# Abstract Supplement Abstracts from AIDS 2022 ‐ the 24th International AIDS Conference, 29 July – 2 August 2022, Montréal, Canada & Virtual

**DOI:** 10.1002/jia2.25935

**Published:** 2022-08-02

## ORAL ABSTRACT

### OAA0102 Pharmacological enhancement of IL‐15 signaling to improve “shock‐and‐kill” strategies against latent HIV

J.N. Howard ^1^, A. Bosque^1^

^1^George Washington University, Microbiology, Immunology, and Tropical Medicine, Washington DC, United States

**Background**: Despite effective anti‐retroviral therapy (ART), the largest barrier to HIV cure remains the formation of a latent reservoir early after initial infection that cannot be cleared by subsequent ART treatment. Finding novel therapeutic strategies that can **S**hock latent HIV, enhance **T**ranslation of viral transcripts, enhance immune **E**ffector functions, and **S**ensitize reactivated cells to apoptosis could enhance **K**illing and elimination of latent HIV reservoirs. As such, development of **STESK** strategies have the potential to improve the efficacy of current “shock and kill” strategies. Among the clinically relevant latency reversing agents (LRA) under investigation, IL‐15 or the IL‐15 superagonist N‐803 have been shown to reactivate latent HIV *ex vivo* and *in vivo*. However, the clinical benefit of IL‐15 can be hindered by the transient nature of cytokine signaling. We previously identified a small molecule, HODHBt, that enhances the biological activity of IL‐15 by increasing STAT5 phosphorylation and transcriptional activity leading to enhanced IL‐15‐ mediated viral reactivation *ex vivo* in cells isolated from ART‐suppressed participants.

**Methods**: We used the Connectivity Map to identify compounds with similar transcriptional profiles to HODHBt and identified five clinically relevant FDA‐approved candidates. We evaluated their ability to promote viral reactivation from latency.

**Results**: From the 5 tested compounds, only one, a retinoid derivative, shared a similar transcriptional profile to HODHBt in CD4T cells. Next, we tested the ability of the retinoid to reactivate latent HIV in a primary cell model of latency. The retinoid (10mM) increased viral reactivation mediated by IL‐15 to a similar extent as HODHBt (100mM) but unlike HODHBt, specifically promoted cell death of latently infected cells compared to controls when combined with IL‐15. In contrast to HODHBt, the retinoid did not increase IL‐15‐induced STAT5 phosphorylation. This indicates that the retinoid is able to reactivate latent HIV through a mechanism mediated by IL‐15 but not directly dependent on STAT5 phosphorylation. There are a number of additional retinoid structural analogues, which we are now investigating for their potential LRA activity.

**Conclusions**: In conclusion, retinol derivatives have the potential to enhance IL‐15 LRA activity and can be ideal candidates for the development of **STESK** strategies against latent HIV.

### OAA0103 Pharmacological targeting of REV‐ERB to modulate HIV transcription and viral outgrowth in CD4+ T cells

C.‐D. Ngassaki‐Yoka ^1,2^, D. Chatterjee^1,2^, Y. Zhang^1,2^, T.R. Wiche Salinas^1,2^, L. Raymond Marchand^2^, N. Cermakian^3,4^, J.‐P. Routy^5^, L. Solt^6^, P. Ancuta^1,2^

^1^Université de Montréal, Montréal, Canada, ^2^Centre de Recherche du CHUM, Montréal, Canada, ^3^McGill University, Montréal, Canada, ^4^Douglas Mental Health University Institute, Montréal, Canada, ^5^McGill University Health Centre: Glen Site, Research Institute, Montréal, Canada, ^6^The Scripps Research Institute, Jupiter, United States

**Background**: Current antiretroviral drugs block the different steps of the viral replication cycle, except the transcription, a process under the control of the host‐cell machinery. Residual HIV transcription in viral reservoirs (VR) persisting during antiretroviral therapy (ART) is a major cause of chronic immune activation and non‐AIDS co‐morbidities. The Th17‐polarized CD4^+^ T cells are highly enriched in VR in people living with HIV (PLWH) receiving viral‐suppressive ART. In previous studies, we demonstrated that the transcriptional signature associated with HIV permissiveness in Th17 cells includes the circadian clock components/regulators REV‐ERBa/b, BMAL1 and RORC2. Of note, REV‐ERB acts as a transcriptional repressor of BMAL1 (a transcriptional activator binding to E‐boxes in the HIV promoter) and RORC2 (the master regulator of Th17 polarization). Thus, we hypothesized that REV‐ERB regulates both BMAL1‐mediated HIV transcription/replication and RORC2‐mediated effector functions in Th17 cells.

**Methods**: To test this hypothesis, we used the REV‐ERB agonist SR9011 and antagonist SR8278. Memory CD4^+^ T cells from uninfected individuals were stimulated with CD3/CD28 antibodies and exposed to replication‐competent HIV_Nbal_ and single‐round VSV‐G‐pseudotyped HIV (HIV_VSVG_) *in vitro* in the presence/absence of drugs. A viral outgrowth assay (VOA) was performed with memory CD4^+^ T cells of ART‐treated PLWH activated *via* CD3/CD28. Cytokines and HIV‐p24 levels were measured by ELISA and/or flow cytometry. HIV‐DNA integration was quantified by nested real‐time PCR.

**Results**: CD3/CD28‐mediated triggering in memory CD4^+^ T cells resulted in a significant downregulation of both REV‐ERBa/β mRNA. As expected, the expression of BMAL1 and RORC2 transcripts was upregulated. Upon HIV_VSVG_ exposure, the antagonist SR8278 increased HIV‐DNA integration and intracellular HIV‐p24 expression (p=0.0103, p=0.0001 respectively), revealing its latency reversing potential. However, SR8278 did not increase HIV‐p24 release from HIV_VSVG_‐infected cells, which indicates a post‐translational REV‐ERB‐dependent block in HIV replication. In contrast, the agonist SR9011 potently inhibited HIV_Nbal_ replication *in vitro* (p=0.0024) and viral outgrowth in cells of ART‐treated PLWH. The antiviral effects of SR9011 coincided with a decrease of IL‐17A and IFN‐γ production.

**Conclusions**: These results provide a strong rationale for further evaluating the possibility to therapeutically target REV‐ERB in an effort to modulate BMAL1/RORC2‐dependent HIV transcription and subsequently improve the efficacy of current ART regimen in PLWH.

### OAA0104 Interactome of HIV proteins and their host RNA interaction partners

T. Schynkel ^1^, W. van Snippenberg^1,2^, K. Verniers^2^, E.J. de Bony^2^, G.M. Jang^3^, N.J. Krogan^3^, P. Mestdagh^2^, L. Vandekerckhove^1^, W. Trypsteen^1^

^1^Ghent University, HIV Cure Research Center, Ghent, Belgium, ^2^Ghent University, OncoRNALab, Center for Medical Genetics (CMGG), Ghent, Belgium, ^3^University of California, Department of Cellular and Molecular Pharmacology, San Francisco, United States

**Background**: The HIV genome encodes a limited set of proteins, depending heavily on the exploitation of host cell molecules to complete its viral life cycle and maintaining latency. Recent findings show that HIV hijacks cellular (non‐coding) RNA molecules to aid in these crucial viral processes. Therefore, this study aims to systematically determine this new layer of physical interactions of each for the 18 HIV proteins with host RNA molecules.

**Methods**: An RNA immunoprecipitation (RIP)‐seq strategy was established in Jurkat cell lines that express a single FLAG‐ and streptavidin‐tagged HIV protein upon doxycycline induction. For each of the 18 HIV proteins, FLAG‐based immunoprecipitations (IPs) were performed (triplicate), followed by RNA purification, stranded total RNA library preparation and sequencing (50M reads/sample, Illumina NextSeq). Background controls included mouse IgG antibody and FLAG‐tagged GFP protein IP. Enriched RNA transcripts were identified after mapping (STAR), background filtering based on background controls and performing a differential expression analysis (DEseq2). For 6 HIV proteins also Streptavidin‐based RIPseq was performed (triplicate).

**Results**: The identified interactome comprises a set of 1162 HIV protein – host RNA interactions (FC > 4 and padj < 0.05) across 8 HIV proteins: Nucleocapsid (939), Rev (94), Gag (57), Matrix (47), Tat (12), Integrase (5), Pol (5) and Protease (3). The majority of the identified RNA interaction partners are mRNAs (55%), and also include tRNAs (7%), pseudogenes (25%) and (long) non‐coding RNAs (3%), indicating a wide variety of RNA families that are recruited during replication. Furthermore, this interactome corroborates previous work, as known interactors were identified for Matrix, Gag, Rev (tRNAs) and Tat (7SKRNA). For Tat in specific, additional RNA interaction partners include mRNAs for which the translation protein products are known interactors of Tat within the super elongation complex (SEC), hinting at a regulatory role of Tat in the expression of these SEC subunits.

**Conclusions**: This unique and comprehensive dataset of HIV protein – human RNA interactions broadens our understanding on how HIV manipulates the RNA component of the host's cellular machinery during the course of infection and to establish and maintain latency. This information will support the search for latency reversing agents for a potent shock‐and‐kill strategy.

### OAA0105 Inducibility and distribution of HIV proviruses in early treated Thai children on suppressive ART

M. Massanella ^1^, C. Dufour^1^, A. Pagliuzza^1^, C. Richard^1^, J. Ananworanich^2^, L. Leyre^1^, T. Jupimai^3^, J. Mitchell^4^, P. Sawangsinth^5^, M. de Souza^5^, P. Suntarattiwong^6^, K. Chokephaibulkit^7^, L. Trautmann^4^, R. Fromentin^1^, T. Puthanakit^3^, N. Chomont^1^, HIVNAT209 and HIVNAT194 study groups

^1^Centre de Recerche du CHUM, Montreal, Canada, ^2^Amsterdam Medical Center‐University of Amsterdam, Department of Global Health, Amsterdam, Netherlands, the, ^3^Chulalongkorn University, Center of Excellence in Pediatric Infectious Diseases and Vaccines, Bangkok, Thailand, ^4^Vaccine and Gene Therapy Institute, Beaverton, United States, ^5^SEARCH, Thai Red Cross AIDS research Centre, Bangkok, Thailand, ^6^Queen Sirikit National Institute of Child Health, Bangkok, Thailand, ^7^Siriraj Hospital ‐ Mahidol University, Pediatrics, Bangkok, Thailand

**Background**: Although vertically infected infants display detectable levels of HIV DNA in CD4 T‐cells, inducible assays such as QVOA and TILDA often yield negative results. These observations suggest that CD4 T‐cells from children are refractory to stimulation, possibly because they mostly display a naïve phenotype. We assessed the distribution and inducibility of HIV proviruses in CD4 T‐cell subsets over time, since frequencies of memory cells increase as children age.

**Methods**: Eight vertically‐infected children who initiated ART within 5 months of life were followed longitudinally and provided blood samples collected at median ages of 1.7 (V1) and 4.3 (V2) years. Frequencies of CD4 T‐cells producing msRNA and p24 protein upon PMA/ionomycin stimulation were measured by TILDA and HIV‐Flow, respectively. Stimulated CD4 T‐cells for HIV‐Flow analysis were concomitantly sorted by flow cytometry to obtain naïve (CD45RA+CCR7+CD27+), central memory (CM, CD45RA‐CCR7+CD27+), transitional memory (TM, CD45RA‐CCR7‐CD27+) and effector memory (EM, CD45RA‐CCR7‐CD27‐) cells. Integrated HIV DNA was quantified in sorted subsets.

**Results**: Despite high frequencies of naïve CD4 T‐cells (>70%), naïve cells were rarely infected (median 38 [6‐143] and 7 [0‐12] integrated HIV DNA copies/10^6^ cells at V1 and V2, respectively). Most proviruses were detected in memory subsets, and infection frequencies increased with cell differentiation (EM>TM>CM, median 2820 [176‐9469], 1432 [129‐6711] and 507 [142‐1309] integrated HIV DNA copies/10^6^ cells, respectively at V1 and 260 [74‐2161], 195 [89‐1350] and 100 [40‐426] integrated HIV DNA copies/10^6^ cells, respectively at V2). Frequencies of infected naïve and CM cells decreased over time (p=0.05 and p=0.008, respectively). Despite the low frequency of memory cells, CM cells were the main contributor to the pool of cells carrying integrated proviruses at both visits (46% and 50% at V1 and V2, respectively). Importantly, there were no significant changes in TILDA or HIV‐Flow values over time, which were barely detectable at both visits.

**Conclusions**: Although high levels of HIV genomes are present in memory cells from vertically infected children on ART, they do not produce detectable levels of p24 protein upon stimulation. The latent reservoir seems poorly inducible in children and inducibility does not increase over time.

### OAA0202 An immunological signature for subclinical atherosclerosis in people living with HIV‐1 receiving antiretroviral therapy

T.R. Wiche Salinas ^1^, A. Gosselin^1^, Y. Zhang^1^, N. Fonseca Do Rosario^1^, A. Filali^1^, J.‐P. Routy^2^, C. Chartrand‐Lefebvre^3^, A.L. Landay^4^, M. Durand^1^, M. El‐Far^1^, C. Tremblay^1^, P. Ancuta^1^

^1^Centre de recherche du Centre Hospitalier de l'Université de Montréal, Montreal, Canada, ^2^Research Institute of the McGill University Health Centre, Chronic Viral Illness Service and Division of Hematology, Montreal, Canada, ^3^Université de Montréal, Département de Radiologie, Radio‐oncologie et Médecine Nucléaire, Faculté de Médecine, Montreal, Canada, ^4^Rush University Medical Center, Chicago, Canada

**Background**: Cardiovascular disease (CVD) is an important co‐morbidity in people living with HIV (PLWH) receiving antiretroviral therapy (ART+PLWH). This study explores immunological patterns associated with subclinical coronary artery atherosclerosis during ART‐treated HIV infection in relationship with alterations in gut‐associated lymphoid tissues.

**Methods**: Uninfected (HIV‐; n=61) and ART+PLWH (n=21) with/without subclinical atherosclerosis participants were included in the Canadian HIV and Aging Cohort Study (CHACS)/CVD Cohort. Total plaque volume (TPV), and low attenuated plaque volume (LAPV) were determined by coronary CT angiography.Markers of microbial translocation (LBP, I‐FABP, sCD14, CCL20, MIF and CX3CL1), lipid profiles (LDL, HDL and triglycerides) and coagulation (D‐dimer, fibrinogen) were quantified in plasma. Flow cytometry analysison peripheral blood mononuclear cells were performed to characterize the frequency and expression of chemokine receptors involved in atherosclerotic plaque infiltration (CCR2, CCR6, CCR9, CX3CR1) in Th17 (CCR6+CD26+CD161+), Tregs (CD25highCD127‐FOXP3+), classical/intermediate/non‐classical monocyte (CD14/CD16/M‐DC8) and myeloid (CD1c+HLA‐DR+)/plasmacytoid (BDCA2+/CD123+) dendritic cells.

**Results**: ART+PLWH distinguished from HIV‐ by lower levels of HDL and higher levels of sCD14, FABP2, CCL20, MIF, CX3CL1, and triglycerides. Additionally,ART+PLWHshowed higher frequencies of Tregs and lower Th17/Treg ratios compared to HIV‐ participants. The stratification of ART+PLWH based on the presence (TPV+) or the absence (TPV‐) of subclinical coronary atherosclerotic plaque demonstrated reduced Th17 frequencies and Th17/Treg ratios in TPV+*versus*TPV‐.Also, there was a superior frequency of non‐classical CCR9‐ and M‐DC8+ monocytes expressing high levels of HLA‐DR in TPV+*versus*TPV‐ ART+PLWH. A logistic regression model was used to determine the association between covariates and the presence of coronary atherosclerotic plaque. In crude analyses, the frequency of non‐classical CCR9+HLADRlow [OR: 0.30 (0.15‐0.63)] and non‐classical CCR9‐HLADRhigh [OR:3. 56(1.45‐8.74)] monocytes, as well as the Th17/Treg ratio [0.38(0.18‐0.83)] were associated with TPV values. After adjusting for age, smoking and LDL or statins, smoking and triglycerides, only the frequency of CCR9+HLADRlow and CCR9‐HLADRhigh monocytes remained significantly associated with coronary plaque, whereas Th17/Tregs ratio kept its significant association exclusively when adjusted for age, smoking and LDL.

**Conclusions**: We identified a new immunological signature associated with presence of coronary plaque that may serve in clinical practices for an improved management of CVD risk in ART+PLWH.

### OAA0203 Investigating the development of T cell immunity in acute HIV‐1 infection through a longitudinal analysis of the TCR repertoire

M.M. Magnoumba Legnanga ^1^, K. Reddy^1^, T. Ndung'u^1,2^, A. Leslie^1,3^

^1^Africa Health Research Institute, Immunology, Durban, South Africa, ^2^HIV Pathogenesis Programme, Health and Science Research, Durban, South Africa, ^3^University College London, Immunology, London, United Kingdom

**Background**: Evidence suggests that many events leading to the long‐term T‐cell dysfunction that is a hallmark of HIV infection, occur during the acute phase. Previous longitudinal study of individuals identified in hyper‐acute infection showed those mounting a rapid and large CD8 cytotoxic T‐cells (CTL) response achieved superior natural control of HIV. However, due to sample availability, the dynamic response of the T‐cell compartment during this acute phase remains poorly understood, as does the potential involvement of non‐classical T‐cells. Consequently, Antigen‐specific T cell responses remain a key feature in understanding the relationship between the disease and host.

**Methods**: This study used longitudinal DNA samples of pre‐infected and post infected young african women between 18‐23 years of age. For TCRα sequencing, purified genomic DNA was sequenced by Adaptive Biotechnologies using the ImmunoSEQ assay. Analysis were carried using R and python to determine TCR clusters and predict antigen‐driven TCR clustering, additional methodologies like VDJtools were also used to determine the TCR repertoire diversity.

**Results**: In this study, we used bulk TCR sequencing to track the dynamics of the TCR repertoire longitudinally in HIV individuals sampled before infection and serially through the untreated acute phase. Using this approach, we find that, in multiple individuals, the TCR landscape is highly dynamic during the acute phase of infection. This landscape is characterised by large clonal expansions, reaching 30% frequency during Pre‐infection to 60%; 51% and 52% in early, mid and late infection respectively. Interestingly, some TCR clonotypes persist while others rapidly disappear and are replaced by new and previously unexpanded clonotypes. Antigen prediction tools are being employed to determine the specificity of persisting TCRs and those that are lost. In contrast to convention TCRs, invariant and semi‐invariant clonotypes, including MAITS, iNKTs, and gamma delta TCRs, show no significant expansions during the acute phase. This observation implies that Donor Unrestricted T‐cells (DURTS) do not respond directly to HIV antigen or acute phase cytokines and that HIV‐associated changes in these subsets occur during the chronic phase.

**Conclusions**: Together these data demonstrate extensive skewing of the T‐cell repertoire that occurs during acute HIV infection and may impact long‐term immune health.

### OAA0204 Optimization of a VLP‐forming HIV‐1 env‐gagmRNA vaccine by inclusion of gag‐pol mRNA to express the viral protease

P. Zhang^1^, S. Falcone^2^, G. Stewart‐Jones^3^, Y. Seo^1^, D. Rogers^1^, H. Miao^1^, Q. Liu^1^, Y.‐T. Lai^2^, I. Renzi^2^, E. Narayanan^3^, S. Himansu^2^, A. Carfi^2^, P. Lusso ^1^

^1^NIH, LIR, NIAID, Bethesda, United States, ^2^Moderna Inc., Cambridge, United States, ^3^Moderna Inc, Cambridge, United States

**Background**: The development of a protective vaccine remains a top priority for the control of the HIV/AIDS pandemic. Taking advantage of recent advances in mRNA technology, we developed an *env‐gag* mRNA vaccine that yielded promising results in macaques.

**Methods**: Five groups of wild‐type (WT) Balb‐c mice (n=8 per group) were sequentially immunized with mRNA encoding different forms of a clade‐C HIV‐1 envelope (Env), 426c, bearing a truncated gp41 cytoplasmic tail to enhance expression, starting with two immunizations (weeks 0, 4) with an open form lacking three N‐glycans (276, 460, 463) around the CD4‐binding site (3Δgly), followed at week 16 by a partially glycan‐restored form lacking only the 276 glycan (Δ276), and finally at week 20 by WT 426c *env*. The *env* mRNA was inoculated at 2.5 μg/dose either alone (Arm 1) or co‐formulated with SIV *gag* mRNA at 2.5 μg/dose in order to induce the *in vivo* formation of virus‐like particles (VLP) (Arm 2), or with SIV *gag* and *gag‐pol*mRNA in order to express the viral protease, which is essential for processing Gag to its mature form. To identify the optimal dose of *gag‐pol*mRNA, different *gag:gag‐pol*molar ratios were tested (5:1, 10:1, and 20:1, Arms 3‐5). Serum was collected after each immunization and tested for trimer‐binding antibodies by ELISA and neutralizing antibodies (NAbs) by the TZMbl assay.

**Results**: *In vitro*, co‐transfection of *gag‐pol*mRNA with *env* and *gag*mRNA resulted in both quantitative and qualitative improvements in VLP production. All groups of immunized mice developed trimer‐binding antibodies. NAbs against 426c‐3Δgly, and to a lesser extent 426c‐Δ276, started to appear after the second immunization, with the highest titers in Arm 3 (with *gag‐pol*mRNA at 1:5). Boosting with 426c‐Δ276 enhanced NAb titers, especially against 426c‐Δ276, with *gag‐pol‐*containing regimens showing the highest titers, followed by *env+gag*and, lastly,*env* alone. No neutralization of WT virus was observed.

**Conclusions**: Our results illustrate a further improvement of our VLP‐forming HIV‐1 *env‐gag* mRNA vaccine platform through the addition of *gag‐pol* mRNA to promote Gag processing. The triple mRNA co‐formulation provides an optimized platform to test the efficacy of different HIV‐1 Env immunogens in pre‐clinical and clinical studies.

### OAA0205 Exceptional post‐treatment control associated with strong NK and ϒɗ cytotoxic T cells

N. Climent ^1,2^, J. Ambrosioni^1,2^, T. González^1,2^, M. Casadellà^3^, M. Noguera^3^, R. Paredes^3^, M. Plana^1,2^, J. Mallolas^1,2^, J. Alcamí^1,4,2^, S. Sánchez‐Palomino^1,2^, J.M. Miró^1,2^

^1^Hospital Clinic‐IDIBAPS/University of Barcelona, HIV Unit, Barcelona, Spain, ^2^CIBER of Infectious Diseases (CIBERINFEC), Madrid, Spain, ^3^IrsiCaixa AIDS Research Institute. Hospital Universitari Germans Trias i Pujol, Badalona, Spain, ^4^Instituto de Salud Carlos III (ISCIII)., Madrid, Spain

**Background**: Although ART is effective in suppressing viral replication, HIV persists in reservoirs and rebounds after stopping therapy. However, there are few patients, such as post‐treatment controllers (PTC), who are able to maintain viral loads below detection limits without ART, being a realistic model for the HIV‐functional‐cure. We describe the mechanisms of control of an exceptional PTC (>15 years).

**Methods**: A 59‐year woman with sexually‐acquired acute HIV‐infection was included in the `Immune‐mediated PHI trial´ (NCT00979706), involving several interventions: short course of low doses of CsA, IL‐2, GM‐CSF and Peg‐α‐IFN followed by analytical STI. Virological studies were performed: total and integrated HIV‐1 DNA in CD4^+^ T‐cells and rectal tissue, viral outgrowth assay (qVOA), HIV‐1 infectivity in PBMC and CD4^+^ T‐cells cultures and viral inhibitory activity (VIA) of autologous CD4^+^T‐cells with NK and CD8^+^ T‐cells. NK and T‐cell phenotype was determined by flow‐cytometry.HLA class I, Δ32CCR5 and NKG2C alleles were genotyped.

**Results**: After antiretroviral and immunomodulatory treatment, the patient maintained undetectable viral load in plasma for 15 years. HIV‐1 subtype was CFR_02AG, R5‐tropic. We found a pronounced and progressive fall of the viral reservoir (VR): total HIV‐DNA (from 4573.50 to 95.33 copies/10^6^ CD4^+^T‐cells) and integrated proviral DNA (from 85.37 to 5.25 copies/10^6^ CD4^+^T‐cells).VR in rectal biopsy was 3 HIV DNA total copies/10^6^ cells and qVOA detected 1.61 UIMP at year 9. VIA assay showed strong inhibition of *in vitro* replication in co‐cultures with autologous NK‐cells or CD8^+^T‐cells at 1:2 ratio (75% and 62%, respectively). Co‐cultures with NK and CD8^+^T‐cells resulted in 93% inhibition of HIV‐replication. Higher levels of both NKG2C^+^‐memory‐like NK‐cells and NKG2C^+^ϒɗ^+^T‐cells than referenced data from untreated normal HIV‐infected progressors were detected (46.2% versus 24.0% and 64.9% versus 19.7%, respectively). The patient has A*29:01/A*29:01, B*44:03/B*44:03, C*16:01/C*16:01 HLA‐I, wt/wt CCR5 and wt/wt NKG2C alleles.

**Conclusions**: We describe the case of functional cure in a 59‐years‐old woman treated during PHI that has maintained undetectable viral load for 15 years without ART. Replication‐competent HIV‐1 could be isolated by qVOA. NKG2C^+^‐memory‐like NK‐cells and ϒɗ^+^CD8^+^T‐cells contribute to the control of viral‐replication and functional‐cure observed. Strategies able to expand these cells could help to achieve HIV‐functional‐cure.

### OAA0302 Scarcity of intact HIV genomes in vertically infected Thai children who initiated ART during the first months of life

C. Dufour ^1^, M. Massanella^1^, A. Pagliuzza^1^, C. Richard^1^, J. Ananworanich^2^, L. Leyre^1^, T. Jupimai^3^, J. Mitchell^4^, P. Sawangsinth^5^, M. de Souza^5^, P. Suntarattiwong^6^, K. Chokephaibulkit^7^, L. Trautmann^4^, T. Puthanakit^3,8,9^, R. Fromentin^1^, N. Chomont^1^

^1^Université de Montréal/Centre de Recherche du CHUM, Microbiology, Infectiology and Immunology, Montreal, Canada, ^2^University of Amsterdam/Amsterdam Medical Center, Department of Global Health, Amsterdam, Netherlands, the, ^3^Chulalongkorn University/Center of Excellence in Pediatric Infectious Diseases and Vaccines, Bangkok, Thailand, ^4^Oregon Health & Science University/Vaccine and Gene Therapy Institute, Portland, United States, ^5^SEARCH/Thai Red Cross AIDS research Centre, Bangkok, Thailand, ^6^Queen Sirikit National Institute of Child Health, Bangkok, Thailand, ^7^Mahidol University/Siriraj Hospital, Faculty of Medicine, Department of Pediatrics, Bangkok, Thailand, ^8^Chulalongkorn University, Faculty of Medicine, Department of Pediatrics, Bangkok, Thailand, ^9^The HIV Netherlands Australia Thailand Research Collaboration (HIV‐NAT), the Thai Red Cross AIDS Research Centre (TRC‐ARC), Bangkok, Thailand

**Background**: Latently infected cells harboring intact HIV genomes persist in children living with HIV receiving suppressive ART. However, the dynamics of these genetically intact viral genomes over time remains unclear.[Fig jia225935-fig-0001]

**Methods**: Thai children vertically infected with HIV who initiated ART within the first 6 months of life were enrolled in the HIVNAT209 & HIVNAT194 studies and followed longitudinally. We used cross sectional blood samples collected from infants before initiation of ART (n=3) as well as in virally suppressed children on ART for 2 years (n=6), 3 years (n=5) and more than 3 years (n=5). Near‐full length (NFL) proviral sequences were obtained by FLIPS on enriched CD4 T cells and PacBio sequencing.

**Results**: We obtained a total of 939 NFL HIV genomes (200 before ART and 282, 263 and 194 after 2, 3,and >3 years of ART, respectively). Prior to ART initiation, 48% of the viral genomes were genetically intact (Fig. 1). This proportion drastically decreased to 11%, 6% and 1% after 2, 3 and >3 years of ART, respectively, while proviruses presenting large internal deletions largely dominated (>79%). Clonally expanded proviruses (i.e., 100% identical sequences) were rare before ART initiation (7%) and observed in only 1 of the 3 samples studied. However, the proportion of identical genomes dramatically increased to 36% after 2 years of ART and remained stable afterwards (36% and 40% at 3 and >3 years of ART, respectively). None of these clonally expanded genomes during suppressive ART were genetically intact.

**Abstract jia225935-fig-0001:**
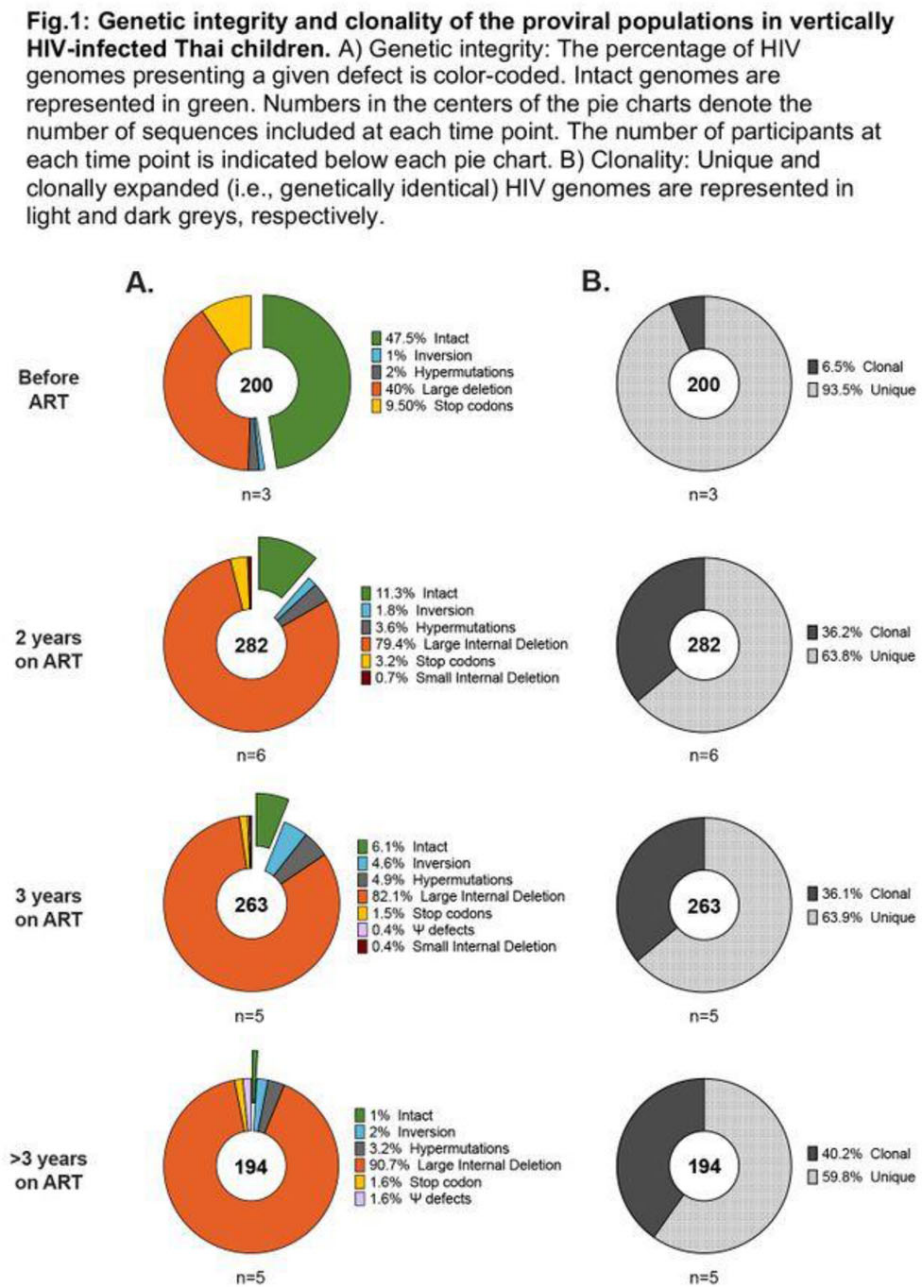
**Abstract OAA0302‐Figure 1**.

**Conclusions**: These results suggest that genetically intact HIV genomes are massively depleted during the first years of ART in children who initiated treatment during the first months of life. Although clonal expansions of defective proviruses were frequently observed during ART, genetically intact HIV genomes were scarce and always unique.

### OAA0303 HIV SMRTcap, a novel single molecule, long‐read sequencing assay to characterize the HIV‐1 reservoir in cells and tissues across multiple subtypes

D. Sachs^1^, W. Lauer^2^, P. Singh^3,4^, P. Warburton^1^, M. Emery^1^, J. Powell^1^, J. Soto^1^, G. Deikus^1^, J. Kasule^5^, J. Prodger^6^, A. Tobian^7^, A. Redd^8,9^, S. Morgello^10^, A. Engelman^3,4^, R. Sebra^1,11^, M. Smith ^2^

^1^Icahn School of Medicine at Mount Sinai, Genetics and Genomic Sciences, New York, United States, ^2^University of Louisville, Biochemistry and Molecular Genetics, Louisville, United States, ^3^Dana‐Farber Cancer Institute, Cancer Immunology and Virology, Boston, United States, ^4^Harvard Medical School, Medicine, Boston, United States, ^5^Rakai Health Sciences Program, Kalisizo, Uganda, ^6^Western University, Microbiology and Immunology, London, Canada, ^7^Johns Hopkins University, Pathology, Baltimore, United States, ^8^Johns Hopkins University, Division of Infectious Disease, Baltimore, United States, ^9^National Institutes of Health, National Institutes of Allergy and Infectious Disease, Bethesda, United States, ^10^Icahn School of Medicine at Mount Sinai, Neurology, Pathology, and Neuroscience, New York City, United States, ^11^Sema4, Stamford, United States

**Background**: HIV reservoir characterization has focused on classifying integration sites and examining the integrity of proviral genomes, many of which contain defects rendering them replication incompetent. Current methods mainly examine either integration sites or proviral integrity, and perform robustly in cell‐based assays, with limited application to tissue‐specific reservoirs. There has also been scant effort to expand these assays to non‐subtype B samples. To address these limitations, we developed a novel single molecule assay, HIV SMRTcap, that provides simultaneous resolution of the “HIV integron” (proviral genome and matched integration sites) in cells and tissues across all major HIV‐1 subtypes.

**Methods**: HIV+ genomic DNA is extracted from cells or tissues and sheared into 11‐15kb fragments. Oligo‐based enrichment is performed using a custom panel of biotinylated 120‐mers specific for HIV genomes representing all major subtypes. Oligo‐bound fragments are enriched with streptavidin beads and used for sequencing library preparation. Sequencing is performed on the Sequel IIe system to obtain highly accurate (>99.99%) long reads, which allow for simultaneous characterization of the integration site, proviral intactness, and clonality.

**Results**: HIV SMRTcap successfully enriched HIV integron events by >2400‐fold from multiple sources: *in vitro* infection, primary PBMC, multiple autopsy‐ and biopsy‐derived tissues (including spleen, kidney, liver, lymph node, basal ganglia), and across samples infected with HIV subtypes A, B, C, and D. HIV SMRTcap resolved both intact and defective proviral genomes, demonstrating that partial tiling of oligo baits was sufficient for capture. Intriguingly, analysis of samples using HIV SMRTcap revealed integration into repeat elements (i.e., LINEs, SINEs) absent from matched data obtained with linker‐mediated PCR, likely due to limitations in mapping short‐read data.

**Conclusions**: Standard short‐read HIV reservoir measurement methods do not provide direct simultaneous evaluation of the complete HIV integron, limiting investigations of the impact of integration site on clonal expansion and retention of intact proviral genomes in patient samples. Moreover, specialized methods that can link this information (i.e., MIP‐seq) are often prohibitively expensive and labor intensive. HIV SMRTcap utilizes long‐read sequencing to characterize complete HIV integrons in a cost‐effective, scalable manner, and has tremendous potential to accurately investigate HIV reservoir dynamics during evaluation of HIV cure strategies.

### OAA0304 Intact HIV proviruses persist in the central nervous system despite viral suppression with antiretroviral therapy

C. Cochrane^1,2^, T. Angelovich^1,3,4^, A. Guanizo^1^, G. Trollope^1^, L. Senior^1^, E. Wanicek^1^, J. Jamal Eddine^1^, M. Gartner^5^, E. Waring^1^, J. Estes^6^, B. Brew^7^, S. Lewin^3,8^, M. Roche ^1,3^, M. Churchill^1,4,9^

^1^RMIT University, School of Health and Biomedical Sciences, Melbourne, Australia, ^2^University of Melbourne, Department of Medicine, Melbourne, Australia, ^3^The University of Melbourne, Department of Infectious Diseases, Melbourne, Australia, ^4^Burnet Institute, Life Sciences, Melbourne, Australia, ^5^University of Melbourne, Department of Microbiology and Immunology, Melbourne, Australia, ^6^Oregon Health and Science University, Vaccine and Gene Therapy Institute, Oregon National Primate Research Centre, Portland, United States, ^7^St Vincent's Hospital, University of New South Wales, Departments of Neurology and Immunology, Sydney, Australia, ^8^Alfred Hospital and Monash University, Department of Infectious Diseases, Melbourne, Australia, ^9^Monash University, Departments of Microbiology and Medicine, Melbourne, Australia

**Background**: HIV persistence in blood and tissue reservoirs represents the major barrier to HIV cure and is a possible cause of comorbid disease. HIV is known to infect the central nervous system (CNS); however, to date the size and replication competent nature of the CNS reservoir is unclear.

**Methods**: Here we employed a droplet digital PCR assay to detect total HIV DNA and the intact proviral DNA assay (IPDA) to provide the first quantitative assessment of the intact and defective HIV reservoir in well‐characterized brain tissues from autopsies. Further phenotypic characterization of brain reservoir cells was provided by in situ hybridization (DNAscope) targeting HIV DNA and laser capture microdissection and PCR of CD68+ brain cells.

**Results**: HIV DNA was present at similar levels in brain tissues from untreated viremic or antiretroviral (ART)‐suppressed individuals (n=36; median: 22.3 vs 26.2 HIV *pol* copies/10^6^ cells), reflecting a stable CNS reservoir that persists despite therapy. Furthermore, 9/12 viremic and 5/8 virally suppressed individuals also harbored intact proviruses in the CNS (13.5 vs 4.63 intact copies/10^6^ cells). CNS and peripheral reservoirs harbored a similar frequency of intact proviruses (∼20% of proviruses). In situ hybridization (DNAscope) identified the presence of HIV DNA in brain myeloid cells and sequences of proviruses isolated from purified brain myeloid cells compartmentalized relative to those from matched peripheral lymphoid tissue reservoirs, indicating that the CNS harbors a distinct reservoir.

**Conclusions**: Thus, here we provide the first evidence of an intact, potentially replication competent, HIV reservoir in the CNS of virally suppressed people living with HIV.

### OAA0305 SARS‐CoV‐2 mRNA vaccination exposes latent HIV to Nef‐specific CD8+ T cells

E. Stevenson^1^, S. Terry^1^, D. Copertino^1^, L. Leyre^1^, A. Ward^1^, P. Khadka^1^, K. Bernard^1^, G. Ellsworth^1^, C. Johnston^1^, M. Caskey^2^, C. Gaebler^2^, T. Wilkin^1^, G. Lee^1^, R.B. Jones ^1^

^1^Weill Cornell Medicine, Infectious Diseases Division, New York city, United States, ^2^Rockefeller University, New York City, United States

**Background**: SARS‐CoV‐2 mRNA vaccines activate TLR and inflammatory signaling pathways, and thus may activate transcription from HIV proviruses. We hypothesized that, in addition to virological measures, a key manifestation of this would be*in vivo*boosting of T‐cell responses specific for the early gene product HIV‐Nef. This was based upon our previously reported findings that Nef‐specific T‐cells disproportionately recognize residual antigen expression during long‐term antiretroviral therapy (ART), enforcing an effector profile (granzyme‐B release).

**Methods**: HIV RNA in PBMC supernatants was measured by RT‐qPCR. T‐cell responses to HIV gene products were measured at baseline and ∼2 weeks after SARS‐CoV‐2 mRNA vaccine prime and boost in 13 ART‐treated adults using IFN‐gor granzyme‐B ELISPOT, as well as activation induced marker (AIM) assays. Total and unspliced HIV mRNA, as well as intact and defective (IPDA) HIV DNA were measured in parallel by digital droplet PCR (ddPCR).

**Results**: Treatment of PBMCs from ART‐treated donors with SARS‐CoV‐2 mRNA vaccines drove release of HIV RNA into supernatants (n=6, p=0.03). Within days of vaccinations, we observed transient *in vivo* increases in cell‐associated HIV RNA. Nef‐specific T‐cell responses increased following vaccine‐prime by granzyme‐B ELISPOT (3.1‐fold increase, p=0.002), with a parallel trend by AIM assay (1.5‐fold, p=0.06). Analogous increases were not observed in responses to late gene products. Unspliced and total HIV mRNA decreased modestly between baseline and 2 weeks post vaccine‐boost, unspliced‐1.6‐fold decrease p=0.03; total‐1.5‐fold decrease p=0.05. Changes in total HIV mRNA showed strong inverse correlations with Nef‐specific granzyme B‐producing (spearman'sr=‐0.73, p=0.006) and Nef‐specific CD8+ AIM T‐cell responses (r=‐0.76, p=0.006) following vaccine prime. Neither total nor intact HIV DNA changed significantly across the study.

**Conclusions**: SARS‐CoV‐2 mRNA vaccination induced a degree of HIV latency reversal, resulting in engagement of HIV‐Nef‐specific CD8+ T‐cells with a cytotoxic profile. The strong correlations between increases in these responses and subsequent decreases in cell‐associated HIV RNA suggest some elimination of transcriptionally‐active cells. However, this was not accompanied by reductions in intact or total HIV DNA. This may reflect that only a minority of proviruses were responsible for transcription, both at baseline and following latency reversal, consistent with recent observations in the field.

### OAA0402 Potent latency reversal enables in‐depth transcriptomic analyses of the HIV reservoir

M. Pardons ^1^, B. Cole^1^, L. Lambrechts^1^, S. Rutsaert^1^, Y. Noppe^1^, E. Nijs^2^, E. Van Gulck^2^, D. Boden^3^, L. Vandekerckhove^1^

^1^HIV Cure Research Center, Ghent University, Department of Internal Medicine and Pediatrics, Ghent, Belgium, ^2^Janssen Infectious Diseases, Janssen Research and Development, Division of Janssen Pharmaceutica NV, Beerse, Belgium, ^3^Janssen Infectious Diseases, Division of Janssen Pharmaceutica NV, South San Francisco, United States

**Background**: Extensive characterization of the translation‐competent reservoir has been hampered by the limited capacity of current latency reversing agents (LRAs) at inducing HIV reactivation *in vitro*. Here, we describe a new LRA combination (JNJ877+PNB) that induces potent latency reversal without inducing global T cell activation, and we took advantage of these unique properties to study transcriptomic features of the inducible reservoir.

**Methods**: CD4 T cells from 22 ART‐treated individuals were stimulated for 24H with PMA/ionomycin (PMA/i) or with JNJ877 in combination with panobinostat (PNB). The frequency of cells expressing p24 and viral release in the supernatant were assessed by HIV‐Flow and p24‐SIMOA, respectively. HIV clones reactivated by JNJ877+PNB were compared to those reactivated with PMA/i using the STIP‐seq assay, which allows for the simultaneous assessment of the integration site and proviral sequence from p24+ cells. Single‐cell RNA‐seq on sorted p24‐/p24+ cells from 7 ART‐treated individuals was used to study cellular and viral transcripts following stimulation with JNJ877 or JNJ877+PNB.

**Results**: JNJ877+PNB induced HIV reactivation in a larger fraction of CD4 T cells than PMA/i (n=22, p<0.00001, fold increase=5X). Similar results were obtained with SIMOA (n=4), confirming an effective release of viral particles in the supernatant. Clones reactivated with JNJ877+PNB were mostly shared with the ones induced by PMA/i, although some were represented in different proportions. Single‐cell RNA‐seq analyses showed that JNJ877 does not modify the cellular transcriptome of CD4 T cells. Following JNJ877 treatment, p24+ cells significantly expressed higher levels of a novel long non‐coding RNA, *SOD1P3*, *CCL5* and *GZMA*, while expressing lower levels of *ATG10* and *IL7R* when compared to p24‐ cells. Finally, transcriptomic analyses on 321 p24+ cells revealed that proviruses with a defective major splice donor (MSD) site use alternative splice sites up‐ and/or downstream of the MSD, suggesting an underestimated role of these proviruses in HIV pathogenesis.

**Conclusions**: We report a combination of LRAs that induces latency reversal in a higher proportion of latently infected cells compared to PMA/i, without inducing global T cell activation. Therefore, JNJ877+PNB appears as a promising LRA combination to reactivate HIV *in vitro* and *in vivo*, paving the way to an HIV cure.

### OAA0403 In vivo preclinical efficacy of MGD014 and MGD020 (HIV‐1 envelope x CD3 DART molecules) and first‐in‐human phase 1 clinical safety evaluation of MGD014

J.L. Nordstrom ^1^, C.L. Gay^2^, C. Bohac^1^, A.R. Wahl^3^, T. Morgan^3^, J.L. Schmitz^4^, H. Li^1^, J. Cotshott^1^, J. Gobburu^5^, A.N. Macintyre^6^, R.J. Gorelick^7^, G. Ferrari^8^, D.M. Margolis^2^, J.V. Garcia^3^

^1^MacroGenics, Inc., Rockville, United States, ^2^University of North Carolina at Chapel Hill School of Medicine, UNC HIV Cure Center and Department of Medicine, Chapel Hill, United States, ^3^University of North Carolina at Chapel Hill, International Center for the Advancement of Translational Science, Division of Infectious Diseases and Center for AIDS Research, Chapel Hill, United States, ^4^University of North Carolina at Chapel Hill School of Medicine, Department of Pathology and Lab Medicine, Chapel Hill, United States, ^5^University of Maryland, School of Pharmacy and School of Medicine, Baltimore, United States, ^6^Duke University, School of Medicine and Duke Human Vaccine Institute, Durham, United States, ^7^Leidos Biomedical Research, Inc., AIDS and Cancer Virus Program, Frederick, United States, ^8^Duke University, Department of Surgery and Department of Molecular Genetics and Microbiology, Durham, United States

**Background**: MGD014 and MGD020 are bispecific DART® molecules that bind CD3 and HIV‐1 env; anti‐env specificities are from non‐neutralizing mAbs A32 and 7B2, respectively. They redirect CD3+ T lymphocytes to kill HIV‐1‐infected CD4+ T cells. We evaluated the in vivo antiviral efficacy of MGD014 and MGD020 in HIV‐1‐infected humanized mice on antiretroviral therapy (ART). We also completed an initial phase 1 safety study of MGD014 in persons with HIV‐1 (PWH) on ART [NCT03570918].

**Methods**: MGD014 and MGD020 were administered (300 mcg/kg, QW) to HIV‐1‐infected humanized mice on ART. To assess the effects on cell‐associated HIV‐1 RNA (caRNA), tissues (spleen, liver, bone marrow, lymph node, organoid) were collected after 2 DART molecule doses. To assess the effects on rebound viremia, 7 DART molecule doses were administered; ART was discontinued after the 4^th^ DART molecule dose. The MGD014 clinical study evaluated 21 participants in Part 1 (single dose escalation, 0.1 to 300 mcg/kg) and 3 participants in Part 2 (300 mcg/kg, Q2Wx3). Endpoints included incidence of DLTs, PK, ADA, cytokines, immunophenotype, and residual plasma viremia.

**Results**: The efficacy study in HIV‐1‐infected humanized mice revealed that MGD014, MGD020, or MGD014+MGD020 [combination] reduced mean caRNA levels in tissues by 3.7‐fold (p=0.0161), 1.9‐fold (p=0.0132) or 6.2‐fold (p <0.0001), respectively (Mann‐Whitney test). Importantly, following ART discontinuation, median time to viremia rebound was 7 days for MGD014 (p=0.0444), 12 days for MGD020 (p=0.009) or 19 days for MGD014+MGD020 (p=0.0001) (Kaplan‐Meier analysis). In the MGD014 clinical study, no DLT or SAE was observed. At 300 mcg/kg, MGD014 bound an average of 92% and 72% of circulating CD4+ and CD8+ T cells, respectively, without inducing activation markers or serum cytokines. MGD014 half‐life was ∼12 days and trough serum concentrations exceeded EC_90_ for redirected CD8+ T cell killing of HIV‐infected CD4+ T cells in vitro by ≥ 20‐fold.

**Conclusions**: Administration of MGD020+MGD014 mediated greater HIV‐1 clearance activity than individual DART molecules in HIV‐1‐infected humanized mice. MGD014 was well‐tolerated in PWH on ART. A first‐in‐human study with MGD020+MGD014 in PWH on ART will begin in 2022.Our data support future clinical studies combining DART molecules and latency reversing agents. [Funded by NIAID (HHSN272201500032C) and NCI (75N91019D00024) contracts.]

### OAA0404 AZD5582 and SIV Env‐targeting rhesus monoclonal antibodies (RhmAbs) ± N‐803 in SIV‐infected ART‐suppressed rhesus macaques

A. Dashti ^1^, S. Sukkestad^1^, A.M. Horner^1^, M. Neja^1^, A. Lin^1^, N. Schoof^1^, M. Mavigner^1,2^, J.D. Lifson^3^, S.D. Falcinelli^4^, J. Sacha^5,6^, H. King^7^, R.D. Mason^7^, R.M. Dunham^4,8^, N.M. Archin^4^, J.T. Safrit^9^, D.M. Margolis^4^, M. Roederer^7^, G. Silvestri^10^, A. Chahroudi^1,2,10^

^1^Emory University, Pediatrics, Atlanta, United States, ^2^Center for Childhood Infections and Vaccines of Children's Healthcare of Atlanta and Emory University, Atlanta, United States, ^3^AIDS and Cancer Virus Program, Frederick National Laboratory for Cancer Research, Frederick, United States, ^4^UNC HIV Cure Center and Department of Medicine, University of North Carolina at Chapel Hill, Chapel Hill, United States, ^5^Vaccine & Gene Therapy Institute, Portland, United States, ^6^Oregon National Primate Research Center, Oregon Health & Science University, Portland, United States, ^7^Vaccine Research Center, National Institute of Allergy and Infectious Disease, National Institutes of Health, Bethesda, United States, ^8^HIV Drug Discovery, ViiV Healthcare, Research Triangle Park, United States, ^9^ImmunityBio, Inc., Culver City, United States, ^10^Yerkes National Primate Research Center, Emory University, Atlanta, United States

**Background**: Latency reversal and clearance of infected cells is a major strategy for HIV cure. The IAP inhibitor AZD5582 reverses latency systemically in animal models. Here, we investigated the ability of AZD5582 in combination with a cocktail of 4 rhesus‐derived SIV Env‐specific monoclonal antibodies (RhmAbs) ± the IL‐15 superagonist N‐803 to reduce viral reservoirs in ART‐suppressed rhesus macaques (RMs).

**Methods**: 30 RMs were infected with SIV_mac239_. ART was initiated 8 wks after infection. After 90 wks of ART, RMs were divided into 4 groups with continuous ART exposure: ART control (n=6), RhmAbs control (n=6), RhmAbs+AZD5582 (n=9), and RhmAbs+AZD5582+N‐803 (n=9). RhmAbs targeting V2, CD4 binding site, CD4 binding site proximal, and MPER were dosed twice at 20 mg/kg s.c. each; AZD5582 at 0.1 mg/kg i.v. wkly for 10 wks, and N‐803 at 0.1 mg/kg s.c. twice. Concentrations of RhmAbs were evaluated by ELISA. Plasma SIV RNA and CD4+ T‐cell SIV*gag* DNA/quantitative virus outgrowth were measured to quantify latency reversal and reservoir frequency, respectively.

**Results**: ART was successful in suppressing viremia. Anti‐SIV Env RhmAbs peaked in all treated RMs 24h after infusion with mean half‐life of 7.4 ± 2d. Serum concentrations were reduced for 3/4 RhmAbs in RMs who received N‐803, likely due to development of anti‐drug antibodies (ADA). Latency reversal (on‐ART viremia) was observed in 7/9 RhmAbs+AZD5582‐treated and 9/9 RhmAbs+AZD5582+N‐803‐treated RMs. At study end, RMs treated with RhmAbs+AZD5582±N‐803 had reduced splenic CD4+ T‐cell SIV*gag* DNA (p=0.03 for each comparison) and trended toward lower viral outgrowth compared to the combined ART and RhmAbs control groups. Only RMs that received RhmAbs+AZD5582 without N‐803 showed reduced lymph node CD4+ T‐cell SIV*gag* DNA (p=0.01) and virus outgrowth compared to controls (p=0.04). Finally, increased levels of CD4+ T‐cell activation/proliferation in lymph nodes following N‐803 was observed.

**Conclusions**: Our findings give insight into HIV cure interventions in a relevant preclinical model, confirm the efficacy of AZD5582 in inducing SIV reactivation, and provide new *in vivo* data showing latency reversal in all RMs treated with the combination of AZD5582+N‐803. This study also suggests that elimination of infected cells in tissues using an IAP inhibitor and anti‐SIV Env RhmAb cocktail is possible.

### OAA0405 Temsavir treatment of HIV‐1‐infected cells decreases envelope glycoproteins recognition by broadly‐neutralizing antibodies

M. Boutin ^1,2^, D. Vézina^1^, S. Ding^1^, J. Prévost^1,3^, A. Laumaea^1,2^, L. Marchitto^1,2^, S.P. Anand^4^, H. Medjahed^1^, G. Gendron‐Lepage^1^, C. Bourassa^1^, G. Goyette^1^, A. Clark^5^, J. Richard^1,2^, A. Finzi^1,2,4^

^1^Centre de Recherche du Centre Hospitalier de l'Université de Montréal (CRCHUM), Fundamental Research, Montréal, Canada, ^2^Université de Montréal, Microbiologie, Infectiologie et Immunologie, Montréal, Canada, ^3^Université de Montréal, Microbiology et immunology, Montréal, Canada, ^4^McGill University, Microbiology and Immunology, Montreal, Canada, ^5^ViiV Healthcare, Global Medical Affairs, Brentford, United Kingdom

**Background**: The heavily glycosylated HIV‐1 envelope glycoprotein (Env) is the sole viral antigen present on virion and infected cells, representing the main target for antibody responses. The FDA‐approved small molecule temsavir acts as an HIV‐1 attachment inhibitor by preventing Env‐CD4 interaction. This molecule also stabilizes Env in a prefusion “closed” conformation that is preferentially targeted by several broadly neutralizing antibodies (bNAbs). A recent study showed that an analog of temsavir (BMS‐377806), affects the cleavage and addition of complex glycans on Env. In this study, we investigated the impact of temsavir on the overall glycosylation, proteolytic cleavage, cell‐surface expression and antigenicity of Env.

**Methods**: SinceBMS‐377806limits Env conformational flexibility, we first investigated temsavir's impacton overall Env processing and glycosylation by immunoprecipitation. Next, we evaluated if this alteration affected the recognition of Env at the surface of infected cells and virionsby a panel of bNAbs and non‐neutralizing antibodies (nnAbs).Finally, we investigated ifthe capacity of bNAbs to eliminate infected cells by ADCC was also affected.

**Results**: We found that temsavir substantially impacts Env glycosylation and processing at physiological concentrations. This significantly alters the capacity of several bNAbs to recognize Env present on virions and HIV‐1‐infected cells. Temsavir treatment also reduces the capacity of bNAbs to eliminate HIV‐1‐infected cells by ADCC.

**Conclusions**: Temsavir has a profound impact on Env antigenicity at the surface of viral particles and infected cells.This new information needs to be considered for the development of new antibody‐based approaches in temsavir‐treated individuals.

### OAB0102 Chronic injection opioid use increases the systemic inflammation and T cell immune activation in virally suppressed people living with HIV

C. Dang ^1^, S. Pallikkuth^1^, A. Kizhner^1^, C.M. Nelson^1^, D. Forrest^1^, D. Feaster^1^, R. Pahwa^1^, D. Jayaweera^1^, A. Rodriguez^1^, H. Tookes^1^, S. Pahwa^1^

^1^University of Miami, Miami, United States

**Background**: Opioid dependence is a major health problem in the US. Our group is investigating the immunologic consequences of opioid dependence in HIV‐infected (HIV+) and HIV‐uninfected (HIV‐) participants. We hypothesize that virally controlled HIV+ injecting opioid users manifest immune perturbations.

**Methods**: In an ongoing study, opioid users (OP+) are recruited from our needle exchange program and opioid non‐users (OP‐) from the clinics. Participants are grouped as HIV+OP+ (Gp1), HIV‐OP+ (Gp2), HIV+OP‐ (Gp3), and HIV‐OP‐ (Gp4), median age 48yr with 62% Male: 36% Female: and 2% Transgender Women. HIV+ were on ART with <HIV 200 copies/mL. Statistical methods include non‐parametric group comparisons (Kruskal‐Wallis), Spearman correlations and Multiple regression. (Table 1)

**Results**: Plasma biomarkers showed differences in OP+ (Gp1 and Gp2) compared to Gp4 (HIV‐OP‐), with higher soluble activation markers (sTNFR‐I, ‐II), inflammatory biomarkers (TNFa, IL‐6, sCD25), adhesion mediators (ICAM‐1, VCAM‐1), monocyte chemotaxis protein (CCL2) and monocyte activation (sCD14). Cytokine scores for OP+ were higher than the OP‐ groups, and among the OP‐ groups, HIV+ (Gp3) were higher than HIV‐ (Gp4). In a regression model that included gender, race, ethnicity, and age, cytokine scores differed by HIV status (HIV+:higher scores, p<0.002) and opioid use (OP+:higher scores, p<0.0000001) and moderated relationship by race, p<0.02. T cell phenotype (flow cytometry) revealed inverted CD4/CD8 ratio. Immune activation (IA), measured as frequencies of HLA‐DR+CD38+ T cells, was significantly higher in the CD8 compartment in OP+ groups, compared to Gp4. CD8 T cell IA positively associated with cytokine score (rho= 0.5, p<0.0001).

**Conclusions**: Chronic injection opioid use (Gp1 and Gp2) increases systemic inflammation and T cell immune activation. Contribution of virally controlled HIV status was less striking and limited to cytokines. Exacerbation of immune dysfunction by opioids in the syndemic of HIV and chronic injection opioid use may pose a risk for comorbidities in this population.

**Figure:**
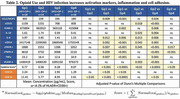
**Abstract OAB0102‐Table 1**.

### OAB0103 Impact of gender identity‐ and HIV‐related stigmas and psychosocial resources on mental health and alcohol use among transgender women newly diagnosed with HIV in India: a longitudinal cohort study

N.S. Chopra^1^, D. Datta^1^, A. Gupta^2^, F. Gulfam^2^, J. Kaur^2^, J. Ross^1^, S. Reddy^2^, A. Ahalawat^2^, S. Safren^3^, S. Golub^4^, V. Chakrapani^2^, V.V. Patel ^1^

^1^Albert Einstein College of Medicine, Division of General Internal Medicine, The Bronx, United States, ^2^HIV/AIDS Alliance India, New Delhi, India, ^3^University of Miami, Psychology, Coral Gables, United States, ^4^Hunter College, CUNY, Health Psychology, New York, United States

**Background**: Transgender women (TGW) living with HIV may be subject to intersectional stigma at multiple levels – enacted (experiences of discrimination/devaluation); anticipated (expectations of discrimination); internalized (negative self‐valuation due to stigmatized status) – that are associated with negative health outcomes, as posited by the health stigma framework. However, few studies have examined the association between stigma and mental health or hazardous alcohol use among TGW living with HIV. We investigated the longitudinal influence of multiple stigmas on depression, anxiety, and alcohol use in TGW living with HIV in India.

**Methods**: We enrolled a prospective cohort of 140 TGW newly diagnosed with HIV from 11 Indian states. Participants completed surveys via telephone‐interviews at three timepoints – baseline, three‐months, and six‐months (Aug. ‘20 ‐ Oct. ‘21) – about enacted, anticipated, and internalized stigmas related to positive HIV status and transgender identity, as well as: gender affirmation in healthcare settings, social support, and resillency resources. Surveys also captured depression (PHQ‐9), generalized anxiety (GAD‐2), and hazardous alcohol use (AUDIT‐C) scores. Longitudinal analyses used multivariable regression with generalized estimating equations (which account for clustering within individuals and provide robust population‐averaged estimates of the outcomes) for each of the three outcomes (results reported as β [95% CI]).

**Results**: Increasing anticipated HIV‐stigma (3.2, [1.7,4.7], p<.001), enacted HIV‐stigma (3.5, (2, 5.1), p<.001), anticipated TG‐stigma (1.7 [.4, 3], p=.01), internalized TG‐stigma (3.1 [1.2,4.9], p=.001), and hazardous alcohol use scores (0.1 [0.03,0.2], p=.006) were associated with increased depression; increasing resiliency resources (‐0.3 [−0.5,‐0.1], p=.002) and gender affirmation in healthcare (‐0.08 [−0.2,‐0.004], p=.039) was associated with decreased depression. Increasing enacted HIV‐stigma (0.9 [0.5,1.3], p<.001) and internalized HIV‐stigma (2.1 [1.4,2.8], p<.001) were associated with increased generalized anxiety; increasing gender affirmation(‐0.2 [−0.3,‐0.1], p<.001), social support(‐0.8 [−1.3,‐0.3], p=.001.) was associated with decreased generalized anxiety. Finally, increasing gender affirmation in healthcare‐settings was associated with lower hazardous alcohol use scores (‐.1 [−0.16,‐0.004], p=.04).

**Conclusions**: Transgender identity‐ and HIV‐specific stigmas strongly impacted depression and anxiety. Increasing levels of psychosocial resources including gender affirmation in healthcare‐settings were associated with improved mental health and reduced alcohol use scores. These findings provide empirical evidence for intervention targets for addressing the well‐being of TGW newly diagnosed with HIV.

### OAB0104 Forty‐eight week outcomes of a combined cognitive behavioral therapy and medication management algorithm for treatment of depression among youth living with HIV in the United States (IMPAACT 2002)

L. Brown ^1^, K. Baltrusaitis^2^, B. Kennard^3^, G. Emslie^3^, M. Chernoff^2^, S. Buisson^4^, L. Levy^4^, L. Whiteley^5^, S. Traite^2^, C. Krotje^6^, K. Knowles^6^, E. Townley^7^, J. Deville^8^, M. Wilkins^9^, D. Reirden^10^, M. Paul^11^, C. Beneri^12^, D. Shapiro^2^

^1^Brown University; Rhode Island Hospital, Psychiatry, Providence, United States, ^2^Harvard T.H. Chan School of Public Health, Center for Biostatistics in AIDS Research, Boston, United States, ^3^University of Texas Southwestern Medical Center, Psychiatry, Dallas, United States, ^4^FHI 360, Durham, United States, ^5^Brown University, Psychiatry, Providence, United States, ^6^Frontier Science Foundation, Amherst, United States, ^7^National Institute of Allergy and Infectious Diseases, Rockville, United States, ^8^University of California, David Geffen School of Medicine, Los Angeles, United States, ^9^St. Jude Children's Research Hospital, Memphis, United States, ^10^University of Colorado School of Medicine, Children's Hospital Colorado, Aurora, United States, ^11^Baylor College of Medicine, Texas Children's Hospital, Houston, United States, ^12^Stony Brook Children's Hospital, Stony Brook, United States

**Background**: Studies suggest that manualized depression treatmentguided by symptom measurementis more efficacious than usual care but that its impact can wane. Our study among youth living with HIV (YLWH), ages 12‐24 years at United States clinical research sites in the International Maternal Pediatric Adolescent AIDS Clinical Trials Network (IMPAACT), found a significant reduction in depressive symptoms at Week 24 among YLWH who received a manualized, measurement‐guided intervention (Brown, L. JAIDS, 2021). This abstract reports outcomes at Weeks 36 and 48, after the study's intervention ended.

**Methods**: Eligibility included diagnosis of nonpsychotic depression and current depressive symptoms. Using restricted randomization, sites were assigned to either combination cognitive behavioral therapy and medication management algorithm (COMB‐R) tailored for YLWH or Enhanced Standard of Care (ESC), which provided standard psychotherapy and medication management. Site‐level mean Quick Inventory for Depression Symptomatology Self‐Report (QIDS‐SR) scores and proportion of youth with treatment response (>50% decrease from baseline) and remission (QIDS‐SR£5) were compared across arms using t‐tests.

**Results**: Thirteen sites enrolled 156 YLWH, with baseline demographic factors, depression severity, and HIV status comparable across arms. At Weeks 36 and 48, the mean proportion of youth with a treatment responsewas greaterat COMB‐R sites compared to ESC sites (52.0% vs. 18.8%, p=0.015; 58.7% vs. 33.4%, p=0.047). At Week 36,a greater mean proportion of youth at COMB‐R sites reported remission(37.9%vs. 19.4%, p=0.05), and the mean QIDS‐SR was lower (7.45 vs. 9.75, p=0.05). At Week 48,the COMB‐R improvement was generally maintained, while ESC continued to improve,with differences no longer significant (remission:43.7% vs. 27.5%, p=0.24; QIDS‐SR:7.09 vs. 9.08, p=0.14).

**Conclusions**: The evidence for maintenance of the intervention's impact after the intervention concluded suggests promise. The intervention is feasible in clinic settings with existing staff and can be implemented without increasing the burden of additional clinic visits for patients. Future research can investigate how to better prevent relapse for those who achieve remission and promote a treatment response for those whom treatment is not effective in the first 24 weeks.

### OAB0105 Long‐term risk of hospitalization and chronic disease among children who were HIV‐exposed uninfected (cHEU) compared to population controls in Montreal, Canada

B. Jeanne ^1^, T. Ducruet^1^, S. Taillefer^1^, M.E. Metras^1^, S. Valois^1^, V. Lamarre^1^, I. Boucoiran^1^, H. Soudeyns^1^, J. Puyat^2^, J. Singer^2^, F. Kakkar^1^

^1^CHU Sainte Justine, Université de Montréal, Montréal, Canada, ^2^CIHR Canadian HIV Clinical trials Network, Vancouver, Canada

**Background**: While studies have demonstrated increased risk of morbidity and mortality among cHEU in early life, the need for specialized follow‐up of cHEU once their HIV status has been confirmed negative is not clear. The primary objective of this study was to determine the long‐term risk of hospitalization, and incidence of chronic disease, among cHEU compared to HIV‐unexposed uninfected (cHUU) controls.

**Methods**: Longitudinal cohort study linking data from the Centre Maternel et Infantile sur le SIDA (CMIS) cohort to administrative data from the Régie de l'Assurance Maladie du Quebec (RAMQ), a universal health system with unique single patient identifiers, covering all care services provided. ICD‐9 codes were extracted and grouped by system for measures of chronic diseases and cHEU matched 1:3 by age, gender and postal code to cHUU controls. Survival analysis was used to determine risk of hospitalization and chronic disease.

**Results**: Among 847 cHEU enrolled in the CMIS cohort between 1988 and 2015, 726 were linked to the RAMQ database, and matched to 2178 cHUU. Median follow‐up was 11.1 [6.6 – 15.9] years. There was a significantly higher risk of hospitalization among cHEU *vs*. cHUU over their life‐span (HR 1.42 [1.26 – 1.61], p<0.001), which remained significant after adjusting for gestational age (aHR 1.23 [1.08 – 1.40], p=0.001), and in a sensitivity analysis excluding prolonged (>5 days) birth hospitalization (HR 1.21 [1.06 – 1.41] (Figure 1). Over their lifespan, cHEU had a significantly higher risk of neuropsychiatric disorders (33.3% *vs*. 26.1%, RR 1.28 [1.13 – 1.45], p<0.001) and congenital anomalies (5.5% *vs*. 3.5%, RR 1.58 [1.09 – 2.3], p=0.016), though there was no difference in risk of chronic cardiovascular, respiratory or neoplastic disease.

**Figure:**
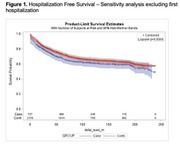
**Abstract OAB0105‐Figure 1**.

**Conclusions**: In this resource‐rich setting with universal health‐care, cHEU had increased risk of hospitalization and neuro‐psychiatric disorders, suggesting that cHEU would benefit from enhanced pediatric care, including early neurodevelopmental assessment.

### OAB0202 The fast and the continuous: dolutegravir‐based antiretroviral therapy achieves impressive viral load suppression in CALHIV in the short‐ and long‐term

J. Bacha ^1,2,3^, S. Dlamini^2,4^, F. Anabwani^4^, J. Gwimile^5^, J.B. Kanywa^6^, P.J. Elyanu^6^, J. Farirai^7^, M. Bvumbwe^8^, M. Tsotako^9^, T. Steffy^1,2,9^, D. Nguywen^1,2,10^, H. Haq^1,2^

^1^Baylor College of Medicine, Pediatrics, Houston, United States, ^2^Baylor College of Medicine International Pediatric AIDS Initiative (BIPAI) at Texas Children's Hospital, Houston, United States, ^3^Baylor College of Medicine Children's Foundation ‐ Tanzania, Mbeya, Tanzania, The United Republic of, ^4^Baylor College of Medicine Children's Foundation ‐ Eswatini, Mbabane, Eswatini, ^5^Baylor College of Medicine Children's Foundation ‐ Tanzania, Mwanza, Tanzania, The United Republic of, ^6^Baylor College of Medicine Children's Foundation ‐ Uganda, Kampala, Uganda, ^7^Botswana‐Baylor Children's Clinical Centre of Excellence Trust, Gabarone, Botswana, ^8^Baylor College of Medicine Children's Foundation ‐ Malawi, Lilongwe, Malawi, ^9^Baylor College of Medicine Children's Foundation ‐ Lesotho, Maseru, Lesotho, ^10^Baylor College of Medicine, Department of Education, Innovation, and Technology, Houston, United States

**Background**: As dolutegravir (DTG)‐based antiretroviral therapy (ART) is used as the preferred ART for children and adolescents living with HIV (CALHIV), questions remain about its short‐ and long‐term effectiveness in the real‐world. We describe longitudinal viral load suppression (VLS) trends of CALHIV using DTG in six African countries.

**Methods**: Retrospective chart review from 2016 through 2021 analyzing clinical characteristics and VLS rates of CALHIV ages 0‐19 years old prescribed DTG at clinics in Botswana, Eswatini, Lesotho, Malawi, Tanzania, and Uganda. VLS was defined as VL<1000 copies/mL. VL results were coded at 6 month intervals from DTG start date, with +/‐ 90 day buffer range. Initial VLS rate at 6 month post‐DTG was used as the comparator for the longitudinal VLS rates and differences.

**Results**: 11,799 CALHIV received DTG; 56.0% (6604/11799) were female and average age of 13.4 years (SD 4.0 yr). By study end, 93.7% (11059/11799) remained active in care, 4.7% (549/11799) transferred out, 1.2% (145/11799) were lost to follow up, and 0.4% (46/11799) died. 22,577 VL results were documented, ranging from 6 months to 60 months post‐DTG and average follow up time post‐DTG was 22.4 months (SD 12.4). Initial 6 month post‐DTG VLS rate was 92.1%, similar across sexes (Females 91.6%, Males 92.7%) and ages (Figure 1). VLS rates were maintained without significant loss across the entire cohort, by sex and by age groups (Figure 2).

**Figure:**
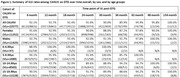
**Abstract OAB0202‐Figure 1**.

**Conclusions**: DTG rapidly achieved and consistently maintained VLS for years among CALHIV in real world settings. These findings support the widespread use of DTG in CALHIV.

**Figure:**
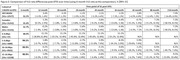
**Abstract OAB0202‐Figure 2**.

### OAB0203 Optimal timing for ART initiation in ART‐naive children and adolescents living with HIV following the diagnosis of HIV‐associated TB

A. Kay ^1,2^, J. Mendez Reyes^1^, T. Devezin^1^, S. Dlamini^2^, A. Msekandiana^3^, T. Steffy^4,5^, J. Bacha^6,1^, P. Amuge^7^, B. Lukhele^2,5^, H.L. Kirchner^8,1^, A. Mandalakas^1^

^1^Baylor College of Medicine, Pediatrics, Global TB Program, Houston, United States, ^2^Baylor Children's Foundation Eswatini, Mbabane, Eswatini, ^3^Baylor Children's Foundation Malawi, Lilongwe, Malawi, ^4^Baylor Children's Foundation Lesotho, Maseru, Lesotho, ^5^Baylor College of Medicine, Pediatrics, Houston, United States, ^6^Baylor Children's Foundation Tanzania, Mbeya, Tanzania, The United Republic of, ^7^Baylor Children's Foundation Uganda, Kampala, Uganda, ^8^Geisinger Health System, Danville, United States

**Background**: There is no direct evidence in children and adolescents living with HIV (CALHIV) to guide the timing of antiretroviral treatment (ART) initiation following TB treatment. To address this gap, we evaluated the risk of mortality associated with the timing of ART initiation in ART‐naive CALHIV treated for TB in a large, multinational retrospective cohort from high‐burden settings throughout sub‐Saharan Africa.

**Methods**: Data was extracted from electronic medical records of ART‐naïve patients, aged 0‐19 years treated for HIV‐associated TB at a Baylor Center of Excellence in Botswana, Eswatini, Malawi, Lesotho, Tanzania, or Uganda between 2014 and 2020. Data was analyzed against a primary outcome of all‐cause mortality with Cox proportional hazard models. The models included time of ART initiation as a time dependent variable to limit immortal time bias and were adjusted for age, body mass index, immune status, clinical site, and location of TB disease.

**Results**: The study population included 731 CALHIV with variable timing of ART initiation following TB treatment initiation: ART started within two weeks of TB treatment (n=260), ART started two weeks after but in less than two months of TB treatment (n=379), ART started after two months of TB treatment (n=64) and no ART (n=28). Adjusted Cox proportional hazard models demonstrated an increased risk of mortality by year three from TB treatment initiation in children never started on ART compared to children initiating ART between two weeks and two months from TB treatment; however, the risk of mortality did not differ in the less than two week group (((adjusted Hazard Ratio (aHR)) 0.93; 95% CI 0.30 to 2.84) or in the greater than two month group (aHR 1.87; 95% CI 0.22 to 15.96) as compared to ART initiation between two weeks and two months from TB treatment.

**Conclusions**: Our study demonstrates no increased risk of mortality in CALHIV initiating ART less than two‐weeks from TB treatment initiation. Given the broad health benefits of ART, this evidence supports the World Health Organization recommendation for CALHIV to initiate ART within two‐weeks of initiating TB treatment.

### OAB0204 Subcortical brain volumes of children who are HIV‐exposed and uninfected in the first three years of life: a South African birth cohort study

C.J. Wedderburn ^1,2,3^, S. Yeung^2^, N.A. Groenewold^1^, A.M. Rehman^2^, S. Subramoney^1^, J.‐P. Fouche^1,4^, S.H. Joshi^5^, K.L. Narr^5^, N. Hoffman^1^, A. Roos^1,4^, D.M. Gibb^3^, H.J. Zar^1^, D.J. Stein^1^, K.A. Donald^1^

^1^University of Cape Town, Cape Town, South Africa, ^2^London School of Hygiene & Tropical Medicine, London, United Kingdom, ^3^MRC Clinical Trials Unit at University College London, London, United Kingdom, ^4^Stellenbosch University, Stellenbosch, South Africa, ^5^University of California Los Angeles, Los Angeles, United States

**Background**: Children who are HIV‐exposed and uninfected (CHEU) are at risk for early neurodevelopmental impairment. Neuroimaging studies are scarce, although recently we reported smaller volumes of basal ganglia structures in CHEU compared to children who are HIV‐unexposed (CHU) at 2‐6 weeks of age. However, no studies have examined subcortical brain volumes through childhood. We aimed to investigate whether the effects of *in utero* exposure to HIV/ART on subcortical brain structures detected in infancy are evident at age 3 years.

**Methods**: The Drakenstein Child Health Study is a population‐based birth cohort in South Africa. Pregnant women were enrolled in the second trimester of pregnancy and mother‐child pairs received HIV testing per local guidelines; all mothers with HIV were initiated on ART. A subgroup of children had magnetic resonance imaging (MRI) aged 2‐3 years. Structural T1‐weighted images were acquired on a 3T Siemens MRI scanner during natural sleep and images were processed using FreeSurfer software, blinded to HIV status. Bilateral subcortical volumes were extracted from segmented images, and CHEU and CHU groups were compared using multivariable linear regression.

**Results**: One hundred and sixty‐two children (70 CHEU; 92 CHU) (mean age 34 months; 58% male) had high resolution scans. Socioeconomic characteristics were similar between groups. CHEU had lower overall putamen volume compared to CHU (4381 mm^3^ versus 4597 mm^3^, p=0.016, Cohen's d −0.37 [−0.69 to −0.06]), with similar reductions when analysed by hemisphere (left p=0.023; right p=0.018). The findings held after adjusting for multiple covariates including age, sex, socioeconomic status and intracranial volume. Compared to CHU, CHEU also had lower hippocampus volume (3043 mm^3^ versus 3149 mm^3^, p=0.046, Cohen's d −0.31 [−0.62 to 0.00]). The left hemisphere difference persisted after adjusting for covariates (p=0.038).

**Conclusions**: Altered morphometry in the basal ganglia region can be detected in CHEU across the first 3 years of life. Further, CHEU showed smaller volumes of the hippocampus at 3 years, a region known to be vulnerable to early‐life exposures. These findings suggest that *in utero* HIV/ART exposure may affect early subcortical brain development with enduring impact. Follow up studies are needed to determine the underlying mechanisms and long‐term trajectories.

### OAB0205 Probability of AIDS and non‐AIDS‐related mortality of early‐treated children living with HIV‐1

C. Bréhin ^1^, A. Tagarro^2^, S.D.‐R. Domínguez‐Rodríguez^2^, L. Kuhn^3^, T. Nhampossa^4^, K. Otwombe^5^, A. Janse van Rensburg^6^, N. Klein^7^, M.G. Lain^8^, A.I. Maiga^9^, C. Giaquinto^10^, P. Rossi^11^, P. Rojo^2^, EPIICAL Consortium

^1^Toulouse University Hospital, Toulouse, France, ^2^Fundación para la Investigación Biomédica del Hospital Universitario 12 de Octubre, Madrid, Spain, ^3^Gertrude H. Sergievsky Center, College of Physicians and Surgeons, Columbia University Irving Medical Center, New York, United States, ^4^Centro de Investigação em Saúde de Manhiça, Maputo, Mozambique, ^5^School of Public Health, Faculty of Health Sciences, University of the Witwatersrand, Johannesburg, South Africa, ^6^Tygerberg Children's Hospital and Family Clinical Research Unit, Stellenbosch University, Cape Town, South Africa, ^7^Infection, Immunity and Inflammation Programme, UCL Great Ormond Street Institute of Child Health, London, United Kingdom, ^8^Fundação Ariel Glaser Contra O Sida Pediátrico, Maputo, Mozambique, ^9^SEREFO (HIV/TB Research and Training CenteHIV/TB Research and Training Center, FMOS, University of STT, Bamako, Mali, ^10^PENTA Onlus, Padova, Italy, ^11^Bambino Gesù Children's Hospital, Rome, Italy

**Background**: The mortality of early treated children born with HIV in Sub‐Saharan Africa during first years of life is still higher than baseline. We assessed the probability of AIDS‐related mortality of a cohort of early treated children born with HIV, and factors related to them.

**Methods**: EARTH‐EPIICAL Cohort is underway in Mozambique, Mali, and South Africa. From May 1^st^, 2018 to May 1^st^, 2021, infants with HIV who started ART in the first 3 months of life, are followed up for 24 months. To describe the probability of AIDS‐related death, a competing risk joint model was performed consisting of a multivariable mixed linear model for longitudinal CD4 trajectory and a survival Cox proportional model. The model was adjusted by ART initiation regimen, age at HIV diagnosis, and weight‐for‐age.

**Results**: 212 participants were enrolled and followed during a median time of 17 [6.8;27.5] months; 84 reached 2 years of follow‐up. ART started at 34 [26;74] days of life.

23 patients (10.8%) died, at a median of 2.5 [0.6;6.8] months of age; 12 due to AIDS‐related causes . At 2 years, overall probability (P) of death was 12% (CI95%,7 to 17). The excess of mortality compared to baseline mortality was 7%; the excess of mortality due to AIDS‐related causes was 5.7%, and due to non‐AIDS related causes was 1.4%. According to the joint model, there was an inverse statistically significant association between the probability of AIDS‐related mortality and the percentage of CD4 (%CD4) during the time to follow up and (HR:0.9 [CI95%, 0.86‐0.98], p=0.046). An increase in CD4 count decrease a 10% the probability of AIDS‐related mortality. Notably, these estimates had opposite sign for the non‐AIDS‐attributable deaths (HR:1.09 [CI95%, 1.02‐1.15], p=0.003). Interestingly, baseline VL was significantly associated with non‐AIDS‐related mortality (HR:4.34 (CI95%, 1.84‐20.7), p=0.026).

**Conclusions**: Despite early treatment, excess of AIDS‐ and non‐AIDS‐related mortality remains high in children living with HIV‐1. Differentiating AIDS and non‐AIDS related mortality in children with HIV may allow us to understand better the risk factors associated with mortality. CD4 percentage changes over time, and it impacts the probability of death. Infants with high baseline VL and low CD4% require specific attention.

### OAB0302 Efficacy and safety of dolutegravir plusemtricitabinevs combined antiretroviral therapy for the maintenance of viral suppression: 144‐week results of the SIMPL'HIV trial

A. Marinosci ^1^, D. Sculier^2^, G. Wandeler^3^, S. Yerly^4^, M. Stoeckle^5^, E. Bernasconi^6^, D.L. Braun^7^, P. Vernazza^8^, M. Cavassini^9^, M. Buzzi^10^, L. Decosterd^9^, P. Schmid^8^, M. Branca^11^, A. Limacher^11^, M. Egger^12^, H.F. Günthard^13^, K.J. Metzner^14^, A. Calmy^4^

^1^Hôpitaux Universitaires de Genève, Infectious Diseases, Geneva, Switzerland, ^2^Private office, Geneva, Switzerland, ^3^Inselspital, Bern, Switzerland, ^4^Hôpitaux Universitaires de Genève, Geneva, Switzerland, ^5^University Hospital Basel, Basel, Switzerland, ^6^Ospedale Regionale Lugano, Lugano, Switzerland, ^7^University Hospital Zurich, Zurich, Switzerland, ^8^Kantonsspital St. Gallen, St. Gallen, Switzerland, ^9^Centre hospitalier universitaire vaudois, Lausanne, Switzerland, ^10^Private Office, Geneva, Switzerland, ^11^CTU, Bern, Switzerland, ^12^University of Bern, Bern, Switzerland, ^13^University Hospital of Zurich, Zurich, Switzerland, ^14^University Hospital of Zuirch, Zurich, Switzerland

**Background**: Simplified treatment is needed to address challenges associated with daily oral HIV treatment in people living with HIV.

**Methods**: SIMPL'HIV is a phase 3, randomized, open‐label, multicentre, factorial study conducted among HIV‐1 infected adults on cART in Switzerland. At baseline, patients with HIV‐RNA <50 copies/ml for at least 24 weeks were randomised to switching to dolutegravir (DTG) plus emtricitabine (FTC) or continuing standard combined antiretroviral therapy (cART), and to a reduced biological and medical surveillance *vs* continuation of standard 3‐monthly monitoring. The main endpoint was the proportion of participants maintaining HIV‐1 RNA <100 copies/mL throughout 144 weeks.

**Results**: 93 participants were randomly assigned to DTG+FTC and 94 to cART (mean nadir CD4 count, 259 cells/mm3 [SD=187]; 17%, female). Through 144 weeks, 3 participants in the DTG+FTC group and 6 in the cART group had HIV‐RNA levels >100 copies/ml with an adjusted difference of −3.1% (95% CI −9.2‐3.1%) in the intention‐to‐treat population. At week 144 HIV‐RNA was >50 copies/ml in one patient in the DTG+FTC group and four patients in the cART group (adjusted difference −3.2%; 95%‐CI −7.7‐1.5%). Mean CD4 gain between baseline and week 144 was 8.4 (±195.3) and 34.7 (±193.6) cell/mm^3^ with DTG+FTC and cART, respectively (adjusted difference −19.4; 95% CI −74.2‐35.4). Twelve (12.9%) participants in the DTF+FTC group and 15 (18.1%) in the cART group presented with serious AEs through week 144. Adjusted average weight gain between baseline and 144 weeks was 2.4 (±4.7) *vs* 2.3 (±4.1) kg with DTG+FTC and cART, respectively; participants with 10% or more weight gain were 7.5% and 6.4% in the DTG+FTC and cART groups, respectively.

**Figure:**
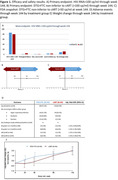
**Abstract OAB0302‐Figure 1**.

**Conclusions**: DTG+FTC remains safe and non‐inferior to standard care in the maintenance of viral suppression in adults with HIV‐1 at 144 weeks. No new safety signals were observed and change weight was similar between groups.

### OAB0303 Dual therapy based on DRVr plus 3TC in HIV‐1 naïve patients: global 48 week results from ANDES Study

M.I. Figueroa ^1^, O. Sued^1^, P. Patterson^1^, D. Cecchini^2^, M. Sanchez^3^, M.J. Rolon^4^, G. Lopardo^5^, M. Ceschel^1^, G. Mernies^1^, M. Destefano^1^, A. Gun^6^, V. Fink^1^, Z. Ortiz^7^, P. Cahn^7^, on behalf ANDES Study Group

^1^Fundacion Huesped, Research Department, Ciudad Autónoma de Buenos Aires, Argentina, ^2^Hospital Argerich, Infectious Disease Unit, Ciudad de Buenos Aires, Argentina, ^3^Hospital Italiano, Infectious Disease Service, Ciudad de Buenos Aires, Argentina, ^4^Hospital Juan A Fernandez, Infectious disease Division, Ciudad de Buenos Aires, Argentina, ^5^Centro de Estudios Infectologicos SA (CTD Stamboulian), Research Department, Ciudad de Buenos Aires, Argentina, ^6^Fundacion Huesped, Laboratory Department, Ciudad Autónoma de Buenos Aires, Argentina, ^7^Fundacion Huesped, Research Direction, Ciudad Autónoma de Buenos Aires, Argentina

**Background**: Dual therapy has been explored in different drug combination. A generic fixed dose combination(FDC)DRV/r 800/100 mg is available in Argentina. We designed a study to compare efficacy(non‐inferiority)and safety of this FDC plus 3TC to standard‐of care antiretroviral therapy based on the same drugs plus tenofovir.ClinicalTrials.gov:NCT02770508

**Methods**: ANDES is a randomized, open‐label, phase IV study designed to compare dual therapy(DT)with DRV/r 800/100mg FDC plus 3TC(300mg)vs. triple therapy(TT) with DRV/r plus 3TC/TDF(300/300mg)in treatment‐naïve HIV‐1 infected patients.Primary endpoint: proportion of patients with HIV viral load(pVL) <50 copies/mL at week 48(FDA snapshot‐ITTe analysis).The study was carried out in two steps as pre‐planned protocol study design.First step results were presented at CROI 2018.Final results (first step/second step) at week 48 are presented here.

**Results**: Out of 395 patients screened,336 were randomized to receive:DT(n:171) or TT(n:165).Demographic characteristic were similar between arms:94% CDC stage A, 90% males, 23% pVL>100,000 copies/mL.Median (IQR) HIV pVL log: 4.5 (4.1‐5.0), Median (IQR) CD4 T‐cell/uL: 415 (300‐599). At week 48, 92.7% on TT vs 90.6% on DT achieved pVL<50 copies/mL,difference (95%CI): −2.1%(‐7.0; 2.9%). Patients with baseline pVL>100,000 copies/mL showed 90.3% response in TT vs. 86.7% in DT, difference (95%CI):‐3.7% (‐15.7;8.4%).Per‐protocol population analysis:98.7% were responder in TT vs. 98.1% in DT,difference (95%CI):‐0.6% (‐2.9;1.7%).Seven patients had Protocol‐Defined virologic failure:2 at week 24(1 TT/1 DT) and 5 at week 48 (2 TT/ 3 DT). Mean pVL at week 48 failure:73.8 copies/mL.Mean CD4+ change from baseline:TT:+238.1 cells/uL, DT:+275.3 cells/mm3 (p:0.4).Fifty‐nine grade 2‐3 related adverse events (AEs) were reported among 53 patients(TT:33 / DT:20, p:0,04). Most frequent were:rash (TT:5%; DT: 6%; p:0.70), diarrhea (TT: 5%; DT: 1%; p:0.06**),abdominal pain (TT: 5%; DT: 1%; p:0.03). AEs leading to discontinuation were rare (TT:2, DT:2).Two possible treatment‐related SAEs were reported (both lipase increase G4)in TT arm.Laboratory changes from baseline to week 48 were similar except to lipid profile with higher change in DT arm:total cholesterol (TT:8%;DT:20%;p<0.01) ,LDL‐cholesterol (TT:8%;DT:19%;p:0.01),Triglycerides(TT:38%;DT:56%;p=0.05).

**Conclusions**: A generic FDC of DRV/r plus 3TC showed non‐inferiority to standard TT with DRV/r plus TDF/3TC at 48 weeks in both ITTe and per‐protocol populations.The DT strategy was safe and well tolerated and could be considered as an alternative option for treatment naïve population.

### OAB0304 Dolutegravir versus efavirenz‐400 as first‐line ART in Cameroon: week 192 data of NAMSAL trial

M. Mpoudi‐Etame ^1^, T. Tovar Sanchez^2^, P. Omgba Bassega^3^, J. Olinga^4^, R. Pelloquin^2^, M. Foalem^4^, C. Njonkep^4^, E. Mimbé^4^, M. Varloteaux^4^, M. Tongo^5^, N. Lamare^5^, M. Peeters^2^, J. Reynes^6,2^, E. Delaporte^6,2^, S. Koulla‐Shiro^4^, C. Kouanfack^7,8,4^, NAMSAL ANRS 12313 study group

^1^Military Hospital of Yaoundé, Yaoundé, Cameroon, ^2^TransVIHMI, University of Montpellier‐IRD‐INSERM, Montpellier, France, ^3^District of the Cité Verte Hospital, Yaoundé, Cameroon, ^4^Cameroon ANRS‐site, Yaoundé, Cameroon, ^5^CREMER, Yaoundé, Cameroon, ^6^Montpellier University Hospital Center, Montpellier, France, ^7^Faculty of Medicine and Pharmaceutical Sciences University of Dschang, Dschang, Cameroon, ^8^Yaoundé Central Hospital, Yaoundé, Cameroon

**Background**: The NAMSAL main objective was the comparison of two first‐line antiretroviral treatments (ARTs) in real‐life conditions in low‐ and middle‐income countries (LMIC): dolutegravir 50 mg (DTG) and low‐dose of efavirenz (ie 400 mg; EFV400) daily both combined with tenofovir‐disoproxil‐fumarate [TDF]/lamivudine [3TC]. The 48‐week outcomes provided decision‐making elements to World Health Organization (WHO) to recommend DTG as first‐line ART in 2019. Outcomes were confirmed at 96 weeks. We assessed long‐term efficacy and safety of these two regimens.

**Methods**: NAMSAL was an open‐label, multicenter, randomized, phase 3 non inferiority trial conducted in Cameroon over 96‐week, extended as post‐trial follow‐up as a prospective cohort until 192‐week. HIV‐1 infected ARV‐naive adults with HIV‐RNA viral load (VL)>1000 copies/mL were randomized and maintained in the base arm (1‐DTG:1‐EFV). The primary end point was the proportion of participants with a VL of less than 50 copies/mL at week 48; secondary outcomes were assessed with superiority‐test.

**Results**: At week 192, proportions of participants with a VL of less than 50 copies/mL in intention‐to‐treat (ITT) were 69% (DTG: 214/310) and 62% (EFV400: 187/303) respectively (difference, 7.3%; CI‐95%, [−0.20;14.83], p‐value=0.057; Figure1). *Per‐protocol* results were 75% (DTG: 172/230) and 66% (EFV400: 178/271) respectively (difference, 9.1%; CI‐95%, [1.13;17.07], p‐value=0.027). During the four‐year of follow‐up, five (DTG: 2; EFV400: 3) new virological failures (WHO‐definition) without related resistance mutations and 24 new severe adverse‐events (SAE) were observed (DTG: 13, EFV400: 11). Over four years, weight gain was more important on DTG group compared to EFV400 group: Median weight‐gain (Women (W): DTG +8.0 Kg, EFV400 +5.0 Kg, p‐value=0.010; Men (M): DTG +6.0 Kg, EFV400 +4.0 kg; p‐value=0.024); Obesity incidence (W: DTG 17%, EFV400 11%, p‐value=0.140; M: DTG 26%, EFV400 4%; p<0.001); Proportion of patients who had a weight‐gain of at least 15% compared to their initial weight (W: DTG 43%, EFV400 31%, p‐value=0.030; M: DTG 23%, EFV400 25%; p‐ value= 0.848; Figure 2).

**Conclusions**: DTG‐based and low dose EFV‐based regimens has durable efficacy and safety for use in treatment‐naïve patients with HIV‐1. There was significantly more weight gain with the DTG‐containing regimen.

### OAB0305 Survival in advanced AIDS patients treated with efavirenz or dolutegravir in Brazil: a multicenter, observational study

C. Brites ^1^, M. Lacerda^2^, S. Eduardo^3^, M. Bay^4^, G. Pinto^5^, P. Azevedo^1^, I. Luz^1^, E. Luz^1^, E. M. Netto^1^

^1^Federal University of Bahia, Medicine, Salvador, Brazil, ^2^Fundação Instituto de Medicina tropical de Manaus, Infectious Diseases, Manaus, Brazil, ^3^Universidade Federal do Rio Grande do Sul, Infectious Diseases, Porto Alegre, Brazil, ^4^Universidade Federal do Rio Grande do Norte, Infectious Diseases, Natal, Brazil, ^5^Universidade Federal de Santa Catarina, Infectious Diseases, Florianopolis, Brazil

**Background**: Most of studies on integrase inhibitors efficacy were conducted on healthy patients. There is scarce information on DTG use in late‐presenters HIV patients. We compared the effect of ART regimens based on Efavirenz (EFV) or Dolutegravir (DTG) on survival of patients with advanced AIDS.

**Methods**: We enrolled symptomatic AIDS patients starting therapy with a CD4 count<50 cells/ml in 5 Brazilian cities. We compared patients starting DTG‐based ART (2018 to 2020) or EFV‐based regimens (2013 to 2016), as controls regarding early mortality, rates of viral suppression at 24 and 48 weeks, changes in CD4 count, incidence of adverse events, and therapy discontinuation.

**Results**: We included 92 patients per arm mean age 39.4 (DTG) and 37.3 years (EFV), 68 % males, mean baseline CD4 count=23 cells/ml, mean HIV viral load= 5.5 copies/ml log_10_. Viral suppression rates (<50 copies/ml) were higher in DTG than in EFV group at 24 (67% vs 42%,) and at 48 weeks (65% vs 46%, p<0.01). At 48 weeks median CD4 count was similar for DTG and EFV groups (213 cells/ml vs. 222 cells/ml), but more patients in DTG group presented with CD4 >200 cells/ml (45% vs. 29%, p=0.03). Levels of total cholesterol(189 vs 168 mg/dL), triglycerides (188 vs 129 mg/dL) and VLDL cholesterol(35 vs 26 mg/dL) were higher in EFV than in DTG group (p<0.01 for comparisons). Creatinine levels were higher in DTG (0.97 mg/dL) than in EFV (0.86 mg/dL, p=0.02) group. Survival was higher in DTG group (figure 1), mostly driven by treatment changes (1% vs. 17%, p<0.0001) or loss to follow up (11% vs. 15%).

**Conclusions**: Advanced AIDS patients treated with DTG had a higher proportion of viral suppression/survival rate/ immune restoration, less lipids changes, and lower discontinuation rates after 48 weeks than patients treated com EFV. DTG is confirmed as a preferential option to treat advanced AIDS patients.

### OAB0402 Efficacy and safety results in participants co‐infected with HIV from TB‐PRACTECAL Clinical Trial

I. Motta ^1^, C. Berry^1^, E. Kazounis^1^, M. Dodd^2^, K. Fielding^2^, B.‐T. Nyang'wa^3^, TB‐PRACTECAL team

^1^Medecins Sans Frontieres UK, London, United Kingdom, ^2^London School of Hygiene and Tropical Medicine, London, United Kingdom, ^3^Medecins Sans Frontieres Holland, Amsterdam, Netherlands, the

**Background**: Globally, TB/HIV coinfection accounts for 477461 notified cases and 456000 cases of rifampicin‐resistant tuberculosis (RR‐TB) are estimated. TB‐PRACTECAL clinical trial (NCT02589782) evaluated the safety and efficacy of three 24‐week all‐oral regimens for the treatment of pulmonary RR‐TB in adults and adolescents above 15 years from Uzbekistan, Belarus and South Africa. Participants were randomised to receive one of three investigational regimens or the control. BPaL arm consisted of bedaquiline, pretomanid and linezolid. Clofazimine was added in BPaLC or moxifloxacin in BPaLM arm. The primary efficacy outcome was the percentage of patients with a composite unfavourable outcome (treatment failure, death, treatment discontinuation, recurrence, loss to follow‐up) at 72 weeks post‐randomization. We present the efficacy and safety of regimens in the HIV coinfected patients.

**Methods**: All patients were offered a HIV test and were eligible irrespective of CD4 count. Antiretroviral treatment was modified to minimise drug‐drug interactions and co‐trimoxazole prophylaxis offered. Pre‐specified analyses were conducted for primary efficacy and safety outcomes at 72 weeks post‐randomization for HIV status in mITT and ITT populations, respectively.

**Results**: 552 patients were enrolled, of whom 153 were HIV positive. Median CD4 count at baseline was 330, 297, 326 and 250 cells/μL in BPaLM, BPaLC, BPaL and control arm, respectively. In mITT population 28.6%, 35.7%, 28.6% and 40% of HIV‐positive patients experienced unfavourable outcomes in BPaLM, BPaLC, BPaL and control arm, respectively. The small sample size and P‐values don't provide strong evidence of interaction. In ITT population grade 3 or above and/or serious adverse events (SAEs) were no more frequent in HIV‐positive versus negative patients across arms.

**Table:** 

**Abstract OAB0402‐Table 1**.								
HIV status	Unfavourable outcome Controln/N (%)	Unfavourable outcome Experimental Arm n/N (%)	Risk difference (one‐sided 98.3% CI)	Interaction p‐value	Grade ≥3 and/or SAEs Controln/N (%)	Grade ≥3 and/or SAEsExperimental Armn/N (%)	Risk difference (one‐sided 98.3% CI)	Interactionp‐value
**BPaLM versus control**								
Negative Positive	26/51 (51.0) 6/15 (40.0)	3/48 (6.3) 4/14 (28.6)	−44.7% (−∞ to −28.1%) −11.4% (−∞ to 25.6%)	p = 0.08	33/56 (58.9) 10/17 (58.8)	11/55 (20.0) 3/17 (17.6)	−38.9% (−∞ to −20.9%) −41.2% (−∞ to −9.2)	p = 0.89
**BPaLC versus control**								
Negative Positive	26/51 (51.0) 6/15 (40.0)	7/50 (14.0) 5/14 (35.7)	−37.0% (−∞ to −18.9%) −4.3% (−∞ to 33.9%)	p = 0.10	33/56 (58.9) 10/17 (58.8)	17/57 (29.8) 6/15 (40.0)	−29.1% (−∞ to −10.1%) −18.8% (−∞ to 18.0%)	p = 0.60
**BPaL versus control**								
Negative Positive	26/51 (51.0) 6/15 (40.0)	10/46 (21.7) 4/14 (28.6)	−29.2% (−∞ to −9.6%) −11.4% (−∞ to 25.6%)	p = 0.37	33/56 (58.9) 10/17 (58.8)	11/51 (21.6) 4/18 (22.2)	−37.4% (−∞ to −18.8%) −36.6% (−∞ to −3.9%)	p = 0.97

**Conclusions**: Current TB‐PRACTECAL data supports the use of 24‐week regimens irrespective of HIV status. A trend towards the shorter regimens being more efficacious in HIV‐negative patients was observed. However, this trend was not seen in the safety outcomes for the BPaL and BPaLM arms. The trial is accruing more data and will update at a later date.

### OAB0403 Impact of a community‐wide HIV test and treat intervention on population‐level tuberculosis transmission in rural Uganda

C. Marquez ^1^, M. Atukunda^2^, J. Nugent^3^, E. Charlebois^1^, G. Chamie^1^, F. Mwangwa^2^, E. Ssemmondo^2^, J. Kironde^2^, J. Kabami^2^, A. Owaraganise^2^, E. Kakande^2^, R. Abbott^1^, B. Ssekaynzi^2^, J. Ayieko^4^, T. Ruel^1^, D. Kwariisima^2^, M. Kamya^2,5^, M. Petersen^6^, D. Havlir^1^, L. Balzer^3^

^1^University of California, San Francisco, San Francisco, United States, ^2^Infectious Diseases Research Collaboration, Kampala, Uganda, ^3^University of Massachusetts‐ Amherst, Amherst, United States, ^4^Kenya Medical Research Institute, Nairobi, Kenya, ^5^Makerere University School of Medicine, Kampala, Uganda, ^6^University of California, Berkeley, Berkeley, United States

**Background**: HIV and TB are linked epidemics. We tested whether a community‐wide universal HIV testing and treat strategy would reduce population‐level TB transmission in the SEARCH trial.

**Methods**: SEARCH was 32‐community cluster‐randomized trial conducted in rural Uganda and Kenya from 2013‐2017 (NCT:01864603). Intervention communities received population‐level HIV test and treat; control communities received enhanced standard‐of‐care. We measured incident TB infection in a nested cohort of children and adults ages (>5 years) in in 4 intervention and 5 control communities (8 and 10 parishes, respectively) in Eastern Uganda. All people in the cohort had a negative tuberculin skin test (TST) at baseline (induration <10mm or <5 mm among PWH); the cohort was enriched with PWH. Incident TB infection (primary outcome) was defined as TST conversion from negative to positive one year after baseline TST (2015‐2017). Incident TB was compared between arms, adjusted for sampling and participation, and parish‐level drivers of incident TB infection and accounting for clustering using Targeted Maximum Likelihood Estimation.

**Results**: The TST negative cohort was comprised of 3,242 persons in the intervention and 4,125 persons in the control arms; 2,922 (39.7%) were children (5‐11 years) and 4,445 (60.3%) youth and adults (ages 12+ years). Within the cohort 53.3% were women, and PWH comprised 16.6% of adults, 1.1% of children. One‐year cumulative incidence of TB infections was 16% in the intervention and 22% in the control; the population‐level intervention reduced risk of incident TB infection by 27% (aRR of 0.73; 95% CI: 0.58‐0.93, one‐sided p=0.007). The effect was largest among children aged 5‐11 years (Figure 1).

**Conclusions**: A universal HIV test and treat intervention reduced incident TB infection, a marker of population‐level TB transmission. Investments in community‐level HIV interventions have direct impacts on HIV and broader population‐level benefits, including reductions in TB.

**Figure:**
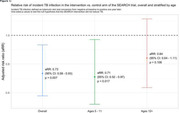
**Abstract OAB0403‐Figure 1**.

### OAB0404 Are people living with HIV at higher risk of severe and fatal COVID‐19?

S. Bertagnolio ^1^, R. Silva^1^, S. Nagarajan^1^, S.S. Thwin^1^, W. Jassat^2^, R. Fowler^3^, R. Haniffa^4^, L. Reveiz^5^, N. Ford^1^, M. Doherty^1^, J. Diaz^1^

^1^World Health Organization, Geneve, Switzerland, ^2^National Institute for Communicable Diseases, Johannesburg, South Africa, ^3^Sunnibrook Health Science Centre, Toronto, Canada, ^4^University of Oxford, Oxford, United Kingdom, ^5^Pan American Health Organization, Washington, United States

**Background**: WHO has established a Global Clinical Platform aiming to assess clinical features and risk factors for severe/fatal COVID‐19 among hospitalized individuals.

**Methods**: Between January 2020‐June 2021 anonymized individual‐level clinical data from 338,566 patients hospitalized in 38 countries were reported to WHO using a standardized case report form. Descriptive and regression analyses assessed whether HIV status was a risk factor for severity at admission and in‐hospital mortality among people hospitalized for COVID‐19.

**Results**: Of 197,479 patients reporting HIV status, 8.6% (16,955) were living with HIV (PLHIV), and 94.6% (16,283) were from Africa; 37.1% were male, mean age was 45.5 years, 38.3% were admitted with severe or critical illness and 24.7% died in‐hospital. 91.5% of 10,166 PLHIV were on antiretroviral therapy (ART). Compared to those without HIV, PLHIV had 15% increased odds of severe/critical presentation (aOR=1.15, 95%CI 1.10–1.20) and were 38% more likely to die in‐hospital (aHR=1.38, 95%CI 1.34‐1.41). Among PLHIV, male sex, age 45‐75 years, and having chronic cardiac disease or hypertension increased the odds of severe/critical COVID‐19. Male sex, age>18 years, diabetes, hypertension, malignancy, TB, or chronic kidney disease increased the risk of in‐hospital mortality. In an exploratory subgroup analysis in a subset of 9097 hospitalized individuals reporting ART information, PLHIV on ART were 17% less likely to die (p<0.048) and 40% less likely to be admitted with severe disease than those not on ART (p<0.001). However, both PLHIV on ART (aHR=1.48, 95%CI 1.39‐1.57) and those not on ART (aHR=1.79, 95%CI 1.48‐2.16) had a higher risk of death relative to HIV negative people.

A similar exploratory analyses on a sample of 5793 hospitalized individuals reporting viral load (VL) information showed that both people with VL<1000 c/ml (aHR=1.77, 95% CI 1.57‐1.99) and those with VL>1000 c/ml (aHR=1.45, 95% CI 1.32‐1.58) have an increased risk of death compared to HIV negative individuals.

**Conclusions**: In this sample of hospitalized people contributing data to the WHO Global Clinical Platform, HIV was an independent risk factor for both severe/critical COVID‐19 at admission and in‐hospital mortality. These findings have informed the WHO COVID‐19 Clinical Management Guidelines and SAGE recommendations around COVID‐19 vaccination prioritization.

### OAB0405 HIV/HBV coinfection in pregnancy and response to antiretroviral therapy

D. Bhattacharya ^1^, C. Tierney^2^, A. Chang^2^, D. Moodley^3^, V. Govender^3^, T. Vhembo^4^, N. Mohtashemi^1^, H. Ship^5^, D. Dula^6^, K. George^7^, N. Chakhtoura^8^, F.K. Matovu^9^, M.G. Fowler^10^, M. Peters^11^, J.S. Currier^1^

^1^University of California, Los Angeles David Geffen School of Medicine, Los Angeles, United States, ^2^Harvard TH Chan School of Public Health, Boston, United States, ^3^University of KwaZulu‐Natal, Durban, South Africa, ^4^Uz‐UCSF Collaborative Research Programme, Harare, Zimbabwe, ^5^University of Miami Miller School of Medicine, Miami, United States, ^6^Johns Hopkins Research Project, Blantyre, Malawi, ^7^FHI 360, Durham, United States, ^8^National Institutes of Health, Bethesda, United States, ^9^Makerere University–Johns Hopkins University Research Collaboration, Kampala, Uganda, ^10^The Johns Hopkins University, Baltimore, United States, ^11^University of California San Francisco, San Francisco, United States

**Background**: Hepatitis B virus (HBV) affects 3‐12% of pregnant women with HIV in Africa; however, the impact of HBV infection on HIV outcomes in pregnant women is unclear. We evaluated the association of HIV/HBV coinfection with HIV virologic response, CD4 cell count, and hepatotoxicity in pregnant women in secondary analyses of the IMPAACT PROMISE study.

**Methods**: The PROMISE study enrolled pregnant women living with HIV who were ART‐naive and had not met criteria for initiating ART. Women at ≥14 weeks gestation were randomized to either: Arm A: ZDV alone, Arm B: 3TC+ZDV+LPV/r, or Arm C: FTC+TDF+LPV/r. HBV was defined as HBsAg(+). We compared women with HIV alone to HIV/HBV with outcomes of HIV viral load, CD4, and ALT elevation at delivery and through 74 weeks postpartum using Fisher's exact, t‐tests. Time to ALT used the log‐rank test. Analyses also compared women with HIV alone to HIV/HBV who were also HB**e**Ag(+).

**Results**: Among 3537 women analyzed, 138 had HBV. Median age, CD4, and HIV VL were 27 years, 505 cells/mm^3^ and 4.0 Log_10_ copies/mL, respectively. Thirty‐four women (26%) with HBV were HB**e**Ag(+). Median ALT at baseline was 15 and 12 IU/ml amongst HBV‐infected and uninfected. There was no statistically significant difference by HBV/HIV co‐infection status in HIV VL suppression or CD4 at delivery, at one year postpartum (PP) (primary) or 74 weeks PP. Those with HIV/HBV had more frequent grade 3/4 ALT events (8% vs 3%) overall. Numerically more grade 3/4 ALT events occurred in HIV/HBV coinfected women in Arms B and C (10.4%, 10.4%) vs Arm A (2.4%) but this did not reach statistical significance. Women with HIV/HBV also had earlier first grade 3/4 ALT elevation (hazard ratio (HR) 3.08, 95% CI: 1.56, 5.50). Compared to those with HIV alone, HBV infected women with HB**e**Ag(+) had earlier Grade 3/4 ALT elevation (HR 6.93, 95% CI: 2.70, 14.51).

**Conclusions**: In pregnant women with HIV initiating ART, HIV RNA suppression and CD4 cell response did not differ between HIV and HIV/HBV coinfection. However, grade 3 or 4 ALT elevations occurred at a higher rate in those with HBV, with HBeAg+ status conferring increased risk.

### OAC0102 Progress in scaling up HIV recent infection surveillance in 13 countries: October 1, 2019 ‐ June 30, 2021

H. Truong ^1,2^, M. Arons^1^, M. Schmitz^3,4^, M. Pasipamire^5^, G. Abera^6^, A. Haile^6^, Y. Tedla^6^, N. Farach^7^, C. Gutierrez^7^, N. Wadonda‐Kabondo^8^, D. Payne^8^, A. Beukes^9^, I. Pietersen^9^, M. Alagi^10^, G. Asalou^10^, I. Kankindi^11^, S. Malamba^11^, C. Kittinunvorakoon^12^, A. Mubiru^13^, B.T.T. Hien^14^, Q. Nguyen^14^, P. Minchella^15^, P. Chikwanda^16^, E. Gonese^16^, B. Moyo^16^, S. Behel^1^, E. Yufenyuy^1^, S. Ahmed^1^, N. Zender^17^, H. Dale^1^, P. Hosseini^17^, O. Nzoutchoum^4,3^, E. Sharp^4,3^, B. Parekh^1^, M. Martin^1^, P. Monita^1^

^1^Centers for Disease Control and Prevention, Atlanta, United States, ^2^Public Health Institute/Centers for Disease Control, Global Health Fellowship Program, Atlanta, United States, 3United States Military HIV Research Program, Walter Reed Army Institute of Research, Silver Spring, United States, ^4^Henry M. Jackson Foundation for the Advancement of Military Medicine, Bethesda, United States, ^5^Centers for Disease Prevention and Control, Mbabane, Eswatini, ^6^Centers for Disease Prevention and Control, Addis Ababa, Ethiopia, ^7^Centers for Disease Control and Prevention, Guatemala City, Guatemala, ^8^Centers for Disease Control and Prevention, Lilongwe, Malawi, ^9^Centers for Disease Control and Prevention, Windhoek, Namibia, ^10^Centers for Disease Control and Prevention, Abuja, Nigeria, ^11^Centers for Disease Control and Prevention, Kigali, Rwanda, ^12^Centers for Disease Control and Prevention, Bangkok, Thailand, ^13^Centers for Disease Control and Prevention, Kampala, Guatemala, ^14^Centers for Disease Control and Prevention, Hanoi, Viet Nam, ^15^Centers for Disease Control and Prevention, Lusaka, Zambia, ^16^Centers for Disease Control and Prevention, Harare, Zimbabwe, ^17^Office of the U.S. Global AIDS Coordinator and Health Diplomacy, United States Department of State, Washington, D.C., United States

**Background**: In 2018, countries supported by the United States President's Emergency Plan for AIDS Relief (PEPFAR) began implementing recent HIV infection surveillance among newly diagnosed people living with HIV (PLHIV) to provide signals of ongoing HIV transmission. Recent infection surveillance uses results from rapid tests for recent infection (RTRIs) with viral load results to improve accuracy as part of a recent infection testing algorithm (RITA). We assessed global progress in recent infection surveillance in PEPFAR‐supported countries.

**Methods**: We collected recency data for adults ≥15 years from October 1, 2019–June 30, 2021 and pooled results from 13 countries (Eswatini, Ethiopia, Guatemala, Malawi, Namibia, Nicaragua, Nigeria, Rwanda, Thailand, Uganda, Vietnam, Zambia, Zimbabwe). We described quarterly trends in RTRI site coverage (% of sites reporting ≥1 RTRI) and RTRI testing coverage (% of newly diagnosed PLHIV who had an RTRI). In a subset of seven countries (Eswatini, Nigeria, Rwanda, Thailand, Uganda, Vietnam, Zambia), we described trends in RITA site and testing coverage (% of sites and PLHIV reporting RITA results) for the October 1, 2019–June 30, 2021 study period.

**Results**: Among the 13 countries, RTRI site coverage increased from 10.3% (733/7,107) to 21.5% (1,900/8,818) during the study period, and RTRI testing coverage increased from 9.0% (13,864/153,268) to 18.0% (41,688/231,410). During April 1–June 30, 2020, a quarter within the study period coinciding with the COVID‐19 epidemic, RTRI site and testing coverage decreased 9.6% (22,437/234,164) to 3.2% (85,791/183,085). For the subset of seven countries, RITA site coverage decreased from 71.0% (88/124) to 48.1% (276/574) during the study period, and RITA testing coverage decreased from 70.2% (290/413) to 58.7% (1,651/2,814).

**Conclusions**: For the 13 countries, RTRI site and testing coverage increased during the study period but remained low. RTRI coverage decreased temporarily April 1–June 30, 2020. For the subset of seven countries, RITA site and testing coverage decreased during the study period. These decreases may be due to COVID‐19 mitigation efforts, disruptions in HIV and viral load testing, and PEPFAR guidance to temporarily pause recent infection testing. Expanding access to recent infection testing can provide data for public health action to prevent new HIV infections.

### OAC0103 Use of a robust health information system to improve accuracy of recent HIV infection testing in Bangkok, Thailand, 2020‐2021

S. Aungkulanon ^1^, C. Kittinunvorakoon^1^, T. Tasaneeyapan^1^, S. Tanpradech^1^, S. Nookhai^1^, N. Pattarapayoon^2^, P. Mookleemas^2^, A. Vittangkrun^3^, P. Chaiphosri^4^, T. Naiwatanakul^1^, S. Northbrook^1^

^1^U.S. Centers for Disease Control and Prevention, Division of Global HIV and TB, Nonthaburi, Thailand, ^2^Thailand Ministry of Public Health, Division of AIDS and STIs, Department of Disease Control, Nonthaburi, Thailand, ^3^Bangkok Metropolitan Administration (BMA), Department of Medical Services, Bangkok, Thailand, ^4^Bangkok Metropolitan Administration (BMA), Department of Health, Bangkok, Thailand

**Background**: Based on the Asia Epidemic Model, Bangkok has the highest number of estimated new HIV infections in Thailand. We integrated HIV recent infection testing into routine HIV testing services to monitor recent infection trends and accelerate efforts to ending AIDS in Bangkok.

**Methods**: HIV recency testing was integrated in 16 facilities in Bangkok. Blood from consenting newly diagnosed adults with HIV (PLHIV) collected during October 2020–November 2021 was tested with a rapid test for recent infection (RTRI). Because PLHIV who have suppressed viral load (VL) can produce false recent results, we tested RTRI‐recent specimens with VL (recent infection testing algorithm [RITA]); specimens with VL≥1,000 copies/ml were defined as RITA‐recent, <1,000 copies/ml as RITA‐long term (LT). Cross‐checking clinical history data with the national AIDS database (NAD) and electronic medical records was conducted for all RTRI‐recent cases to enhance the validity and accuracy of results. Logistic regression was used to identify associations between recent infection and demographic variables (sex, age, population).

**Table:** 

**Abstract OAC0103‐Table 1**.				
Demographic		% RITA recent infection (n/total)	OR	p‐value
Age	15‐19 years	16% (7/44)	2.5	0.04
	20‐29 years	8% (23/302)	1.1	0.75
	>30 years	7% (24/345)	1	Ref
Sex	Female	9% (14/164)	1	Ref
	Male	8% (40/532)	0.9	0.67
Population group	General	6% (23/356)	1	Ref
	MSM	10% (27/267)	1.6	0.10
	Other	4% (4/73)	0.8	0.75

**Results**: 694/1,291 (53.8%) newly diagnosed PLHIV provided consent and were tested for recent infection: 59 (8.5%) tested RTRI‐recent and, after VL testing, 54 (7.8%) tested RITA‐recent and 5 tested RITA‐LT. After cross‐checking with NAD, 4/5 RITA‐LT cases were among 21‐25 year old men who had a history of pre‐exposure prophylaxis, antiretroviral treatment (ART) <28 days at the time of RTRI testing or no ART history, and no previous HIV diagnosis, suggesting potential misclassification. Young clients aged 15‐19 were more likely to test RITA‐recent than other age groups (OR=2.5, p<.05).

**Conclusions**: While 15‐19 year‐olds showed a high proportion of RITA positives, recent HIV infections were diagnosed in all age groups, sexes and populations, warranting continued surveillance of HIV‐1 recent infections in Bangkok. Cross‐checking the clinical history with other records through a robust national health information system can be used to improve the accuracy of tests for recent infection.

### OAC0104 A rapid, integrated method to monitor HIV viral load, drug resistance, and transmission patterns from finger‐prick blood samples

S.E. Chaudron ^1^, L. Zhao^2^, G. Macinthyre‐Crockett^1^, L. Thompson^2^, I. Baudi^1^, M. Limbada^3^, S. Floyd^4^, B. Kosloff^3,5^, K. Shanaube^3^, S. Fidler^6^, R. Hayes^4^, H. Ayles^3,5^, J. Herbeck^7^, C. Fraser^2,1^, D. Bonsall^2,1^, The PANGEA Consortium, The AMPHEUS research group

^1^Wellcome Centre for Human Genetics, Nuffield Department of Medicine, University of Oxford, Oxford, United Kingdom, ^2^Big Data Institute, Nuffield Department of Medicine, University of Oxford, Oxford, United Kingdom, ^3^Zambart House, School of Medicine, University of Zambia, Lusaka, Zambia, ^4^Department of Infectious Disease Epidemiology, London School of Hygiene and Tropical Medicine, London, United Kingdom, ^5^Department of Clinical Research, London School of Hygiene and Tropical Medicine, London, United Kingdom, ^6^Imperial College, United Kingdom and Imperial College NIHR BRC, London, United Kingdom, ^7^Department of Global Health, School of Public Health, University of Washington, Seattle, United States

**Background**: By applying sequencing and phylogenetics to tens‐of‐thousands of plasma samples from various cohorts of people living with HIV, we have characterised the dominant sources and flows of HIV transmission, tracked changing patterns of drug resistance, and discovered a highly infectious HIV lineage with faster disease progression. As global incidence of HIV/AIDS declines, we need broader individual access to viral load monitoring and more‐efficient molecular surveillance to track population‐level changes in drug resistance and transmission. Our findings and modelled projections incentivised development of an integrated method that monitors HIV viral load, drug resistance and co‐infections, while producing anonymised HIV‐transmission data to inform adaptive public health strategies.

**Methods**: In this study, venepuncture‐derived plasma from 61 epidemiologically‐characterised transmission pairs from The Partners in Prevention Transmission Study, and plasma collected from HPTN‐071 (PopART) by venepuncture and 'finger‐prick’ sampling, were deep‐sequenced using bait‐capture (veSEQ). Sample processing was optimised at key steps for long‐read PacBio sequencing. Transmission pairs were identified using Phyloscanner software, and drug resistance was predicted using the Stanford algorithm (HIVdb). Viral loads measurements were validated against clinically‐accredited assays.

**Results**: Our optimised protocol generated whole‐genome sequences and drug resistance predictions from 200 μl of finger‐prick derived plasma, with viral loads greater than 500 copies per ml. Viral loads were obtained after 5 hours of processing from the same ‘finger‐prick’ specimens and the overall cost of the combined assay was kept below that of commercially available viral load tests. Whole hepatitis B genomes were sequenced concurrently by the same method in a subset of samples. PacBio sequencing correctly identified the direction of transmission in 85% (52/61) of pairs compared to 36% (22/61) using Illumina sequencing. The longer reads generated by PacBio (250bp‐2.5kb; 95% CI) reduced the likelihood of misreporting transmission direction.

**Conclusions**: Our combined sequence‐based assay accurately measures HIV VL and characterises drug resistance from ‘finger‐prick’ derived blood and generates anonymised, high‐resolution, molecular‐epidemiological data for public health. This low‐cost combined approach could be transformative to global health management of HIV and related blood born infections in resource‐limited settings.

### OAC0105 Phylogenetic surveillance of HIV epidemic control from 2014 to 2020 among MSM and heterosexual groups in Quebec

B. Brenner ^1^, R.‐I. Ibanescu^1^, M. Oliveira^1^, J.‐P. Routy^2^, R. Thomas^3^, M. Roger^4^, Montreal Primary HIV Infection Study Group

^1^Lady Davis Institute, McGill AIDS Centre, Montreal, Canada, ^2^McGill University Health Centre, Montreal, Canada, ^3^Clinique Médicale l'Actuel, Montreal, Canada, ^4^University of Montreal, Montreal, Canada

**Background**: The UNAIDS 90‐90‐90 initiative has led to 38% declines in heterosexual (HET) epidemics in Africa since 2014. In 2019, The Ending the HIV Epidemic for America added phylogenetics as fourth pillar for epidemic control by 2030. Here, phylogenetics was combined with available epidemiological data to track drivers of HIV‐1 spread among Men having Sex with Men (MSM), People Who Inject Drugs (PWID) and recent migrants.

**Methods**: Phylogenetic analyses, using MEGA‐10 and Microbe‐TRACE methodologies, ascertained the linkage of sequences obtained from newly‐diagnosed persons (2002─2020). Infections were stratified into groups according to HIV‐1 subtype, sex, and cluster size, including i) the subtype B MSM epidemic (male singletons/male‐male clusters); ii) the subtype B PWID epidemic (mixed gender large clusters); iii) subtype B HET infections originating from the Caribbean and Americas (female singletons, mixed gender clusters) and iv) non‐B subtype epidemics originating outside Canada.

**Results**: Amongst MSM, there were 56%, 43% and 27% declines in singleton, small (1‐5 members) and large cluster networks (6‐152 members) from 2014─2020 compared to 2007─2013. Epidemic control among MSM was thwarted by 35 super‐spreader, large cluster micro‐epidemics, adding 8─‐96 infections/cluster from 2014‐2020. Notably, 18 clusters gained 4‐12 infections during the COVID 19 era (post‐2019). The subtype B epidemic among PWID is controlled showing 54% declines in new infections from 2014‐2020 compared to 2007‐2013. Recent migration has led to the steady growth in the subtype B and non‐B subtype HET epidemics. To date, 65% and 20% of HET epidemics were singletons or small clusters (2‐4 members). PHI cohort data revealed that 28% of infected MSM born outside Canada acquired large cluster provincial variants. Of note, isolated non‐B subtype HET outbreaks occurred in Quebec City, Richelieu, and Northern Quebec.

**Figure:**
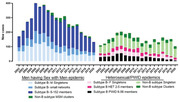
**Abstract OAC0105‐Figure 1**.

**Conclusions**: Declines in HIV‐1 infections are promising. Public health measures must address emerging needs of vulnerable migrants and younger MSM populations.

### OAC0202 Collision of HIV and non‐communicable disease epidemics: mapping chronic health needs among a HIV hyperendemic community in rural South Africa

D. Cuadros ^1^, U. Singh^2^, S. Olivier^2^, Y. Moosa^2^, A. Edwards^3^, H.‐Y. Kim^4^, M. Siedner^5^, E. Wong^2^, F. Tanser^3^

^1^University of Cincinnati, Cincinnati, United States, ^2^Africa Health Research Institute, Durban, South Africa, ^3^University of Lincoln, Lincoln, United Kingdom, ^4^New York University Grossman School of Medicine, New York, United States, ^5^Massachusetts General Hospital, Boston, United States

**Background**: With improvements in survival among people living with HIV (PLHIV), the co‐occurrence of HIV and non‐communicable diseases is emerging as a public health priority. Using a comprehensive population‐based disease survey in rural South Africa (SA), we aimed to characterize the spatial structure of multimorbidity and unmet health system needs for HIV, diabetes, and hypertension.

**Methods**: Data for HIV, diabetes and hypertension were collected as part of the Vukuzazi study, a population‐based health assessment conducted in uMkhanyakude district in KwaZulu‐Natal, SA. Participants were categorized by a novel health needs scale including: healthy/absence of disease (needs score 0), diagnosed and well controlled (1), diagnosed and sub‐optimally controlled (2), diagnosed and not engaged in care (3) and undiagnosed and uncontrolled (4). Scores 2‐4 indicated individuals experiencing unmet needs. We explored the geospatial structure of unmet needs using different multivariate spatial clustering methods.

**Results**: The analytic sample included 18,041 individuals. HIV had a similar spatial structure for those with a combined needs score 2‐3 (diagnosed but uncontrolled) and 4 (undiagnosed and uncontrolled), with most PLHV with unmet needs been clustered (red areas in maps Figure A) in the southern urban/peri‐urban area. Multivariate KMeans clustering analysis identified a significant overlap of all three diseases for individuals with undiagnosed and uncontrolled diseases (unmet needs score 4) in the southern part of the surveillance area (Cluster 3 in red in map Figure B).

**Figure:**
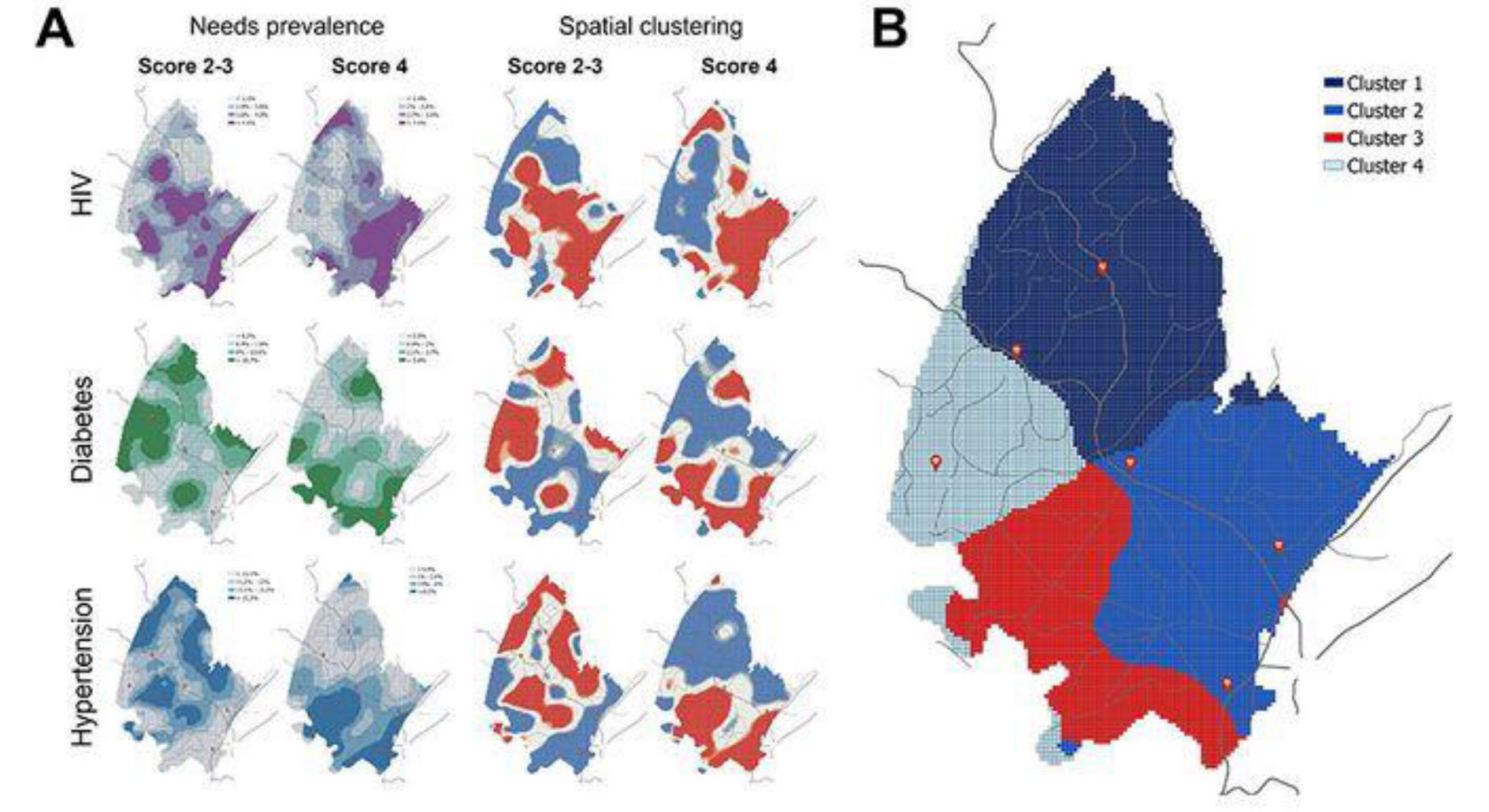
**Abstract OAC0202‐Figure 1**.

**Conclusions**: Areas where most PLHIV are experiencing the highest needs with undiagnosed and uncontrolled disease are also areas suffering the highest burden of unmet needs for other diseases like diabetes and hypertension in this rural community in SA. The identification and prioritization of high needs areas where health systems needs for both HIV and non‐communicable diseases collide provides a rationale for policy and implementation strategies to improve health outcomes with optimal efficiency and impact.

### OAC0203 The impact of COVID‐19 non‐pharmaceutical interventions on incidence and case‐fatality ratios of sexually transmitted diseases in China

X. Wu ^1^, X. Zhou^1^, Y. Chen^1^, K. Zhai^1^, G. Luo^1^, B. Liang^1^, C. Yang^1^, H. Zou^1,2^

^1^School of Public Health (Shenzhen), Sun Yat‐sen University, Shenzhen, China, ^2^Kirby Institute, University of New South Wales, Sydney, Australia

**Background**: China implemented nationwide non‐pharmaceutical interventions (NPIs) to contain COVID‐19 at the early stage. We aimed to elucidate the impact of COVID‐19 NPIs on incidence and case‐fatality ratios of sexually transmitted diseases (STDs) in China.

**Methods**: Cases and deaths data for STDs by month were extracted from the notifiable disease reporting database of the official website of the National Health Commission of China between January 2015 and August 2021. We used descriptive statistics to summarize data on case and death of HIV, gonorrhea, syphilis, hepatitis B, and hepatitis C, and calculated incidence and case‐fatality ratios before and after the implementation of massive NPIs (January 2020). We used Poisson segmented regression models to estimate the immediate and long‐term impacts of NPIs on these outcomes in January 2020 and August 2021, respectively.

**Results**: A total of 14,071,484 cases and 118,399 deaths of the five STDs were reported from January 2015 to August 2021, with an incidence of 149.10/100,000 before NPIs and 150.72/100,000 after, and a case‐fatality ratio of 8.21/1000 before NPIs and 9.02/1000 after. In the Poisson model, accounting for seasonal fluctuations and long‐term trends, there was a 19.2% (IRR 0.808, 95% CI 0.714‐0.914) decline in overall incidence and a 20.9% (0.791, 0.680‐0.921) decline in overall deaths of the five STDs in January 2020. There was no significant change in incidence or case‐fatality ratios of HIV in January 2020, but a 22.1% (0.779, 0.647‐0.938) decline in deaths. Deaths and case‐fatality ratios for both gonorrhea and syphilis showed an increase of 152.1%‐897.6% compared with a small counterfactual in January 2020. Incidence of hepatitis B and C showed significant decreases in January 2020, but the changes in death and case‐fatality ratio were not statistically significant. By August 2021, the incidence, deaths, and case‐fatality ratios for each of the five STDs returned to or were below expected levels.

**Conclusions**: During the COVID‐19 pandemic, the implementation of massive NPIs had a positive impact on the overall incidence and deaths of STDs in China. However, under the current strategy of holistic NPIs in China, one should be alert to the increase in death and case‐fatality ratio from gonorrhea and syphilis.

### OAC0204 The effect of the COVID‐19 pandemic on access to HIV Treatment and vertical transmission: results from the Canadian Perinatal HIV Surveillance Program

J. Singer^1^, L. Sauve ^2^, A. Bitnun^3^, F. Kakkar^4^, J. Brophy^5^, I. Boucoiran^4^, D. Money^2^, T. Lee^6^, J. Comeau^7^, A. Tse‐Chang^8^, W. Vaudry^8^, Canadian_Perinatal_HIV_Surveillance_Program

^1^University of British Columbia, School of Population and Public Health, Vancouver, Canada, ^2^University of British Columbia, Women's Hospital and Health Centre of British Columbia, Vancouver, Canada, ^3^University of Toronto, Hospital for Sick Children, Toronto, Canada, ^4^Université de Montréal, CHU Ste‐Justine, Montreal, Canada, ^5^University of Ottawa, Children's Hospital of Eastern Ontario, Ottawa, Canada, ^6^Canadian HIV Trials Network, Vancouver, Canada, ^7^Dalhousie University, IWK Health Centre, Halifax, Canada, ^8^University of Alberta, Department of Pediatrics, Edmonton, Canada

**Background**: We describe demographics, antiretroviral treatment during pregnancy, and vertical transmission rates in the Canadian perinatal HIV surveillance cohort of births to women living with HIV (WLWH) and assess the effect of the COVID‐19 pandemic on access to optimal therapy and perinatal transmission.

**Methods**: 22 Canadian pediatric and HIV centres update data including demographics, antiretroviral treatment during pregnancy, and perinatal transmission, on births in WLWH yearly each January. The results reported in this abstract reflect births up to the end of 2020 but will be updated to include 2021 results.

**Results**: The number of HIV exposed infants per year has increased over time in Canada, with 250 infants born in 2020; 32% came from Ontario, 24% from Quebec, 17% from Alberta, 14% from Saskatchewan, 7% from British Columbia and 4% from Manitoba; 60% were Black, 21% were Indigenous, and 13% were white. Overall, 63% of this population acquired HIV heterosexually, 13% through injection drug use and 4.4% perinatally. The proportion and number of pregnant women sub‐optimally treated in May‐December 2020 was 7.7% (12/155) compared to 6.6% (86/1297) in the period from 2015‐2019.The corresponding transmission rates were 3.2% (5/155) versus 1.3% (17/1297), respectively. Among those who had acquired HIV through IDU, the sub‐optimal treatment rate was 26.1% during the COVID‐19 pandemic, versus 13.6% in the pre‐COVID‐19 period.

**Conclusions**: The perinatal transmission rate increased from 1.3% (2015‐2019) to 3.2% during the pandemic, the highest reported rate in over 5 years.Pregnant women who acquired HIV through IDU may have been at highest risk of vertical transmission because of sub‐optimal treatment.These data signal disturbing problems in accessing care for addictions, prenatal care and HIV‐specific care in the first waves of the pandemic.Additional attention to at‐risk populations is needed as the pandemic continues to affect Canada.

### OAC0205 Impact of COVID‐19 on TB case findings and TB/HIV co‐infection rates at a Princess Marina Hospital, Gaborone, Botswana, during the first year of COVID‐19

K. Fane ^1^, B. Kgwaadira^1^, B. Nkomo^2^, T. Molefi^3^, O. Fane^4^

^1^Botswana University of Maryland School of Medicine (Bummhi), TB/HIV Program, Gaborone, Botswana, ^2^Ministry of Health and Wellness, HIV/AIDS Department, Gaborone, Botswana, ^3^Ministry of Health and Wellness, Botswana National TB Program, Gaborone, Botswana, ^4^Sir Ketumile Masire Teaching Hospital, Nursing Department, Gaborone, Botswana

**Background**: Tuberculous incidence in HIV infected patients has steadily decreased in Botswana from 327/100,000 in 2010 to 115/100,000 in 2020. TB/HIV co‐infection rates also declined from 65% to 48%. However, it remains unclear how the COVID‐19 pandemic affected this progress following two countrywide lockdowns in 2020. We sought to determine the impact of COVID‐19 on TB/HIV case finding and TB/HIV co‐infection rates through retrospective analysis of 2019 and 2020 TB and TB/HIV indicators at Princess Marina Hospital (PMH) in Gaborone, the largest urban tertiary hospital in Botswana.

**Methods**: TB Case finding and TB/HIV co‐infection rates from 2019 and 2020, were extracted from individual patient records in electronic Integrated Patient Management System (IPMS), TB/HIV laboratory registers and admission registers. Data was disaggregated by gender, age, use of (Anti‐Retroviral Therapy) ART and (Cotrimoxazole Prophylaxis Therapy) CPT at the time of TB diagnosis.

**Results**: During 2020 ‐ the first year of COVID‐19 ‐ the number of TB diagnostic tests increased. Overall the number of TB cases decreased from 228 (65.6% TB positivity) to 79 (21.1% TB positivity). The percentage of children (<14 years) identified with TB were similar for both years. TB/HIV co‐infection rates increased from 64% to 77.2%. ART treatment for TB/HIV patients increased from 93.8% to 100% and 100% of TB/HIV patients received CPT in both years.

**Table:** 

	**2019**			**2020**		
**Total TB tests completed**:	347			373		
**TB cases confirmed**	228 (65.6%)			79 (21.1%)		
	97 female (42.5%)	131 Male (57.5%)		27 female (34%)	52 Male (66%)	
**TB Cases**	**≤14 years**	**≥15 years**		**≤14 years**	**≥15 years**	
	36 (15.8%)	192 (84.2%)		13(16.5%)	66(83.5%)	
**HIV Status**	**POS**	**NEG**	**UNKNOWN**	**POS**	**NEG**	**UNKNOWN**
	146 (64 %) Female:19.8%	79 (34.6%)	3 (1.3%)	61(77.2%) Female:24.6%	18 (22.8%)	0(%)
**TBHIV on ART**	**137 (93.8%)**			**61 (100%)**		

**Conclusions**: During 2020, the number of TB investigations by microscopy and GeneXpert at the PMH modestly increased. However, TB/HIV co‐infection rates increased by 13.3%. While it appears that the COVID‐19 pandemic had a significant effect on the number of TB cases identified ‐ likely due to social distancing and the use of masks ‐ further surveillance and analysis of TB/HIV indicators at PMH and across the country for 2021 is required.

### OAC0302 The 95‐95‐95 UNAIDS targets mask the underlying number of people with transmissible viral load: case study of England

A. Brown ^1^, V. Martin^1^, N. Connor^1^, R. Harris^1^, A. Presanis^2^, D. De Angelis^2^, V. Delpech^1^

^1^UK Health Security Agency, London, United Kingdom, ^2^MRC Modelling and Statistics Unit, Cambridge, United Kingdom

**Background**: In 2020, England met the 95‐95‐95 UNAIDS targets with 95% of the 97,740 (95% CrI 96,400‐100,060) people with HIV diagnosed, 99% diagnosed on treatment and 97% treated being virally suppressed; we explore the number of people with transmissible virus, comparing the UNAIDs method with a new approach.

**Methods**: The HIV and AIDS Reporting System (HARS) is the comprehensive health surveillance system for adults (<15 years) in England diagnosed with HIV. Serial records

For the UNAIDS metrics, we calculated the number of people with transmissible virus by using the Multi‐Parameter Evidence Synthesis (MPES) statistical model to estimate the number of people living with HIV and using HARS data to provide: the number of people diagnosed (in care in 2020), the number diagnosed and not treated (in care in 2020 but no treatment evidence); and the number treated with detectable virus (viral load >200 copies/mL adjusted for missing information).

The novel approach also incorporated those not linked to care (diagnosed in 2020 but not in care that year); not retained in care (in care in 2019 but not 2020); and treated without viral suppression evidence (on treatment with no viral load information).

**Results**: Using the UNAIDS method, an estimated 9% (8,800) of people with HIV had transmissible virus. Of these, 57% were estimated to be undiagnosed, 11% diagnosed and untreated and 32% treated but not virally suppressed.

With the new approach, up to 20% (19,800) people with HIV had transmissible virus levels. Of these, 24% were undiagnosed, 37% were diagnosed but not referred/retained in care, 6% untreated, 9% treated but not virally suppressed and 24% treated with no evidence of viral suppression.

**Conclusions**: The UNAIDS metric masks the absolute number of people living with transmissible virus, specifically excluding those not in care and those with missing information. Using the new approach, only a quarter of people living with transmissible HIV infection in England were estimated to be undiagnosed compared to over half using the UNAIDS approach. The focus on HIV prevention must be expanded from testing to include support for those with diagnosed HIV to remain in care, on treatment and virally suppressed.

### OAC0303 Characteristics associated with viral suppression among transgender women with HIV in 7 U.S. cities, NHBS‐Trans, 2019‐2020

J. Chapin‐Bardales ^1^, S. Harris^1,2^, S. Whitby^1^, S. Masciotra^1^, A. Smith^1^, J. Johnson^1^, K. Lee^1^, E. Olansky^3^, T. Finlayson^1^, L. Faucher^1^, C. Wejnert^1^, for the NHBS‐Trans Study Group

^1^U.S. Centers for Disease Control and Prevention, Division of HIV Prevention, Atlanta, United States, ^2^Oak Ridge Institute for Science and Education, assigned to the Division of HIV Prevention, U.S. Centers for Disease Control and Prevention, Atlanta, United States, ^3^ICF International, Atlanta, United States

**Background**: Sustained viral suppression can improve quality of life for transgender women with HIV. Understanding which transgender women with HIV may need additional support towards becoming virally suppressed can inform future interventions.

**Methods**: During 2019‐2020, National HIV Behavioral Surveillance recruited transgender women via respondent‐driven sampling in 7 U.S. cities (Atlanta, Los Angeles, New Orleans, New York City, Philadelphia, San Francisco, Seattle). Participants eligible for this analysis included those who were ≥18 years old, assigned male at birth or intersex, identified as a transgender woman or a woman, completed the survey, completed rapid HIV testing, had an HIV‐positive result, and provided dried blood spots that were tested for HIV viral load. Viral suppression was defined as a viral load result <1000 copies/mL. We assessed viral suppression prevalence and obtained adjusted prevalence ratios (aPR) and 95% confidence intervals (CI) for key associations using log‐linked Poisson regression models with robust standard errors accounting for clustering by recruitment chain and adjusting for city and network size.

**Results**: Overall, 80.8% of HIV‐positive participants were virally suppressed. Viral suppression was greater among participants who were Hispanic/Latina (vs. Black/African American; aPR=1.12, 95% CI: 1.02, 1.23) and had a college degree (vs. high school or less; aPR=1.13, 95% CI: 1.03, 1.24). Those who were younger (aPR[30‐39 vs. ≥40 years]=0.87, 95% CI: 0.79, 0.95), experienced homelessness within the past 12 months (aPR=0.89, 95% CI: 0.83, 0.96), had not visited an HIV care provider recently (aPR=0.81, 95% CI: 0.72, 0.91), and had an unmet need for healthcare due to cost within the past 12 months (aPR=0.77, 95% CI: 0.67, 0.89) were less likely to be virally suppressed.

**Figure:**
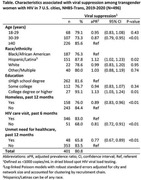
**Abstract OAC0303‐Table 1**.

**Conclusions**: About 1 in 5 transgender women with HIV were not virally suppressed. Programs to support higher education, regular HIV care visits, stable housing, and healthcare costs could assist transgender women with HIV in becoming virally suppressed.

### OAC0304 Using two‐stage inverse probability weights to correct mortality estimates for LTFU among children, adolescents and young adults living with HIV in Southern Africa: results from linkage and multi‐country tracing studies

P. Nyakato ^1^, B. Christ^2^, N. Anderegg^2^, J. Muhairwe^3^, L. Jefferys^4^, J. van Dijk^5^, M. J. Vinikoor^6^, M. van Lettow^7^, C. Chimbetete^8^, S. J Phiri^9^, M. Egger^10,11^, M. Ballif^12^, C.T. Yiannoutsos^13^, R. Kassanjee^14^, M.‐A. Davies^10^, M. Cornell^10^, M. Schomaker^10,15^

^1^Centre for Infectious Disease Epidemiology and Research, University of Cape Town, School of Public Health and Family Medicine, Cape Town, South Africa, ^2^University of Bern, Institute of Social and Preventive Medicine, Bern, Switzerland, ^3^SolidarMed, Maseru, Lesotho, ^4^SolidarMed, Pemba, Mozambique, ^5^SolidarMed, Masvingo, Zimbabwe, ^6^Centre for Infectious Diseases Research in Zambia, Lusaka, Zambia, ^7^Dignitas International, Zomba, Malawi, ^8^Newlands Clinic, Harare, Zimbabwe, ^9^Lighthouse Trust Clinic, Lilongwe, Malawi, ^10^Centre for Infectious Disease Epidemiology and Research, University of Cape Town, Cape Town, South Africa, ^11^Institute of Social and Preventive Medicine, University of Bern, Bern, Switzerland, ^12^Institute of Social and Preventive Medicine, Bern, Switzerland, ^13^R.M. Fairbanks School of Public Health,Indiana University, Department of Biostatistics, Indianapolis, United States, ^14^Centre for Infectious Disease Epidemiology and Research, University of Cape, Cape Town, South Africa, ^15^Institute of Public Health, Medical Decision Making and Health Technology Assessment, UMIT ‐ University for Health Sciences, Medical Informatics and Technology, Tyrol, South Africa

**Background**: Our study examined the correction of mortality estimates based on additional mortality data among children, adolescents and young adults living with HIV (CAYHIV) who were lost in the International epidemiology Databases to Evaluate AIDS (IeDEA‐SA) in six Southern Africa ART programs.

**Methods**: We estimated all‐cause mortality from; five IeDEA‐SA (Lesotho, Malawi, Mozambique, Zambia and Zimbabwe) and Western Cape, South Africa, IeDEA‐SA ART programs. Additional mortality among patients LTFU was ascertained through tracing in the sites outside South Africa and linkage to the Western Cape Provincial Health Data Centre (WCPHDC) and the National Population Register (NPR) in South Africa. We included a total of 85,286 CAYHIV aged 0‐24 years who initiated ART between 2004‐2019. We estimated mortality from ART start using i) routine data without and with additional mortality ascertainment from tracing in sites outside South Africa and iii) routine data in South Africa without and with additional ascertainment from linkage. We used two‐stage inverse probability weighting to correct estimates of mortality for LTFU.

**Results: Tracing study**: Out of a total of 79,867 CAYHIV, 46,375 were defined to be LTFU, of these 680 were randomly sampled for tracing. Of these, 462/680 were successfully traced and vital status ascertained. At two and eight years from ART start, uncorrected mortality was approximately 5% and 12% and after correction: 7% and 20% respectively (Figure 1).

**Linkage study**: There were a total of 5,410 children in routine Western Cape ART programs. Of these, 1,462 were LTFU and 1,048 were successfully linked to the WCPHDC and NPR. At two and eight years from ART start, uncorrected mortality was approximately 5% and 8% and after correction, it was 7% and 12%.

**Conclusions**: Mortality estimates increase when we account for unreported mortality among those LTFU. This is the first long term accurate mortality estimation in CAYHIV in Southern Africa.

**Figure:**
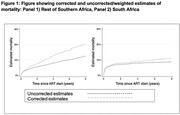
**Abstract OAC0304‐Figure 1**.

### OAC0305 Malawi's progress towards the UNAIDS 95‐95‐95 HIV testing and treatment targets: comparison of the 2015‐16 and 2020‐21 Malawi Population‐based HIV Impact Assessments (MPHIA)

N. Wadonda^1^, D. Payne ^1^, A. Kabaghe^1^, L. Tenthani^2^, C. Braccio^3^, E. Kim^1^, T. Dobbs^3^, M. Nyangulu^1^, A. Jahn^4,5^, K. Brown^3^, N. Kalata^1^, C.A. West^3^, F. Ogollah^2^, F. Kayigamba^2^, A. Auld^1^, G. Bello^4,5^, S.M. Farley^6^, B. Muyunda^7^, K. Mircovic^1^, R. Nyirenda^4^, MPHIA Study Team

^1^U.S. Centers for Disease Control and Prevention, Malawi, Lilongwe, Malawi, ^2^ICAP at Columbia University, Malawi, Lilongwe, Malawi, ^3^U.S. Centers for Disease Control and Prevention, Atlanta, United States, ^4^Ministry of Health, Malawi, Lilongwe, Malawi, ^5^University of Washington, I‐TECH, Seattle, United States, ^6^ICAP at Columbia University, New York, United States, ^7^U.S. Centers for Disease Control and Prevention, Zambia, Lusaka, Zambia

**Background**: The 2015‐16 Malawi Population‐based HIV Impact Assessment showed notable coverage gaps in HIV diagnosis, antiretroviral therapy access and retention, and viral load suppression (VLS). Targeted interventions were implemented by the Government of Malawi, PEPFAR and other partners to close these gaps. The second MPHIA was conducted between January 2020 and April 2021 to measure progress towards the UNAIDS 90‐90‐90 goals.

**Methods**: MPHIA is a nationally representative survey, which enrolled over 23,000 participants. Participants were interviewed and a blood sample was tested for HIV infection using the national algorithm. Results were returned to participants. All HIV positive samples were tested for viral load (VL) and presence of antiretrovirals (ARV); a suppressed VL was defined as <1,000 viral copies per milliliter. All results were weighted and self‐reported awareness and treatment status were adjusted to account for ARV detection results. This analysis was restricted to participants aged 15‐64 years with HIV test results.

**Results**: HIV prevalence decreased significantly from 10.6 % [95% confidence interval (CI): 9.9%‐11.2%] in MPHIA 2015‐16 to 8.9% (95% CI: 8.4%‐9.5%) in MPHIA 2020‐21. Awareness of HIV status among all adults increased from 76.8% (95%CI: 74.7%‐79.0%) to 88.4% (95% CI: 86.7%‐90.1%), with females increasing from 80.2% (95%CI: 77.8%‐82.5%) to 90.4% (95%CI: 88.5%‐92.2%). Among 15‐24‐year‐olds, awareness increased from 53.7% (95%CI: 45.3%‐62.0%) to 76.2% (95%CI: 69.4%‐83.1%). Among all adults aware of their HIV status, ART use increased from 91.4% (95%CI: 89.8%‐93.0%) to 97.8% (95%CI: 97.1%‐98.5%). Viral suppression (VLS) among those on treatment increased from 91.3% (95% CI: 89.3%‐93.3%) to 96.9% (95% CI: 96.0%‐97.8%). Population VLS among all adults living with HIV increased from 68.3% (95%CI: 66.0%‐70.7%) to 87.0% (95%CI: 85.4%‐88.6%). However, VLS remained lowest in the major urban centers of Lilongwe and Blantyre cities, and among participants aged 15–24 years.

**Conclusions**: Targeted investments by district and subpopulation in HIV testing, ART linkage, adherence, and retention have resulted in significant progress towards achievement of the UNAIDS 90‐90‐90 targets. These results show that Malawi has exceeded the more recent UNAIDS 95‐95‐95 treatment and VLS targets. Continued targeted efforts and tailored interventions are needed to close remaining gaps, particularly among young people and in urban centers.

### OAC0402 Determinants of long‐term survival in late HIV diagnosed individuals: the PISCIS Cohort study

R. Martin‐Iguacel ^1,2^, J. Reyes‐Urueña^1^, J. Casabona^1^, A. Bruguera^1^, J. Aceiton Cardona^1^, Y. Diaz Rodriguez^1^, S. Moreno Fornes^1^, P. Domingo^3^, V. Falcó^4^, A. Imaz^5^, I.S. Johansen^2^, J.M. Miró^6^, J.M. Llibre^7^, PISCIS study group

^1^Centre for Epidemiological Studies on Sexually Transmitted Infections and HIV/AIDS of Catalonia (CEEISCAT), Badalona, Spain, ^2^Odense University Hospital, Infectious Diseases, Odense, Denmark, ^3^Hospital Sant Pau, Barcelona, Spain, ^4^Hospital de Vall d' Hebron, Barcelona, Spain, ^5^Hospital de Bellvitge, Barcelona, Spain, ^6^Hospital Clínic‐Institut d'Investigacions Biomèdiques August Pi i Sunyer, University of Barcelona, Barcelona, Spain, ^7^Infectious Diseases Department and Fight AIDS and Infectious Diseases Foundation, University Hospital Germans Trias i Pujol, Badalona, Spain

**Background**: Half of the people living with HIV (PLWH) in Western Countries are still diagnosed late, having a negative impact in their life expectancy and comorbidities. Neither the best determinants of their long‐term mortality nor the potential impact of starting an integrase inhibitors (INSTI)‐based antiretroviral treatment (ART) are completely understood.

We assessed the impact of immune recovery and INSTI‐based ART in their long‐term mortality.

**Methods**: From the PISCIS prospective cohort we included all adult treatment‐naïve PLWH starting ART in 2005‐2020 and surviving the first 2 years. We estimated mortality rates (MR) upon immune recovery 2 years after ART initiation and associated prognostic factors using Poisson regression. We also assessed risk‐factors for incomplete immune recovery at 2 years (defined as CD4 counts ≤500cells/μL) in a nested case‐control study using logistic regression with propensity score matching.

**Results**: We included 2719 persons (15566.8 person‐years of follow‐up); 1441 (53%) were late presenters, decreasing from 78.5% in 2005‐2008 to 40.9% in 2015‐2020 (p<0.01). Among late presenters, 44% achieved CD4 counts >500 cells/μL at 2 years. Overall, 113 patients (4.2%) died (crude all‐cause MR 7.3/1000PY [95%CI:6.0‐8.7]). MR were higher in late compared to non‐late presenters, except for those achieving CD4 counts >500 cells/μL at 2 years (MRR 1.13 [95%CI:0.56‐2.30], independent of nadir CD4 counts (test‐interaction p=0.48).

In multivariate analysis, risk factors for death included: CD4 recovery <500cells/μL (<200cells/μL:aMRR 4.45 [95%CI:2.17‐9.11]; 200‐350cells/μL:aMRR 1.71 [95%CI:0.85‐3.44]; >350‐500cells/μL:aMRR 2.14, [95%CI:1.09‐4.18]); viral load >200 c/ml at 2 years (aMRR 2.04 [95%CI:1.13‐3.68]); Charlson comorbidity index ≥4 (aMRR 4.11 [95%CI:1.90‐8.86]), heterosexual men (aMRR 1.97 [95%CI:1.12‐3.46]) and injection drug use (aMRR 2.60 [95%CI:1.37‐4.95]).

Overall, 979 PLWH initiated an INSTI‐based regimen, which was associated with a trend towards decreased mortality compared to other regimens (aMRR 0.60 [95%CI:0.34‐1.05]) and with favorable immune recovery (CD4 counts >500cells/μL, aOR 0.70 [95%CI:0.54‐0.90]).

No significant changes in MR were observed over calendar time.

**Conclusions**: ART‐associated immune recovery at 2 years was a better predictor of long‐term mortality than nadir baseline CD4 counts in late ART initiators. Nearly half experienced a favorable immune recovery with a life expectancy similar to non‐late presenters. INSTI‐based regimens were associated with higher rates of successful immune recovery and survival.

### OAC0403 High HIV incidence and mortality in a multi‐site cohort of transgender women in the eastern and southern United States

A.L. Wirtz ^1^, E. Humes^2^, K.N. Althoff^2^, T. Poteat^3^, A. Radix^4^, K.H. Mayer^5,6^, C. Beyrer^1^, J. Schneider^7^, J.S. Haw^7^, A.J. Wawrzyniak^8^, A.E. Rodriguez^9^, D. Adams^1^, M. Stevenson^1^, E.E. Cooney^10^, S.L. Reisner^5,11,12,13^

 

**Figure:**
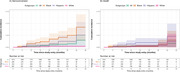
**Abstract OAC0403‐Figure 1**.

^1^Johns Hopkins University School of Public Health, Department of Epidemiology, Center for Public Health and Human Rights, Baltimore, United States, ^2^Johns Hopkins University, Epidemiology, Baltimore, United States, ^3^University of North Carolina School of Medicine, Center for Health Equity Research, Chapel Hill, United States, ^4^Callen‐Lorde Community Health Center, New York, United States, ^5^Fenway Health, The Fenway Institute, Boston, United States, ^6^Harvard Medical School, Beth Israel Deaconess Medical Center, Boston, United States, ^7^Emory University, School of Medicine, Atlanta, United States, ^8^University of Miami Miller School of Medicine, Department of Psychiatry and Behavioral Sciences, Miami, United States, ^9^University of Miami, Miller School of Medicine, Division of Infectious Diseases, Miami, United States, ^10^Johns Hopkins University, School of Public Health, International Health, Baltimore, United States, ^11^Brigham Women's Hospital, Division of Endocrinology, Diabetes, and Hypertension, Boston, United States, ^12^Harvard University, Department of Medicine, Harvard Medical School, Boston, United States, ^13^Harvard University, Harvard TH Chan School of Public Health, Department of Epidemiology, Boston, United States

**Background**: Transgender women are a priority population in the US HIV strategy due to social vulnerabilities and HIV burden, yet epidemiologic monitoring of HIV, premature death, and other events to inform public health is almost non‐existent.

**Methods**: We established a multi‐site cohort for transgender women in eastern and southern US across two arms: 1) technology‐enhanced site‐based (Boston, New York City, Baltimore, Washington DC, Atlanta, Miami); 2) exclusively online (spanning 72 matched cities). Eligibility criteria: transfeminine; ages>=18 years; negative baseline HIV test; not in a PrEP trial. Participants were followed for >=24 months, completing surveys, rapid oral fluid HIV tests with confirmatory testing referrals and medical record reviews. Retention efforts (e.g., comprehensive locator, community outreach, events) permitted other event ascertainment, including death. HIV incidence and mortality rates were estimated as the number of observed events (HIV seroconversions or deaths) divided by the number of person‐years(py) accumulated. We visualized Kaplan‐Meier estimates of cumulative incidences (Figure) with a time‐to‐event approach that defines time‐of‐origin as study entry.

**Results**: Enrollment launched March 2018 in the site‐based arm and January 2019 in the online arm. 1,313 participants were enrolled with 83% retention, 2,479 person‐years accumulated, and 12 identified seroconversions as of December 2021. HIV incidence was 4.8/1,000py (95%CI:2.1‐7.6) overall and by group: online IR:1.8/1,000py, site‐based IR:7.3/1,000py, Black participants IR:15.9/1,000py, Latinx participants IR:8.6/1,000py, and residence in South IR:8.6/1,000py. Seven deaths were identified (attributed causes: homicide, suicide, overdose, unknown). Mortality rates were 2.8/1,000py overall (95%CI:1.1‐5.8); site‐based: 4.4/1,000py and online: 0.9/1,000py, 3.5/1,000py in Black and 8.6/1,000py in Latinx participants.

**Conclusions**: HIV incidence and mortality are high in transgender women, disparate across race and ethnicity, and underscore community calls to for combination approaches that address structural and other health concerns alongside HIV. Differences across cohort arms highlight the need for continued community and location‐based efforts as HIV research and interventions are increasingly delivered online.

### OAC0404 Prevalence and individual and community‐level risk factors of late diagnosis among newly diagnosed people living with HIV from nine African countries

K. Ganesan ^1^, W. El‐Sadr^1^, A. Low^1^

^1^Columbia Univeristy, Epidemiology, New York, United States

 

**Figure:**
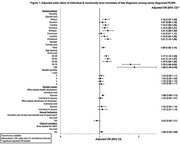
**Abstract OAC0404‐Figure 1**.

**Background**: People living with HIV (PLWH) with late diagnosis (LD) (CD4 cell count <350 cells/mm^3^ and no prior HIV diagnosis) are at higher risk of opportunistic infections, non‐AIDS defining comorbidities, and death compared to stable patients due to delayed diagnosis. We used Population‐based HIV Impact Assessment (PHIA) survey data from Cameroon, Eswatini, Ethiopia, Lesotho, Malawi, Tanzania, Uganda, Zambia, and Zimbabwe to examine LD prevalence and identify individual and community‐level correlates of LD.

**Methods**: The PHIAs are cross‐sectional, household‐based surveys that use two‐stage sampling to collect nationally representative data from adults aged ≥15 years. Between 2015‐2017, data from interviews, home‐based HIV testing, and laboratory testing were collected. Blood samples were analyzed for HIV RNA, detectable antiretrovirals, and CD4+ cell counts. Community‐level variables were generated at each enumeration area‐level using weighted data. Logistic regression using fixed‐effects to account for cross‐country variation was used to determine individual and community‐level factors associated with LD in adults aged ≥15 years.

**Results**: Of 4,408 newly diagnosed PLWH, 42.8% (95% CI: 40.9‐44.8) had LD. LD prevalence ranged from 29.6% (95% CI: 25.3%‐34.3%) in Uganda to 53.3% (95% CI: 49.5%‐57.1%) in Zimbabwe. Newly diagnosed PLWH who resided in higher LD prevalence countries such as Ethiopia, Lesotho, Malawi, Tanzania, Zambia, and Zimbabwe, were older, of male sex, and had never tested for HIV, had higher adjusted odds of LD (Figure 1). PLWH in communities where gender norms supported a lack of health‐related decision‐making autonomy, had higher adjusted odds of LD.

**Conclusions**: Late diagnosis of HIV remains a challenge despite increases in HIV testing services. Such services should highlight the importance of early diagnosis of HIV and for individuals, particularly older men in high LD prevalence countries, to get repeatedly tested. Gender norms that inhibit health agency should be addressed by providing community‐based support for promotion of health autonomy to optimize testing services.

### OAC0405 Prevalence of adverse birth outcomes and external birth defects among women living with HIV in Malawi

G. Bello^1,2^, J. Smith‐Sreen ^3^, D. Williamson^4^, F. Taulo^5^, A.N. Kabaghe^6^, K. Thomson^7^, M. Kagoli^2^, S. Chipeta^2^, R. Nyirenda^2^, Y. Babaye^1^, A.F. Auld^6^, E. Kim^6^, B. Matatiyo^8^, E. Zenengeya^8^, A.S. Muula^5^, I. Nyasulu^9^, M.F. Gomes^10^, L. Chiwala^1^, M. Kamzati^1^, J. Mkungudza^2,1^, D. Valencia^4^, C. Moore^4^, G. O'Malley^7^, N. Wadonda‐Kabondo^6^

^1^International Training and Education Centre for Health, Lilongwe, Malawi, ^2^Ministry of Health, Lilongwe, Malawi, ^3^PHI/CDC Global Health Fellowship Program, Lilongwe, Malawi, ^4^Centers for Disease Control and Prevention, Atlanta, Georgia, ^5^Kamuzu University of Health Sciences, Blantyre, Malawi, ^6^Centers for Disease Control and Prevention‐Malawi, Lilongwe, Malawi, ^7^The University of Washington, Seattle, United States, ^8^National AIDS Commission, Lilongwe, Malawi, ^9^World Health Organization, Lilongwe, Malawi, ^10^World Health Organization, Geneva, Switzerland

**Background**: Routine surveillance for birth outcomes is essential to monitor safety of antiretroviral therapy (ART) during pregnancy among women living with HIV (WLHIV). We examined the prevalence of adverse birth outcomes and major external birth defects (BDs) by maternal HIV and ART status in Malawi.

**Table:** 

**Abstract OAC0405‐Table 1**.					
**Outcome**	**Women living with HIV on ART** N = 16,349 (9.9%)	**ART naïve women living with HIV** N = 321 (0.2%)	**HIV‐negative women** N = 147,399 (89.1%)	**Women with unknown HIV status** N = 1,333 (0.8%)	**Total** N = 165,402 (100.0%)
**Premature delivery (<37 weeks)** (prevalence per 100 births)	**21.4** (20.8, 22.1)	**28.3** (23.5, 33.6)	**19.2** (19.0, 19.4)	**27.2** (24.8, 29.6)	**19.5** (19.3, 19.7)
**Low birthweight (<2500g)** (prevalence per 100 births)	**14.6** (14.0, 15.1)	**18.4** (14.3, 23.1)	**11.9** (11.8, 12.1)	**19.2** (17.1, 21.4)	**12.3** (12.1, 12.4)
**Selected external birth defects under surveillance** (prevalence per 10,000 births)	**60.6** (49.2, 73.7)	**31.2** (0.8, 172.3)	**48.2** (44.8, 51.9)	**75.0** (36.0, 137.5)	**49.6** (46.3, 53.1)
–Neural tube defects (anencephaly, encephalocele, spina bifida)	**15.9** (10.4, 23.3)	0.0	**8.6** (7.2, 10.3)	0.0	**9.3** (7.8, 10.8)
–Orofacial clefts (cleft lip with and without cleft palate)	**0.6** (0.01, 3.4)*	0.0	**1.1** (0.6, 1.8)	0.0	**1.0** (0.6, 1.6)
–Hypospadias	**11.0** (6.5, 17.4)	0.0	**7.9** (6.6, 9.5)	**7.5** (0.2, 41.7)	**8.2** (6.9, 9.7)
–Talipes equinovarus (clubfoot)	**26.9** (19.6, 36.1)	**31.2** (0.8, 172.3)	**20.5** (18.2, 22.9)	**52.5**(21.1, 107.9)	**21.4** (19.3, 23.8)
–Limb reduction defects	**2.4** (0.7, 6.3)	0.0	**1.2** (0.7, 1.9)	0.0	**1.3** (0.8, 2.0)
–Gastroschisis and omphalocele	**3.1** (1.0, 7.1)	0.0	**2.5** (1.8, 3.5)	0.0	**2.5** (1.8, 3.4)

**Methods**: Adverse birth outcomes (prematurity, low birthweight) and BDs were recorded for all live and stillbirths delivered at four Malawian hospitals from January 2016 to July 2020 and July 2021 to November 2021. BDs were confirmed by experts at the Centers for Disease Control and Prevention. Maternal characteristics were collected from interviews and health records. Pooled prevalence and crude prevalence ratios (cPRs) were calculated using maximum likelihood estimates for adverse outcomes and BDs.

**Results**: Among 165,402 women with informative births, the median age was 24.0 years (IQR: 20.0‐30.0) and 10.1% were HIV‐positive. The prevalence of prematurity and low birthweight, respectively, was significantly higher for the following populations: ART naïve WLHIV (28.3%, 18.4%), WLHIV on ART (21.4%, 14.6%) and women with unknown HIV status (27.2%, 19.2%) than HIV‐negative women (19.2%, 11.9%). The most prevalent BDs (excluding syndromes) were talipes equinovarus (21.4 per 10,000 births, 95% CI: 19.3, 23.8), neural tube defects (NTDs) (9.3, 95% CI: 7.8, 10.8), and hypospadias (8.2, 95% CI: 6.9, 9.7); higher prevalence of these conditions was observed among WLHIV on ART than HIV‐negative women. There was a slightly higher likelihood of WLHIV on ART delivering a baby with an NTD than HIV‐negative women (cPR: 1.85, 95% CI: 1.07, 2.62).

**Conclusions**: Higher prevalence of adverse birth outcomes and BDs was observed among HIV‐positive women. Further analyses are needed to understand the impact of a COVID‐related data collection pause between 2020 and 2021, and to explore risk factors of HIV and ART status by ART regimen and timing for adverse outcomes and BDs among WLHIV in Malawi.

### OAC0502 Association of prenatal PrEP exposure with neurodevelopmental and growth outcomes beyond 24 months among Kenyan children

L. Gómez ^1^, J. Kinuthia^2^, A. Larsen^1^, J. Stern^1^, J. Dettinger^1^, M. Marwa^2^, F. Abuna^2^, B. Ochieng^2^, N. Ngumbau^2^, P. Omondi^2^, G. John‐Stewart^1^, J. Pintye^1^

1University of Washington, Seattle, United States, ^2^Kenyatta National Hospital, Nairobi, Kenya

**Background**: Safety data of prenatal PrEP use are reassuring, yet studies to date have less than 1 year of follow‐up and do not assess neurodevelopmental outcomes among PrEP‐exposed infants. Evaluating safety outcomes beyond infancy following maternal PrEP use could help complete the safety profile for PrEP use during pregnancy.

**Methods**: We utilized data from mother‐child pairs enrolled in an ongoing evaluation of perinatal PrEP use in Western Kenya. In the parent study (NCT03070600), HIV‐negative women were enrolled and offered PrEP during pregnancy at 20 public sector maternal child health (MCH) clinics and followed through 9 months postpartum regardless of PrEP status. An extension cohort to evaluate safety outcomes enrolled mother‐child pairs at 4 sites to be followed until the child's 5^th^ birthday. Between October 2020 and January 2022, trained study nurses conducted anthropometric measurements on children and assessed neurodevelopment using the Ages and Stages Questionnaire (ASQ), an early developmental screener. Using data from 24‐30 months, we evaluated the association of prenatal PrEP exposure and growth and ASQ scores using linear regression models, clustered by facility, and adjusted for gestational age at birth.

**Results**: Among 472 mother‐child pairs included in the analysis, median maternal age was 27.8 years (IQR: 24.6‐33.0) and median child age was 25 months (IQR: 21‐28) at enrollment into the extension cohort; 16.3% had any PrEP exposure during pregnancy for a median duration of 3.0 months (IQR: 2.0‐4.3).At 24‐month visits, there was no difference in mean weight (mean difference −0.04 kg, 95% CI: −0.78, 0.70, p=0.886), mean height (mean difference ‐0.43 cm, 95% CI: ‐2.32, 1.46, p=0.520), frequency of underweight (5.6% vs. 4.0%, adjusted prevalence ratio[aPR]=1.49, 95% CI: 0.27‐8.09, p=0.647) and frequency of stunting (22.2% vs. 21.4%, aPR=1.07, 95%CI: 0.64‐1.78, p=0.805) between children with and without prenatal PrEP exposure. Results were similar at 30‐month visits. Prenatal PrEP exposure was not associated with overall ASQ scores at 24‐months (p=0.243) or 30‐months (p=0.664).

**Conclusions**: Among Kenyan mother‐child pairs followed from pregnancy through early childhood, we found no differences in growth or neurodevelopmental outcomes between children with and without prenatal PrEP exposure. Our results support prior data indicating safety of prenatal PrEP use.

### OAC0503 Introduction of gain‐framed pre‐exposure prophylaxis counseling increased uptake among transgender women at the Tangerine Clinic, Bangkok, Thailand

P. Srimanus ^1^, P. Parkpien^1^, R. Janamnuaysook^1^, K. Samitpol^1^, A. Chancham^1^, J. Kongkapan^1^, T. Amatsombat^1^, J. Rueannak^1^, P. Getwongsa^1^, W. Tasomboon^1^, M. Bucha^1^, M. Avery^2^, S. Mills^2^, P. Phanuphak^1^, N. Phanuphak^1^, R. Ramautarsing^1^

^1^Institute of HIV Research and Innovation, Bangkok, Thailand, ^2^FHI 360, Bangkok, Thailand

**Background**: Transgender women continue to be disproportionally affected by HIV in Thailand. Pre‐exposure prophylaxis (PrEP) remains underutilized by transgender women despite its efficacy and availability due to several barriers, including stigma related to sexual behaviors and to taking PrEP. Loss‐framing counseling, in which risk behavior is emphasized, further exacerbates PrEP stigma and can impede PrEP uptake. In June 2020, Tangerine Clinic in Bangkok, Thailand, introduced gain‐framed PrEP counseling to encourage clients to focus on protection and healthy behavior and ultimately increase PrEP uptake. Here we assess PrEP uptake before and after implementation of gain‐framed PrEP counseling.

**Methods**: We analyzed data from transgender women visiting Tangerine Clinic from June 2020 through July 2021, including demographic and sexual behavioral characteristics from self‐administered questionnaires, data related to HIV and sexually transmitted infections (STIs) (syphilis, gonorrhea, chlamydia). Although not the focus of counseling messages, HIV risk was determined by asking about self‐perceived HIV risk and through self‐reported behavioral risk factors. PrEP uptake during this period was compared to uptake before implementation of gain‐framed counseling (June 2019–May 2020).

**Results**: From June 2020 to July 2021, 1,149 transgender women not on PrEP visited Tangerine, of whom 398 (34.6%) accepted PrEP after gain‐framed counseling. Median age was 26.2 years. A total of 319 (80.2%) were new initiations, and 79 (19.8%) restarted PrEP after previous discontinuation. Among the 398 transgender women accepting PrEP, 106 (26.6%) did not perceive themselves to be at risk for HIV, while 41/106 (38.7%) reported risk behaviors and 26/106 (24.5%) were diagnosed with an STI. In the year before gain‐framed counseling implementation, PrEP uptake was 161/1,191 transgender women (13.5%), with 13 transgender women having no self‐perceived risk accounting for 8.1% of initiations, indicating an increase in PrEP uptake of 147% overall, and a 715% increase among transgender women without self‐perceived HIV risk.

**Conclusions**: Replacing traditional risk‐based counseling with empowering messages focusing on health improved PrEP uptake overall, particularly among transgender women without self‐perceived risk, despite a discrepancy between self‐perceived and actual HIV risk. Gain‐framed messages should be integrated with PrEP counseling to optimize PrEP use and its impact on the HIV epidemic.

### OAC0504 Evaluating adaptive HIV pre‐exposure prophylaxis adherence interventions for young South African women: results from a sequential multiple assignment randomized trial

J. Velloza ^1^, N. Poovan^2^, A. Meisner^3^, N. Ndlovu^2^, N. Khoza^2^, J. Morton^1^, J. Omony^2^, E. Mkwanazi^2^, C. Grabow^1^, D. Donnell^3^, J. Baeten^4^, S. Hosek^5^, C. Celum^1^, S. Delany‐Moretlwe^2^

^1^University of Washington, Global Health, Seattle, United States, ^2^Wits Reproductive Health & HIV Institute, Johannesburg, South Africa, ^3^Fred Hutchinson Cancer Research Center, Seattle, United States, ^4^Gilead Sciences, Foster City, United States, ^5^Stroger Hospital of Cook County, Chicago, United States

**Background**: Pre‐exposure prophylaxis (PrEP) is a highly effective HIV prevention strategy for adolescent girls and young women (AGYW). Widespread PrEP delivery will require identification of layered support strategies for AGYW with diverse needs. We conducted the first sequential multiple assignment randomized trial (SMART) to evaluate stepped PrEP adherence support interventions for AGYW in South Africa.

**Methods**: “PrEP SMART” was conducted in Johannesburg from 2019–2022. Sexually active, HIV‐negative women ages 18–25 years were offered PrEP and randomized to receive standard PrEP counseling with either weekly two‐way SMS or WhatsApp support. Those with low PrEP adherence through Month 2 (“non‐responders”) were re‐randomized to quarterly visits with drug‐level feedback (DLFB) or monthly visits with issue‐focused counseling. The primary outcome was high adherence (tenofovir diphosphate [TFV‐DP] ≥700 fmol/punch) from dried blood spots (DBS) at Month 9. We assessed the effects of the initial interventions on TFV‐DP at Month 9, the effects of the intensified interventions among non‐responders, and the optimal intervention sequence.

**Results**: Of 360 AGYW, the median age was 21, 31.4% had sexually transmitted infections at enrollment, and 77.5% were retained through Month 9 despite COVID‐19 disruptions. Of those with DBS, 58.6% (N=164) had TFV‐DP ≥700 fmol/punch at Month 2 and 24.7% (N=66) at Month 9. At Month 9, 34/133 (25.6%) AGYW in the two‐way SMS arm and 32/134 (23.9%) in the WhatsApp arm had high PrEP adherence (relative risk [RR]=1.07; 95% confidence interval [95% CI]=0.70–1.63; p=0.75). Among non‐responders, 4/49 (8.2%) in the DLFB arm and 3/51 (5.9%) in the monthly counseling arm had high adherence at Month 9 (RR=1.39; 95% CI=0.33–5.88; p=0.66). Across the four dynamic treatment strategies, the estimated probability of high adherence at Month 9 was 23–27% (p=0.94).

**Conclusions**: In this study, PrEP adherence was higher than in comparable cohorts, despite the COVID‐19 pandemic. Re‐engaging non‐responders after two months was challenging; individual‐level interventions may not overcome structural PrEP barriers suggesting that longer‐acting PrEP formulations may benefit this population. Our data show that individual tailored adherence approaches (SMS, WhatsApp) have similar impact on PrEP adherence and PrEP programs can adopt approaches based on likelihood of scalability.

### OAC0505 Pragmatically approaching social network testing (SNT): using a peer‐driven community outreach model to extend reach of HIV testing services (HTS) to networks of people who inject drugs (PWID) in Ukraine

S. Leontieva ^1^, T. Gaborets^1^, N. Roman^1^, K. Gamazina^1^, D. Canagasabey^2^

^1^PATH, Ukraine Country Program, Kyiv, Ukraine, ^2^PATH, Washington, DC, United States

**Background**: The HIV prevalence among PWID in Ukraine is 20.3%, up to four times higher than other key population groups, reinforcing the need for focused HIV case‐finding and linkage strategies that tap into PWID networks (2020 IBBS). The USAID/PATH Serving Life project introduced and scaled a peer‐driven community outreach model, leveraging SNT principles to reach contacts of PWID peer case‐finders with HTS in 12 oblasts.

**Description**: Project‐supported non‐governmental organizations (NGO) hired peer case‐finders among former or soon‐to‐be released PWID prisoners as seed recruiters to mobilize social, sexual, and drug injecting contacts for HTS. Peer case‐finders and NGO social workers provided HIV counseling, offered HIV self‐testing (HIVST) services, followed up with recruited peers to confirm HIVST results, and provided referrals for treatment or prevention services. Newly identified HIV‐positive peers (first outreach wave) offered index testing services to sexual/injecting partners and biological children (second outreach wave), and could be hired as peer case‐finders themselves. In 2021, to maximize reach among hidden PWID networks, NGOs prioritized hiring in underserved geographies and more frequently rotated case‐finders to source new networks. We analyzed program data from October 2020 through September 2021 to understand the model's success in reaching HIV‐positive PWID and their contacts.

**Lessons learned**: PWID peer case‐finders mobilized 10,184 peers and their contacts for HTS, among whom 333 were confirmed HIV‐positive (3.3% positivity) and 95% initiated on treatment. The second outreach wave was more efficient at reaching PWID contacts more likely to be HIV‐positive (testing positivity: 10.6% [second wave] versus 1.3% [first wave]; see table), given targeted outreach through index testing. There was also an increase in both volume of people tested and confirmed HIV‐positive in 2021 (versus 2020) due to refinements to the SNT model.

**Table:** 

**Abstract OAC0505‐Table 1**.			
	**First wave (PWID peers)**	**Second wave (index testing)**	**Total**
**# tested**	8,035	2,149	10,184
**# confirmed HIV‐positive**	106	227	333
**% testing positivity**	1.3%	10.6%	3.3%

**Conclusions/Next steps**: These results highlight the promise of using a peer‐driven, SNT‐based outreach model to efficiently tap into networks of HIV‐positive PWID and their contacts and link them to HIV services. Further expansion of this approach to reach PWID and their partners/contacts with HIV services is essential to achieving epidemic control in Ukraine.

### OAD0102 Turning the tide towards eliminating mother to child transmission: lessons from Murang'a County Government HIV program

J. Kisio ^1^, D.G. Kinyanjui^1^, C. Ochola^2^, E. Karanja^3^

^1^Murang'a County Government, Murang'a, Kenya, ^2^Center for Health Solutions, Nairobi, Kenya, ^3^Aids Health Foundation, Nairobi, Kenya

**Background**: According to the Kenya National Aids Control Council (NACC) 2017 HIV estimates, mother to child transmission rate for Murang'a county stood at 20.4% way above the national rate of 11.5%, in that same year 44 babies in the county were confirmed to be infected with HIV through mother to child transmission. With the help of our local implementing partners; Center for Health solutions (CHS) and Aids Health Foundation (AHF), the county HIV program embarked on a mission towards reducing the mother to child transmission rates to a rate equal or less than the national level.

**Description**: Health workers (HIV testing service providers, nurses and clinical officers) working in the identified 199 prevention of mother to child transmission (PMTCT) clinics were comprehensively trained on the national PMTCT guidelines with the emphasis made on the four prongs of PMTCT which are; keeping HIV negative women negative, prevention of unintended pregnancy for the already positive women, test and treat strategy for HIV positive pregnant women with issuance of prophylaxis for their HIV exposed infants and finally care and treatment for both the positive mother and the child. All pregnant women are tested for HIV at their first antenatal visit (ANC) and if they test negative, the HIV test is repeated at 3rd trimester, labor and delivery, at 6 weeks post natal clinic and after every six months during breastfeeding. Audits are done (including maternal) for all HIV exposed infants (HEI) who turn HIV positive to determine likely root for mother to child transmission.

**Lessons learned**: Through this strategy, the number of HIV exposed infants turning positive have been gradually declining as follows 44 in 2017, 27 in 2018, 18 in 2019, 16 in 2020 and 5 in 2021. The MTCT rate has also reduced to 9% according to the National Aids Control Council (NACC) 2020 estimates. Murang'a county has also been listed among the counties on the right gear towards elimination of mother to child transmission.

**Conclusions/Next steps**: Elimination of mother to child transmission can be achieved when all health care workers receive the right training and are supported to offer quality services as prescribed by national guidelines.

### OAD0103 The protective association of social cohesion on sex workers’ experiences of violence and access to tailored services: findings of a community‐based cohort in Vancouver, Canada (2010‐2019)

J. Pearson ^1,2^, K. Shannon^3,2^, D. Kerrigan^4^, A. Krüsi^2,3^, M. Brachel^2^, S. Goldenberg^5,2,6^

^1^University of British Columbia, Interdisciplinary Studies Graduate Program, Vancouver, Canada, ^2^Centre for Gender and Sexual Health Equity, Vancouver, Canada, ^3^University of British Columbia, Department of Medicine, Vancouver, Canada, ^4^George Washington University, Department of Prevention and Community Health, Washington, DC, United States, ^5^San Diego State University, Division of Epidemiology and Biostatistics, School of Public Health, San Diego, United States, ^6^Simon Fraser University, Faculty of Health Sciences, Burnaby, Canada

**Background**: While community mobilization and social cohesion have been identified as key drivers of HIV prevention and improved safety for sex workers in the global south, we know less about social cohesion's impacts on safety and access to community‐driven HIV prevention services in North America and under partial‐criminalization models. COVID‐19, in addition, has highlighted the critical need and role of community supports. Our aim was to measure recent (in the last six months) social cohesion (perceptions of mutual aid, trust and support) and its association with (1) sexual/physical violence, and (2) engagement with sex work‐specific services (e.g., drop‐in spaces, HIV/harm reduction outreach) among women sex workers in Metro Vancouver, Canada.

**Methods**: Prospective data (January 2010‐August 2019) were drawn from an open cohort, operated by experiential and community‐based staff, of 900+ women sex workers across diverse work environments (An Evaluation of Sex Workers' Health Access). We used multivariable logistic regression confounder models with generalized estimating equations (GEE) for repeated measures to examine the association between social cohesion and recent outcomes of (1) physical/sexual violence and (2) use of sex work‐specific services, over a ten‐year period.

**Results**: The study sample included 860 sex workers, of whom 315 (36.6%) were Indigenous and 283 (32.9%) Black/Women of Colour. Overall, 36.4% identified as a sexual minority and 8.0% as gender‐diverse. At baseline, the median social cohesion score was 19 (IQR 15‐22), out of a possible 36. In bivariable GEE analysis, increased social cohesion was associated with formal indoor work environments, good self‐rated health, and working with other sex workers as a safety strategy, and was negatively associated with living with HIV. In separate multivariable GEE confounder models, social cohesion was independently associated with lower odds of recent physical/sexual violence (Adjusted Odds Ratio (aOR) 0.99 per point on scale, 95% Confidence Interval (CI) 0.97, 1.00) and increased odds of recently using sex work‐specific services (aOR 1.02 per point on scale, 95% CI 1.00, 1.04).

**Conclusions**: The findings affirm community calls to fully decriminalize sex work to better promote sex workers’ social cohesion, physical safety and access to tailored, sex work‐specific sexual health and HIV services.

### OAD0104 The efficiency of index contact testing approach in community HIV case identification

A. Hoejrup^1^, M. Lichtenberg ^2^, A. Zulu^1^, J. Kanyanda^3^, E. Zimunhu^4^

^1^Development Aid from People to People in Zambia, HQ, Lusaka, Zambia, ^2^Planet Aid Inc., Elkridge, Maryland, United States, ^3^DAPP Zambia, Total Control of the Epidemic (TCE) in Southern Province, Pemba, Zambia, ^4^Federation Humana People to People, HQ, Shamwa, Zimbabwe

**Background**: Approximately 1.2 million people in Zambia are living with HIV. It is estimated that 92% of PLHIV know their HIV status while there is still 8% unaware that they have HIV, thereby posing risk for further HIV transmission. To speedily mitigate transmission resulting from those unaware of their HIV positive status, a more efficient community‐based HIV case finding approach is essential.

**Description**: DAPP in Zambia partners with the Ministry of Health to implement the Total Control of the Epidemic (TCE) project, which has been under implementation since 2006 and currently implemented in four provinces. The project employs two community‐based HIV testing approaches, namely; 1) Venue/Hotspot HIV testing, which involves testing individuals in their work venues such as fishing camps, etc. and 2) Index Testing Services (ITS). The latter involves:

- Identifying known HIV positives, offering them ITS, and eliciting their sexual partners and biological children otherwise known as “Contacts”;
- Screening for intimate partner violence and tracing their named “Contacts”;
- Screening the traced “Contacts” for HIV testing;
- Testing and linking newly HIV positive diagnosed contacts to treatment (ART);

Offering ITS to new positives and named known HIV positive “Contacts.”

**Lessons learned**: Results in Lusaka Province, Oct 2020‐Sept 2021.

*Venue/Hotspot HIV testing*: Under this approach, 5,854 were tested for HIV and 1,476 (25%) were diagnosed HIV positive. 98% of all HIV positives were successfully initiated on ART.

*Index Testing Services*: Using this approach, 22,332 PLHIV were offered ITS and 99% accepted the service. 53,974 contacts were named of which 24% had a known HIV positive status. 69% of the contacts named were eligible and tested for HIV. Of those tested,11,899 (31%) were diagnosed HIV positive and 99.5% of the positives were successfully initiated on ART.

**Conclusions/Next steps**: ITS had a comparatively higher positivity rate of the two approaches, proving to be a more efficient community‐based HIV case finding approach than Venue/Hot Spot testing. Successful implementation of the ITS approach was due in large part to the exceptional psychosocial counseling and contact elicitation skills of DAPP TCE's psychosocial counselors.

### OAD0105 Working with cultural leaders to create demand for HIV services: lessons from an engagement with Buganda Kingdom in Uganda in April 2020

D. Kwarisiima^1^, R. Kabanda^1^, J. Musinguzi^1^, A. Mukundane ^2^, D. Atwine^1^

^1^Ministry of Health, Kampala, Uganda, ^2^John Hopkins University CCP, Kampala, Uganda

**Background**: Data from routine surveillance and population surveys indicate that HIV prevalence amongst adults reduced from a peak of 18% in the 1990s to 6.2% in 2016. New HIV infections declined from 160,000 in 2010 to 52,000 in 2018. Vertical infections declined from 25,000 in 2010 to less than 4,000 in 2018. Furthermore, AIDS related mortality declined from approximately 50,000 to 25,000 in 2018. However, the central region continues to have a higher prevalence despite the combination of high impact evidence based behavioral, structural and biomedical interventions. Ministry of Health (MOH) engaged the Buganda Kingdom leadership to support in mobilising their communities to utilise available HIV services.

**Description**: MOH together with the Buganda kingdom leadership conducted an orientation of 180 cultural leaders/ gatekeepers, who include kingdom ministers, clan heads, county chiefs, and youth leaders. The orientation equipped these leaders/ gatekeepers with Key HIV information so that they can aide the scaling down of HIV messaging in the central region community. Following this orientation, the leaders organized fireplace discussions (ebyooto) in 118 sub counties in Buganda kingdom reaching over 4,720 young people.The trained elders imparted knowledge of HIV Prevention, care and treatment services upon the selected youth.There is evidence that the young people did not only listen to information from their elders, but they understood it, acted on it and it changed their behavior. For example, there were less teenage pregnancies and increased uptake of HIV testing services in the central region as compared to other regions.

**Lessons learned**: Cultural leaders can play a significant role in behavior change however; they need to be exemplary in their way of life to influence young people. The cultural leaders also reported that use of appropriate/slang language while communicating about HIV, was more acceptable and relatable to the young people. Finally, the cultural leaders had significant respect and a big opportunity to continuously engage their subjects on wider health issues.

**Conclusions/Next steps**: If cultural leaders are supported to integrate HIV awareness activities in their regular programs the results can be immense and as custodians of norms and cultural practices, they can successfully influence behaviors.

### OAD0202 "I feel that things are out oF my hands": the impact of the COVID‐19 prevention measures on the lives of young people living with HIV (YPLHIV) in Uganda

B. Ssekajja ^1^, R. Both^2^

^1^Reproductive Health Uganda, Gender and Youth Department, Kampala, Uganda, ^2^Rutgers, International Department, Amsterdam, Netherlands, the

**Background**: With an estimated 1.4 million PLHIV In Uganda, youth account for 170,000 infections. These are expected to rise as the youth remain highly vulnerable to the infection. Analyses conducted by the Uganda Harm Reduction Network (UHRN) in July 2020 on the effects of the COVID‐19 pandemic showed a decline in access to HIV prevention information, services and Psychosocial support. This particular study was conducted among various groups of youth, and put emphasis on YPLHIV to examine how the COVID‐19 prevention measures affected their access to HIV care information and services.

**Methods**: In October 2020, Rutgers coordinated a study among youth aged 18‐30, using a mobile web survey to collect quantitative data from 640 respondents (326 males; 314 females), and Focus Group Discussions (FGDs) to collect qualitative data from 39 Youth (14 males, 22 females, 3 non‐binary) in four districts of Eastern Uganda. Participants included YPLHIV, teen mothers, students, and LGBTQI. Quantitative data were analyzed using descriptive statistics, while qualitative data were analyzed using a grounded theory approach which allowed for the identification of common patterns and salient themes

**Results**: COVID‐19 interconnectedly, negatively affects YPLHIV, as responses revealed that they experienced worse outcomes than those living without HIV. Evidence shows that for YPLHIV, adherence to prevention measures was sometimes misinterpreted by others as being infected with COVID‐19. Given the stigma associated with them, such accusations are difficult to bear. In vain, 65% of YPLHIV said they needed ART services, and 30% said they needed information regarding STDs/STIs. Additional study findings indicate that the prolonged prevention measures have resulted in a reduction in HIV testing services and ART initiation.

**Conclusions**: Clearly, the pandemic negatively impacted YPLHIV, presenting concerns of double stigma if they test positive for COVID‐19, and increased psychosocial afflictions caused by stress and isolation. Dealing with 2 pandemics resultantly affects their mental health, presenting another risk of additional barriers to care, potentially leading to further disenfranchisement. Therefore, addressing such inequalities, challenges and barriers is critical to maintaining continuity of care and strong psychosocial support systems and YPLHIV recommended that mitigating the Pandemic's direct impact on access to HIV information and services is key.

### OAD0203 Impact of COVID‐19 on economic well‐being, mental health and HIV risk among MSM: a mixed methods study in a north Indian city

A. Sebastian ^1^, V. Chakrapani^2^, P. A. Newman^3^, S. Rawat^4^, M. Shunmugam^2^, S. Mittal^5^, V. Gupta^5^, M. Kaur^6^, G. Siddhant^4^, D. Kumar^4^, D. Singh^4^, S. Romio^4^

^1^National Institute of Advanced Studies (NIAS), Bangalore, India, ^2^Centre for Sexuality and Health Research and Policy (C‐SHaRP), Chennai, India, ^3^University of Toronto, Factor‐Inwentash Faculty of Social Work, Ontario, Canada, ^4^The Humsafar Trust, Mumbai, India, ^5^Chandigarh State AIDS Control Society (CSACS), Chandigarh, India, ^6^Postgraduate Institute of Medical Education and Research (PGIMER), Chandigarh, India

**Background**: Limited empirical data are available on the impact of the COVID‐19 pandemic on the lives of sexual and gender minority communities in the global south. We aimed to understand the pandemic's impact on economic well‐being, mental health, health care access, and HIV risk among men who have sex with men (MSM) in India.

**Methods**: In March 2020, we conducted a concurrent mixed‐methods study among MSM in Chandigarh, North India. A convenience sample was recruited through three non‐governmental organizations implementing HIV prevention interventions among MSM. Maximum diversity sampling (identity, sex work status and HIV status) was used to identify MSM for in‐depth interviews. The survey assessed the impact of sexual minority stigma, internalized homonegativity and stress due to social distancing on depressive and anxiety symptoms. We used multivariable logistic regression to analyze survey data, and thematic analysis for qualitative data.

**Results**: Among survey participants (n=132), most (61%) identified as Kothi (feminine/receptive role), were single (79%) and college graduates (64%). Mean monthly income was INR 8375 (USD 120). 43% engaged in sex work, 34% were unemployed and 8% HIV positive. Participants reported reduced access to condoms (19%), HIV testing (38%), and counseling services (74%) during COVID‐19 lockdown. Social distancing stress was significantly associated with depressive (aOR=7.89, 95% CI 2.71−22.97, p<.001) and anxiety symptoms (aOR=5.69, 95% CI 2.73−11.86, p<.001). Internalized homonegativity was significantly associated with both depressive (aOR = 1.31, 95% CI 1.04−1.65, p=.04) and anxiety symptoms (aOR = 1.52, 95% CI 1.09−1.62, p<.001). MSM in sex work had higher odds (aOR=8.06, 95% CI 1.49−43.57, p <.001) of reporting depressive symptoms compared to those not involved in sex work. Qualitative data (n=10) highlighted how economic distress due to job loss or income reduction and survival sex contributed to mental health distress and HIV risk.

**Conclusions**: The COVID‐19 pandemic has significantly impacted the economic well‐being and mental health of lower socioeconomic status MSM. Limited access to HIV preventive interventions among MSM involved in sex work exacerbates HIV risk due to economic hardship. Tele‐counseling/prescription, low‐interest loans, and better access to mental health and HIV services are needed to support MSM in India.

### OAD0204 ‘COVID‐19 should be learned through illustration’: COVID‐19 effects on HIV‐affected youth in South Africa through participatory visual methodologies

L. Gittings ^1,2^, N. Ralayo^2^, J. Kelly^2^, S. Medley^2^, Y. Price^2^, A. Thomas^2^, L. Cluver^3^, C. Logie^1^, E. Toska^2^

^1^University of Toronto, Factor‐Inwentash Faculty of Social Work, Toronto, Canada, ^2^University of Cape Town, Centre for Social Science Research, Cape Town, South Africa, ^3^University of Oxford, Social Policy and Intervention, Oxford, United Kingdom

**Background**: Participatory visual methodologies have been used to take action against pressing social and health issues, challenge HIV‐related stigma, and forefront the realities of marginalised populations. We engaged participatory visual methodologies to explore the COVID‐19 experiences, challenges and coping of HIV‐affected youth.

**Methods**: Two groups of HIV‐affected adolescent advisors –recruited from studies of HIV‐affected young people and adolescents living with HIV – shared their COVID‐19 experiences in telephonic in‐depth, semi‐structured interviews (n=41), and over social media in closed Facebook groups (n= 27 activities) in 2020‐2021. We conducted thematic analysis, identifying seven themes. Each theme was visually translated into a draft illustration by a local artist. Illustrations were verified with participant groups over Facebook, and on the telephone with a sub‐set of adolescent advisors (n=14).

**Figure:**
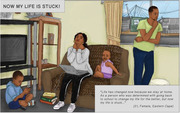
**Abstract OAD0204‐Figure 1**.

**Results**: Participants included 42 youth (aged 16‐29) from the Eastern Cape (n=19) and Western Cape (n=23) provinces. Themes included: feeling anxious and 'stuck' about uncertain futures; lacking basic necessities; inadequate social services; fear of COVID‐19; mental health challenges; non‐biomedical COVID‐19 beliefs; concerns over policing; and lack of protective equipment. We created illustrations to visually depict these themes, then sought feedback over Facebook (n=27 participants) and calls (n=14) with the same group.

Participants suggested that the illustrations:

1. visibilised and documented their experiences, making them feel ‘seen’ during an isolating time;
2. helped them feel connected to other youth, even if they had not seen each other physically;
3. elicited positive reflections on their coping strategies and abilities through COVID‐19 related adversities.

They suggested that the illustrations should be engaged as a tool to advocate for improved health and social services, and that they be accompanied by an exemplary quote to contextualise visual data.

**Conclusions**: Visual participatory methodologies with HIV‐affected youth are possible remotely, and can be engaged via social media to promote psychosocial well‐being, reflection and knowledge mobilisation.

### OAD0205 Pandemic déjà vu: reflections of long‐term AIDS survivors and activists on the first year of COVID‐19

S. Schwartz ^1^, J. Fockele^2^, J. Flanzer^1^, M. Larsen^1^, K. Tinkham^1^, W. Cholico^1^

^1^University of Southern California, Suzanne Dworak‐Peck School of Social Work, Los Angeles, United States, ^2^National AIDS Memorial, San Francisco, United States

**Background**: COVID‐19 surfaced forty years after initial reports of the virus now known as HIV. The rapid spread of both viruses killing seemingly healthy people and early confusion about transmission has garnered attention on pandemic parallels. Current research examines similarities related to biology, disease management, social inequities, and government response. To compliment existing work, this paper explores pandemic similarities and differences from the perspectives of long‐term AIDS survivors and early activists.

**Methods**: Using non‐probability sampling methods, 52 semi‐structured interviews were conducted in real time as the pandemic unfolded between April and November 2020. Fifteen follow‐up interviews were completed in March and April 2021, during vaccine distribution. All interviews occurred over zoom, were recorded, and transcribed. Thematic analysis reveals insights on AIDS/COVID similarities and differences.

**Results**: The sample represents 8 countries, offering diverse perspectives on community response to COVID‐19. Sixty‐one percent identify as Male, 31% Female and 8% Transgender. Participants reflect upon several pandemic similarities to include stigma and ‘othering’; health disparities; slow and inconsistent government response; and crushing fear and anxiety. Participants were quick to address a vital difference between the two pandemics specific to stigma surrounding disease transmission. Since HIV/AIDS is spread through bodily fluids and COVID‐19 through breathing, the social shaming associated with HIV is not evidenced with COVID‐19. Some participants connect transmission differences with government response. The role of media in information sharing and social isolation from support networks were also identified as chief differences in pandemic experiences. Traumatic memories from the 1980s and 1990s were triggered for all participants during COVID‐19 shelter‐in‐place and many expressed anger over what lessons could and should have been learned from the past.

**Conclusions**: Alongside the physicians serving at the front lines of both pandemics are the AIDS activists and long‐term survivors. These voices have received little attention despite the unique lens they offer into surviving two global pandemics. As COVID‐19 continues to destabilize communities around the world, important lessons can be learned from these survivors as we navigate virus mutations and consider the long‐term emotional trauma that can result from surviving a pandemic.

### OAD0302 “Just like you would with an STI or HIV”: sexual risk mitigation during COVID‐19 among gay, bisexual, and other men who have sex with men (GBM) in Canada

E. Daroya ^1^, C. Grey^1^, S. Skakoon‐Sparling^2^, B. Klassen^3^, D. Lessard^4^, J. Jollimore^3^, N. Lachowsky^5^, D. Moore^6^, T. Hart^2,1^, J. Cox^4^, D.H.S. Tan^7,1^, D. Grace^1^

^1^University of Toronto, Dalla Lana School of Public Health, Toronto, Canada, ^2^Ryerson University, Toronto, Canada, ^3^Community‐Based Research Centre, Vancouver, Canada, ^4^McGill University, Montreal, Canada, ^5^University of Victoria, Victoria, Canada, ^6^BC Centre for Excellence in HIV/AIDS, Vancouver, Canada, ^7^St. Michael's Hospital, Toronto, Canada

**Background**: The COVID‐19 pandemic has impacted the sexual behaviours of gay, bisexual, and other men who have sex with men (GBM). Some GBM decreased their sexual behaviours, while others kept engaging in sex with people outside of their households or increased their number of sex partners. However, few studies have focused on how GBM who continued to engage in sexual behaviours assessed and managed risks. We examined the strategies GBM have implemented to negotiate and mitigate sexual risks associated with COVID‐19, HIV, and other sexually transmitted infections (STIs) during COVID‐19 lockdowns.

**Methods**: We conducted semi‐structured interviews with 93 GBM as part of Engage‐COVID‐19, a mixed‐methods study examining the impacts of COVID‐19 on GBM living in Vancouver, Toronto, and Montreal. Participants were recruited along four key dimensions: ethno‐racial backgrounds, age, gender identity, and HIV status. Two rounds of online interviews took place between November 2020‐January 2021 and June‐October 2021. Interviews were transcribed verbatim and thematically coded using NVivo.

**Results**: Participants ranged in age from 24‐76 years old. 73 participants self‐identified as HIV‐negative and 20 as living with HIV. We identified four key preventive practices: (1) Asking about health status: participants who reported engaging in sexual behaviours mitigated sexual risks by inquiring about potential partners’ HIV, STI, and COVID‐19 status; (2) Reducing physical contact during sex: a few men implemented mask‐wearing, avoided kissing, and engaged in voyeuristic masturbation to minimize physical contact; (3) Sex with regular partners: some engaged in sexual behaviours only with people they already knew and trusted; and (4) Vaccine status sorting: most participants reported increased sexual activities after receiving COVID‐19 vaccinations and used vaccine status sorting by only having sex with partners who have received COVID‐19 vaccines.

**Conclusions**: Our results show that GBM who continued engaging in sexual behaviours during the COVID‐19 pandemic implemented mitigation strategies to reduce risks associated with HIV, other STIs, and COVID‐19. Our findings shed light on how GBM are using knowledge gained from HIV prevention to inform their protective behaviours during the COVID‐19 pandemic. Significant efforts are needed to provide resources acknowledging the multiple pandemics GBM are attempting to navigate at this time.

### OAD0303 Syndemic factors and sexual risk behaviors among men who have sex with men in Guangzhou, China: a latent variable structural equation modeling approach

Y. Huang ^1^, J. Li^1^, W. Cheng^2^, Y. Yang^1^, H. Jiang^1^

^1^Guangdong Pharmaceutical University, Department of Epidemiology and Biostatistics, Guangzhou, China, ^2^Guangdong Second Provincial General Hospital, Guangzhou, China

**Background**: Men who have sex with men (MSM) were vulnerable to encounter syndemic factors, resulting in a higher risk of sexual risk behaviors. This study aimed to explore the interactions between the coexisting syndemic factors and condomless anal intercourse (CAI) and multiple sexual partners among MSM in Guangzhou, China.

**Methods**: A cross‐sectional study was conducted to recruit MSM in Guangzhou from June 2017 to April 2018. Data on syndemic factors including childhood sexual abuse (CSA), intimate partner violence (IPV), depression, internalized homophobia (IH), higher level of Sexual sensation seeking (SSS), alcohol/rush popper use before sex were collected. Multi‐order latent variable structural equation modeling (SEM) were performed to explore the syndemic effects of the coexisting psychosocial factors on sexual risk behaviors.

**Results**: A total of 500 MSM who had sex in the last 6 months were included. The proportions of CAI and multiple sexual partners in the last 6 months were 44.40% and 60.40%. The proportion of syndemic factors CSA, IPV, depression, higher level of SSS, IH, alcohol use and rush popper before sex in the last 6 months was 23.60%, 12.80%, 25.00%, 54.80%, 59.40%, 33.80% and 33.40%, respectively. A larger number of syndemic factors was associated with higher risks of CAI (aOR=1.23, 95%CI:1.05∼1.45) and multiple sexual partners (aOR=1.33, 95%CI:1.13∼1.56). The second‐order latent variable SEM was better than the first‐order latent variable SEM with the ability to explain sexual risk behavior variation of *R* ^2^_second order_=91% and *R* ^2^_first order_=66%. There was a strong linear relationship (Standard regression coefficient=0.96) between sexual risk behaviors and the syndemic burden which was formed by the potential interaction of violence (CSA and IPV), mental health (depression and SSS), and substance use (alcohol/rush popper use before sex). Mental health contributed the most to the syndemic burden (*R* ^2^_mental health_=96%,*R* ^2^_violence_=58%, *R* ^2^_substance use_=34%).

**Conclusions**: MSM in Guangzhou were confronted with a high burden of multiple psychosocial problems. Multiple syndemic factors and their interactions should be fully taken into consideration when tailoring targeted interventions and health care policies to improve the effectiveness of the intervention. Mental health screening and psychological counseling should be prioritized under limited health resources.

### OAD0304 Characterizing substance use typologies and their association with sexual risk behaviors: a latent class analysis among men who have sex with men in Mexico

A. Algarin ^1^, M. Valenzuela Lara^2^, R. Baruch‐Dominguez^3^, M. Hernandez‐Avila^4^, T. Sanchez^2^, S. Strathdee^1^, L. Smith^1^

^1^University of California, San Diego, Division of Infectious Diseases and Global Public Health, La Jolla, United States, ^2^Emory University, Epidemiology, Atlanta, United States, ^3^International Planned Parenthood Federation, Western Hemisphere Region, Mexico City, Mexico, ^4^Mexican Institute of Social Security, Economic and Social Benefits, Mexico City, Mexico

**Background**: Substance use behaviors are closely associated with increased risk for HIV and other STIs, but little has been reported about substance use among men who have sex with men (MSM) in Mexico. We characterized classes of substance use among a nationwide sample of MSM in Mexico and examined the associations between substance use classes, sexual behaviors and STI diagnosis.

**Methods**: The *Encuesta de Sexo Entre Hombres* study collected online survey data on substance use, sexual behavior and STI diagnosis in the past 12 months from 15,875 Mexican MSM between May‐June 2017. Latent class analyses characterized substance use patterns and multiple multivariable regression models examined substance use class associations with sexual behaviors while controlling for age, education, sexuality, HIV status, and geographical region.

**Results**: We identified five distinct substance use classes: No Drug Use (75.4%), Marijuana Only (15.1%), Marijuana + Poppers (4.3%), Marijuana + Stimulants (4.2%), and Assorted Drug Use (e.g. Marijuana + Poppers + Stimulants + Other Substances) (1.0%). Demographic makeup of Classes were significantly different, where the Assorted Drug Use class was majority 25‐39 years of age (71.4%; p<0.001), received a Bachelor's degree or more (73.6%; p<0.001), gay (89.0%; p<0.001), HIV negative/ unknown status (62.8%; p<0.001), and lived in the City/State of Mexico (50.0%; p<0.001) We found that participants in substance use classes (e.g. Classes 2‐5) were significantly more likely to engage in condomless anal intercourse (adjusted prevalence ratio [aPR]=1.14‐1.39; all p<0.001), exchange sex for goods (aPR=1.37‐4.99; all p<0.001), anonymous sex (aPR=1.22‐2.01; all p<0.001), group sex (aPR=1.50‐3.28; all p<0.001), and have an STI diagnosis (aPR=1.24‐2.20; all p<0.002) in comparison to participants in the No Drug Use class, where the largest estimates were among the Assorted Drug Use class.

**Conclusions**: Understanding substance use groups and how group classification impacts sexual health in Mexico can better inform future interventions focused on reducing HIV incidence among MSM with varying substance use behaviors, particularly as policies begin to change following the Supreme Court ruling for the legalization of recreational marijuana in 2021 and methamphetamine use is growing exponentially.

### OAD0305 Exploring the mental health experiences and perceived social and sexual risks among female sex workers in Nairobi, Kenya

M. Panneh ^1^, M. Gafos^1^, H.A. Weiss^2^, E. Nyariki^1^, H. Babu^1^, J. Seeley^1^, P. Shah^1^, J. Liku^3^, M. Wanjiru^3^, R. Wanjir^3^, R. Kaul^4^, J. Kimani^3^, J. Bradley^2^, T. Beattie^1^

^1^London School of Hygiene and Tropical Medicine, Global Health and Development, London, United Kingdom, ^2^London School of Hygiene and Tropical Medicine, MRC International Statistics and Epidemiology, London, United Kingdom, ^3^Partners for Health and Development in Africa, Nairobi, Kenya, ^4^University of Toronto, Kings College Toronto Canada, Toronto, Canada

**Background**: Female sex workers in Kenya are at an increased risk of HIV infection, violence, poverty and harmful alcohol and other substance use, which are all linked to poor mental health and suicidal ideation/behaviours. Some of these distressful events may precipitate entry into sex work for some women. There has been limited qualitative research investigating the mental health experiences of female sex workers in Kenya. In this study we examine female sex workers’ mental health experiences and perceived social and sexual risk factors over their life course.

**Methods**: We randomly selected 40 female sex workers enrolled in a longitudinal study in Nairobi, for baseline in‐depth semi‐structured interviews. Participants were asked to detail their life stories, including narrating specific events such as entry into sex work, HIV testing and diagnoses, experiences of violence, mental health, alcohol use etc. Interviews were recorded, transcribed and translated. Data were coded thematically using the Hierarchical Conceptual Framework to explore risk factors for mental health and suicidal ideation/behaviours.

**Results**: Based on the women's personal and second hand experiences, they related mental health to stress, depression and suicide. A few believed in the supernatural causes of mental health problems like witchcraft. Structural factors such as low levels of education, poor job opportunities, the lack of family support, harmful gender norms, intimate partner violence and subsequent relationship breakdowns, and family bereavement all contributed to poor mental health and subsequent entry into sex work. Their entry into sex work was despite the recognised risk of HIV, even though the majority were HIV negative when they started. The consequences of sex work such as sexual risks, concern about HIV acquisition, ongoing violence from police and clients, all exacerbated their poor mental health.

**Conclusions**: There is a need for both micro‐ and macro interventions to address poverty and gender‐based violence among vulnerable women in Kenya, thereby reducing mental health problems, entry into sex work and risk of HIV acquisition. FSW programmes should include health promotion and screening for mental health problems to increase health seeking behaviour and access to services for FSWs.

### OAD0402 Predictive modelling to determine defaulting from antiretroviral therapy (ART) services amongst adolescent girls and young women (AGYW)

M. Majachani ^1^, J. Murungu^2^

^1^Chinhoyi University of Technology, Graduate Businesss school, Harare, Zimbabwe, ^2^University of Zimbabwe, Medicine, Harare, Zimbabwe

**Background**: Zimbabwe has made significant progress towards the 95%‐95%‐95% targets. However, outcomes are worse among adolescents and young people compared to other population groups. Only 49% of AGYW aged 15‐24 years on ART are virally suppressed compared to the national suppression rate of 90%. We therefore developed a supervised machine learning model to predict the risk of ART defaulting amongst AGYW.

**Methods**: Design science methodology was used to develop and assess the performance of algorithms to predict the risk of defaulting among AGYW initiated on ART between 2013 and 2016 at Mbare Polyclinic in Zimbabwe. The Cross Industry Standard Process for Data Mining (CRISP‐DM) was applied to mine and analyse the data before modelling and evaluation. We used Decision Tree Classifier algorithms with neural networks to predict ART defaulting. A ten‐fold cross‐validated area under the receiver operating characteristic curve was used to assess the model's performance at identifying AGYW who defaulted ART. The best‐performing algorithm was obtained with least absolute shrinkage and selection operator. The demographic and clinical characteristics of the 2055 patients who were filtered out were analysed.

**Results**: Data for 2,055 AGYW was analyzed of which 1,007 were AGYW, (median age 21 years), in the development cohort. The model was applied and successfully predicted defaulter outcomes for 606 AGYW with similar social characteristics to those who already had a defaulter outcome, and in‐care outcomes for the remaining 442. Factors associated with defaulting ART included lack of disclosure and illegal cohabitation.

The predictive model yielded positive results, with the chosen algorithm's accuracy reaching 100 percent. The prediction model is likely to produce encouraging results when applied to other age groups of patients to evaluate their ART defaulting likelihood. Using factors associated with a higher risk of defaulting, the model can predict ART defaulters with accuracy and precision.

**Conclusions**: Automated algorithms efficiently identify patients at increased risk of ART defaulting. Integrating these models into Electronic Health Records to alert providers about patients who might default ART could improve adherence tracking.

### OAD0403 Determining preferred attributes of a “virtual village" platform to halt isolation among people aging with HIV: a community‐engaged project

A.L. Nguyen ^1^, B. Brown^2^, K.Y. Greene^3^, E. Ruiz^4^, A.N. Polonijo^5^, M. Yoo‐Jeong^6^, J. Taylor^7^, C. Christensen^7^, J.T. Galea^3^

^1^University of Southern California, Keck School of Medicine, Los Angeles, United States, ^2^University of California, Riverside, School of Medicine, Riverside, United States, ^3^University of South Florida, School of Social Work, Tampa, United States, ^4^University of South Florida, College of Public Health, Tampa, United States, ^5^University of California, Merced, Department of Sociology, Merced, United States, ^6^Northeastern University, School of Nursing, Boston, United States, ^7^HIV+Aging Research Project‐Palm Springs, Palm Springs, United States

**Background**: COVID‐19 exacerbated existing social isolation, depression, and anxiety among older people living with HIV (OPLWH). While the use of existing social networking platforms can help reduce social isolation, they lack specificity for addressing the needs of OPLWH. In response, we are developing a “virtual village” platform to reduce social isolation among OPLWH. As a first step, we investigated what OPLWH perceive to be the platform's most important attributes.

**Methods**: In collaboration with a community advisory board (CAB) of 24 OPLWH ≥50‐years‐old (from three sites: Palm Springs, CA, Los Angeles, CA, and Tampa Bay, FL), we constructed a list of 28 potential attributes for the virtual village. Next, the CAB rank‐ordered the attributes to identify the top‐five most important and chose mutually exclusive levels (private chat [yes/no]; cost [free/paid]; sub‐communities [yes/no]; social service directory [yes/no]; registration required [yes/no]) for each to create a choice‐based conjoint experiment. English‐speaking OPLWH ≥50‐years‐old who resided in a study city and had internet access were then recruited to participate in the experiment and received $50 for completion. Participants compared eight groups of different combinations of four hypothetical virtual village “scenarios” comprised of the 5 attributes at differing levels, selecting the most acceptable scenario from each group. The relative importance of the attributes was calculated using Sawtooth Software's (2021) Hierarchical Bayesian Analysis.

**Results**: Participants (N=57) were 50‐82 years‐old (mean=59.8 years). Most (78.6%; n=45) identified as male. 64.1% (n=35) identified as White, 29.8% (n=17) as Black/African American, and 17.5% (n=10) as Hispanic/Latino/a/x.The preferred attributes for the virtual village in order of their relative preference, and their corresponding levels when compared to all other options (all p's < 0.05) were: cost (24.74%, free); chat function (22.91%, yes); communities (15.58%, yes); services (17.86%, yes); and registration (18.9%, required).

**Conclusions**: Participants identified the attributes/levels most important for inclusion in the Virtual Village. Continued development of our Virtual Village will prioritize these attributes during prototype development.

### OAD0404 Interest of people living with HIV in injectable long‐acting antiretroviral treatment: results from a flash AIDES survey

C. Lacoux ^1,2^, C. Rouquette^1^, M. Salcedo^1,2^, T. Alain^1,2^, C. Le Gouez^1^, D. Michels^1,2^, B. Spire^1,3^

^1^AIDES, Pantin, France, ^2^Coalition PLUS, Community‐based Research Laboratory, Pantin, France, ^3^Aix Marseille University, Inserm, IRD, SESSTIM, Sciences Economiques & Sociales de la Santé & Traitement de l'Information Médicale, ISSPAM, Marseille, France

**Background**: The end of 2021 in France was marked by the important therapeutic innovation of injectable long‐acting antiretroviral (iARV) treatment for HIV. While healthcare professionals and pharmaceutical industries are hoping for better adherence and improved quality of life in people living with HIV (PLHIV), the latter's perceptions have not been explored. To ensure good adherence to iARV, it is essential to identify PLHIV expectations of its benefits, their potential related fears, and obstacles they perceive to its implementation. We aimed to identify factors associated with interest in iARV among PLHIV frequenting the French association AIDES.

**Methods**: From July to October 2021, an online and paper‐based survey was distributed through the AIDES network via social networks and the association's newspaper *Remaides*. It comprised 16 questions collecting data on sociodemographics, interest in iARV, confidence in its efficacy, expected improvements in quality of life, and perceived potential obstacles to its implementation. A multivariate logistic regression helped identify factors associated with a high level of interest in iARV (“very” versus “quite”, “not really” and “not at all” interested).

**Results**: Among the 581 respondents, the majority were men (n=459; 79 %) and were born in France (n=477; 82 %). Median age was 52 years [42‐59]. Approximately half (n=276, 47 %) were very interested in iARV. Factors associated with a high level of interest were daily intake of non‐ARV treatments (aOR=1.9[1.2‐3.2]), cohabiting with persons unaware of the respondent's HIV status (aOR=2.3[1.3‐4.1]), confidence in iARV efficacy (aOR=2.8[1.7‐4.6]), expected improvements in quality of life (aOR=6.4[4.1‐10.1]) and willingness to continue iARV despite potential side effects (aOR=4.4[2.6‐7.6]). In contrast, travelling to the hospital for iARV was seen as a constraint (aOR=0.6 [0.4‐0.9]), while the ease of taking current (i.e., non‐injectable) ARV (aOR=0.6[0.4‐0.9]) was associated with a low level of interest in iARV.

**Conclusions**: PLHIV are interested in iARV, especially those whose current situation is complicated due to the confidentiality of their HIV status, or to difficulties following several treatments simultaneously. Having to go to hospital for iARV to ensure good adherence could constitute a barrier to uptake. One possible alternative is the use of ambulatory care providers.

### OAD0405 DOTS PLUS: a promising approach to increasing adherence to tuberculosis, drug resistant tuberculosis and antiretroviral HIV treatment in Mozambique

A. Cassamo Omar Abdula ^1^, H. Hallstrom^2^, M. Lichtenberg^3^

^1^ADPP Mozambique, Project Implementation, Maputo, Mozambique, ^2^ADPP Mozambique, Partnership, Maputo, Mozambique, ^3^Planet Aid, Inc, USA, Elkridge, United States

**Background**: According to World Health Organization Global Tuberculosis (TB) Report (2021), Mozambique is among the highest‐burden countries for TB, TB/HIV co‐infection and drug resistant TB (DR‐TB), and is among 10 countries where over 75% of estimated DR‐TB cases remain undetected. Currently, almost half (47%) of DR‐TB cases are HIV+. Addressing barriers to treatment adherence must be a national priority to ensure people with TB are successful in completing their treatment regimens.

Since 2019, ADPP Mozambique has been applying a holistic, patient centered approach through the enhanced Direct Observation Treatment Strategy (DOTS) PLUS, funded by USAID.

**Description**: ADPP, a local Mozambican civil society organization, leads the implementation of the Local TB Response project in four provinces of Mozambique, in partnership with FHI 360, Comusanas, Kupulumusana and DIMAGI. DOTS PLUS includes direct observation of treatment, **plus** psychosocial support, financial support (for nutrition, transport, and other costs), and provision of pill boxes to keep patients on track. It includes medication monitoring, regularly scheduled refills, home deliveries and accompanying patients to follow up appointments.

**Lessons learned**: During the first two years, the project identified 32,676 new TB cases (46% of all TB cases in the 50 target districts), and 86 DR‐TB cases (16% of newly identified TB cases in targeted districts, and 7% at national level). From May 2020 to September 2021, 250 DR‐TB patients received support from DOTS PLUS, with 99% adhering to TB and antiretroviral drug treatment (for those with HIV), and 65% achieving culture conversion upon treatment completion, with zero lost to follow up. These impressive results compare to a national average of only 60% for TB treatment completion, likely due to the added features of DOTS PLUS.

**Conclusions/Next steps**: Providing DOTS PLUS for TB, TB/HIV and DR‐TB patients is crucial for ensuring treatment adherence as it provides more wrap around care and follow up support, improving treatment success and reducing morbidity and mortality. ADPP will continue to promote DOTS PLUS for all TB and HIV response efforts, in a collaborative effort with the Ministry of Health and partners.

### OAD0502 A behavior‐based intervention investigating the effects of a gender‐based violence (GBV) and sexual assault educational curriculum on improving male attitudes toward women in 4 sub districts in South Africa

N. Madubela ^1^, S. Voster^1^, C. Wagner^1^, M. De Vos^1^, M. Vaynos^1^

^1^NACOSA, Cape Town, South Africa

**Background**: The rights of women, girls and other vulnerable populations continue to be compromised by high levels of GBV in South Africa. The prevalence of the issue, compounded by the impact of the Covid‐19 pandemic, has triggered a ‘secondary pandemic’ marred by rising GBV and femicide (GBVf). In 2021, No Means No Worldwide (NMNW) and NACOSA launched the IMPOWER Boys program, an evidence‐based intervention, delivering an educational sexual and GBV prevention curriculum.

**Description**: Overall, 16 male instructors were trained to facilitate an 8‐hour curricular (4 classes, 2 hours each) in 4 sub districts namely: Klipfontein, Mitchells Plain, Tshwane and Bojanala. The target population was n=1120 boys (280 per sub district) aged between 10‐24 years, in 7 months (August 2021 – December 2021). The program was designed to increase gender equitable attitudes, learn skills to defend equality, avoid violence, ask for consent, and intervene when witnessing or anticipating sexual assault. The Intervention was delivered using 3 models: In school (within school hours), after school (extracurricular) and out of school (in community spaces). Data on attitudes toward women were collected anonymously at baseline by administering pre‐questionnaires and post questionnaires, and compared with baseline

**Lessons learned**: Overall participants had significantly higher positive attitudes toward women at follow‐up. Median age is 19 years. 65% participants > 19 years. The percentage of boys who successfully intervened when witnessing violence was 78% for verbal harassment, 75% for physical threat, and 74% for physical or sexual assault. Data shows noteworthy gains in knowledge towards consent, intervening during cases of violence and gender roles shift in gender equitable attitudes with 82% able to provide desired responses. An average of 42% change between the pre/post intervention data, a significant 85% were able to accurately recollect core knowledge topics in the post questionnaires.

**Conclusions/Next steps**: A multifaceted response is needed to enhance the country's fight against GBVf and HIV/AIDS in a COVID‐19 pandemic. This standardized 4‐week training program proved to be effective in improving attitudes toward women and increasing the likelihood of successful intervention when witnessing GBV.

### OAD0503 Impacts of intimate partner violence and sexual violence on antiretroviral adherence among adolescents living with HIV in South Africa

L. Cluver ^1,2^, S. Zhou^3,4^, M. Orkin^5^, S. Dzumbunu^3^, F. Meinck^6,7^, N. Langwenya^1,3^, M. Vicari^8^, L. Sherr^9^, E. Toska^3,10^

^1^University of Oxford, Department of Social Policy and Intervention, Oxford, United Kingdom, ^2^University of Cape Town, Department of Psychiatry and Mental Health and Centre for Social Science Research, Cape Town, South Africa, ^3^University of Cape Town, Centre for Social Science Research, Cape Town, South Africa, ^4^University of Cape Town, Department of Public Health and Family Medicine, Faculty of Health Sciences, Cape Town, South Africa, ^5^University of the Witwatersrand, Medical Research Council Development Pathways to Health Research Unit, School of Clinical Medicine, Johannesburg, South Africa, ^6^University of Edinburgh, School of Social and Political Science, Scotland, United Kingdom, ^7^University of the Witwatersrand, School of Public Health, Johannesburg, South Africa, ^8^International AIDS Society, Geneva, Switzerland, ^9^University College London, Institute for Global Health, London, United Kingdom, ^10^University of Oxford, Social Policy and Intervention, Oxford, United Kingdom

**Background**: We are failing to reach 95‐95‐95 for adolescents living with HIV (ALHIV). Adolescents in Sub‐Saharan Africa are exposed to high rates of sexual violence and intimate partner violence (IPV). However, evidence on associations of sexual violence and ART adherence remains limited, with only three cross‐sectional studies globally.

**Methods**: We conducted a longitudinal cohort, with interviews and clinical records from 1046 ALHIV aged 10‐19 years, recruited from 53 government health facilities in South Africa's Eastern Cape (2014‐2018; 57% female, 90% uptake, 94‐97% retention, 3.4% mortality).Ethical approvals were given by the University of Cape Town, University of Oxford, provincial government and health facilities. We used a repeated‐measures random effects model and marginal predicted probabilities to assess multivariable associations of self‐reported sexual violence and IPV with ART adherence, validated against viral load suppression (<50 copies/ml). We fitted moderation models by gender.

**Results**: 51% of adolescents reported consistent ART adherence. ART adherence was associated with viral suppression (aOR 1.49, CI:1.03‐2.14, p=0.033) controlling for age, sex, location, poverty, housing, and HIV acquisition mode. Exposure to IPV was associated with lower ART‐adherence (aOR 0.39, CI:0.21‐0.72, p=0.003), and so was sexual violence (aOR 0.54, CI:0.29‐0.99, p=0.048). Marginal predicted probabilities showed that adolescents with no sexual violence or IPV exposure had a 72% (CI:0.70‐0.74) probability of ART adherence compared to 38% (CI:0.20‐0.56) for those exposed to both sexual violence and IPV. Moderation showed similar impacts of violence by gender.

**Conclusions**: Effective sexual violence prevention and post‐violence care are essential in supporting adolescent ART adherence. There is now increasing evidence of effective services across sectors, with systematic reviews identifying parenting programmes, classroom and community‐based programmes in sub‐Saharan Africa, and social protection such as government cash transfers in reducing sexual violence. There is an urgent need to link violence prevention and adolescent HIV services.

**Figure:**
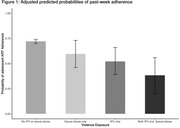
**Abstract OAD0503‐Figure 1**.

### OAD0504 Targeted violence as a risk factor for posttraumatic stress disorder and HIV acquisition risks among cisgender gay, bisexual, and other men who have sex with men in the United States

J.M. Wiginton ^1^, S. Murray^2^, S. Baral^3^, T. Sanchez^4^

^1^San Diego State University, School of Social Work, San Diego, United States, ^2^Johns Hopkins University Bloomberg School of Public Health, Department of Mental Health, Baltimore, United States, ^3^Johns Hopkins University Bloomberg School of Public Health, Center for Public Health & Human Rights, Department of Epidemiology, Baltimore, United States, ^4^Emory University Rollins School of Public Health, Department of Epidemiology, Atlanta, United States

**Background**: Posttraumatic stress disorder (PTSD) has been linked to HIV transmission risk behaviors among cisgender gay, bisexual, and other men who have sex with men (MSM) in the United States (US), and interpersonal violence carries the highest conditional risk of developing PTSD. Among MSM who have experienced interpersonal violence, characterizing risk factors for PTSD is critical to inform preventive and therapeutic intervention strategies.

**Methods**: Using a 2020 nationwide cross‐sectional survey of 2,886 MSM (21.5% of 13,433 MSM surveyed) who reported ever experiencing interpersonal violence, we performed multivariable modified Poisson regressions with robust variance estimators to examine differences in prevalence of current PTSD by how participants attributed the experience of violence (as occurring because of one's same‐sex practices, as not occurring because of one's same‐sex practices, or unsure of whether or not it occurred because of one's same‐sex practices). We also examined the relationship between PTSD and past‐year serodiscordant condomless anal sex. Control variables included age, education, race/ethnicity, sexual identity, urbanicity, and HIV status. Model results are reported as adjusted prevalence ratios (aPR) and 95% confidence intervals (CI).

**Results**: Median age of participants who experienced interpersonal violence was 27 years (interquartile range: 22‐43); 78.8% identified as gay (n=2,273), and 62.2% (n=1,794) were non‐Hispanic white. Interpersonal violence was attributed to same‐sex practices by 45.8% (n=1,321) of participants; 46.3% (n=1,335) did not make this attribution, and 7.0% (n=203) were unsure. Overall, 23.0% (n=665) had PTSD, and PTSD prevalence was greater among participants who attributed the violence to their same‐sex practices (25.9%[342/1,321]; aPR[CI]=1.54[1.33‐1.78]) or who were unsure (33.5%[68/203]; aPR[CI]=1.80[1.44‐2.25]) compared to those who did not make the attribution (18.1%[242/1,335]). Those who met criteria for PTSD were more likely to report serodiscordant condomless anal sex (27.0%[237/879] versus 21.3%[428/2,007]; aPR[CI]=1.22[1.08‐1.38]).

**Conclusions**: Findings reveal the potential role of attribution in PTSD risk for violence‐exposed US MSM and suggest a likely pathway between violence exposure, PTSD, and serodiscordant condomless anal sex. Future research with longitudinal designs will be needed to establish temporal ordering to test this pathway. Moreover, progress on ending the US HIV epidemic will require interventions that simultaneously address mental and sexual health among violence‐exposed MSM.

### OAD0505 Impact of intimate partner violence on women's risk of HIV acquisition and engagement in HIV care cascade in sub‐Saharan Africa: a meta‐analysis of population‐based surveys

S. Kuchukhidze ^1^, D. Panagiotoglou^1^, M.‐C. Boily^2^, S. Diabaté^3^, J. Eaton^2^, F. Mbofana^4^, L. Sardinha^5^, L. Schrubbe^6^, H. Stöckl^7^, R. Wanyenze^8^, M. Maheu‐Giroux^1^

 

**Figure:**
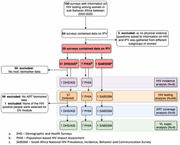
**Abstract OAD0505‐Figure 1**.

^1^McGill University, Department of Epidemiology, Biostatistics and Occupational Health, Montreal, Canada, ^2^Imperial College London, Medical Research Council (MRC) Centre for Global Infectious Disease Analysis, London, United Kingdom, ^3^Université Laval, Département de Médecine Sociale et Préventive, Quebec City, Canada, ^4^Conselho Nacional de Combate ao HIV/Sida, Maputo, Mozambique, ^5^University of Bristol, Bristol Poverty Institute, School for Policy Studies, Bristol, United Kingdom, ^6^London School of Hygiene & Tropical Medicine, Department of Population Health, Faculty of Epidemiology and Population Health, London, United Kingdom, ^7^Ludwig‐Maximilians‐Universität München, Institute for Medical Information Processing, Biometry, and Epidemiology, Munich, Germany, ^8^Makerere University, Department of Disease Control and Environmental Health, School of Public Health, Kampala, Uganda

**Background**: Achieving the 95‐95‐95 targets for HIV diagnosis, treatment, and viral load suppression (VLS) to end the AIDS epidemic hinges on eliminating manifestations of structural inequalities, including intimate partner violence (IPV). Sub‐Saharan Africa (SSA) has among the world's highest prevalence of IPV and HIV but an examination of the impact of IPV on HIV incidence, and women's engagement in HIV care cascade is yet to be conducted.

**Methods**: We pooled individual‐level data from all available nationally representative surveys with information on physical and/or sexual IPV in SSA (2000‐2020;Figure). We used generalized estimating equations with robust standard errors to estimate adjusted prevalence ratios (aPR) of lifetime and past year experience of IPV on HIV incidence (measured cross‐sectionally by recent infection testing algorithm), past‐year HIV testing (self‐reported), antiretroviral therapy (ART) uptake, and VLS among ever‐partnered women. Models were adjusted for age, age at first sex, residence type, women's marital status, women's education, and survey as a proxy of time and country.

**Results**: Fifty‐nine surveys were available from 30 countries, encompassing over 273,000 (N_i_) respondents. Most surveys were from East Africa (48%); median survey year was 2013. Overall, 32% of women reported lifetime physical and/or sexual IPV (N_i_=255,564) and 22% experienced IPV in the past year (N_i_=273,603). Women exposed to past year IPV were 2.75 times (95%CI:1.26‐6.00;N_i_=19,852) more likely to have a recent HIV infection, adjusting for potential confounders. Past year IPV was not associated with HIV testing (aPR=1.00, 95%CI:0.98‐1.01;N_i_=273,603), but women living with HIV experiencing IPV in the last year were 10% less likely to be on ART (aPR=0.90; 95%CI:0.82‐0.99;N_i_=5,205) and to achieve viral suppression (aPR=0.90; 95%CI:0.81‐0.99;N_i_=5,205).

**Conclusions**: IPV was associated with increased HIV incidence and, among women living with HIV, lower ART uptake and VLS. Preventing IPV is inherently imperative, and a crucial milestone in reducing population‐level HIV incidence and burden.

### OAD0602 Examining engagement in and initial efficacy of a structured peer health navigator intervention to improve HIV and related outcomes among YBMSM living with HIV

D. Gerke ^1^, J. Glotfelty^2^, M. Freshman^2^, A. Ochs^1^, K. Plax^2^

^1^University of Denver, Graduate School of Social Work, Denver, United States, ^2^Washington University School of Medicine in St. Louis, Pediatrics, Saint Louis, United States

**Background**: HIV health disparities among young black men who have sex with men (YBMSM) are often the result of a complex intersection of trauma, psychosocial cofactors, and low access to socioeconomic resources. To address these disparities and needs, the WITH U intervention employed peer health navigation that attended to behavioral health needs, linkage to services, and psychosocial support. This study reports on engagement and efficacy of the intervention.

**Methods**: This was a longitudinal mixed‐methods single‐arm study. Data were collected from enrollment and 6‐month self‐administered quantitative surveys with all participants and semi‐structured qualitative interviews with a sub‐sample of 22 participants. Quantitative analyses included frequencies, means, and bivariate correlations. Deductive and inductive content analysis was used to analyze qualitative data.

**Results**: WITH U participants were 65 YBMSM, average age of 25‐26 years (M=25.55, *SD*=2.51). Approximately one quarter of participants scored at or above the clinical cutoff for depression or anxiety symptoms, and over 40% reported being bothered by past traumatic stressors in the last month. More than half of participants reported food insecurity in the last three months, over a third were concerned about housing, and a quarter were unable to pay their utilities when needed.

Overall, participants reported high satisfaction with WITH U and attended an average of 5‐6 of 12 possible sessions with health navigators (M=5.69, SD=3.05). Attending a greater number of sessions was significantly associated with being virally suppressed at enrollment (*rs*(50)=.36, *p*<.05) and six month‐follow‐up (*rs*(38)=.34, *p*<.05), as well as greater concern about housing (*rs*(54)=.29, *p*<.05) and experiencing unemployment for at least three months in the last year (*rs*(54)=.29, *p*<.05). This aligned with qualitative findings that navigator support around basic needs was critical to participants. Although participants identified mental health support from navigators as important, experiencing more depressive symptoms was significantly associated with attending fewer sessions (*rs*(54)= ‐.32, *p*<.05). A switch to virtual sessions due to the COVID19 pandemic was a barrier to engagement for some participants.

**Conclusions**: Engagement in WITH U was associated with improved health outcomes and social determinant health assistance needs. Novel methods for better engaging YBMSM virtually, especially those with depression, are needed.

### OAD0603 Improving HIV outcomes amongst adolescents and young people living with HIV through adolescence and young people mentorship program

P. Ogunsanya ^1^, A. Ogunsanya^1^, E. Kihika^1^, D. Mpagi^1^, J. Nampungu^1^, L. Kansiime^1^, Z. Timothy^1^, I. Gulemye^1^, C. Adongo^1^, C. Ndeloa^2^, D. Schlosser^2^

^1^Alive Medical Services, Clinical, Kamapala, Uganda, ^2^Frntline AIDS, Programs, Brighton, United Kingdom

**Background**: According to the 2020 UNAIDS report there were over 170,000 adolescents and young people living with HIV(AYPLHIV) in Uganda. Viral load suppression rates were below 50% for the under 24years and there were over 2800 AIDS related deaths a figure that has stagnated since 2015. Research has shown that poor adherence to ART, amongst AYPLHIV can be attributed to several factors including small support networks, forgetfulness, individual resilience factors and stigma. Alive Medical Services (AMS) implemented AYP mentorship program aimed at supporting AYP with non‐suppressed viral loads improve adherence and treatment outcomes.

**Description**: Between July‐December 2021, AMS Identified consenting AYP who have achieved viral load suppression (VLS) and those with nonviral load suppressed (NVLS) matching them in groups disaggregated by age no more than 20 members. The groups met monthly at the youth corner and would dialogue, share their experiences, play in door games and offer support. VLS AYP would follow up their partners in the community with the help of the community linkage facilitator both virtually and physically. The ministry of health treatment protocols for the two groups were adhered to including intense adherence counselling sessions for the NVLS AYP. Those who suppressed at the end of the 6 months were engaged to support new AYP with NVLS

**Lessons learned**: Out of the 28 young people who were none suppressed at the start 22 have achieved viral load suppression and are now being engaged to support another group of young people with newly none suppressed viral loads 2 have been switched to the second line and are being followed up. Friendships have been established, attitudes and behaviours have changed, adherence and appointment keeping has greatly improved

**Conclusions/Next steps**: Social factors play a crucial role in ensuring adherence to ART medication by AYPLHIV.

AMS plans to Scale up the AYP adherence mentorship program to all AYP in other facilities while working with the AYP to reach more AYP.

Key Words: Mentorship, Adolescents, Adherence, Young people

### OAD0604 Zambia on the rise: addressing the challenges of young key populations inclusion

B.D. Nibogora ^1^, K. Mkandawire^2^, W. Mulwanda^2^

^1^UNDP, BPPS, Nepean, Canada, ^2^UNDP, BPPS, Lusaka, Zambia

**Background**: The linking policy to programming project in Southern Africa aimed at reducing HIV risks and improving SRHR outcomes amongst young key populations (YKP), namely young gay men and men who have sex with men, sex workers of all genders, drug users, transgender and inmates aged from 10 to 24 years. The programme's objectives were to (1) build capacity for YKPs to claim their rights; (2) support policymakers to understand YKP needs and deliver tailored HIV and SRHR services ; (3) strengthen regional mechanisms providing standards to countries to end AIDS and achieve SRHR outcomes; (4) generate strategic information through research for programmes’ improvement. This abstract presents lessons from Zambia.

**Description**: The project used a people's centred approach: putting YKPs at the centre of implementation, ensuring that project's focal staff, trainees, research personnel and advocacy task force members were from and accountable to YKPs. A regional team with expertise in policy advocacy and movement building supported in‐country safe‐space retreats to identify priority issues; development of a multi‐stakeholders’ roadmap for policy change and its roll‐out by an advocacy task team. The advocacy process involved, among others, a media training to shift the narrative around sexuality and drug use. Regional convenings allowed for further sharing, exposure and learning.

**Lessons learned**: The programme allowed for an emergence of amore resilient movement of YKP advocates despite the hostile political context. In addition to existing groups[1], the programme strengthened new vibrant groups ‐ such as the Intersex Society of Zambia, Umoto and Decisive Minds ‐ led by young leaders from the intersex and gay community as well as drug users. Their engagement led to a development of an intersex model law supported by health professionals despite a hostile environment. Their media engagement also resulted in nuanced positive reporting on YKP and SRHR in Zambia.

[1] Groups such as Transbantu Zambia and Friends of Rainka

**Conclusions/Next steps**: The model teaches us that despite growing homophobia, investing in YKP using youth and health as entry‐points is key to ending AIDS. In an era of increased online presence, positive media discourse is important to shifting social norms and acceptance of YKPs.

### OAD0605 Young key populations speak out! This is what we want

A.N. Kyendikuwa ^1^, J. Ouma^1^

^1^Global Network of Young People Living with HIV, Cape Town, South Africa

**Background**: The Love Alliance is a five‐year programme created to address the challenges in health and human rights that members of the Key Population group in all their diversities experience in their countries.In August 2021, Y+ Global alongside GNP+ convened a virtual global consultation with Young Key Populations(YKP) with the objective of understanding the advocacy visions and voices of young people within PWUD, LGBTQI, sex worker,s and PLHIV movements in the different regions to inspire Y+ Global, GNP+ and the Love Alliance in its work around young key populations living with or affected by HIV. The consultations were to provide a better understanding of the issues affecting the cohort, interventions needed and provide a focus on advocacy for the Love Alliance.

**Description**: The virtual consultation was attended by 31 participants ,from more than 15 countries across the African continent and one country from Asia. The countries represented included; Zimbabwe, Lebanon, Kenya, Uganda, South Africa, Angola, Morocco ,Nigeria, Zambia, Malawi, Burundi, Mozambique , Tanzania, Eswatini and Vietnam. The technicalities of the session including recordings and translations and identity sharing were shared with the participants.To promote inclusivity and active participation of the participants,the session had French and Portuguese translations as well as language based break out rooms.

**Lessons learned**: Gender inequality and criminalization, Stigma and discrimination, access to quality and YKP friendly HIV/SRH services and mental health issues are the major issues affecting YKP across the African continent hence they need revamped advocacy efforts, the main trends such as tokenistic and unethical engagement of YKP, criminalization and oppressive laws and policies such as age of consent within the spaces impact YKPLHIV advocacy and the disruptions of COVID‐19.

**Conclusions/Next steps**: YKP need to participate in key spaces, activities and calendar moments that impact their lives. Y+ global under the love alliance continues to support the online and offline engagement of YKP and to strengthen youth‐led advocacy with the recommendations that the participants provided.

### OAD0702 Social support attenuates the syndemic of poor HIV care and stigma on suicidal tendencies among South African young women living with HIV

W. Saal ^1^, B. Banougnin^1^, L. Hertzog^1^, E. Toska^1,2,3^, L. Cluver^3,4^

^1^University of Cape Town, Centre for Social Science Research, Cape Town, South Africa, ^2^University of Cape Town, Department of Sociology, Cape Town, South Africa, ^3^University of Oxford, Department of Social Policy and Intervention, London, United Kingdom, ^4^University of Cape Town, Department of Psychiatry and Mental Health, Cape Town, South Africa

**Background**: Young women living with HIV (YWHIV) are at increased risk of developing suicidality. Yet little is known about the combined effects of living with HIV on suicidality among YWHIV.

**Methods**: We analysed data from 791 YWHIV who participated in two large prospective South African cohort studies, Mzantsi Wakho (2017‐2018) and HEY BABY (2018‐2019). Standardised questionnaires were used to measure the syndemic factors, mental health issues (mediator), social support (moderator) and suicidality (outcome). A moderated mediation analysis was used to investigate (1) the direct (individual and syndemic) effects of poor HIV care retention and HIV‐related stigma on suicidality, (2) the indirect effects as mediated through mental health issues, and (3) the moderating effects of social support on mental health issues, and suicidality, controlling for potential confounders (age, rural residence, informal housing, household poverty, and mode of HIV acquisition).

**Results**: Higher HIV‐related stigma (AOR=2.19, *p*=0.012) and poor HIV care retention (AOR=3.47, *p* < 0.001) were directly associated with higher suicidality. The combined direct effects of poor HIV care retention and HIV‐related stigma on suicidality (14% points increased risks of suicidality, *p*<0.001) are higher than their individual effects (12% points increased risks of suicidality,*p*<0.001). Furthermore, both HIV‐related stigma and poor HIV care retention were indirectly associated with suicidality via mental health issues. Social support buffered the direct and indirect effects of poor HIV care retention and HIV‐related stigma on suicidality.

**Conclusions**: Combined effects of poor HIV care retention and HIV‐related stigma are associated with higher suicidality, indicating a syndemic interaction. Programmes that simultaneously address these factors and strengthen social support services may improve mental health and reduce suicidality among YWHIV.

### OAD0703 Mental health services utilization among young Black gay, bisexual, and other men who have sex with men living with HIV

K. Doraivelu ^1^, D. Camp^1^, S. Moore^1^, A. Kalokhe^1,2^, M. Ali^1,2^, E. Farber^2^, S. Hussen^1,2^

^1^Rollins School of Public Health, Emory University, Hubert Department of Global Health, Atlanta, United States, ^2^Emory University School of Medicine, Atlanta, United States

**Background**: Mental health (MH)comorbidities are prevalent amongyoung Black gay, bisexual, and other men who have sex with men(YB‐GBMSM) living with HIV. However, it remains unclear what factors are associated with utilization of MH services among YB‐GBMSM engaged in HIV care.

**Methods**: We conducted a cross‐sectional survey of YB‐GBMSM from two HIV clinics. Utilization of MH services was defined as at least one self‐reported MH visit in their lifetime. Psychological symptoms were assessed using the Generalized Anxiety Disorder assessment‐7, Center for Epidemiologic Studies Depression scale, Primary Care Post‐Traumatic Stress Disorder Screen, and self‐reported substance use in the last six months. Multivariate logistic regression models were used to evaluate covariates of lifetime MH care utilization.

**Results**: Among 100 YB‐GBMSM, over half (51%) reported utilizing MH services, and 40% had been referred to a MH provider in the past year. In multivariate logistic regression analyses, non‐organizational religious activity (OR: 1.33, CI: 1.01‐1.77), severe anxiety (OR: 5.23, CI: 1.08‐25.26), and homelessness in the past three months (OR: 4.03, CI: 1.08‐15.07) were associated with MH care utilization. HIV stigma, discrimination in medical settings, and other psychological symptoms (depression, trauma, substance misuse) were not associated with utilization of MH services.

**Conclusions**: Our findings that MH utilization was associated with homelessness, NORA, and severe anxiety suggest that service providers should consider promoting MH services to a wider range of YB‐GBMSM clients, specifically to clients that do not present with psychological symptom complexes. Additionally, future research should explore the complex relationships between religiosity and MH.

### OAD0704 Loneliness and ARV adherence: results from a cohort study of people living with HIV in Ontario, Canada

L. Light^1^, C. Hui^2,3^, T. Hart^3,4^, D. Brennan^5^, A.E. Kroch ^6,1,4^

^1^Ontario HIV treatment Network, OHTN Cohort Study, Toronto, Canada, ^2^Ontario Positive Asians, Toronto, Canada, ^3^Ryerson University, Toronto, Canada, ^4^Dalla Lana School of Public Health, University of Toronto, Toronto, Canada, ^5^University of Toronto, Factor‐Inwentash Faculty of Social Work, Toronto, Canada, ^6^Public Health Ontario, Toronto, Canada

**Background**: Social connectedness is important to human beings while loneliness can be detrimental to both mental and physical health. Our goal was to investigate the degree of loneliness experienced by people living with HIV (PLWH) and its impact on adherence to ARV medications using Ontario HIV Cohort Study (OCS).

**Methods**: OCS, a study of people Living with HIV (PLWH), collects clinical and socio‐behavioral data from 15 HIV clinics in Ontario, Canada. In 2020, a 3‐item short UCLA Loneliness Scale was added to the annual questionnaire. A loneliness score was categorized into 3 levels (low:3‐4, medium:5‐6, high:7‐9). Descriptive statistics and proportional odds models were used to identify factors correlated with loneliness. Impact of loneliness on ARV adherence was examined using logistic regression.

**Results**: Mean age (STD) of the 1,870 participants who completed loneliness scale was 52.2 (12.1), 22.8% were females, 57.6% gay men, 60% white, 21.7 % black, and 2.5% Indigenous. 19.6% of participants often felt lacking companionship, 13.2% felt left out, and 17.9% felt isolated.Meanwhile, the majority hardly ever lacked companionship (49.6%), felt left out (60.7%) or isolated (53%). On the combined loneliness scale (range= 3‐9, median (IQR) = 3(4‐6)), 27.1% had medium level, and 20.7% had high level. Multivariable analysis reveals predictors of higher loneliness Score with OR(95%CI) are younger age 2.84(1.80,4.48) for <35, 3.18(2.22,4.55) for 35‐49, 2.18(1,57,3.03) for 50‐64 vs 65+ years old), being female 1.38(1.06,1.79), single 4.04(3.26,5.00), low income 1.51(1.23,1.85) for <$20,000/year, and using alcohol 1.49(1.08,2.07) and non‐medicinal drugs 1.53(1.13,2.07). We found strong association between ARV non‐adherence and higher loneliness scores [OR(95%CI)=1.45(1.14,1.84) for medium vs low, and 1.41(1.08,1.85) for high vs low)], adjusted for age, sex, race, alcohol, and drug use.

**Conclusions**: PLWH with higher loneliness scores are more likely to skip their ARV medications. About 13% to 20% of OCS participants experienced at least one of the three aspects of loneliness, almost half have high scores on the combined loneliness scale. More research is needed to identify relationship between loneliness and co‐morbid conditions, such as substance use. Intervention strategies are need in communities of PLWH to combat loneliness, especially during current COVID‐19 pandemic.

### OAD0705 HIV and suicide risk across adolescence and young adulthood: an examination of sociodemographic, contextual, and psychosocial risk factors for attempted suicide in a longitudinal cohort of youth affected by HIV

P. Kreniske ^1^, C. Morrison^2^, B.H. Spencer^3^, A. Levine^4^, L. Liotta^2^, P.W. Fisher^5^, N. Nguyen^6^, R.N. Robbins^1^, C. Dolezal^1^, L. Kluisza^1^, A. Wiznia^7^, E.J. Abrams^8,9^, C.A. Mellins^1^

^1^HIV Center for Clinical and Behavioral Studies, New York State Psychiatric Institute and Columbia University, Psychiatry, New York, United States, ^2^HIV Center for Clinical and Behavioral Studies, New York State Psychiatric Institute and Columbia University, New York, United States, ^3^Mailman School of Public Health, Columbia University, Department of Population and Family Health, New York, United States, ^4^Research Foundation for Mental Hygiene, Mental Health Data Science, New York, United States, ^5^New York State Psychiatric Institute and Columbia University, Child and Adolescent Psychiatry, New York, United States, ^6^The Aaron Diamond AIDS Research Center, New York, United States, ^7^Jacobi Medical Center, Albert Einstein College of Medicine, Bronx, United States, ^8^ICAP at Columbia University, Mailman School of Public Health, New York, United States, ^9^Columbia University, Vagelos College of Physicians & Surgeons, New York, United States

**Background**: Risk for attempting suicide increases dramatically as children become adolescents and young adults (AYA), with chronic health conditions being a risk factor. To date, no studies have examined correlates of suicidality across development in AYA living with perinatal HIV‐infection (AYALPHIV) and those perinatally HIV‐exposed but uninfected (AYAPHEU). Findings can inform much‐needed interventions to support AYALPHIV and AYAPHEU as they age.

**Methods**: Data come from a longitudinal New York City‐based study of health and psychosocial functioning in AYALPHIV and AYAPHEU (mean enrollment age = 12 years; current mean age = 27 years) interviewed every 12‐18 months. Psychiatric disorders and first‐reported suicide attempt were assessed with the DISC. Generalized estimating equations were used to examine associations between first‐reported suicide attempt and sociodemographic, contextual, and psychosocial correlates measured concurrently across 6 time‐points.

**Results**: At enrollment, 51% were female, 72% heterosexual, 57% Black, and 50% Latinx. Attempted suicide was significantly higher among AYALPHIV (27%) than AYAPHEU (16%) (OR = 1.74, p = 0.02). In the full and AYALPHIV samples, sexual minority identity, lower self‐concept, negative life events, and past‐year arrest were associated with increased odds of attempted suicide. For all groups, past‐year anxiety, mood, or behavior disorders were associated with increased odds of attempted suicide. Among AYALPHIV, pregnancy and HIV stigma were associated with increased odds of attempted suicide. Interactions by HIV status and age group were found: substance use was more strongly associated with attempted suicide among AYAPHEU than AYALPHIV, while negative life events and higher religiosity were more strongly associated with increased odds of attempted suicide among AYA ages 19 and older than those 18 and younger.

**Conclusions**: Adolescence and young adulthood is a critical period when risk for attempted suicide rises precipitously. As our cohort aged into adulthood sociodemographic, contextual and psychosocial factors placed them at risk for suicidality and only higher self‐concept was protective. Unique risks for attempted suicide were evident by PHIV‐status with HIV stigma and pregnancy impacting AYALPHIV, and substance use a risk among AYAPHEU. Assessing for suicide risk and correlates with attention to aging can inform preventive interventions tailored to meet AYALPHIV and AYAPHEU needs.

### OAD0802 Intersecting stigma experiences among refugee youth sexual violence survivors in a humanitarian context in Uganda: implications for HIV cascade engagement in conflict settings

C. Logie ^1^, M. Okumu^2^, A. McAlpine^1^, M. Loutet^3^, N. Kisubi^4^, S. Odong^4^

^1^University of Toronto, Social Work, Toronto, Canada, ^2^University of Illinois Urbana‐Champaign, Urbana, United States, ^3^University of Toronto, Toronto, Canada, 4Uganda Refugee and Disaster Management Council, Yumbe, Uganda

**Background**: Youth sexual violence survivors’ experiences accessing HIV services in the aftermath of humanitarian crises are understudied. This is a notable gap as refugee youth disproportionately experience sexual violence. We explored experiences accessing HIV and post‐rape care among refugee youth sexual violence survivors in Bidi Bidi refugee settlement in Uganda.

**Methods**: We conducted a community‐based qualitative study in Bidi Bidi, the world's second largest refugee settlement, implementing purposive sampling to recruit young refugee survivors (aged 16‐24), community elders (aged>50), and service providers working in Bidi Bidi. We conducted 6 focus groups and 12 in‐depth individual interviews (IDI) with refugee youth, 8 IDI with elders, and 10 IDI with service providers. We applied thematic analysis informed by the Health Stigma and Discrimination Framework to explore deductive and inductive themes associated with HIV and post‐rape care engagement following sexual violence.

**Results**: Participants included: 60 youth (n=30 men; n=30 women; mean age: 20.9, standard deviation [SD]: 2.17) from South Sudan (83.3%) and the Democratic Republic of Congo (DRC) (16.7%), and 10 elders (n=4 women, n=4 men; mean age: 58.3, SD: 3.88) from South Sudan (87.5%) and the DRC (12.5%). Healthcare providers (n=5 men, n=5 women; mean age 31.5, SD: 4.88) included midwives (n=4), clinical officers (n=4), a nurse (n=1) and a lab technician (n=1). Participant narratives revealed profound experiences of sexual violence stigma, including blame, shame, and mistreatment. This stigma was gendered, particularly targeting young women. Sexual violence stigma often resulted in young women's forced/early marriage to violence perpetrators. HIV stigma contributed to further violence exposure following an HIV diagnosis. Fear of HIV and sexual violence stigma presented substantial barriers to accessing post‐rape care. Stigma spanned social‐ecological levels, including mistreatment and rejection from family, friends, health providers and community members, contributing to internalized stigma. Limited emergency contraception access following sexual violence resulted in unwanted and early pregnancy, further exacerbating social exclusion.

**Conclusions**: Findings signal the intersection of sexual violence stigma, HIV stigma, and inequitable gender norms reduces post‐rape care engagement and harms wellbeing among refugee youth. Gender transformative, multi‐level intersecting stigma reduction strategies can both increase HIV cascade engagement and advance sexual rights with refugee youth.

### OAD0803 From homophobic and sexist attitudes to tolerance toward LGBTQ+ individuals: critical consciousness as a tool against stigma

R. M. Pinto ^1^, C. Lee^2^, M. Arthur^1^, L. Windsor^1,2^

^1^University of Michigan, Social Work, Ann Arbor, United States, ^2^University of Illinois Urbana‐Champaign, Social Work, Urbana‐Champaign, United States

**Background**: Research shows that Critical Dialogues (CD) after viewing illustrations of personal and historic oppression can help people develop critical consciousness and address internalized prejudices. This NIH‐funded study uses data from recorded critical dialogue sessions from a behavioral group intervention to reduce substance use and recidivism among men. We focused on homophobia and sexist attitudes as these are public health issues that predict harmful behavior. Critical consciousness raising – understanding of and action against structural roots of oppression – takes place when an individual moves from internalized prejudice to acceptance of self and others.

**Methods**: We used qualitative data collected during 13 sessions where 28 men of color with histories of incarceration and substance misuse (2‐4 per group) viewed illustrations concerning gay/lesbian relationships. Four coders used NVivo to code transcripts and develop codes in an iterative process. We open‐coded independently, discussed preliminary codes, and came to a 100% agreement about codes. Saturation occurred after 10 transcripts. “Member check” involved a Collaborative Board who reviewed findings and contributed to data interpretation.

**Results**: We identified four key themes – *Critical Appraisal* and *Critical Reactions* toward the illustrations; and *Critical Reflections* and *Critical Consciousness* around 14 sub‐themes. The initial discussion revolved around homophobic and sexist attitudes involving pejorative and stigmatizing terms – e.g., “*I don't like men datin’ men. That's fuckin’ disgustin’*.”Halfway through the sessions, participants slowly understood that stigmatization supported oppressive structures that impacted them – e.g., *“Oh, man, why do I think like that? Are there other things that I need to consider?”* Participants realized that clinging to beliefs/morals used to uphold homophobia and sexism was, in their words, “crazy” – e.g., “…*because your family is a certain way, now you hate this person. How could you hate somebody if you don't even know ‘em?”*

**Conclusions**: By engaging in CD, participants progressed from homophobic and sexist attitudes to greater understanding toward LGBTQ+ individuals and women. CD inspired by illustrations can help individuals develop critical consciousness around myriad issues that may help abate other forms of oppression, such as racism and xenophobia.

### OAD0804 Partner social support and sexual satisfaction is associated with lower anticipated HIV stigma in Malawian couples living with HIV

A. Conroy ^1^, S. Gutin^1^, A. Ruark^2^, L. Darbes^3^, T. Neilands^1^, J. Mkandawire^4^

^1^University of California San Francisco, Center for AIDS Prevention Studies, San Francisco, United States, ^2^Wheaton College, Applied Health Sciences, Wheaton, United States, ^3^University of Michigan, School of Nursing, Ann Arbor, United States, ^4^Invest in Knowledge, Zomba, Malawi

**Background**: Fear of HIV stigma can lead to non‐disclosure of HIV status to partners and negative impacts on couple relationships; at the same time, partner support has the potential to reduce the harms of HIV stigma on health. Little research has focused on couples who have disclosed their HIV status and whether a supportive relationship could protect against anticipated experiences of HIV stigma outside the partnership. We investigated the association between HIV stigma and relationships dynamics in couples living with HIV to identify dyadic targets for intervention.

**Methods**: Married couples (N=211) with at least one partner on antiretroviral therapy were recruited from HIV clinic waiting rooms in Zomba, Malawi. Partners completed separate surveys on anticipated HIV stigma outside of the relationship and relationship dynamics (e.g., intimacy, trust, sexual satisfaction, general social support from the partner, and couple communication patterns). Linear mixed models tested for associations between relationship dynamics and anticipated stigma, and whether this association varied by gender, after controlling for socio‐demographics and relationship characteristics.

**Results**: Couples were together for 12.5 years, on average, and two‐thirds were sero‐concordant positive. In multivariable models, higher sexual satisfaction (b=‐0.22, 95%CI= ‐0.41; ‐0.03, p=0.020) and partner social support (b=‐0.02, 95%CI=‐0.04; ‐0.01, p=0.006) were associated with less stigma, while negative communication styles such as withdrawal (b=0.13, 95%CI=0.04; 0.21, p=0.003), demanding (b=0.17, 95%CI=0.09; 0.24, p<0.001), and avoidant communication (b=0.26, 95%CI=0.13; 0.39, p<0.001) were associated with higher stigma. Associations did not vary by gender.

**Conclusions**: Couple‐based interventions that promote constructive forms of couple communication, strengthen emotional and practical support within couples, and improve sexual satisfaction could reduce extra‐dyadic HIV stigma and its negative impacts on the health of couples living with HIV in sub‐Saharan Africa. Relationship dynamics such as sexual satisfaction and social support may be of equal importance for both men and women in efforts to reduce anticipated HIV stigma.

### OAD0805 Social support, food insecurity and HIV stigma among men living with HIV in rural southwestern Uganda

I. Arinaitwe ^1^, H. Amutuhaire^1^, D. Atwongyeire^1^, E. Tusingweire^1^, P.C. Kawungezi^2^, G.Z. Rukundo^3^, S. Ashaba^3^

^1^Mbarara University of Science and Technology, Faculty of Medicine, Mbarara, Uganda, ^2^Mbarara University of Science and Technology, Faculty of Medicine, Department of Community Health, Mbarara, Uganda, ^3^Mbarara University of Science and Technology, Faculty of Medicine, Department of Psychiatry, Mbarara, Uganda

**Background**: HIV stigma is a significant factor in HIV/AIDS care, influencing uptake of HIV care services including HIV testing, initiation on antiretroviral therapy (ART) and retention in care. This is due to the fear of status disclosure and social discrimination. Internalized HIV stigma has been documented to be more common in men and it has the most impact on treatment adherence among people living with HIV. Information about HIV stigma and its associated factors among men living with HIV (MLWHIV) in rural Uganda is limited. This study determined the burden of HIV stigma and its associated factors among men accessing HIV/AIDS care at a rural health facility in southwestern Uganda.

**Methods**: This was a clinic‐based cross sectional study. We consecutively enrolled 252 adult men accessing HIV/AIDS care at a rural health centre in southwestern Uganda during the Corona virus pandemic. We collected information on sociodemographic information, HIV stigma using the Berger stigma scale, social support using the Multidimensional Scale of Perceived Social Support and food insecurity using the Household food insecurity Access Scale. We fitted modified Poisson regression models to determine the associations between social support, food insecurity and HIV stigma.

**Results**: The mean HIV stigma score of the study participants was 70.08 (SD 19.34) and almost half (48%) had high level HIV stigma. Most participants (75%) reported food insecurity, 5% of whom had severe food insecurity. The risk of HIV stigma was lower among those aged 35 years and above (adjusted risk ratio [ARR]=0.89; 95% CI 0.83–0.96; P=0.003, those who had been on ART for more than 5 years (ARR=0.92; 95% CI=0.84–0.99; P=0.04), and those who had social support (ARR=0.99; 95% CI=0.98–0.99; P=<0.001). Food insecurity was associated with an increased risk of HIV stigma (ARR=1.07; 95% CI 1.00–1.15; P=0.03). Social support moderated the effect of food insecurity on HIV stigma (P=0.45).

**Conclusions**: HIV stigma is common among MLWHIV in rural Uganda and is significantly associated with food insecurity. Social support moderated the effect of food insecurity on HIV stigma. We thus recommend social support interventions and economic empowerment of MLWHIV to improve their HIV treatment outcomes.

### OAE0102 Medical drones to support HIV differentiated service delivery in an island population in Uganda

R. Parkes‐Ratanshi ^1,2^, J. Akullo^1^, J.L. Ssemata^1^, P. Ssesaazi^1^, D. Masoni^1^, J. Germann^3^, T. Mutabazi^4^, T. Pattery^5^, T. Amukele^6^, A. Kambugu^7^, A. Kiragga^7^, R. Kimbui^8^

^1^Infectious Diseases Institute, Academy for Health Innovation, Kampala, Uganda, ^2^University of Cambridge, Clinical School, Cambridge, United Kingdom, ^3^WeRobotics, Zurich, Switzerland, ^4^Uganda Flying Labs, Kampala, Uganda, ^5^Janssen, The Pharmaceutical Companies of Johnson & Johnson, Global Public Health, Beerse, Belgium, ^6^Icon Plc, Dublin, Ireland, ^7^Infectious Diseases Institute, Kampala, Uganda, ^8^Johnson & Johnson, Supply Chain, Nairobi, Kenya

**Background**: Kalangala district, Uganda is comprised of 84 islands in Lake Victoria. These island‐dwelling communities have the highest HIV prevalence (27%) and lost to follow up from HIV care (50%) in Uganda. Delivery of antiretroviral therapy (ART) is a challenge due to the geography and the nomadic nature of the community.

**Description**: Between July – September 2019 we undertook a survey of medical supply chain gaps for HIV care at health facilities in two sub‐counties Bufumira and Mazinga) in Kalangala district. At baseline 5.3% of PLHIV were accessing care from differentiated service delivery (DSD). Most ART refills were managed through outreach visits to remote boat landing sites. Between 50‐90% health care worker time outreaches is spent on ART refills; the remaining spent on maternal and child health and non‐communicable diseases. Annual cost of outreach activities by these facilities boat is approximately $90,000. Data were used to design a quasi‐experimental pilot to evaluate the feasibility and acceptability of using medical drones for ART delivery to peer support groups.[Table jia225935-tbl-0003]

**Lessons learned**: Since September 2021 two DJI Matrice 300 drones have flown from Bufumira health centre to five remote landing sites previously receiving ART through boat outreaches. In September 2021 the medical drone delivered three months of ART to 43 PLHIV in seven DSD groups on three landing sites. In December 2021 drones replaced boat deliveries to 64 PLHIV in 11 DSD groups across five landing sites. Average flight time was 9.3 minutes for medical drone compared to 35 minutes per boat trip. Average distance from Bufumira to landing site was 6.6km (max 10km). Six peer support workers have been sensitised on how to prepare the drone landing pad, secure the area, safely unload ART from the drone and load documentation for return to Bufumira.

**Conclusions/Next steps**: The pilot will continue until June 2022, aiming to deliver to 100 PLHIV. The pilot will be evaluating (i). PLHIV: ART stock outs, lost to follow up, viral load and (ii) impact on health facility: number of DSD groups, number of outreaches and impact on outreach costs. Mazinga health centre and associated landing sites will be used as control comparison.

### OAE0103 How efficient are HIV self‐testing models? A comparison of community, facility, one‐stop‐shop and pharmacy retail distribution models in Nigeria

V.A. Adepoju ^1^, C. Umebido^2^, Z. Hassan^3^, A. Adetosoye^4^, A. Olowu^5^, T.E. Ademola‐Kay^6^, F. Quaitey^7^, U. Ijeoma‐Johnson^8^, C. Laube^9^, K. Grabbe^10^, M. Strachan^11^, H. Anyasi^12^, D. Ndukwe^13^, A. Ajijelek^14^, J. Iseniyi^15^, I. John‐Dada^16^, S. Omoighe^16^, E. Atuma^17^, A. Oniyire^18^, A. Ikpeazu^19^

^1^Jhpiego Nigeria (an affiliate of John Hopkins University), HIV and Infectious Diseases, Abuja, Nigeria, ^2^Jhpiego Nigeria, Strategic Information‐HIV and Infectious Disease, Abuja, Nigeria, ^3^Jhpiego, HIV and Infectious Disease, Abuja, Nigeria, ^4^Jhpiego Nigeria, Technical Advisor‐HIV Testing Services, TMEC/RISE Project, Abuja, Nigeria, ^5^Jhpiego Nigeria, Supply chain Lead, Abuja, Nigeria, ^6^Jhpiego Nigeria, HIV and Infectious Disease‐STAR Project, Lagos, Nigeria, ^7^Jhpiego Nigeria, HIV and Infectious Disease‐STAR Project, Akwa‐Ibom, Nigeria, ^8^Jhpiego, HIV and Infectious Disease‐STAR Project, Porthacourt, Nigeria, ^9^Jhpiego, Senior Technical Advisor‐HIV and Infectious Disease, Maryland, United States, ^10^Jhpiego, HIV and Infectious Disease, Baltimore, Maryland, United States, ^11^Jhpiego, Strategic Information Directorate, Baltimore, Maryland, United States, ^12^FHI 360, Reproductive Health/HIV, Abuja, Nigeria, ^13^National Agency for the Control of AIDS(NACA), Deputy Director, Prevention and SBCC, Abuja, Nigeria, ^14^Jhpiego, Private Sector‐ Demand Side Financing, Abuja, Nigeria, ^15^Jhpiego Nigeria, Laboratory Consultant‐HIV Self Testing, Abuja, Nigeria, ^16^Federal Ministry of Health, National AIDS/STI Control Program, Abuja, Nigeria, ^17^Jhpiego Nigeria, Chief of Party‐TMEC/RISE Project, Abuja, Nigeria, ^18^Jhpiego Nigeria, Country Director, Abuja, Nigeria, ^19^Federal Ministry of Health, National Coordinator‐National AIDS/STI Control Program, Abuja, Nigeria

**Background**: In many Nigerian states, more than 95% of persons living with HIV now know their status. Nigeria is scaling up HIV self‐testing (HIVST) to close testing gaps among populations not reached with conventional testing. Jhpiego, in close partnership with the National AIDS and STDs Control Program within the Federal Ministry of Health and National Agency for the Control of AIDS, has been providing catalytic support to enable the scale up of HIVST in Nigeria under the Unitaid‐funded Self‐Testing Africa (STAR) initiative since 2020.

**Methods**: We compared HIVST outcomes across 4 distribution models implemented by non‐governmental and community‐based organizations in Nigeria between October‐December 2021, with technical and commodity support from STAR. A community‐based distribution model targeted men, key populations (KP), adolescents, young people, and orphans and vulnerable children in hotspots within high burden Nigerian states; a facility‐based model offered primary and secondary distribution via various healthcare services; KP one‐stop shops (OSS) integrated HIVST distribution within their services, and a pharmacy model provided HIVST at reduced cost equivalent to USD 2.00.

**Results**: HIVST cascade data were collected, documented in relevant HIVST tools and descriptively analyzed as shown in table 1.

**Abstract OAE0103‐Table 1 jia225935-tbl-0003:** 

		Distributed	Results returned %	Reactive %	Linked to testing %	Concordant test %	Linked to ART
Community		33023	33023 (100%)	192 (0.6%)	156 (81%)	124 (79%)	116 (94%)
Facility		15225	15100 (99%)	263 (1.7%)	250 (95%)	240 (96%)	235 (98%)
KP OSS		10301	10149 (99%)	340 (3.4%)	340 (100%)	338 (99%)	338 (100%)
Pharmacy		245	238 (97%)	1 (0.4%)	0	N/A	N/A

**Conclusions**: HIV testing yield and performance across the cascade was optimized through KP OSS. Trends warranting examination include 1) low yield in community and pharmacy settings suggest need for better targeting; 2) the absence of confirmatory testing following reactive tests from pharmacy distribution suggests a need for linkage support; 3) low test concordance in community‐based distribution suggests possible test or data quality issues; and, 4) high linkage to ART across models, with potential to improve following community‐based HIVST. More research is needed to understand the potential of each model as the Nigerian program advances.

### OAE0104 How soon should patients be eligible for differentiated service delivery models for antiretroviral treatment?

L. Jamieson^1,2^, S. Rosen ^3,1^, B. Phiri^4^, A. Grimsrud^5^, M. Mwansa^6^, H. Shakwelele^4^, P. Haimbe^4^, M.M. Mwenechanya^7^, P. Lumano‐Mulenga^6^, I. Chiboma^6^, B.E. Nichols^3,2,1^

^1^University of Witwatersrand, Health Economics and Epidemiology Research Office, Johannesburg, South Africa, ^2^Amsterdam University Medical Centre, Department of Medical Microbiology, Amsterdam, Netherlands, the, ^3^Boston University School of Public Health, Department of Global Health, Boston, United States, ^4^Clinton Health Access Initiative, Lusaka, Zambia, ^5^International AIDS Society, Cape Town, South Africa, ^6^Ministry of Health, Lusaka, Zambia, ^7^The Centre for Infectious Disease Research in Zambia, Lusaka, Zambia

**Background**: Attrition from HIV treatment (ART) is highest during patients’ first 6 months after initiation. Although ≥6 months on ART is an eligibility criterion for most differentiated service delivery (DSD) model guidelines, some patients enroll earlier. We used routinely‐collected data on DSD models in Zambia to evaluate loss to follow‐up (LTFU) comparing patients enrolling in DSD models early vs those who did so later (>6 months).

**Methods**: We extracted data from electronic medical records for adults (≥15 years) initiated on ART between 01/01/2019 and 31/12/2019 and evaluated LTFU (>90 days late for last scheduled medication pickup) at 18 months for “early enrollers” (DSD enrolment within <6 months on ART) and “established enrollers” (DSD enrolment with >6 months on ART). We used a log‐binomial model to compare LTFU risk between groups, adjusting for age, sex, urban/rural status, ART refill intervals and DSD model.

**Results**: For 6,340 early enrollers and 25,857 established enrollers, there were no important differences between the groups by sex (61% female), age (median 37 years), or setting (65% urban). ART refill intervals were longer for established vs early enrollers (72% vs 55% were given 4‐6 month refills). LTFU at 18 months was 3% (192/6,340) for early enrollers and 5% (24,646/25,857) for established enrollers. Early enrollers were 41% less likely to be LTFU than established patients (adjusted risk ratio [95% confidence interval] 0.59[0.50‐0.68]).

**Figure:**
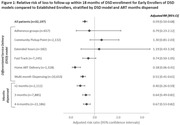
**Abstract OAE0104‐Figure 1**.

**Conclusions**: Patients enrolled early after ART initiation in DSD models in Zambia were more likely to be retained in care than patients referred after they were established on ART. A limitation of the analysis is that early enrollers may have been selected for DSD participation due to providers’ and patients’ expectations about future retention. Offering DSD model entry to at least some ART patients <6 months after ART initiation may help address high attrition during the early treatment period.

### OAE0105 The effect of six‐month PrEP dispensing supported with interim HIV self‐testing on PrEP continuation at 12 months in Kenya: a randomized implementation trial

K.F. Ortblad ^1^, P. Mogere^2^, A.R. Bardon^3,4^, C. Kiptinness^2^, D. Mangale^3^, S. Gakuo^2^, S. Mbaire^2^, J. Nyokabi^2^, K. Thomas^3^, N.R. Mugo^5,3,2^, J.M. Baeten^3,4,6,7^, K. Ngure^8,2^

^1^Fred Hutchinson Cancer Research Center, Public Health Sciences Division, Seattle, United States, ^2^Partners in Health and Research Development, Thika, Kenya, ^3^University of Washington, Department of Global Health, Seattle, United States, ^4^University of Washington, Department of Epidemiology, Seattle, United States, ^5^Kenya Medical Research Institute, Center for Clinical Research, Thika, Kenya, ^6^University of Washington, Department of Medicine, Seattle, United States, ^7^Gilead Sciences, Foster City, United States, ^8^Jomo Kenyatta University of Agriculture and Technology, Department of Community Health, Nairobi, Kenya

**Background**: In Kenya, HIV pre‐exposure prophylaxis (PrEP) is primarily delivered at HIV clinics where client barriers to continuation include privacy concerns, long wait times, and setting‐associated stigma. Six‐month PrEP dispensing supported with interim HIV self‐testing (HIVST) would reduce the number of clinic visits and potentially address some of these continuation barriers.

**Methods**: We conducted a non‐inferiority trial to test this model of PrEP delivery in Thika, Kenya. Eligible participants were PrEP clients ≥18 years who returned for their first one‐month follow‐up visit. We randomized participants 2:1 to: 1) six‐month PrEP dispensing with semiannual clinic visits and interim HIVST at three months, or 2) standard‐of‐care (SOC) PrEP delivery with quarterly clinic visits and clinic‐based HIV testing. Pre‐specified outcomes at 12 months included HIV testing (any in past six months and ≥2 times since enrollment) and PrEP refilling (at 12 months and both six and 12 months). We used binomial regression models, adjusted for sex and serodifferent partnership status, to estimate risk differences (RDs) and interpreted one‐sided 95% confidence interval (CI) lower bounds (LB) ≥‐10% as non‐inferior.

**Results**: From May 2018 to February 2020, we enrolled and followed 495 participants. At 12 months, 73.3% (241/329) in the intervention and 72.3% (120/166) in the SOC arm returned to clinic. In the intervention arm, 69.9% (230/329) tested for HIV in the past six months and 72.3% (238/329) tested ≥2 times since enrollment, compared to 69.9% (116/166, RD ‐0.3%, 95% CI LB ‐7.4%) and 71.7% (119/166, RD 0.2%, 95% CI LB ‐6.8%) in the SOC arm, respectively. Additionally, 59.6% (196/329) in the intervention arm refilled PrEP at 12 months compared to 62.7% (104/166) in the SOC arm (RD ‐3.3%, 95% CI LB ‐10.8%, thus failing to demonstrate non‐inferiority). However, 56.5% (186/329) in the intervention and 56.6% (94/166) in the SOC arm (RD ‐0.2%, 95% CI LB ‐7.9%) refilled PrEP at both at six and 12 months.

**Conclusions**: Six‐month PrEP dispensing with interim HIVST resulted in high PrEP continuation at one year. HIV testing and PrEP refilling were generally comparable to SOC PrEP dispensing. This novel model has the potential to optimize PrEP delivery in Kenya and similar settings.

### OAE0202 Responding to the challenges of a dual pandemic: how outreach mobile health clinics maintained HIV testing and linkage services in the face of COVID‐19

N. Khozomba ^1^, J. Jere^1^, M. Phiri^1^, E. Geoffroy^2^, M. Brostrom^2^

^1^GAIA Malawi, Limbe, Malawi, 2GAIA Global Health, Oakland, United States

**Background**: In September 2021, The Global Fund declared COVID‐19 a major setback in the fight against HIV/AIDS, reporting that HIV testing services (HTS) dropped 22% over the previous year globally. In Malawi, the COVID‐19 pandemic threatens the dramatic progress made in recent years to achieve UNAIDS 95‐95‐95 targets, particularly in hard to reach communities.

**Description**: GAIA operates outreach mobile clinics, improving access to integrated HIV and primary health care services for communities far off the healthcare grid. In a public‐private partnership with Malawi District Health Offices, seven clinics serve 35 sites weekly across three districts, where adult HIV prevalence is 16%, providing 250,000 client visits per year. In these remote regions, GAIA's HTS and linkage to care are critical to sustain epidemic control.

During the pandemic, GAIA mobile clinics maintained access to these essential health services for our vulnerable, rural clients through smart pivots. GAIA added staff to reduce client visit length and promote distancing; protected staff, clients and government partners with PPE; altered clinic workflow and care protocols to enhance safety; and improved hand‐washing protocols and ventilation.

**Lessons learned**: In stark contrast to the global trend, HIV testing at GAIA mobile clinics increased by 22%, averaging 1272 tests per clinic between April 2020‐December 2021 compared with 1042 during the 21 months prior. Of the 7,736 people tested, 86% were female and 3% tested positive (3% of females, 5% of males), of whom 90% were successfully reached for follow‐up and 88% initiated ART.

During this period, the clinics operated 98% of the time, missing only 58 of 2715 clinic working days (83% COVID‐related closures), providing HIV and COVID‐19 related health talks to 54,761 and 242,358 attendees respectively, and facilitating vaccinations by government community health workers at mobile clinic sites.

**Conclusions/Next steps**: COVID‐19 challenged many health providers' ability to provide HIV testing services, threatening progress in the fight against HIV/AIDS. Leveraging community trust and local government partnership, GAIA's mobile clinics maintained and increased access to high quality, easily accessible HTS throughout the pandemic. This flexible, community‐based outreach approach to care provision is an effective model for rural health system strengthening and rapid crisis response.

### OAE0203 Expanding role of village health workers in Lesotho: from supporting HIV patients to COVID‐19 contact tracing

A.T. Andom ^1^, L. Ntlamelle^1^, M. Matoko^1^, J. Mabathoana^1^, P. Chetane^1^, M. Chere^1^, P. Nkundanyirazo^1^, T. Mohlouoa^1^, S. Motsamai^1^, R. Tlali^1^, M. Mokoena^1^, M. Msuya^1^, M. Asfaw^1^, M. Zulu^1^, M. Ndayizigiye^1^

^1^Partners In Health, Maseru, Lesotho

**Background**: The COVID‐19 globally has strained health systems, particularly in developing countries like Lesotho, which have experienced a shortage of health workers during the pandemic. Since 2006, Partners In Health Lesotho (PIHL) has been supporting comprehensive HIV services at seven health centers in hard‐to‐reach areas of Lesotho. Prior to the pandemic, PIH had engaged and trained two village health workers (VHW) in each village, one who is dedicated to providing services to HIV patients and the second who is dedicated to maternal and child health.

**Description**: PIHL collaborated with the Ministry of Health of Lesotho to expand the scope of the work of the VHWs who support HIV patients to include COVID‐19 screening and contact tracing. Through this intervention, 938 VHWs were trained on COVID‐19 symptoms, infection prevention and control measures, contact tracing and case reporting. The VHWs received three day in‐person training and personal protective equipment (PPE), including face masks, face shields and hand sanitizers. The VHW coordinator and health facility staff supervised and coached the VHWs to undertake COVID‐19 related activities in addition to HIV patients' care at the community level.

**Lessons learned**: From May 2020 to December 2021, 87,634 people were screened for COVID‐19, 430 clients were diagnosed with COVID‐19, and 444 were contacts traced and isolated with the help of VHWs. During this time, VHWs continued to support 5,279 HIV patients, which was comparable to a pre‐pandemic patient load (N=5168). Between January 2020‐December 2021, we completed 10,684 viral load tests, which was only a 7% reduction from the 11,533 tests performed during the two previous years (Jan 2018‐December 2019). However, average testing coverage during the pandemic remained greater than one test per patient per year. From January 2020 to December 2021, 9,737 viral loads tests (91%) were virally suppressed (<1,000 copies/ml), which was a higher proportion than observed during the previous two years (January 2018 to December 2019, N=9,048, 78.5%).

**Conclusions/Next steps**: Providing VHWs with PPE and training to integrate COVID‐19 infection services into their routine HIV prevention and care activities was an effective strategy to staff COVID‐19 prevention and control programs without sacrificing high‐quality HIV care.

### OAE0204 MPOWER yourself: an online HIV self‐test service to increase testing among gbMSM during COVID‐19 restrictions

A. Shanley ^1^, D. Quinlivan^1^

^1^HIV Ireland, MPOWER Programme, Dublin, Ireland

**Background**: Opportunities to test for HIV in Ireland have increased in recent years, however, the COVID‐19 crisis resulted in complete closure or significantly restricted access to HIV testing services across the country. In November 2020, the MPOWER Programme at HIV Ireland devised a free online HIV self‐test ordering service to bridge the gap created by COVID‐19 restrictions and increase HIV testing among gbMSM.

**Description**: An online portal was developed that allows gbMSM to order a free HIV self‐test and have it delivered to their home address. MPOWER staff is available via helpline, WhatsApp, email, and on Zoom to assist in the use of the kit, and to offer support and referrals if needed. Demographic and behavioral data is collected during the ordering process and is stored separately from personal data. Service users receive an SMS two weeks after dispatch with a link to an evaluation survey.

**Lessons learned**: Such was demand, the total project supply of 2,000 HIV self‐test kits were ordered within 13 days of launching the service. With the support of a mix of funding partners, the service resumed with limited weekly availability from April 2021 to the end of that year. 3,572 people in total received a self‐test, 78% identified as gbMSM. 58% of users were aged 17‐29. Across all users, 23% had never tested for HIV before. Notably, 95% of gbMSM were not using PrEP, of these men 32% had condomless anal sex in the 3 months prior to testing. 13 gbMSM received a reactive result, 5 reported their result through our support team, and 8 through the evaluation form. The evaluation received a response rate of 33%. Inability to access a sexual health service (20%), prefer not to attend a clinic or GP (20%), and having never tested before (28%) were cited as reasons for using the service.

**Conclusions/Next steps**: Online HIV self‐testing services offer a unique opportunity to increase access to testing, engage first‐time testers and key populations who experience barriers, including those created by COVID‐19 restrictions. The development of a bespoke online portal allowed a seamless connection between data collection, analysis, kit dispatch, support, and referrals.

### OAE0205 “Endeavor to reach the furthest behind first”: promoting HIV prevention services using social media intervention in the era of COVID 19 among FSW in Benue State

B. Okeke ^1^, P.E. Durojaye^2^, E. Maduabum^3^

^1^Concerned Women International Development Initiative, Benue, Nigeria, ^2^University of Western Cape, Cape Town, South Africa, ^3^University of Abuja, Sociology, Abuja, Nigeria

**Background**: Sex workers in Nigeria are affected by the COVID‐19 pandemic . Also, lockdown and other measures by government to reduce the spread of COVID 19 limit access to HIV prevention services among sex workers. It is against this backdrop that CWIDI with support from Restless development fund uses social media to promote HIV prevention service among FSW.

**Description**: 20 FSW were trained as social media mobilizers to engage FSW through social networking platforms such as Facebook, WhatsApp, and Instagram. IEC materials were developed in local dialects and posted on selected social media platforms to raise awareness regarding HIV prevention strategies as well as COVID 19 prevention messages for FSW. The trained social media mobilizers engaged FSW that access these platforms through one–on‐one interpersonal communication and provided HIV testing and refereed FSW for COVID‐19 test.

**Lessons learned**: Data from August to December 2020 shows that social media is a safe space for communication among FSW and an effective tool to reach more FSW. 400 FSW were recruited through social media. 320 (80%) had not been tested for HIV within the last six months. Physical outreach reached 112 FSW; 52(46.4%) had not been tested within the last six months. Out of 320 recruited through social media who has not been tested for HIV within the last six month. 300 (93.8%) got tested for HIV, 56(18.7%) tested positive while 244(81.3%) tested Negative. Out of 52(46.4%) FSW reached through physical outreach who has not been tested for HIV within the last six months 40 (76.9%) tested negative while 12(23.1%) were positive. 20% of FSW recruited through social media engaged in inconsistent use of condoms compared to 60% identified through physical out reach. HIV positivity rate was higher among those reached through social media outreach compared to those reached through physical outreach. 100% of FSW reached through social media outreach got tested for COVID 19

**Conclusions/Next steps**: Linking FSW to services through social media has shown to deliver higher HIV prevention result among FSW. Hence, implementing partners should use social media as an effective tool for sharing HIV behavior change messages alongside COVID 19 messages for FSW.

### OAE0302 The effect of a conditional cash transfer program on AIDS morbidity and mortality among the poorest: a quasi‐experimental study of a cohort of 22.7 million Brazilians

A.F. Silva^1,2^, I. Lua^1,2^, G.S. Jesus^1,3^, N.S. Guimarães^1^, G.A.S. Morais^1^, R.V.R. Anderle^1^, J. Pescarini^2^, D.B. Machado^2,4^, C.A.S.T. Santos^2^, M.Y. Ichihara^2^, M.L. Barreto^2,1^, L. Magno^1,5^, L.E. Souza^1^, J. Macinko^6^, I. Dourado^1^, D. Rasella ^1,2,7^, DSAIDS

^1^Collective Health Institute, Federal University of Bahia (UFBA), Salvador, Brazil, ^2^Center for Data and Knowledge Integration for Health (CIDACS) Gonçalo Moniz Institute, Oswaldo Cruz Foundation (FIOCRUZ), Salvador, Brazil, ^3^Faculty of Medicine, Federal University of Bahia (UFBA), Salvador, Brazil, ^4^Department of Global Health and Social Medicine, Harvard Medical School Boston, Boston, United States, ^5^Department of Life Sciences, State University of Bahia (UNEB), Salvador, Brazil, ^6^Departments of Health Policy and Management and Community Health Sciences, UCLA Fielding School of Public Health, Los Angeles, United States, ^7^ISGlobal, Hospital Clinic ‐ Universitat de Barcelona, Barcelona, Spain

**Background**: Poverty is a risk factor for HIV/AIDS but previous studies on the impact of conditional cash transfer programs (CCT) have shown inconsistent results. We evaluated the effects of one of the world's largest CCTs, the *Programa Bolsa Família* (PBF), on all sequential AIDS outcomes, using data from a nationwide cohort of the poorest Brazilian people on the Unified Registry for Social Programs (*Cadastro Único*).

**Methods**: We analyzed a cohort of 22.7 million low‐income Brazilian people for the period between 2007 and 2015, comparing PBF beneficiaries and non‐beneficiaries, using a quasi‐experimental impact evaluation design. We used inverse probability of treatment weighting (IPTW) to adjust for selection into receipt of BFP benefits and then fitted multivariable Poisson regressions, adjusted for all relevant socioeconomic and demographic confounding variables, to estimate the effect of PBF on AIDS incidence, mortality, and case‐fatality rates. We also performed subgroup analyses.

**Results**: Exposure to PBF was associated with a lower incidence of AIDS (RR: 0.59; 95% CI: 0.57‐0.61), mortality (RR: 0.61; 95% CI: 0.57‐0.64) and case‐fatality rates (RR: 0.75; 95% CI: 0.66‐0.85). PBF associations were significantly stronger among individuals living in extreme poverty, in comparison with those experiencing poverty (RR0.53 versus RR0.84 for incidence; RR0.54 versus RR0.90 for mortality, and RR0.72 versus RR1.00 for case‐fatality). PBF impact was also stronger among females and adolescents.

**Conclusions**: Conditional cash transfers could significantly reduce AIDS morbidity and mortality, especially in extremely poor populations. During the current dramatic rise in global poverty, due to the COVID‐19 pandemic, CCT investments could protect against potential increases in the HIV/AIDS burden, and contribute towards achieving AIDS‐related Sustainable Development Goals (SDGs).

### OAE0303 Cost‐effectiveness of broadly neutralizing antibodies for HIV prophylaxis for all infants born in high‐burden settings

C. Alba ^1^, S. Malhotra^2^, S. Horsfall^1^, M. Barnhart^3^, K. Chapman^2^, C.K. Cunningham^4,5^, P. Fast^2,6^, G.G. Fouda^7,8^, K.A. Freedberg^1,9,10,11^, L. Ghazaryan^3^, V. Leroy^12^, C. Mann^3^, M.M. McCluskey^3^, E.J. McFarland^13^, V. Muturi‐Kioi^14^, S.R. Permar^15,16^, D. Sok^2^, L. Stranix‐Chibanda^17^, M.C. Weinstein^18^, A.L. Ciaranello^1,9,10^, C.M. Dugdale^1,9,10^

 

**Figure:**
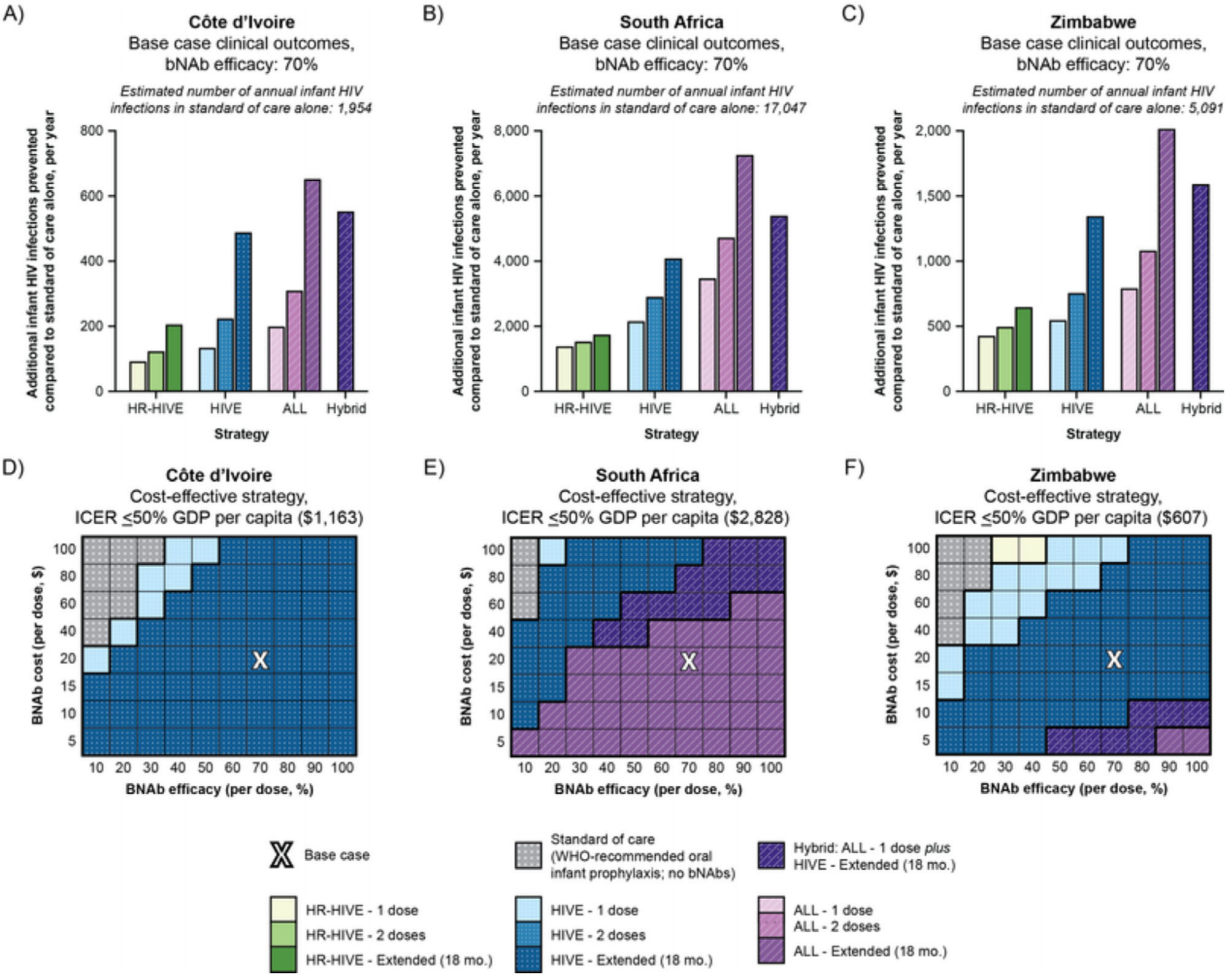
**Abstract OAE0303‐Figure 1**.

^1^Massachusetts General Hospital, Medical Practice Evaluation Center, Boston, United States, ^2^IAVI, Global Headquarters, New York, United States, ^3^U.S. Agency for International Development (USAID), Office of HIV/AIDS, District of Columbia, United States, ^4^University of California Irvine, Department of Pediatrics, Irvine, United States, ^5^Children's Hospital of Orange County, Department of Pediatrics, Orange, United States, ^6^Stanford University School of Medicine, Pediatric Infectious Diseases, Palo Alto, United States, ^7^Duke University Medical Center, Department of Pediatrics, Durham, United States, ^8^Duke Human Vaccine Institute, Durham, United States, ^9^Massachusetts General Hospital, Division of Infectious Diseases, Boston, United States, ^10^Harvard Medical School, Boston, United States, ^11^Massachusetts General Hospital, Division of General Internal Medicine, Boston, United States, ^12^Inserm and Université Toulouse III, Centre d'Epidémiologie et de Recherche en santé des POPulations (CERPOP), Toulouse, France, ^13^Children's Hospital Colorado at University of Colorado Anschutz Medical Campus, Department of Pediatrics, Aurora, United States, ^14^IAVI, Eastern Africa Regional Office, Nairobi, Kenya, ^15^Weill Cornell Medicine, Department of Pediatrics, New York, United States, ^16^New York‐Presbyterian/Weill Cornell Medical Center, Department of Pediatrics, New York, United States, ^17^University of Zimbabwe Faculty of Medicine and Health Sciences, Child and Adolescent Health Unit, Harare, Zimbabwe, ^18^Harvard T.H. Chan School of Public Health, Department of Health Policy and Management, Boston, United States

**Background**: Approximately 150,000 infants acquire HIV annually despite maternal antiretroviral therapy scale‐up. We evaluated the potential clinical impact, cost, and cost‐effectiveness of offering anti‐HIV broadly neutralizing antibody (bNAb) prophylaxis, once clinically approved, to infants in various high‐burden settings.

**Methods**: We simulated birth cohorts in Côte d'Ivoire, South Africa, and Zimbabwe using the Cost‐Effectiveness of Preventing AIDS Complications (CEPAC) model. We modeled strategies offering a three‐bNAb combination–in addition to standard‐of‐care prophylaxis, where WHO‐recommended–to infants: a) with known, WHO‐defined high‐risk HIV exposure at birth (*HR‐HIVE*), b) with known HIV exposure at birth (*HIVE*), or c) regardless of known HIV exposure (*ALL*). Infants received one, two, or extended (every 3 months through 18 months) bNAb doses. We also modeled a strategy offering one birth bNAb dose to all infants plus extended dosing to infants with known exposure. Base‐case model inputs, varied in sensitivity analyses, included bNAb efficacy (70%), efficacy duration/dosing interval (3 months), and cost ($20/dose). Outcomes included infant HIV infections, life expectancy, lifetime HIV‐related costs, and incremental cost‐effectiveness ratios (ICERs, in US$/year‐of‐life‐saved, assuming a <50% GDP per capita cost‐effectiveness threshold).

**Results**: Under base‐case assumptions, *HIVE* and *ALL* strategies would prevent 6‐42% of infant HIV infections across settings (Figure 1A‐C). Extended bNAbs for at least all known HIV‐exposed infants would be cost‐effective in all settings (Figure 1D‐F). *HR‐HIVE* strategies would result in greater lifetime costs and smaller life expectancy gains than *HIVE* strategies. At various bNAb costs and efficacies, *HIVE* strategies would be cost‐effective in Côte d'Ivoire and Zimbabwe, and *ALL* strategies would be cost‐effective in South Africa, partially driven by relatively higher maternal HIV prevalence (Figure 1D‐F).

**Conclusions**: Adding long‐acting bNAbs to current maternal‐infant prophylaxis would be cost‐effective over plausible cost and efficacy ranges, with the cost‐effective target population varying by setting. Infant bNAb prophylaxis development and implementation should be prioritized in high‐burden settings.

### OAE0304 The relative cost‐effectiveness of long‐acting injectable cabotegravir versus oral pre‐exposure prophylaxis: a modelled economic evaluation and threshold analysis in South Africa based on the HPTN 083 and 084 trials

L. Jamieson^1,2^, L.F. Johnson^3^, B.E. Nichols^4,2,1^, S. Delany‐Moretlwe^5^, M.C. Hosseinipour^6,7^, C. Russell^4,2^, G. Meyer‐Rath ^1,4^

^1^University of Witwatersrand, Health Economics and Epidemiology Research Office, Johannesburg, South Africa, ^2^Amsterdam University Medical Centre, Department of Medical Microbiology, Amsterdam, Netherlands, the, ^3^University of Cape Town, Centre of Infectious Disease Epidemiology and Research (CIDER), Cape Town, South Africa, ^4^Boston University School of Public Health, Department of Global Health, Boston, United States, ^5^Wits RHI, Faculty of Health Sciences, University of the Witwatersrand, Johannesburg, South Africa, ^6^University North Carolina, Chapel Hill, United States, ^7^UNC Project, Lilongwe, Malawi

**Background**: Long‐acting cabotegravir (CAB‐LA), administered 2‐monthly, is more effective at preventing HIV infection than daily oral tenofovir (TDF)/emtricitabine (FTC), but its cost‐effectiveness in a high‐prevalence setting is not known. We estimated the cost‐effectiveness of CAB‐LA compared to TDF/FTC in South Africa and determined the threshold price at which CAB‐LA is as cost‐effective as TDF/FTC.

**Methods**: We used deterministic HIV transmission modelling, evaluating the impact of CAB‐LA provision compared to scaling up standard‐of‐care, TDF/FTC, to adolescent girls, young women, female sex workers, adolescent boys, young men, and men who have sex with men. We estimated the average cost by population using ingredients‐based costing (costs in 2021 USD). We model the cost‐effectiveness over 2022‐2041, assuming two coverage scenarios (medium, high), assuming higher uptake of CAB‐LA compared to TDF/FTC throughout based on preference studies. Under CAB‐LA we modelled two scenarios defined by average duration of use (minimum: same duration as TDF/FTC of 5‐11 months; maximum: longer duration than TDF/FTC, 12‐24 months). We compare scenarios to the current baseline of low TDF/FTC roll‐out.

**Results**: Across CAB‐LA scenarios, 15%‐28% of new HIV infections were averted over baseline compared to 5%‐8% in oral TDF/FTC scale‐up scenarios (Table 1). Assuming equivalent drug costs between CAB‐LA and TDF/FTC, the incremental cost of CAB‐LA to the HIV programme was higher than TDF/FTC (5%‐14% vs 2%‐4%) due to higher assumed uptake of CAB‐LA. The cost per infection averted was $4,553‐$6,803 (CAB‐LA) and $6,053‐$6,610 (TDF/FTC). The cost per CAB‐LA injection needed to be less than twice that of a 2‐month supply of TDF/FTC to remain as cost‐effective, with threshold prices ranging between $8.99/injection (high coverage; maximum duration) and $14.21/injection (medium coverage; minimum duration).

**Figure:**
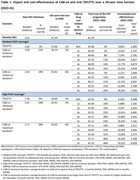
**Abstract OAE0304‐Table 1**.

**Conclusions**: CAB‐LA is potentially game‐changing for HIV prevention. However, for its implementation to be financially feasible across low‐ and middle‐income countries with high HIV incidence, CAB‐LA must be reasonably priced.

### OAE0305 Variation in average unit prices (2020) of antiretroviral drugs in generic accessible low‐ and middle‐income countries

D. Mattur ^1^, V. Habiyambere^2^

^1^UNAIDS, Geneva, Switzerland, 2WHO(retired), Geneva, Switzerland

**Background**: By end 2020, 37.7 million people were living with HIV and 27.5 million people were accessing antiretroviral therapy (ART). Price transparency of anti‐retroviral drugs (ARVs) will be key to optimize procurement and support in sustainable financing of HIV commodities.

**Methods**: Data on average ARV unit prices were extracted from reports to UNAIDS (Global AIDS Monitoring) and government customs to estimate the price trends in 2020 across 1^st^ line and 2^nd^ line ARV regimens & variations for ARVs in generic accessible low‐and‐middle‐income countries (LMICs).

Estimates for person‐years on treatment and unit price per person‐year (ppy) were calculated from the volumes, expected dosage frequency for each regimen and price of ARV exports in LMICs. Analyses were stratified by year, regimen, income group and region.

**Results**: In 2020, the average unit price ppy of 1st line ARVs in LMICs was US$ 95. It was US$ 77 in low‐income countries, US$ 92 in lower middle‐income countries and US$ 117 in upper middle‐income countries. The 1st line ART regimen was estimated to be most expensive in Eastern Europe and Central Asia with an average price of US$116 ppy. The West and Central Africa region, with an average price for 1st line ART of US$ 76 was the least expensive followed by US$82 in East and Southern Africa and US$83 in the Caribbean.

Overall, average unit price ppy of the second line ARV regimen in LMICs was US$256, 2.7 times more expensive than the 1st line ARV regimens.

The most expensive 2nd line ART regimen was Abacavir+Lamivudine+Raltegravir (US$ 747).The least expensive 2nd line ARV regimen was Zidovudine+Lamivudine+Dolutegravir with an average unit cost ppy of US$102.

**Conclusions**: There are large variations in average procurement prices across regions and income groups of countries. In the current environment of flat lined international resources for HIV, countries must look for options to optimize their procurement cost and thereby reallocate the resources saved through supply chain optimization to other programme needs.

### OAE0402 Is local production of HIV commodities a feasible strategy for improving domestic ownership and financial sustainability of HIV epidemiological control in low‐ and middle‐income settings?

F. Ilika ^1^, U. Ihekanandu^1^, J. Nwizu^1^, C. Ohazurike^1^

^1^Palladium, Health Policy Plus, Abuja, Nigeria

**Background**: Nigeria has about 1.9 million people living with HIV, with 1,228,100 on antiretroviral therapy in June 2020. Approximately US$126 million was invested in HIV commodity expenditure in 2018 with more than 81% from donors, while public and private funds accounted for 18% and 1%, respectively. Recently, HIV donor funding has decreased—from 92.3% in 2008 to 82.8% in 2018. Given this decline, the government is making efforts toward a more sustainable HIV financing approach. Local manufacture of HIV drugs was proposed as one of the sustainable strategies for mobilizing domestic resources for HIV in Nigeria's National Domestic Resource Mobilization and Sustainability Strategy for HIV (2021–2025).

**Methods**: A two‐pronged approach was adopted to explore the feasibility of this strategy: (1) key informant interviews with multiple stakeholders, including national and state governments, pharmaceutical manufacturers and associations, biomedical companies, donors, and partner organizations, and (2) a desk review of available national reports, peer‐reviewed and gray literature. This obtained understanding of the requirements, risks, and benefits of local production of ARVs in Nigeria.[Table jia225935-tbl-0005]

**Results**: Local production of HIV commodities is a viable and sustainable strategy that can increase ARV access, improve efficiency, contribute to economic growth, and reduce dependence on external sources, however some challenges must be overcome. These include difficulty with obtaining prequalification approvals from the World Health Organization (WHO) and U.S. Food and Drug Administration (FDA); lack of guaranteed market for products, high import duties and taxes, financial constraints, and insufficient government commitment.Recommendations to overcome barriers include enabling policies like direct fiscal and non‐fiscal incentives by the government to local manufacturers, review of heavy tax burden, advance purchase commitment by the government, supporting pharmaceutical industries to attain prequalification, strategic joint venture with international pharmaceutical companies and multisectoral collaboration.

**Conclusions**: Local production of HIV commodities can be a viable and sustainable strategy for enhancing country ownership of HIV response. Local manufacturing companies are willing to take on this venture, if needed support and commitment are obtained. HIV actors in low‐and middle‐income settings can explore mechanisms of creating an enabling regulatory and policy environment with guaranteed buyer commitments through multisectoral partnerships.

### OAE0403 Quantifying the health and economic impact of voluntary licensing of HIV medicines in low‐ and middle‐income countries: putting numbers on additional uptake, deaths averted, and money saved by MPP licences

S. Morin ^1^, M. Das^2^, O. Bubb‐Humfryes^3^, C. von Drehle^3^, E. Burrone^1^

^1^Medicines Patent Pool, Geneva, Switzerland, ^2^Medicines Patent Pool, Mumbai, India, ^3^Cambridge Economic Policy Associates, London, United Kingdom

**Background**: Public‐health licences enable the development, manufacturing, and uptake of generic versions of patented medicines in low‐ and middle‐income countries (LMICs) before patent expiry. Generic competition reduces prices and supports accelerated uptake of optimal treatment regimens, leading to positive health outcomes. However, quantifying this impact has seldom been done. Here, we build on previous work and present a thorough modelling study quantifying the health and economic impact in LMICs of Medicines Patent Pool (MPP) voluntary licences for multiple HIV medicines.

**Methods**: Building on a rigorous, evidence‐based methodology previously applied to MPP licences for dolutegravir (DTG) and daclatasvir (Morin et al., The Lancet Public Health, 2021), we present an adaptation of the model, now applied to other MPP licences for HIV medicines and with updated results on DTG reflecting the most recent uptake data. Results for atazanavir (ATV), DTG (now also for paediatric use), lopinavir/ritonavir (LPV/r), and tenofovir disoproxil fumarate (TDF), together representing > 95% of MPP impact so far, are presented.

**Results**: By the end of 2020, MPP licences for HIV medicines ATV, DTG, LPV/r, and TDF are modelled to have led to an additional uptake of 1.0 [0.86 – 1.7] million patient‐years treated, 830 [470 – 1,500] million USD saved, 7,500 [1,800 – 16,000] deaths averted, 63,000 [15,000 – 150,000] DALYs averted and 79,000 [17,000 – 190,000] virological failures averted. Results for each licence, including projections until 2030, are presented in Table 1.

**Abstract OAE0403‐Table 1 jia225935-tbl-0005:** Disaggregated impact results – achieved by 2020 and projected until 2030 – for MPP licences for ATV, DTG, LPV/r, and TDF

	**Additional uptake (patient‐years treated)**	**Costs saved (million USD)**	**Deaths averted**	**DALYs averted**	**Virological failures averted**
**ATV**	* 240,000 by 2020 * 330,000 by 2030	* 67 by 2020 * 76 by 2030	* 300 by 2020 * 410 by 2030	* 7,900 by 2020 * 11,000 by 2030	* 9,600 by 2020 * 13,000 by 2030
**DTG** **(adult use)**	* 710,000 by 2020 * 16,000,000 by 2030	* 280 by 2020 * 2,800 by 2030	* 7,000 by 2020 * 160,000 by 2030	* 50,000 by 2020 * 1,100,000 by 2030	* 64,000 by 2020 * 1,500,000 by 2030
**DTG** **(paediatrics)**	* No uptake as of 2020 * 42,000 by 2030	* No uptake as of 2020 * 61 by 2030	* No uptake as of 2020 * 180 by 2030	* No uptake as of 2020 * 4,700 by 2030	* No uptake as of 2020 * 5,700 by 2030
**LPV/r** **(adult use)**	* 0 by 2020 * 0 by 2030	* 89 by 2020 * 89 by 2030	* Not applicable * Not applicable	* Not applicable * Not applicable	* Not applicable * Not applicable
**LPV/r** **(paediatrics)**	* 36,000 by 2020 * 36,000 by 2030	* 7 by 2020 * 7 by 2030	* 92 by 2020 * 92 by 2030	* 2,400 by 2020 * 2,400 by 2030	* 2,900 by 2020 * 2,900 by 2030
**TDF**	* 49,000 by 2020 * 49,000 by 2030	* 380 by 2020 * 380 by 2030	* 87 by 2020 * 87 by 2030	* 2,300 by 2020 * 2,300 by 2030	* 2,800 by 2020 * 2,800 by 2030
**Total for MPP licences for HIV medicines**	* **1,000,000 by 2020** * 17,000,000 by 2030	* **830 by 2020** * 3,400 by 2030	* **7,500 by 2020** * 160,000 by 2030	* **63,000 by 2020** * 1,200,000 by 2030	* **79,000 by 2020** * 1,500,000 by 2030

**Conclusions**: Modelling the impact of MPP licences for HIV medicines offers visibility on the overall contribution to the global HIV response of the MPP model of public‐health oriented voluntary licensing and a means to assess the investment (additional expenditure) that would be needed for the same level of optimal drug uptake without MPP licences: 22 billion USD in cumulative theoretical expenditures avoided by 2030.

### OAE0404 Estimated costs to address unmet financial needs for HIV pre‐exposure prophylaxis, United States, 2018

R. Bonacci ^1^, M. Van Handel^2^, R. Huggins^1^, D. Smith^1^

^1^U.S. Centers for Disease Control and Prevention, Division of HIV Prevention, Atlanta, United States, ^2^U.S. Centers for Disease Control and Prevention, National Center for HIV, Viral Hepatitis, STD, and TB Prevention, Atlanta, United States

**Background**: An estimated 1.2 million persons in the United States (US) had indications for pre‐exposure prophylaxis (PrEP) in 2018. The cost of PrEP medications and care are frequently cited as barriers to increased PrEP uptake. We sought to understand the burden of financial needs for PrEP care and the cost to address them.

**Methods**: Using population‐based surveys and published information, we modeled the number of persons who had unmet financial needs for PrEP care among US adults with PrEP indications, stratified by transmission risk group (men who have sex with men [MSM], heterosexual males [HET males] and females [HET females], and persons who inject drugs [PWID]), insurance status, and income level. We estimated the annual cost to address unmet financial needs for PrEP medication, clinical visits, and lab testing based on the 2021 PrEP clinical practice guideline.

**Results**: Of 1.2 million US adults with PrEP indications, we estimated that 64% had private insurance, 21% had public insurance, and 15% were uninsured. In total, 49,860 (4%) persons required PrEP financial assistance, including 32,350 MSM, 7,600 HET females, 5,070 HET males, and 4,840 PWID (Table). Of those, 3,160 required assistance for PrEP medications ($16.8 million), clinical visits ($2.1 million), and lab testing ($3.5 million) at a cost of $22.4 million (Table); 46,700 required assistance only for clinical visits ($31.3 million) and lab testing ($52.2 million) at a cost of $83.5 million. The total annual cost to address unmet financial needs for adults with PrEP indications was $106.0 million.

**Table:** 

**Abstract OAE0404‐Table 1**.						
		MSM	HET Females	HET Males	PWID	Total
Persons needing financial assistance for medication, clinical visits, labs^1^(n)		2,020	460	680	0	3,160
	PrEP medication, cost per person^2^	$5,324	$5,324	$5,324	$5,324	$16,822,660
	Clinical visits, cost per person^3^	$671	$671	$671	$671	$2,120,390
	Lab testing, cost per person^3^	$1,458	$478	$478	$506	$3,490,150
	Total, cost per person	$7,453	$6,472	$6,472	$6,501	$22,433,200
Persons needing financial assistance for clinical visits and labs only^4^(n)		30,330	7,140	4,390	4,840	46,700
	Clinical visits, cost per person^3^	$671	$671	$671	$671	$31,336,170
	Lab testing, cost per person^3^	$1,458	$478	$478	$506	$52,185,520
	Total, cost per person	$2,129	$1,149	$1,149	$1,177	$83,521,690
Grand Total						$105,954,890

Table. Estimated cost for PrEP medication, clinical visits, and labs for adults with unmet financial needs, by HIV transmission risk group — United States, 2018

Abbreviations: HET, heterosexual; MSM, men who have sex with men; PrEP, pre‐exposure prophylaxis; PWID, persons who inject drugs

^1^Persons with income ≥500% of the federal poverty line (FPL) were ineligible for medication assistance programs and needed assistance for PrEP medication, clinical visits, and lab testing

^2^Medication costs calculated as weighted average of 340B price for Truvada, Descovy, and generic tenofovir disoproxil fumarate/emtricitabine

^3^Costs obtained from Centers for Medicare & Medicaid Services

^4^Persons with income <500% FPL were eligible for medication assistance programs and needed assistance for PrEP clinical visits and lab testing only

**Conclusions**: The number of persons with unmet financial needs for PrEP is <5% among adults with PrEP indications, but costs to meet those needs are significant. Population size estimates and costs to address unmet needs can inform policy makers about resources needed to overcome financial barriers for PrEP.

### OAE0406 The economic returns of achieving the 2021‐2030 AIDS targets to end the AIDS epidemic by 2030

E. Lamontagne ^1,2^, M. Over^3^, J. Stover^4^, A. Yakusik^1^

^1^UNAIDS, Strategic Information, Geneva, Switzerland, ^2^Aix‐Marseille University, School of Economics, Marseille, France, ^3^Center for Global Development, Washington, DC, United States, ^4^Avenir Health, Glastonbury, United States

**Background**: In 2019, several countries have achieved or were on track to end AIDS. Despite progress towards that goal, AIDS remains a global crisis. The gains achieved are still fragile in many countries. In June 2021, the General Assembly of the United Nations adopted the Political Declaration on HIV and AIDS: Ending Inequalities and Getting on Track to End AIDS by 2030. We estimated the benefits and costs of this ambitious commitment.

**Methods**: We estimated the incremental costs benefits and economic returns of a scenario which fulfils the AIDS targets stated in the Political Declaration, compared to a counterfactual scenario defined as maintaining coverage of HIV‐related services at 2020 levels. The benefits are calculated using the full‐income approach, which values both the change in income and in mortality. We value both the health gain and the intervention cost to each HIV‐affected country from the perspective of that country, converting national benefits and costs to purchasing‐parity‐equivalent (PPP) 2019 US dollars. We estimated the value of the projected reduction in the mortality rate of the HIV programmes as the amount an average person would pay to reduce their risk of death by one in 10,000 for one year. From the literature, this amount varies between 1.0 and 1.6% of GDP per capita. We allowed the income‐elasticity of the willingness to pay for mortality risk reduction to decline at either 1% or 1.5% for every percentage decline of the country's income.

**Results**: Using the full‐income approach, we found that each additional dollar invested between 2021 and 2030 generates US$ 7.37 [4.52‐11.79] in economic returns. Returns on investment vary substantially between countries, regions, and income categories. Returns are higher in countries with higher purchasing power as measured by their GDP per capita. The benefits of investment are also highly correlated with the total number of adults living with HIV.

**Conclusions**: Using the latest scientific evidence in terms of benefit‐cost analysis, it appears that investing to achieve the 2025 targets in the UNAIDS Strategy and the 2030 target in the Agenda for Sustainable Development provides significant returns from both human and economic perspectives.

### OAE0502 Screening and treatment of common mental disorders at HIV clinics within the International epidemiology Databases to Evaluate AIDS (IeDEA) consortium

A. Parcesepe ^1^, M. Remch^2^, M. Stockton^3^, C. William Wester^4^, C. Bernard^5^, L.A. Enane^6^, A. Haas^7^, J. Ross^8^, R. Ajeh^9^, K. Althoff^10^, W. Pape^11^, A. Minga^12^, E. Kwobah^13^, M. Tlali^14^, J. Tanuma^15^, D. Nsonde^16^, A. Freeman^10^, D. Nash^17^, K. Lancaster^18^, IeDEA Consortium

**Table:** 

**Abstract OAE0502‐Table 1**.				
	All n=223 n (%)	Low‐income countries n=51 n (%)	Middle‐income countries n=121 n (%)	High‐income countries n=51 n (%)
**Depression** Screening	112 (50)	27 (53)	48 (40)	37 (73)
Screening and counseling	102 (46)	24 (47)	44 (36)	34 (67)
Screening and psychiatric medication	80 (36)	18 (36)	27 (22)	35 (69)
**PTSD** Screening	27 (12)	3 (6)	14 (12)	10 (20)
Screening and counseling	24 (11)	3 (6)	12 (10)	9 (18)
Screening and psychiatric medication	17 (8)	2 (4)	6 (5)	9 (18)
**Anxiety** Screening	32 (14)	2 (4)	17 (14)	13 (25)
Screening and counseling	29 (13)	2 (4)	16 (13)	11 (22)
Screening and psychiatric medication	24 (11)	2 (4)	10 (8)	12 (24)

^1^University of North Carolina at Chapel Hill, Department of Maternal and Child Health, Chapel Hill, United States, ^2^University of North Carolina at Chapel Hill, Department of Epidemiology, Chapel Hill, United States, ^3^Columbia University, Department of Psychiatry, New York, United States, ^4^Vanderbilt University Medical Center, Department of Medicine, Nashville, United States, ^5^University of Bordeaux, National Institute for Health and Medical Research, Research Institute for Sustainable Development, Bordeaux Population Health Research Centre, Bordeaux, France, ^6^Indiana University School of Medicine, The Ryan White Center for Pediatric Infectious Disease and Global Health, Department of Pediatrics, Indianapolis, United States, ^7^University of Bern, Institute of Social and Preventive Medicine, Bern, Switzerland, ^8^TREAT Asia/amfAR, The Foundation for AIDS Research, Bangkok, Thailand, ^9^Clinical Research Education and Networking Consultancy, Yaounde, Cameroon, ^10^Johns Hopkins University, Bloomberg School of Public Health, Baltimore, United States, ^11^Groupe Haitien d'Etude du Sarcome de Kaposi et des Infections Opportunistes (GHESKIO), Port au Prince, Haiti, ^12^Centre Medical de Suivi de Donneurs de Sang/CNTS/PRIMO‐CI, Abidjan, Cote D'Ivoire, ^13^Moi Teaching and Referral Hospital, Department of Mental Health, Eldoret, Kenya, ^14^University of Cape Town, Centre for Infectious Disease Epidemiology & Research (CIDER), School of Public Health & Family Medicine, Cape Town, South Africa, ^15^National Center for Global Health and Medicine, Division of the AIDS Medical Information of AIDS Clinical Care, Tokyo, Japan, ^16^CTA Brazzaville, Brazzaville, Congo, the, ^17^City University of New York, Institute for Implementation Science in Population health, New York, United States, ^18^The Ohio State University, Department of Epidemiology, Columbus, United States

**Background**: Common mental disorders (CMDs), including depression, anxiety, and post‐traumatic stress disorder (PTSD) are highly prevalent among people living with HIV and associated with poor HIV treatment outcomes. Integrating screening and treatment of CMDs into HIV care can improve mental health, HIV treatment outcomes, and quality of life. Data regarding the availability of mental health screening and treatment at HIV clinics remain scarce.

**Methods**: We describe the reported availability of mental health screening and treatment from a survey of HIV treatment sites in Africa, the Asia‐Pacific, the Caribbean, Central and South America, and North America regions participating in the International epidemiology Databases to Evaluate AIDS (IeDEA) consortium. The survey captured information on site characteristics and reported availability of screening and treatment for depression, anxiety, and PTSD in 2020.

**Results**: Among the 223 HIV treatment sites that completed the mental heath portion of the survey, 67% were located in urban settings, 50% served adult and pediatric populations, and 38% served only adults. Most sites (78%) were located in low‐ or middle‐income countries (LMIC). Overall, 50%, 14%, and 12% of sites reported using a validated instrument to screen for depression, anxiety, and PTSD, respectively. Screening and counseling/psychotherapy were available for treatment of depression, anxiety, and PTSD at 46%, 13%, and 11% of sites, respectively. Screening and psychiatric medication was available for the treatment of depression, anxiety, and PTSD at 36%, 11%, and 8% of clinics, respectively. Screening and treatment for all disorders assessed was more commonly reported at urban compared to rural sites and at sites in high‐income countries compared to sites in LMIC.

**Conclusions**: Substantial gaps exist in the availability of mental health services at HIV treatment sites, particularly in rural and LMIC settings. Identification of barriers and implementation of feasible and sustainable strategies to integrate mental health services into HIV care is needed.

### OAE0503 Using the RE‐AIM framework to evaluate the implementation and effectiveness of a WHO HEARTS based implementation strategy to integrate the management of hypertension into HIV care in Uganda

M. Muddu ^1^, F.C. Semitala^1^, I. Kimera^1^, M. Mbuliro^1^, R. Ssenyonjo^1^, S. Kigozi^2^, R. Katwesigye^1^, F. Ayebare^2^, C. Namugenyi^1^, F. Mugabe^3^, G. Mutungi^3^, C. Longenecker^4^, A. Katahoire^5^, J. Schwartz^6^, I. Ssinabulya^7^

^1^Makerere University Joint AIDS Program (MJAP), Clinical Servcies, Kampala, Uganda, ^2^Infectious Diseases Research Collaboration (IDRC), Kampala, Uganda, ^3^Uganda Ministry of Health, NCDs, Kampala, Uganda, ^4^University of Washington, Washington, United States, ^5^Makerere University College of Health Sciences, Kampala, Uganda, ^6^Yale School of Medicine, Medicine, New Haven, United States, ^7^Uganda Heart Institute, Kampala, Uganda

**Background**: World Health Organization (WHO) HEARTS packages are increasingly used to control hypertension. However, their feasibility for hypertension control in persons living with HIV (PLHIV) is unknown. We studied the effectiveness and implementation of a WHO HEARTS based implementation strategy to integrate the management of hypertension into HIV care.

**Methods**: This was a mixed methods study at Uganda's largest HIV clinic. Components of the implementation strategy were: lifestyle counseling, free hypertension medications, hypertension treatment protocol, task shifting and monitoring tools. We used a pre‐post study to determine effectiveness of the implementation strategy on hypertension and HIV outcomes among PLHIV over 21 months. The RE‐AIM framework evaluated implementation of the strategy from stakeholders’ perspectives. We conducted four focus group discussions with PLHIV (n=42), in‐depth interviews with PLHIV (n=9), health care providers (n=15) and Ministry of Health (MoH) policy makers (n=2).

**Results**: Reach: WHO HEARTS based integrated hypertension‐HIV care was acceptable as all 48 (100%) healthcare providers in the clinic were trained. All 15,953 (100%) adult PLHIV were screened and 3,892 (24.4%) diagnosed with hypertension. Of these, 1,084 (28%) initiated HTN treatment compared to 39(1%) at baseline. Among enrolled patients, mean age was 51.5±9.7 years and 679(62.6%) were female. Effectiveness: Among patients treated, controlled hypertension improved from 5% to 75% (p<0.0001), mean systolic BP 152.9 ± 0.7 to 130.2 ± 0.9 mmHg (p<0.0001) and mean diastolic BP 96.7 ± 0.5 to 83.7 ± 0.6mmHg (p<0.0001). Overall, 1098(95.6%) of patients were retained by month 21. HIV viral suppression remained high, 97% to 100% (p=0.063). Patients who received integrated hypertension‐HIV care felt healthy and saved more money. Adoption: Training healthcare providers on WHO HEARTS, task shifting, synchronizing clinic appointments for hypertension and HIV and relative advantage promoted adoption. Implementation:WHO HEARTS strategy was feasible and implemented with fidelity.Maintenance: Leveraging HIV program resources, adopting the WHO HEARTS protocol by MoH and integrating it into national guidelines will promote sustainability.

**Conclusions**: WHO HEARTS implementation strategy promoted integration of hypertension management into HIV care. It was acceptable, feasible and effective in controlling hypertension and maintaining optimal viral suppression among PLHIV. Integrating this strategy into national guidelines will sustain the implementation.

### OAE0504 Successful transition to tenofovir/lamivudine/dolutegravir (TLD) in PEPFAR‐supported countries

J. da Silva ^1^, S. Vallabhaneni^1^, R. Pati^1^, H. Chun^1^, C. Malati^2^, D. Kiesa^2^, J. Rosenfield^2^, M. Belayneh^2^, K. Fisher^1,1^, G. Siberry^2^, E. Raizes^1^, C. Godfrey^3^

^1^Centers for Diseases Control and Prevention, Division of Global HIV and Tuberculosis, Atlanta, United States, ^2^United States Agency for International Development, Washington, DC, United States, ^3^Office of Global AIDS Coordination, Washington, DC, United States

**Background**: PEPFAR begun supporting the transition to tenofovir/lamivudine/dolutegravir (TLD) for people living with HIV (PLHIV) in 2017. In 2018, the possible association of maternal DTG use and offspring neural tube defects delayed uptake of TLD. In 2019, based on reassuring additional data, PEPFAR renewed efforts to transition several million supported clients to TLD. We describe the lessons learned and the results of a massive multisectoral effort to provide optimized treatment to all PLHIV.

**Description**: From 2017 to 2021,PEPFAR promoted policies and programmatic interventions among its 35 supported countries to increase the uptake of TLD. Key elements were the removal of programmatic barriers such as pre‐transition viral load (VL) test and the streamlining of technical guidance by recommending TLD as preferred regimen for naïve, suppressed, non‐suppressed and individuals with unknown VL status, and to male and female adults, adolescents and eligible children, thus simplifying supply chain and implementation at site level.

**Lessons learned**: Based on routine PEPFAR programmatic data on antiretroviral dispensation, TLD made up 41% of drugs dispensed to adults in PEPFAR‐supported countries in April 2020, this proportion doubled to 81% by October 2021.For all but one PEPFAR‐supported country with >1 million PLHIV, TLD accounted for at least 80% of antiretrovirals dispensed. Viral suppression has increased in all age bands since TLD rollout begun, but remains lowest among children.

**Conclusions/Next steps**: Roll out of DTG‐based regimens to children should be prioritized with the recent pediatric formulations approval. Strategies to improve adherence among PLHIV non‐suppressed on TLD may be needed to achieve the 3rd 95 UNAIDS target.

**Figure:**
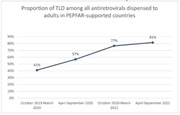
**Abstract OAE0504‐Figure 1**.

**Figure:**
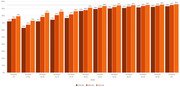
**Abstract OAE0504‐Figure 2**.

### OAE0505 Integration of hepatitis B and C testing into HIV services: an opportunity to achieve dual elimination of viral hepatitis and HIV in Vietnam

B. Vu Ngoc ^1^, K. Do Tuan^1^, A. Tran Khanh^1^, L. Tran Khanh^1^, K. Green^1^, K. Nguyen Trong^2^, P. Cao Duc^2^, N. Ngo Thuy^1^, L. Tran Thi Huong^1^, K. Do Quang^3^, P. Nguyen Anh^4^, T. Le Van^5^

^1^PATH, SE Asia, Hanoi, Viet Nam, ^2^Vietnam Administration of Medical Services, Ministry of Health, Hanoi, Viet Nam, ^3^Galant Clinic, Ho Chi Minh City, Viet Nam, ^4^My Home Clinic, Ho Chi Minh City, Viet Nam, ^5^Glink Clinic, Ho Chi Minh City, Viet Nam

**Background**: Testing is the gateway for access to hepatitis prevention and treatment services, but there remains a large burden of undiagnosed hepatitis B (HBV) and C (HCV) infection in Vietnam. We implemented HepLINK initiative to demonstrate integration of HBV/HCV testing into HIV services to accelerate access to viral hepatitis treatment among key populations (KPs) in Hanoi and Ho Chi Minh City.

**Description**: We engaged 9 KP‐led community‐based organizations (CBOs) and 18 clinics (12 public; 6 private) in providing community‐based testing (CBT) and facility‐based testing (FBT) and linkage to HBV/HCV care and treatment. Clients seeking HIV testing, pre‐exposure prophylaxis (PrEP), antiretroviral therapy (ART), and methadone maintenance treatment (MMT) were offered HBV/HCV screening using a single blood‐based rapid diagnostic test. Those with a reactive result were linked to confirmatory testing and treatment.

**Lessons learned**: From April‐October 2021, we reached 8,840 KP, of which 86.9% accepted HBsAg testing, yielding 689 (9%) positive cases. CBT yielded a higher HBsAg positivity rate than FBT (11.5% and 4.9%, respectively), whereas FBT had a higher rate of enrollment on treatment than CBT (90% and 62.1%, respectively). HBsAg+ infection rate was highest in female sex workers‐FSW (20.8%), followed by people injecting drugs‐PWID (11.1%), drug users‐DU (9.5%), men having sex with men‐MSM (5.9%) and transgender women‐TGW (2.6%). HBV‐HIV co‐infection rate was 9.6%. Of 689 people with HBsAg+, 45.4% received evaluation for treatment eligibility, and 69.9% of those eligible enrolled on HBV treatment or TDF containing ART or PrEP.

Of 8,840 KP offered testing, 94% accepted anti‐HCV testing, yielding 941 (11.3%) positive cases. CBT reached and tested more people, but unlike with HBV, yielded lower positivity rate than FBT (6.7% and 18.4%, respectively). Anti‐HCV positivity rate was highest in PWID (20.9%), followed by DU (6.8%), FSW (3.9%), TGW (2.6%), and MSM (1.9%). HCV‐HIV co‐infection rate was exceptionally high (28.4%). Of 941 people with anti‐HCV+, 55.3% received confirmatory testing, and 38.1% of those confirmed initiated HCV treatment.

**Conclusions/Next steps**: Community‐based and HIV integrated HBV/HCV testing is a promising approach to accelerate HBV/HCV case finding and treatment access among KPs. Further work is needed to improve access to and affordability of HBV/HCV confirmatory testing and treatment.

### OAF0102 Ensuring HIV treatment continuity for Haitian migrants to the Dominican Republic during the COVID‐19 pandemic

E. Emmanuel^1^, G. Guethina ^1^, L. Daniel^1^, L. Jutile^1^, N. Bernadine^1^, M.L. Excellent^1,2^, M. Léonard^1^, J.W. Domerçant^1^

^1^Institut pour la Santé, la Population et le Développement (ISPD), Pétion‐Ville, Haiti, ^2^University of North Carolina at Chapel Hill, Chapel Hill, North Carolina, United States

**Background**: Nearly 75% of People Living with HIV/AIDS (PLHIV) in the Caribbean live either in Haiti or the Dominican Republic (DR). Individuals of Haitian descent represent 36.8% of all PLHIV in DR, the largest priority group. Historically, migration flows from Haiti to DR, except during the deportation campaigns. During the covid‐19 pandemic, the flow was inverted. From 17 March 2020 to 31 July 2021, 711,736 movements were observed from Haiti to DR compared to 921,103 from DR to Haiti ‐ including 337,147 voluntary returns. With an HIV prevalence of 3‐5% among Haitian migrants, these population movements represent a potential challenge for epidemic control in both countries.

**Description**: With USAID funding, BRIDGE project was implemented to increase access to comprehensive HIV services for mobile populations while strengthening program collaboration between Haiti and DR. In collaboration with the Ministry of Health, ISPD implemented two HIV service delivery points at the main entry points (Malpasse, Ouanaminthe) and a coordination committee comprised of organizations intervening on the border. Comprehensive HIV services are provided to deportees, migrants, drivers, police officers, FSW, and their clients working in the border area.

**Lessons learned**: During FY21, 944 individuals were reached (284 deportees and 235 other migrants) of which 434 received assisted HIV Self‐testing and 414 received standard HIV‐testing. Twenty individuals who were newly identified as HIV‐positive (9 males‐11 females) and two who interrupted treatment were enrolled in care. Clinical management is challenging as many patients returned symptomatic (AIDS stage 4) or had no documentation of their treatment history. Haiti and DR have different ART first‐line regimens. Additionally, migrants do not stay near the border, they move to other parts of the country and the deportees plan their returning back to DR right away. Follow‐up and proper tracking of those who received HIV services are further complicated.

**Conclusions/Next steps**: In order to accelerate the process towards the UNAIDS 95‐95‐95 goal, there is a need to build a partnership between Haiti and the Dominican Republic to provide better options to the Haitians crossing the border through improved referrals and establishing accessible ART dispensing points.

### OAF0103 The use of community paralegals to improve the HIV and TB treatment cascade: comparing approaches in Mozambique, Senegal, Ghana, Indonesia, and Kyrgyzstan

J. Amon ^1^, N. Sun^2^, K. Hinman^2^, J. Csete^3^, M. Golichenko^4^, C. Kazatchkine^4^, D. Lohman^5^, M. McLemore^6^, S.K.H. Chu^4^

^1^Drexel University Dornsife School of Public Health, Philadelphia, United States, ^2^Drexel University, Philadelphia, United States, ^3^Columbia University, New York, United States, ^4^HIV Legal Network, Toronto, Canada, ^5^Consultant, New York, United States, ^6^Consultant, Philadelphia, United States

**Background**: The Global Fund's *Breaking Down Barriers*(BDB) initiative provides support in 20 countries to scale‐up to comprehensive levels programs to remove human rights‐related barriers to HIV and tuberculosis (TB) services.

**Description**: A mid‐term evaluation was conducted from 2020‐21. One of the program areas assessed was increasing access to legal services. Within this area, the Global Fund provides support to a range of access to justice activities, including the use of community paralegals.

**Lessons learned**: Community paralegals are community members trained to advocate for the rights of individuals and to liaise with legal professionals when necessary. With the support of the BDB initiative, community paralegals in many countries work to address rights violations that impede access to HIV and TB services. We compared approaches used in five BDB countries during the assessment period (from 2017‐2021). In Mozambique, with Global Fund support, community paralegal programs had expanded significantly during the period assessed, with NGOs training and deploying paralegals in 11 provinces. In Senegal, NGOs have trained 118 sex workers as paralegals. In Ghana, paralegal trainings had been conducted for 88 persons living with HIV, sex workers, MSM and former TB patients. In Indonesia, the 4 Pillars program used paralegals in outreach teams to support key populations. In Kyrgyzstan, “street lawyers”, most of whom are from key populations themselves or have extensive experience working directly with them, expanded, amidst the COVID‐19 pandemic, to provide onlineservices to HIV NGOs. Paralegals in these programs were able to advocate for treatment access, negotiate with police for the release of key population members, conduct anti‐stigma programs with communities, address discrimination in schools and workplaces, and ensure access to medicines in closed settings.

**Conclusions/Next steps**: Community paralegals can address a diverse range of barriers to HIV and TB services, strengthen trust and build relationships between communities and the health system.

### OAF0104 Differentiated service delivery for people with HIV and non‐communicable diseases: South African policy enabler for integration

M. Manganye ^1^, Z. Pinini^2^, L. Seshoka^1^, L. Malala^1^, D. Gavhi^1^, T. Nyawasha^1^, T. Molewa^1^, M. Pilusa^1^, M. Mkhize^1^, M. Kgokolo^1^, T. Dladlama^1^

^1^National Department of Health, Care and Treatment Directorate, Pretoria, South Africa, ^2^National Department of Health, HIV/AIDS & STI Cluster, Pretoria, South Africa

**Background**: Non‐communicable diseases (NCDs), are undoubtedly leading cause of mortality and disabilities in the world accounting for about half of global disease burden. NCDs are increasingly causing health threats for people living with HIV. Whilst South Africa has the largest ART programme worldwide with over 5.5 Million people on ART. The rising burden of NCDs is placing considerable strain and presenting challenges of maintaining high‐quality public health care services. The overall prevalence of hypertension was 14.3% in 2017, while the overall prevalence of diabetes was 3.2% in 2017. As South Africa scale‐up differentiated service delivery (DSD), this optimization presents an opportunity to integrate HIV and management of NCDs into DSD models.

**Description**: DSD is the framework of the Adherence Guidelines (AGL) for HIV, TB and NCDs adopted in March 2020. It aims to strengthen linkage, adherence, and retention in care using a patient‐centered approach throughout the treatment cascade for both HIV, TB, and NCDs patients. It makes provision for the three DMoCs (Facility Pick Up Points (FAC PuP), External Pick‐Up Points (EX‐PuP), and Adherence Clubs (AC) both at facility and community based) for those who are established in ART care and living with hypertension and/or diabetes.

**Lessons learned**: FAC‐PuP model allows for direct and quick access to the pharmacy for healthy and stable clients on treatment. AC is facility and community‐based and allows stable patients to be grouped, voluntarily for routine check‐ups. EX‐PuP model takes various forms, but all involve the patient collecting their treatment supply individually outside of the facility or from an automated system; thus, including from private pharmacies, lockers, etc. As of the end of October 2021, data by DSD and type of patient showed a significant number of clients decanted (2 901 452). EX‐PuP showed a high proportion of 60% (1 486 684) followed by FAC‐PuP 25% (658 671) and lastly, the AC at 15% (500 590).

**Conclusions/Next steps**: DSD policy on HIV and NCDs provides the opportunity to optimize 90% of people on ART including the Integration of NCDs, TB/HIV services into less‐intensive. Conduct the comprehensive review of AGL Policy to inform on options to strengthen the HIV and NCDs integration.

### OAF0105 Legal self‐defense and access to justice for people who use drugs

A. Levin^1^, D. Subeliani ^2^, A. Fedosik, L. Vinc

^1^UnMode, Moscow, Russian Federation, ^2^UnMode, Tbilisi, Georgia

**Background**: In Russia, the equality of all citizens before the law is legally enshrined. However, people who use drugs (PWUD), due to their marginal status, do not have access to high‐quality legal assistance. As a result, in most cases they are deprived of access to a fair trial, replenish the prison population and contribute to the increase in the incidence of HIV in the penitentiary system. In 2021, there were 509 thousand prisoners in Russia. 165 thousand of them were convicted under anti‐drug articles. 53 thousand prisoners live with HIV, which is more than 10% of the total prison population.

**Description**: RuNPUD (Russian‐language Network of People Who Use Drugs) is a regional network that protects PWUD rights, including in Russia. In the current situation RuNPUD directs its efforts to reduce prosecution and protect the rights of PWUD in the country. RuNPUD trains PWUD to be defenders of their rights and interests along with lawyers. RuNPUD teaches these people the basics of legal self‐defense, provides basic knowledge on the current anti‐drug legislation, provides mentoring support in protecting their rights, the rights of their loved ones, the rights of those people who do not yet have the resource to protect themselves on their own. In addition, RuNPUD members themselves act as public defenders in drug‐related criminal cases, which is allowed by Russian law, and are engaged in strategic support of court cases to the European Court of Human Rights (ECHR).

**Lessons learned**: As part of this work, 349 consultations on drug‐related cases and more than 100 representations in courts were held in 2021. This efficiency is due to the fact that Runpad members have stable connections and a high level of trust on the part of PWUD. All members of RuNPUD are successfully socialized people with similar life experiences (imprisonment, criminal prosecution, including for drug use).

**Conclusions/Next steps**: RuNPUD is changing discriminatory practices towards democratic positive changes not through political decisions and changes in the current legislation from above, but through grassroots democracy ‐ the practice of applying this legislation on the ground, by ensuring access to justice for detainees, suspects and accused under anti‐drug articles.

### OAF0202 Trained community peers enhance access to justice, response to violence, discrimination and structural inequities for LGTBiq+ communities in India

R. Lairikyengbam ^1^, M.S. Mudaliar^2^, N. Senapati^3^, T Mahender^4^, S. Yumnam^1^, K. Patnaik^3^, R. Dash^3^, A. Moirangthem^1^, K. Golmei^1^, N. Nampalli^5^, K. Shivakumar^4^, T. Behera^6^, S. Dash^3^, S. Swain^3^, K. Gollapalli^4^, L. Athokpam^1^, J.R. Pasupula^5^, S.C. Mohanty^6^, L. Ramakrishnan^7^, S.S. Raghavan^8^

^1^Solidarity and Action Against The HIV Infection in India (SAATHII), East Imphal, India, ^2^Solidarity and Action Against The HIV Infection in India (SAATHII), Nagpur, India, ^3^Solidarity and Action Against The HIV Infection in India (SAATHII), Bhubaneswar, India, ^4^Solidarity and Action Against The HIV Infection in India (SAATHII), Hyderabad, India, ^5^Modern Architects for Rural India (MARI), Secunderabad, India, ^6^RRDC, Mayurbhanj, India, ^7^Solidarity and Action Against The HIV Infection in India (SAATHII), Chennai, India, ^8^Solidarity and Action Against The HIV Infection in India (SAATHII), New Delhi, India

**Background**: LGBTIQ+ communities in India face multiple human rights violations including violence, discrimination, and exclusion (VDE) due to prevailing cis‐binary‐heteropatriarchal norms across families, institutions, and service providers. Despite the existence of protective laws and policies, low community awareness concerning rights and VDE redressal mechanisms, compounded by the limited sensitivity of law‐enforcement and legal service authorities, collectively impede access to justice.

**Description**: The SAATHII‐led consortium implemented project Sangraha with support from the EU, to reduce VDE and promote access to justice and social protection among LGBTIQ+ communities in 11 districts of Manipur, Odisha, and Telangana states, between 2018 ‐ 2021. The project served 3,679 LGBTQI+ individuals including 53% trans women, 37% cis gay, bi, and other queer men, 7% trans men, 2% cis lesbian, bi, and other queer women, and 1% Intersex individuals and gender non‐conforming children. The project interventions included; a) peer‐led capacity building of community members on current laws on decriminalisation, transgender rights, and domestic violence, b) sensitisation of and advocacy with 5,911 stakeholders on promoting access to LGBTIQ+ inclusive justice, social protection, education, and gender‐affirmation services, c) crisis redressal, and d) facilitating access to social protection, legal and law enforcement services.

**Lessons learned**: Trained LGBTIQ+ peers (one per district) were instrumental in increasing community awareness and successfully advocating with the stakeholders, which helped report 542 incidents of VDE and redressal of 468 of these crises. They facilitated 968 LGBTIQ+ members access social protection services and 628 transgender persons change their name and gender legally. A large proportion (37%) of VDE was found to be perpetrated by the general community, followed by natal family (17%), community peers (17%), and intimate partners (15%). Most crises (78%) were resolved through counseling and mediation by the peer teams and community leaders and 22% through the police and legal service authorities. Key social protection services facilitated included obtaining government identity, voter, labor and food security cards, domicile certificates, housing schemes, insurance and bank loans to individuals and self‐help groups, admission to colleges, and access to vocational training and livelihood opportunities.

**Conclusions/Next steps**: Scaling‐up, financing, and mainstreaming of community‐led interventions are critical for reducing VDE and promoting LGBTIQ+ Inclusive services.

### OAF0203 Sex worker led campaigns to decriminalise sex work in Australia

J. Kim ^1^

^1^Scarlet Alliance Australian Sex Workers Association, Sydney, Australia

**Background**: Decriminalisation of sex work has been recognised as definitively linked to the reduction of HIV risk and rates yet progress has been slow. NSW, Australia was the first jurisdiction in the world to decriminalise sex work and recently there has been growing momentum throughout Australia towards positive sex work law reform. Strong partnerships between sex workers and government and supported by allied organisations and unions has led to the decriminalisation of sex work in NT in 2020 and a commitment to decriminalise in VIC and QLD. This paper outlines the sex worker led campaigns to decriminalise sex work, how it has impacted on sex workers lives and why it is essential for the rights, health and safety of sex workers.

**Description**: Scarlet Alliance, Australian Sex Workers Association is the national peak organisation representing sex workers and sex worker organisations throughout Australia. Scarlet Alliance and our member orgs throughout the country have been leading the push to fully decriminalise sex work. This has involved public awareness campaigns, lobbying and briefing of politicians, government departments and allies, involvement in submissions processes, motions and bills and most importantly ensuring that local sex workers lead, inform and guide in all stages and levels of the law reform process.

**Lessons learned**: There are a number of misconceptions of what decriminalisation of sex work is, what it does and does not mean and how it works in practice. Through lessons learnt in all stages of the decriminalisation process, from initiation of campaigns to the implementation of decriminalisation, we will share our experiences and demonstrate why decriminalisation of sex work is the best practice model of sex work regulation.

**Conclusions/Next steps**: It is widely recognised that decriminalisation is the optimal model for sex work legislation. A decriminalised framework removes police as regulators of the sex industry, repeals criminal laws specific to the sex industry, regulates sex industry businesses through standard business, planning and industrial codes, and does not single out sex workers for specific legislation. In doing so, a decriminalised system removes barriers to HIV prevention, amplifies opportunities for health promotion and magnifies capacities for peer education.

### OAF0204 Integration of a peer‐led depression screening and linkage‐to‐care intervention among transgender women living with and at risk for HIV at a transgender‐led health clinic in Bangkok, Thailand

R. Janamnuaysook ^1,2^, K. Janthawilai^1^, S. Wainipitapong^3^, P. Srimanus^1^, A. Tangmunkongvorakul^4^, J. Ross^5^, A.H. Sohn^5^, C.A. Mellins^6,7^, M.L. Wainberg^8^, M.M. Philbin^7^, N. Phanuphak^1,2^

^1^Institute of HIV Research and Innovation, Bangkok, Thailand, ^2^Center of Excellence in Transgender Health, Chulalongkorn University, Bangkok, Thailand, ^3^Chulalongkorn University and King Chulalongkorn Memorial Hospital, Bangkok, Thailand, ^4^Research Institute for Health Sciences, Chiang Mai University, Chiang Mai, Thailand, ^5^TREAT Asia, amfAR, Bangkok, Thailand, ^6^HIV Center for Clinical and Behavioral Studies, New York State Psychiatric Institute and Columbia University, New York, United States, ^7^Columbia Mailman School of Public Health, Columbia University, New York, United States, ^8^Department of Psychiatry, New York State Psychiatric Institute and Columbia University, New York, United States

**Background**: We aimed to demonstrate the feasibility of mental health service integration by implementing a peer‐led depression screening and linkage‐to‐care intervention at the Tangerine Clinic in Bangkok, Thailand, which provides transgender‐led and gender‐affirming health services for HIV prevention and treatment and sexual health for transgender women (TGW) living with and at risk for HIV.

**Description**: We used the Consolidated Framework for Implementation Research (CFIR) to develop strategies to integrate depression screening, diagnosis, and linkage‐to‐treatment services into routine clinical practice. Implementation strategies were developed in consultation with transgender clinic staff and peer counselors, including 1) identifying early adopters; 2) collaborating with a psychiatrist to strengthen the capacity of clinic staff; 3) developing a formal implementation blueprint; 4) providing access to ongoing psychiatric consultation. We piloted an intervention to conduct these activities with four transgender counselors and two nurses, trained by a psychiatrist. Screening tools included the Patient Health Questionnaire (PHQ2/PHQ9). Participants with PHQ‐9 score ≤4 received psychosocial support counseling; those with more severe symptoms were referred to a psychiatrist. Safety planning for mental health emergencies and referral systems for diagnosis and treatment were established. Implementation outcomes included the numbers and proportions of TGW screened, identified with depressive symptoms, and referred for specialist care.

**Lessons learned**: From 10/2021‐1/2022, clinic staff recruited 205 TGW; 177 (86%) agreed to depression screening, of whom 2.3% were living with HIV and 47% were taking HIV pre‐exposure prophylaxis (PrEP). Overall, 51% met clinical cut points on PHQ‐2 requiring PHQ9 administration; 80% reported none‐to‐minimal symptoms (PHQ‐9 score ≤4), 10% mild symptoms (score=5‐9), and 6.7% moderate to moderately severe symptoms (score ≥10). Those with none‐to‐minimal symptoms received psychosocial support counseling by trained transgender counselors or nurses. Those with more severe symptoms were all successfully linked to psychiatric evaluation and treatment. The high rates of acceptance of screening and linkage‐to‐care showed that integration of peer‐led mental health services into our transgender clinic was feasible and acceptable.

**Conclusions/Next steps**: Development of mental health care implementation strategies that are adapted to local cultural contexts and available resources can expand available health resources. Qualitative data are needed to guide further development of mental health service integration strategies.

### OAF0205 Human rights violations faced by women from key populations in Ukraine: evidence collected through the community‐based monitoring approach

N. Semchuk ^1^, O. Pashchuk^1^

^1^ICF "Alliance for Public Health", Monitoring and Evaluation, Kyiv, Ukraine

**Background**: Women who belong to vulnerable communities such as injecting drug users, people living with HIV, sex workers, opioid substitution therapy (OST) patients and other are widely experiencing multiple stigma and discrimination in Ukraine. Human rights related barriers influence actual availability of HIV and other health services, as well as has an overall impact on HIV‐related national outcomes.

**Methods**: Data was collected using the Rights ‐ Evidence ‐ ACTion (REAct) system that allows to document barriers and rights violations of the key groups in their access to HIV and other health care services, as well as to respond to those barriers identified. REAct is implementing in 18 regions out of 24 in Ukraine by 70 community‐based organizations.

**Figure:**
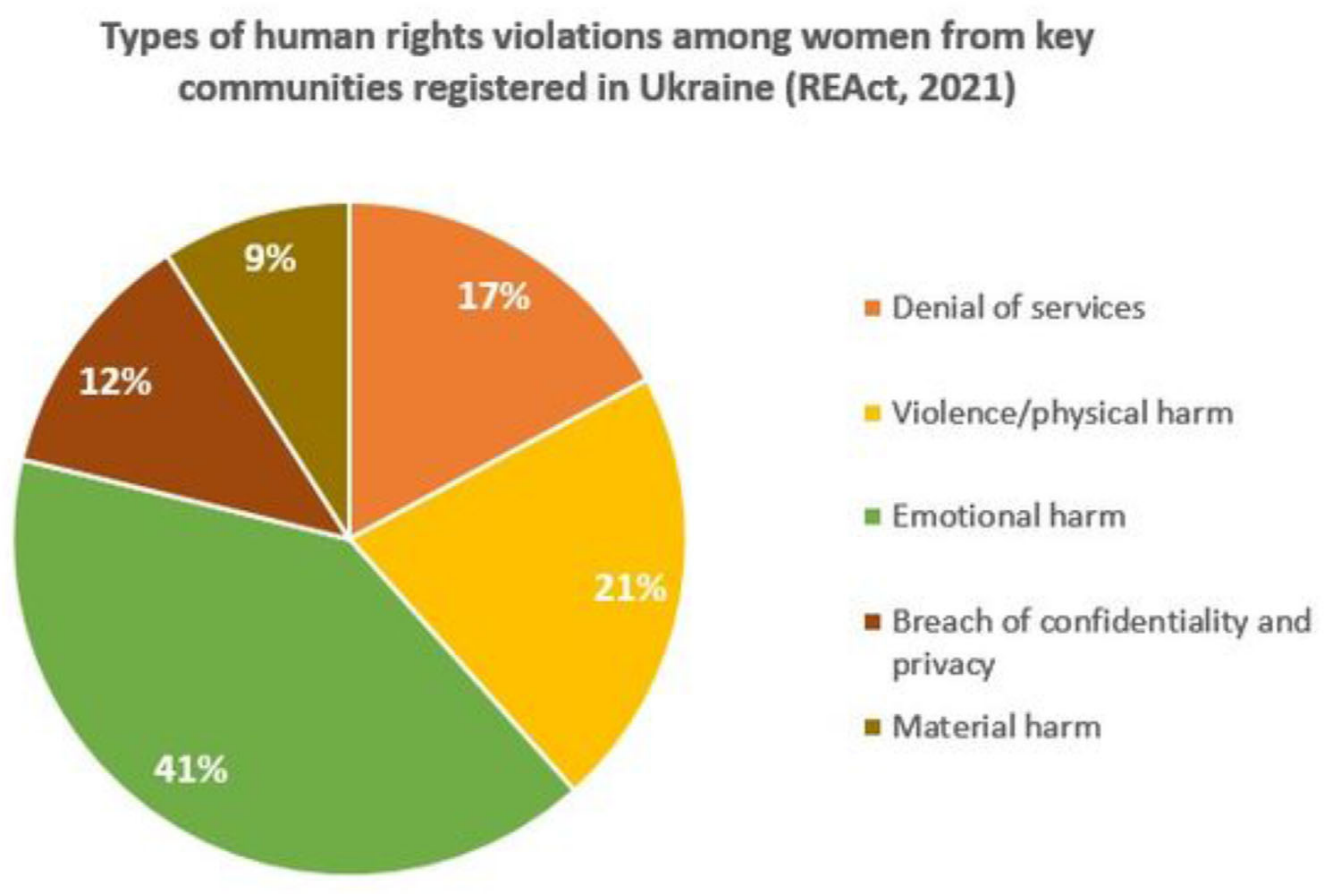
**Abstract OAF0205‐Figure 1**.

**Results**: More than 2000 cases of violations were registered in total in the REAct system in 2021, among them 818 cases were registered among women who belong to key populations. Disaggregation by key population is presented in the Table.

**Table:** 

**Abstract OAF0205‐Table 1**.	
Key populations	% of all registered cases in REAct among women (N=818) in 2021
People living with HIV	37%
OST patient	23%
People who inject drugs	17%
Sex worker	13%
People living with TB	6%
Homeless person	1%
Ex‐Prisoner	1%

Key types of human rights violations identified are: verbal abuse, denial in health services, in particular general primary and secondary outpatient health care, OST provision, denial in social services, denial of police to investigate a case of human rights violation, extortion and blackmail from the partner, sex client or police worker.

**Conclusions**: Understanding of human rights violations among women from key populations enable to identify their actual needs in health and legal services, ensure access to HIV‐related, sexual and reproductive health services, facilitate women empowerment and gender equality in the context of HIV, as well as to produce evidence‐informed recommendations to promote effective HIV response.

### OAF0302 Missing: meaningful trans inclusion in HIV national strategic plans in Eastern and Southern Africa

E. Castellanos ^1^, E. Lankiewicz^2^, A. Ogeta^3^, J. Mulucha^4^, S. Reggee^5^, N. O'Connor^1^, J. Sherwood^2^

^1^GATE, Global Action for Trans Equality, New York, United States, ^2^amfAR, The Foundation for AIDS Research, Washington DC, United States, ^3^Jinsiangu Transgender, Intersex, Non‐binary Kenya, Nairobi, Kenya, ^4^Fem Alliance Uganda, Kampala, Uganda, ^5^Transbantu Association Zambia, Lusaka, Zambia

**Background**: Trans people are disproportionately impacted by HIV, but rarely prioritized in HIV programming. The inclusion of trans people in policy and planning documents, including HIV National Strategic Plans (NSPs), is a crucial step towards increasing the national prioritization of trans people and guiding international investments. We investigate the current state of trans inclusion in NSPs in Eastern and Southern Africa through a mixed methods approach to highlight gaps and opportunities for meaningful trans inclusion.

**Methods**: NSPs from 16 high HIV prevalence countries in Eastern and Southern Africa (current as of January 2021) were reviewed for trans‐inclusion in five sections: NSP narratives, epidemiological data, monitoring and evaluation (M&E) indicators and targets, activities, and budgets. In‐depth interviews with government officials (n=2) and trans organizations (n=3) in Kenya and Uganda were conducted. Key informants were community‐selected experts and interviews focused on challenges and successes in trans engagement during strategic planning. Virtual interviews were recorded, transcribed, and reviewed for key themes.

**Results**: The majority (68%, 11/16) of NSPs mentioned trans people in at least one of the five key sections, however no NSPs mentioned them in all key sections. Trans people were most often included in the narrative (63%, 10/16), followed by indicators/targets (19%, 3/16) and activity (19%, 3/16) sections of NSPs. No NSPs included trans‐specific epidemiological data or provided budgets for trans programming. In‐depth interviews revealed stakeholders were hesitant to elevate the needs of trans people where they did not have epidemiological data to demonstrate need. Barriers to community engagement with government included the under‐funding and under‐capacitation of trans organizations. Successes occurred when trans groups built within‐country coalitions, when governments explicitly involved trans people in working groups, and when international stakeholders helped push governments on critical advocacy points.

**Conclusions**: Inclusion of trans people in NSPs in Eastern and Southern Africa is low, with critical gaps in epidemiological data and budgeting for trans programming. In addition to moving towards inclusion of trans people in all key sections of NSPs, government and international funders have roles to play in funding trans epidemiological data studies and creating accessible opportunities for trans engagement in strategic planning.

### OAF0303 Disability inclusion in the national response to HIV/AIDS in Nigeria: an analysis of the national strategic plan and framework

A. Ibrahim ^1^, D.E. Lucero‐Prisno III^2^

^1^University of Ilorin, Pharmacy, Ilorin, Nigeria, ^2^Global Health Focus, London, United Kingdom

**Background**: National Strategic Plans (NSP) and National Strategic Frameworks (NSF) provide the tools and blueprints for ensuring a comprehensive national response to HIV/AIDS. Often, people with disabilities (PWDs) have been overlooked in the context of HIV risk, prevention, and services due to the common assumption that they are not at risk for HIV. Research indicates that people with disabilities (PWDs) are at an equal, if not greater, risk of HIV compared to their non‐disabled peers. As such, this paper analyzes the extent to which disability is included in the national response to HIV in Nigeria while suggesting good examples to follow.

**Methods**: A review of the most recent Nigeria National Strategic Plans (2017–2021) and National Strategic Framework (2019–2021) was conducted to determine the extent to which disability is included. The six principles employed in an earlier study, which was based on relevant rights in the UN Convention on the Rights of Persons with Disabilities, the UNAIDS International Guidelines on HIV and Human Rights, and the UNAIDS Disability and HIV Policy Briefs, were used in the analysis process as key focus areas.

**Results**: The most recent National Strategic Plan and Framework failed to integrate the needs of people with disabilities into the national response to HIV/AIDS. While provisions and plans were put in place for other vulnerable and special groups, there is little to no provision for treatment, care, and support services for persons with disability in the context of HIV and AIDS. PWDs were not recognized in the documents, not as key populations or even vulnerable groups.

**Conclusions**: Disability inclusion in the Nigeria NSP and NSF is extremely poor, particularly when compared with some other African countries like Niger or Ghana, and is indicative of the need for a review. As they are truly due for a review, it provides an opportunity to implement disability‐focused approaches, inclusive policies and strategies in the national response toward HIV/AIDS in the country as could be seen in the NSPs of Niger and Ghana. Achieving this requires utilizing a rights‐based approach, involvement of disability‐focused organizations and individuals, and the allocation of financial resources.

### OAF0304 Meaningful inclusion and effective participation of people who use drugs in shaping access to HIV/AIDS services: implications of a donor funding policies in 11 Latin American countries

C. Brentari^1^, S. Shirley‐Beavan^2^, P. Cymerman^3^, R. Torruella^4^, E. Cortes^5^, L.V. Russo ^6^

^1^Harm Reduction International, London, United Kingdom, ^2^Harm Reduccion International, London, United Kingdom, ^3^Intercambios, Buenos Aires, Argentina, ^4^Intercambios, San Juan, Puerto Rico, ^5^Lanpud, San Josè, Costa Rica, ^6^LANPUD, Buenos Aires, Argentina

**Background**: The Global Fund to Fight AIDS, Tuberculosis and Malaria (the GF) has long influenced the programming of funding on HIV/AIDS in Latin America. The GF recognises people who use (but do not inject) drugs (PUD) are a key population for HIV. Within a GF Community, Rights and Gender (CRG) short‐term technical assistance, Harm Reduction International, at the request of Lanpud – the Latin American Network of PUD, sought to understand why PUD are not included as key populations in any of the 11 GF Country Coordination Mechanisms (CCMs) analysed in Latin America.

**Description**: There are 5.5 million people who use non‐injected drugs in Latin America and the Caribbean; the number of people who inject drugs is low compared to other regions. CCMs, GF staff and civil society report that PUDs face barriers to accessing health services and are more vulnerable to HIV: they are a key population.

The GF recognises key population representation as essential within CCMs. Qualitative multi‐stakeholder consultation evidenced the absence of PUD within the 11 CCMs. CCMs reported insufficient evidence for a link between people who do not inject and vulnerability to HIV/AIDS: they currently emphasize people who inject drugs based on historical data. PUD are considered ineligible for representation. Civil society, CCMs and representatives of PUD also agreed there is a grave lack of data on the link between HIV and non‐injecting drug use.

**Lessons learned**: Evidence on HIV and tuberculosis among people who use but do not inject drugs is needed, on prevalence but also on accessibility of HIV prevention, treatment and care due to stigma, discrimination and criminalisation and the lack of harm reduction. Greater co‐ordination among networks of PUD is needed and with other key populations for their voices to be heard.

**Conclusions/Next steps**: The GF and the CCMs can play an important role in ensuring PUD are represented in relevant fora through active engagement, supporting evidence gathering and capacity building.

### OAF0305 Towards more inclusive and feminist approaches in HIV programming evaluations: transforming principles into practice

S. Shahi ^1^, J. Whitbread^2^, C. Nyambura^3^, M. Tholanah^4^, J. Tatenda Bhila^5^, N. Lahouel^6^, M.M. Shadie^7^, C. Rodriguez^8^, L. Benjamin Mwakyosi^9^, E. Boucicault^10^, M. Jean^11^, T. Khaydarova^12^, A. Oktariani^13^, M. Iacono^8^, B. Rivona^13^, F. Hale^14^, E. Bell^14^, J. Feather^15^, V. Ahlenback^16^

^1^International Community of Women living with HIV Asia Pacific (ICWAP), Kathmandu, Nepal, ^2^International Community of Women living with HIV, Toronto, Canada, ^3^ATHENA Network, Nairobi, Kenya, ^4^Making Waves Network, Harare, Zimbabwe, ^5^Zimbabwe Young Positives, Bulawayo, Zimbabwe, ^6^Association El Hayet des personnes vivants avec le VIH, Alger, Algeria, ^7^AFI Santé, Kinshasa, Congo, Democratic Republic of the, ^8^International Community of Women living with HIV ‐ ICW Argentina, Buenos Aires, Argentina, ^9^DARE Organization, Dar‐es‐Salaam, Tanzania, The United Republic of, ^10^Fondation Esther Boucicault Stanislas, Saint Marc, Haiti, ^11^AFIAVIH (Association de Femmes Haitiennes vivant avec et affectées par le VIH), Port au Prince, Haiti, ^12^Tajik network of women living with HIV, Dushanbe, Tajikistan, ^13^IPPI Indonesian Network of Women living with HIV, Jakarta, Indonesia, ^14^Making Waves Network, London, United Kingdom, ^15^Social Development Direct, Bristol, United Kingdom, ^16^Social Development Direct, London, United Kingdom

**Background**: In 2020, UNAIDS contracted SDDirect to conduct aGlobal Evaluation of the Joint Programme's work addressing linkages between HIV and VAWG. Data was collected in nine countries. Its strategic recommendations on integrating VAWG and HIV were accepted by the Co‐Sponsors'Management Response.

To achieve lasting change in the HIV response, our approach aimed to address structural inequalities inherent in standard evaluation practice, be guided by priorities of women and girls living with and affected by HIV, and be accountable to them and their networks.

**Description**: ‘Nothing about us without us’ is vital to HIV programming, research and evaluation, following principles ofGreater Involvement of People living with HIV/AIDS(GIPA), and Meaningful Involvement ofWomen living with HIV/AIDS (MIWA).

To embed these principles, SDDirect worked with ICW and ATHENA Network to establish the accountability and advisory group (TAAG), a group of 13 women living with / affected by HIV, from the 9 case study countries. They supported the evaluation process, interviewed members of networks and CSOs, and reviewed findings and recommendations.

**Lessons learned**: All evaluations should be guided by members of communities.TAAG ensured community members’ priorities were addressed and broadened the scope of who was consulted.

Multilingual working is important to make evaluations accessible to people in the countries involved. We worked in English, French, Spanish, Russian, Khmer.

Proper compensation for women's and girls’ (and their networks/organisations) contributions is essential. This recognises the value of their expertise, time and commitment, and can help remove barriers to participation when paid in advance.

Flexible internal systems facilitate involvement. Drawing on feminist practice, SDDirect adapted contracts, Conflict of Interest statements, and payment modes, funded data bundles, and translated critical elements.

Promoting transformative change: This approach aids accountability to women and girls in the implementation of evaluation recommendations.

**Conclusions/Next steps**: To meaningfully promote anti‐racist approaches, community and feminist leadership, and embrace GIPA/MIWA, concepts of 'independent evaluation' should be revisited. Women and girls who are active in their communities have commitment to lasting change and a wealth of expertise. They can play a vital role in evaluations as evaluators & experts. This needs to be properly facilitated, compensated and recognised.

### OAF0402 Bad blood: why blood donations by people living with HIV should not be a crime

E. Hatt^1^, S. Beaumont^1^, E. Bernard ^1^

^1^HIV Justice Network, Amsterdam, Netherlands, the

**Background**: At least 23 countries have laws that criminalise blood donations by people living with HIV, despite the removal of blood donation bans for gay men due to scientific advances in screening for HIV. Although these laws are invariably implemented with the legitimate objective of protecting public health, we seek to demonstrate that they fail to meet this objective and are discriminatory.

**Methods**: The HIV Justice Network's Global HIV Criminalisation Database contains case reports of HIV‐related criminal cases and criminal laws that target people living with HIV. Following recent reports of blood donation‐related prosecutions in Russia, Singapore, and the United States, we undertook desk‐based research between September to November 2021, collating and categorising all known country and jurisdictional laws that specifically criminalise blood donations by people living with HIV, and known prosecutions under these laws. We analysed these laws and cases using a global policy guidance and human rights law framework, informed by international and state‐level scientific data assessing risks of transmission via blood transfusion.

**Results**: The Global Commission on HIV and the Law, UNAIDS and UNDP all state that the use of criminal law in relation to HIV can only be legitimate where harm is intentionally caused, there is actual risk of harm, and harm actually occurs. Although some prosecutions involved people aware they were living with HIV, others ‐ notably in Singapore ‐ involved gay men who *ought to have known* their HIV status. In addition, due to advances in blood screening capabilities allowing for the removal of blood donation bans for gay men in a growing number of countries, there is now an extremely low risk of transmission through blood donations, especially in countries with advanced health systems.

**Conclusions**: These laws and prosecutions fail the proportionality test due to the low risk of transmission and the fact that they single out people with HIV. Ultimately these laws and prosecutions fail to achieve their stated aim of protecting public health, cause harm through their stigmatising effect, and violate international human rights law as they discriminate based on a characteristic protected under international law. These laws should be repealed.

### OAF0403 Measuring potential impacts of parental consent laws on adolescent HIV testing globally: multinational insights from 51 population‐based surveys

J.G. Rosen ^1^, E.M. Stone^2^, M.T. Mbizvo^3^

^1^Johns Hopkins Bloomberg School of Public Health, International Health, Baltimore, United States, ^2^Johns Hopkins Bloomberg School of Public Health, Health Policy and Management, Baltimore, United States, ^3^Population Council, Lusaka, Zambia

**Background**: HIV testing remains imperative to close gaps in both the prevention and treatment cascades, but pervasive social and structural barriers—including national policies—inhibit HIV testing uptake among priority populations, including adolescents. We assessed the relationship between age‐of‐consent laws for HIV testing and adolescent HIV testing prevalence in 51 low‐ and middle‐income countries.

**Methods**: We pooled 51 nationally representative household surveys (Demographic and Health Surveys, AIDS Indicator Surveys, and Population‐Based HIV Impact Assessments) from 2010 to 2020. We estimated the weighted country‐level prevalence of lifetime HIV testing separately for adolescent girls and boys (ages 15‐19). We then abstracted age‐of‐consent laws for HIV testing across countries. Using multivariable linear regression, we estimated the average difference in national HIV testing coverage estimates for adolescent girls and boys by age‐of‐consent restrictions for HIV testing.

**Results**: National HIV testing coverage estimates were substantially heterogeneous, ranging from 0.7% to 72.5% among girls and 0% to 73.2% among boys in Pakistan and Lesotho, respectively. Median national HIV testing prevalence estimates were 18.0% among girls and 7.5% among boys. Adjusting for region, World Bank income classification, and per‐capita health expenditure, HIV testing coverage in countries requiring parental consent for HIV testing in individuals younger than 18 years was, on average, 7.8% lower (95%CI: –14.6% to –1.0%) among girls and 8.2% lower (95%CI: –14.6% to –1.9%) among boys. Comparing countries with more restrictive (age‐of‐consent: 18 years) to less restrictive (age‐of‐consent: 14 years or younger) HIV testing laws, HIV testing prevalence was significantly lower among girls (*β* = –7.7%, 95%CI: –15.1% to –0.3%) and boys (*β* = –8.3%, 95%CI: –15.2% to –1.3%) in countries with more restrictive parental consent policies.

**Figure:**
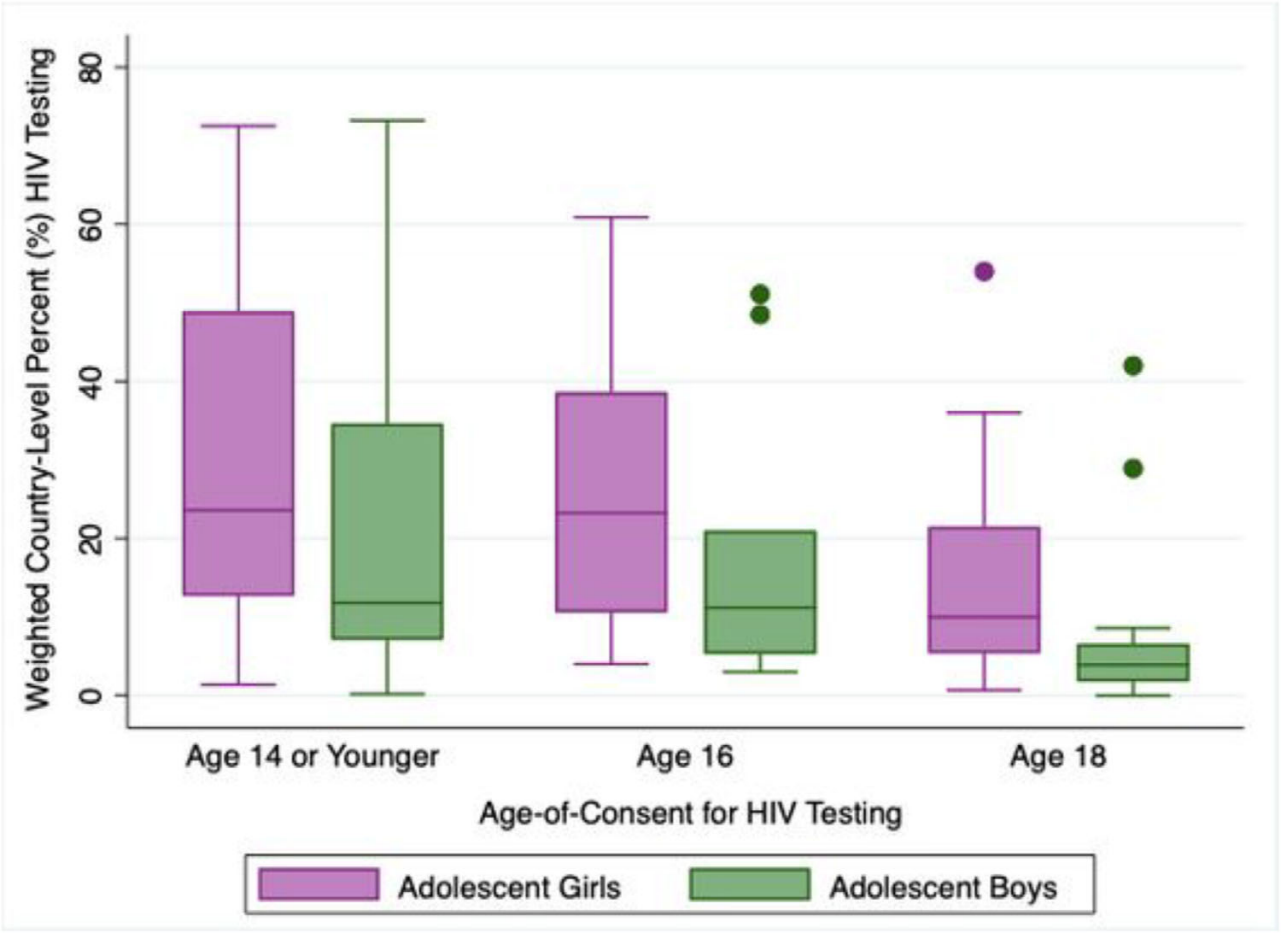
**Abstract OAF0403‐Figure 1**.

**Conclusions**: Age‐of‐consent laws are persistent obstacles to adolescent HIV testing. Revoking parental consent requirements for HIV testing is needed to expand coverage and ensure equity.

### OAF0404 Ending inequalities in access to justice: scaling up and sustaining judicial sensitization across sub‐Saharan Africa

M. Tabengwa ^1^, K. Grant^1^, O. Dingake^2^, F. Githiru^3^, S. Mia^1^, M. Ngugi^3^

^1^UNDP, Johannesburg, South Africa, ^2^UNDP, Johannesburg, Seychelles, ^3^UNDP, Nairobi, Kenya

**Background**: In 2019, the African Regional Judges’ Forum initiated and developed a Judicial Training Manual, the first of its kind in Africa, to expand sustainable judicial sensitization on HIV, law and human rights affecting key and vulnerable populations across sub‐Saharan Africa, and to promote rights‐focused jurisprudence that challenges laws discriminating against women and young people, and criminalizing HIV transmission, drug use, sex work, same‐sex sex and transgender people.

**Description**: The Forum, initiated by prominent African judiciary and supported by UNDP, arose out of the Global Commission on HIV and the Law's recommendations. It allows for sharing expertise, sensitizing judiciary and promoting judicial excellence. It has contributed to progressive jurisprudence repealing punitive laws or practices, and strengthened enabling environments, human rights and access to justice for populations in Africa, consistent with national bills of rights, regional and global commitments.

In 2019, noting the limited resources available and wishing to draw on successes and scale up efforts, the Forum worked with national judicial training institutes(JTIs) to develop the innovative Manual. The consultative process drew on the expertise and networks of the Forum and its partners, and included a Judicial Roundtable with JTIs,a needs assessment and ongoing feedback.

**Lessons learned**: The regular engagement and judicial sensitization by and amongst judges over the years has yielded rich pro‐rights jurisprudence in the region. This engagement also enriched and informed the format, methodological approach and content of rights‐based training materials including e.g. (i) the focus on criminalization of HIV and key populations as well as gender‐based violence; (ii) the inclusion of scientific and medical evidence; (iii) the importance of involving affected populations in methodologies; and (iv) materials capable of updating and adaptation to national contexts and differing jurisprudential systems within Francophone, Anglophone and Lusophone Africa.

**Conclusions/Next steps**: Critical next steps include working with JTIs to create awareness and disseminate the Manual, including on online platforms, and to identify technical support needs for its adaptation, integration and use within national judicial training, to expand judicial sensitization and mitigate the impact of stigma, discrimination and violence on key and vulnerable populations in the African justice sector.

### OAF0405 The race to end AIDS: Ghana's prospects in light of a proposed anti‐LGBTQ bill

P. Ayamah ^1^, K. Atuahene^1^, F. Nana Poku^1^, R. Sackitey^1^, S. Abbah^2^, B. Adjei^2^

^1^Ghana AIDS Commission, Accra, Ghana, ^2^Micdak Charity Foundation, Kumasi, Ghana

**Background**: UNAIDS has identified inequality as a foremost factor for many countries’ inability to reach the 90‐90‐90‐targets by 2020. Discriminatory laws and policies drive inequality which hinders the achievement of health goals especially relating to HIV. Ghana's Parliament is currently considering a bill to among others “proscribe LGBTQ+ and related activities; proscribe the promotion, propagation of, advocacy for, support of or funding” of LGBTQ+ activities. In this study, we explored Ghana's prospects at ending AIDS from the perspectives of key stakeholders in light of the proposed bill. Relevant data were analysed for context.

**Methods**: This was a qualitative cross‐sectional study. Data collection done virtually (via zoom and phone call/WhatsApp); respondents selected by convenience and snow‐balling. There was focus‐group discussions with peer‐educators(n=19); semi‐structured interviews with service‐providers(n=15) and independent experts (n=2). Transcripts were coded, themes identified and analysed.

**Results**: Ghana's HIV prevalence as at 2020 was 1.68% with an estimated 346,120 PLHIV; total new infections was 18,928; ART(15+) and PMTCT coverage stood at 63% and 72% respectively. Regarding the 90‐90‐90, by 2020, 63.20% of all PLHIV knew their status, 95.46% and 72.97% of which were on ART, and had achieved viral‐suppression respectively. Ghana has an estimated MSM‐population of 54,759; 52% being bisexual (bridging‐population) with a very high HIV prevalence (18%) among same. Against this background, key stakeholders contend that the ensuing law will exacerbate Ghana's difficulties at attaining the 95‐95‐95 targets by 2025 and ending AIDS by 2030. Peer‐educators and service‐providers have noted a spike in violence‐and‐stigma against KPs since the bill was introduced. HIV service‐providers contend that the ensuing law will undermine their mandate, drive KPs underground and stifle external funding. Independent experts and other opponents contend that it will “establish a system of state‐sponsored discrimination and violence” against sexual‐minorities.

**Conclusions**: There's unanimity among respondents that the ensuing law (whose passage they deem ‘very likely’) will pose a structural‐barrier and deepen inequality against KPs thereby posing a considerable obstruction to Ghana's prospects at ending AIDS by 2030. Stakeholders should advance necessary amendments to assuage its impact and commission a study to determine the actual impact if passed into law.

## POSTER EXHIBITION ABSTRACTS

### PESAA01 PICH115: a new potential target to control HIV latency

M. Da Rocha ^1^, T. Habib^1^, A. Delannoy^1^, E. Wilhelm^1^, B. Bell^1^

^1^Université de Sherbrooke, Département de Microbiologie et d'Infectiologie, Sherbrooke, Canada

**Background**: Latency is the main obstacle toward Human Immunodeficiency Virus (HIV) eradication. Current approaches to fight latency, Shock and Kill and Block and Lock, both focus on modulating HIV gene expression. Our laboratory recently identified a cellular protein complex, the *Pre‐Initiation Complex of HIV* (PICH), that binds the HIV promoter. Mass spectrometry was then used to identify PICH proteins, and depletion of PICH proteins by siRNA demonstrated their positive impact on HIV gene expression. PICH are potential drug targets and understanding their role on HIV transcription could help develop molecules to control latency. Here, we present new data on PICH115, a protein known to interact with Tat.

**Methods**: First, we mapped the interaction site between Tat and PICH115. We designed plasmids coding for the different domains of GST‐Tat and His‐PICH115 to produce recombinant proteins for GST‐Pulldowns. *In cellulo* experiments have also been performed, by overexpressing the different domains of PICH115‐HA and a Tat‐Flag protein in HEK293T to perform a Co‐ImmunoPrecipitation with the protein extracts.

Then, we studied the impact of PICH115 on the interaction between Tat and the viral TAR RNA, which is essential for HIV transcription. To determine if PICH115 influence this interaction, we performed ElectroMobility Shift Assays using a fluorescent TAR RNA probe incubated with recombinant Tat and PICH115.

**Results**: *In vitro*, a direct interaction happens between the core domain of Tat and both the N‐acetyltransferase and the tRNA‐Binding domains of PICH115.*In cellulo*, the N‐acetyltransferase and the tRNA‐Binding domains of PICH115 are required for the interaction with Tat.

Our data further show that the presence of PICH115 increases the binding of Tat to TAR *in vitro*.

**Conclusions**: In conclusion, we identified the interaction site between Tat and PICH115 and demonstrated its enhancement of the Tat‐TAR interaction. In the future, we will investigate the potential role of PICH115 on viral mRNA post‐transcriptional modification and its impact on HIV gene expression. This could lead to a better understanding of the role of this cellular protein on HIV replication and could allow the development of new Shock and Kill or Block and Lock strategies. Indeed, an inhibitor of PICH115 have been identified recently.

### PESAA02 Novel roles for PSGL‐1 in HIV infection: virions with incorporated PSGL‐1 can be captured by selectins and transferred to permissive cells

J. Burnie ^1,2^, A.T. Persaud^1,2^, L. Thaya^1,2^, Q. Liu^3^, H. Miao^3^, S. Grabinsky^1^, V. Norouzi^1^, P. Lusso^3^, V.A. Tang^4^, C. Guzzo^1,2^

^1^University of Toronto Scarborough, Biological Sciences, Toronto, Canada, ^2^University of Toronto, Cell and Systems Biology, Toronto, Canada, ^3^National Institute of Allergy and Infectious Diseases (NIAID), Viral Pathogenesis Section, Laboratory of Immunoregulation (LIR), Bethesda, United States, ^4^University of Ottawa, Faculty of Medicine, Department of Biochemistry, Microbiology, and Immunology, Ottawa, Canada

**Background**: P‐selectin glycoprotein ligand‐1 (PSGL‐1/CD162) was recently identified as a restriction factor present within the HIV envelope. While it strongly inhibits infection in viruses generated through transfection to overexpress PSGL‐1, viruses produced through infection with endogenous levels of PSGL‐1 remain infectious. To date, no studies have shown whether PSGL‐1 is present on a range of viral isolates or on viruses circulating *in vivo*. Similarly, it is unknown whether virion‐incorporated PSGL‐1 can bind its natural receptors (selectins), which may have implications on the ability of HIV to traffic *in vivo*.

**Methods**: Using transfection and infection, we generated viruses that express differential levels of PSGL‐1. With these viruses we compared the antiviral effect of PSGL‐1 and quantified levels of PSGL‐1 on virions using virion capture assays and flow virometry. Finally, we assessed the capacity of virion‐incorporated PSGL‐1 to facilitate binding to selectins, and whether selectin‐captured viruses could be transferred to nearby cells for infection.

**Results**: PSGL‐1 was far more abundant on our viruses produced through transfection compared to viruses produced through infection of primary cells. Infectivity assays confirmed the dose‐dependent antiviral activity of PSGL‐1 in viruses engineered to express varying levels of PSGL‐1, while viruses produced through infection of T cells remained infectious. Notably, virion‐incorporated PSGL‐1 was present in viruses of all clades tested, including a range of clinical isolates. Most importantly, we found that PSGL‐1 on virions could be captured by selectins, and that captured virus could be transferred to nearby permissive bystander cells.

**Conclusions**: PSGL‐1 is a potent antiviral when expressed at high levels in the HIV envelope, however, it is less efficacious at endogenous levels on virions. These results caution interpretations of the effects of PSGL‐1 on HIV infection when using viruses produced through transfection. The presence of PSGL‐1 on a broad range of viral isolates and patient samples demonstrates the importance for further studies of how PSGL‐1 can impact HIV biology. Strikingly, our finding that virion‐associated PSGL‐1 can facilitate capture by its cognate receptors and subsequent transfer to bystander permissive cells, suggests that PSGL‐1 may have additional roles in facilitating HIV infection, in addition to its antiviral activity.

### PESAA03 The HCV infection in HIV patients drives chromosome 14 microRNA cluster (C14MC) dysregulation

D. Valle Millares ^1^, Ó. Brochado‐Kith^1^, L. Martín‐Carbonero^2^, L. Domínguez^3^, P. Ryan^4^, I. De los Santos^5^, S. De la Fuente^6^, J.M. Castro‐Álvarez^2^, M. Lagarde^3^, G. Cuevas^4^, M. Mayoral‐Muñoz^2^, C. Crespo‐Bermejo^1^, V. Lara‐Aguilar^1^, M.Á. Jiménez‐Sousa^1^, S. Resino^1^, V. Briz^7^, A. Fernández‐Rodríguez^1^

^1^Institute of Health Carlos III, Unit of Viral Infections and Immunity, Majadahonda, Spain, ^2^Hospital La Paz Institute for Health Research (IdiPAZ), Madrid, Spain, ^3^Research Institute Hospital 12 de Octubre (i+12), Internal Medicine Service, Madrid, Spain, ^4^Infanta Leonor Teaching Hospital, Department of Infectious Diseases, Madrid, Spain, ^5^Hospital Universitario de La Princesa, Internal Medicine Service, Madrid, Spain, ^6^Hospital Puerta de Hierro, Internal Medicine Service, Madrid, Spain, ^7^Institute of Health Carlos III, Laboratory of Reference and Research on Viral Hepatitis, Majadahonda, Spain

**Background**: Around 2.75 million of HIV individuals worldwide are co‐infected with hepatitis C virus (HCV), showing higher rate of cirrhosis, liver failure, and hepatocellular carcinoma among others. miRNAs have a key role in postranscripcionally regulating these biological processes. The chromosome 14 miRNA cluster (C14MC) in the 14q32 region is one of the largest miRNA cluster of the genome, harbouring more than 50 maternally imprinted miRNAs‐encoded genes. Dysregulation of this cluster have a key role in cancer prognosis, and pathogenesis of non‐alcoholic liver diseases, but the impact of viral infections in the C14MC remains unknown. We aim to analyse the C14MC in HIV patients with different exposure to HCV, to explore their possible association with risk of cancer development and sex bias.

**Methods**: SmallRNA sequencing analysis was performed in PBMCs from 117 HIV+ infected patients: 45 HIV+ patients chronically infected with HCV (HIV/HCV+), 36 HIV+ that spontaneously clarified HCV after an acute infection (HIV/HCV‐) and 36 HIV+ patients without previous HCV infection (HIV+). Thirty‐two healthy patients were used as controls (HC). Significantly differentially expressed (SDE) miRNAs were calculated (fold‐change > 1.5 and p‐value < 0.05 adjusted by Benjamin–Hochberg correction). Only those SDE miRNAs located at the C14MC were considered.

**Results**: Significant clinical differences were observed between HIV patients for the HIV transmission route (p < 0.001). There were no differences in CD4+ or CD8+ T‐cell parameters between any HIV+ patients. We identified SDE miRNAs of the C14MC with respect to healthy controls in HIV (n = 22), HIV/HCV+ (n = 25) and HIV/HCV‐ (n = 32). After analyzing each group of patients by gender, we found that the dysregulation of C14MC was mainly limited to a strong upregulation in males rather than females. The highest differences were observed for HIV/HCV‐ male patients, where 41 out of 54 miRNAs of the C14MC were dysregulated (37 up and 4 down‐regulated).

**Conclusions**: Our findings indicate that HCV exposure strongly disrupts C14MC expression. Furthermore, we found a sexual bias in the dysregulation of miRNAs at the C14MC especially in male HCV spontaneous clarifiers. Additional studies should be performed to decipher the role of C14MC in infectious diseases and HCV‐cancer related development.

### PESAA04 The host factor p32 facilitates HIV infection by stabilizing the HIV‐1 transcriptionaltrans‐activatorTat protein

L. Mori ^1^, C. Li^1^, S. Lyu^1^, A. Getzler^1^, M. Pipkin^1^, S. Valente^1^

^1^Scripps Florida, Department of Microbiology and Immunology, Jupiter, United States

**Background**: Following HIV integration into the host genome, viral expression is modulated by the combinatorial activity of the HIV transcriptional activator Tat, host transcription factors and chromatin remodeling complexes. To expand our knowledge of the mechanisms regulating HIV transcription, we sought to identify novel proteins associating with the HIV promoter.

**Methods**: We used a chromatin affinity purification approach that takes advantage of specific single guide RNAs (sgRNAs) and endonuclease deficient Cas9 (dCAS9) to enrichonintegrated HIV promotersfollowed by mass spectrometry (ChAP‐MS).

**Results**: We identified a total of 161 proteins that were ranked based on their enrichment in active versus transcriptionally silenced promoters with theTat inhibitor, didehydro‐Cortistatin A (dCA). Among the top 30 hits enriched in active promoters we identified several Tat interacting proteins and subunits of RNAPII holoenzyme, while in latent promoters we identified several histones (H1, H2, H4), an expected outcome since dCA promotesheterochromatinization of theHIV promoter. Genes chosen for follow‐up were prioritized based on novelty in HIV transcriptional regulation, reproducibility and biological insight as to potential function(s).p32, also called ASF/SF2 splicing factor‐associated protein, wasenriched inactively transcribing HIV promoters and absent in silenced ones. Chromatin immunoprecipitation analysis confirmed the presence of p32 on active HIV promoters and its recruitment enhanced by Tat. The RNA interference of p32 significantly reduced HIV transcription in primary CD4^+^T cells as well as in HIV chronically infected cells, independently of either HIV splicing or p32 splicing activity. Conversely, overexpression of p32 specifically increased Tat‐dependent HIV transcription. In effect, p32 was found to directly interact with Tat's basic domain enhancing Tat stability and half‐life. The stabilization of Tat by p32 converged in an increased Tat association with the HIV LTR and RNAPII.

**Conclusions**: Using a novel chromatin affinity purification strategy, we identified p32 as a novel host factor that physically interacts and stabilizes Tat protein enhancing Tat‐dependent HIV transcription. These results highlight p32 as a potential novel target for HIV transcriptional modulation andfurthered our understanding of the mechanisms regulating Tat mediated transcription.

### PESAA05 Activation of anti‐viral innate immunity in epithelial cells as a potential cellular mechanism for preferential R5 transmission at genital mucosa

A. Nazli ^1,2^, M. Atif Zahoor^1,2^, C. Kaushic^1,2^

^1^McMaster University, Department of Medicine, Hamilton, Canada, ^2^McMaster University, McMaster Immunology Research Center, Hamilton, Canada

**Background**: Women make up approximately half the population living with HIV/AIDS. Majority of HIV‐1 transmission in women occurs through heterosexual intercourse via the female reproductive tract (FRT). Although both CCR5‐tropic (R5) and CXCR4‐tropic (X4) HIV‐1 strains are present in semen, primary infection in FRT occurs almost exclusively through R5 HIV‐1. The mechanism underlying this preferential selection of HIV‐1 R5 during mucosal transmission is not completely understood. We examined the interactions between X4 and R5 HIV‐1 and genital epithelial cells (GECs) to gain a better understanding of the preferential selection of R5 strains.

**Methods**: This study was conducted on primary GECs isolated from tissues obtained from women undergoing hysterectomy in McMaster Hospital, following informed consent. Fluorescently labelled X4 and R5 HIV‐1 was added to primary GECs and virus coming through cells on basolateral side was titrated on TZMb‐l cell line. HIV‐1 was measured by P24 ELISA. Intracellular trafficking of HIV‐1 was studied by immunofluorescence microscopy. Expression of interferon stimulated genes (ISGs) was measured by quantitative PCR. Interferon‐β production was measured by ELISA.

**Results**: GECs showed significantly higher interferon‐β production, IFNAR1 and ISG expression in response to X4 HIV‐1, compared to R5 strains. The IFN‐β response against X4 HIV‐1 was mediated through TLR2 signaling in the endosomal compartment. TLR2 pathway activation resulted in upregulation of BST‐2 and ISG‐15, resulting in sequestration of the virus in the endosomal compartment for more than 96 hours. Blocking endosomal pathway blocked the anti‐viral response and sequestration of X4 virus. Blocking BST2 and ISG15 by siRNA inhibited sequestration of X4 HIV‐1. In contrast, R5 virus was rapidly transcytosed through the cells to the basolateral side, avoiding recognition through TLR2, and with minimal activation of IFN‐β and ISGs. Further examination showed that X4 and R5 viruses were directed into different cellular compartment through differential binding to CXCR4 and CCR5 co‐receptors, respectively.

**Conclusions**: Our results indicate thatX4 HIV‐1 induces a robust anti‐viral immune response, resulting in entrapment of virus within GECs, while R5 HIV‐1 evades the innate immunity resulting in preferential selection of R5 HIV‐1 for mucosal transmission. Understanding the mechanism of transmission will help develop prevention strategies.

### PESAA06 Dual role of HIV‐1 Envelope Signal Peptide in Immune Evasion

C. Upadhyay ^1^, P. Gadam Rao^1^, R. Feyznezhad^1^

^1^Icahn School of Medicine at Mount Sinai, Medicine, New York, United States

**Background**: The HIV‐1 Env signal peptide (SP) initiates Env biogenesis and is an important contributor to Env glycosylation and functions. HIV‐1 Env is generated from Vpu/Env encoded bicistronic mRNA such that the 5`end of Env N‐terminus, that encodes for Env SP overlaps with the 3` end of Vpu. Env SP displays high sequence diversity, which also translates into high variability in Vpu sequence. This study was aimed to understand the effect of sequence polymorphism in the Vpu‐Env overlapping region (VEOR) on the functions of two vital viral proteins i.e., Vpu, and Env.

**Methods**: We used infectious molecular clone (IMC) pNL4.3‐CMU06 (WT) and swapped its SP (i.e., VEOR) with that from other HIV‐1 isolates (MW, 398F1, CH119 and 271.1). We examined the effects of VEOR on Env (as SP) and Vpu functions.

**Results**: Swapping VEOR did not affect virus production in the absence of tetherin however, presence of tetherin significantly altered the release of virus progeny compared to the WT. Notably, MW VEOR abrogated Vpu's ability to augment the release of progeny virions from infected cells. When MW VEOR was tested in context of two other HIV‐1 isolates (SF162 and REJO) similar reduction in virus release was observed. Swapping VEOR also changed Vpu's ability to down‐modulate surface expression of human CD4. We next tested the effect of these swaps on Env properties and functions. Analyzing the binding of monoclonal antibodies to membrane‐embedded HIV‐1 Env revealed changes in the antigenic landscape of swapped Envs vs the WT and also altered the virus sensitivity to antibody‐mediated neutralization. These swaps affected the oligosaccharide composition of N‐glycans as shown by changes in DC‐SIGN‐mediated virus transmission. Importantly, all IMCs differ only in their VEOR and otherwise have identical sequences.

**Conclusions**: Collectively, this study shows that polymorphisms in the VEOR has direct implications on HIV‐1 infection. This overlapping region 1) regulate Vpu functions and Vpu‐host interactions, facilitating virus replication and infection establishment, 2) impact Env glycosylation altering Env interaction with antibodies as Env‐SP; facilitating immune escape and virus transmission via DC‐SIGN. Thus, by incorporating changes in this region the virus uses it as another mechanism for immune evasion.

### PESAA07 Overt IL‐32 Isoform Expression at Intestinal Level During HIV‐1 Infection is Negatively Regulated by IL‐17A *via* PPARgamma and Retinoic Acid‐Dependent Mechanisms

E. Moreira Gabriel ^1,2^, T. Wiche Salinas^1,2^, A. Gosselin^1,2^, E. Larouche‐Anctil^2^, M. Durand^2^, A. Landay^3^, M. El‐Far^2^, C. Tremblay^1,2^, J.‐P. Routy^4^, P. Ancuta^1,2^

^1^Université de Montréal, Departement de Microbiologie, Infectiologie et Immunologie, Montréal, Canada, ^2^CHUM‐Research Centre, Montréal, Canada, ^3^Rush University Medical Center, Chicago, United States, ^4^McGill University Health Centre, Montréal, Canada

**Background**: The interplay between intestinal epithelial cells (IEC) and Th17 cells is key for mucosal immunity homeostasis. HIV infection is associated with impaired intestinal barrier functions leading to chronic immune activation, a process not normalized by antiretroviral therapy (ART). Such alterations coincide with the overexpression of interleukin (IL)‐32, a cytokine family composed of multiple isoforms. IL‐32 overexpression was associated with the loss of HIV control in elite controllers and linked to non‐AIDS co‐morbidities, such as cardiovascular disease (CVD). The involvement of specific IL‐32 isoforms in HIV gut pathogenesis remains poorly investigated.

**Methods**: Sigmoid colon biopsies (SCB) and blood were collected from ART‐treated PLWH (HIV+ART; n = 17; median age: 55 years; CD4 counts: 679 cells/ml; ART: 72 months) and age‐matched HIV‐uninfected controls (HIVneg; n = 5). Cells were isolated by enzymatic digestion/gradient centrifugation. The IEC HT‐29 line was exposed to TNF‐α, IL‐17A, and HIV, in the presence/absence of T0070907 (PPARγ antagonist) and/or *all‐trans* retinoic acid (ATRA). IL‐32α/β/γ/d/ε/θ and IL‐17A mRNA were quantified by real‐time RT‐PCR. IL‐32 protein was quantified by ELISA. The IEC HIV *trans*‐infection capacity was assessed upon co‐culture with CD3/CD28‐activated CD4+ T‐cells.

**Results**: Among all isoforms tested, IL‐32β was the predominant one, with expression levels upregulated in SCB of HIV+ART compared to HIVneg. IL‐17A mRNA levels negatively correlated with IL‐32β levels. IL‐32β/γ/ε isoforms were also detected in HT‐29 exposed to TNF‐α and HIV. IL‐17A significantly decreased IL‐32 β/γ/ε mRNA and cell‐associated IL‐32 protein expression induced by TNF‐α. IL‐17A increased HIV *trans*‐infection. ATRA boosted the IL‐17A effects and further increased HIV *trans*‐infection with coincidental reduction of IL‐32 expression; T0070907 exhibited opposite effects.

**Conclusions**: Our results provide a cartography of IL‐32 isoform expression in the colon and blood of ART‐treated PLWH. They also reveal the capacity of the Th17 hallmark cytokine IL‐17A to attenuate overt IL‐32 expression in inflamed IEC, while promoting HIV dissemination *via* PPARγ and RA‐dependent mechanisms. This is consistent with the documented antiviral properties of IL‐32. Our results support a model in which inflamed IEC are an important source of IL‐32, especially upon HIV‐mediated Th17 depletion, and reveal the opposite role of IL‐17A in reducing overt IL‐32 expression and favoring HIV dissemination at intestinal level.

### PESAA08 Estradiol inhibits HIV‐1_BaL_ infection and induces CFL1 expression in peripheral blood mononuclear cells and endocervical mucosa

N. Verma ^1^, S. Mukhopadhyay^1^, P. Barnable^1^, M.G. Plagianos^1^, N. Teleshova^1^

^1^Population Council, HIV, New York, United States

**Background**: Although several reports suggested an inhibitory effect of estradiol (E2) on HIV infection, the mechanism of this effect remains understudied. Analysis of endocervical transcriptome in proliferative and secretory phases of the menstrual cycle demonstrated upregulated gene expression of actin‐binding protein CFL1 in the E2‐dominated proliferative phase. Actin cytoskeleton plays an integral role in the regulation of HIV infection. This study was designed to explore the role of CFL1 in E2‐mediated effects on HIV infection in PBMCs and endocervical mucosa.

**Methods**: PBMCs were isolated from anonymous healthy HIV uninfected blood donors. Human endocervical tissues without gross pathological changes from 32–50 years old subjects were obtained from routine hysterectomies through the National Disease Research Interchange. PBMCs and endocervical tissue explants were incubated with E2 (100–10000 pg/ml) for 48h and then challenged with HIV‐1_BaL_ (1000 TCID_50_/10^6^ PBMCs and 500 TCID_50_/explant), washed and cultured for 14 days in the presence of E2 (vs. untreated control). Select experiments included 3TC, Raloxifene (selective estrogen receptor modulator), and LIMKi3 (LIMK1/2 inhibitor blocking CFL1 phosphorylation). The infection was monitored by HIV *gag* one‐step qRT‐PCR. CFL1 expression was analyzed by qRT‐PCR, Immunofluorescence microscopy (IF) and Western Blot (WB). p24 expression was monitored by IF. Cytokines and chemokines in infected tissue supernatants were measured using 25‐plex Luminex kit.

**Results**: E2 dose‐dependently inhibited HIV‐1_BaL_ infection in PBMCs and endocervix (*p* < 0.01). Raloxifene blocked E2‐mediated HIV‐1_BaL_ inhibition. No consistent significant increase in CFL1 mRNA expression was induced by E2. At the protein level, E2 augmented total CFL1 and phosphorylated CFL1 (pCFL1) and increased the pCFL1/CFL1 ratio in uninfected and infected PBMCs and endocervix. LIMKi3 reverted the phenotype and restored infection levels; blocked E2‐induced increase in total CFL1 and pCFL1; and decreased the pCFL1/CFL1 ratios in PBMCs and endocervix. Additionally, Luminex analysis revealed decrease in pro‐inflammatory chemokines CXCL10 and CCL5 in endocervix incubated with E2 (*p* < 0.05).

**Conclusions**: Our data propose a link between E2‐mediated anti‐HIV activity and CFL1 expression in PBMCs and endocervical mucosa. The data support exploration of cytoskeletal pathway targets for development of prevention strategies against HIV.

### PESAA09 Excess BAFF alters the Breg potential of human marginal zone B‐cells in the context of HIV‐1 infection

K. Doyon‐Laliberté ^1,2^, M. Aranguren^1,2^, M. Byrns^1,2^, J. Chagnon‐Choquet^1,2^, M. Paniconi^3^, J.‐P. Routy^4^, C. Tremblay^1,2^, N. Brassard^2^, M.‐C. Quintal^5,6^, D. Kaufmann^7,2^, J. Poudrier^1,2^, M. Roger^1,2^

^1^Université de Montréal, Microbiologie, Infectiologie et Immunologie, Montréal, Canada, ^2^CRCHUM, Montréal, Canada, ^3^Université de Montréal, Faculté des Arts et Sciences, Montréal, Canada, ^4^Université McGill, Département de Médecine, Montréal, Canada, ^5^Université de Montréal, Département de chirurgie, Montréal, Canada, ^6^CHU Sainte‐Justine, Montréal, Canada, ^7^Université de Montréal, Département de Médecine, Montréal, Canada

**Background**: We have previously reported that excessive levels of B‐cell activating factor (BAFF) are concomitant with increased frequencies of precursor‐like marginal zone (MZp) B‐cells in the blood of HIV‐infected progressors, despite antiretroviral therapy (ART). Recently, we have shown that MZp from healthy individuals possess strong Breg capacities, which are characterized by high expression levels of NR4A1, NR4A2, NR4A3 and CD83 among others, as well as Breg function involving CD83 signals. Our objective was to better understand the impact of HIV‐infection and excess BAFF on the Breg potential of MZp.

**Methods**: We have performed transcriptomic analyses by RNA‐seq of MZp sorted from the blood of HIV‐infected progressors from the Montreal Primary HIV Infection (PHI) cohort. Furthermore, the Breg profile and function of blood MZp B‐cells from HIV infected progressors, with or without HAART, were also assessed by flow‐cytometry and high content screening (HCS) analyses, respectively. In addition, the effects of high amounts of soluble recombinant BAFF on the Breg profile of MZp B‐cells from healthy donors was investigated *in vitro*.

**Results**: We report highly significant downregulation of NR4A1, NR4A2, NR4A3 and CD83 gene transcripts in blood MZp B‐cells from HIV‐infected progressors when compared to elite controllers (EC) and healthy individuals. Accordingly, NR4A1, NR4A3 and CD83 protein expression levels and Breg function are also downregulated in blood MZp B‐cells from HIV‐infected progressors and not restored by HAART. Importantly, we observe decreased expression levels of NR4A1, NR4A3, CD83 and IL‐10 by MZp B‐cells following treatment with excess BAFF, which significantly diminished their regulatory function.

**Conclusions**: Our data thus suggest that excess BAFF contributes to the reduced immune surveillance that precipitates the development of co‐morbidities such as atherosclerosis in the context of HIV and is likely to do so in other chronic inflammatory diseases where BAFF is found in excess.

### PESAA10 Cerebrospinal fluid cellular inflammatory gene expression profile in people with acute HIV‐1 infection

K. McGuckin Wuertz ^1,2^, V. Sharma^1,3^, A. Yates^1,3^, M. Creegan^1,3^, P. Chan^4^, C. Sacdalan^4^, N. Phanuphak^4^, K. Benjapornpong^4^, P. Prueksakaew^4^, S. Spudich^5^, S. Vasan^1,3^, D. Bolton^1,3^, on behalf of the RV254/304 Study Team

^1^Walter Reed Army Institute of Research, U.S. Military HIV Research Program, Silver Spring, United States, ^2^University of Washington, Center for Innate Immunity and Immune Diseases, Seattle, United States, ^3^Henry M. Jackson Foundation for the Advancement of Military Medicine, Bethesda, United States, ^4^Institute of HIV Research and Innovation (IHRI), SEARCH, Bangkok, Thailand, ^5^Yale University, Department of Neurology, New Haven, United States

**Background**: Despite effective antiretroviral therapy, neurocognitive dysfunction associated with HIV‐1 infection remains a significant source of morbidity. The mechanisms underlying the impairment are unclear, though likely involve widespread inflammation and early HIV‐1 replication within the central nervous system (CNS). We aimed to identify sources of CNS inflammation through transcriptional analysis of cerebrospinal fluid (CSF) cells from people living with HIV‐1 (PLWH) in acute infection.

**Methods**: PBMC and CSF were obtained from PLWH in the RV254 acute HIV‐1 cohort (Thailand), during untreated Fiebig stages III‐IV (n = 9, male, median age 23 ± 2.8) and from age and sex matched Thais without HIV‐1 (PWOH) (n = 2 (PBMC) and 5 (CSF)). CD4+ T cells and monocytes were FACS sorted in replicate (n = 4–8) for targeted 96‐gene expression profiling by multiplexed RT‐qPCR. Gene expression differences were assessed using Welch test on the means.

**Results**: Median HIV‐1 RNA values in PLWH were 6.1E6 copies/mL in plasma and 1.4E4 copies/mL in CSF. Median peripheral blood CD4+ T cell counts were 310 cells/uL, with a median CD4:CD8 ratio of 0.33. Multiple host genes involved in type I interferon regulation were upregulated in monocytes and CD4+ T cells in the CSF during acute HIV‐1 infection compared to PWOH, including *IFI44, IFIT1, RSAD2, STAT1, USP18* and *OAS* family members (*P < 0.05*). These genes were upregulated 2–8‐fold in CD4+ T cells and 5–40,000‐fold in monocytes. Cell cycle regulatory genes were also increased. Cytokines and chemokines involved in cellular recruitment and inflammation, *CCL2, CXCL9* and *TNF*, were only upregulated in monocytes (30–100,000‐fold). Similar results were observed in PBMC, though interestingly, the magnitude of the response in monocytes was more limited than that in CSF. These results indicate a robust antiviral inflammatory response in both CSF and peripheral blood.

**Conclusions**: Our findings provide unique insight into the cellular signaling pathways initiated within the CNS during the earliest stages of HIV‐1 infection. Greater understanding of these early events will inform therapeutic strategies designed to limit HIV‐1‐associated neurologic inflammation and disease.

### PESAA11 A longitudinal assessment of the impact of antiretroviral therapy and HIV‐1 associated inflammation on neurocognitive outcomes in perinatally infected (PHIV) children in South Africa

S. Naidoo ^1^, B. Laughton^1^, T. Maponga^1^, K. Veldsman^1^, M. Cotton^1^, R. Glashoff^1^

^1^Stellenbosch University, Cape Town, South Africa

**Background**: Early diagnosis and improved treatment options in paediatric HIV‐1 infections necessitates comprehensive characterisation of the neuropathology of HIV‐associated neurocognitive disorders (HAND) during viral suppression. Longitudinal studies on clinical and immunological biomarker associations with neurological outcomes in PHIV children are limited.

**Methods**: A dual longitudinal and cross‐sectional study design was implemented to investigate the impact of clinical, immunological, and virological parameters on neurodevelopmental outcomes in PHIV children from the Children with HIV Early antiRetroviral (CHER) randomised trial. Longitudinal neurocognitive assessments included the Griffiths Mental Development Scales (GMDS) administered at 11, 18, 30, 42 and 60 months of age and included assessments of locomotor, personal‐social, hearing, language and eye‐hand co‐ordination. The Beery‐Buktenica Development Test for Visual‐Motor Integration (Beery‐VMI) was implemented at 5, 7 and 9 years of age. Forty immunological plasma biomarkers were measured by Luminex^®^Multiplex Assays and ELISA. A sensitive qPCR adapted for HIV‐1 subtype C targeting the integrase gene was implemented for the measurement of total HIV‐1‐cell‐associated DNA (CAD).

**Results**: A total of 139 participants were assessed. We observed significant positive associations between pro‐inflammatory biomarkers including IL‐1β (*r* = 0.39; p = 0.01), sCD14 (*r* = 0.34; p = 0.03), sCD163 (*r* = 0.45; p < 0.01), IL‐18 (*r* = 0.36; p = 0.02) and LBP (*r* = 0.32; p = 0.04) and early Locomotor and General Griffiths scores. These biomarkers indicate innate immune activity of monocyte/macrophage activation and PAMP stimulation and may depict a neurological protective response. Negative associations with neurodevelopmental outcomes were observed for IL‐1RA (*r* = −0.50; p < 0.01), IL‐6 (*r* = −0.42; p < 0.01), MCP‐1 (*r* = −0.35; p = 0.02), MIP‐1α (*r* = −0.36; p = 0.02) and IFN‐α (*r* = −0.37; p = 0.02). Interleukin‐17F (*r* = −0.20; p = 0.02), IL‐12 (*r* = −0.19; p = 0.04), IL‐13 (*r* = −0.19; p = 0.03), MIP‐1α (*r* = −0.20; p = 0.02) and TNFβ (*r* = −0.18; p = 0.04) may serve as early predictors of late neurodevelopmental outcomes whereas IL‐13, IFN‐α, IL‐6 and TGF‐β_2_ can predict both early and late neurodevelopmental parameters. Early measures of HIV‐1‐CAD were significantly associated with Locomotor and General Griffiths scores. Early clinical parameters showed significant associations with both early and late neurodevelopmental outcomes. These include time‐to‐viral suppression (*r* = 0.34; p = 0.03), gestation (*r* = 0.33; p = 0.03), %CD8 at birth (*r* = 0.41; p = 0.03), CD8 count at birth (*r* = 0.40; p = 0.02), time‐to‐therapy initiation (*r* = −0.41; p < 0.01) and %CD4 at birth (*r* = 0.36; p = 0.02).

**Conclusions**: Neurocognitive outcomes can be predicted by early immunological, virological and clinical parameters. Early initiation of and continuous cART is important for mitigating excess inflammation and immune activation which significantly impacts the neuro‐immune relationship.

### PESAA12 No evidence that ongoing HIV‐specific immune responses contribute to persistent inflammation and immune activation in persons on long‐term suppressive ART

A. Ward ^1^, A. Thomas^2^, E. Stevenson^3^, S.‐H. Huang^3^, S. Keating^4^, R. Gandhi^5^, D. McMahon^6^, R. Bosch^7^, B. Macatangay^6^, J. Cyktor^6^, J. Eron^8^, J. Mellors^6^, R.B. Jones^1^, ACTG A5321 Team

^1^Weill Cornell Medicine, Medicine, New York City, United States, ^2^Boston University School of Medicine, Boston, United States, ^3^Weill Cornell Medicine, New York City, United States, ^4^GigaGen, Inc., San Francisco, United States, ^5^Massachusetts General Hospital, Boston, United States, ^6^University of Pittsburgh School of Medicine, Pittsburgh, United States, ^7^Harvard T.H. Chan School of Public Health, Boston, United States, ^8^University of North Carolina at Chapel Hill, Chapel Hill, United States

**Background**: People with HIV (PWH) have persistently elevated levels of inflammation and immune activation despite suppressive antiretroviral therapy (ART), with specific biomarkers showing associations with AIDS‐ and non‐AIDS‐defining morbidities and mortality. Because adaptive immune responses against HIV also persist in PWH on ART, and show evidence of ongoing antigenic stimulation, we hypothesized that they contribute to this clinically‐relevant inflammatory profile. We therefore investigated potential associations between HIV‐specific T‐cell and antibody responses with on‐ART inflammation and immune activation.

**Methods**: T‐cell responses (IFN‐γ ELISPOT) to each HIV gene product as well as to CMV‐pp65, along with HIV‐specific antibody concentrations, were measured in n = 101 virally suppressed participants from the AIDS Clinical Trials Group A5321 cohort at study entry (median 7 years on ART). HIV persistence measures including cell‐associated (CA)‐DNA, CA‐RNA, plasma HIV RNA by integrase single‐copy assay (iSCA), and intact proviral DNA assay (IPDA, in a subset of n = 33 participants) were also assessed at study entry. Plasma inflammatory biomarkers and T‐cell activation and cycling biomarkers were measured at a pre‐ART time point and at study entry.

**Results**: Magnitudes of HIV‐specific T‐cell responses, CMV‐pp65‐specific responses, and HIV antibody levels were not correlated with levels of inflammatory or immune activation biomarkers, including hs‐CRP, IL‐6, neopterin, sCD14, sCD163, or %CD38^+^HLA‐DR^+^ or %Ki67^+^ CD8^+^ and CD4^+^ cells – including after adjustment for pre‐ART biomarker level (all Spearman |r| < 0.20, p > 0.05). Magnitudes of T‐cell responses to HIV‐Pol were correlated with TNF‐α levels, but this was confounded by several factors including pre‐ART plasma viral load, CD4^+^ T‐cell count, and years on ART at A5321 entry. iSCA levels were correlated with CD8^+^ T‐cell activation (Spearman r = 0.25, p = 0.027), and defective (but not intact) HIV DNA levels with CD4^+^ T‐cell cycling (%Ki67^+^; r = 0.51, p = 0.003), but other HIV persistence parameters were not associated with these biomarkers. In statistical mediation analysis, relationships between HIV persistence parameters and inflammatory biomarkers were not influenced by HIV‐specific T‐cell responses or antibody levels.

**Conclusions**: HIV‐specific immune responses do not appear to contribute to the elevated inflammatory and immune activation profile associated with morbidity and mortality in PWH on long‐term suppressive ART.

### PESAA13 Soluble interferon receptor (sIFNAR2) inhibits HIV‐1 infection in macrophages through IFN‐β‐independent pathways

E. Calonge ^1^, F. Diez‐Fuertes^1^, I. Hurtado^2^, B. Oliver^3^, J. Alcamí^1^

^1^Institute of Health Carlos III. National Centre of Microbiology, AIDS immunopathology, Madrid, Spain, ^2^Biotech Research & Innovation Centre (BRIC), University of Copenhagen, Copenhagen, Denmark, ^3^Institute of Biomedical Investigation (IBIMA), Malaga, Spain

**Background**: The soluble IFNβ receptor (sIFNAR2) plays an immunomodulatory role in autoimmune diseases by reducing inflammation and tissue damage at the same levels as IFN‐β. Besides IFN‐β interferes with HIV replication mainly in monocyte‐derived macrophages (MDM). This study aims to evaluate the impact of sIFNAR2 on HIV‐1 infection in MDM and to compare the biochemical pathways induced by sIFNAR2 and IFN‐β.

**Methods**: MDM were infected with the YU2 HIV‐1 strain for 72h and infection was assessed by p24 levels in infection supernatants in the presence of IFNAR (30μgr/ml) or IFN‐β (20U). Soluble IFN binding protein B18 was used to exclude IFN‐β activation by SIFNAR2. Differential gene expression was measured by RNAseq of cells treated with IFNAR2 and IFN‐β (Illumina). Changes in protein expression were analyzed by quantitative phosphoproteome analysis (LC‐MS/MS and isobaric labeling with TMT)

**Results**: sIFNAR2 does not affect cell viability in culture. HIV‐1 infection of macrophages and PBLs was inhibited by at least 85% when sIFNAR was added to cultures at similar levels as IFN‐β. sIFNAR2 reduced the production of inflammatory cytokines (CXCL9,10,11) in MDM. sIFNAR2 effects were independent of IFN‐β activation since its action was not affected by treatment with the IFN inhibitor B18

**Figure:**
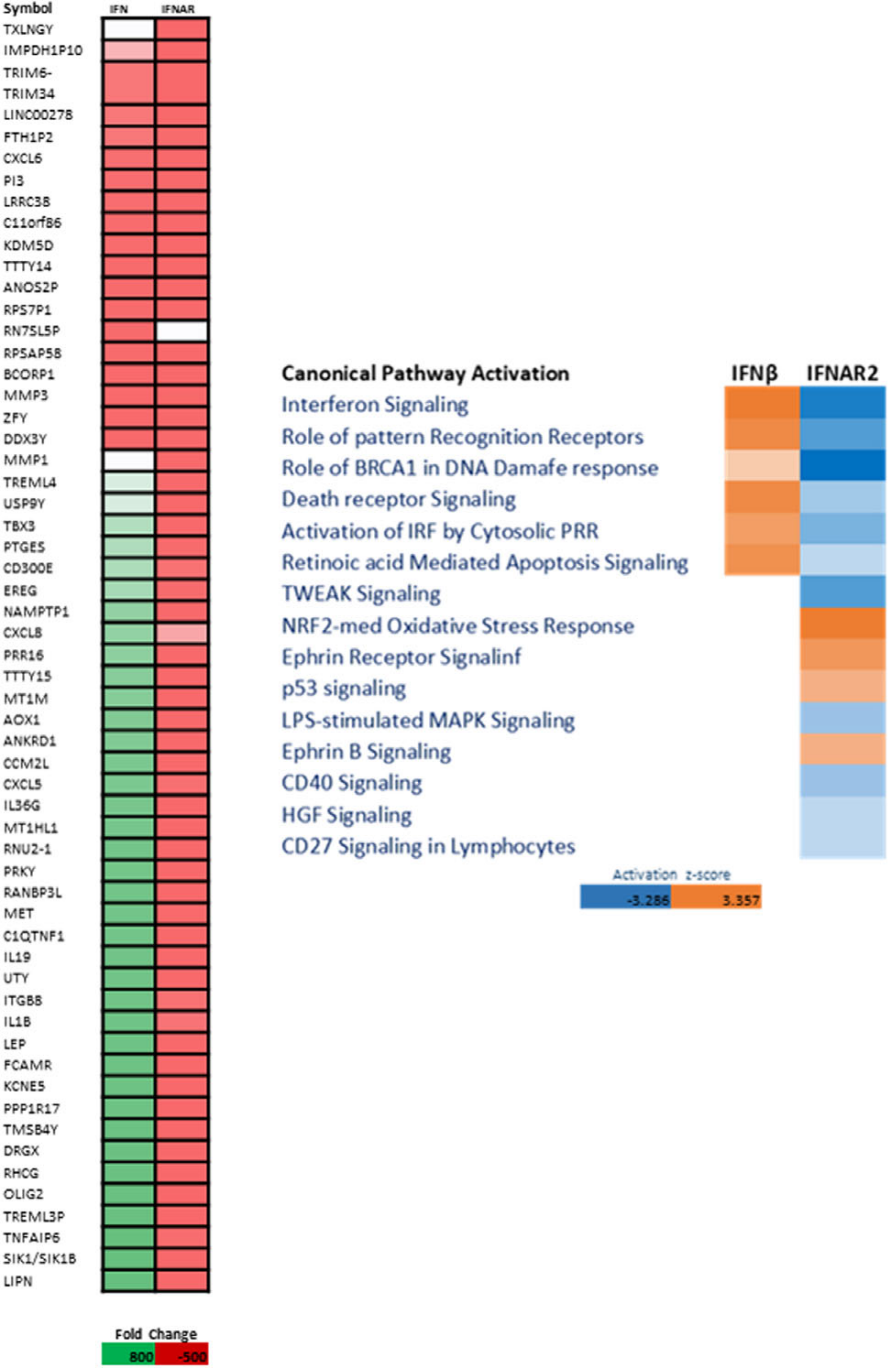
**Abstract PESAA13‐Figure 1**.

Differential gene expression measured by transcriptome sequencing shows that sIFNAR2 generates a functional pattern totally different from the profile shown by IFN‐β (Image) and remarkably, the Jak‐STAT pathway was not induced by sIFNAR2. Phosphoproteome analysis demonstrates activation of the IL37 pathway while classical IFNα/β signaling pathways, ISG15 antiviral mechanism or OAS antiviral response were not altered.

**Conclusions**: sIFNAR2 interferes with HIV‐1 replication in MDM and decreases the production of proinflammatory cytokines. Transcriptomic and proteomic analysis confirmed that the sIFNAR2 mechanism of action is different from the pathways elicited by INF‐β. The use of sIFNAR2 in the treatment of HIV‐1 infection deserves further consideration.

### PESAB01 Successful recruitment of Youth with untreated HIV infection in Adolescent Trials Network 147: Early treatment of Acute HIV Infection

T. Kerin ^1^, R. Cortado^1^, S. Paiola^1^, S.E. Abdalian^2^, D. Swendeman^3^, M. Ocasio^2^, R. Bolan^4^, B. Ark^1^, Y. Bryson^1^, K. Nielsen‐Saines^1^, ATN CARES Team

^1^David Geffen UCLA School of Medicine, Division of Infectious Diseases, Department of Pediatrics, Los Angeles, United States, ^2^Tulane University School of Medicine, Department of Pediatrics, New Orleans, United States, ^3^University of California, Los Angeles, Department of Psychiatry and Biobehavioral Sciences, Los Angeles, United States, ^4^Los Angeles LGBT Center, Los Angeles, United States

**Background**: Gay, bisexual, transgender, and African‐American adolescents are at elevated risk of acquiring HIV infection but diagnosis is often delayed. Early antiretroviral treatment (ART) of acute HIV infection can reduce viremia and viral reservoir burden, promoting long‐term HIV control. Adolescent Trials Network (ATN) 147 aimed to identify and recruit youth with acute/recent HIV infection for early ART. Baseline demographic and clinical data for the cohort are reviewed.

**Methods**: Treatment‐naïve, recently identified HIV+ youth, aged 12 to 24 years, from Los Angeles and New Orleans were recruited from community centers, clinics, social media, and a high‐risk seronegative cohort (n = 1727, ATN 149) using point‐of‐care assays. Acute HIV infection was determined by Fiebig staging. HIV RNA viral load (VL) and CD4 cell counts were assessed at enrollment.

**Figure:**
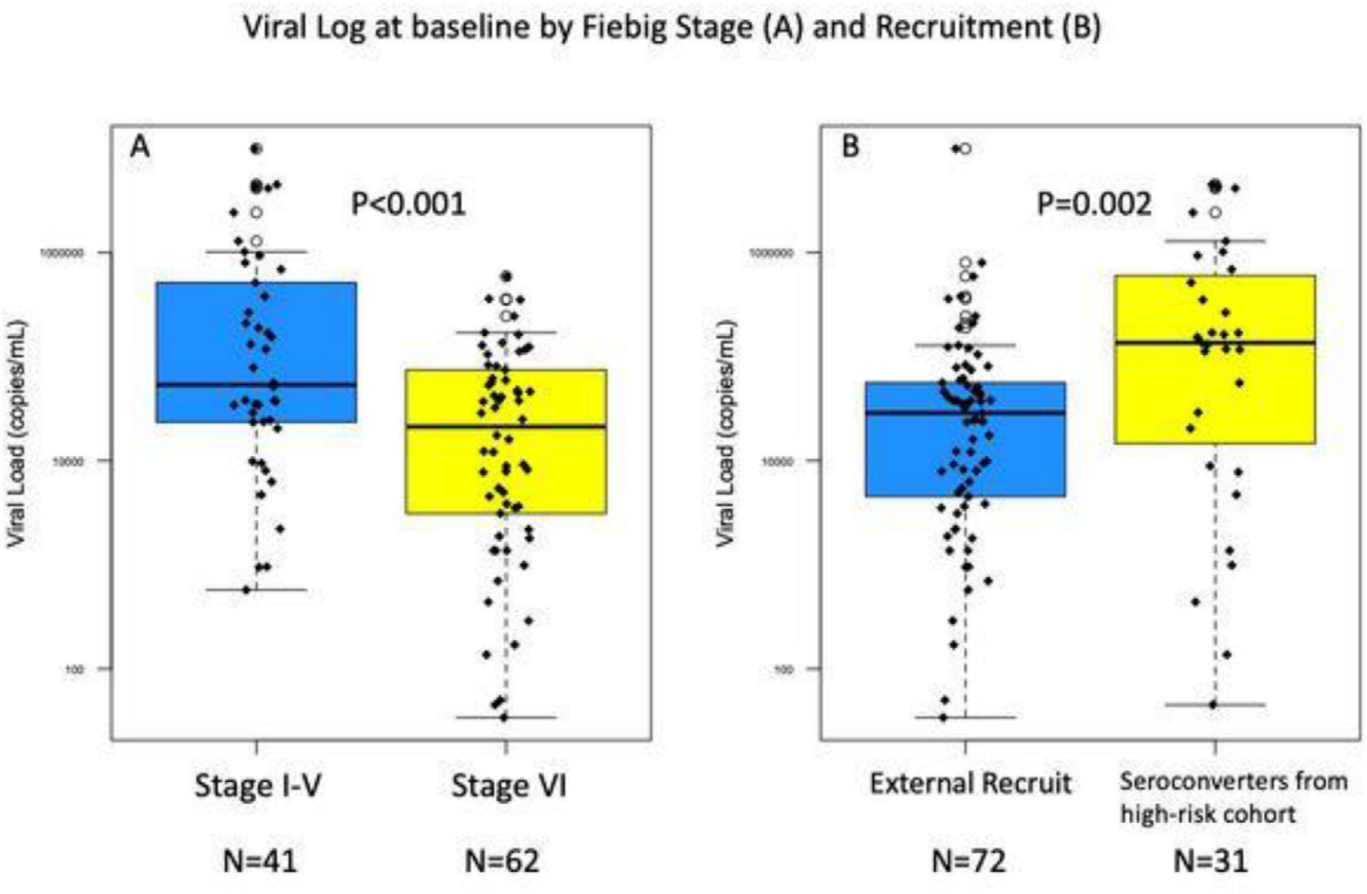
**Abstract PESAB01‐Figure 1**.

**Results**: Between July 2017‐July 2021, 103 newly diagnosed youth were enrolled and started ART within a week. Mean age was 20.8 years (sd: 2.4); 90.3 % of youth identified as cis‐male, 83.5% were single or in casual relationships, 71.8% were gay, bisexual, and other MSM, and 60.2% were Black. One‐fourth (24.3%) reported homelessness ever; 10.7% within the last 4 months. At enrollment, median VL was 37,313 copies/ml (IQR: 5849–126162) and median CD4 count 445.5 cells/mm3 (IQR: 357–613). 40% of youth reported acute retroviral symptoms prior to or at enrollment. Median VL differed significantly by Fiebig stage and recruitment source (Figure). Acutely‐infected, seroconverting youth had higher VL. STI co‐infections were present at enrollment in 63%, with syphilis most frequent (39%).

**Conclusions**: Despite challenges in identification and recruitment of youth with HIV infection to clinical studies, ATN 147 successfully enrolled youth at the time of HIV diagnosis. A high STI burden was present in recently HIV‐infected youth, likely facilitating HIV acquisition. Acute retroviral symptoms were reported by only 40%, demonstrating that broad universal HIV screening is needed for better identification of recent infection in youth.

### PESAB02 In‐hospital mortality among persons with HIV (PWH) in the US and Canada, 2005–2018

T. Davy‐Mendez ^1^, S. Napravnik^1^, J.J. Eron^1^, K.A. Gebo^2^, K.N. Althoff^2^, B.C. Hogan^2^, M.J. Silverberg^3^, M.A. Horberg^4^, J.N. Martin^5^, A.C. Justice^6^, M.J. Gill^7^, A. Nijhawan^8^, J.A. Colasanti^9^, M.Y. Karris^10^, J.E. Thorne^2^, C.S. Rabkin^11^, K.A. McGinnis^12^, J.M. Jacobson^13^, M. Loutfy^14^, R.D. Moore^2^, S.A. Berry^2^

 

**Figure:**
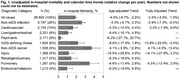
**Abstract PESAB02‐Figure 1**.

^1^UNC Chapel Hill, Chapel Hill, United States, ^2^Johns Hopkins University, Baltimore, United States, ^3^Kaiser Permanente Northern California, Oakland, United States, ^4^Kaiser Permanente Mid‐Atlantic States, Rockville, United States, ^5^University of California, San Francisco, United States, ^6^Yale University, New Haven, United States, ^7^Southern Alberta HIV Clinic, Calgary, Canada, ^8^University of Texas Southwestern Medical Center, Dallas, United States, ^9^Emory University, Atlanta, United States, ^10^University of California, San Diego, United States, ^11^National Cancer Institute, Bethesda, United States, ^12^Veterans Affairs Connecticut Healthcare System, West Haven, United States, ^13^Case Western Reserve University, Cleveland, United States, ^14^University of Toronto, Toronto, Canada

**Background**: In‐hospital mortality can reflect incidence of severe events, quality of inpatient and outpatient disease management, and delayed presentation to care for acute or chronic illness. Examining in‐hospital mortality among PWH can help identify potential areas for improvement in preventing disease progression in the context of increasing age and comorbidity burden.

**Methods**: For hospitalizations in 2005–2018 among PLWH in care in six cohorts of the NA‐ACCORD, we categorized ICD codes for the primary discharge diagnosis using modified Clinical Classifications Software (CCS). Using Modified Poisson regression, we estimated calendar time trends in the probability of in‐hospital death overall and by diagnostic category, adjusting for age, gender, race/ethnicity, HIV risk factor, CD4 count, HIV viral load (VL), and NA‐ACCORD cohort, all measured at hospitalization.

**Results**: We examined 26,600 hospitalizations among 9,076 PLWH who were 73% cisgender men, 38% White, 37% Black, and 19% with IDU risk factor. PLWH hospitalized in 2018 vs. 2005 were older (median 54 vs. 44 years), likelier to have VL < 400 copies/mL (83% vs. 48%), and had higher CD4 counts (median 469 vs. 267 cells/μL). Over the study period, unadjusted in‐hospital mortality was 1.9% (95% CI 1.7%‐2.1%) for all‐cause hospitalizations and ranged from 0.3% (0.1%‐0.6%) for psychiatric to 3.7% (2.9%‐4.8%) for non‐AIDS‐defining cancer hospitalizations (Fig. 1). For all‐cause hospitalizations, unadjusted in‐hospital mortality decreased from 2.3% (1.7%‐3.2%) in 2005 to 1.6% (1.1%‐2.4%) in 2018. The age‐adjusted relative change in in‐hospital mortality was −4.5% (−6.7% to −2.2%) per year for all‐cause hospitalizations. In fully adjusted analyses, the relative change per year was −3.3% (−5.5% to −1.0%) for all‐cause, −3.9% (−7.3% to −0.4%) for non‐AIDS infection, and −13.8% (−23.0% to −3.4%) for AIDS‐defining illness hospitalizations.

**Conclusions**: Among PLWH in care, in‐hospital mortality decreased over 2005–2018. Further research should investigate risk factors and potential prevention strategies for hospitalizations with higher mortality, e.g. for pulmonary conditions.

### PESAB03 High accuracy of an enzyme‐linked immunoasssay for detection of tenofovir alafenamide: implications for point‐of‐care antiretroviral adherence monitoring

M. Spinelli ^1^, W. Rodrigues^2^, G. Wang^2^, J. Chen^2^, K.A. Johnson^1^, D.V. Glidden^1^, M. Morrow^3^, H. Okochi^1^, P.L. Anderson^4^, M. Gandhi^1^

^1^University of California, San Francisco, San Francisco, United States, ^2^Abbott Rapid Diagnostics, Pomona, United States, ^3^Colorado School of Public Health, Aurora, United States, ^4^University of Colorado Anshutz Medical Campus, Aurora, United States

**Background**: We previously developed a urine point‐of‐care (POC) immunoassay to measure urine tenofovir (TFV) levels for patients on tenofovir disoproxil fumarate (TDF) as an objective adherence metric. Tenofovir alafenamide (TAF), a prodrug of TFV, is metabolized intracellularly and gives ∼80% lower urine TFV levels than TDF. Previous modelling demonstrated that 300 ng/mL of TFV is an optimal cut‐off for detecting non‐daily adherence to TAF. The goal of this study was to demonstrate accuracy of the enzyme‐linked immunoassay (ELISA) for TFV from TAF compared to liquid chromatography tandem mass spectrometry (LC‐MS/MS) at this cut‐off.

**Methods**: The TAF‐DBS study recruited HIV‐negative participants to take TAF/emtricitabine using directly‐observed‐therapy. The Point‐of‐Care Urine Monitoring of Adherence (PUMA) Study collected urine from people with HIV using TAF‐based ART. Ten urine samples were included as negative controls from healthy participants on no medications. A previously validated ELISA using the anti‐TFV antibody was recalibrated for concentration ranges appropriate for individuals taking TAF and levels compared to those from LC‐MS/MS.

**Results**: Overall, 131 samples from 94 participants were included. TAF‐DBS included 36 participants (17 cisgender women) providing 2 samples, median age 29 (range 18–41); PUMA included 48 participants providing one sample (6 cisgender women, 2 transgender women, 1 transgender man), median age 56 (range 26–73); Controls included 10 participants not on TAF (5 cisgender women), median age 32 (range 25–45). Of the 109 samples with TFV levels above the 300 ng/mL cut‐off by LC‐MS/MS, 107 were above 300 ng/mL via ELISA, with 98.2% sensitivity (95% confidence interval (CI): 93.5–99.8%). Of 21 samples with TFV levels below the cut‐off by LC/MS/MS, 20 were below via ELISA, with 95.2% specificity (95% CI: 76.2–99.9%). ELISA was 97.7% (95% CI: 93.4–99.5%) accurate compared to LC‐MS/MS (**Fig**.). The log‐transformed correlation coefficient between the two methods was 0.86 (p < 0.001).

**Conclusions**: As TAF uses expands for PrEP and ART, a POC urine assay for TAF adherence is needed. We demonstrate that an immunoassay can accurately measure TFV in urine above and below an appropriate cut‐off for TAF with high accuracy compared to LC‐MS/MS. The assay is now optimized to support development of a lateral flow POC adherence test for TAF.

### PESAB04 Comparison of Advanced HIV Disease identification using CD4 results from a semi‐quantitative CD4 point of care test and CD4 flow cytometry in Nigeria

N. Otubu ^1^, O. Abudiore^1^, M.‐M. Akanmu^1^, B. Levy‐Braide^1^, W. Eigege^1^, O. Sowale^1^, A. Inyang^1^, D. Rathakrishnan^2^, I. Amamilo^1^, J. Conroy^3^, F. Lufadeju^1^, C. Amole^3^, O. Wiwa^4^, P. Nwaokenneya^5^, C. Adesigbin^5^, A. Ikpeazu^5^, G. Olorunfemi^6^, R. Oladele^7^, O. Agbaji^8^, S. Akanmu^7^

^1^Clinton Health Access Initiative, HIV Access Program, Abuja, Nigeria, ^2^Clinton Health Access Initiative, HIV Access Program, Kigali, Rwanda, ^3^Clinton Health Access Initiative, HIV Access Program, Boston, United States, ^4^Clinton Health Access Initiative, Global Resources for Health, Abuja, Nigeria, ^5^Federal Ministry of Health, National AIDS and STIs Control Program, Abuja, Nigeria, ^6^University of Witwatersrand, Division of Epidemiology & Biostatistics, Johannesburg, South Africa, ^7^Lagos University Teaching Hospital, Lagos, Nigeria, ^8^Jos University Teaching Hospital, Jos, Nigeria

**Background**: CD4 testing is critical in identifying people living with HIV with Advanced HIV Disease (AHD) and is conducted at enrollment/re‐enrollment into care. Nigeria introduced VISITECT CD4 Advanced Disease rapid test (VISITECT), a semi‐quantitative point of care test pre‐qualified by WHO, to address gaps in CD4 coverage. Conducting VISITECT test requires time‐sensitive procedural steps and visual acuity for interpretation, and there was no experience with its use in Nigeria. To allay concerns of the impact of operational differences on the quality of VISITECT results in the Nigerian context, we compared results from VISITECT to CD4 flow cytometry, the current gold standard in‐country.

**Methods**: We recruited patients >10years old enrolling into HIV care across 4 states (Akwa‐Ibom, Anambra, Lagos, Rivers) implementing the AHD package of care between February and June 2021. Venous or capillary blood samples were collected at enrollment, and parallel CD4 tests were conducted via VISITECT and CD4 flow cytometry platforms (BD FACSPresto and Partec Cyflow). We determined how many results reported by healthcare workers (HCWs) as <200cells/mm^3^ (AHD), or ≥200cells/mm^3^ by CD4+ cell flow cytometry was correctly identified by VISITECT. The paired tests were tested for agreement using Cohen's Kappa test. STATA version 16 was used for analysis.

**Results**: 603 patients were recruited from 10 ART facilities. The prevalence of CD4 <200cells/mm^3^ was 47.6%, (95% CI: 43.6% ‐ 51.6%, 287/603) and 50.9% (95% CI: 46.9% ‐ 54.9%, 307/603) on the flow cytometry and VISITECT respectively. In all, 268 of 307 VISITECT results that were <200cells/mm^3^ were identified as correct by the gold standard, giving a positive predictive value of 87.3%. 277 of 296 VISITECT results ≥200cells/mm^3^ were identified as correct by the gold standard, with giving a negative predictive value of 93.6% (Kappa = 0.81, Agreement = 90.38%, P = <0.001).

**Conclusions**: The observed high agreement between VISITECT and flow cytometry results demonstrates that VISITECT can correctly identify patients with AHD and has the potential to improve access to CD4+ testing and linkage to care. The findings show that operational differences have minimal effect on the accuracy of VISITECT results at facilities and Nigeria can deploy the test across the country with minimal concerns.

### PESAB05 Circulating mir21 and mir125b in women living with human immunodeficiency virus: utility of biomarkers for monitoring cervical carcinogenesis

J. Okoye ^1^, C. Onyenekwe^2^, A. Ngokere^2^, O. Omotuyi^3^

^1^Faculty of Health Sciences and Technology, Nnamdi Azikiwe University, Nnewi Campus, Medical Laboratory Science, Nnewi, Nigeria, ^2^Nnamdi Azikiwe University, Medical Laboratory Science, Nnewi, Nigeria, ^3^Adekunle Ajasin University, Biochemistry, Akungba, Nigeria

**Background**: As of 2018, the prevalence of Human immunodeficiency virus (HIV), and cervical cancer (Ca) attributable to HIV was higher in Africa than in other continents. Identifying individuals at a high risk of developing Ca among immunocompromised persons, using less invasive techniques, remains a major challenge. The study evaluated HIV infection‐associated dysregulation of Ca‐linked oncomirs (miR‐21, miR‐146a, miR‐155, miR‐182, and miR‐200c) and tumor suppressors (miR‐let‐7b, miR‐125b, miR‐143, miR‐145, and p53 gene), in a bid to identify early indicators of genetic instability, and biomarkers for monitoring of high‐risk individuals.

**Methods**: This case‐control study included 173 women without abnormal Pap smear; confirmed HIV seropositive women (HIV+ = 103) and HIV seronegative women (HIV‐ = 70). Relative expressions of miRNAs and p53 gene in blood and cervical cells) were determined following RNA extraction, reverse transcriptase Polymerase Chain Reaction (PCR), and gel electrophoresis. T‐test was used to compare the data from HIV+ and HIV‐ women. Significance was set at*p*≤ 0.05.

**Results**: Similar pattern of miR‐21, miR‐146a, miR‐182, miR‐200c, miR‐125b, and miR‐145 expression was observed in both samples. Higher expressions of miR‐155 and p53 gene were observed in cervical cells of HIV+ women compared with HIV‐ women (p = 0.046, and 0.033, respectively) whereas lower expressions of miR‐155 and p53 gene were observed in the blood of HIV+ women compared with HIV‐ women (p = 0.539 and 0.049, respectively). In both blood and cervical cells, higher miR‐21 expression (p = 0.032 and 0.198, respectively) and lower miR‐125b expression (p = 0.050 and 0.004, respectively) were observed in HIV+ women compared with HIV‐women. In blood, a lower expression of miR‐146a was observed in HIV+ women compared with HIV‐ women (p = 0.036) whereas in cervical cells, lower expressions of miR‐182 and miR‐200c were observed in HIV+ women compared with HIV‐ women (p = 0.035 and 0.045, respectively). The higher expression of miR‐21, and lower expression of miR‐125b and p53 could be early indicators of genetic instability prior to epithelial transformation.

**Conclusions**: This study suggests that circulating high expression of miR‐21 and low expression of miR‐125b and p53 gene could be used in identifying individuals at risk of developing Cervical cancer, especially among immunocompromised patients.

### PESAB06 Cryptococcus qPCR assays: the future for routine mycology labs and clinical trials dealing with HIV‐associated cryptococcosis

T. Mbangiwa ^1,2^, A. Sturny‐Leclere^3^, K. Lechiile^2^, C. Kajanga^4^, T.B. Chammard^5^, D. Lawrence^6^, J.C. Hoving^1^, O. Lortholary^3^, F. Dromer^3^, M. Mosepele^2,7^, H. Mwandumba^4,8^, T. Harrison^9^, J.N. Jarvis^6^, A. Alanio^3^

^1^University of Capetown, Division of Immunology, Cape Town, South Africa, ^2^Botswana Harvard AIDS Institute Partnership, Research Lab, Gaborone, Botswana, ^3^Institut Pasteur, Molecular Mycology Unit and National Reference Centre for Invasive Mycoses, Paris, France, ^4^Kamuzu University of Health Science, Research, Blantyre, Malawi, ^5^Centre Hospitalier d'Ajaccio, Department of Infectious Diseases and Tropical Medicine, Ajaccio, France, ^6^London School of Hygiene and Tropical Medicine, Clinical Research, London, United Kingdom, ^7^University of Botswana, Department of Internal Medicine, Gaborone, Botswana, ^8^Liverpool School of Tropical Medicine, Liverpool, United Kingdom, ^9^St George's University of London, Centre for Global Health, Institute for Infection and Immunity, London, United Kingdom

**Background**: Routine laboratory testing for cryptococcal meningitis currently consists of Cryptococcal antigen (CrAg) testing in blood and cerebrospinal fluid (CSF), CSF India ink, and CSF fungal culture. Quantitative cryptococcal culture (QCC) is labor intensive and not feasible in most settings. We evaluated quantitative (qPCR) and reverse transcriptase qPCR (RT‐qPCR) assays to quantify cryptococcal load in CSF, plasma, and blood. We investigated the dynamics of fungal DNA and RNA detection during antifungal treatment.

**Methods**: We developed a qPCR assay that can differentiate serotypes A, D and B/C of *Cryptococcus neoformans* and *Cryptococcus gattii* based on the amplification of a unique nuclear Quorum sensing protein 1 (QSP1) and a multicopy 28S rRNA gene and evaluated the assays on 205 patients samples from the AMBITION‐cm trial in Botswana and Malawi (2018–2021). CSF, plasma and whole blood samples were stored per patient and were sampled at day 0 (baseline), day 7 and 14 for CSF and at day 1, 3 and 7 for plasma and whole blood post antifungal treatment initiation. A Roche LightCycler480 and Graph pad prism were used for data analysis.

**Results**: Using the *QSP1* qPCR, 138 (81.7%) were serotype A, 28 (16.6%) were serotype B/C and 3 (1.8%) were a mixed infection of serotype A and B/C. There was no amplification with 36 (17.6%) samples. QCC showed a good correlation with *QSP1* qPCR (slope = 0.797, R^2^ = 0.73) and with 28S rRNA qPCR (Slope = 0.771, R^2^ = 0.778) assays. The fungal load at D0 was significantly higher in patients who died at week 10 (w10) as compared to patients who survived post week 10 (p < 0.01). Detection of *Cryptococcus* DNA (28S rRNA qPCR) in plasma or whole blood within the first 24 hours of treatment was significantly associated with early mortality at w10 (p < 0.01). *QSP1* RT‐qPCR showed that detection of DNA was due to viable fungal cells as the quantification of *QSP1* whole nucleic acids was systematically higher (2 to 5‐fold) than that of DNA.

**Conclusions**: Quantification of *C. neoformans* and *C. gattii* load in CSF and plasma at D0 is useful in identifying patients at risk of death and may be a promising tool for monitoring treatment response in the future.

### PESAB07 Antenatal systemic inflammation and mortality of children born to mothers with HIV in rural Zimbabwe

C. Evans ^1,2^, B. Chasekwa^1^, K. Mutasa^1^, S. Rukobo^1^, M. Govha^1^, P. Mushayanembwa^1^, F.D. Majo^1^, N.V. Tavengwa^1^, J.H. Humphrey^1,3^, A.J. Prendergast^1,2,3^

^1^Zvitambo Institute for Maternal and Child Health Research, Harare, Zimbabwe, ^2^Queen Mary University of London, Blizard Institute, London, United Kingdom, ^3^Johns Hopkins Bloomberg School of Public Health, Department of International Health, Baltimore, United States

**Background**: Despite increasing availability of antiretroviral therapy (ART) and falling vertical transmission rates, children born to mothers living with HIV have higher mortality than children born to mothers without HIV. We tested the hypothesis that maternal systemic inflammation during pregnancy is associated with mortality of children born to mothers with HIV.

**Methods**: Women from the SHINE trial in rural Zimbabwe were recruited during pregnancy and infants were followed for 18 months. C‐reactive protein (CRP) and soluble CD14 (sCD14) were measured in plasma by ELISA at a median of 16 gestational weeks. Cox regression models were used to estimate hazard ratios (HR) for infant mortality. Covariates for adjusted models included maternal HIV viral load, CD4 count, cytomegalovirus co‐infection, antiretroviral therapy exposure, gestational age at blood sampling and randomised trial arm.

**Results**: Among 636 children born to mothers with HIV, 82% had ART exposure antenatally. The median maternal CRP was 4.72mg/L (interquartile range (IQR) 1.54, 11.85), and was higher in mothers of children who died by 18 months (9.18mg/L, IQR 5.28, 18.02) compared to mothers of children who survived (4.35mg/L, 1.50, 10.99). After adjusting for plausible confounders, risk of child mortality doubled for each log rise in maternal CRP (adjusted HR (aHR) 2.12, 95%CI 1.33, 3.40; *P* = 0.002). By contrast, maternal CRP was lower among 1744 mothers without HIV (2.67mg/L, IQR 1.04, 6.02) and was not associated with the risk of infant mortality (aHR 1.05, 95%CI 0.76, 1.46; *P* = 0.763). Maternal sCD14 was not associated with the risk of mortality in children born to mothers with HIV (aHR1.65, 95%CI 0.25, 10.80; *P* = 0.600) or without HIV (aHR1.62, 95%CI 0.26, 10.19; *P* = 0.606).

**Conclusions**: We show for the first time that that maternal systemic inflammation measured by CRP is independently associated with infant mortality in HIV‐affected mother‐child pairs. This finding has two major public health implications. First, CRP is cheap and simple to measure, meaning antenatal point‐of‐care CRP could be utilised to identify those most at risk of child mortality. Second, antenatal anti‐inflammatory interventions may be required to improve clinical outcomes of children born to mothers with HIV.

### PESAB08 Viral suppression among adults with HIV receiving dolutegravir‐based antiretroviral therapy and 3HP in Kampala, Uganda

L. Chaisson ^1^, F. Semitala^2^, D. Dowdy^3^, K. Aman^4^, S. Steinmetz^3^, D. Armstrong^5^, B. Opira^4^, M. Kamya^2^, P. Phillips^6^, C. Yoon^6^

 

**Figure:**
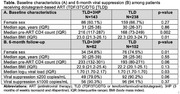
**Abstract PESAB08‐Table 1**.

^1^University of Illinois at Chicago, Medicine, Chicago, United States, ^2^Makerere University College of Health Sciences, Medicine, Kampala, Uganda, ^3^Johns Hopkins University, Epidemiology, Baltimore, United States, ^4^Infectious Diseases Research Collaboration, Kampala, Uganda, ^5^Johns Hopkins University, Pathology, Baltimore, United States, ^6^University of California San Francisco, Medicine, San Francisco, United States

**Background**: A Phase I/II study evaluating the pharmacokinetics of TB preventive therapy with 3HP (3 months weekly rifapentine/isoniazid) co‐administered with dolutegravir (DTG)‐based antiretroviral therapy (ART) found 3HP to be well‐tolerated and that 3HP could be given without dose adjustment of DTG, although DTG clearance was increased. We assessed 1) safety of DTG‐based ART and 3HP and 2) 6‐month viral suppression, in a routine setting.

**Methods**: TB SCRIPT is an ongoing Phase 3 randomized trial comparing TB screening with point‐of‐care C‐reactive protein versus symptoms among adults with CD4≤350 cells/μL initiating routine ART in Kampala, Uganda. TB screen‐negative participants without contraindications are referred for self‐administered 3HP two weeks following ART initiation. HIV viral load (VL) is measured at 6‐month follow‐up. Here, we evaluated 3HP discontinuation due to drug toxicity and 6‐month viral suppression among participants without prevalent TB at baseline who initiated once‐daily DTG‐based ART.

**Results**: From 11/2020–10/2021, 381 participants without TB initiated TDF/3TC/DTG (TLD), of whom 143 (37.5%) initiated 3HP. Median pre‐ART CD4 and BMI were higher for participants who initiated 3HP (Table). To date, 129/143 (90.2%) TLD+3HP participants completed 3HP; reasons for discontinuation included drug toxicity (rash, n = 1), pregnancy (n = 1), and active TB diagnosis (n = 3). Six‐month VL testing was completed by 164/381 (43.0%) participants, including 62/143 (43.4%) TLD+3HP participants and 102/238 (42.9%) TLD only participants. TLD+3HP participants had higher median VL than TLD only participants (p = 0.03). Furthermore, compared to TLD only participants, a higher proportion of TLD+3HP participants were viremic at >200 (21.0% [13/62] vs. 9.8% [10/102], p = 0.06) and >1000 copies/mL (9.7% [6/62] vs. 5.9% [6/102], p = 0.37), although the differences were not statistically significant.

**Conclusions**: Co‐administration of 3HP with DTG‐based ART was well‐tolerated. Viral suppression was high overall, but early evidence suggests that receipt of 3HP with once‐daily DTG‐based ART may be associated with a lower probability of achieving viral suppression within 6‐months.

### PESAB09 Pharmacokinetic and 48 week Efficacy of Once‐Daily vs Twice‐Daily Dolutegravir among patients with Human Immunodeficiency virus/Tuberculosis coinfection receiving rifampicin based tuberculosis therapy: A Randomized control trial

A. Avihingsanon ^1,2^, T. Ueaphongsukkit^3^, S. Gatechompol^1,2^, J. Sophonphan^1^, S.J. Kerr^1,4,5^, H.M.S. Lwin^1^, S. Ubolyam^1,2^, P. Chaiyahong^1^, N. Wacharachaisurapol^6^, P. Chaiyavilaskul^6^, Y.S. Cho^7^, J.G. Shin^7^, HIV‐NAT 254

^1^HIV‐NAT, Thai Red Cross AIDS Research Centre, Bangkok, Thailand, ^2^Center of Excellence in Tuberculosis, Division of pulmonary disease, Department of Medicine, Faculty of Medicine, Chulalongkorn University, Bangkok, Thailand, ^3^Division of Infectious Disease, Department of Medicine, Faculty of Medicine, Chulalongkorn University, Bangkok, Thailand, ^4^Biostatistics Excellence Centre, Faculty of Medicine, Chulalongkorn University, Bangkok, Thailand, ^5^The Kirby Institute, University of New South Wales, Sydney, Australia, ^6^Clinical Pharmacokinetics and Pharmacogenomics Research Unit and Center of Excellence for Pediatric Infectious Diseases and Vaccines, Faculty of Medicine, Chulalongkorn University, Bangkok, Thailand, ^7^Center for Personalized Precision Medicine of Tuberculosis, Inje University College of Medicine, Busan, Korea, The Republic of

**Background**: Concurrent use of Rifampicin (RIF) and dolutegravir (DTG) reduces DTG exposure, thus, DTG 50 mg twice‐daily is currently recommended. Food increases DTG concentrations in healthy volunteers by 33 – 66%. We investigated the effect of RIF on DTG exposure when dosed at 50 mg once daily with food. DTG 50mg once daily could be more convenient than 50 mg twice daily and generic FDC of TDF/3TC/DTG (TLD) could be easily used without extra 50 mg DTG.

**Methods**: Forty ARV‐naïve HIV‐positive participants with newly diagnosed TB receiving stable RIF‐based anti‐TB therapy in Bangkok, Thailand were randomized to initiate 50 mg once‐daily with food (study arm; TLD 1 pill/day) or DTG 50 mg twice‐daily (control arm; TLD 1 pill plus additional DTG). Intensive PK was scheduled at week 4. Blood samples were collected pre‐dose, 1, 2, 4, 6, 8, 12, and 24‐hour post‐dose (by study arm). HIV‐1 RNA, liver and renal function tests were monitored. DTG concentrations were determined by validated LC‐MS/MS. PK parameters were estimated by WinNonLin.

**Results**: The majority of the participants were male (87.5%); with median age of 32 years; and median body weight was 60.4 kg. Median baseline CD4 was 194 (IQR 46–238) cells/μL. Median baseline HIV‐1 RNA was 4.9 (IQR 3.6–5.6) log_10_copies/mL; 43% had HIV‐1 RNA >100,000 copies/mL. Table 1 shows that GMR (90%CI) trough concentration (C_trough_), maximal concentration (C_max_) and area under curve (AUC_0‐τ_) were not within the bioequivalence range of 0.8–1.25: [0.19 (0.1–0.35), 0.72 (0.49–1.06) and 0.42 (0.28–0.64)] respectively. In addition, 70% and 95% of study and control arm participants had DTG C_trough_ > 0.064 μg/mL. At week 48, 90% of the participants in the study arm (18/20) and control arm (18/20) had HIV‐RNA <40 copies/mL using ITT analysis. Premature study discontinuation occurred in 2 cases (1 in study arm: RIF‐induced cholestasis; 1 control arm: hypersensitivity reaction).

**Conclusions**: Although there was substantial reduction in DTG concentration when co‐administered with RIF, DTG once‐daily regimen with food had robust virological suppression at week 48. Larger study of once‐daily and twice‐daily DTG is underway to confirm this finding.

### PESAB10 Sitafloxacin Therapy for Mycoplasma Genitalium in Men who have Sex with Men

N. Ando ^1^, D. Mizushima^1^, M. Takano^1^, T. Aoki^1^, Y. Yanagawa^1^, K. Watanabe^1^, H. Uemura^1^, M. Mitobe^2^, K. Kobayashi^2^, H. Kubota^2^, H. Miyake^2^, T. Shinkai^2^, K. Sadamasu^2^, H. Gatanaga^1^, S. Oka^1^

^1^National Center for Global Health and Medicine, AIDS Clinical Center, Shinjuku‐ku, Japan, ^2^Tokyo Metropolitan Institute of Public Health, Laboratory, Shinjuku‐ku, Japan

**Background**: *Mycoplasma genitalium* infection has been recognized as an alarming STI in recent years. Reportedly, 89.6% and 68.3% of the strains detected in Japan carried mutations associated with macrolide and quinolone respectively. Due to these high rates of macrolide‐resistance strains, sitafloxacin monotherapy is used as the first choice for treating *M. genitalium* infections in Japan. In this study, we aimed to assess the efficacy of sitafloxacin monotherapy for rectal and urogenital *M. genitalium* infection.

**Methods**: Patients diagnosed with *M. genitalium* infections in National Center for Global Health and Medicine between 2019 and 2021 were treated with sitafloxacin 200 mg for 7 days. A rectal swabs and/or urine sample were collected for assessing quinolone‐ and macrolide‐resistance associated mutations (*parC*, *gyrA* and *23S‐rRNA*) before treatment. Test of cure was recommended at approximately four weeks post‐treatment.

**Results**: Among 114 patients included in this study, the mean age was 34 year‐old, all were MSM, and 49.0% were HIV‐positive. *M. genitalium* was detected in 91 rectal samples and 24 urine samples were observed. Among the strains diagnosed with *M. genitalium*, 70.3%(78/111) were successfully analyzed for *parC* mutations, 59.5%(66/111) for *gyrA* mutations and 78.4%(87/111) for *23S‐rRNA* mutations. Microbiological cure rate of whole strains was 88.6%(101/114). That of the strains carrying *S83I* was 80.0%(44/55). Among them, the rate of the strains carrying *S83I* without *gyrA* mutations was 90.3%(28/31). The cure rate of the wild type strains was 100% (15/15). There was no significant difference in cure rate by anatomical site.

**Conclusions**: The efficacy of sitafloxacin therapy for wild type *M. genitalium* infections was very high. However, the cure rate for the strains carrying both *parC* and *gyrA* mutations was limited. Resistance profile of both *parC* and *gyrA* is important to predict clinical course. According to the result of this study, we can treat rectal MG infections in the same way as urogenital infections.

**Table:** 

Abstract PESAB10‐Table 1.						
*Par* Cmutation (AAA)	Cure rate for each *parC* mutated MG infection	*GyrA* mutation (AAA)	Cure rate for combined parC and *gyrA* mutated MG infection	Cure rate for rectal MG infections	Cure rate for urogenital infections	Cure rate for concurrent rectal and urogenital
**G248T(S83I)**	80% (35/44, 95CI 64.7–90.2)	**G285T(M95I)**	77.8% (7/9, 95CI 40.0–97.2)	71.4% (5/7, 95CI 29.0–96.3)	100% (2/2, 95CI 15.8–100)	
		**G277T(G93C)**	0% (0/2, 95CI 0–84.2)	0% (0/2, 95CI 0–84.2)		
		**G295A(D99N)**	0% (0/1, 95CI 0–97.5)		0% (0/1, 95CI 0–97.5)	
		**G285C(M95I)&G295A(D99N)**	0% (0/1, 95CI 0–97.5)	0% (0/1, 95CI 0–97.5)		
		**Wild type**	90.3% (28/31, 95CI 74.2–98.0)	87.5% (21/24, 95CI 67.6–97.3)	100% (6/6, 95CI 54.1–100)	100% (1/1, 95CI 2.5–100)
**Wild type**	100% (15/15, 95CI 78.2–100)	**Wild type**	100% (15/15, 95CI 78.2–100)	100% (14/14, 95CI 76.8–100)	100% (1/1, 95CI 2.5–100)	
**Others**	95% (51/55, 95CI 83.1–99.4)	**Others**	95% (51/55, 95CI 83.1–99.4)	96.6% (39/42, 95CI 82.2–99.9)	90.9% (12/13 95CI 58.7–99.8)	
**Total**	88.6% (101/114, 95CI 81.3–93.8)		88.6% (101/114, 95CI 81.3–93.8)	87.8% (79/90, 95CI 79.2–93.7)	91.3% (21/23, 95CI 72.0–98.9)	100% (1/1, 95CI 2.5–100)

AAA: amino acid substitution, MG: Mycoplasma genitalium

### PESAB11 The acceptability of the AMBITION treatment regimen for HIV‐associated cryptococcal meningitis: Findings from a qualitative study of patients and providers in Botswana and Uganda

D.S. Lawrence ^1,2^, A. Ssali^3,4^, N. Moshashane^2^, G. Nabaggala^3^, L. Maphane^2^, T.S. Harrison^5,6,7^, D. Meya^8^, J.N. Jarvis^1,2^, J. Seeley^3,4^

^1^London School of Hygiene and Tropical Medicine, Department of Clinical Research, London, United Kingdom, ^2^Botswana Harvard AIDS Institute Partnership, Gaborone, Botswana, ^3^MRC/UVRI & LSHTM Uganda Research Institute, Social Aspects of Health Programme, Entebbe, Uganda, ^4^London School of Hygiene and Tropical Medicine, Department of Global Health and Development, London, United Kingdom, ^5^St George's University London, Institute of Infection and Immunity, London, United Kingdom, ^6^St George's University Hospitals NHS Foundation Trust, Clinical Academic Group in Infection and Immunity, London, United Kingdom, ^7^University of Exeter, MRC Centre for Medical Mycology, Exeter, United Kingdom, ^8^Makerere University, Infectious Diseases Institute, Kampala, Uganda

**Background**: HIV‐associated cryptococcal meningitis remains a significant contributor to AIDS‐related mortality. The AMBITION trial found a single, high‐dose of intravenous liposomal amphotericin (AmBisome) given alongside 14‐days of oral flucytosine and fluconazole non‐inferior in terms of all‐cause mortality when compared to 7‐days of intravenous amphotericin B deoxycholate and flucytosine followed by 7‐days of fluconazole. The AmBisome regimen was associated with significantly fewer adverse events. We explored the acceptability of the AmBisome regimen.

**Methods**: We embedded a qualitative study within the AMBITION sites in Gaborone, Botswana and Kampala, Uganda. We conducted in‐depth interviews with trial participants, surrogate decision makers, and researchers and combined these with direct observations. Interviews were transcribed and translated and data underwent thematic analysis.

**Results**: We interviewed 38 trial participants, 20 surrogate decision makers and 31 researchers. Participant understanding of the intricacies of the treatment regimens was limited however there was a broad preference for the AmBisome regimen due to the single intravenous doses and fewer side effects, with some in the control arm stating that they would have preferred the single dose. The single AmBisome dose took roughly 30 minutes to reconstitute compared to 5–10 minutes for each daily amphotericin B deoxycholate dose. The AmBisome regimen was associated with fewer episodes of amphotericin related rigors, a reduced need for intravenous hydration, fewer cases of thrombophlebitis and a reduction in the number of intravenous cannulae required. The reduced toxicity profile resulted in less intensive monitoring and management of participants in the AmBisome arm. A particular challenge was accessing blood transfusions which were needed more often in control arm participants who has significantly higher rates of anaemia. A challenge of the AmBisome arm was the extended duration of oral flucytosine which was given six hourly and involved participants taking a dose in the night.

**Conclusions**: Participants, surrogate decision makers and researchers found the AmBisome arm to be highly acceptable, being simpler to administer despite the initial time investment required. The single dose was well tolerated and associated with less toxicity and resultant management and monitoring. Widespread implementation of this regimen would reduce the clinical workload of caring for patients with HIV‐associated cryptococcal meningitis.

### PESAB12 Efficacy of cefixime for the treatment of *Neisseria gonorrhoeae* infection at three anatomic sites: a systematic review and meta‐analysis

K.J. Yang ^1^, N. Kojima^2^, J. Klausner^1^

^1^Keck School of Medicine of the University of Southern California, Los Angeles, United States, ^2^UCLA David Geffen School of Medicine, Department of Internal Medicine, Los Angeles, United States

**Background**: *Neisseria gonorrhoeae* infection is the most common co‐infection among patients with HIV. Additionally, rectal gonorrhea increases the risk of HIV infection acquisition. *Neisseria gonorrhoeae* is also highly prone to developing antibiotic resistance; thus, identification of effective antibiotics for treatment is important. We conducted a systematic review and meta‐analysis to describe the efficacy of the cephalosporin cefixime in treating gonorrhea at different anatomic sites.

**Methods**: We searched PubMed using the query “(“Gonorrhea”) AND (“Cefixime”).” Reports published between January 1, 1980, and December 7, 2022, were included. We excluded studies that were not in English, not accessible, non‐human, did not specify the cefixime dose/frequency, or case reports/series. Of the included reports, we abstracted treatment success rates and cefixime dosage/frequency. One investigator reviewed each article. The relevance and assessment of bias and cohort characteristics for each article was determined by two investigators. We performed a meta‐analysis on a minimum of 3 studies to determine the overall success of cefixime in treating urogenital, rectal, and pharyngeal gonorrhea. Via OpenMeta[Analyst] software (Brown University, Rhode Island), we then calculated 95% confidence intervals using a binary random effects model.

**Results**: Of the 347 studies returned by PubMed, 15 met our inclusion criteria.

Of patients who received a 400 mg single dose of cefixime, 756 of 777 patients with urogenital infections (estimate: 98.0%; CI: 97.0%‐99.0%), 107 of 112 patients with rectal infections (estimate: 95.9%; CI: 92.3%‐99.4%), and 183 of 218 patients with pharyngeal infections (estimate: 86.0%; CI: 76.9%‐95.0%) were cured.

Of patients who received a 800 mg single dose of cefixime, 224 of 228 patients with urogenital infections (estimate: 98.7%; CI: 97.3%‐100%) and 21 of 26 patients with pharyngeal infections (estimate: 81.3%; CI: 66.3%‐96.2%) were cured. There were fewer than 3 studies of rectal infections treated with 800 mg.

**Conclusions**: Cefixime was found to be an effective treatment for gonorrhea. At both single 400 mg and 800 mg doses, cefixime is most effective at treating urogenital infections and least effective at treating pharyngeal infections.

### PESAB13 Cumulative HIV‐1 viremia is associated with multimorbidity among U.S. women with HIV

Z.P. Morton ^1^, C.C. Mehta^2^, T. Wang^3^, F.J. Palella, Jr.^4^, S. Naggie^5,6^, E.T. Golub^7^, K. Anastos^8^, A.L. French^9^, S. Kassaye^10^, T.N. Taylor^11^, M.A. Fischl^12^, A.A. Adimora^13,14^, M.‐C. Kempf^15^, P.C. Tien^16^, I. Ofotokun^2,17^, A.N. Sheth^2,17^, L.F. Collins^2,17^

 

**Figure:**
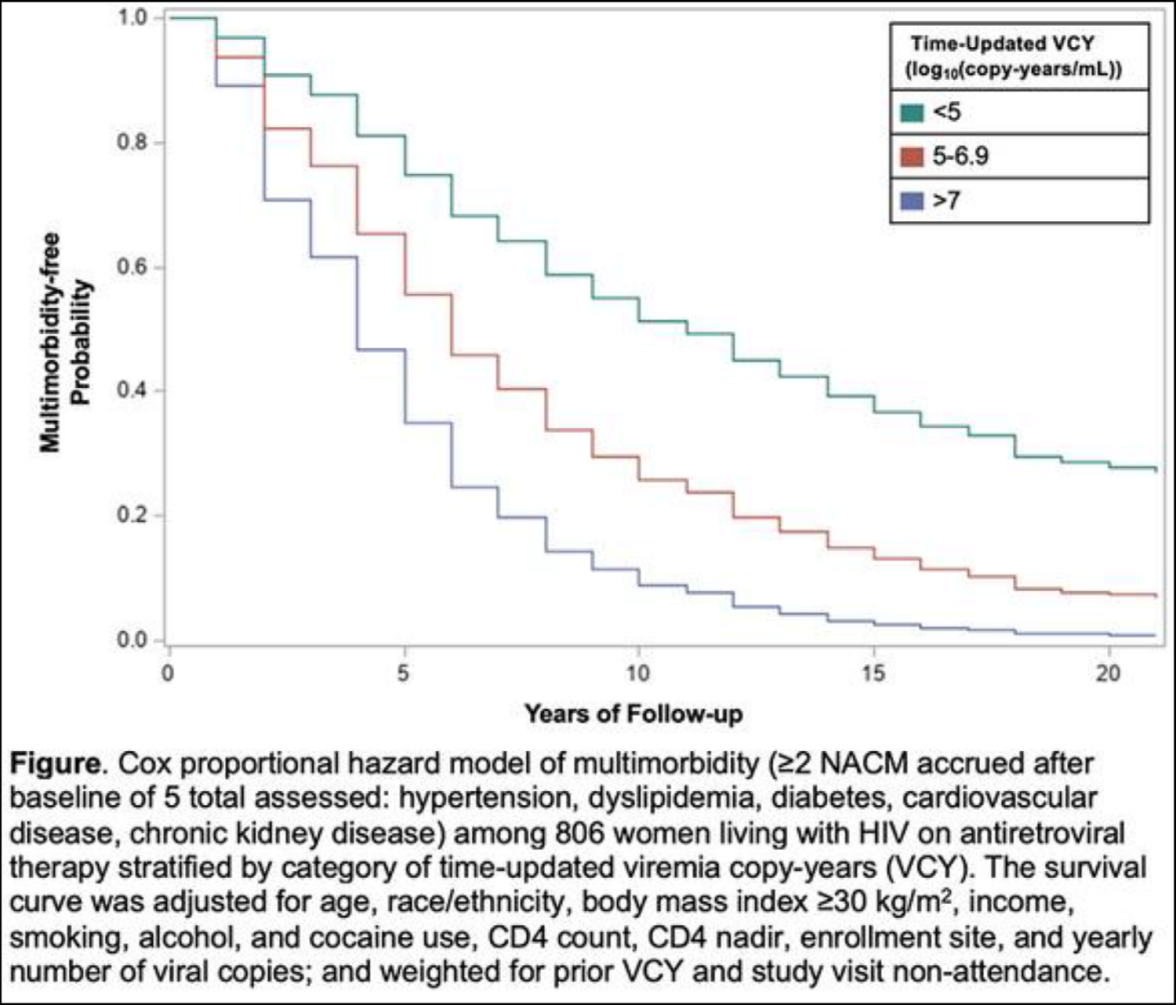
**Abstract PESAB13‐Figure 1**.

^1^Emory University School of Medicine, Atlanta, United States, ^2^Emory University School of Medicine, Division of Infectious Diseases, Atlanta, United States, ^3^Emory University Rollins School of Public Health, Atlanta, United States, ^4^Northwestern University Feinberg School of Medicine, Division of Infectious Diseases, Chicago, United States, ^5^Duke Clinical Research Institute, Durham, United States, ^6^Duke University School of Medicine, Durham, United States, ^7^Johns Hopkins Bloomberg School of Public Health, Department of Epidemiology, Baltimore, United States, ^8^Albert Einstein College of Medicine, Department of Medicine, Bronx, United States, ^9^CORE Center, Stroger Hospital of Cook County, Division of Infectious Diseases, Chicago, United States, ^10^Georgetown University Medical Center, Washington D.C., United States, ^11^SUNY Downstate Health Sciences University, Brooklyn, United States, ^12^University of Miami Miller School of Medicine, Division of Infectious Diseases, Miami, United States, ^13^University of North Carolina at Chapel Hill School of Medicine, Chapel Hill, United States, ^14^University of North Carolina Gillings School of Global Public Health, Chapel Hill, United States, ^15^University of Alabama at Birmingham Schools of Nursing, Public Health and Medicine, Birmingham, United States, ^16^University of California, San Francisco, Division of Infectious Diseases, Department of Medicine, San Francisco, United States, ^17^Grady Healthcare System, Infectious Diseases Program, Atlanta, United States

**Background**: Ongoing HIV‐1 replication despite antiretroviral therapy (ART) use may contribute to higher burden of aging‐related non‐AIDS comorbidities (NACM) among women living with HIV (WLWH) versus women without HIV. We evaluated effects of cumulative HIV‐1 viremia copy‐years (VCY) on NACM among WLWH.

**Methods**: We included WLWH in the Women's Interagency HIV Study through 9/13/2019 with ≥2 HIV‐1 RNA viral loads (VL) <200 copies/mL within a two‐year‐period (baseline) following self‐reported ART use. Primary outcome was multimorbidity (≥2 NACM accrued of 5 assessed: hypertension, dyslipidemia, diabetes, cardiovascular disease, kidney disease); presence of any NACM at baseline was exclusionary. VCY measures were calculated using the trapezoidal rule as area‐under‐the‐VL‐curve. A Cox proportional hazard model with time‐dependent covariates was fit to estimate the association of time‐updated cumulative VCY and multimorbidity, after adjusting for age, race/ethnicity, body mass index ≥30 kg/m^2^, income, smoking, alcohol, and cocaine use, CD4 count, CD4 nadir, enrollment site, yearly number of viral copies.

**Results**: 806 WLWH contributed 6,892 women‐years, with median 12 (Q1‐Q3 7–23) VL measured per participant on a median interval of 182 (Q1‐Q3 167–197) days. Baseline characteristics were median age 39 years, 56% Black, 36% reported smoking, and median CD4 count of 534 cells/mm^3^. Median time‐updated cumulative VCY was 5.4 (Q1‐Q3 4.7–6.9) log_10_ copy‐years/mL. Of 211 (26%) WLWH who developed multimorbidity, 324 (40%) had hypertension, 193 (24%) dyslipidemia, 69 (9%) diabetes, 66 (8%) cardiovascular and 44 (5%) chronic kidney disease. Compared with WLWH who had time‐updated cumulative VCY <5 log_10_, multimorbidity was associated with an adjusted hazard ratio of 2.03 (95% CI 1.29–3.20) and 3.63 (95% CI 2.04–6.44) for those with VCY 5–6.9 and ≥7 log_10_ copy‐years/mL, respectively (overall *p* < 0.0001) (Figure).

**Conclusions**: Among women on ART, time‐updated cumulative VCY was associated with multimorbidity and hence may be a prognostically useful biomarker to assess risk for aging‐related NACM in this population.

### PESAC01 Prevalence and individual and community‐level risk factors of advanced HIV disease among people living with HIV from nine African countries

K. Ganesan ^1^, W.M. El‐Sadr^1^, A. Low^1^

^1^Columbia Univeristy, Epidemiology, New York, United States

**Background**: People living with HIV (PLWH) with advanced HIV disease (AHD) (CD4 cell count <200 cells/mm^3^) are at higher risk of opportunistic infections, non‐AIDS defining comorbidities, and death. We used Population‐based HIV Impact Assessment (PHIA) survey data from a random sample of the population in Cameroon, Eswatini, Ethiopia, Lesotho, Malawi, Tanzania, Uganda, Zambia, and Zimbabwe to examine prevalence of AHD and identify individual and community‐level correlates of AHD among PLWH aware of their status (PLWHA).

**Methods**: Between 2015–2017, data from interviews and home‐based HIV testing were collected. Blood samples were analyzed for HIV RNA, detectable antiretrovirals, and CD4+ cell counts. PLWH were considered aware of their status based on self‐report or if antiretrovirals were detectable. Community‐level variables were created at each enumeration area (EA)‐level. Logistic regression using weighted data and clustered analysis to account for cross‐country and EA variation was used to determine individual and community‐level factors associated with AHD among PLWHA aged 15–59 years.

**Results**: Of 14,329 PLWHA, 11.6% (95% CI: 10.9%‐12.4%) had AHD. AHD prevalence ranged from 6.59% (95% CI: 5.59%‐7.76%) in Eswatini to 15.3% (95% CI: 13.7%‐17.0%) in Zimbabwe. By sex, 17.4% (95% CI: 15.8%‐19.0%) of men and 8.63% of women (95% CI: 7.91%–9.41%) had AHD. In multivariable analysis, higher odds of AHD was associated with male PLWHA, those aged 25–54 years, reporting individual‐level stigmatizing behavior, not having sexual intercourse in the last year, not being on antiretroviral therapy (ART), and residing in communities where there was denial of health services due to HIV status (Figure 1).

**Figure:**
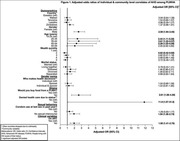
**Abstract PESAC01‐Figure 1**.

**Conclusions**: AHD among PLWHA remains a common challenge despite increased access to ART. Interventions are needed to enhance early diagnosis and sustained care, particularly among older men. Stigma at the community and health facility‐level hinders access and engagement in care, suggesting the need to implement and scale‐up community and health‐systems focused HIV stigma interventions.

### PESAC02 High transmitted drug resistance in Brazil: unprecedented levels of INSTI resistance

D.B. Caldeira^1^, T.R.C. Vergara^2^, E.L.B.d. Santos^3^, C.A.F. Lopes^4^, C.R.B. Alves^5^, M.M. Caseiro^6^, M.S. Treistman^2^, M.D.G. Sousa^2^, J.C. Fernandes^7^, E. Sprinz^8^, J. Galinskas^1^, M.T.d.O. Mota^1^, M. Schechter^1^, L.M.R. Janini^1^, R. Sobhie Diaz ^1^

^1^Univerisdade Federal de São Paulo (UNIFESP), São Paulo, Brazil, ^2^ONCOHIV Serviços Médicos Especializados, Rio de Janeiro, Brazil, ^3^Secretaria de Saúde do Distrito Federal (SES DF)/CLIDIP, Brasília, Brazil, ^4^Hospital Universitário João de Barros Barreto, Belém, Brazil, ^5^Universidade Federal da Bahia (UFBA), Salvador, Brazil, ^6^Secretaria municipal de saúde de Santos /Seção Centro de referência em AIDS, Santos, Brazil, ^7^Centro de referência em doenças infecciosas, Itajaí, Brazil, ^8^Universidade Federal do Rio Grande do Sul (UFRS), Porto Alegre, Brazil

**Background**: As of September 2021, 775.805 individuals were on antiretroviral therapy (ART) in Brazil. Until January 2017, the only Integrase Strand Transfer Inhibitor (INSTI) available in Brazil was Raltegravir, mainly used for salvage therapy when resistance to protease inhibitors (PIs) was detected. In January 2017, dolutegravir was introduced for first‐line treatment. We evaluate the national prevalence of transmitted drug resistance (TDR) mutations in treatment‐naïve patients initiating ART.

**Methods**: The HIV Threshold Survey methodology was utilized. From September 2020 to February 2022, subjects were selected from seven highly populated cities representative of all Brazilian macro‐regions: Belem (North), Salvador (Northeast), Brasilia (Central), Rio de Janeiro and Santos (Southeast), and Itajai and Porto Alegre (South). Dried Blood Spots were collected on SS903 cards and transported to a central laboratory for genotyping of the reverse transcriptase, protease, and integrase of the *pol* gene.

**Results**: Of 244 individuals analyzed, 56 (22.95%) harbored TDR mutations. The mean CD4+T‐cell count was 425 cells/μL, and the mean viral load was 312.923 copies/mL. The regional TDR prevalence was 16.66% in the Northeast, 22.05% in the Southeast, 14.89% in the Central region, 35.71% in the North, and 24% in the South. Overall, TDR prevalence was 4.09% for nucleoside reverse transcriptase inhibitors, 11.47% for non‐nucleoside reverse transcriptase inhibitors, 2.87% for PIs, and. 2.05% for INSTI (Table). TDR to two and three antiretroviral classes was 0,82% and 0.41%, respectively. The prevalence of Non‐B subtypes was 32.79%, being 20.49% of C, 4.92% of F, and 7.38% of recombinants.

**Conclusions**: We identified variable TDR prevalence, ranging from intermediate to more frequently high levels. Previous use of Raltegravir in salvage therapy may have contributed to this unprecedented level of INSTI TDR.

**Table:** 

Abstract PESAC02‐Table 1. Prevalence of resistant associated mutations in percentages for each antiretroviral class.							
NRTI (%)		NNRTI (%)		PI (%)		INSTI^*^ (%)	
M184V, I	2.70	K103N	6,48	V82A	0.53	T97A	1.79
V75I	0.54	V106I	2,70	M46I	0.53	E138K	0.90
K219Q	0.54	V179D, M203I	4,86	V32I	0.53	G140S	0.45
A62V	1.08	V108I	1.08	V43T	0.53	G140R	0.45
L210W	0.54	M230I	1.08	N88D	0.53		
		A98G	0.54	L10F	0.53		
		E138A, K, G, Q	7,02	L33F	0.53		
		G190A	0.54				
		Y181C	0.54				

^*^Substitution N155K detected in one patient infected with clade B virus

### PESAC03 “MENTORS MOTHERS”! The Link between community and health facilities for PMTCT Programs in Tanzania

N. Makyao ^1^, A. Maro^1^, A. Nyirenda^1^, A. Kosia^2^

^1^Amref Health Africa, Prevention, Dar es salaam, Tanzania, The United Republic of, ^2^Cristian Social Services Council, Prevention, Dar es Salaam, Tanzania, The United Republic of

**Background**: In the implementation of GF 2020–2023, the target is to eliminate new HIV infection among HIV exposed infants from 8% in 2018 to below 5% in 2023 and to increase access to ART among HIV infected children from 60% in 2016 to 95% by 2023. The use of the community approach through mentor mothers is a new strategy to ensure early identification and linkage, retention throughout the cascade of care. The intervention is implemented in a total of 10 regions, 57 councils, and 330 health facilities.

**Description**: Selection of MM was done with program team in collaboration with facility PMTCT supervisors. Criteria's for selection were developed including having good adherence to clinics, willingness to disclose or have disclosed status and influential who can perform the task. MM were responsible for giving one‐on‐one support to HIV‐infected pregnant/postpartum women; encourage enrollment, adherence and retention in HIV care, perform tracing for women who miss clinic visits; and educate on PMTCT and health‐related topics. Sites positivity rates of more than 5% with HIV pregnant or breastfeeding attending PMTCT were identified. Each facility selected a total of 4 MM and one facility supervisor who attended a special 10 days training using National curriculum.

**Lessons learned**: Findings indicated that majority of participants, their male partners, and health care providers accepted the intervention. Our community‐based mentor mothers have contributed to filling the critical gap in the quality and continuum of care for mothers living with HIV. One year of implementation indicated an increase for early ANC booking, couple counseling and HIV testing as well as early infant diagnosis. We reached 49,171 women who tested for HIV. 540 were HIV+ which is 1.1%, their male partners reached were 30,845 and 395 were HIV positive which is 1.3% yield.

**Conclusions/Next steps**: We learnt that there was high acceptance of use of mentor mother intervention as strategy for linkage between facility and community. This is in agreement with previous studies which have found that task shifting to lay health workers who are also HIV patients is acceptable and results in increased early identification, linkage, adherence and retention to HIV care.

### PESAC04 HIV incidence and death among orphaned and non‐orphaned children and adolescents living in family‐based settings in Western Kenya

D. Apedaile ^1^, A. DeLong^2^, E. Sang^3^, D. Ayuku^4^, L. Atwoli^4,5^, P. Braitstein^1,3,4^

^1^University of Toronto, Dalla Lana School of Public Health, Toronto, Canada, ^2^Brown University, School of Public Health, Providence, United States, ^3^Academic Model Providing Access to Healthcare, Eldoret, Kenya, ^4^Moi University, School of Medicine, Eldoret, Kenya, ^5^Aga Khan University, Medical College, East Africa, Nairobi, Kenya

**Background**: In Kenya, most of the 1.8 million orphaned children live with members of their extended family. These households can face severe poverty and have difficulty meeting the needs of all the children in their care. While children have reported experiencing discrimination at home and in their communities based on their status as orphans, little is known about the health outcomes of orphans compared to non‐orphaned children. The objective of this analysis was to compare HIV incidence and death among orphaned children to non‐orphaned children.

**Methods**: A random sample was taken of 300 households caring for at least one orphaned child in Uasin Gishu County, Kenya. From each household, all orphaned and non‐orphaned children were enrolled in a prospective cohort study between May 2010 and April 2013. Annual follow‐up visits, including HIV testing, were conducted until 2019. Mixed‐effects survival analyses with a random slope to account for clustering within households were used to estimate the association between orphan status, defined by the number of parents the child had lost (none, 1, or 2), and HIV incidence, death, and combined HIV incidence or death.

**Results**: The study enrolled 1488 children, including 487 double orphans, 743 single orphans, and 258 non‐orphans. At enrollment, 52% of participants were female, the median age was 10.4 years old, and 23 participants were HIV‐positive (0 non‐orphans, 15 single orphans, 8 double orphans). Over the course of the study, 16 participants died (0 non‐orphans, 8 single orphans, 8 double orphans) and 11 acquired HIV (0 non‐orphans, 6 single orphans, 5 double orphans). Among participants who were HIV‐negative at enrollment, having one more deceased or missing parent was strongly associated with incident HIV or death (AHR 2.34 per parent, 95% CI: 1.17–4.71) after accounting for gender and age at enrollment.

**Conclusions**: Within similar households, orphans experience a higher risk of HIV and death than non‐orphans. This indicates that both orphans themselves and the families caring for them need additional support to prevent serious health outcomes.

### PESAC05 Stigma in youth with HIV is associated with depression, school dropout and adult clinic attendance

C. Mugo ^1^, M. Kumar^2^, J. Badia^3^, J. Kibugi^3^, K. Agot^3^, J. Dyer^4^, P. Kohler^4^, D. Wamalwa^5^, G. John‐Stewart^4^

^1^Kenyatta National Hospital, Research and Programs, Nairobi, Kenya, ^2^University of Nairobi, Psychiatry, Nairobi, Kenya, ^3^Impact Research and Development Organization, Kisumu, Kenya, ^4^University of Washington, Global Health, Seattle, United States, ^5^University of Nairobi, Pediatrics and Child Health, Nairobi, Kenya

**Background**: Few studies have looked at the risk factors and outcomes of HIV stigma among youth with HIV (YWHIV) in sub‐Saharan Africa.

**Methods**: YWHIV in nine Western Kenya facilities were enrolled in an observational cohort in 2019–2020. Participants completed an enrollment survey assessing their sociodemographics, HIV history, adherence, depression (PHQ‐9), exposure to physical, emotional and sexual violence and HIV stigma (10‐item scale by Wright 2007). Correlates of overall HIV stigma (overall score: 10–50) were assessed using generalized linear models. We report mean differences (MD) adjusted for age and gender, and bootstrapped 95% confidence intervals (95%CI) and p values accounting for clustering by facility.

**Results**: Of 1,011 YWHIV (aged 15–24), 59% were 15–19 years old, 69% were female, 22% had dropped out of school, and 59% received care in adolescent/youth clinics. Twenty‐one percent had missed ≥2 days’ medication, and 64% reported ever having sex. Eighteen percent had mild depressive symptoms, while 3% had moderate/severe symptoms; 28% experienced physical violence, 18% emotional violence and 7% sexual violence. The median (interquartile range) overall stigma score was 25 (21–29).

Compared to YWHIV receiving care in adolescent/youth clinics, those in general/adult HIV clinics had higher stigma scores (MD: 1.58 [95%CI: 0.13–3.04], p = 0.042). YWHIV who had dropped out of school had higher stigma scores compared to those in school (2.56 [0.81–4.31], p = 0.016), as was those ever in a sexual relationship (2.59 [1.43–3.73], p = 0.004). YWHIV who had missed ≥2 days’ medication had higher stigma scores compared to those fully adherent (2.16 [0.92–3.41], p = 0.011). Those with mild, and moderate/severe depression had higher stigma scores (3.37 [2.57–4.17], p < 0.001 and 7.08 [1.32–12.84], p = 0.028) compared to those with no depression. YWHIV who experienced any violence before the last 6 months, and within the last 6 months had higher stigma scores compared to those with no experience of violence (2.51 [−0.12–5.13], p = 0.058 and 2.91 [1.38–4.44], p = 0.002).

**Conclusions**: This study identified intrapersonal, interpersonal and structural factors to consider when developing HIV stigma interventions for YWHIV. This includes exposure to violence, sexual relationships, and service points in facilities. Possible outcomes targeted by these interventions may include depression, adherence and keeping YWHIV in school.

### PESAC06 Alcohol use among people who inject drugs living with HIV in Kenya is associated with needle sharing, more sex partners, and poor engagement in care

N. DesLauriers ^1^, N. Ludwig‐Barron^2,3^, B. Sambai^4^, L. Mbogo^4^, D. Bukusi^4^, E. Juma^4^, B. Chohan^2,5^, R. Bosire^5^, H. Kingston^6^, E. Wilkinson^7,8^, E. Gitau^9^, W. Sinkele^9^, S. Masyuko^2,10^, J. Herbeck^2^, B. Guthrie^2,3^, C. Farquhar^1,2,3^, A. Monroe‐Wise^2^

^1^University of Washington, Department of Medicine, Seattle, United States, ^2^University of Washington, Department of Global Health, Seattle, United States, ^3^University of Washington, Department of Epidemiology, Seattle, United States, ^4^Kenyatta National Hospital, HIV Testing and Counseling and HIV Prevention, Nairobi, Kenya, ^5^Kenya Medical Research Institute, Centre for Clinical Research, Nairobi, Kenya, ^6^University of Washington, Institute for Public Health Genetics, Seattle, United States, ^7^University of KwaZulu‐Natal, KwaZulu‐Natal Research and Innovation Sequencing Platform, Durban, South Africa, ^8^Stellenbosch University, Centre for Epidemic Response and Innovation (CERI), School of Data Science and Computational Thinking, Stellenbosch, South Africa, ^9^Support for Addictions Prevention and Treatment in Africa, Nairobi, Kenya, ^10^Kenya Ministry of Health, Nairobi, Kenya

**Background**: People who inject drugs living with HIV (PWID‐LWH) are a high‐risk population in the HIV epidemic in Kenya. It is essential to elucidate factors contributing to risk behavior and suboptimal care among PWID‐LWH to decrease HIV transmission. Within an assisted partner services implementation science study, we evaluated the association between alcohol use and HIV risk behaviors and care outcomes among a cohort of PWID‐LWH in Kenya.

**Methods**: Participants were recruited through 8 sites in Nairobi and coastal Kenya with the following eligibility criteria: age ≥18 years, injected drugs within the past 30 days, HIV‐positive, and willing to provide partner contact information. Participants reported type and frequency of alcoholic beverages consumed in the past 30 days and use was stratified into heavy (>14 drinks/week for men, >7 for women), moderate (any lesser amount), or none based on CDC/NIAAA definitions. Logistic regression was used to calculate odds ratios for factors associated with alcohol use.

**Results**: Of 870 PWID‐LWH, 527 (60.6%) reported no alcohol use, 210 (24.1%) moderate use and 133 (15.3%) heavy use. 49.8% were female, 7.8% reported needle sharing within the past 30 days, 18.6% had more than 3 new sex partners in the past 3 months, 13.7% were not enrolled in HIV care, and 18.5% were not on ART. Of 510 PWID‐LWH on ART from whom viral load was obtained, 28.2% were virologically unsuppressed. In bivariate analysis, heavy alcohol use was associated with needle sharing (OR = 3.87, 95%CI: 2.13–7.01). In multivariate analysis (table), heavy alcohol use was independently associated with more new sex partners. Moderate and heavy alcohol use were independently associated with not being on ART and non‐enrollment in care. Among those on ART, there was no association between alcohol use and viral non‐suppression.

**Table:** 

**Abstract PESAC06‐Table 1**.					
Variable	>3 new sex partners in past 3 months	Needle sharing	Not enrolled in HIV care	Not on ART	Unsuppressed Viral load^‡^
	Adjusted odds ratio (95% confidence interval)^*^				
No alcohol use	1 (ref)	1 (ref)	1 (ref)	1 (ref)	1 (ref)
No alcohol use	1 (ref)	1 (ref)	1 (ref)	1 (ref)	1 (ref)
Moderate alcohol use	1.47 (0.89, 2.43)	0.98 (0.47, 2.00)	**2.23 (1.33, 3.72)**	**1.88 (1.18, 2.97)**	0.82 (0.48, 1.37)
Heavy alcohol use	**1.76 (1.01, 3.07)**	1.89 (0.91, 3.89)	**2.04 (1.10, 3.76)**	**1.99 (1.15, 3.43)**	0.63 (0.30–1.27)

^*^Adjusted odds ratios from logistic regression models controlling for age, sex, region, history of sex work, khat use (associated with alcohol use), and number of substances used in the past 30 days. Needle sharing, non‐enrollment in care, ART, and viral load aORs also control for enrollment in a methadone clinic.

^‡^Among participants on ART

**Conclusions**: Alcohol use is prevalent among PWID‐LWH in Kenya and a potential risk factor for suboptimal care and HIV risk behavior. Our findings reinforce the importance of addressing alcohol use among this group through expanded clinical and public health interventions.

### PESAC07 A Quantitative Intersectionality Analysis of HIV/STI Testing, Positivity and Current PrEP Use among Transgender People in Washington State, USA

D. Tordoff ^1^, C. Khosropour^1^, S. Glick^2^, L. Barbee^2^, A. Duerr^3^, The Seattle Trans and Non‐binary Sexual Health (STARS) Advisory Board

^1^University of Washington, Epidemiology, Seattle, United States, ^2^University of Washington, School of Medicine, Seattle, United States, ^3^Fred Hutchinson Cancer Research Center, Seattle, United States

**Background**: Transgender people, especially transgender women of color, are disproportionately impacted by HIV/STIs. We applied quantitative intersectional methods to identify HIV/STI‐related disparities among multiply marginalized transgender populations.

**Methods**: We pooled data from five 2019–2021 cross‐sectional data sources in Washington State. We considered three self‐reported outcomes: past year HIV/STI testing, current PrEP use, and a composite measure of HIV/STI positivity (past year bacterial STI diagnosis and/or HIV positive). We defined groups by gender and race/ethnicity and calculated the risk difference (RD) for each outcome (reference = White transgender men). For transgender women and non‐binary participants of color, we used Poisson regression to estimate two surrogate measures of additive interaction–the attributable proportion (AP) and ratio of the observed to expected relative joint effects (RJE)–that measure the excess risk attributable to the intersection of gender and race/ethnicity.

**Results**: Our analysis included 1648 transgender participants (70.4% White, 10.7% Latinx, 5.8% Black). HIV/STI positivity was statistically significantly higher among transgender women and non‐binary people assigned male at birth (AMAB) compared to White transgender men (RD, Table). From the AP, we estimated that 50% and 67% of the excess HIV/STI prevalence among Black and Latinx transgender women, respectively, was attributable to the intersection of gender and race/ethnicity. Although the RJE was not statistically significant for any outcomes/groups, it suggests that, compared to White transgender men, HIV/STI prevalence is 101%and 206%higher among Black and Latinx transgender women, respectively, than what would be expected if gender and race/ethnicity alone were sufficient to explain it. Similar patterns were observed for HIV/STI testing and PrEP use.

**Table:** 

Abstract PESAC07‐Table 1. Quantitative Intersectionality Analysis for Self‐reported HIV/STI Positivity among Transgender and Non‐binary People in Washington State, 2019–2021						
Gender Identity	Race/Ethnicity	N	HIV/STI Positive (%)	Risk DifferenceRD (95% CI)	Attributable ProportionAP (95% CI)	Ratio of observed to expected relative joint effectsRJE (95% CI)
Trans Men	White [ref]	242	4.1	ref	ref	ref
Trans Women	White	212	7.5	−0.06 (−0.16, 0.03)	NA	NA
	Black	37	5.4	**0.43 (0.25, 0.60)**	**0.50 (0.07, 0.93)**	2.01 (0.27, 3.76)
	Latinx	32	46.9	**0.21 (0.10, 0.33)**	**0.67 (0.27, 1.08)**	3.06 (−0.70, 6.83)
Non‐binary People Assigned Male at Birth	White	165	21.2	**0.39 (0.11, 0.67)**	NA	NA
	Black	22	31.8	**0.27 (0.02, 0.52)**	−0.18 (−2.68, 2.31)	0.84 (0.27, 1.42)
	Latinx	12	30.8	**0.29 (0.15, 0.43)**	0.29 (−0.83, 1.40)	1.40 (0.13, 2.68)

*The null value for RD and AP is 0, and the null value for RJE is 1*.

**Conclusions**: Our findings highlight the heterogeneity in HIV/STI positivity, testing, and PrEP use within the transgender population. We observed that a large proportion of the increased HIV/STI risk among transgender women of color is explained by intersection of gender and race/ethnicity.

### PESAC08 Sexual behavior and HIV prevalence among Venezuelans immigrants in Peru: a study among men who have sex with men (MSM) and transgender women (TGW) screened for pre‐exposure prophylaxis (PrEP)

O.A. Elorreaga ^1^, K.A. Konda^1^, J. Guanira^1^, G.M. Calvo^1^, S.K. Vargas^1^, A. Borquez^2^, C.F. Caceres^1^, for the ImPrEP Study Group

^1^Universidad Peruana Cayetano Heredia, Centro de Investigación Interdisciplinaria en Sexualidad, SIDA y Sociedad, Lima, Peru, ^2^University of California San Diego, California, United States

**Background**: The Venezuelan economic crisis has generated an unprecedented migratory wave. According to World Bank estimates, Peru is now home to 1.2 million Venezuelan immigrants. However, little is known about the epidemiological profile of MSM and TGW Venezuelan immigrants, who may be at risk of acquiring or transmitting HIV due in part to challenging social and economic circumstances.

**Table:** 

**Abstract PESAC08‐Table 1**.				
Characteristics of the n = 205 participants who tested HIV positive during PrEP screening		Peruvians (N = 131) N (column %)	Venezuelans (N = 74) N (column %)	Chi‐2 p‐value
Age (years)	▪ <25 ▪ 25 or more	70 (53.4%) 61 (46.6%)	21 (28.4%) 53 (71.6%)	0.001
Schooling	▪ <=Complete secondary ▪ Completed tertiary	16 (12.2%) 38 (29.0%)	1 (1.4%) 38 (51.4%)	0.001
Sex w/o condom	▪ Insertive condomless anal sex	75 (57.3%)	63 (85.1%)	<0.001
Sex with HIV+ partner w/o condom	▪ None ▪ Yes, with partner of unknown HIV status ▪ Yes, with HIV+ partner	16 (12.2%) 106 (80.9%) 9 (6.9%)	12 (16.2%) 48 (64.9%) 14 (18.9%)	0.016
Sex work	▪ Non sex workers ▪ MSM sex workers ▪ TGW sex workers	100 (76.3%) 20 (15.3%) 11 (8.4%)	57 (77.0%) 17 (23.0%) 0 (0.0%)	0.021

**Methods**: Between May 2018 and February 2021, participants answered a structured questionnaire on sexual behaviors and were tested for STI/HIV to determine eligibility for PrEP services as part of ImPrEP, a demonstration project implemented in six Peruvian cities. All participants were MSM/TGW, 18+ years‐old, and provided informed consent. We compared social and behavioral characteristics among participants who screened HIV‐positive and were therefore ineligible for PrEP enrolment. Differences were assessed using Chi‐square tests.

**Results**: Among the 2526 participants screened for ImPrEP, we identified 623 (24.7%) immigrants, of whom 590 (94.7%) were Venezuelan. Among all immigrants, 74 (11.9%) were HIV positive, of which all (74/74, 100%) were Venezuelan. Comparing the HIV prevalence among participants screened for ImPrEP, Venezuelans (12.5%, 95%CI 10.1–15.5) had twice the HIV prevalence of Peruvians (6.8%, 95%CI 5.7–7.9). Comparing Peruvian and Venezuelans, among all HIV positive participants (N = 205), Venezuelans were older and more educated (p = 0.001), reported more insertive condomless anal sex (p < 0.001), condomless sex with HIV+ partners (p = 0.016), and more MSM who engaged in sex work (23.0% vs 15.3%, p = 0.021), which may increase their risk for HIV transmission.

**Conclusions**: The Venezuelan migrant crisis demands adequate health policies and services in host countries. Our findings suggest that HIV prevalence among Venezuelan MSM/TGW is high, and engagement in sex work and HIV‐associated risk behaviors are common in this vulnerable group. Free and easily accessible HIV testing and treatment must be made available. Likewise, HIV prevention opportunities, including PrEP, should be made available within the public health program.

### PESAC09 Cervical cancer screening among women living with HIV: a systematic analysis of population‐based surveys in sub‐Saharan Africa

L. Yang ^1^, M.‐C. Boily^2^, M. Rönn^3^, S. Delany‐Moretlwe^4^, D. Obiri‐Yeboah^5^, I. Morhason‐Bello^6^, N. Meda^7^, T. Gauthier^8^, P. Mayaud^9^, M. Pickles^2^, M. Brisson^10^, M. Maheu‐Giroux^1^

^1^McGill University, Montreal, Canada, ^2^Imperial College London, London, United Kingdom, ^3^Harvard University, Cambridge, United States, ^4^University of the Witwatersrand, Johannesburg, South Africa, ^5^University of Cape Coast, Cape Coast, Ghana, ^6^University of Ibadan, Ibadan, Nigeria, ^7^Centre Muraz, Bobo‐Dioulasso, Burkina Faso, ^8^Ministère de la Santé, Ouagadougou, Burkina Faso, ^9^London School of Hygiene & Tropical Medicine, London, United Kingdom, ^10^Université Laval, Québec, Canada

**Background**: Cervical cancer (CC) is the leading cause of cancer death in women in sub‐Saharan Africa (SSA) where 1 in 5 CC cases can be attributable to HIV. Screening for CC is highly effective in reducing disease burden and is essential to the global CC elimination strategy, but estimates of screening uptake in SSA are limited. We aim to estimate CC screening coverage for women in SSA by HIV status.

**Methods**: We collected all publicly available nationally representative surveys with CC screening information from SSA. We estimated screening coverage using a Bayesian multilevel model (levels: survey, country, region), accounting for the recall period, age groups, and time trends (nested within regions). The effect of living with HIV on screening was modeled using country‐level random slopes. For surveys without HIV biomarkers, standardization with the UNAIDS HIV prevalence estimates was used. Post‐stratification was used to aggregate estimates among women aged 25–49y for countries with HIV biomarker data.

**Results**: We pooled 46 surveys across 25 countries (253,285 respondents) between 2000–2019, of which 12 countries (132,629 respondents) had HIV biomarker data. In 2019, the proportion of women ever screened for CC varied by region and was higher for WLHIV than women without HIV in Central/Western Africa (WLHIV = 7% [95% Credible Intervals (CrI): 3–15%] vs. 4% [95%CrI: 2–8%]) and Eastern Africa (WLHIV = 21% [95%CrI: 15–27%] vs. 9% [95%CrI: 6–12%]), but similar in Southern Africa (WLHIV = 48% [95%CrI: 33–65%] vs. 50% [95%CrI: 34–65%]). Odds ratios (age‐adjusted) for CC screening among WLHIV compared to women without HIV ranged from 0.93 (95%CrI: 0.84–1.05) in South Africa to 2.80 (95%CrI: 2.28–3.50) in Tanzania.

**Conclusions**: WLHIV have equal or greater odds of being screened for CC in comparison to those not living with HIV. However, overall coverage remains low in SSA. These findings are aligned with current practises as many CC screening initiatives have been introduced alongside existing HIV care structures. As funding and priorities shift away from HIV, these screening rates among WLHIV may not be sustained. Greater investment and improved approaches to screening are essential, particularly for WLHIV, to improve coverage, and reduce disparities in CC burden between women living and not living with HIV.

### PESAC10 National and Regional Rates of Hospitalizations and In‐Hospital Mortality for Opportunistic Infections for People with HIV in the United States, 2012–2018

C. Bielick ^1^, A. Strumpf^1^, K. McManus^1^

^1^University of Virginia, Infectious Diseases and International Health, Charlottesville, United States

**Background**: Contemporary rates of hospitalizations for Human Immunodeficiency Virus (HIV)‐related Opportunistic Infections (OIs) in the United States (US) are unknown. We aimed to quantify the national and regional OI hospitalization rate and OI hospitalizations’ in‐hospital mortality rate for people with HIV (PWH) in the US for 2012–2018.

**Methods**: All hospitalizations in the Agency for Healthcare Research and Quality's National Inpatient Sample that included an HIV diagnosis and an OI diagnosis (as defined by the Centers for Disease Control and Prevention's (CDC) Stage‐3‐Defining OIs) during 2012–2018 were included. For the hospitalizations, we analyzed demographics (age, sex, race) and the in‐hospital mortality rate. Using the number of PWH from CDC data, we estimated national and regional OI hospitalization rates per 100,000 PWH‐years with 95% Poisson confidence intervals.

**Results**: There were 145,710 total weighted hospitalizations for PWH with an OI diagnosis. PWH with OI hospitalizations were predominately male (68.7%), 45–55 years‐old (31.7%) and Black (52.6%). The national rate of OI hospitalization per 100,000 PWH‐years declined from 2,664.0 (95% CI, 2,664.3–2,665.6) in 2012 to 1,784.5 (95% CI, 1,783.9–1,785.1) in 2018 (Figure 1). Regionally, the South had the highest rate every year which declined from 3,074.4 (95% CI, 3,073.4–3,075.4) in 2012 to 2,125.4 (95% CI, 2,124.5–2,126.3) in 2018. The percentage of OI hospitalizations in the US resulting in in‐hospital mortality was 6.4% in 2012 and 6.0% in 2018.

**Figure:**
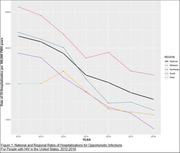
**Abstract PESAC10‐Figure 1**.

**Conclusions**: OI hospitalization rates for PWH in the US declined from 2012–2018, but there are regional disparities with PWH in the South experiencing the highest burden of OI hospitalizations. Studies should examine what gaps in services are contributing to these OI hospitalizations. Communities and clinics may need region‐specific interventions. Additionally hospitalizations due to specific OIs may also contribute to these regional disparities and should be investigated.

### PESAC11 Declining incidence of chlamydia and gonorrhea, but not mycoplasma in a high‐risk population eligible for PrEP experiencing a test‐and‐treat‐setting

T.A. Crowell ^1,2^, K. Jansen^3^, A. Esber^2,1^, C. Spinner^4^, S. Esser^5^, C. Lehmann^6^, C. Boesecke^7^, C. Tiemann^8^, A. Stoehr^9^, J. Hartikainen^10^, M. Bickel^11^, S. Schneeweiß^12^, S. Scholten^12^, C. Cordes^13^, N.H. Brockmeyer^14^, H. Jessen^15^, V. Bremer^3^, U. Marcus^3^, G. Steffen^3^, H. Streeck^16,17^,, for the BRAHMS Study Team^17^

^1^U.S. Military HIV Research Program, Walter Reed Army Institute of Research, Silver Spring, United States, ^2^Henry M. Jackson Foundation for the Advancement of Military Medicine, Bethseda, United States, ^3^Robert Koch Institute, Department for Infectious Disease Epidemiology, Unit for HIV/AIDS, STI and Blood‐borne Infections, Berlin, Germany, ^4^Technical University of Munich, University Hospital rechts der Isar, München, Germany, ^5^University Hospital, University of Duisburg‐Essen, Essen, Germany, ^6^University Clinic Köln, Köln, Germany, ^7^University Bonn, Department of Internal Medicine, Bonn, Germany, ^8^MVZ Labor Krone GbR, Bad Salzuflen, Germany, ^9^Institut für interdisziplinäre Medizin, Hamburg, Germany, ^10^Zentrum für Infektiologie, Berlin, Germany, ^11^Infektiologikum, Frankfurt/Main, Germany, ^12^Praxis Hohenstaufenring, Köln, Germany, ^13^Praxis Dr. Cordes, Berlin, Germany, ^14^Center for Sexual Health and Medicine, University of Bochum, Bochum, Germany, ^15^Praxis Jessen2 + Kollegen, Berlin, Germany, ^16^Institute for HIV Research, University Bonn, Bonn, Germany, ^17^Institute of Virology, Medical Faculty, University Bonn, Bonn, Germany

**Background**: Pre‐exposure prophylaxis (PrEP) can prevent HIV, but could lead to behavioral changes increasing risk of other sexually transmitted infections (STIs). We evaluated changes in STI incidence and associated risk factors in men eligible for PrEP.

**Methods**: The prospective, multicenter BRAHMS study enrolled participants without HIV, 18–55 years, who reported recent STI or condomless anal intercourse with ≥2 male partners with HIV or unknown status. Every three months, participants were offered PrEP, completed behavioral questionnaires, and were screened and treated for STIs including syphilis as well as *Chlamydia trachomatis* (CT), *Mycoplasma genitalium* (MG), and *Neisseria gonorrhoeae* (NG) at the rectal, urethral and pharyngeal sites. We calculated three‐months‐incidence rates and used multi‐level mixed effects logistic regression to calculate odds ratios (ORs) and 95%‐confidence intervals (CIs) for factors associated with any STI.

**Results**: From 6/2018–3/2021, 1,017 participants were followed with mean age 33 years, 99.1% cisgender men, and 96.8% gay/bisexual. PrEP was used at enrollment 54.0%, 82.2% at any point during follow‐up.

Between 3‐month and 12‐month visits, MG incidence was higher than other STIs and relatively stable (figure). CT incidence decreased by 27.2% among PrEP users and 49.2% for non‐PrEP users, NG incidence by 28.1% and 21.7%. Syphilis incidence was lowest and decreased by 2.2% and 15.4%. Incidence was higher in PrEP‐users only for anorectal localizations.

**Figure:**
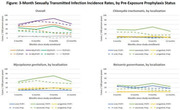
**Abstract PESAC11‐Figure 1**.

STI incidence was independently associated with PrEP use (OR = 1.3; CI: 1.1–1.6), condomless anal sex with >5 casual partners (OR = 1.8; CI: 1.5–2.2), STI‐related symptoms (OR = 1.8; CI: 1.4–2.3), recreational drug use (OR = 1.7; CI: 1.4–2.0), born outside of Germany (OR = 1.5; CI: 1.2–1.9), and moderately or largely increased self‐perceived risk of HIV (OR = 1.3; CI: 1.1–1.6 and OR = 1.4; CI: 1.1–1.8).

**Conclusions**: A structured test‐and‐treat setting appears to have reduced CT and NG incidence in men with sexual risk behavior. Besides other behavioral factors, PrEP use increased STI risk in our cohort slightly, and services providing PrEP may be an effective setting for intensive STI‐related risk reduction counselling considering the risk factors identified.

### PESAC12 Incidence of HCV reinfection among people with HIV prior to and during periods of limited and broad access to direct‐acting antiviral therapies for HCV in five countries

M. Stoove ^1,2^, R. Sacks‐Davis^1,2^, D. van Santen^1,2,3^, A. Boyd^3,4^, J. Young^5^, T. Spelman^1^, A. Stewart^1,2^, M. van der Valk^4,6^, A. Rauch^7^, I. Jarrin^8^, K. Lacombe^9^, L. Wittkop^10^, G. Matthews^11^, J. Doyle^1,12^, M. Klein^5^, M. Prins^3,4^, M. Hellard^1,12,2^, InCHEHC Study Group

^1^Burnet Institute, Disease Elimination, Melbourne, Australia, ^2^Monash University, School of Public Health and Preventive Medicine, Melbourne, Australia, ^3^Public Health Service of Amsterdam, Amsterdam, Netherlands, the, ^4^Amsterdam University Medical Centers, Amsterdam, Netherlands, the, ^5^McGill University Health Center, Montreal, Canada, ^6^Stichting HIV Monitoring, Amsterdam, Netherlands, the, ^7^University Hospital Bern, Bern, Switzerland, ^8^Instituto de Salud Carlos III, Madrid, Spain, ^9^Sorbonne Universites, Paris, France, ^10^University of Bordeaux, Bordeaux, France, ^11^Kirby Institute, Sydney, Australia, ^12^Alfred Hospital and Monash University, Department of Infectious Diseases, Melbourne, Australia

**Background**: Direct‐acting antivirals (DAA) may reduce HCV incidence through a treatment‐as‐prevention effect. Reinfection incidence after successful treatment has been a major concern for HCV elimination, particularly among people with HIV (PHIV). We aim to assess changes in HCV reinfection incidence following limited and broad DAA introduction among PHIV.

**Methods**: We used data from six cohorts from the International Collaboration on Hepatitis C Elimination in HIV‐coinfection (InCHEHC), including data from the Netherlands, Switzerland, Australia, Spain, and France (2010–2019). Participants were considered at risk of reinfection if they had a HCV positive test followed by spontaneous or treatment clearance. We measured the incidence of first reinfection per participant. Time zero was the first negative HCV RNA test indicating treatment or spontaneous clearance. Data were censored at the last negative HCV RNA test or infection date, which was estimated as the midpoint between the last negative and first positive test dates. The rate of reinfection was compared between three periods: prior to DAA access, and during limited access, and broad access to DAA in each jurisdiction using a piecewise exponential survival model, with a fixed effect for risk group (men who have sex with men [MSM], people who inject drugs [PWID] vs. other/unknown) and a random intercept at the cohort level.

**Results**: Overall, 2,818 (57%) MSM, 1237 (25%) PWID, and 880 other participants were included. The median (IQR) age at spontaneous or treatment clearance was 47 (40–53). During 13,527 person‐years (py), we observed 790 reinfections (5.8 per 100py, 95%CI 5.4–6.3). Relative to the pre‐DAA period, reinfection incidence was 25% lower in the limited DAA access period (IRR: 0.75, 95%CI 0.62–0.91), and 44% lower in the broad access period (IRR: 0.56, 95%CI 0.48–0.66). Compared to MSM, reinfection incidence was 54% lower in PWID (IRR = 0.46, 95%CI 0.37–0.56), and 57% lower in those with other/unknown risks (IRR = 0.43, 95%CI: 0.34–0.55).

**Conclusions**: HCV reinfection rates among PHIV were high prior to DAA introduction, particularly among MSM. Our data suggests that DAA introduction was associated with declines in HCV reinfection incidence rates among PHIV. With HCV treatment uptake resulting in a growing pool of individuals at risk of reinfection, continued monitoring is warranted.

### PESAC13 Pooled estimates of hepatitis C incidence among gay and bisexual men using PrEP by country‐level availability of hepatitis C DAA treatment: a systematic review and meta‐analysis

M. Traeger ^1,2^, B. Harney^1,2^, J. Doyle^1,3^, M. Hellard^1,2,3^, M. Stoové^1,2^

^1^Burnet Institute, Melbourne, Australia, ^2^Monash University, School of Public Health and Preventive Medicine, Melbourne, Australia, ^3^Alfred Health and Monash University, Department of Infectious Diseases, Melbourne, Australia

**Background**: Sexual transmission of hepatitis C virus (HCV) among gay and bisexual men (GBM) has been historically concentrated among people living with HIV, but concerns exist that the widespread scale‐up of PrEP may increase HCV incidence among HIV‐negative GBM. Given community‐level HCV viremia at the time of PrEP scale‐up is likely to moderate risk among PrEP users, we examined the impact of broad DAA availability on HCV incidence in studies of GBM using PrEP.

**Methods**: We searched PubMed, Medline, EMBASE and relevant conference databases to 9^th^ August 2021 for studies reporting HCV incidence among GBM using PrEP (daily or event‐driven). Cohort studies and open‐label clinical trials were included. Pooled estimates of HCV incidence were calculated using random‐effects meta‐analysis. Using a literature and policy review of the timing of broad/universal HCV DAA availability in included countries, we conducted a subgroup analysis of PrEP studies that commenced participant follow‐up before or after country‐level DAA availability.

**Results**: Seventeen studies published between 2015–2021 reported on HCV incidence among GBM using PrEP and were included; all were from high‐income countries. One study reported HCV incidence among daily and event‐driven PrEP users separately and was included as two observations in the meta‐analysis. Overall 18,363 participants were included; 145 incident HCV infections were captured over 24,023 person‐years. The pooled estimate of HCV infection across studies was 0.91 per 100 person‐years (range, 0.00 to 2.93/100py). Heterogeneity was high across studies (I^2^ = 84.0%, χ^2^ p < 0.001). Twelve studies (5,186 individuals) commenced follow‐up before broad‐access to DAAs, with a pooled incidence of 1.27/100py (95% CI, 0.69–1.86; I^2^ = 81.8%, χ^2^p = 0.187) and five studies (13,177 individuals) started follow‐up after broad‐access to DAAs, with a pooled incidence of 0.30/100py (95% CI, 0.11–0.50; I^2^ = 33.2%, χ^2^p = 0.187).

**Conclusions**: Our pooled estimates of HCV incidence among PrEP users were lower than reported in a previous meta‐analysis (1.48/00py), and lower in settings where broad access to DAAs had occurred prior to study initiation. Findings suggest that reductions in community‐level HCV viremia driven by DAA uptake among respective GBM populations, which occurred prior to changes in sexual networks and behaviours associated with PrEP uptake, may have contributed to lower HCV transmission among PrEP users.

### PESAC14 Decision analytic model estimates of the predicted benefits and anticipated costs associated with HIV self‐testing versus standard provider‐administered testing modalities if implemented in Kenya

B. Akasreku ^1^, E. Kelvin^2,3^, N. Sabounchi^4,5^

^1^CUNY Graduate School of Public Health and Health Policy, Community Health and Social Science, New York City, United States, ^2^CUNY Graduate School of Public Health and Health Policy, Epidemiology and Biostatistics, New York City, United States, ^3^CUNY Institute for Implementation Science in Population Health, New York City, United States, ^4^CUNY Graduate School of Public Health and Health Policy, Health Policy Management, New York City, United States, ^5^Center for Systems and Community Design, New York City, United States

**Background**: To reach the UNITAIDS 95‐95‐95 goals by 2030, countries need information about the potential benefits and costs associated with various HIV testing modalities to make informed policies. We used decision‐analytic modeling to estimate the benefit and cost of HIV self‐testing (HIVST) relative to the standard provider‐delivered HIV testing (HTC).

**Methods**: We developed a decision‐analytic model to estimate the predicted number of HIV‐diagnosed infected individuals and on treatment and the estimated cost associated with the following scenarios if implemented in Kenya: (*1*) Provider‐supervised HIVST, 2) un‐supervised HIVST, and 3) HTC. The model focused on the 2020 Kenyan population aged 15–64 (n = 31,671,946), who were assumed to be sexually active. We conducted a literature review to determine costs for all points in the HIV care continuum (testing, treatment for those who test positive, PrEP for those at high risk who test negative) from the provider perspective in US dollars and probabilities of accessing each stage in the HIV care continuum (i.e., probability that someone will test, probability that someone who tests positive will access treatment and that someone who is high risk and tests negative will access PrEP).

**Results**: Among the three scenarios, unsupervised HIVST resulted in correctly diagnosing and initiating treatment for more than twice the number of people living with HIV (PLWH) (n = 102,389 PLWH on treatment) than for supervised HIVST (n = 42185) and HTC (n = 38470). However, the estimated cost for both provider‐supervised and unsupervised HIVST was higher ($60,117,771 and $57,597,242, respectively) than HTC ($20,203,585). Offering unsupervised HIVST over provider‐supervised HIVST resulted in a cost‐saving of $0.27 per additional person tested. All these costs were sensitive to changes in the cost of testing, the number who test under each scenario and the linkage to care and treatment initiation rates.

**Conclusions**: Unsupervised HIVST had a cost advantage over provider‐supervised HIVST and led to a higher number of PLWH on treatment than supervised HIVST and HTC. Countries should consider the benefits of making unsupervised HIVST easily available for free but take into consideration preferences for supervised vs. non‐supervised testing among different population groups and the relatively higher cost of getting more people into care.

### PESAC15 Recent HIV acquisition and age‐disparate relationships predict rapid repeated pregnancies among adolescent mothers in a large South African cohort

W. Saal ^1^, E. Toska^1,2^, J. Tolmay^1^, N. Langwenya^2^, J. Jochim^2^, C. Wittesaele^2^, L. Cluver^2^

^1^University of Cape Town, Centre for Social Science Research, Cape town, South Africa, ^2^University of Oxford, Department of Social Policy and Intervention, Oxford, United Kingdom

**Background**: Pregnant/breastfeeding adolescent girls and young women are at greater risk of HIV infection, poor retention in care, and higher rates of secondary transmission. Understanding the timing and predictors of rapid repeated pregnancies is critical to ensuring HIV service provision aligns with their HIV prevention and reproductive needs.

**Methods**: We analysed data from 1,045 young mothers who had their first child before <20 years old, 30% of whom were living with HIV (88% recently infected). Participants were recruited in the HEY BABY cohort through a six‐prong sampling approach including health facilities, schools, community organizations and referrals, to minimise selection bias (>95% enrolment for each recruitment channel). Self‐reported HIV status was verified through medical records where available. Ethical approvals were given by the Universities of Cape Town and Oxford, local government, and participating facilities. We explore factors experienced during and post first childbearing associated with rapid repeated childbearing, using multivariate regression models in STATA16.

**Results**: 99% of first‐child pregnancies were unintended. 8.8% of participants had multiple children, with 18.5% among adolescent mothers living with HIV experiencing multiple pregnancies. Rapid repeated pregnancies, occurring when first child is < = 2, were 5.3% in the overall study, 9.9% among participants living with HIV. A quarter of first children were born while the mother was an in age‐disparate relationship (5+ years older partner), this rose to 50% among adolescent mothers living with HIV. In multivariate analyses, adolescent mothers with children < = 2 years (aOR = 4.71 95%CI 2.76–8.05, p < 0.001), who recently acquired HIV (aOR = 2.54 95%CI 1.41–4.57, p = 0.002), and who had their first child in an age‐disparate relationship (aOR = 1.83 95%CI 1.10–3.04, p = 0.02) had higher odds of rapid repeated pregnancies. These risks were cumulative: 14.1% of participants who recently acquired HIV and had their first child in an age‐disparate relationship were at risk of rapid repeated childbearing, compared to 3.9% among adolescent mothers who were HIV‐free and did not have their first child with a significantly older partner.

**Conclusions**: Adolescent mothers, especially those living with HIV need access to consistent family planning and dual protection urgently. Young mothers with recently‐acquired HIV need additional support to time future childbearing, remain retained in care and attain positive HIV outcomes.

### PESAC16 FCGR3Agene duplication, FcγRIIb‐232TT and FcγRIIIb‐HNA1a associate with an increased risk of vertical acquisition of HIV‐1

J. Ebonwu ^1,2^, R. Lassaunière^3^, M. Paximadis^1,4^, R. Strehlau^5,6^, G.E. Gray^7,8^, L. Kuhn^9^, C.T. Tiemessen^4,1^

^1^University of the Witwatersrand, Faculty of Health Sciences, Johannesburg, South Africa, ^2^National Institute for Communicable Diseases, Division of Public Health Surveillance and Response, Johannesburg, South Africa, ^3^Statens Serum Institut, Department of Virus and Microbiological Special Diagnostics, Copenhagen, Denmark, ^4^National Institute for Communicable Diseases, Centre for HIV &STIs, Johannesburg, South Africa, ^5^Rahima Moosa Mother and Child Hospital, Empilweni Services and Research Unit, Johannesburg, South Africa, ^6^University of the Witwatersrand, Pediatrics and Child Health, Faculty of Health Scinces, Johannesburg, South Africa, ^7^University of the Witwatersrand, Perinatal HIV Research Unit, Faculty of Health Sciences, Johannesburg, South Africa, ^8^South African Medical Research Council, Cape Town, South Africa, ^9^Mailman School of Public Health, Columbia University, Gertrude H. Sergievsky Centre, College of Physicians and Surgeons, and Department of Epidemiology, New York, United States

**Background**: Different mother‐to‐child transmission (MTCT) studies suggest that allelic variations of Fc gamma receptors (FcγR) play a role in infant HIV‐1 acquisition, but findings are inconsistent. To address potential confounding of small samples sizes, the present study investigates the association between perinatal HIV‐1 transmission and FcγR variability (*FCGR* point mutations and gene copy number variation) in a large cohort of South African infants born to women living with HIV‐1.

**Methods**: This retrospective, nested case‐control study combines *FCGR* genotypic data from three perinatal cohorts at two hospitals in Johannesburg, South Africa. Children with perinatally‐acquired HIV‐1 (cases, n = 395) were compared to HIV‐1‐exposed uninfected children (controls, n = 312). All study participants were black South Africans and received nevirapine for prevention of MTCT. Functional variants were genotyped using a multiplex ligation‐dependent probe amplification assay and Sanger sequencing, and their representation compared between groups using logistic regression analyses.

**Results**: *FCGR3A* gene duplication independently associated with HIV‐1 acquisition (OR = 3.70; 95% CI 1.05–12.99; *P* = 0.041). The FcγRIIb‐232TT genotype significantly associated with increased odds of HIV‐1 acquisition in a multivariate model, which controlled for *FCGR3A* copy number and *FCGR3B* genotype (AOR = 1.72; 95% CI 1.07–2.76; *P* = 0.024). When adjusted separately for *FCGR2C* c.134–96C>T that associated with infant HIV‐1 acquisition in a different South African cohort, the strength of association increased (AOR = 2.28; 95% CI 1.11–4.69; *P* = 0.024). Homozygosity for FcγRIIIb‐HNA1a did not significantly associate with HIV‐1 acquisition in a univariate model (OR = 1.42; 95% CI 0.94–2.16; *P* = 0.098); however, it attained significance after adjustment for *FCGR3A* copy number and *FCGR2B* genotype (AOR = 1.55; 95% CI 1.01–2.38; *P* = 0.044). Both FcγRIIb‐232TT (AOR = 1.84; 95% CI 1.11–3.03; *P* = 0.017) and homozygous FcγRIIIb‐HNA1a (AOR = 1.67; 95% CI 1.06–2.62; *P* = 0.028) retained significance when birthweight and breastfeeding were added to the model. We did not observe an association between the common *FCGR2A* and *FCGR3A* polymorphisms and HIV‐1 acquisition.

**Conclusions**: Collectively, our findings suggest that the FcγRIIb‐232TT genotype exerts a controlling influence on infant susceptibility to HIV‐1 infection. We also show a role for less studied variants – *FCGR3A* duplication and homozygous HNA1a. These findings provide additional insight into a role for FcγRs in HIV‐1 infection in children.

### PESAC17 The association of HIV‐1 subtypes and transmission clustering with late diagnosis: the first nationwide study in Japan

T. Shiino ^1,2^, M. Otani^2,3^, M. Nishizawa^2^, A. Hachiya^4,5,6^, H. Gatanaga^7^, D. Watanabe^8^, R. Minami^9^, M. Imahashi^4^, K. Yoshimura^10^, W. Sugiura^1^, T. Matano^2,3^, T. Kikuchi^2^, Japanese Drug Resistance HIV‐1 Surveillance Network

^1^National Center for Global Health and Medicine, Center for Clinical Sciences, Tokyo, Japan, ^2^National Institute for Infectious DIseases, AIDS Research Center, Tokyo, Japan, ^3^The University of Tokyo, The Institute of Medical Science, Tokyo, Japan, ^4^National Hospital Organization Nagoya Medical Center, Clinical Research Center, Aichi, Japan, ^5^Tokyo Medical University, Tokyo, Japan, ^6^Nitobe Bunka College, Tokyo, Japan, ^7^National Center for Global Health and Medicine, AIDS Clinical Center, Tokyo, Japan, ^8^National Hospital Organization Osaka National Hospital, AIDS Medical Center, Osaka, Japan, ^9^National Hospital Organization, Kyushu Medical Center, Internal Medicine, Clinical Research Institute, Fukuoka, Japan, ^10^Tokyo Metropolitan Institute of Public Health, Tokyo, Japan

**Background**: Late HIV diagnosis is a major concern worldwide. While previous studies in some countries have reported association of late diagnosis with qualitative and/or demographic factors, clinical and phylogenetic factors remain unclear. In the present study, we conducted a large‐scale analysis to explore the demographic, clinical, and phylogenetic factors associated with late HIV diagnosis in Japan.

**Methods**: Japanese Drug Resistance HIV‐1 Surveillance Network collects anonymized demographic, clinical, and sequences data for about 40% of newly diagnosed HIV cases in Japan from collaborating hospitals nationwide. Of the 9,866 newly diagnosed cases enrolled in the surveillance network between 2003–2019, 7,853 cases with available CD4 count at diagnosis were included. Late diagnosis was defined as HIV diagnosis with a CD4 count below 350 cells/μL. Factors associated with late diagnosis were determined using Logistic regression. Molecular transmission clusters were identified by HIV‐TRACE with genetic distance threshold 1.5%.

**Results**: The study population was predominantly male (95.0%) and Japanese (90.8%). Median age at diagnosis was 37 (IQR: 30–45) years, and 5,594 (71.2%) and 3,636 (46.3%) were diagnosed with CD4<350 cells/μL and with CD4<200 cells/μL, respectively. Demographic and clinical factors independently associated with late diagnosis were period of diagnosis (2009–2014: aOR 1.16, p < 0.05, versus 2015–2019), transmission risk (Heterosexual: aOR 1.37, p < 0.01, Other risk and Unreported: aOR 2.15, p < 0.0001, versus MSM), older age (≧45 years: aOR 2.13, p < 0.0001, 30 to 44: aOR 1.43, p < 0.0001, versus ≦29), geographical area (other than Tokyo: aOR 1.20, p < 0.01, versus Tokyo), and HCV co‐infection (aOR 1.42, p < 0.05). Individuals in molecular transmission clusters were less likely to be diagnosed with CD4<350 cells/μL than those not in clusters (aOR 0.80, p < 0.01). HIV‐1 CRF07_BC was negatively associated with late diagnosis (aOR 0.24, p < 0.001, versus subtype B). Country of origin, gender, or HBV co‐infection was not associated with late diagnosis.

**Conclusions**: It was revealed that over 70% of newly diagnosed HIV cases in Japan were diagnosed with CD4<350 cells/μL. This study indicates for the first time the association of late HIV diagnosis with HCV co‐infection, HIV‐1 subtypes, and transmission clustering. Consideration of these factors would contribute to efficient enhancement of early diagnosis of HIV.

### PESAD01 Improving HIV knowledge and gender‐based attitudes amongst male inmates through football in correctional facilities in Zambia, Malawi and Zimbabwe

P. Dias ^1^, J. Haney^2^, M. Wolfe^2^, T. Ponde^3^, C. Gamble^4^, P. Diouf^5^

^1^Umunthu Foundation, Blantyre, Malawi, ^2^TackleAfrica, Lusaka, Zambia, ^3^Volunteer Services Overseas, Harare, Zimbabwe, ^4^TackleAfrica, Brighton, United Kingdom, ^5^Volunteer Services Overseas, Phnom Penh, Cambodia

**Background**: In Southern Africa, incarcerated people experience higher HIV prevalence than the general population and have been identified as a critical group for HIV programming in order to achieve the “95‐95‐95” goals. However, preliminary data collected from VSO and TackleAfrica, in a pilot program across 11 correctional facilities in Malawi, Zimbabwe and Zambia in 2019, suggested that both HIV knowledge and positive attitudes towards women was low. We report the results of an inmate led, football‐based intervention designed to improve HIV and SRHR knowledge and positive attitudes towards women amongst male inmates.

**Methods**: The program was implemented over two years in ten‐week blocks. Baseline and end‐line surveys were collected before and after each block by youth volunteers working with inmate coaches. HIV knowledge was measured using the UNAIDS Comprehensive HIV Knowledge indicator. Questions around SRHR and attitudes to women were based on similar DHS & WHO indicator questions.

**Results**: Over 2 years, 126 inmates were trained as peer football coaches to reach 2920 male inmates with 1047 football sessions integrated with sexual health messaging. 1,109 male inmates completed baseline surveys, of which 562 (51%) completed end‐line surveys. The primary reason for leaving the program was release or transfers. Comprehensive HIV knowledge improved from 35% at baseline (n = 1109) to 51% at end‐line (n = 562). Knowledge of STIs improved from 52% (n = 1109) at baseline to 65% at end‐line (n = 491). The question, “Should a girl be able to refuse unwanted sex from her husband or boyfriend?” saw an increase from 76% to 88% at end‐line in Zambia (n = 531/n = 230), while in Malawi and Zimbabwe the question “having sex with many women is a sign of man‐hood” saw a correct answer increase from 64% at baseline (n = 656) to 76% at end‐line (n = 278).

**Conclusions**: Football based interventions are an effective mechanism to engage with male inmates in Southern Africa around sensitive issues related to HIV, SRHR and attitudes towards women. While issues associated with inmate transfer and release may disrupt program completion, inmate‐led sports‐based interventions represent a sustainable and valuable mechanism to engage with a highly disenfranchised and alienate group that are critical to reaching UNAIDS targets.

### PESAD02 Survey Measurements of Community Norms on Adolescent Girls and Young Women's (AGYW) Sexual Behaviour and Use of Condoms for HIV Prevention in Manicaland, East Zimbabwe

S. Gregson ^1^, T. Dadirai^1^, R. Maswera^1^, L. Moorhouse^2^, T. Museka^1^, P. Mandizvidza^1^, F. Dzamatira^1^, B. Tsenesa^1^, C. Nyamukapa^1^, M. Skovdel^3^

^1^Biomedical Research and Training Institute, Manicaland Centre for Public Health Research, Harare, Zimbabwe, ^2^Imperial College London, Department of Infectious Disease Epidemiology, London, United Kingdom, ^3^University of Copenhagen, Copenhagen, Denmark

**Background**: Qualitative data suggest pre‐marital sex stigma presents a major obstacle to AGYW's use of HIV prevention methods. Lack of social acceptability therefore is included as a barrier to motivation to use condoms in HIV prevention cascades. Representative survey data on community norms are rare but necessary to test the validity of this assumption and measure their contributions to gaps in prevention cascades.

**Figure:**
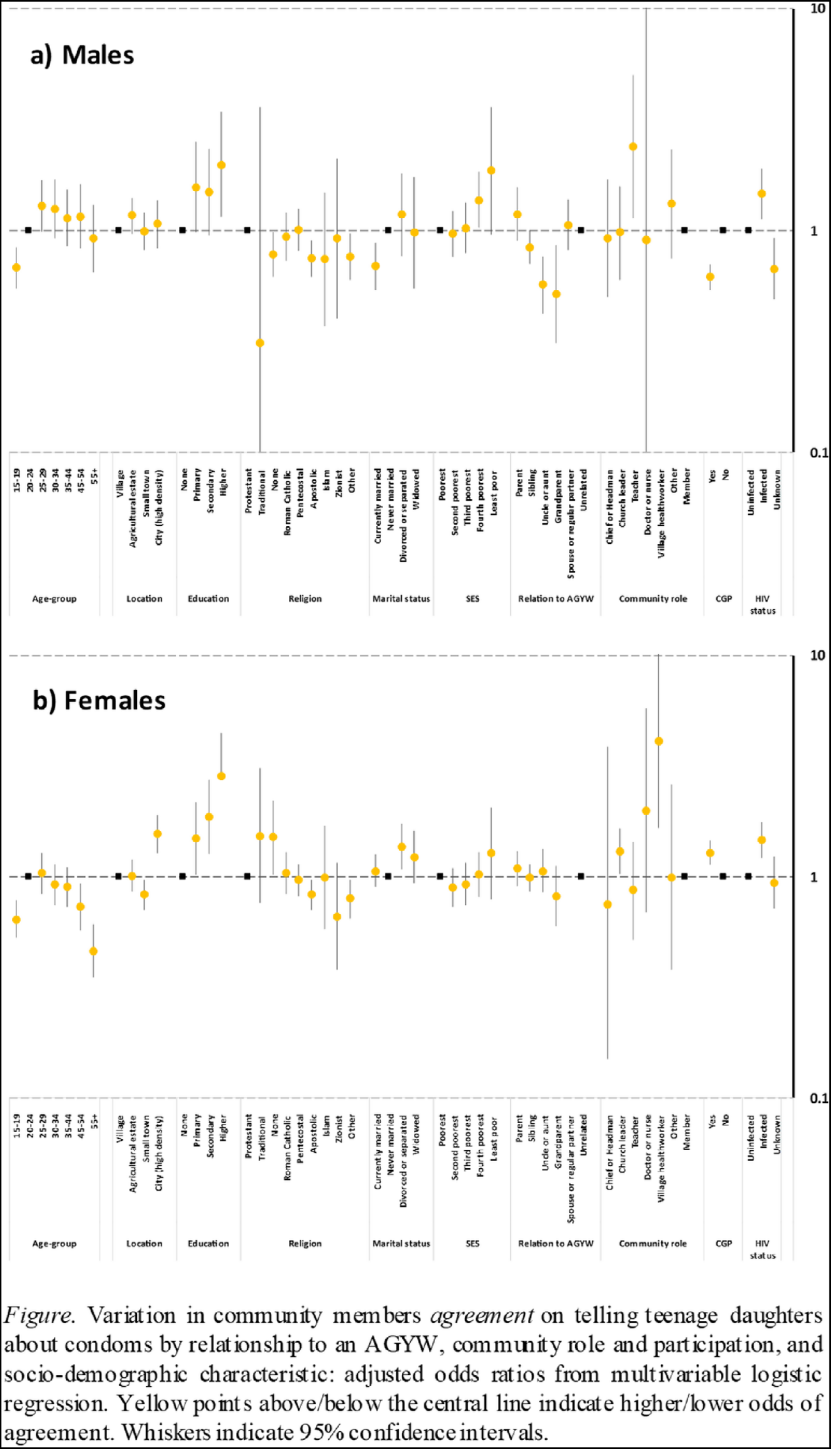
**Abstract PESAD02‐Figure 1**.

**Methods**: General‐population survey participants in Manicaland (ages≥15,N = 9803) were asked if they agreed/disagreed with statements on social norms. AGYW were asked whether community views are an obstacle to their using condoms. Proportions agreeing/disagreeing with these statements were calculated, variations in community members’ views were investigated in multivariable logistic‐regression models, and the association between AGYW's perceiving negative community norms and condom use was measured.

**Results**: 93.5%(95%CI,93%‐94%) of respondents agreed that ‘Many young women have sex before marriage these days’. 57%(56%‐59%) of men and 70%(69%‐71%) of women disagreed that ‘If I have a teenage daughter and she has sex before marriage, I would be ok with this’; and 41%(40%‐43%) of men and 57%(56%‐59%) of women disagreed that ‘If I have a teenage daughter, I would tell her about condoms’. Fathers but not mothers were more likely to disagree with their daughters having sex before marriage (Figure). Similar proportions of parents and other community members were against telling daughters about condoms. 68%(61%‐75%) of sexually‐active unmarried AGYW said negative community views were unimportant in decisions to use condoms. Condom use didn't differ between those who agreed/disagreed that negative community views are important (46.9% vs. 50.0%; AOR = 0.88, 95%CI, 0.48–1.62; N = 202).

**Conclusions**: Community resistance to condom promotion based on pre‐marital sex stigma may be weakening as a barrier to AGYW's motivation to use condoms in Manicaland. Community‐led interventions to accelerate this dynamic in social norms and support AGYW's agency could reduce HIV incidence.

### PESAD03 From mobile phone to health center: WhatsApp as persuasive tools towards HIV testing among college students of the remote district of Pakistan

F. Ahmad ^1^, M.N. Qaisar^2^

^1^University of Okara, DOMS, Okara, Pakistan, ^2^NUML, Islamabad, Pakistan

**Background**: HIV testing targets in remote areas of Pakistan remain low. Due to this issue, many people were unable to know about their status and cannot be linked to HIV care. The objective of this study was to check the effectiveness of interventions using WhatsApp as a platform to improve HIV testing among college students in the Larkana district of Pakistan.

**Methods**: We used a randomized controlled trial approach, and recruited, HIV‐negative, 18 years or older college students, who did not get HIV testing in the last 6 months. These were recruited online and randomly were assigned to two groups i.e. an intervention or control group. Participants were recruited using google forms. After being recruited online, participants completed a short baseline survey. Later, a trained health worker used the mobile application WhatsApp to follow each participant for 8 weeks. They shared different topics of interest with an emphasis on HIV prevention in the second half of the period of follow‐up. Participants in control groups received standard of care. The main outcome was the number of participants who got an HIV test at one of the health centers of the research project.

**Results**: Participants were recruited between Jan and March 2021. 500 participants were randomly assigned to the intervention group (n = 250) or the control group (n = 250). 91 participants (36.4%) in the intervention group and 19 (7.6%) in the control group went to the health center to receive an HIV test (adjusted odds ratio: 7.54; 95% CI: 4.1 ‐13.4).

**Conclusions**: This strategy where trained health workers using a mobile application for follow‐up, was good to divert traffic towards health centers for HIV testing. It is important to use WhatsApp through mobile to get traffic for HIV testing, mobile applications are already been widely used in remote parts of Pakistan as well.

### PESAD04 Structural level factors and PrEP stigma influence on PrEP Use among Black MSM (18 to 29) in the US

D. Boyd ^1^, G.M. Abubakari^2^, J. Allen^3^

^1^The Ohio State University, Social Work, Columbus, United States, ^2^Yale University, School of Public Health, New Haven, United States, ^3^Wayne State University, Social Work, Detroit, United States

**Background**: Albeit the proven efficacy of PrEP to prevent HIV transmission and reduce spread among HIV key populations such as Black men who have sex with men (MSM), uptake may remain slow among such populations due to structural and individual‐level factors in the United States (US). As such, we examined the relationships between structural factors (food insecurity, homelessness, employment), PrEP stigma on PrEP use among a national sample of Black MSM between the ages 18 to 29.

**Methods**: The survey was programmed for two sampling and social media sites (Twitter, Facebook) with Qualtrics software, targeting 400 Black MSM between 18 and 29 years. The survey was completed directly on the Qualtrics portal for the Qualtrics sampling site, and a link was provided for M‐Turk sampling site. The research team distributed an anonymous link to the survey embedded on the flyer posted on social media. Respondents were recruited for all three locations from December 1, 2021, to January 31, 2022. We used a logistic regression analysis to examine whether structural factors and PrEP stigma correlate with the outcome variable PrEP use.

**Results**: Our results from our logistic regression indicated that Black MSM who scored higher on the PrEP stigma scale were less likely to use PrEP (OR: 0.73; 95%CI: 0.52, 0.99). Black males who stated that they lost their job due to Covid 19 were 2.9 times more likely to use PrEP than those who did not (OR: 2.93; 95%CI: 1.36, 6.29). Individuals who reported reducing the number of meals at least once or twice were more likely to use PrEP (OR: 4.28, 95CI: 2.17, 8.42). Lastly, those who reported being homeless in the past 12 months were more likely to use PrEP than those who never reported being homeless (OR: 1.00; 95%CI: 1.00,1.00).

**Conclusions**: Our results suggest reduced uptake of PrEP among BLMSM who experience PrEP stigma but show an increased uptake among those who are food insecure or homeless. Thus, highlighting that despite possible available resources that link low‐income BMSM to PrEP, stigma remains a key issue that would continue to impede use, hence, the need for innovative interventions to address PrEP stigma among BMSM in the US.

### PESAD05 Young Heroes Initiative – High Five (YHI‐HF): Creative Contributory Contest (CCC) and Social Innovation Hackathon as an Entry Point in Engaging Adolescents for HIV and AIDS

A. Dicto ^1^, J.C. Arana^2^, J.C. Borja^3^

^1^Council for the Welfare of Children, Quezon City, Philippines, the, ^2^Council for the Welfare of Children, Quezon City, Philippines, the, ^3^Positive Youth Development Network, Iloilo, Philippines, the

**Background**: In December 2020, the HIV and AIDS and Art Registry of the Philippines recorded 273 (25%) cases among the youth 15–24 years old. The Integrated HIV and Behavioral and Serologic Surveillance presented that HIV knowledge is lowest among 15–20 years old. Both evidence shows adolescents’ risky sexual behaviors start when they have insufficient knowledge on HIV and AIDS and less access to ASRH services. In December 2020, with funding from the Council for the Welfare of Children (CWC), Unicef, and UNAIDS, YHI‐HF was launched.

**Description**: The intervention has 3 phases. Phase 1 is the Creative‐Contributory‐Contest, where teams (4 adolescents, one adult mentor) are asked to submit their 2030 Vision of Young People and HIV and AIDS using creative outputs. Thirty‐two (32) teams joined this phase with outputs varying from dances to paintings. Phase 2 is the social innovation hackathon where they build on their creative outputs to pitch for concrete solutions. Mentors and experts were tapped to help them fine‐tune their ideas utilizing the Human‐Centered Design. Six teams proceed on the final phase, the project implementation. They are given 2,000 USD to implement their social innovation for a year with the local CWC office. The projects include traveling play, magazine publication, audience‐led performing arts, and a musical designed for and by adolescents.

**Lessons learned**: There were 451,428 directly engaged adolescents in the project. An increase of 54% from baseline in basic knowledge and awareness about HIV and AIDS was observed among participants. 48% showed increased health‐seeking behavior, as indicated by their willingness to be tested, and an increase of 72% in the level of knowledge and awareness in condom use, PREP, and combination methods was also observed. Psychological safety (32%), fun and creative environment (63%), and peer‐to‐peer approach (55%) were among the top 3 identified reasons why adolescents joined the project.

**Conclusions/Next steps**: CCC and Hackathon are effective approaches to attract adolescents to HIV‐related activities. Children can implement effective HIV interventions if given the proper support and resources. The next phase of the project is to design a training module for duty‐bearers working on HIV on how they can effectively engage adolescents in their area.

### PESAD06 Perspectives and Preferences for Multi‐Purpose Prevention Technologies (MPTs) to Address Sexual and Reproductive Health (SRH) Needs Among Adolescent Girls and Young women (AGYW) in Kenya and Uganda

L.L. Lunani ^1^, S. Namukwaya^2^, S. Lipesa^1^, B. Kombo^1^, D. Omondi^1^, J. Shikuku^1^, C. Omom^1^, A. Maina^1^, R. Malogo^1^, K. Katumba^2^, I. Kayesu^2^, Z. Nabalwanyi^2^, R. Naluwooza^2^, Y. Mayanja^2^, G. Omosa‐Manyonyi^1^, O. Anzala^1^, P. Indravudh^3^, M. Quaife^3^, L. Gao^4^, M. Kuteesa^5^, Y. Machira^6^, A. Kamali^5^, M. Gafos^3^

^1^KAVI‐ICR, University of Nairobi, Nairobi, Kenya, ^2^MRC/UVRI & LSHTM Uganda Research Unit, Entebbe, Uganda, Entebbe, Uganda, ^3^London School of Hygiene and Tropical Medicine, London, United Kingdom, ^4^Busara Center for Behavioral Economics, Nairobi, Kenya, ^5^International AIDs Vaccine Initiative (IAVI), Entebbe, Uganda, ^6^International AIDS Vaccine Initiative (IAVI), Nairobi, Kenya

**Background**: Adolescent girls and young women in Sub Saharan Africa are disproportionately affected by high rates of HIV, sexually transmitted infections (STIs) and unintended pregnancies. To inform development of MPTs and delivery of SRH services, an innovative behavioral science research project (UPTAKE) seeks to determine factors that facilitate future acceptability and uptake of long‐acting (LA) technologies to prevent HIV and unintended pregnancy among AGYW in Nairobi, Kenya and Kampala, Uganda.

**Methods**: We conducted in‐depth interviews with 30 AGYW aged 15–24 years in Nairobi and Kampala. We explored participants’ perceptions of future MPTs, building on their experiences and views of existing family planning (FP) and HIV prevention products. Interviews were audio recorded, transcribed, and translated. Data were analyzed thematically in NVivo and reconciled iteratively during reflective sessions with the research teams.

**Results**: An interim analysis shows that AGYW generally have a limited understanding of the FP and HIV prevention options available to them. Most of them report early sexual debut, premature school dropout, a lack of family and social support, and engaging in age‐disparate sexual relationships, transactional sex or sex work. Participants reported unintended pregnancies and initiated FP only after childbirth. There were misconceptions and a lack of trust in modern HIV prevention methods, with some viewing PrEP as a drug for HIV positive individuals. Only AGYW engaging in sex work were aware of PrEP. In terms of future MPTs, most participants preferred long‐acting injectable, for >1‐year, inserted in the arm, providing HIV and pregnancy prevention. Participants expressed limited interest in prevention for other STIs, which they considered treatable. Government facilities were the preferred choice for future delivery of MPTs due to affordability. AGYW described themselves as decision‐makers in relation to their SRH, while acknowledging the need for new products citing women's’ limited ability to negotiate HIV testing and condom use with male partners.

**Conclusions**: Long‐acting MPTs offer considerable potential for reducing HIV infection and unintended pregnancies among AGYW. Product developers should consider MPTs during manufacturing. Policy makers and programme planners need to make these products and their information readily available and accessible to AGYW using youth friendly health services and other approaches.

### PESAD07 Body image dissatisfaction is positively associated with poorer mental health outcomes but not linked to HIV risk behaviours among young gay, bisexual, transgender and queer men in Singapore

A. Tyler ^1^, Z.H. Mah^2^, D. Le^1^, A. Tan^1^, C. Tan^1^, C. Kwok^1^, S. Banerjee^1^, A.R. Cook^3^, M.L. Wong^3^, R.K.J. Tan^3,4^

^1^Action for AIDS Singapore, Singapore, Singapore, ^2^National University of Singapore, Yong Loo Lin School of Medicine, Singapore, Singapore, ^3^National University of Singapore, Saw Swee Hock School of Public Health, Singapore, Singapore, ^4^University of North Carolina Project‐China, Guangzhou, China

**Background**: No prior study has been published on the impact of body image dissatisfaction in the Gay, Bisexual, Transgender and Queer (GBTQ) community in Singapore. Furthermore, while many studies have found a positive link between poor mental health outcomes and HIV risk behaviours, few have explored the role of body dissatisfaction in driving such risks. This study aims to explore associations between varying measures of body image dissatisfaction with measures of mental and sexual health risks, including depression severity, suicidal ideation and attempts, as well as recent condom‐less anal intercourse.

**Methods**: Data were derived from a cross‐sectional follow‐up survey of the Pink Carpet Y Cohort Study, Singapore's first prospective cohort study among young GBTQ men. Participants comprised HIV‐negative, young GBTQ men aged 18–25 years old. Body image dissatisfaction was measured through the Male Body Attitudes Scale (MBAS‐R), Drive for Muscularity Scale (DMS), and two measures of distance between perceived and ideal body types: Body Fat Difference (BFD) and Muscularity Difference (MD). Higher scores indicate greater dissatisfaction. Multivariable logistic and linear regression were employed.

**Results**: The study recruited a total of 396 participants. Multivariable analyses revealed that MBAS‐R (Coeff. = 0.08, 95% CI 0.04, 0.12) and BFD (Coeff. = 0.04, 95% CI 0.01, 0.08) were positively correlated with depression severity. MD (aOR = 1.02, 95% CI 1.00, 1.03) was positively associated with ever having suicidal ideation, whereas DMS (aOR = 0.97, 95% CI 0.96, 1.00) was negatively associated with suicidal ideation. Analyses also revealed that MBAS‐R (aOR = 1.03, 95% CI 1.01, 1.05) and BFD (aOR = 1.02, 95% CI 1.00, 1.03) were positively associated with ever having attempted suicide. No measures of body dissatisfaction were significantly associated with recent condom‐less anal intercourse.

**Conclusions**: Body image dissatisfaction in young GBTQ men was positively associated with negative mental health outcomes. Future health promotion interventions should be targeted at developing a positive body image in young GBTQ men, in hopes of achieving better mental health outcomes. Given that poor mental health outcomes may drive syndemics of HIV acquisition, further research into the lasting impact of body image dissatisfaction on sexual risk in young MSM is warranted.

### PESAD08 Integrating human‐centered design, public health, and behavioral science to improve access to HIV services among young men who have sex with men in Kenya

A. Beeman^1^, J. Levine^2^, R. Hope ^2^, A. Lacorazza^2^, S. Olabode‐Dada^2^, M. Omondi^3^, J.M. Wambui^4^, T. Gibbs^2^

^1^Youth Development Labs (YLabs), Kigali, Rwanda, ^2^Youth Development Labs (YLabs), Berkeley, United States, ^3^Men Against AIDS Youth Group (Maaygo), Kisumu, Kenya, ^4^Health Options for Young Men on HIV/AIDS/STI (Hoymas), Nairobi, Kenya

**Background**: The criminalization and stigmatization of same‐sex sexual activity contribute to social, community, and healthcare‐related barriers that prevent young men who have sex with men (YMSM) from accessing high‐quality HIV services. Using a human‐centered design (HCD) approach, research and design firm YLabs partnered with community YMSM‐serving organizations Maaygo and Hoymas to develop an HIV self‐testing (HIVST) campaign and service delivery model. The pilot aimed to expand programmatic reach to YMSM ages 15–24 in Nairobi and Kisumu counties, Kenya, not currently engaged in services to increase access to HIV prevention and care.

**Description**: The program consisted of a behavior change‐informed digital and physical demand generation campaign co‐designed with YMSM. This included YMSM‐focused messaging to address common fears about HIV testing; an automated SMS messaging platform to connect YMSM to peer educators for delivery of HIVST kits and incentives at convenient pick‐up points; and streamlined, confidential access to sensitized clinicians to encourage reporting of HIVST results and facilitate linkage to follow‐up care.

**Lessons learned**: Compared to 2019 data, the pilot demonstrated a 265% increase in testing of new YMSM clients to participating clinics, an 834% increase in YMSM who had never tested for HIV, and a 123% increase in YMSM who had not tested within 12 months. Successful engagement strategies included paid advertisements with YMSM‐specific language across multiple social media platforms promoted by LGBTQ influencers. Distinct differences in engagement by YMSM by geography, social media channel, campaign subscription channels, and preferred incentives illustrated the need to tailor programming to sub‐populations of YMSM. Barriers to HIVST kit use included supply chain shortages of tests, emotional preparedness to accept a positive result, and low willingness to report results to clinic personnel.

**Conclusions/Next steps**: The success of this pilot indicates the value of using HCD to create customized programming that engages key populations such as YMSM in the development of interventions intended to serve them. The tailored demand generation approaches and messaging addressed key fears and barriers to testing, enabling the program to test harder‐to‐reach youth. Preferred awareness channels and testing incentives differed between YMSM geographically, emphasizing the need to tailor future programs' contextual nuances to maximize reach and effectiveness.

### PESAD09 Factors associated with initiation of selling sex as a minor among adult female sex workers in Eswatini

A. Grosso ^1^, B. Sithole^2^, S. Maziya^3^, M. Akinmade^4^, S. Matse^5^, S. Baral^6^

**Table:** 

**Abstract PESAD09‐Table 1**.										
	**Age at the time of the study [mean]**	**Education [completed primary school or higher]**	**Has any living children**	**Sold sex in multiple geographic regions in the past 12 months**	**Must share earnings with a person who arranges clients or provides protection**	**Ever beaten up as a result of selling sex**	**Can count on sex worker colleagues to help deal with a violent or difficult client**	**Tested positive for syphilis in the study or reported a previous diagnosis with syphilis in the past 12 months**	**Tested positive for HIV in the study**	**Was forced to have sex after age 18**
Adjusted odds ratio (95% CI)	0.82 (0.76, 0.88)	0.66 (0.52, 0.87)	0.39 (0.20, 0.79)	1.51 (1.14, 1.99)	2.82 (1.07, 7.41)	1.89 (1.01, 3.54)	0.69 (0.50, 0.96)	3.31 (1.26, 8.71)	N/A	N/A
Started 18+ 74% (237/320)	28	80% (189/237)	84% (199/236)	38% (90/236)	7% (15/229)	35% (81/234)	86% (199/231)	8% (20/236)	74% (171/233)	38% (89/232)
Started <18 26% (83/320)	22	69% (57/83)	52% (43/83)	51% (42/83)	17% (14/82)	48% (40/83)	77% (64/83)	16% (13/83)	60% (48/80)	44% (32/72)

Note: Multivariable logistic regression analysis controlled for all variables listed above except HIV status and experience of forced sex

^1^Rutgers University Rutgers Institute for Health, Healthcare Policy and Aging Research, Urban‐Global Public Health, New Brunswick, United States, ^2^FHI 360, Mbabane, Eswatini, ^3^HealthPlus 4 Men, Mbabane, Eswatini, ^4^Rutgers University Rutgers Institute for Health, Healthcare Policy and Aging Research, New Brunswick, United States, ^5^Ministry of Health, National AIDS Program, Mbabane, Eswatini, ^6^Johns Hopkins School of Public Health Center for Public Health and Human Rights, Epidemiology, Baltimore, United States

**Background**: Eswatini has a population of 1.16 million; 26.8% of all adults and 61% of female sex workers (FSW) are living with HIV. There have been recent reports of underage girls selling sex due to economic hardships during COVID‐19 and while schools were closed. Moreover, an estimated 800+ children in Eswatini have lost a parent due to COVID‐19, and in a 2014 study, adult FSW in Eswatini who were orphaned before the age of 18 were more likely to have started selling sex as minors. Here, we aimed to describe associations of initiation of selling sex as a minor in Eswatini.

**Methods**: In 2011, FSW aged 18+ who exchanged sex for money, favors, or goods in the past 12 months were recruited through respondent‐driven sampling in Eswatini, completed a questionnaire, and were tested for HIV and syphilis. Unadjusted multivariable logistic regression analysis was conducted to compare participants who reported their age of initiation of selling sex was <18 years old to those whose age of initiation was 18+.

**Results**: 60% of FSW who started selling sex <18 tested positive for HIV, and this did not significantly differ from those who started as adults after controlling for current age (p = 0.953). Participants who started selling sex <18 were younger at the time of the study, less educated, and more likely to be mobile. They had higher odds of testing positive for syphilis or reporting a recent syphilis diagnosis, being forced to share earnings with someone who arranges clients or provides protection, and being beaten up due to selling sex and lower odds of counting on other sex workers to help deal with violent clients.

**Conclusions**: Selling sex as a minor has potentially lasting effects on social determinants of adverse sexual health outcomes. Strengthening education, economic support and child protective services is necessary to prevent sexual exploitation of children in Eswatini, and health services are needed address the vulnerabilities of those who are selling or previously sold sex as minors.

### PESAD10 Challenges to HIV Prevention and Sexual Health Promotion Among Impoverished Youth Living in Slums/Favelas of São Paulo, Brazil in the First Two Years of the COVID‐19 Pandemic

M. Jardim dos Santos ^1^, L. Oliveira da Silva^2^, V.S. Facciolla Paiva^2^

^1^University of São Paulo/Medicine School, Preventive Medicine, São Paulo, Brazil, ^2^University of São Paulo/Institute of Psychology, Social Psychology, São Paulo, Brazil

**Background**: The emergence of the COVID‐19 pandemic and its concomitant socio‐economic and humanitarian crises magnified youth's vulnerability to HIV/AIDS, which was already high in impoverished suburban territories. Community‐based peer education projects had to innovate to guarantee young people's right to health.

**Description**: The H.I.V‐Project (Heliópolis Investindo na Vida) promotes sexual health among young people in Heliópolis, the largest slum in the metropolitan area of São Paulo. Starting in January 2020, the primary local CBO selected a group of 9 young leaders (age 15–19) to be peer‐educators in HIV/STI and pregnancy prevention through a Human Rights and Vulnerability perspective and Freire's pedagogy framework. Seventeen of the twenty months of the project were mostly online, respecting COVID prevention measures and included weekly supervision workshops, interventions using livestreams and social media posts, activities focused on afterschool programs aimed at 13–17 yr‐olds, and mask+condom distribution to young people at Street‐Funk parties and public gatherings.

**Lessons learned**: A lack of housing infrastructure and internet access hampered participants’ engagement during online activities. Participants kept quiet when trying to discuss sexuality, HIV/STI prevention, and especially “sex during a pandemic.” In small houses without privacy, participants had little space to talk about their sexual experiences or explore their thinking and feelings about these themes. Furthermore, in times of COVID, as parents became unemployed, nurseries and daycares closed for long periods, and the domestic workload increased, girls had less time to work on the project. Two girls also had unexpected pregnancies. Boys, on the other hand, had to work long hours to support their family income. Black boys in particular experienced increased police violence and were also more absent from the project. Working COVID and mental health into the program was inescapable while inventing methodologies and producing content, mixing online with in‐person encounters.

**Conclusions/Next steps**: It remains crucial to consider the coalescence of the ongoing COVID‐19 pandemic with the HIV/AIDS and mental health epidemics. In order to innovate HIV/STI prevention peer‐education, social, structural, psychosocial, interpersonal, and intrapersonal barriers should be considered. The integrality of human rights and the notion of “comprehensive prevention” needs to encompass “combination prevention”.

### PESAD11 Effectiveness of a multifaceted intervention *(KUJA‐KUJA)* to reduce violence and increase condom use in intimate partnerships among female sex workers: a randomized controlled trial in Nakivale Refugee settlement in Uganda

N. Nabulumba ^1^, J. Ssemanda^2^, C. Kamwiine^1^

^1^ALIGHT, Protection, Nsingiro, Uganda, ^2^University of Florida, Center for African/Latin American Studies, Gainesville, United States

**Background**: Intimate Partner Violence (IPV) is among the prevalent problems among sex workers in Nakivale settlement. Evidence suggests that about 41% of sex workers have experienced IPV during Covid‐19 lockdown. The high rate of participation in sex work in the settlement is associated with poverty, alcoholism, explore for better shelter and misuse of drugs. However, the effectiveness of the present interventions to reduce IPV among sex workers isn't well streamlined. “Kuja‐kuja” a multifaceted intervention, working with sex workers, their intimate partners (IPs) and communities could help reduce IPV and increase condom use among sex workers and refugees in Nakivale settlement.

**Methods**: A total of 36 villages (most sex workers) and 2 towns were visited in a two arm cluster randomized control study in Nakivale refugee settlement. 15 villages and 1 town were randomized to Kuja‐kuja intervention while the remaining on a wait‐list control. Female sex workers above 20 years with IPs in the last 4 months were interviewed and participated in both surveys from Dec 2019 to August 2021. A baseline survey was given 6months after which an end line surveys was conducted in all villages and towns to assess the effectiveness of “Kuja‐kuja” intervention.

**Results**: With a baseline (n = 410) imbalance was observed with reference to age (32.8 vs 34.1) and IPV (32.6% vs 46.1%), study results indicate no differences in physical/sexual IPV (7.1% vs 8.2%), severe physical/sexual IPV (5.8% vs 7.4%) and consistent condom use with IPs (62.5% vs 57.3%) by trial arm at end line (n = 257). Kuja‐kuja was connected with reduced acceptance of IPV (adjusted OR (AOR) = 0.62, 95% CI 0.40 to 0.94, p = 0.025) and solidarity of sex workers around issues of IPV (AOR = 1.69, 95% CI = 1.02–2.82, p = 0.042). We observed a rise in IPV between baseline (25.9%) and midline (63.5%) among women in Kuja‐kuja villages but lower in parallel villages (41.8%–44.3%) and a sharp decrease at end line in both arms (∼8%).

**Conclusions**: The kuja‐kuja had a significant effect on both increasing awareness of self‐protection strategies and solidarity around IPV among sex workers within the kuja‐kuja villages. However, we found insignificant evidence that the proposed intervention increased condom use.

### PESAD12 Resilient & Empowered, Adolescents and Young PLHIV (READY++)‐a pilot initiative working with adolescent and young PLHIV in India

F. Khan ^1^, R. Mitra^2^, P. K^3^, S. Kumar^1^, R. Sharma^1^

^1^India HIV/AIDS Alliance, Care & support, Delhi, India, ^2^India HIV/AIDS Alliance, Director‐Care & support, Delhi, India, ^3^India HIV/AIDS Alliance, Associate Director‐Care & support, Delhi, India

**Background**: Adolescents and young people contribute 40% of the Indian population. The evidence suggested that adolescents and young people engage in sexual experimentation and sexual risk taking, the accessibility of this group to accurate information on SRH and sexuality related services is poor. Such situations expose to adverse SHR outcomes. Nearly 10% of PLHIV in India are adolescents and young people in the age group. AYPLHIVs face unique challenges related to their HIV status and related to their SRH needs such as growing up changes, developing relationships, sexuality, marriage, and love.

**Description**: a pilot peer lead initiative namely READY++ was rolled out in five states of India by Alliance India, to address the specific unmet SRHR and mental health needs of AYPLHIVs to build a cadre of informed and empowered AYPLHIVs with information and skills on SRHR and HIV, who in turn reached their fellow AYPLHIVs with comprehensive knowledge on a range of SRHR issues, following activities carried out

1. identification peer champions pre‐training assessment
2. capacity building through state level consultations and national level training
3. one2one session with AYPLHIVs by peer champions
4. support group meetings by peer champions
5. peer champions engaging parents and families
6. advocacy meetings by peer champions with prominent state level officials and
7. Engaging AYPLHIVs and youth affected by HIV through social media

**Lessons learned**: The READY++ program employed an intensive process to create powerful, positive and sustainable changes at the level of peer champions. For the first time, they found a platform to discuss issues of guilt about their HIV status and relationship with an HIV negative person, love, sex, marriage, myths, and misconceptions associated with their bodies. Over time, peer champions reached thousands of their peers (both online and offline) with knowledge on HIV and SRHR and facilitated their linkages to HIV services, social schemes and vocational skills building programs.

**Conclusions/Next steps**: The peer champion is playing an active role in the states linking young people with HIV related services, the empowered peer are taking lead to ensure the rights, policy development and active as pressure group and raising issues of the unmet needs of AYPLHIV community

### PESAD13 What are the practices of people attending erotica shows in terms of psychoactive substance use, sexual behavior and HIV testing?

M. Chollier^1^, P. Enel ^2^, M. Bonierbale^3^, A. Maquigneau^1^, T. Korchia^1^, R. De Wever^2^

^1^Aix Marseille University, Psychiatry Dept, CRIR‐AVS Paca, Sainte‐Marguerite Hospital, Marseille, France, ^2^Aix Marseille University, Public Health Dept, COREVIH POC, Marseille, France, ^3^Aix Marseille University, Psychiatry Dept, AIUS, Marseille, France

**Background**: Our action‐research aimed to achieve a specific key‐population estimated vulnerable to HIV‐risk transmission, as people attending Erotic industry shows (ES). While ES seem appropriate events to raise awareness and survey people potentially with an interest for sex, there is little recent data on screening practices, risk perception and lifestyle behaviors in this population.

**Methods**: A cross‐sectional study was conducted in ES in Dec. 2017 to document substance uses, sexual behaviours, and HIV‐screening practices. The intervention was designed as a detached outreach one, with a stand provided information and guidance materials, and an anonymous questionnaire wich investigated knowledge about HIV, HCV and STIs screening history, substance use, sexual behavior and attraction.

**Results**: Overall,781 respondents, 58% male, mean age 34 years, completed the survey. 18% reported substance use in the last 3 months, 51% with alcohol. Among them, 26% reported sexual purposes to substance use: disinhibition (14%), conditioning (13%) and feeling better (11%). Main sexual partners were: spouse (68%), regular (21%), unknown (18%) and multi‐partners (17%).The 3 main sexual practices were: libertinism (22%), partner‐swapping (15%) and threesome (15%). 27% of respondents reported contactless sexual behaviors: BDSM (13%), voyeurism (10%) and exhibitionism (10%). At last 18% of the respondents reported no previous HIV‐test.

In univariate analysis the lack of previous HIV‐test was significantly associated (p < 0.05) with gender, alcohol use, number of drugs, same‐sex attraction, sexual partnership with spouse and multiple partner practice. On logistic regression analysis, the independent predictors of the lack of previous HIV‐test in men were: alcohol (OR: 1.79), same‐sex attraction (OR: 0.13), sexual partnership with spouse (OR: 0.47), and multiple partners practice (OR: 0.60); in women: same‐sex attraction (OR: 8.74), sexual context of drug use (OR: 3.07), number of drugs (OR: 0.66).

**Conclusions**: This innovative intervention explored testing practices, sexual behaviors, substance use and sexual motives for substance use in an audience that was interested in sex but far from perceiving HIV risks. It highlighted the usefulness of HIV education and testing interventions in fun events context such as ES, and the receptivity of participants to reflect on their risk behaviors. This intervention model is intended to complement existing HIV‐testing services.

### PESAD14 Perspectives of migrant people living with HIV on multidisciplinary HIV care: a call for greater patient‐empowerment and formalized community‐engagement

A. Arora ^1,2^, S. Vicente^2,3^, A. Rodriguez‐Cruz^1,2^, K. Engler^2^, N. Kronfli^2,4^, J.‐P. Routy^4^, M. Klein^4^, J. Cox^4^, A. de Pokomandy^1,4^, G. Sebastiani^4^, B. Lemire^5^, R. Wittmer^6^, I. Vedel^1^, A. Quesnel‐Vallée^7,8^, B. Lebouché^1,2,4^

^1^McGill University, Family Medicine, Montréal, Canada, ^2^Research Institute of the McGill University Health Centre, Centre for Outcomes Research & Evaluation, Montréal, Canada, ^3^Université de Montréal, Mathematics and Statistics, Montréal, Canada, ^4^McGill University Health Centre, Chronic Viral Illness Service, Division of Infectious Diseases, Montréal, Canada, ^5^McGill University Health Centre, Pharmacy, Montréal, Canada, ^6^Université de Montréal, Family Medicine and Emergency Medicine, Montréal, Canada, ^7^McGill University, Epidemiology, Biostatistics and Occupational Health, Montréal, Canada, ^8^McGill University, Sociology, Montréal, Canada

**Background**: Multidisciplinary models can facilitate care and treatment engagement for migrants living with HIV (MLWH). However, the perspectives of MLWH around such models have rarely been explored. Our objective was to understand how MLWH experience multidisciplinary HIV care models and their suggestions for improving care.

**Methods**: In January 2020, we initiated a 96‐week prospective longitudinal cohort study with a convergent mixed‐method design at a hospital‐based clinic serving the largest proportion of MLWH in Montreal, Quebec. Currently, 26 patients have been enrolled. All patients received bictegravir/emtricitabine/tenofovir alafenamide for free and were provided care by a multidisciplinary team composed of physicians, nurses, social workers, and pharmacists. Preliminary qualitative data are presented here. Eighteen interviews were conducted with 10 MLWH at two time‐points (10 after 1 week of starting treatment and 8 after 24 weeks) and were analyzed via thematic analysis.

**Results**: Three themes were identified: (1) *multidisciplinary care enables holistic, humanizing, and personalized care* – MLWH expressed that their needs extend beyond HIV treatment dispensation and that the different clinicians, together, were able to address their complex bio‐psycho‐social needs; (2) *multidisciplinary models need to optimize communication, coordination, and empowerment* – MLWH suggested that multidisciplinary teams are only useful when (a) consistent and regular contact is maintained between patients and clinicians, (b) all team members are aware of each other's responsibilities, and (c) clinicians seek to educate MLWH about their HIV and engage them in decision‐making; and (3) *HIV care extends beyond multidisciplinary teams and requires a transition to community‐engaged models to better address patient needs* – MLWH explained that they navigate various clinics and services across their HIV care trajectory for various reasons (e.g., free and anonymous blood tests from certain clinics and social support from community groups), and that these services should be better integrated to ensure efficient care coordination.

**Conclusions**: Multidisciplinary care settings can address the needs of MLWH, particularly through the availability of complimentary care. Improved multidisciplinary care for MLWH could result through optimizing communication, coordination, and empowerment. Furthermore, such care models must evolve to incorporate communities and allied services to better meet the needs of this growing and often vulnerable population.

### PESAD15 Perceptions of healthcare accessibility and medical mistrust among African American women living with HIV in the United States

L. Small ^1^, S. Godoy^2^, C. Lau^1^

^1^University of California, Los Angeles, Social Welfare, Los Angeles, United States, ^2^University of North Carolina at Chapel Hill, School of Social Work, Chapel Hill, United States

**Background**: African American women living with HIV frequently endure structural racism, racial biases, and discrimination in healthcare systems that affect their access to care. The present study sought to explore the lived experiences of these women in healthcare settings as it relates to HIV‐treatment accessibility and medical mistrust.

**Methods**: Four focus groups were conducted with seropositive African American women (*N* = 20) residing in a large urban region. Participants were asked about their experiences with healthcare providers including (a) their relationship with healthcare providers; (b) accessibility of community‐based medical and mental health services; and (c) experiences while attending HIV‐treatment appointments. Utilizing Dedoose software, interviews were transcribed and coded for themes using analytic induction techniques.

**Results**: Analyses revealed four interrelated themes: (1) multilevel stigma and discrimination; (2) medical mistrust of multiple providers; (3) mixed responses to stigma, discrimination, and medical mistrust; and (4) preferences for patient‐provider relationship. Participants expressed that medical providers and staff perpetuated negative treatment that included multiple forms of discrimination and stigmatization based on their HIV/AIDS status and social identities. In particular, our participants perceived that their negative experiences were connected to their HIV/AIDS diagnosis, race, class, and gender. Participants described ways their mistrust moved beyond their primary doctors to include nurses, medical staff, and pharmaceutical staff. The stigma, discrimination and resulting mistrust that they experienced often resulted in hurt feelings and decisions to disengage from treatment or remain with providers while feeling unwelcome. Participants described their desire to feel seen, supported and validated by their entire team of care providers.

**Conclusions**: This study provides insight about the myriad of challenges Black women living with HIV/AIDS face when navigating the healthcare system. Narratives reveal that feelings of being discriminated against or minimized can cultivate mistrust not only towards primary physicians, but across various healthcare providers and staff. Findings from this study can inform care models for low‐income African American women living with HIV/AIDS. Specifically, medical education and healthcare trainings should include the stories and perspectives of marginalized communities, including seropositive African American women, in an effort to actively work against negative effects due to stigma and discrimination.

### PESAD16 Villains to Superheroes: Supporting adolescents and young people living with HIV through a social media‐based psychosocial support intervention

V. Munatsi ^1^

^1^University of Cape Town, Sociology, Cape Town, South Africa

**Background**: Sexually transmitted diseases cause shame. Therefore, having HIV is shameful and results in a spoiled identity, vilification, self‐ loathing and stigma. Although shame can be experienced by all people with HIV, its impact is greater on adolescents, young people and key populations. For these sub groups, shame and HIV infection are developmental traumas that affect mental health, identity formation, self‐esteem, worth and confidence. They cause hiding, isolation, secrecy, poor health seeking behaviour and adherence which is slow suicide.

**Description**: Positive Konnections (PK) is a mobile application with a mental health intervention for young people with HIV, designed to counter the effects of shame and help them access services privately and anonymously. From October 2020 to December 2021, 100 young people with HIV, 18–28 years of age, (60% females) signed up to use the intervention. They engaged with 10 chapters of stories designed to help them discover their true identity, value and self ‐worth . In each chapter, a short video clip was taken from a movie where a super hero was facing a situation. The video was accompanied by a short story that demonstrated how the situations the super heroes were facing were similar to experiences of having HIV. After watching the videos and reading the stories, participants were asked to reflect on how the stories relate to their lives. This was done in the PK Work Book, a personal reflective space were participants expressed their thoughts or feelings which they discussed with a counsellor . Participants also interacted with others in weekly group sessions. https://play.google.com/store/apps/details?id=com.health.positive_konnections&hl=nl&gl=US

**Lessons learned**: Process evaluation suggests that showing young people that their experiences of living with HIV are similar to those of superheroes helped them to reframe negative self or social perceptions and see themselves in a positive light, increasing self‐worth, love and acceptance. Reframing helped them see their true identity, re‐write the narrative of their lives mentally shifting from villains to superheroes . A reduction in shame increased openness to disclose, health seeking behaviour, mental health and adherence to medication.

**Conclusions/Next steps**: Various packages of the intervention are being developed or strengthened to scale up to more people including key populations

### PESAD17 Intensifying community testing strategies to achieve improved case identification in the context of COVID‐19: lessons from Eastern Uganda

O. Apok ^1^, C. Cheptoris^1^, E. Serioni^1^, I. Mirembe^1^, J. Nakazzi^1^, T. Ijagason^1^, A. Mugabe^1^, D. Wamboya^1^, D. Ako‐Arrey^1^

^1^IntraHealth International/USAID RHITES‐E Activity, Mbale, Uganda

**Background**: UNAIDS’ Fast‐Track strategy focuses on enhanced HIV case finding toward accelerating the end of AIDS by 2030. WHO echoes that without deliberate follow‐up interventions, less than 90% of identified positives get linked into care, while retention decreases among those linked to less than 70% in the first six months of care. In Kapchorwa district in Eastern Uganda, where HIV prevalence is 4.8%, myths and misconceptions surrounding COVID‐19, as well as fear of acquiring COVID‐19, directly impacted access to and uptake of health services, including HIV testing and treatment. DHIS2 data in March 2021, at the height of the pandemic, indicated that HIV case identification and linkage to care had fallen off significantly compared to pre‐pandemic outcomes.

**Description**: To improve HIV case identification using a community model, the USAID RHITES‐E Activity, led by IntraHealth International, engaged HIV ART clinic staff to intensively search for positive clients via targeted testing and linkage to ART. Approaches included active follow‐up of enlisted clients from assisted partner notification/index line lists and targeted community/home testing in known high HIV burden villages. In addition, home‐based counseling was provided to address COVID‐19 myths/misconceptions that hindered accessibility and utilization of health facility services. Virtual tools were adapted to improve reporting.

**Lessons learned**: The team quickly adapted to the COVID‐19 pandemic to safely deliver quality services using personal protective equipment and virtual tools. DHIS2 data comparison on entry point of all new positives (see figure) shows a steady increase from 56% in the last quarter of 2020 to 88% by September 2021.

**Figure:**
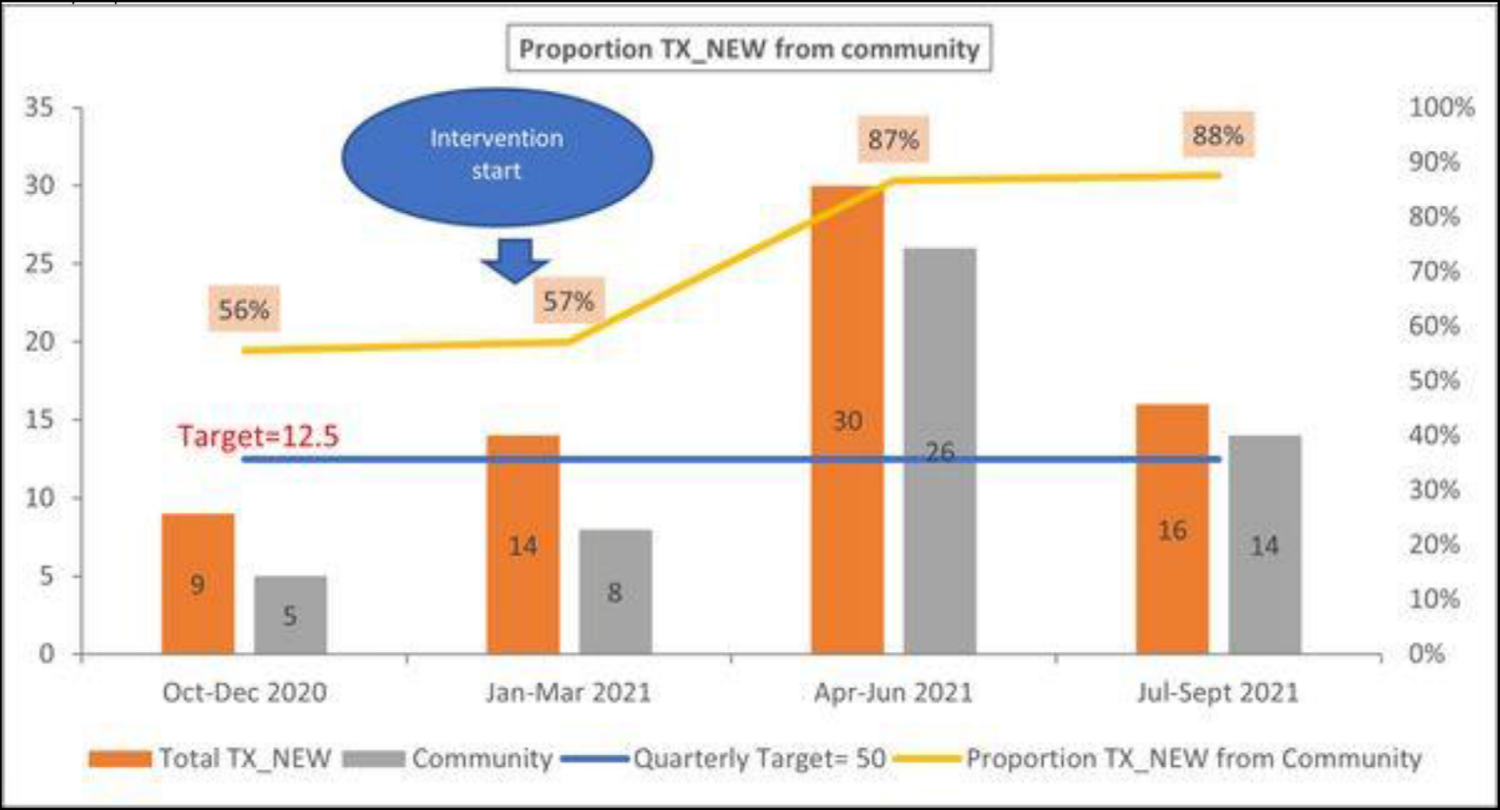
**Abstract PESAD17‐Figure 1**.

**Conclusions/Next steps**: Deliberate integrated community engagement during the pandemic improved continuity of health services in this setting and can be duplicated in similar settings.

### PESAD18 Engaging PrEP Champions to improve uptake and continuation on PrEP among AGYW in the DREAMS Program in Matabeleland North Province, Zimbabwe

P. Moyo ^1^, E. Molande^1^, J. Murungu^1^, I. Mahaka^1^, S. Nyakuwa^1^, J. Ncube^1^, P. Ndlovu^1^, A. Musonza^1^, S. Ruzibe^1^, B. Chingombe^2^, A. Muchara^3^, R. Mundingi^3^, R. Mashapa^3^, A.K. Korn^4^, G. Gonese^3^, T. Mharadze^5^, S. Wallach^6^, R. Malaba^5^, B. Makunike^3^, S. Wiktor^4^

^1^Pangaea Zimbabwe AIDS Trust, Harare, Zimbabwe, ^2^Ministry of Health and Child Care, Harare, Zimbabwe, ^3^Zimbabwe Technical Assistance, Training & Education Center for Health, Harare, Zimbabwe, ^4^International Training and Education Center for Health, University of Washington, Seattle, United States, ^5^Division of Global HIV & TB, U.S. Centers for Disease Control and Prevention, Harare, Zimbabwe, ^6^Public Health Institute (PHI)/U.S. Centers for Disease Control and Prevention (CDC) Global Health Fellowship, Harare, Zimbabwe

**Background**: Oral pre‐exposure prophylaxis (PrEP) implemented as part of combination prevention reduces the risk of HIV infection by 99%. The level of protection provided by PrEP depends on adherence. Pangaea Zimbabwe AIDS Trust (PZAT) supports the delivery of PrEP as part of the Determined, Resilient, Empowered, AIDS‐Free, Mentored and Safe (DREAMS) program aiming to reduce new HIV infections among adolescent girls and young women (AGYW) aged 15–24 years. PrEP interventions for AGYW include monitoring PrEP uptake, continuation, and psychosocial support.

**Description**: Starting January 2021, PZAT engaged 16 AGYW (mean age 23 years) as PrEP Champions (PCs) in Matabeleland North province and trained them on effective engagement of AGYW using a standard Ambassador Toolkit developed by the Optimizing Prevention Technology Introduction on Schedule project to provide peer‐to‐peer support, mobilization, education, and linkage to PrEP. PCs supported effective use and continuation on PrEP through telephone calls, text messages and in‐person follow up. Community dialogues with AGYW were conducted around catchment areas of the 16 supported health facilities and PrEP support groups were formed. Feedback from AGYW was used to address gaps and improve programming. Data were captured and reported using DHIS2 through supported facilities.

**Lessons learned**: The proportion of newly initiated AGYW continuing on PrEP at month 1 increased from 40% in November 2020 to a peak of 91% in July 2021. A temporary PrEP stock‐out affected performance in August 2021.

**Figure:**
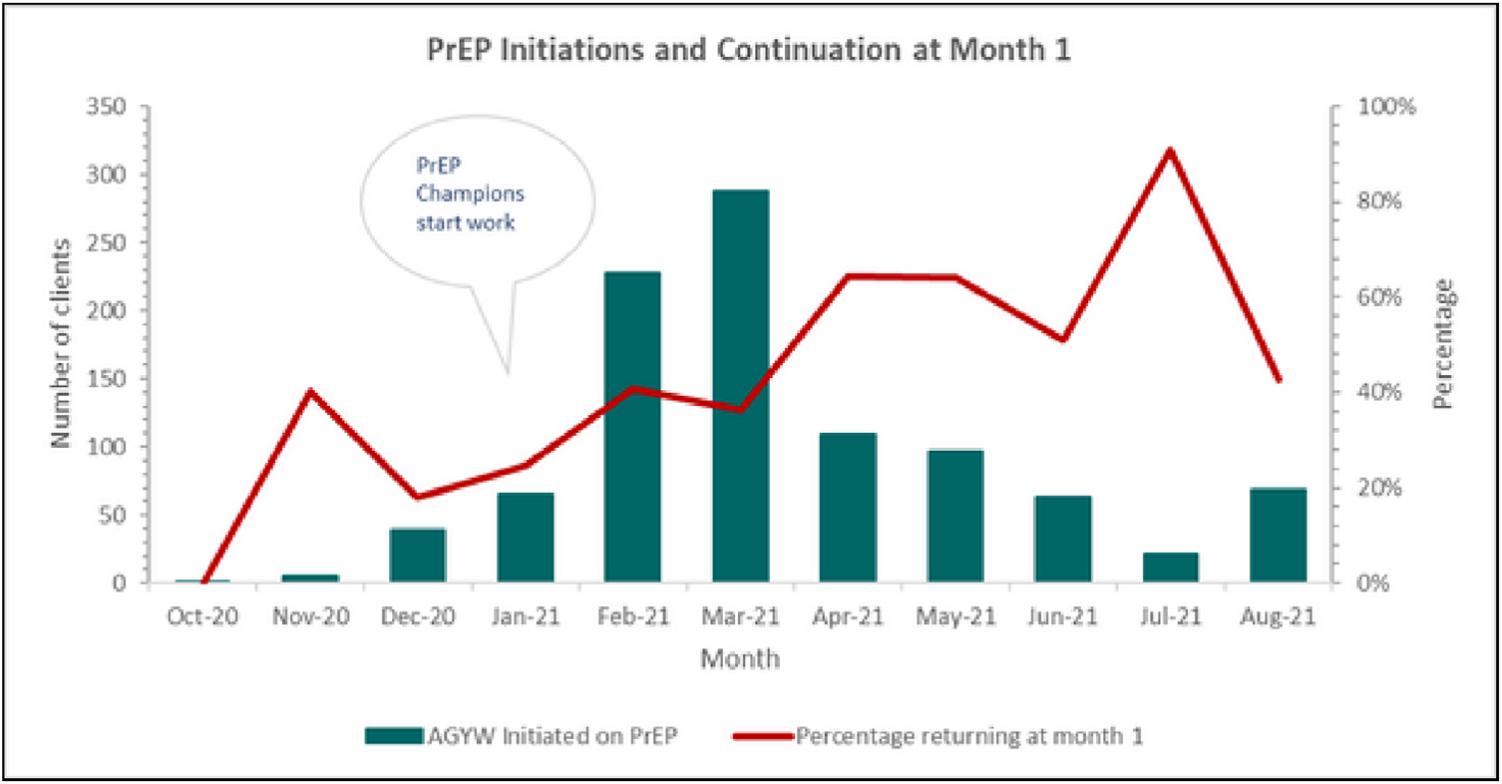
**Abstract PESAD18‐Figure 1**.

**Conclusions/Next steps**: PCs were cited in community dialogues as sources of accurate information that led to an increase in knowledge and linkage to PrEP services for AGYW. Coordination between the health care workers and PrEP Champions resulted in joint defaulter tracking. Peer‐to‐peer support through interpersonal and mobile platforms leads to successful awareness raising, improved uptake and continuation on PrEP. Collection of long‐term continuation data in future promotes further analysis of the impact of PCs.

### PESAD19 The Race to End AIDS and COVID‐19: How edutainment and a rallying call to run for a cause boosted HIV prevention and COVID‐19 vaccination uptake in Zambia

B. Simpasa ^1^, M.N.J.MN.S.H. M. Chikuba‐McLeod,^1^, K.P. M. Bwembya,^2^, M. Musonda^3^

^1^JSI Research & Training Institute, Inc. (JSI), Lusaka, Zambia, ^2^John Snow Health Zambia (JSH), Lusaka, Zambia, ^3^United States Agency for International Development (USAID)/Zambia Mission, Lusaka, Zambia

**Background**: In Zambia, Adolescent Girls and Young Women AGYW (15–24 years) and men (20–34 years) are among the most vulnerable groups to HIV infection (ZAMPHIA 2019). In addition, COVID‐19 has negatively affected access to HIV testing and treatment services (MOH COVID‐19 Media Briefing, Jan 2022). In response to this, the USAID ZAM‐Health and USAID DISCOVER‐Health Projects, in collaboration with the Ministry of Health (MOH) and National AIDS Council (NAC), designed a #Run2EndAIDS#Run4COVIDVaccination event to promote COVID‐19 and HIV service uptake, primarily targeting these groups.

**Description**: Over a 14‐day period between October and November 2021, the #Run2EndAIDS#Run4COVIDVaccination event attracted 7,457 runners from Zambia and 5 other countries, who collectively covered 74,410 kilometers. The event far surpassed the target of 2,000 runners. Runners took photos on their routes of health facilities that provide HIV and COVID‐19 vaccination services to raise awareness about their location and submitted them with their run data. During the event, healthcare providers distributed 22,027 condoms, vaccinated 680 against COVID‐19, tested 775 for HIV, initiated 193 on PrEP, and distributed 1,500 PrEP factsheets. The Zambia Ending AIDS Facebook page recorded 1,945,425 impressions, and 64,553 people were actively engaged. The event culminated in a live‐streamed ‘Virtual Fest’, bringing music and messaging together; the momentum continued with the artists and other participants continuing to champion COVID‐19 vaccination and HIV prevention.

**Lessons learned**: Targeted edutainment events appeal to both young and old, and effectively rally target audiences around a cause, providing correct HIV prevention and COVID‐19 vaccine information and increasing knowledge levels and numbers accessing services. It is, however, crucial to harness the momentum for ongoing activism. A website or mobile app is recommended for ease of registration, to capture run‐data in real‐time, and to provide targeted messaging/engagement, for events that might attract a similarly overwhelming response.

**Conclusions/Next steps**: The #Run2EndAIDS #Run4COVID‐19Vaccination event empowered primarily young people, but also others, to make smart and safe choices about COVID‐19 and take control of their physical, mental and sexual health. It created a group of HIV and COVID‐19 prevention champions who continue to champion these causes.

### PESAD20 Does normalizing sexual conversations increase HIV prevention demand in young women aged 20–24 years? Lessons from the *Mo'ghel, Get Your Life Pack* campaign in five South African districts

Z. Penzhorn ^1^, S. Stafford^1^, M. Kelly^1^, C. Wendler^1^, R. Makiwa^1^, A. Machinda^1^

^1^Shout‐It‐Now, Cape Town, South Africa

**Background**: Shout‐It‐Now (Shout), a PEPFAR/DREAMS partner in South Africa, experienced difficulties mobilizing young women aged 20–24 years to access community‐based HIV prevention services during the country's first two COVID‐19 waves between May 2020‐May 2021. During the third wave, Shout engaged clients, staff and a creative/media partner to develop a demand creation campaign that tapped into young women's desire to move forward in their lives and relationships after feeling stalled by COVID‐19 lockdowns.

**Description**: Using human‐centered design principles, Shout engaged clients and staff to provide input on messaging and service journey mapping. Informed by this, Shout's *Mo'ghel, Get Your Life Pack* campaign featured an aspirational value proposition: a free pack of youth‐friendly services that improve young women's lives and relationships. Campaign promotion hinged on sexual conversations in a youthful but trustworthy vernacular using print media, targeted radio broadcasts/podcasts and social media via igniters and Shout's platforms. Shout also improved staff engagements with young women about HIV prevention through a training video and IEC materials that facilitated organic sexual health conversations. The campaign was implemented August 12‐October 8, 2021 in five districts.

**Lessons learned**: Clients and staff valued contributing to the campaign design, and their input was instrumental to the core message. Shout surveyed clients during the 6‐week campaign to assess its impact on HIV prevention uptake. A combined 2,854 of the total 3,499 (82%) clients served reported they heard of the campaign, including 2,697 females of whom 1,368 (51%) were aged 20–24 years. A comparison of service data among females aged 20–24 years between the six weeks leading up to the campaign and the six weeks of the campaign found a 668% increase in HIV testing and a 44% increase in PrEP initiations. Analysis of the engagement channels found the podcasts and one radio promoter were most effective at mobilizing young women.

**Conclusions/Next steps**: Increased uptake of HIV prevention services among young women during the campaign shows that promoting empowerment and normalizing sexual health conversations can lead to HIV prevention seeking behaviors among a highly vulnerable population. This approach is now used to design all Shout communications and is easily replicable.

### PESAD21 Fake porn videos helped save real lives ‐ creative approaches to increase demand in Ukraine in the context of the HIV epidemic

L. Gavrysh ^1^

^1^All‐Ukrainian Network of People Living with HIV, HealthLink Project, Kyiv, Ukraine

**Background**: Ukraine is the second‐fastest HIV‐spreading country in Europe and Central Asia. HealthLink project is aimed at HIV case‐finding in Ukraine through testing populations at risk of HIV. In Ukraine 257000 people live with HIV, 30% among them don't know their HIV‐status and one of the largest key risk groups is MSM (near 179400 people). Almost 90% of HIV testing within the project is implemented in healthcare facilities. A project's study indicated that men are the priority group for HIV testing and detection, but are less likely to visit the doctors than women. The uptake of HIV testing is especially low among MSM who widely experience stigmatization in the Ukrainian context.

**Description**: HealthLink project set a goal to inform MSM about the option to order the oral HIV self‐tests online completely confidential. Porn sites are those digital places where people seek for anonymity and which are extremely popular at the same time. For the second year in a row, Ukraine is in the top 20 countries in which the PornHub site is used the most.

We created 4 high‐quality videos, which had one detail making them different from most porn site content. There was no sex. Different scenes with real actors and dialogues, but always with the same message — a passionate relationship must be followed by a test which is easy to order. The videos have been uploaded on PornHub and more than 20 famous gay category resources.

**Lessons learned**: For 3 month of campaign in 2020: total reach of 85000 people had a positive effect on the level of knowledge about HIV testing. 9000 clicks were received. The level of clickability reached 3.35%, indicating that the advertising visual was interesting to the audience. More than 700 self‐tests were ordered online.

**Conclusions/Next steps**: Crucial aspect of motivation for HIV testing is meeting the need for protection and staying confidential, therefore it's important to emphasize that testing is confidential, completely safe, fast and easy. The information space is so full of content that it is important to be bold and choose communication channels, messages and visuals that will definitely attract the attention of the target audience.

### PESAD22 “Delivery of clinical HIV prevention care and treatment services for Key Populations through safe spaces in Harare”

C. Muchemwa ^1^, P. Moyo^1^, E. Molande^1^, J. Murungu^1^, S. Nyakuwa^1^, I. Mahaka^1^, P. Manyanga^2^, B. Mushangwe^2^, H. Bara^3^, B. Chingombe^4^, G. Gonese^2^, T. Mharadze^5^, S. Wallach^5^, R. Malaba^5^, R. Weber^5^, B. Makunike^2^, S. Witkor^6^

^1^Pangaea Zimbabwe AIDS Trust, Programmes, Harare, Zimbabwe, ^2^Zimbabwe Technical Assistance Training and Education Centre for Health, Programmes, Harare, Zimbabwe, ^3^City of Harare, Health Department, Harare, Zimbabwe, ^4^Ministry of Health, AIDS and TB Unit, Harare, Zimbabwe, ^5^CDC Zimbabwe, Harare, Zimbabwe, ^6^University of Washington, International Training and Education Centre for Health, Washington, United States

**Background**: Key Populations (KPs) bear the burden of HIV yet have poorer access to HIV services. Pangaea Zimbabwe AIDS Trust (PZAT) is building the capacity of public health facilities to provide KP‐friendly services to improve uptake of HIV prevention, care, and treatment services. KPs experience barriers to care due to stigma, discrimination, criminalization, and socio‐economic challenges. COVID‐19 worsened access to comprehensive HIV services. PZAT conducted differentiated service delivery services at safe spaces to ensure access for KPs in areas around 18 high‐burden public health facilities in Harare.

**Description**: From October 2020 to September 2021, PZAT supported the delivery of comprehensive HIV services for KPs at safe spaces, including secluded outdoor spots. KPs identified safe spaces based on privacy, convenience, safety, and accessibility to many KPs. Thirty Community Facilitators (female and male sex workers, men who have sex with men, transgender and people who use drugs) identify and mobilize KPs ahead of the visit, link them to services, and follow‐up. A multidisciplinary team provides services including HIV testing, Pre‐Exposure Prophylaxis, Antiretroviral Therapy, condoms and lubricants, intimate partner violence screening and referral, sexually transmitted infections (STI) screening, and management.

**Lessons learned**: KPs prefer accessing services where they feel safe and comfortable. A total of 640 KPs were reached with HIV services through safe spaces, among these 547 (85%) were tested for HIV, 524 (96%) were negative, and were all screened for PrEP. 357 (68%) were initiated on PrEP. All 23 KPs that tested HIV positive were initiated on ART. Of the 609 KPs screened, 163 (27%) were diagnosed with STIs and 157 received treatment on site.

**Figure:**
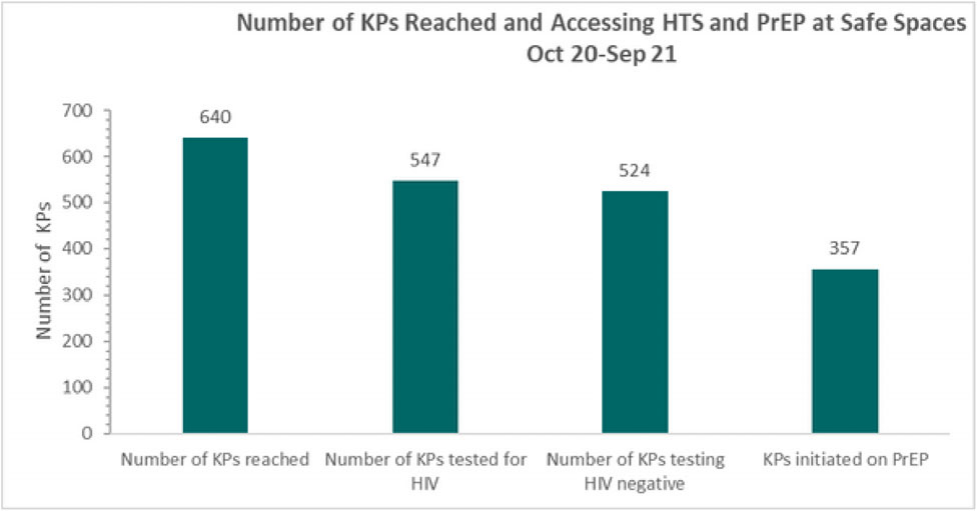
**Abstract PESAD22‐Figure 1**.

**Conclusions/Next steps**: The involvement of KPs in designing, planning, implementation, and monitoring of HIV services provision is important and can improve uptake and utilization of services. Peer‐to‐peer support is essential for identifying eligible clients, mobilizing for services, and supporting adherence and retention.

### PESAD23 Awareness and acceptability of undetectable = untransmittable among a national sample of HIV‐negative young adults in Nigeria

K. Nworie ^1,2^, U. Agbom^1,2^, E. Duru^1,2^, U. Eze^1,2^, D. Aluh^3^, A. Aliu^2^, A. Ezeh^4^, S. Abah^2^, C. Onyekwum^2^, S. David^1^

^1^University of Nigeria, Pharmaceutical Outcomes Research Group, Nsukka, Nigeria, ^2^YOUALIVE AIDS, Enugu, Nigeria, ^3^Nova medical School, Universidade Nova de Lisboa, Lisbon Institute of Global Mental Health, Lisbon, Portugal, ^4^Taraba State Specialist Hospital, Jalingo, Department of Family Medicine, Jalingo, Nigeria

**Background**: Despite widespread support for the U = U statement as an empowering initiative aimed at raising awareness about treatment as prevention (TasP) and ending stigma towards PLWH, information about its reach and impact in developing countries is sparse. In our study, we described the socio‐demographic characteristics and sexual behaviors that are associated with awareness of and trusting U = U in a Nigerian national sample of HIV‐negative participants.

**Methods**: Cross‐sectional cohort analysis of an internet‐based survey of HIV‐negative young adults in Nigeria (n = 1,016) between February and September 2021. Measures included socio‐demographics, sexual behaviors, and awareness of and trust in U = U. Descriptive statistics and multivariable logistic regression were used to identify the characteristics associated with awareness of and trust in U = U, as well as patterns of willingness to engage in condomless sex based on trust in U = U.

**Results**: The participants’ mean age was 26.94±4.68 years. Of the participants, 52.1% of participants reported having heard of U = U. Among those who were aware of U = U, 51.0% reported they trusted it, 20.4% did not trust it, and 28.5% were unsure. Gender identity, sexual orientation, and having tested for HIV in the last 6 months were significantly associated with being aware of U = U. Non‐gay participants were 2 times more likely to be aware of the U = U message (OR = 2.99; 95% CI: 1.08–8.25) than gay participants. Similarly, gender identity, sexual orientation, and having tested for HIV in the last 6 months were significantly associated with trust in U = U. No significant differences were observed by age, level of education, geographic region, or recent condomless sex in the study. Overall, participants were more likely to engage in condomless sex with HIV‐negative partners than with HIV‐positive partners. However, willingness to engage in condomless sex with an HIV‐positive but undetectable partner was associated with trust in U = U.

**Conclusions**: Although we observed moderate U = U awareness and trust in this cohort, crucial populations and minorities are still unaware and distrustful of the U = U message. This study will serve as a basis for further elaborate studies and to develop community‐based health education and awareness initiatives regarding U = U in Nigeria.

### PESAD24 Differences in drug use and HIV stigma among people who inject drugs in Kyrgyzstan

S. Yeager ^1^, N. Shumskaya^2^, A. Kurmanalieva^2^, A. Blyum^1^, T.L. Patterson^1^, D. Werb^3,1^, J. Cepeda^4^, L.R. Smith^1^

^1^University of California San Diego, San Diego, United States, ^2^AIDS Foundation East‐West, Bishkek, Kyrgyzstan, ^3^University of Toronto, Toronto, Canada, ^4^Johns Hopkins, Baltimore, United States

**Background**: The Eastern Europe and Central Asia (EECA) region has the highest annual rate of HIV infections worldwide, primarily concentrated among people who inject drugs (PWID). In Kyrgyzstan, where stigma appears to be reducing syringe service program (SSP) access, approximately 51% of people living with HIV are PWID. We aimed to characterize differences in drug use and HIV stigma by SSP access. We hypothesized that PWID who did not report recent (i.e., past 6 month) SSP access would have higher mean levels of structural and internalized drug use (DU) and HIV stigma, but lower mean levels of anticipated DU and HIV stigma compared to those with recent SSP access.

**Methods**: A cohort of 279 PWID were recruited via community‐based HIV service agencies and word of mouth in the Kyrgyzstan capital city of Bishkek and surrounding rural Chuy region. Participants completed DU and HIV stigma measures that included structural, anticipated, and internalized stigma subscales (1 = low stigma, 5 = high stigma). Anticipated stigma items distinguished between three stigma sources (e.g., healthcare workers, family, other PWID). Internalized HIV stigma was only assessed among PWID who reported living with HIV at baseline (n = 57). Chi‐square and t‐tests were used to assess group differences by SSP utilization.

**Results**: Participants were primarily male (75.3%) with a median age of 40 years. Compared to PWID that did not recently access SSP, those that did access SSP had a higher prevalence of public injection (16.2% vs. 5.3%, p = .018) and a lower prevalence of sharing syringes and equipment (14.2% vs. 22.7%, p = .092). Compared to PWID that did not access SSP, those that did access SSP had significantly higher internalized DU stigma (M = 3.52 vs. M = 3.04, p < 0.001) and anticipated DU stigma from family (M = 3.28 vs. M = 2.89, p = 0.020). Compared to PWID without SSP access, those that did access SSP had significantly greater anticipated HIV stigma from other PWID (M = 3.52 vs. M = 3.20, p = 0.005) and internalized HIV stigma (M = 2.88 vs. M = 1.83, p = 0.004).

**Conclusions**: DU and HIV stigma levels are high, but significantly higher among PWID with recent SSP access, whose drug use is more publicly visible, than PWID without recent SSP access who remain at higher risk for HIV transmission.

### PESAE01 At risk in the emergency department: can the electronic health record identify patients with indications for PrEP?

L. Walter ^1^, N. Carlisle^2^, J. Rodgers^1^, J. Booth^1^, S. Heath^2^

^1^University of Alabama at Birmingham, Emergency Medicine, Birmingham, United States, ^2^University of Alabama at Birmingham, Internal Medicine, Birmingham, United States

**Background**: Persons newly diagnosed with HIV often had previous, recent contact with healthcare, including emergency departments (EDs). While EDs have become vital partners in screening and linkage to care for persons living with HIV, ED engagement in HIV prevention efforts, to include risk assessment and pre‐exposure prophylaxis (PrEP) referral, are rare. We sought to assess the proportion of patients presenting to the ED with HIV risk factors and PrEP indications.

**Methods**: In an ED with a preexisting universal HIV screening program, a retrospective electronic health record (EHR) query was performed for all patients who screened negative for HIV in 2019–2020. Objective laboratory evidence of sexually transmitted infection (chlamydia, syphilis, gonorrhea, and/or trichomoniasis) at the time of ED visit or within six months prior was utilized to assess risk of HIV acquisition through sexual behavior. Urine drug screen positive for commonly injected substances at the time of ED visit or within twelve months prior, was utilized to assess risk of HIV acquisition through injection drug use practices. Descriptive and chi‐square analyses were performed.

**Results**: 26,914 ED patients screened negative for HIV during the study timeframe. Of these, 1,403 (5.2%) were found to have one or more potential HIV risk factors and PrEP indications; 304 (21.7%) had a sexual behavior risk identified and 1,111 (79.2%) had an injection drug use risk identified; of note, 12 (0.9%) had both. Median age was 40; the majority (58.2%) were male and white/non‐Hispanic (50.8%), followed by Black/non‐Hispanic (43.5%). Injection drug use as a risk factor represented a significant proportion as compared to the general population (79.2% versus 9.3% nationally; p < .0001). In consideration of sexual behavior as a risk factor, women in this study represented a significant proportion as compared to national estimates (62.2% versus 41.9%; p < .0001). During the study period, 52 ED patients were newly diagnosed with HIV; retrospective application of ‘EHR query’ approach successfully identified 29% of this cohort.

**Conclusions**: In addition to screening, EDs may represent an opportune setting for HIV risk assessment and PrEP referral. Given time constraints and competing demands inherent to EDs, an objective EHR approach represents a low resource option for risk factor identification.

### PESAE02 The fidelity of a pharmacy‐based PrEP delivery model in Kenya: an unannounced standardized patient actor assessment

V. Omollo ^1^, M. Asewe^1^, P. Mogere^2^, G. Maina^2^, A. Kuo^3^, J. Odoyo^1^, J. M. Baeten^4,5,6,7^, T. Owens^8^, E. Anne Bukusi^9,6,10^, K. Ngure^11^, K. F. Ortblad^12^, P. Kohler^5,13^

^1^Kenya Medical Research Institute, Kisumu, Kenya, ^2^Partners in Health and Research Development, Thika, Kenya, ^3^University of Washington, Department of Pharmacy, Seatle, United States, ^4^Gilead Sciences, Foster City, United States, ^5^University of Washington, Department of Global Health, Seattle, United States, ^6^University of Washington, Department of Epidemiology, Seattle, United States, ^7^University of Washington, Department of Medicine, Seattle, United States, ^8^Howard University, Washington, DC, United States, ^9^Kenya Medical Research Institute, Centre for Microbiology Research, Nairobi, Kenya, ^10^University of Washington, Department of Obstetrics and Gynecology, United States, ^11^Jomo Kenyatta University of Agriculture and Technology, Department of Community Health, Nairobi, Kenya, ^12^Fred Hutchinson Cancer Research Center, Public Health Sciences Division, Seattle, United States, ^13^University of Washington, Department of Nursing, Seattle, United States

**Background**: The delivery of HIV PrEP at private pharmacies is a promising new model that may address barriers to clinic‐based delivery. In Kenya, a pilot study testing this new model found it reached populations at HIV risk and had comparable PrEP continuation to clinic‐based PrEP delivery models. We used unannounced standardized patient (USP) visits to measure the fidelity of pharmacy PrEP delivery in a pilot study.

**Methods**: Five retail pharmacies were purposefully selected to participate in the pilot. Following a two‐day training, pharmacy providers delivered counseling on HIV risk and PrEP safety, provider‐assisted HIV self‐testing, and PrEP to eligible clients as per the Kenya national guidelines. We trained eight USPs and asked them to visit each pharmacy acting the following cases: 1) a young woman seeking emergency contraception, 2) a young woman who fears HIV testing, 3) a young man who has sex with men seeking treatment for a sexually transmitted infection, and 4) a young man seeking sexual performance enhancement drugs. After each visit, the USP actors completed a 40‐item checklist that assessed PrEP promotion, behavioral risk assessment, counseling, medical safety assessment, HIV testing, PrEP dispensing, and service quality at the pharmacy. We descriptively analyzed our data.

**Results**: From February 2021 to August 2021, eight USPs completed 15 pharmacy PrEP visits. At these visits, 60% (9/15) of the USP were asked about their interest in PrEP and 80% (12/15) about behaviors associated with HIV risk. All USP actors (100%, 15/15) reported being counseled on PrEP safety and side effects and most (87%, 13/15) on the PrEP efficacy Almost all USP actors (87%, 13/15) were assessed for a history of kidney or liver disease and all (100%, 15/15) received assisted HIVST and PrEP in a private room during the visits. Of the USPs, 60%(9/15) agreed and 40%(6/15) strongly agreed that the quality of service at their visits was as expected.

**Conclusions**: The fidelity of PrEP delivery at private pharmacies in the pilot study was high, as assessed by USP actors. This suggests pharmacy providers can deliver high‐quality PrEP services supporting possible scale up of pharmacy‐based PrEP delivery models in Kenya and similar settings.

### PESAE03 Community Preparedness to embrace new Biomedical HIV Prevention Technologies. CHEDRA's Experience with the Dapivirine Vaginal Ring and Female Sex Workers (FSWs) amidst a volatile atmosphere in Masaka, Uganda

M. Kigozi ^1^, N. Georgina^2^

^1^CHEDRA (Community Health Empowerment Development and Relief Agency), Research and Advocacy, Masaka, Uganda, ^2^CHEDRA (Community Health Empowerment Development and Relief Agency), Health Education and Community Mobilization, Masaka, Uganda

**Background**: In Uganda, HIV prevalence is high among FSWs (37%) and their partners (18%). The brutal law against sex work creates institutional obstacles in accessing HIV prevention services for FSWs. Since 2015 todate, the International Partnership for Microbicides supports CHEDRA (Community Health, Empowerment, Development and Relief Agency) introduced the Dapivirine Vaginal Ring among FSWs within fishing communities to assess preparedness towards uptake of new Biomedical HIV prevention technologies.

**Description**: We popularized the Dapivirine Ring among key populations.‘Bar to Bar’, ‘With the Brothel’ and ‘Personalized HTS’, strategies were developed to intensify advocacy and awareness raising for the Dapivirine Ringas well as assessment for uptake and acceptance preparedness. Permission to operate from within brothels was obtained from owners, Trained front line staff used a snowball approach to penetrate FSWs networks, demonstration on how to use the vaginal ring, personalized HTS, STI management, Counseling, condom and contraceptive distribution was done within brothels. 314 FSWs were secretly mobilized, 154(49%) of them were between 14–22 years, others were above 23 years.283 (90%) of them received HTS, 108 (38%) of them were HIV positive. 65 (60%) enrolled in HIV care, all brothel owners yearned for our services. 100% of FSWs reported that the ring is the best HIV prevention option they had ever heard, those aged 26 and above showed much willingness to use it compared their younger counterparts, 100% of them desired to have a ring that would prevent both HIV and pregnancy.

**Lessons learned**: New biomedical HIV prevention strategies need to be tailor made to address gender specific imbalances in HIV prevention. ‘Within the Brothel’ and ‘Personalized HCT, strategies proven highly effective in increasing HIV prevention services to FSWs, Communities need to be educated about a new HIV prevention option even before it becomes available for public consumption.

**Conclusions/Next steps**: HIV prevention programs urgently need to widen HIV prevention options. No one size its all.

### PESAE04 Optimising provider‐initiated indicator condition guided testing for HIV to identify undiagnosed individuals: preliminary results of a multifaceted, multicentre intervention study

S. Bogers ^1^, M. Schim van der Loeff^1,2^, U. Davidovich^2^, A. Boyd^2,3^, M. van der Valk^1,3^, K. Brinkman^4^, K. Sigaloff^1^, J. Branger^5^, N. Bokhizzou^6^, G. de Bree^1^, P. Reiss^1,3,7,8^, J. van Bergen^9,10^, S. Geerlings^1^, on behalf of the HIV Transmission Elimination AMsterdam (H‐TEAM) Consortium

^1^Amsterdam University Medical Centers, Department of Internal Medicine, Amsterdam, Netherlands, the, ^2^Public Health Service of Amsterdam, Department of Infectious Diseases, Amsterdam, Netherlands, the, ^3^HIV Monitoring Foundation, Amsterdam, Netherlands, the, ^4^Onze Lieve Vrouwe Gasthuis, Department of Internal Medicine, Amsterdam, Netherlands, the, ^5^Flevoziekenhuis, Department of Internal Medicine, Almere, Netherlands, the, ^6^BovenIJ ziekenhuis, Department of Internal Medicine, Amsterdam, Netherlands, the, ^7^Amsterdam Institute for Global Health and Development, Amsterdam, Netherlands, the, ^8^Amsterdam University Medical Centers, Department of Global Health, Amsterdam, Netherlands, the, ^9^Amsterdam University Medical Centers, Department of General Practice, Amsterdam, Netherlands, the, ^10^STI AIDS Netherlands, Amsterdam, Netherlands, the

**Background**: In the Netherlands, approximately 7% of people with HIV (PWH) remained undiagnosed in 2020. Indicator Condition (IC)‐guided HIV testing is a cost‐effective strategy to identify such undiagnosed individuals. We implemented a multicentre intervention study in hospitals in the Amsterdam region to increase provider‐initiated IC‐guided HIV testing.

**Methods**: Two university hospitals, two teaching hospitals, and one non‐teaching hospital participated. Seven ICs were selected (Table 1). Relevant departments participated in a multifaceted intervention from June‐December 2020, including competitive audit and feedback and multimedia materials. Department‐specific solutions such as guideline adaptation, implementation of order sets and reflex‐testing varied by IC and hospital. HIV testing among patients ≥18 years not known to have HIV and presenting with an IC was assessed using electronic health records during pre‐intervention (January 2015‐May 2020) and post‐intervention (June 2020‐December 2021) periods. The primary endpoint was documentation of an HIV test ≤3 months around IC diagnosis. The difference in endpoints between periods was compared using logistic regression.

**Results**: Data from 8,017 patients were included. During the pre‐intervention period, HIV testing varied widely, (range 0.7%‐83.7%), and increased significantly in 4/7 ICs post‐intervention, but not in the two ICs with the highest‐ and the one with the lowest baseline testing (Table 1). Of 3,074 tested patients, 18 (0.6%) were HIV positive [4 (22%) female; median age 45 years (IQR = 34–54)], exceeding the consensus cost‐effectiveness threshold of 0.1%. Eight (44%) had tuberculosis, 7 (39%) malignant lymphoma, 2 (11%) hepatitis B and 1 (6%) hepatitis C. Median CD4 count at diagnosis was 97 cells/mm3 (IQR = 50–130); 94% had CD4 <350 cells/mm3.

**Table:** 

**Abstract PESAE04‐Table 1. Patients tested for HIV ≤3 months around indicator condition diagnosis, by period before and after intervention**						
Indicator condition	Tested before interventionn/N (%)	Tested after interventionn/N (%)	OR	95% CI	aOR*	95% CI
**Tuberculosis**	312/373 (83.7%)	57/65 (87.7%)	1.39	0.63 – 3.07	1.61	0.69 – 3.80
**cervical carcinoma/intraepithelial neoplasia grade III**	46/1065 (4.3%)	78/295 (26.4%)	7.96	5.38 – 11.79	14.30	9.04 – 22.62
**vulvar carcinoma/intraepithelial neoplasia grade III**	2/279 (0.7%)	0/56 (0.0%)	2.06	0.00 – 26.67	0.72	0.00 – 10.76
**Malignant lymphoma**	993/1580 (62.9%)	316/398 (79.4%)	2.28	1.75 – 2.97	2.27	1.73 – 2.98
**Hepatitis B virus**	514/798 (64.4%)	84/112 (75.0%)	1.66	1.06 – 2.60	1.64	1.02 – 2.62
**Hepatitis C virus**	345/491 (70.3%)	54/75 (72.0%)	1.09	0.63 – 1.87	1.07	0.61 – 1.88
**Peripheral neuropathy**	217/2041 (10.6%)	56/389 (14.4%)	1.41	1.03 – 1.94	1.74	1.24 – 2.45

^*^ *Odds ratio adjusted for hospital and patients’ sex, age category and socio‐economic status*

**Conclusions**: Overall HIV positivity underlined this testing strategy's cost‐effectiveness to identify undiagnosed PWH. IC‐guided testing improved in some, but not all ICs. Differences in intervention elements and observed effect by IC and hospital must be used for tailored, impactful strategies to optimise IC‐guided HIV testing by setting.

### PESAE05 Index testing approaches for early diagnosis of PLHIV and treatment initiation for HIV epidemic control

A. Enugu ^1^, P. Mugundu^1^, A.R. Yeruva^1^, A. Devanga^1^, S. Chintala^1^, J. Thakker^1^, A. Singh^1^, Aylur Kailasom^2^, S. Ghosh^2^, V. Arumugam^2^, V.R. Munagala^2^, R. Pollard^1^, A. McFall^3^, J. Bell^1^, S. Mehta^3^, S. Solomon^1^

^1^Johns Hopkins University, School of Medicine, Baltimore, United States, ^2^YR Gaitonde Centre for AIDS Research and Educa, ACCELERATE, Chennai, India, ^3^Johns Hopkins University, Bloomberg School of Public Health, Baltimore, United States

**Background**: Innovative case identification strategies remain critical for countries to achieve the UNAIDS fast track targets. Telangana is one of the Indian states with the highest HIV prevalence. Of the estimated 158,000 PLHIV in Telangana, ∼35% remain undiagnosed. We evaluate the impact of index testing as a case identification strategy.

**Description**: Under the PEPFAR/USAID funded ACCELERATE, index testing was implemented across 50 facilities (38 testing and 12 treatment) in five high prevalence districts of Telangana. Contacts were elicited from newly diagnosed clients at testing centers and PLHIV with either unsuppressed viral load, low CD4, demonstrated poor adherence, or previous loss‐to‐follow‐up at treatment centers. Contacts elicited were contacted and offered testing as per National guidelines.

**Figure:**
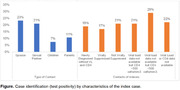
**Abstract PESAE05‐Figure 1**.

**Lessons learned**: From Aug 2020‐Dec 2021, from 8,868 index clients, 13,375 contacts were elicited and tested (7,200 sexual partners; 4,155 spouses; 1,973 children; and 47 mothers) of whom 2,634 (20%) were newly diagnosed with HIV infection. Positivity was highest among spouses (23%), followed by sexual partners (21%), parents (11%) and children (7%). There was also heterogeneity in positivity by district (range, 17–24%). Positivity was comparable by type of facility (testing vs, treatment sites = 19% vs 21%). However, within treatment sites, positivity among contacts of virally suppressed index clients, virally unsuppressed index clients and clients with no viral load data but CD4<500 cells/mm^3^were 17%, 21% and 29%, respectively. 2,433 (92%) of all new cases diagnosed were initiated on treatment.

**Conclusions/Next steps**: We demonstrated the efficiency of index testing in identifying and linking cases to ART at testing and treatment facilities. It is critical to ensure that all clients at treatment facilities undergo index‐testing when initiating ART with regular updating of contacts particularly for clients who are virologically unsuppressed. If scaled, such efforts could significantly support new case identification globally and achieve HIV/AIDS epidemic control.

### PESAE06 Scale‐Up of “Same‐Day ART Initiation” in South Africa

J. Bor ^1,2^, N. Jinga^2^, K. Shumba^2^, W. MacLeod^1,2^, M.P. Fox^1,2^, S. Rosen^1,2^, D. Onoya^2^

^1^Boston University, Boston, United States, ^2^Health Economics and Epidemiology Research Office (HE2RO), University of Witwatersrand, Johannesburg, South Africa

**Background**: Initiation of HIV antiretroviral therapy (ART) on day of diagnosis has been shown to increase treatment uptake and viral suppression in randomized trials. South Africa implemented “same‐day ART” in September 2017, a year after expanding ART eligibility to all patients under Universal Test‐and‐Treat (UTT). We assessed the impact of same‐day ART and UTT on time from diagnosis to ART initiation.

**Figure:**
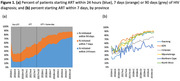
**Abstract PESAE06‐Figure 1**.

**Methods**: The study included all patients diagnosed with HIV at public‐sector health facilities in six South African provinces from 2016–2019 who started ART within 90 days. Data were extracted from the Three Interlinked Electronic Registers (TIER.Net) clinical database, and patients were followed up for 90 days. For each patient, we computed days from HIV diagnosis (or first clinical presentation with HIV) to ART initiation and defined this duration as same‐day (within 24 hours), rapid (≤7 days), or 90‐day. We stratified the analysis by province and CD4 count at presentation. We also assessed trends in the share of facilities that had adopted rapid ART as standard of care, which we defined as initiating at least 80% of patients within 7 days.

**Results**: Among 1,193,618 ART‐initiators who entered care between 2016 and 2019, the proportion starting ART on the same day and ≤7 days increased from 16% and 38% in 2016 to 70% and 87% in 2019, respectively (**Figure 1a**). The increase in same‐day and rapid initiation occurred starting during the Universal Test‐and‐Treat (UTT) in September 2016 and continuing with the formal implementation of same‐day ART in 2017. The pace of same‐day rollout varied by province (**Figure 1b**). In 2019, the share of patients starting ART within 7 days ranged from 53% in Northern Cape to 90% in KwaZulu‐Natal and Mpumalanga.

**Conclusions**: By 2019, over two‐thirds of people starting ART did so on the day they were diagnosed with HIV.

### PESAE07 Long term effects of donor transition processes and their potential influence on the realization of the country's 90‐90‐90 targets. Experiences from a subnational level in Uganda

E. Ssegujja ^1^, M. Mukuru^1^, H. Zakumumpa^1^, F. Ssengooba^1^

^1^School of Public Health, Makerere University, Health Policy Planning and Management, Kampala, Uganda

**Background**: Great improvements in the quality of HIV service delivery coupled with performance of some national key HIV/AIDS delivery indicators arising from donor support bestowed hope that realization of the 90‐90‐90 targets by 2030 were within reach. However, experiences from some districts following implementation of donor transition activities reflected potential threats to the country's ability to achieve some of the set targets. We explore this phenomenon in‐depth to document the long‐term effects of donor transition processes on HIV service provision at subnational level and its potential influence on the country's 90‐90‐90 aspirations over a ten‐year period.

**Methods**: A exploratory qualitative study was conducted between November 2021 and January 2022 in three districts representing three regions of the country that received donor support. Interviews were conducted among a purposively selected sample of 25 health managers and providers. Data was collected using audio recorders with analysis conducted following a thematic content analysis technique

**Results**: Results reflected mixed experiences with the first 90 of the population tested being compromised by withdrawal of community linkage facilitators which greatly affected the community HIV counselling and testing (HCT) activities. HIV services to key population were worst affected as they greatly rely on community component and having been set in project mode rendered it challenging to integrate into routine care. Disruptions in medical supplies and human resource for health following scale‐down of formally supported activities affected the second 90 of provision of appropriate care. However, whereas some previously supported health workers were absorbed into the government pay roll, not all were lucky particularly community linkage facilitators. Scale down of service delivery for the community component affected retention in care while disruptions in the hub system caused delayed delivery of test results which impacted clinical decision making.

**Conclusions**: Despite progress in realizing national 90‐90‐90 targets, transitional processes presented challenges to maintenance of these achievements. Effective transition calls for orderly and consultative processes beyond focusing more on set outcome targets calling for dedicated efforts to prioritize transition processes as a target for achievement in its self.

### PESAE08 High Rates of Interruptions in HIV Treatment in People Living with HIV on ART Less than Three Months Across the Age Continuum

M. Williams Sherlock ^1^, P. Bachanas^1^, L. Lee^2^, C. Biedron^1^, J. Aberle‐Grasse^1^, P. Agaba^3^, L. Bailey^2^, D. Connor^4^, J. Devine^2^, J. Stephens^2^, I. Zulu^1^, C. Godfrey^5^

 

**Figure:**
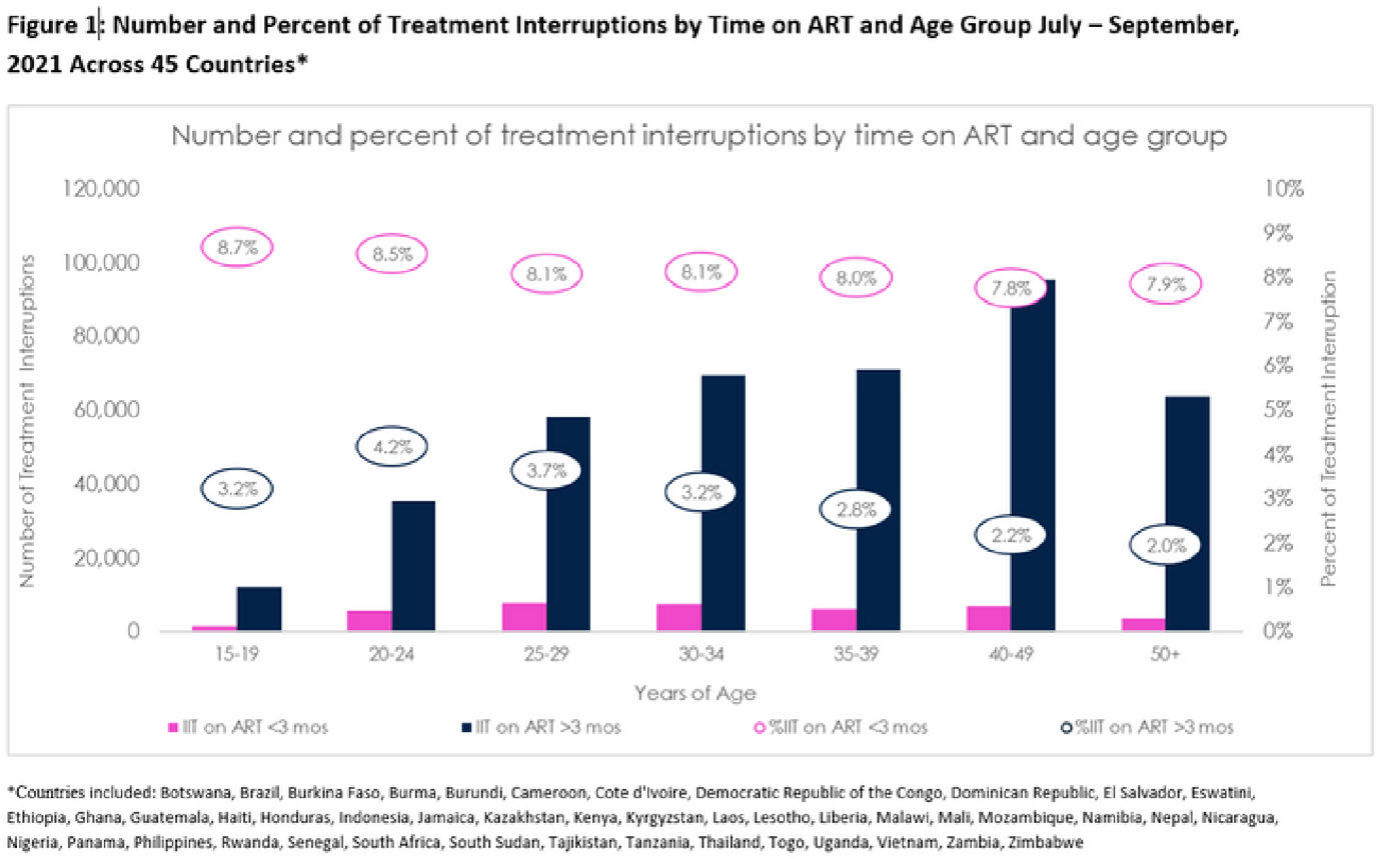
**Abstract PESAE08‐Figure 1**.

^1^U.S. Centers for Disease Control and Prevention, Division of Global HIV/AIDS and TB, Atlanta, United States, ^2^US Agency for International Development, Washington DC, United States, ^3^U.S. Military HIV Research Program, Walter Reed Army Institute of Research, Silver Spring, United States, ^4^Department of Defense, Defense Health Agency, DOD HIV/AIDS Prevention Program, San Diego, United States, ^5^Department of State, Office of the Global AIDS Coordinator, Washington DC, United States

**Background**: The importance of retaining people living with HIV (PLHIV) on treatment has been established. Emerging evidence indicates that many newly initiated persons experience treatment interruptions within the critical first few months after antiretroviral (ART) initiation. We analyzed treatment interruption rates among PLHIV on ART less than or more than three months in President's Emergency Plan for AIDS Relief‐ (PEPFAR) supported ART programs.

**Methods**: Aggregate routine program data from 45 countries receiving PEPFAR support for HIV services between July and September 2021 were included in this analysis. Interruptions in treatment (IIT) were defined as more than 28 days since a client's last expected clinic appointment or medication pick‐up date. Trends in IIT were examined by age, sex and time on ART for PLHIV aged 15 and older.

**Results**: Overall, 2.7% (292,722/10,790,728) of females and 3.0% (167,934/5,674,008) of males on ART experienced IIT during the last quarter of the fiscal year (July – September, 2021). PLHIV who were on ART less than three months experienced IIT approximately three times more often than PLHIV on ART more than three months (7.8% versus 2.6% p < .0001), respectively. Significant differences in IIT rates for females (8.0% and 2.6%, p < .0001) and males (7.6% and 2.8%, p < .0001) between time on ART disaggregates were also observed. Across all age groups of PLHIV on ART less than 3 months, IIT rates were significantly higher than of those on ART for more than three months (p < .0001 for each age group comparison; Figure 1).

**Conclusions**: The overall rate of treatment interruptions across PEPFAR‐supported programs was low during this investigation period. However, the risk of interruptions for those on ART less than three months is a critical challenge across sex and age groups. Targeted interventions for PLHIV newly initiating ART should be prioritized to ensure treatment continuity, especially in the era of multi‐month dispensing.

### PESAE09 Asynchronous prescribing of ART and antihypertensives results in frequent clinic visits despite multi‐month dispensing of ART in Malawi

H.S. Whitehead ^1^, C. Moucheraud^2^, K. Phiri^3^, B.A. Banda^3^, A. Moses^3^, J. van Oosterhout^3^, S. Phiri^3^, R.M. Hoffman^1^

^1^David Geffen School of Medicine at the University of California, Department of Medicine, Division of Infectious Diseases, Los Angeles, United States, ^2^Fielding School of Public Health, University of California, Department of Health Policy and Management, Los Angeles, United States, ^3^Partners in Hope, Lilongwe, Malawi

**Background**: While multi‐month dispensing (MMD) of antiretroviral therapy (ART) up to 6 months has become routine in Malawi, hypertension care often involves dispensing a 1–2‐month supply of medication, diminishing the benefits of MMD for ART. We assessed the degree of asynchronous dispensing in patients on ART and antihypertensives and estimated visit savings that would result from synchronized three‐ and six‐month prescribing of these medications.

**Methods**: We performed a cross‐sectional study of adults receiving care for both HIV and hypertension at an urban, PEPFAR/USAID‐supported clinic in Malawi between June‐December 2017. During this timeframe, the clinic provided integrated ART and hypertension care, with free ART and antihypertensives available for purchase. The quantity of ART and antihypertensive medications prescribed at individuals’ three most recent clinic visits was abstracted, and the median quantity for each medication was used to estimate the total number of refill visits per year (including visits where both meds were prescribed at the same time and any additional visits where prescribing of ART or antihypertensives would be required for a continuous supply). We then estimated visit savings that would result from synchronized three‐month and six‐month dispensing of ART and antihypertensives.

**Results**: We evaluated 193 adults (≥18 years): 65% female, median age 53 years (IQR 44–59), and median of 4 years (IQR 2–7) on antihypertensive medication. Four‐month dispensing was most common for ART (64.8%), while one‐month dispensing was most common for antihypertensive medications (59.6%). Only 16.6% of individuals received synchronous dispensing for both conditions at all three visits. The majority (62.2%) of participants were estimated to have 12 annual refill visits, 22.8% had 6–8 annual visits, and 15.0% had 3–4 annual visits. Under synchronized six‐month dispensing nearly two‐thirds of patients (64.8%) would eliminate 6+ visits. Under three‐month synchronized dispensing, 85.0% of respondents would eliminate at least 2 visits per year.

**Conclusions**: We found a high rate of asynchronous dispensing of ART and antihypertensive medication in an integrated care setting, with most individuals requiring monthly clinic visits despite MMD of ART. Expanding integrated care with synchronized MMD for stable individuals with HIV and hypertension has the potential to reduce patient and health system burdens.

### PESAE10 Using a robust approach to rapidly scale up integration of cervical cancer screening and treatment of pre‐cancerous lesions in HIV clinics

S. Namaganda Aleserwe Hope ^1,2^, B. Babirye^2^, J. Businge^3^, T. Nsubuga ‐Nyombi^4^, J. Nakaweesi^3^, S. Alger^4^, J.N. Kalamya^4^, C. Senyimba^3^, B. Mukasa^3^

^1^MildMay Uganda, Health, Kampala, Uganda, ^2^Mildmay Uganda, Kampala, Uganda, ^3^Mildmay Uganda, Health, Kampala, Uganda, ^4^CDC Uganda, Health, Kampala, Uganda

**Background**: Cervical cancer (CxCa) is the second most common cancer among women worldwide, causing significant mortality. In 2020, an estimated 604,237 women were diagnosed with CxCa globally. In Uganda, CxCa is the leading cause of cancer death. Women living with HIV (WLHIV) are 4–5 times more likely to develop CxCa. In October 2020, with support from CDC/PEPFAR, Mildmay Uganda (MUg) started implementing CxCa screening and treatment of precancerous lesions among WLHIV in central Uganda. This paper describes MUg's experience and successes in implementing the program.

**Description**: The CxCa screening program was implemented in 49 health facilities (HFs) in 8 districts of central Uganda. The program implemented from October 2020 to September 2021 targeted to reach 17,464 WLHIV aged 25 to 49 years. A needs assessment conducted to identify hindrances to CxCa screening found that HFs lacked equipment for screening and health workers lacked technical capacity to conduct screening and treatment of precancerous lesions. Mildmay provided the equipment, trained, and mentored the health workers. Two screening approaches used were visual inspection under acetic acid (VIA) at all HFs and Human Papilloma Virus (HPV) testing at 4 HFs with GeneXpert capacity.

**Lessons learned**: A total of 250 health workers from 49 participating HFs were trained on CxCa screening and treatment of precancerous lesions.

The number of WLHIV screened for CxCa markedly improved from 560 (3.2%) in Jan‐March to 14,101 (81%) in July‐September 2021. The positivity rate was 7% and treatment rate 83%. Twenty‐one women with suspicious lesions were supported to access biopsies. Five had invasive carcinoma and were referred for further management.

The scale up of differentiated service delivery models and multi‐ month dispensing of ARVs and COVID‐19 lockdown restrictions affecting access to HFs by clients and health workers were key challenges. Strategies implemented to mitigate these included pre‐appointment reminders, screening at community drug distribution points, cancer camps, mentorship, and flexi ‐hours for HPV testing and treatment

**Conclusions/Next steps**: It is feasible to integrate CxCa screening into HIV care using the screen & treat approach. The roll out was able to achieve 81% of the target despite the challenges from COVID‐19.

### PESAE11 Community antiretroviral therapy dispensation in Cameroon associated with superior client outcomes: A national evaluation

T. Epie ^1^, M. Bateganya^2^, P. Sadate‐Ngatchou^2^, L. Nishimoto^2^, M. Abass^2^, M. Nsoh^1^, P.‐P. Ewane^1^, M. Anastasie^3^, N. Kamgaing^3^, J.d.D. Anoubissi^3^, R. Wilcher^2^, H. Mahler^2^, V. Nzima^4^, L. Bonono^3^, S. Billong^3^

^1^FHI 360, Yaounde, Cameroon, ^2^FHI 360, Durham, United States, ^3^Cameroon National AIDS Control Committee, Yaounde, Cameroon, ^4^USAID, Washington D.C, United States

**Background**: The USAID‐ and PEPFAR‐funded Meeting Targets and Maintaining Epidemic Control (EpiC) project and the Government of Cameroon developed and evaluated a model in which some health facilities providing antiretroviral therapy offered clients the option to receive antiretroviral (ARV) drug refills at community‐based organizations (CBOs).

**Methods**: A mixed‐methods evaluation was conducted from October to December 2020 in10 regions of Cameroon to determine the CBO model's impact on client retention and viral suppression.

Retention was compared cross‐sectionally at 3, 6, 12, and 24 months between clients receiving ARV refills at 50 CBOs (n = 2633, 2549, 2425, 2063 respectively) and clients receiving refills at 38 health facilities offering the CBO option/model (offering facility) (n = 2017, 1916, 1805, 1606 respectively).

Additionally, retention at 3, 6, 12, and 24 months was compared between a cohort of clients receiving ARV refills at a subset of 3 health facilities offering the CBO model (offering facility) (n = 126) and 3 health facilities that did not (non‐offering facility) (n = 114).

Lastly, viral suppression was compared each year from 2016–2020 cross‐sectionally between clients receiving ARV refills at CBOs (n = 91, 217, 550, 664, 964 respectively) and at offering health facilities (n = 31, 130, 342, 347, 543 respectively). Program data from August 2014 to October 2020 was used for descriptive and inferential analysis.

**Results**: Clients receiving ARV refills at CBOs had higher retention than those at offering health facilities at 3 (94% vs. 90%, p‐value < 0.000), 6 (91% vs. 86.1%, p‐value < 0.000), 12 (86.6% vs. 81.1%, p‐value < 0.000), and 24 (86.1% vs. 72.2%, p‐value < 0.079) months. Clients receiving ARV refills at offering facilities had higher retention than at non‐offering facilities, but significantly only at 3 (100% vs. 93.1%, p‐value = 0.0013) and 24 months (90.5% vs.79.0%, p‐value = 0.0127). Similarly, viral suppression was higher among clients receiving ARV refills at CBOs than at offering health facilities each year, but significantly only in 2018 (98.6 vs. 92.4%, p‐value < 0.00) and 2020 (95.1% vs. 92.3%, p‐value = 0.02).

**Conclusions**: Dispensation of ARV through CBOs was associated with higher retention and viral suppression. The model has potential to improve clinical outcomes for clients who receive ARV refills at CBOs and those who continue to receive refills at health facilities offering the CBO option/model.

### PESAE12 Awareness of U = U among sexual and gender minorities in Brazil, Mexico, and Peru: Differences according to self‐reported HIV status

K. Konda ^1^, T.S. Torres^2^, H. Vega‐Ramirez^3^, O. Elorreaga^1^, C. Guillén‐Díaz‐Barriga^3^, D. Diaz^3^, B. Hoagland^2^, J.V. Guanira^4^, M. Benedetti^2^, C. Pimenta^5^, H. Vermandere^6^, S. Bautista‐Arredondo^6^, V.G. Veloso^2^, B. Grinsztejn^2^, C.F. Caceres^1^, for the ImPrEP Study Group

**Table:** 

**Abstract PESAE12‐Table 1**.						
Characteristics	PLHIV Model A	PLHIV Model B	HIV Negative Model A	HIV Negative Model B	Unknown HIV Status Model A	Unknown HIV Status Model B
Country (ref. Brazil) Mexico Peru	**1.04 [1.03–1.06]** **0.93 [0.88–0.98]**	**1.04 [1.02–1.05]** **0.92 [0.87–0.97]**	**1.02 [1.01–1.04]** **0.78 [0.75–0.82]**	1.01 [0.99–1.02] **0.79 [0.76–0.83]**	1.01 [0.95–1.08] **0.59 [0.51–0.68]**	0.95 [0.89–1.00] **0.60 [0.51–0.71]**
Trans/non‐binary (ref. Cisgender men)	0.96 [0.91–1.01]	0.97 [0.92–1.02]	**0.91 [0.88–0.95]**	**0.95 [0.92–0.99]**	0.89 [0.77–1.04]	0.90 [0.77–1.06]
Age 25+ (ref. Age 18–24)	1.00 [0.96–1.03]	0.98 [0.95–1.02]	1.00 [0.98–1.02]	1.01 [0.99–1.03]	0.97 [0.90–1.03]	0.96 [0.90–1.02]
Black/Mixed‐race (ref. White)	**0.98 [0.96–1.00]**	0.99 [0.97–1.00]	**0.98 [0.97–1.00]**	**0.98 [0.98–1.00]**	0.95 [0.90–1.01]	0.95 [0.89–1.01]
<Secondary education (ref. >0 = secondary)	0.97 [0.91–1.03]	0.96 [0.90–1.02]	**0.86 [0.81–0.90]**	**0.91 [0.86–0.96]**	**0.79 [0.66–0.95]**	0.86 [0.71–1.04]
<Minimum wage (ref. > = Minimum wage)	**0.97 [0.94–0.99]**	0.98 [0.96–1.00]	**0.94 [0.92–0.95]**	**0.95 [0.93–0.96]**	**0.93 [0.87–1.00]**	0.95 [0.88–1.01]
ART use / PrEP use	‐	**1.18 [1.09–1.26]**	‐	**1.07 [1.06–1.08]**	‐	0.98 [0.75–1.29]
High HIV risk perception	‐	‐	‐	0.99 [0.97–1.01]	‐	1.02 [0.91–1.14]
> = 10 (ref. <10, MSM risk index)	‐	‐	‐	**1.01 [1.01–1.03]**	‐	1.01 [0.95–1.07]

^1^Universidad Peruana Cayetano Heredia, Centro de Investigación Interdisciplinaria en Sexualidad, Salud, y SIDA, Lima, Peru, ^2^Instituto Nacional de Infectologia Evandro Chagas, Fundação Oswaldo Cruz (INI‐Fiocruz), Rio de Janeiro, Brazil, ^3^Instituto Nacional de Psiquiatria Ramon de la Fuente Muñiz, Mexico City, Mexico, ^4^Inmensa, Lima, Peru, ^5^Brazilian Ministry of Health, Departmento de Doenças de Condições Crônicas e Infecções Sexualmente Transmissiveis, Rio de Janeiro, Brazil, ^6^Instituto de Salud Pública (INSP), Mexico City, Mexico

**Background**: The slogan “Undetectable = Untransmittable” (U = U) was created to translate the message that people living with HIV (PLHIV) using antiretroviral treatment (ART) with undetectable viral load will not transmit HIV to sex partners. We describe U = U awareness among sexual and gender minorities (SGM) in Latin America and investigate differences by self‐reported HIV status.

**Methods**: We conducted an online survey among SGM (≥18 years) living in Brazil, Mexico, and Peru during 2021. We used Poisson regression to calculate prevalence ratios of factors associated with awareness of U = U stratified by self‐reported HIV status (PWH, negative and unknown). First, we estimated initial models (Model A), including socio‐demographic factors (country, gender, age, race, education, and income) and then subsequent models (Model B), including risk behavior, ever taking PrEP, and HIV risk perception for HIV‐negative/unknowns or taking ART for PLHIV.

**Results**: A total of 21,374 respondents were included (Brazil: 61%, Mexico: 30%, Peru: 8%); median age was 32(IQR: 26–39), 96% cisgender man, 57% Black/Mixed‐race; 3% <secondary education and 23% <minimum wage. Among HIV‐negative (16338/21374; 76%) and unknown status (2169/21374; 10%) SGM, 9% reported high self‐perceived HIV risk, 58% were classified as high‐risk for HIV, and 13% ever used PrEP. Among HIV‐positive SGM (2867/21374; 10%), 93% were using ART. Awareness of U = U was 89% in both Brazil and Mexico, higher than Peru 65%. Awareness of U = U was higher among HIV‐positive (96%) than negative (88%) and unknown (69%) SGM. In multivariate models, U = U awareness was lower among lower‐income and Black/Mixed‐race SGM. Among HIV‐negative SGM, trans/non‐binary had lower awareness of U = U compared to cisgender men, while those reporting high HIV‐risk and ever using PrEP had higher awareness.

**Conclusions**: Awareness of U = U varied according to HIV status, sociodemographic characteristics, and HIV risk behavior. Broad educational strategies, including teaching U = U, focusing on SGM vulnerable to HIV infection are urgent to decrease stigma against PLHIV.

### PESAE13 Changes in “Undetectable Equals Untransmittible” (U = U) Knowledge and Practices by HIV and PrEP Status Among Gay, Bisexual, Queer, and Trans Men and Two‐Spirit and Non‐Binary People Across Canada, 2015–2021

N. Lachowsky ^1,2^, A. Hu^1^, C. Draenos^2^, B. Klassen^2^, K. Card^2,3^, R. Higgins^1,2^, F. Ibáñez‐Carrasco^4^

^1^University of Victoria, Public Health & Social Policy, Victoria, Canada, ^2^Community Based Research Centre, Vancouver, Canada, ^3^Simon Fraser University, Burnaby, Canada, ^4^University of Toronto, Toronto, Canada

**Background**: Canada officially endorsed “Undetectable equals Untransmittible” (U = U) in 2018, but few longitudinal and nation‐wide studies on the impacts exist. We sought to examine population‐level trends of U = U‐related knowledge and sexual behaviour among gay, bisexual, trans, Two‐Spirit, and queer men and non‐binary people (GBT2Q) across Canada, by HIV and pre‐exposure prophylaxis (PrEP) status.

**Methods**: Data are from community‐based repeated cross‐sectional bilingual (English/French) surveys: 2015 (online), 2018 (pride festivals), 2019 (online), 2020 (online), and 2021 (online). Online recruitment used advertisements on sociosexual websites/apps, and community‐based organizations’ social media and email lists. Eligible participants were at least 15 years old, lived in Canada, and either identified as non‐heterosexual or reported recent sex with a man. Women were ineligible. Temporal trends were evaluated using separate multivariate logistic regressions by HIV/PrEP status, with survey year (continuous) as the primary explanatory variable, and controlling for age, education, ethnoracial identity, sex/gender identity (cisgender man, transgender man, non‐binary), and number of recent sexual partners (for behavioural outcomes). Adjusted odds ratios (AOR) with 95% confidence intervals are shown.

**Results**: The pooled sample included 24,160 responses: 8.6% from GBT2Q living with HIV, 14.4% HIV‐negative PrEP users, and 77.1% HIV‐negative non‐PrEP users. Knowledge that HIV medications effectively suppress viral load increased from 2015–2021 for those living with HIV (68.8%‐100%, AOR = 3.43 [2.69–4.37]), PrEP users (70.3%‐97.3%, AOR = 1.77 [1.56–1.99]), and non‐PrEP users (69.6%‐84.1%, AOR = 1.26 [1.22–1.27]). Knowledge of the U = U scientific consensus on sexual transmission increased from 2018–2021 for those living with HIV (95.2%‐98.3%, AOR = 1.61 [1.04–2.47]), PrEP users (92.3%‐95.5%, AOR = 1.20 [1.02–1.42]), and non‐PrEP users (68.9%‐77.7%, AOR = 1.25 [1.19–1.30]). Reporting recent anal sex with an undetectable partner decreased from 2018–2021 for those living with HIV (55.3%‐52.7%, AOR = 0.78 [0.67–0.90]), PrEP users (46.3%‐24.5%, AOR = 0.73 [0.68–0.79]), and non‐PrEP users (7.2%‐6.8%, AOR = 0.91 [0.84–0.99]) after additionally controlling for number of recent sex partners. In 2021, participants living with HIV reported whether U = U impacted stigma (37.9% decreased, 55.6% no change, 6.5% increased) and access to sexual partners (14.2% decreased, 57.9% no change, 28.0% increased).

**Conclusions**: While U = U knowledge increased over time among GBT2Q, behavioural uptake remains incommensurate. The self‐reported impacts of U = U for GBT2Q living with HIV are mixed, and require further investigation.

### PESAE14 U = U awareness promotes engagement in HIV care among HIV Negative men who have sex with men in Mississippi and Alabama

T. Mckay, Phd ^1^, J.C. Henne^2^, C. Shinners^2^, S. Schwartz, MBA^2^, A. Dyson, PharmD^3^, T. Evans^3^, M.C. Penner, BSW^4^, C. Kinker^5^, R.C. Quarles, PhD^6^

^1^Vanderbilt University, Department of Medicine, Health, and Society, Nashville, United States, ^2^The Henne Group, Inc., San Francisco, United States, ^3^ViiV Healthcare, Research Triangle Park, United States, ^4^Prevention Access Campaign, Washington, D. C., United States, ^5^Prevention Access Campaign, New York, United States, ^6^Q Catalytics, Arlington, United States

**Background**: Expanding public awareness of the “Undetectable = Untransmittable” (U = U) message is central to a status neutral approach prioritizing engagement with health care providers (HCP) to monitor HIV status and viral load. This study tests the dissemination of a U = U communication campaign on HCP engagement among HIV negative men who have sex with men (MSM) in Alabama and Mississippi, two US states with high rates of new HIV diagnoses and deaths.

**Methods**: We use a two‐group pre/post study design with data from MSM in Alabama and Mississippi. Three interactive social media ads designed by Prevention Access Campaign (PAC) were run in both states from April to June 2021. In Mississippi, PAC ambassadors provided additional peer‐to‐peer engagement around U = U from February to May 2021. Survey data were collected from December 2020 to April 2021 (N = 801; pre) and June to July 2021 (N = 504; post) with similar retention across states (62% vs 64%). HIV negative men comprised 72% of the sample. Analyses below are restricted to HIV negative men who completed follow‐up (N = 368).

**Results**: Before campaign exposure, 38% of HIV negative men reported having an HCP monitoring their HIV status and 29% were aware of and understood U = U. Campaign exposure significantly increased overall U = U awareness with comprehension (29% to 52%), with larger increases in Mississippi (28% to 41%) than Alabama (30% to 38%). One in five (20%) HIV negative men who were unaware of U = U and reported no HCP monitoring their HIV status before the campaign (N = 175) reported having an HCP at follow‐up. Men who experienced a positive change in U = U awareness at follow‐up were three times more likely to also report a positive change in having an HCP monitoring HIV status (OR 3.27; 95% CI = 1.51–7.08). The effect of the U = U campaign is larger in Mississippi, where 73% of newly U = U aware men also reported a new HCP connection compared with 47% in Alabama.

**Conclusions**: Engagement with care is an important step for improving timely HIV testing and PrEP uptake for at‐risk MSM. The U = U communication campaign increased HCP engagement among HIV negative men, with larger increases in Mississippi where men received the social media campaign with peer‐to‐peer outreach.

### PESAE15 Amazon smart locker collaboration as a pick up point preference for medicine distribution programs in Sub‐Saharan Africa

S. Hendriksz ^1^, B. Meyer^1^, C. Kraamwinkel^1^

^1^Right e‐Pharmacy, Pretoria, South Africa

**Background**: The Collect & Go Smart Locker Solution serves as a Pick‐up Point (PuP) for pre‐dispensed medicines from Centralised Dispensing Facilities with built in monitoring and reporting capabilities. There are currently 101 Smart Locker sites located in South Africa, Lesotho, Eswatini, Zambia and Botswana. In response to the COVID‐19 pandemic, PuPs were required to encourage medication collection outside overburdened facilities. Right ePharmacy deployed Collect & Go Smart Locker PuPs as an alternative and scalable solution for various distribution programs, including ART refills.

**Description**: Commodity distribution programs in Africa utilises various PuPs to cater for patient and program needs. Alternative PuPs are a priority to alleviate burden on clinical staff and to reduce patient waiting times and exposure. Collect & Go Smart lockers further reduce waiting times and eliminate interpersonal contact, ultimately improving patient experience. In May 2020 the 1st smart lockers were deployed and continue to be scaled due to patient preference, positive impact on adherence and accurate supply chain management capabilities.

**Lessons learned**: apid uptake of Collect & Go lockers were observed in Southern Africa with more than 250 000 prescriptions loaded and 220 000 successfully collected by January 2022. Smart lockers addressed critical challenges related to patient and commodity management, decreasing exposure risk and increasing capacity, with the goal to facilitate more convenient and safe collection practices for patients.

**Figure:**
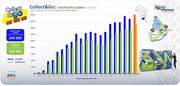
**Abstract PESAE15‐Figure 1**.

**Conclusions/Next steps**: While alternative PuPs remain a critical solution for convenient access to chronic and HIV medication, the locker solution does provide an additional layer of patient satisfaction in terms of preference for quality, privacy and safety when compared to other PuP options. The integrated temperature control unit, technology and parcel monitoring also allows for accurate commodity and supply change management, and ultimately positive patient adherence management. In addition, this solution proved to be an ideal solution to limit exposure during the COVID‐19 pandemic.

### PESAE16 Impact of a community health worker intervention on PrEP knowledge and utilization in Rakai, Uganda: a mixed methods assessment

A. Edwards ^1^, R. Pollard^1^, C.E. Kennedy^1,2^, G. Nakigozi^2^, J. Mulamba^2^, I. Mbabali^2^, A. Anok^2^, X. Kong^3,4,5^, N. Nakyanjo^2^, W. Ddaaki^2^, H. Nishimura^1^, M. Wawer^2,3,6^, L.W. Chang^1,2,3,6^

^1^Johns Hopkins Bloomberg School of Public Health, International Health, Baltimore, United States, ^2^Rakai Health Sciences Program, Rakai, Uganda, ^3^Johns Hopkins School of Medicine, Division of Infectious Diseases, Baltimore, United States, ^4^Wilmer Eye Institute, Johns Hopkins School of Medicine, Baltimore, United States, ^5^Johns Hopkins Bloomberg School of Public Health, Biostatistics, Baltimore, United States, ^6^Johns Hopkins Bloomberg School of Public Health, Epidemiology, Baltimore, United States

**Background**: Highly mobile individuals living in Ugandan fishing communities face increased HIV acquisition risk. From 2015–2018, the Rakai Health Sciences Program (RHSP) implemented a novel mHealth, motivational interviewing‐informed counseling intervention led by community health workers (CHW) to improve uptake of HIV treatment and prevention services in an HIV hyperendemic fishing village. Following introduction of PrEP in 2017, a PrEP counseling module was incorporated into the intervention to increase PrEP awareness and uptake. This mixed‐methods study evaluated the intervention's impact on PrEP knowledge and utilization.

**Methods**: Survey data were collected from all available community members aged 15–49 through the Rakai Community Cohort Study. Multivariable logistic regressions with generalized estimating equations were used to estimate the effect of the CHW intervention on PrEP knowledge and use. We also conducted 74 in‐depth interviews with 5 types of informants: clients, CHWs, program staff, community leaders, and health clinic staff. Transcripts were analyzed using a deductive, iterative approach. Findings were triangulated with programmatic data from an electronic mobile application used by CHWs during counseling visits.

**Results**: 1848 individuals were surveyed; 46% were female; mean age was 31.8 years (SD: 8.2). In as‐treated analyses, individuals exposed to the CHW intervention reported significantly higher PrEP knowledge (N = 1848, PRR: 1.10, 95% CI: 1.06–1.14, p = <.0001), lifetime PrEP use ((N = 1176 (HIV‐negative participants only) PRR: 1.77, 95% CI: 1.33–2.36, p = <.0001)), and current PrEP use (N = 1176, PRR: 1.86, 95% CI: 1.22–2.82, p = 0.0039) compared to those unexposed. Qualitative data supported quantitative findings and attributed positive PrEP outcomes to CHW counseling and effective use of motivational interviewing skills by CHWs. Barriers to PrEP uptake included misinformation about PrEP, HIV‐related stigma, and pill burden. Mobile application data demonstrated that the PrEP module was delivered consistently by CHWs throughout the implementation period.

**Conclusions**: CHWs positively influenced PrEP knowledge, use, and retention among clients of a motivational‐interviewing informed counseling intervention in an HIV hyperendemic fishing community. A mixed methods approach provided important insights to inform implementation of future PrEP programs. Findings suggest use of CHWs and motivational interviewing‐informed counseling can be effective components of interventions to improve PrEP outcomes.

### PESAF01 The role of the *Canadian Coalition to Reform HIV Criminalization* in changing Canada's approach to HIV criminalization: 5 years of community‐led strategies, advocacy and consensus‐building

I. Annamanthadoo^1,2^, C. Clarke^2^, R. Elliott^1,2^, C. Johnson^2^, C. Kazatchkine^1,2^, A. McClelland^3,2^, R. Peck^4,2^, L. Pelletier‐Marcotte ^5,2^, S. Rahim^6,2^

^1^HIV Legal Network, Toronto, Canada, ^2^Canadian Coalition to Reform HIV Criminalization (CCRHC), Toronto, Canada, ^3^Carleton University, Ottawa, Canada, ^4^HIV & AIDS Legal Clinic Ontario (HALCO), Toronto, Canada, ^5^Coalition des Organismes Communautaires Québécois de Lutte Contre le Sida (COCQ‐SIDA), Montréal, Canada, ^6^Kastner Lam LLP, Toronto, Canada

**Background**: Canada has one of the world's highest number of recorded HIV‐related criminal cases, resulting from a broad interpretation of the sexual assault provisions in the *Criminal Code*. The most commonly laid charge in cases of alleged non‐disclosure is *aggravated sexual assault*, where conviction carries a maximum penalty of life imprisonment and mandatory registration as a sex offender. People living with HIV (PLWH) have been convicted even in cases where no transmission occured, when they used a condom or had an undetectable viral load.

**Description**: In 2016, a number of organizations and advocates, including people with lived experience of HIV criminalization, created the Canadian Coalition to Reform HIV Criminalization (CCRHC) to change Canada's approach to HIV criminalization. In 2017, CCRHC conducted its first national consultation, leading to a widely endorsed Community Consensus Statement that makes specific demands to the federal and provincial governments to limit HIV criminalization.

**Lessons learned**: Involving people with lived experience and adapting strategies to shifting (political and social) landscapes are key to the CCRHC's work, and led to positive developments. In 2017, Justice Canada made recommendations to both federal and provincial officials to limit HIV criminalization. In 2018, following a call for action by the CCRHC, the Attorney General of Canada (AGC) issued a (limited) directive based on Justice Canada's recommendations. In 2019, CCRHC members testified before the House of Commons Standing Committee on Justice and Human Rights (SCJHR) on the issue of HIV criminalization. The SCJHR majority report concluded that Canada's approach is too broad, too punitive and unscientific. It recommended limiting the use of criminal law in cases of HIV non‐disclosure, including through *Criminal Code* reform. In 2021, the CCRHC launched a second community consultation on *Criminal Code* reform advocacy. Since 2017, some provinces have also adopted policies or directives to limit HIV‐related prosecutions.

**Conclusions/Next steps**: While significant, these developments remain insufficient to end the overbroad criminalization of PLWH in Canada. While results from the ongoing consultation around Criminal Code reform will inform advocacy strategies at the federal level, CCRHC must continue to demand concrete actions from both levels of government to implement recommendations to limit HIV criminalization.

### PESAF02 Harms of Sex Offender Registries in Canada among people living with HIV

L. Michaud ^1^, I. Annamanthadoo^2^, S. Ka Hon Chu^2^, A. McClelland^3^, R. Nobleman^4^, R. Peck^4^

^1^York University, Toronto, Canada, ^2^HIV Legal Network, Toronto, Canada, ^3^The Institute of Criminology and Criminal Justice, Carleton University, Toronto, Canada, ^4^HIV & AIDS Legal Clinic Ontario, Toronto, Canada

**Background**: The number of prosecutions of people living with HIV in Canada for alleged HIV non‐disclosure is among the highest in the world, and Canada is unique in applying aggravated sexual assault charges in the majority of these cases. Following amendments to Canada's Criminal Code in 2011, all individuals living with HIV convicted of aggravated sexual assault for non‐disclosure became subject to mandatory sex offender registration, in many cases for life.

**Methods**: This research is based on qualitative interviews conducted from 2017 to 2019 with 16 people living with HIV from across Canada who have faced criminalization and incarceration for alleged non‐disclosure of their HIV status, and compulsory registration with the National Sex Offender Registry due to their conviction. We combined this qualitative research with a legal analysis of the interplay between federal legislation, correctional policy, and the related regulatory practices of law enforcement.

**Results**: Mandatory sex offender registration triggers substantial harms among people living with HIV, further exacerbating the harms caused by ongoing criminalization and incarceration of non‐disclosure. These harms include: family estrangement and internalized stigma, negative impacts on re‐entry and reintegration, an excessive burden due to onerous registration requirements, as well as psychological harms arising from potential lifetime government and police surveillance. These impacts also interact with – and are reinforced by – harms resulting from news media reporting of sex offender status to the public and resulting stigma and social violence, as well as psychological harms from inappropriate sex offender treatment stemming from correctional policy.

**Conclusions**: This study provides an assessment of the complexity of interlocking legal and social harms experienced by people living with HIV as a result of sex offender designation and registration. The research makes visible and contextualizes these harms, providing important knowledge for individuals supporting and caring for people living with HIV who have been subject to criminalization. Our analysis also offers critical knowledge related to how sex offender registration laws operate in the daily lives of people living with HIV for individuals engaged in advocacy and law reform regarding the criminalization of HIV non‐disclosure.

### PESAF03 Flawed by Design: Canada's first Prison Needle Exchange Program

S.K.H. Chu^1^, R. Elliott ^1^, S. Simons^2^, J. Rowe^3^, L. Edmiston^4^, T. Stratton^5^

^1^HIV Legal Network, Toronto, Canada, ^2^Former Prison Peer Health Worker, Toronto, Canada, ^3^PASAN, Toronto, Canada, ^4^CATIE, Toronto, Canada, ^5^Communities, Alliances and Networks (CAAN), Fort Qu'Appelle, Canada

**Background**: Prisoners have long been recognized as a “key population” disproportionately affected by HIV. Yet despite ample evidence demonstrating the effectiveness of needle and syringe programs in reducing the risk of HIV infection and other harms to prisoners’ health, for decades Correctional Service Canada (CSC) refused to implement this essential harm reduction measure in prison. This had an especially severe impact on the disproportionate number of Indigenous people in federal prisons, and particularly Indigenous women in prison — 11.7% of whom are reported to be living with HIV.

**Description**: In 2012, a former prisoner and four HIV organizations challenged CSC's failure to provide prisoners with equivalent access to sterile injection equipment as breaching their constitutional rights to life, liberty, and security of the person and to equal benefit of the law without discrimination. Eventually, in response, the federal government announced that it would introduce a prison needle exchange program (PNEP) in two prisons in 2018, and gradually extend the program to all federal prisons.

**Lessons learned**: Serious deficiencies in the PNEP's design have impeded access to the program. Participation involves a four‐step assessment process that requires disclosure of one's drug use to the prison warden, among others — without any guarantee of approval of participation. Those approved to participate are subject to daily “visual inspections” by security staff to verify accountability for the equipment distributed, breaching prisoners’ confidentiality regarding their participation. CSC's own interim evaluation revealed extremely low rates of participation, and as of September 2021, only 9 (out of 43) federal prisons were identified by CSC as having a functioning PNEP, despite CSC's initial pledge to fully implement the program by August 2020. Of those 9 prisons, CSC identified only 43 people participating in the program across four prisons, and only two participants in one women's prison.

**Conclusions/Next steps**: CSC's PNEP is seriously compromised by design deficiencies, low uptake and the indefinite suspension of its roll‐out during the COVID‐19 pandemic. There is an urgent need to continue monitoring the program and address barriers to participation. If the benefits of a PNEP are to be realized, prisoners need easy, confidential access.

### PESAF04 “Investigating premarital HIV testing in Indonesia and its risks of human rights violations”

N. Puspitasari ^1^, A. Wirya^1^, A. Larasati^2^

^1^Community Legal Aid Institute, Jakarta, Indonesia, ^2^Harm Reduction International, London, United Kingdom

**Background**: HIV programs in Indonesia often place families as the basis of intervention, believing that resilient families will result in HIV prevention. One of such policies is mandatory premarital HIV testing. This study aims to review this intervention, using the lens of human rights.

**Methods**: This research uses a qualitative method, combining literature reviews and interviews. The literature includes laws, modules, and healthcare guidelines. In total, the researchers collected 218 laws, spanning across different provinces in Indonesia. The data was then complemented by online interviews with stakeholders and civil societies.

**Results**: Fourteen regions have local regulations imposing premarital HIV testing. There are four forms of the regulation, namely (1) mandatory premarital HIV testing, (2) recommendations for testing through the provision of information, (3) mandatory health check, or (4) mandatory notification to the partner of HIV status. Most of them require people who want to get married to provide health certificates detailing their health alongside recommendations from doctors. Clients are often not free to opt‐out of the test since the Office of Religious Affairs or the Civil Registration Service is only willing to marry people when there is a health certificate. This requirement results in marriage cancellations in Gorontalo, Banyuwangi, and Jakarta. In addition, when the test result is positive, a health certificate will be issued only when the person is willing to undergo ARV therapy. The test also does not guarantee one's right to privacy since the notification of HIV status is conducted together with the partner. Some displayed a lack of risk mitigation efforts, causing reluctance to undergo the test for fear of being abandoned or experiencing violence, especially when the positive is the bride.

**Conclusions**: Mandatory premarital HIV testing, which has been implemented in various regions in Indonesia and used different strategies to compel people to take HIV tests poses many human rights issues. It is not based on voluntariness that should be the underlying principle of HIV tests. Without comprehensive risk mitigation strategies, this effort could potentially result in violence, stigma and discrimination from partners—counterproductive to the idea of ‘making the family resilient’.

### PESAF05 An Advocacy Campaign to Increase Access to Post‐Exposure Prophylaxis (PEP) through Expansion of Pharmacist Scope of Practice in North Carolina

M. Martin ^1^, D. Ward, JD^2^, D. Rowan, Ph.D., MSW, LCSW^3^, G. Wilkins, RN, MPA, MSN, FNP‐BC^4^, L. Storrow^5^

^1^NC AIDS Action Network, Raleigh, United States, ^2^Southern AIDS Coalition, Bluffton, United States, ^3^University of North Carolina at Charlotte, School of Social Work, Charlotte, United States, ^4^East Carolina University, Brody School of Medicine, ECU Adult Specialty Care, Greenville, United States, ^5^Community Education Group, Charleston, United States

**Background**: Since 2010, North Carolina AIDS Action Network (NCAAN) has led HIV/AIDS treatment and prevention advocacy/lobbying efforts in North Carolina (NC), a state in the southern United States. In 2020 & 2021, NCAAN partnered with Southern AIDS Coalition (SAC) to lead an educational advocacy campaign to increase access to Post Exposure Prophylaxis (PEP) through policy change that expands the scope of practice of NC pharmacists to prescribe PEP medications.

**Description**: In 2020 there were 1,079 reported new cases of HIV in NC, with disproportionate incidence in rural counties. There are five rural counties without a family physician, while licensed community‐based pharmacists often serve as the main healthcare contact. There is a negative correlation between the availability of specialized medical services and the rate of incidence of HIV transmission. Therefore, expanding the scope of practice of pharmacists to prescribe PEP promotes public health outcomes in rural and other under‐resourced communities.

To build support for pharmacists prescribing PEP, NCAAN hosted virtual advocacy trainings and organized an advocacy day at the NC state capitol. Advocates met with NC legislators to discuss HIV trends and barriers to care in rural communities. Professional lobbyists worked closely with key legislators and medical associations to ensure expansion of pharmacist scope of practice was included in key legislation introduced in 2021.

**Lessons learned**: Based on interviews with key legislators, lobbyists, and stakeholders, NCAAN and SAC's joint advocacy efforts were critical to the passage of NC State House Bill 96 (2021), which allows NC pharmacists to prescribe PEP and other public health‐promoting agents. Framing PEP as medication to be prescribed with urgency and demonstrating support from other medical providers was politically strategic and helped generate support from more conservative, Republican lawmakers.

**Conclusions/Next steps**: In 2022, NCAAN and SAC will partner with the NC Association of Pharmacists to train pharmacists on their new authority in the expanded scope of practice, with a focus on rural communities that have limited PEP prescribers. This successful advocacy case study serves as a model for other states and communities on advancing policy change to increase access to HIV biomedical prevention tools.

### PESAF06 Community‐led law making in HIV field as a Ukrainian know‐how

V. Rachynska ^1^

^1^All‐Ukrainian Network of PLWH, Kyiv, Ukraine

**Background**: Over the last 15 years only 3 laws regulating issues in the field of HIV and key populations were adopted in Ukraine. While the world is moving forward protecting the rights of PLHIV and other KPs legislation of our country slows down this progress being an impediment to innovations in medicine, decreasing retention in treatment and strengthening stigma towards PLWH and KPs.

**Description**: For acceleration of changes in laws most relevant to the interests of PLWH and other key affected populations a Parliamentary platform “Fight for Health” was established.

Taking into account the fact that key populations should be the final beneficiary and the main drivers of the mentioned changes the structure of the Platform includes:

9 Platform Community representatives collecting the needs of key communities (WHIV, AHIV, ex‐prisoners, SW, MSM, TG, PWID; PLHIV, TB) on national level;
Lawyers developing draft bills and recommendations on changes to legal acts for submission to the Parliament, Ministries, CCM and regional Coordinating bodies based on needs ofKPs;
Coordination of activities is conducted by supervisors. Supervisors communicate with Parliamentarians, Ministries, CCM presenting proposals of communities;
27 Community representatives taught to collaboration with Legislative, Executive and Judicial branches of power at the School of Power.
Technical support visits to regions improve communication with communities and regional coordinating bodies at regional level.

**Lessons learned**: The intervention has had the following outcomes:

5 draft bills for decriminalization of sex work with 1 parliamentarian as a lead;
3 draft bills for decriminalization of HIV with 3 parliamentarians as leads;
a draft bill on HIV and a Parliament Committee with participants representing key communities;
a draft bill on decriminalization of drug use;
a concept and draft bill on civil partnerships for LGBTIQ+;
communities of Trans* people, women and adolescents living with HIV developed and submitted their proposals for changes in the regulatory documents of different relevant Ministries.
community of ex‐prisoners initiated penitentiary system reform on all levels.

**Conclusions/Next steps**: Further work of the Platform will focus on passing of the draft bills by the Parliament of Ukraine and strengthening capacity of KPs in law making initiatives improving their lives and protecting their rights.

### PESAF07 Strengthening youth voices for change: The role of advocacy training in enhancing meaningful engagement of young people in youth‐friendly HIV service provision

C. Zinyemba ^1^, R. Igweta^2^, C. Audi^3^, H. Paul^4^

^1^Elizabeth Glaser Pediatric AIDS Foundation, Public Policy & Advocacy, Harare, Zimbabwe, ^2^Elizabeth Glaser Pediatric AIDS Foundation, Public Policy & Advocacy, Harare, Kenya, ^3^Elizabeth Glaser Pediatric AIDS Foundation, Public Policy & Advocacy, Washington DC, United States, ^4^Elizabeth Glaser Pediatric AIDS Foundation, Public Policy & Advocacy, Kampala, Uganda

**Background**: In 2020, 3.3 million young people were living with HIV. To meet the global goal of ending AIDS by 2030, specific attention must be paid to adolescents and youth living with HIV, including provision of youth‐responsive programming and meaningful engagement of adolescent and youth voices in decision‐making fora

**Description**: The Elizabeth Glaser Pediatric AIDS Foundation, supported through ViiV Breakthrough Partnership project, led an advocacy training for 20 adolescent leaders living with HIV in Uganda. The three‐day virtual training was designed according to principles of adult learning and adapted to suit participants through adolescent‐friendly language, case studies, and real‐life scenarios. Digital tools were utilized to adapt to challenges of the COVID‐19 pandemic. Through a range of activities targeting diverse learning styles, the training focused on three key themes: 1) the foundations of advocacy, 2) planning for advocacy, and 3) building advocacy skills.

**Lessons learned**: All participants noted in a post‐training assessment an increase in knowledge about advocacy and the development of new skills relevant to their work as youth leaders living with HIV which includes advocating for other adolescents living with HIV, providing peer psychosocial support and bridging the gap between the health care system and the client. Additionally, the advocacy training was found to refine participants’ strategies and skill for engaging decision makers and stakeholders, including service providers. Using their new skills, all participants successfully organized World AIDS Day activities, related to community sensitization, increasing psychosocial support visibility, and ending stigma. Furthermore, 25% of participants have since engaged with media programs to advocate for improved HIV service delivery.

**Conclusions/Next steps**: A virtual advocacy training, tailored specifically to adolescents and youth, was found to be an appropriate and effective method for building youth advocacy capacity, engaging adolescent voices in the HIV response, and enhancing youth‐friendly service provision. Further research is needed to confirm durability of knowledge and skills gained, and investigate the long‐term impacts on youth advocacy amongst participants. Moving forward, a skills‐based advocacy training targeted at adolescents and youth should be replicated to increase the substantive participation of this population in HIV policy development and program implementation.

### PESAF08 Community based organizations: the lastest bastion against abusive patent protection on life saving medicines. The experience of Make Medicines Affordable consortium

O. Mellouk ^1^, S. Kondratyuk^2^

^1^ITPC, Marrakech, Morocco, ^2^ITPC, Kyiv, Ukraine

**Background**: For decades, patent barriers have been identified as key barriers for access to HIV, TB, and HCV treatment and more recently to COVID‐19 vaccines and antivirals in several low and middle‐income countries (LMICs). The use of the TRIPS flexibilities to remove patent barriers remains important component of ensuring access to affordable medicines. Today, civil society and community based organizations (CBOs) play a major role in challenging patents on medicines by filing patent oppositions.

**Description**: Since 2015, under Make Medicines Affordable (MMA) campaign, an ITPC‐led consortium of 13 CBOs managed to file 69 challenges on patent applications and patents aimed at protecting monopolies on 10 ARVs (31 oppositions), 5 DAAs (10 oppositions), 3 COVID (12 oppositions) and 4 anti‐TB medicines (16 oppositions) in 13 countries in Latin America, EECA and SEA regions. Oppositions were prepared by local multidisciplinary teams consisting of community representatives (PLHIV, people affected by TB, HCV and COVID), lawyers and chemists. Targeted medicines were informed by consultation with local community organizations and health officials.

**Lessons learned**: Although filing patent opposition remains a technical process, it is possible to build local CBOs capacity to file oppositions, as it was done by MMA consortium since 2019 in Belarus, Georgia, Guatemala, Kazakhstan, Kyrgyzstan, Moldova. In order to define most optimal target medicines wide consultation with local CBOs in combination with clinical trials, patent and market intelligence work was beneficial. To develop strong patent oppositions arguments that will succeed it is also important to conduct capacity building of local chemists involved in patent oppositions work.

**Conclusions/Next steps**: While patent oppositions to succeed take some time to prepare and support ‐ from months to several years, ‐ and not all of oppositions succeed, from 69 oppositions ‐ 17 oppositions (25%) led to positive outcomes generating significant savings for state budgets in relevant countries (estimated $472 million savings in Argentina, Brazil, Thailand, Ukraine), which led to significant increases in HIV and HCV treatment programs coverage. Thus, among TRIPS Flexibilities patent oppositions is one of the most accessible and impactful options that can be used by local community organizations.

### PESAF09 Brazilian civil society role in opposing HIV/AIDS patent applications: 15 years of experience defending the Brazilian public health system

P. Villardi^1^, F. Fonseca ^1^, G. Zucoloto^2^

^1^ABIA, GTPI, Rio de Janeiro, Brazil, ^2^IPEA, Rio de Janeiro, Brazil

**Background**: The Brazilian IP law foresees the possibility of any interested party to fill patent oppositions (PO), i.e., document with technical grounds for a given patent application to be rejected. ABIA/GTPI is a civil society coalition that challenges the impact of pharmaceutical monopolies on public health. Over the last fifteen years (2006–2021), ABIA/GTPI have filed PO against eight patent applications related to six ARVs, to prevent unfair monopolies.

**Methods**: We analyze the PO's outcome using: patent application granting or rejection, price reductions: yes or not, entry or not of generic versions. We set three categories: (a) total success, (b) partial success, and (c) unsuccessful. Total success means the patent was rejected, and there was a price reduction. Partial success means when the patent is rejected without the entry of generics or a price reduction. Unsuccessful means when the patent was granted, there was no entry of generic competitors nor price reduction.

**Results**: Two drugs were total successes (TDF and TDF+FTC), three were partial success (atazanavir, dolutegravir, and lopinavir/ritonavir) and one was unsuccessful (TAF). Considering TDF and TDF+FTC: rejected patent applications and price reduction with the entry of generics. Granted patents for atazanavir and dolutegravir without generic entry, but with price reductions after PO. In the Lopinavir/ritonavir case, the patent application was rejected, but there was no price reduction or generic entry. The unsuccessful case was TAF. The patent was granted, but there was no purchase by the Brazilian government until the date of the data collection.

**Table:** 

Abstract PESAF09‐Table 1.				
Drug	Patent #	Rejected or granted?	Price reduction?	Generic entry?
**TDF**	PI0406760‐6	rejected	Yes	Yes
**TDF+FTC**	PI9811045‐2 PI9816239‐0	rejected	Yes	Yes
**Lopinavir+ritonavir**	PI1101190‐4 PI0413882‐1	rejected rejected	Yes	Yes
**Dolutegravir**	PI0610030‐9	granted	Yes	No
**TAF**	PI0112646‐6	granted	‐	No
**Atazanavir**	PI0509595‐6	granted	Yes	No

**Conclusions**: The study showed total or partial success in five out of six cases. The ABIA/GTPI's POs have helped to decrease prices for five ARVs. We highlight the TDF and TDF+FTC cases. TDF was the backbone of treatment in Brazil for almost a decade. The purchase of generic versions of TDF contributed to the universal access public policies. Generic version of TDF+FTC, used in PrEP, made possible this policy's expansion.

### PESAF10 Where the World Stands on the ‘10‐10‐10’ Social Enabler Policies: Mapping and Analyzing Progress and Gaps

M. Pillinger^1^, J.R. Moon^2^, J. McHardy ^1^, S. Light^1^, K. Aneja^1^, S. Lynch^1^, T. Erkola^3^, M. Kavanagh^3^

^1^The O'Neill Institute for National and Global Health Law, Georgetown University, Washington, United States, ^2^University of Sussex, Brighton, United Kingdom, ^3^Joint United Nations Programme on HIV/AIDS (UNAIDS), Geneva, Switzerland

**Background**: Criminalization and stigma/discrimination against PLHIV and key and marginalized populations create significant barriers to achieving global HIV/AIDS goals. In the 2021 UN Political Declaration on HIV/AIDS, governments committed to address these barriers by adopting the UNAIDS‐proposed ‘10‐10‐10’ societal enabler targets. We identify seven laws/policies that countries should adopt in order to create a legal/policy environment conducive to achieving these targets. (They are: non‐criminalization of (1) same‐sex sex, (2) sex work, (3) drug use, (4) HIV transmission; (5) creation of national human rights institutions; and legal protections against (6) discrimination and (7) gender‐based violence.)

**Methods**: Using data from Georgetown University's HIV Policy Lab on 194 countries, we analyze whether each country has adopted each law/policy. We then map and compare policy adoption globally and across regions and other country groupings to describe the current state of policy progress towards the 10‐10‐10 goals and pinpoint where policy change is needed.

We then apply network analysis methods to map the co‐occurrence, clusters and intersections of different sets of policies to deepen and quantify our “3D” understanding of how policies overlap and interact in practice and pinpoint underlying cross‐national patterns in policy adoption. Finally, we investigate correlations between the adoption of different combinations of social enabler policies and key HIV outcomes (e.g., % of PLHIV who know their status, are on ART, and are virally suppressed).

**Results**: Preliminary descriptive findings indicate that, on average, countries have adopted three of the seven laws/policies (range: 1–6 policies). The adoption rates for individual policies vary widely; whereas 78% of countries have gender‐based violence laws, only 3% and 4% do not criminalize drug use and sex work, respectively. Regionally, we observe the greatest variation in adoption rates for: same‐sex sex non‐criminalization, where WCENA and EECA countries have significantly higher rates of policy adoption than other regions; and HIV transmission non‐criminalization, where sub‐Saharan African countries have significantly higher rates of policy adoption than other regions.

**Conclusions**: These findings will further deepen our understanding of the patterns and political, economic, and geographic factors that shape policy adoption and the importance of supportive law/policy environments in the fight against HIV. They also inform policy change advocacy around the 10‐10‐10 targets.

### PESAF11 Violence against people living with HIV in the context of Colombian armed conflict: findings from a report presented to the Colombian Truth Commission

J.F. Serrano Amaya ^1^, C. Martinez Quesada^2^, H. Mejia Mercado^3^, M.S. Luna Siachoque^4^

^1^Universidad de los Andes, Lenguas y Cultura, Bogotá, Colombia, ^2^Corporación Red Somos, Bogotá, Colombia, ^3^Corporación Caribe Afirmativo, Barranquilla, Colombia, ^4^Universidad de los Andes, Bogotá, Colombia

**Background**: In 2020 a group of scholars and activists with expertise on HIV and LGBTQ rights from *Universidad de los Andes*, *Caribe Afirmativo* and *Red Somos* allied to produce a report on how internal armed conflict affected people living with HIV –PLHIV‐ in Colombia. It was produced as a research document and as a tool for advocacy. The report was submitted to the Colombian Truth Commission in 2021. It intended to make visible that PLHIV were victims of internal armed conflict due to stigma, vulnerability and lack of institutional support. They were targeted as individuals and as a specific social group for the benefit of armed actors.

**Methods**: The report describes the patterns of violence suffered by PLHIV from 1995 up to 2020. It has a focus on the experiences of victims, taking a distance from the more common epidemiological approaches in the analysis of interactions between war and HIV (Elbe, 2000; Whiteside, 2006; McInnes, 2009). 17 in‐depth interviews to HIV activists in 11 municipalities and a meta‐analysis of newspapers and human rights reports were conducted.

**Results**: Two main patterns of violence were identified: (i) direct violence, including threats, forced displacement and selective killings of PLWHIV; (ii) indirect violence, including threats, persecution of marginalised populations and gendered and sexualised violence against PLHIV or collectives associated with HIV, as sex workers and LGBTQ people. Those patterns acted against individuals with real or assumed diagnostic of HIV and their organisations; occurred all through the period of analysis and in urban and rural areas; were committed by armed actors from all sides of political spectrum in their disputes for territorial control; affected the fabric of community based activism and leadership that has been pivotal in the response to HIV and its consequences.

**Conclusions**: Findings suggest that a crime of persecution was committed against PLHIV and their organisations during internal armed conflict. Results are useful in advocacy for rights of PLHIV and in the implementation of transitional justice measures, particularly for truth telling mechanisms such as truth Commissions; in the prosecution of perpetrators; and in the design of reparatory measures for PLHIV victims of armed conflict.

### PESAF12 Benchmarks and beyond: assessing and addressing structural barriers to rights‐based HIV programming for LGBTI key populations in six African countries

M. Tabengwa ^1^, M. Judge^1^, J. O'Malley^1^, F. Damazio^2^, A. Kra^3^, B. Loots^1^, S. Tamundele^4^, I. Yekeye^1^

^1^UNDP, Johannesburg, South Africa, ^2^UNDP, Johannesburg, Angola, ^3^UNDP, Johannesburg, Congo, Democratic Republic of the, ^4^UNDP, Johannesburg, Cote D'Ivoire

**Background**: There has been significant progress in reducing new HIV infections and related morbidity and mortality in the general population in Africa, but much less among key populations. According to UNAIDS, by 2019, approximately 50% of new infections in Sub‐Saharan Africa were among key populations and their sexual partners. There is considerable evidence that stigma, discrimination, criminalization and social exclusion make key populations more vulnerable to HIV infection and less likely to access and use relevant services. The UNDP's Inclusive Governance Initiative commissioned baseline research in six African countries to benchmark progress and barriers to the rights and inclusion of LGBTI people in national laws, policies, strategies and programmes, in the health sector and beyond.

**Methods**: One of the aims of this research is to contribute to strengthening the structural conditions for effective and accessible HIV programming for diverse LGBTI populations in Africa. Country baseline studies were undertaken in Namibia, Zimbabwe, Angola, Côte d'Ivoire, Kenya and the Democratic Republic of Congo. Data was gathered through desktop research, targeted policy analysis, and stakeholder interviews in each country including with state decision‐makers (in the judiciary, parliament, the executive, national human rights institutions and government departments) and leaders from development agencies and civil society.

**Results**: Given the links between regulatory environments and HIV outcomes for key populations, structural interventions are necessary to tackle human rights barriers to HIV related services. Extrapolations from the country‐level studies identified key policy and legislative developments where significant progress has been achieved, along with critical entry points for the further reduction of structural impediments to effective HIV programming for key populations. Structural factors that perpetuate the vulnerabilities of LGBTI people are also identified, along with potential strategies to address those.

**Conclusions**: The findings, which highlight contextual dynamics and explore their implications for structural reform efforts, are highly relevant to understanding LGBTI rights and inclusion as a critical step to reducing structural obstacles facing key populations. This provides contextually relevant knowledge to inform advocacy, law reform, and country and multi‐country programming, aimed at challenging the structural dynamics that undermine right‐based HIV prevention, care and support for marginalised groups in Africa.

### PESUA14 Administration of the broadly neutralizing, CD4‐binding site targeting antibody VRC07‐523LS in dual‐ and triple‐antibody combinations with 10–1074, PGT121, and/or PGDM1400: Impact on pharmacokinetics compared to VRC07‐523LS administration alone

S. Walsh ^1^, C. Gay^2^, M. Sobieszczyk^3^, S. Mannheimer^3^, O. Hyrien^4^, C. Yu^4^, K. Seaton^5^, T. Skalland^4^, J. Dumond^2^, P. Andrew^6^, C. Karg^4^, C. Paez^4^, T. Gamble^6^, Z. He^4^, B. Hanscom^4^, B. Dye^6^, E. Piwowar‐Manning^7^, J. Hural^4^, L. Polakowski^8^, M. Yacovone^8^, W. Chege^8^, D. Montefiori^5^, L. Gama^8^, J. Mascola^8^, G. Tomaras^5^, Y. Huang^4^, S. Karuna^4^, HVTN127/HPTN087 and HVTN130/HPTN089 Study Teams

^1^Brigham & Women's Hospital, Infectious Diseases, Boston, United States, ^2^University of North Carolina, Chapel Hill, United States, ^3^Columbia University, New York, United States, ^4^Fred Hutchinson Cancer Research Center, Seattle, United States, ^5^Duke University, Durham, United States, ^6^FHI 360, Durham, United States, ^7^Johns Hopkins University, Baltimore, United States, ^8^National Institute of Allergy and Infectious Diseases, Bethesda, United States

**Background**: Broadly neutralizing antibodies (bnAbs) are a promising approach for HIV‐1 prevention. In the only bnAb HIV prevention efficacy studies to date (the AMP studies), intravenous (IV) administration of a CD4‐binding site targeting bnAb (VRC01) prevented infection only against highly susceptible viruses. BnAb combinations, particularly using bnAbs engineered for increased potency, breadth, and half‐life, may be more efficacious. Clinical data assessing potential interactions between co‐administered antibodies is limited. We present the first interim clinical data comparing VRC07‐523LS pharmacokinetics (PK) when administered alone (HVTN 127/HPTN 087) versus co‐administered with other bnAbs that bind non‐overlapping HIV Env epitopes, including V2‐binding PGDM1400 and V3‐binding 10–1074 and PGT121 (HVTN 130/HPTN 089).

**Figure:**
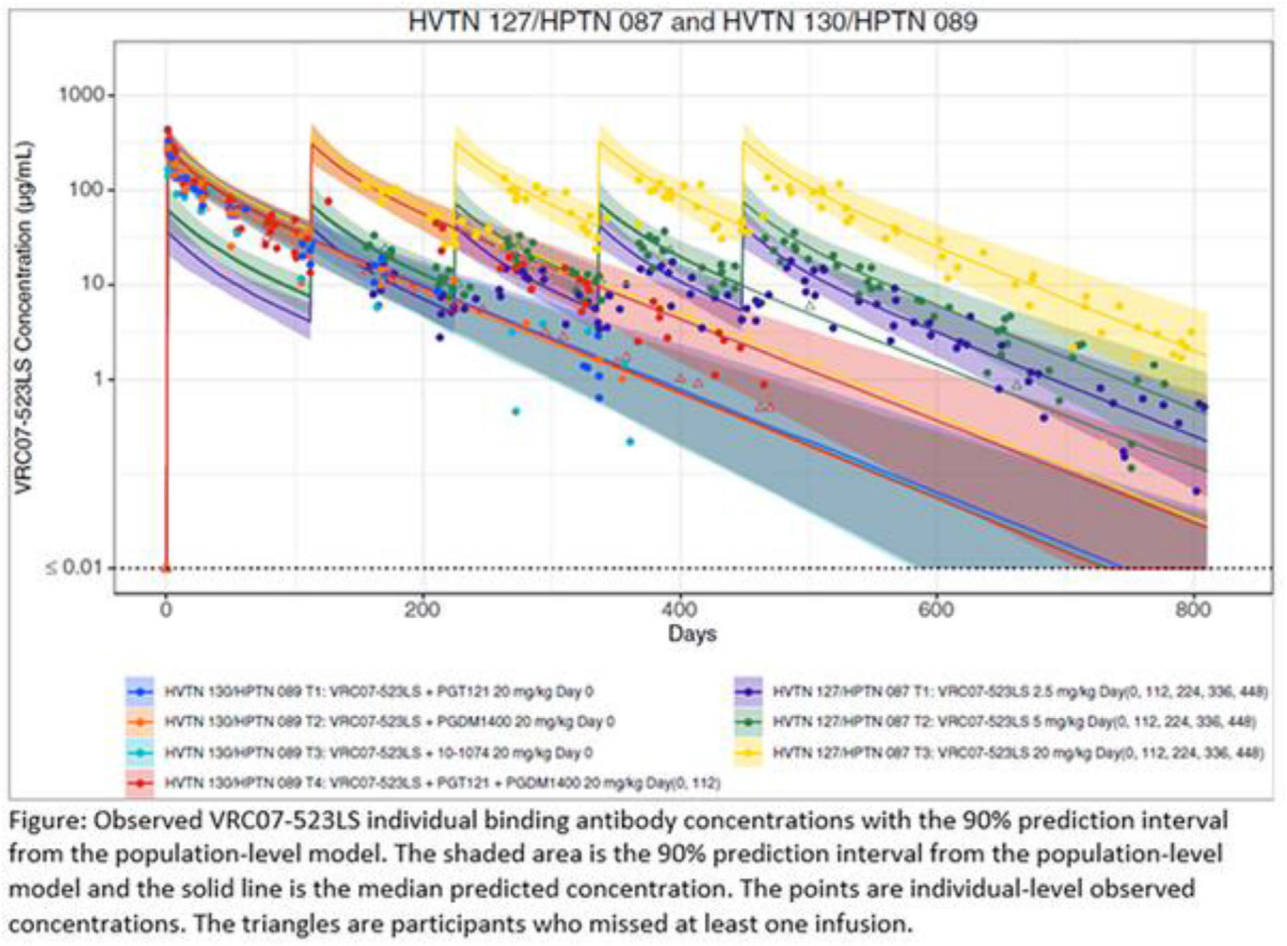
**Abstract PESUA14‐Figure 1**.

**Methods**: Healthy, HIV‐uninfected adults received VRC07‐523LS administered IV at four‐month intervals alone at five timepoints (n = 29; 2.5, 5, or 20 mg/kg), sequentially in dual combination with 10–1074, PGT121, or PGDM1400 at one timepoint (n = 18; 20 mg/kg), or in triple combination with PGDM1400 and PGT121 at two timepoints (n = 9; 20 mg/kg). VRC07‐523LS serum concentration kinetics were measured by anti‐idiotype Binding Antibody Multiplex Assay. A two‐compartment population PK model was fitted to estimate PK parameters.

**Results**: Participant demographics and baseline characteristics were similar between the two studies. Predicted VRC07‐523LS levels from the fitted model were in excellent agreement with observed levels (see Figure). Small changes in clearance, intercompartmental clearance, and peripheral volume PK parameters of VRC07‐523LS were observed with co‐administration of 10–1074, PGT121, and/or PGDM1400. VRC07‐523LS alone has an estimated median half‐life of ∼54.8 days versus ∼52.3 days when co‐administered with 10–1074, PGT121, and/or PGDM1400 (p = 0.55).

**Conclusions**: VRC07‐523LS appears to be cleared more rapidly with a larger peripheral volume when co‐administered with 10‐1074, PGT121, and/or PGDM1400 versus administration alone. With an insignificant impact on elimination half‐life, these data suggest that the duration of PK coverage for single anti‐HIV bnAbs, like VRC07‐523LS, is preserved in bnAb combinations.

### PESUA15 Depot‐medroxyprogeterone acetate use alters immune modulation function of cortisol and thyroid hormones

K. Omollo ^1,2^, J. Oyugi^1,3^, M.M. Kowatsch^3^, J. Kimani^2^, C. Kaushic^4,5^, J. Lajoie^3,1^, K.R. Fowke^3,6,2,1^

^1^University of Nairobi, Medical Microbiology, Nairobi, Kenya, ^2^Partners For Health And Development in Africa, Nairobi, Kenya, ^3^University of Manitoba, Medical Microbiology and Infectious Diseases, Winnipeg, Canada, ^4^McMaster University, McMaster Immunology Research Centre, Michael G. DeGroote Centre for Learning and Discovery, Hamilton, Ontario, Canada, ^5^McMaster University, Department of Pathology and Molecular Medicine, Canada, ^6^University of Manitoba, Department of Community Health Science, Winnipeg, Canada

**Background**: Depot‐medroxyprogesterone acetate (DMPA) is a popular contraceptive; however, some studies report an association with increased HIV susceptibility through mechanisms that are not yet fully elucidated. We investigated the plasma levels of cortisol, free triiodothyronine (T3), and free thyroxine (T4) and the correlation of these hormones with markers of inflammation and CD4+ T cell activation in female sex workers and non‐sex workers using DMPA and those not using any hormonal contraception (non‐HC).

**Methods**: From a group of Kenyan female sex workers (FSW (51)) and non‐sex workers (60), blood and cervical cytobrush‐derived T cells were phenotyped using flow cytometry. Cytokine concentrations were determined in plasma and cervico‐vaginal lavages and comparisons were done between the women according to contraception use.

**Results**: In FSW, we observed that DMPA users (27) had significantly higher levels of Cortisol (p < 0.001), T3 (p < 0.001), and T4 (p < 0.001) while in non‐sex workers, only T3 levels were significantly higher in DMPA users (p < 0.01). Correlation of the three hormones revealed a direct relationship in the levels of T3 and T4 (r = 0.67, p < 0.001), T3 and Cortisol (r = 0.54, p = 0.002), and T4 and Cortisol (r = 0.56, p = 0.001) in DMPA‐using non‐sex workers. Increased cortisol levels in DMPA‐using FSW inversely correlated with plasma MIP‐1β levels but had no effect on genital cytokines. In FSW non‐HC users, cortisol and T4 positively correlated with MIP‐1α at the genital tract. T3 positively correlated with plasma IL‐8 and sCD40L in DMPA‐using FSW but had no impact in non‐HC users. Cortisol had an inverse correlation with the proportion of mucosal CD4+CCR5+ T cells in DMPA‐using FSW, yet positively correlated with the same cells in non‐HC users. Levels of T4 correlated with the per cell expression of CD69, and the proportions of CD4+ T cells expressing CD38, HLADR and CCR5 in DMPA‐using FSW.

**Conclusions**: These data demonstrate that DMPA use results in elevated levels of cortisol, T3 and T4 which then mediate T cell activation and inflammation in HIV uninfected women. This effect of DMPA may be an important yet under investigated mechanism by which the contraceptive DMPA may influence susceptibility to HIV in women.

### PESUA16 Role of the *pol*gene enhancer in HIV‐1 transcription and replication in myeloid infected cells

C. Van Lint ^1^, O. Hernalsteens^1^, R. Verdikt^1^, C. Vanhulle^1^, A. Dutilleul^1^, S. Cristinelli^2^, T. Marray^1^, L. Nestola^1^, V. Monceaux^3^, A. David^3^, B. Verhasselt^4^, H. Galons^5^, A. Ciuffi^2^, A. Saez‐Cirion^3^, O. Rohr^6^

^1^Université Libre de Bruxelles (ULB), Gosselies, Belgium, ^2^Lausanne University Hospital and University of Lausanne, Lausanne, Switzerland, ^3^Institut Pasteur de Paris, Paris, France, ^4^Ghent University, Ghent, Belgium, ^5^CNRS UMR8258 ‐ U1267 INSERM, Paris, France, ^6^IUT Louis Pasteur de Schiltigheim, Strasbourg, France

**Background**: There is increasing evidence for the physiological relevance of myeloid HIV‐1 reservoirs such as brain microglia and urethral macrophages. However, the molecular mechanisms of HIV‐1 expression in myeloid infected cells are still poorly understood. The HIV‐1 intragenic *cis*‐regulatory region (IRR) located in the *pol* gene exhibits an enhancer activity on the 5’LTR promoter. The IRR possesses multiple binding sites for various cellular transcription factors (TF). Here, we characterized several of these binding sites and their functional involvement in the IRR‐mediated control of HIV‐1 gene expression in monocytes/macrophages. Special emphasis was put on studying several binding sites for the myeloid‐specific PU.1 TF, known to be a pioneer factor inducing the opening of heterochromatin in enhancers.

**Methods**: Electrophoretic Mobility Shift Assays (EMSAs), chromatin immunoprecipitations, infection studies, ATAC‐seq (Assay for Transposase‐Accessible Chromatin sequencing).

**Results**: We demonstrated the *in vivo* recruitment of PU.1 to the HIV‐1 intragenic enhancer in latently‐infected cell lines from myeloid origin. We physically characterized *in vitro* PU.1 binding to the different intragenic PU‐boxes by EMSAs and identified mutations abolishing PU.1 binding without altering the underlying amino acid sequence of the *pol* gene. We demonstrated the role of the PU‐boxes in the IRR enhancer activity, its chromatin remodeling and HIV‐1 epigenetic regulation, in concert with other IRR transcription factor binding sites. We showed the importance of intragenic TF binding sites in HIV‐1 replication using mutated viral particles in single‐round infection experiments using primary monocytes‐derived macrophages isolated from uninfected individuals. To overcome HIV‐ 1 persistence, targeted approaches for each specific cellular reservoir are needed. As a proof‐of‐concept, we revealed the potential therapeutic application of a specific inhibitor interfering with PU.1 binding as a new anti‐HIV‐1 approach.

**Conclusions**: The HIV‐1 intragenic enhancer brings an additional factor in an already complex network of regulators affecting the level of HIV‐1 transcription. Such complexity could allow a finer‐tuned regulation that might find its purpose when HIV‐1 transcription needs to be moderately or transiently modified within different cellular and chromatin environments. The cell specificity of the IRR in HIV‐1 gene expression regulation opens new avenues for HIV cure approaches targeting the heterogeneous cellular and tissue latent reservoirs of virus.

### PESUA17 Role of UHRF1 in HIV‐1 transcriptional regulation

M. Bendoumou ^1^, R. Verdikt^1^, S. Bouchat^1^, L. Nestola^1^, A.O. Pasternak^2^, G. Darcis^3^, V. Avettand‐Fenoel^4^, C. Vanhulle^1^, A. Ait‐Ammar^1^, M. Santangelo^1^, E. Plant^1^, V. Le Douce^5^, N. Delacourt^1^, A. Cicilionytè^2^, C. Necsoi^6^, F. Corazza^7^, C. Pereira Bittencourt Passaes^8^, C. Schwartz^9^, M. Bizet^10^, F. Fuks^10^, A. Sáez‐Cirión^8^, C. Rouzioux^11^, S. De Wit^6^, B. Berkhout^2^, V. Gautier^5^, O. Rohr^9^, C. Van Lint^1^

^1^Université Libre de Bruxelles, Service of Molecular Virology, Gosselies, Belgium, ^2^Amsterdam UMC, University of Amterdam, Amsterdam, Netherlands, the, ^3^Infectious Diseases Departement, Liege University Hospital, Liège, Belgium, ^4^INSERM, U1016, Institut Cochin, Paris, France, ^5^Centre for Research in Infectious Deseases, University College Dublin, Dublin, Ireland, ^6^Service des Maladies Infectieuse, CHU St‐Pierre, Université Libre de Bruxelles, Brussels, Belgium, ^7^Laboratory of Immunology, IRISLab, CHU Brugmann, Université Libre de Bruxelles, Brussels, Belgium, ^8^Institut Pasteur, Unité HIV, Inflammation et Persistance, Paris, France, ^9^Université de Strasbourg, Schiltigheim, France, ^10^Laboratory of Cancer Epigenetics, Faculty of Medecine, Université Libre de Bruxelles, Brussels, Belgium, ^11^AP‐HP, Hôpital Necker‐Enfants‐Malades, Service de Microbiologie clinique, Paris, France

**Background**: DNA methylation is one of the epigenetic mechanisms involved in HIV‐1 latency. The latent 5’LTR methylation profile is heterogeneous in latency model cell lines and in patient cells where it increases with the duration of cART. We have previously demonstrated that 5‐Azadeoxycytidine (decitabine) treatment, an inhibitor of DNA methylation, resulted in variable levels of HIV‐1 reactivation in latently infected T‐cell lines and in *ex vivo* patient cell cultures. Nevertheless, the mechanisms mediating HIV‐1 latency through DNA methylation remain unclear.

**Methods**: Sodium bisulfite sequencing, Electrophoretic mobility shift Assay, ChIP‐qPCR, RNA interference, GFP fluorescence FACS, p24 ELISA and purification of primary cells from HIV+ patient blood.

**Results**: Using latently infected J‐Lat cell lines, displaying different integration sites, we showed that decitabine‐induced reactivation of HIV‐1 was accompanied by differential DNA demethylation profiles at the 5’LTR, occurring at specific CpG positions, termed DDMP (*Differentially DeMethylated Positions*). Interestingly, we showed that UHRF1 (Ubiquitin‐like with PHD and Ring Finger domain 1) bound *in vitro* to several of these DDMPs through different binding modalities where DNA methylation was either non‐essential, either essential, or enhancing UHRF1 binding. Moreover, since UHRF1 was originally identified as an CCAAT/enhancer binding protein (C/EBP), we were able to show *in vivo* recruitment of UHRF1 to four C/EBPs motifs localized in the 5’LTR independently of DNA methylation, in both cell line and primary cell models for HIV‐1 latency. UHRF1 depletion through RNA interference induced an increase in HIV gene expression accompanied by global DNA and histone demethylation of the 5’LTR . We showed that UHRF1 repressed HIV‐1 in absence and presence of Tat, independently of 5’LTR DNA methylation. Pharmacological inhibition of UHRF1 in PBMCs isolated from aviremic cART‐treated HIV+ individuals reactivated expression of HIV‐1 RNAs.

**Conclusions**: We demonstrate an important role in HIV‐1 latency of UHRF1 which binds to multiple sites throughout the 5’LTR in a methylation‐dependent and ‐independent manner. As UHRF1 is known to maintain heterochromatic profiles during replication, our results suggest its involvement in HIV‐1 latency by maintenance of the 5’LTR methylation and other mechanisms. In this regard, UHRF1 constitutes a new therapeutic target for HIV cure strategies.

### PESUA18 *In vivo* and *in vitro* evidence for infection of naïve CD4 T cells with CCR5‐tropic HIV

C. Kearns ^1^, M. Pinzone^1^, A. Oceguera^1^, A. Ginda^1^, U. O'Doherty^1^

^1^University of Pennsylvania, Department Of Pathology and Laboratory Medicine, Philadelphia, United States

**Background**: Historically, the field of HIV research has largely ignored the viral reservoir in naïve CD4+ T cells, due to lower HIV DNA levels compared to memory subsets. Our analysis reveals unique proviral sequences within naïve CD4 T cells suggesting that the naïve reservoir is distinct from the memory reservoir. The naïvereservoir represents a unique hurdle because it is persistent, diverse, and resistant to immune clearance. It may also serve to replenish the more differentiated memory reservoir. Curiously, we detected CCR5‐tropic HIV in naïve cells in Persons Living with HIV (PLWH) using computer algorithms to determine tropism. Given that naïve T cells appear to lack the CCR5 co‐receptor, we first wanted to determine tropism phenotypically *in vivo* and then explore mechanisms of naïve infection *in vitro*.

**Methods**: We tested the *in vivo* tropism bystimulating sorted naïve CD4 T cells (distinguished as CD95‐,CD45RA+,CCR7+,CD27+) to release virus at limiting dilution from PLWH on antiviral therapy. Tropism of HIV RNA+ supernatants were then determined by infecting cells engineered to express CCR5 or CXCR4.

We next investigated *in vitro* conditions that promote naïve infections. We infected mixtures of CD4 subsets, as well as pre‐sorted naïve cells alone with a CCR5‐tropic HIV utilizing fibroblast reticular stromal cells to preserve the naïve phenotype.

**Results**: We showed CCR5‐tropic HIV can be isolated from naïve CD4 T cells of PLWH by performing infection studies with out‐growth virus in the presence and absence of CCR5 inhibitors.

We also found CCR5‐tropic infection occurred *in vitro* in phenotypically naïve cells when CD4+ T cells were infected in bulk, but not when naïve cells were pre‐sorted and then infected. Thus, memory T cell reversion or transient upregulation of CCR5 expression may provide a mechanism for CCR5‐tropic naïve infection which may be promoted by cellular interactions that occur in the lymph node milieu.

**Conclusions**: We present *in vivo* and *in vitro* data that CCR5‐tropic infection occurs in naïve cells. Additionally, we developed an infection model that promotes naïve infection, potentially by reversion. This model can be utilized to explore mechanisms that underlie naïve infection and for preclinical studies to probe the naïve reservoir.

### PESUA19 High prevalence of HIV persistence in CSF of adolescents and young adults with perinatally‐acquired HIV and cognitive impairment in the IMPAACT 2015 study

T. Wagner^1^, C. Tierney^2^, S. Huang^2^, S. Nichols^3^, K. Malee^4^, N. Montañez^5^, A. Coletti^5^, H. Spiegel^6^, M. Wilkins^7^, L. Abuogi^8^, M. Purswani^9^, A. Bearden^10^, A. Wiznia^11^, A. Agwu^12^, E. Chadwick^4^, D. Richman^3^, M. Gandhi^13^, P. Mehta^14^, B. Macatangay^14^, S. Spector^3^, S. Spudich^15^, D. Persaud^12^, A. Chahroudi ^16^, IMPAACT 2015 Protocol Team

^1^Center for Global Infectious Disease Research, University of Washington, Seattle, United States, ^2^Center for Biostatistics in AIDS Research, Harvard TH Chan School of Public Health, Boston, United States, ^3^University of California, San Diego, San Diego, United States, ^4^Ann & Robert H Lurie Children's Hospital of Chicago, Chicago, United States, ^5^IMPAACT Operations Center, FHI360, Durham, United States, ^6^Kelly Government Solutions, Contractor to the National Institute of Allergy and Infectious Diseases, Rockville, United States, ^7^St. Jude Children's Research Hospital, Memphis, United States, ^8^University of Colorado, Denver, United States, ^9^Bronx‐Lebanon Hospital Center, Bronx, United States, ^10^University of Southern California, Los Angeles, United States, ^11^Jacobi Medical Center, Bronx, United States, ^12^Johns Hopkins University School of Medicine, Baltimore, United States, ^13^University of California, San Francisco, San Francisco, United States, ^14^University of Pittsburgh, Pittsburgh, United States, ^15^Yale University, New Haven, United States, ^16^Emory University School of Medicine, Atlanta, United States

**Background**: HIV persistence in the central nervous system (CNS) may be an important barrier to cure/remission strategies and may impact long‐term cognitive outcomes in adolescents and young adults with perinatal HIV (AYAPHIV). IMPAACT 2015 systematically examined AYAPHIV with cognitive impairment and receiving effective antiretroviral therapy (ART) to quantify HIV persistence in blood and cerebrospinal fluid (CSF).

**Methods**: AYAPHIV (13–30 years old) with cognitive impairment and on suppressive ART were consented and enrolled into IMPAACT 2015, an IRB‐approved U.S.‐based cross‐sectional, multi‐site, exploratory, observational study. Cognitive impairment was defined as NIH Toolbox Fluid Cognition composite standard score >1 S.D. below the normative group mean. Participants underwent lumbar puncture (LP), phlebotomy, and hair collection. CSF and blood were measured for HIV‐RNA and HIVpol/gag‐DNA and 11 biomarkers of inflammation and neuronal injury. Hair was used to quantify ART exposure levels. Exact binomial confidence intervals (CIs) were calculated, and 41 comparisons evaluated with Wilcoxon rank sum tests.

**Results**: Among 24 enrolled participants, 22 underwent LP, and 20/22 (91%) had successful CSF collection. 18 participants met ART suppression criteria, and had plasma HIV‐RNA <20 copies/ml from entry through the day of LP. 9/18 (50%) were cisgender females and 14/18 (78%) were Black. Median (range) age was 20 years (13–27), time on ART 18.3 years (8.0–25.5), CD4 count 701 cells/mm^3^ (143–1342), and Fluid Cognition T‐score 68 (53–80). HIV‐DNA was detected in PBMCs in all participants. In CSF, 2/18 participants had detectable HIV‐RNA, one of whom was quantifiable (5.6% 95% CI (0.1%, 27.3%)) and HIVgag and/or pol‐DNA was detectable in 13/18 (72% 95% CI (47%, 90%)). Detectable HIV‐DNA in CSF was associated with higher levels of HIVpol‐DNA copies in PBMCs (medians 227, 27 per million cells, p = 0.04), and trended with lower scores on a Fluid Cognition subtest measuring Inhibitory Control and Attention (medians 49, 65 p = 0.09). Measured biomarkers and ART levels were not statistically associated with presence of detectable HIV‐DNA in CSF.

**Conclusions**: Findings from IMPAACT 2015 suggest that the CNS is a site of HIV persistence in the majority of AYAPHIV with cognitive impairment, warranting further evaluation in pediatric HIV treatment and eradication studies.

### PESUA20 Subtype A1 and D HIV‐1 proviral genome landscape in Rakai, Uganda

G.Q. Lee ^1^, P. Khadka^1^, R.B. Jones^1^, Z.L. Brumme^2,3^, J. Kasule^4^, T. Kityamuweesi^4^, P. Buule^4^, S. Reynolds^5,6^, T. Quinn^5,6^, J. Prodger^7^, A.D. Redd^5,6^

^1^Weill Cornell Medical College, Department of Medicine, Division of Infectious Diseases, New York, United States, ^2^Simon Fraser University, Burnaby, Canada, ^3^BC Centre for Excellence in HIV/AIDS, Vancouver, Canada, ^4^Rakai Health Sciences Program, Kalisizo, Uganda, ^5^National Institute of Allergy and Infectious Diseases, Division of Intramural Research, Bethesda, United States, ^6^Johns Hopkins University School of Medicine, Department of Medicine, Baltimore, United States, ^7^Western University, Department of Microbiology and Immunology, London, Canada

**Background**: Whether various HIV‐1 subtypes have similar reservoir profiles, such as the frequencies of intact proviruses and extent of clonal expansion, remains a major knowledge gap in cure research. Here, we describe and compare near‐full‐genome DNA sequences of subtype A1 and D HIV‐1 and their associated recombinant forms observed in Rakai, Uganda.

**Methods**: Blood was collected from 7 male and 16 female participants with chronic HIV‐1 who were virologically‐suppressed for >1‐year. Resting CD4+ cells were negatively selected from total PBMC (CD69^−^/CD25^−^/HLA‐DR^−^) and extracted for DNA. Total HIV‐1 DNA levels were quantified via *gag*‐specific droplet digital PCR, followed by limiting dilution, nested near‐full‐viral‐genome PCR (HXB2 638–9632) and Illumina sequencing. Viral genome‐intactness was assessed using a previously published software suite HIVSeqinR adapted for non‐B HIV subtypes.

**Results**: We obtained 607 near‐full‐genome HIV‐1 DNA sequences after sampling ∼two million cells per individual. Among the 23 donors, subtype distribution was 4 A1, 9 D, 9 A1/D and 1 A1/C/D. Intact genomes were relatively rare and made up <1.6% to 33%, whereas clonal expansion was detected in both intact and defective genomes and made up <2–14% (intact) and <3–70% (defective) of the intrahost viral DNA population. Total HIV‐1 DNA load per million CD4 cells, relative proportions of intact genomes, proportions of clonally expanded genomes, and proportions of hypermutated genomes did not differ among subtypes (Kruskal‐Wallis all p >= 0.1) and did not differ by sex (Mann‐Whitney all p >= 0.2). Large deletions were significantly less frequently observed in *gag* relative to reverse transcriptase, RNaseH, integrase, *vif*, *vpr*, *vpu*, and *env* (intra‐host median 50% *gag‐retained* versus 18–35% other genes, Mann‐Whitney p = 0.0004–0.03). Among all the defective genomes, 85% (A1), 87% (D), 87% (A1/D), 79% (A1/C/D) lacked one or both of the Intact Proviral DNA Assay (IPDA) psi/*env* primer binding sites, whereas 100% of intact genomes in this study contained both regions.

**Conclusions**: Similar to subtype B reservoirs reported in the literature, persisting HIV DNA pools in subtypes A1, D, A1/D and A1/C/D had high proportions of defective genomes and/or had undergone clonal expansion. Future research should explore whether re‐activatability differs across HIV‐1 subtypes and utilize these sequence data to validate IPDA for non‐B HIV‐1.

### PESUA21 The LRA HODHBt synergizes with IL‐15 to enhance the cytotoxic capacity of HIV‐specific CTL

D. Copertino ^1^, C. Stover^2^, T. Zaikos^2^, A.B. Macedo^2^, A. Bosque^2^, R.B. Jones^1^

^1^Weill Cornell Medicine, Infectious Diseases, New York, United States, ^2^The George Washington University, Department of Microbiology, Immunology and Tropical Medicine, Washington, United States

**Background**: More potent “shock and kill” therapeutics are likely needed to achieve reductions in HIV reservoirs. We previously showed that 3‐hydroxy‐1,2,3‐benzotriazin‐4(3H)‐one (HODHBt) enhances IL‐15‐mediated HIV reactivation in cells from people living with HIV (PLWH), by increasing occupancy of STAT5 on the HIV‐LTR. Since IL‐15 can also enhance CD8+ T‐cell effector functions through STAT5 activation, we hypothesized that HODHBt would also synergize with IL‐15 to enhance cytotoxic functions of HIV‐specific CD8+ T‐cells.

**Methods**: CD8+ T‐cells from 9 HIV‐negative donors were treated with HODHBt and IL‐15, and assessed by flow cytometry for phosphorylation of STAT‐1, ‐3, and ‐5, and expression of Granzymes A (GZMA) and B (GZMB), Perforin and Granulysin. GZMB ELISpots were performed on PBMCs from 14 PLWH – suppressed on ART for an average of 10.9 years ‐ stimulating with peptide pools spanning Gag, Pol, Nef, Env, or CMVpp65, with or without IL‐15 and HODHBt. Secreted cytokines were measured in supernatants using the CorPlex Human Cytokine Panel 10‐Plex array.

**Results**: HODHBt alone increased STAT‐1 phosphorylation (p = 0.031) but did not increase STAT‐3 or STAT‐5 phosphorylation, nor expression of GZMB, GZMA, perforin and granulysin. Relative to IL‐15 alone, CD8+ T‐cells treated with IL‐15 and HODHBt showed increases in phosphorylation of STAT‐1 and STAT‐5 (p = 0.031) but not STAT‐3, as well as expression of GZMB (p < 0.001). HIV‐specific GZMB‐releasing responses were enhanced by treatment with HODHBt in combination with IL‐15, relative to medium only as follows: Gag 6.8‐fold (p = 0.005), Pol 6.8‐fold (p = 0.005), Nef 12.8‐fold (p < 0.001), and Env 3.7‐fold (p = 0.438, ns). These were substantially increased relative to enhancements with IL‐15 alone (IL‐15+HODHBt/L‐15+DMSO) Gag 2.4‐fold (p < 0.001), Pol 1.8‐fold, (p < 0.001), Nef4.3‐fold, (p < 0.001), and Env 2.6‐fold, (p < 0.001). HODHBt alone did not increase background (no peptide) above IL‐15 alone. Across all conditions, except CMV stimulation, GZMB was significantly positively correlated with cytokines IFNγ (r = 0.89, p < 0.001) and IL‐22 (r = 0.73, p < 0.001) by CorPlex.

**Conclusions**: HODHBt synergizes with IL‐15 to significantly enhance HIV‐specific cytotoxic T‐cell responses in *ex vivo* PBMCs from ART‐treated PLWH. Our results highlight that pharmacologic enhancement of IL‐15 mediated STAT activation can be a therapeutic strategy with the potential to enhance both the ‘shock’ and the ‘kill’ components of strategies aimed at depleting HIV reservoirs.

### PESUA22 Productively‐infected CD4 T cells are resistant to ADCC mediated by non‐neutralizing antibodies

J. Richard * ^1,2^, G. Sannier^1,2^, J. Prévost^1,2^, H. Medjahed^1^, G. Gabrielle Delgado^1^, G. Gendron‐Lepage^1^, M. Dubé^1^, D. E. Kaufmann #^1,3^, A. Finzi #^1,2^, * # Equal contribution

^1^Centre de Recherche du CHUM, Montreal, Canada, ^2^Université de Montreal, Département de Microbiologie, Infectiologie et Immunologie, Montreal, Canada, ^3^Université de Montreal, Département de Médecine, Montreal, Canada

**Background**: The conformation of the HIV‐1 envelope glycoprotein (Env) substantially impacts antibody recognition and antibody‐dependent cellular cytotoxicity (ADCC) responses. In its unliganded form, the Env samples a “closed” conformation that is preferentially recognized by broadly‐neutralizing antibodies (bNAbs). CD4 engagement drives Env into the “open” CD4‐bound conformation, preferentially targeted by non‐neutralizing Abs (nnAbs). The virus prevents exposure of CD4‐induced epitopes by downregulating CD4 via Nef and Vpu. Despite significant advances on the understanding of HIV resistance to ADCC, the capacity of nnAbs to mediate ADCC against productively‐infected cells remain controversial.

**Methods**: We used a multiplexed viral RNA detection by flow cytometric fluorescent *in situ* RNA hybridization (RNAflow‐FISH) technique to characterize cell populations targeted by bNAbs and nnAbs in the context of primary CD4+ T cells infected with a primary HIV‐1 isolate or isolated from HIV‐1‐infected individuals

**Results**: Productively‐infected cells are recognized by bNAbs, efficiently downregulate CD4, express high levels of Nef and p24 proteins and are enriched in HIV‐1 mRNA (CD4^−^p24^+^Nef^+^HIV mRNA^+^). In contrast, cells targeted by nnAbs are CD4‐positive, express little or no p24 and are negative for Nef expression and HIV‐1 mRNA (CD4^+^p24^−^/p24^low^Nef‐HIV mRNA^−^). Moreover, cells recognized by nnAbs are *env* mRNA negative, suggesting that they represent cells coated with either shed Env and/or non‐infectious viral particles. As expected, we observed that CD4 downregulation precedes the expression of HIV‐1 late transcripts, thus confirming that the CD4^+^p24^low^Nef^−^HIV mRNA^−^cells targeted by nnAbs are not part of the viral replication cycle. Finally, we found that *ex vivo* expanded CD4+ T cells isolated from HIV‐1‐infected individuals are sensitive to ADCC mediated by bNAbs but resistant to those mediated by nnAbs.

**Conclusions**: Our results demonstrate that productively‐infected cells are resistant to nnAbs. This information is important for the development of immunotherapy‐based strategies aimed at targeting and eliminating productively‐infected cells.

### PESUA23 Longer intervals between SARS‐CoV‐2 infection and SpikeVax doses improve the neutralization of different variants of concern

J. García‐Pérez^1^, M. Pérez‐Olmeda^2^, F. Díez‐Fuertes ^1^, A. Ramírez‐García^3^, H.E. De La Torre‐Tarazona^1^, M. Bermejo^1^, A. Cascajero^1^, M. Castillo‐de la Osa^2^, P. Jiménez^1^, M. Aparicio‐Gómez^4^, M. Aparicio‐Herguedas^4^, R. Layunta‐Acero^3^, L. Vicente‐Izquierdo^3^, C. Miñano‐González^4^, C. Avendaño‐Solá^3^, J. Alcamí^1^

^1^Instituto de Salud Carlos III, AIDS Immunopathogenesis Unit, Majadahonda, Spain, ^2^Instituto de Salud Carlos III, Laboratorio de Serología, Majadahonda, Spain, ^3^Hospital Universitario Puerta de Hierro Majadahonda, Instituto de Investigación Sanitaria Hospital Puerta de Hierro‐Segovia de Arana, Clinical Pharmacology Department, Majadahonda, Spain, ^4^Hospital Universitario Puerta de Hierro Majadahonda, Instituto de Investigación Sanitaria Hospital Puerta de Hierro‐Segovia de Arana, Prevention of Occupational Risks Department, Majadahonda, Spain

**Background**: Optimal time to administrate COVID‐19 vaccines after natural infection is a matter of debate. This study aims to evaluate the humoral immune response elicited against the variants of concern (VOCs) alpha, beta, and delta in convalescent and naïve people vaccinated with SpikeVax (Moderna). To evaluate the effect of extending the vaccination schedule we compared the impact of immunization 1–3 months versus 4–12 months after the natural infection in recovered patients.

**Methods**: Sera from 66 health care workers were collected at pre‐vaccination, at pre‐boost, and at post‐boost. A total of 31 out of the 66 participants had a documented prior history of SARS‐CoV‐2 infection, including 17 (53.1%) lately‐infected (LI) within three months before vaccination and 14 (43.7%) with early infection (EI) documented from 4 to 12‐months before vaccination. Antibody‐mediated immune responses were assessed by three commercial immunoassays and a SARS‐CoV‐2 lentiviral‐based pseudovirus neutralization assay.

**Results**: Levels of immunoglobulins (Ig) to SARS‐CoV‐2 were lower in naive participants at post‐boost as compared with convalescents after a single dose of SpikeVax (p < 0.05). In recovered patients, after two vaccine doses total Ig to RBD were higher in EI (21,618 BAU/ml; 95% CI: 18,092–25,831) as compared to LI (10,219 BAU/ml; 95% CI: 7572–13,792 BAU/ml) (p < 0.001). These differences were also observed for anti‐trimeric spike IgG levels and anti‐spike IgA.

The SARS‐CoV‐2 neutralization titer 50 (NT50) observed in EI was consistently higher than in LI against VOCs alpha, beta, and delta. Specifically, after the second dose of SpikeVax, the geometric mean NT50 against alpha were 6306 (95% CI: 4548–8743) for EI and 2575 (95%CI: 1737–3817) for LI; 2607 (95% CI: 1614–4211) vs 922 (95% CI: 553–1536) for beta, and 4991 (95% CI: 3319–7506) vs 1795 (95% CI: 1135–2838) for delta. These levels involve fold reductions in NT50 of 2.4‐, 2.8‐, and 2.8‐fold against alpha, beta, and delta respectively in LI in comparison with the EI group.

**Conclusions**: Increasing more than 4 months the interval between SARS‐CoV‐2 infection and the immunization with mRNA‐based vaccines generates a more efficient humoral immune response against VOCs. This improvement can be related with the time requested to mount a strong recall memory B cell response.

### PESUA24 Chronic CNS inflammation in ART‐suppressed SIV‐infected rhesus macaques is associated with immune activation and viral persistence in the gut

S. Byrnes ^1^, T. Angelovich^1^, K. Busman‐Sahay^2^, S. Younger^2^, M. Nekorchuk^2^, C. Cochrane^1^, M. Roche^3^, C. Deleage^4^, B. Brew^5^, J. Estes^2^, M. Churchill^1^

^1^RMIT University, School of Health and Biomedical Sciences, Melbourne, Australia, ^2^Oregon Health & Science University, Vaccine and Gene Therapy Institute, Oregon National Primate Research Centre, Portland, United States, ^3^Peter Doherty Institute for Infection and Immunity, Melbourne, Australia, ^4^Leidos Biomedical Research, Inc., Frederick National Laboratory for Cancer Research (FNLCR), AIDS and Cancer Virus Program, Frederick, United States, ^5^St Vincent's Hospital, Peter Duncan Neurosciences Unit, Departments of Neurology and Immunology, Sydney, Australia

**Background**: HIV‐associated neurocognitive disorders (HAND) affect ∼30% of virally suppressed people with HIV (PWH), suggesting that HAND pathogenesis may be driven by mechanisms other than direct viral replication in the brain including chronic systemic inflammation. However, to date, the precise viral dependent and independent changes to the brain of virally suppressed PWH remains unclear.

**Methods**: Here we comprehensively characterised the CNS reservoir and immune environment of SIV‐infected (SIV+) rhesus macaques during acute (n = 4), chronic (n = 16) or ART‐suppressed SIV infection (n = 11). Multiplex immunofluorescence for markers of SIV infection (vRNA/DNA) and immune activation was performed on frontal lobe and matched gut tissue. CNS and gut inflammation was also measured in an SIV‐uninfected model of chronic colitis, validated to mimic SIV‐induced gut damage, to determine the effect of gut damage on neuroinflammation independent of SIV infection.

**Results**: SIV+ animals contained viral DNA+ cells that were not reduced in the brain or gut by ART (P < 0.05), supporting the presence of a stable viral reservoir in these compartments. SIV+ animals had heightened levels of activated astrocytes (GFAP+) and microglia (Iba1+) producing antiviral (Mx1 and/or TGF‐β1) and oxidative stress markers (SOD1) as well as reduced blood‐brain barrier integrity than uninfected animals, and these dysfunctions were not abrogated by ART (P < 0.05 for all). Interestingly, measures of CNS immune activation and blood brain barrier integrity correlated with gut, but not CNS, viremia and immune activation in virally suppressed animals, supporting the role of systemic inflammation as a contributor to neuroinflammation. Furthermore, SIV‐uninfected animals with experimentally induced gut damage showed a similar immune activation profile in the brain to animals with SIV, supporting the role of chronic gut damage as an independent source of neuroinflammation.

**Conclusions**: Collectively, we show that ART‐suppressed SIV+ rhesus macaques exhibit impaired blood brain barrier integrity and heightened microglial and astrocyte activation which is associated in part with viral reservoirs and immune activation in the gut.

### PESUA25 Unvaccinated individuals admitted to the ICU due to fatal COVID‐19 showed progressive decay of unresponsive cytotoxic cells

G. Casado ^1^, M. Corona^2^, M. Torres^1^, L. Vigón^1^, A.J. Saez^2^, F. Ramos Martín^1^, E. Mateos^1^, S. Rodríguez Mora^1^, V. García‐Gutierrez^2^, M. Coiras^1^

^1^Instituto de Salud Carlos III, AIDS Immunopatology Unit, National Center of Microbiology, Madrid, Spain, ^2^Hospital Universitario Ramón y Cajal, Hematology Service, Madrid, Spain

**Background**: Individuals admitted to the ICU due to critical COVID‐19 show an ineffectual cytotoxic activity against SARS‐CoV‐2. We determined whether this impaired cytotoxic activity could be restored in PBMCs from individuals with critical and fatal COVID‐19.

**Methods**: 23 patients with critical COVID‐19 admitted to the ICU were divided in two groups according to the outcome: Exitus (n = 13) or Survival (n = 10). Blood samples were collected every 10–15 days during 80 days of hospitalization. Cytotoxic activity against SARS‐CoV‐2‐infected Vero E6 cells was analyzed after co‐culture (2:1) with PBMCs treated or not with IL‐15 (0.13μL/mL) 48h.

**Results**: 1) Median age was 65.0 (IQR 62.0–69.0) and 63.0 years (IQR 59.0–68.5) in Exitus and Survival groups, respectively. 73.9% individuals were men with dyslipidemia (52.2%), hypertension (40.1%), and/or diabetes mellitus (26.1%). 2) Mean length of hospital stay was 65.3 (SD: 37.1) and 68.1 days (SD: 29.3), respectively, whereas mean length of ICU stay was 53.8 (SD: 30.6) and 46.3 days (SD: 23.3). 3) Cytotoxic activity against SARS‐CoV‐2 of PBMCs in the Exitus group increased 1.9‐fold (p = 0.0313) after 21–35 days of hospitalization, but this response decayed steadily until the fatal outcome (**Figure 1**). In the Survival group, cytotoxic activity was 5.6‐fold (p = 0.0290) higher than the Exitus group at days 21–35 and it increased steadily until days 36–50. 4) Treatment with IL‐15 increased cytotoxic activity of PBMCs in both groups but it remained 6.2‐fold (p = 0.0303) lower in the Exitus group after 21–35 days. 5) CD8 count increased 2.1‐fold (p = 0.0409) in the Exitus group (t = 0) and remained enhanced until the fatal outcome.

**Figure:**
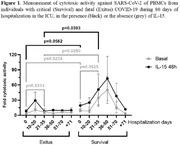
**Abstract PESUA25‐Figure 1**.

**Conclusions**: Individuals with fatal COVID‐19 showed increased levels of CD8 with impaired cytotoxic activity that were unresponsive to IL‐15‐induced proliferation. The reactivation of this impaired cytotoxic response appears to be essential to avoid a fatal outcome and has to be considered to develop new strategies against critical COVID‐19.

### PESUB14 Low frequency of activated T cells in people living with HIV, aviremic under treatment, and presenting neurocognitive disorders

L. Kundura ^1^, R. Cezar^2^, M. Pastore^3^, L.‐A. Gutierrez^4^, C. Berr^4^, C. Reynes^3^, J. Reynes^5,6^, A. Makinson^5,6^, P. Corbeau^1,6^

^1^Institute of Human Genetics ‐ CNRS, Homing, immune activation and infection, Montpellier, France, ^2^Nîmes University Hospital, Immunology Department, Nîmes, France, ^3^Institute of Functional Genomics ‐ CNRS, Montpellier, France, ^4^Institute for Neurosciences of Montpellier ‐ CNRS, Montpellier, France, ^5^Montpellier University Hospital, Infectious and Tropical Diseases Department, Montpellier, France, ^6^Montpellier University, Montpellier, France

**Background**: Prevalence of HIV‐associated neurocognitive disorders (HAND), either asymptomatic neurocognitive impairment (ANI), minor neurocognitive disorder (MND), or HIV‐associated dementia in people living with HIV (PLWH) under efficient combined antiretroviral therapy is high. Here, we looked for links between immune activation and HAND in an HIV‐population over 55 years age with controlled HIV‐disease.

**Methods**: This study is an ancillary study of the French national ANRS EP58 HAND 55–70 project (Clinical trial registration NCT02592174). We recruited 71 PLWH with a mean (±SD) age of 62±2.4 years; CD4 count, 553±249 cells/μL; CD4:CD8 ratio, 1.03±0.58; duration of infection, 20.0±7.7 years under efficient treatment during at least two years (<50 copies/mL and less than 2 viremic blips). We used the Frascati criteria to classify neurocognitive performances. We analyzed 31 peripheral blood soluble and cell surface markers of T cell, NK cell, monocyte, endothelial cell activation and inflammation by ELISA and flow cytometry. We performed two hierarchical clustering analyses, one at a participants’ level, and the other one at a markers’ level.

**Results**: We found that 53% of PLWH were classified as HAND (ANI, n = 21; MND n = 12). The proportion of CD8+ (44.3±10.3 vs. 52.6±10.4 %, p = 0.002), activated (HLA‐DR‐positive) CD4+ (19.6±10.3 vs. 28.9±14 %, p = 0.002) and activated (HLA‐DR‐positive) CD8+ (19.6±10.3 vs. 28.9±14.0 %, p = 0.025) T cells were lower in participants with ANI or MND than in participants without HAND. The double hierarchical clustering identified six different immune activation profiles in participants. Participants with one of these profiles, also characterized by a low frequency of circulating activated CD4+ T cells, presented more frequently ANI and MND (odds ratio 8.8 [95% CI 1.0–77.0], p = 0.041) than the participants with other profiles.

**Conclusions**: We did not find a positive correlation between HAND and circulating markers of immune activation in ageing PLWH. Our observation of a low percentage of activated T cells in peripheral blood raises the interesting hypothesis of a recruitment of these lymphocytes into the central nervous system.

### PESUB15 Incidence of and risk factors for heart failure subtypes in women and men living with HIV

D.B. Hanna ^1^, C.A. Bravo^2^, M.A. Weinreich^3^, S. Murthy^1^, P.O. Chavez^1^, K. Anastos^1^, M.J. Feinstein^4^, M.S. Ginsberg^1^, I.L. Pina^5^, U.R. Felsen^1^, P. Mirhaji^1^, M.J. Fazzari^1^, R.C. Kaplan^1,6^, J.R. Kizer^7,8^

^1^Albert Einstein College of Medicine, Bronx, United States, ^2^University of Washington, Seattle, United States, ^3^University of Colorado, Fort Collins, United States, ^4^Northwestern University, Chicago, United States, ^5^Central Michigan University, Mount Pleasant, United States, ^6^Fred Hutchinson Cancer Research Center, Seattle, United States, ^7^University of California San Francisco, San Francisco, United States, ^8^San Francisco Veterans Affairs Medical Center, San Francisco, United States

**Background**: People living with HIV (PLWH) have increased heart failure (HF) risk, but HF subtypes among PLWH remain less well‐characterized, especially among women. We estimated incidence of HF with preserved ejection fraction (HFpEF) and reduced ejection fraction (HFrEF) among PLWH receiving care in a large urban U.S. health system, and determined factors associated with each subtype.

**Methods**: We followed adult PLWH in outpatient care, defined as 2+ visits in 2011–2015, from the Einstein‐Rockefeller‐CUNY CFAR Clinical Cohort Database of PLWH in the Montefiore Health System (Bronx, NY). Incident HF cases were identified for review using diagnosis codes and NT‐proBNP levels, and confirmed independently by two cardiologists using MESA criteria (k = 0.70). HF was categorized as HFpEF or HFrEF based on left ventricular EF (≥50% vs. <50%). We estimated the age‐standardized incidence of HFpEF and HFrEF, and determined factors associated with each using Poisson regression, adjusting for time‐varying demographic, behavioral, and cardiometabolic characteristics.

**Results**: Among 8,199 PLWH (44% women, 44% Black, 41% Hispanic, 67% with suppressed viremia <200 copies/mL) contributing 31,612 person‐years, 123 incident HF cases were confirmed (53% HFrEF, 37% HFpEF, 10% EF unavailable). Age‐standardized HF incidence rates per 10,000 person‐years (95% CI) were 27.3 (21.6–32.9) overall, 14.2 (10.2–18.2) for HFrEF and 9.3 (6.1–12.5) for HFpEF. Men had higher HFrEF incidence than women (16.8 vs 11.1/10,000), whereas HFpEF incidence was more similar by sex (8.0 vs. 11.3/10,000). At HF diagnosis, median age was 56 for both subtypes; median BMI was 25.1 for HFrEF and 24.7 for HFpEF. NT‐proBNP, a marker of HF prognosis, was lower in HFpEF vs. HFrEF (median 2,686 vs. 4,075 pg/mL, p = 0.26). After adjustment, time‐varying hypertension history and diabetes were associated with HFpEF (incidence rate ratio, IRR 2.92, 95% CI 1.31–6.53 and 2.03, 95% CI 1.07–3.88), whereas male sex and unsuppressed viremia were associated with HFrEF (IRR 1.96, 95% CI 1.12–3.44 and 2.25, 95% CI 1.32–3.83). Lower CD4 count was associated with both HFpEF and HFrEF.

**Conclusions**: We identified differential risk factors associated with incident HFpEF and HFrEF among PLWH. Given the aging HIV population, HF prevention through virologic suppression and hypertension and diabetes control should be prioritized.

### PESUB16 The burden of and risk factors for maternal mental health and the impact on parenting by mothers living with HIV in rural Zimbabwe

R.M.S. Chingono ^1,2^, V. Simms^3^, F. Cowan^4^, L. Sherr^1^

^1^University College London, Institute of Global Health, London, United Kingdom, ^2^Biomedical Research and Training Institute, Social Science, Harare, Zimbabwe, ^3^London School of Hygiene and Tropical Medicine, London, United Kingdom, ^4^Liverpool School of Tropical Medicine, London, United Kingdom

**Background**: Mothers in limited‐resource settings, especially those living with HIV, are at increased risk of comorbidities, including mental health disorders. In sub‐Saharan Africa, mental health disorders are common in the population living with HIV, yet they remain undetected and untreated. We explored the prevalence of, and factors associated with prolonged depression and parenting stress in HIV positive mothers and their association with parenting behaviour. We hypothesised that prolonged depression leads to stress and hostile parenting.

**Methods**: We conducted a secondary analysis of a cluster‐randomised controlled trial in two districts in Zimbabwe between 2016–2017, enrolling 485 HIV positive mothers of infants. Outcomes included prolonged depression, measured using the 14‐item Shona Symptom Questionnaire (and defined as depression at baseline and 12 months follow up) and parenting stress using the Parenting Stress Index Short Form. We assessed mother‐child interaction, maternal social interaction, and health. Logistic regression was used to determine the correlates of prolonged depression and parenting stress.

**Results**: Overall, 26% of mothers experienced prolonged depression with no difference by the trial group. Risk factors for prolonged depression included being the only adult in the household (aOR = 2.49); food insecurity (aOR = 1.90); domestic violence (aOR = 3.45); mobility problems (aOR = 3.77); lack of social support (aOR = 1.33) and poor postpartum bonding (aOR = 2.52). For those in a relationship, prolonged depression was independently associated with impaired postpartum bonding and lower relationship quality. Overall, 73% of mothers with parenting stress were at risk of symptoms suggestive of prolonged depression. Factors associated with parenting stress included symptoms of prolonged depression and having pain or discomfort. 70% of mothers with prolonged depression had slapped, shaken or spanked their child and it was associated with increased parenting stress.

**Conclusions**: We found a high prevalence of prolonged depression and parenting stress among HIV positive mothers associated with food insecurity, experiencing pain or mobility problems, being less resilient and having poor relationships and low social support. There is a critical need to address depression and parenting stress both for its own sake and to benefit child outcomes.

### PESUB17 DTG associated weight gain: real or perceived? Real world experiences from Zimbabwe

T. Shamu ^1,2,3^, C. Chimbetete^1^, T. Mudzviti^1,4^, J. Manasa^5^, M. Egger^2,6,7^, N. Anderegg^2^

^1^Newlands Clinic, Harare, Zimbabwe, ^2^University of Bern, Institute of Social and Preventive Medicine, Bern, Switzerland, ^3^University of Bern, Graduate School of Health Sciences, Bern, Switzerland, ^4^University of Zimbabwe, Department of Pharmacy and Pharmaceutical Sciences, Harare, Zimbabwe, ^5^University of Zimbabwe, Innovation Hub, Harare, Zimbabwe, ^6^University of Cape Town, Centre for Infectious Disease Epidemiology and Research, School of Public Health and Family Medicine, Cape Town, South Africa, ^7^University of Bristol, Population Health Sciences, Bristol Medical School, Bristol, United Kingdom

**Background**: Dolutegravir (DTG) based regimens have been associated with weight gain in clinical trials. We compared real‐world weight changes after starting or switching of treatment for DTG, efavirenz (EFV), and atazanavir (ATV/r) based regimens.

**Figure:**
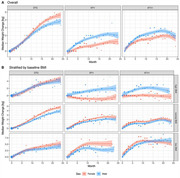
**Abstract PESUB17‐Figure 1**.

**Methods**: We included adults (≥18 years) starting or switching (defined as baseline) to EFV, ATV/r, or DTG between 2008 and 2021 at Newlands Clinic, Zimbabwe. We calculated weight changes for subsequent visits from the baseline weight. We aggregated data by treatment regimen, sex, and month after baseline and calculated median weight change for each data cell. We analyzed trends in median weight changes after baseline by generalized additive models. We included sex, regimen, and their interaction as fixed effects, and smoothed monthly trends by sex and regimen. We analyzed overall and baseline BMI category (<18.5 “underweight”, 18.5–24.9 “normal”, ≥25 “overweight/obese”) stratified weight gain.

**Results**: We included 59,564 weight measurements of 7047 adults, with 5342, 1108, and 597 being on DTG, EFV, and ATV/r, respectively. Two years post‐baseline, estimated median weight (95% CI) increased by 3.81kg (3.43–4.19), 2.01kg (1.58–2.44), and 1.92kg (1.52–2.31) for DTG, EFV and ATV/r, respectively in males and by 4.63kg (4.24–5.01), 1.21kg (0.81–1.61), and 1.61kg (1.23–2.00) for DTG, EFV and ATV/r, respectively in females (Figure). Overall, DTG‐based regimens showed a strong, almost linear increase in weight over time, with the inflection point towards the end of the two years, while weight gain plateaued with time for ATV/r and EFV‐based regimens. For patients underweight at baseline BMI, increases in weight were similar among treatment groups, while normal or overweight/obese patients had substantially larger weight gains with DTG‐based regimens.

**Conclusions**: Patients receiving DTG based regimens had a two‐ to four‐fold weight gain compared to EFV and ATV/r over two years, with little evidence of plateauing of the trend among those in on DTG.

### PESUB18 Progression of hepatic steatosis in people with HIV on integrase inhibitors

D. Kablawi ^1^, J. Milic^2^, A.S. Al Hinai^1^, B. Lebouche^1^, M. Klein^1^, M. Deschenes^1^, G. Guaraldi^2^, G. Sebastiani^1^

^1^McGill University, Montreal, Canada, ^2^University of Modena and Reggio Emilia, Modena, Italy

**Background**: Hepatic steatosis (HS) is frequent in people with HIV (PWH), due to viral hepatitis coinfection, overweight and antiretroviral therapy (ART). Recent data suggest weight increase in PWH on integrase inhibitors (INIs)‐based ART. The effect of this ART regimen on HS progression is not known.

**Figure:**
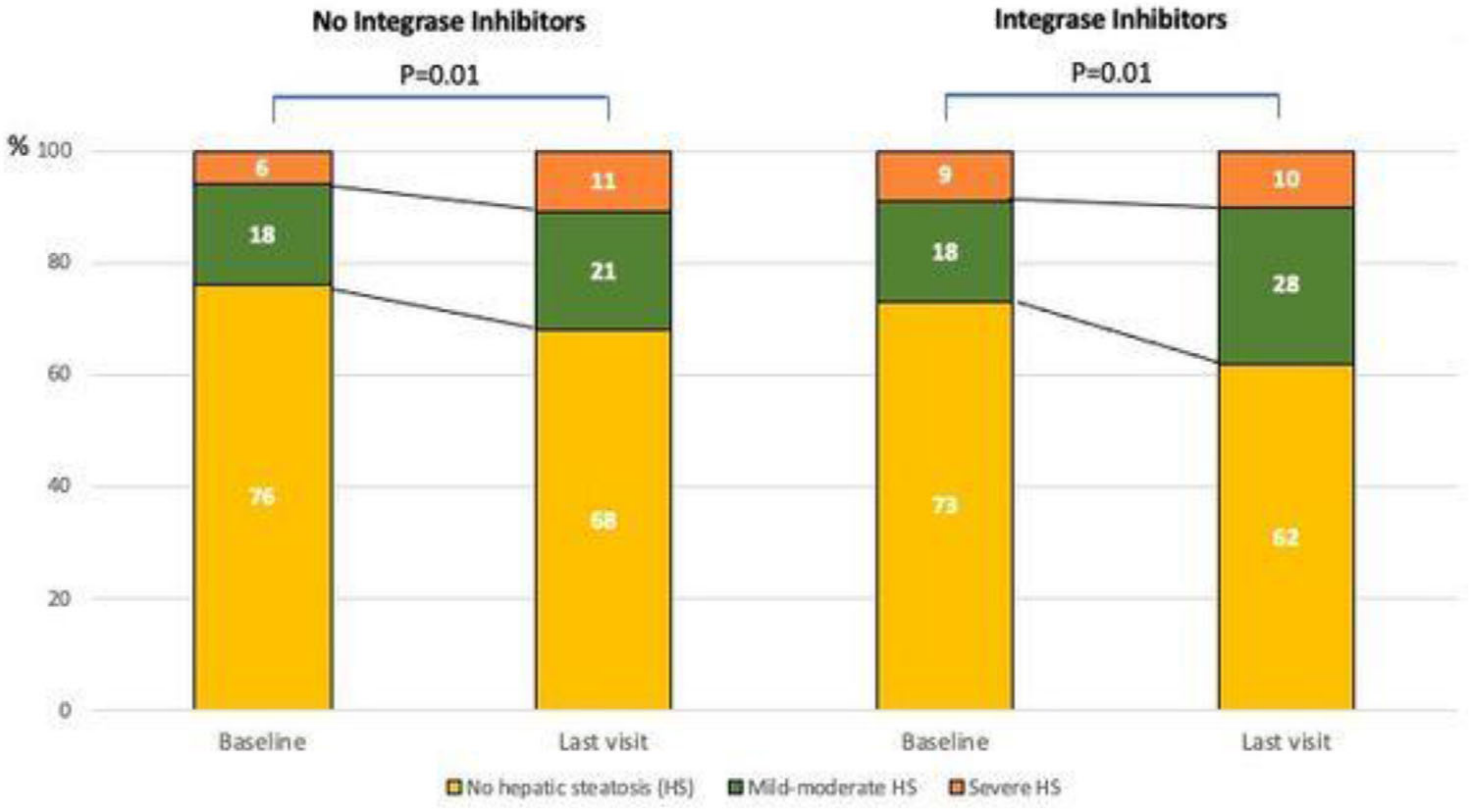
**Abstract PESUB18‐Figure 1**.

**Methods**: Fibroscan with controlled attenuation parameter (CAP) was performed in consecutive PWH from the LIVEHIV Cohort in Montreal. Overweight was defined as body mass index >25Kg/m^2^. Any grade and severe HS were defined as CAP > 270 and CAP > 330dB/m, respectively. HS progression was defined as development of any grade HS, or transition to severe HS if CAP < 330 at baseline. We compared incident overweight and HS progression in PWH with and without INIs‐based ART. Covariate adjustments for HS progression were evaluated by multivariable Cox regression analysis.

**Results**: We included 393 PWH (mean age 50yrs, HIV duration 16yrs, BMI 26Kg/m^2^; 76% male, 32% with viral hepatitis coinfection, 29% on INIs). Prevalence of any grade and severe HS at baseline was 25% and 7%, respectively. During a median follow‐up of 2.7yrs, incidence rate of overweight was similar in PWH with (28.8 per 100 person‐years [PY], 95% CI 23.8–50.5) or without (36.2 per 100 PY, 95% CI 29–45.1) INIs‐based ART (log‐rank: p = 0.50). Progression of HS between baseline and last visit was observed in both PWH with and without INIs‐based ART (see Figure). Progression rate of HS was 14.3 (95% CI 11.5–17.9) and 11.9 (95% CI 7.1–20.9) per 100 PY, in PWH with or without INIs‐based ART (log‐rank: p = 0.46). In multivariable analysis and after adjusting for HIV duration, overweight (adjusted hazard ratio 2.91, 95% CI 1.33–6.36) was associated with progression of HS while INIs‐based ART was not.

**Conclusions**: PWH on INIs‐based ART did not exhibit accelerated progression of HS. Incident overweight was similarly observed in PWH with or without INIs‐based ART. Overweight was associated with HS progression.

### PESUB19 Rapid ART initiation: a four‐years observational study

R. Pincino ^1,2^, L. Taramasso^3^, M. Berruti^1,2^, G. Cenderello^1^, M. Bassetti^2,3^, A. Di Biagio^2,3^

^1^Borea Hospital, Infectious Diseases Clinic, Sanremo, Italy, ^2^University of Genoa, Health and Science Department, Genoa, Italy, ^3^Policlinico San Martino Hospital, Infectious Diseases Clinic, Genoa, Italy

**Background**: Rapid initiation of antiretroviral therapy (ART) benefits both people living with HIV (PLWH) and the community. We assessed predictors of virological suppression and adverse outcome in a cohort of newly diagnosed PLWH, comparing rapid ART initiation with other treatment strategies.

**Figure:**
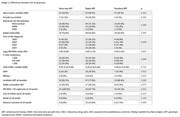
**Abstract PESUB19‐Figure 1**.

**Figure:**
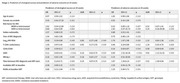
**Abstract PESUB19‐Figure 2**.

**Methods**: Observational, single center, retrospective study.

The study population was divided into: “same‐day ART” (0–3 days post diagnosis/referral), “rapid ART” (4–14 days post‐diagnosis/referral), and “early ART” (more than 14 days after diagnosis).

Predictors of virological suppression (HIV RNA < 50 copies/mL) and of adverse outcome (composite of lack of virological suppression, loss to follow up and death) within 24 weeks were analyzed using a binomial regression model.

**Results**: 116 PLWH were enrolled from January 2018 to December 2021, 33 (28.4%) in same‐day ART, 61 (52.6%) in rapid ART and 22 (19.0%) in early ART group; baseline characteristics are shown in image 1. Same day and rapid ART strategies became more used, with two (9.1%) people starting ART more than 14 days after HIV diagnosis in 2021 compared with 9 (40.9%) in 2018. PLWH in the same day ART group showed higher rates of virological suppression and lower rates of adverse outcomes.

AIDS diagnosis (p 0.025) and a longer time between HIV diagnosis and ART initiation (p 0.014) were negatively associated with virological suppression; the same variables were predictors of adverse outcome at multivariate analysis (Image 2).

**Conclusions**: Rapid ART initiation is associated with improved clinical outcomes and represents a key strategy to control the HIV epidemic and optimize the health of PLWH.

### PESUB20 Ibalizumab long‐term efficacy is not impacted by partially active antiretrovirals

J. Leider ^1^, T. McLaughlin^2^, A. Lee^2^, C. McGary^2^, P. Mesquita^2^

^1^Jacobi Medical Center, Medicine, Bronx, United States, ^2^Theratechnologies, Inc., Medical Affairs, Montreal, Canada

**Background**: Ibalizumab (IBA) is a long‐acting post‐attachment inhibitor approved for heavily treatment‐experienced (HTE) adults with multidrug resistant HIV‐1 failing their current antiretroviral (ARV) regimen. We have previously demonstrated IBA's efficacy in the TMB‐301/311 clinical trials. Complex resistance profiles in HTE patients limit the number of fully active agents. Therefore, partially active ARVs are required to build optimized background regimens (OBR) to maintain suppression. As such, we sought to characterize the complexity of OBR in TMB‐301/311 and determine its impact on the durability of response to IBA.

**Methods**: In TMB‐301/311, 40 viremic patients received a 2000 mg loading dose of IBA followed by 800 mg doses every 2 weeks up to 96 weeks. OBR was incorporated 7 days after starting IBA. Baseline resistance mutations were entered into the Stanford database to scale the activity of each ARV in the patient's OBR 0–1. Drug activities were summed to generate a continuous genotypic susceptibility score (CGSS), which was averaged when OBRs were adjusted. The impact of CGSS on virologic outcomes was evaluated by linear or logistic regression, controlling for baseline viral load and CD4 count.

**Results**: The study population was 85% male with a median age of 53 and 23 years of HIV infection. Baseline median viral load and CD4 count were 35,350 copies/mL and 73 cells/mm^3^, respectively. The median number of drugs in participants’ OBRs was 4. Despite this, the median CGSS of OBR was only 1.58, indicating many partially active agents, the most common being tenofovir. Average CGSS was not associated with decrease in viral load at week 96 (p = 0.18), maximal decrease in viral load (p = 0.59), or viral suppression (p = 0.69). In addition, no CGSS threshold was associated with virologic outcomes, even after weighting boosted PIs and DTG to account for differences in potency and barrier to resistance amongst ARVs.

**Conclusions**: The high disparity between the number of drugs in patients’ OBR and their median CGSS highlights the challenge of building OBR in HTE patients. Importantly, IBA remained effective across a range of CGSS scores through week 96, demonstrating the durability of IBA in HTE patients despite combination with partially active agents.

 

**Figure:**
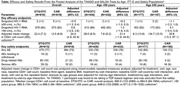
**Abstract PESUB21‐Table 1**.

### PESUB21 Efficacy and Safety of Switching to Dolutegravir/Lamivudine (DTG/3TC) in Treatment‐Experienced, Virologically Suppressed PLHIV Aged ≥50 Years: Pooled Results From the TANGO and SALSA Studies

S. Walmsley ^1^, D.E. Smith^2^, M. Górgolas^3^, P.E. Cahn^4^, T. Lutz^5^, K. Lacombe^6^, P.N. Kumar^7^, B. Wynne^8^, R. Grove^9^, G. Bontempo^8^, R. Moodley^10^, F. Spinelli^8^, B. Jones^10^, C. Okoli^10^, M. Ait‐Khaled^10^

^1^University Health Network, Toronto, Canada, ^2^Albion Centre, Sydney, Australia, ^3^Fundación Jiménez Díaz, Universidad Autónoma de Madrid, Madrid, Spain, ^4^Fundación Huésped, Buenos Aires, Argentina, ^5^Infektiologikum, Frankfurt, Germany, ^6^Hôpital Saint‐Antoine, Paris, France, ^7^Georgetown University Medical Center, Washington, DC, United States, ^8^ViiV Healthcare, Research Triangle Park, United States, ^9^GlaxoSmithKline, Brentford, United Kingdom, ^10^ViiV Healthcare, Brentford, United Kingdom

**Background**: As older adults living with HIV (OALHIV) are one of the fastest growing populations, concerns over managing age‐related comorbidities and polypharmacy while maintaining virologic suppression highlight the importance of their inclusion in clinical trials. DTG/3TC is an international guidelines–recommended 2‐drug regimen demonstrating high efficacy and barrier to resistance. We present pooled TANGO and SALSA efficacy and safety analyses in participants aged ≥50 years.

**Methods**: Week 48 data from the phase 3 TANGO and SALSA trials evaluating switch to DTG/3TC vs continuing current antiretroviral regimen (CAR) were pooled. Proportions of participants with HIV‐1 RNA ≥50 and <50 c/mL (Snapshot, ITT‐E) and safety were analyzed by age. Adjusted mean change from baseline in CD4+ cell count was assessed using mixed‐models repeated‐measures analysis.

**Results**: Of 1234 participants, 29% were aged ≥50 years (9% female; 3% aged ≥65 years). At baseline, participants aged ≥50 vs <50 years had greater concomitant medication use (median (range): 2.0 [0–20] vs 1.0 [0–16], respectively) and more comorbidities (86% vs 71%); baseline characteristics were otherwise similar. Among those aged ≥50 years, 1 (0.6%) DTG/3TC participant and 3 (1.6%) CAR participants developed HIV‐1 RNA ≥50 c/mL; proportions with HIV‐1 RNA <50 c/mL were high, consistent with overall efficacy (Table). CD4+ cell count increased from baseline in DTG/3TC participants in both age groups. No participants in the DTG/3TC group had confirmed virologic withdrawal (CVW); 1 participant in the CAR group had CVW (no resistance detected). Proportions of AEs, AEs leading to withdrawal, and serious AEs in DTG participants were similar between age groups.

**Conclusions**: Although participants aged ≥50 years used a higher number of concomitant medications and had a greater prevalence of comorbidities, switching to DTG/3TC maintained virologic suppression, demonstrating robust efficacy, a high barrier to resistance, and good safety and tolerability.

### PESUB22 Pharmacokinetics of a Simplified Subcutaneous Lenacapavir Regimen versus Phase 2/3 Regimen

V. Jogiraju ^1^, H. Graham^1^, S. West^1^, J. Ling^1^, J. Cuvin^1^, M. Rhee^1^, R. Palaparthy^1^, R. Singh^1^

^1^Gilead Sciences, Inc., Foster City, United States

 

**Figure:**
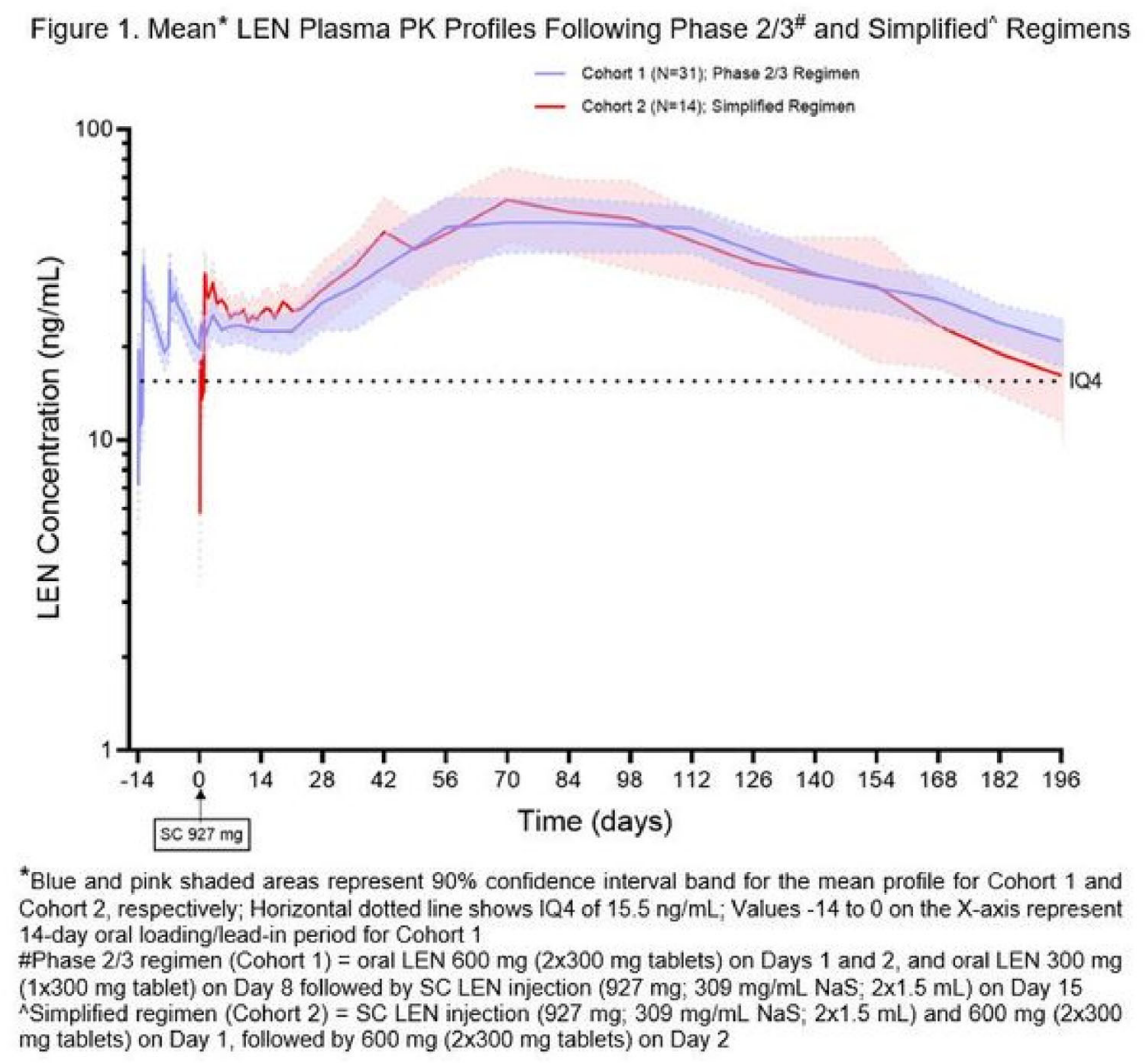
**Abstract PESUB22‐Figure 1**.

**Background**: Lenacapavir (LEN) is a potent, first‐in‐class, HIV‐1 capsid inhibitor currently in clinical development for HIV‐1 infection treatment and prevention. The ongoing Phase 2/3 studies in people with HIV‐1 uses every 6 months subcutaneous (SC) dosing injection with oral loading/lead‐in (oral LEN 600 mg on Days 1 and 2, and oral LEN 300 mg on Day 8 followed by SC LEN 927 mg on Day 15 and every 6 months thereafter). While this Phase 2/3 regimen has been shown to be safe and effective, a more simplified regimen (oral LEN 600 mg on Days 1 and 2, with SC LEN 927 mg on Day 1 and every 6 months thereafter) can be more convenient. Our objective was to compare the pharmacokinetics (PK) of the simplified regimen with that of the Phase 2/3 regimen.

**Methods**: 31 and 14 healthy participants received the Phase 2/3 regimen (Cohort 1) and the simplified regimen (Cohort 2), respectively, in a Phase 1 single subcutaneous dose study. Intensive LEN PK and safety through Day 196 were summarized. PK was evaluated using noncompartmental analysis.

**Results**: LEN C_max_ (within ±8%) and AUC_0–196 days_ (within ±15%) were comparable between regimens. Mean LEN concentrations achieved the efficacy target (inhibitory quotient of 4 [IQ4] = 15.5 ng/mL) rapidly and were maintained above IQ4 through the dosing interval. LEN was well tolerated with no Grade 3 or 4 adverse events (AEs), serious AEs or deaths reported. Most common AEs were injection site reactions.

**Conclusions**: LEN concentrations of the simplified regimen were generally comparable to those of the Phase 2/3 regimen. LEN concentrations reached efficacious target rapidly and were maintained through the dosing interval. These results suggest that the simplified regimen provides similar exposures to the Phase 2/3 regimen and can be utilized as a potential clinical regimen for treatment and prevention of HIV‐1 infection.

### PESUB23 Simulations for Once Weekly Dosing of Oral Lenacapavir

N. Shaik ^1^, H. Zhang^1^, S. Girish^1^, M. Rhee^1^, R. Palaparthy^1^, R. Singh^1^

^1^Gilead Sciences, Clinical Pharmacology, Foster City, United States

**Background**: Lenacapavir (LEN), a potent first‐in‐class inhibitor of HIV‐1 capsid function, is in development for thetreatment and prevention of HIV‐1 infection. Current data indicates that LEN exhibits near maximal antiviral activity when the lower bound of the 90% confidence interval (CI) of mean C_trough_ are maintained above inhibitory quotient 4 (IQ4) (at least 4‐fold greater than the *in vitro* protein adjusted 95% effective concentration). The objective of this analysis was to utilize a population pharmacokinetic (PopPK) model to simulate various oral weekly dosing regimens of LEN that would rapidly achieve and maintain concentrations above IQ4.

**Methods**: A 2‐compartment PopPK model with first order absorption and linear elimination was previously developed to describe LEN concentration data from multiple clinical studies (384 participants). This model was utilized to simulate various dosing regimens (loading + maintenance doses) that can achieve efficacious LEN concentrations rapidly and maintain it throughout the dosing interval. Additionally, various scenarios of missed oral doses were simulated to evaluate the forgiveness window.

**Results**: Simulations showed that an oral loading dose of 600 mg on day 1 and day 2 followed by 300 mg oral once weekly doses maintained the lower bound of the 90% CI of mean C_trough_ above IQ4 (15.5 ng/mL) throughout the dosing interval (Figure 1). This dosing regimen reached IQ4 rapidly within 4 hours. In addition, simulations also showed that oral LEN administered once weekly is expected to maintain concentrations above IQ4 with a forgiveness window of up to 7 days after the last missed dose.

**Conclusions**: Once weekly oral LEN 300 mg is expected to maintain concentrations above IQ4 throughout the dosing interval while allowing for a 7‐day forgiveness window after the last missed oral dose. Oral LEN can be developed as part of a complete oral weekly regimen for the treatment of HIV‐1 infection.

**Figure:**
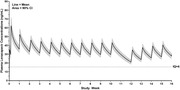
**Abstract PESUB23‐Figure 1**.

### PESUB24 A study evaluating the safety, tolerability, and pharmacokinetics of a high‐concentration (CAB 400 mg/ml) cabotegravir long‐acting injectable formulation following subcutaneous and intramuscular administration in healthy adult participants

P. Benn ^1^, K. Han^2^, J. Sievers^1^, J. Sadik Shaik^2^, M. Warwick‐Sanders^3^, B. Win^3^, D. Dorey^4^, M. Baker^5^, C. Leemereise^6^, K. Offenbecker^7^, C. Garis^8^, D.B. Brimhall^9^, C. Boyle^10^, C. Schwabe^11^, D. Taylor^12^, M.A. Hassman^13^, A. Wolstenholme^2^, S. Knowles^14^, W. Spreen^8^, M. Lataillade^15^

^1^ViiV Healthcare, Brentford, United Kingdom, ^2^GlaxoSmithKline, Collegville, United States, ^3^GlaxoSmithKline, Brentford, United Kingdom, ^4^GlaxoSmithKline, Mississauga, Canada, ^5^ViiV Healthcare, Nyon, Switzerland, ^6^GlaxoSmithKline, Amersfoort, Netherlands, the, ^7^GlaxoSmithKline, Upper providence, United States, ^8^ViiV Healthcare, North Carolina, United States, ^9^PPD, Inc, Las Vegas, United States, ^10^PPD, Inc., Austin, United States, ^11^New Zealand Clinical Research, Auckland, New Zealand, ^12^PPD, Inc., Orlando, United States, ^13^Hassman Research Institute, Berlin, United States, ^14^Halozyme Therapeutics, Inc., San Diego, United States, ^15^ViiV Healthcare, Branford, United States

**Background**: Long‐acting (LA) injectable cabotegravir (CAB200mg/mL) administered intramuscularly is approved for HIV‐1 prevention (every‐2‐months), and treatment (suppressed switch) with rilpivirine (monthly or every‐2‐months). A high‐concentration (CAB400mg/mL) formulation was developed to support less frequent dosing and/or potential self‐administration via subcutaneous or thigh injections.

**Methods**: The safety and pharmacokinetics of single/repeat administration of CAB400mg/mL 200–800mg (Cohorts 1–4, 4b, 4h, 5) intramuscularly (*gluteus medius*, *vastus lateralis*) or subcutaneously (abdominal) in healthy adults was evaluated in this ongoing Phase I study (NCT04484337). CAB200mg/mL active controls (n = 1–2 per cohort) were matched by dose or volume. In Cohort 4h, recombinant human hyaluronidase rHuPH20 will be co‐administered subcutaneously. Pharmacokinetic parameters were estimated via noncompartmental analysis and population pharmacokinetic (PPK) modelling. Simulations were performed using PPK model to assess various CAB400mg/mL regimens. Participant‐reported outcome measures (PROMs) were also assessed.

**Table:** 

**Abstract PESUB24‐Table 1. Safety Summary of CAB400mg/mL in Healthy Adult Participants (Cohorts 1–4)**						
	IM gluteal		IM thigh		SC abdominal	
	600mg [1.5mL] (n = 18)	400mg [1mL] (n = 34)	600mg [1.5mL] (n = 11)	400mg [1mL] (n = 12)^*^	600mg [1.5mL] (n = 9)	200mg [0.5mL] (n = 24)
Any AE, n (%) Drug‐related AEs, n (%) Drug‐related serious AEs, n (%) Drug‐related AEs leading to withdrawal, n (%)	17 (94) 17 (94) 0 2 (11)	33 (97) 33 (97) 0 1 (3)	11 (100) 11 (100) 0 1 (9)	12 (100) 12 (100) 0 0	9 (100) 9 (100) 0 0	24 (100) 24 (100) 0 0
Any ISR AE, n (% of injections) Grade 1 events, n (% of ISRs)^†^ Grade 2 events, n (% of ISRs)^†^ Grade 3 events, n (% of ISRs)^†^	17 (94) 8 (47) 8 (47) 1 (6)	33 (97) 19 (58) 10 (30) 4 (12)	11 (100) 4 (36) 6 (55) 1 (9)	11 (92) 4 (36) 6 (55) 1 (9)	9 (100) 2 (22) 6 (67) 1 (11)	24 (100) 6 (25) 16 (67) 2 (8)

Note. Participants receiving CAB400mg/mL across Cohorts 1–4 were pooled by administration route and dose. All participants were scheduled to receive two injections. Each participant received a single injection at one dose/administration route (except the one detailed below), with the other injection being at a different dose/administration route (as seen in Table 2). Therefore, the n numbers are event level and represent the number of participants receiving an injection at each dose/administration route.

^*^One participant received two injections at the indicated dose/volume for injection 1 and 2 and is therefore counted twice.

^†^Maximum grade reported following each injection. The denominator is the total number of injections leading to ≥1 ISR. There were no Grade 4 or 5 ISRs.

AE, adverse event; CAB, cabotegravir; gluteal, *gluteus medius*; IM, intramuscular; ISR, injection site reaction; SC, subcutaneous.

**Table:** 

**Abstract PESUB24‐Table 2. Plasma Pharmacokinetic Parameters of CAB400mg/mL in Healthy Adult Participants (Cohorts 1–4)**								
Cohort route	Cohort 1 IM gluteal		Cohort 2 SC abdominal		Cohort 3 IM thigh		Cohort 4 IM gluteal	Cohort 4 SC abdominal
Injection dose	Injection 1600mg (n = 18)	Injection 2400mg (n = 16)	Injection 1600mg (n = 9)	Injection 2200mg (n = 8)	Injection 1600mg^*^ (n = 13)	Injection 2400mg (n = 10)	Injection 1400mg (n = 18)	Injection 2200mg (n = 16)
C_max_ (μg/mL)	6.51 (28.6%)	7.10 (25.8%)	6.76 (37.7%)	4.32 (33.5%)	7.14 (69.4%)	5.87 (75.3%)	3.98 (61.6%)	3.03 (21.1%)
Concentration at: Week 4 (μg/mL) Week 8 (μg/mL)	2.78 (45.2%) NA	2.90 (37%) 0.745 (131.5%)	1.31 (82.4%) NA	1.29 (65.3%) 0.308 (95.8%)	0.784 (198.6%) NA	1.59 (57.2%) 0.24 (215.6%)	1.22 (31.7%) NA	1.26 (38.6%) 0.328 (13.8%)
LA absorption rate constant (h^–1^)	NA	0.00155 (56.2%)	NA	0.00203 (62.8%)	NA	0.00191 (112.3%)	NA	0.00186 (40.8%)
Terminal half‐life (weeks)	NA	2.67 (56.2%)	NA	2.03 (62.8%)	NA	2.16 (112.3%)	NA	2.22 (40.8%)

PK parameters were estimated using noncompartmental analysis. Values displayed are geometric mean (CV%).

^*^Two participants in Cohort 3 received 400mg instead of 600mg for injection 1, and their plasma concentrations were increased by 50% (dose normalized to 600mg) for estimating PK parameters.

CAB, cabotegravir; C_max_, maximum plasma concentration post IM injection; gluteal, *gluteus medius*;

IM, intramuscular; LA, long‐acting; NA, not applicable; PK, pharmacokinetic; SC, subcutaneous (abdominal); thigh, *vastus lateralis*.

**Results**: Seventy participants received CAB (oral and/or injection) across Cohorts 1–4; 40% were female (sex), 40% were non‐White. Median age, weight, and BMI were 34y, 76.9kg, and 26.1kg/m^2^, respectively. Overall, safety profiles, including injection site reaction (ISR) frequency, were similar between formulations. CAB400mg/mL ISRs occurred in 92–100% of injections; most were Grade 1–2 (88–94%, maximum grade) (**Table 1**). Erythema and induration/swelling occurred more commonly after subcutaneous versus intramuscular injections. CAB400mg/mL pharmacokinetics were similar across administration routes (**Table 2**). CAB400mg/mL C_max_ was higher and half‐life was 62% shorter than CAB200mg/mL after adjusting for demographics. CAB400mg/mL administered monthly, regardless of route, was predicted to exceed the plasma concentrations of approved CAB200mg/mL regimens. The conference presentation will include Cohort 4b, 4h, and 5 and PROM results.

**Conclusions**: CAB400mg/mL could potentially expand options for LA injectable ART, and these interim safety and pharmacokinetic data support further clinical evaluation.

### PESUB25 Bridging the gap between patient perceptions and delivered care among people living with HIV in the Asian region

S. Park^1^, Y.‐H. Hsiao^2^, F. Yu^3^, K. Kambara^4^, B. Allan^5^, G. Brough^6^, T.‐F. Hwang ^7^, N. Dang^7^, B. Young^8^, R. Patel^9^, A. Maldonado^10^, N. Nwokolo^9^, C. Okoli^9^

^1^Love4One, South Korea, Seoul, Korea, The Republic of, ^2^Positive Alliance, Taiwan, Taipei, Taiwan, Province of China, ^3^Danlan Beijing Media Limited, China, Beijing, China, ^4^Japanese Network of People living with HIV/AIDS, Japan, Tokyo, Japan, ^5^Australasian Society for HIV, Viral Hepatitis and Sexual Health Medicine, Melbourne, Australia, ^6^Positively UK, United Kingdom, London, United Kingdom, ^7^ViiV Healthcare, Medical Affairs, Singapore, Singapore, ^8^ViiV Healthcare, RTP Durham, United States, ^9^ViiV Healthcare, Brentford, United Kingdom, ^10^ViiV Healthcare, Wavre, Belgium

**Background**: Client‐centered care is recommended for the care of people living with HIV (PLHIV) to improve client retention and treatment adherence. We assessed how Asian PLHIV perceived their personal healthcare needs/priorities were met, and how these beliefs were associated with health‐related outcomes.

**Methods**: 230 PLHIV aged ≥18 years on anti‐retroviral therapy (ART) completed the 2019 Positive Perspectives survey from Taiwan (n = 55), Japan (n = 75), China (n = 50), and South Korea (n = 50). Using logistic regression adjusting for age, gender, and HIV duration, we explored how having personal health needs met was associated with ART‐related perceptions and self‐rated health.

**Results**: Overall, 57.4% perceived their provider met their personal healthcare needs (South Korea, 38.0%; China, 56.0%; Japan, 62.7%; Taiwan, 69.1%). Prevalence of ART satisfaction was 50.0% (South Korea, 34.0%; China, 40.0%; Japan, 54.7%; Taiwan, 67.3%). However, 27.0% (China, 38.0%; South Korea, 28.0%; Japan, 24.0%; Taiwan, 20.0%) would not discuss treatment concerns with providers believing, “I don't think my main HIV care provider's priorities are the same as mine”. Participants perceiving their personal needs were met had higher odds of reporting ART satisfaction (AOR = 5.83, 95%CI = 3.19–10.64), optimal overall health (AOR = 2.98, 95%CI = 1.67–5.31), and greater self‐efficacy in managing their daily ART (AOR = 2.31, 95%CI = 1.32–4.04). Yet, over one‐fourth of those wanting more involvement in healthcare decisions indicated their viewpoint was not sought by their provider before prescribing new treatments (overall, 29.1%[46/158]; China, 31.1%[14/45], Japan, 25.0%[10/40]; South Korea, 41.4%[12/29]; Taiwan, 22.7%[10/44]). Regional variations were seen in the extent to which PLHIV's concerns were collaboratively addressed with providers. For example, while Chinese participants reported the highest overall percentage of those who missed ≥one ART dose in the past month because of concerns over long‐term impacts of ART (China, 70.0%; South Korea, 42.0%; Taiwan, 29.1%; Japan, 26.7%), Chinese participants reported the lowest percentage of those whose ART had been changed in the past year by their provider to mitigate their concerns about long‐term impacts among those with such concerns (China, 4.4%[2/46]; Taiwan, 9.5%[4/42]; South Korea, 10.5%[4/38]; Japan, 25.9%[14/54]).

**Conclusions**: Improving client‐provider relationships may improve care continuum and treatment satisfaction. ART planning should be done proactively and collaboratively between PLHIV and providers to deliver person‐centered care.

### PESUB26 The first‐in‐human clinical trial of STP0404, a novel potent HIV‐1 allosteric integrase inhibitor

X. Meng ^1^, U.‐I. Kim^1^, Y. Donazzolo^2^, B. Kim^3,4^, K. Kim^1^

^1^ST Pharm Co., Ltd., Seoul, Korea, The Republic of, ^2^Eurofins Optimed S.A.S, Grenoble, France, ^3^Emory University, School of Medicine, Department of Pediatrics, Atlanta, United States, ^4^Children's Healthcare of Atlanta, Center for Drug Discovery, Atlanta, United States

**Background**: STP0404 is a novel HIV‐1 allosteric integrase inhibitor with potent *in vitro* anti‐HIV‐1 activities, an *in vitro* resistance profile different from those of other catalytic‐site integrase inhibitors, and favorable nonclinical safety and PK profiles.

**Methods**: The safety and PK of STP0404 was evaluated in a double‐blinded, placebo‐controlled, randomized phase 1 clinical trial in healthy male adult volunteers with once daily regimen. Single and repeated administration with ascending doses (200, 400, 600 and 800 mg for SAD (Single Ascending Doses), 200 and 400 mg for 10 days for MAD (Multiple Ascending Doses)) and food effect (200 mg) were evaluated through this trial.

**Results**: A total of 65 male subjects were enrolled (aged 18 to 45 years old). Most AEs were mild (75%, 21/28), headache (9/28) and diarrhea (5/28) were most frequently observed. No severe AE, SAE and withdrawn due to AE observed or occurred during the trial.

PK was linear but less‐proportional over the dose ranged administered except for SAD 800 mg. C_max_ were reached at 4 to 6 hours throughout the whole study. Accumulation ratio is around 1.3 at steady‐state (D10). Food effect factor was around 1.5 to 2, and resulted in a lower variability in drug exposure. AUC_0‐τ_ and C_max_ in plasma ranged from 23.6 h·μg/mL and 2.05 μg/mL at a 200 mg dose (fasted) to 67.7 h·μg/mL and 6.16 μg/mL at a 200 mg dose (fed, steady‐state), respectively. Steady state was reached between Day 3 and Day 6, The mean C_ss,24h_ with a 200 mg dose was 1.37 μg/mL, approximately 600‐fold of the protein‐adjusted EC_95_ (0.0022 μg/mL).The elimination half‐life were about 18 to 33 hours throughout the study. MAD 400 mg PK data will be ready before presentation.

**Conclusions**: STP0404 was well tolerated and its PK profile indicated a once‐daily regimen at low dose level given after meal will achieve therapeutic concentrations. A Phase 2a clinical trial of STP0404 is planned to start at 3Q, 2022.

### PESUC18 Disproportionate HIV burden among key populations in sub‐Saharan Africa: national estimates of population size, HIV prevalence, and ART coverage

O. Stevens ^1^, K. Sabin^2^, S. Arias García^2^, R. Anderson^1^, K. Willis^3^, E. Grard^1^, A. Stuart‐Brown^1^, F. Cowan^4^, L. Degenhardt^5^, J. Zhao^6^, M. Maheu‐Giroux^7^, L. Platt^8^, B. Rice^8^, I. Sathane^9^, M. Boothe^10^, S. Baral^3^, M. Mahy^2^, J. Eaton^1^

^1^Imperial College London, London, United Kingdom, ^2^UNAIDS, Geneva, Switzerland, ^3^Johns Hopkins University, Baltimore, United States, ^4^Centre for Sexual Health and HIV/AIDS Research, Harare, Zimbabwe, ^5^University of New South Wales, Sydney, Australia, ^6^The Global Fund to fight AIDS, TB, and Malaria, Geneva, Switzerland, ^7^McGill University, Montreal, Canada, ^8^London School of Hygiene and Tropical Medicine, London, United Kingdom, ^9^Ministry of Health, Maputo, Mozambique, ^10^UNAIDS, Maputo, Mozambique

**Background**: The Global AIDS Strategy 2021–2026 calls for equitable programme access to end HIV/AIDS by 2030. Robust HIV data among key populations (KP) are important for monitoring and reducing inequality in the global HIV/AIDS response.

**Figure:**
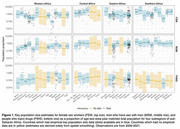
**Abstract PESUC18‐Figure 1**.

**Figure:**
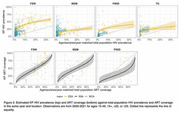
**Abstract PESUC18‐Figure 2**.

**Methods**: We systematically collated population size (PSE), HIV prevalence, and ART coverage data for female sex workers (FSW), men who have sex with men (MSM), people who inject drugs (PWID), and transgender (TG) populations in sub‐Saharan Africa (SSA) 2000–2021 from existing databases and primary source review. We used spatial random‐effects regression to pool and extrapolate data relative to age‐sex‐year‐area matched total population estimates.

**Results**: We extracted 1557 datapoints for PSE [FSW (n = 670); MSM (n = 522); PWID (n = 296); TG (n = 69)], 1248 HIV prevalence datapoints, and 212 ART coverage datapoints. Across countries, median 0.65% of women were FSW (interquartile range [IQR] 0.44–1.2%); 0.56% of men were MSM (IQR 0.33–0.72%); and 0.1% of adults injected drugs (IQR 0.08–0.15%) (Figure 1).

In Eastern and Southern Africa, HIV prevalence among FSW, PWID, and TG was correlated with and higher than total population prevalence, but less correlated for MSM. In Western and Central Africa, KP HIV prevalence was higher than population prevalence with weak correlation. FSW and MSM ART coverage was similar to population ART coverage, and PWID ART coverage was lower (Figure 2). Insufficient data were available to estimate TG PSE or ART coverage.

**Conclusions**: National‐level KP estimates may guide HIV programming to reduce inequality, but highlight insufficient data for many countries. HIV surveillance for PWID and TG should be prioritised to improve programmatic responses.

### PESUC19 Assessing levels of provincial HIV virological suppression in the public health sector in South Africa during the COVID‐19 pandemic

L. Hans ^1,2^, N. Cassim^1,2^, S. Sarang^2^, S. Ndlovu^2^, P. Da Silva^1,2^, W.S. Stevens^1,2^

^1^University of the Witwatersrand, Faculty of Health Sciences, Molecular Medicine and Haematology, Johannesburg, South Africa, ^2^National Health Laboratory Service, National Priority Programme, Johannesburg, South Africa

**Background**: In December 2019, the first reports of a novel coronavirus infection originated from the city of Wuhan, China. The World Health Organization named the new disease COVID‐19, which spread globally and was classified as a pandemic in March 2020. Many countries, including South Africa, introduced social distancing and lockdown rules to limit transmission. South Africa has experienced four waves of infection with rises in the number of diagnosed cases. A local study has reported reductions in average weekly HIV viral load (VL) testing due to legislated lockdown levels. This study aims to assess the impact of COVID‐19 on provincial HIV viral load (VL) suppression (<50 copies/ml).

**Methods**: Specimen‐level VL data were extracted from the corporate data warehouse (CDW) for the period January 2019 to December 2021. We assessed the provincial percentage of samples with virological suppression (<50 copies/ml) by calendar year. Data without a province indicated in the CDW were excluded. The change in the provincial proportion of samples with virological suppression (VS) in 2019 was compared to the subsequent years where lockdown was implemented (percentage change).

**Results**: Data was reported for 17,460,264 samples, of which 61,073 did not have a valid province populated (%0.35%) and were excluded. Overall, VS was reported for 67.7%, 70.3% and 70.0% of samples for the 2019, 2020 and 2021 calendar years respectively. In 2019, VS ranged from 58.6% (Limpopo) to 76.3% (KwaZulu‐Natal). The provincial percentage change in VS between 2019 and 2020 ranged from −1.3% (Northern Cape) to 8.6% (Eastern Cape). Similarly, between 2019 and 2021, the provincial percentage change in VS ranged from −6.0% (North West) to 1.5% (Western Cape). Between 2019 and 2021, the Limpopo province reported a percentage change decrease in VS of 5.8%.

**Conclusions**: Our findings indicate that Covid‐19 has not had a substantial impact on the percentage of samples with virological suppression when compared with 2019 at the national level. However, at the provincial level decreases in VS have been shown particularly for the 2021 year for the Limpopo and North West provinces. Further analysis is required to understand why VS decreased in these two provinces.

### PESUC20 Projection of age of people living with HIV and time since ART initiation in high‐income countries in 2030: estimates for France

D. Costagliola ^1^, M. Lise^1^, Y. Diawara^1^, A. Rachas^2^, S. Grabar^3^, V. Supervie^1^

^1^Sorbonne Université, INSERM, Institut Pierre Louis d'Epidémiologie et de Santé Publique, Paris, France, ^2^Direction de la Stratégie, des Etudes et des Statistiques, CNAM, Paris, France, ^3^Sorbonne Université, INSERM, Institut Pierre Louis d'Epidémiologie et de Santé Publique, AP‐HP, Hôpital St Antoine, Paris, France

**Background**: Thanks to antiretroviral treatment (ART), HIV‐infected individuals are aging. This involves increased co‐morbidity risks, which also depend on when individuals started ART. These upcoming challenges require knowledge of the future demographic profile of HIV population. Here, we forecast the demographic profile of the adult population diagnosed with HIV (aPDHIV) in France up to 2030, accounting for the impact of ART initiation period on mortality.

**Methods**: Using national data from the French Hospital Database on HIV (ANRS CO4‐FHDH) and a sample of the National Health Data System, we characterized the aPDHIV in 2018 and estimated their mortality rates according to age, sex, and ART initiation period. Using national surveillance data, we defined three scenarios for the numbers of newly‐diagnosed HIV cases over 2019–2030: 30% decrease (S1), status quo situation (S2), and epidemic elimination (S3). Combining these data, we projected the age structure of the aPDHIV and time since ART initiation.

**Results**: In 2018, there was an estimated 161,125 aPDHIV (67% men), of which 55% were aged 50 or older (50+), 22% 60+ and 8% 70+. In 2030, the aPDHIV would be 192,181 under S1, 204,813 under S2 and 164,224 under S3. Whatever the scenario, the age distribution would shift towards older ages: with in 2030, 64 to 71% aPDHIV aged 50+, 41–47% 60+ and 16–18% 70+. This corresponds to ∼80,800 aPDHIV (72% men) aged 60+, among which ∼69% started ART ≥20 years ago and ∼39% ≥30 years ago, and to ∼31,500 aPDHIV (72% men) aged 70+, among which ∼72% started ART ≥20 and ∼43% ≥30 years ago (Figure).

**Figure:**
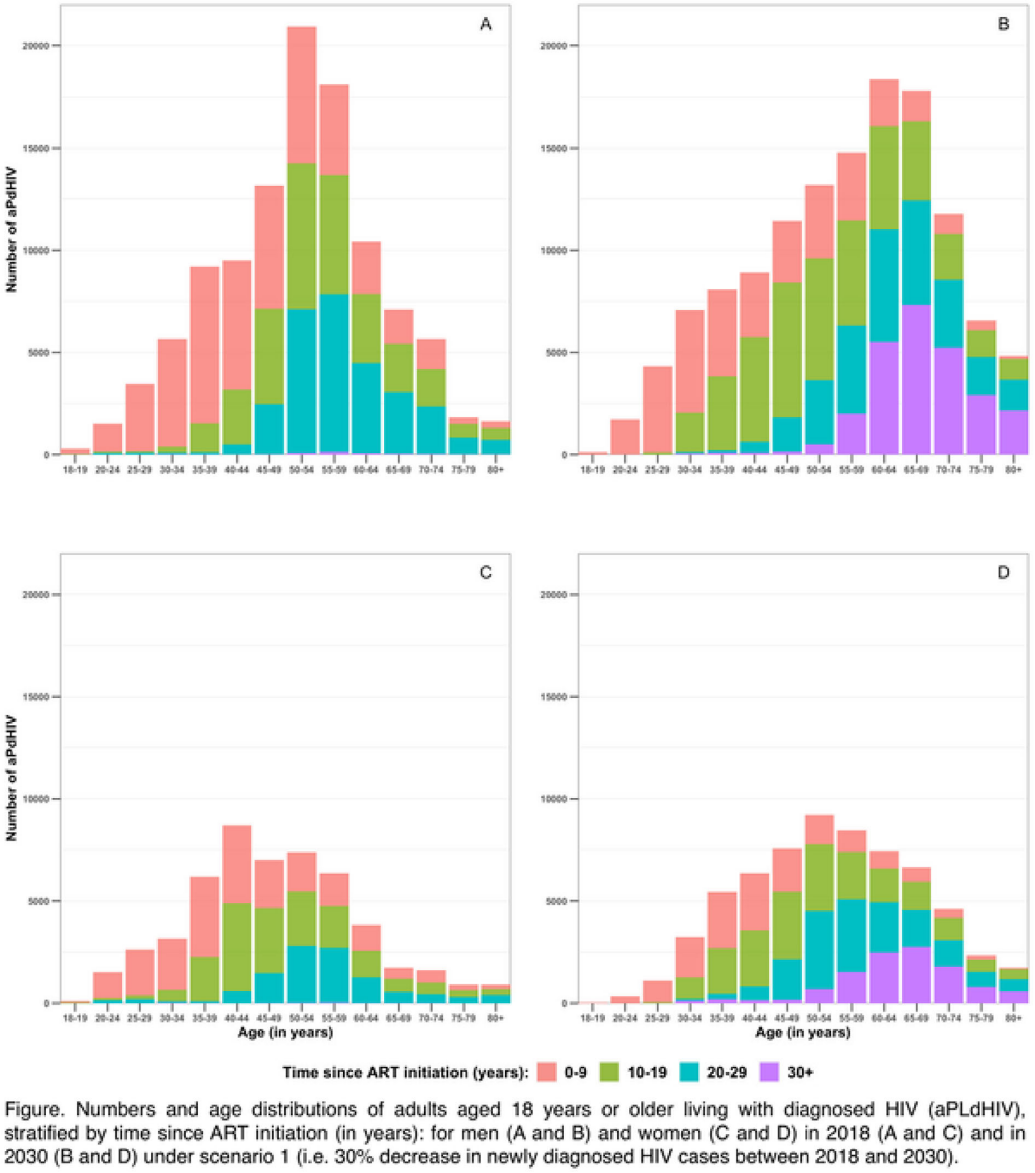
**Abstract PESUC20‐Figure 1**.

**Conclusions**: By 2030, in France, close to 20% of theaPDHIV will be aged 70+, of which >40% would have spent ≥30 years on ART. These estimates are essential to anticipate co‐morbidity screening and resource provision in the aged care sector.

### PESUC21 Life years gained with improved HIV care among non‐Hispanic Black and White people who have ever injected drugs (PWID) with HIV: a modeling study

K. Rich ^1^, F. Shebl^1,2^, A. Pandya^3^, J. Chiosi^2^, A. Ciaranello^1,2^, E. Losina^1,4,5^, K. Freedberg^1,2,3^, E. Hyle^1,2^

^1^Harvard Medical School, Boston, United States, ^2^Massachusetts General Hospital, Boston, United States, ^3^Harvard T.H. Chan School of Public Health, Boston, United States, ^4^Brigham and Women's Hospital, Boston, United States, ^5^Boston University School of Public Health, Boston, United States

**Background**: HIV remains a major health burden among people who have ever injected drugs (PWID). We projected life expectancy among non‐Hispanic Black and White PWID with HIV (PWID‐HIV) and assessed the impact of improving the HIV care cascade on life expectancy.

**Methods**: We applied race‐ and sex‐stratified data for US PWID to the validated CEPAC microsimulation model (Figure 1A). Race‐ and sex‐specific life tables for non‐HIV‐related mortality were adjusted for excess all‐cause mortality among PWID. We projected life expectancy from age 15 years among PWID‐HIV under four scenarios:

1. status quo HIV care,
2. annual HIV testing,
3. 95% retention in HIV care, and
4. annual testing and 95% retention in care.

We also compared life expectancy of PWID‐HIV to PWID without HIV.

**Results**: Among PWID‐HIV who receive *status quo* care, we projected life expectancy from age 15 to be highest among White females (56.6y) and lowest among Black males (50.7y) (Figure 1B‐E). With annual HIV testing, years of life gained (YLG) were higher among White PWID (females: 0.3y, males: 0.3y) than Black PWID (females: 0.2y, males: 0.2y). Increased retention in care (to 95%) would also result in additional YLG for White PWID (females: 1.0y, males: 0.6y) than Black PWID (females: 0.3y, males: 0.4y) compared with annual testing. When combining annual testing and 95% retention, YLG ranged from 0.2y to 0.3y. Comparing PWID‐HIV receiving *status quo* care to PWID without HIV, White PWID had a greater difference in LE (female: 2.2y, male: 1.7y) than Black PWID (female: 1.0y, male: 1.1y).

**Figure:**
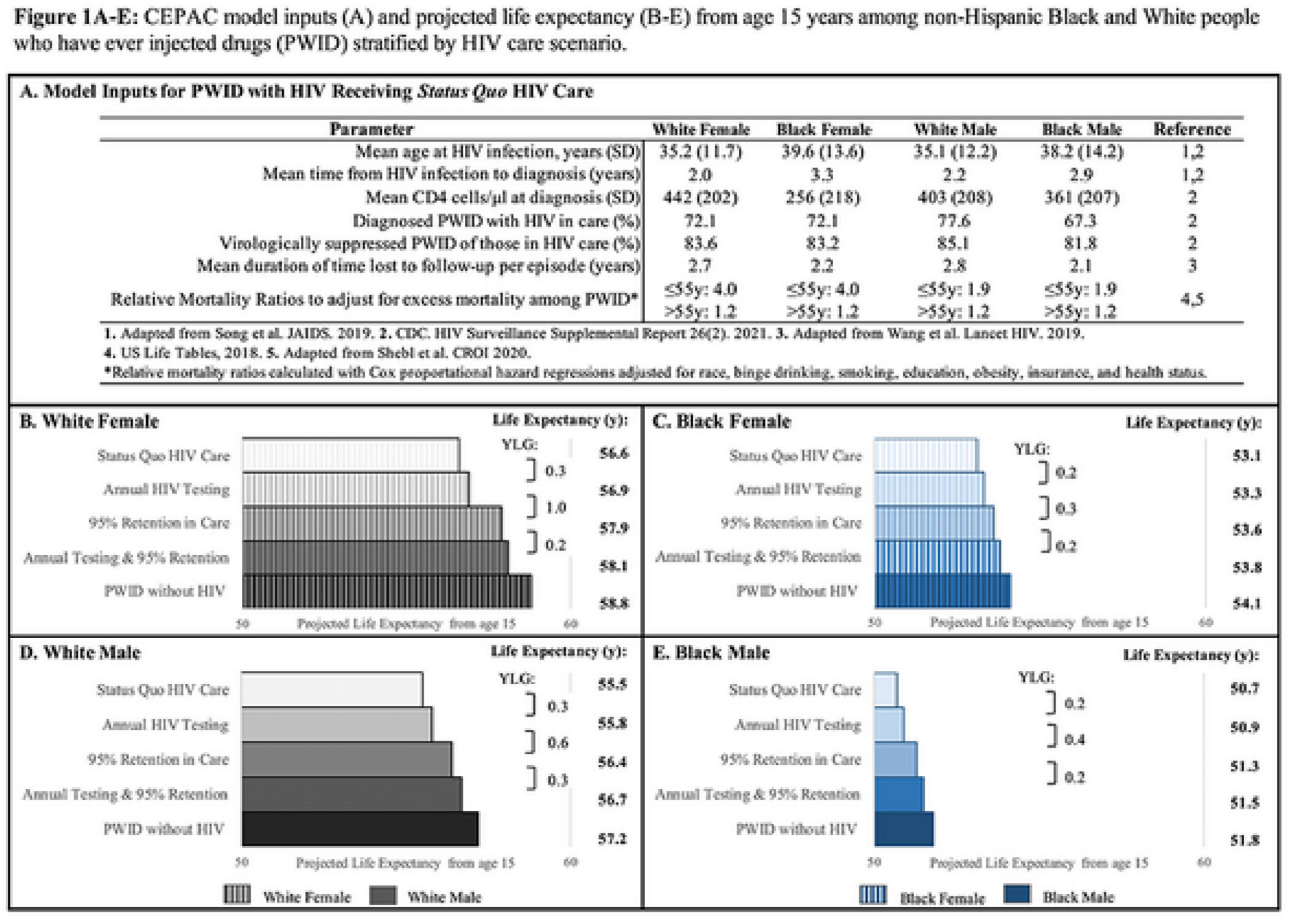
**Abstract PESUC21‐Figure 1**.

**Conclusions**: Among most PWID‐HIV, improving retention in care would gain more life‐years than increased testing, yet disparities in life expectancy between Black and White PWID‐HIV would persist. Attaining equivalent HIV testing and retention rates across Black and White PWID would be insufficient to address disparities in life expectancy.

### PESUC22 Is Thailand on track to achieve 95‐95‐95 targets by 2025?

S. Aungkulanon ^1^, M. Vasantiuppapokakorn^2^, S. Northbrook^1^, R. Triamwichanon^3^, T. Naiwatanakul^1^, A. Kanphukiew^1^, W. Kiatchanon^1^, B. Baipluthong^1^, C. Lertpiriyasuwat^2^

^1^U.S. Centers for Disease Control and Prevention, Division of Global HIV and TB, Nonthaburi, Thailand, ^2^Thailand Ministry of Public Health, Division of AIDS and STIs, Department of Disease Control, Nonthaburi, Thailand, ^3^National Health Security Office, Bangkok, Thailand

**Background**: Thailand made progress in expanding coverage of antiretroviral treatment (ART) for people living with HIV (PLHIV). We developed a model to predict national HIV cascade trends and estimate effort needed to achieve 95‐95‐95 targets in 2025.

**Methods**: We developed a Markov model on disease progression to project national HIV cascade trends from 2022 to 2025 using current epidemic (Model A) and accelerated response trends (Model B). The cycle of the Markov model was 1 year. We used 2021 National AIDS Program (NAP) data to classify PLHIV into six states:

1. diagnosed but not initiated ART,
2. initiated ART but not on ART,
3. on ART but not tested for VL,
4. tested for VL but not suppressed (VL > 1,000),
5. virally suppressed, and
6. death.

We calculated the probability of moving from one state to another or remaining in the same state by analyzing patient data from 23 health facilities. We applied a linear trend using NAP data from 2014 to 2021 to estimate the number of new and undiagnosed HIV‐infections from 2022 to 2025 and added them to the model. While we used the probability matrix from 2021 to develop Model A, we applied the exponential growth of the annual transition probability from current state to state 3 and state 5 by 1% to 10% to develop Model B.

**Results**: Of 503,459 PLHIV who knew their HIV status in 2021, 81% (405,634) were on ART and, of these, 81% (327,577) were virally suppressed. Under Model A, we projected ART coverage and VLS to increase to 86% and 84% in 2025, respectively. Under Model B, we achieved 2^nd^ and 3^rd^95 targets after intensifying treatment and viral suppression efforts by 5% each year.

**Table:** 

**Abstract PESUC22‐Table 1**.							
	Model B: Increase 5% annually					Model A	
Year	PLHIV Alive Diagnosed	On ART	Virally Suppressed	Second 95	Third 95	Second 95	Third 95
2022	509,263	431,472	367,259	85%	84%	83%	83%
2023	514,395	457,112	406,412	89%	89%	85%	83%
2024	518,395	478,936	443,798	92%	93%	86%	84%
2025	521,086	492,929	466,684	95%	95%	86%	84%

**Conclusions**: Current efforts will increase ART coverage and viral suppression but are insufficient to achieve 95‐95‐95 targets. Defining which populations, geographic areas, and interventions to intensify efforts are needed to optimize resource allocation and control the HIV epidemic.

### PESUC23 Characterising the HIV Care Cascade in Saskatoon, Saskatchewan, 2018 ‐ 2021

C. Spence ^1,2^, S. Sanche^1,3^, B. Wudel^1,3^, L. Kiesman^4^, S. Takaya^1,3^, M. Klein^2^, S. Kogilwaimath^1,3^

^1^University of Saskatchewan, Medicine, Saskatoon, Canada, ^2^McGill University, Medicine, Montreal, Canada, ^3^Saskatchewan Health Authority, Infectious Disease, Saskatoon, Canada, ^4^Westside Community Clinic, Saskatoon, Canada

**Background**: Saskatchewan has experienced a unique HIV epidemic in Canada, driven largely by injection drug use and disproportionately affecting younger women through heterosexual transmission. HIV care in Saskatoon is primarily accessed at two clinical sites: the Royal University Hospital (RUH) and the Westside Community Clinic (WSCC). While the clinic at RUH is a specialized Infectious Diseases clinic, WSCC provides community‐based access to primary care and addictions support. These clinics serve over 1200 active patients living with HIV. Offering different, yet complementary clinical care, the HIV care cascades of these two sites offer insights into the HIV epidemic over time, specifically, the characteristics of each of the care models; patient populations; intersectional considerations; and the impact of the COVID‐19 pandemic on patient outcomes.

**Description**: With demographic and clinical data for diagnosed Persons With HIV (PWH) across the two clinic sites over a four‐year period, from 2018 – 2021, care continuum data is compared across the two sites during the four‐year time periods, characterizing the HIV care cascade for Saskatoon, SK. The data demonstrates where the gaps in care exist between the two models.

**Lessons learned**: The community‐based care model has seen a progressive advancement in cascade outcomes, while being relatively undisrupted during the COVID‐19 period, with a consistent 79% PWH engaged in care. However, the pandemic period adversely impacted cascade outcomes for the hospital‐based clinic, reflected by a drop from 67% to 56% of patients on ART, and an overall trend of moderate cascade outcomes over the four‐year period of analysis. The rates of virologic suppression are noted to be lowest during the peak period of the pandemic lockdown in May 2020 for both clinic sites, (65% and 46% respectively) indicating a clear impact of the pandemic lockdown mandate. A marked shift in patient demographics over the analysis period includes an increase of younger females with new infections.

**Conclusions/Next steps**: Gaps in the care continuum offer insights to advocate for adaptation of the community‐based delivery model to develop targeted solutions to expand outreach, supporting the petition for more resources for access and engagement in care for a large cohort of PWH seeking care in Saskatoon.

### PESUC24 Willingness to use PrEP among gay, bisexual, and other men who have sex with men in five Asian countries: Results of the Asia Pacific MSM Internet Survey

B. Bavinton ^1^, A. Hill^2^, N. Amos^2^, S.H. Lim^3^, T. Guadamuz^4^, N. Kaneko^5^, M. Holt^6^, A. Bourne^2^

^1^University of New South Wales, Kirby Institute, Sydney, Australia, ^2^La Trobe University, Australian Research Centre in Sex, Health and Society, Melbourne, Australia, ^3^University of Malaya, Kuala Lumpur, Malaysia, ^4^Mahidol University, Bangkok, Thailand, ^5^Nagoya City University, Nagoya, Japan, ^6^University of New South Wales, Centre for Social Research in Health, Sydney, Australia

**Background**: PrEP is highly effective at preventing HIV infection among gay, bisexual, and other men who have sex with men (GBM). However, across Asia, PrEP programs are limited, and little is known about men's willingness to use PrEP.

**Methods**: A cross‐national online survey targeting GBM in Indonesia, Japan, Malaysia, Thailand, and Vietnam was conducted from May 2020–January 2021. Factors independently associated with willingness to use PrEP among non‐PrEP‐users were determined by multivariable logistic regression.

**Results**: We recruited 10,339 HIV‐negative/untested GBM who reported ≥1 male sexual partners in the previous year (Japan = 5,660; Vietnam = 2,257; Thailand = 1,076; Indonesia = 803; Malaysia = 543). Overall, 7.4% were currently using PrEP (Thailand = 14.0%; Vietnam = 11.1%; Malaysia = 7.4%; Japan = 5.4%; Indonesia = 2.0%). After restricting to the 9,578 non‐PrEP‐users, 54.4% were willing to use PrEP (Malaysia = 73.6%; Thailand = 65.2%; Indonesia = 62.6%; Vietnam = 51.2%; Japan = 50.6%). Among these non‐users, 2.0% (n = 194) had previously used it (range = 0.8% in Indonesia/Japan–5.4% in Thailand). Factors independently associated with PrEP willingness among non‐users were: younger age (adjusted odds ratio [AOR] = 0.98, 95% confidence interval [CI] = 0.97–0.98, p < 0.001); higher levels of education (diploma: AOR = 1.54, 95%CI = 1.18–2.01, p = 0.001; university: AOR = 1.34, 95%CI = 1.04–1.72, p = 0.022); having more than 10 sexual partners in the previous 12 months (AOR = 1.21, 95%CI = 1.01–1.44, p = 0.041); awareness of the “undetectable = untransmittable (U = U)” message (AOR = 1.23, 95%CI = 1.12–1.36, p < 0.001); condomless anal intercourse with regular (AOR = 1.16, 95%CI = 1.05–1.28, p = 0.003) and casual (AOR = 1.29, 1.16–1.43, p < 0.001) male partners in the previous 12 months; use of Viagra (AOR = 1.33, 95%CI = 1.17–1.50, p < 0.001); being a former PrEP‐user (AOR = 1.93, 95%CI = 1.34–2.77, p < 0.001); and being willing to pay for PrEP (AOR = 4.61, 95%CI = 4.09–5.20, p < 0.001). Of those willing to use PrEP, only 36.1% were willing to pay for it (versus 10.8% of those not willing to use it).

**Conclusions**: PrEP use was very low overall. However, more than half of non‐PrEP‐users were willing to use it, indicating that there is a large pool of potential users; these men were younger and at higher risk (having more sexual partners, reporting condomless intercourse). In some countries, the gap between willingness and use can be explained by the lack of PrEP programs, but other factors may be important. Affordability of PrEP is critical, as most men – even many willing to use it – were not willing to pay for it out‐of‐pocket.

### PESUC25 Recovery in the uptake of pre‐exposure prophylaxis (PrEP) in Australia after COVID‐19 restrictions: analysis of national pharmacy dispensing data

D. Fraser^1^, H. McManus^1^, N. Medland^1^, A. Grulich ^1^, R. Guy^1^, B. Bavinton^1^

^1^Kirby Institute, UNSW, Sydney, Australia

**Background**: Initial COVID‐19 restrictions in 2020 were accompanied by reductions in HIV pre‐exposure prophylaxis (PrEP) prescribing in Australia. We analysed the rate of PrEP uptake nationally before and after COVID‐19 restrictions to examine whether PrEP use returned to pre‐COVID levels.

**Figure:**
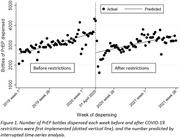
**Abstract PESUC25‐Figure 1**.

**Methods**: Data were extracted from all publicly‐subsidised PrEP prescriptions dispensed in Australia between 01 January 2019 and 30 June 2021. We used interrupted time series analyses of time trends in the weekly number of bottles (30 pills) of PrEP dispensed nationally and by state/territory of residence, age group, and socioeconomic status of residential postcode. We also report total dispensing in March 2020, the month immediately before COVID‐19 restrictions were implemented with June 2021, the final month of follow‐up.

**Results**: Over the 30‐month period, 45,147 people (98.4% male) were dispensed 395,707 bottles of PrEP. Before restrictions, PrEP dispensing increased by 17 bottles each week (p < 0.001; 95%CI: 13.0, 21.6). In the week following implementation of restrictions, 1,023 fewer bottles were dispensed than the previous week (p < 0.001; 95%CI: −1,339.8, −707.4). Dispensing then increased at an average of 13.1 bottles per week (p < 0.001, 95%CI: 6.9, 19.3). The rate of increase was not significantly different to the pre‐restrictions rate (p = 0.272; 95%CI:‐11.8, 3.3). By the end of follow‐up there were 15,330 bottles dispensed in June 2021, approaching the 16,635 bottles dispensed in March 2020 (immediately before COVID‐19 restrictions were implemented). There were no significant differences between the rate of post‐restriction dispensing between subpopulations of state/territory, age groups or socioeconomic status.

**Conclusions**: PrEP use steadily increased in Australia before COVID‐19 restrictions led to a sudden large decrease in dispensing. By June 2021, nationwide PrEP use recovered to pre‐pandemic levels. There were no disparities by jurisdiction, age, or socioeconomic status in the rate of recovery. This suggests that, as restrictions were lifted and sex increased, PrEP uptake also recovered.

### PESUC26 Fostering access to PrEP among high‐risk adolescent girls and young women aged 16 to 24 years through the DREAMS initiative in 4 districts in Zambia

J. Chipukuma ^1^, P. Olowski^1^, B. Lindsay^1,2^, L. Mwango^3^, K. Tembo^1^, C. Bwale^3^, P. Makasa^3^, M. Muchoka^3^, S. Tembo^3^, W. Mbokile^3^, B. Bwembelo^3^, E. Fundulu^3^, C. Munsongo^4^, K. Watala^4^, B. Musonda^4^, J. Okuku^5^, A. Mwila^5^, C. Muleya^5^, C.W. Claassen^1,2^

^1^Maryland Global Initiatives Corporation Zambia, Lusaka, Zambia, ^2^Center for International Health, Education, and Biosecurity, University of Maryland School of Medicine, Baltimore, United States, ^3^Ciheb Zambia, Lusaka, Zambia, ^4^Ministry of Health, Zambia, Lusaka, Zambia, ^5^U.S. Center for Disease Control and Prevention, Lusaka, Lusaka, Zambia

**Background**: In Zambia, adolescent girls and young women (AGYW) remain at high risk of HIV infection due to social, cultural, and economic vulnerabilities. Pre‐exposure prophylaxis (PrEP) is effective at preventing HIV especially when used as part of a combination prevention approach. However, limited data exists on PrEP eligibility, uptake and persistence among AGYW in sub‐Saharan Africa. The University of Maryland Baltimore CIRKUITS and Z‐CHECK projects implemented the Determined Resilient Empowered AIDS‐free Mentored Safe (DREAMS) initiative aimed at educating young girls, communities and families on HIV prevention using evidence‐based interventions, as a holistic HIV prevention intervention, including PrEP provision to AGYW.

**Methods**: We examined PrEP uptake among AGYW aged 16 to 24 years using a retrospective cohort enrolled in DREAMS between October 2020 and September 2021 in four districts of Zambia. AGYW were screened for substantial HIV risk; those eligible were consented and voluntarily initiated on PrEP at DREAMS centers by health care workers. Follow ups were conducted for refills and laboratory investigations. Multivariable logistic regression was used to examine variables associated with obtaining at least one PrEP refill following initiation.

**Results**: A total of 1,734 AGYW were assessed for PrEP eligibility, of whom 1,733 (99.9%) were eligible and 1,716 (99.0%) started PrEP. The median age at PrEP initiation was 21 years (IQR: 19, 23). Of those who started, 938 (54.7%) had at least one PrEP refill. Factors associated with obtaining at least one refill included age 20–24 years (aOR 1.88, 95% CI 1.51–2.34), being married (aOR 1.80, 95% CI 1.37–2.36) or sexually active in last six months (aOR 2.82, 95% CI 2.29–3.48), and reporting an STI in the prior six months (aOR 10.13, 95% CI 3.08–33.34). Among AGYW who discontinued PrEP and had at least one follow‐up visit (99, 10.0%), reasons for discontinuation included: no longer at risk (56, 57%), relocation (12, 12%), and side effects (7, 7%).

**Conclusions**: The DREAMS initiative was successful at reaching AGYW with PrEP services. A high proportion at risk of HIV elected to initiate PrEP for at least some time. More evidence is needed to assess reasons for discontinuation and improve persistence for those with sustained risk.

### PESUC27 Transforming Social and Behaviour Communication (SBC) using digital platforms: the case of USSD HIV self‐assessment and PrEP adherence support tools

R.M. Muleya ^1^

^1^JSI‐ USAID DISCOVER ‐ Health, Prevention and Behavioral Interventions, Lusaka, Zambia

**Background**: In 2018 in Zambia, the USAID DISCOVER‐Health Project, implemented by JSI, was one of the first partners to roll out PrEP. In Zambia, PrEP is only provided to individuals at substantial risk of HIV, using a paper‐based national eligibility screening tool, administered by healthcare workers (HCWs) during service delivery. This is sometimes challenging to do in busy clinics, from both time‐constraints and privacy perspectives.

**Description**: To help address this, the Project developed an Unstructured Supplementary Service Data (USSD) information management system, available to the public, to support self‐risk assessment and improve linkage to services. Using mobile phones, clients access the HIV self‐risk assessment platform to ascertain their risk level using the USSD. If an individual is at substantial risk, the USSD links them geographically to their nearest PrEP service provider (SP). TheSP reaches out to the client and makes an in‐person appointment to initiate them on PrEP. Once the client is initiated, the USSD, in combination with Short Message Service (SMS), provides PrEP adherence support, through periodic reminders.

**Lessons learned**: The Project analysed PrEP USSD/SMS access log to determine the volume of access for this service. 123,388 clients accessed this service between October 2018 and June 2019. Out of the 123,388 clients that accessed the USSD platform and used the HIV self‐risk assessment, 16,430 were found to be at risk and requested the system to link them to the next step of in‐person PrEP services. In addition, 916 clients also requested to be linked to PrEP clinics, before full completion of the HIV risk assessment. Through the USSD, 17,346 people were linked to PrEP clinics from January to December 2021. Duringthe same period, 33,776 clients accessed PrEP Adherence support using the USSD.

**Conclusions/Next steps**: The Project has demonstrated that simple digital solutions, such as USSD platforms, can facilitate health service linkage and adherence support for clients. The USSD platform provides privacy through self‐risk assessment and reduces the initial screening burden on busy HCWs, while significantly opening up access to essential HIV prevention services to a wider beneficiary universe.

### PESUC28 Primary care providers’ perspectives regarding PrEP care at primary care settings: a qualitative analysis

C. Zhang ^1^, S. Przybylek^1^, Y. LiU^2^

^1^University of Rochester, School of Nursing, Rochester, United States, ^2^University of Rochester, Public Health Science, Rochester, United States

**Background**: Primary care providers (PCPs) are crucial for preventing HIV and promoting sexual wellbeing for their patients. PCPs are considered the ideal pre‐exposure prophylaxis (PrEP) care providers as they usually encounter more HIV‐negative patients with indications for PrEP use. Nevertheless, limited data are available to assess PCP's perspectives regarding PrEP care. This current study will explore their perceived barriers and potential facilitators on providing PrEP care at primary care settings in a cohort of PCPs recruited from a northeastern state in the United States.

**Methods**: In the current study, we employed a semi‐structured in‐depth interview to collect information about barriers and facilitators in PrEP care implementation among 18 PCPs who practice in New York State. We coded texts using coding themes related to the central questions in our interview guides and new themes that emerged in the coding process. Mixed methods including content and grounded theory analyses were used to analyze the transcribed narrative data.

**Results**: The recruited PCPs aged 30–68 years old reported a few specialties, including internal medicine, family medicine, and adolescent health. We identified a few themes related to their perspectives on PrEP care in primary care settings. Perceived barriers included: reluctance for discussion on sexual health‐related topics, lack of navigations for patients and providers, the complexity of the regimen for engaging and monitoring patients, patients' low perceived risks, and HIV and PrEP associated stigma. In addition to barriers, a few potential solutions have been proposed by PCPs, such as screening for PrEP eligibility before the doctor visit, increasing self‐awareness of HIV risk among patients, the flexible regime being available (e.g., long‐term injectable PrEP), providing comprehensive PrEP navigation system to both providers and patients, and providing PCPs with peer education and sufficient training for PrEP care.

**Conclusions**: Our study is one of the first to explore PrEP care implementation in primary care settings. Findings suggested a navigation system for PrEP care in patients and providers is urgently needed. Future studies should facilitate PrEP discussion, engagement, and monitoring in primary care settings.

### PESUC29 PrEP use and HIV incidence among young Thai men and transgender women who sell sex in Bangkok and Pattaya, Thailand: results of the COPE effectiveness study

C. Beyrer ^1^, B.W. Weir^1,2^, A.L. Wirtz^1^, S.H.H. Mon^1^, N. Qaragholi^1^, T. Chemnasiri^3,4^, A. Warapornmongkholkul^3,4^, D. Linjongrat^5^, S. Janyam^6^, P. Sirivongrangson^7^, B. Chua‐Intra^7^, A.C. Hickey^3,4^

 

**Figure:**
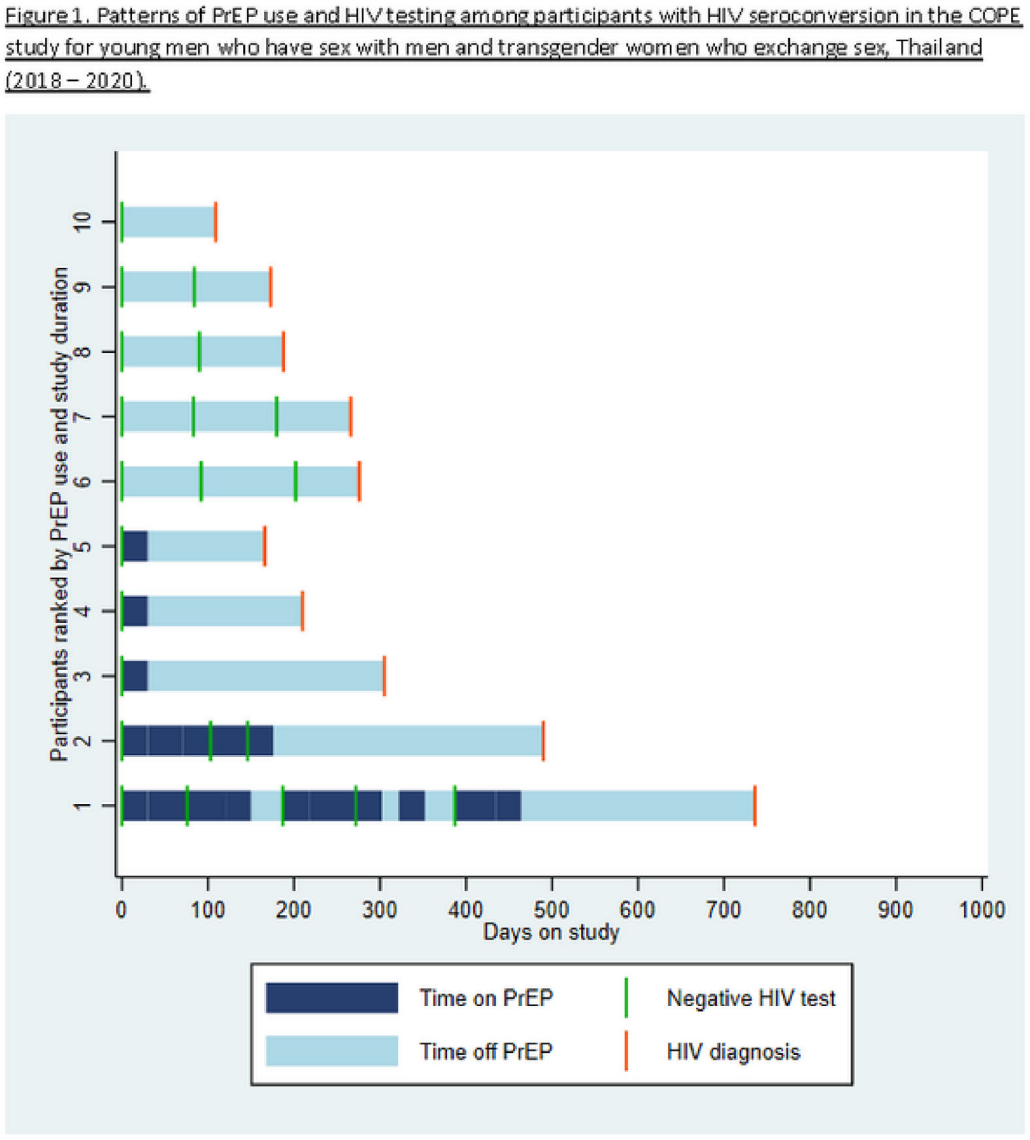
**Abstract PESUC29‐Figure 1**.

^1^Johns Hopkins Bloomberg School of Public Health, Center for Public Health and Human Rights, Department of Epidemiology, Baltimore, United States, ^2^Johns Hopkins Bloomberg School of Public Health, Department of Health, Behavior, and Society, Baltimore, United States, ^3^U.S. Centers for Disease Control and Prevention, Division of HIV/AIDS Prevention, Atlanta, United States, ^4^Thailand Ministry of Public Health‐U.S. Centers for Disease Control and Prevention Collaboration (TUC), Nonthaburi, Thailand, ^5^Rainbow Sky Association of Thailand (RSAT), Bangkok, Thailand, ^6^Service Workers in Group Foundation (SWING), Bangkok & Pattaya, Thailand, ^7^Thailand Ministry of Public Health, Department of Disease Control, Nonthaburi, Thailand

**Background**: Young men who have sex with men (MSM) and transgender women (TGW) who exchange sex in Thailand have high HIV incidence. Access to PrEP for combination HIV prevention is imperative.

**Methods**: The COPE study recruited HIV‐negative Thai MSM and TGW aged 18–26 years, who exchanged sex in the last year through online and community outreach. Participants were offered oral PrEP and could start and stop PrEP as desired. Participants were followed for 1 year, with some extending up to 2 years, and completed quarterly assessments, weekly SMS surveys and HIV and STI testing. The primary outcome was HIV seroconversions per 100 person‐years (PY) on PrEP and not on PrEP, based on monthly pill pickups.

**Results**: Among 846 participants at 4 community clinics (October 2017‐August 2019), 531 (62.8%) initiated PrEP at baseline, 104 (12.3%) subsequently initiated PrEP, and 211 (24.9%) never initiated PrEP. Participants contributed 598.4 PY on PrEP and 335.5 PY not on PrEP. Sufficient adherence was self‐reported during 96.2% of SMS surveys while on PrEP. In a sub‐sample of 66 dried blood spots with high self‐reported adherence (≥4 pills per week), 53 (80%) had protective levels of intracellular tenofovir diphosphate. Of the 10 participants diagnosed with HIV during study participation, none were using PrEP at the time of diagnosis (Figure 1), corresponding to an on‐PrEP incidence rate of 0.0 per 100 PY (95% CI = 0.0, 0.62), an off‐PrEP incidence rate of 2.98 per 100 PY (95% CI = 1.43, 5.48), and an IRR of 0.0 (95% CI = 0.0, 0.25; p <.0001). Sensitivity analyses accounting for uncertainty in time of seroconversion provided an IRR of 0.041 (95% CI = .006, .311; p = .001).

**Conclusions**: Combination HIV prevention with PrEP for young Thai MSM and TGW who exchange sex can achieve high PrEP uptake, high adherence, and reduced HIV incidence.

### PESUC30 Preferences for potential long‐acting pre‐exposure prophylaxis (PrEP) dosing regimens among gay, bisexual and other men who have sex with men (GBMSM) in Taiwan: 2021 HEART Survey

S.W.‐W. Ku ^1,2^, T.‐W. Chen^3^, C.‐W. Li^4^, P. Huang^5^, H.‐J. Wu^6^, T.‐H. Wu^2^, A. Bourne^6,7^, C. Strong^3^

 

**Figure:**
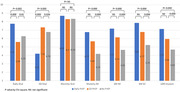
**Abstract PESUC30‐Figure 1**.

^1^Taipei City Hospital Renai Branch, Division of Medicine, Department of Infectious Diseases, Taipei City, Taiwan, Province of China, ^2^HIV Education And Research Taiwan (HEART) Association, Taipei City, Taiwan, Province of China, ^3^National Cheng Kung University, College of Medicine, Department of Public Health, Tainan City, Taiwan, Province of China, ^4^National Cheng Kung University, College of Medicine, National Cheng Kung University Hospital, Center for Infection Control and Department of Internal Medicine, Tainan City, Taiwan, Province of China, ^5^National Taiwan University, Institute of Health Behavior and Community Sciences, Taipei City, Taiwan, Province of China, ^6^Kirby Institute, University of New South Wales, Sydney, Australia, ^7^Australian Research Centre in Sex, Health and Society, La Trobe University, Melbourne, Australia

**Background**: Various antiretrovirals have been examined as potential candidates for long‐acting PrEP. While event‐driven (ED) PrEP is currently the most prevalent dosing regimen among GBMSM in Taiwan, there is a need to determine preferences for other possible regimes and what factors might inform such preferences, in order to support future PrEP uptake.

**Methods**: Between November 28^th^‐December 28^th^ 2021, a survey comprising 67 questions was administered online to GBMSM using social networking applications in Taiwan. Beyond demographics, HIV serostatus and HIV risk behaviors, respondents were asked to indicate their preference for different PrEP formulations on a 10‐point Likert scale (1 = least preferred; 10 = most preferred). Options included: daily oral, ED oral, monthly oral, monthly or bimonthly intramuscular injectable (2M IM), 6‐month subcutaneous injectable (6M SC), and 12‐month implant.

**Results**: In total, 1,880 survey responses were included in this analysis. Among 1,728 respondents who reported HIV‐negative or unknown serostatus, 52 took PrEP daily and 198 event‐driven. Regardless of cost, current daily PrEP users were most likely to prefer monthly oral, followed by 6M SC, daily oral, and 2M IM. Current ED PrEP users preferred monthly oral form the most, followed by ED oral, 6M SC, and 2M IM. Overall, the majority (67.7%) were only willing to pay less than 150 USD per injection to receive 2M IM PrEP. Multivariable logistic regression revealed current daily PrEP users (AOR 2.19 vs. ED user), those willing to take PrEP (AOR 1.45), those with more sex partners (AOR 1.39), and those not feeling happy about their sex life (AOR 1.60) had significant correlation with preference of 2M IM over ED oral PrEP.

**Conclusions**: Monthly oral was the most preferred dosing regimen. Bimonthly intramuscular injectable PrEP could be an alternative to ED PrEP for certain populations. Cost and means of administration could affect new regimen uptake and should be further investigated.

### PESUC31 Contact Tracing an Innovative Approach to Fight HIV Epidemic in Haiti

G.E. Thermidor ^1^, J. Ukwishaka^2^, J.F. Gakindi^3^, J. Deas Van ONACKER^4^, N. Duval^4^, E.G. Robin^4^

^1^Universite Libre de Bruxelles, Public Health School, Bruxelles, Belgium, ^2^Universite Libre de Bruxelles, School of Public Health, Bruxelles, Belgium, ^3^Univesite Libre de Bruxelles, School of Public Health, Bruxelles, Belgium, ^4^MSPP/PNLS, Port‐au‐Prince, Haiti

**Background**: Significant progress has been made in controlling the HIV epidemic in the past 20 years. In 2020, 84% of people living with HIV (PLHIV) knew their status in Worldwide. During the last decade, the Haitian Ministry Health (MSPP) through the National program against STIs and HIV/AIDS (PNLS) has concentrated on discovering creative techniques to expanding HIV testing in line with WHO, CDC and USAID strategies. One of implemented strategy was contact tracing which was adapted to the Haitian settings to be more effective. This study's objectives were to identify the percentage of PLHIV (People Living with HIV) who accepted to participate in contact notification services, to determine the prevalence of HIV in tested contacts and to determine the number and profile of new cases enrolled in HIV‐care and treatment services.

**Methods**: It was a retrospective cross‐sectional descriptive study conducted between July 2018 to July 2021. The data were collected from the MESI (monitoring evaluation surveillance integree) national database. The search included (149) health facilities involved in providing care and treatment to PLHIV in the whole country with available data on partner services platform viamesi.ht. The data were collected on the platform from Index patient and their contact information forms.

**Results**: Among the Index cases contacted, **94%** accepted the contact notification services. From July 2018 to July 2021, the contact tracing unveiled 52742 contacts. Among them (85.9%) 45324 were contacted. Among those contacted only (92.7%) were found. Among the interviewed people, 31806 persons were not aware of their HIV status. Among those who were not aware of their status (95.5%) 30393 were tested **and (33.7%)** 10252 of them were positive. **(94%)** 9636 of new cases were enrolled in the service of care and treatment. The analysis per index cases priority group revealed that 52% of MSM contacts and 43% of FSW contacts tested positive for HIV.

**Conclusions**: Contact tracing approach adapted to local settings is an effective targeted method of identifying and enrolling to treatment many patients. It helps finding the high number of contacts including the key population.

### PESUC32 Potential use of pooled point‐of‐care HIV‐1 viral load to detect HIV infection at PrEP initiation and follow‐up visits in key population‐led PrEP clinics in Thailand

N. Thammajaruk ^1^, S. Thongsuksangcharoen^1^, R. Trichavaroj^1^, P. Thapwong^2^, S. Saiwaew^3^, S. Sangtong^4^, M. Avery^5^, S. Mills^5^, P. Phanuphak^1^, R.A. Ramautarsing^1^, N. Phanuphak^1^

^1^Institute of HIV Research and Innovation (IHRI), Bangkok, Thailand, ^2^The Service Workers In Group Foundation, Bangkok, Thailand, ^3^Rainbow Sky Association of Thailand, Bangkok, Thailand, ^4^Mplus Foundation, Chiang Mai, Thailand, ^5^FHI 360, Bangkok, Thailand

**Background**: Use of HIV‐1 viral load (VL) assay can improve detection of HIV infection before pre‐exposure prophylaxis (PrEP) initiation and during follow‐up, as PrEP can delay detection by fourth‐ and third‐ generation immunoassays. We studied potential use of individual and pooled point‐of‐care VL assays to detect acute/early HIV infection in key population (KP)‐led PrEP clinics in Thailand.

**Methods**: From August 2019 to September 2021, men who have sex with men and transgender women PrEP clients were enrolled from four KP‐led clinics in Bangkok, Chiang Mai, and Pattaya. EDTA plasma was collected at entry and quarterly visits and tested for HIV VL (Xpert HIV‐1 VL) and conventional HIV rapid testing algorithm (Alere Determine HIV‐1/2, if reactive, then followed by Wantai Colloidal Gold Device and SD Bioline HIV‐1/2 3.0). Participants with detectable VL had their leftover plasma tested by Wantai and SD Bioline (regardless of Alere Determine result) and Alere HIV COMBO (fourth‐generation rapid test). Their samples and those from other PrEP clients who came on the same day were pooled for VL testing by Xpert.

**Table:** 

**Table 1**: Results of individual and pooled virological and immunological response to HIV‐1 infection among PrEP users								
Participants	HIV testing algorithm at KP‐led clinics						Pooled HIV‐1 VL	
PrEP users	Conventional HIV test result	A1: Alere Determine HIV‐1/2	A2: Wantai Colloidal Gold Device	A3: SD Bioline HIV‐1/2 3.0	Individual HIV‐1 VL result (copies/mL)	Alere HIV COMBO (4th generation rapid HIV test)	Number of samples per pool	HIV‐1 VL result (copies/mL)
PrEP initiation	negative	negative	negative	negative	> 10,000,000	positive (Ag band)	11	3,670,000
PrEP follow‐up	negative	negative	negative	positive	810	positive (Ab band)	12	43
PrEP follow‐up	negative	negative	positive	positive	5,940,000	positive (Ab band)	7	670,000

**Results**: Of 3,828 visits (390 PrEP initiation and 3,438 PrEP follow‐up), VL was detected in three participants; one at PrEP initiation (>10,000,000 copies/mL) and two at PrEP follow‐up visits (810 and 5,940,000 copies/mL). Pooled VL showed detectable VL at 3,670,000 (11 samples/pool), 43 (12 samples/pool), and 670,000 (7 samples/pool) copies/mL, respectively (see Table 1). Alere HIV COMBO showed antigen positivity for the participant with detectable VL at initiation, and antibody positivity for the follow‐up participants. All tested HIV negative by conventional algorithm.

**Conclusions**: Pooled point‐of‐care HIV VL testing and fourth‐generation rapid test detected HIV infections at PrEP initiation and follow‐up visits that were missed by conventional third‐generation rapid testing algorithm. Both assays have high potential and affordability to be integrated into KP‐led PrEP clinics in resource‐limited settings.

### PESUD25 Burnout among service providers for people living with HIV: Factors related to coping and resilience

S. Dale ^1^, R. Reid^1^, A. Madhu^1^, S. Gonzalez^1^, H. Crosby^1^, M. Stjuste^1^

^1^University of Miami, Miami, United States

**Background**: Individuals who provide services for people living with HIV (PLWH) face numerous challenges at work, including psychosocial and structural factors, which affect quality of care and service delivery. Little is known about the factors that relate to burnout among case managers/workers, peer counselors, group facilitators, social workers, and HIV testers who may share overlapping identities with their clients, which may increase their effectiveness in their roles, yet also exacerbate burnout. The current study seeks to examine the factors associated with burnout, as well as the role of resilience and coping among service providers for PLWH.

**Methods**: Data was collected from 28 service providers for PLWH (67.9% Black/African American, 53.6% HIV negative and 57.1% HIV testers) in the United States from January to October 2021 using a mixed methods research design. Participants completed quantitative measures on sociodemographics, organizational factors, discrimination, trauma, depression and burnout. A sub‐sample of 19 participants also provided in‐depth qualitative data via semi‐structured interviews, which explored the aforementioned factors as well as the impact of COVID‐19, coping, and resilience on burnout.

**Results**: Thematic content analysis revealed important themes on the factors related to burnout (e.g. discrimination, microaggressions, shared identities, limited financial and housing resources, and COVID‐19), rejuvenating factors (e.g. finding purpose in one's work), coping with burnout (e.g. psychotherapy and spirituality), and intervention strategies that are individually tailored. Results of Pearson correlations were consistent with the stories and insights shared by participants and revealed significant associations between mental health variables (i.e. depressive and posttraumatic stress disorder symptomology), discrimination and microaggressions, and burnout.

**Conclusions**: Our findings highlight structural barriers within organizations and discrimination that are impacting professionals in this sample who share identities (e.g. LGBTQ+, living with HIV, race) with the PLWH whom they serve. This research also highlights the relationship between discrimination and the mental health and well‐being of key professionals who serve PLWH. These findings may inform the development of an intervention targeting burnout among individuals providing services to PLWH as well as incite change to remove structural barriers that may improve the work environments for providers and ultimately the quality of care for PLWH.

### PESUD26 Using technology to increase STI/HIV case finding through pharmacies in Cambodia

J. Neukom ^1^, S. Suon^2^, J.M. Lailo^3^, E.G. Ramoran^4^, M.A. Tripon‐Manguiat^5^, O. Vichea^6^

^1^mClinica Pharmacy Solutions, Public Sector, Ho Chi Minh City, Viet Nam, ^2^mClinica Pharmacy Solutions, Public Sector, Phnom Penh, Cambodia, ^3^mClinica Pharmacy Solutions, Public Sector, Taguig City, Philippines, the, ^4^mClinica Pharmacy Solutions, Data, Taguig City, Philippines, the, ^5^mClinica Pharmacy Solutions, Technology, Taguig City, Philippines, the, ^6^National Center for HIV/AIDS, Dermatology and Sexually Transmitted Diseases, Phnom Penh, Cambodia

**Background**: Pharmacies serve as an initial source of health care for key populations at risk of HIV and sexually transmitted infections (STIs) in Cambodia and other low and middle income countries. Despite this, public health programs have struggled to engage pharmacies at scale given the fragmented nature of the pharmacy channel in Southeast Asia. In this context, the National Center for HIV/AIDS, Dermatology and Sexually Transmitted Diseases (NCHADS) collaborated with mClinica to leverage the SwipeRx network to facilitate HIV/STIs e‐referral practices in Cambodia.

**Description**: With support from the Global Fund and technical assistance from USAID EpiC, Linkages, and Enhancing Quality Health Activity (EQHA), NCHADS and mClinica tested an e‐Referral system. The system was built using an e‐Referral tool within the SwipeRx app with linkages to three referral facilities (1 private and 2 public) in Phnom Penh. Clients who consent to be referred through SwipeRx provide a mobile phone contact through which they receive automated SMS messages to encourage them to seek referral care. Pharmacies invited to join the program were identified based on their pre‐existing key population client volume, technology capacity, and willingness to participate in the program.

**Lessons learned**: Data from June‐December 2021 suggest that 252 clients were referred through SwipeRx by 39 pharmacies trained to use e‐Referral system. Among the clients referred, 93 were referred for STI/HIV diagnosis, the average number of STI/HIV clients referred through e‐Referral system increase by 130% based on comparison of 3‐month averages. Close to 3 out of 4 clients (73%) referred for STI/HIV services chose Chouk Sar (private, non‐governmental) referral site while 27% chose one of the two (public) referral sites accessible through the system. During the initial 6.5 months of e‐Referral, 17 STI cases and 4 HIV cases were diagnosed and treated.

**Conclusions/Next steps**: These findings highlight the potential for pharmacies linked to e‐Referral system to contribute to national STI/HIV program goals. Future efforts to expand e‐Referral system coverage to include additional pharmacies serving key populations and to include pharmacy access to HIV self‐testing are sustainable strategies to increase coverage of key populations and link with the full cascade of care available at referral health facilities.

### PESUD27 Integrated HIV Testing Service Delivery: An Innovative Approach to Reaching Female Head Porters and High Risk Men in Ghana amid COVID‐19

R. Afriyie ^1^, P. Ayamah^2^, N.A. Acheampong^1^, C. Adobea Asante^2^, S. Oppong^3^, D.K. Atuahene^4^, F.N. Poku^1^, B. Amankwa^5^

^1^Ghana AIDS Commission, Technical Services, Accra, Ghana, ^2^Ghana AIDS Commssion, Accraa, Ghana, ^3^NHS, UK, United Kingdom, ^4^Ghana AIDS Commssion, Director General, Accraa, Ghana, ^5^UNDP, Accra, Ghana

**Background**: In Ghana, head porterage is an urban phenomenon dominated by young women who have migrated from other parts of the country. These young migrants live in deplorable conditions in the city and engage in risky sexual behaviours. UNDP and GAC collaborated on an integrated outreach programme to reach them and other vulnerable populations with an integrated HIV service in the COVID‐19 era.

**Description**: To ensure sufficient coverage of the target population, leaders of the Female Head Porters (FHP) were engaged in mapping their sites with support of Peer‐Educators in the nine communities (located in the Accra Metropolis and GA Central Municipality) targeted for the intervention. Other vulnerable groups including seafarers, truck drivers and mates, HRM were reached and served. Event‐based and mobile based testing was adopted to reach the target population with integrated health outreach services namely HTS, Malaria and Hepatitis B test, BMI checking, Blood Glucose Testing, TB Sputum collection and COVID‐19 vaccination. The participants benefited health education on HIV, nutrition, sexual and gender‐based violence and substance abuse. Condom demonstration and BCC leaflets were also distributed.

**Lessons learned**: With the integrated services approach to HTS, 3,580 FHP and other vulnerable groups were reached in 9 days as against 821 and 488 reached in the whole of 2020 and 2021 respectively at the same catchment area. Moreover, the integrated HTS approach yielded HIV positivity of 2.2% as against 0.01% for each of 2020 and 2021. Over 90% of positive clients were linked to care and treatment. 1,680 were screened for Blood Pressure and Body Mass Index Screening and 1,172 blood Sugar were checked with 104 receiving COVID‐19 vaccines. Mobile testing strategy helped to overcome some barriers to HTS for persons who would otherwise not seek it. Ghetto clients had opportunity to interact with health care staff and the nutritionist and discuss matters of concern to them.

**Conclusions/Next steps**: Integrated services approach as against standalone HTS encourages more vulnerable people to test and removes stigma relating to HIV service uptake. GAC is therefore encouraged to pursue more such collaborations to reach hidden populations with the services they need.

### PESUD28 High linkage to treatment among key populations who self‐test through a peer HIV self‐test distribution and community‐based ART program among key populations in Lagos, Nigeria

A. Fernandez^1^, W. Tun^2^, E. Shoyemi^3^, J. Pulerwitz^4^, A. Adedimeji ^4^

^1^Population Council, HIV/AIDS, Lagos, Nigeria, ^2^Population Council, Washington, United States, ^3^Centre for Population Health Initiative, Lagos, Nigeria, ^4^Population Council, Washington DC, United States

**Background**: HIV self‐testing (HIVST) presents an opportunity to increase HIV testing uptake. It offers a confidential alternative particularly for highly stigmatized and criminalized populations such as men who have sex with men (MSM), transgender persons (TG), and female sex workers (FSW). However, follow‐up of HIVST recipients is often a challenge, thus hindering appropriate referral to prevention and treatment services. We report on implementation outcomes and lessons learned from community‐based HIVST distribution and linkage to community‐based ART and PrEP for key populations (KPs) in Lagos, Nigeria.

**Description**: In 2020, as part of outreach services of a community‐based health clinic (CBHC) serving KPs, HIVST kits were distributed to 1,174 MSM, 224 FSWs, and 102 TG persons (TG men: 12; TG Women: 90) by peer educators (PEs). PEs (N = 10) reached their peers at physical hotspots and through social media (WhatsApp followed by in‐person distribution) and provided HIV education and shared an HIVST demonstration video and brochure on post‐test services (e.g., confirmatory testing, ART, PrEP). Contact information was obtained from clients for follow‐up (e.g., referral to the CBHC or linked with a community health extension worker for ART or PrEP enrollment).

**Lessons learned**: 17% of recipients were first‐time testers. PEs reached 100% of kit recipients within five days of distribution (majority by phone call); all reported having unassisted self‐testing. The self‐reported positivity rates were 3.1% in MSM, 0.4% in FSWs, and 4.9% in TG persons. All KPs who self‐tested positive initiated ART; 10 of 1,458 who self‐tested negative initiated PrEP (all MSM). Employing trusted peers was an effective way of reaching KPs with HIVST. Follow‐up calls were successful due to: i) verifying the number at the time of kit distribution, ii) following‐up soon after distribution by the PEs themselves. Successful linkage to treatment was likely due to referral to a KP‐friendly community‐based clinic supplemented by community‐based ART initiation.

**Conclusions/Next steps**: The high uptake of HIVST and ART was facilitated by strategies led by PEs and post‐test services being offered in KP‐friendly clinics and in the community. HIVST is especially essential given that the COVID‐19 pandemic has limited numbers of in‐person testing at clinic and outreach.

### PESUD29 “Coming out or not”: sexual orientation disclosure, HIV testing, and homoprejudiced violence for MSM in China

X. Yan^1,2^, Y. Ni^1,2^, Y. Lu^1,2^, Q. Wang^1,2^, R.K.J. Tan^1,2^, G. Marley^1,2^, Y. Zhou^3^, W. Tang ^4,2,1^, D. Wu^5,2^

^1^University of North Carolina at Chapel Hill Project‐China, Guangzhou, China, ^2^Social Entrepreneurship for Sexual Health (SESH) Global, Guangzhou, China, ^3^Zhuhai Center for Disease Control and Prevention, Department of HIV/AIDS Prevention and Control, Zhuhai, China, ^4^Dermatology Hospital of Southern Medical University, Guangzhou, China, ^5^London School of Hygiene and Tropical Medicine, International Diagnostics Centre, London, United Kingdom

**Background**: Disclosure of sexual orientation to health professionals has been advocated in clinical settings because it could be an entry point to providing HIV testing and optimal care for men who have sex with men (MSM). Yet, sexual orientation disclosure is stigmatized or even criminalized in certain societies. This study aimed to test the association between disclosure of sexual orientation and the uptake of HIV testing services, and explore whether disclosing one's sexual orientation would increase the risk of experiencing homoprejudiced violence (i.e., violence against individuals based on their actual or perceived sexual orientation) for Chinese MSM.

**Methods**: We obtained the data from a cross‐sectional survey among Chinese MSM in January 2021. Participants were recruited online through a popular gay dating app called BlueD. The measurements ‘ever disclosed sexual orientation’, ‘ever tested for HIV’, and ‘ever self‐tested for HIV’ were all dichotomous variables. Ever experiencing homoprejudiced violence was measured on a 12‐item scale and recoded as a dichotomous variable (i.e., ever experienced any one of the twelve items). Multivariable logistic regressions were used to explore the relationships, adjusted for sociodemographic characteristics.

**Results**: A total of 1828 MSM were enrolled in the survey, of whom 73% (1334) identified as gay, 56% (1023) had ever disclosed their sexual orientation to others, 77.5% (1417) have ever tested for HIV, and 68% (1244) have ever self‐tested for HIV. 46.3% (847) reported ever experiencing homoprejudiced violence. Ever disclosing one's sexual orientation was associated with greater odds of HIV testing (aOR = 1.90, 95%CI: 1.50∼2.41) and HIV self‐testing (aOR = 1.39, 95%CI: 1.13∼1.71). However, sexual orientation disclosure was also positively associated with ever experiencing homoprejudiced violence (aOR = 1.70, 95%CI: 1.48∼2.07).

**Conclusions**: Sexual orientation disclosure is a double‐edged sword for the MSM population in China. While it showed public health benefits in improving HIV prevention by increasing the uptake of HIV testing/self‐testing services, we need to be cautious about encouraging disclosure in high‐stigma settings as it may subject MSM to greater exposure of homoprejudiced violence. Future researches should verify the causal relationship between these factors, and our findings call for an inclusive social environment that safeguards disclosure of sexual orientations.

### PESUD30 Marijuana use goals, patterns, and perceptions of health impact among persons with HIV in care in the era of state‐level legalization

R. Fredericksen ^1^, E. Fitzsimmons^1^, M. Sigal^2^, S. Dougherty^3^, J. Pearce^1^, M. Powell^1^, J. Nguyen^1^, S. Ruderman^1^, B. Whitney^1^, L. Drumright^1^, J. Ma^1^, R. Nance^1^, J. Delaney^4^, K. Mayer^2^, A. Willig^3^, H. Crane^1^, A. Hahn^1^

^1^University of Washington, Seattle, United States, ^2^Fenway Institute, Boston, United States, ^3^University of Alabama‐ Birmingham, Birmingham, United States, ^4^University of Manitoba, Winnipeg, Canada

**Background**: We sought to determine whether state‐level marijuana policy changes were associated with marijuana use behaviors and perceptions among people with HIV (PWH), including access to marijuana, goals for use, patterns of use (e.g., frequency, modality), use of other substances, and perceived health impacts.

**Methods**: We recruited PWH aged ≥18 for 1:1 interviews at three U.S.‐based HIV clinics within the Centers for AIDS Research Network of Integrated Clinical Systems (CNICS) between 2019–2020. Two clinics were in states that legalized marijuana use. PWH reporting weekly or more frequent marijuana use on routinely administered, clinic‐based, electronic assessments of patient reported outcomes (PROs) were eligible for inclusion. We transcribed and coded interviews based on sub‐topic areas of interest.

**Results**: We interviewed 29 PWH who reported weekly‐or‐greater marijuana use [80% cisgender male; mean age = 50; 48% Black, 34% White, 17% Hispanic, 10% multiracial, 7% Asian‐American]. One‐quarter of participants reported increased marijuana use since legalization in their state, primarily due to increased accessibility and a desire to explore products and varieties for therapeutic needs. The most cited therapeutic goals included relaxation/sleep (66%), increased appetite (41%), stress/anxiety relief (31%), pain relief (28%), fun/recreation (28%), to reduce cravings for another substance (17%), and to alleviate physical symptoms other than pain (10%); overall, 69% of interviewees reported they used marijuana for more than one purpose. Reasons for use were not reported to be influenced by legalization. In legalized settings, increased product diversity and attribute labeling (e.g., cannabinoid type and strength) facilitated decision‐making, allowing PWH to tailor to their specific goals. Several PWH reported marijuana's role in helping maintain sobriety from other substances. Edible marijuana products were regarded as a healthier alternative to smoking and very few reported concerns with over‐use or addiction. PWH reported stopping use once a goal was met (e.g., being pain‐free). Short‐term therapeutic benefits were prioritized over what were believed to be ambiguous, potential long‐term negative health consequences.

**Conclusions**: Among a sample of PWH who used marijuana, the broad availability of products following legalization offered a means for facilitating decision‐making for targeted therapeutic use, including stress reduction and minimization of craving alcohol and ‘harder’ substances.

### PESUD31 HIV remission trials with treatment interruption of fixed duration: Trial investigators’ perceptions of strategies to mitigate HIV transmission risk

C. Golin ^1,2^, E.A. Okumu^2^, G.E. Henderson^3^, K.J. Kuczynski^3^, N. Ormsby^3^, H.L. Peay^4^

^1^UNC Gillings Global School of Public Health, Health Behavior, Chapel Hill, United States, ^2^UNC Center for AIDS Research, Chapel Hill, United States, ^3^University of North Carolina at Chapel Hill, Social Medicine, Chapel Hill, United States, ^4^RTI International, Durham, United States

**Background**: Early phase HIV remission (“cure”) trials aim to develop interventions to eradicate HIV from the body, or to sustainably control HIV without antiretroviral treatment (ART). Controversy over analytic treatment interruption (ATI) has been generated by trials with longer duration before re‐starting ART, which involves greater risk for individuals and partners.

**Methods**: In a 2021 online survey of international HIV remission trial investigators and study team members registered on clinicaltrials.gov, we assessed perceptions of feasibility, acceptability, and efficaciousness of six HIV transmission risk mitigation strategies during trials with ATI of fixed duration, using 7‐point semantic differential scales (−3; 0; +3).

**Results**: Of 170 recruited, 52 participated. Respondents were diverse in demographic characteristics, current geographical location, and clinical trial/professional role. Mean scores indicate respondent concerned about risk of HIV transmission during ATI (M = 1.11). The mean scores for feasibility, acceptability, and efficacy for each of the transmission mitigation strategies are reported in Table 1. Requiring counseling for potential participants was judged feasible, acceptable, and efficacious. Respondents were, overall, neutral on the feasibility, acceptability, and efficacy of requiring that participants’ sexual partner(s) participate in risk counseling. Limiting participation to those who commit to abstaining from sex during the entire ATI period was judged as not feasible and acceptable, but respondents were more neutral on its efficacy. Providing referrals so that all HIV negative partners of participants can obtain PrEP, providing PrEP directly to all HIV negative partners of participants without cost, and monitoring participants for new STD acquisition as an effort to assess sexual activity were all judged as feasible, acceptable, and efficacious.

**Conclusions**: Our study demonstrates that HIV remission trial investigators are concerned about the risk of transmission. Their assessment of risk mitigation strategies for transmission risk demonstrates the importance of examining feasibility, acceptability, and efficacy when designing risk reduction programs.

**Table:** 

**Abstract PESUD31‐Table 1. Feasibility, acceptability, and efficacy of HIV transmission risk mitigation strategies during trials with ATI of fixed duration‐ longer duration before re‐starting ART**.			
Anticipate how feasible, acceptable, and efficacious you anticipate the following actions would be to reduce transmission risk during trials with ATI of fixed duration. *(−3: Not at all; 3: Extremely)*	Mean (n)		
	Feasible	Acceptable	Efficacious
Require counseling for potential participants that is focused on reducing transmission risk during ATI of fixed duration	2.31 (42)	2.14 (42)	1.07 (42)
Require that participants’ sexual partner(s) participate in risk counseling targeted to reducing transmission risk during ATI of fixed duration.	−.22 (40)	−.03 (39)	.23 (40)
Limit participation to those who commit to abstaining from sex during the entire ATI period.	−.78 (36)	−.70 (37)	−.24 (38)
Provide referrals so that all HIV negative partners of participants can obtain PrEP	1.31 (42)	1.33 (42)	1.48 (42)
Provide PrEP directly to all HIV negative partners of participants without cost.	1.02 (41)	1.45 (42)	1.60 (42)
Monitor participants for new STD acquisition as an effort to assess sexual activity.	1.85 (41)	1.40 (40)	.98 (41)

### PESUD33 Addressing the unique needs of learners living with HIV in the education setting

J. Ouma ^1^, T. Rufurwadzo^2^, M. Katekwe^1^, G. Caswell^3^, A. Stahmer^4^

^1^Global network of young people living with HIV (Y+ Global), Capetown, South Africa, ^2^Global network of young people living with HIV (Y+ Global), Amsterdam, Netherlands, the, ^3^Global Network of People Living with HIV, Capetown, South Africa, ^4^UNESCO, Paris, France

**Background**: In 2012, UNESCO collaborated with the Global Network of People living with HIV (GNP+) to produce the publication “positive Learning: Meeting the needs of young people living with HIV (YPLHIV) in the education sector”. The tool identified key issues faced by YPLHIV in education settings and recommendations for the education sector on areas including confidentiality and disclosure. In 2021 UNESCO commissioned the Global Network of Young People Living with HIV (Y+ Global) with support from GNP+ to revise the tool to reflect the current needs of YPLHIV in schools and to develop recommendations for stakeholders.

**Description**: The positive Learning revision process capitalized on youth leadership and leveraged on the experiences of the YPLHIV in schools engaging 145 young people from Asia (21),Middle East and North Africa (19),Anglophone and Francophone Africa (43),Eastern Europe and Central Asia (20) and Latin America and Hispanic Caribbean (17) and Global (25) . The revised tool highlights recommendations in seven thematic areas; comprehensive sexuality education, confidentiality and sharing information about HIV status, end HIV related stigma, discrimination, bullying and violence, HIV treatment and care, sexual reproductive health and rights, mental health and psychosocial well being and creating an inclusive and health promoting learning environment.

**Lessons learned**: YPLHIV globally are facing a compendium of cross cutting challenges such as stigma and discrimination, mental health issues and adherence to medication in the school settings. As YPLHIV we are aware of the issues and we have the solutions to these issues. YPLHIV leadership is critical as we are the agents of change in addressing these challenges and holding stakeholders accountable.

**Conclusions/Next steps**: The Positive Learning tool provides YPLHIV in schools with safe and conducive learning environments to improve treatment, education and mental wellbeing outcomes. The tool will be disseminated on a rolling basis through all platforms to ensure that relevant stakeholders in countries are reached.

### PESUD34 Pathways from multiple stigmas to ART adherence among transgender women and men who have sex with men newly diagnosed with HIV in India: a prospective cohort study

V.V. Patel ^1^, V. Chakrapani^2^, A. Gupta^3^, F. Gulfam^3^, J. Kaur^4^, N. Chopra^1^, J. Ross^1^, J. Rendina^5^, A. Ahalawat^3^, S. Reddy^3^, S.A. Safren^6^, S.A. Golub^7^

^1^Albert Einstein College of Medicine, Montefiore Health System, General Internal Medicine, Bronx, United States, ^2^Centre for Sexuality and Health Research and Policy (C‐SHaRP), Chennai, India, ^3^Alliance India (India HIV/AIDS Alliance), New Delhi, India, ^4^Post Graduate Institute of Medical Education & Research, Chandigarh, India, ^5^Whitman‐Walker Institute, Washington D.C., United States, ^6^University of Miami, Psychology, Coral Gables, United States, ^7^Hunter College, City University of New York, Hunter HIV/AIDS Research Team, Department of Psychology, New York, United States

**Background**: Stigma remains a major barrier to ART adherence among persons newly diagnosed with HIV. However, little research exists on the impact of multiple stigmas on treatment outcomes for transgender women (TGW) or men who have sex with men (MSM) living with HIV in South Asia. Guided by the Health Stigma Discrimination Framework, we conducted a longitudinal study among Indian TGW/MSM to understand how multiple stigmas (enacted, anticipated, and internalized) related to HIV and either gender (for TGW) or sexuality (for MSM) may influence ART adherence.

**Methods**: Between 2020–2021, 140 TGW and 227 MSM from 11 Indian states (≥18 years, diagnosed with HIV in past six‐months) completed interviews at baseline, three‐ and six‐months. Using mediational analyses, we tested the associations of baseline stigma scores (HIV‐ and gender‐identity for TGW, or sexual‐identity for MSM) stigmas with past 30‐day self‐reported ART adherence (0–100) at six‐months, and the indirect effects of stigma on ART adherence through three‐month depression, anxiety, and alcohol use scores.

**Results**: Among TGW, mean age was 31.1 years and 23% were illiterate. At six‐months, 92% of TGW had been prescribed ART; mean (sd) adherence was 77.9±17.1. Mediational analysis (Fig.1a) revealed a significant direct‐effect of anticipated HIV‐stigma in healthcare on adherence and indirect‐effect of enacted HIV‐stigma via alcohol use. Among MSM, mean age was 33.3 years and 18.5% were illiterate. At six‐months, 98% of MSM were prescribed ART; mean (sd) adherence was 74.9±20.1. Mediational analysis (Fig.1b) revealed significant direct‐effects from internalized MSM‐stigma and enacted community HIV‐stigma on adherence and indirect‐effects of enacted HIV‐stigma in healthcare via alcohol use.

**Figure:**
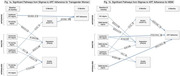
**Abstract PESUD34‐Figure 1**.

**Conclusions**: Adherence was suboptimal and distinct forms of stigmas, either directly or indirectly differentially influenced ART‐adherence among TGW and MSM. For both TGW and MSM, interventions to reduce stigma faced in healthcare settings and beyond and by addressing alcohol use could improve ART adherence.

### PESUD35 Racial residential segregation as a structural barrier to viral suppression among people living with HIV in Southern United States from 2013 to 2018: a county‐level longitudinal study

F. Shi ^1^, J. Zhang^2^, X. Yang^1^, Z. Li^2^, X. Sun^2^, C. Zeng^2^, H. Ning^2^, B. Olatosi^2^, S. Weissman^3^, X. Li^1^

^1^University of South Carolina, Health Promotion, Education, and Behavior, Columbia, United States, ^2^University of South Carolina, Columbia, United States, ^3^University of South Carolina, School of Medicine, Columbia, United States

**Background**: Achieving viral suppression (VS) is one of the crucial goals of HIV care cascade. Residential segregation by race has long been framed as a potential structural barrier to successful VS at the individual level, but evidence is scarce at the population level, which has strong implication for healthcare policymaking and resources allocation. This study aims to examine the longitudinal relationship between county‐level racial segregation and VS rate among people living with HIV (PLWH) in 46 counties of South Carolina (SC) from 2013 to 2018.

**Methods**: De‐identified laboratory reports of all PLWH in SC were extracted from the electronic HIV/AIDS reporting system in the SC Department of Health and Environment Control from January 2013 to December 2018. Based on CDC's definition, county‐level VS rate was calculated as the percentage of PLWH who have viral load (VL) less than 200 copies/ml in the last VL report at each calendar year (excluding those newly diagnosed in that year). Racial residential segregation was calculated using Massy and Denton's formula of isolation index for Black residents. The association between racial segregation and VS rate was tested by generalized linear mixed model, adjusting for potential confounders (e.g., sociodemographic characteristics, social capital, HIV care resources) and time.

**Results**: From 2013 to 2018, the average VS rate in SC increased from 64.3% to 65.4% among all PLWH. Counties in Upstate (Spartanburg and Cherokee) and Lowcountry (Orangeburg) reported low VS rate. Final model revealed that counties with higher residential isolation experienced lower VS rate (b = −0.354, 95%CI: −0.614 ∼ −0.095). However, stronger county‐level social capital, which was indicated by community health index, was related to higher VS rate in SC (b = 0.757, 95%CI: 0.277∼1.237).

**Conclusions**: This study described the temporal and spatial distribution of VS rate in SC. Structural influence of residential segregation on viral suppression was found. It is also suggested that more social cohesion at the county level was a protective factor of VS. These findings emphasize the need to address racial disparities in social capital based on racial residential segregation as part of a comprehensive strategy to curb the HIV epidemic.

### PESUD36 Improving our understanding of how structural determinants impact HIV epidemics: A scoping review of dynamic models to guide future research

J. Stannah ^1^, J.L. Flores Anato^1^, K.M. Mitchell^2^, J. Larmarange^3^, M. Maheu‐Giroux^1^, M.‐C. Boily^2^

^1^McGill University, Department of Epidemiology, Biostatistics, and Occupational Health, Montreal, Canada, ^2^Imperial College London, Department of Infectious Disease Epidemiology, London, United Kingdom, ^3^Université Paris Descartes, Institut de Recherche pour le Développement, Paris, France

**Background**: Dynamic models of HIV transmission have proven valuable tools for informing HIV prevention strategies. Including structural determinants in models is crucial to estimate their population‐level impacts on HIV transmission and inform efforts towards HIV elimination. However, this is challenging due to a lack of coherent conceptual frameworks, limited understanding of their specific causal pathways, and few empirical estimates of their impacts on downstream mediators.

**Methods**: With the overarching aim to improve models, we conducted a scoping review of studies that used dynamic HIV transmission models to evaluate the impact of structural determinants. From included studies, we extracted information on the types of structural determinants and methods used to model their impacts on HIV transmission. We appraised studies on how they conceptualized structural exposures and represented their causal relationships over time within models.

**Results**: We identified 9 dynamic transmission modelling studies that incorporated structural determinants of HIV, including violence (N = 3), incarceration (N = 2), stigma (N = 2), housing instability (N = 2), migration (N = 1), and education (N = 1). Only one study modelled multiple determinants simultaneously. In most models, structural determinants were conceptualized using current, recent, non‐recent and/or lifetime exposure categories. Modelled structural determinants largely impacted HIV transmission through mediated effects on one or more proximate risk factors, including sharing injection equipment, condom use, number of partners, and access to treatment. However, causal pathways were simplistic, with few mediators and/or lack of clear empirical justification. To measure impact, most studies simply assumed the elimination of structural determinants in counterfactual comparison scenarios. Few models included long‐term and/or delayed effects of past, recurrent, or acute exposure, potentially overestimating impacts of determinants.

**Conclusions**: Despite the importance of structural determinants for HIV prevention, methods for including them in dynamic HIV transmission models remain insufficient. Few studies have attempted to incorporate structural determinants in HIV models, and methods vary considerably. To improve inferences, models should adopt precise exposure definitions, deconstruct and estimate their complex causal pathways, and translate them into their mechanistic components. The need for development of coherent frameworks to conceptualize the synergistic interplay between strengthened empirical data analysis and the inclusion of structural determinants in dynamic models is pressing.

### PESUD37 “Guys are different”: Young women's views on heterosexual relationship dynamics and how they influence women's potential PrEP uptake and disclosure in Durban, South Africa

L. Miller ^1^, A. Harrison^2^, N. Bhengu^3^, N. Tesfay^3^, S. Magutshwa^3^, S. Khumalo^3^, S. Bergam^4^, T. Exner^5^, J. Hanass‐Hancock^3^, S. Hoffman^5,6^

^1^Columbia University, ICAP, Mailman School of Public Health, New York, United States, ^2^Brown University, Behavioral and Social Sciences, International Health Institute, Providence, United States, ^3^South African Medical Research Council, Gender and Health Research Unit, Durban, South Africa, ^4^Brown University, Providence, United States, ^5^NYS Psychiatric Institute and Columbia University Irving Medical Center, HIV Center for Clinical and Behavioral Studies, New York, United States, ^6^Columbia University, Department of Epidemiology, Mailman School of Public Health, New York, United States

**Background**: Considerable evidence demonstrates that heterosexual relationship dynamics influence women's decisions around HIV prevention methods, but little research has been conducted among educated South African women. In the context of oral pre‐exposure‐prophylaxis (PrEP) becoming publicly available in South Africa (2019), we explored urban, educated young women's views on relationship dynamics with male partners, how these dynamics might impact women's use of PrEP, and how women might navigate those dynamics if they chose to use PrEP. Understanding and taking into account the realities of the lives of women is key to designing successful PrEP programs.

**Methods**: This analysis utilized qualitative data from a study to develop a gender‐focused PrEP information‐motivation workshop to introduce young women to PrEP, in Durban, South Africa. Participants were aged 18–25, educated, and recruited from urban clinic and community settings. Six focus group discussions and eight in‐depth interviews were conducted with 46 women. Data were analyzed thematically.

**Results**: Women described men as having a different culture and set of behaviors than women and as experiencing different societal gender norms, which leads to women being at a greater risk for HIV. These differences bring complexity to women's relationships and influence their choices around PrEP use and disclosure. While acknowledging the potential benefits of PrEP, women stated that risks included: potential for anger and loss of trust in relationships, breakup, physical violence, pregnancy or other sexually transmitted infections. Despite these concerns, woman expressed desire for mutuality in relationships and shared suggestions to manage choices around PrEP use and disclosure, including willingness to end relationships.

**Conclusions**: These results document the challenges that even urban, educated women experience in heterosexual relationships with respect to gender dynamics and HIV prevention and add to the growing body of evidence that women's use and adherence to PrEP in Africa is shaped by male partners and women's perceptions of their male partners’ reactions. For PrEP to be rolled out successfully, implementation programs need to provide women with concrete methods to improve self‐agency and communication skills that address conflict. Women need these skills to navigate the complex power dynamics they experience in heterosexual relationships.

### PESUD38 HIV testing experiences and priorities among refugee youth in a humanitarian setting in Uganda

K. Malama ^1^, C. Logie^2^, M. Okumu^3^, M. Loutet^1^, M. Coelho^1^, S. Odong^4^, N. Kisubi^4^, R. Lash^2^, P. Kyambadde^5^

^1^University of Toronto, Toronto, Canada, ^2^University of Toronto, Social Work, Toronto, Canada, ^3^University of Illinois Urbana‐Champaign, Urbana, United States, ^4^Uganda Refugee and Disaster Management Council, Yumbe, Uganda, ^5^Uganda Ministry of Health, Kampala, Uganda

**Background**: Refugee youth experience social drivers of HIV, including violence, poverty, and constrained access to HIV prevention services. Scant research has focused on youth‐centred HIV testing strategies—including HIV self‐testing (HIV‐ST)—in humanitarian crises. We explored HIV testing experiences and preferences among refugee youth in Bidi Bidi refugee settlement, Uganda to inform development of an HIV self‐testing intervention.

**Figure:**
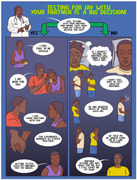
**Abstract PESUD38‐Figure 1**.

**Methods**: We implemented a multi‐method community‐based study in Bidi Bidi. We conducted four focus groups (FG) with refugee youth, 2 with ages 16–19 (women: n = 1 FG; men; n = 1 FG) and 2 with ages 20–24 (women: n = 1 FG; men; n = 1 FG). We developed a 9‐page comic series illustrating FG findings and then conducted a 3‐day human‐centred design (HCD) workshop with refugee peer navigators (n = 8). We conducted thematic analyses across methods.

**Results**: Focus group participants (n = 40; n = 16 ages 16–19, n = 24 ages 20–24; 20 men, 20 women) were largely from South Sudan (83%). Most had a lifetime HIV test (83%) yet three‐quarters (78%) had not tested in the past year. FG narratives revealed HIV testing decision‐making considerations of a) positive outcomes (self‐care, post‐rape awareness, protecting others from infection, self‐knowledge) and b) negative outcomes (HIV‐related stigma, relationship mistrust/violence, healthcare mistreatment). Participants expressed enthusiasm regarding HIV‐ST (e.g., confidentiality, convenience) yet women detailed concerns of relationship violence with HIV‐positive results. Comics detailed these HIV testing decision‐making processes, including risk‐assessment for partner testing. HCD findings identified ways to create youth‐friendly HIV testing spaces, address HIV‐ST misinformation, and reduce community and health‐care stigma.

**Conclusions**: Findings provide unique insight into refugee youth HIV testing experiences and priorities. This research highlights the salience of the disclosure process model to understand antecedent goals (approach goals for positive outcomes, avoidance goals for negative outcomes) for HIV testing, including HIV‐ST. Refugee youths' experiences of cumulative violence signal the need for trauma‐informed, youth‐centred HIV testing strategies in humanitarian crises.

### PESUD39 Partner social support and sexual satisfaction buffer the effects of stigma on ART adherencein Malawian couples

S.A. Gutin ^1^, A. Ruark^2^, L.A. Darbes^3^, T.B. Neilands^1^, J. Mkandawire^4^, A.A. Conroy^1^

^1^UCSF Center for AIDS Prevention Studies, Division of Prevention Science, San Francisco, United States, ^2^Wheaton College, Wheaton, United States, ^3^University of Michigan, Ann Arbor, United States, ^4^Invest in Knowledge, Zomba, Malawi

**Background**: For couples affected by HIV, relationship dynamics can have a positive impact on adherence to antiretroviral therapy (ART) via social support and coping. While research suggests that general partner support can enhance ART adherence and that stigma may exert its influence dyadically, few if any studies in sub‐Saharan Africa have explored whether relationship dynamics can buffer the experience of anticipated stigma from family members and healthcare providers on ART adherence. We examined this question among a sample of couples living with HIV in Malawi.

**Methods**: Married couples (N = 211) with at least one partner on ART were recruited from HIV clinic waiting rooms in Zomba, Malawi. Partners completed separate surveys on anticipated HIV stigma from family members of healthcare providers, relationship dynamics (e.g., intimacy, trust, sexual satisfaction, general partner social support, communication), and ART adherence. Using generalized estimating equation (GEE) regression models, we tested for associations between anticipated stigma and ART adherence at the individual level, and whether this association was moderated by relationship dynamics at the couple level, after controlling for socio‐demographics and relationship duration.

**Results**: Couples had been together for a mean of 12.5 years (SD = 9.0), 66% were sero‐concordant, and 95.6% of participants reported 90–100% adherence. The mean anticipated stigma score was 1.6 (SD = 0.74, scale range 1–5), with higher scores indicating higher stigma. In multivariable models, the odds of having 90–100% adherence were 45% lower for each one‐unit increase in anticipated HIV stigma (aOR = 0.55, 95%CI = 0.34; 0.89). There were significant interactions between partner social support and anticipated stigma from family members and healthcare providers (p = 0.032) and between sexual satisfaction and anticipated stigma (p = 0.039), showing that the association between higher stigma and non‐adherence was moderated in couples with higher social support and sexual satisfaction.

**Conclusions**: Increased anticipated HIV stigma is associated with higher non‐adherence to ART. Supportive and fulfilling relationships, particularly those with greater partner support and sexual satisfaction, may buffer the experience of stigma on ART adherence. Couple‐based interventions that intervene on these important aspects of relationships may help lessen the negative impact of HIV stigma on HIV treatment engagement such as ART adherence.

### PESUD40 Anticipated stigma among recently diagnosed HIV clients in the UTT era in Johannesburg, South Africa

I. Mokhele ^1^, T. Sineke^1^, J. Bor^1,2^, D. Onoya^1^

^1^Health Economics and Epidemiology Research Office, Department of Internal Medicine, School of Clinical Medicine, Faculty of Health Sciences, University of the Witwatersrand, Johannesburg, South Africa, ^2^Boston University School of Public Health, Department of Global Health, Boston, United States

**Background**: Anticipated stigma – the fear that HIV diagnosis and status disclosure could have negative social implications – may adversely affect engagement with HIV care and treatment, despite universal eligibility for treatment under universal‐test‐and‐treat (UTT). We aimed to determine prevalence and predictors of anticipated stigma among newly HIV‐diagnosed individuals under the UTT policy in Johannesburg, South Africa.

**Methods**: We analyzed a cross‐sectional survey of 652 newly HIV‐diagnosed adults (≥18 years) (64.1% female, median age was 33 years, interquartile range [IQR]: 28–39) enrolled in a cohort study from October 2017 to August 2018 from four primary clinics in Johannesburg. Participants were interviewed immediately after receiving their HIV test results. We used an adapted five‐item, four‐point scale measuring agreement with statements regarding HIV disclosure concerns and HIV status concealment (Cronbach's alpha = 0.82). Mean scores were categorized as “low‐to‐medium” (score <= 2.5), or “high” (score > 2.5). We used Modified Poisson regression to assess for predictors of high anticipated stigma and report adjusted risk ratios (aRR) with 95% confidence intervals (CIs).

**Results**: Overall, 55% of study participants had high anticipated stigma; 55.8% for males, 61.1% for 18–29‐year‐olds, and 43% for those married. Unmarried individuals who were in a relationship had a higher risk of high anticipated stigma than those married (aRR 1.10, 95% CI: 1.01–1.18). Risk of high anticipated stigma was lower among: older individuals (aRR 0.94 for being 30–39 vs 18–29 years, 95% CI: 0.88–0.99), those having a primary house in another province/rural (aRR 0.82 for primary house in another country vs current house, 95% CI: 0.78–0.87), (aRR 0.83 for primary house in another country vs current house, 95% CI: 0.78–0.88), those living in current homes for ≥5 years (aRR 0.93 for >5 years vs <1 year, 95% CI: 0.88–0.99), those with low ART concerns (aRR 0.86, 95 % CI: 0.82–0.90), and those with low perceived social‐support (aRR 0.79 for low vs high, 95 % CI: 0.70–0.88).

**Conclusions**: Over 50% of adults diagnosed with HIV in the UTT era experienced high anticipated stigma. Findings highlight the need to address factors that continue to drive anticipated stigma, to mitigate the potential impact on engagement in HIV care.

### PESUD41 Experiences of viral detectability, undetectability, and stigma among recently diagnosed people living with HIV in Australia

N. Wells ^1^, S. Philpott^1^, D. Murphy^1^, J. Ellard^2^, G. Prestage^1^

^1^The Kirby Institute, Sydney, Australia, ^2^Australian Research Centre in Sex, Health and Society, Melbourne, Australia

**Background**: HIV treatments can improve the health and wellbeing of people living with HIV (PLHIV) and eliminate the risk of sexual transmission for those who can maintain undetectable viral load (UVL). This has been accompanied by a promise of reducing HIV‐related stigma. We explored how detectable and undetectable viral loads were experienced by PLHIV, including how these experiences interplayed with experiences of self‐ and anticipated stigma.

**Methods**: The RISE study is an ongoing qualitative cohort study of 34 PLHIV diagnosed from 2016 onward in Australia. Of these, 25 had participated in follow up interviews, providing 59 interviews for analysis. Interviews were conducted between January 2019 and November 2021.

**Results**: Median age was 41; 32 were male and 2 were female; 21 were gay, 8 were bisexual, and 5 were heterosexual. All participants except 1 were on treatment and had UVL when first interviewed.

Participants frequently associated the period in which they were detectable with feeling “dirty,” “viral,” and “a risk” to sexual partners. During this period, most participants limited or ceased sex, even when in an ongoing romantic relationship. Participants commonly framed reaching UVL as an important goal in their HIV care, with UVL acting as a marker of good health and as reenabling sexual and/or romantic relationships. This helped reduce experiences of self‐stigma and enabled participants to envision living a “normal” life, something they could not imagine while detectable. However, some participants described that despite having UVL, and their belief in its preventative efficacy, they remained hesitant to reengage with sex. This was mainly due to a perceived lack of awareness among the broader, HIV‐negative population around UVL, concerns of sexual rejection, and anticipated stigma.

**Conclusions**: UVL can improve the physical and emotional wellbeing of PLHIV. However, these experiences occur in a context of persistent HIV‐related social stigma and a lack of awareness around UVL more broadly. While UVL can reduce experiences of self‐stigma, many PLHIV continue to anticipate HIV‐related stigma and sexual rejection. This highlights the limits of biomedicine alone as a stigma‐reduction strategy and the need for strategies, programs, and interventions that aim to reduce HIV‐related stigma in the broader community.

### PESUD42 “Fear really comes from the unknowns”: navigating ‘unknowable’ stigma and discrimination among people living with HIV in Singapore

J. Dewaele^1^, S. Hyder^1^, R.K.J. Tan^2,3^, C.S. Wong^4^, B.C. Ng^1^, E. Cambria^1^, S. Banerjee^5^, R. Chan^5^, R. Jain ^1^

^1^Nanyang Technological University, Singapore, Singapore, ^2^University of North Carolina Project‐China, Guangzhou, China, ^3^National University of Singapore, Saw Swee Hock School of Public Health, Singapore, Singapore, ^4^National Centre for Infectious Diseases, Singapore, Singapore, ^5^Action for AIDS Singapore, Singapore, Singapore

**Background**: Structural stigma and institutionalised forms of discrimination towards people living with HIV (PLHIV), especially in the areas of health insurance, immigration, and employment remain pervasive in many settings. However, no study in Singapore has qualitatively explored how PLHIV navigate such forms of discrimination, its impact on health/social service seeking behaviour and quality of life, and interventions required to address such inequities.

**Methods**: Semi‐structured qualitative interviews were conducted with 73 participants. These included 56 PLHIV (30 men who have sex with men, 23 heterosexual men, 3 women) and 17 stakeholders including healthcare professionals and other allied workers. Interviews focused on participant perspectives or experiences of HIV diagnosis, navigating healthcare, attitudes towards and impact of HIV on relationships. Data were analysed through inductive thematic analysis.

**Results**: Participants highlighted that they were aware of institutionalised discrimination towards PLHIV (or experienced it, for participants who were PLHIV) across various aspects of their lives. These included experiences – overt and covert – of discrimination in education, workplace, and healthcare settings. However, participants disclosed a greater discriminatory impact due to the manifold unknowns; that is, experiences resulting from ‘unknowable’ discrimination. We interpreted this as participants' fear of potential legal and/or social repercussions resulting from the disclosure of their HIV status that they may not be able to anticipate. Even though employers may not overtly discriminate, the fear of such ‘unknowable’ discrimination influenced decisions to conceal HIV status in job applications and workplaces. This shroud of fear restricted agency for PLHIV, and impacted their regular medical follow‐ups, socialising behaviours, and overall quality of life. Consequently, many participants felt that concealment of their status, and bypassing potential educational, employment, and even health opportunities were the only ways of protecting themselves from such ‘unknowable’ stigma. Both PLHIV and stakeholders articulated that an anti‐discrimination framework is urgently needed to address such uncertainties in their lives.

**Conclusions**: An anti‐discrimination framework, enforceable by law, on the institutional treatment of PLHIV would remove unpredictability and address the manifold unknowns surrounding discriminatory experiences and improve their quality of life. Subsequent elimination of fear for unknown discrimination may also greatly facilitate timely testing, linkage to care and treatment.

### PESUD43 Engagement in HIV care and on‐going substance use: A qualitative sub‐study of BASE participants

L. Heerten‐Rodriguez ^1^, J. Coleman^2^, J. Havens^3^

^1^University of Nebraska at Omaha, Grace Abbott School of Social Work, Omaha, United States, ^2^University of Nebraska at Omaha, School of Health and Kinesiology, Omaha, United States, ^3^University of Nebraska Medical Center, College of Pharmacy, Omaha, United States

**Background**: Of the 1.2 million people living with HIV (PWH) in the United States, an estimated 48% are also experiencing a substance use disorder (SUD). Inconsistencies in treatment adherence and retention in HIV care have been reported among PWH that use illicit drugs, often leading to poor treatment outcomes and increased HIV transmission. This qualitative sub‐study explored factors that influence engagement in HIV care among PWH and ongoing SUD.

**Methods**: In‐depth, semi‐structured interviews were conducted in 2021 with 15 PWH/SUD and were enrolled in a pilot study assessing the efficacy and safety of bictegravir/emtricitabine/tenofovir alafenamide (BASE study; NCT03998176) in Omaha, NE, USA. Interviews addressed participants' experiences living with HIV, substance use, and engagement in HIV care. Transcripts were analyzed using the methods of constructivist grounded theory, including initial coding, focused coding and categorization.

**Table:** 

**Abstract PESUD43‐Table 1**.	
Participant Sex	80% Male, 20% Female
Race/Ethnicity	73% White Non‐Hispanic, 13% White Hispanic, 13% Black/African‐American
Age	Average 43, Range 30–62
Risk factors	53% MSM, 40% IVDU
Reported Substance Use	100% Methamphetamine, 7% Cocaine, 7% Opiate, 7% Sedative

**Results**: Participants described complex, interconnected, and mutually reinforcing barriers to engagement in HIV care. For example, experiences of homelessness often disrupted HIV care and medication adherence. At the same time, participants experiencing homelessness also experienced stigma from social service providers related to drug use, HIV status, sexual orientation, and participation in sex work. Many participants internalized responsibility for these experiences, viewing themselves as unworthy of HIV care which would improve their health or extend their lifespan. In the absence of strong intrapersonal motivation to engage in HIV care, many participants relied heavily on interpersonal motivation, including caring for themselves in order to support loved ones, honoring the efforts of clinic staff, or attempting to prove others wrong. Engagement in HIV care in respectful and supportive environments increased some participants’ sense of self‐worth and their own intrapersonal motivation to continue engaging in HIV care.

**Conclusions**: The development of this mid‐range explanatory theory of engagement in HIV care for PWH/SUD highlights the significant role of layered stigma, as well as the importance of recognizing and utilizing interpersonal motivation to help overcome barriers to engagement in HIV care. Results have implications for clinical care and intervention development.

### PESUD44 A longitudinal examination of anticipated HIV stigma as a mediator of the relationship between enacted and internalized stigma and self‐rated health

J.M. Lo Hog Tian ^1,2^, J. Watson^1^, R.G. Maunder^3,4^, J.A. Parsons^5^, S.B. Rourke^1,4^, The Ontario HIV Stigma Index Team

^1^Unity Health Toronto, MAP Centre for Urban Health Solutions, Toronto, Canada, ^2^University of Toronto, Institute of Medical Science, Toronto, Canada, ^3^Mount Sinai Hospital, Department of Psychiatry, Toronto, Canada, ^4^University of Toronto, Department of Psychiatry, Toronto, Canada, ^5^Unity Health Toronto, Applied Health Research Centre, Toronto, Canada

**Background**: HIV stigma remains high in Canada, causing significant impact on the health and wellbeing of people living with HIV. There is a limited understanding, however, around how different types of stigma act interact to impact overall health. This study examines how enacted and internalized stigma may lead to worse self‐rated physical, mental, and overall health through anticipated stigma as a mediator.

**Methods**: We recruited 724 participants in Ontario from September 2018 – August 2019 to complete the People Living with HIV Stigma Index at baseline (t_1_) using validated measures for stigma and quality of life. Approximately two years later we resurveyed participants (n = 407) with the same instruments (t_2_). Five separate mediation models were created with enacted and internalized stigma at t_1_ as the antecedents and physical health, mental health, and overall health at t_2_ as the outcomes. Anticipated stigma at t_2_ was entered as the mediator in all models. Age, years since HIV diagnosis, gender, ethnicity, sexual orientation, geographic region, education, employment, and anticipated stigma at t_1_ were entered into the models as covariates.

**Results**: With internalized stigma (t_1_) as the antecedent, anticipated stigma (t_2_) was a significant mediator leading to both decreased mental health (indirect effect = −0.48, 95% CI = −1.05, −0.01) and overall health (indirect effect = 0.04, 95% CI = 0.00, 0.08). The model with physical health as the outcome was not significant, nor were any of the models with enacted stigma (t_1_) as the antecedent.

**Conclusions**: We found that higher internalized stigma can lead to an increase in anticipated stigma which resulted in worse mental health and overall health. These findings show how internalizing negative thoughts and feelings about living with HIV can lead to anticipating experiences of discrimination and stereotyping with consequences for health and wellbeing. This highlights potential points for intervention to reduce the negative impacts of stigma.

### PESUD45 Influence of multiple stigmas on psychosocial problems and condom use among MSM and transgender women in India: Findings from a longitudinal S3 (stigma, syndemics and sex) cohort study

V. Chakrapani ^1^, J. Kaur^2^, A. Sebastian^3^, S. Rawat^4^, R. Nelson^1^, D. Baruah^4^, M. Shunmugam^1^, A Jaya^5^, P.A. Newman^6^

^1^Centre for Sexuality and Health Research and Policy (C‐SHaRP), Chennai, India, ^2^Post Graduate Institute of Medical Education and Research (PGIMER), Chandigarh, India, ^3^National Institute of Advanced Sciences (NIAS), Bangalore, India, ^4^The Humsafar Trust, Mumbai, India, ^5^Sahodaran, Chennai, India, ^6^University of Toronto, Toronto, Canada

**Background**: The disproportionate HIV burden among MSM and transgender women (TGW) in India persists along with multiple forms of stigma and psychosocial problems such as depression and problematic alcohol use. Amid limited research in India on the associations between multiple stigmas, psychosocial problems and condomless anal sex (CAS), we explored these associations, informed by minority stress and syndemic theories.

**Methods**: We used two‐wave data (November 2020 to Jan 2022) from an ongoing cohort study with 500 MSM and 500 TGW recruited through community‐based organisations that conduct HIV preventive interventions in Chennai/Mumbai. Path analyses were conducted (Mplus‐8) to predict CAS with male non‐primary partners (wave‐2) from stigma scores (wave‐1 sexual stigma, transgender identity stigma, sex work stigma) and psychosocial variables (wave‐2 depression, anxiety, internalized homonegativity, internalized transprejudice and alcohol use).

**Results**: Among MSM (mean age = 28.2 years; HIV = 4.2%)) and TGW (mean age = 27.6 years; HIV = 4.8%), CAS (wave‐2) was 23.2% and 72.3%, respectively. Compared to MSM, TGW had higher prevalence of moderate depression (MSM‐4.3%; TGW‐21.1%) and anxiety (MSM‐16.2%; TGW‐45.9%), but lower prevalence of problematic alcohol use (MSM‐13.4%; TGW‐12.1%). Among TGW, sex work stigma, internalized transprejudice, depression and anxiety had significant direct effects on CAS (Figure‐1). Transgender identity stigma had significant direct effects on depression, anxiety and alcohol use, and significant indirect effect on CAS through anxiety. Among MSM, sexual stigma had significant direct effects on CAS, depression and anxiety, and sex work stigma had significant direct effects on internalized homonegativity and alcohol use (Figure‐1).

**Figure:**
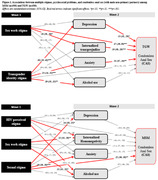
**Abstract PESUD45‐Figure 1**.

**Conclusions**: Stigmas faced by MSM (sexual, sex work) and TGW (transgender identity) contributed directly and indirectly to HIV risk through multiple psychosocial problems. Expanded efforts to reduce societal stigma against transgender people, MSM and those involved in sex work, and to address population‐specific psychosocial problems, are needed to promote safer sex and mental health among TGW and MSM in India.

### PESUD46 Impacts of intersecting stigma towards Gay, Bisexual men and other Men Who have Sex with Men (GBMSM) on HIV care in a clinical setting: breaking the vicious cycle in Zambia

S. Qiao ^1^, O. Adeagbo^2^, G.S. Mohammad^2^, L. Ngosa^3^, M. Kabwe^4^, A. Sharma^5^, A. Menon^6^, X. Li^2^, G. Harper^7^, C. Lwatula^8^

^1^Shan Qiao, Health Promotion Education and Behavior, Columbia, United States, ^2^University of South Carolina, Health Promotion Education and Behavior, Columbia, United States, ^3^DZL, Lusaka, Zambia, ^4^The Lotus Identity, Lusaka, Zambia, ^5^CIDRZ, Lusaka, Zambia, ^6^University of Zambia, Department of Psychology, Lusaka, Zambia, ^7^University of Michigan, Department of Health Behavior and Health Education, Ann Arbor, United States, ^8^University of Zambia, Lusaka, Zambia

**Background**: GBMSM in Zambia face tremendous challenges in accessing HIV care services due to cultural, legal and religious context. A prominent barrier for GBMSM is intersecting stigma in HIV/STI clinic setting. However, few studies have examined impacts of stigma at various ecological levels based on GBMSM's lived experience in Zambia from a holistic perspective. We aim to explore how the impacts of stigma at different ecological levels (intrapersonal, interpersonal and institutional level) interact with each other as a vicious cycle of impeding GBMSM from HIV‐related care and services.

**Methods**: In‐depth interviews were conducted with 19 GBMSM participants aged 18 to 35 purposively recruited from Lusaka in 2021. Interviews were conducted in English in private rooms, lasted 30–80 minutes and were audio‐recorded with participant consent. The audio files were transcribed verbatim and then managed and iteratively coded with Nvivo. Inductive approach was applied to conduct thematic analysis.

**Results**: The impacts of intersecting stigma at different ecological levels interacted with each other as a vicious cycle preventing GBMSM from the linkage, engagement and retention in HIV prevention and treatment cascade. Mental health and substance abuse issues negatively affected their relationship with family/friends, causing social isolation which increased their stress, and reduced social support. Confidentiality breaches in clinical setting caused unintentional disclosure of sexual orientation or HIV/STI status to family, friends, and communities, exaggerating the tensions between GBMSM and their social environment. Detention and harassment by law enforcement increased the distrust in institutes including some public/government facilities, which drove many GBMSM to select NGOs/private clinics. However, not all GBMSM could access affordable and sustainable HIV care clinics/programs. GBMSM with negative self‐images and mental health and substance abuse issues gave up self‐care and self‐management, which influenced their health seeking behaviors and medicine adherence (e.g., PrEP use). The negative experiences in health facilities further reinforced the existing social stigma against GBMSM and threatened their self‐image.

**Conclusions**: To increase GBMSM's HIV care access and improve their psychosocial wellbeing in Zambia, integrated intervention strategies are needed to break the vicious cycle of the impacts of intersecting stigma at intrapersonal, interpersonal and institutional levels.

### PESUD47 Motivations behind seeking religious and spiritual support and their impact on health and social outcomes for PLHIV in Singapore

S. Hyder ^1^, R.K.J. Tan^2,3^, J. Dewaele^1^, C.S. Wong^4^, B.C. Ng^1^, E. Cambria^1^, R. Chan^5^, S. Banerjee^5^, R. Jain^1^

^1^Nanyang Technological University, Singapore, Singapore, ^2^University of North Carolina Project‐China, Guangzhou, China, ^3^National University of Singapore, Saw Swee Hock School of Public Health, Singapore, Singapore, ^4^National Centre for Infectious Diseases, Singapore, Singapore, ^5^Action for AIDS, Singapore, Singapore

**Background**: Existing literature on religion and HIV identified that people living with HIV (PLHIV) who have a religious or spiritual affiliation believe faith helps with coping with illness and finding a renewed sense of purpose in life. However, there is no existing study on religion and HIV in Singapore, much less a study on religion as a resource for treatment or support in clinical interventions for PLHIV.

**Methods**: Semi‐structured qualitative interviews were conducted with a total of 73 participants. These included 56 PLHIV (30 men who have sex with men, 23 heterosexual men, 3 women) and 17 stakeholders including healthcare workers, contact tracers, religious leaders, social workers, and volunteers. Interviews focused on PLHIV and stakeholders’ perspectives or experiences of HIV diagnosis, navigating healthcare, attitudes towards HIV, and impact of HIV on relationships. Data were analysed through inductive thematic analysis.

**Results**: Most PLHIV reported having a religious or spiritual affiliation with varying degrees of practice. Participants report that their faith communities are not directly involved with HIV/AIDS treatment, support, or resource provision. Instead, participants turn to religion to cope with their illness through practice (prayer, reflection) and support‐seeking. Our analysis revealed that those who practice Abrahamic religions (Islam, Christianity, Catholicism) are more inclined to seek support from their community members, while those of non‐Abrahamic faiths (Buddhism, Taoism, Hinduism) seek spiritual support from their practice. PLHIV from both groups are reluctant to disclose their HIV‐status to members from their faiths, not because their religions, religious leaders, or fellow practitioners condemn HIV/AIDS, but because of existing prejudices against homosexuality, promiscuity, and infidelity in their religions. This indicates that cultural stigmas are being reproduced in religious groups in Singapore, as well as conflated with HIV/AIDS by our religious participants.

**Conclusions**: This paper delineates the motivations of religious/spiritual practice in PLHIV. Understanding the differences between Abrahamic and non‐Abrahamic faiths is paramount to conceiving of religion or spirituality as a resource for treatment/support in clinical interventions. Further, understanding how cultural stigmas are reproduced in religious settings illuminates barriers to health and social outcomes for PLHIV, including a reluctance to seek support from social groups due to a fear of discrimination.

### PESUD49 Photovoicing social ecological resilience and resources for youths living with HIV/AIDS in Western Uganda: towards empowering representations

E. Kimera ^1,2^, S. Vindevogel^2^

^1^Mountains of the Moon University, Public Health, Fort Portal, Uganda, ^2^University of Applied Sciences and Arts Gent (HOGENT), Social Educational Carework & EQUALITY Research Collective, Gent, Belgium

**Background**: The adversities and challenges faced by youths living with HIV/AIDS (YLWHA) are manifold, but their disproportional documentation results in overly disempowering representations of YLWHA as weak, suffering and vulnerable. What enables resilience has received subpar attention. This study sought to explore YLWHA's perceptions and representations of social ecological resources that drive resilience. Through stimulating self‐representation, the study further pursued critical consciousness and affirmation of voice and agency to challenge dominant disempowering representations and social injustices around YLWHA.

**Methods**: The study was conducted at a regional referral hospital in Kabarole district, western Uganda. Eleven YLWHA, aged 14–21 years, were recruited from an existing peer support group in the hospital. Drawing on photovoice methodology, seven consecutive sessions were organized to guide participants in producing and discussing their photos and associated narratives. These were subjected to preliminary inductive content analysis by participants, and then analyzed and interpreted by the researchers against the theoretical framework of social ecological resilience.

**Results**: Through their photos and narratives, participants portray tensions in resilience and document an array of multisystemic resources that are both informal and institutionalized. Yet, these resources appear rather incidental and not structurally available to YLWHA. Six overarching themes emerged: (1) availability of structural provisions, (2) personal and collective senses of purpose, (3) self‐appraisal of strengths, (4) achieving what society does not expect of them, (5) expression of appreciation for supportive relationships, and (6) being of value to others and getting recognition.

**Conclusions**: The representations shared by participants challenge the trope of the weak, suffering, and vulnerable YLWHA and contribute to an understanding of the multisystemic resources that foster resilience and empower YLWHA. Yet, the study also shows that resources need to become more structurally available for these youths.

### PESUD50 Frameworks and Measures for HIV‐Related Internalized Stigma, Stigma and Discrimination in Healthcare and in Laws and Policies: A Systematic Review

L. Ferguson ^1^, S. Gruskin^1^, M. Bolshakova^2^, S. Yagyu^2^, N. Fu^3^, N. Cabrera^2^, M. Rozelle^2^, K. Kasoka^4^, T. Oraro‐Lawrence^4^, L. Stackpool‐Moore^5^, A. Motala^2^, S. Hempel^2^

^1^University of Southern California, Institute on Inequalities in Global Health, Los Angeles, United States, ^2^University of Southern California, Southern California Evidence‐based Practice Center, Los Angeles, United States, ^3^Shanghai University of Finance and Economics, School of Economics, Shanghai, China, ^4^International AIDS Society, Geneva, Switzerland, ^5^Watipa, Sydney, Australia

**Background**: While global progress towards eliminating HIV‐related stigma and discrimination remains of key importance, there is wide variation in efforts to measure and assess existing interventions.

**Figure:**
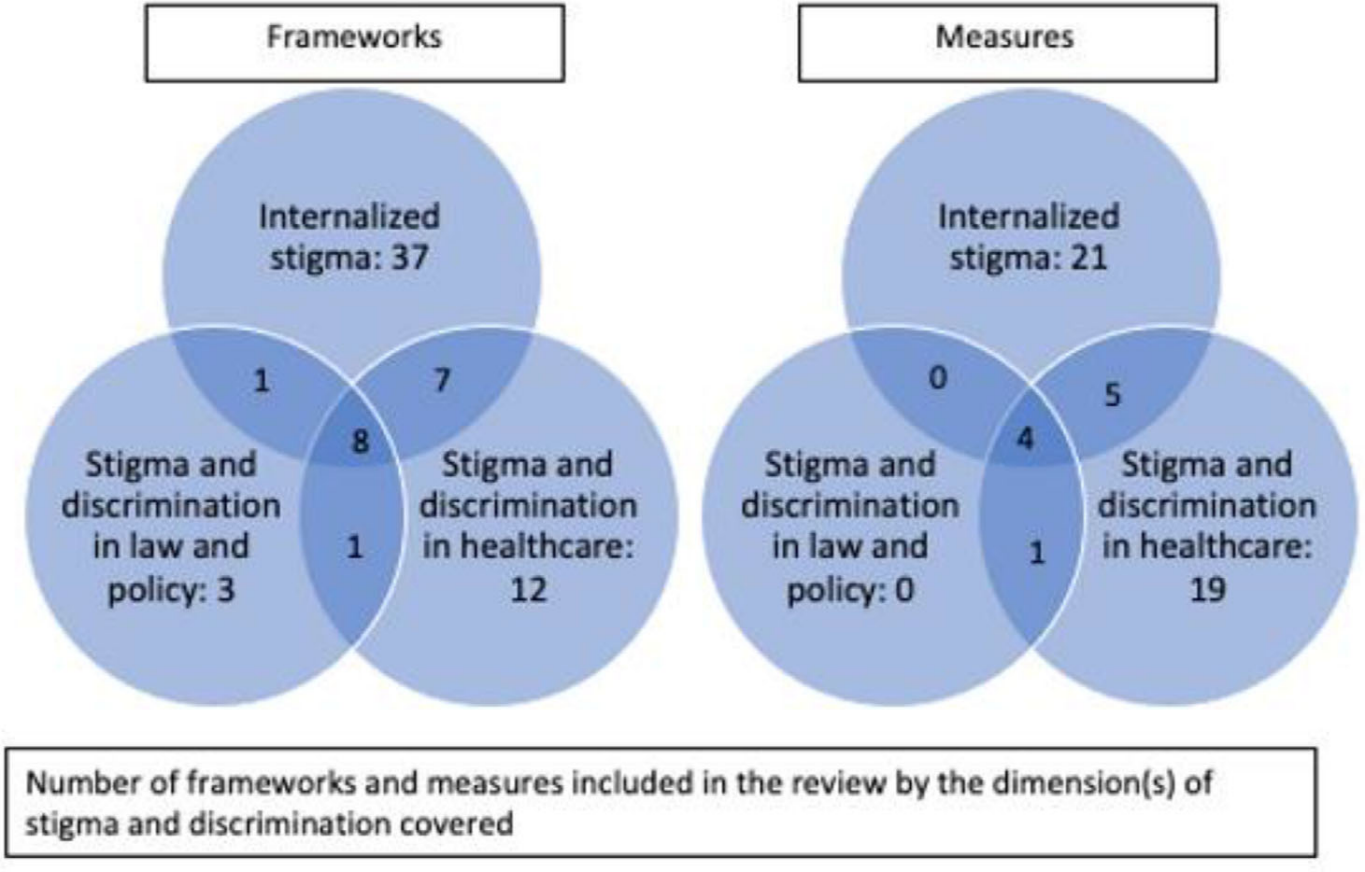
**Abstract PESUD50‐Figure 1**.

**Methods**: Given this variability, we carried out a systematic review to address two questions: What conceptual frameworks exist to assess internalized stigma, stigma and discrimination experienced in healthcare settings, and stigma and discrimination entrenched in national laws and policies? Which measures of different types of stigma and discrimination have been proposed and what are their descriptive properties? Searches, completed on 05/06/21, cover publications from 2008 onwards. The review is registered in PROSPERO (CRD42021249348), the protocol incorporated stakeholder input, and the data are available in the Systematic Review Data Repository.

**Results**: Sixty‐nine frameworks and fifty measures met the inclusion criteria. While frameworks generally seek to highlight the complex web of factors affecting different types of stigma and their impacts, their scope varies tremendously as do outcomes of interest. Measures are equally diverse with substantial differences even between those purporting to measure the same dimension of stigma. Overall, there was disproportionately little attention to stigma and discrimination in laws and policies.

**Conclusions**: To achieve global targets will require rigorous measurement of stigma and discrimination across different spheres, including those studied here. The challenge remains how to do this with frameworks and measures that are both locally appropriate and globally comparable. Frameworks and measures must be fit to help direct investment, prioritize appropriate actions, and strengthen learning about effectiveness. Most importantly, the goal must be to understand, measure and help mitigate and alleviate the impact of different types of stigma on people's health and quality of life. This review provides a basis to seek consensus about appropriate concepts and measures to understand the experiences and drivers of stigma for different people in diverse contexts around the world.

### PESUE17 Triangulating orphans and vulnerable children program and health facility data to improve HIV treatment outcomes for children and adolescents living with HIV

B. Sousa ^1^, H. Bryant^1^, T. Medrano^2^, S. Chiale^3^

^1^FHI 360, Programs, Maputo, Mozambique, ^2^FHI 360, Programs, Durham, United States, ^3^CARE, Programs, Inhambane, Mozambique

**Background**: COVida (2016‒2022) is a PEPFAR/USAID‐funded orphans and vulnerable children (OVC) project implemented in 30 districts in seven provinces in Mozambique by FHI 360 and local partners. A project priority is to help children and adolescents living with HIV (CALHIV) achieve viral load (VL) suppression, but lack of accurate data on their treatment status prevented COVida from providing appropriate support at the community level. To address this challenge, COVIDA introduced a data‐triangulation approach in October 2019.

**Description**: The data‐triangulation approach compares self‐reported OVC program data with data in the health facility (HF) patient information system to identify data gaps and discrepancies and inform corrective actions. This approach was piloted during October–December 2019 in 14 HFs in five districts in Inhambane province in collaboration with the PEPFAR HIV clinical partner.

**Lessons learned**: Of the1,555 CALHIV self‐reported as on ART in OVC program data, HFs confirmed only 1,473 as on ART. Among those confirmed, only 767 (52%) had VL data, of whom only 462 (60%) were virally suppressed. These results led to improvement actions such as finding defaulters, referring CALHIV to VL testing, and providing enhanced adherence counseling to those with high VL loads. In September 2020, the number of CALHIV on ART had increased to 1,647, those with known VL to 1,183, and those virally suppressed to 552, a 12%, 54%, and 19% increase, respectively. Given these results, data triangulation was scaled up to all 203 HFs in COVida's program sites during the remainder of 2020.

Data triangulation has contributed to improve treatment outcomes among CALHIV in all project sites. The project's overall VL coverage rate increased from 50% in 2020 to 91% in 2021, and VL suppression from 61% to 82%.

**Conclusions/Next steps**: Data triangulation helps OVC and clinical partners work together to improve treatment outcomes for CALHIV. It also allows OVC programs to access real‐time clinical data to meet the needs of CALHIV at the community level. This approach is critical to reduce the pediatric and adolescent HIV treatment gap and should be used by all OVC and HIV clinical partners working in the same geographic areas.

### PESUE18 Take home dose of Methadone: new arena for OST adherence during COVID‐19 in Bangladesh

M.S. Farid ^1^, D.L. Rahman^2^, D.S. Islam^1^, E.I. Chowdhury^1^

^1^Save the Children, HIV/AIDS Program, Dhaka, Bangladesh, ^2^Save the Children, Health, Nutrition and HIV/AIDS, Dhaka, Bangladesh

**Background**: Bangladesh has an estimated 33,067 people who inject drugs (PWID) who are the major drive for HIV among the Key population (KP). In 2015–16, HIV prevalence in Dhaka among the male PWID was 22% and among female PWID was 5%. The Global Fund HIV project in Bangladesh, implemented by Save the Children, targets high impact & cost‐effective interventions towards 14,035 PWID through 35 centers in 13 districts that was 60% of the national target (23,371).

**Description**: This project offers differentiated HIV prevention and treatment services to PWID in the 13 districts, contextualized by epidemiological and other factors. Save the Children provides Oral Substitution Therapy (OST) to 2,351 PWIDs in 2021, that was 2,013 in 2020 and 1,257 in 2019. After the outbreak of COVID‐19, OST could not be dispensed from some centers due to restricted movement and lockdowns. Many centers also were forced to close. Some PWIDs were shifted to other nearby centers temporarily for OST, and also to the Central Drug Addiction Treatment Center (CTC), Tejgaon. In addition to the Directly Observed Therapy, a take‐home dose (THD) of OST for 3–10 days was introduced to the stable and home‐based PWID upon getting approval from DNC. PWID Network members were engaged as volunteers to dispense OST, manage crowds, maintain social distance, and ensure adherence. The proportion of THD in any given location varied with the intensity of lockdown requirements. THD was highest (90%) in April‐June 2020 when lockdown was most strict, and gradually dropped to 64% in October 2020 when lockdown became relaxed.

**Lessons learned**: During July‐ Dec 2019, the retention rate of OST was 68.1%. In initial months of COVID‐19, OST intake reduced to 62–63% due to restriction. After taking different approaches and THD, OST intake increased and the OST retention rate increased to 72.9% during Jan‐Jun 2020; 82.73% in Jul‐Dec 2020; and 87.33% during Jan‐March 2021.

**Conclusions/Next steps**: Use of THD as an option for OST and use of outreach workers for follow up, had a positive impact on adherence of OST. THD need to continue as alternative options in reasonable scale for better compliance of PWID when COVID will no longer exist.

### PESUE19 What are the 12‐month retention and viral suppression outcomes for South African ART clients enrolled in DSD models compared to conventional care?

A.N. Huber ^1^, N. Jinga^1^, L. Jamieson^1,2^, B. Nichols^2,3^, S. Rosen^1,3^, S. Pascoe^1^

^1^Health Economics and Epidemiology Research Office, Department of Internal Medicine, School of Clinical Medicine, Faculty of Health Sciences, University of the Witwatersrand, Johannesburg, South Africa, ^2^Amsterdam University Medical Center, Department of Medical Microbiology, Amsterdam, Netherlands, the, ^3^Boston University School of Public Health, Department of Global Health, Boston, United States

**Background**: South Africa has implemented several differentiated service delivery (DSD) models for HIV treatment. Few comparisons of treatment outcomes between the country's DSD models and conventional care are available. We analyzed routine data to determine one‐year retention and viral suppression of clients enrolled in DSD models.

**Figure:**
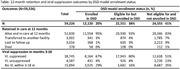
**Abstract PESUE19‐Table 1**.

**Methods**: We analyzed individual data from South Africa's electronic patient record (TIER.Net) for 24 clinics across 4 districts. We followed clients alive and in care on 01/02/2019 and estimated outcomes of retained at 12 months after follow‐up start and virally suppressed (<400 copies/ml^3^) ≥3–18 months. We classified clients as eligible for DSD models if they were ≥18 years old, on ART ≥12 months and had two suppressed viral load (VL) measurements, per national guidelines at the time. We compared outcomes for those enrolled in a DSD model to those eligible but not enrolled and for those ineligible, compared outcomes by reason ineligible for DSD.

**Results**: Among 12,120 clients enrolled in DSD and 22,551 ART clients eligible but not enrolled in DSD, retention was 95% and 93%, respectively (risk ratio [95% confidence interval] 1.02[1.02–1.03])(Table). Viral suppression for those with a VL measure was 95% for both groups, but 29% of those in DSD models and 16% in conventional care had no VL measurement recorded. Of the 3,298 recently enrolled into a DSD model (≤6 months), 35% (n = 1,153) did not meet the eligibility criteria (0.5% <18yrs, 3% on ART <12 months, 99% missing two suppressed VLs). Retention and VL suppression were higher for those with a known suppressed VL prior to DSD enrollment (93%, n = 498) than for those with a known unsuppressed VL prior to DSD enrolment (87%, n = 46).

**Conclusions**: DSD model enrolment conferred a minor benefit to retention and equivalent viral suppression over one year of follow‐up compared to conventional care for clients eligible for DSD enrolment.

### PESUE20 The effect of multi‐month dispensing of ART on viral load suppression rates in 18 PEPFAR‐supported countries

L. Bailey ^1^, T. Essam^1^, L. Lee^1^, P. Agaba^2,3^, I. Zulu^4^, G. Alemnji^5^, D. Patel^1^, C. Godfrey^5^

^1^U.S. Agency for International Development, Washington, DC, United States, ^2^U.S. Military HIV Research Program, Walter Reed Army Institute of Research, Silver Spring, United States, ^3^Henry M. Jackson Foundation for the Advancement of Military Medicine, Bethesda, United States, ^4^U.S. Centers for Disease Control and Prevention, Atlanta, United States, ^5^U.S. Department of State, Office of the Global AIDS Coordinator, Washington, DC, United States

**Background**: Research studies demonstrate that people living with HIV (PLHIV) receiving multi‐month dispensing (MMD) of antiretroviral therapy (ART) can maintain high rates of viral load suppression (VLS), but little is known about the programmatic effect of MMD on VLS rates rolled out at scale in global HIV programs. The President's Emergency Plan for AIDS Relief (PEPFAR) recommends three to six‐month MMD of ART and collects data on ART dispensing and viral load testing at PEPFAR‐supported ART sites. This multi‐country analysis aims to describe the effect of MMD scale‐up on VLS rates across multiple countries under real world conditions.

**Figure:**
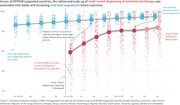
**Abstract PESUE20‐Figure 1**.

**Methods**: We analyzed quarterly PEPFAR program data from Oct 2018 to Sept 2021 in 18 PEPFAR‐supported countries with an average MMD reporting completeness rate > 80%. We compared the proportion of ART patients receiving at least three‐month dispensing of ART to VLS rates (proportion of viral load results <1,000 copies/ml) using descriptive statistics, correlations, and a parsimonious fixed‐effects regression model. The analysis incorporated a six‐month lag between quarterly MMD coverage and VLS data points to account for time between MMD initiation and subsequent VL testing.

**Results**: In the 18 countries and 1,472 subnational units analyzed, the scale‐up of MMD was moderately positively correlated [r = 0.275] with improved viral load suppression. A fixed‐effect regression on VLS and MMD using robust standard errors, suggests a positive correlation between lagged MMD and VLS (beta = 0.032; SE = 0.017, p‐value = 0.087, CI = −0.006 to 0.069). Graphical summaries of the data show similar results and point towards a narrowing of the distribution of VLS outcomes with time [Figure1].

**Conclusions**: Large HIV programs have successfully scaled‐up MMD to patients while sustaining and increasing VLS rates in real‐world settings, supporting data from clinical trials. Limitations include the use of aggregate program data rather than patient‐level data.

### PESUE21 Domestic public spending in low‐and‐middle‐income countries 2006–2020: levels and trends

D. Mattur R ^1^, S. Javadekar^2^

^1^UNAIDS, Geneva, Switzerland, ^2^University of Bath, Bath, United Kingdom

**Background**: Domestic public investments in HIV/AIDS have been the main driver of increase in HIV resources in LMICs during the last decade. For the first time, there is a sign of flattening for last two years and a 2% annual decrease of domestic resources in 2020. Understanding the correlates and predictors of domestic public spending on HIV can inform sustainability plans.

**Methods**: A panel data regression using reports to UNAIDS for 2006–2020 from 114 low‐and‐middle‐income countries was used for the analysis resulting in 1452 country year data points. All the regression estimates are based on panel data random effects models.

**Results**: There are significant positive associations between the Gross Domestic Product (GDP) per capita (co‐efficient 0.2%, p value <0.01) of a country and its level of domestic public spending on HIV. The HIV Prevalence (8.61, <0.001), the non‐GDP residuals of the Human Development Index (HDI) (0.25, <0.001) and share of health in total government expenditures (0.05, <0.01) were also significant predictors. No significant effect was found for ODA for HIV or other independent variables. The domestic public resources per person living with HIV in 2020 were estimated at US$174 in East and Southern Africa, US$36 in West and Central Africa, US$369 in Asia and the Pacific, US$196 in the Caribbean, US$859 in Eastern Europe and Central Asia, US$1699 in Latin America, and US$320 in Middle East and North Africa. Domestic public spending has increased 70% between 2010 and 2020. In 2020, domestic public resources constituted 50.4 % of all resources in LMICs. [1] There are large differences in donor dependency across geographies, for example while 98% of AIDS resources in Latin America come from domestic resources, they constitute 35% and 31% in West Central Africa and the Caribbean respectively.

[1] UNAIDS Global report, 2021

**Conclusions**: The main determinants of domestic public spending for HIV are ability to pay (GDP percapita), burden of disease (HIV prevalence), non‐GDP residuals of HDI and the national prioritization of health within government spending. With the observed flat lining of international resources, sustained and efficient domestic public spending will be key in achieving resource mobilization targets set by 2021 Political Declaration.

### PESUE22 Stakeholders’ perspectives on the financial sustainability of HIV response in Nigeria

D. Ogbuabor ^1^, O. Onwujekwe^1^

^1^University of Nigeria, Health Administration and Management, Enugu, Nigeria

**Background**: Transitioning from donors to government requires an understanding of the contextual factors shaping financial sustainability in low‐resource settings. As this evidence is scarce in Nigeria, we assessed the perspectives of HIV response stakeholders to understand how domestic funds can be mobilised, pooled, and strategically used to pay for HIV services.

**Methods**: The study adopted the framework of health financing functions including revenue mobilization, pooling, and purchasing. We conducted document reviews and semi‐structured interviews with stakeholders at national and sub‐national levels (n = 32) between December 2021 and January 2022. We adopted maximum variation sampling to purposively select individuals whose roles included financing in the HIV response. Data were analysed thematically using NVivo software (version 11).

**Results**: Public spending is low nationally and sub‐nationally due to low resource allocation and low budget execution. Few state governments implemented the policy earmarking 0.5–1% of states’ federal allocation to the HIV response. Decision‐makers and budgeting staff perceive the HIV response as getting substantial external assistance. Although private sector investment is low, the establishment of an HIV trust fund might increase its contribution to the HIV response. On pooling and fund management, appropriations are need‐based, but releases do not reflect needs. In contrast, external assistance reflects geographic variations in the HIV burden. Notwithstanding a national strategy for integrating HIV into social health insurance schemes, HIV services have not been prioritised by the schemes. Coverage of some HIV services in the Basic Health Care Provision Fund has not translated into practice. Users pay for some HIV services previously supported by donors. Regarding purchasing, a parallel procurement system between donors and government, and high supply‐side spending undermine the financial sustainability of the HIV response. Purchasing of services for the key populations is limited by a lack of reliable estimates due to demographic shifts and stigma. Dysfunctional inter‐agency relationships hinder scaling up HIV testing and treatment in primary health facilities despite its efficiency gains. Civil society organisations can be financed through partnerships with the government.

**Conclusions**: This study highlights the financing and governance factors that can inform the development of a financial sustainability plan for the HIV programme in Nigeria.

### PESUE23 Do differentiated models of care for HIV treatment result in lower costs for recipients of care in Zambia?

C. Hendrickson ^1,2^, B. Phiri^3^, N. Lekodeba^1^, I. Mokhele^1^, A. Huber^1^, V. Ntjikelane^1^, P. Haimbe^3^, H. Shakwelele^3^, P. Mulenga^4^, B. Nichols^1,2,5^, S. Pascoe^1^, S. Rosen^1,5^

^1^Health Economics and Epidemiology Research Office, Department of Internal Medicine, School of Clinical Medicine, Faculty of Health Sciences, University of the Witwatersrand, Johannesburg, South Africa, ^2^Amsterdam University Medical Center, Department of Medical Microbiology, Amsterdam, Netherlands, the, ^3^Clinton Health Access Initiative, Lusaka, Zambia, ^4^Ministry of Health, Lusaka, Zambia, ^5^Boston University School of Public Health, Department of Global Health, Boston, United States

**Background**: One of the benefits that differentiated service delivery (DSD) models for HIV treatment are assumed to generate is a reduction in direct and indirect costs to recipients of care (RoC), but the savings that come with reduced costs must vary among the widely diverse DSD models. We estimated the costs to RoC of nine discrete models currently in routine use in Zambia, compared to conventional care.

**Methods**: From May to November 2021 we surveyed RoC at 12 clinics in two provinces of Zambia. Participants were selected consecutively on their arrival for routine visits, with stratification by DSD model participation, and asked about time spent and transport costs incurred when accessing care. We calculated the cost/health system interaction (clinic and out‐of‐facility) and multiplied by the participant‐reported number of interactions per year to estimate an opportunity cost (using the country‐specific minimum wage of $1.99/day) and transport cost/RoC/year by model of care.

**Results**: We surveyed 558 RoC (median age 38, 72% female). Conventional care required four facility visits year, while most (but not all) DSD models reduced facility visits to two per year, with or without additional external interactions such as adherence club meetings or community medication pickups (Figure). Depending on the model, opportunity costs to RoC ranged from roughly 1 to 3 days’ minimum wage. Fewer than half of RoC incurred any transport costs; for those who did, the cost averaged 1–1.5 days’ minimum wage. Variation in transport costs among models may reflect RoC choices about paying for transport based on how many interactions will be required and the locations of the interactions.

**Figure:**
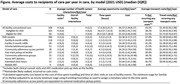
**Abstract PESUE23‐Figure 1**.

**Conclusions**: DSD models generally minimise costs and time for RoC as compared to conventional care, but this depends entirely on model design (number of interactions required/year). Implementing models that minimize RoC interactions with the healthcare system and model events may improve outcomes.

### PESUE24 PLHIV refill booklet: an innovative way to reduce interruption in treatment among highly mobile PLHIV in Ghana

A. Dambasea ^1^, M. Owusu^1^, S. Wosornu^1^, E. Adiibokah^2^, E. Owusu^1^, H. Tagoe^3^

^1^Maritime Life Precious Foundation, Sekondi‐Takoradi, Ghana, ^2^JSI Research & Training Institute, Inc., Takoradi, Ghana, ^3^JSI Research & Training Institute, Inc, Takoradi, Ghana

**Background**: PLHIV who experience interrupted HIV care usually have poor clinical outcomes, and they also contribute to further HIV transmission. HIV‐related mortality and morbidity significantly increases when there is interruption in treatment (IIT). Follow‐up calls made to PLHIV who interrupted treatment revealed that high mobility from the nature of their jobs or circumstances was a major casual factor. Such clients find it difficult to get their refill when they are due. In October 2019, Maritime Life Precious Foundation (MLPF) a key population focus Civil society organization (CSO), collaborated with Tarkwa Municipal Hospital (TMH) in Western Region, Ghana to find an innovative way to mitigate this situation. We deployed a portable refill booklet system that is easy to use, convenient and ensures that refills are documented.

**Description**: In October 2019, MLPF and the ART team designed a pocket‐sized booklet that enables PLHIV to conveniently get ARV refill from other ART sites. The phone numbers of the ART nurses and data officers were printed in the booklet. 85% of all clients diagnosed HIV‐positive (316 PLHIV) at the Tarkwa Municipal Government Hospital accepted the booklet. 68% (214) of them were female whereas 32% (102) were male. Health facility data officers documented refills on the e‐tracker network.

**Lessons learned**: 91% (212) of all newly diagnosed PLHIV accepted the booklet. After 24 months, 48% (102) of the clients who accepted the booklet visited TMH for their refills and so, did not use their booklet, while 52% (110) used their booklets successfully. 65% (72) of the PLHIV who used the booklet got refills from facilities outside the municipality and 35% (38) got refills from outside the Western region. The booklet was helpful to PLHIV might interrupt treatment as a result of their high mobility. It also reduced the need for client transfers in‐between ART facilities. Data officers verified refill on the e‐tracker network. Clients experienced difficulties ART sites that did not have prior information about the pilot.

**Conclusions/Next steps**: The PLHIV refill booklet is effective and has the potential to improve ART retention and records keeping. Therefore it is recommended that this refill booklet system be supported and scaled up nationwide.

### PESUE25 Optimal treatment and prevention mix for South Africa's UTT program

S. Nyamhuno ^1^

^1^Ashley Nyamhuno, Durban North, South Africa

**Background**: South Africa has over 8 million people living with HIV/AIDS. In 2016, it adopted the UTT program in a bid to control, the epidemic. There is room to fine‐tune the current UTT program to get high impact, less costly programs that give the country a great value for its money.

**Methods**: We used Goals Model which is a dynamic compartmental model to project the results for the period, 2020–2030 for South Africa's entire population, including PLWHA. We fitted the epidemic model to South Africa's epidemic using current data. For the program components under consideration (PrEP coverage, ART coverage and retention on care), we subjected them to a 3% discount for costs and infections averted. We further used a scenario analysis in which we varied the program components for different coverage levels to assess each impact on the HIV investment case for South Africa.

**Results**: Increased ART coverage is more cost‐effective than any option with less coverage. The 95% scenario yielded better health benefits than both90% and the current coverage (72%). It records fewer AIDS deaths, gain more life years and averts more new HIV infections than the scenarios with less coverage. It also has an ICER of R13,111/QALY gained which was cost‐effective. A lower migration rate was much beneficial to South Africa. It showed the least amount of deaths recorded, lower costs, the highest number of new HIV infections averted than all scenarios with higher migration rates. It further had the lowest ICER which proved that it was the most cost‐effective option. Increased PrEP coverage is associated with more health benefits however at a higher cost at a high cost. However, the PrEP average to all‐risk categories was quite phenomenally high and unsustainable. PrEP coverage was highly beneficial when given to high‐risk populations.

**Conclusions**: The best investment option for South Africa is high ART coverage, low migration to second regimens as well as PrEP average to high‐risk groups. With this, South Africa can save costs and redirect savings to other equally important areas. UTT costs are manly driven by ART costs. South Africa must consider local production of drugs to reduce imported inflation costs.

### PESUE26 Mapping opportunities for CSO financial sustainability through domestic funding access to ensure the continuation of HIV community‐led response in Indonesia

A. Nugroho ^1^, M. Lubis^2^, Y. Nurhidayat^2^, S. Sarwitri^2^, M. Maryono^2^, F. Riyadi^2^

^1^UNAIDS Indonesia, Jakarta, Indonesia, ^2^Konsil LSM, Jakarta, Indonesia

**Background**: To support the sustainability of HIV community‐led response, Indonesia has launched a pilot project with the support of the Global Fund to strengthen the sustainability of HIV‐CSO financing in ten cities through capacity building for CSOs and local governments in developing social contracting initiatives. The objectives of this mapping were (1) to evaluate the legality and competence of HIV‐CSOs engaged in lobbying, advocacy, and social contracting in ten cities; and (2) to identify policy and financial opportunities for HIV‐CSOs at the national level and in ten pilot cities.

**Description**: This mapping applied the civic and fiscal space analysis approach and involved 84 HIV‐CSOs and 35 local government officials as respondents/participants. The civic space analysis employed both quantitative and qualitative methods and gathered data via literature reviews, online surveys, in‐depth interviews, and FGDs. While the fiscal space approach gathered data through literature reviews and examination of relevant government papers.

**Lessons learned**: Local governments continue to be unsure about how to interpret the division of responsibilities in dealing with HIV‐AIDS. Fiscal capacity is not a big driver, but commitment is. Accountability of HIV CSOs, particularly upward accountability, remains a challenge in comparison to internal and downward accountability. 26 organizations met the eligibility criteria for social contracting. According to the Organizational Performance Index's score, 20 CSOs (51%) are in a nascent state, which means they are improving their effectiveness, internal efficiency, and human resource strength. And 49% are at an emergent state, which means that practice and skills at the fledgling stage have been achieved.

**Conclusions/Next steps**: General recommendations include: (1) To determine the area that will serve as the locus of advocacy for the social contract, the most critical factor to map, aside from fiscal capacity, is the commitment of local government, as well as the capacity of local CSOs. (2) Need to identify champions who come from local government leaders as primary partners in social contracting; (3) Invest in raising awareness and understanding of the HIV‐AIDS issue as part of local governments' work mandate; (4) Strengthening CSOs' organizational capacity and accountability in order to build public trust in CSOs as strategic partners, particularly among local governments.

### PESUE27 Optimizing Diagnostic Technologies for Pediatric HIV– Function or Location? Modelling analysis of Point of Care Technologies in Matabeleland South, Zimbabwe

A.K. Amick ^1^, M. Yildrim^1,2^, K.A. Webb^3,4^, A. Mushavi^5^, C. Flanagan^1^, A. Chimwaza^5^, N.C. McCann^1^, T. Murape^3^, A. Claypool^1^, K.A. Freedberg^1,6^, A. Ciaranello^1,1,6^, M.S. Jalali^2^

^1^Medical Practice Evaluation Center, Massachusetts General Hospital, Harvard Medical School, Boston, MA, United States, ^2^Institute for Technology Assessment, Massachusetts General Hospital, Harvard Medical School, Boston, MA, United States, ^3^Organization for Public Health Interventions and Development, Harare, Zimbabwe, ^4^London School of Hygiene and Tropical Medicine, Epidemiology and Population Health, London, United Kingdom, ^5^Ministry of Health and Child Care, National PMTCT Program, Harare, Zimbabwe, ^6^Division of Infectious Diseases, Massachusetts General Hospital, Harvard Medical School, Boston, MA, United States

**Background**: Novel point‐of‐care (POC) devices for infant HIV testing provide prompt receipt of results and increase ART initiation, improving survival among HIV‐exposed infants with HIV. POC device functionality (proportion of days devices are operational) varies with power supply, machine maintenance and testing commodities supply. Program planners must decide in which health facilities to locate a limited supply of POC devices.

**Figure:**
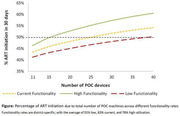
**Abstract PESUE27‐Figure 1**.

**Methods**: We developed a location‐optimization model to identify the placement of 11 currently available POC devices in Matabeleland South Province, Zimbabwe, that would maximize the number of infants with HIV initiating ART within 30 days of testing. We first examined the current and optimal placement of the currently available devices, then determined the number of new POC machines that would need to be added and optimally located to achieve 50% 30‐day ART initiation. We modelled 4724 infants who received HIV testing from Jan2019‐Jan2020 using routine program data from 122 health facilities.

**Results**: With current placement of 11 existing POC machines, 37% of all tested infants with HIV would receive their results and 35% would initiate ART within 30 days. With optimal placement of existing machines, 46% would receive their HIV test results and 44% would initiate ART within 30 days; retaining 2 machines in their current locations and moving 9 machines to new facilities. Requiring >/ = 1 machine/district reduced 30‐day ART initiation to 42%. The number of optimally placed POC devices required to achieve 50% 30‐day ART initiation depended on device functionality: 38 devices would be needed with low (51%) functionality, 25 with current (63%) functionality, and 15 with high (75%) functionality.

**Conclusions**: We demonstrate substantial increases in 30‐day result return and ART initiation among infants with HIV through optimization‐based location of available POC machines for infant testing. The benefits of optimal location and/or adding new POC machines are dramatically influenced by machine functionality.

### PESUE28 Digital Interventions through an online community portal to engage historically hard‐to‐reach populations of same‐sex attracted men living in a heterosexual relationship or lifestyle

C. Hawk ^1^, S. Ruth^2^, C. Batrouney^1^

^1^Thorne Harbour Health, Health Promotion, Policy, & Communications, Melbourne, Australia, ^2^Thorne Harbour Health, Melbourne, Australia

**Background**: Same‐sex attracted men living in a heterosexual relationship or lifestyle (often referred to ‘gay and married men’) have been historically difficult to connect with HIV prevention and sexual health education, peer support, and sexual health services due to a range of factors including structural, social, and self stigma associated with their same‐sex attraction. With limited success achieved through previous face‐to‐face interventions, this multidisciplinary project sought to realise the potential of digital interventions in engaging this population in Australia.

**Description**: Originally a two‐year research partnership, the development of the DALE project was informed by preliminary qualitative interviews with same‐sex attracted men living in a heterosexual relationship or lifestyle to develop a digital wireframe for the delivery of a dual education and contact approach through an online community. The community website included a feed of articles integrated with health promotion messaging alongside peer‐moderated discussion boards and live chat sessions. The website also connected site visitors with an online survey about their mental health, sexual behaviours, sexual health, disclosure of same‐sex attraction, substance use, and experiences of stigma.

**Lessons learned**: The online community at DALE has had more than 20,000 visitors and connected with men who have sex with other men identify as identified as gay, bisexual, and heterosexual. These men have reported to experience greater levels of anxiety, depression, stress, and stigma. Survey participants also showed higher rates of condomless anal intercourse with casual and regular partners alongside lower rates of testing for HIV and other sexually transmitted infections. As the time of the original study period's completion, the findings from the survey combined with the quantitative data from site analytics represented the largest quantitative research to be conducted among this cohort of men.

**Conclusions/Next steps**: The original research partnership developed an online intervention to address compound forms of stigma and the resulting online community created a valuable connection with a historically hard‐to‐reach population of men who have sex with other men to disseminate health promotion messaging, peer support, and referrals to additional resources. The developed digital wireframe also has the potential to be adapted for other hard‐to‐reach communities.

### PESUE29 Finding local models for comprehensive HIV/AIDS management in closed settings: Documenting the experience of the Bureau of Jail Management and Penology (BJMP) in Cebu City, Philippines

M.J. Villaester^1^, E. Bagasol ^1^, J.O. Corciega^1^, R. Rizarri^2^, M. Encarnacion^3^, I. Tac‐an^3^, F. Rubio^4^, V.P. Baton^4^, M.L. Averilla^5^

^1^LoveYourself Inc., Mandaluyong, Philippines, the, ^2^Bureau of Jail Management and Penology, Cebu City, Philippines, the, ^3^Social Hygiene Clinic, Cebu City Health Department, Cebu City, Philippines, the, ^4^Department of Health, Central Visayas Center for Health Development, Cebu City, Philippines, the, ^5^Australian Federation of AIDS Organisations, Bangkok, Thailand

**Background**: HIV prevalence in Philippine jails, compounded by congestion and inadequate health services, pose challenges in treating PLHIV and preventing transmission among persons deprived of liberty (PDL). Acknowledging the public health imperative and opportunity to address HIV‐related risks and deaths among PDL, BJMP issued a memorandum circular (MC) to guide jails in establishing HIV/AIDS programs.

**Methods**: LoveYourself, through the Global Fund SKPA program, helped document and analyze Cebu City Jail's HIV/AIDS program implementation to provide a model for other jails in the country to operationalize the BJMP MC. The process documentation involved policy reviews, stakeholder meetings, and key informant interviews with jail personnel, health officials, and select PDL.

Interventions and challenges throughout the PDL confinement stages (commitment, detention, transfer and release), as well as opportunities to extend HIV interventions for PDL's family members were explored.

**Results**: Despite PDL's access to condoms, knowledge retention on safer sex practices and HIV/STI prevention remains a challenge. Voluntary testing, offered upon commitment and during detention, is hampered by peer‐driven stigma. PDL who test reactive are immediately enrolled to treatment assisted by peer health aides. Health aide accounts, however, reveal PLHIV hesitancy in accessing/maintaining treatment caused by fear of ARV side effects, ARV access issues, and perceived effects of family disclosure. PLHIV‐PDL's continued access to treatment, care and support, following transfer or release, is also unsecured due to the lack of standardized procedure for referral and monitoring.

Local government social hygiene clinic and community‐based organization collaborations help address these gaps, including through periodic mass HIV screening, conduct of PDL/PLHIV learning group sessions, treatment monitoring, and ARV refill.

**Conclusions**: Documenting Cebu City Jail's experience surfaced gaps on the BJMP MC and how it can be operationalized at facility level. The study recommends addressing jails’ health human resource limitations; mobilizing and enhancing health‐related capacity of PDL aides and jail personnel; improving access of PDL and family members to HIV information and prevention commodities; better documenting PDL/PLHIV learning group sessions to inform service enhancements; firming‐up case monitoring, reporting and referral mechanisms; integrating mental health interventions; and ensuring better access to food, water and medicine in‐facility.

### PESUE30 Expedited development and registration resulting in successful uptake of generic, pediatric dolutegravir products for low‐ and middle‐income countries through an innovative public‐private partnership

M. Watkins^1^, H. McDowell^2^, L. Lewis^1^, K. Hayward^1,2^, M. Louey ^1^, A. Djemai^2^, S. Ghelani^1^, K. Grainger^2^, C. Amole^1^

^1^Clinton Health Access Initiative (CHAI), Boston, United States, ^2^ViiV Healthcare UK Ltd, London, United Kingdom

**Background**: There are 1.7M children living with HIV (CLHIV) globally, with only 54% on treatment and 100,000 deaths each year. Poor viral suppression is observed in CLHIV due to lack of accessible, effective child‐friendly formulations. A significant contributor is the time delay between adult and pediatric formulation development, followed by generic development via voluntary licensing mechanisms. Dolutegravir (DTG) was originally approved in 2013 by the US FDA for HIV treatment in adults and children (≥12 years, ≥40kg). In 2018, WHO guidelines recommended DTG, in combination with NRTI backbone, as the preferred first‐line treatment for adults and children. However, ViiV Healthcare's child‐friendly formulation (≥4 weeks, ≥3kg), under development, was not yet available and generic development had not yet commenced.

**Description**: A public‐private partnership, consisting of CHAI (funded by Unitaid), ViiV, Mylan and Macleods, was initiated to expedite the development and registration of generic, pediatric DTG dispersible tablets for use in low‐ and middle‐income countries (LMICs) within ViiV's pediatric licensing territory. CHAI/Unitaid provided a financial incentive for product development. ViiV provided a comprehensive technical package. CHAI and ViiV provided ongoing technical and regulatory support. A novel regulatory strategy enabled generic filing to the US FDA under the PEPFAR program during ViiV's review period. FDA tentative approvals for pediatric DTG were obtained by Mylan and Macleods within 5 and 9 months of ViiV's approval, respectively. The financial incentive enabled a global price agreement (75% price reduction from the existing standard of care).

**Lessons learned**: Technical support throughout the generic development process was critical to reducing timelines. Early engagement with the US FDA prior to generic filing was key to gain alignment on the proposed novel regulatory strategy and timing.

**Conclusions/Next steps**: The collaborative partnership between CHAI, ViiV, Mylan and Macleods significantly accelerated development and registration of generic, pediatric DTG products. The gap between innovator and generic US FDA product approvals was reduced to months. The generic products launched at an affordable and sustainable price improving pediatric patient access in LMICs.

### PESUE31 Sexual, physical, economic and emotional violence faced by women and adolescent girls living with HIV and at high risk of HIV in South Africa and Nigeria in time of COVID‐19

E. Lamontagne^1,2^, A. Yakusik ^1^, H. Humphries^3^, L. Lewis^3^, S. Choonara^3^, O. Arije^4^, K. Dikgale^3^, A. Enemo^5^, A. Matendawafa^6^, R. Mohammed Abdullah^7^, A. Muhammad^8^, D. Nyagani^9^, H. Okiwu^10^, P. Sibisi^11^, A. Sunday^12^, H. Yunusa Nyako^13^, M. Oluwatoyin Folayan^4,14^, Q. Abdool Karim^3,15^

^1^UNAIDS, Strategic Information, Geneva, Switzerland, ^2^Aix‐Marseille University, School of Economics, Marseille, France, ^3^CAPRISA, Durban, South Africa, ^4^Obafemi Awolowo University, College of Health Sciences, Ile‐Ife, Nigeria, ^5^Nigeria Sex Workers Association, Kubwa, Nigeria, ^6^African Alliance, Johannesburg, South Africa, ^7^National Association of Persons with Physical Disability, Abuja, Nigeria, ^8^Northern Nigerian Transgender Initiative, Abuja, Nigeria, ^9^Youth Health Africa, Johannesburg, South Africa, ^10^YouthRise, Abuja, Nigeria, ^11^AIDS Foundation of South Africa, Berea, South Africa, ^12^African Network of Adolescent and Young Persons Development, Barnawa, Nigeria, ^13^Jami Al Hakeem Foundation, Jimeta‐Yola, Nigeria, ^14^Nigeria Institute of Medical Research, Lagos, Nigeria, ^15^Columbia University, Department of Epidemiology, New York, United States

**Background**: The COVID‐19 crisis is associated with a global surge in reports of gender‐based violence (GBV). Little is known of its impact on adolescent girls and women living with HIV (W&GLHIV) or at high risk of HIV. This study aimed to determine the risk factors for physical, sexual, emotional and economic violence during the COVID‐19 crisis.

**Methods**: National cross‐sectional surveys were conducted among W&GLHIV or at high risk of HIV ≥15 years in South Africa and Nigeria from July to December 2021. Participants were recruited using a combination of venue‐based and snowball convenience sampling methods. The questionnaires were completed online or with assistance from a data collector for those with reading/writing impairment or in the absence of an internet‐connected device. Multivariable logistic regression was used for identifying risk factors for GBV and controlled for age, education and working status.

**Results**: A third (30%) of the 6,689 participants reported experiencing physical or sexual gender‐based violence, with 7% facing less violence, 10% facing the same level of violence and 13% facing more violence than before the COVID‐19 crisis. W&GLHIV were more likely (adjusted odds ratio (AOR) 1.27, 95%CI 1.09–1.49) to experience violence compared to those HIV‐negative. There was a monotonic relationship between violence and mental health with those experiencing violence more than twice likely to report severe symptoms of depression and anxiety (AOR 2.33, 95%CI 1.82–2.99). Violence was also strongly associated with housing insecurity (AOR 1.59, 95%CI 1.27–2.02). Participants engaging in sex work (AOR 2.74, 95%CI 1.62–4.61) or transactional sex (AOR 1.86, 95%CI 1.40–2.46) were more likely to face sexual and physical violence compared to those not engaging in transactional or sex work. Furthermore, using validated instruments, 35% of all participants reported emotional as well as economic abuse with W&GLHIV reporting higher rates of emotional (AOR 1.19, 95%CI 1.04–1.37) and economic abuse (AOR 1.19, 95%CI 1.04–1.37) respectively.

**Conclusions**: The COVID‐19 crisis resulted in an increase in already high rates of gender‐based violence faced by W&GLHIV or at risk of HIV with substantial mental health and economic consequences. COVID‐19 and HIV policy and programmes need to pay more attention to the multi‐faceted manifestations of GBV.

### PESUF13 Sex workers educating sex workers, www.plaperts.org and its online school: LXS expertxs

K. Bravo ^1^, J. Campi‐Portaluppi^2^

^1^Plaperts, Machala, Ecuador, ^2^Universidad Casa Grande, Applied Research Projects, Guayaquil, Ecuador

**Background**: Strengthen the capabilities of people who exercise sex work in order to grant them the abilities to better advocate for their access to basic rights, including access to integral health.

**Description**: Design and creation of an online learning ecosystem. Designed as a MOOC and with courses conceived through an inclusive and constructivist approach where sex workers use backwards design to create courses that help other sex workers strengthen their capabilities around advocacy, project design and human rights. As of now one course has been launched and has started to be applied in 12 countries in Latin America where Plaperts' allies are working every day to better the conditions of sex workers and their access to healthcare and other basic rights.

**Lessons learned**: Online tools have become a viable alternative in LATAM because of the exponential growth in internet penetration during the covid pandemic.

An online learning ecosystem created by sex workers for sex workers is a space that can potentially grow to become a tool that allows to better educate more women and men, cis or trans, H or LGBTQI+ in matters related to HIV, politics, human rights, gender diversity and other topics connected to the possibility of improving the conditions of the lives of people in a position of vulnerability.

The platform has helped the students but also has contributed to helping those who participated from the design learn new ideas and concepts from their peers and help them also feel more empowered because of the experience of teaching others and creating a product such as this.

**Conclusions/Next steps**: This initiative is an ongoing project that aims at the creation of 4 more courses in the next 4 years and that is looking to formally evaluate its results within the region, in order to better tune its resources to be able to achieve its goals. We believe that sharing this initiative in this space will allow for others to be motivated by our efforts and that the input obtained will also allow it to evolve and grow.

### PESUF14 Community‐based assessment on criminalization costs in the Central and Eastern Europe, and Central Asia region

E. Kurcevič ^1^, G. Dovbakh^1^

^1^Eurasian Harm Reduction Association, Vilnius, Lithuania

**Background**: Criminalization of people who use drugs in the Central and Eastern Europe, and Central Asia (CEECA) region, instead of maintenance of public health increases the financial and social burden on the states. This community‐based assessment aimed to evaluate and compare costs of incarceration versus costs of health and social services for people who use drugs; as well, to analyze how incarceration and health costs changed in two years (same assessment was conducted in 2019).

**Methods**: Assessment was done in 2021, in 29 countries of CEECA region. It was conducted in two stages: 1) desk research, and 2) verifying data with national partners, working in the harm reduction field. The cost of incarceration was calculated by multiplying 365 days to the cost of maintenance of one prisoner/per day. This sum doesn't include law enforcement work, court proceedings and lost taxes, which person cannot pay, because of the incarceration. The costs of health and social services included cost of needle and syringe exchange services, opioid substitution therapy and unemployment benefit for one person per year.

**Results**: In most of countries of CEECA region, incarceration costs are 1,5 to even 17 times more than health and social services. The biggest difference of costs is assessed in Georgia (17 times), Hungary (17 times), North Macedonia (13 times), Romania (8 times). Meanwhile the lowest difference of costs is assessed in countries, which has numerous and gross human rights violations in prisons ‐ Belarus, Bulgaria, Azerbaijan and Kyrgyzstan. In comparison with 2019, few countries made improvements in increasing unit costs for health and social support: Azerbaijan (5,5 time), Croatia (1,6 time) and Czechia (1,5 time). In some countries both ‐ costs of health and social services and incarceration increased on the equal level, in average ‐ Lithuania (1,7 time), Estonia (1,4 time), Moldova (1,1 time).

**Conclusions**: Assessment shows that there are opportunities for governments to invest into the health and social services for people who use drugs. Governments should take evidence‐based health and human rights approaches and reallocate money from policing, prosecuting, and incarceration of people who use drugs to community harm reduction and health services.

### PESUF15 Experiences with criminal justice system and HIV/Hepatitis C testing among people who inject drugs (PWID) in Selangor, Malaysia

J. Pang ^1^, W.H. Siew^1^, A.M. Jailani^2^, A. Ahmad^3^, N.A. Mohd Salleh^4,5^, A. Kamarulzaman^5,1^

^1^University of Malaya, Department of Medicine, Kuala Lumpur, Malaysia, ^2^Persatuan Insaf Murni, Kajang, Malaysia, ^3^Yale University, Department of Medicine, Section of Infectious Diseases, AIDS Program at the Yale School of Medicine, New Haven, United States, ^4^University of Malaya, Department of Social and Preventive Medicine, Faculty of Medicine, Kuala Lumpur, Malaysia, ^5^Centre of Excellence for Research in AIDS (CERiA), Faculty of Medicine, Kuala Lumpur, Malaysia

**Background**: Early screening of people who inject drugs (PWID) co‐infected with HIV and Hepatitis C (HCV) facilitates timely treatment and better management of these co‐morbidities. However, the scale‐up of HIV and HCV testing can be hampered by punitive criminal justice approaches for this key population. This study aims to describe the characteristics of PWID, experiences with police and incarcerated settings and HIV/Hepatitis C testing status.

**Description**: Using a cross‐sectional, respondent‐driven sampling (RDS) survey, a total of 367 PWID were recruited in Selangor, Malaysia between September 2021 to January 2022. The survey assessed the current situation of sociodemographic factors; drug use patterns; experiences with the criminal justice system; health status and access to social support or healthcare services, including HIV/HCV testing.

**Lessons learned**: Overall, the study recruited 345 (94%) male and 22 (6%) female, with 297 (81%) participants used Methamphetamine and 318 (87%) used Heroin in the past month. A total of 34 (94%) participants had ever been in lock up, 314 (85%) in prisons and 174 (47%) in compulsory drug detention centers. Among these participants, 163 (44%) reported that they had a rushed injection for fear of police, 129 (35%) experienced confiscation of injecting equipment by the police; 182 (50%) have been beaten up or tortured by police, and 191(52%) avoided carrying injecting equipment for fear of the police. Only 283 (77%) participants and 186 (51%) ever had HIV and HCV screening, respectively. At the study visit, a number of 22 (6%) individuals were detected HIV positive, and 157(43%) were detected HCV positive.

**Conclusions/Next steps**: Results showed high Hepatitis C prevalence among PWID, in the context of high levels of negative experiences with the criminal justice system which may affect safer injecting practices. A change towards a more health‐based policy will lead to a more enabling environment for PWID to access health services and implementation of a more effective harm reduction services for PWID.

### PESUF16 Does travel time matter? Transportation vulnerability and access to HIV care among people living with HIV in South Carolina

P. Hung^1,2^, S. Harrison ^2,3^, K. Green^4^, S.J. Miller^3^, M. Paton^3^, D. Ahuja^4^, S. Weissman^4^, C.A. Rudisill^5^, T. Evans^6^

^1^University of South Carolina Arnold School of Public Health, Health Services Policy and Management, Columbia, United States, ^2^University of South Carolina Arnold School of Public Health, South Carolina SmartState Center for Healthcare Quality, Columbia, United States, ^3^University of South Carolina College of Arts and Sciences, Department of Psychology, Columbia, United States, ^4^University of South Carolina School of Medicine, Department of Internal Medicine, Columbia, United States, ^5^University of South Carolina Arnold School of Public Health, Health Promotion, Education and Behavior, Columbia, United States, ^6^ViiV Healthcare, Research Triangle Park, Columbia, United States

**Background**: Access to safe and reliable transportation is necessary for people living with HIV (PLHIV), particularly in areas where public transportation is limited. Transportation barriers and long travel times might result in delayed linkage to care and missed appointments, leading to disease progression for PLHIV. This study examined travel burdens, transportation barriers, and the associated HIV care outcomes among PLHIV in South Carolina—a rural southern state in the United States (US).

**Methods**: A total of 160 PLHIV from a large immunology center – who were re‐engaging with HIV care after a prolonged absence or were in care but not virally suppressed – were enrolled in a randomized clinical trial from January 2020 to June 2021. During the enrollment, each participant completed an intake survey reporting their sociodemographic characteristics, barriers to HIV care, and transportation vulnerability. Using Kruskal–Wallis tests, sociodemographic characteristics, transportation vulnerability, and negative care outcomes (e.g., missed or delayed appointments) were compared across residential proximity to HIV care, measured by number of minutes needed to travel to the immunology center. Multivariable logistic regressions were employed to identify the likelihoods of negative outcomes for PLHIV living <15 minutes, 15–30 minutes and >30 minutes from the center.

**Results**: A majority of participants were male (63.8%), aged 45–64 years (54.4%), never married (77.0%), Black or African‐American (77.5%), and non‐Hispanic (82.5%). Many were unemployed (40.6%), receiving public insurance (50.6%), and reported transportation barriers (59.4%). Nearly 20% of participants lived <15‐minutes from the clinic, 59.1% lived 15–30‐minutes, and 21.4% lived >30‐minutes from the clinic. Compared to participants living <15‐minutes away, those living >30‐minutes from the center were more likely to be late for appointments (aOR = 5.25, 95% CI, 1.06–25.92), miss appointments (aOR = 3.85, 95% CI, 1.04–15.89), and have difficulties seeing doctors (aOR = 7.06, 95% CI, 1.61–30.99).

**Conclusions**: Long travel time is a barrier to care for PLHIV in South Carolina and is associated with negative care outcomes including missed HIV appointments. Lack of transportation is likely to further aggravate HIV disparities in the southern US. Local and statewide efforts such as expanding public infrastructure and developing ridesharing approaches should be prioritized for underserved and low‐resource communities.

### PESUF17 Digital health and rights of young adults living with HIV and young key populations in Ghana, Kenya and Vietnam: a participatory action research study

C. Nininahazwe ^1^, S.L.M. Davis^2^, E. Ayeh^3^, G. Caswell^4^, D.D. Dong^5^, T. Ha^6^, T. Imalingat^7^, I.E. Kpodo^8^, N. Mjwana^4^, A. Muthui^7^, T. Pham^5^, M. Podmore^6^, T.J. Sandset^9^, N. Were^7^, A. Maleche^7^

^1^Global Network of People Living with HIV (GNP+), Amsterdam, Netherlands, the, ^2^Graduate Institute of International and Development Studies, Global Health Centre, Geneva, Switzerland, ^3^Ghana National Association of People Living with HIV (NAP+ Ghana), Kumasi, Ghana, ^4^Global Network of People Living with HIV (GNP+), Cape Town, South Africa, ^5^Vietnam Network of People Living with HIV (VNP+), Hanoi, Viet Nam, ^6^STOPAIDS, London, United Kingdom, ^7^KELIN, Nairobi, Kenya, ^8^Ghana National Association of People Living with HIV (NAP+ Ghana), Accra, Ghana, ^9^University of Oslo, Centre for Sustainable Healthcare Education, Oslo, Norway

**Background**: Digital health is rapidly scaling up in low‐ and middle‐income countries, transforming access to health services for young people living with HIV and key populations. However, human rights experts have highlighted related threats to privacy, autonomy and equity, given weak governance of digital platforms.

**Methods**: To understand the experience of young adults with digital health and how they see their human rights, we conducted a qualitative study in Ghana, Kenya and Vietnam using a participatory action research approach. We combined a legal and policy review of digital health governance and access in each country, with digital ethnography, focus group discussions, and key informant interviews. Global and national networks of people living with HIV, AIDS advocates, youth activists and human rights lawyers collaborated with anthropologists in design, data‐gathering, analysis, and drafting of policy recommendations.

**Results**: During the Covid‐19 pandemic, the 200 respondents described a major shift onto mobile apps and social media platforms to gain information and advice on sexual and reproductive health, which would otherwise be inaccessible to them due to stigma and confidentiality concerns. In an ungoverned environment of conflicting health advice, many reported relying on social media influencers as trusted sources. Young adults shared positive experiences of empowerment and peer support online, and a growing interest in cybersex as “safe sex”. They also reported online and offline harm, including data breaches, extortion, cyberbullying and censorship. Most respondents lacked basic understanding of online security or data management. They expressed an interest to learn more, and to have a greater voice as youth in design and governance of digital technologies.

**Conclusions**: To achieve the aim of ending AIDS as a public health threat, more robust rights‐based digital policy and governance are critically needed, complemented by digital literacy training for young people. Young people should be fully empowered and engaged in design, planning and governance of digital technologies used for health.

### PESUF18 Understanding intersectional oppressions experienced by Immigrants and Refugees Living with HIV: Equity and social determinants of health lenses

R. Dhungel ^1^, K. Karki^2^, J. Wang^3^

^1^The University of the Fraser Valley, School of Social Work and Health and Community Studies, Abbotsford, Canada, ^2^University of the Fraser Valley, School of Social Work and Health Services, Abbotsford, Canada, ^3^University of Calgary, Phychology, Calgary, Canada

**Background**: This presentation examines the intersectional oppression experienced by Immigrants and Refugees Lining with HIV (IRLWH) in Alberta, Canada with a focus on COVID‐19 pandemic. The pandemic has had an unprecedented impact on IRLWH. In response, the Public Health Agency of Canada has expanded its list of risk factors to include occupational, socio‐economic, and other life circumstances that increase risk for COVID‐19 infection and severe outcomes. This is certainly an advance; however, gaps continue to exist. By recognizing the limited extant knowledge in relationship to the COVID‐19 in particular in Alberta, a community‐based study was designed. The primary goal of the collaboration was to critically examine the challenges faced by IRLWH with a particular focus on the mental health and psychosocial wellbeing of IRLWH during the pandemic.

**Methods**: We employed the concurrent parallel mixed‐methods approach. Quantitative data were collected using a self‐developed survey (n = 124), and qualitative data were collected through three photovoice sessions (n = 13) among IRLH across Alberta between May 2021 and December 2021. The survey and the photovoice sessions assessed/captured the experiences of the IRLH on social determinants of health and its impacts on mental health and social wellbeing, both prior to and during the COVID‐19.

**Results**: This study claimed that national HIV policies, programs and services do not address their issues, including affordable housing, food security/ employment opportunities, health plan, and racism, impacted on their mental health. These results corroborated the findings from the surveys, for instance 51.5% reported having issues accessing healthcare services during COVID‐19 compared to 38.4% before the COVID‐19 pandemic. Similarly, 45.4% reported having difficulty accessing an HIV organization during the COVID‐19 pandemic compared to 35.3% before the pandemic. We also found that about 58% reported having problems finding housing services during COVID‐19 compared to 47.5% before COVID‐19.

**Conclusions**: This study found that COVID‐19 had escalated their vulnerabilities to mental health and socio‐economic marginalization. Based on the findings of the results, we developed the “Emerging community‐led transformative HIV post COVID‐19 Model” which speaks to the need of the active involvement of border communities including policy makers, health professionals, practitioners, educators and communities at large with IRLWH in addressing the challenges experienced.

### PESUF19 Association between LGBTI+ health equity and achieving UNAIDS 2030 targets: Findings from a global study of Fast‐Track Cities

C. Prachniak‐Rincón ^1^, J.M. Zuniga^1,2^

^1^International Association of Providers of AIDS Care (IAPAC), Washington, United States, ^2^Fast‐Track Cities Institute, Washington, United States

**Background**: Lesbian, gay, bisexual, transgender, and intersex (LGBTI+) individuals face inequities with respect to HIV prevalence, syndemic and comorbid conditions, and accessing services. Progress on LGBTI+ equity is essential to meeting the UNAIDS goals that at least 95% of people living with HIV know their status, 95% of those individuals are on treatment, and 95% of those individuals are virally suppressed.

**Methods**: Relevant laws and policies for LGBTI+ equity were assessed in 32 Fast‐Track Cities. Key informants (131) from these cities were surveyed on LGBTI+ issues using a Likert scale. These responses were averaged and trends were assessed at regional and global levels. The results were then compared to the cities’ average percentage across the three 95 targets. Cities were divided into those with 95‐95‐95 averages below 85% (“limited progress group”), between 86% and 90% (“mid‐progress group”), and at or above 91% (“high progress group”).

**Results**: Cities in the limited progress group had lower LGBTI+ policy scores (2.27, wherein 0 was least inclusive and 4 was most inclusive) than did the mid‐progress (2.33) or high progress (3.08) groups of cities. Similarly, the limited progress group had the lowest‐rated LGBTI+ primary care and HIV care, and the most serious problems with respect discrimination, according to key informants. In contrast, the high progress group of cities had the highest levels of LGBTI+ inclusive care and lower levels of discrimination.

**Quality of LGBTI+ care (1** = **“poor,” 5** = **“excellent”)**

**Table:** 

Abstract PESUF19‐Table 1.		
Group	Primary care	HIV care
Limited progress	3.08	3.75
Mid‐progress	3.24	3.89
High progress	3.64	4.10

**LGBTI+ discrimination (1** = **“not a problem,” 4** = **“serious problem”)**

**Table:** 

Abstract PESUF19‐Table 2.		
Group	Sexual orientation discrimination	Gender identity discrimination
Limited progress	2.85	3.12
Mid‐progress	2.73	3.08
High progress	2.19	2.69

**Conclusions**: While this study did not explore causality, an association was found between progress on the 95‐95‐95 targets and inclusive LGBTI+ policies, better LGBTI+ care, and less discrimination. Additional research, including surveying LGBTI+ communities directly and using larger sample sizes, could further contribute to knowledge of LGBTI+ health at the local level and aid cities in ending HIV.

### PESUF20 Human rights violations against key populations in South Africa Public health facilities: findings from the Ritshidze Community‐led Monitoring Programme

N. Mavasa^1,2^, N. Rambau ^1^, M. Sebotsa^1^, S. Sibisi^1^, L. Rutter^3^, J. Sherwood^4^

^1^Ritshidze Community‐Led Monitoring Project, Johannesburg, South Africa, ^2^Treatment Action Campaign, Johannesburg, South Africa, ^3^Health GAP, Johannesburg, South Africa, ^4^amfAR, The Foundation for AIDS Research, Washington DC, United States

**Background**: Members of key populations (KPs), including men who have sex with men (MSM), sex workers (SW), trans* people, and people who use drugs (PWUD), have increased vulnerability to HIV and experience legal and social barriers to healthcare. The HIV response is dependent on the healthcare system's ability to serve these populations, yet they are often the most excluded from care. Through the Ritshidze Community‐led Monitoring Programme we track the quality of healthcare for KPs in South Africa with implications for improving services and rectifying abuses.

**Methods**: Key populations were recruited for a cross‐sectional survey via community‐based snowball sampling in 18 PEPFAR‐supported districts across seven provinces from August to October 2021. Initial participants were community‐recruited and had previously engaged in Ritshidze qualitative data collection. Survey data on KP healthcare experiences were collected electronically by trained KP data collectors. Data were analysed using descriptive statistics for key service quality and human rights indicators by population and province.

**Results**: A total of 5,979 KP members, including MSM (n = 1,476), PWUD (n = 2,397), SW (n = 1,344) and trans* people (n = 762) were surveyed. Over the past year, 14% of MSM (n = 207), 13% of SW (n = 175), 12% of PWUD (n = 288), and 11% of trans* people (n = 84) reported being denied health services at a public health facility (PHF) because they are a member of a key population. Denial of services was highest in Limpopo (30% n = 218) and KwaZulu‐Natal province (20% n = 81). Overall, 24% (n = 849) of KPs reported experiencing privacy violations at a facility, including disclosure of their HIV and KP member status. Among those who were not receiving services at a PHF, the primary reasons were lack of friendly services (66%, n = 114), lack of privacy (49% n = 84) and unsafe conditions (26%, n = 45).

**Conclusions**: Human rights violations and unfriendly services at PHFs were frequently reported by KP members in South Africa. These violations are a likely detriment to the health of KP members as well as to the broader HIV and public health outcomes in South Africa. Denial of healthcare to KPs in South Africa violates their Constitutional right to access health and requires immediate attention by the National Department of Health.

### PESUF21 If not now, when? A unique opportunity to reduce HIV‐related stigma & discrimination, criminalization & other human rights‐related barriers

R. Jurgens ^1^, A. Iovita^1^, H. Lim^1^, A. Shaw^1^, N. Avani^1^

^1^Global Fund to Fight AIDS, TB and Malaria, Community, Rights and Gender, Geneva, Switzerland

**Background**: Stigma and discrimination (S&D), criminalization, gender inequality and other human rights (HR) barriers have impeded access to HIV services and attainment of global HIV goals. Despite UN Member States´ commitments to support programs that reduce such barriers, until recently these programs had nowhere been sufficiently scaled up. To address this, the Global Fund's “Breaking Down Barriers” (BDB) initiative has been supporting and evaluating the scale‐up of seven key HR programs and undertaken sustained efforts to increase country ownership of HR problems and solutions.

**Description**: 20 countries are part of BDB: Benin, Botswana, Cameroon, Côte d'Ivoire, DRC, Ghana, Honduras, Indonesia, Jamaica, Kenya, Kyrgyzstan, Mozambique, Nepal, Philippines, Senegal, Sierra Leone, South Africa, Tunisia, Uganda, Ukraine. In these countries, GF investment in HR programs increased more than 12‐fold, to levels never seen before, from $10.57 million in 2014–16 to 130 million in 2020–22. Scale, scope and quality of programs, as assessed by independent researchers, also significantly increased. All countries developed country‐owned, costed strategies to address HR barriers and established HR working groups to oversee and coordinate implementation.

**Lessons learned**: Elevating HR to one of four strategic objectives in the 2017–22 GF Strategy enabled the BDB initiative. Providing financial incentives, establishing programmatic conditions, making available long‐term TA to assist development of national strategies and scale up of HR programs, and closely monitoring and evaluating the initiative, have been critical. The initiative itself has evolved, integrating lessons learned and expanding partnerships in countries and at the global level.

**Conclusions/Next steps**: The experience of this initiative is providing unprecedented, well‐evaluated and soundly costed models for rights‐based approaches to HIV services. Already, the new GF Strategy (2023–2028) is integrating lessons learned, with an even greater focus on HR, equity and gender inequality and commitment to even greater action against S&D and criminalization and greater support for community‐led efforts. There is also unprecedented commitment to addressing these issues in the Global AIDS Strategy (2021–2026) and there are new and promising initiatives specifically to eliminate S&D. This provides for a unique opportunity we cannot afford to miss to make a real difference, with great expected benefits for health and human rights.

### PESUF22 Evaluating Legal Obstacles to Provider‐Assisted Injection in Ontario's Supervised Consumption Services

M.F. Sana Ullah^1^, I. Annamanthadoo ^1^, S. Ka Hon Chu^1^

^1^HIV Legal Network, Toronto, Canada

**Background**: Approximately 14% of new HIV infections in Canada were acquired through injection drug use,[1] and research indicates significantly higher HIV prevalence among people who require assistance injecting than those who do not. Provider‐assisted injection within supervised consumptions services (SCS) could reduce this risk, particularly for people with disabilities, women, and youth. However, concerns about providers’ legal liability impede the provision of this critical service within SCS. We interviewed SCS providers in Ontario to identify key questions and concerns raised by SCS nurses and other SCS staff about potential criminal liability, civil liability, and professional discipline associated with providing assisted injection.

[1] “The epidemiology of HIV in people who inject drugs in Canada” (2018) at 2, online (pdf): *CATIE* 〈www.catie.ca/sites/default/files/fs‐epi‐idu‐EN‐2018‐08‐15.pdf〉.

**Methods**: SCS providers (n = 11) were interviewed via videoconference in the fall of 2021. Nine respondents identified as nursing staff, one as management staff and one as a peer worker. SCS were selected from across the province to ensure geographical representation. Interviews were transcribed and consolidated. Respondents were asked to share their experience with assisted injection in SCS, including whether they observed a need for assisted injection, the consequences of refusing assistance, and the perceived legal barriers to providing assisted injection.

**Results**: All respondents observed a significant need for provider‐assisted injection and believed that it should be permitted. However, respondents identified several barriers that impede their ability to provide this service, including the threat of professional discipline (by the nursing regulatory body, for nurses) and a lack of clear guidelines condoning the practice from regulatory bodies and/or employers. Few respondents expressed serious concern about civil or criminal liability. Respondents reported that clients seeking provider‐assisted injection most often turn to peer‐assisted injection to meet this need – although such assistance is not always accessible or safe.

**Conclusions**: There is a significant unmet need for provider‐assisted injection within Ontario SCS. Although SCS staff are willing and competent to provide this support, legal and non‐legal barriers mean that provider‐assisted injection is unavailable to people who use drugs in Ontario, hampering the ability of staff to engage in vital HIV and overdose prevention work.

### PESUF23 The impact of scaling up human rights interventions on reducing inequality and increasing access to care and treatment for HIV and TB: Mid‐term results from the Breaking Down Barriers initiative

J. Amon ^1^, N. Sun^2^, S.K.H. Chu^3^, J. Csete^4^, R. Elliott^3^, M. Golichenko^3^, C. Kazatchkine^3^, D. Lohman^5^, J. Mabilat^3^, M. McLemore^6^, K. Hinman^2^, A. Iovita^7^, H. Lim^7^, A. Shaw^7^, R. Jurgens^7^

^1^Drexel University Dornsife School of Public Health, Philadelphia, United States, ^2^Drexel University, Philadelphia, United States, ^3^HIV Legal Network, Toronto, Canada, ^4^Columbia University, New York, United States, ^5^Consultant, New York, United States, ^6^Consultant, Philadelphia, United States, ^7^Global Fund, Geneva, Switzerland

**Background**: The Global Fund's *Breaking Down Barriers* (BDB) initiative provides support for countries to scale‐up to comprehensive programs to remove human rights‐related barriers to HIV and tuberculosis (TB) services, with the aim to increase the effectiveness of Global Fund grants and ensure that health services reach those most affected.

**Description**: A mid‐term evaluation, conducted in 2020–21, examined progress in scaling up programs identified as effective in reducing human rights‐related barriers to health services for HIV and TB in 20 BDB countries (Benin, Botswana, Cameroon, Cote d'Ivoire, Democratic Republic of Congo, Ghana, Honduras, Jamaica, Indonesia, Kenya, Kyrgyzstan, Mozambique, Nepal, Philippines, Senegal, Sierra Leone, South Africa, Tunisia, Uganda, Ukraine). These included programs to: reduce HIV‐related stigma and discrimination; train health care workers on human rights and ethics; sensitize lawmakers and law enforcement; provide legal literacy; provide HIV‐related legal services; monitor and reform laws, regulations and policies; and reduce discrimination against women and girls. For tuberculosis, in addition to these programs, it also included programs that: protect confidentiality and privacy in TB services; mobilize and empower TB patient and community groups; and improve TB services in prisons and other closed settings. The evaluation included both qualitative and semi‐quantitative assessment, focusing upon measuring scale up of programs to ensure nationwide coverage and identifying emerging evidence of impact.

**Lessons learned**: All countries saw progress in removing human rights‐related barriers to HIV and TB services. For HIV, Ukraine had the highest coverage of programs to reduce barriers, and Sierra Leone had the greatest increase compared to baseline, followed by Jamaica, Senegal, Cameroon, and Mozambique. For TB, program coverage was highest in Ghana, and increases in coverage were greatest in Ukraine and Côte d'Ivoire. Evidence of impact was found in relation to success in challenging rights violations in the courts, countering the criminalization of key populations, addressing discrimination and stigma, advancing harm reduction programs, working with police and responding to gender‐based violence.

**Conclusions/Next steps**: Across a diverse set of countries, the scale up of human rights‐based programs is possible and effective in reducing barriers and increasing access to HIV and TB services.

### PESUF24 Capacity strengthening of local civil society organizations offering HIV services: Learning experiences from Liberia

P. Gobah ^1^, G. Kamanga^1^, C. Kerbay^1^, L. Harris^1^, N. Fosua Clement^1^, D. Darrow de Mora^2^, H. Rodriguez Sherman^2^

^1^FHI 360, Monrovia, Liberia, ^2^FHI 360, Washington DC, United States

**Background**: With support from PEPFAR and USAID, the FHI 360‐led LINKAGES project and subsequent EpiC project implemented a comprehensive package of HIV prevention, care, and treatment services among 10 civil society organizations (CSOs) serving key populations (KPs) in Montserrado County, Liberia. When LINKAGES began, the technical and management capacity of these CSOs was limited. LINKAGES, in collaboration with Pact and EpiC, implemented capacity‐building innovations to enhance technical delivery of HIV Services and improve organizational management systems.

**Description**: The project conducted baseline capacity‐building assessments with all 10 CSO partners using two standardized toolkits: the integrated technical and organizational capacity assessment and the Organization Performance Index (OPI). Institutional strengthening plans were then developed and implemented to address the issues identified and monitor progress. Project engagement with the 10 CSO partners around internal control systems, operational management, and governance helped foster ownership of HIV service delivery in Montserrado County.

**Lessons learned**: From April 2019 through September 2021, EpiC Liberia trained all 10 CSOs in HIV community interventions to empower peer outreach workers, people living with HIV (PLHIV), and KPs, including the members of 23 HIV support groups, to understand and manage their needs, risks, and rights regarding HIV services. The project also trained CSO leadership in organizational system development, which focused on organizational management and operations policies, resource mobilization, sustainability, partnership, and networking to enable delivery of optimal HIV services. At the first OPI assessment conducted to measure performance and impact, two out of four performance measurements — “efficiency” and “effectiveness” — achieved the maximum score of 4.0. “Relevance in meeting the needs of beneficiaries” scored 2.5 and “sustainability” scored 1.0. Measures to improve relevance and sustainability are being implemented, and some CSOs already have won new grants and developed successful small business enterprises. These efforts contributed to reaching 15,000 individuals with a comprehensive HIV service package, testing 12,000 individuals for HIV, and identifying 850 individuals as living with HIV, 94% of whom were linked to treatment.

**Conclusions/Next steps**: Ongoing Support for CSO capacity building in technical and management operations facilitated the building of a strong KP HIV program and network of KPs and PLHIV.

### PEMOA26 Developing a novel HIV cure strategy: retargeting potent cytotoxic T cells to kill HIV‐infected cells

E.F. Iversen ^1,2^, C.V. Konrad^1,2^, J.D. Gunst^2^, I.M. Johannsen^1,2^, L.J. Østergaard^1,2^, O.S. Søgaard^1,2^, M.H. Schleimann^2^, M. Tolstrup^1,2^

^1^Aarhus University, Institute of Clinical Medicine, Aarhus, Denmark, ^2^Aarhus University Hospital, Department of Infectious Diseases, Aarhus, Denmark

**Background**: Cytotoxic T lymphocytes (CTL) are potent killers of virus infected cells. In most HIV‐1‐positive people, HIV‐specific CTLs are exhausted with limited capacity to control or eliminate HIV‐1 infection. Therefore, we developed a novel immunotherapy concept in which potent *de novo* vaccine‐induced effector CTLs can be redirected to target and eliminate HIV‐1‐infected cells.

**Methods**: We developed a bispecific molecule (RoVER: **R** edirector **o** f **V** accine‐induced cytotoxic T **E** ffector **R** esponses) comprising two functionally distinct domains:

1. a scFv‐domain targeting HIV Env, and
2. an HLA‐I molecule carrying a yellow fever vaccine epitope.

Following Yellow Fever (YF‐17D Stamaril, Novartis) vaccination of 52 healthy volunteers, YF epitope‐specific CTL responses were quantified by tetramer staining and multicolour flow cytometry. The ability of RoVER to mediate killing of HIV‐infected cells by linking YF vaccine‐induced CTLs was assessed in a series of killing assays. As target cells, Raji‐Env and autologous CD4+ cells infected *in vitro* with a full‐length HIV‐1‐egfp were used. Moreover, extracellular release of IFN‐y, Granzyme B and TNF‐a were analysed by mesoscale multiplex assays.

**Results**: YF‐17D vaccination induced strong epitope‐specific CTL responses in all study participants. In HLA*A2 individuals, a mean of 2.8% of CTLs (range 0.1–10.3%) targeted the immunodominant NS4B_214–222_epitope. RoVER‐mediated redirection of NS4B‐specific effector CTLs to Raji‐Env cells resulted in killing of target cells regardless of whether the two domains of RoVER were linked through streptavidin or recombinantly expressed. Redirection to HIV‐egfp‐infected autologous CD4 target cells resulted in 65% killing at E:T ratio 3:1. In contrast, no target killing was observed using autologous CTLs obtained prior to YF‐17D vaccination or without exposure to RoVER. Moreover, RoVER‐mediated target cell killing could be achieved using different HLA‐molecules and YF epitopes. Lastly, target cell killing was associated with pronounced secretion of IFN‐y.

**Conclusions**: We have developed a novel immunotherapy concept in which epitope‐specific CTLs induced by vaccination can be redirected towards HIV‐infected cells. This novel technology is highly specific and easily adaptable to recognize any target of interest while obviating the need for *ex vivo* modification and expansion, thus holding great potential for various diseases.

### PEMOA27 Clinical and Immunometabolic Patterns Determining Efficacy of DC‐treatment reinvigorating HIV‐1‐specific CD8+ T cells in PLWH

M. Calvet‐Mirabent ^1,2^, I. Sánchez‐Cerrillo^1,2^, N. Martín‐Cófreces^1,2,3^, H. de la Fuente^1,3^, I. Tsukalov^2^, C. Delgado‐Arévalo^1,2^, M.J. Calzada^2^, I. de los Santos^4,5^, J. Sanz^4,5^, L. García‐Fraile^4,5^, F. Sánchez‐Madrid^1,2,3^, A. Alfranca^1^, M.Á. Muñoz‐Fernández^6^, M.J. Buzón^7,8^, E. Martín‐Gayo^1,2,5^

^1^Hospital Universitario de La Princesa, Immunology Unit, Madrid, Spain, ^2^Universidad Autónoma de Madrid, Medicine Faculty, Madrid, Spain, ^3^Centro de Investigación Biomédica en Red Cardiovascular, CIBERCV, Madrid, Spain, ^4^Hospital Universitario de La Princesa, Infectious Diseases Unit, Madrid, Spain, ^5^Centro de Investigación Biomédica en Red Infecciosas, CIBERINF, Madrid, Spain, ^6^Instituto de Investigación Sanitaria Gregorio Marañón (IiSGM), Hospital General Universitario Gregorio Marañón, Immunology Section, Madrid, Spain, ^7^Institut de Recerca Hospital Univesritari Vall d'Hebrón (VHIR), Infectious Diseases Department, Barcelona, Spain, ^8^Universitat Autònoma de Barcelona, Barcelona, Spain

**Background**: Heterogeneous dysfunctional states of CD8+ T cells in people living with HIV‐1 (PWLH) has limited the efficacy of dendritic cell (DC)‐based immunotherapies. Here, we studied associations between improved functional response to Gag‐loaded adjuvant‐primed DCs of CD8 T cells from PLWH with ART duration, memory subset distribution and exhaustion and metabolic profiles in these cells.

**Methods**: A cohort of n = 49 PLWH on ART with undetectable plasma viremia and CD4+ T counts above 400cells/ml were recruited. Monocyte‐derived DC were activated with Poly I:C and 2′3′c‐diAM(PS)2 adjuvants in the presence of a pool of HIV‐1 Gag peptides and co‐cultured with autologous CD8+ T cells. Induction and polyfunctionality of HIV‐1 specific CD8+ T responses was evaluated by IFNg and CD107a expression by FACS. Functionality of DC‐stimulated CD8+ T cells was evaluated by co‐culture with autologous CD4+ T cells and the ability to reduce proportions of p24+ CD4+ T cells. Individual or combined anti‐PD1, TIGIT, TIM3 antibodies and Metformin were used in some functional assays. Characterization of CD8+ T cell memory subset and exhaustion markers was analyzed by FACS. Metabolic profiles of CD8+ T cells were analyzed by Seahorse.

**Results**: Polyfunctionality and functional capacities to eliminate p24+ CD4+ T cells of HIV‐1 specific CD8+ T cell responses from PLWH on ART for more than 10 years (LT‐ARTp) significantly improved after activation with adjuvant‐engineered DC *in vitro* (p = 0.001 and p = 0.0039; respectively). In contrast, CD8+ T cells from PLWH on ART for less than a decade (ST‐ARTp) were less responsive to DC (p = 0.0024) and unable to increase cytotoxic function (p = 0.0156). This was associated with lower frequencies of central memory CD8+ T cells, increased co‐expression of PD1 and TIGIT (p = 0.0362) and reduced mitochondrial respiration and glycolytic induction after TCR activation (p = 0.002). In contrast, enrichment on TIM3+ PD1‐ cells (p = 0.001) and preserved glycolytic induction (p = 0.0005) was observed in CD8+ T cells from LT‐ARTp. Finally, combined treatment of anti‐PD1, anti‐TIGIT antibodies and metformin restored cytotoxic properties of dysfunctional CD8+ T cells from ST‐ARTp (p = 0.0156).

**Conclusions**: We identified new immunometabolic parameters potentially useful to personalize DC‐based HIV‐1 vaccines and improve specific CD8+ T cell response in different PLWH populations.

### PEMOA28 Pharmacologic intervention to reduce chronic inflammation in people with HIV

S. Rodriguez‐Mora^1^, G. Casado‐Fernandez^1^, M. Manzanares^1^, J. Canton^2^, L. Vigon^1^, F. Ramos‐Martin^1^, M. Torres^1^, C. Hoffmann^3^, C. Wyen^4^, M. Cervero^2^, M.A. Murciano‐Anton^5^, V. Planelles^6^, M. Coiras ^1^

^1^Instituto de Salud Carlos III, Madrid, Spain, ^2^Severo Ochoa University Hospital, Madrid, Spain, ^3^ICH Study Center, Hamburg, Germany, ^4^University Hospital of Cologne, Cologne, Germany, ^5^Laín Entralgo Health Center, Madrid, Spain, ^6^University of Utah School of Medicine, Utah, United States

**Background**: Chronic inflammation and persistent immune activation are critical for HIV‐1 disease pathogenesis and progression. HIV‐infected monocyte‐derived macrophages (MDMs) contribute to long‐lived reservoirs and chronic inflammation in people with HIV (PWH) due to persistent release of proinflammatory mediators. Tyrosine kinase inhibitor dasatinib, which is used to treat chronic myeloid leukemia (CML), inhibits HIV‐1 infection in CD4+ T cells by preserving SAMHD1 antiviral activity. SAMHD1 also downregulates interferon (IFN) and other inflammatory responses to viral infections. Our objective was to evaluate if dasatinib may control both infection and proinflammatory effects of HIV‐1 on MDMs from PWH, as well as reduce the levels of proinflammatory cytokines in plasma of PWH on ART and dasatinib.

**Methods**: 15 ART‐treated PWH, 3 PWH with CML on ART and dasatinib, and 11 healthy donors were recruited. CD14+ cells from PBMCs were differentiated to MDMs and then infected with JR_FL_Renilla strain for 48h with or without dasatinib. HIV‐1 infection was analyzed by flow cytometry. SAMHD1 phosphorylation and synthesis of IFNγ and TNFα were analyzed by flow cytometry after stimulation with lipopolysaccharide (LPS). Plasma cytokines were quantified by Luminex.

**Results**: 1) Dasatinib reduced 3.0‐(p = 0.0104) and 2.1‐fold the LPS‐induced synthesis of IFNγ from MDMs of PWH and healthy donors, respectively. TNFα synthesis remained unchanged. 2) Plasma levels of proinflammatory cytokines IL‐15, IL‐18, IL‐21, and IL‐23 were reduced 2.17‐, 1.55‐, 1.61‐, and 4.86‐fold in PWH on ART+dasatinib, in comparison with ART‐treated PWH. IFNβ was undetectable in plasma of PWH on ART+dasatinib. 3) Dasatinib reduced 1.8‐(p = 0.0420) and 2.6‐(p = 0.0459) fold SAMHD1 phosphorylation in MDMs from PWH and healthy donors, respectively. 4) Dasatinib interfered with proinflammatory NF‐κB‐dependent transcriptional activity (p < 0.0001). 5) HIV‐1 infection was reduced 2.4‐(p = 0.0006) and 5.9‐fold in MDMs from PWH and healthy donors, respectively, after treatment with dasatinib.

**Conclusions**: New therapeutic interventions are needed to reverse chronic inflammation caused by HIV‐1 persistence. Dasatinib reduced the levels of proinflammatory cytokines in plasma of ART‐treated PWH and reverted SAMHD1 constitutive phosphorylation of MDMs, protecting them from HIV‐1 infection and reducing their inflammatory potential. The use of dasatinib as adjuvant of ART would decrease the inflammatory environment characteristic of chronic infection, thereby improving health of PWH.

### PEMOA29 In Vitro Release and In Vivo Pharmacokinetics of Antiretroviral Drugs from Ultra‐Long‐Acting Polymeric Implants: Towards Better Outcomes for HIV Prevention and Treatment

 

**Figure:**
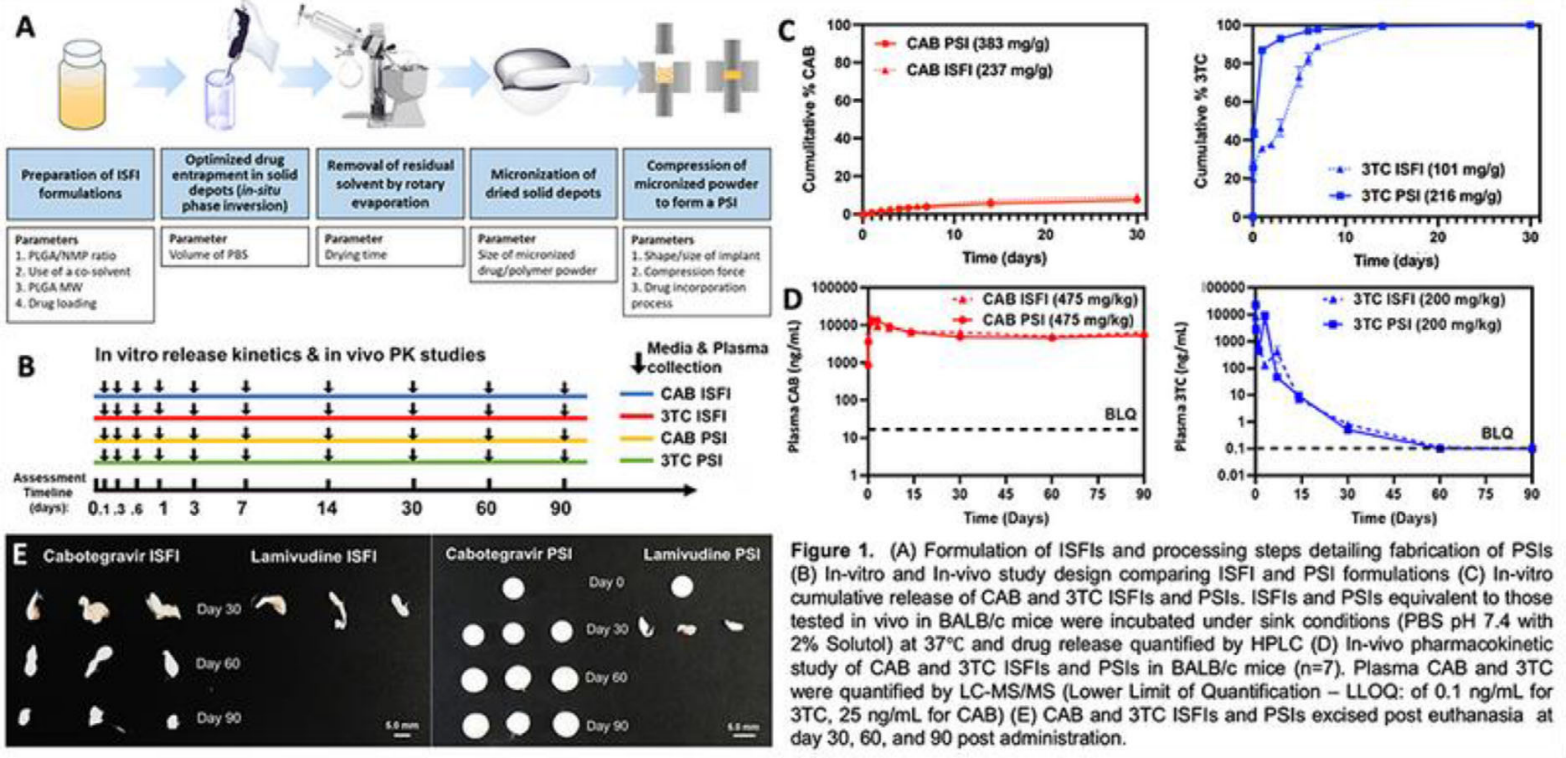
**Abstract PEMOA29‐Figure 1**.

P. Maturavongsadit^1^, R. Shrivastava^1^, A. Prasher^1^, S.A. Howard^2^, C. Sykes^2^, M. Cottrell^2^, A. Kashuba^2^, S.R. Benhabbour ^1^

^1^University of North Carolina at Chapel Hill, UNC_NCSU Joint Department of Biomedical Engineering, Chapel Hill, United States, ^2^University of North Carolina at Chapel Hill, Eshelman School of Pharmacy, Chapel Hill, United States

**Background**: Long‐acting pre‐exposure prophylaxis or treatment formulations that can provide sustained drug release over weeks or months can potentially reduce the incidence of new HIV infections and improve adherence.

**Methods**: Ultra‐long‐acting (ULA) biodegradable, removable in‐situ forming implants (ISFIs) and polymeric solid implants (PSIs) were generated via phase inversion upon injection (ISFIs) or using a simple process combining phase inversion and compression (PSIs; **Fig. 1A**). These formulations can accommodate one or more antiretrovirals (ARVs) in a single injection or implant. ISFIs and PSIs of Cabotegravir (CAB) and Lamivudine (3TC) were tested for in vitro release (n = 3) by HPLC, and in vivo pharmacokinetics in BALB/c mice (n = 7) to establish correlations of CAB and 3TC, which have distinct physicochemical properties (**Fig. 1B**). Plasma CAB and 3TC concentrations were quantified by HPLC‐MS/MS with a lower limit of quantification (LLOQ) of 25 and 0.1ng/ml, respectively.

**Results**: CAB ISFIs and PSIs exhibited minimal burst release (∼1%) followed by sustained zero order release both in vitro and in vivo (**Fig. 1C‐D**). PSIs and ISFIs (475 mg/kg CAB) resulted in nearly identical plasma CAB levels with sustained zero order kinetics over 90 days (**Fig. 1D**). 3TC ISFIs and PSIs exhibited high burst (38% and 68% respectively) and reached complete release within 14 days (**Fig. 1C**). PSIs and ISFIs (200 mg/kg 3TC) demonstrated nearly identical 3TC plasma concentrations; however, plasma 3TC rapidly declined to below LLOQ at day 60 (**Fig. 1D**). Drug physicochemical properties also influenced in vivo implant degradation rate. 3TC (log P −1.5, pKa 1.09) ISFIs and PSIs exhibited faster degradation in vivo compared to CAB (log P 1.04, pKa 10.04) and reached complete degradation by day 60 (**Fig. 1E**).

**Conclusions**: ULA tunable, biodegradable ISFIs and PSIs can accommodate ARVs of distinct physicochemical properties and exhibited near identical plasma drug concentrations for both CAB and 3TC when administered at equivalent doses.

### PEMOA30 Persistent AP‐1‐induced immune activation during early SARS‐CoV‐2 infection revealed by simultaneous single‐cell epigenetic and gene expression profiling

Y. Wei ^1^, J. Collora^1^, R. Liu^1^, J. Klein^2^, C. Lucas^2^, A. Iwasaki^2^, Y.‐C. Ho^1^

^1^Yale School of Medicine, Microbial Pathogenesis, New Haven, United States, ^2^Yale School of Medicine, Immunology, New Haven, United States

**Background**: SARS‐CoV‐2 induces cytokine response dysregulation and immune dysfunction. What remains unclear is how cytokine signaling shapes immune responses during early SARS‐CoV‐2 infection when adaptive immunity is developing. Our goal is to identify immune pathways that shape the early development of adaptive immune responses in COVID‐19 patients.

**Methods**: We performed paired single‐cell transcriptomic and epigenomic profiling at two time‐points of early SARS‐CoV‐2 infection to determine immune signatures of acute infection and epigenetic drivers that underpin immune response dynamics. PBMC samples from Yale IMPACT cohort were collected from four moderate to severe COVID‐19 patients at two early time‐points (n = 3 for Week 1 and n = 3 for Week 2 after symptom onset, including 2 participants having paired blood sampling at both time points) and from two healthy controls (n = 2). Using paired scRNA‐Seq and scATAC‐Seq, we captured transcriptomic and epigenomic profiles in the same single cells to identify chromatin accessibility changes as a potential mechanism for the surge and decline of immune responses elicited during acute SARS‐CoV‐2 infection. Using bioinformatic approaches, we identified heterogeneous immune cell populations, determined dysregulated immune pathways through gene set enrichment analysis, and connected RNA expression profiles with chromatin co‐accessible landscapes.

**Results**: We captured transcriptomic and epigenomic profiles of 34,954 single cells and identified paired transcriptional and epigenetic landscapes. We found that SARS‐CoV‐2 infection induced an early surge in IL‐2, IL‐6, IFN‐α, IFN‐γ, and SMAD‐dependent TGF‐β responses at Week 1 that declined at Week 2 in adaptive immune cells (CD4+ T, CD8+ T, and B cells). In contrast, we found steady increase in AP‐1‐induced immune responses that persisted at Week 1 and 2 in adaptive immune cells despite convalescence. Expression levels of the AP‐1 transcription factors *JUN* and *FOS* were upregulated in tandem with increased chromatin accessibility at the AP‐1 binding sites and increased expression of host genes epigenetically regulated by AP‐1 signaling, such as MAP kinase activation genes (*PPIA* and *ADAP1*), *NFKB1*, and the CXCR5 ligand *CXCL13*.

**Conclusions**: Our finding suggests that AP‐1 signaling shapes early adaptive immune responses during early stages of acute SARS‐CoV‐2 infection.

### PEMOA31 HIV skews the B cell response to SARS‐CoV‐2 toward extrafollicular maturation

R. Krause ^1,2^, J. Steyn^3,2,4^, D. Muema^4,2^, F. Karim^4,2^, Y. Ganga^4^, A. Ngoepe^1^, Y. Zungu^4,2^, I. Gazi^5,2^, S. Cele^4,2^, M. Bernstein^4^, K. Khan^4^, M. Mazibuko^4^, N. Mthabela^6^, A. Sigal^4,2,7^, H. Kloverpris^4,8,9^, T. Ndung'u^4,8,10,7^, A. Leslie^1,8^, COMMIT‐KZN Team, SARS Cellular Immunology Team

^1^Africa Health Research Institute, TB Immunology, Durban, South Africa, ^2^University of KwaZulu‐Natal, School of Laboratory Medicine and Medical Sciences, Durban, South Africa, ^3^HIV Pathogenesis Programme, University of KwaZulu‐Natal, HIV virology, Durban, South Africa, ^4^Africa Health Research Institute, HIV Immunology, Durban, South Africa, ^5^KwaZulu‐Natal Research Innovation and Sequencing Platform, HIV, Durban, South Africa, ^6^Africa Health Research Institute, TB Immunology, Duban, South Africa, ^7^Max Planck Institute for Infection Biology, Berlin, Germany, ^8^University College London, Division of Infection and Immunity, London, United Kingdom, ^9^University of Copenhagen, Department of Immunology and Microbiology, Copenhagen, Denmark, ^10^Doris Duke Medical Research Institute, Durban, South Africa

**Background**: HIV dysregulates the B cell compartment affecting the nature and quality of the memory B cell, antibody secreting cell (ASC) and resulting antibody response to infections. Understanding the B cell response to COVID‐19 in people living with HIV (PLWH) could explain the increased morbidity, reduced clearance and intra‐host evolution of SARS‐CoV‐2. We compared the B cell response to SARS‐CoV‐2 infection in PLWH and HIV negative patients.

**Methods**: Our cohort from Durban, KwaZulu‐Natal, South Africa comprised primarily of mild to moderate COVID‐19 severity and was recruited during the first wave of the pandemic, in July 2020. Most HIV positive patients were on effective ART (n = 28), of which five were HIV viremic, and 32 were HIV negative. Patient blood samples were collected weekly for five weeks from symptom onset and positive diagnostic swab. Peripheral blood cells (PBMC) were isolated for B cell phenotyping by flow cytometry using three phenotyping panels to assess maturation, homing and regulatory populations.

**Results**: Using an unbiased tSNE analysis we observed a coordinated B cell homing response after infection. The ASC frequency associating with disease severity; where class switched memory and transitional B cells associated with resolution of disease. PLWH had lower germinal center (GC) homing capacity and class switched memory responses. In stark contrast to HIV negative patients, the COVID‐19 B cell response in PLWH had pronounced EF activity, despite only mild to moderate disease. This included expanded activated double negative (DN2) and activated naïve responses. In turn the higher early plasma blast frequencies also supported an active EF pathway in PLWH. The SARS‐CoV‐2 specificity of the EF response was confirmed using viral spike and RBD bait proteins.

**Conclusions**: HIV primes an EF B cell response to COVID‐19 even with mild to moderate disease, contrasting with the response in HIV negative patients. This suggests a potentially suboptimal B cell response to infection in PLWH. These results could explain the reduced antibody affinity and B cell memory responses in PLWH and support future studies aimed at monitoring both responses, especially considering new SARS‐CoV‐2 variants and vaccine dosing for optimal protection in PLWH.

### PEMOA32 The impact of delta and omicron variants in the T‐cell response to mRNA vaccination in people living with HIV

J.L. Casado ^1^, P. Vizcarra^1^, S. Martín‐Colmenarejo^1^, J. del Pino^1^, M.J. Pérez‐Elías^1^, A. Moreno^1^, M.J. Vivancos^1^, A. Martín‐Hondarza^2^, A. Abad^1^, A. Vallejo^1,2^

^1^Ramon y Cajal University Hospital, Infectious Diseases, Madrid, Spain, ^2^Ramon y Cajal University Hospital, Laboratory of Immunovirology, Madrid, Spain

**Background**: SARS‐CoV‐2 variant‐of‐concern (VOC) B.1.1.529 (Omicron) presents a surprisingly large number of mutations in its spike protein escaping from antibody neutralization. Thus, it is important to determine how well T‐cell responses perform against different variants including Omicron in people living with HIV (PWH) following SARS‐CoV‐2 vaccination.

**Methods**: Pilot study of PWH who underwent blood tests for humoral and cellular immunogenicity testing 30 days after the second dose of a SARS‐CoV‐2 mRNA vaccine. Humoral (anti‐S IgG, CLIA) and IFN‐γ producing T‐cell responses to spike peptides of the ancestral virus, delta, and omicron variants were performed.

**Results**: Overall, 24 PWH were included. Median age was 53 (interquartile range, IQR, 33–57) years, 71% were male, 4% were obese, and 42% had at least one comorbidity. Median nadir CD4+ count was 287 cells/mm3, and 13% had a previous AIDS diagnosis. Median current CD4+ count was 746/mm3 and HIV‐RNA viral load was ≤ 50 copies/ml in all the individuals. After the second vaccine dose, humoral and T‐cell responses to ancestral SARS‐CoV‐2 were observed in 96% and 92% of PWH, respectively, and were highly correlated (rho = 0.657; p < 0.01 between IgG and CD4+). Additionally, there was a high correlation between T‐cell responses to the ancestral strain, delta, and omicron variants. However, the magnitude of CD4+ and CD8+ T‐cell responses were significantly lower to delta (−13%, p = 0.004; and −32%, p = 0.007, respectively), and to omicron variants (−40%, p < 0.001; and −27%, p = 0.012,respectively) compared to the ancestral strain. In any case, 75% and 87%% of PWH continued to have CD4+ and CD8+ T‐cell responses to the omicron variant. As expected, those with the best cellular response to delta or omicron variants were those with the highest humoral response (rho = 0.62, p < 0.01 for CD4+, rho = 0.42, p = 0.03 for CD8+).

**Conclusions**: We report that IFN‐γ producing T cell responses against delta and omicron spike peptides, although preserved in an important proportion of PWH, were significantly lower than to the ancestral strain in individuals who received two doses of SARS‐CoV‐2 mRNA vaccine. The clinical importance of these findings should be further evaluated, as the presence of T‐cell responses could avoid the progression to severe disease in most cases.

### PEMOA33 Serological responses to SARS‐CoV2 vaccination in people with HIV: The SCAPE‐HIV Study

T.J. Barber ^1,2^, D. Peppa^3,1^, F. Burns^2,1^, J.R.G. Brown^4^, C. Smith^4^, N. Hemat^1^, S. Madge^1^, A. Hunter^1^, M. Lipman^1^, S. Bhagani^1^, T. Mahungu^1^, M.A. Johnson^1^

^1^Royal Free London NHS Foundation Trust, Ian Charleson Day Centre, London, United Kingdom, ^2^University College London, Institute for Global Health, London, United Kingdom, ^3^University College London, Institute of Immunity & Transplantation, London, United Kingdom, ^4^University College London, London, United Kingdom

**Background**: People with HIV (PWH), despite efficient virological suppression on antiretroviral therapy (ART) often display blunted responses to vaccination. There is a need to establish correlates of vaccine efficacy in PWH to tailor vaccine strategies to maximise protection against disease and new emerging variants. The SCAPE‐HIV Study (SARS‐CoV‐2 antibody prevalence in an HIV cohort) was established to determine antibody responses in PWH following SARS‐CoV2 infection and vaccination and evaluate parameters/clinical variables relating to antibody seropositivity.

**Methods**: SCAPE‐HIV is an ongoing cross‐sectional study in our adult PWH cohort. This interim analysis is restricted to 384 participants recruited between July‐September 2020 reporting 2 doses of SARS‐CoV2 vaccines. Participants completed questionnaires about sociodemographics, medical history, prior COVID19, and SARS‐COV2 vaccine uptake. Anti SARS‐CoV2 spike and nucleocapsid antibodies were quantified using commercial Roche assays at least 2 weeks after the last vaccine dose.

**Results**: 73.69% white; 82.55% male; 96.35% virally suppressed. 382/384 (99.47%) generated SARS‐CoV2 anti‐spike antibodies. 2/384 (organ transplant recipients) failed to seroconvert post two vaccines. Antibodies to nucleocapsid detected in 80/384 (20.8%) consistent with prior infection. 91/384 (23.69%) had an anti‐spike titre that fell below the lowest level reported in a health care workers study (<400 after second dose vaccine). Low titre was associated with age ^3^60y (p = 0.018). No clear associations were observed with current CD4 count or CD4 nadir. Participants with history of SARS‐CoV2 infection had higher anti‐spike antibody titres (p < 0.001). 7.8% of participants had an anti‐spike titre of <100. These were more likely to be on immunosuppressants (p = 0.016), have CD4:CD8 ratio <0.5 (p = 0.009) and/or have other medical conditions (p = 0.018).

**Conclusions**: SCAPE‐HIV is ongoing. This preliminary analysis shows high levels of seroconversion in our study population (majority of whom are well controlled on ART) and highlights an inverse relationship between age and antibody responses. Immunosuppressants, receipt of a solid organ transplant, and a low CD4:CD8 ratio may all be indicators of poor/no response to SARS CoV2 vaccination. It remains to be determined how antibody titres correlate with functional protection against reinfection and cross protection against variants of concern, especially in people with suboptimal serological responses.

### PEMOA34 Seroprevalence of cross‐reactive anti‐SARS‐CoV‐2 antibodies in pre‐COVID‐19 samples collected from Cameroonian women during pregnancy and at delivery

R. Seumko'o^1,2^, H. Tene^1,3^, C. Nana^1,2^, P. Gwanmesia^1^, O. Kenji Mfuh^1,4^, M. Besong^1^, B. Zambo^1,2^, M. Nana^1,2^, E. Mpoudi Ngole^5^, J. Bigoga^1,3^, R. Leke^1,5^, R. Magnekou^1,2^, F. Esemu Livo ^1,5,6^

^1^University of Yaounde I, The Biotechnology Center, Yaounde, Cameroon, ^2^University of Yaounde I, Department of Animal Biology and Physiology, Yaounde, Cameroon, ^3^University of Yaounde I, Department of Biochemistry, Yaounde, Cameroon, ^4^Stanford Medicine, Stanford Health Care, Palo Alto, United States, ^5^Insititute Medical Research and Medicinal Plant Studies, Center For Research on Emerging and Reemerging Diseases, Yaounde, Cameroon, ^6^University of Buea, Faculty of Health Sciences, Buea, Cameroon

**Background**: TheCOVID‐19 Pandemic still causes significant morbidity and mortality worldwide. Significantly more cases of COVID‐19 and its related deaths are reported in High‐Income countries compared to Low‐ and Middle‐Income Countries (LMIC). This might be due to pre‐existing cross‐reactive antibodies to other human coronaviruses in the LMIC setting. It is also unclear whether these antibodies circulate amongst pregnant women and can be acquired transplacentally. Our study aimed to determine the seroprevalence of cross‐reactive anti‐SARS‐CoV‐2 antibodies in pre‐COVID‐19 samples collected during pregnancy and at delivery from women in three settlements in Cameroon.

**Methods**: A total of 1,711 archival plasma from pregnant women and 84 cord blood plasma collected from 2009 to 2019, were tested for COVID‐19 using the Abbott Panbio ^TM^COVID‐19 IgG/ IgM rapid diagnostic test that captures antibodies to viral N protein. Samples from 128 (7.5%) women were collected from the rural area, 1115 (65.2%) in the peri‐urban area, and 468 (27.3%) in an urban area at different antenatal visits. Samples from the peri‐urban area were split into pregnancy (293) and delivery (876) arms. Data was summarized in proportions.

**Results**: Overall, 13.7% (235/1711) of pregnant women were seropositive for COVID‐19, among whom, 27.3% (35/128) were from rural areas, 14.3% (160/1115) from peri‐urban areas, and 22.0% (103/468) from urban areas. During pregnancy 22.8% (8/35), 2.2% (1/46), 30.09% (31/103) of the women were IgG positive while 2.8 % (1/35), 2.2% (1/46), 3.9% (4/103) of pregnant women were seropositive for both IgG and IgM in rural areas, peri‐urban and urban respectively. At delivery, the seroprevalence of IgG, IgM, and both were 13.1% (15/114), 83.3% (95/114), and 3.5% (4/114) respectively. Transplacental transfer of cross‐reactive anti‐ SARS‐CoV‐2 IgG was found in 5.9 % (5/84) of these women.

**Conclusions**: This study provides evidence of existing of cross‐reactive anti‐SARS‐CoV‐2 antibodies among pregnant Cameroonian women in the Pre‐COVID‐19 era and that these antibodies can be transferred transplacentally. However, the protective nature of these antibodies should be investigated further.

### PEMOA35 Side‐by‐side comparison of SARS‐CoV‐2 neutralizing antibody responses after various COVID‐19 vaccine regimens

D. Planas ^1^, I. Staropoli^1^, F. Guivel‐Benhassine^1^, F. Porrot^1^, D. Veyer^2^, H. Péré^2^, C. Planchais^1^, A.P. Pessoa Vilela^3^, S. Maia Acuña^3^, M. dos Passos Cunha^3^, J. Hadjad^4^, B. Terrier^4^, A. Seve^5^, H. Mouquet^1^, P. Minoprio^3^, T. Prazuck^5^, L. Hocqueloux^5^, T. Bruel^1^, O. Schwartz^1^

^1^Institut Pasteur, Virology, Paris, France, ^2^Hôpital Européen Georges Pompidou, Laboratoire de Virologie, Service de Microbiologie, Paris, France, ^3^Institut Pasteur, São Paulo, Brazil, ^4^Hôpital Cochin, Department of Internal Medicine, Paris, France, ^5^CHR d'Orléans, Infectious Diseases, Orléans, France

**Background**: Waning immunity and emergence of SARS‐CoV‐2 variants impact COVID‐19 vaccine efficacy. Here, we studied longitudinally the humoral response induced by Pfizer, AstraZeneca, Janssen, Coronavac and Sputnik Vaccines, with or without booster doses. We also asked how breakthrough Omicron infection in Pfizer‐vaccinated individuals enhances antibody levels and cross‐reactivity.

**Methods**: We analyzed 349 sera from individuals immunized with five vaccines, Pfizer/BioNTech (BNT162b2), AstraZeneca (ChAdOx1 nCoV‐19), Janssen (Ad26COV2.S), Sinovac biotech (Coronavac) or Sputnik (Gam‐COVID‐Vac). We also examined in 92 sera the impact of a Pfizer booster dose in individuals immunized with Pfizer, Janssen or Sinovac regimens. Samples were collected up to 13 months after the first injection, and 5 months after the boost. We measured anti‐S antibodies by flow cytometry with the S‐Flow assay, and neutralization titers against infectious D614G, Alpha, Beta, Delta and Omicron isolates.

**Results**: Administration of two doses of Pfizer, AstraZeneca, Sputnik vaccines, or an heterologous AstraZeneca/Pfizer regimen, induced seroconversion of 95% of individuals and neutralization activity against D614G, Alpha, Beta and Delta, but not Omicron. Janssen and Sinovac vaccines elicited lower levels of anti‐S antibodies, and no detectable neutralization of Delta and Omicron. During the first 8 months, the antibody levels and neutralization activity progressively declined with all vaccines. A booster dose of Pfizer strongly increased antibody response and elicited neutralizing antibodies against Omicron. However, titers were 8‐ to 36‐ fold lower against Omicron relative to Delta. We observed a waning of the humoral response after the boost and estimated that neutralizing antibodies against Omicron will no longer be detectable in the sera after 6 months. Breakthrough Omicron infections strongly increased the levels of cross‐reactive antibodies with titers only 2.5‐fold lower against Omicron compared to Delta.

**Conclusions**: Our results highlight differences between vaccines and support the use of an mRNA‐based vaccine as a booster regardless of prior regimens. The duration of the neutralizing humoral response after the boost is estimated to be about 6 months. A high level of cross‐reactivity is observed in Omicron breakthrough cases. Our data suggest that an Omicron‐specific booster may improve cross‐immunity.

### PEMOA36 Immunogenicity of the BNT 162b2 mRNA vaccine against COVID‐19 variants in people living with HIV on antiretroviral therapy in Malaysia

R. Rajasuriar ^1^, A. Kukreja^1^, N.S. Zulhaimi^2^, C.Y. Loh^2^, C.S. Lee^2^, W.Y. Ho^1^, S. Shaharuddin^1^, W.M.H. Wan Alias^1^, J. I‐Ching^3^, S. Hisham^1^, A. Ahsan^1^, S.F. Syed Omar^1^, A. Kamarulzaman^1^

^1^Centre of Excellence for Research in AIDS, University of Malaya, Kuala Lumpur, Malaysia, ^2^Immunotherapeutics Laboratory, Faculty of Medicine, University of Malaya, Kuala Lumpur, Malaysia, ^3^University of Malaya, Department of Medical Microbiology, Kuala Lumpur, Malaysia

**Background**: Few studies have explored the immunogenicity of COVID‐19 vaccines in people living with HIV (PLWH) from low‐middle income settings where late presentation to care may lead to persistent immune deficiencies despite antiretroviral therapy (ART). In this study, we compared humoral immune responses following BNT162b2 mRNA primary vaccination in PLWH on ART to age‐matched controls and explored the clinical correlates of immunogenicity.

**Methods**: This prospective study recruited PLWH on ART and HIV uninfected controls (≥18 years) attending vaccination appointments at University Malaya Medical Centre, Malaysia. All participants received two doses of the BNT162b2 vaccine 21 days apart and had serum collected at D0 and D35 after dose 1. Immunogenicity was assessed by anti‐S1 antibody levels (Elecsys Anti‐SARS‐CoV‐2 S assay, Roche) and percent inhibition of neutralization against wild‐type D614G, beta and delta variants using cPass SARS‐CoV‐2 Surrogate Virus Neutralization Test (GenScript). Participant characteristics were extracted from medical records. Clinical factors associated with immunogenicity markers were assessed using univariate non‐parametric analyses.

**Results**: A total of 68 PLWH and 52 HIV‐negative controls were recruited with a median age of 37 years. The majority were males (PLWH = 96%, controls = 64%).94% of PLWH had HIV RNA < 50 copies/ml. Median nadir and current CD4 T‐cell counts were 182 (44–351) cells/ul and 554 (361–790) cells/ul, respectively. All participants were seropositive (>0.8IU/ml) following vaccination and no difference in median anti‐S1 antibody levels were observed at D35 in both groups (p = 0.871). Neutralising activity was lower in PLWH compared to controls for the beta (73.1% vs 83.1%, p < 0.001) and delta variants (92.7% vs 95.1%, p = 0.008) but not significantly different for the D614G variant. Older PLWH (≥45 years), CD4 T‐cell counts <800 cells/ul and CD4:CD8 ratio <0.8 were associated with significantly lower inhibition activity against the beta and delta variants compared to controls (p < 0.05). These factors however, had no impact on anti‐S1 levels and inhibition activity against the wild‐type D614G variant.

**Conclusions**: Older PLWH with suboptimal immune recovery on ART had lower immunogenicity compared to age‐matched controls against variants of concern and should be prioritized for SARS‐CoV‐2 vaccine boosters. Larger studies are needed to examine clinical correlates of vaccine effectiveness among PLWH on ART.

### PEMOA37 Vaccine scheme, age and previous COVID‐19 predict humoral response to SARS‐CoV‐2 vaccination in HIV‐infected indivudals

P. Gantner ^1^, A. Ursenbach^2^, C. Cheneau^3^, Y. Ruch^2^, C. Melounou^3^, P. Fischer^3^, F. Danion^2^, C. Bernard‐Henry^3^, N. Lefebvre^2^, E. Rougier^2^, C. Kaeuffer^2^, Y. Hansmann^2^, M.‐J. Wendling^1^, N. Meyer^4^, S. Fafi‐Kremer^1^, D. Rey^3^

^1^Université de Strasbourg / Faculté de Médecine, Medical Virology, Strasbourg, France, ^2^Hôpitaux Universitaires de Strasbourg, Infectious Diseases, Strasbourg, France, ^3^Hôpitaux Universitaires de Strasbourg, HIV‐Infection Care Center, Strasbourg, France, ^4^Hôpitaux Universitaires de Strasbourg, GMRC, Strasbourg, France

**Background**: Humoral response to SARS‐CoV‐2 vaccination in HIV‐infected individuals on successful antiretroviral therapy (ART) remain undercharacterized. Here, weobtained anti‐SARS‐CoV‐2 Receptor binding domain (anti‐RBD) and neutralization titers detected after vaccination.

**Figure:**
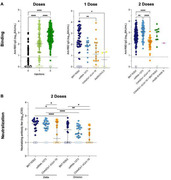
**Abstract PEMOA37‐Figure 1**.

**Methods**: We collected longitudinal serum samples (pre‐ [D0] and post‐vaccination era [M6]) from 447 participants (>200 CD4/mm^3^) enrolled in the SECOVIHA study (NCT04515225, Strasbourg, France). We measured anti‐RBD and neutralizing antibody titers (Delta and Omicron variants pseudoparticle‐based assay), and analyzed predictors of humoral response.

**Results**: At D0 8.9% of individuals had developed COVID‐19 and an additional 9.7% were subsequently infected at M6. Women were at higher risk of COVID‐19 (logistic regression, p < 0,01). At M6, 132, 93 and 222 participants had received 0, 1 or 2 doses of vaccine, respectively **(Fig 1A)**. Median anti‐RBD titers were of 2.0 and 3.2 log_10_binding antibody unit [BAU]/mL after 1 and 2 doses. Adjusting for time since last vaccine dose, a multivariate analysis showed that an older age was associated to lower anti‐RBD titers (−0.09 log_10_BAU/mL per 10‐year increase) and COVID‐19 prior to vaccination was associated with higher titers (+0.98 log_10_BAU/mL). Decreased titers were associated to an incomplete vaccine scheme (−1.06, −0.81 and −1.87 log_10_BAU/mL for 1 dose of BNT162b2, mRNA‐1273 and ChAdOx1nCov19, respectively) and 2 doses of ChAdOx1nCov19 vaccination (−0.27 log_10_BAU/mL) but not with 2 doses of mRNA‐1273 or heterologous ChAdOx1nCov19/ BNT162b2 compared to 2 doses of BNT162b2. No HIV‐related parameters were associated to humoral response. Delta and Omicron‐neutralizing antibody titers from paired participants receiving 2 doses of BNT162b2, mRNA‐1273 or ChAdOx1nCov19 (n = 24 each) showed a higher neutralizing ability after mRNA‐based vaccination and a weaker Omicron‐neutralizing ability compared to Delta **(Fig 1B)**.

**Conclusions**: Collectively, these data indicate that determinants of anti‐SARS‐CoV‐2 humoral response in HIV‐infected individuals on succesfull ART seem similar to those observed in the general population.

### PEMOA38 People living with HIV who are receiving suppressive antiretroviral therapy mount strong humoral responses to two and three doses of COVID‐19 vaccine

H.R. Lapointe ^1^, F. Mwimanzi^2^, P.K. Cheung^1,2^, Y. Sang^2^, F. Yaseen^3^, S. Speckmaier^1^, N. Moran‐Garcia^1^, R. Kalikawe^2^, L. Young^4^, G. Umviligihozo^2^, F.H. Omondi^1,2^, M.C. Duncan^1,2^, S. Datwani^2^, O. Agafitei^2^, S. Ennis^2^, K. Ng^2^, H. Ali^5^, B. Ganase^6^, H. Sudderuddin^1,7^, J. Toy^1^, P. Sereda^1^, L. Burns^8^, C.T. Costiniuk^9^, C. Cooper^10,11^, A.H. Anis^12,13,14^, V. Leung^4,15^, D. Holmes^8,15^, M.L. DeMarco^8,15^, J. Simons^8,15^, M. Hedgcock^16^, N. Prystajecky^15,17^, R. Pantophlet^2,3^, C. Lowe^4,15^, M.G. Romney^4,15^, R. Barrios^1,12^, S. Guillemi^1,7,18^, C.J. Brumme^1,7^, J.S.G. Montaner^1,7^, M. Hull^1,7^, M. Niikura^2^, M. Harris^1,18^, M.A. Brockman^1,2,3^, Z.L. Brumme^1,2^

^1^BC Centre for Excellence in HIV/AIDS, Vancouver, Canada, ^2^Simon Fraser University, Faculty of Health Sciences, Burnaby, Canada, ^3^Simon Fraser University, Department of Molecular Biology and Biochemistry, Burnaby, Canada, ^4^St. Paul's Hospital, Division of Medical Microbiology and Virology, Vancouver, Canada, ^5^St. Paul's Hospital, John Ruedy Clinic, Vancouver, Canada, ^6^St. Paul's Hospital, AIDS Research Program, Vancouver, Canada, ^7^University of British Columbia, Department of Medicine, Vancouver, Canada, ^8^Providence Health Care, Department of Pathology and Laboratory Medicine, Vancouver, Canada, ^9^The Research Institute of the McGill University Health Centre, Division of Infectious Diseases and Chronic Viral Illness Service, Montreal, Canada, ^10^University of Ottawa, Department of Medicine, Ottawa, Canada, ^11^Ottawa Hospital Research Institute, Ottawa, Canada, ^12^University of British Columbia, School of Population and Public Health, Vancouver, Canada, ^13^University of British Columbia, CIHR Canadian HIV Trials Network, Vancouver, Canada, ^14^Centre for Health Evaluation and Outcome Sciences, Vancouver, Canada, ^15^University of British Columbia, Department of Pathology and Laboratory Medicine, Vancouver, Canada, ^16^Spectrum Health, Vancouver, Canada, ^17^British Columbia Centre for Disease Control Public Health Laboratory, Vancouver, Canada, ^18^University of British Columbia, Department of Family Practice, Faculty of Medicine, Vancouver, Canada

**Background**: Immune responses to COVID‐19 vaccines, particularly to third doses, remain incompletely characterized in people living with HIV (PLWH).

**Methods**: We are monitoring immune responses to COVID‐19 vaccination in a cohort of 99 adult PLWH and 152 controls, aged 22–88 years, in British Columbia, Canada. All PLWH were receiving suppressive ART, with median CD4+ T‐cell counts of 715 (Q1‐Q3 545–943) cells/mm^3^at cohort entry. In samples collected one month after the second and third COVID‐19 vaccine doses, we quantified serum antibodies against the SARS‐CoV‐2 spike protein receptor‐binding domain (RBD) using the Roche Elecsys anti‐SARS‐CoV‐2 assay, and measured viral neutralization activity in plasma against the original (USA‐WA1/2020) and Omicron (BA.1) SARS‐CoV‐2 strains.

**Results**: We previously reported that, at one month following the second COVID‐19 vaccine dose, and after adjustment for sociodemographic, health, and vaccine‐related variables, HIV infection was not associated with a difference in either anti‐RBD antibody concentration nor live virus neutralization activity compared to responses observed in controls. In PLWH, there was no significant correlation between the most recent (or nadir) CD4+ T‐cell count and vaccine responses after two doses. Rather, at this timepoint, older age, a higher burden of chronic health conditions, and having received two ChAdOx1 doses (as opposed to mRNA or heterologous regimens) were associated with lower responses. One month following the third dose, anti‐RBD serum antibodies increased by 0.4 log_10_higher on average, and viral neutralization by four‐fold higher on average, than values observed one month after the second dose, in both PLWH and controls (Wilcoxon paired test p < 0.0001 for all within‐group comparisons between time‐points). Importantly, there was no significant difference between PLWH and controls in terms of the magnitudes of post‐3rd‐dose responses. In a subset of 24 participants assessed to date, the ability to neutralize Omicron after three doses was on average 8‐fold lower than the pandemic founder strain (p < 0.0001).

**Conclusions**: In PLWH with well‐controlled viral loads on therapy and CD4+ T‐cell counts in a healthy range, humoral responses to two and three COVID‐19 vaccine doses are comparable to individuals without HIV. Third COVID‐19 vaccine doses induce responses capable of neutralizing Omicron to some extent.

### PEMOB27 Persistent Low‐Level Viremia in the Era of Dolutegravir in Four African Countries

J. Cavanaugh ^1^, N. Dear^2,1^, A. Esber^2,1^, N. Shah^1^, M. Iroezindu^3,1^, E. Bahemana^4,1^, H. Kibuuka^5^, J. Owuoth^6^, J. Maswai^7^, V. Singoei^8^, C. Polyak^1,2^, J. Ake^1^

^1^Walter Reed Army Institute of Research, Military HIV Research Program, Silver Spring, United States, ^2^Henry Jackson Foundation, Bethesda, United States, ^3^Henry Jackson Foundation Medical Research International, Abuja, Nigeria, ^4^Henry Jackson Foundation Medical Research International, Mbeya, Tanzania, The United Republic of, ^5^Henry Jackson Foundation Medical Research International, Kampala, Uganda, ^6^Henry Jackson Foundation Medical Research International, Kericho, Kenya, ^7^US Army Medical Research Directorate ‐ Africa, Nairobi, Kenya, ^8^Henry Jackson Foundation Medical Research International, Kisumu, Kenya

**Background**: HIV programs frequently use a viral load (VL) <1000 copies/mL as the threshold for VL suppression (VLS). Consequently, persistent low‐level viremia (pLLV), VL between 50–999 copies/mL on at least two consecutive measurements, is often undetected even though it has been associated with worse clinical outcomes.

**Methods**: The African Cohort Study (AFRICOS) enrolls people living with HIV (PLWH) who are engaged in routine HIV care in Uganda, Kenya, Tanzania and Nigeria. Semiannually, participants come to study sites for more intensive evaluation, including quantified VL testing. In this analysis, we included participants who were taking tenofovir/lamivudine/dolutegravir (TLD) for at least three months and had two subsequent VL measurements; we used the two most recent VL measurements. We calculated frequencies for VLS using the number of participants with two VL measurements as the denominator. We documented participants with VL >1000 copies/mL and stratified results for those with VL <1000 copies/mL by: both VL measurements <50 copies/mL, pLLV, one VL 50–999 copies/mL and one VL <50 copies/mL. We further stratified results by site, age group, and sex. We used Pearson's Chi‐squared and Fisher's exact tests to compare frequencies.

**Results**: Between January 2013 and November 2021, 1439 participants were eligible. Across all sites, 1388 participants had VL <1000 copies/mL on both tests, 28 (2.0%) of whom had pLLV. Three participants were above 1000 copies/mL on both VL tests, 39 had one VL <50 copies/mL and one VL ≥1000 copies/mL, and 9 had one VL 50–999 copies/mL and one VL ≥1000 copies/mL. Prevalence of pLLV, by strata, was highest in Nigeria, among young adults, and among males (Table 1).

**Table:** 

**Abstract PEMOB27‐Table 1**.							
	N	Both tests <50 c/mL^*^	p‐value	Persistent low‐level viremia^*^	p‐value	One test <50 c/mL and one test 50–999 c/mL^*^	p‐value
Uganda	193	165 (85.5%)	<0.001	3 (1.5%)	<0.001	21 (10.9%)	<0.001
Kenya	777	704 (90.6%)		5 (0.6%)		48 (6.2%)	
Tanzania	264	195 (73.9%)		7 (2.6%)		49 (18.6%)	
Nigeria	205	130 (63.4%)		13 (6.3%)		48 (23.4%)	
15–24 years	64	43 (67.2%)	<0.001	3 (4.7%)	0.091	10 (15.6%)	<0.001
25–49 years	844	679 (80.4%)		19 (2.2%)		119 (14.1%)	
50+ years	531	472 (88.9%)		6 (1.1%)		37 (7.0%)	
Male	717	575 (80.2%)	0.005	19 (2.6%)	0.054	94 (13.1%)	0.061
Female	722	619 (85.7%)		9 (1.2%)		72 (10.0%)	

**Conclusions**: Studies to investigate associations with unsuppressed and pLLV are needed in these populations, including documentation of differences in viral genomics (e.g., viral subtypes and drug resistance mutations) and local participant demographic factors associated with adherence.

### PEMOB28 Safety Outcomes Among HIV‐1 Positive Zambian Adults Receiving Tenofovir Alafenamide Combined With Dolutegravir: Results From The VISEND Clinical Trial

D. Engamba ^1,2^, A. Kumar^1^, N. Mbewe^1,2^, S. Fwoloshi^3,2,4^, G. Phiri^1^, A. Mweemba^5,4^, S. Sivile^1,4,2^, M. Siwingwa^1,6^, D. Kampamba^1,4^, B. Simons^7^, A. Hill^8^, L. Chirwa^1^, C.W. Wester^9,10^, L. Mulenga^1,2,4,10^

^1^University Teaching Hospital, Adult Infectious Diseases Center, Lusaka, Zambia, ^2^University of Zambia, Internal Medicine, Division of Infectious Diseases, Lusaka, Zambia, ^3^University Teaching Hospital, Infectious Diseases, Lusaka, Zambia, ^4^Ministry of Health, Lusaka, Zambia, ^5^Levy Mwanawasa Medical University Teaching Hospital, Internal Medicine, Lusaka, Zambia, ^6^University of Zambia, Department of Medicine, Lusaka, Zambia, ^7^Imperial College, Faculty of Medicine, London, United Kingdom, ^8^Liverpool University, Department of Translational Medicine, Liverpool, United Kingdom, ^9^Vanderbilt University Medical Center (VUMC), Department of Medicine, Division of Infectious Diseases, Nashville, United States, ^10^Vanderbilt Institute for Global Health (VIGH), Nashville, United States

**Background**: Tenofovir disoproxil fumarate (TDF), isassociated with higher risks of renal and bone adverse events, a reason why the WHO had recommended the use ofTenofovir alafenamide (TAF) as a favorable option especially in those with preexisting renal or bone comorbidities. However, there has been limited use of TAF in resource limited settings with scanty data on its safety especially among pregnant women. We thus evaluated the ARVsafety among HIV‐positive, Zambian adults receiving TDF/lamivudine(3TC)/dolutegravir (DTG) or TAF/emtricitabine (FTC)/DTG after being switched from non‐nucleoside reverse transcriptase inhibitors (NNRTI)‐based ART.

**Methods**: The VISEND trial is a 144 week, randomized, open label, phase 3 noninferiority study in which weenrolled HIV‐1 positive Zambian adults individuals who were receiving TDF/lamivudine (3TC) /efavirenz (EFV) or nevirapine (NVP) ART. Individuals receiving TDF/3TC/EFV400 or TDF/3TC/NVP with baselineHIV‐1 RNA < 1,000 copies/mL (arm A) and those with baseline HIV‐1 RNA≥1,000 copies/mL (Arm B) wererandomizedto either TDF/3TC/DTG or TAF/FTC/DTG. Participants who became pregnant after study enrollment were maintained on the study medicines. Safety was monitored using various biomarkers including serum creatinine with creatinine clearance (CrCl) calculated using the cock‐croft gault equation. Creatinine clearance < 50mLs/min and <30 mL/min warranted TDF and TAF discontinuations respectively.

**Results**: 837 were randomized toTDF/3TC/DTG or TAF/FTC/DTG. At Week 48, eight (8) individuals receiving TLD were discontinued due to kidney events and 2 due to bone related events. Individuals receiving TAFED had a slightly higher mean weight gain (BMI change +1.84) compared to those on TDF/3TC/DTG (BMI change +1.54) although higher among the female. Twenty‐four (24) participants (from both arms) became pregnant with 19 (79%) viable babies delivered. There were 4 miscarriages (2 on TDF/3TC/DTG and 2 on TAF/FTC/DTG). 1 whose mother was on TDF/3TC/DTG had congenital cardiac anomaly.

**Conclusions**: Individuals on TAF/FTC/DTG had better renal and bone safety profiles whereas pregnancy outcomes were comparable in both groups. TAFED was associated with increased weight gain. Tenofovir alafenamide offers a choice for an ARV with balancedoptimal efficacy and potential for improved long‐term safety among HIV positive individuals on ART. However, long term follow up is needed to ascertain metabolic complications in women.

### PEMOB29 Selection of Cabotegravir Dosing Regimens for HIV Treatment and Pre‐exposure Prophylaxis (PrEP) in Adolescents by Leveraging Adult Data

K. Han^1^, N. Goyal^1^, S. Ford^2^, S.Y.A. Cheung^1^, L. Tan^3^, C. McCoig^4^, P. Patel^5^, E. Capparelli^6^, B. Best^6^, M. Marzinke^7^, C. Bolton Moore^8,9^, A.H. Gaur^10^, C. Harrington^5^, D. Izard^1^, J. Huang^1^, M. Baker ^11^

^1^GlaxoSmithKline, Collegeville, United States, ^2^GlaxoSmithKline, Research Triangle Park, United States, ^3^ViiV Healthcare, Brentford, United Kingdom, ^4^ViiV Healthcare, Madrid, Spain, ^5^ViiV Healthcare, Research Triangle Park, United States, ^6^University of California San Diego, La Jolla, United States, ^7^Johns Hopkins University, Baltimore, United States, ^8^Centre for Infectious Disease Research in Zambia, Lusaka, Zambia, ^9^University of Alabama at Birmingham, Birmingham, United States, ^10^St. Jude Children's Research Hospital, Memphis, United States, ^11^ViiV Healthcare, Nyon, Switzerland

**Background**: Cabotegravir (CAB) is the first complete long‐acting (LA) regimen for HIV treatment (with rilpivirine) in adults and PrEP in adults and adolescents (≥35kg). CAB LA is an important alternative to daily oral regimens for adolescents. Oral and LA CAB pharmacokinetics (PK) and safety were characterized in virologically‐suppressed adolescents (≥35kg) on stable combination antiretroviral therapy in the ongoing MOCHA study. Population PK (PPK) modelling was used to support extrapolation from adults to adolescents and bridge dose regimens and therapeutic use.

**Methods**: Interim PK from MOCHA (8 adolescents) was compared to adult PK to establish PK similarity and determine feasibility of extrapolation. Adolescent PK profiles following monthly and every‐2‐months regimens were simulated by incorporating adolescent weight and BMI in a previously developed adult PPK model, and compared to adult PK targets.

**Results**: Observed and predicted adolescent PK were similar to that in adults receiving treatment or PrEP regimens (Figure 1). Adult regimens in adolescents had resulting PK most similar to adults with similar weight range. PPK model accurately predicted PK from adolescents ≥35kg and bridged dose frequency and HIV‐infection status. The observed and simulated data demonstrated that adolescents, receiving adult CAB regimens, remained above PK targets observed in adults and below safety thresholds (Figure 2).

**Figure:**
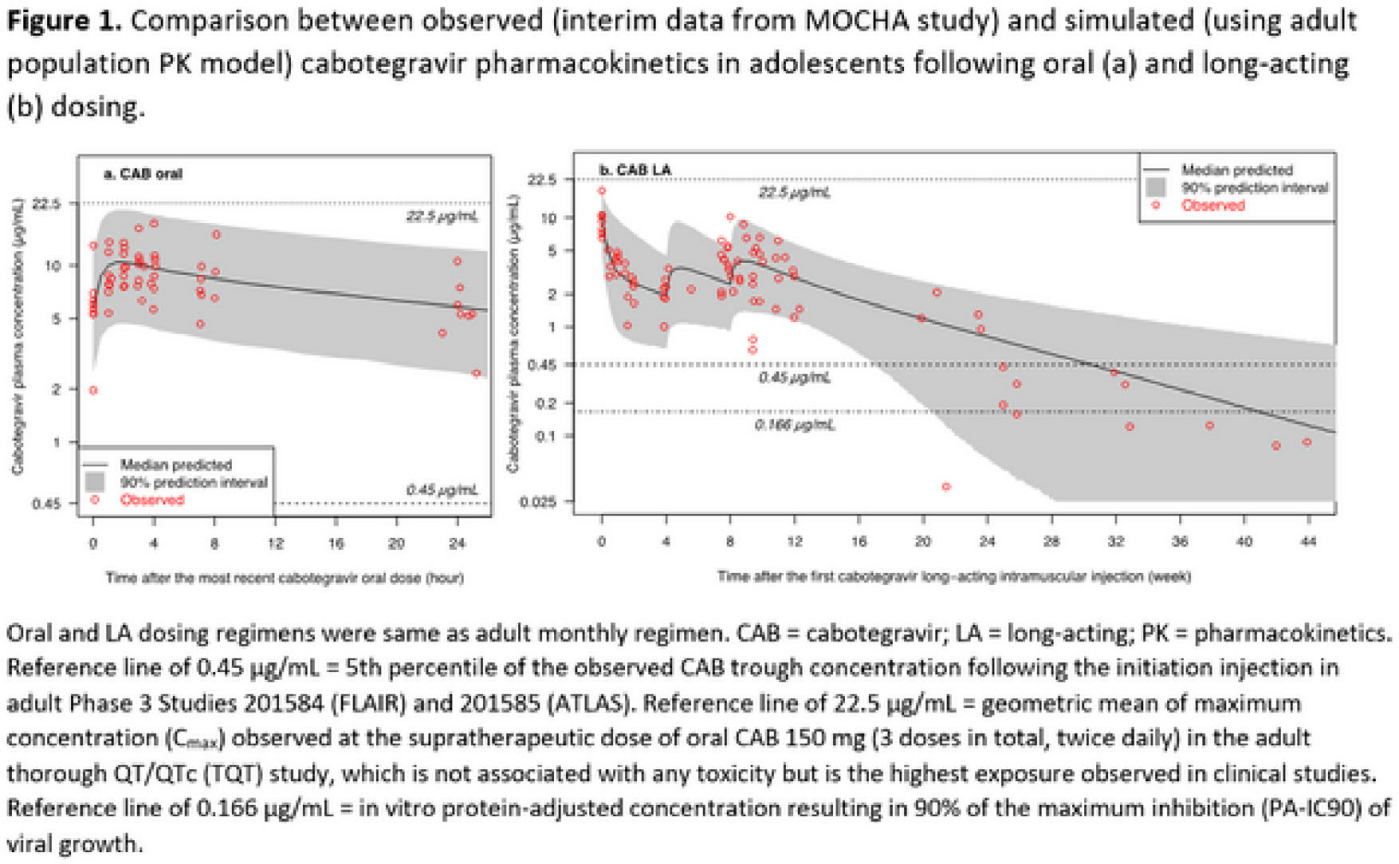
**Abstract PEMOB29‐Figure 1**.

**Figure:**
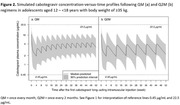
**Abstract PEMOB29‐Figure 2**.

**Conclusions**: Robust modelling and simulation approaches, combined with adolescent PK data from MOCHA, allowed bridging and extrapolation across several factors, and inform future study design. The consistency of adolescent and adult PK supports the use of adult CAB regimens in adolescents ≥12 years and ≥35 kg.

### PEMOB30 Optimizing DTG uptake amongst Children Living with HIV in Ikom rural communities attending ART clinic at Comprehensive Health Center (CHC) Ikom, Cross River State

E. Chukwu ^1^, D.L. Buzaalirwa^2^, D.V. Poopola^3^, D.E. Nwabueze^3^, D.I. Udenkwo^4^, C. Michael^5^, P.F. Lumenze^6^, J.U. Okey^6^, R. Okon^6^, S. Akanbi^6^, I. Ubi^6^, N. Oto‐Obong^6^, I. Igbebor^6^, S. Usang^4^, A. Ene^4^, I. Essien^4^, E. Agada^4^, P. Ochinokwu^6^, Dr Victor Poopola Dr Emmanuel Nwabueze Charles Micheal Dr Ifeanyi Udenkwo Pharm Frank Lumenze Solomon Akanbi Idang Essien Joseph Ugar Okey Aruk Ene Rebecca Okon Oto‐Obong Ntekim Itam Igbebor Stanley Usang Janet Ogar Esther Obogo Alice Erim Eyiwa Dominic

^1^AIDS Healthcare Foundation, Nigeria, Medical laboratory science, Ikom, Nigeria, ^2^AIDS Healthcare Foundation, Kampala, Uganda, ^3^AIDS Healthcare Foundation, Nigeria, Abuja, Nigeria, ^4^AIDS Healthcare Foundation, Nigeria, Calabar, Nigeria, ^5^AIDS Healthcare Foundation, Nigeria, Medical laboratory science, Abuja, Nigeria, ^6^AIDS Healthcare Foundation, Nigeria, Ikom, Nigeria

**Background**: Treatment of HIV infection among children remains very challenging due to several reasons, including difficulties surrounding the availability of effective, palatable, dosage and storage friendly Antiretroviral medications. The introduction of pediatric friendly dolutegravir (DTG) based regimen addressed some of the challenges with previous regimens and this project worked on optimizing its uptake among children living with HIV in rural communities in Ikom Cross River State.

**Description**: Description

Most of the rural communities in Cross river state are hard to reach making it difficult for children in these communities to have access to effective treatment. Also, due to experience with previous unpalatable regimen, adherence among them was not optimal. To address this challenge, this project was commissioned in one of the rural clinics, CHC Ikom supported by AIDS Healthcare Foundation. Uptake of the newly introduced DTG was found to be low (25%) amongst children attending ART clinics in CHC Ikom despite being available on site for more than 3 months. Viral suppression among the children was about 35%. Strategies employed include capacity building on DTG administration for healthcare workers, weekly facility‐based treatment literacy sessions for caregivers, geographical mapping of the children in care, followed by intensive follow‐up through home visits.

**Lessons learned**: After 6months of this project, DTG uptake among the children increased to 100% and the clinical scores among the children improved significantly. Adherence increased to over 85%, viral load suppression among the children increased to 90% and there was no report of any adverse drug reactions. There was no record of missed appointment within the period and most of their care givers understand better how ART treatment works. Access to treatment also improved among the children due to the geographical mapping and availability of differentiated services for them

**Conclusions/Next steps**: Conclusion/Next steps: Uptake of key interventions like Pediatric DTG is critical for improved treatment outcome for children living with HIV and should be offered in a comprehensive manner. A tripartite strategy of healthcare worker capacity building, Treatment literacy sessions for caregivers and geographical mapping followed by intensive follow up is recommended to improve DTG uptake among Children living with HIV in rural communities.

### PEMOB31 The cognitive development of children born to adolescent mothers – does child HIV status matter?

K. Roberts ^1^, L. Sherr^1^, K. Haag^1^, C. Smith^1^, J. Jochim^2^, E. Toska^3^, M. Marlow^4^, L. Cluver^2,3^

^1^University College London, Institute for Global Health, London, United Kingdom, ^2^University of Oxford, Oxford, United Kingdom, ^3^University of Cape Town, Cape Town, South Africa, ^4^Stellenbosch University, Stellenbosch, South Africa

**Background**: HIV, both directly and indirectly, impacts child development outcomes. The most severe impacts are for children infected with HIV, and those exposed but uninfected are also shown to have challenges – though less severe. However, little is known regarding the development of children born to adolescent mothers affected by HIV. This study aims to examine cognitive development for children born to adolescent mothers, comparing those children living with HIV, those HIV exposed and uninfected (HEU) and those HIV unexposed (HU).

**Methods**: Analyses utilise cross‐sectional data from 920 adolescent mother (10–19 years)‐first born child dyads residing in the Eastern Cape Province, South Africa. Participants completed detailed study questionnaires relating to sociodemographic characteristics, HIV, and maternal and child health. Trained assessors administered standardised child development assessments (Mullen Scales of Early Learning). Chi‐square tests and ANOVA tests were used to explore maternal and child characteristics according to child HIV status on cognitive development. Linear regression models were used to explore associations between child HIV status and child cognitive development.

**Results**: 1.2% of children were living with HIV, 20.5% were classified as being HEU and, 78.3% were classified as HU. Overall, children living with HIV were found to perform lower across developmental domains compared to both HEU and HU groups (composite score of early learning: 73.0 vs 91.2 vs. 94.1, respectively: *F* = 6.45, p = 0.001). HEU children on average scored lower on all developmental domains compared to HU children, reaching significance on the gross motor domain (p < 0.05). Exploratory analyses identified maternal education interruption as a potential risk factor for lower child cognitive development scores and, higher maternal age to be protective of child cognitive development scores.

**Conclusions**: Analyses identify stepwise differences in the average child cognitive development scores according to child HIV status among children born to adolescent mothers affected by HIV; with children living with HIV performing worse overall. Young mothers and their children may benefit from adapted interventions aimed at bolstering child development outcomes. Targeted programming particularly among younger adolescent mothers and those experiencing education interruption may identify those families, particularly in need.

### PEMOB32 No difference in prevalence of TB infection among infants in two Southern African cohorts by *in utero* HIV exposure status

S.C. Iwase ^1^, P.T. Edlefsen^2^, L. Bhebhe^3^, K. Motsumi^3^, S. Moyo^3,4^, A.‐U. Happel^1^, N. Mmasa^5^, S. Schenkel^6^, M.A. Gasper^7^, M. Dubois^8^, F. Duffy^9^, J. Aitchison^9^, M.G. Netea^10,11^, J. Jao^3,12,13^, D.W. Cameron^14^, C.M. Gray^15^, H.B. Jaspan^1,16^, K.M. Powis^3,4,17^

^1^University of Cape Town, Division of Immunology, Institute of Infectious Disease and Molecular Medicine, Cape Town, South Africa, ^2^Fred Hutchinson Cancer Research Center, Statistical Center for HIV/AIDS Research and Prevention, Vaccine and Infectious Disease Division, Seattle, United States, ^3^Botswana Harvard AIDS Institute Partnership, Gabarone, Botswana, ^4^Harvard T.H. Chan School of Public Health, Department of Immunology and Infectious Diseases, Boston, United States, ^5^Darlington Memorial Hospital, County Durham and Darlington NHS trust, Surgical Department, England, United Kingdom, ^6^Massachusetts General Hospital, Department of Pediatric Global Health, Boston, United States, ^7^Seattle Children's Research Institute, Seattle, United States, ^8^Boston Children's Hospital, Division of Infectious Diseases, Department of Pediatrics, Boston, United States, ^9^Seattle Children's Research Institute, Center for Global Infectious Disease Research, Seattle, United States, ^10^Radboud University Medical Center, Department of Internal Medicine and Radboud Center for Infectious Diseases, Nijmegen, Netherlands, the, ^11^University of Bonn, Department of Immunology and Metabolism, Life & Medical Sciences Institute, Bonn, Germany, ^12^Ann and Robert H. Lurie Children's Hospital of Chicago, Department of Pediatrics, Division of Infectious Diseases, Chicago, United States, ^13^Northwestern University Feinberg School of Medicine, Department of Pediatrics, Department of Medicine, Chicago, United States, ^14^University of Ottawa at the Ottawa Hospital, Divisions of Infectious Diseases and Respirology, Ottawa, Canada, ^15^Stellenbosch University, Division of Molecular Biology and Human Genetics, Biomedical Research Institute, Cape Town, South Africa, ^16^Seattle Children's Hospital, Seattle, United States, ^17^Massachusetts General Hospital, Departments of Internal Medicine and Pediatrics, Boston, United States

**Background**: Children under 5 years old are at risk for tuberculous (TB) disease. Bacillus Calmette–Guérin (BCG) mitigates this risk and may also improve all‐cause mortality. However, whether this is the same for infants exposed *in utero* to HIV yet uninfected (iHEU) is unknown.

**Methods**: Leveraging mother‐child health studies conducted in Botswana and South Africa, women living with HIV and without were enrolled during pregnancy. Mother‐child pairs were followed prospectively, and samples collected at 9–12months and/or 18‐month of life in Botswana and 9‐month or 12‐month of life in South Africa were used. All infants were BCG vaccinated at birth. T‐SPOT.TB assays were performed on cryopreserved peripheral mononuclear cells. Cells were stimulated with phytohaemagglutinin (PHA), ESAT‐6, CFP‐10, and medium. Results were interpreted as positive, negative, borderline, or invalid, according to the manufacturer's instruction. For invalid or borderline results, re‐testing was performed using another aliquot collected at the same visit or in follow‐up, if available. Valid re‐tested results from later time points were considered reflective of the initial testing date, acknowledging the potential TB exposure between the tests. Proportions of T‐SPOT.TB positive tests were compared by infant HIV exposure status using Fisher's exact test. Median PHA stimulated spot forming cells were compared using Wilcoxon rank sum test.

**Results**: Overall, 418 infants were tested; 293 (70%) iHEU and 125 (30%) HIV‐unexposed infants (iHUU). Women with HIV were older with higher gravidity compared to women without HIV. No infant presented with TB disease. TB infection prevalence did not differ by infant HIV exposure overall or in either cohort. In Botswana, 6 iHEU (4.4%) and 1 iHUU (3.0%) tested positive (p = 1.0). In South Africa, 4 iHEU (2.5%) and 3 iHUU (3.3%) tested positive (p = 0.71). Two seroreversions occurred in iHEU. South African iHEU showed a significantly higher PHA stimulation response compared to iHUU (median 575 vs. 409 spots; p = 0.004). This was not observed in Botswana (median 289 vs. 426 spots; p = 0.059).

**Conclusions**: TB infection prevalence was similar between two Southern African infant cohorts regardless of fetal HIV exposure status, contrary to previous reports where overall prevalence was higher in infants. Studies with larger cohorts are needed to confirm these findings.

### PEMOB33 Causes of death and hospitalisations in a cohort of sex workers in South Africa

H. Hausler ^1,2^, S. Schwartz^3^, C. Comins^3^, L. Shipp^3^, M. Mcingana^1^, N. Mulumba^1^, S. Makama^1^, S. Baral^3^

^1^TB HIV Care, Cape Town, South Africa, ^2^Univeristy of Pretoria, Family Medicine, Pretoria, South Africa, ^3^Johns Hopkins University, Department of Epidemiology, Johns Hopkins Bloomberg School of Public Health, Baltimore, United States

**Background**: In South Africa, 60% of female sex workers (FSW) are living with HIV, and many face structural and individual‐level barriers to initiating, accessing and adhering to antiretroviral therapy (ART). FSW are criminalized, stigmatized and marginalized. The Siyaphambili (we are moving forward) study enrolled 777 FSW with sustained viremia into an 18 month sequential multistage adaptive randomized trial (SMART) to compare the effectiveness and durability of a nurse‐led decentralized treatment program (DTP) and individualized case management (ICM) in isolation or in combination to achieve sustained viral suppression. This study reports on morbidity and mortality in the cohort.

**Methods**: Non‐virally suppressed FSW 18+ years living with HIV in Durban, South Africa were enrolled into the Siyaphambili trial from June 2018‐March 2020; follow‐up ended January 2022. Attempted contact was made with women every 1 to 2 months dependent on intervention arm and 6‐monthly for study visits/blood draws. We describe morbidity and mortality over 18‐months of follow‐up; mortality rates were estimated per 1000 person‐years of follow‐up.

**Results**: Average age of FSW was 31 years. Sixteen deaths were reported among 777 trial participants during follow‐up at a rate of 18.7/1000 PY. Causes of death included TB (n = 5), unspecified illness (n = 5), murder (n = 3), COVID‐19 (n = 1), suicide (n = 1) and hypothermia (n = 1). A further 6 women were known to have been incarcerated during study follow‐up. Adverse events outside of death were commonly reported in the study visits at 6, 12 and 18 months: A range of 10.1–15.7% of FSW reported hospitalization in the past 6 months across these each of these visits; 19.4–32.2% reported physical violence in the past 6 months; and 10.6–14.8% reported sexual violence (rape) across the past 6 months. No adverse events were determined to be due to the study.

**Conclusions**: FSW have high morbidity and mortality relative to their age group in the general population with TB being the major cause of mortality. Hospitalization, physical and sexual violence are also highly prevalent among FSW. Interventions to prevent and treat TB and violence need to be integrated into care for FSW.

### PEMOB34 Prevalence and risk factors for anal dysplasia among men who have sex with men living with HIV: the HPV Screening and Vaccine Evaluation (HPV‐SAVE) Study

A.K. Gupta ^1,2^, T. Tattersall^1,3^, A. Ablona^1^, R. Azmin^1^, A.N. Burchell^4,5^, J. Edward^1^, M. Gaspar^5^, D. Grace^5^, J. Gillis^6^, B. Lyons^1^, P. MacPherson^7^, B. Okocha^1^, R. Rosenes^8^, D.H.S. Tan^4,5^, I. Salit^9,5^, T. Grennan^1,10^

^1^BC Centre for Disease Control, Vancouver, Canada, ^2^University of British Columbia, Faculty of Pharmaceutical Sciences, Vancouver, Canada, ^3^University College London, London, United Kingdom, ^4^St. Michael's Hospital, Toronto, Canada, ^5^University of Toronto, Toronto, Canada, ^6^Vaccine Evaluation Centre, BC Children's Hospital Research Institute, Vancouver, Canada, ^7^The Ottawa Hospital, Vancouver, Canada, ^8^Progressive Consultants Network of Toronto, Toronto, Canada, ^9^Toronto General Hospital, Toronto, Canada, ^10^University of British Columbia, Faculty of Medicine, Division of Infectious Diseases, Vancouver, Canada

**Background**: HPV‐associated anal cancer is a common malignancy in men who have sex with men living with HIV (MSMLWH). Despite recent indications that screening reduces the incidence of anal cancer, access is limited to urban centres and is coupled with substantial waitlists requiring the need for triaging care. We assessed the prevalence of and risk factors for anal dysplasia among MSMLWH.

**Methods**: The HPV‐SAVE study examines anal cancer screening in MSMLWH in Vancouver, Ottawa and Toronto, Canada. Between 01/2016 and 05/2021, participants were recruited from HIV clinics and completed a questionnaire pertaining to demographics and medical history. We screened participants for anal dysplasia via anal pap, defined as any non‐normal result on cytology using the Bethesda classification. We completed descriptive statistics to report the prevalence of anal dysplasia and binomial logistic regression to identify risk factors for dysplasia.

**Results**: Among 720 participants screened, most were white (70.0%), over 50 years‐old (52.1%) and unpartnered (53.8%). Most had dysplasia (344/663; 51.9%): ASCUS (223/663; 33.6%), LSIL (77/663; 11.6%), HSIL (18/663; 2.7%); and ASC‐H (16/663; 2.4%). Many participants reported past anogenital warts (269/699; 38.5%) however, this did not increase the odds of dysplasia (odds ratio [OR] 1.03 95% confidence interval [CI] 0.75, 1.41). Black participants were less likely to have dysplasia than non‐Black participants (OR 0.47, 95% CI 0.23, 0.96), as were individuals who acquired HIV between 2000–2010 relative to those who were diagnosed after 2010 (OR 0.63 95% CI 0.42, 0.94). Elevated odds of dysplasia were observed for current smokers and individuals who quit within the past 5‐years (OR 1.42; 95% CI 1.01, 2.01) and individuals with quarterly physician visitation compared to individuals with biannual visitation (OR 1.61, 95% CI 1.00, 1.93). There was no association between dysplasia and low self‐reported CD4+ count (OR 1.10 95% CI 0.75, 1.62) or elevated age (OR 1.10 95% CI 0.80, 1.50).

**Conclusions**: Anal dysplasia is common among MSMLWH. Smoking, frequent physician visitation, recent HIV acquisition and non‐Black ethnicity were associated with dysplasia. These interim results highlight the importance of further research addressing mediators and confounders of engagement in anal cancer screening to support treatment and care for MSMLWH.

### PEMOB35 Addressing sexual health in trans masculinities: lessons learned from TransCITAR transgender cohort study in Argentina

N.K. Panis ^1^, L. Spadaccini^1^, N. Cabrera^1^, C.F. Perez^1^, M.V. Iannantuono^1^, M.M. Sandoval^1^, N. Doudtchitzky^1^, M.I. Figueroa^1^, C. Cesar^1^, V. Fink^1^, C. Frola^1^, O. Sued^2^, I. Aristegui^1^, TransCITAR Study Group

^1^Fundación Huésped, Research Department, Capital Federal, Argentina, ^2^OPS, Washington, United States

**Background**: Trans masculinities (TM) are usually underrepresented in clinical settings, and data involving HIV and other sexually transmitted infections (STIs) are scarce, highlighting health disparities among this community. This study aimed to describe sexual health status of TM attending a transgender cohort at Buenos Aires, Argentina

**Methods**: We conducted a retrospective review of TransCITAR cohort study TM medical records (September 2019‐December 2021). Gathered data included age, sexual orientation, sex work history, age of sexual initiation, history of pregnancy, and use of gender‐affirming hormone therapy (GHT). STI screening included: HIV (by rapid test), syphilis (by nontreponemal test‐VDRL‐), hepatitis A (HAV), B (HBV) and C (HCV) serologies, rectal chlamydia and gonorrhea and cervical cytology. Diagnosis of gonorrhea and chlamydia were made by in‐house PCR on rectal swabs performed by a healthcare professional.

**Results**: Thirty‐six TM were included, median age 25 years (IQR 21–30). Only two participants reported history of sex work. Regarding sexual orientation a great variation was reported: 15(42%) heterosexual, 9(25%) bisexual, 6(17%) pansexual, 1(3%) gay and 1(3%) asexual. Most participants 69%(n = 25) were receiving GHT. Four participants had not initiated sexual activity. All participants were tested for HIV and syphilis with negative results. Five (14%) had rectal gonorrhea and chlamydia screening, with negative results. Among TM who had initiated sexual activity, only 10/32 (31%) performed the cervical cytology, among these, 2 had low‐grade intraepithelial lesions. At baseline visit, all participants consented to hepatitis serology, and all had IgG HAV positive, IgG HCV and HBsAg negative. Ten TM had HBsAb < 10 IU/ml. One person had pregnancy history with miscarriage.

**Conclusions**: Despite facilitated access to a trans competent facility, only few TM completed STI and genital cancer screening, and almost one third had HBV serology compatible with missing or incomplete vaccination. These results highlight the urgent need for more formative research to understand how to offer adapted and acceptable genital evaluation and screening of STIs and cancer to this population.

### PEMOB36 Connecting vulnerable people with opioid use disorder to care: Expanding accessibility and building trust through a community‐based telemedicine partnership in Montreal, Canada

C. de Montigny ^1^, S. Hoj^2^, R. Leandre^2^, M.‐E. Beauchemin Nadeau^1^, G. Boyer‐Legault^3^, S. Chougar^1^, A. Goyette^3^, S.‐K. Lamont^1^, J. Bruneau^2^

^1^CHUM, Addiction medicine, Montreal, Canada, ^2^CR CHUM, Montreal, Canada, ^3^CACTUS Montréal, Montréal, Canada

**Background**: The COVID‐19 pandemic has greatly impacted health service delivery, with unprecedented expansion of telemedicine. To address the needs of people with opioid use disorder (PWOUD), the Centre hospitalier de l'Université de Montréal Addiction Medicine service (CHUM‐A) began to initiate new patients on opioid agonist treatment (OAT) – a key intervention for reducing HIV transmission and overdose risk – via telemedicine.

However, PWOUD often lack access to the technological resources necessary for telemedicine. Likewise they often have complex needs while being disengaged from mainstream health services. The CHUM‐A and CACTUS Montreal (a community‐based harm reduction organisation) therefore co‐constructed a unique telemedicine program aiming to provide evidence‐based treatment and health care for PWOUD.

**Description**: Procedures were developed jointly to enable flexible and rapid appointment schedules. CACTUS Montreal workers, known and trusted by their clientele, inform PWOUD of the program, facilitate telemedicine connection in a private room located within their facilities, introduce the CHUM‐A team and can accompany the patient throughout the appointment if requested. Treatments offered by the CHUM‐A team include a long‐acting opioid, often combined with a short‐acting opioid to increase comfort and reduce risk of illicit use and overdose, and other services including HIV and HCV prevention and treatment as needed. CACTUS Montreal workers maintain follow up and support to the participants following an holistic approach.

**Lessons learned**: Between April 2020 and October 2021, 66 people initiated OAT through the program and 83% currently remain engaged in care, much higher than reported 1‐year OAT retention rates of 30–70%. Five participants commenced HIV treatment and 16 were treated for hepatitis C. Qualitative interviews with 20 participants suggest an enthusiastic response; the initiative was perceived to be convenient and protective in the pandemic context, and the implication of CACTUS Montreal was highly valued. Several participants reported having reduced their drug consumption and experiencing greater stability in other areas of their lives.

**Conclusions/Next steps**: Our telemedicine program provides a flexible approach with alternative treatment options for PWOUD disengaged from traditional care, integrated within a local organisation. It represents an affordable solution to reduce the gap between patients and health providers, and is promising to increase access in remote settings.

### PEMOB37 COVID‐related barriers associated with suboptimal adherence during India's second “delta” wave: Results from a South India HIV cohort

M.L. Ekstrand ^1^, S. Chandy^2^, M. Pereira^3^, E. Heylen^1^, K. Srinivasan^4^

^1^University of California, San Francisco, Medicine, San Francisco, United States, ^2^Wayanad Institute of Medical Sciences, Department of Medicine, Wayanad, India, ^3^St John's National Academy of Health Sciences, SJRI, Bangalore, India, ^4^St John's National Academy of Health Sciences, Psychiatry, St John's Medical College, Bangalore, India

**Background**: Successful management of HIV requires excellent adherence and timely prescription refills to avoid treatment interruptions. The COVID‐19 pandemic restrictions have resulted in unintended treatment barriers for people living with HIV (PWH). It is important to document the impact that these barriers had on ability to visit ART clinics, obtain prescription refills, and adhere to medical regimens during different phases of the pandemic.

**Methods**: The “Tel‐Me‐Box” study was designed to validate novel measures of medication adherence by enrolling and following a cohort of 526 PWH in South India for 24 months. During COVID‐19‐related government restrictions on travel and face‐to‐face visits, we conducted telephone surveys, adding questions on pandemic‐related adherence barriers. This abstract includes data collected in 1) Jan‐Feb (n = 442) 2) May‐June (n = 451) and 3) Aug‐Sept (449) 2021, i.e. pre‐, during, and post‐ India's “second wave,” which occurred during the surge of the delta variant. We assessed past month HIV adherence and >48hr treatment interruptions, combined into a measure of “suboptimal adherence,” as well as individual and structural adherence barriers.

**Results**: In 2021, <95% past month adherence was reported by 7% in Jan‐Feb, 8% in May‐June, and 4% in Aug‐Sept of cohort participants. While these differences were not statistically significant, perceived pandemic‐related barriers to clinic visits and medication adherence were significantly more common during the May‐June surge of the delta variant than they were pre‐ or post‐surge. These barriers were associated with suboptimal adherence during the surge (see table), but not before or afterwards.

**Table:** 

**Abstract PEMOB37‐Table 1**.					
Perceived pandemic‐related adherence barriers	% who agreed with each statement (^*^p < 0.05)Pre‐surge (Jan‐Feb)During surge (May‐June)Post‐surge (Aug‐Sept)	% who reported suboptimal adherence during surge among those who agreed with barriers at that time	% who reported suboptimal adherence during surge among those who disagreed with barriers at that time	p (chi‐square test bivariate)	Multivariate modelaOR (95% CI)
“I worry that I will run out of my medication and not be able to get refills during the pandemic”	Pre: 13.3% (n = 59/442) During: 34.8%* (n = 157/451) Post: 8.2% (n = 37/449)	16.6% (n = 26/157)	7.8% (n = 23/294)	p≤0.005	1.13 (0.53, 2.39)
“I don't have transportation to get me to the clinic”	Pre: 10.4% (n = 46/442) During: 40.6%* (n = 183/451) Post 7.8% (n = 35/449)	16.4% (n = 30/183)	7.1% (n = 19/268)	*p≤0.002*	*1.96 (0.98, 3.91)*
“I'm afraid that taking my HIV meds will make me more vulnerable to COVID infection or complications.”	Pre: 13.8% (n = 61/442) During: 20.6%* (n = 93/451) Post: 6.2% (n = 28/449)	20.4% (n = 19/93)	8.4% (n = 30/358)	p≤0.001	2.13 (1.01, 4.48)
“I'm afraid that doctors won't be available or will treat me differently if I seek care at the hospital”	Pre: 19.7% (n = 87/442) During: 31.7%* (n = 143/451) Post: 12.0% (n = 54/449)	15.4% (n = 22/143)	8.8% (n = 27/308)	p≤0.036	1.13 (0.55, 2.30)

**Conclusions**: Although self‐reported adherence levels in this cohort were similar throughout 2021, pandemic‐related concerns were significantly greater during India's devastating “second wave” in May‐June, during the surge of the delta variant. During this time, COVID‐related worries and lack of transportation were significantly associated with suboptimal HIV medication adherence. These findings have implications for policy and clinical care during future surges and suggest that PWH could benefit from additional counseling to reduce worries together with transportation assistance or medication deliveries to ensure consistent medication access.

### PEMOC33 Real‐world utilization of HIV pre‐exposure prophylaxis (PREP) by cisgender and transgender individuals in the united states

J. Thorburn^1^, J. Paone^1^, A. Kong^1^, C. Bush^1^, D. Patel^1^, C. Carter^2^, M. Das^2^, L. Tao ^2^

^1^Aetion, New York, United States, ^2^Gilead Sciences, Foster City, United States

**Background**: This study describes real‐world utilization of daily oral PrEP regimens emtricitabine (F)/tenofovir disoproxil fumarate (TDF) and F/tenofovir alafenamide (TAF) by gender.

**Methods**: HIV‐1 negative individuals receiving PrEP (F/TDF or F/TAF) were identified from a pharmacy claims database linked with medical claims from physicians' offices across the US. Transgender men (TGM) and women (TGW) were identified using an algorithm which incorporated claims for gender dysphoria, gender‐affirming surgery, and gender‐affirming hormone therapy. Individuals not identified as TGM or TGW were classified as cisgender men (CGM) or women (CGW).

**Results**: Most people receiving PrEP were CGM while the proportions of individuals classified as CGW, TGM, and TGW increased from 2015 to 2020 (CGW: 8.1% to 11.9%; TGM: 0.3% to 0.6%; TGW: 0.4% to 1.4%). Among 104,354 people receiving PrEP from 10/3/2019 (approval date of F/TAF) to 3/31/2021, 90.2%, 7.9%, 0.6%, and 1.2% were CGM, CGW, TGM, and TGW, respectively, and 37% were from the South (Table). The proportion of people receiving PrEP of each state ranged from 79.2% (Louisiana) to 95.4% (Utah) for CGM, from 2.7% (Vermont) to 19.6% (Florida) for CGW, and from 0.0% (North Dakota) to 3.7% (Ohio) for TGM/TGW (Figure). Higher proportion of people on F/TAF received prescriptions from an infectious disease physician compared with F/TDF (9.2% vs. 6.7%); this difference was greatest among CGW (13.7% vs. 7.1%).

**Table:** 

**Abstract PEMOC33‐Table 1**.					
	Total N = 104,354	CGM N = 94,159 (90.2%)	CGW N = 8,291 (7.9%)	TGM N = 605 (0.6%)	TGW N = 1,299 (1.2%)
**F/TAF Users**	28,372	27,211 (95.9%)	725 (2.6%)	114 (0.4%)	322 (1.1%)
**F/TDF Users**	75,982	66,948 (88.1%)	7,566 (10.0%)	491 (0.6%)	977 (1.3%)
**Median Age at Initiation (IQR)**	36 (28, 48)	36 (28, 48)	36 (27, 47)	32 (25, 44)	29 (23, 37)
**Region: Northeast**	24,143	21,703 (89.9%)	1,913 (7.9%)	154 (0.6%)	373 (1.5%)
**Region: Midwest**	17,253	15,787 (91.5%)	1,077 (6.2%)	106 (0.6%)	283 (1.6%)
**Region: South**	38,177	33,597 (88.0%)	4,078 (10.7%)	175 (0.5%)	327 (0.9%)
**Region: West**	24,287	22,631 (93.2%)	1,182 (4.9%)	167 (0.7%)	307 (1.3%)

**Figure:**
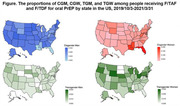
**Abstract PEMOC33‐Figure 1**.

**Conclusions**: This study describes the changing gender composition of people on PrEP, with CGW, TGM, and TGW accounting for increased proportions over time in the real‐world. Demographic and geographic variation across genders highlights the importance of improving access for people who would benefit from PrEP.

### PEMOC34 Considerations for the delivery of long‐acting HIV prevention methods in South Africa: provider perspectives on uptake and facilitating choice and informed decision‐making

N. Vundamina ^1^, C.M. Wong^2^, M. Pleaner^1^, E. Briedenhann^1^, N. Naidoo^1^, S. Mullick^1^

^1^Wits Reproductive Health and HIV Institute (Wits RHI), University of Witwatersrand, Implementation Science, Johannesburg, South Africa, ^2^FHI 360, Global Health, Population and Nutrition, Durham, United States

**Background**: Clinical trials of the dapivirine vaginal ring (PrEP ring or ring) and long‐acting injectable cabotegravir (CAB‐LA) have shown promising results, with both products exhibiting efficacy in preventing HIV acquisition. Successful product introduction and scale‐up will require in‐depth understanding of the knowledge, perceptions, and attitudes of healthcare providers (HCPs) related to these new products.

**Description**: Through the USAID‐funded PROMISE collaboration, we used semi‐structured discussion guides to conduct conversations with family planning (FP) and HIV prevention HCPs from public health facilities and project sites in South Africa from May to June 2021. These conversations explored perceptions of the ring and CAB‐LA; experiences with oral PrEP and FP provision; counseling on multiple methods; and strategies to enable informed choice and decision‐making among potential clients. Conversations were audio‐recorded and thematic analysis was conducted using a two‐step rapid analysis process.

**Lessons learned**: A total of 59 (53 female and four male) HCPs providing integrated FP‐HIV prevention services participated in the conversations. 57 HCPs had heard about the ring; about half had heard about CAB‐LA. Perceived advantages of the ring included fewer side effects compared to oral PrEP and ease of insertion and removal. HCP concerns included low ring uptake due to low efficacy compared to oral PrEP; incorrect use with clients removing the ring before 28 days; or potential intimate partner violence. For CAB‐LA, HCPs mentioned its discreet use and long‐acting qualities as advantages. Concerns about CAB‐LA included the risk of developing HIV drug resistance and lasting side effects. Overall, HCPs said the introduction of new PrEP products would be beneficial to clients, allowing them to choose methods based on their lifestyles. They expected uptake of CAB‐LA to be higher among women who already use injectable contraceptives. HCPs worried that introducing new products would lead to increased workloads, inadequate counseling, and improper stock control and storage measures, particularly for CAB‐LA.

**Conclusions/Next steps**: New product introduction will require health system strengthening with a focus on building the capacity of HCPs to provide new methods. Job aids should be developed to facilitate counseling on choice while client‐facing materials should support choice and informed decision‐making.

### PEMOC35 Effect of combination Needle and Syringe Program and Opioid Agonist Therapy on HIV and hepatitis C virus acquisition among people who inject drugs: a comparison between Amsterdam, Melbourne and Vancouver

A. Boyd ^1,2^, D.K. van Santen^1,3,4^, S. Lodi^5^, W. van den Boom^3^, P. Dietze^3^, K. Hayashi^6,7^, H. Dong^6^, Z. Cui^6^, L. Maher^8^, M. Hickman^9^, M. Prins^1,10^

^1^Public Health Service of Amsterdam, Infectious Diseases, Amsterdam, Netherlands, the, ^2^Stichting HIV Monitoring, Amsterdam, Netherlands, the, ^3^Burnet Institute, Disease Elimination, Melbourne, Australia, ^4^Monash University, Department of Epidemiology and Preventive Medicine, Melbourne, Australia, ^5^Boston University School of Public Health, Department of Biostatistics, Boston, United States, ^6^British Columbia Centre on Substance Use, Vancouver, Canada, ^7^Simon Fraser University, British Columbia Centre on Substance Use, Burnaby, Canada, ^8^The Kirby Institute for Infection and Immunity, Faculty of Medicine, Sydney, Australia, ^9^University of Bristol, Population Health Sciences, Bristol Medical School, Bristol, United Kingdom, ^10^Amsterdam UMC, University of Amsterdam, Department of Infectious Diseases, Amsterdam Infection and Immunity Institute (AI&II), Amsterdam, Netherlands, the

**Background**: Harm reduction programs for people who inject drugs (PWID) have been available in Amsterdam, Vancouver, and Melbourne since the 1980s. Compared to the current situation in Vancouver and Melbourne, coverage of harm reduction programs is higher in Amsterdam, fewer individuals initiate injection drugs, and HIV and HCV transmission has been largely stopped among PWID. We aimed to assess whether the effect of needle and syringe program (NSP) and opioid agonist therapy (OAT) participation on HIV and HCV incidence differs in these three settings.

**Methods**: We emulated the design of a target randomized trial using observational data from the Amsterdam Cohort Studies (ACS, 1985–2014), Vancouver Injection Drug Users Study (VIDUS, 1997–2009), and Melbourne Injecting Drug User Cohort Study (SuperMix, 2008–2021). We included PWID with a recent history of injecting drug use and opioid use, and who tested negative for HIV or HCV. We compared the effect of complete NSP/OAT participation (current OAT and 100% NSP coverage, or current OAT if no recent injection drug use) versus no or partial NSP/OAT participation combined (no OAT and/or <100% NSP coverage) on HIV and HCV risk, per cohort (only HCV in SuperMix as transmission of HIV is rare among PWID in Australia). Marginal structural models were used to analyze data.

**Results**: During follow‐up among participants included in analysis, there were 61/624 HIV seroconversions in ACS and 37/1,399 in VIDUS, and 34/129 HCV seroconversions in ACS, 30/216 in VIDUS, and 21/122 in SuperMIX. Compared with no/partial NSP/OAT participation, complete participation led to lower risk of HIV and HCV acquisition, with the strongest effect on HIV observed in the ACS (Hazard ratio (HR) = 0.38, 95%CI = 0.23–0.66) compared to VIDUS (HR = 0.45, 95%CI = 0.11–1.84), while its effect on HCV was strongest in VIDUS (HR = 0.11, 95%CI = 0.01–0.85) and ACS (HR = 0.29, 95%CI = 0.12–0.67) compared to SuperMix (HR = 0.49, 95%CI = 0.14–1.71).

**Conclusions**: Complete NSP and OAT participation led to a reduction of HIV and HCV acquisition compared to no/partial participation in regions from two and three continents, respectively. Findings reinforce the crucial role of combined NSP and OAT in infection prevention and the need for comprehensive access to both interventions.

### PEMOC36 The effect of universal testing and treatment for HIV on health‐related quality of life – data from the HPTN 071 (PopART) cluster randomised trial in Zambia and South Africa

K. Davis ^1^, M. Pickles^1^, S. Gregson^1,2^, J. Hargreaves^3^, H. Ayles^4^, P. Bock^5^, T. Pliakas^3^, R. Thomas^6^, J. Ohrnberger^1^, J. Bwalya^7^, N. Bell‐Mandla^5^, K. Shanaube^7^, W. Probert^8^, G. Hoddinott^5^, V. Bond^7,9^, R. Hayes^10^, S. Fidler^11^, K. Hauck^1^

^1^Imperial College London, MRC Centre for Global Infectious Disease Analysis and the Abdul Latif Jameel Institute for Disease and Emergency Analytics, School of Public Health, London, United Kingdom, ^2^Biomedical Research and Training Institute, Harare, Zimbabwe, ^3^London School of Hygiene and Tropical Medicine, Department of Public Health, Environments and Society, Faculty of Public Health and Policy, London, United Kingdom, ^4^London School of Hygiene and Tropical Medicine, Department of Clinical Research, Faculty of Infectious and Tropical Diseases, London, United Kingdom, ^5^University of Stellenbosch, Desmond Tutu TB Centre, Department of Paediatrics and Child Health, Faculty of Medicine and Health, Cape Town, South Africa, ^6^London School of Economics, Department of Health Policy, London, United Kingdom, ^7^University of Zambia, Zambart, School of Medicine, Lusaka, Zambia, ^8^University of Oxford, Big Data Institute, Nuffield Department of Medicine, Oxford, United Kingdom, ^9^London School of Hygiene and Tropical Medicine, Department of Global Health and Development, Faculty of Public Health and Policy, London, United Kingdom, ^10^London School of Hygiene and Tropical Medicine, Department of Infectious Disease Epidemiology, Faculty of Epidemiology and Population Health, London, United Kingdom, ^11^Imperial College London, Department of Infectious Disease, Faculty of Medicine, London, United Kingdom

**Background**: HIV treatment has clear Health‐Related Quality‐of‐Life (HRQoL) benefits. However, little is known about how Universal Testing and Treatment (UTT) for HIV affects HRQoL. We examined the effect of a combination prevention intervention, including UTT, on HRQoL among PLHIV.

**Methods**: Data were from HPTN 071 (PopART), a three‐arm cluster randomised controlled trial in 21 urban and peri‐urban communities in Zambia and South Africa (2013–2018). Arm A received the full UTT intervention of door‐to‐door HIV testing plus access to antiretroviral therapy regardless of CD4 count, Arm B received the intervention but followed national treatment guidelines (universal ART from 2016) and Arm C received standard care. The intervention effect was measured in an open cohort of randomly selected adults (18–44 years) in randomly selected households, using data from baseline and 36‐months. HRQoL scores (range: 0–1), and the prevalence of problems in five dimensions of HRQoL (mobility, self‐care, performing daily activities, pain/discomfort, anxiety/depression) were assessed among all participants using the EuroQol‐5‐dimensions‐5‐levels questionnaire (EQ‐5D‐5L). HRQoL among PLHIV with laboratory confirmed HIV status was compared between arms. This was achieved using two‐stage cluster‐level analyses, controlling for baseline imbalances in language(s) used to complete the survey, wealth and HRQoL, as well as age and gender.

**Results**: Data from 10,900 PLHIV (women, n = 9,205, 84.4%; men, n = 1,695, 15.6%) were examined. At 36‐months, the mean HRQoL score was 0.893 (95% confidence interval: 0.891–0.894) in Arm A, 0.888 (0.886–0.890) in Arm B and 0.891 (0.889–0.892) in Arm C. There was no evidence of a difference in HRQoL scores between arms (adjusted mean difference, A vs C: 0.003, −0.001–0.006; B vs C: −0.004, −0.014–0.005). However, the geometric mean prevalence of problems with pain/discomfort was 2.4% in Arm A, 7.5% in Arm B and 7.8% in Arm C, with prevalence lower in Arm A than C (adjusted prevalence ratio: 0.37, 0.14–0.97). There was no evidence of a difference in effect between men and women.

**Conclusions**: The PopART UTT intervention did not change overall HRQoL, suggesting that improving HRQoL among PLHIV might require more than access to testing and treatment. However, PLHIV had fewer problems with pain/discomfort under the full intervention; this benefit of UTT should be maximised in further roll‐out.

### PEMOC37 *Men's voices on LA_PrEP*: The acceptability and preferences of Long acting Pre‐Exposure Prophylaxis (LA‐PrEP) among Cis‐gender men and Men who have sex with men in South Africa

M. Atujuna ^1^, Z. Duby^2^, A. Minnis^3^, T. Palanee‐Phillips^4^, S. Tenza^4^, K. Reddy^4^, N. Nkomana^1^, L.‐G. Bekker^1^, E. Montgomery^3^

^1^University of Cape Town, Faculty of Health Sciences, School of Medicine, Desmond Tutu HIV Centre, Cape Town, South Africa, ^2^University of Cape Town, Division of Social and Behavioural Sciences, School of Public Health and Family Medicine, Cape Town, South Africa, ^3^Research Triangle Institute, International, Berkeley, United States, ^4^Wits Reproductive Health and HIV Institute, University of Witwatersrand, Johannesburg, South Africa

**Background**: Cisgender men who have sex with women (MSW) and men who have sex with men (MSM) are often under‐represented in HIV prevention research despite their roles as key drivers of the HIV epidemic. As HIV prevention research around long‐acting pre‐exposure prophylaxis (LA‐PrEP) options expand in sub‐Saharan Africa (SSA), it is essential to engage these key populations to ensure their buy‐in. We investigated perceptions of implants and injectables as LA‐PrEP delivery platforms among MSW and MSM in SAMURAI, an end‐user acceptability and preference study.

**Methods**: In‐depth interviews were conducted with 40 MSW (n = 20) and MSM (n = 20) from resource‐restricted communities in Cape Town and Johannesburg, aged 18–35 years who self‐reported as being HIV‐negative and currently sexually‐active. We explored themes around sexual behaviour and relationships, masculinities and gender, and other factors influencing attitudes towards LA‐PrEP. Data analysis followed a thematic framework approach.

**Results**: Both MSW and MSM felt that the proposed modes of administering LA‐PrEP were acceptable and more appealing than daily oral PrEP because they offered longer lasting protection, while reducing the burden related to frequent clinic visits for refills. They described these new products as having potential to overcome the various challenges relating to consistent condom use, while offering a more acceptable tool to reduce the risk of acquiring HIV. MSW voiced hesitancy around the use of ‘*foreign products*’, expressing concerns about infertility and fears that these products may cause birth defects in their future children. The convenience of implants with long dosing duration was acknowledged by both populations, but injections were deemed to be more discreet and familiar. Implant use was described to be potentially stigmatizing with a greater chance of causing tissue scarring, or lead to implant robbery, a narrative that implants are forcefully removed and used as recreation drugs.

**Conclusions**: Evidence about men's engagement in HIV prevention and what modalities of HIV prevention may be acceptable and preferred is limited. We found that both groups were enthusiastic about LA‐PrEP. This research will inform development of a clinical study to provide further insight into safety, acceptability and use of placebo versions of LA‐PrEP among MSW and MSM.

### PEMOC38 Adherence with antiretroviral therapy among recently pregnant HIV‐positive women in 8 African countries

S. Chung ^1^, M. Farahani^2,1^, C. Wang^2^, W. El‐Sadr^2,1^, J. Justman^2,1^

^1^Columbia University Mailman School of Public Health, Epidemiology, New York, United States, ^2^ICAP at Columbia University, New York, United States

**Background**: Adherence to antiretroviral therapy (ART) is essential for reducing morbidity and mortality among people living with HIV and HIV transmission, particularly mother‐to‐child transmission for pregnant and postpartum women. This study compared self‐reported ART use with antiretroviral drug (ARV) detection in blood among HIV+ women aged 15–49 who had delivered within three years before the survey, using population‐based HIV surveys (PHIAs) in Eswatini, Lesotho, Malawi, Namibia, Tanzania, Uganda, Zambia, and Zimbabwe (2015–2019), conducted by the ministries of health in collaboration with ICAP and CDC.

**Methods**: Consenting participants from randomly selected households provided demographic and clinical information and blood for household HIV testing. Household HIV+ results were laboratory‐confirmed. Viral load suppression (VLS) was defined as VL < 1000 cp/mL. Commonly prescribed ARVs, namely, efavirenz, nevirapine, atazanavir, and lopinavir, were assayed in dried blood spots. All analyses accounted for complex survey design, and Taylor Series Linearization methods were used for variance estimation.

**Results**: Of all 91,728 female participants, 2,108 were included in this analysis. Most women took ARVs before their first antenatal visit, ranging from 46% (95% CI: 44%‐49%) in Tanzania to 82% (95% CI: 79%‐85%) in Namibia. VLS ranged from 77% (95% CI: 73%‐80%) in Lesotho to 88% (95% CI: 85%‐91%) in Malawi. ARVs were detected in the blood of most women who initiated ART before their first antenatal visit, ranging from 88% (95% CI: 84%‐92%) in Lesotho to 94% (95% CI: 90%‐98%) in Malawi.

Adjusted for other demographic characteristics, women who initiated ART before the first antenatal visit were more likely to have detectable ARVs than those who initiated ART during pregnancy (adjusted odds ratio (aOR): 2.2; 95% CI: 1.7–2.9). Women aged 35–49 were more likely to have detectable ARVs than those aged 15–24 (aOR: 2.2; 95% CI: 1.5–3.1).

**Conclusions**: ART adherence, proxied by ARV detection in blood, was lower among women who initiated ART during pregnancy than those who started ART before pregnancy, particularly among women aged 15–24 years. Women, particularly young women, who initiate ART during pregnancy require specific attention to enhance their own outcomes and prevent mother‐to‐child HIV transmission.

### PEMOC39 Effect of client profiling for tailored adherence support on ART retention among Children and Adolescents Living with HIV/AIDS in an OVC program in Nigeria

U. Ezenwa ^1^, O. Onyezue^1^, D. Atagher^1^, S. Adamu‐Oyegun^1^, O. Olabanjo^1^, I. Onwuatuelo^1^, J. Osi‐Samuels^1^

^1^APIN Public Health Initiatives, Prevention and Community Services, FCT, Nigeria

**Background**: ART retention rate among Children and Adolescents Living with HIV/AIDS (C/ALHIV) enrolled in a CDC funded OVC program in Nigeria has been steadily low over the last four years. Despite significant efforts, which ensure drug pickup at supported health facilities and also in non‐conventional locations like Patent Medicine Stores, C/ALHIVs continue to be poorly retained, because most interventions do not take into cognizance the underlying uniqueness of members of this population. The objective of this study was to analyze the effectiveness of client profiling as a strategy for identifying characteristic barriers inhibiting ART retention and by the provision of corresponding tailored adherence support, improve ART retention rates among C/ALHIV.

**Description**: Of 12,440 C/ALHIV on ART in 10 HIV high burden Local Government Areas in Benue State, in‐depth interviews were conducted on 10,433 who are enrolled in OVC program using pre‐designed client profile forms administered by 126 Community caseworkers, to determine unique characteristics driving vulnerability to missed appointments and the type of support or incentives required to address it. Data was analyzed using descriptive and summary statistics after a six months period following provision of required support or incentives.

**Lessons learned**: Absence of mobile phone 1,262 (12%), attending school or vocational center 1,640 (16%), not pleased with venue for ARV refill 3,025 (29%) and absence of transport fare 4,506 (43%) were identified as key barriers. Following profiling, 1,094 (10%) required home visits 1,659 (16%) required reminder phone calls/text messages, 3,696 (35%) escort to facility for ARV refill, and 3,984 (38%) required minimum fare support. Retention rate among 10,433 profiled CALHIV who received tailored adherence support or incentive improved from 71% at baseline to 92% after six months representing 21% increase while for the 2,007 C/ALHIV who are not enrolled in OVC program and weren't profiled or received intervention, retention rate was 75% after the same period, representing 4% increase.

**Conclusions/Next steps**: The study revealed that client profiling and provision of corresponding tailored adherence support or incentive is effective in improving retention rate among C/ALHIV. Program interventions, which will strengthen provision of escort services and minimum fare support to C/ALHIV is highly recommended.

### PEMOC40 Leveraging faith communities to test and treat men living with HIV in Uganda

L.J. Nelson ^1^, A.G. Fitzmaurice^1^, R. Kamara^2^, L. Sekimpi^2^, H. Mwesezi^2^

^1^US Centers for Disease Control and Prevention (CDC), Division of Global HIV & TB, Kampala, Uganda, ^2^Uganda Episcopal Conference, Uganda Catholic Medical Bureau, Kampala, Uganda

**Background**: In Uganda, disproportionately fewer men living with HIV (MLHIV) than women are aware they have HIV. This is partly due to not seeking out healthcare, so providing testing services where men frequent might help MLHIV get tested and treated for HIV. Uganda's population comprises 84.4% Christians and 13.7% Muslims, so faith communities are effective venues for reaching many Ugandans. Here, we describe how national faith‐based medical organizations introduced HIV testing to faith communities in Uganda; we compare results from Catholic churches with those from regional HIV testing programs to demonstrate the potential of faith community HIV programs.

**Description**: In April—September 2021, Uganda's Catholic, Protestant, Muslim, and Orthodox medical bureaux conducted two‐day trainings of 794 faith leaders in three regions using curriculum endorsed by the Ministry of Health. Faith leaders used sermons, free/subsidized Christian radio and television, and social media to mobilize 53,826 community members for counseling and HIV test screening. Some men came to HIV testing events when COVID‐19 vaccination and hypertension screening were offered concurrently. Faith leaders administered 15,578 HIV tests, including 9,101 rapid test kits. We used a two‐sample proportion test to compare results from a subset (5,449 rapid tests administered by Catholic faith leaders) with HIV test data in Uganda's Electronic Health Information System (eHMIS) from the same time period and regions.

**Lessons learned**: Catholic churches tested more men than women (62%; 3,378/5,449), while traditional testing programs tested fewer men (31%; 168,649/546,956). Tests in Catholic churches were more seropositive than in traditional testing programs (4.6% vs. 3.7%; OR = 1.25; P = 0.0005). Churches needed fewer tests than traditional programs to identify each person living with HIV. Of the 138 men and 114 women with positive results in Catholic churches, 111 men (80%) and 106 women (93%) went to facilities and had confirmatory positive tests; 109 men (98%) and 98 women (92%) initiated treatment.

**Conclusions/Next steps**: Faith communities can be leveraged to test and treat those who do not typically seek HIV services. We spent only 2 USD per person for one‐on‐one counseling and screening. Further implementation will help reveal whether faith communities can identify MLHIV at an impactful and cost‐effective scale.

### PEMOC41 A geospatially targeted field‐based approach is more efficient than a network‐driven approach for reaching PWID living with HIV in Punjab, India

T. Loeb ^1^, A.M. McFall^1^, A.K. Srikrishnan^2^, L.R. Sangma^2^, P. Narayan^3^, P. Nandagopal^2^, S. Anand^2^, V. Swaminathan^2^, S.H. Mehta^1^, S.S. Solomon^3^

^1^Johns Hopkins Bloomberg School of Public Health, Baltimore, United States, ^2^YR Gaitonde Center for AIDS Research and Education, Chennai, India, ^3^The Johns Hopkins University School of Medicine, Baltimore, United States

**Background**: Respondent‐driven sampling (RDS) ‐ network referral/recruitment ‐ is an effective approach to reach people who inject drugs (PWID) at risk for or living with HIV. Yet, in most RDS samples, ∼30–60% of recruitment coupons remain unreturned. We explored the impact of integrating a geospatially targeted field‐based HIV testing strategy with an RDS survey.

**Methods**: PWID were first recruited in the town of Patti, Punjab near the India‐Pakistan border using RDS (Nov 2019‐Feb 2020) in which they reported on injection venues. HIV prevalence by venue informed field‐based testing (Feb‐May 2021; paused Mar 2020‐Feb 2021 for COVID‐19). Biometric data ensured individuals could only enroll in one of the two approaches. All participants completed a survey and underwent rapid onsite HIV testing. HIV RNA was quantified for positive participants. For each sampling approach, we calculated the number of: 1) HIV+; 2) undiagnosed (HIV+ but unaware of status [self‐report]), and 3) viremia (≥150 copies/mL). We compared the prevalence and identification rate (average number identified per day) by each approach for each outcome.

**Results**: 501 PWID were recruited using RDS in 81 days; median age was 29 years, <1% were female, and 94% reported active injection. In the geospatially targeted field‐based testing, 500 PWID were recruited in 81 days; median age was 31 years, 4% were female and 99.8% reported active injection. The prevalence of HIV in the RDS vs. field‐based testing was 12.6% vs. 28.6% (p < 0.01); and prevalence of viremia were 9.4% vs 14.8% (p < 0.01; Figure). The field‐based approach was significantly faster than RDS at identifying both PWID with HIV (1.77/day vs. 0.78/day; p < 0.01) and PWID with viremia (0.91/day vs. 0.58/day; p = 0.014).

**Figure:**
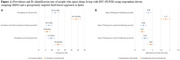
**Abstract PEMOC41‐Figure 1**.

**Conclusions**: A geospatially targeted field‐based testing approach was faster than a network‐driven approach at identifying PWID with transmission potential. Surveillance programs should consider capturing injection venues to facilitate such field‐based HIV testing approaches.

### PEMOC42 Adding to the HIV testing services toolkit! Caregiver‐assisted oral HIV screening of children 18 months – 14 years in Uganda and Zambia

C. Stecker ^1^, K. Paris^2^, F. Okello^3^, M.G. Alwano^3^, Z. Zyambo^4^, C. Chungu^4^, D. Oliver^1^, T. Lyon^1^, N.M. Tumwesigye^5^, A. Mukose^5^, C. Biribawa^5^, J. Kagaayi^5^, S. Kagongwe^5^, A. Nabuduwa^5^, C. Namanda^5^, M. Kaakyo^5^, M. Nsenga^5^, C. Pounds^5^, M. Hast^2^, S. Mutembo^6^, N. Moyo^7^, J. Matoba^7^, O. Chilyabanyama^7^, P. Ndubani^7^, A.C. Awor^8^, E. Nazziwa^8^, M. Adler^8^, M. Itoh^9^, M. Boyd^9^, G. Taasi^10^, G. Munthali^11^, M. Mwiya^11^, D. Mabirizi^1^, T. Fenn^1^, E. Rivadeneira^2^, J. Gross^2^

^1^Catholic Relief Services, Baltimore, United States, ^2^Centers for Disease Control and Prevention, Maternal Child Health Branch, Division of Global Health & TB, Atlanta, United States, ^3^Catholic Relief Services, Kampala, Uganda, ^4^Catholic Relief Services, Lusaka, Zambia, ^5^Makerere University, School of Public Health, Kampala, Uganda, ^6^Johns Hopkins, Baltimore, United States, ^7^Macha Research Trust, Choma, Zambia, ^8^Centers for Disease Control and Prevention, Kampala, Uganda, ^9^Centers for Disease Control and Prevention, Lusaka, Zambia, ^10^Ministry of Health, Government of Uganda, HIV Testing Services, Kampala, Uganda, ^11^Ministry of Health, Government of Zambia, Lusaka, Zambia

**Background**: An estimated 26,727 children living with HIV (CLHIV) in Uganda and 31,461 in Zambia need antiretroviral therapy (ART). Pediatric ART coverage is 68.7% in Uganda and 59.5% in Zambia for CLHIV 0–14 years. Innovative methods are needed to improve the identification of undiagnosed CLHIV. Studies in Uganda and Zambia evaluated the acceptability, feasibility, and effectiveness of caregiver‐assisted oral HIV screening of children 18 months – 14 years.

**Methods**: Forty‐seven facilities in Uganda (32) and Zambia (15) recruited parents/caregivers living with HIV who had children with an unknown HIV status from February‐October2021 to screen their children with OraQuick *Advance* ^©^ Rapid HIV‐1/2 Antibody test kits at home. Children with reactive oral HIV self‐test (HIVST) results received confirmatory testing per respective national guidelines. Children confirmed HIV‐positive were linked to ART. Acceptability, feasibility and effectiveness were evaluated through study registers documenting testing uptake/results returned, and a post‐use survey administered to parents/caregivers.

**Results**: Of the 4,059 eligible index parents/caregivers, 3,931 (96.8%) accepted to screen their 7,593 children with oral HIVST kit; 7,416 (97.7%) children completed oral HIVST with returned results. Among 2,722 caregivers surveyed, 2,639 (97.0%) reported HIVST was easy to use and 2,615 (96.1%) would recommend HIVST to other caregivers. One hundred‐nineteen (1.6%) children had a reactive HIVST, decreasing the need for facility testing by 98.4%. Of these, 116 (97.5%) completed blood‐based confirmatory testing. Forty‐three (37.1%) children were confirmed HIV‐positive and initiated on treatment with 97.7% (42) same‐day ART initiations. Eleven (0.4%) caregivers surveyed reported a child had minor reactions to oral HIVST.

**Figure:**
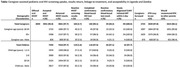
**Abstract PEMOC42‐Table 1**.

**Conclusions**: Caregiver‐assisted oral HIVST is an acceptable, feasible and effective option to screen high‐risk children who might not otherwise receive HIV testing and decongest health facilities. Policy makers may consider revised guidance to promote caregiver‐assisted oral HIV screening for children 18 months‐14 years, expanding community‐based pediatric testing options during the COVID‐19 pandemic.

### PEMOC43 Understanding the potential for harm among men who have sex with men using HIV self‐testing in the SELPHI online randomised controlled trial in England and Wales: a multi‐method study

T.C. Witzel ^1,2^, E.J. Nicholls^2,1^, L. McCabe^3^, P. Weatherburn^2^, S. McCormack^3^, C. Bonell^2^, M. Gafos^2^, F.C. Lampe^1^, A. Speakman^1^, D.T. Dunn^3^, D. Ward^3^, A.N. Phillips^1^, R. Peabody^4^, M.M. Gabriel^3^, Y. Collaco‐Moraes^3^, A.J. Rodger^1^, F.M. Burns^1^

^1^University College London, Institute for Global Health, London, United Kingdom, ^2^London School of Hygiene and Tropical Medicine, London, United Kingdom, ^3^University College London, Medical Research Council Clinical Trials Unit, London, United Kingdom, ^4^NAM: AidsMap, London, United Kingdom

**Background**: The potential of social‐harms (e.g. coercion to test, relationship breakdown) resulting from HIV self‐testing (HIVST) is a longstanding concern hindering widespread implementation. SELPHI (An HIV Self‐Testing Public Health Intervention) is the largest randomised trial of HIVST in a high‐income country. We aim to explore the relationship between HIVST and harm.

**Table:** 

**Abstract PEMOC43‐Table 1**.					
Type of harm	nBT %(n)	BT %(n)	nRT %(n)	RT %(n)	Overall
**Relationships**	N/A	1% (15/1626)	0.2% (1/468)	2% (9/581)	1% (25/2675)*
**Wellbeing**	N/A	1% (18/1611)	2% (8/467)	2% (11/580)	1% (37/2658)*
**Pressured/persuaded to test**	1% (14/1013)	1% (21/1615)	2% (8/471)	2% (11/579)	1% (54/3678)
**False positive**	1% (3/280)	3% (8/273)	2% (11/470)	2% (10/580)	2% (32/1603)

^*^Data not collected for nBT

**Methods**: Cis/trans men who have sex with men (MSM) aged 16+, reporting life‐time anal intercourse, resident in England and Wales were recruited through apps/social media, and randomised 60/40 to baseline HIVST (BT) or standard of care (no baseline HIVST (nBT)) between Feb2017‐Mar2018. A subset (received HIVST in BT, remained HIV‐negative, reported risk at 3‐months) were randomised to 3‐monthly HIVST (RT) or not (nRT). All received an exit survey including harms to relationships, well‐being, false positive results or being pressured/persuaded to test when they did not want to. Nine reporting harm in surveys were interviewed in‐depth; interviews were audio‐recorded, transcribed and analysed narratively.

**Results**: The sample of 10,111 were predominantly cis‐MSM (99%), 90% white, 88% gay, 47% university educated and 7% current/former PrEP users. Final survey response rate was: nBT = 26% (1056/4062), BT = 47% (2802/3567), nRT = 42% (510/1228), RT = 48% (581/1210).

Reports of harm were rare. In BT, nRT and RT combined, harms to relationships and to well‐being were reported by 1% (n = 25/2675) and 1% (n = 37/2658) respectively. In all arms combined, being pressured/persuaded to test was reported by 1% (n = 54/3678), and false positive results in 2% (n = 32/1603). Table 1 provides details by trial arm.

Qualitative analysis revealed harms emerged primarily from the kit itself (technological harm), the wider psychosocial intervention (intervention harms) or from the social context of the participant (socially emergent harms). Generally, intervention and socially emergent harms did not reduce HIVST acceptability, whereas technological harms did.

**Conclusions**: Reported harms were extremely rare in this large RCT of HIVST. Potential for harm should be considered in each context, and individuals experiencing negative impacts from HIVST linked to appropriate support services, but these concerns should not undermine roll‐out.

### PEMOC44 Offering HIV index contact testing to improve case identification and linkage to appropriate care among sexual partnersand biological children of PLHIV; Kavango East and West Namibia

U. Banda ^1,2^, H. Chirairo^2,1^, R. Mulang^2,1^, F. Munyayi^2,1^, E. Dzinotyiweyi^3^, G. Barnabee^1^, M. Golden^1^, T. Danda^2,1^, E. Likoro^3^, J. Kambanzera^2,1^, M. Sindimba^2,1^, N. Forster^2,1^, A. Ensminger^1^, G. O'Bryan^1^, D. Muronga^2,1^, R. Njengwa^3^, R. Muruti^2,1^

^1^International Training and Education Centre for Health, Department of Global Health, University of Washington, Seattle, United States, ^2^International Training and Education Centre for Health (I‐TECH), University of Washington, Windhoek, Namibia, ^3^Ministry of Health and Social Services, Windhoek, Namibia

**Background**: Despite progress in identification of people living with HIV (PLHIV), sexual contacts and biological children of PLHIV remain at significant risk of acquiring HIV. Introduction of Index Contact Testing (ICT) in routine HIV Testing Services (HTS) has improved case identification including in settings nearing epidemic control. In the Kavango regions, ICT was conducted at the facilities and in the community.

**Methods**: A retrospective data review of program outcomes from October 2020 to September 2021 was conducted at five selected high‐volume facilities in Kavango East and West. Newly diagnosed HIV‐positive individuals and PLHIV with viral loads >1000 copies/ml were offered ICT services using a structured interview guide. Recent sexual contacts and exposed biological children <18 years were listed. Index clients selected referral options for contacts following verbal informed consent and intimate partner violence (IPV) screening. Contact notification approaches included passive/self, provider, contract, and dual referral options. Contacts with unknown HIV status were notified, tested, and linked to ART or HIV preventions services depending on result. Data was captured using REDCap.

**Results**: ICT services were offered to 449 HIV‐positive individuals, 413 (92%) consented and listed 538 contacts (1.3 contacts elicited per index client) with 443 contacts eligible for testing and 254 (57%) tested. Of contacts eligible for testing, 78 (31%) newly tested HIV‐positive and 77 (99%) were linked to ART; 176 tested HIV‐negative of which 110 were sexual contacts. Of the 110 sexual contacts testing HIV‐negative, 78 (71%) were linked to PrEP. Overall, 32% of the listed contacts were HIV‐positive.

**Conclusions**: Offering ICT services to sexual contacts and biological children of PLHIV identified many newly diagnosed HIV positive cases at selected high‐volume sites. ICT services had a high rate of acceptance (consent), and more than one contact was elicited from each index client. A high proportion of contacts were linked to either ART or PrEP services.

### PEMOC45 A latent class analysis of combination HIV prevention strategies enacted by a prospective cohort of midlife and older men who have sex with men in the United States

S. Meanley ^1^, J.E. Egan^2^, D. Ware^3^, M. Brennan‐Ing^4^, S. Haberlen^5^, R. Detels^6^, A.L. Brown^2^, S. Wolinsky^7^, M.R. Friedman^8^, M.W. Plankey^3^

^1^University of Pennsylvania School of Nursing, Family and Community Health, Philadelphia, United States, ^2^University of Pittsburgh Graduate School of Public Health, Behavioral and Community Health Sciences, Pittsburgh, United States, ^3^Georgetown University Medical Center, Washington, D.C., United States, ^4^Hunter College, Brookdale Center for Healthy Aging, New York City, United States, ^5^Johns Hopkins Bloomberg School of Public Health, Epidemiology, Baltimore, United States, ^6^University of California ‐ Los Angeles Fielding School of Public Health, Epidemiology and Infectious Diseases, Los Angeles, United States, ^7^Northwestern University Feinberg School of Medicine, Infectious Diseases, Chicago, United States, ^8^University of Pittsburgh Graduate School of Public Health, Infectious Diseases and Microbiology, Pittsburgh, United States

**Background**: Midlife and older (age 40+) men who have sex with men (MSM) in the United States remain disproportionately affected by HIV. The HIV prevention toolbox has evolved over the past decade, offering biomedical methods, like pre‐/post‐exposure prophylaxis [PrEP/PEP] and HIV treatment as prevention alongside traditional methods, like condom use, anal sex alternatives, and discussions about HIV status. We aimed to identify the typologies of combination HIV prevention (CHP) methods used by midlife and older MSM during recent sexual encounters.

**Figure:**
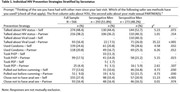
**Abstract PEMOC45‐Table 1**.

**Methods**: Participants were sexually‐active, midlife and older adult MSM (N = 566; mean age 61.1±8.2 years; 28.4% men of color) from the Multicenter AIDS Cohort Study/Healthy Aging Substudy (2016–2019). We asked about their/their partners’ enacted CHP methods (Table 1). Latent class analyses were performed cross‐sectionally to identify CHP typologies stratified by HIV serostatus.

**Results**: Seronegative men yielded a 3‐class CHP solution (Table 2); Class 1: high prevention orientation overall [43%], Class 2: low prevention orientation/anal sex abstention [15%], and Class 3: low prevention orientation overall [42%]. Seropositive men yielded a 4‐class CHP solution; Class 1: high prevention orientation overall [21%], Class 2: high prevention orientation/low condom use [27%], Class 3: low prevention orientation/moderate condom use [22%], and Class 4: low prevention orientation overall [30%].

**Figure:**
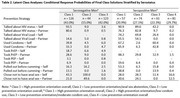
**Abstract PEMOC45‐Table 2**.

**Conclusions**: Participants' CHP typologies differed by serostatus suggesting that midlife and older adult MSM's prevention behaviors are not one‐size‐fits‐all. These findings support scaling up health providers’ service capacity to ascertain MSM's prevention behaviors to promote and build self‐efficacy for enacting the combination of effective strategies that align with patients’ preferences.

### PEMOC46 Attrition from antiretroviral treatment among adults in Mozambique, 2015–2019

L. Chaguala ^1^, A. Juga^2^, F. Ponda^1^, M.V. Jossefa^1^, N. Kamat^2^, D. Mugabe^1^

^1^National Institute of Health‐ Mozambique, National Health Observatory, Maputo, Mozambique, ^2^Centers for Disease Control and Prevention (CDC)‐ Mozambique, Epidemiology Branch, Maputo, Mozambique

**Background**: Mozambique has one of the largest HIV epidemics in Africa. Although access to antiretroviral therapy (ART) has increased, Ministry of Health reports 32% are not retained in care. We used national data to estimate 12‐month attrition and identify associated factors.

**Methods**: Data from adult (≥15 years) patients diagnosed with HIV who started ART between January 2015 and December 2019 were extracted from MozART, a national database covering 70% of all patients on ART in Mozambique. Attrition was defined as individuals who were either reported dead or who did not return to care for 90 days since their last clinical visit. Descriptive and multivariate regression analyses were conducted to identify attrition rates and associated factors.

**Results**: Of 15,634 patients who started ART between 2015–2019, 5% died and 17% did not return to care, for a 12‐month attrition rate of 22% (95% CI: 21.16–22.46%). Of all patients 53% were men, 38% were married, 40% completed primary education, 84% reported no history of alcohol consumption, and 43% were in WHO clinical stage III. The median age was 35 years (IQR: 29–43) and median CD4 cell count at ART initiation was 155 cells/mm^3^. WHO clinical stages IV (AOR = 2.09, 95% CI: 1.49 ‐ 2.91) and III (AOR = 1.56, 95% CI 1.28 ‐ 1.90), male sex (AOR = 1.53, 95% CI: 1.28 ‐ 1.81), and increased number of partners (AOR = 1.21, 95% CI: 1.02 ‐ 1.44) were associated with increased odds of attrition. Completing secondary education (AOR = 0.72, 95% CI: 0.56 ‐ 0.92) and residing in the northern region of Mozambique (provinces of Cabo Delgado, Nampula and Niassa) (AOR = 0.68, 95% CI: 0.56 ‐ 0.82) were associated with decreased odds of attrition.

**Conclusions**: This study shows that WHO clinical stages III or IV, and being male, were strongly associated with attrition. Reinforcement of interventions which target patients when they are still in early WHO clinical stages of disease and added support for patient groups at higher risk of attrition, could improve ART retention and thereby reduce HIV mortality in Mozambique.

### PEMOC47 Contrasting COVID‐19 and AIDS Orphanhood – How can AIDS inform this urgent need?

L. Sherr ^1^, S. Hillis^2^, L. Cluver^3^, S. Flaxman^4^, J. Unwin^4^, A. Butchart^5^, A. Vilaveces^2^, P. Goldman^6^, L. Rawlings^7^, P. Green^8^, C. Desmond^9^, C. Nelson^10^, Global Reference Group for Children Affected by COVID‐19

^1^University College London, Institute of Global Health, London, United Kingdom, ^2^CDC, Washington, United States, ^3^Oxford Unversity/UCT, Oxford, United Kingdom, ^4^Imperial College, London, United Kingdom, ^5^WHO, Geneva, Switzerland, ^6^Maestral, Washington, United States, ^7^World Bank, Washington, United States, ^8^World without Orphans, Washington, United States, ^9^University KwaZulu Natal, Durban, South Africa, ^10^Harvard, Boston, United States

**Background**: The AIDS Epidemic highlighted the plight of orphans and vulnerable children infected and affected by HIV globally. Specific funding and evidence based responses are needed. This study firstly contrasts global COVID‐19 orphanhood and parental loss with AIDS orphanhood figures to examine the global burden and inform the global response. Then secondly examines how the global HIV and AIDS evidence base can inform the COVID‐19 response.

**Description**: Parental or caregiver loss in childhood has long term consequences for development. An analysis of global data over time has enabled the creation of a model showing the global level of COVID‐19 losses of parent or primary caregivers. This analysis shows that over 6.7 million children have lost a parent or caregiver to COVID‐19. This number is greater than the current COVID‐19 global death rate. The rate of orphanhood has doubled every six months.A detailed examination of the data shows that two out of three children are adolescents with the remaining third under the age of 10. Furthermore three quarters of losses worldwide are paternal deaths leaving children vulnerable to exploitation, abuse, poverty and HIV‐infection, through elevated risk behaviours. The COVID‐19 data was contrasted with the HIV orphanhood and vulnerability data. It took 10 years for 5 million children to become orphaned and vulnerable due to AIDS. In contrast it has taken 2 years for 5 million children to experience orphanhood and vulnerability due to COVID. The impact of the overlap is unknown and uncharted . The HIV response to orphaned and vulnerable children provides a blueprint for action and a potential vehicle for an integrated and informed response.

**Lessons learned**: A strategy of ‘*prevent deaths, prepare families, and protect children*’ should be followed based on the learnings from the HIV AIDS epidemic. Evidence based cost effective programmes should inform the urgent response with accelerator evidence guiding programmes.

**Conclusions/Next steps**: COVID‐19 orphanhood and caregiver loss bears similarity with AIDS related orphanhood and vulnerability – although the rate of loss is accelerated. An integrated approach for all children is urgently needed with ongoing HIV and AIDS resources supplemented.

### PEMOC48 Trends in HIV viral load testing and viral suppression in US HIV cohort, 2017–2020

E. Tedaldi ^1^, Q. Hou^2^, C. Armon^2^, F. Palella^3^, J. Mahnken^2^, G. Simoncini^1^, J. Fuhrer^4^, C. Mayer^5^, K. Carlson^2^, K. Chagaris^2^, J. Li^6^, K. Buchacz^6^

 

**Figure:**
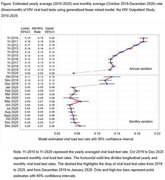
**Abstract PEMOC48‐Figure 1**.

^1^Lewis Katz School of Medicine at Temple University, Medicine/General Internal Medicine, Philadelphia, United States, ^2^Cerner Corporation, Kansas City, United States, ^3^Northwestern University Feinberg School of Medicine, Medicine, Chicago, United States, ^4^Renaissance School of Medicine Stony Brook University, Medicine, Stony Brook, United States, ^5^St. Joseph's Comprehensive Research Institute, Tampa, United States, ^6^Centers for Disease Control and Prevention, Division of HIV/AIDS Prevention, Atlanta, United States

**Background**: COVID‐19 pandemic effects on ambulatory care services for persons living with HIV in the United States, including HIV viral load (VL) testing frequency, and viremia (≥ 200 copies/mL) have not been well described.

**Methods**: We analyzed longitudinal data of study participants seen since January 1, 2019 at 8 HIV Outpatient Study (HOPS) sites. HIV VL test monthly rates were derived from generalized linear mixed models (GLMM), using data collected January 2010 to December 2020. We examined demographic and clinical correlates of viremia before and during the pandemic using GLMM logistic regression with the 2017–2020 data.

**Results**: Of 2,249 active patients, 75.8% were male, 58.1% aged ≥ 50 years, 40.6% non‐Hispanic White (NHW), 38.7% non‐Hispanic Black (NHB), 16.9% Hispanic/Latino (H/L), and 51.4% publicly insured. Median CD4 count was 669 cells/mm^3^ and 93% had a suppressed (< 200 copies/mL) VL on their last test before January 1, 2020. Monthly VL test rates as times/month (95% Confidence Interval) declined from 0.12 (0.10–0.14) in December 2019 to 0.05 (0.05–0.06) in January 2020, and subsequently stabilized with a rate of 0.07(0.05–0.08) in December 2020 (Figure). The model‐estimated percentage of VL tests ≥ 200 copies/mL out of all VL tests was 5.6% before the pandemic (2017–2019), and 4.9% in 2020 (P = 0.14). We detected no associations between key sociodemographic factors. Comparing the period before (2017–2019) and during (2020) the pandemic, the frequency of viremia over time was not significantly different by ethnicity, age, gender, type of insurance.

**Conclusions**: In the HOPS, HIV VL testing rates dropped by about half in early 2020 without year‐end recovery. The decrease in VL testing was not associated with changes in the relative frequency of viremia, both overall and by demographics including age, sex, race/ethnicity, and healthcare insurance. Longer term effects of the COVID‐19 pandemic upon HIV clinical care and outcomes, including viral suppression, require ongoing investigation.

### PEMOC49 Strategies to support effective use of the vaginal ring and oral PrEP among adolescent girls and young women in sub‐Saharan Africa: Qualitative findings from MTN‐034/REACH

S.T. Roberts ^1^, N. Mancuso^2^, K. Williams^3^, H.N. Kalule^4^, H. Mposula^5^, C. Mugocha^6^, P. Mvinjelwa^7^, M. Garcia^8^, D. Szydlo^9^, L. Soto‐Torres^10^, K. Ngure^11,12^, S. Hosek^13^

^1^RTI International, Women's Global Health Imperative, Berkeley, United States, ^2^RTI International, Global Public Health Impact Cener, Research Triangle Park, United States, ^3^RTI International, Applied Public Health Research Center, Research Triangle Park, United States, ^4^Makerere University‐Johns Hopkins University Research Collaboration, Kampala, Uganda, ^5^Wits Reproductive Health and HIV Institute, Johannesburg, South Africa, ^6^University of Zimbabwe Clinical Trials Research Centre, Harare, Zimbabwe, ^7^Desmond Tutu HIV Centre, Cape Town, South Africa, ^8^FHI360, Durham, United States, ^9^Fred Hutchinson Cancer Research Center, Statistical Center for HIV/AIDS Research and Prevention, Seattle, United States, ^10^National Institute of Allergy and Infectious Diseases, Division of AIDS, Bethesda, United States, ^11^Jomo Kenyatta University of Agriculture and Technology, Nairobi, Kenya, ^12^University of Washington, Seattle, United States, ^13^Stroger Hospital of Cook County, Chicago, United States

**Background**: Effective use of pre‐exposure prophylaxis (PrEP) has been low among adolescent girls and young women (AGYW) in sub‐Saharan Africa. The MTN‐034/REACH trial offered AGYW a menu of adherence support strategies and achieved high adherence to both daily oral PrEP and the monthly dapivirine vaginal ring. Understanding how these strategies promoted product use could inform design of adherence support systems in programmatic settings.

**Methods**: REACH was a randomized crossover trial evaluating safety of and adherence to the ring and oral PrEP among 247 AGYW (ages 16–21) living without HIV in South Africa, Uganda, and Zimbabwe. Adherence support included monthly counseling sessions with drug level feedback for all participants plus optional daily SMS reminders, weekly phone or SMS check‐ins, peer support groups, “peer buddies”, and additional counseling sessions. Through 16 focus groups, 24 sets of 3 serial in‐depth interviews (IDIs), and 37 single IDIs (n = 119 total), we used semi‐structured guides to explore experiences with adherence support options and how they encouraged product use. Coded transcripts were analyzed thematically using the Psychological Empowerment Framework.

**Results**: All strategies except for peer buddies were frequently used and highly valued. Counselors were perceived as friendly, trusted, and non‐judgmental; feeling ‘cared for’ by counselors during monthly sessions, additional sessions, and weekly check‐ins increased AGYW's perceived self‐worth and motivated adherence. Drug level feedback showing high adherence increased perceived competence, while results indicating medium‐to‐low adherence motivated improvement and facilitated open counseling conversations to identify and address challenges. Counseling and education increased perceived control over HIV acquisition by teaching AGYW how the products worked. In support groups, participants motivated each other, shared adherence challenges—especially on side effects, disclosure, and opposition to product use—and helped each other find solutions. These groups built self‐efficacy by normalizing experiences of experiencing and overcoming difficulties. SMS reminders and counseling exercises helped AGYW integrate oral PrEP into daily routines.

**Conclusions**: REACH empowered participants to adhere by creating positive, supportive environments and offering a choice of additional strategies to help identify and address use‐related challenges. PrEP implementation programs could support effective use through counseling and peer support groups focused on building motivation, self‐efficacy, and problem‐solving skills.

### PEMOD51 HIV care continuum outcomes after transition from pediatric to adult care

K. Doraivelu ^1^, M. Goldstein^2,3^, N. Shenvi^4^, K. Easley^4^, B. Zanoni^3^, A. Camacho‐González^2,3^, C. Del Rio^1,3^, S. Hussen^1,3^

^1^Rollins School of Public Health, Emory University, Hubert Department of Global Health, Atlanta, United States, ^2^Children's Healthcare of Atlanta, Atlanta, United States, ^3^Emory University School of Medicine, Atlanta, United States, ^4^Rollins School of Public Health, Emory University, Biostatistics, Atlanta, United States

**Background**: Youth living with HIV (YLH) receiving care in pediatric/adolescent settings will ultimately undergo healthcare transition (HCT) to adult‐centered care. HCT is viewed as a high‐risk time for care disengagement; however, there is a paucity of research documenting HIV care outcomes after HCT.

**Methods**: This is a prospective, observational cohort study of HCT among 70 YLH at an HIV care center in Atlanta, Georgia. Patients within three months of HCT were followed to determine clinical care outcomes through medical record abstractions at five timepoints: baseline, 6‐,12‐,18‐ and 24‐months. Outcomes were defined as

1. Linkage to adult care: at least one visit with an adult provider;
2. Retention in adult care: one visit in each post‐HCT abstraction period;
3. Viral suppression: maintaining HIV RNA <200 copies/mL.

Kaplan‐Meier curves were used to estimate time to linkage. Retention in care and viral suppression were estimated using generalized estimating equation analyses.

**Results**: The majority of our cohort identified as male (88.6%), Black (92.9%), horizontally‐infected (80%), and were virally suppressed (73%). Most of our cohort was linked to adult care by 6‐month (74%) and 12‐month (84%) follow‐up periods. Mean time to linkage was 105.8 days. Of those who linked to adult care by 12‐months, the retention rate was 86% (CI: 78–94%) at 6‐months, 76% (CI: 66–86%) at 12‐months, and 66% (CI: 55–78%) at 18‐ and 24‐months (Figure 1). Among those retained in care, the proportion with viral suppression was stable (74% at 6‐months, 77% at 12‐months, 67% at 18‐months, and 78% at 24‐months).

**Figure:**
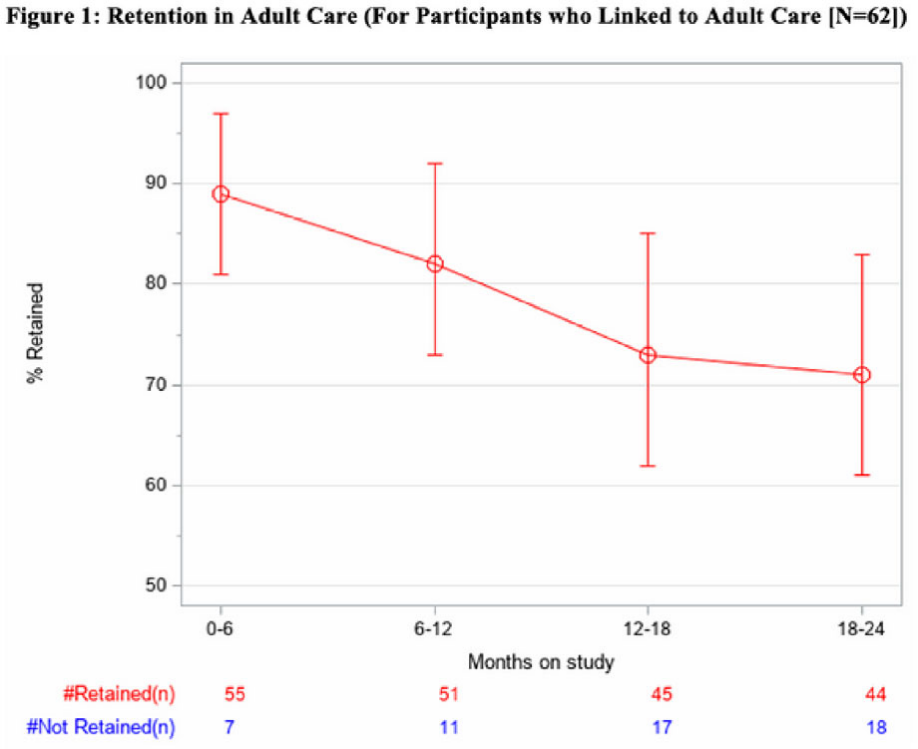
**Abstract PEMOD51‐Figure 1**.

**Conclusions**: Although a majority of YLH in our cohort linked to adult care, rates of retention decreased over the 24‐month follow up period. Rates of viral suppression were suboptimal, though stable. Current interventions focus mostly on preparation for linkage to adult care; however, our data suggest that strategies to enhance retention in care after HCT will be critically important for YLH.

### PEMOD52 It's time to think positive about HIV – a strengths‐based campaign to end HIV stigma from New South Wales, Australia

M. Vaughan ^1^, K. Johnson^1^, L. Rabie^1^

^1^ACON, Surry Hills, Australia

**Background**: Campaigns to address HIV stigma often aim to raise awareness by communicating the experiences of people living with HIV (PLHIV) with stigma. In a new approach, ACON's innovative anti‐stigma campaign *It's Time to Think Positive About HIV* reorientates how campaigns address HIV stigma. Rather than focus on the harms of HIV stigma, *Think Positive* celebrates HIV allyship and calls on the broader community, with a focus on those who are HIV negative, to confront HIV stigma together. Featuring Australian community members, *Think Positive* providesa blueprint to challenge HIV stigma through an uplifting, strengths‐based message that celebrates allyship.

**Description**: Aligned with best practice and linked to the Greater Involvement and more Meaningful Engagement of PLHIV (GIPA/MIPA) principals, staff living with HIV led the conceptualisation, development, and delivery of the campaign. *Think Positive* launched on 1 September 2021 and ended promotions on 31 October 2021. Hosted on YouTube and embedded on ACON's Ending HIV campaign platform, the *Think Positive* landing page achieved 12,640 page views, suggesting robust engagement with the message. Beyond ACON web channels, *Think Positive* video content achieved 395,533 views across Facebook, Instagram, and YouTube.

**Lessons learned**: In an internal evaluation, 97% of survey respondents considered the main campaign video messaging to be extremely, very, or moderately effective. *Think Positive* demonstrates that anti‐stigma campaigns can be constructed as uplifting messages that are empowering and optimistic for PLHIV. Anti‐stigma messaging does not need to shame behaviour or showcase harms, but rather can encourage better allyship and collective community care. This new approach relieves the burden on PLHIV around stigma.

**Conclusions/Next steps**: Addressing HIV stigma and improving quality of life for PLHIV should be prioritised in the global HIV response with equal importance to testing, treatment, and prevention. Reducing HIV stigma is much more than building resilience in PLHIV, who have carried this burden through the history of the epidemic. Instead, positive examples that engage all of community and provide people with both a blueprint and vocabulary to end HIV stigma should be developed. Guiding people towards high quality HIV allyship should be core to these efforts.

### PEMOD53 “My attitude towards my own journey changed the way others see me and treat me”: Insights from male peers living with HIV on challenging and changing stigma

L. Rambally Greener ^1^, S. Malone^1^, S. Shabalala^2^, P. Pitsillides^2^, T. Grenville ‐ Grey^2^, N. Hasen^1^

^1^Population Services International, HIV / TB, Johannesburg, South Africa, ^2^Matchboxology, Johannesburg, South Africa

**Background**: HIV‐related stigma and discrimination remain significant barriers to achieving the UNAIDS 95‐95‐95 targets and realizing optimal outcomes for people living with HIV (PLHIV). Among healthcare workers (HCW) living with HIV, there is a complex and multi‐layered intersection of both externalized and internalized stigma particularly among peer navigators who work closely with communities. Efforts are needed to directly address stigma reduction in HIV programs and create a safe and supportive working environment for HCWs.

**Description**: Programmes that engage with stigma at both inter‐personal and community support environments in which stigma and discrimination are no longer accepted or practiced. We explored the experiences and perceptions of male peer navigators living with HIV‐ referred to as coaches‐, with the aim of understanding how they have addressed stigma in their personal and work lives. We used a qualitative design involving individual semi‐structured in‐depth discussions with 45 coaches in 5 provinces in South Africa.

**Lessons learned**: By embracing their HIV status publicly and countering outdated notions of what it means to live with HIV coaches reported being able to overcome internal stigma by reframing their experience of HIV as a resource for advising and supporting other men living with HIV.

*“Self‐acceptance is the first step to healing and setting the tone on how others will treat you.” – Coach, Free State*

*“Stigma will always be there from lack of information, but we move on, and now we are comfortable in our own skin.” – Coach, Gauteng*

*“People do not believe when I disclose my status during campaigns because I am healthy and confident.” – Coach, Free State*

The social and professional standing that they hold as healthcare workers and the purpose and meaning that they find in their work appear to serve as protective factors.

**Conclusions/Next steps**: The Coach intervention has demonstrated that empathy and disclosure from a healthcare worker can reduce stigma and encourage others to seek care. These approaches are interdependent and mutually reinforcing and have consequences for the way in which people react to others in their community as well as within healthcare facilities. This suggests that greater visibility of PLHIV could be a significant element of stigma reduction strategies.

### PEMOD54 “I am a rural woman and living with HIV, how bad can it get?”: Exploring the challenges of women living with hiv and AIDS in rural ghana

N.A. Acheampong ^1^, T. Nyarko^1,2^, R. Afriyie^1^, O. Graham^3^

^1^Ghana AIDS Commission, Accra, Ghana, ^2^Cocoa Research Institute of Ghana, Rural Sociologist, Akyem New Tafo, Ghana, ^3^Ghana AIDS Commission, Kumasi, Ghana

**Background**: The stigma and discrimination against Persons Living with HIV and AIDS (PLHIV) in Ghana is nothing short of abominable and for PLHIV in rural Ghana where traditional cultures are entrenched, it is virtually abusive. Women living in rural Ghana and having HIV therefore, face unique vulnerabilities that need to be explored in order to appreciate their distinct challenges and make programmatic interventions more effective.

**Description**: Qualitative data collection method of Focus Group Discussions was employed in the study in 2021. 5 FGD made up of 7 participants each were organized in 5 rural districts in the Ashanti Region of Ghana. The 35 respondents of rural women living with HIV were purposely selected. The discussions were audio recorded after getting the informed consent of participants. The recorded data was transcribed and the transcripts were thematically analyzed.

**Lessons learned**: It was revealed during the interactions that the participants face the triple burden of being women, living in a rural area, and living with HIV. Negotiating how to lessen the effects of these burdens is both problematic and extremely difficult. The perception that HIV is the curse of the gods, and hence has a spiritual aetiology makes these women ultimately responsible for their condition. The women living with HIV are considered to be prostitutes or have caused a sacrilege. Their male counterparts are treated differently and more positively. It was further observed that women living with HIV and AIDS have limited economic opportunities, as those selling food see their businesses folding up because their customers after knowing their status stop buying from them.

**Conclusions/Next steps**: There is a need to increase facility lead community outreach services for both HIV sensitization and education and testing. The majority of the residents will be reached through outreach led by a health facility.

### PEMOD55 Acceptability of raltegravir granule use for neonates diagnosed with HIV at birth by healthcare workers and caregivers in Zimbabwe: A qualitative analysis

M. Murandu ^1^, L. Katirayi^2^, C. Stecker^3^, P. Andifasi^4^, A. Mushavi^4^, T. Maphosa^5^, V. Thorsen^6^, G. Gombakomba^1^, M. Mungati^1^, L. Denoeud^7^, E. Rivadeneira^6^, R. Weber^5^, S. Hrapcak^6^

^1^Elizabeth Glaser Pediatric AIDS Foundation, Harare, Zimbabwe, ^2^Elizabeth Glaser Pediatric AIDS Foundation, Washington D.C., United States, ^3^Catholic Relief Services, Baltimore, Maryland, United States, ^4^Ministry of Health and Child Care, Harare, Zimbabwe, ^5^Centers for Disease Control and Prevention, Harare, Zimbabwe, ^6^Centers for Disease Control and Prevention, Atlanta, Georgia, ^7^Elizabeth Glaser Pediatric AIDS Foundation, Washington D.C, United States

**Background**: In 2020, Zimbabwe adopted the World Health Organization recommendation to use raltegravir (RAL) granule‐based regimens for the treatment of neonates identified with HIV through birth testing. This study explores the acceptability of RAL granule use by caregivers and health care workers (HCWs).

**Methods**: Interviews were conducted with 15 caregivers and 12 HCWs from a subset of 14 health facilities in Zimbabwe participating in the initial roll‐out of RAL granules. HCWs identified eligible caregivers that had administered RAL to their infant and attended the 8^th^ and/or 28^th^ day of life appointments. Caregivers were selected in the order of whose neonates were most recently initiated on RAL, and HCWs were identified from these same facilities. Through convenience sampling, eligible HCWs who provided RAL preparation, administration instructions, and support to caregivers with neonates on RAL for at least three months were recruited. Caregivers and HCWs were interviewed on the same day. Transcripts were coded using the MAXQDA software and thematically analyzed.

**Results**: Caregivers reported their babies looking healthier after initiating RAL, noting improvements in skin appearance and weight. Some caregivers wanted their child to remain on RAL at the day 28 appointment instead of switching as recommended by national guidelines, and others recommended the national roll‐out of RAL. HCWs felt that RAL granules were an improvement from previous neonatal antiretroviral medications stating beliefs that RAL improved health outcomes compared to previous regimens.

HCWs reported challenges with caregivers understanding dosing instructions, measuring with a syringe, the need to swirl and not shake the medicine, discarding unused medication, and changes in dosing schedule and amount if RAL was initiated a few days after birth. HCWs stated that adequate counseling, demonstrations, and repeat demonstrations were crucial to ensure that caregivers clearly understood RAL dosing instructions. HCWs requested more standardized training targeting nurses, guidance on handling missed doses, and clarity on instructions to not mix RAL granules with breastmilk.

**Conclusions**: While positive feedback was provided by caregivers who used RAL granules and HCWs, additional steps are needed for adequate training of HCWs and sufficient caregiver instruction and support to ensure that RAL granules are prepared and administered correctly.

### PEMOD56 Risk, vulnerability, and protective factors for HIV and STI infection among adolescent girls and young women in West and Central Africa: pooled analysis of available Demographic and Health survey data

S.K. Brar ^1^, J.G. Rosen^2^, P.T. Yeh^2^, L. Dongmo Tsague^3^, D. Walker^4^, C. Lwamba^1^, J. Harris Requejo^1^, C.E. Kennedy^2^

^1^UNICEF, Data & Analytics Section, Division of Data, Analytics, Planning, and Monitoring (DAPM), New York, United States, ^2^Johns Hopkins Bloomberg School of Public Health, Department of International Health, Social and Behavioral Interventions Program, Baltimore, United States, ^3^UNICEF, West and Central Africa Regional Office, Dakar, Senegal, ^4^UNICEF, HIV/AIDS, Health Programme Group, New York, United States

**Background**: Adolescent girls and young women (AGYW) are considered at heightened risk for HIV in sub‐Saharan Africa, but most data on risk and vulnerability factors for this group comes from Eastern and Southern Africa, where HIV prevalence is highest. We assessed risk and vulnerability factors across nationally representative household surveys in West and Central Africa to better understand relationships and inform policy and programmatic decision‐making in this region.

**Methods**: We used cross‐sectional data from sexually‐active AGYW participating in Demographic and Health Surveys from 17 West and Central African countries. Sexually transmitted infection (STI) symptomatology was used as a proxy for HIV risk. Modified Poisson regression models (for binomial outcomes) estimated prevalence ratios for associations between risk and vulnerability factors and past‐year STI symptomatology among AGYW. Models were stratified by five‐year age bands (15–19, 20–24, 25–29 years) as well as current marital status to identify stratum‐specific risk and vulnerability factors associated with STI symptomatology.

**Results**: Almost three‐fourths of AGYW were married/cohabiting and 40% had completed secondary or higher education. Prevalence of past‐year symptomatology was 20.7%. Consistent and strong associations existed between several risk and vulnerability factors and STI symptomatology. Older age (25–29‐year‐olds) and secondary or higher education were associated with increased prevalence of past‐year STI symptomatology in the pooled AGYW sample and among married AGYW, specifically. Urbanicity and secondary or higher education were associated with increased prevalence of past‐year STI symptomatology across five‐year age bands. Employment, migration and accepting spousal violence were strongly associated with past‐year STI symptomatology among all three age groups, with adjusted prevalence ratios (aPR) ranging from 1.13–1.33 (p < 0.0001). Multiple sexual partners and unprotected last sex were also strongly associated among all three age groups, with aPR ranging from 1.35–1.64 (p < 0.0001).

**Conclusions**: These findings indicate heterogeneities in STI symptomatology by key sociodemographic characteristics and corresponding sources of risk and vulnerability among AGYW, highlighting the need to develop effective policies to address increased risk and vulnerability of AGYW to HIV and STI infection. HIV/STI prevention interventions should focus on supporting individual agency to engage in safer sexual practices and addressing harmful gender norms and practices, including acceptance of spousal violence.

### PEMOD57 How can text‐messaging change the context of living with HIV and engaging with treatment in Iran: building a realist programme theory

V. Ameli ^1^, G. Wong^2^, J. Barlow^3^, F. Meinck^4^

^1^Vira Ameli, Social Policy and Intervention, Oxford, United Kingdom, ^2^University of Oxford, Primary Healthcare Sciences, Oxford, United Kingdom, ^3^University of Oxford, Social Policy and Intervention, Oxford, United Kingdom, ^4^University of Edinburgh, School of Social and Political Sciences, Edinburgh, United Kingdom

**Background**: Text‐messaging interventions are increasingly used to address the complex challenges of lifetime antiretroviral therapy (ART). A wealth of evidence supports the efficacy of text‐messaging in improving treatment adherence, yet there is a dearth of evidence on *how* text‐messaging is improving outcomes and *why* it works in a particular context. This study uses stakeholder views to theorise *how* and *why* text‐messaging could change the context of living with HIV and engaging with ART in Iran.

**Methods**: The study is part of an ongoing research project on ART engagement among Iranians living with HIV. We draw on the perspectives of recently‐diagnosed and treatment‐experienced patients and their providers, using purposive sampling, conducting 17 individual interviews and 9 focus group discussions. A realist framework of inquiry is utilised to conceptualise how interventions alter the context of people's lives by providing new paths for individual reasoning and [re]action, thereby producing altered outcomes.

**Results**: Our findings build on the previously identified socio‐ecological pathways that disrupt ART in Iran (Figure 1).

We found that participants perceive text‐messaging can improve adherence by changing socio‐ecological pathways and the relation between the health‐system and patients in terms of support, motivation, initiative, and information (Figure 2).

**Conclusions**: We believe this is the first study to explain how and why text messaging works to reduce disruption in ART treatment. It identifies the important contexts that interventions need to address for desired outcomes to occur. The realist programme theory developed here can inform future design and evaluation of interventions and trials.

**Figure:**
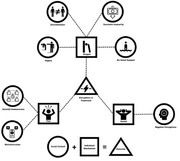
**Abstract PEMOD57‐Figure 1**.

**Figure:**
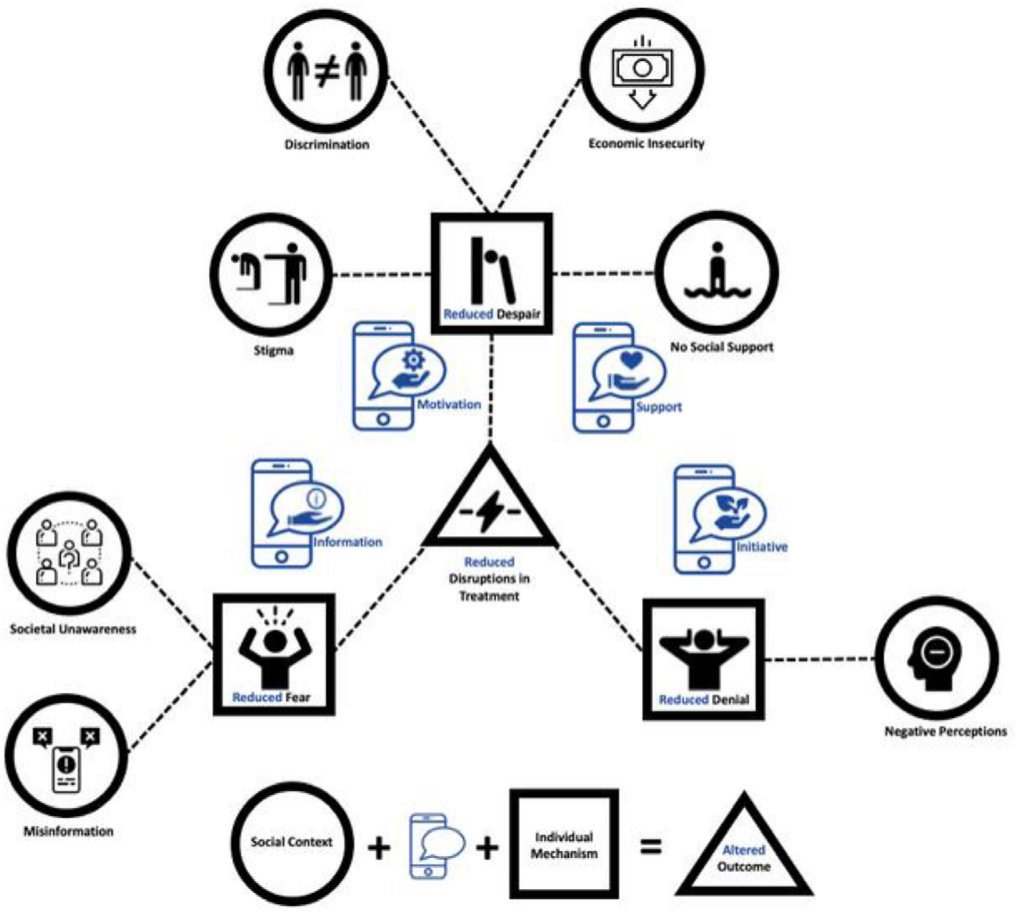
**Abstract PEMOD57‐Figure 2**.

### PEMOD58 Impact of COVID‐19 prevention measures including restrictions on socio‐economic lives of people living with, at high risk of and those affected byhivonaccess, utilization of essential health services

N. Jackie ^1^, L. Mworeko^1^, B. Ajonye^1^

^1^International Community Of Women Living with HIV Eastern Africa, Advocacy, Kampala, Uganda

**Background**: Through its COP20, PEPFAR is supporting ICWEA, HEPS Uganda and SMUG to implement a scale up phase of the Community Led Monitoring model of health services delivery in Uganda. The model trains, supports, equips affected PLHIV communities carry out routine monitoring of HIVTB services aimed at improving quality, availability, accessibility and utilization of these services. the quality and accessibility of HIV services.

**Description**: In June 2021, An assessment on the impact of COVID‐19 prevention measures including restrictions on the socio‐economic lives of people living with, at high risk of and those affected by HIV on access and utilization of essential health services. The process involved consultations with key stakeholders and communities of people living with HIV and at high risk of HIV infection.

Data was collected through face‐to‐face interviews and online and telephone. The assessment involved 300 respondents across 46 districts in Uganda. Most of people living had difficulties accessing social or essential health services and these were more likely to experience drop out of treatment, relapses, stigma and discrimination in the communities, shortage of some essential health commodities such as ARVs, condoms and lubricants.

**Lessons learned**: A total of 310 people living with HIV consented and participated in the survey. Overall, 60% were female, 38.5% were males while 1.5% preferred not to identify their gender. The survey considered participants above 18 years of age and the results show that majority of the respondents were aged above 38 years. 72% of them suffered a reduction in the number of health workers. Only 41% of the clients were able to go for their clinic appointments of the 67% who had appointments during the COVID‐19 era. 75% of the respondents acknowledge that they were stigmatized, discriminated against especially by law enforcement officers while travelling for ART refills on clinic days.

**Conclusions/Next steps**: Interventions to reduce the spread of COVID‐19 in some situations as the attention is focused on controlling the epidemic, implications on access to health services such as drug stock outs were noted at facilities. Mitigating collateral impact of lockdowns where possible interventions, ensure continuity of access to essential services and strengthen support systems of PLHIV.

### PEMOD59 Assessing the impact of COVID‐19 on HIV prevention among rural populations in South‐West Uganda

R. Gumbie ^1^, L. Akullu^2^, J. Van Leeuwen^1^, H. Nabimanya^2^, C. Buchholz^1^, J. Amanya^1^, I. Nkonge^2^, B. Muhindo^2^, H Amutuhaire^2^, R. Grundy^1^

^1^Global Livingston Institute, Denver, United States, ^2^Reach a Hand Uganda, Kampala, Uganda

**Background**: The global focus and prioritization of health systems was altered as a result of the COVID‐19 pandemic. Social distancing and stay‐at‐home measures, termed “lockdowns” by the WHO, dominated public health approaches. Uganda was one of the countries with the strictest lockdown measures in the world, only re‐opening schools in January 2022, 20 months after the pandemic began. The full impact of the pandemic on HIV incidence and prevention is still under investigation, as the Gates’ Foundation Goalkeepers report 2021 found that the global goal to fight HIV had been set back 25 years in 25 weeks.

**Methods**: Since 2014, the ‘iKnow Kati’ HIV awareness and prevention program implemented by RAHU and the GLI, has provided channels for youth to obtain HIV prevention information, testing, counselling, and treatment services. As a result, the campaign held on World AIDS Day 2020 targeted vulnerable groups that were disproportionately affected by the COVID‐19 pandemic. We trained peer educators within 5 districts to collect data through a cross‐sectional survey. Participants who accessed health outreach services responded to questions about the campaign and COVID‐19.

**Results**: Out of 655 respondents, 65% were female and 35% male with the highest percentage age groups falling between 20–29 years old. When asked about how COVID‐19 had impacted their health seeking behaviour, nearly 25% of respondents mentioned that lack of transportation was a barrier to seeking health services. About 12% indicated that finances played a role, while 3.7% mentioned the lockdowns, and 3% mentioned stigma and fear.

**Conclusions**: While the full impact of the COVID‐19 pandemic on HIV services is still being researched, the Gates Foundation Goalkeeper's report identified that some of the most effective interventions have happened at the hyperlocal level, headed by leaders who have worked long and hard to earn the trust of their communities. Our results show that access to health services through provision of accessible programs designed with specific communities in mind is effective. There is evidence that lack of transportation, finances and stigma and fear play related to COVID‐19 played a role in health seeking behaviour.

### PEMOD60 Impact of the COVID‐19 pandemic and related restrictions on the lives of young people living with HIV in Kisumu, Kenya

J.M. Zech ^1^, A. Zerbe Buba^1^, M. Mangold^2^, S. Akoth^3^, R. David^3^, J. Odondi^3^, D. Naitore^3^, K. Ndede^3^, M. Hawken^3^, T.G. Harris^1^, E.J. Abrams^1^

^1^ICAP at Columbia University, New York City, United States, ^2^Columbia University Irving Medical Center, New York City, United States, ^3^ICAP Kenya, Nairobi, Kenya

**Background**: Adolescents and young adults with HIV (AYAHIV) may be particularly vulnerable to the impact of the COVID‐19 pandemic and associated mitigation measures which can adversely impact fragile social and economic systems. We examined the impact of the pandemic and related government mandated restrictions among AYAHIV in Kisumu, Kenya.

**Methods**: Between April‐May 2021, a cross‐sectional survey was conducted among a convenience sample of AYAHIV 18–25 years aware of their HIV status and receiving HIV care at Jaramogi Oginga Odinga Teaching and Referral Hospital. Information on demographics, COVID‐19 knowledge, protective measures, and the impact of the pandemic and related restrictions (i.e., curfews, lockdowns, school/workplace closures) on their daily lives and well‐being since the start of the pandemic was collected. Responses were analyzed using descriptive statistics.

**Results**: Of 275 AYAHIV enrolled: median age 22 years (IQR: 19–24 years); 178 (65%) female; 222 (81%) completed some secondary education or higher; 108 (39%) lived in an informal housing area. Awareness of COVID‐19 was high (99%), mean COVID‐19 knowledge score was 4.32 (SD: 0.93; range 1–5) and most reported taking protective measures, including frequent handwashing (91%) and face mask use (85%). Over half (55%) reported recently going to a crowded place, including church (78%) and bars/clubs (13%). Overall, 193 (70%) felt COVID‐19 and associated restrictions impacted them including affecting their daily routine (38%), views on travel/immigration (22%), and relationships (14%). Almost half (49%) reported changes in living situation; 24% living with different people, 11% moved/relocated, and 5% newly living on the street. Additionally, AYAHIV reported increased verbal arguments (30%) and physical conflict (16%) at home with 8% reporting someone having used/threatened them with a weapon, 12% experiencing physical abuse, 7% were touched in a sexual way without permission, and 5% had forced sex.

**Conclusions**: AYAHIV in Kenya were knowledgeable about COVID‐19 and prevention practices despite inconsistent adherence. Impacts of the pandemic and related restrictions were felt across various aspects of AYAHIV's lives, including disrupted living situations and increased exposure to verbal and physical conflict, including sexual violence. Interventions are needed to address the impact and potential negative long‐term effects of the pandemic on AYAHIV health and well‐being.

### PEMOD61 Improving access to HIV services while minimizing potential exposure to COVID‐19 among men who have sex with men (MSM) in Ghana

S.E. Owusu ^1^, C. Asamoah Adu^2^, S.K. Wosornu^3^, K. Diaba^4^, C. Yalley^1^, G. Ekem‐Ferguson^5^

^1^Maritime Life Precious Foundation, Programs, Takoradi, Ghana, ^2^Ghana‐West Africa Program to Combat AIDS and STIs (WAPCAS), Executive Directorate, Accra, Ghana, ^3^Maritime Life Precious Foundation, Executive Directorate, Takoradi, Ghana, ^4^Ghana‐West Africa Program to Combat AIDS and STIs (WAPCAS), Programs, Accra, Ghana, ^5^Ghana Health Service, Clinical Psychology, Accra, Ghana

**Background**: Ghana experienced disruptions in providing HIV services to MSM due to COVID‐19. Factors such as social distancing which restricts large group outreaches; reduced demand for services because of fear of COVID‐19 transmission in facilities; and reduced availability of services as providers are assisting with pandemic response affect delivery of HIV services for MSM. Maintaining uninterrupted access to essential HIV services for MSM during the pandemic require using integrated and community‐based strategies that minimizes potential risk for COVID‐19 exposure.

**Description**: Maritime‐Foundation introduced various community‐based approaches to HIV service delivery for MSM during the pandemic in three districts. Peer educators were trained to provide education on COVID‐19 during their community outreach activities. Authorization was sought for outreach workers in lockdown areas and provided with PPEs during delivery of physical outreach services.

Flexible strategies were implemented to preserve access to HIV services and promote safety of staff and clients during the pandemic: (1) Social media platforms were used to engage peers for HTS and support PLHIVs through virtual case management; (2) HTS and treatment took place at homes and safe locations identified and agreed by peers at their own convenience; (3) Condoms and lubricants were made available at community‐led Drop‐In‐Centers and outlets for easy access; (4) The program promoted multi‐month dispensing of ART and PrEP to eliminate clinic visits.

**Lessons learned**: Introduction of community‐based strategies during the pandemic reached out to more MSM and increased HIV+ yield across the three districts. During the pandemic between February to April 2020, 445 new MSM were reached and tested for HIV; 32 were diagnosed positive. After the introduction of community‐based strategies, between May and July 2020, 634 new MSM were reached and tested; 89 were diagnosed positive.

**Conclusions/Next steps**: CSOs can adopt tailored community‐based approaches that can be integrated into HIV programs to improve results in reaching, testing and linking MSM in times of a pandemic.

Scaling up community‐based approaches to HIV service delivery can help safeguard the hard‐fought gains of the global HIV response. If these solutions are sustained beyond the pandemic, they may serve to modernize KP programming and position it to be more effective in our new reality

### PEMOD62 Factors influencing COVID‐19 Vaccine Acceptability in Women living with, or at High Risk of HIV in South Africa

H. Humphries ^1^, L. Lewis^1^, S. Choonara^1^, E. Lamontagne^2^, A. Yakusik^2^, K. Dikgale^1^, N. Mkhize^3^, B. Kuwane^4^, D. Massawe^5^, A. Matendawafa^5^, O. Arije^6^, M. Folayan^6^, Q. Abdool Karim^1^

^1^Centre for the AIDS Programme of Research in South Africa (CAPRISA), Durban, South Africa, ^2^UNAIDS, Geneva, Switzerland, ^3^AIDS Foundation South Africa, Durban, South Africa, ^4^Youth Health Africa, Johannesburg, South Africa, ^5^African Alliance, Johannesburg, South Africa, ^6^Obafemi Awolowo University, College of Health Sciences, Ile‐Ife, Nigeria

**Background**: COVID‐19 vaccines offer hope for a return to ‘normality’ but uptake varies. Previous research reported a 67% acceptability rate amongst the general South African population. This study investigated factors influencing vaccine hesitancy amongst adolescent young girls and young women (AGYW), and key populations (KPs) living with, or at high risk of HIV living in South Africa.

**Methods**: We analyzed data from a cross‐sectional survey of women (AGYW, HIV‐positive women, sex workers, LGBTQ+ women, migrants and women using drugs) from four provinces in South Africa, conducted between September‐November 2021. Surveys collected information on demographics, self‐reported HIV status, vaccine hesitancy, and the impacts of COVID‐19. We defined vaccine hesitancy as disagreeing with the statement: ‘When a vaccine for COVID‐19 is available to me, I will get it’. Prevalence of vaccine hesitancy was quantified and factors associated with hesitancy were measured using multivariable logistic regression.

**Results**: Of the 2,812 women interviewed, 2,763 (98%) responded to the question on vaccine hesitancy and 2,332 (82.4%) reported their HIV status. From the 2,320 women with complete vaccine and HIV status data, 14.4% reported being vaccine‐hesitant. Prevalence of vaccine hesitancy was 13.4% among HIV‐positive women, 18.3% among women 15–24 years‐old, 16.9% among LGBTQ+ individuals, 29.3% among migrants (n = 99), 17.6% among sex workers, 18.1% among women with disabilities (n = 166) and 25.6% among drug‐users. The three most common reasons for vaccine hesitancy regardless of HIV‐status or sub‐population were being scared of side‐effects (57.9%), a lack of trust in authorities (32.5%) and being anti‐vaccines (23.9%). Vaccine hesitancy was positively associated with age ˂25 years (adjusted odds ratio (aOR): 1.73(95% confidence interval (CI): 1.35–2.22), incomplete secondary schooling (aOR: 1.52(95% CI: 1.06–2.16), economic vulnerability (aOR: 1.43(95% CI: 1.00–2.05) and reporting anxiety/depression (aOR: 1.81(95% CI: 1.35–2.42). Previous COVID‐19 infection in the household was associated with reduced vaccine hesitancy (aOR: 0.41 (95% CI: 0.28–0.61).

**Conclusions**: This study demonstrated low vaccine hesitancy amongst AGYW and KP's. Our more granular analysis highlighted that broader contextual socioeconomic concerns, anxiety and youth affect vaccine uptake ‐ mediated by concerns about side‐effects, vaccine safety and social status. Customized interventions, adjusted with time, that build trust in vaccines and enhance uptake are needed.

### PEMOD63 Pregnancy history and unmet need for contraception among female sex workers living with HIV in Durban, South Africa

C. Comins ^1^, K. Rucinski^2^, M. Mcingana^3^, N. Mulumba^4^, L. Shipp^1^, H. Hausler^5^, S. Baral^1^, S. Schwartz^1^

 

**Figure:**
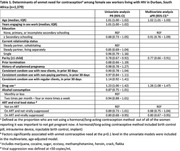
**Abstract PEMOD63‐Table 1**.

^1^Johns Hopkins Bloomberg School of Public Health, Epidemiology, Baltimore, United States, ^2^Johns Hopkins Bloomberg School of Public Health, International Health, Baltimore, United States, ^3^TB HIV Care, Key Populations Program, Cape Town, South Africa, ^4^TB HIV Care, Key Populations Program, Durban, South Africa, ^5^TB HIV Care, Cape Town, South Africa

**Background**: In South Africa, an estimated 60% of female sex workers (FSW) are living with HIV, with studies indicating that the majority of FSW are mothers and primary caregivers. This analysis aims to characterize pregnancy history, fertility intentions, and unmet need for contraception among FSW living with HIV.

**Methods**: Non‐pregnant cisgender FSW living with HIV ≥18 years of age were enrolled into the Siyaphambili trial in Durban, South Africa (June 2018‐March 2020). Fertility intentions and contraceptive and antiretroviral therapy (ART) use were captured at baseline through an interviewer‐administered questionnaire; viral load was measured. Multivariable robust Poisson regression was used to estimate the association between correlates of unmet need for contraception at enrollment, controlling for age and education.

**Results**: Of the 1,379 FSW living with HIV enrolled in Siyaphambili, 85.2% had previously been pregnant. History of unplanned and unwanted pregnancy was high (90.7% and 90.2%, respectively). Nearly all (95.2%) reported it was important to “avoid pregnancy now”, but 18.8% desired future children. Use of hormonal/long‐acting contraception was low (35.2%), and consistent condom use in the prior 30 days was limited with both clients and non‐paying partners (59.7% and 49.5%, respectively). Of the 485 using hormonal/long‐acting contraception, 80.0% used injectable birth control. Unmet need for contraception was 60.1%. Participants with ≥1 child and those on ART and virally suppressed in comparison to those not on ART were less likely to have unmet contraceptive needs (aPR: 0.77, 95% CI: 0.66–0.91; aPR: 0.80, 95% CI: 0.67–0.95, respectively), and participants reporting current drug use were more likely to have unmet contraceptive needs (aPR: 1.26, 95% CI: 1.08–1.47) [Table 1].

**Conclusions**: Moving forward, strategies to couple fertility intention screening and contraceptive care with ART are needed to optimize reproductive health and decrease vertical HIV transmission. Further, understanding unmet contraceptive needs is critical to preventing unintended pregnancies and promoting safer conception among FSW living with HIV.

### PEMOD64 Project Samajh – Leveraging Social‐Media for promoting COVID safety behaviors, safer sex practices, and ART adherence among the LGBTQ+ community during Covid‐19 pandemic in India: lessons learned

A. Dange ^1^, S. Abbasi^1^, Y. Singh^2^, S. Kumar^1^, R. Pujari^1^, S. Rawat^1^, M. Sivasubramanian^1^, V. Anand^1^

^1^The Humsafar Trust, Mumbai, India, ^2^The Humsafar Trust, Delhi, India

**Background**: In India, Covid‐19 pandemic messaging/communication was linear thereby creating a severe knowledge gap among LGBTQ+ on transmission, prevention and HIV‐related safety behaviors/measures. The Humsafar Trust (HST) envisaged, designed and implemented an LGBTQ+ community‐led social media campaign to bridge this gap. Project *Samajh* (Understanding) was funded by UNAIDS India country office.

**Description**: Three‐pronged communication strategy was implemented for LGBTQ+ communities awareness, improved services access and linkage to care. Communication content was designed by community members and focused on COVID‐appropriate behaviors while addressing misconceptions, safer sex, STI protection, HIV/ART treatment, mental health, and information on NACO‐govt and other helplines. Overall, 46 posters, 7 videos, 18 infographics and 24 quizzes were promoted on HST's social media pages; organisational Facebook groups, and HST's Youtube, Instagram, Twitter, and Linkedin channels. Whatsapp groups helped promote content amongst `hard ‐to‐reach LGBTQ+’. Influencers/micro‐influencers were engaged to outreach specific local LGBTQ+ population. Better‐performing posts were boosted for wider reach. In four months, *Samajh* reached 4 million persons across India. Largest audience reach 78% was achieved from paid promotions; 18% reach was achieved by eleven community influencers who also attracted 9% engagement. HST's social media handles achieved 6% engagement rate.

**Lessons learned**: Community inputs in designing content is critical for presenting COVID‐19 information for LGBTQ+ communities. Content must be holistic, engaging and address issues such as mental health, partner safety, sexual health along with COVID‐19 information. Content needs to be mobile‐screen oriented as maximum consumption is on mobile phone. Age 18–24 years emerge as focus audience on social media. Existing support services such as mental health and NACO helplines can be leveraged for further support. Social media paid promotion is able to reach a diverse set of audience based on campaign objective at a low cost however, LGBTQ+ community influencers attract higher engagement rate.

**Conclusions/Next steps**: Social media‐based health communication is the new frontier for community based organizations to reach young/hard‐to‐reach populations. Effective virtual promotion strategies can be low cost and effective in behaviour change messaging. Project Samajh experiences will be leveraged in Global Fund‐supported NETREACH and other virtual outreach projects targeting virtual populations vulnerable to HIV/AIDS.

### PEMOD65 South African adolescents living with HIV talk priorities! Longitudinal analysis of priorities of adolescent advisors living with HIV prior‐to and during the COVID‐19 pandemic

L. Gittings^1,2^, Y. Price^1^, A. Thomas^1^, N. Kannemeyer^1^, J. Kelly ^1^, N. Ralayo^1^, L. Cluver^3^, C. Logie^2^, E. Toska^1^

^1^University of Cape Town, Centre for Social Science Research, Cape Town, South Africa, ^2^University of Toronto, Factor‐Inwentash Faculty of Social Work, Toronto, Canada, ^3^University of Oxford, Social Policy and Intervention, Oxford, United Kingdom

**Background**: Growing evidence documents the indirect effects of the COVID‐19 pandemic on youth. We explore and compare the priorities of youth living with HIV in the Eastern Cape province of South Africa prior to, and during the COVID‐19 pandemic using longitudinal qualitative and participatory data.

**Methods**: Findings were co‐generated with adolescent advisors in the Eastern Cape Province of South Africa (n = 19, ages 16–21), recruited from studies of adolescents living with HIV, and young parents. Advisors engaged in an exercise to generate youth health and development‐related priorities prior to COVID‐19 in 2019 and 2020. During the COVID‐19 pandemic in 2020/2021, they shared their experiences, challenges and coping strategies in semi‐structured telephone interviews (n = 14) and group‐based social media activities (n = 27). We thematically analysed COVID‐19 data, then compared themes with pre‐pandemic priority lists.

**Results**: The most common priorities pre‐COVID‐19 were substance abuse, unemployment and career, health and medication, pregnancy, peer pressure and bullying and blessers (age disparate transactional sexual partners). These pre‐COVID‐19 priorities, apart from blessers, presented as strong themes in the COVID‐19 data. Additional COVID‐19‐related priorities that were not in the pre‐pandemic exercise included: mental health and emotional well‐being, describing feeling depressed and anxious over uncertain futures and new concerns over crime and policing.

**Conclusions**: While many topics of concern to adolescents living with HIV remain unchanged before and during COVID‐19, there were also notable differences. New COVID‐19 reported challenges included crime and policing as well as mental health and emotional challenges. This data dovetails with a growing literature on mental health concerns exacerbated COVID‐19 and warrants further consideration ofyouth mental health needs brought about by COVID‐19. While this data demonstrates how COVID‐19 may have exacerbated pre‐existing issues, analysis also suggest that COVID‐19 may have brought about a new paradigm for adolescents to make sense of, and articulate their challenges.

**Figure:**
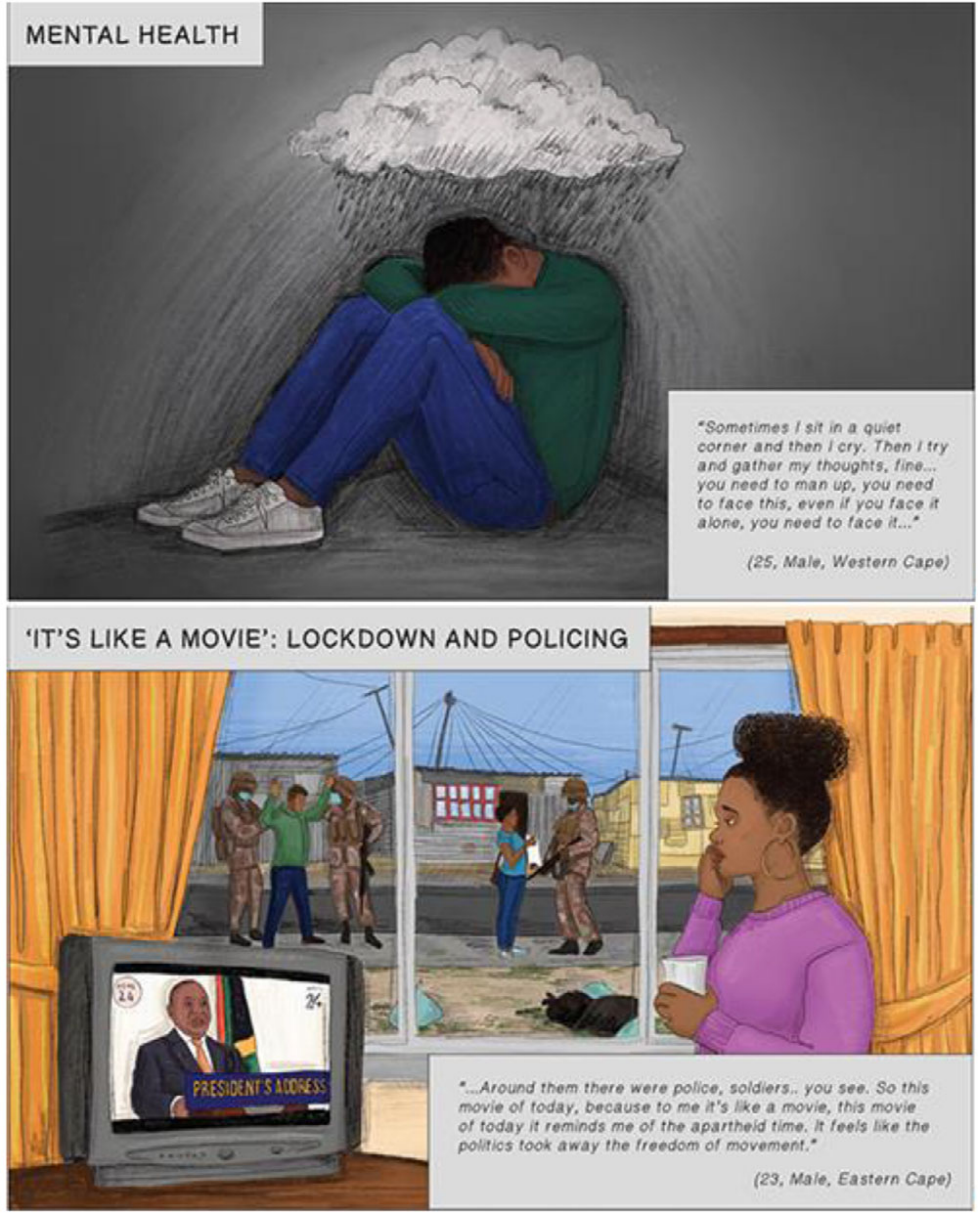
**Abstract PEMOD65‐Figure 1**.

### PEMOD66 Impact of coronavirus disease 2019 social distancing and curfews on the people of western Uganda: implications for access to HIV care

Z. Naiga ^1^, R. Najjeke^2^

^1^Kyambogo University, Social Sciences, Kampala, Uganda, ^2^Kyambogo University, Information Technology, Kampala, Uganda

**Background**: The world has been hit by the covid‐19 pandemic which led to the loss of several lives across the globe. Uganda following the WHO recommendations imposed social distancing and curfew restrictions on its population as measures to combat the rapid spread of covid‐19. Little or no studies have been carried out on the impact of these restrictions on HIV care by people of western Uganda.

**Methods**: The study was conducted between March to December 2021 at the health care centers in western Uganda. A cross‐sectional study design was used by interviewing the people with HIV (n = 672) attending health care centers in the region using. XLSTAT, statistical software was used to obtain the descriptive data. Significant differences between means were determined using the T‐test and Analysis of variance.

**Results**: The study revealed that 43% of people with HIV aged (18–30) years reported being beaten by security persons denying them access to health care centres for medication. The majority (65%) of the female people with HIV reported having run out of their ARV drugs as they could not walk long distances to access the health care centres due to halting of transport means. A good number (95%) of the male persons with HIV that got to the health care centres were not attended to. The health care practitioners were reported to have attended much more on covid‐19 cases than the HIV related cases. The people ageing with HIV (above 50years) reported having contracted co‐morbidities resulting from reduced HIV care access. Significant differences were observed in access to HIV care before and during the imposing of the restrictions.

**Conclusions**: The covid‐19 social distancing and curfew restrictions denied the people with HIV access to health care centers which put their lives at stake. The government under the ministry of health should use this study data to establish HIV care centers at the village level. This will allow the People with HIV in western Uganda to easily access these facilities even in times of crisis.

### PEMOD67 Female sex workers (FSW) and police violence during the Covid‐19 health crisis in 2020–21: Results from the EPIC multi‐country community‐based research program in Argentina

I. Aristegui ^1,2^, J. Castro Avila^3^, V. Villes^3^, G. Orellano^4^, M. Aguilera^5^, M. Romero^5,6^, L. Riegel^3^, L. Kretzer^3^, N. Cardozo^1,5,6^, P. Radusky^1,7^, D. Rojas Castro^3,8^, EPIC Study Group

^1^Fundacion Huesped, Research Department, Buenos Aires, Argentina, ^2^Universidad de Palermo, Buenos Aires, Argentina, ^3^Coalition PLUS, Community‐based Research Laboratory, Pantin, France, ^4^Asociación de Mujeres Meretrices de Argentina (AMMAR), Buenos Aires, Argentina, ^5^Asociación de Travestis, Transexuales y Transgenero de Argentina (ATTTA), Buenos Aires, Argentina, ^6^RedLacTrans, Buenos Aires, Argentina, ^7^Univesidad de Buenos Aires, Buenos Aires, Argentina, ^8^Aix Marseille Université, Sciences Economiques & Sociales de la Santé & Traitement de l'Information Médicale, Marseille, France

**Background**: Female sex workers (FSW) have been disproportionately impacted by the Covid‐19 crisis. Data shows increases of police violence towards key populations (KP), a consequence of their greater attributions to enforce health government measures. This study aimed to identify factors associated with police violence experienced among FSW during the Covid‐19 crisis in Argentina.

**Methods**: EPIC is a multi‐country, cross‐sectional, community‐based research program evaluating the impact of Covid‐19 among KP. In Argentina, the study was conducted by community‐based organizations (CBO) working with and for FSW. 173 FSW completed an online survey (October 2020‐April 2021). Police violence was measured as having experienced episodes of at least one type of violence (physical, verbal, psychological or sexual) by security forces since the start of the health crisis. Factors associated with police violence were assessed in logistic regression models.

**Results**: Among respondents (N = 173, median age 34[IQR 27–42]), 39.3% were transgender women (TW), 78.1% declared that sex work was their only income source and 71.7% declared that their financial situation has deteriorated considerably with the health crisis. 44.5% of FSW reported experiencing police violence. Furthermore, 59 (76.6%) of them experienced more frequent violent episodes. After adjustment for age, being a TW (aOR[95%CI] = 4.00[1.69;9.45], deterioration of quality of life (4.59[1.28;16.51]), fear of being harassed/arrested by the police (6.39[2.12;19.68]) and having received medical assistance from a CBO (5.01[1.98;12.68) were independently associated with police violence.

**Conclusions**: FSW in Argentina have experienced an increase in police violence since the beginning of the health crisis. Belonging to multiple KP (FSW and TW) increases the likelihood of experiencing police violence, highlighting the need of an intersectional approach to develop interventions to reduce stigma and violence against FSW. Police violence is related to fear of the police, possibly impeding street‐based sex work, and may contribute to a decline in the financial situation and the quality of life of FSW. CBOs have responded to the needs of FSW by offering medical assistance, among other services, to support KP.

### PEMOD68 Who cares if you're poz right now? Learning and adapting risk and responsibility from HIV to COVID‐19 among barebackers

J. Garcia‐Iglesias ^1^, C. Ledin^1^

^1^University of Edinburgh, Usher Institute, Centre for Biomedicine, Self and Society, Edinburgh, United Kingdom

**Background**: COVID‐19 has had major impacts on sexuality and sexual practices. Barebackers, gay men who engage in condomless anal intercourse, have adapted their experiences and risk management practices from HIV to navigate the new risks of COVID‐19 and balance risk and desire.

**Methods**: We conducted an online ethnography of the most popular barebacking online forum in English during July 2021, retrieving 112 conversations comparing HIV and COVID‐19, exploring how to navigate risk and retain pleasure. These were thematically analyzed.

**Results**: In our findings, we found significant differences between those users who had first‐hand experience of living through the AIDS crisis, who were seen as ‘experts’ or as having a ‘badge of honor’, and younger forum members. Overall, barebackers compared the AIDS crisis (not the current HIV‐as‐treatable‐moment) with COVID‐19 in several ways. First, they repurposed individual risk reduction practices (e.g. reducing the number of partners, limiting casual sex) as a way of preventing the spread of the virus both among barebackers and to society at large. Second, they emphasised the role of ‘individual responsibility’ for preventing the spread of COVID‐19 and chastised those they saw as ‘reckless’ (e.g. those engaging in casual sex while infected with COVID‐19), repurposing serophobic language (e.g. ‘slut‐shaming’). However, third, they also strongly emphasised their desire to retain pleasurable barebacking practices, such as group sex in clubs, and suggested that temporary risk reduction would make these possible in a post‐COVID‐19 future.

**Conclusions**: This is, to our knowledge, the first study of how barebackers have adapted HIV learnings to COVID‐19. Barebackers are a community historically disproportionally affected by HIV and culturally seen as ‘risk prone’ or ‘hedonistic’. This paper evidences not only that barebackers have adopted narratives of individual responsibility deeply influenced by the history of the AIDS crisis, but that they have done so to preserve their future ‘pleasurable practices’, such as group sex, after COVID‐19. Thus, this paper reveals how one key community disproportionaely affected by HIV have adapted to COVID‐19 and imagined their future after it.

### PEMOD69 Understanding the lived experiences of people at the intersection of HIV and COVID‐19

A. Hundal ^1^, D.L. Bose^1^, S. Rawat^2^, D. Baruah^2^, S. ul Hadi^1^, K. Seth^1^, P. Saha^1^, J. Mukherjee^1^

^1^IAVI, New Delhi, India, ^2^The Humsafar Trust, Mumbai, India

**Background**: COVID‐19 has been particularly challenging for marginalized communities affected by HIV. For many, this meant reliving experiences of fear, confusion, stigma and economic disenfranchisement. To understand lived realities of those at the intersection of COVID‐19 and HIV, IAVI, with Humsafar Trust, conducted a series of Community‐Researcher Dialogues in India.

**Description**: Between November 2020 and June 2021, three virtual dialogues were conducted with 60 participants comprised of community members from FSW, PWID, MSM, AGYW and TG populations, CBOs, activists, scientists and researchers to initiate conversations and identify pressing programmatic and research needs. Conversations were audio recorded with due participant consent and accompanied by detailed notes. This data was then coded using an inductive framework of analytic domains based on key areas of enquiry.

**Lessons learned**: These dialogues highlighted the following:

**Poor testing and increased HIV risk‐taking behaviours ‐** Disruptions in services, fear of contracting COVID‐19 at health centers, lockdown‐related interruptions in transportation and mass migration led to poor testing and adherence. Food insecurity, fear of stigma and discrimination, lack of family/peer support and isolation, loss of employment, lack of access to care added to pandemic stress, pushing some to opt for unsafe sex for economic sustenance or due to limited access to safer sex options.

**Mental health crises** emerged as major concerns along with increased incidences of violence, especially among sexual minorities.

**Existing networks/collectives of HIV‐affected communities** were instrumental in facilitating/creating innovative prevention/treatment service/delivery models, working with local/government health agencies to initiate strategies like multi‐month dispensing and ART home delivery. In HIV knowledgeable communities, where targeted interventions have been sustained for years, adoption of COVID‐19 appropriate behaviours and sharing of information among peers was easier.

**Digital media** also played a critical role in facilitating referral‐, emergency care‐, and peer support in real‐time.

**Conclusions/Next steps**: The COVID‐19 crisis has highlighted the need to rethink approaches to shared global health practices by putting communities at the centre. The role of HIV knowledgeable communities, along with the vitality of newly created networks during the pandemic, necessitate sharpened insights into how these networks were leveraged to support service uptake, and how they can be further bolstered for future pandemic preparedness.

### PEMOD70 ‘Of the youth, by the youth, for the youth’: a peer‐led model for building resilience among youth living with HIV during the COVID‐19 pandemic

M. Babu Raj ^1^, L. Reddy^1^, F.T. Thomas^1^, A. S^1^, K. Tippanna Chitti^1^, K. Lakshmikanth^1^, B. Seenappa^1^, G. Nethra Raju^1^, K. Mehta^2^, A. Shet^2^

^1^Sneha Charitable Trust, Snehagram, Bangalore, India, ^2^Johns Hopkins University, Johns Hopkins Maternal and Child Health India Center, Baltimore, United States

**Background**: HIV care access and delivery was adversely impacted due to the COVID‐19 pandemic. Youth living with HIV were particularly affected and were vulnerable to reversal of health gains. In response to this need, Sneha Charitable Trust (SCT) mobilized youth peer leaders to build a model of continued healthcare access and support for those affected during the COVID‐19 pandemic in southern India.

**Description**: The peer‐led model consisted of ten HIV‐postive youth leaders, who have have demonstrated leadership skills during crises. Between January 2020‐September 2021, 500 adolescents and youth aged 12–25yrs with poor healthcare acess and social support were selected as beneficiaries. During this period, the peer leaders in a 1:50 ratio, reached out to beneficiaries and built an effective program focused on addressing misinformation related to COVID‐19, strengthening COVID‐19‐appropriate behaviours, promoted continued support ofone another, ensuring access to ART and nutrition, and support for building healthy lives during pandemic‐related adversity. Contact was maintained weekly, and additional nutrition and counselling support for affected families were provided by peer leaders.

**Lessons learned**: COVID‐19 lockdowns impeded ART access among 83.0% of children and youth. Through coordination with the State AIDS Control Society, the program facilitated a 3‐month supply of ART through home delivery, which helped to restore ART acesss to 93.0%. During the intervention period, 84.0% remained healthy, 5.8% developed tuberculosis, and 3.2% required hospital admission for opportunistic infections. COVID‐19 infections were present in 8.0% adolescent’ households. Nutritional support was provided to 60.0% of the households for over 6 months. The peer leaders were able to build strong partnerships with clinical service teams, and facilitated the beneficiaries’ healthcare access. Informal surveys administered with the beneficiaries highlighted that the support extend by the peers helped in positive adaptation to adversity, recovery from illness and adaptation and resilience amidst crises.

**Conclusions/Next steps**: Our unique peer‐led model of care provision and confidence‐building activities during the COVID‐19 restrictions led to HIV‐positive youth handling pandemic adversity with resilience. This model demonstrates that amidst unprecedented crises, peer‐led mutual support and partnerships at the community and systems level are critical to ensure ongoing success.

### PEMOD71 How the Covid Lockdown and Government Measures affected Gay Men and Transwomen's Health Care, Sexuality, and Life in Peru: A qualitative study from the EPIC multinational community research program

C. Sandoval Figueroa ^1^, M. Reyes Díaz^1^, J.C. Enciso Durand^1^, O. Apffel Font^2,3^, N. Lorente^2,4,5^, J. Castro Avila^2^, L. Riegel^2^, M. Di Caccio^2,6^, D. Rojas Castro^2,7^, C.F. Cáceres^1^, EPIC study group

^1^Universidad Peruana Cayetano Heredia, Centro de Investigación Interdisciplinaria en Sexualidad, SIDA y Sociedad, Lima, Peru, ^2^Community‐based Research Laboratory, Coalition PLUS, Pantin, France, ^3^Coalition des Organismes Communautaires Québécois de Lutte Contre le Sida (COCQ‐SIDA), Montréal, Canada, ^4^Centre d'Estudis Epidemiològics sobre les Infeccions de Transmissió Sexual i Sida de Catalunya (CEEISCAT), Departament de Salut, Generalitat de Catalunya, Badalona, Spain, ^5^• Centro de Investigación Biomédica en Red de Epidemiología y Salud Pública (CIBERESP), Madrid, Spain, ^6^Groupe de Recherche en Psychologie Sociale (GRePS), Université Lyon 2, Lyon, France, ^7^Aix Marseille Univ, Inserm, IRD, SESSTIM, Sciences Economiques & Sociales de la Santé & Traitement de l'Information Médicale, ISSPAM, Marseille, France

**Background**: EPIC‐international community‐based program is coordinated by Coalition PLUS to collect comparable multinational data regarding the impact of the COVID‐19 crisis in key populations (KP) concerning HIV.

**Methods**: As part of the EPIC‐international community‐based program by Coalition PLUS, we designed a qualitative study to gather experiences from Peruvian MSM and transwomen (TW) about the Covid lockdown and how they dealt with it regarding health/medical care, education, and work. From September/December 2021, 16 in‐depth‐interviews with TW and MSM, who identified as gay men, focused on how lockdown and government measures affected their health/medical/care, work, and education.

**Results**: Gay Men (GM) and TW associated Covid‐19 with illness/death/stress. All had friends/relatives who had been severely ill or had died from Covid‐19. Many experienced stress after being forced to reorganize their lives and budget, as their education/work routines had changed.

Normally excluded from stable, formal jobs, TW lost their livelihoods as hairdressers, peddlers, or sex workers, facing serious economic difficulties during the lockdown. The government formally includes KPs among vulnerable populations, but did not include TW as recipients of COVID‐19 relief packages (food/money) due to technicalities, despite their clear financial vulnerability.

Regarding sexuality, GM/TW dropped out of their networks, but quickly returned to them when measures relaxed. However, sexual health services were suspended from the start of the pandemic, affecting condom and comprehensive care provision. Fortunately GM/TW with HIV picked up their treatment at the health services every three months.

Inconvenient for the general public, public movement restrictions by gender were harmful for TW, who were arrested by the police or ridiculed by the media when going out on women's days. Although this measure only lasted one week, it remained etched on the memory of those interviewed as an example of structural discrimination suffered by TWs.

**Conclusions**: During lockdown, TW were greatly affected, due to their social and financial vulnerability. They are not receiving government COVID‐19 relief packages signals their social and structural exclusion. Public policies for vulnerable populations in Peru should take GM and TW into account. A strategic plan in sexual health services is needed for sizeable emergencies, so as not to leave aside vulnerable populations.

### PEMOD72 Economic, social, and behavioural impacts of the COVID‐19 pandemic on women and girls living with or at high risk of HIV in Nigeria

A. Yakusik ^1^, E. Lamontagne^1,2^, K. Sabin^1^, O. Arije^3^, A. Enemo^4^, A. Sunday^5^, A. Muhammad^6^, H. Yanusa Nyako^7^, R.M. Abdullahi^8^, H. Okiwu^9^, M. Oluwatoyin Folayan^3,10^

^1^UNAIDS, Strategic Information, Geneva, Switzerland, ^2^Aix‐Marseille University, School of Economics, Marseille, France, ^3^Obafemi Awolowo University, College of Health Sciences, Ile‐Ife, Nigeria, ^4^Nigeria Sex Workers Association, Kubwa, Nigeria, ^5^African Network of Adolescent and Young Persons Development, Barnawa, Nigeria, ^6^Northern Nigerian Transgender Initiative, Abuja, Nigeria, ^7^Jami Al Hakeem Foundation, Jimeta‐Yola, Nigeria, ^8^National Association of Persons with Physical Disability, Abuja, Nigeria, ^9^YouthRISE, Abuja, Nigeria, ^10^Nigeria Institute of Medical Research, Lagos, Nigeria

**Background**: Little is known about how the COVID‐19 pandemic affected women and girls living with HIV (WLHIV) or at high risk of HIV, who experience disproportionate levels of comorbidity and stigma. We describe the economic, social, and behavioural impacts of the pandemic on WLHIV or at high risk of HIV in Nigeria.

**Methods**: A cross‐sectional survey was conducted between July and December 2021, collaboratively with community‐based organizations to investigate the impact of the pandemic on WLHIV or at high risk of HIV, including those who sell sex, live with disabilities, use drugs, migrants and displaced people, and transgender women. Participants 15 years and older were recruited voluntarily, both online and face‐to‐face, using a combination of venue‐based and snowball convenience sampling in ten states covering six geopolitical zones.

**Results**: There were 4541 respondents; 50% were between 19 and 30 years old, 47% reported living with HIV. 61% (2676/4411) reported a negative impact on their income, and 76% (3342/4409) had to cut back on food. About 10% (468/4541) received assistance or cash relief. Some 25% (722/2835) started selling sex to meet their financial obligations. WLHIV or at high risk of HIV said they experienced issues accessing health services for HIV, 55% (1988/3581), sexually transmitted infections, 36% (1104/3073), tuberculosis, 22% (609/2773), family planning, 19% (510/2702), and safe abortion care, 13% (335/2570). The biggest obstacles were financial: 54% affording transport, 39% medicines or tests, and 28% user fees at a health‐care facility (Figure 1). Social and structural barriers were also reported (Figure 1).

 

**Figure:**
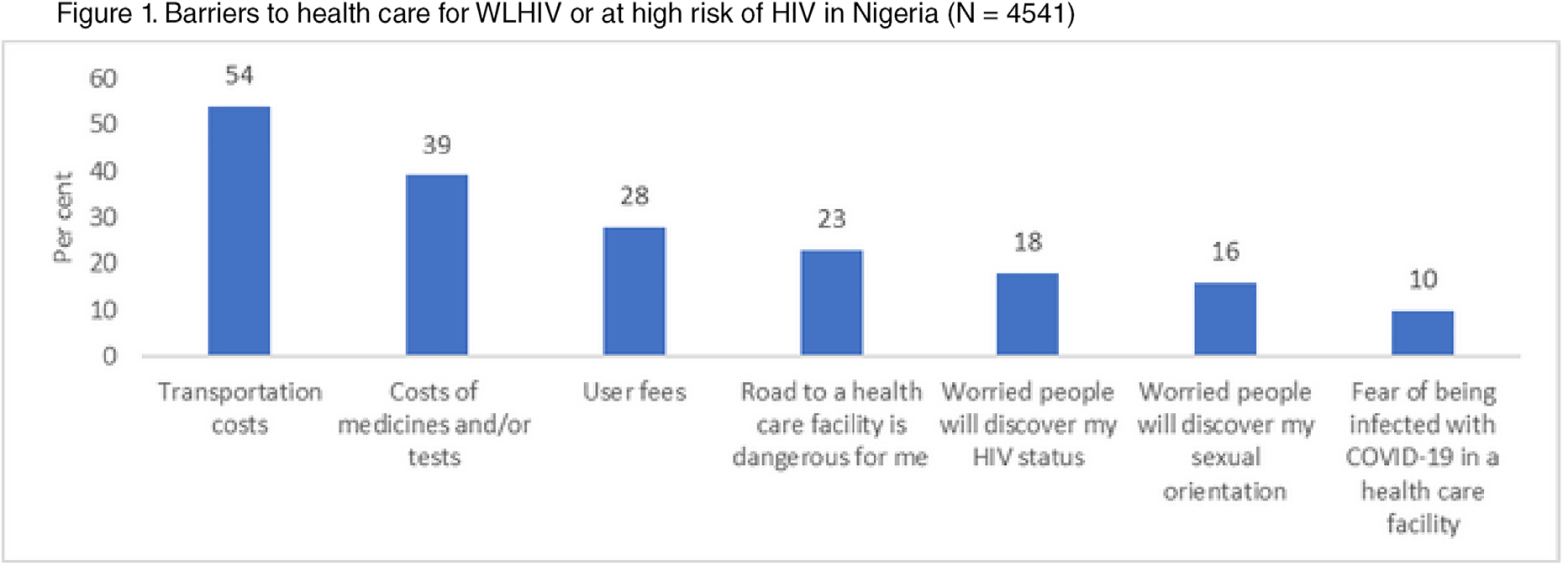
**Abstract PEMOD72‐Figure 1**.

**Conclusions**: Our study demonstrated the significant negative effects of the COVID‐19 pandemic on WLHIV or at high risk of HIV in Nigeria. Interventions are necessary to mitigate socio‐economic challenges, address structural inequality, and ensure access to health services.

### PEMOD73 Covid‐19 and self‐perceived changes in psychosocial and behavioral outcomes of people living with HIV in community‐based clinics of Mali: The EPIC programme of Coalition PLUS

L. Sagaon‐Teyssier ^1,2^, D. Dondbzanga^3,2^, M. Guindo^2^, D. Traoré^2^, A. Kamissoko^2^, M. Cissé^2^, B. Dembélé Keïta^2^, L. Riegel^4^, T. Cerveau^3^, R. Delabre^4^, D. Rojas Castro^1,4^, The EPIC working group

^1^Aix Marseille Univ, INSERM, IRD, SESSTIM, Economic & Social Sciences of Health & Medical Information Processing, ISSPAM, SanteRCom, Marseille, France, ^2^ARCAD Santé PLUS, Centre Intégré de Recherche, de Soins et d'Action Communautaire (CIRSAC), Bamako, Mali, ^3^Community Research Laboratory, Coalition PLUS, Dakar, Senegal, ^4^Community Research Laboratory, Coalition PLUS, Pantin, France

**Background**: In Mali, around 23% of the 57000 people living with HIV (PLHIV) on antiretroviral treatment (ART) are followed‐up in community‐based clinics. Their functioning was reorganized to guarantee the HIV‐care continuum during Covid‐19. We investigated changes in PLHIV's psychosocial and behavioral outcomes, associated factors to these changes, and their interrelationship.

**Methods**: The EPIC multi‐country community‐based research coordinated by Coalition PLUS was conducted in 33 countries including Mali, studying the impact of Covid‐19 among PLHIV and key populations. Analyses used data collected in March 2021 among PLHIV of 18 community‐based clinics of ARCAD Santé PLUS. Face‐to‐face questionnaires collected participants’ characteristics, and assessed perceived changes in psychosocial/behavioral and HIV‐care related aspects compared before Covid‐19. Outcomes: perceived changes in day‐to‐day life (DDL) (negative = 1/unchanged = 0), quality‐of‐life (QoL) (worse = 1/unchanged = 0), and ART‐adherence (worse = 1/unchanged = 0). Three‐equation multivariate probit model was estimated to investigate associated factors and the link between outcomes.

**Results**: Among 695 participants, 72.3% were female and median age was 38 years IQR[31–45]. Negative changes in DDL were declared by 74.5%; 27.2% and 40% declared poorer QoL and ART‐adherence, respectively. Estimations showed deteriorated financial situation associated with worse DDL (coeff: 0.805/p < 0.001) and QoL (coeff: 0.278/p = 0.036). Negative changes in DDL were less likely for older (coeff: −0.276/p = 0.030) and those in rural areas (coeff: −0.507, p < 0.013), although more likely for those with difficulties finding social support (coeff: 0.640/p = 0.003). Female (coeff: 0.263/p = 0.040), older (coeff: 0.468/p < 0.001) and those in urban areas (coeff: 0.568/p = 0.002) were associated with poorer QoL. Long‐term ART delivery reduced QoL decline (coeff:‐0.346/p = 0.008). Accommodation difficulties (coeff: 0.503/p = 0.017), and perceiving that community‐based response to Covid‐19 was inadequate to PLHIV (coeff: 0.331/p = 0.002) were associated to negative changes in ART‐adherence. Easy healthcare access (coeff: −0.653/p = 0.010) and long‐term ART delivery (coeff: −0.243/p = 0.048) reduced ART‐adherence issues. Finally, ART‐adherence and QoL changes are strongly related (correlation: 0.308/p < 0.001), but not ART‐adherence with DDL (correlation: 0.127/p = 0.063). However, DDL and QoL are processes that evolve together (correlation: 0.184/p = 0.011).

**Conclusions**: QoL seems to be a key aspect in the management of negative changes in ART‐adherence and DDL among Malian PLHIV, although the mechanism is different. Community‐based related factors to QoL should contribute to the improvement of ART‐adherence, while focusing on demographic/socioeconomic factors should contribute to the attenuation of the Covid‐19 impact over DDL through the improvement of QoL.

### PEMOD74 Impact of COVID‐19 on access to sexually transmitted and blood‐borne infections (STBBI) and harm reduction services for people who use drugs or alcohol in Canada

J. Cox ^1^, J. Zhang^1^, M. Wong^1^, D. Shublaq^1^, K. Zhang^1^, L. Jonah^1^, J. Tarasuk^1^, J. Sorge^1^, E. Wong^1^, M. Bryson^1^, D. Paquette^1^

^1^Public Health Agency of Canada, Centre for Disease and Infection Control, Ottawa, Canada

**Background**: The COVID‐19 pandemic has disproportionately affected people who use drugs or alcohol (PWUD). This population faces health inequities including increased risk of STBBI and severe COVID‐19 illness. Using national survey data, we describe changes in social determinants of health (SDOH) and access to STBBI‐related health services among PWUD.

**Figure:**
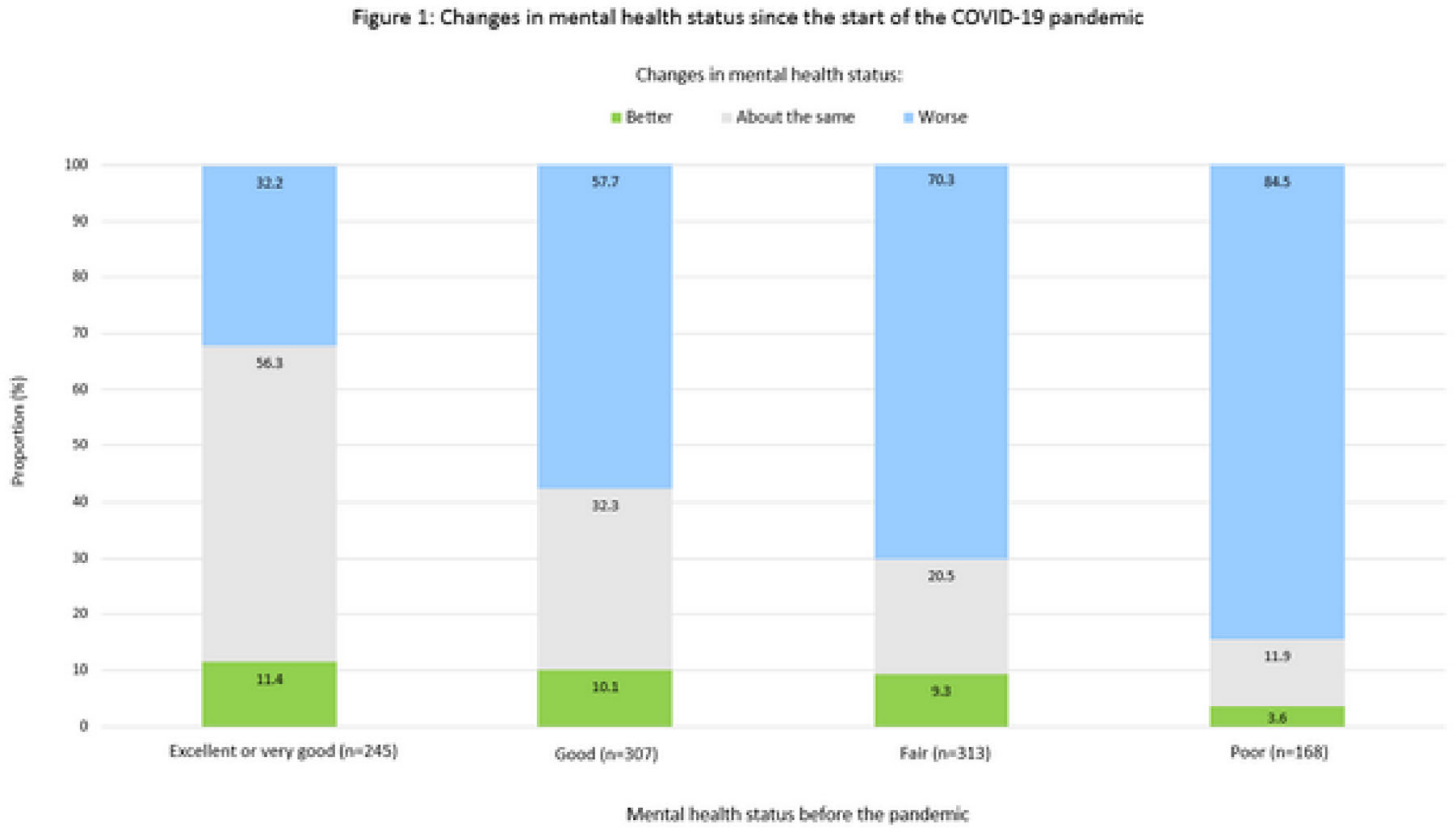
**Abstract PEMOD74‐Figure 1**.

**Methods**: From January to February 2021, an anonymous, self‐administered, online survey was conducted among anyone living in Canada aged 18+ years who identified as using substances, including alcohol or cannabis, in the past 6 months. Information collected included social determinants of health, substance use, and access to STBBI‐related services, including harm reduction.

**Results**: 1034 individuals participated (61.2% cisgender female, 32.6% cisgender male; mean age 40.5, range 18–84 years). Increased use in cannabis (64.9%), alcohol (56.7%), and opioids (56.9%) was reported. Those who experienced discrimination pre‐pandemic reported the highest increase in discrimination (59.7%) compared to those who never experienced it (4.4%). Worsening mental health since the beginning of the pandemic was noted, particularly among participants reporting fair‐to‐poor mental health prior to the pandemic (Figure 1). Those who felt the least safe pre‐pandemic reported the largest increase in feeling less safe (50.0%) since the pandemic‐start, compared to those who felt somewhat (35.6%) or very safe (19.3%) pre‐pandemic. Participants reported pandemic‐related difficulties accessing HIV, STI, or hepatitis C testing (58.8%) and harm reduction services (i.e. needle/syringe distribution programs, drug checking services, or drug consumption rooms) (80.6%). Main barriers included: public health measures; reduced clinic hours; getting a referral or appointment; and concerns about stigma, discrimination, or violence.

**Conclusions**: COVID‐19 has exacerbated existing SDOH inequities among PWUD in Canada, and introduced challenges accessing STBBI and harm reduction services. Worsening mental health and increased substance use signal the need for improved and innovative support measures (e.g., mobile outreach) to overcome barriers from the pandemic for PWUD.

### PEMOD75 PrEParados: A Spatially Explicit Network Visualization Framework to Identify Latino MSM who Qualify for PrEP Use

M. Kanamori ^1^, C.H. Shrader^2^, A. Johnson^1^, J. Arroyo‐Flores^1^, J. Skvoretz^3^, E. Rodriguez^1^, S. Fallon^4^, J. Stoler^5^, S. Doblecki‐Lewis^1^, A. Carrico^1^, S. Safren^5^

^1^University of Miami Miller School of Medicine, Public Health Sciences, Miami, United States, ^2^Columbia University, New York, United States, ^3^University of Central Florida, Tampa, United States, ^4^Latinos Salud, Miami Beach, United States, ^5^University of Miami, Miami, United States

**Background**: Frameworks are needed to increase PrEP initiation among Latino men who have sex with men (LMSM) in Miami Florida, a US epicenter of the HIV epidemic. Our PrEParados Network Framework identifies LMSM who qualify for PrEP through friendship, sexual, and spatially‐explicit networks.

**Description**: This framework incorporates sociocentric friendship, egocentric sexual and geospatial networks. Findings are based on cross‐sectional data collected from October 2018 to August 2019 (N = 130 PrEP eligible LMSM and their 507 sexual partners). Participants were predominantly White, employed full‐time, single, mean age of 28 years; and half were foreign‐born.

**Figure:**
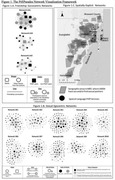
**Abstract PEMOD75‐Figure 1**.

**Lessons learned**: Friendship network structures identified correlates of PrEP communication, which included PrEP use within the network. LMSM may feel pressure to use PrEP, after hearing about or observing PrEP use through mutual acquaintances. The framework identified the characteristics of popularLMSM and their intention to disseminate PrEP messages and convince friends to use PrEP. The PrEParados Framework also identified unconnected LMSM and opportunities to connect them through LMSM “bridges” to other members who use PrEP or encouraged their friends to use PrEP. The majority of participants did not disclose their PrEP use status with sexual partners. We identified an increase in receptive condomless anal sex among sexual partners who reported at least one partner was on PrEP. The spatial network found the absence ofSpanish language PrEP services in areas where LMSM find sexual partners and/or have sex, and priority locations for advertisements about HIV services.

**Conclusions/Next steps**: The PrEParados Network Framework incorporated sociocentric, egocentric, and geospatial networks to identify LMSM who qualify for PrEP use. This model can: a) identify, reach and engage key populations who qualify for PrEP but are not using PrEP, b) increase diffusion of accurate PrEP information and guidance to key populations, and c) identify policies to increase access to PrEP services.

### PEMOE32 Women's legal and economic empowerment project to improve HIV care and treatment outcomes: Lessons from cross‐border trading initiative in Northern Rwanda and Democratic Republic of Congo

H.N. Gerard Audace ^1^

^1^Universite Laic Adventiste, Economics, Kigali, Rwanda

**Background**: Despite remarkable investments in health and social education, gender inequity and poverty remain the major predictors of health outcomes among HIV patients in rural and resource‐constrained settings. To improve women's health and HIV outcomes among women from villages surrounding Rwanda‐Congo border, LegEcoAccelerator, an innovative program combining legal and economic support were implemented. Family planning and child care services were provided. We present results following five years of active implementation period. A mixed method evaluation was conducted to assess changes in household income, clinical outcomes before and after 60 months of active implementation. A qualitative assessment captured perceptions and acceptability of the program.

**Description**: Following poor outcomes among women crossing the Rwanda‐RDC border for micro‐businesses, we have deployed a micro‐grant and saving program to empower women who had very little or no investment capital. We also launched a one‐stop center for HIV care and treatment, facility planning and day care services were provided. This integrated and innovative approach was named LegEcoAccelerator program.

**Lessons learned**: A legal support established a joint agreement between local leadership across two borders and improved women's freedom and social economic development. Of 7623 women enrolled in the program at baseline 72% and 28% were extremely poor and very poor, respectively. A total 15,869 women participated in our LegEcoAccelerator program between January 2017‐December 2021. The percent of women living in extreme poverty reduced significantly from 72% to 15%, p < 0.001. The percent of women who shifted from extreme poverty and poverty to any other upper wealth categories from 20% to 81%, p < 0.001.Over 70 women received childcare and nutrition support. Adherence to ARV increased from 75% to 85%, *p* = *0.06*. The percent of women with suppressed viral load improved by 50%. Domestic violence incidents which declined by 80%.

**Conclusions/Next steps**: Combining legal and social economic support improve the effectiveness of HIV care and treatment. This suggest that combining a legal and economic empowerment could be a strategy to improve health outcome and accelerate the pace toward ending HIV endemic. Although cross‐border programs bring about better impact, there is a need for shared accountability and responsibility among neighboring countries.

### PEMOE33 A Community‐centric Study of Male Clients of Female Sex Workers using Respondent Driven Sampling

J. Coetzee ^1^, M. Milovanovic^1^, K. Hlongwe^1^, K. Otwombe^1^, V. Mbowane^1^, G. Gray^2^, R. Jewkes^3^

^1^Perinatal HIV Research Unit, Prevention in Key Populations, Soweto, South Africa, ^2^South African Medical Research Council, Office of the President, Capet Town, South Africa, ^3^South African Medical Research Council, Cape Town, South Africa

**Background**: In South Africa, male clients of sex workers (MCSWs) account for 40% of new HIV infections. There are currently limited understanding of the population with no interventions targeting MCSWs. Understanding their health status and risk factors for adverse health outcomes is foundational for developing evidence‐based health care for this population. Describe the methodology used to successfully implement a community‐centric respondent driven sampling (RDS) study of psycho‐social circumstances, HIV, violence perpetration and associated factors amongst MCSWs in South Africa.

**Methods**: A community‐centric, RDS, survey of 660 adult MCSWs in Soweto, South Africa was conducted (March–November 2021). Formative interviews and focus group discussions helped inform the RDS study design and inform the questionnaire development. Men from the sex work sector were involved in the study design and questionnaire development. Questions included: demographic, sexual behaviour, HIV testing and treatment history, and violence exposure and perpetration. HIV rapid testing, viral load, CD4 count, and HIV drug resistance genotypic testing were undertaken.

**Results**: 19.1% of MCSWs were HIV positive, two thirds were aged 25–34 and 35–44, the median age of purchasing sex was 21, and condoms were consistently used by 16.7%. Almost all MCSWs had experienced childhood trauma, with two thirds of men reporting some form of sexual violence in their lifetimes. Just under 30% reported perpetrating sexual violence against their intimate partner, more than half (54.1%) had raped a non‐intimate parter in the past year, and almost two thirds of men had symptoms of PTSD. Older men were more likely to be HIV positive (<0.0001). HIV positive men were twice as likely to consistently use condoms (0.0006), and to suffer from PTSD symptoms (0.004) as compared to HIV negative men.

**Conclusions**: This is the first study of MCSWs in South Africa, and globally, the first to use an RDS methodology for MCSWs. Data highlight the vulnerability of this population to HIV, violence and mental health. Based on the unique methodology and successful implementation, the outcome will inform tailored interventions. Our rate of enrolment, low rate of screening failure and low proportion of missing data showed the feasibility and importance of community‐centric research with marginalised, vulnerable populations

### PEMOE34 Case‐finding, linkage to antiretroviral treatment (ART), and continuity of care: Findings from “Siyenza” facilities in South Africa, 2019–2021

S. De Anda^1,2^, J. Cattle ^1,2^, M.E. Patton^1^, J. Olivier^1^, G. Kindra^1^, R. Taback‐Esra^1^, C. Biedron^1^, H. Paulin^1^, C. Scott^1^, E. Raizes^1^, S. Dawad^3^, J. Otchere‐Darko^4^, L. Mashudu^5^, S. Prusente^6^, N. Kamere^7^, Z. Pinini^3^, J. Blandford^1^, J.M. Grund^1^

^1^Centers for Disease Control and Prevention, Pretoria, South Africa, ^2^Public Health Institute, Oakland, United States, ^3^South African National Department of Health, Johannesburg, South Africa, ^4^Wits Reproductive Health and HIV Institute (Wits RHI), Atteridgeville, South Africa, ^5^Health Systems Trust, Durban, South Africa, ^6^TB HIV Care, Cape Town, South Africa, ^7^The Aurum Institute, Johannesburg, South Africa

**Background**: The President's Emergency Plan for AIDS Relief (PEPFAR) and the South African National Department of Health launched *Siyenza* in February 2019 to improve retention of people living with HIV (PLHIV) on antiretroviral therapy (ART). *Siyenza* used weekly program data and site visits to provide intensive technical support to 241 public facilities comprising 45% of PLHIV on ART across the Centers for Disease Control and Prevention's twelve PEPFAR‐supported districts.

**Description**: To assess resiliency throughout COVID‐19, we compared weekly numbers of persons newly diagnosed with HIV and linked to ART, PLHIV on ART at 28 days, PLHIV with missed appointments 29–89 days after appointment dates, and index contacts tested and found positive for HIV from 142 facilities with continuous *Siyenza* participation from March 30, 2019–March 27, 2020 (Y1) to March 28, 2020–March 26, 2021 (Y2). Weekly facility means were analyzed using paired t‐tests. Missed appointment data for 34 facilities in eThekwini district were excluded from the analysis due to missing data.

**Lessons learned**: In Y1 and Y2, 108,970 and 61,286 persons were newly diagnosed with HIV, respectively, and 104,537 (95.9%) and 59,817 (97.6%) were linked to ART; 85,486 and 122,261 PLHIV missed an appointment, respectively. The number of PLHIV on ART at 28 days increased by 2.3% between the end of Y1 and Y2, from 591,230 to 604,847. In Y1, 5,272/29,854 (17.7%) of index contacts tested positive for HIV; 8,134/49,429 (16.5%) tested positive in Y2. Weekly facility means significantly decreased between Y1–Y2 for: persons newly diagnosed (Y1: 767.4 [95% Confidence Interval (CI): 704.3–830.4], Y2: 431.6 [CI: 397.3–465.9]) and PLHIV linked to ART (Y1: 736.2 [CI: 675.6–796.7], Y2: 421.2 [CI: 387.3–455.2]), and increased for index contacts tested (Y1: 210.2: [CI: 179.6–240.8], Y2: 348.1 [CI: 296.2–399.9]) and found positive (Y1: 37.1 [CI: 31.7–42.5], Y2: 57.3 [CI: 46.4–68.1]). There were no significant differences in weekly means for PLHIV on ART at 28 days (Y1: 4,163.6 [CI: 3,885.9–4,441.3], Y2: 4,259.5 [CI: 3,980.4–4,538.5]) and missed appointments (Y1: 791.5 [CI: 678.8–904.2], Y2: 1,132.0 [CI: 685.5–1,578.6]).

**Conclusions/Next steps**: Despite COVID‐19 challenges that caused decreases in new diagnoses, *Siyenza's* hands‐on approach ensured maintenance of active PLHIV on ART, prevented increases in missed appointments, and increased index contacts tested.

### PEMOE35 Evaluation of Ritshidze community‐led monitoring programme in South Africa

A. Yawa^1^, N. Rambau ^2^, B. Setshogelo^2^, M. Nyathi^2^, S. Xaba^2,3^, L. Rutter^4^, B. Honermann^5^, A. Sharp^6^

^1^Treatment Action Campaign (TAC), Johannesburg, South Africa, ^2^Ritshidze, Johannesburg, South Africa, ^3^South African Network of Religious Leaders Living With or Personally Affected by HIV and AIDS (SANERELA+), Randburg, South Africa, ^4^Health Global Access Project (Health GAP), Johannesburg, South Africa, ^5^amfAR, the Foundation for AIDS Research, Washington, United States, ^6^The O'Neill Institute for National and Global Health Law, Georgetown University, Washington, United States

**Background**: Community‐led monitoring (CLM), where civil society collects data on services for people living with HIV to advocate for improved services, is an emerging and powerful approach to improving quality healthcare. In 2019, the Ritshidze CLM programme was launched in South Africa to monitor HIV, TB and other health service delivery to advocate for improved primary healthcare services for all people in the country. We compared CLM measures of service delivery after year 1 and year 2 of the programme to determine Ritshidze's impact.

**Figure:**
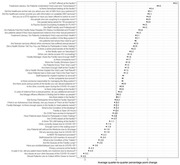
**Abstract PEMOE35‐Figure 1**.

**Methods**: Between July 2019 and September 2021, data were collected from 427 public health facilities in 30 health districts. Survey data taken from observation, patient and facility managers were gathered electronically by community monitors using the CommCare application. Priority indicators were analyzed, focusing on seven metrics: antiretroviral therapy (ART) collection and access; HIV treatment; tuberculosis; facility staff and attitudes; access to prevention, care, and support; infrastructure; and COVID‐19 disruption. Longitudinal trends were calculated by the average of the quarter‐by‐quarter percentage change in each metric between October to December 2020 and the same period in 2021.

**Results**: Between quarter 4 (July to September) 2020 and quarter 4 2021, large changes in several indicators were observed. The largest changes were in access to PrEP (9.2% average quarter‐to‐quarter increase) and GeneXpert testing (5.4%), treatment literacy on viral load (6.5%), healthcare workers asking about gender‐based violence (5.1%), and information on GBV services (5.1%) (Figure 1). In this same time period, the percent of clinics reporting any COVID‐19 disruption decreased an average of 5.2% each quarter.

**Conclusions**: Results from the Ritshidze CLM programme show early promise and suggest that CLM's can positively contribute to improving the quality, accessibility, and acceptability of HIV, TB and other health services.

### PEMOE36 Effect of district ART access on young people's wellbeing in South Africa: an econometric analysis of panel data

D. Govindasamy ^1^, T. Barnighausen^2^, M. Neuman^3^, P. Bodzo^4^, C. Mathews^5^, J. Seeley^3^, G. Ferrari^6^

^1^South African Medical Research Council, Health Systems Research Unit, Durban, South Africa, ^2^University Heidelberg, Heidelberg Institute of Global Health, Faculty of Medicine, Heidelberg, Germany, ^3^London School of Hygiene and Tropical Medicine, London, United Kingdom, ^4^University of Cape Town, Cape Town, South Africa, ^5^South African Medical Research Council, Cape Town, South Africa, ^6^London School of Economics, London, South Africa

**Background**: Expanded antiretroviral therapy (ART) is associated with substantial health and economic benefits. However, evidence on the population‐level effects of ART scale‐up on broader Sustainable Development Goals (SDGs) such as wellbeing are scarce. We aimed to study the effects of ART access on young people's in South Africa.

**Methods**: We sought to assess how temporal increases in the number on ART at a district‐level affected young people's life satisfaction, a measure of wellbeing. We used panel data from South Africa's National Income Dynamics Survey, which followed participants over 5 waves (2008–2017) and collected both individual‐ and household‐level health, social and economic data. We restricted this panel data to participants aged 15–24 years in wave 1. We then overlaid this panel dataset with district ART count and population estimates, derived from routine national HIV laboratory and census datasets, respectively. We used individual fixed effects regression models, with time and district fixed effects, to control for unobserved heterogeneity. We assessed the sensitivity of our findings using alternate regression models and a balanced sample.

**Results**: We analysed data on 5685 individuals (N = 27 739 observations), mean age 23 years (SD = 4.30), 50% female. On average, a 1‐unit increase in the number of people living with HIV and on ART, was associated a 5% increase in life satisfaction scores after controlling for observed and unobserved time‐invariant confounders. Our results were robust to alternate specifications.

**Conclusions**: Our findings suggest that further investments into ART scale‐up programmes could yield substantial wellbeing gains for young people in this region and should be leveraged as a key SDG 3 strategy.

### PEMOE37 Successful methods to ensure continuity of cervical cancer screening and pre‐cancerous lesion treatment services for women living with HIV during COVID‐19 in Mozambique

J. Barroso Pacca ^1^, E. Ferreira^2^, A. Chauca^2^, M. Prieto^3^

^1^Abt Associates, Mozambique, ^2^Pathfinder International, Maputo, Mozambique, ^3^ThinkWell, Maputo, Mozambique

**Background**: As a consequence of the COVID‐19 pandemic, from March to September 2020 Mozambique experienced severe disruption to cervical cancer screening and treatment services. The Ministry of Health (MOH), along with its stakeholders, developed new guidelines to ensure the continuity of cervical cancer services despite lockdown restrictions. We describe the implementation of these new guidelines and their impact on ensuring access to cervical cancer services in four provinces in Mozambique.

**Description**: In September 2020, USAID's Efficiencies for Clinical HIV Outcomes (ECHO) project started implementing new guidelines for cervical cancer services in 148 health facilities supported by the project, including: (1) online trainings for health providers; (2) revisions of clinical files to identify patients eligible for cervical cancer screening at reception desks, pharmacies, and clinical consultations; (3) improved organization of patients in waiting rooms to avoid crowding and provide better health education; (4) escorting of screening‐eligible patients from waiting rooms to consultation rooms; (5) weekly data assessments to evaluate site‐level performance; (6) technical assistance and formative supervision at high‐volume sites; (7) the provision of medical supplies and equipment for visual inspection with acetic acid (VIA), cryotherapy, and loop electrosurgical excision procedures (LEEP); (8) service expansion to 54 additional sites, and (9) the provision of personal protective equipment to prevent COVID‐19.

**Lessons learned**: As a result of the implementation of the new guidelines, the number of women living with HIV and on antiretroviral therapy (ART) who were screened for cervical cancer increased over time, with a monthly average of 6,967 women screened from October 2020 to September 2021, compared with a monthly average of 4,116 women screened from October 2019 to September 2020—a 69% increase. Treatment coverage, defined as the percentage of women who were screened as positive and received treatment, grew on average from 43% to 72%. Key contributors to these achievements included collaboration between the MOH and its stakeholders and the provision of medical supplies and equipment, which helped ensure service continuity and even increased coverage.

**Conclusions/Next steps**: A well‐coordinated effort between key stakeholders has been essential to the continuity and uptake of cervical cancer services in Mozambique during the COVID‐19 pandemic.

### PEMOE38 Impact of COVID‐19 on HIV testing volume and positivity in 16 countries

K. Stankevitz ^1^, C. Fischer Walker^2^, V. Ranebennur^3^

^1^FHI 360, Durham, NC, United States, ^2^FHI 360, Cincinnati, KY, United States, ^3^FHI 360, Mumbai, India

**Background**: The COVID‐19 pandemic presents many challenges to HIV testing. We aimed to describe the impact of COVID‐19 and country‐level COVID‐19 stay‐at‐home policies on HIV testing services provided by a large global HIV project.

**Methods**: We performed a retrospective analysis of monthly HIV testing data in 16 countries from January 2020 to September 2021. To account for differences in testing volume across countries, we calculated each county's percentage reduction/increase in testing by month compared to the average monthly country‐level HIV testing volume in the first three months of 2020 (pre‐COVID‐19). Regression analysis was used to quantify the relationship between HIV testing volume, HIV case‐finding rates, and national stay‐at‐home policies (from the Oxford COVID‐19 government response tracker) by month.

**Results**: HIV testing volume declined dramatically early in the pandemic, with countries averaging 37.8% and 33.1% fewer HIV tests per month in April and May, respectively. Those months also saw the highest average case‐finding rates, at 16.7% and 16.0%. In the ensuing months, programs adapted to the threats and restrictions of the pandemic and demonstrated a rebound in testing volume and an overall average case‐finding rate consistent with the pre‐COVID‐19 comparator months.

More stringent stay‐at‐home policies were associated with decreased HIV testing. Countries completed an average of 60.9% fewer tests during months when national policies required not leaving the house (p < 0.001). Positivity of testing increased an average of 1.7% during those months (p = 0.09).

**Figure:**
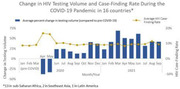
**Abstract PEMOE38‐Figure 1**.

**Conclusions**: While initial decreases in HIV testing were observed, focused testing strategies led to rapid programmatic recovery and, ultimately, HIV testing volume was maintained above pre‐COVID‐19 levels. Tighter stay‐at‐home orders were associated with decreases in testing, yet HIV case finding increased during these months. These results demonstrate the resiliency of HIV programs to adapt during the pandemic and highlight opportunities for increased emphasis on innovative strategies for HIV testing.

### PEMOE39 Measuring the impact of the COVID‐19 lockdown on testing services in specialized HIV facilities in Mexico

F. Macías‐González ^1^, H. Vermandere^1^, A. Piñeirúa‐Menendez^1^, S. Bautista‐Arredondo^1^

^1^National Institute of Public Health, Health Economics, Cuernavaca, Mexico

**Background**: Mexico's response to mitigate the spread of COVID‐19 reduced the use of non‐urgent health services, including those related to HIV/AIDS. This study aims to estimate the impact of the COVID‐19 pandemic on HIV testing.

**Methods**: We used monthly data on HIV testing (2018–2021), of 78 public health facilities that provide HIV/AIDS‐associated services in Mexico. We conducted a piecewise regression estimating the immediate change in HIV testing, positive test, and positivity rate. We used difference‐in‐difference models to measure the average impact of the lockdown in 2020 and 2021 using the average services of 2018–2019 as baseline/comparison. We distinguished the analyses by sex, subpopulation (MSM, heterosexuals, pregnant women, transgenders, IDU, and sex workers), region, and COVID‐19 mortality calculated as municipality mortality tertiles in 2020–2021.

**Results**: The lockdown in 2020 caused a sharp drop of 87% in testing and positive tests fell by 37%. The positivity rate, however, increased by 145%. Testing especially decreased among women, in northern Mexico and regions with low COVID19 mortality, while the positivity rate increase was highest among these same groups. Testing practices and positivity rate among MSM changed less than in any other risk group, even though testing decreased by 55% and the positivity rate increased by 53%. Throughout 2020, testing services remained far less used yet in 2021, the testing increased by 40% compared with 2018–2019, especially among pregnant women and people living in South Mexico. The positivity rate decreased however in 2021, reaching a lower level compared with 2018–2019 yet the difference was not statistically significant.

**Conclusions**: The confinement measures negatively affected testing services during 2020, especially among those other than MSM; those who did get tested were more likely to be positive. Health facilities have increased the number of tests carried out in 2021 and even exceeded pre‐pandemic values. Despite this increase, the incidence values of HIV in 2021 seem to be lower than those recorded in 2018–2019, indicating that those most at risk might get tested less now. Research is needed to better understand these changes in positivity rate and measures must be taken to properly target at‐risk populations.

### PEMOE40 The effects of COVID‐19 on HIV care and treatment programs: a multicountry review

S. Ramachandran ^1^, R. Machekano^1^, S. Argaw^1^, A. Tiam^1^

^1^Elizabeth Glaser Pediatric AIDS Foundation, Washington DC, United States

**Background**: Since the onset of COVID‐19, governments across the globe have implemented strategies to curb the spread of the pandemic. We reviewed HIV clinical cascade data to assess the effect of COVID‐19 and these pandemic mitigation measures on HIV programs.

**Description**: We analyzed the aggregate number of individuals who received HIV testing services, number of individuals newly initiated on ART, number of individuals currently receiving ART, and the number of ART individuals who were virally suppressed (<1000 copies/ml). A relative ratio was derived by comparing EGPAF's program data from October to December 2019 (pre‐pandemic period) to data from January 2020 to September 2021 (intra‐pandemic period). The analysis included data from Cameroon, Cote d'Ivoire, DRC, Eswatini, Kenya, Lesotho, and Malawi.

**Lessons learned**: With the exception of DRC, results revealed declines across a majority of countries in the number of individuals who received HIV testing services and number of individuals who newly initiated on ART from January to September 2020 as compared to data from the pre‐pandemic period (*see figure below*). While in some countries these declines showed gradual recovery starting in October 2020, in Eswatini, Lesotho, and Malawi figures for the two indicators continued falling, decreasing by an average of 55% and 37%, respectively, by September 2021 as compared to the pre‐pandemic data. By contrast, the number of individuals currently on ART and the number of ART individuals virally suppressed remained steady throughout the intra‐pandemic period for the majority of countries, and in some countries, these figures actually increased.

**Figure:**
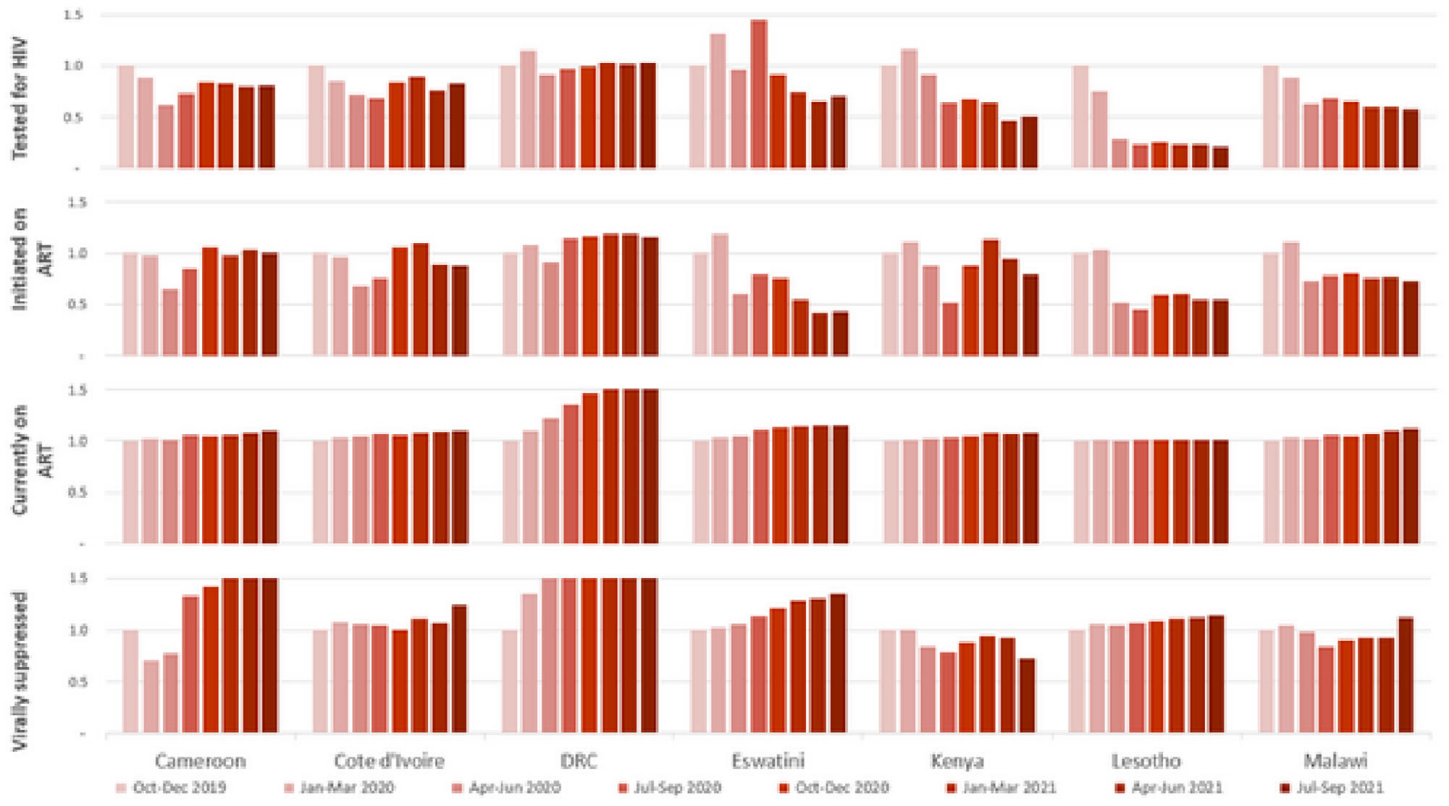
**Abstract PEMOE40‐Figure 1**.

**Conclusions/Next steps**: While the COVID‐19 pandemic and mitigation measures have impacted HIV testing and ART initiations, there were no discernable effects on the numbers of ART patients continuing to access treatment and those who were virally suppressed. The latter finding points to utility of interventions and adaptations by HIV programs, which deserve further study and replication.

### PEMOE41 Impact of COVID‐19 public health measures on art use among ugandans living with hiv in sero‐different couples

T.R. Muwonge ^1^, E. Feutz^2^, A. Meisner^2^, K. Thomas^3^, N. Ware^4^, M.A. Wyatt^4^, F. Nambi^1^, L. Nakabugo^1^, J. Kibuuka^1^, D. Thomas^2^, J. Simoni^2^, I.T. Katz^2^, H. Kadama^5^, J.M. Baeten^6^, A. Mujugira^1^, R. Heffron^2^

^1^Makerere University, Infectious Diseases Institute, Kampala, Uganda, ^2^University of Washington, Seattle Washington, United States, ^3^University of Washington, Seattle Washington, United States, ^4^Harvard Medical School, Boston, United States, ^5^Ministry Of Health, AIDS Control Program, Kampala, Uganda, ^6^Gilead Sciences, California, United States

**Background**: Approximately 30% of new HIV infections in sub‐Saharan Africa occur among heterosexual HIV serodifferent couples. Effective antiretroviral therapy (ART) eliminates HIV transmission risk and is a priority intervention. We describe how the onset of COVID‐19, which yielded restrictions to public transportation and strict curfews, impacted ART initiation and HIV viral load among people living with HIV in Uganda.

**Methods**: In a stepped‐wedge cluster‐randomized trial of an integrated PrEP and ART intervention for HIV‐serodifferent couples at 12 ART clinics in Kampala/Wakiso, Uganda (ongoing at the outset of the Covid 19 pandemic), we compared ART initiation and viral suppression among participants enrolled during different time points defined by the initial COVID‐19 lockdown. Period‐1 included participants who enrolled and had a 6‐month viral load assessment before the first COVID‐19 lockdown in Uganda on 18‐March‐2020. Period‐2 includes participants enrolled before 18‐March‐2020 with viral load measured thereafter (straddling pre‐COVID and COVID times). Period‐3 includes participants enrolled with viral load quantified after 18‐March‐2020 (entirely during COVID‐19). ART and viral load data, available through standard of care, were abstracted from clinic records.

**Results**: We enrolled 1,381 partners living with HIV, including 896 (64.9%) in Period‐1, 260 (18.8%) in Period‐2, and 225 (16.3%) in Period‐3. Almost all participants (1371, 99.3%) initiated ART within 90 days of enrollment and more than half (59.2%) had CD4 >350 cells/mm3at enrollment. Among those enrolled in Period‐1, 88.8% were virally suppressed within 6‐months of ART initiation, among those enrolled in period‐2, 80.5% were suppressed, and among those in period‐3, 88.2% were suppressed. In a generalized estimating equation model with adjustment for clustering by clinic, the small number of clusters, and the intervention phase, no pairwise comparisons of viral suppression across periods were statistically significant. The median time from ART initiation to VL assessment was greatest in period‐2: Period‐1 median time = 128 days (IQR 95–173), Period‐2 median time = 175.5 (IQR 146–206.5), Period‐3 median time = 130.5 (IQR 97.8–168.3).

**Conclusions**: Despite COVID‐19 lockdown measures, people living with HIV initiated ART and achieved viral suppression. Any potential challenges faced during the initial restricted conditions of lockdown waned and levels of ART initiation and viral suppression rebounded.

### PEMOE42 Increased prevalence of depression and anxiety among adults initiating antiretroviral therapy during the COVID‐19 pandemic in Tanzania

A. Sabasaba ^1^, S. Winters^2^, C. A Fahey^2^, P. F Njau^3^, E. Katabaro^1^, Y. Ndugile^4^, L. Packel^2^, S. I McCoy^2^

^1^Health For A Prosperous Nation (HPON), Dar es salaam, Tanzania, The United Republic of, ^2^University of California, Berkeley, School of Public Health, Berkeley, United States, ^3^Ministry of Health, National AIDS Control Program, Dodoma, Tanzania, The United Republic of, ^4^President's Office ‐ Regional Administration and Local Government (PO‐RALG), Dodoma, Tanzania, The United Republic of

**Background**: Depression and anxiety are common among people living with HIV and can impair adherence to antiretroviral therapy (ART) and increase disengagement from care. We compared the prevalence of depression and anxiety among ART initiates before and during COVID‐19 in Tanzania.

**Table:** 

**Abstract PEMOE42‐Table 1**.						
	“*A lot”*			*“Extreme” amount*		
Reported Frequency	pre‐COVID‐19	COVID‐19	Adjusted Prevalence Difference (PD_a_) (95% CI)	pre‐COVID‐19	COVID‐19	Adjusted Prevalence Difference (PD_a_) (95% CI)
Feeling little interest in things	5.3%	45.8%	38.6 (35.3,41.8)	1.3%	12.5%	9.1 (7.1,11.2)
Feeling hopeless about the future	3.8%	50.7%	46.7 (43.5,49.9)	1.7%	7.7%	4.2 (2.6,5.9)
Uncontrolled worrying	7.7%	42.9%	34.7 (31.3,38.1)	3.4%	7.7%	2.1 (0.3,4.0)

**Methods**: We analyzed baseline data from two randomized controlled trials of adults initiating ART in Shinyanga, Tanzania between April‐December 2018 (pre‐COVID‐19 period, n = 530) and May 2021‐January 2022 (COVID‐19 period, n = 542), respectively. Depression and anxiety were measured using the Hopkins Symptom Checklist‐25 in the pre‐COVID‐19 period and the Patient Health Questionnaire‐2 and Generalized Anxiety Disorder‐2 scales in the COVID‐19 period, respectively, and classified as binary indicators per each scale's threshold. We also examined three mental health indicators that were similarly measured in both surveys: loss of interest, hopelessness, and uncontrolled worrying. To account for temporal differences in participant characteristics, adjusted prevalence differences (PD_a_) in mental health over time were estimated using stabilized inverse probability of treatment weighting. Propensity scores (weights) were generated from logistic regression with age, sex, primary language, education, marital status, household head, overall health, and work status as covariates.

**Results**: The prevalence of depression was 26.6% before and 68.6% during COVID‐19 (PD_a_ = 38.0 percentage points (pp), 95% CI: 34.1,41.9). Anxiety increased from 19.8% to 63.2% (PD_a_ = 41.3pp, 95% CI: 37.4,45.0). Significant temporal increases were also noted in the prevalence of feeling “a lot” or “extreme” loss of interest, hopelessness, and uncontrolled worrying

**Conclusions**: After applying a quasi‐experimental weighting approach, the prevalence of depression and anxiety among those starting ART during COVID‐19 was dramatically higher than before the pandemic. Although depression and anxiety were measured using different, validated scales, the concurrent increases in similarly measured mental health indicators lends confidence to these findings and warrants further research to assess the impact of COVID‐19 on mental health among adults living with HIV.

### PEMOE43 Describing the impact of COVID‐19 on AIDS Drug Assistance Program operations

K. McManus^1^, A. Strumpf^1^, A. Steen^1^, Z. An^1^, E. Schurman^1^, A. Killelea^2^, T. Horn ^3^, A. Hamp^3^, J. Keim‐Malpass^1^

^1^University of Virginia, Charlottesville, United States, ^2^Killelea Consulting, Arlington, United States, ^3^National Alliance of State & Territorial AIDS Directors (NASTAD), Washington, United States

**Background**: In the United States (U.S.), state AIDS Drug Assistance Programs’ (ADAPs) are a key part of the US HIV healthcare delivery safety net. ADAPs provide free antiretroviral therapy to people with low incomes who are uninsured/underinsured. As a public service program, ADAPs’ operations were impacted by the COVID‐19 pandemic. To better understand how operations were affected and what changes were implemented in response, the National Alliance of State and Territorial AIDS Directors (NASTAD) surveyed ADAPs in 2021. The objective of this mixed methods study was to characterize the programmatic challenges and subsequent innovations.

**Methods**: Data about COVID‐19‐related challenges and innovations were collected via the 2021–2022 NASTAD National Ryan White HIV/AIDS Program Part B and ADAP Monitoring Project, a cross‐sectional survey of state, district, and territorial ADAPs. Descriptive statistics were used to assess proportional differences in Likert‐style responses. Qualitative responses were coded and analyzed using a thematic analysis framework.

**Results**: Forty‐seven state and D.C. ADAPs responded to the survey (92%, response rate). The majority of ADAPs reported that maintaining client eligibility (78%) and working remotely (70%) were the most challenging aspects of the pandemic, particularly in regards to implementing new telehealth and e‐certification eligibility platforms. In response to COVID‐19, ADAPs introduced enrollment “grace periods” (19%) while bolstering client outreach (11%), expanded medication supply for more than 30 days (79%), and provided pharmacy home delivery for clients (80%). Figure 1 provides a situational map of themes and subthemes from open‐ended questions.

**Figure:**
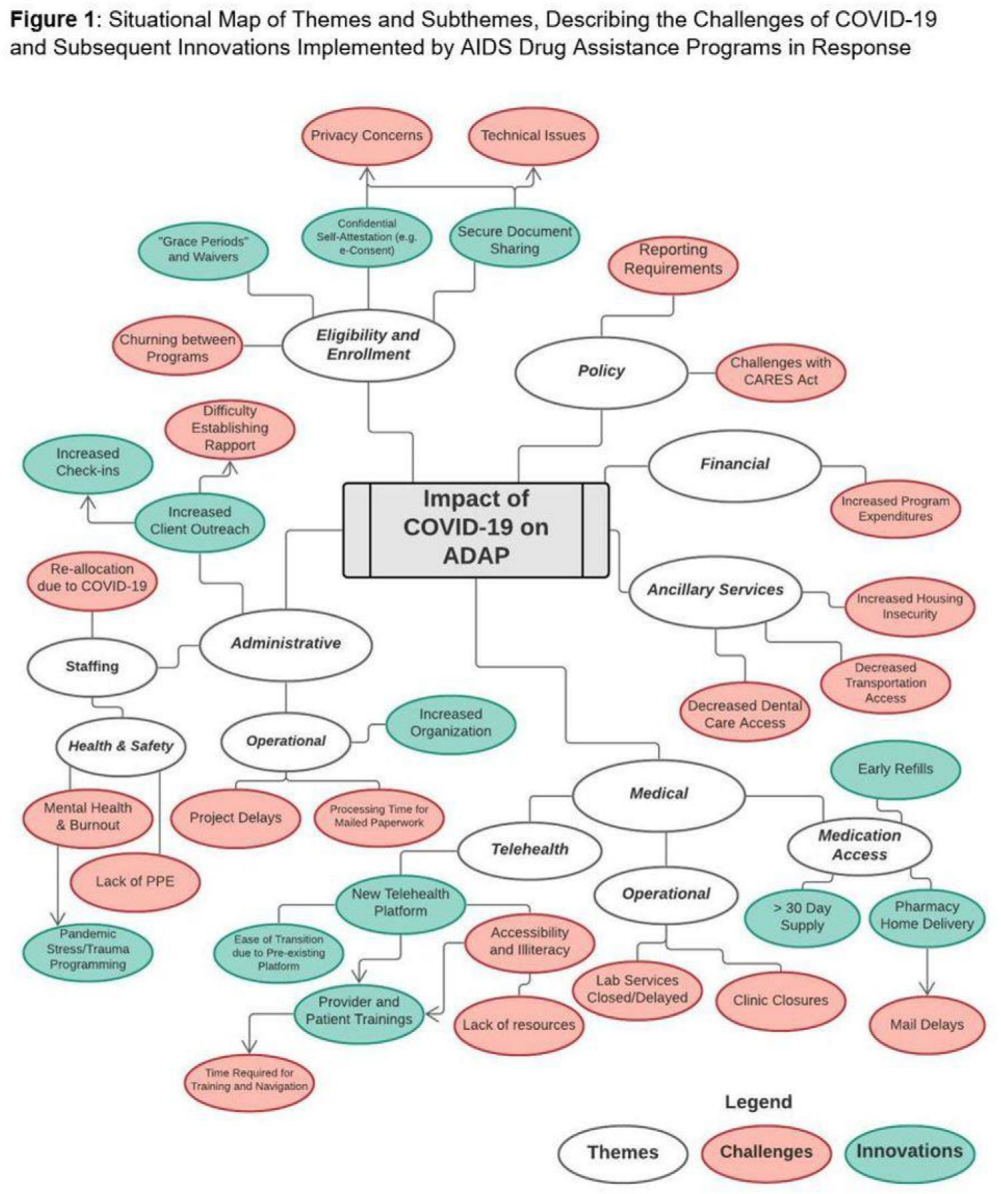
**Abstract PEMOE43‐Figure 1**.

**Conclusions**: Despite the multifaceted challenges of the COVID‐19 pandemic, state ADAPs implemented several operational innovations in order to continue providing prescription drug assistance. Additional studies are warranted to assess the retention of successful policies over time, what impact they had on the individual client level, and what factors may improve the acceptability of telehealth and e‐certification platforms.

### PEMOE44 Maintaining access to HIV services to persons living with HIV amid the COVID‐19 pandemic in Nepal

R. Baskota ^1^, R. Baral^2^

^1^Ministry of Health and Population, Department of Health Services, Kathmandu, Nepal, ^2^Ministry of Health and Population, Department of Drug Administration, Kathmandu, Nepal

**Background**: The surge of COVID‐19 infection and subsequent lockdown resulted in closure of many public and private health facilities, including those providing HIV services. While the national prevalence of HIV was 30300 in 2020, many persons with HIV could not continue to access services from the routine health facilities. The National Centre for AIDS and STD Control (NCASC) practiced innovative strategies with the main objective of ensuring uninterrupted access to HIV services amid the pandemic.

**Description**: Innovations were practised since June 2020 through May 2021 in the HIV centres of high burden districts based on key population estimates, HIV prevalence and COVID‐19 hotspot mapping. During the period of travel restrictions, risk assessment was conducted virtually to the key populations. NCASC initiated travel pass program to support organizations that provide needle syringe exchange and opioid substation therapy. Antiretroviral drugs were delivered at home through multiple national networks of people with HIV. The targeted beneficiaries of these activities included people with HIV, pregnant women, HIV infected infants and children, and people who inject drugs.

**Lessons learned**: Nearly 50% of the targeted clients benefitted by the interventions. A total of 8542 virtual consultations were conducted. Through travel pass program, 2702947 syringes were distributed; benefiting 3364 people who inject drugs. Through the same program, 328 people received Methadone and 92 people received Buprenorphine. Among the 20883 people with HIV currently on ART, 9900 received home delivery of ART. This program included 157 (73%) pregnant women with HIV, 121 (56%) mothers with HIV and 900 (71%) HIV infected infants and children. These approaches were feasible and were acceptable by the communities as they provided continuity of care while lockdown had imposed barriers to access to most HIV services.

**Conclusions/Next steps**: Virtual consultations, travel pass program and home delivery of medicines were effective strategies of maintaining continuity of HIV services amid the COVID‐19 pandemic in Nepal. Such models can be implicated to other chronic health conditions that require long‐term care in situations like the COVID‐19 pandemic and related lockdowns that impose substantial barriers to access to health services. Supportive policies are needed to sustain such decentralized services in communities.

### PEMOE45 Building infection prevention and control capacity across PEPFAR programs in response to the COVID‐19 pandemic

D. Gomes ^1^, I. Lawal^2^, N. Zender^3^, P. Kerndt^4^, T. Lucas^1^, D. Forno^1^, D. Goldstein^4^, S. Vallabhaneni^1^, B. Park^1^, A. Date^1^, E. Bancroft^1^, C. Godfrey^3^

^1^Centers for Disease Control and Prevention, Atlanta, United States, ^2^United States Department of Defense, Abuja, Nigeria, ^3^United States Department of State, District of Columbia, United States, ^4^United States Agency for International Development, District of Columbia, United States

**Background**: Effective infection prevention and control (IPC) practices are essential for safe healthcare delivery. In low‐ and middle‐income countries, the COVID‐19 pandemic exposed ineffective IPC systems which increased healthcare worker and patient risk for infection and reduced patient access to life‐saving medical care. Therefore, the President's Emergency Plan for AIDS Relief (PEPFAR), committed to ensuring the safe delivery of high‐quality healthcare, identified IPC as an urgent priority.

**Description**: Early in the COVID‐19 pandemic, a technical working group with public health and IPC experts, from United States (U.S.) Government agencies responsible for implementing PEPFAR programs, began collaborating to improve IPC in PEPFAR programs. In July 2021, the Office of the U.S. Global AIDS Coordinator formally identified this group as the site safety short term task team (ST3).This ST3 is responsible for defining minimum IPC standards for PEPFAR‐supported facilities and developing strategies for IPC implementation and monitoring.

**Lessons learned**: IPC priorities identified by the ST3 were added to PEPFAR's minimum program requirements and include respiratory hygiene, standard and transmission‐based precautions, and healthcare worker safety such as post‐exposure prophylaxis for HIV. Recommendations to support program alignment with IPC priorities were included in PEPFAR's COVID‐19 guidance and in country and regional operational plan guidance. IPC was prioritized for additional COVID‐19 funds received by PEPFAR; the ST3 developed recommendations to guide rational use of these funds. In addition, the ST3 developed indicators to assess adherence to IPC standards as a mandatory component of PEPFAR's facility‐level quality assurance tool, a personal protective equipment forecasting calculator, and aids to prevent infections in PEPFAR‐supported surgical procedures.

**Conclusions/Next steps**: The site safety ST3 quickly developed IPC standards for PEPFAR programs and ensured that IPC was incorporated into PEPFAR's minimum program requirements to enforce accountability across programs and facilities. Implementation of IPC standards will improve quality of care, protect healthcare workers and patients, and strengthen the resiliency of the healthcare system beyond COVID‐19. This experience highlights a cross‐agency collaboration developed to rapidly respond to a critical public health need and demonstrates how PEPFAR programs can be mobilized to respond to COVID‐19‐like pandemics and impact health systems and health security moving forward.

### PEMOE46 A pandemic over an epidemic: surveying LGBTQI+ situation during COVID‐19 pandemic in Thailand

K. Honglawan ^1^, I.Y.‐H. Chu^2^, R. Olete^3^, I.Q. Rendon^4^, M. Poonkasetwattana^5^

^1^Adelphi University, Garden City, United States, ^2^London School of Hygiene & Tropical Medicine, Department of Public Health, Environments and Society, London, United Kingdom, ^3^National Cheng Kung University, Department of Nursing, Tainan, Taiwan, Province of China, ^4^APCOM Foundation, Bangkok, Thailand, ^5^SOAS, University of London, London, United Kingdom

**Background**: COVID‐19‐related mobility restrictions have devastated the lives of PLHIV and other key populations (KPs) worldwide. After Thailand's first national lockdown in April 2020, in collaboration with eight community‐based organizations (CBOs), APCOM initiated the Khormoon (“Information” in Thai) project to identify post‐COVID‐19 lockdown health situations and the critical needs of KPs in Thailand.

**Description**: From September 2020 to November 2020, the Khormoon team administered a cross‐sectional online survey co‐designed by APCOM, the eight CBOs, and KP representatives. The 51‐item questionnaire contained four dimensions: access to HIV services and information on COVID‐19, socioeconomic status, mental health status, and life challenges. We recruited participants and distributed the online survey through both websites and service facilities of all partnered organisations.

**Lessons learned**: The project reached 1,323 KPs across all regions of Thailand. Of all, 69% were identified as PLHIV, 82% of whom underwent antiretroviral treatment (ART). Most (90%) of the PLHIV suffered from psychological distress, while almost all respondents (93%) reported income losses with 44% being unemployed. Affording daily expenses and accessing medicine were considered the two most urgent needs (85% and 56%, Figure). Although 80% of respondents reported receiving combination HIV prevention/treatment and information on COVID‐19, the majority (66%) worried that sexual health services (eg., ART, PrEP, and PEP) could become inaccessible if COVID‐19‐related travel restrictions persist. Regarding community support, 85% agreed that organisations had ensured their access to HIV/STI care during the COVID‐19 lockdown with adaptive/innovative measures (eg., online consultations, 24‐hour hotlines and door‐to‐door ART delivery).

**Figure:**
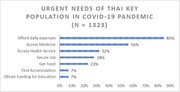
**Abstract PEMOE46‐Figure 1**.

**Conclusions/Next steps**: Our Khormoon project highlights the urgent needs of KPs in Thailand, who continue requiring differentiated support to tackle socioeconomic disparities and mental distress heightened by the ongoing COVID‐19 pandemic. The findings illuminate our future projects on delivering user‐centered, innovative, and comprehensive community support to people affected by HIV and COVID‐19.

### PEMOE47 Analyzing FY 19–21 PEPFAR PrEP uptake focusing on adolescent girls and young women and key populations

S. Straitz ^1^, T. English^1^, L. Stubbs^1^, S. Blatz^1^

^1^U.S. Department of State, Office of the Global AIDS Coordinator, Washington, United States

**Background**: From FY 19–21, PEPFAR significantly scaled PrEP uptake, particularly for adolescent girls and young women and key populations. Despite the impact of SARS‐CoV‐2 on community mobilization for, and delivery of, PrEP to these vulnerable populations, the number of individuals newly initiated on PrEP still increased across all age and sex bands from FY 19–20 and accelerated from FY 20–21 with commensurate increases in PrEP expenditures.

**Description**: Annual FY19–21 (October 1, 2018‐September 30, 2021) PrEP expenditures and semiannual FY19–21 PrEP_NEW, the number of individuals newly enrolled on PrEP in the past reporting period, results are included for 48 PEPFAR‐supported countries.

**Lessons learned**: From FY19–20, global PrEP uptake increased by 91% (163,452 to 312,017) while PrEP expenditures increased 112% ($16,822,939 to $35,726,992). Adolescent girls and young women drove FY 20–21 PrEP uptake. FY20–21 global PrEP uptake accelerated by 225% (312,017 to 1,015,094) and global PrEP expenditures by 189% ($35,726,992 to $103,133,294).

**Figure:**
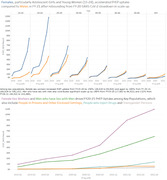
**Abstract PEMOE47‐Figure 1**.

Among key populations, female sex workers increased PrEP uptake from FY19–20 by 150% (28,018 to 69,924) and again by 185% from FY 20–21 (69,924 to 199,152). Men who have sex with men also contributed significant scale‐up by 148% from FY19–20 (17,983 to 44,522) and 132% from FY20–21 (44,522 to 103,480).

**Conclusions/Next steps**: From FY19–21, PrEP uptake increased 712% from 163,452 to 1,327,111 clients on PrEP while PrEP expenditures increased 725% from $16,822,939 to $138,860,286. Future analysis should evaluate countries with most efficient PrEP expansion by age/sex bands to determine how enabling environments to support PrEP scale‐up in those countries can be replicated in other country contexts.

### PEMOF25 Litigating against forced and coerced sterilisation in Kenya

V. Njogu ^1^

^1^KELIN, Strategic Litigation, Nairobi, Kenya

**Background**: As programs for the prevention of mother to child transmission of HIV were launched around the world, discriminatory attitudes and practices toward women living with HIV continued to emerge. There were untrue beliefs that women living with HIV could not, or should not, bear children which led to a great number of them being subjected to involuntary sterilization. Between 2005 and 2010, there was a sustained unofficial policy that led to the forced and coerced sterilization of women. They had several things in common. They were: living with HIV, of lower social‐economic status, receiving medicine and food rations for themselves and their children, as part of programs to prevent mother to child transmission, and were threatened with withdrawal of the medical and food assistance if they did not produce evidence of permanent family planning . They were subjected to unwanted sterilization despite the fact that science had proven that with adequate medical care and essential medication, they could bear healthy children.

**Description**: In 2014, KELIN assisted five of these women to file suit in court against the facilities that coerced them into undergoing forced sterilization highlighting the violations they suffered. In their claim, they are also seeking state interventions to ensure that forced sterilization never happens again. That litigation is still pending, now in its 8^th^ year.

**Lessons learned**:

1. The factors that resulted in the vulnerability of the victims to forced sterilization must be addressed alongside litigation.
2. Litigation is a lengthy, technical and sometimes emotional process, and the likelihood of retraumatising the victims is ever‐present. We must anticipate delays and the needs of the victims
3. It is only one step in the journey of redress for women who are living with consequences of forced and coerced sterilization.

**Conclusions/Next steps**: We are expecting the conclusion to this process in 2022. Litigation is useful to secure individual redress and social change, but does not guarantee success. We will scale up other avenues for advocacy through which women living with HIV can secure justice, including social advocacy programmes to increase education about living with HIV, economic empowerment, and availability of medical and psychosocial support.

### PEMOF26 Youth‐Led Monitoring for Accountability in the HIV Response

C. Modi ^1^, G. Jones^2^, E. Dupuis^3^, A. Sanchez^2^, T. Rufurwadzo^1^, A.R. Khan^3^

^1^Global network of Young People Living with HIV (Y+ Global), Amsterdam, Netherlands, the, ^2^UNAIDS, Geneva, Switzerland, ^3^The PACT, Bangkok, Thailand

**Background**: Youth‐led data generation remains an underfunded yet potentially key aspect of the HIV response for young people in all our diversity. To address this, a youth‐led monitoring tool (#UPROOT scorecard) was developed with youth‐led organisations to measure progress on key issues that impact young people in the context of HIV at the country level.

From 2017–2020, The PACT, with support from UNAIDS, implemented the scorecard in 16 countries across five regions (LAC, EECA, WCA, ESA, AP). Youth‐generated data was used to catalyse targeted advocacy at the country level to improve the legal, policy, and social landscape for young people.

**Description**: Qualitative and quantitative data are gathered to create a youth‐generated snapshot of national HIV responses.

Data from the NCPI, GAM, and national databases are analysed and coupled with a participatory consensus‐based exercise to determine the impact of laws, policies, and the effects of stigma and discrimination on young people in all their diversity.

**Lessons learned**: Young people reported that they felt empowered throughout the monitoring process.

National profiles that identify which areas of the HIV response for young people require more attention and where advocacy should be focused were created

Areas for improvement included putting more emphasis on supporting advocacy plans after the scorecard creation, and managing the consensus process to ensure a balance report of the situation in the country.

Funded by UNAIDS Technical Support Mechanism and managed globally by Y+ Global with support from The PACT, the revised methodology will be conducted in 7 countries in 2022. Results from the current implementation in 7 countries will be available by June 2022.

**Conclusions/Next steps**: Youth‐led data generation and monitoring have the potential to more accurately represent the views of young people than mechanisms that are not designed by and for youth but that include them. Data are not often disaggregated enough for young people and do not focus on their specific needs, therefore the youth‐led approach of the #UPROOT scorecard allows for young people to have a say on our experience of the HIV response. Scaling up of youth‐led monitoring can help identify priority action areas for tackling the HIV epidemic in young people.

### PEMOF27 PLWH Stigma Index 2.0. in the countries of Central Asia

L. Vorontsova ^1^

^1^ALE Central Asian Association of People Living with HIV, Almaty, Kazakhstan

**Background**: The purpose of the study is to obtain information about the problems of PLHIV related to stigmatization, discrimination and violation of their rights. This abstract presents a comparison of study data in three countries ‐ Kazakhstan, Tajikistan, Kyrgyzstan. These countries share the same epidemic history, HIV laws, education and public opinion. The main issues of stigma and discrimination here affect women and key populations.

**Methods**: The methodology of this study is based on the method developed and recommended by the GNP+, ICW, and the Joint United Nations Program on HIV/AIDS (UNAIDS).

**Figure:**
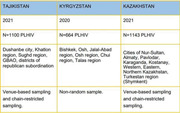
**Abstract PEMOF27‐Table 1**.

**Results**: Almost all PLHIV tend to hide their status from other people. About two‐thirds feel devalued because of their status and many (71%) feel ashamed about it.

A large majority (87%) find it difficult to share their status with others. Women are verbally reprimanded 6 times more often than men. The majority of PLHIV are not aware of the existence of laws protecting their rights in the country, only less than a third declared their knowledge of such laws. CPs face higher levels of stigma and discrimination for reasons other than HIV.

**Figure:**
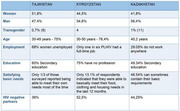
**Abstract PEMOF27‐Table 2**.

**Conclusions**: In all three countries, there is a high level of internalized stigma. There is low legal literacy among PLHIV. Stigma and discrimination not related to HIV is quite high. There is a problem with the confidentiality of diagnosis and informed consent in the provision of medical services. It is necessary to change a number of legal acts.

### PEMOF28 Technical Support for PLHIV Leadership in Implementation of the PLHIV Stigma Index 2.0

P. Looze^1^, K. Lalak^1^, O. Syarif^1^, K. Dunaway ^2^, S. Brion^3^, L. Sprague^4^, M. Chisenga Chungu^5^, D. Matyushina‐Ocheret^6^, C. Garcia de Leon Moreno^4^, G. Turpin^7^, L. Lucas^7^, S. Baral^7^, C. Lyons^7^

^1^Global Network of People living with HIV (GNP+), PLHIV Stigma Index, Amsterdam, Netherlands, the, ^2^International Community of Women Living with HIV (ICW), Stigma Index Equity Officer, Buenos Aires City, Argentina, ^3^International Community of Women Living with HIV (ICW), Sarasota, United States, ^4^UNAIDS, Community Engagement, Geneva, Switzerland, ^5^UNAIDS, Community Mobilization Community Support, Social Justice & Inclusion, Geneva, Switzerland, ^6^Consultant, Geneva, Switzerland, ^7^Johns Hopkins Bloomberg School of Public Health, Department of Epidemiology, Baltimore, United States

**Background**: In 2022, stigma is understood as a fundamental barrier to ending the HIV pandemic by 2030, in just 8 years. The People Living with HIV (PLHIV) Stigma Index 2.0 (SI) represents an instrument to monitor HIV‐related and intersectional stigma affecting PLHIV including key populations (KP). Standardizing the instrument and implementation of the SI facilitates measurement of progress in stigma mitigation over time and across geographies. The leadership of PLHIV in SI implementation and their ownership of the results is critically important and PLHIV networks need financial and/or technical support (TS) to implement.

**Description**: Since 2020, the International Partnership for SI (GNP+, ICW, John Hopkins University, and UNAIDS), funded through the UNAIDS Technical Support Mechanism, provides TS covering PLHIV leadership, partnership building and advocacy, as well as research protocol development, data collection, analysis, and report writing. Four data analysis toolkits and guidelines have been developed to strengthen implementation steps. TS has been provided to support PLHIV‐led implementations in over forty countries, eight of which have finalized implementations. Non‐negotiables of implementation emphasize PLHIV leadership, inclusion of all KPs living with HIV as respondents, and overall compliance with the approved methodology.

**Lessons learned**: The measurement and interventions focused on stigma remain controversial in many settings, resulting in challenges including leadership of PLHIV contested by national stakeholders, and the criminalization of KPs requiring coordinated efforts of communities, donors, and technical partners to safeguard the engagement of KPs as respondents. In response, we have learned that tailored technical support provision, capacity‐building exercises for PLHIV‐led organizations, and the clarification of the non‐negotiables from the outset, can ensure quality implementation of the SI. Moreover, iteration of the SI has uncovered opportunities to enhance TS leading to the development of toolkits aimed at improving data analysis, report writing, and advocacy.

**Conclusions/Next steps**: Quality and long‐term technical support provision, based on a partnership between communities, academia, and technical agencies create a strong foundation for community‐led monitoring. Data from the stigma index 2.0 can inform advocacy approaches, policy, and community leadership—all of which are central in improving HIV outcomes and ultimately, responding to the HIV pandemic.

### PEMOF29 Increased frequency of experiencing multiple forms of discrimination in healthcare settings during the COVID‐19 pandemic among African, Caribbean, and Black (ACB) people across Canada

S. Baidoobonso ^1^, A. Kaida^2^, J. Etowa^3^, J. Osuji^4^, A. Ogunleye^5^, L. Azangtsop^6^, E. Etowa^7^, A. Odhiambo^8^

^1^Dalhousie University, Department of Community Health and Epidemiology, Halifax, Canada, ^2^Simon Fraser University, Burnaby, Canada, ^3^Univerity of Ottawa, Ottawa, Canada, ^4^Mount Royal University, Calgary, School of Nursing & Midwifery, Calgary, Canada, ^5^Black Cultural Society of PEI, Charlottetown, Canada, ^6^University of Ottawa, Ottawa, Canada, ^7^University of Windsor, Department of Sociology, Anthropology & Criminology, Windsor, Canada, ^8^Public Health Agency of Canada, Surveillance and Epidemiology Division Centre for Communicable Diseases and Infection Control Infectious Disease Prevention and Control Branch, Ottawa, Canada

**Background**: In Canada, racialized communities, including African, Caribbean, and Black (ACB) people, are disproportionately affected by HIV and COVID‐19. Experiencing multiple forms of discrimination (e.g., racism, sexism, genderism, ageism, classism, ableism) in healthcare settings compromises care engagement and health outcomes. Among a national sample of ACB people, we examined changes in experiencing discrimination in healthcare settings during the COVID‐19 pandemic.

**Methods**: Cross‐sectional data were collected using an online survey co‐led by the Public Health Agency of Canada, University of Ottawa, and ACB community leaders and researchers to examine COVID‐19 impacts on ACB people aged 18+ (May 25‐July 12, 2021). Participants reported on experiences of discrimination when accessing healthcare services in the year prior to and during the pandemic. Descriptive statistics are provided.

**Results**: The 1,556 participants were diverse by age, ethnic identity (Black African (63.2%), Black Caribbean (28.3%), Black Indigenous or Black Canadian (7.3%)), gender (trans‐gender (3.0%)), and sexual orientation (11.9% identified as LGB). Among those who accessed healthcare in the year prior to COVID‐19 (n = 982), participants reported that they “Often” (10.2%), “Sometimes” (32.8%), or “Rarely” (19.1%) experienced discrimination. Among those who also accessed healthcare during COVID‐19 (n = 902), 25.2% reported increased frequency of experienced discrimination (9.2% decreased).

During the pandemic, participants reported an increase in discrimination by race (including anti‐Black racism) (31.2% reported an increase), economic status (18.7%), age (18.1%), (dis) ability (17.0%), substance use (15.9%), gender (15.3%), and sexual orientation (10.8%). Between 2.9%‐13.5% reported a decrease in various forms of discrimination.

Among participants living with HIV (10.3%), 26.7% cited concerns about experiencing stigma, discrimination, or violence as barriers to accessing HIV services during the pandemic, with 20.0% reporting fear of experiencing racism.

**Conclusions**: A sizable proportion of ACB people in Canada often/sometimes experience discrimination while accessing healthcare services, and for many, discriminatory experiences increased during the COVID‐19 pandemic. One‐fifth of survey participants living with HIV cited racism as a barrier to accessing HIV services. In partnership with communities, concerted efforts are needed to address multiple forms of discrimination in healthcare settings to improve care engagement and health equity among ACB communities during COVID‐19 and beyond.

### PEMOF30 Have mandatory testing laws become a serious threat to HIV responses? Lessons learned from a civil society advocacy campaign in Australia

F. Delhomme ^1^, M. Holt^2^, A. Cogle^3^

^1^ACON, Surry Hills, Australia, ^2^UNSW Sydney, Centre for Social Research in Health, Sydney, Australia, ^3^National Association of People With HIV Australia, Newtown, Australia

**Background**: Since 2015, a coalition of civil society organisations has advocated against the introduction of new mandatory testing laws in Australia. Proposed by police unions, mandatory testing laws have been formally introduced by several state governments. They allow individuals to be tested for HIV and other blood borne viruses (BBV) without consent, including after low or no risk exposures (e.g. to saliva).

**Description**: The “Stop Mandatory Testing” campaign was conducted by a broad coalition of civil society organisations, including HIV and LGBTQ+ organisations, medical associations, legal aid organisations, health worker unions and public health researchers. Aimed at convincing legislators to withdraw or amend the proposed laws, the campaign addressed lobbying efforts by police unions seeking the introduction of mandatory testing in jurisdictions across the country, including Australia's most populous state of New South Wales. The campaign centred on the provision of scientific and empirical evidence and personal testimony to convince Australian decision‐makers that compulsory testing violated rights, increased stigma and fear, could be misused punitively, and was proposed for situations that carried little to no risk of transmission (e.g., spitting). The campaign highlighted that mandatory testing was in conflict with Australia's rights‐based response to HIV/BBV, would hinder testing and stigma reduction targets, and that no occupational HIV transmission had ever been recorded in a police officer. Strategies evolved over the course of the campaign and included the production of reports, submissions, appearances at parliamentary inquiries, a campaign website, media and targeted advertising.

**Lessons learned**: The failure of the campaign to stop mandatory testing laws offers a stark warning about the risk of backsliding and fracturing of HIV responses, and that evidence may be insufficient to prevent regressive laws. This failure offers important lessons for civil society, including the need for stronger influence over policymakers, tactics to generate public attention or shame lawmakers, and strategic multi‐jurisdiction lobbying.

**Conclusions/Next steps**: Considering how evidence was overlooked and the threats posed by growing police powers, we need to consider renewed political and intersectional alliances, including with political parties and organisations and communities affected by police violence. After forty years of HIV responses, the need for community mobilisation remains.

### PEMOF31 Dos and don'ts for community‐led HIV programming in challenging contexts in Eastern Europe and Central Asia: lessons learned for funders and implementers

J. Rashbass ^1^

^1^Elton John AIDS Foundation, London, United Kingdom

**Background**: New HIV infections and AIDS‐related deaths are growing faster in Eastern Europe and Central Asia (EECA) than any other region. The epidemic is concentrated among Key Populations (KPs), who are widely marginalised. Since 2017, international HIV funding in EECA has reduced significantly. Elton John AIDS Foundation (EJAF) implemented the EECAKPs Fund from 2018–2021 to increase KPs’ access to innovative HIV services; reduce stigma and discrimination; and collate best practices. Resources were prioritised in settings with large epidemic burdens where NGOs cannot access alternative resources.

**Description**: Nineteen local NGO‐led projects provided direct services to 149,263 people from KP communities, including 100,302 HIV tests, and initiated 8,924 PLHIV on treatment. EJAF commissioned end‐of‐project mixed‐method evaluations of the 12 largest projects in 2021 to assess results and inform future intervention design.

**Lessons learned**: Marginalisation of KPs in EECA means earning trust is crucial to programmatic success. To build clients’ trust: (1) Engage clients as staff and volunteers to connect with the hardest‐to‐reach; projects fostering collaboration between effective peer‐led NGOs and government institutions also reduce stigma; (2) Adapt harm reduction services for new stimulant users to make services relevant; (3) Integrate with services that resonate with communities’ needs beyond HIV.

Implementers most effectively improve HIV outcomes by: (1) Proactively reaching hard‐to‐reach sub‐populations, such as synthetic stimulant users, women who use drugs, and MSM practicing chemsex. This correlates with higher testing yields; (2) Providing innovative entry points, including online and home‐based services. This was particularly productive during COVID‐19; (3) Ensuring all staff, including in remote implementation sites, can access mental health support to prevent burnout.

To ensure sustainability: (1) Secure cost‐share from government; (2) Mobilise local private donations, proven feasible even for locally controversial causes; (3) Partner with diverse government agencies, including law enforcement, proven an unlikely success; (4) Strive for system changes in initial project design; (5) Bake NGO capacity development into project design so that it happens intentionally.

**Conclusions/Next steps**: With the HIV epidemic growing in EECA and limited domestic resources allocated to KPs, expanded international donor support is urgently needed. The EECAKPs Fund shows effective foreign‐funded community‐led HIV services for KPs in challenging EECA environments are possible, if programmes intentionally earn communities’ trust, optimise service modalities, and bake in sustainability approaches.

### PEMOF32 A digital crowdsourcing open call on adolescent and young adult consent for HIV research participation in low‐ and middle‐income countries

S. Day ^1^, K. Tahlil^2^, T. Gbaja‐Biamila^3,4^, E.C. Wilson^5^, C. Obiezu‐Umeh^3^, U. Nwaozuru^6^, S. Rennie^7,8^, J. Iwelunmor^3^, S. Shah^9,10^, K. Chima^4^, O. Ezechi^4^, J.D. Tucker^1,11^

^1^The University of North Carolina at Chapel Hill, Department of Medicine ‐ Division of Infectious Diseases, Chapel Hill, United States, ^2^The University of North Carolina at Chapel Hill, Department of Epidemiology, Chapel Hill, United States, ^3^St. Louis University, Department of Behavioral Science and Health Education, St. Louis, United States, ^4^Nigerian Institute of Medical Research, Clinical Sciences Department, Lagos, Nigeria, ^5^San Francisco Department of Public Health, San Francisco, United States, ^6^Wake Forest School of Medicine, Department of Implementation Science, Winston‐Salem, United States, ^7^The University of North Carolina at Chapel Hill, Department of Social Medicine, Chapel Hill, United States, ^8^The University of North Carolina at Chapel Hill, UNC Center for Bioethics, Chapel Hill, United States, ^9^Northwestern University Feinberg School of Medicine, Department of Pediatrics, Chicago, United States, ^10^Ann and Robert H Lurie Children's Hospital of Chicago, Mary Ann and J Milburn Smith Child Health Research, Outreach, and Advocacy Center, Stanley Manne Children's Research Institute, Chicago, United States, ^11^London School of Hygiene and Tropical Medicine, Clinical Research Department, Faculty of Infectious and Tropical Diseases, London, United Kingdom

**Background**: Social, ethical and legal barriers to consent for HIV research participation among adolescents and young adults (AYA; 10–24 years old) can hinder AYA inclusion in research. Engaging AYA and other stakeholders in identifying solutions to these barriers is especially important in low‐ and middle‐income countries (LMICs), where youth face high HIV morbidity and mortality.

**Methods**: A digital crowdsourcing open call for ideas to improve AYA consent in LMIC HIV research was held from August 23 – October 15, 2021. Crowdsourcing involves having a group contribute creative solutions to a problem, then sharing the results. Participation was open to anyone living or working in LMICs. Email and social media were used to reach AYA, parents, HIV researchers, community organizers, and ethicists. Submissions were scored on a 1–10 scale by three independent judges for clarity, relevance, feasibility, innovation, and potential impact, with $2000 USD for finalist prizes. Participants’ demographic data were collected, and submissions were qualitatively analyzed for emergent themes in the proposed solutions.

**Results**: Of 110 total submissions, 65 submissions from 10 LMICs were eligible for judging per open call criteria (described a solution to improve AYA consent). Of these, 25 submissions scored 6/10 or greater. Fifty‐eight participants submitted the 65 eligible submissions, including 30 (51.7%) participants age 18–24 years old, 26 (44.8%) cis‐gender women, and 5 (8.6%) members of key populations (e.g. men who have sex with men). Using thematic analysis, seven main themes were identified for solutions to improve AYA consent processes, including ways to enhance AYA and parental research engagement, increase awareness of HIV and research processes, and make research participation more AYA‐friendly (see infographic).

**Conclusions**: Improving AYA consent processes in HIV research will require creative, community‐ and AYA‐oriented solutions. Open calls engaging AYA and other stakeholders in LMICs on the topic of consent can identify promising ideas for developing practical guidance.

**Figure:**
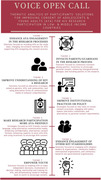
**Abstract PEMOF32‐Figure 1**.

### PEMOF33 The Long Tail of Warp Speed: The politics of HIV science and the role of the AIDS pandemic in generating the COVID‐19 scientific response

D. Werb ^1,2,3^

^1^University of Toronto, Dalla Lana School of Public Health, Toronto, Canada, ^2^University of California San Diego, Department of Medicine, La Jolla, United States, ^3^Centre on Drug Policy Evaluation, Toronto, Canada

**Background**: Humanity's control of the AIDS pandemic in the absence of a viable vaccine is one of the 20^th^ century's greatest scientific achievements. The success of that effort is consequential in ways that go far beyond HIV itself. In fact, it is because of the decades‐long effort to control AIDS that humanity is, two years into the pandemic, equipped with an assortment of vaccines, antiviral treatments, and public health protocols that have the power to stop people from dying.

**Description**: The profound impact of HIV science–both individual scientists and the scientific organizations created to control the AIDS pandemic–is largely unknown. Through qualitative interviews with leading HIV scientists (*n* = 29), this presentation will explore the history of HIV science and describe the key ways that this has intersected with, and driven, the scientific response to COVID‐19.

**Lessons learned**: The scientists interviewed described three pathways by which HIV science generated the COVID‐19 response. First, decades of failure on an HIV vaccine contributed to an expansion of the field of immunology, which directly led to the development and testing of mRNA vaccines. Second, the “scientific‐industrial complex” created to end the AIDS pandemic—parts of which were rebranded as Operation Warp Speed, under the direction of the AIDS Clinical Trial Group—were critical to the development of antivirals and other therapies to treat COVID‐19. Third, the strategy of using HIV antiviral medicines as ‘Treatment as Prevention’ to end the AIDS pandemicis increasingly being considered as a way to control the impact of SARS‐CoV‐2 variants with escape mutations that threaten the effectiveness of vaccines.

**Conclusions/Next steps**: While the public discourse suggests that Operation Warp Speed brought COVID‐19 science from nothing to vaccines within a matter of months, it is the continuous decades‐long public funding devoted to tackling the AIDS pandemic that powered the scientific response to SARS‐CoV‐2. Public funding for monitoring of pandemic threats, and the continued support of scientific efforts to end the AIDS pandemic, are the most critical priority areas to protect humanity from current and future opportunistic pathogens.

### PEMOF34 Post Covid increase of HIV Prevalence among Rural Migrant population and Women in India – Need to re‐design Migrant/Rural Interventions

V.S. Prasad ^1^, E. Michael^2^, M. Kumar^3^, S. Dwivedi^4^, K. Prasanna Viswanath^5^, L. Hong^6^, J. Vandenhombergh^7^, A.S. Benzaken^8^, F.F. Fonseca^8^

^1^AIDS Healthcare Foundation India, Management Health Care Services, New Delhi, India, ^2^AIDS Healthcare Foundation India, Operations and Partnerships, New Delhi, India, ^3^AIDS Healthcare Foundation India, Monitoring and Evaluation, New Delhi, India, ^4^AIDS Healthcare Foundation India, Prevention Services, New Delhi, India, ^5^AIDS Healthcare Foundation India, Clinical Services, New Delhi, India, ^6^AIDS Healthcare Foundation, BUREAU CHIEF INDIA, Los Angeles, United States, ^7^AIDS Healthcare Foundation, Global Quality, Amsterdam, Netherlands, the, ^8^AIDS Healthcare Foundation, Clinical Services, Sao Paulo, Brazil

**Background**: AIDS Healthcare Foundation – India implements community based rapid HIV testing across ten states. The objective of the program is early detection and linkage to treatment and to compliment the efforts of National Program in reaching 95‐ 95 ‐95. During COVID lockdown in 2020 large numbers of male migrant labourers from Delhi and Mumbai (destination districts) returned to their native villages (source districts) due to loss of employment during lockdown.

**Description**: This operational study considered four source migration districts from where male migrants would travel to destination districts for employment. The Community based rapid HIV testing data (gender disaggregated) from 2019 to 2021 was analysed. The objective of the study was to compare the HIV sero positivity before and during Covid between men and women and to revisit the risk profile of the newly identified cases.

**Lessons learned**: The results showed that HIV sero‐positivity in 2019, prior to COVID‐19, in the source districts among men and women was 0.8% and 0.6% respectively. During late 2020 and 2021, when the restrictions were eased and testing resumed, the HIV sero prevalence among men was 1.3% and women 1.1%.Based on this evidence a quick profile of the newly identified men and women was analysed, revealing that these women were wives/partners of male migrants who returned from destination districts. Most women (87%) have children and are between 19 – 30 years old. 93% of women had completed primary education only and have no knowledge about HIV.A total of 1055 (93%) of all identified HIV positives were linked to the Government ART centre for treatment and follow‐up. The project initiated partner testing and as a result these women were identified.

**Conclusions/Next steps**: The study recommends increased access to HIV prevention, testing and treatment services to be made available at source district and FOCUS on strategies like Index Testing and Partner testing. The National program needs to re‐engage and re‐design rural interventions for migrants and women, especially for identifying hidden populations and linking them to care and treatment in the context of Covid 19 pandemic.

### PEMOF35 Working apart and together, across and between: lessons learned from an Indigenous and non‐Indigenous organizational partnership in Indigenous harm reduction research during the COVID‐19 pandemic

C. Kendrick ^1^, S. Swann^2^, P. McDougall^2^, R. Masching^1^, Research group‐ Environmental Scan of Community‐Based Harm Reduction Services for Indigenous Peoples in response to the COVID‐19 pandemic

^1^CAAN (Communities, Alliances & Networks), Fort Qu'Appelle, Canada, ^2^Dr. Peter AIDS Foundation, Vancouver, Canada

**Background**: In Canada, Indigenous (First Nation, Inuit and Metis) harm reduction (IHR) programs, policies, and practices center holistic understandings of health and well‐being. Relational care and connections to kin, culture, and community are foundational in responding to disproportionate impacts of HIV, the drug poisoning epidemic, and COVID‐19 on Indigenous peoples. CAAN (Communities, Alliances & Networks) and the Dr. Peter AIDS Foundation (DPAF) have brought together decades of experience in community‐based research, harm reduction and knowledge translation to identify:

1. how IHR programming for Indigenous Peoples has been impacted by COVID‐19,
2. successful adaptations that frontline organizations in providing IHR programming, and
3. resources to address service gaps that impact Indigenous Peoples.

**Description**: To commemorate our partnership, First Nation Elder James Quatell joined the organizations through ceremony. An exchange of copper symbolized our commitment to working towards better health outcomes for Indigenous Peoples. Framed as a one‐year environmental scan, our national project is informed by an iterative and innovative state‐of‐the‐art literature review; sharing circles and interviews with key informants. Our outputs include a Wise Practices Asset Map of culturally responsive IHR services which will form the foundation of new programming co‐led by CAAN and DPAF.

**Lessons learned**: IHR is a critical part of the response to the HIV and Opioid epidemics in Canada. By beginning our partnership with a Copper ceremony, we made an agreement with each other and the communities that we work with, rooted in Indigenous Ways of Knowing and Doing. For our team of Indigenous and non‐Indigenous researchers, this led us to adapt our research processes to mirror the context of working in a global pandemic. This included: rethinking our sequence of data collection events, grappling with virtual dynamics, and staying reflexively attuned to power differences. The results of our work further reflect the need for adaptations.

**Conclusions/Next steps**: Building meaningful research partnerships is allyship in action between Indigenous and non‐Indigenous researchers, service providers, and communities. Together we are leveraging the wisdom and relationships developed over decades of HIV and harm reduction response to strategically contribute to an evolving evidence base, and improve services over time by ensuring they are accessible, people‐centered, and inclusive.

### PEMOF36 Assessing the impact of COVID 19 on the implementation of the hiv workplace policy in (5) organizations

J. Mensah ^1^

^1^African University college of Communication, Development Communication, Accra, Ghana

**Background**: The National HIV and AIDS workplace policy revision in 2012 has been disseminated to most organizations to serve as a guide in the formulation of individual organizational HIV workplace policies and programs. Since the outbreak of COVID 19 epidemic, there has not been a comprehensive study to assess the implementation of these HIV workplace policies in respective organizations. The objective are to create awareness, reduce stigma and discrimination, promote healthy and safe working environment, prevent transmission of HIV and ensure issues at the not relegated to the background amidst COVID 19

**Description**: The study sought to assess the knowledge of employees on their HIV workplace policy and COVID 19 Protocols, the attitudes of employees towards HIV/COVID positive colleagues, and assess the level of implementation of HIV workplace policies in organizations.

A formative research was conducted as the baseline study, and the data collection used was a semi‐structured questionnaire a combination of open‐ended and close –ended. An online response system was created to reach employees that were not at work due to COVID 19 staff rotation policy. Five organizations were to participate in the study. A cross‐section of ten (10) employees comprising managers, senior, and junior staff were sampled to partake in the study. In total fifty (50) employees were interviewed.

**Lessons learned**: The result showed that, 70% of respondent had received updated information about COVID 19 and not HIV in the past 6 months, organizations had visible COVID 19 preventive informative posters and none on HIV at the workplace, 78% of respondent were more comfortable engaging with colleagues who had tested positive to COVID 19 than HIV positive, COVID 19 preventive commodities like hand sanitizers, facemasks were made available to employees with no provision made for HIV preventive commodity like condoms at the workplace, COVID 19 referral centres have been created at the workplace with no referral point for HIV.

**Conclusions/Next steps**:

- The results shows that little importance is given to HIV amidst COVID 19 at the workplace therefore, the Ghana AIDS Commission must strengthen corporate management capacities in implementing HIV and AIDS workplace policies.
- Monitor the implementation of the workplace policy according national guidelines

### PEMOF37 Legal Gender Recognition Policy Briefs (Botswana and Lesotho): A guide for inclusion

A. Mmolai‐Chalmers ^1^

^1^Southern Africa Litigation Centre, Johannesburg, South Africa

**Background**: In 2019, in partnership with LEGABIBO, Matrix, and WILSA, SALC developed policy briefs to inform procedures on legal gender recognition and change of gender markers for Botswana and Lesotho Governments. The two countries laws require official identification documents that reflect one's name and gender identity to access services. The states issued official identification documents are used in daily life including accessing health. Transgender and gender non‐conforming persons are unable to obtain identity documents that match their gender identity and expression.

**Description**: The policy briefs were developed to support Governments with guidance on the interpretation of legal gender recognition. The research was informed by community consultations with members of the transgender in the two countries where participants shared daily experiences on the impacts of not possessing official identification documents. An analysis of international laws and principles that guide legal gender recognition, case lawand domestic laws were reviewed.

**Lessons learned**: Transgender and gender non‐conforming persons are unable to change documents to reflect their gender identity. In Botswana transgender, persons cannot change their gender marker without a court order. When they present identity documents that do not reflect their identity, they experience violence stigma and discrimination. In Lesotho, legal gender recognition is not prohibited, but trans people still face barriers. Legal gender recognition is possible in Botswana because people can change first names and surnames. The National Registration Act allows for particulars to be changed if “material change” has occurred.

**Conclusions/Next steps**: States can takelegislative and administrative steps to ensure that there are procedures for legal gender recognition based on self‐determination. Lesotho legal frameworks recognise gender; therefore, the terms sex/gender should continue to be used interchangeably to accommodate transgender and gender non‐conforming people. In Botswana, the Government must develop regulations and procedures to change the gender marker based on the principles of self‐determination.

## LATE BREAKING ABSTRACTS

### OALBA0102 Immune correlates analysis of the Imbokodo HIV‐1 vaccine efficacy trial

A. Kenny ^1^, A. Luedtke^2^, O. Hyrien^2^, Y. Fong^3^, R. Burnham^2^, J. Heptinstall^4^, S. Sawant^4^, S. Stanfield‐Oakley^4^, F.L. Omar^5^, S. Khuzwayo^5^, O. Dintwe^3,5^, E. Borducchi^6^, L. Pattacini^7^, W. Willems^8^, L. Lavreys^9^, J. van Duijn^7^, D.J. Stieh^7^, F. Tomaka^10^, M.G. Pau^7^, G.E. Gray^11^, S. Buchbinder^12^, K. Mngadi^13^, M.J. McElrath^3^, L. Corey^3^, D.H. Barouch^6^, S.C. De Rosa^3^, G. Ferrari^4^, E. Andersen‐Nissen^3,5^, G. Tomaras^4^, P.B. Gilbert^2^

^1^Department of Biostatistics, University of Washington, Seattle, United States, ^2^Statistical Center for HIV/AIDS Research and Prevention (SCHARP), Fred Hutchinson Research Center, Seattle, United States, ^3^Vaccines and Infectious Disease Division, Fred Hutchinson Research Center, Seattle, United States, ^4^Duke University, Durham, United States, ^5^Cape Town HVTN Immunology Laboratory, Hutchinson Centre Research Institute of South Africa, Cape Town, South Africa, ^6^Center for Virology & Vaccine Research, Beth Israel Deaconess Medical Center, Boston, United States, ^7^Janssen Vaccines Research & Prevention BV, Leiden, Netherlands, the, ^8^Janssen Research & Development BE, Antwerp, Belgium, ^9^Janssen Infectious Diseases BV, Beerse, Belgium, ^10^Janssen Research & Development, LLC, Titusville, United States, ^11^South African Medical Research Council, Cape Town, South Africa, ^12^San Francisco Department of Public Health, San Francisco, United States, ^13^The Aurum Institute, Johannesburg, South Africa

**Background: **In the phase 2b Imbokodo clinical trial (HVTN 705/HPX2008; NCT03060629), the investigational HIV vaccine regimen consisting of a vector‐based vaccine (Ad26.Mos4.HIV) in combination with clade C gp140 protein yielded a month 7‐24 vaccine efficacy (VE) point estimate of 30% (95% CI: –4.0%, 52.9%; p=0.08). Our analyses evaluated immune response markers as correlates of risk (CoR) for HIV‐1 acquisition and protection (impact on VE).

**Methods: **Immune markers were measured in samples from month 7 (4 weeks post‐third vaccination) in a breakthrough case‐control cohort (n=52 cases, 231 non‐cases) from per‐protocol vaccinees (HIV negative through month 7, received first 3 vaccinations within vaccination windows, without major protocol deviations). Binding and functional antibodies were measured in sera by ELISA, BAMA, ADCC, and ADCP; T‐cell functionality was assessed via IFN‐γ ELISpot and ICS. Forty‐one markers (6 primary, 35 exploratory), including IgG and IgG3 magnitude‐breadth and multi‐epitope function scores, were assessed as univariate CoR and correlates of protection for HIV‐1 acquisition over 550 days post‐month 7 via Cox and nonparametric modelling adjusted for baseline prognostic factors. Multivariable CoR were assessed by Cox modelling (primary markers) and machine learning (all markers).

**Results: **The analyses did not support statistically significant CoR (p‐values for multivariable Cox model of primary markers: 0.11‐0.72). There was a consistent trend toward IgG3 V1V2 breadth being associated with decreased HIV‐1 acquisition, suggesting an immune correlate with a hazard ratio of 0.51 (p=0.11) in the multivariable analysis and 0.67 (p=0.24) in the univariable analysis per 10‐fold increase, and increased VE with this marker (Figure). VE mediated through this marker was 25.3% (95% CI: 6.2%, 40.5%). The preclinical correlate combining ELISA and ELISpot responses was not recapitulated in this clinical trial.

**Figure:**
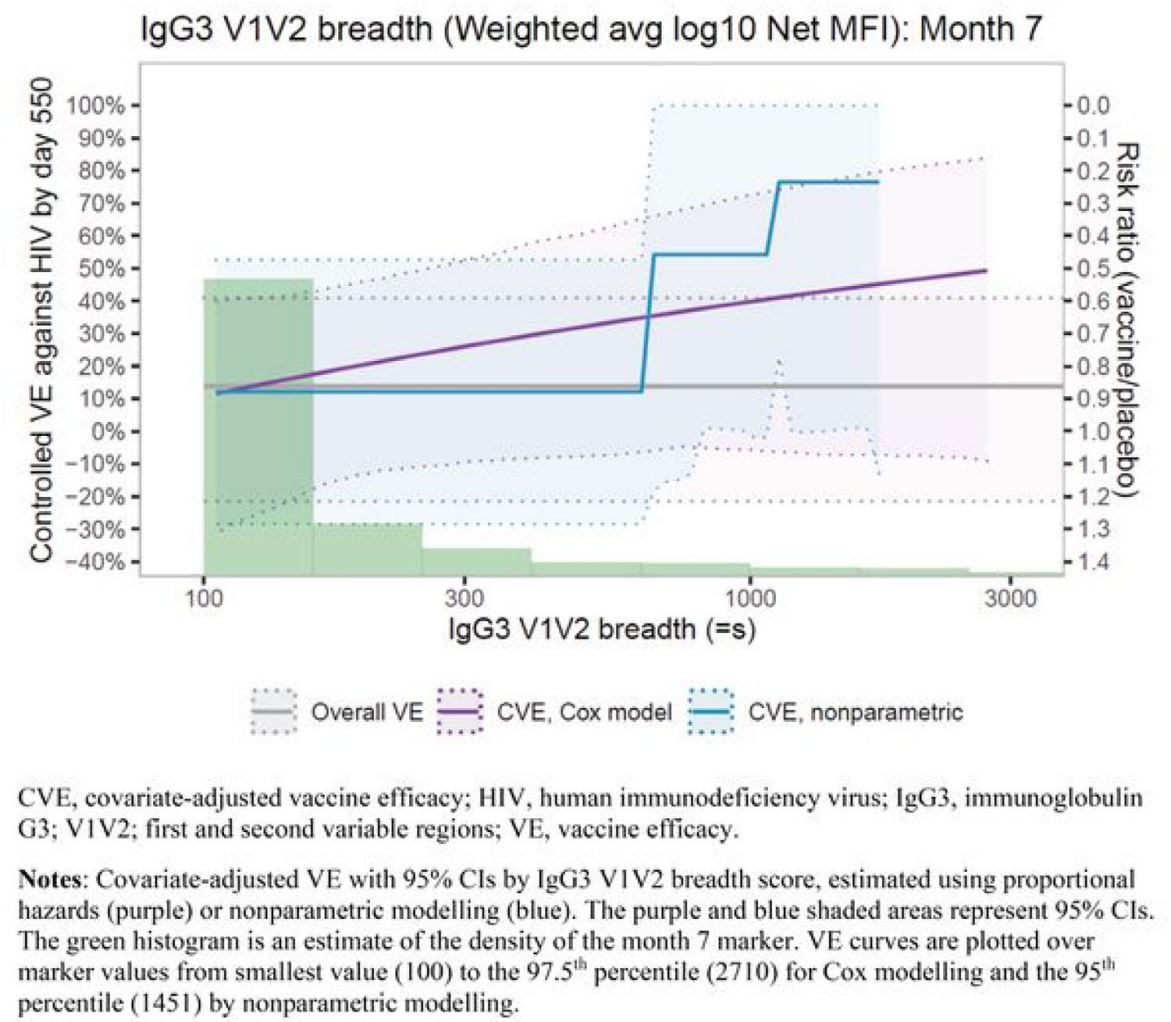
**Abstract OALBA0102‐Figure 1**.

**Conclusions: **Multiple statistical analyses revealed a trend of high IgG3 V1V2 BAMA breadth scores associated with lower infection risk and partial vaccine protection.

### OALBA0103 Flt3 agonist enhances immunogenicity of arenavirus‐vector based vaccines in macaques

A. Boopathy^1^, A. Nekkalapudi^1^, B. Sharma^1^, S. Schulha^2^, R. Wimmer^2^, D. Jin^2^, J. Sung^1^, J. Murry^1^, M. Nagel^1^, B. Carr^1^, S. Ahmadi‐Erber^2^, H. Lauterbach^2^, K. Orlinger^2^, T. Makadzange^1^, M. Kuhne^1^, R. Geleziunas^1^, B. Falkard ^1^, S. Schmidt^2^

^1^Gilead Sciences, Foster City, United States, ^2^Hookipa Pharma, New York, United States

**Background: **Strong virus specific CD8 T cell responses are associated with viral control, in both non‐human primate models of SIV infection and in HIV infected elite controllers. Arenavirus vectors elicit potent antigen specific CD8 T cell responses by infecting and activating dendritic cells (DC) and other antigen presenting cells. In clinical oncology studies, Flt3 receptor activation expands peripheral DCs. Here, we evaluate the potential of Flt3 receptor agonism to enhance immunogenicity of an arenavirus‐based alternating two‐vector SIV vaccine in rhesus macaques.

**Methods: **Healthy rhesus macaques (n=13/group) were immunized with attenuated replicating arenavirus‐based vectors, with artificial genome organization, i.e. artPICV (Pichinde Virus) and artLCMV (Lymphocytic choriomeningitis virus) in alternating sequence. Both vectors encode SIVsmE543 Gag, Env and Pol immunogens and were administered alone (Vaccine vectors on weeks 0, 4, 8, 12) or in combination with Flt3 agonist (Flt3 ligand‐Fc fusion protein dosed on weeks ‐1, 3, 7, 11). Vaccine immunogenicity was assessed by SIV‐specific IFNγ ELISpot, using SIV peptide sub‐pools for cellular breadth, and Env‐binding antibodies by ELISA. SIV‐specific T cell polyfunctionality, activation of T cells, DCs and innate immune cells was evaluated by multi‐parameter flow cytometry.

**Results: **No safety concerns or drug accumulation was observed with repeated Flt3 agonist dosing. In combination with Flt3 agonist, the alternating prime‐boost dosing with artPICV/artLCMV resulted in a robust increase of conventional type 1 DCs (14‐fold, p<0.001) and activated monocytes (2‐fold, p<0.01) over baseline. Combination treatment significantly increased SIV‐specific IFNγ T cell responses (8‐fold, p<0.0001), expansion of SIV‐specific cellular breadth (9‐fold, p<0.0001), T cell activation (2‐fold, p<0.001), and SIV Env‐binding antibodies (3‐fold, p<0.0001) after the 4 vaccine doses. Importantly, Flt3 agonism significantly augmented vaccine induced peak polyfunctional SIV‐specific CD4 and CD8 T cells (by 2‐4‐fold, p<0.05) that express IFNγ, TNFα and IL‐2 after each vaccine dose.

**Conclusions: **Flt3 agonism is a novel immunomodulatory strategy that augments the magnitude, breadth, and polyfunctionality of SIV‐specific T cell responses and SIV‐specific antibody responses induced by arenavirus vaccination with a potential to be part of a combination therapeutic approach for HIV cure.

### OALBA0105 Metabolomic and lipidomic correlates of time‐to‐HIV‐rebound in viremic controllers treated with vesatolimod

Y. Cai ^1^, S. Deeks^2^, L. Zhang^3^, L. Giron^4^, K.Y. Hong^4^, A. Goldman^4^, L. Selzer^3^, L. VanderVeen^3^, S. Guo^3^, C. de Vries^3^, E. Vendrame^3^, D. SenGupta^3^, M. Abdel‐Mohsen^4^, J. Wallin^3^

^1^Gilead Sciences, Biomarker, Foster City, United States, ^2^UC San Francisco, San Francisco, United States, ^3^Gilead Sciences, Foster City, United States, ^4^The Wistar Institute, Philadelphia, United States

**Background: **Metabolites andlipids are biologically active molecules involved in several cellular processes and immunological functions. Recently, several metabolites and lipids were suggested as potential correlates of time to HIV rebound (TTHR) post‐antiretroviral therapy (ART) interruption in chronically‐infected people living with HIV. We examined plasma metabolite and lipid profiles in a placebo‐controlled Phase 1b study of the TLR7 agonist vesatolimod (VES) in ART‐suppressed viremic controllers.

**Methods: **We enrolled 25 ART‐suppressed HIV viremic controllers [pre‐ART plasma viral load (pVL) 50‐5000 copies/mL]. Seventeen participants received ten biweekly doses of VES, and eight received placebo, followed by an analytical treatment interruption (ATI) phase for up to 48 weeks. pVL was measured by qPCR, proviral HIV DNA was measured by the intact HIV proviral DNA (IPDA) assay, and plasma metabolites and lipids were measured by HPLC‐MS/MS. Baseline (predose) levels of metabolites and lipids were used to determine associations with time to pVL of 200 or 1000 copies/mL, or the duration of HIV control below 400 copies/mL during ATI, using the cox proportional hazard model and Spearman's correlations. Data collected at 1‐day after VES dose 10 were used to evaluate the effect of VES. Wilcoxon Signed‐Rank test and Rank‐Sum were used to compare between timepoints and populations.

**Results: **VES treatment resulted in induction of pro‐inflammatory tryptophan metabolism, increased protein‐synthesis‐related aminoacyl‐tRNA biosynthesis, and enhanced the phosphatidylinositol signaling pathway. At baseline, glycoursodeoxycholic acid, some ceramide lipid groups, and cholesterol ester (ChE 20:2) were associated with longer TTHR and larger reduction in IPDA; while phosphatidylcholine (18:3_18:3), and phosphatidylethanolamine (19:1_18:1) were associated with shorter TTHR. Pathways associated with TTHR or changes in IPDA included several pathways which were previously reported to be associated with delayed HIV rebound, such as the Glutamine/Glutamate and Histidine metabolic pathways, and arginine biosynthesis. Additional pathways found in this study included beta‐Alanine metabolism and pantothenate and CoA biosynthesis, which were associated with accelerated immune recovery.

**Conclusions: **This exploratory analysis highlights potential metabolic and lipidomic changes mediated by VES treatment and/or associated with viral control post‐ART‐cessation in HIV viremic controllers. These findings warrant further investigations in larger independent cohorts.

### OALBB0102 Phase I/II study of monoclonal antibody VRC01 with early antiretroviral therapy to promote clearance of HIV‐1 infected cells in infants (IMPAACT 2008)

A. Khaitan ^1^, J. Lindsey^2^, E. Capparelli^3^, C. Tierney^2^, A. Coletti^4^, C. Perlowski^4^, M. Cotton^5^, D. Yin^6^, S. Majji^7^, J. Moye^7^, H. Spiegel^6^, P. Harding^8^, D. Costello^9^, C. Krotje^10^, L. Gama^11^, D. Persaud^12^, E. McFarland^8^, IMPAACT 2008 Protocol Team

^1^Indiana University School of Medicine, Pediatric Infectious Diseases, Indianapolis, United States, ^2^Harvard TH Chan School of Public Health, Center for Biostatistics in AIDS Research, Boston, United States, ^3^University of California San Diego, Pediatric Host‐Microbe Systems and Therapeutics, San Diego, United States, ^4^IMPAACT Operations Center, Durham, United States, ^5^FAMCRU, Stellenbosch University, Department Paediatrics and Child Health, Cape Town, South Africa, ^6^National Institutes of Health, Division of Aids, Rockville, United States, ^7^National Institutes of Health, Eunice Kennedy Shriver National Institute of Child Health and Human Development, Bethesda, United States, ^8^University of Colorado School of Medicine, Pediatric Infectious Diseases, Aurora, United States, ^9^University of California at Los Angeles, MacDonald Research Laboratory, Los Angeles, United States, ^10^Frontier Science & Technology Research Foundation, Amherst, United States, ^11^National Institutes of Health, National Institute of Allergy and Infectious Diseases, Vaccine Research Center, Bethesda, United States, ^12^John Hopkins University, Department of Pediatric Infectious Diseases, Baltimore, United States

**Background: **VRC01 is a broadly neutralizing antibody (bNAb) targeting CD4 binding sites with demonstrated anti‐HIV‐1 activity in adults. Safety and efficacy in infants living with HIV‐1 are unknown.

**Methods: **Infants, age <12 weeks initiating antiretroviral therapy (ART), were randomized to four doses (Weeks 0, 2, 6, 10) of open‐label, subcutaneous VRC01 (VRC01) at 40mg/kg or no VRC01 (No‐VRC01). Follow‐up was 48 weeks, with primary safety and efficacy outcomes assessed at Week 14. Laboratory testing included VRC01 plasma troughs, droplet digital PCR for HIV‐1 DNA and Genosure® MG and PhenoSense® Neutralization assays for ART and VRC01 resistance.

**Results: **Infants enrolled (30 to VRC01; 31 to No‐VRC01) between April 2019‐March 2020 in Malawi, Botswana, Zimbabwe, and Brazil; 84% Black non‐Hispanic, 57% female. Baseline characteristics were (VRC01 vs No‐VRC01): median age (72 vs 73 days), log_10_ plasma HIV‐1 RNA (4.10 vs 4.35 copies/mL), and log_10_cellular HIV‐1 DNA (3.12 vs 3.16 copies/million PBMCs). Initial ART included nevirapine (53% of VRC01; 29% of No‐VRC01) or lopinavir/ritonavir. Baseline ART resistance was detected in 44% (VRC01) and 33% (No‐VRC01) of infants, mostly NNRTI. Baseline resistance to VRC01 (IC50 >50mcg/mL) was detected in 5/17 (29%) infants receiving VRC01. All VRC01 doses were administered. Local injection reactions (all Grade ≤2) occurred in ≥90% of infants. Adverse events Grade >3 (none attributed to VRC01) through Week 14 occurred in 40% of VRC01 (95% CI:23%, 59%) and 47% of No‐VRC01 (95% CI:28%, 66%), and most were anemia, neutropenia and gastrointestinal disorders. Median (Q1, Q3) VRC01 plasma trough was 83.1 (36.1, 111.8) mcg/mL, however 31% were <50mcg/mL. No VRC01 anti‐drug antibodies were detected. HIV‐1 DNA log_10_ copies/million PBMCs median (Q1, Q3) declines from Week 0‐14 were 0.41 (0.30, 0.56) in VRC01 and 0.53 (0.33, 0.70) in No‐VRC01 (Wilcoxon p=0.42).

**Conclusions: **Subcutaneous VRC01 was feasible and no safety concerns were observed in this first treatment study in infants living with HIV‐1. HIV‐1 DNA declines did not differ by treatment arm, however ART and VRC01 resistance and VRC01 troughs <50 mcg/mL may have lessened VRC01 effectiveness. Further studies are needed to determine optimal approaches with more potent bNAbs for early treatment of perinatal HIV infection.

### OALBB0103 Enhanced tuberculosis screening using computer‐aided X‐ray diagnosis and novel point of care urine lipoarabinomannan assay among adults with HIV admitted to hospital (CASTLE study): a cluster randomised trial

R. Burke ^1^, S. Nyirenda^2^, H. Twabi^3,1^, M. Nliwasa^3^, E. Joekes^4^, N. Walker^4^, R. Nyirenda^5^, A. Gupta‐Wright^6^, K. Fielding^7^, P. MacPherson^4^, E. Corbett^6^

^1^Malawi Liverpool Wellcome, Blantyre, Malawi, ^2^Government of Malawi, Zomba Central Hospital, Zomba, Malawi, ^3^Kamuzu University of Health Sciences, Helse Nord Tuberculosis Initiative, Blantyre, Malawi, ^4^Liverpool School of Tropical Medicine, Liverpool, United Kingdom, ^5^Government of Malawi, Department of HIV/AIDS, Lilongwe, Malawi, ^6^London School of Hygiene and Tropical Medicine, Faculty of Infectious and Tropical Disease, London, United Kingdom, ^7^London School of Hygiene and Tropical Medicine, Faculty of Epidemiology and Population Health, London, United Kingdom

**Background: **People living with HIV (PLHIV) have high mortality if admitted to hospital.Tuberculosis (TB) is a major cause of death, reflecting diagnostic challenges.This randomised trial investigated whether screening for TB using high‐sensitivity urine lipoarabinomannan (LAM) testing and digital chest Xray with computer aided diagnosis (dCXR‐CAD) could improve TB diagnosis and reduce mortality.

**Methods: **We conducted an unblinded cluster randomised trial among adult PLHIV admitted to medical wards at Zomba Central Hospital, Malawi, with admission‐day the unit of randomisation. Admission‐days were randomly assigned to: enhanced TB diagnostics using high sensitivity urine LAM (SILVAMP‐LAM, Fujicorp, Japan), dCXR‐CAD (CAD4TBv.6, Delft, Netherlands: provides score 0 to 100 with higher scores more likely to be TB) plus usual care; or usual care alone. The primary outcome was TB treatment initiation during admission. Secondary outcomes were 56‐day mortality, TB diagnosis within 24‐hours and microbiologically‐confirmed undiagnosed TB at discharge. Trial registration NCT04545164.

**Results: **Between 2/September/2020 and 15/February/2022, 415 adults in 207 clusters were included in intention‐to‐treat analysis.At admission, 90.8% (377/415) were taking ART and median CD4 cell count was 249 cells/mm^3^ (IQR 124 ‐ 440). In the enhanced diagnostic arm, the median CAD4TBv6 score among participants was 60 (IQR: 51 ‐ 71), and 4% (9/207) had SILVAMP‐LAM‐positive urine. TB treatment was initiated in 46/208 (22%) in the enhanced TB diagnostics arm and 24/207 (12%) in the usual care arm (risk ratio [RR] 1.93 [95% CI 1.21‐3.08]). There was no difference in mortality by 56 days (enhanced TB diagnosis: 54/207 [26%]; usual care: 52/207 [25%]; hazard ratio 1.05, [95% CI 0.72–1.53]) or TB treatment initiation within 24 hours (enhanced TB diagnosis: 8/207 [3.9%]; usual care: 5/208 [2.4%]; RR 1.61 [95% CI 0.53–4.71]). Undiagnosed TB at discharge based on sputum culture was 0/207 (0.0%) and 2/208 (1.0%) enhanced TB diagnostics and usual care arms, respectively (RR not estimated).

**Conclusions: **High sensitivity urine LAM plus dCXR‐CAD screening led to more hospitalised PLHIV initiating TB treatment, but did not reduce mortality. Better understanding of the causes of death and additional interventions are required to improve clinical outcomes for people with HIV admitted to hospital.

### OALBB0104 The “City of Hope” Patient: prolonged HIV‐1 remission without antiretrovirals (ART) after allogeneic hematopoietic stem cell transplantation (aHCT) of CCR5‐Δ32/Δ32 donor cells for acute myelogenous leukemia (AML)

J. Dickter ^1^, S. Weibel^2^, A. Cardoso^3^, S. Li^3^, K. Gendzekhadze^4^, Y. Feng^5^, S. Dadwal^1^, R. Taplitz^1^, J. Ross^6^, A. Aribi^4^, R. Stan^7^, T. Kidambi^1^, L. Lai^8^, S. Chang^9^, A. Chaillon^2^, M. Al Malki^4^, J. Alvarnas^4^, S. Forman^4^, J. Zaia^3^

^1^City of Hope, Medicine, Duarte, United States, ^2^University of California, San Diego, Medicine, La Jolla, United States, ^3^City of Hope, Center for Gene Therapy, Duarte, United States, ^4^City of Hope, Hematology and Hematopoietic Cell Transplantation, Duarte, United States, ^5^City of Hope, Medical Oncology and Therapeutics Research, Duarte, United States, ^6^City of Hope, Department of Pharmacy Services, Duarte, United States, ^7^City of Hope, Translational Development Center, Duarte, United States, ^8^City of Hope, Surgery, Duarte, United States, ^9^City of Hope, Pathology, Duarte, United States

**Background: **HIV‐1 remission has been described post aHCT. We report a 66‐year‐old Caucasian male, diagnosed with HIV‐1 in 1988 (CD4 nadir <100 cells/uL), undetectable HIV‐1 viral load on ART since 1990s. In 2018, he developed AML, treated with chemotherapy followed by aHCT from unrelated HLA‐matched CCR5‐Δ32 homozygous donor. He continued emtricitabine/tenofovir alafenamide/dolutegravir 25 months (m) post‐aHCT. After analytic treatment interruption (ATI), he remains in HIV‐1 remission.

**Methods: **3/2019‐current, City of Hope.

Pre‐aHCT: HIV‐1 DNA quantification, sequencing of genotypic tropism. Post‐aHCT: blood, intestinal biopsies were obtained for cellular HIV DNA, RNA (by droplet digital PCR); compartmental testing for donor cells; ART levels; HIV‐1 antibody quantification; peripheral blood mononuclear cells (PBMC) challenged with HIV‐1; HIV, CMV T‐cell responses.

**Results: **Pre‐aHCT: 80 HIV‐1 DNA copies/million PBMC, majority R5 tropic virus (10% false‐positive rate). Post‐aHCT, 14m post‐ATI: 100% donor chimerism.

Post‐aHCT: HIV‐1 RNA undetectable (< 20 copies/mL), sporadic (low‐level) detectable cellular HIV‐1 DNA, RNA in PBMC, gut tissue:

**Table:** 

**Abstract OALB0104‐Table 1**.			
Date post‐aHCT months (m)	RNA: msTatRev copies/1 million CD4+ T cells	RNA: skGag copies/1 million CD4+ T cells	DNA: skGag copies/1 million CD4+ T cells
+ 2m PBMC	0	35	0.00
+ 3 months PBMC	0	0	0.00
+ 6m PBMC Gut biopsy	0	0	0.00 4.22
+ 10m PBMC	11.4	0	0.00
+ 13m PBMC Gut biopsy	0	0	0.00 0.00
+18m PBMC	21.9	0	0.00
+ 24m PBMC	0	0	0.00
+ 30m PBMC (5 months post‐ATI)	0	0	0.00
+ 37m PBMC Gut biopsy (12m post‐ATI)	0	0	0.00 0.00

ART levels unremarkable at 7m, 12m post‐ATI. We observed declining HIV‐1 specific humoral, no detectable HIV‐specific cellular immune response. Participant's CD8‐depleted PBMC remained uninfected after ex vivo challenge with HIV R5 strains. Immunological studies 37m post‐aHCT, 12m post‐ATI: viral recall antigen analysis showed robust response to CMV stimulation, no response to HIV (CD4, CD8 T‐cells).

**Figure:**
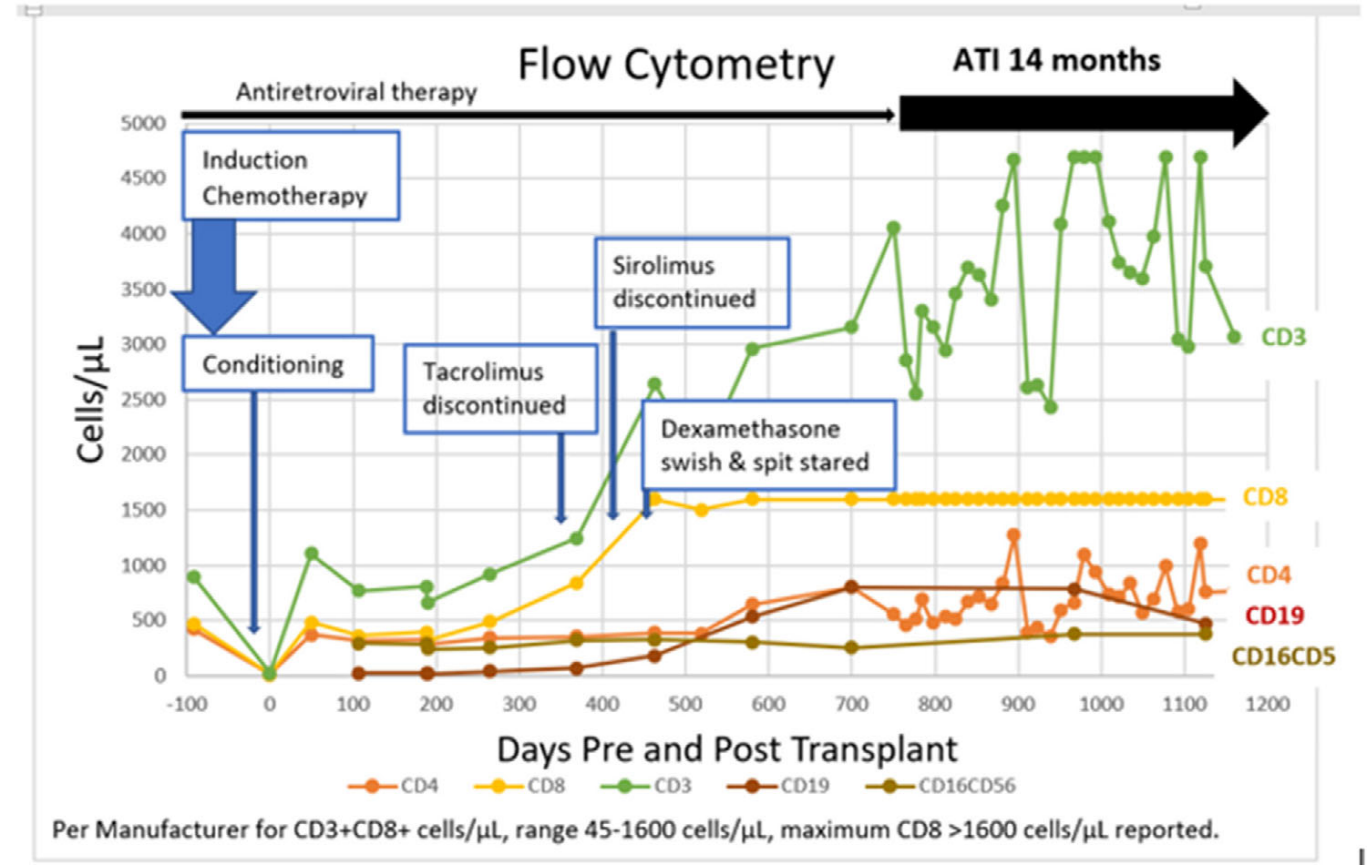
**Abstract OALBB0104‐Figure 1**.

**Conclusions: **We report an individual with HIV‐1 transplanted for AML with CCR5‐Δ32/Δ32 donor cells who at 14m post‐ATI, 39m post‐aHCT, has no evidence of HIV‐1 RNA rebound and no detectable HIV‐1 DNA. HIV cure is feasible post‐aHCT as described here and in previously described reports.

### OALBB0105 Incident tuberculosis as a risk factor for viral non‐suppression 48 weeks among patients switched to dolutegravir based therapy with recycled nucleoside reverse transcriptase inhibitors in Lusaka, Zambia

N. Mbewe ^1^, D. Engamba^1^, S. Fwoloshi^1^, L. Mulenga^2^, S. Lakhi^1^

^1^University of Zambia, Department of Internal Medicine,, Lusaka, Zambia, ^2^Ministry of Health,, Ndeke House, Lusaka, Zambia

**Background: **Dolutegravir (DTG) based therapy has been hailed as the missing key to quickly curb the HIV epidemic in resource‐limited settings. Inadequate viral load testing had slowed the rate at which individuals currently on nucleoside reverse transcriptase inhibitors (NRTI)‐based regimens could be transitioned to DTG based therapy. However, as evidence emerges on the utility of recycled NRTIs, it remains to be known which factors could contribute to viral non suppression on Dolutegravir based therapy.

**Methods: **We conducted a retrospective sub‐analysis for individuals enrolled in the VISEND study at the University Teaching Hospital in Lusaka, Zambia; with a baseline viral load >1000 copies/ml at the time of the switch and analysed virological outcomes at 48weeks post transition. Data on patient demographics, duration of previous antiretroviral therapy, and occurrence of opportunistic infections. Descriptive statistics were computed using STATA version 13. Viral suppression rates, Incidence rate ratios, and Multivariate logistic regressions for common factors associated with treatment failure were computed

**Results: **786 records were screened, with 675 individuals with baseline viral load >1000copies/ml whilst on Tenofovir + Lamivudine + Efavirenz (TLE) meeting eligibility. 375 individuals were transitioned to Tenofovir Disoproxil Fumarate + Lamivudine + Dolutegravir (TLD) or Tenofovir Alafenamide +Emtricitabine + Dolutegravir (TafED) and 300 were to Zidovudine + Lamivudine + boosted Lopinavir or Atazanavir (AZT/3TC/LPV‐r or ATV‐r). Viral suppression rate was 87.9% on PI‐based therapy versus 94.7% on TLD/TafED at 48 weeks (p‐value = 0.002). There was a three‐fold higher chance of virological failure with a TB event during the 48 weeks (p = 0.022) but no significant difference in TB outcomes between the groups nor any other significant factors associated with virological non suppression.

**Table:** 

**Abstract OALBB0105‐Table 1**.			
Variable	Crude OR[95% CI]	Adjusted OR[95% CI]	P value
**Male Gender**	2.14 (1.3 ‐3.69)	1.95 (1.08 ‐3.53)	0.027
**Extremes of age (≤20 or≥60)**	1.71 (0.87 ‐3.38)	1.55 (0.77 – 3.10)	0.215
**TB Event**	**3.19 (1.13‐ 8.93)**	**3.46 (1.19 ‐10.01)**	**0.022**
**Baseline BMI**	0.94 (0.88 ‐1.01)	0.98 (0.91‐1.04)	0.533
**Missed visit(s)**	0.95 (0.44 ‐2.07)	0.98 (0.44 ‐2.16)	0.914

**Conclusions: **The results of this study emphasise the need for thorough screening for TB for patients being transitioned to DTG based therapy with recycled NRTI backbone and the need for prospective follow‐up studies to establish utility of newer molecules such as Tenofovir Alafenamide/Emtricitabine/Dolutegravir with antituberculous therapy.

### OALBC0102 High prevalence of asymptomatic Omicron carriage and correlation with CD4^+^ T cell count among adults with HIV enrolling in COVPN 3008 Ubuntu clinical trial in sub‐Saharan Africa

A. Tapley^1,2^, J. Andriesen ^2^, L. Fisher^2^, Y. Huang^2,3^, N. Ketter^2^, M. Villaran^2^, P. Gilbert^2^, J. Hural^2^, M. Yacovone^4^, L.‐G. Bekker^5^, L. Corey^2^, G. Gray^6^, J. Makhema^7^, H. Nuwagaba‐Biribonwoha^8^, T. Samandari^9^, P. Elyanu^10^, R. Chilengi^11^, Z. Chirenje^12^, S. Dadabhai^13^, N. Mgodi^12^, P. Kotze^14^, N. Garrett^15^, CoVPN 3008 Ubuntu study team

^1^University of Washington, Division of Allergy & Infectious Diseases, Department of Medicine, Seattle, United States, ^2^Fred Hutchinson Cancer Center, Vaccine and Infectious Disease Division, Seattle, United States, ^3^University of Washington, Department of Global Health, Seattle, United States, ^4^National Institutes of Health, National Institute of Allergy and Infectious Diseases, Bethesda, United States, ^5^Desmond Tutu HIV Centre, University of Cape Town, Cape Town, South Africa, ^6^South African Medical Research Council, Pretoria, South Africa, ^7^Botswana Harvard AIDS Institute, Gaborone, Botswana, ^8^Eswatini Prevent Center, Mbabane, Eswatini, ^9^Kisumu CRS/KEMRI‐CDC, Kisumu, Kenya, ^10^Baylor‐Uganda CRS, Kampala, Uganda, ^11^Matero CRS, Lusaka, Zambia, ^12^University of Zimbabwe Clinical Trials Research Centre, Harare, Zimbabwe, ^13^Blantyre CRS, Blantyre, Malawi, ^14^Qhakaza Mbokodo Research Clinic, Ladysmith, South Africa, ^15^Centre for the AIDS Programme of Research in South Africa, University of KwaZulu–Natal, Durban, South Africa

**Background: **The COVID‐19 wave driven by the SARS‐CoV‐2 Omicron variant prompted the need to explore asymptomatic carriage among HIV‐immunocompromised adults.

**Methods: **In the trial we are assessing COVID‐19 mRNA‐1273 vaccine efficacy in persons with HIV (PWH) or another COVID‐19‐associated comorbidity across 7 sub‐Saharan African countries. Previously vaccinated persons were excluded. Baseline testing included HIV screening, CD4^+^ T‐cell count and HIV viral load (if HIV^+^), anti‐SARS‐CoV‐2 antibodies, and nasal swab SARS‐CoV‐2 reverse‐transcriptase polymerase chain reaction (RT‐PCR). Participants had to be without COVID‐19 signs/symptoms to be vaccinated at enrollment. Here we examine December 2021‐April 2022 data to characterize asymptomatic SARS‐CoV‐2 infections and assessed correlation with CD4 count.

**Results: **6397 adults, including 4437 PWH, were enrolled (median age: 38 years; female: 75%). Baseline nasal swab data were available for 5772/6397 (90.2%). 336/5772 (6%) had asymptomatic SARS‐CoV‐2 infection, more frequent among SARS‐CoV‐2 seronegative than seropositive participants (9% vs 4%, p<0.001). Infection was detected among 98/1463 (7%) of PWH with a CD4 count<500 cells/mm^3^ vs 152/2974 (5%) with counts ≥500 cells/mm^3^ (p=0.037), an association irrespective of SARS‐CoV‐2 serostatus. A 10‐fold CD4 decrease corresponded to 1.72‐fold higher odds of PCR positivity, adjusting for serostatus, sex, and non‐linear temporal trends (95% confidence interval [CI]:1.09‐2.72‐fold higher, p=0.019, **Figure 1**). Over time, the adjusted odds of PCR positivity were highest during the Omicron surge in December 2021 and 44% lower in men than women (95% CI: 17%‐62% lower, p=0.004). Gene sequencing on a subset confirmed Omicron.

**Figure:**
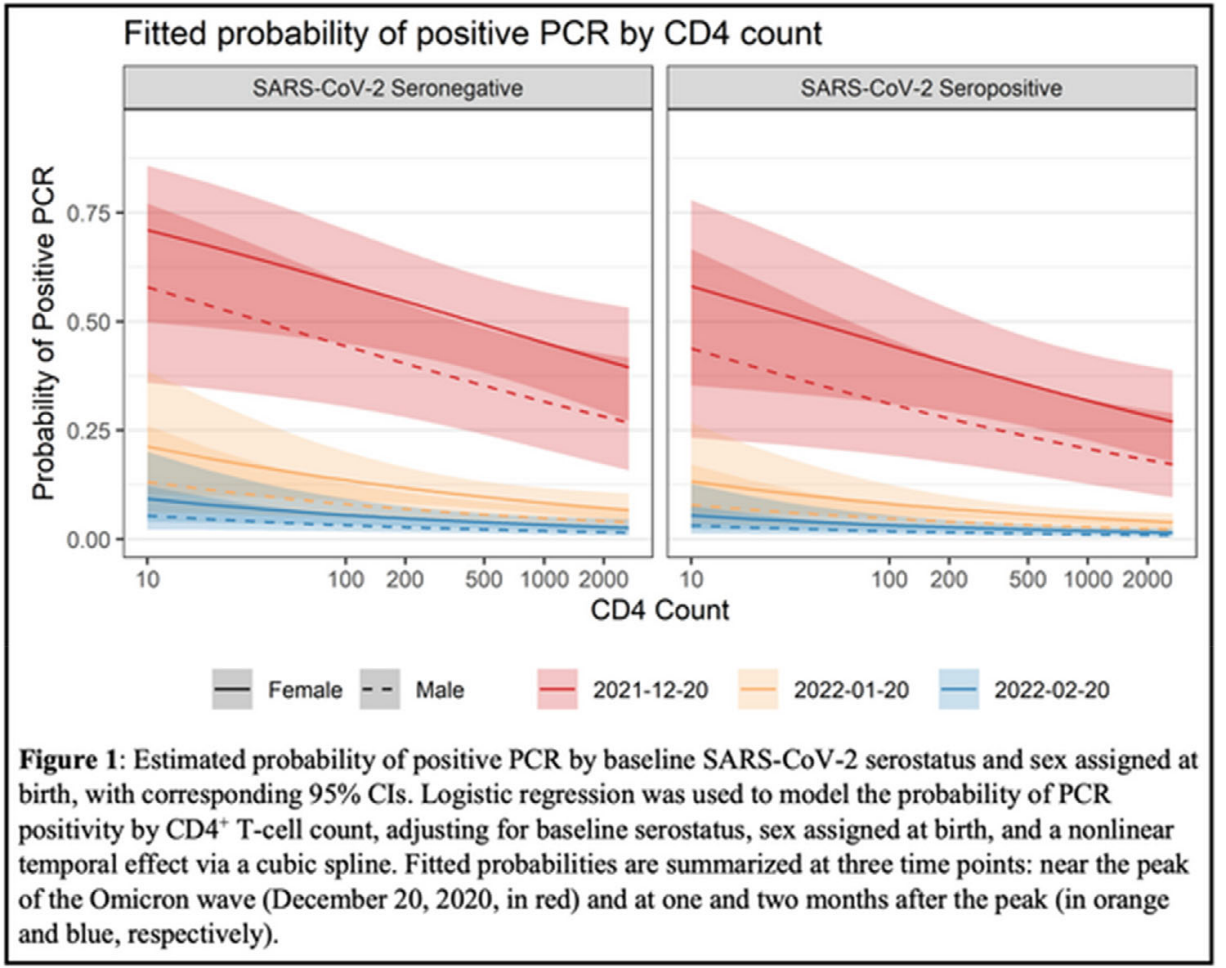
**Abstract OALBC0102‐Figure 1**.

**Conclusions: **Our study of the largest cohort of PWH in a COVID‐19 vaccine clinical trial to date reports the asymptomatic SARS‐CoV‐2 carriage rate was 3‐6‐fold higher than COVID‐19 vaccine trials before Omicron. Additionally, lower CD4 count in PWH strongly correlated with increased odds of SARS‐CoV‐2 PCR positivity. These data highlight the urgent need for larger studies to better characterize how HIV‐associated immunocompromise influences infection acquisition/clearance.

### OALBC0103 A phase IV open‐label evaluation of safety and tolerability of coformulated bictegravir/emtricitabine/tenofovir alafenamide for post‐exposure prophylaxis following potential exposure to HIV‐1

A. Liu^1^, R. Xin^2^, H. Zhang^1^, L. Dai^1^, R.E. Wu^3^, X. Wang^1^, A. Li^1^, H. Wei^1^, J. Li^1^, Y. Shao^1^, Y. Gao^1^, Z. Wang^1^, J. Ye^1^, G. A bu dou re xi ti^4^, Z. Li^1^, L. Sun ^1^

^1^Beijing Youan Hospital, Capital Medical University, Beijing, China, ^2^Beijing Center for Disease Prevention and Control, Beijing, China, ^3^Colgate University, Hamilton, United States, ^4^The eighth Affiliated Hospital of Xinjiang Medical University, Urumqi, China

**Background: **Single‐tablet regimen provides a convenient once‐daily regimen for the prevention of HIV infection. Here, we investigated the adherence and safety of co‐formulated bictegravir/emtricitabine/tenofovir alafenamide (BIC/FTC/TAF) as a 3‐drug, single‐tablet regimen for post‐exposure prophylaxis (PEP) in China.

**Methods: **This was a prospective, open‐label, single‐arm trial conducted in an STD/AIDS clinic of a tertiary hospital in Beijing, from May 2021 to February 2022. Adults requiring PEP were prescribed BIC/FTC/TAF one pill once a day for 28 days. Clinical and laboratory data were collected and analyzed at baseline, weeks 2, 4, 8, 12, and 24.

**Results: **Of 112 participants enrolled in the study, 109 (97.3%) were male and the mean age was 30±8 years. PEP completion was 96.4% (95% CI: 91.1‐99.0). Two participants stopped PEP after 2 days because the source partner was identified as HIV uninfected. One participant was excluded due to hepatitis B virus infection according to the exclusion criteria. One discontinued due to the participant's decision. No participant acquired HIV through week 24. Adherence was 98.9% (standard deviation [SD], 3.3) by self‐reporting and 98.5% (SD, 3.5) by pill count. Only 5 participants experienced mild clinical adverse events attributed to study drug (including headache, diarrhea, and nausea) and 4 participants had elevated serum creatinine (grade 1).

**Conclusions: **A once daily, single‐tablet regimen of BIC/FTC/TAF used as PEP was safe and well‐tolerated with a high rate of completion and adherence. BIC/FTC/TAF may be a good option for PEP.

### OALBC0104 National roll out of community HIV screening among key populations in indonesia: assessment of early results

B. Utomo ^1^, R. Kurniawan^1^, I. Trihandini^1^, K.N. Siregar^1^, S. Rahayu^1^, R.J. BaharuddinNur^1^, P. Loh^2^, A.I. Sirait^2^, I.A. Tasya^2^, R.J. Magnani^2,3^

^1^Universitas Indonesia, Department of Biostatistics and Population Studies, Depok, Indonesia, ^2^PATH, Seattle, United States, ^3^Universitas Indonesia, Faculty of Public Health, Depok, Indonesia

**Background: **Although progress has been made, Indonesia remains far from achieving the first “95” of the UNAIDS 95‐95‐95 framework with only an estimated 43% of PLHIV knowing their HIV status in 2019. In response, the Indonesian Ministry of Health has revised its HIV testing guidelines to permit community‐based HIV screening/self‐testing using oral fluid rapid tests. National roll out of the new strategy began in late 2021. This study assesses the early results in terms of community response, impact on HIV testing coverage, and linkage with treatment.

**Methods: **Test kits distribution and outcome data, including linkage with confirmatory testing and as needed with treatment, were compiled from four MoH implementing partners covering the October 2021 – March 2022 period. Distribution data were analyzed in terms of distribution models and modalities, as well as in terms of the characteristics of persons engaging in community screening. Testing‐to‐treatment initiation cascades were constructed both in the aggregate and for key population sub‐groups.

**Results: **A total of 34,981 valid community screening tests had been performed by April 2022. Hotspot distribution of test kits for assisted screening was the most common distribution mechanism (76.7% of all test kits distributed) followed by workplace distribution with assisted screening (8.4%). 95.2% of persons undergoing community screening were male, reflecting a late start in distribution to female sex workers. Of the persons screened, 75.8% were MSM, 14.3% were male employees of strategically chosen companies, 3.9% were transgendered females, 4.7% person who inject drugs, 0.9% female sex workers, and 0.3% others. 98.7% were first‐time HIV testers. The HIV positivity rate in community screening was 3.6% (5.1% among transgendered females). Among persons with reactive community screening tests, 42.4% had received a confirmatory test at a health facility, and among those with reactive confirmatory tests 50% had initiated ART. The rates of both confirmatory testing and treatment initiation were especially low among FSW and male PWID.

**Conclusions: **Community‐based HIV screening is reaching previously under‐served segments of HIV key populations as evidenced by the high first‐time tester rates. However, linkage with confirmatory HIV testing is low, as is linkage between confirmatory testing and treatment initiation.

### OALBC0105 Predictors of high viral load among adult HIV recipients of care in Zambia, 2021: a cross‐sectional analysis of HIV case‐based surveillance data

S. Nyimbili ^1^, M. Kalubula^2^, S. Bosomprah^3^, P. Somwe^4^, J. Mutale^4^, M. Lumpa^4^, J. Pry^1^, D. Mwamba^4^, B. Hanunka^5^, K. Mweebo^5^, M. Mudenda^5^, P. Funsani^2^, C. Sianyinda^2^, T. Sinkala^1^, M. Mwansa^2^, S. Sivile^2^, L. Tally^5^, P. Minchella^5^, M. Wa Mwanza^1^, T. Savory^1^, C. Bolton^1^, I. Sikazwe^1^, L. Mulenga^2^, C. Phiri^2^

^1^Center for Infectious Disease Research in Zambia, Lusaka, Zambia, ^2^Ministry of Health,, Lusaka, Zambia, ^3^Centre for Infectious Diseases Research in Zambia, Lusaka, Zambia, ^4^Centre for Infectious Diseases Research, Lusaka, Zambia, ^5^Centers for Disease Control and Prevention, Lusaka, Zambia

**Background: **Controlling the HIV epidemic in Zambia will require achieving high viral suppression coverage, as outlined by UNAIDS 95‐95‐95 targets. Identifying and characterizing predictors of high viral load among recipients of care (RoC) could help guide development of interventions critical to improving HIV treatment outcomes.

**Methods: **We conducted a cross‐sectional analysis of HIV case‐based surveillance data from January to December 2021 of adult RoC (≥15 years) in Zambia with record of receiving antiretroviral therapy (ART) for ≥ 6 months and latest valid viral load (VL) measurement per national electronic health record. We assessed association of sociodemographic characteristics and ART duration on 31 December 2021, with high VL load, defined as >1,000 copies RNA/mL, based on the latest VL measurement in 2021. Regression analyses were conducted to estimate unadjusted and adjusted odds ratios to estimate effects of covariates independently associated with high VL.

**Results: **A total of 531,864 HIV RoC met inclusion criteria with majority (64.8%) female, median age of 35 years (interquartile range [IQR] 29‐42), and median ART duration of 52 months (IQR: 29‐99). High VL occurred in 18,540 (3.49%) RoC, of whom 11,083 (59.90%) were females, and 13,822 (74.55%) had been on ART for > 18 months. Factors independently associated with high VL included: being male (adjusted odds ratio [aOR]=1.42, 95% confidence interval [CI]: 1.37‐1.46), younger age: 15‐19 (aOR=2.68, 95% CI: 2.52‐2.85), 20‐24 (aOR=1.78, 95% CI: 1.70‐1.86), 25‐29 (aOR=1.40, 95% CI: 1.35‐1.46) compared to those > 30 year; shorter ART duration: 6 months (aOR =4.54 95% CI: 3.99‐5.17), 7‐12 months (aOR =2.75, 95% CI: 2.64‐2.87), 13‐18 months (aOR =1.42, 95% CI: 1.35‐ 1.49) compared > 18 months; and rural setting (aOR=1.15, 95% CI: 1.10‐ 1.19) compared to urban setting.

**Conclusions: **We identified being new on ART, male and young adult as predictors of high VL and intensifying activities centered on service delivery models for these risk groups will help the Country in its efforts to achieve high viral suppression coverage. Further evaluation of health services in rural settings may identify gaps in HIV service delivery. Identifying pathways for high VL among identified sub‐populations may represent an opportunity to reach the UNAIDS 95‐95‐95 targets.

### OALBD0102 Understanding COVID‐19 vaccine confidence in people living with HIV in Canada: a pan‐Canadian survey

C. Costiniuk ^1^, J. Singer^2,3,4^, J. Needham^3,4^, Y. Yang^5^, H. Qian^3,4^, C. Chambers^6^, A. Burchell^6,7^, H. Samji^8^, I. Colmegna^9^, S del Canto^10^, G.H. Godin^10^, M. Habanyama^10^, C. Hui^11,10^, A. Kroch^12^, E. Mandarino^10^, S. Margolese^10^, C. Martin^10^, M. Owino^10^, T. Mohammadi^3^, W. Zhang^3^, S. Pelaez^13^, C. Cooper^14^, A.H. Anis^3,2,4^

^1^McGill University Health Centre, Department of Medicine, Division of Infectious Diseases and Chronic Viral Illness Service, Montreal, Canada, ^2^School of Population and Public Health, University of British Columbia, Vancouver, Canada, ^3^Centre for Health Evaluation and Outcome Sciences, St. Paul's Hospital, Vancouver, Canada, ^4^Canadian HIV Trials Network, University of British Columbia, Vancouver, Canada, ^5^McGill University, Department of Medicine, Montreal, Canada, ^6^University of Toronto, Dalla Lana School of Public Health, Toronto, Canada, ^7^St Michael's Hospital, Unity Health, Department of Family and Community Medicine, Toronto, Canada, ^8^British Columbia Centre for Disease Control and Faculty of Health Sciences, Simon Fraser University, Burnaby, Canada, ^9^McGill University Health Centre, Department of Medicine, Division of Rheumatology, Montreal, Canada, ^10^Community Advisory Committee, Canadian HIV Trials Network, Vancouver, Canada, ^11^Ryerson University, Yeates School of Graduate Studies, Ryerson University, Toronto, Canada, ^12^Ontario HIV Treatment Network, Toronto, Canada, ^13^School of Kinesiology and Physical Activity Sciences, Faculty of Medicine, University of Montreal, Montreal, Canada, ^14^The Ottawa Hospital and Ottawa Hospital Research Institute, Ottawa, Canada

**Background: **While the advent of safe and effective COVID‐19 vaccines for the general population has led to mass vaccination roll‐outs, certain populations may lack vaccine confidence. Our objectives were to determine demographic factors associated with taking at least 1 COVID‐19 vaccine, and to determine whether there were particular elements of vaccine hesitancy associated with vaccine behaviour.

**Methods: **With community members, we developed a study questionnaire with items from the validated National Advisory Committee on Immunization Acceptability Matrix. PLWH were recruited via social media and community‐based organizations from January‐April 2022.Descriptive statistics were used to summarize results and compare responses between PLWH who have received vs those who have not received COVID‐19 vaccine(s). For each participant, scores on the 5‐point Likert scale were added together (reversing for direction, as necessary). Logistic regression models were used to identify factors associated with COVID‐19 vaccine uptake such as age, sex, gender, and responses to the vaccine confidence questions.

**Results: **205 individuals completed the survey and indicated whether or not they received>1 vaccine dose. Mean age was 47±14 (SD) years and 73% were male. Eighty percent had completed at least some highschool. Mean VHS scores were 33 ± 17 for the 153 individuals, and 17± 6 for the 21 individuals, who did and did not take at least one COVID‐19 vaccine, respectively. Univariate analyses revealed that the odds of taking at least one vaccine dose were increased 2.80‐fold [95% CI 1.91, 4.41] with each increase in age of 10 years (p<0.0001). No effect was observed for sex or education. Individuals accepted COVID‐19 vaccines more for altruistic reasons (i.e., protection of community) than individual reasons (i.e., protection of self). Individuals who felt that the pandemic would linger on longer were more likely to accept the vaccine than those who did not feel the pandemic would last that long.

**Conclusions: **Older PLWH and those who anticipated that the pandemic would be prolonged in duration were more likely to accept COVID‐19 vaccines. PLWH appear to accept COVID‐19 vaccines more for altruistic rather than individual reasons.

### OALBD0104 A community‐based participatory approach to understanding preferences, needs, and barriers to uptake of longer‐acting formulations of PrEP in transgender and gender diverse Texans

P.W. Schnarrs ^1,2^, J. Zuniga^3,2^, G. Benitez^1,2,4^, P. Fliedner^1,2^, A. Norwood^5,2,5^, R. Lane^6^, J. Oeffinger^6^, K. Brookins^7^, A. Andersen^7,8^, K. Hausmann^7,9^, H. Dulce^7^, A. Olujimi^7^, M. Croll^10,7^, E. Schelling^11^

^1^Dell Medical School ‐ The University of Texas at Austin, Population Health, Austin, United States, ^2^Dell Medical School ‐ The University of Texas at Austin, PRIDE Health Lab, Austin, United States, ^3^The University of Texas at Austin, School of Nursing, Austin, United States, ^4^The University of Texas at Austin, School of Architecture, Austin, United States, ^5^Dell Medical School ‐ The University of Texas at Austin, Internal Medicine, Austin, United States, ^6^Texas Health Institute, TransFORWARD, Austin, United States, ^7^PrEP for ALL Community Advisory Board, Austin, United States, ^8^Austin Community College, Department of Physics, Austin, United States, ^9^The University of Texas at Austin, Steve Hicks School of Social Work, Austin, United States, ^10^The University of Texas Rio Grand Valley, Political Science, Edinburg, United States, ^11^Transgender Education Network of Texas, Houston, United States

**Background: **It is estimated that only 3% of transgender and gender diverse (TGD) people currently use PrEP despite the carrying a disproportionate incidence and prevalence burden. Long acting formulations of PrEP (LA PrEP) may improve uptake and adherence, but little is known about TGD individuals’ preferences, needs, and barriers related to LA PrEP. The purpose of this community‐based participatory research (CBPR) project was to better understand the needs of TGD individuals, healthcare providers, and the similarities and differences between these groups.

**Methods: ** Using a CBPR approach, our team consists five researchers, three co‐investigators who represent Texas Health Institute, Trans*FORWARD ‐ a Patient Centered Outcomes Research Institute Engagement Project (EASC‐COVID‐00284)*,Transgender Education Network of Texas,*a*nd a community advisory board (CAB) that includes 6 TGD community members. Participants were recruited online, through existing participant pools, and through the social/professional networks of the CAB and other community partners. We conducted semi‐structured, virtual interviews with health care providers (HP; n = 20) and TGD (n = 10) participations and hosted 2 virtual world café conversations (WCC) with TGD Texans (n = 20) via Zoom. Interviews and WCCs were recorded, transcribed and are currently being thematically coded by three members of the team to ensure reliability and validity of the coding process.

**Results: **Preliminary results identify a number of barriers and facilitators to LA PrEP uptake related to contact with the healthcare system, trauma/discrimination, and reduced worry around adherence. However, of note is a disconnect between HP and TGD participants regarding intramuscular (IM) LA PrEP, with TGD indicating they did not want it, but HP believing this would be the most suitable formulation for TGD patients. Further, there is a also concern among healthcare providers regarding the rollout of LA PrEP.

**Conclusions: **Cultural humility or shared decision‐making strategies are likely needed to increase uptake of LA PrEP in TGD patients, as are interventions that leverage community leaders or trusted members of the community, such as community health worker programs or social network interventions. Also, TGD community members should be included in paid roles to support the development of strategies to increase uptake and support LA PrEP roll out.

### OALBD0105 Assessing stigma as determinant of HIV and sexually transmitted infections among sexual minority men in the United States in 2021 from the American Men's Internet Survey (AMIS)

T. Carpino ^1^, J.M. Wiginton^2,3^, O.W. Edwards^4^, M. Hannah^4^, S. Murray^1^, T. Sanchez^4^, S. Baral^1^

^1^Johns Hopkins University Bloomberg School of Public Health, Baltimore, United States, ^2^San Diego State University School of Social Work, San Diego, United States, ^3^University of California‐San Diego, Department of Medicine, San Diego, United States, ^4^Emory University Rollins School of Public Health, Atlanta, United States

**Background: **The American Men's Internet Survey (AMIS) is an annual electronic survey for sexual minority men (gay, bisexual, same‐gender loving, and other men who have sex with men (MSM)) aged 15+ in the United States. Given a sustained rise in sexually transmitted infections (STI) in the US, we use stigma frameworks to characterize risk factors associated with HIV and any STI (gonorrhea, chlamydia, or syphilis) diagnosis.

**Methods: **Using AMIS data collected September 2021 to February 2022 and STATA 16.1, correlation matrices were developed to inform Pearson's chi‐squared tests and model building. Multivariate logistic regression was used to assess associations between stigma‐related factors and STI diagnosis in the past 12 months, while controlling for HIV status, age, education, race, and region. Another model was developed for individuals not living with HIV infection, additionally controlled for PrEP use in the past 12 months.

**Results: **Overall, 9,061 individuals were included (13.1% Hispanic, 14.1% Black, 64.3% white, non‐Hispanic) with mean age 44.3 years. HIV and STI prevalences were 14.3% and 13.0%, respectively. STI diagnosis was more common among those who experienced stigma from family, friends, and the general community (χ^2 > 23.0, Pr<0.01). Adjusting for age, education, race, HIV status, and region, those with an STI diagnosis were twice as likely to have experienced general community stigma (aOR=2.0; 95% CI= (1.8, 2.3). Among 7,758 people not living with HIV, 2,652 (34.2%) used PrEP in the past 12 months and while controlling for this PrEP use, the association between general community stigma and STI diagnosis remained (aOR=1.8; 95% CI=(1.5,2.1)).

**Conclusions: **Given sustained HIV and STI epidemics among sexual minority men, these results highlight complex relationships between stigma, HIV, PrEP, and STIs. Stigma may be positively associated with STI diagnosis due to (1) screening or treatment‐seeking delays, or (2) differences in outness and provider screening recommendations. If people experiencing stigma are more likely to be diagnosed with an STI, there are potential consequences with how stigma varies treatment behaviors. Characterizing the mechanisms by which stigma increases odds of HIV and STIs is central to intervention development and ultimately, changing the trajectory of these epidemics in the US.

### OALBE0102 SEARCH‐Youth: a cluster randomized trial of a multilevel health system intervention to improve virologic suppression in adolescents and young adults living with HIV in rural Kenya and Uganda

F. Mwangwa ^1^, T. Ruel^2^, L. Balzer^3^, J. Ayieko^4^, M. Nyabuti^4^, W.E. Mugoma^4^, J. Kabami^5^, B. Kamugisha^1^, D. Black^6^, B. Nzarubara^1^, F. Opel^4^, J. Shrom^6^, G. Agengo^4^, J. Nakigudde^7^, H. Nakato^1^, J. Schwab^8^, J. Peng^9^, C. Camlin^10^, S. Shade^11^, E. Bukusi^4^, B. Kapogiannis^12^, E. Charlebois^13^, M. Kamya^14^, D. Havlir^6^

^1^Infectious Diseases Research Collaboration, Kampala, Uganda, ^2^University of California, San Francisco, Pediatrics, San Francisco, United States, ^3^University of Massachusetts – Amherst, Amherst, United States, ^4^Kenya Medical Research Institute, Centre for Microbiology Research, Nairobi, Kenya, ^5^Makerere University, Department of Medicine, Kampala, Uganda, ^6^University of California, San Francisco, Department of Medicine, San Francisco, United States, ^7^Makerere University, Department of Psychiatry, Kampala, Uganda, ^8^University of California, Berkeley;, Division of Biostatistics, Berkeley, United States, ^9^University of Washington, Department of Biostatistics, Seattle, United States, ^10^University of California, San Francisco, Department of Obstetrics, Gynecology & Reproductive Sciences, San Francisco, United States, ^11^University of California, San Francisco, Department of Epidemiology and Biostatistics, San Francisco, United States, ^12^Eunice Kennedy Shriver National Institute of Child Health and Human Development, Maternal and Pediatric Infectious Diseases Branch, Bethesda, United States, ^13^University of California, San Francisco, Medicine, San Francisco, United States, ^14^Makerere University, Medicine, Kampala, Uganda

**Background: **Social and cognitive developmental events disrupt care and medication adherence among adolescents and young adults living with HIV in sub‐Saharan Africa. We hypothesized that a dynamic multilevel health system intervention helping adolescents and young adults and their providers navigate life‐stage related events would increase virologic suppression compared to standard care.

**Methods: **This was a cluster‐randomized trial of 28 clinics and youth aged 15–24 years living with HIV in rural Kenya and Uganda. Participants in intervention clinics received life‐stage specific assessment and counseling at the start of routine visits, choice of flexible clinic access and rapid viral load feedback. roviders had a secure mobile platform for inter‐provider consultation. The primary endpoint was virologic suppression (< 400 copies/ml) two years from enrollment and compared by arm using targeted minimum loss‐based estimation, adjusting for clustering by clinic.

**Results: **Among 1834 participants, median age was 21 years and 82% were female; 85% of intervention participants had life‐stage assessments performed ≥ 4 times, 60% opted for phone visits at least once, and >87% of participants in 13/14 clinics received viral load feedback within 72 hours. The proportion of participants with virologic suppression was higher in the intervention arm [88% (95%CI: 85‐92%)] than in the control arm [80% (95%CI: 77‐84%)], for an adjusted risk ratio of 1.10 (95%CI: 1.03‐1.16; p=0.002). The intervention resulted in increased virologic suppression within subgroups of sex, age, and care‐status at baseline, with greatest improvement among those re‐engaging in care [adjusted risk ratio =1.60 (95% CI:1.00‐2.55; p=0.03)] (Figure).

**Conclusions: **The multilevel SEARCH‐Youth intervention resulted in increased virologic suppression compared to standard care, during a period of transition to dolutegravir and during the COVID19 pandemic. Added to current efforts, systematic life‐stage‐based assessment and support could help bring adolescents and young adults living with HIV closer towards a goal of universal virologic suppression.

**Figure:**
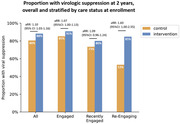
**Abstract OALBE0102‐Figure 1**.

### OALBE0103 A tale of two countries: assessing the transition, scale‐up, and sustainability of HIVST introduction in India and Indonesia

P. Loh^1^, L. Hicks^1^, A.I. Sirait^1^, A. Hegde ^2^, C. Laxmeshwar^2^, K. Mariyappan^2^, R.J. Magnani^3^, K. Green^4^

^1^PATH, Seattle, United States, ^2^PATH, New Delhi, India, ^3^Depok, Faculty of Public Health, Depok, Indonesia, ^4^PATH, Hanoi, Viet Nam

**Background: **Limited research is available to describe the key factors attributable to the introduction and scale‐up of HIV self‐testing products in India and Indonesia. The Unitaid‐funded STAR‐III initiative uses a multidisciplinary monitoring and evaluation (M&E) approach to introduce, evaluate, and transition HIV self‐testing products in its implementing countries by partnering with government entities to strengthen policy and regulatory procedures, fortify monitoring and evaluation processes, and introduce communications and demand generation practices to increase uptake and use of HIV self‐testing products.

**Description: **The STAR‐III Initiative developed an M&E framework to track and monitor progress towards the transition, scale‐up, and sustainability of HIVST across India, Indonesia, and its other implementing countries. This framework consists of 28‐indicators across 6‐domains: (1) Governance & Policies; (2) Funding Security; (3) Supply & Delivery; (4) M & E; (5) Supervision; (6) Scale‐up. Primary and secondary data for these indicators were collected through literature reviews and/or key informant interviews at the start (02/2020) and end (05/2022) of the program and compared to measure progress.

**Lessons learned: **Ourassessment found improved performance across the six evaluative domains, with scalability strongest across Funding Security and Supervision domains.This was largely due to several actions takento operationalize HIVST, including (1) the development of training modules and processes by civil society organizations that have been endorsed by MoH, (2) the operationalization of HIVST delivery channels, and (3) a standing HIVST agenda within the national HIV/AIDS Technical Working Groups.The supporting infrastructure acrossGovernance & Policies, Supply & Delivery, and Scale‐up domains remain a work in progress.

**Conclusions/Next steps: **Although STAR‐III has built the foundation for institutionalization and sustainability of HIVST in Indonesia and India, the policy environment and supporting infrastructure remains a work in progress. HIVST implementation in these countries is being constrained by the unavailability of registered HIVST products which has had direct and indirect impacts on domains of Governance & Policy, Supply & Delivery, and Scale‐up of HIVST. There is an urgent need to work with regulatory authorities to support the registration of HIVST products in India, Indonesia, and also other countries looking to introduce or scale‐up HIVST.

### OALBE0104 What women want ‐ results from discrete choice experiment about preferred PrEP method from Khomas region of Namibia

E. Melese^1^, E. Negumbo^1^, M. Nur^1^, C. Uakuramenua^1^, B. Haufiku^2^, M. Rubeni^1^, S. David^1^, M. Kakende^1^, P. Kamanga^1^, C. Makuyana^1^, M. Shilomboleni^2^, B. Harases^1^, R. Indongo^1^, S. Neri^1^, M. Manyando^3^, N. N Borse ^4^

^1^Project HOPE Namibia, Windhoek, Namibia, ^2^IntraHealth, Windhoek, Namibia, ^3^US Agency for International Development (USAID∖Namibia), Windhoek, Namibia, ^4^Project HOPE, Global Health Technical Unit, Washington, United States

**Background: **Adherence to Pre‐exposure Prophylaxis to reduce new HIV infection among Adolescent Girls and Young Women (AGYW) of age 10 to 24 years remains a critical challenge. Under the USAID/Namibia‐funded DREAMS project, PrEP is offered as part of the package with limited continuation.

Besides daily oral PrEP, with recent positive results from HPTN‐084 on Cabotegravir Long‐Acting Injectable (CAB‐LA) and WHO approval of Dapivirine Vaginal Ring (DAP‐VR), this study had an objective to take end‐user perspectives on their preferred PrEP method of choice.

**Methods: **A survey was conducted using a convenient sampling method with 1,675 active AGYW from the Khomas region. The interview questions included demographics, PrEP knowledge, attitude, practices, and preferred PrEP options. Before the PrEP preferred options section was administered, DREAMS Nurses used visual and narrative descriptions to educate AGYW on the attributes of PrEP options (i.e., oral, DAP‐VR and CAB‐LA). From April 26 to 28, 2022, DREAMS staff utilized the REDCap mobile application to collect data. Data was analyzed in STATAv15.

**Results: **Of the 1,656 respondents, 92% heard about PrEP for HIV prevention, and the primary sources of information were DREAMS staff (85%). Of these, 79% believed PrEP could reduce new HIV infection, and 68% knew PrEP could not prevent other STIs. Moreover, 97% knew where to get oral PrEP, 31% were current/previous users of oral PrEP, and 41% agreed/strongly agreed that PrEP has serious side effects.

Oral PrEP was well known to AGYW (85%), while 3% and 8% were aware of DVR and CAB‐LA, respectively. If all three PrEP options are available, 83% (n=1262) of AGYW were willing to use any of them. Of these, 61% indicated a preference for CAB‐LA, while 27% and 12% preferred oral and DVR. HIV prevention efficacy was a strong determinant of stated reason, followed by the desire for less frequent use and discreteness of the option.

**Conclusions: **The results from this study provide vital information for the successful introduction, scale‐up, and continued use in the target population. CAB‐LA injectable is the preferred PrEP option among AGYW mainly due to its efficacy, every eight‐week administration instead of a daily pill, and its discreteness.

### OALBE0105 The catalytic role of dual HIV/syphilis rapid diagnostic tests in accelerating national elimination of mother‐to‐child‐transmission in Nigeria

C. Nwafor ^1^, O. Alintah^2^, J. Nwi‐ue^1^, S. Igbiri^1^, C. Stillson^3^, A. Storey^2^, O. Wiwa^1^

^1^Clinton Health Access Initiative, Syphilis Program, Abuja, Nigeria, ^2^Clinton Health Access Initiative, Global MNH Program, London, United Kingdom, ^3^Clinton Health Access Initiative, Global HIV Access Program, Boston, United States

**Background: **HIV and syphilis prevalence among pregnant women attending antenatal clinics in Nigeria is 1.4% and 0.7% respectively. The disparity between national maternal syphilis testing rates at 16% and maternal HIV testing at 66% represented an opportunity to leverage higher HIV testing coverage to increase syphilis testing coverage, thereby accelerating progress towards dual elimination targets. Dual HIV/syphilis RDTs allow providers to simultaneously screen clients for HIV and syphilis, eliminating the need for multiple tests.

**Description: **Between 2019 and 2021, CHAI collaborated with NASCP to pilot dual HIV/syphilis RDTs in 31 high volume ANC facilities across all health system tiers in Akwa Ibom, Anambra, and Rivers states. 1,678 healthcare workers were trained to counsel and test pregnant women using dual RDTs, administer Benzathine Penicillin‐G for syphilis treatment, and manage syphilis program data. CHAI institutionalized the dual program within the priorities of existing HIV Technical Working Groups, leveraging the platform to share key learnings and advocate scale up.

**Lessons learned: **45,413 pregnant women were tested for HIV and syphilis using dual RDTs (15,662 in Akwa Ibom, 13,369 in Anambra and 16,382 tested in Rivers state). HIV and syphilis positivity rates were 2.0% and 0.2% respectively. BPG treatment uptake was 90% and partner testing rate for syphilis‐positive pregnant women was 80%. Analysis of HCW feedback from CHAI and MOH facilitated learning sessions revealed a 95% satisfaction rate with the implementation model and 15% improvement in pre‐ and post‐training aptitude assessments. 100% of participants said they would recommend dual RDT scale‐up.

**Conclusions/Next steps: **Evidence generated through these pilots informed national scale‐up efforts, leading to the inclusion of dual RDTs in annual operational plans of not just the three states, but also NASCP and Nigeria's major HIV program funders– PEPFAR and Global Fund. Building on these lessons, the Nigerian Government directly procured 2.5million dual RDTs in late 2021, backed by an additional 1.2million and 350,000 RDTs funded by PEPFAR Nigeria in COP‐22 and Global Fund respectively. The congenital syphilis burden in Nigeria is comparable to other LMICs and Health Ministries are encouraged to learn from this model to scale up such novel diagnostics in support of national dual elimination priorities.

### OALBF0102 Canada's criminal injustice: new national community consensus on law reform to end HIV criminalization

R. Elliott ^1,2,1,2^

^1^HIV Legal Network, Toronto, Canada, ^2^Canadian Coalition to Reform HIV Criminalization, Toronto, Canada

**Background: **Canada has been a global ‘hot spot’ of HIV criminalization. There is no HIV/STI‐specific offence; prosecution of alleged HIV/STI non‐disclosure occurs through application of general Criminal Code offences. The charge most frequently used is *aggravated sexual assault*, following Supreme Court of Canada decisions that if there is a “realistic possibility of HIV transmission,” non‐disclosure amounts to “fraud” invalidating consent to sex. Prosecutors' and courts' interpretations of this legal test have led to prosecutions that perpetuate HIV stigma and are at odds with the international scientific Expert Consensus Statement (released at AIDS 2018), recommendations of the Global Commission on HIV & the Law, and the Global AIDS Strategy. Analyses by Justice Canada and NGOs highlight a disproportionate impact on Black, Indigenous and gay communities.

**Description: **In 2017, the Canadian Coalition to Reform HIV Criminalization, supported by 174 organizations nation‐wide, called on Parliament to legislate an end to sexual assault charges and limit any criminalization to cases of intentional, actual transmission. In 2019, a Parliamentary committee recommended reforms to limit HIV criminalization, but through the adoption of a new offence of transmitting an infectious disease. From 2019‐2022, the Coalition explored options for reform, including through a national community consultation, which underscored widespread concern about the status quo and strong consensus in favour of reform. In July 2022, the Coalition is releasing its new Community Consensus Statement outlining detailed legislative proposals to limit HIV criminalization.

**Lessons learned: **As there is no existing HIV/STI‐specific law in Canada to repeal or modernize, the Coalition's proposed legislative changes would (1) preclude sexual assault charges entirely, and (2) add provisions to the Criminal Code strictly limiting the use of any other, existing offences. The proposed amendments avoid the stigma of creating a new HIV/STI‐specific offence, limit criminalization to purposely (and actually) transmitting HIV/STIs, and avoid expanding criminalization to other transmissible infections.

**Conclusions/Next steps: **The new national Community Consensus Statement is the result of extensive community consultation and reflection regarding the best options to limit HIV criminalization in Canada in line with international recommendations. It will be a key advocacy tool for engaging policymakers to enact necessary legislative reforms.

### OALBF0103 Preliminary report on the provision of HIV care to war refugees living with HIV who are migrating from Ukraine ‐ data from ECEE Network Group

A. Skrzat‐Klapaczyńska^1^, B. Lakatos^2^, J. Kowalska ^3^, Euroguidelines in Central and Eastern Europe Network Group

^1^Hospital for Infectious Diseases in Warsaw, Warsaw, Poland, ^2^South‐Pest Central Hospital, Infectious diseases, AIDS and Clinical Immunology Center, Budapest, Hungary, ^3^Medical University of Warsaw, Department of Adults' Infectious Diseases, Warsaw, Poland

**Background: **The armed conflict in Ukraine resulted in humanitarian crisis with over five million people migrating to neighboring countries and ten million being displaced within Ukraine. The HIV epidemic in Ukraine is the largest in Europe with approximately 130 000 adult people on ART, half of them being women, and 2700 children. It is important to understand the impact of war on continuum of HIV care for displaced people and the impact on national HIV programs, especially in countries from Central and Eastern Europe (CEE).

**Figure:**
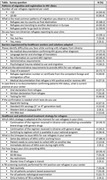
**Abstract OALBF0103‐Table 1**.

**Methods: **The ECEE Network Group consists of 47 experts in infectious diseases from 24 countries, actively involved in patients’ care. The group was established in 2016 to endorse and disseminate the standards of care for HIV and hepatitis. In March 2022, an online survey (28 questions) was created on MonkeySurvey® and disseminated by group members.

**Results: **22 (75.9%) centers from 14 countries (Bulgaria,Croatia, Czech Republic, Estonia, Georgia,Greece, Hungary, Latvia, Lithuania, Malta, Poland, Republic of Moldova, Romania,Slovakia) were admitting war refugees from Ukraine as of 31 March 2022 and completed the survey. Most centers (86%) organized promptly providing access to ARVs on the same day, for 30 days or longer in 77% of the centers. Continuation of ART was manageable with brand or generic drugs in 64% centers, whereas 36% were switching ART (Table). 81.8% of respondents indicated that increased workload with could affect local HIV care.

**Conclusions: **CEE countries receiving war refugees implemented prompt measurements to provide continuity of HIV care including universal healthcare insurance, waiving most administrative requirements, providing same day doctor's consultations and ART disposal. Barriers identified were lack of medical documentation, language barrier (shortage of translators) and psychological trauma. Our study identifies gaps which should informs international stakeholders on how to assist countries in delivering undisrupted HIV care to war refugees from Ukraine.

### OALBF0104 Education as an enabling factor to HIV prevention and adherence to treatment

C. Ouma ^1^, G. Giordana^1^, I. Omondi^1^, M. Hambayi^1^, C. Barkatdaoud^2^, R. Azizi^2^

^1^World Food Programme, Nairobi, Kenya, ^2^World Food Programme, Djibouti Ville, Djibouti

**Background: **The World Food Programme, UNAIDS, the Ministry of Health and the Ministry of Solidarity and Social Affairs conducted a study in Djibouti Ville to better understand the relationship between basic education and HIV treatment and care outcomes among people living with HIV and TB patients.

**Methods: **This was a cross‐sectional study with 805 PLHIV included in the analysis from a total of 9 ART and TB clinics. Sampling was based on the probability proportional to size model. Data collection was done by trained social workers and stored in the encrypted WFP cloud system. Descriptive analysis was done for PLHIV including those co‐infected with TB. Levels of education and the associated HIV prevention and treatment adherence variables were the outcomes of interest.

**Results: **A high proportion of PLHIV have no formal education (47.6%; n=383) with 19.0% (n=153), 19.5% (n=157) and 10.1% (n=81) reporting highest level of education as primary (grade 1‐5), middle (grade 6‐9) and secondary school (grade 10‐13) respectively. Only 3.9% (n=31) of PLHIV attended tertiary school (college/university). An inverse linear relationship exists between one's level of education and skipping doses of ARV in the 30 days prior to the study. Out of a total of 251 PLHIV who reported missing doses of ARV medication in the 30 days prior to the study, 57% (n=143) did not have formal education while the lowest proportion was reported in those who had attended university/college (3.6%; n=9). A similar trend was observed with respondents’ engagement with multiple sexual partners. The study found that from a total of 275 PLHIV who reported engaging with multiple sexual partners in the 12 months prior to the study, 49.8% (n=137) had no formal education.

**Conclusions: **An indirect relationship exists between the study participants’ level of education and their propensity to engage in risky sexual behaviours and their likelihood of non‐adherence to the ARV regime. Literacy coupled with an adequate knowledge of the effects of non‐adherence as well as of the risks associated with risky behaviour are among some of the most effective HIV prevention means, preventing the spread of the virus and fostering treatment outcomes.

### OALBF0105 Strengthening TB/HIV human rights programming to overcome barriers to accessing TB services

J. Malar ^1^, T. Abdullaev^2^, M. Sebayang^3^, C. Nawina^4^, B. Citro^5^, V. Soltan^1^, A. Herna Sari^6^, A. Obiefuna^7^

^1^Stop TB Partnership ‐ UNOPS, Geneva, Switzerland, ^2^TBpeople, Tashkent, Uzbekistan, ^3^JIP, Jakarta, Indonesia, ^4^CITAM+, Lusaka, Zambia, ^5^Consultant, Chicago, United States, ^6^Rekat, Surubaya, Indonesia, ^7^Afro Global Alliance, Accra, Ghana

**Background: **TB remains the biggest killer of PLHIV. Despite this, very little effort and investment has been directed to TB and TB/HIV human rights programming. To help build the evidence base, engagement and investment in TB and TB/HIV human rights programming, Between 2018 and 2021, 20 National Assessments of human rights and gender assessments were undertaken in countries in Asia, Africa and Europe in order to better understand human rights related barriers to accessing TB services and inform human rights programming. Significantly, these assessments were led by TB and TB/HIV affected communities and civil society in close partnership with the National TB Programme to ensure national ownership and sustainability.

Stop TB Partnership provided technical assistance in this USAID and Global Fund funded initiative.

**Description: **In a first for TB and TB/HIV programmes, national assessments of human rights and gender related barriers to accessing TB services were conducted in 20 countries and focused on:

1. identify legal, policy and programmatic human rights and gender related barriers to access;
2. Identify and prioritize TB key and vulnerable population;
3. recommend priority interventions to strengthen human rights related TB and TB/HIV programming in country.

These three areas of investigation were guided by a Communities, Rights and Gender Protocol which included desk review, focus groups, and national validation.

**Lessons learned: **All 20 completed CRG assessments have subsequently been analysed using the right to health framework:

1. accessibility, acceptability, affordability and quality of services;
2. stigma and discimination;
3. freedoms (information, privacy, confidentiality)
4. gender
5. key and vulnerable populations
6. participation of key and vulnerable populations
7. legal remedies.

Responses to these barriers has now been operationalised through costed TB CRG Plans in several countries.

**Conclusions/Next steps: **There is now unprecedented evidence on TB/HIV human rights programming that must be operationalized and implemented in order for countries to realize the UN HLM targets on human rights. Next steps: develop costed CRG Action Plans; integrate recommendations into NSPs; human rights investments in national TB/HIV responses is increased; monitoring and evaluation of human rights investments in national TB/HIV is enhanced; and, community led monitoring of rights is integrated into national responses.

### OALBX0102 Transcriptional programs of HIV silencing and cell survival in HIV‐infected memory CD4 T cells under antiretroviral therapy

E. Boritz ^1^, I. Clark^2^, P. Mudvari^1^, S. Thaploo^3^, S. Smith^1^, M. Hamouda^1^, M. Abu‐Laban^1^, S.H. Ko^1^, D. Bunis^1^, J. Lee^1^, D. Kilam^1^, S. Zakaria^1^, S. Choi^1^, S. Darko^1^, A. Henry^1^, M. Wheeler^3^, R. Hoh^4^, S. Deeks^4^, F. Quintana^3^, D. Douek^1^, A. Abate^5^

^1^Vaccine Research Center, National Institute of Allergy and Infectious Diseases, National Institutes of Health USA, Bethesda, United States, ^2^University of California Berkeley, Department of Bioengineering, Berkeley, United States, ^3^Brigham and Women's Hospital, Harvard Medical School, Ann Romney Center for Neurologic Diseases, Boston, United States, ^4^University of California San Francisco, Department of Medicine, San Francisco, United States, ^5^University of California San Francisco, Department of Bioengineering and Therapeutic Sciences, San Francisco, United States

**Background: **Rare memory CD4 T cells harboring HIV under antiretroviral therapy (ART) represent an important barrier to HIV cure, but the infeasibility of isolating and characterizing these cells in their “natural” state has bred uncertainty about whether they possess distinctive attributes that HIV cure‐directed therapies might exploit.

**Methods: **We developed a custom microfluidic process termed Focused Interrogation of cells by Nucleic acid Detection and Sequencing (FIND‐Seq), which captures polyadenylated RNA and genomic DNA from millions of single cells within water‐in‐oil droplets and then sorts single‐cell transcriptomes based solely on HIV DNA detection. Using blood cells from people with HIV (PWH) who had initiated ART during chronic infection and experienced >1 year of virologic suppression (n = 6), memory CD4 T cell transcriptomes were sorted by FIND‐Seq into HIV DNA^+^ and uninfected cell fractions and sequenced. Host cell transcriptomic profiles of HIV DNA^+^ and uninfected memory CD4 T cells were compared by differential gene expression, co‐expression network analysis, and gene ontology.

**Results: **HIV DNA^+^ memory CD4 T cells from ART‐treated PWH consistently showed inhibition of six transcriptomic pathways including death receptor signaling, necroptosis signaling, and Gα12/13 signaling. Gene co‐expression network analysis revealed two small gene clusters associated with HIV DNA^+^ cells. Gene ontology revealed significant enrichment of these clusters for factors related to epigenetic gene regulation, RNA processing, and the regulation of cell death signaling. Individual genes in these clusters included HIV transcriptional activators that were lower in HIV DNA^+^ cells and HIV silencing factors affecting both transcriptional and post‐transcriptional steps in HIV gene expression that were higher in HIV DNA^+^ cells. Remaining genes in these clusters not previously associated with HIV gene expression had roles in chromatin modification, RNA processing, and the survival and proliferation of CD4 T cells.

**Conclusions: **Whole transcriptome sequencing of unmanipulated HIV DNA^+^ memory CD4 T cells under ART reveals these cells as a highly selected population with distinctive gene expression patterns that can promote HIV persistence through HIV silencing, cell survival, and cell proliferation. These findings help reconcile previous observations made in *ex vivo* and *in vivo* studies, and suggest important next steps in research towards an HIV cure.

### OALBX0103 Doxycycline post‐exposure prophylaxis for STI prevention among MSM and transgender women on HIV PrEP or living with HIV: high efficacy to reduce incident STI's in a randomized trial

A. Luetkemeyer ^1^, J. Dombrowski^2,3^, S. Cohen^4,5^, D. Donnell^2^, C. Grabow^2^, C. Brown^2^, C. Malinski^2,3^, R. Perkins^2^, M. Nasser^4^, C. Lopez^6^, S. Buchbinder^4^, H. Scott^4^, E. Charlebois^5^, D. Havlir^6^, O. Soge^2^, C. Celum^2^

^1^Zuckerberg San Francisco General Hospital, University of California San Francisco, Division of HIV, Infectious Diseases and Global Medicine, San Francisco, United States, ^2^University of Washington, Seattle, United States, ^3^Public Heath ‐ Seattle & King County, Seattle, United States, ^4^San Francisco Department of Public Health, San Francisco, United States, ^5^University of California San Francisco, San Francisco, United States, ^6^Zuckerberg San Francisco General Hospital, University of California San Francisco, San Francisco, United States

**Background: **With a sexually transmitted infection (STI) epidemic among men who have sex with men(MSM) and transgender women(TGW), interventions to reduce STIs are needed.

**Methods: **DoxyPEP is a randomized open‐label trial among Seattle and San Francisco MSM/TGW living with HIV or on PrEP who had *N. gonorroheae* (GC), *C. trachomatis* (CT), or early syphilis in the past year. Randomization was 2:1 to 200 mg doxycycline hyclate within 72 hours of condomless sex or no doxycycline with STI testing at enrollment, quarterly, and when symptomatic. An independent committee adjudicated STIs. The trial had >80% power to detect a 50% reduction in STI incidence, assuming a 10% quarterly STI incidence. The intent to treat analysis compared relative risk of an STI per quarter. A single interim analysis at ∼50% of follow‐up time occurred May 2022; the DSMB recommended stopping the control arm based on prespecified efficacy thresholds measured independently in PLWH and PrEP cohorts.

**Results: **Of 554 enrolled, 360 were on PrEP, 194 PWLH, 65% white, 8% Black, 10% Asian, 30% Latinx. 19 (3%) identified as TGW or nonbinary. Median sex partners in past 3 months was 9. In the past year 67% had GC, 58% CT, 20% syphilis; at enrollment, 18% had GC, 10% CT, 2% syphilis. Among 360 on PrEP, 65 STI endpoints (29.5%) occured in controls and 47 (9.6%) in doxyPEP participants (RR 0.33; 95%CI 0.23‐0.47; p<0.0001). Among 194 PLWH, 30 STI endpoints (27.8%) in controls and 31 (11.7%) in doxyPEP participants (RR 0.42; 95% CI 0.25‐0.75; p=0.0014). GC, CT, and syphilis were each reduced. No serious or ≥ Grade 2 AEs were attributed to doxycycline.

**Figure:**
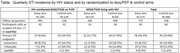
**Abstract OALBX0103‐Table 1**.

**Conclusions: **Doxycycline 200 mg taken within 72 hours after condomless sex significantly reduced STIs in MSM/TGW. Effects on antimicrobial resistance, gut microbiome, and sexual behavior are being assessed as important considerations for this STI prevention strategy.

### OALBX0105 Week 48 results of a Phase 3 randomized controlled trial of bictegravir/emtricitabine/tenofovir alafenamide (B/F/TAF) vs dolutegravir + emtricitabine/tenofovir Disoproxil Fumarate (DTG+F/TDF) as initial treatment in HIV/HBV‐coinfected adults (ALLIANCE)

A. Avihingsanon ^1^, H. Lu^2^, C.L. Leong^3^, C.‐C. Hung^4^, E. Koenig^5^, S. Kiertiburanakul^6^, M.‐P. Lee^7^, K. Supparatpinyo^8^, F. Zhang^9^, S. Rahman^10^, M. D'Antoni Brogan^10^, H. Wang^10^, J. Hindman^10^, H. Martin^10^, J. Baeten^10^, T. Li^11^

^1^HIV‐NAT, Thai Red Cross AIDS Research Centre, Bangkok, Thailand, ^2^Shanghai Public Health Clinical Center, Shanghai, China, ^3^Department of Medicine, Kuala Lumpur General Hospital, Kuala Lumpur, Malaysia, ^4^National Taiwan University Hospital, Taipei, Taiwan, Province of China, ^5^Instituto Dominicano de Estudio Virologicos ‐ IDEV, Santo Domingo, Dominican Republic, the, ^6^Ramathibodi Hospital, Bangkok, Thailand, ^7^Queen Elizabeth Hospital, Kowloon, Hong Kong, SAR of China, ^8^Chiang Mai University, Chiang Mai, Thailand, ^9^Beijing Ditan Hospital, Capital Medical University, Beijing, China, ^10^Gilead Sciences, Foster City, United States, ^11^Peking Union Medical College Hospital, Beijing, China

**Background: **The clinical course of HBV in individuals with HIV coinfection is marked by accelerated disease progression. A tenofovir‐containing anti‐retroviral regimen is recommended in most people with HIV‐1/HBV‐coinfection but there have not been randomized studies of TDF vs TAF in treatment‐naïve HIV‐1/HBV‐coinfected individuals. We report primary endpoint results from a phase 3 study comparing B/F/TAF vs DTG+F/TDF at Week (W) 48 in participants initiating treatment for both viruses.

**Methods: **Adults with HIV‐1/HBV coinfection were randomized 1:1 to initiate blinded treatment with B/F/TAF or DTG+F/TDF (with placebo). Primary endpoints were proportion of participants with HIV‐1 RNA <50 copies/mL (FDA Snapshot) and plasma HBV DNA <29 IU/mL (missing=failure) at Week 48. Noninferiority was assessed with 95% CI (12% margin). Secondary and other endpoints included change from baseline CD4 count, proportion with HBsAg and HBeAg loss/seroconversion, and ALT normalization (AASLD criteria).

**Results: **243 participants were randomized and treated (121 B/F/TAF, 122 DTG+F/TDF) from 11 countries in Asia, Europe, North and Latin America. Baseline characteristics were median age 32 years, 4.5% female, 88% Asian, 30% HIV‐1 RNA >100,000 c/mL, 40% CD4 <200 cells/mL, median HBV DNA 8.1 log_10_ IU/mL, 78% HBeAg+. At W48, B/F/TAF was noninferior to DTG+F/TDF at achieving HIV‐1 RNA <50 copies/mL (95% vs 91%, difference 4.1%; 95% CI ‐2.5% to 10.8%, p=0.21), with mean CD4 gains of +200 and +175 cells/mL, respectively. B/F/TAF was superior to DTG+F/TDF at achieving HBV DNA <29 IU/mL (63% vs 43%, difference 16.6%; 95% CI 5.9% to 27.3%, p=0.0023). Participants treated with B/F/TAF vs DTG+F/TDF had numerically higher HBsAg loss (13%, 6%, p=0.059), HBeAg loss (26%, 14%, p=0.055), HBeAg seroconversion (23%, 11%, p=0.031), and ALT normalization (73%, 55%, p=0.066). Most frequent AEs were upper respiratory tract infection (17%, 11%), COVID‐19 (13%, 11%), pyrexia (9%, 12%), ALT increase (7%, 11%), and nasopharyngitis (11%, 4%). ALT flares (elevations at ≥2 consecutive post‐baseline visits) occurred in 11 participants (7 B/F/TAF, 4 DTG+F/TDF) which resolved.

**Conclusions: **In adults with HIV‐1/HBV‐coinfection starting antiviral therapy, both B/F/TAF and DTG+F/TDF had high HIV‐1 suppression at year 1, with B/F/TAF resulting in superior HBV DNA suppression and significantly more HBeAg seroconversion. Safety findings were similar between groups.

### OALBX0106 Trends in PrEP inequity by race and census region, United States, 2012‐2021

P.S. Sullivan ^1^, S. Whitby^1^, P. Hipp^1^, M. Juhasz^2^, S. DuBose^1^, P. McGuinness^3^, D.K. Smith@@@^4^

^1^Emory University, Rollins School of Public Health, Epidemiology, Atlanta, United States, ^2^Saluda Analytics, Budapest, Hungary, ^3^Gilead Sciences, Foster City, United States, ^4^Centers for Disease Control and Prevention, Atlanta, United States

**Background: **PrEP was approved for HIV prevention in the US in 2012; uptake has been slow. Black and Hispanic people have higher rates of new HIV diagnoses than White non‐Hispanic people in the US. We describe the inequitable use of PrEP by race within US regions from 2012‐2021.

**Methods: **We used commercial pharmacy data to enumerate PrEP users by race and US Census region from 2012‐2021. Race/ethnicity data were available for 124,835 (34%) of PrEP users; to estimate total PrEP users by race, we assumed that the racial distribution was the same in PrEP users with missing race data as in those with reported race data. The PrEP‐to‐Need Ratio (PnR), a metric of PrEP equity, was defined as the number of PrEP users in a group divided by the number of new diagnoses in that group in the same year.

**Results: **PnR increased from 2012‐2021 for all races and regions, but levels of PrEP use were not consistent across regions (See Figure) and were not equitable (defined by differences in PnR by race/ethnicity). In all regions, PnR was highest for White and lowest for Black people. By region, the highest region‐ and race‐specific PnR was for White people in the Northeast in 2021: the PnR was 48.7 and the absolute difference in White versus Black PnRs was 44.5 (White:48.7; Black:4.2).

**Figure:**
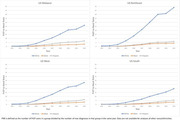
**Abstract OALBX0106‐Figure 1**.

**Conclusions: **Prevention programs should be guided by PrEP equity (use relative to epidemic impact), not PrEP equality (equal use in groups, regardless of HIV diagnosis proportion). By this measure, US prevention programs in all regions demonstrated decreasing PrEP equity over time (e.g., larger gaps in PnR by race/ethnicity). The US South lagged all regions in equitable PrEP use, with the lowest PnR overall compared to other US regions. Better programs are needed to provide PrEP to people at greatest risk for HIV infection.

### OALBX0107 Long acting cabotegravir: updated efficacy and safety results from HPTN 084

S. Delany‐Moretlwe ^1^, J.P. Hughes^2^, P. Bock^3^, S. Dadabhai^4^, D. Gadama^5^, P. Hunidzarira^6^, S. Innes^7^, D. Kalonji^8^, J. Makhema^9^, P. Mandima^6^, C. Mathew^1^, J. Mpendo^10^, P. Mukwekwerere^6^, N. Mgodi^6^, P. Nahirya Ntege^11^, C. Nakabiito^12^, H. Nuwagaba‐Biribonwoha^13^, R. Panchia^14^, F. Angira^15^, N. Singh^8^, B. Siziba^6^, E. Spooner^8^, J. Farrior^16^, S. Rose^16^, R. Berhanu^1^, Y. Agyei^17^, S.H. Eshleman^17^, M.A. Marzinke^17^, E. Piwowar‐Manning^17^, S. Beigel‐Orme^2^, S. Hosek^18^, A. Adeyeye^19^, J.R. Rooney^20^, A. Rinehart^21^, B. Hanscom^2^, M. Cohen^22^, M. Hosseinipour^5,22^, on behalf of the HPTN 084 study team

^1^University of the Witwatersrand, Wits RHI, Johannesburg, South Africa, ^2^Fred Hutchinson Cancer Research Center, Statistical Centre for HIV/AIDS Research and Prevention, Seattle, United States, ^3^University of Stellenbosch, Desmond Tutu TB Centre, Stellenbosch, South Africa, ^4^College of Medicine, Johns Hopkins Research Project, Blantyre, Malawi, ^5^UNC Project‐Malawi, Lilongwe, Malawi, ^6^University of Zimbabwe, Clinical Trials Research Centre, Harare, Zimbabwe, ^7^University of Cape Town, Desmond Tutu Health Foundation, Cape Town, South Africa, ^8^South African Medical Research Council, HIV and other Infectious Diseases Research Unit, Durban, South Africa, ^9^Botswana Harvard AIDS Institute Partnership, Gabarone, Botswana, ^10^UVRI‐IAVI, Entebbe, Uganda, ^11^Baylor College of Medicine Children's Foundation Uganda, Kampala, Uganda, ^12^Makerere University, Johns Hopkins University Research Collaboration, Kampala, Uganda, ^13^Columbia University Mailman School of Public Health, Eswatini Prevention Center, Mbabane, Eswatini, ^14^University of the Witwatersrand, Perinatal HIV Research Unit, Soweto, South Africa, ^15^KEMRI, Kisumu CRS, Kisumu, Kenya, ^16^FHI 360, Durham, United States, ^17^Johns Hopkins University School of Medicine, Department of Pathology, Baltimore, United States, ^18^Stroger Hospital of Cook County, Department of Psychiatry, Chicago, United States, ^19^National Institute for Allergy and Infectious Diseases, Division of AIDS, Washington DC, United States, ^20^Gilead Sciences, Foster City, United States, ^21^ViiV Healthcare, Durham, United States, ^22^University of North Carolina at Chapel Hill, Chapel Hill, United States

**Background: **HPTN 084 is an ongoing Phase 3 randomized, controlled trial that demonstrated the superiority of long‐acting injectable cabotegravir (CAB) compared to daily oral TDF/FTC for HIV prevention in individuals assigned female at birth. The blinded portion of the trial was stopped at a planned interim review in November 2020. Participants were subsequently unblinded and continued on their original randomised study regimen pending a protocol amendment to offer open‐label CAB.

**Methods: **We report on HIV infections detected in the 12‐month period following trial unblinding (11/5/20‐11/5/21, detected through 12/31/21) based on site and HPTN Laboratory Center testing. We estimated cumulative HIV incidence for the combined primary blinded and 12‐month unblinded follow‐up period, by study arm. Grade 2+ adverse events (AEs), injection site reactions (ISR), pregnancy incidence and outcomes are reported for the 12‐month post‐unblinding period only.

**Results: **Twenty‐three incident infections (3 CAB, 20 TDF/FTC) were detected in the 12‐month unblinded period. Of these, two (1 CAB, 1 TDF/FTC) were determined to have occurred during the blinded phase. Only one of the CAB cases (blinded phase case) had ever received an injection. Cumulatively, 62 incident HIV infections (6 CAB, 56 TDF/FTC) have been observed over 6626 person‐years of follow up (HIV incidence 0.94%, 95% CI 0.72, 1.20). The superiority of CAB appears sustained (HR 0.11, 95% CI 0.05, 0.24). No new safety concerns were identified. For the 12‐month unblinded period, 2.4% (32/1318) of participants in the CAB group reported a Grade 2+ ISR. Overall, Grade 2+ AEs in this period were balanced by study group; 20% were assessed as related to study product (CAB 19%, TDF/FTC 21%). Two deaths occurred in the CAB group; both were assessed as unrelated to study product. An additional 83 confirmed pregnancies (43 CAB, 40 TDF/FTC) occurred in the unblinded period (incidence 3.20%, 95% CI 2.56, 3.98). No congenital anomalies were reported.

**Conclusions: **Reductions in HIV incidence were sustained. CAB continues to be superior to TDF/FTC in preventing HIV infection in individuals assigned female at birth. Pregnancy incidence was higher in the unblinded period highlighting the importance of ongoing evaluations of CAB safety in pregnancy.

### PELBA01 Antibody‐mediated prevention of vaginal HIV transmission is dictated by IgG subclass in humanized mice

J. Brady^1^, M. Phelps^1^, S. MacDonald^1^, E. Lam^1^, A. Nitido^1^, D. Parsons^1^, C. Boutros^1^, C. Deal^1^, W. Garcia‐Beltran^1^, S. Tanno^1^, H. Natarajan^2^, M. Ackerman^2^, V. Vrbanac^1^, A. Balazs ^1^

^1^The Ragon Institute of MGH, MIT and Harvard, Cambridge, United States, ^2^Dartmouth College, Department of Microbiology, Hanover, United States

**Background: **HIV broadly neutralizing antibodies (bNAbs) are capable of both blocking viral entry and recruiting innate immunity to HIV‐infected cells through their fragment crystallizable (Fc) region. Vaccination or productive infection results in a polyclonal mixture of class‐switched IgG antibodies comprised of four subclasses, each encoding distinct Fc regions that differentially engage innate immune functions. Despite evidence that innate immunity contributes to protection, the relative contribution of individual IgG subclasses is unknown.

**Methods: **We constructed Adeno Associated Virus (AAV) vectors expressing VRC07, a potent CD4‐binding site direct bNAb, as each of the IgG1‐4 heavy chains. In vitro testing was performed to assess the functionality of each IgG subclass against multiple HIV strains. Vectored ImmunoProphylaxis (VIP) was administered to bone‐liver‐thymus (BLT) humanized mice to interrogate the efficacy of individual IgG subclasses during low‐dose repetitive vaginal HIV challenge by the REJO.c transmitted molecular founder strain of HIV.

**Results: **In vitro studies revealed that each IgG subclass exhibited distinct patterns of innate cell function. In vivo studies found that although IgG1, IgG3 and IgG4 exhibited similar protective efficacy, IgG2, which lacked Fc‐mediated functionality, exhibited significantly reduced protection. A Cox proportional hazards model found that IgG2 was 5.6‐fold less effective at preventing infection than IgG1 when controlling for antibody concentration. Interestingly, these studies also found that concentrations of VRC07‐IgG1 as low as 2 μg/mL yielded substantial protection against vaginal challenge.

**Conclusions: **Our results suggest that antibody dependent cellular cytotoxicity may be dispensable for protective efficacy in BLT humanized mice, but antibody dependent cellular phagocytosis may contribute substantially to prevention at low bNAb concentrations. As such, interventions capable of eliciting modest titers of functional subclasses may provide meaningful benefit against infection.

### PELBA02 First‐in‐human evaluation of safety and pharmacokinetics of intravenous or subcutaneous infusions of PGT121.141.LS, an anti‐V3 HIV‐1 broadly neutralizing antibody in healthy volunteers without HIV

S. Edupuganti ^1^, C. Hurt^2^, K. Stephenson^3^, Y. Huang^4^, C. Paez^4^, T. Gamble^5^, C. Yu^4^, C. Yen^6^, S. Regenold^6^, W. Chege^6^, R.J. Landovitz^7^, K.H. Mayer^8^, M. Siegel^9^, M.E. Sobieszczyk^10^, S.R. Walsh^11^, J. Heptinstall^12^, K. Seaton^12^, D. Montefiori^1^, G. Tomaras^12^

^1^Emory University, Medicine, Atlanta, United States, ^2^University of North Carolina at Chapel Hill, Medicine, Chapel Hill, United States, ^3^Beth Israel Deaconess Medical Center, Harvard Medical School, Center for Virology and Vaccine Research, Boston, United States, ^4^Vaccine and Infectious Disease Division, Fred Hutchinson Cancer Center, Seattle, United States, ^5^FHI 360, Durham, United States, ^6^National Institute of Allergy and Infectious Diseases, Rockville, United States, ^7^UCLA Center for Clinical AIDS Research & Education, Los Angeles, United States, ^8^Boston‐Fenway Health, Harvard Medical School, Boston, United States, ^9^George Washington Medical Faculty Associates, Washington, DC, United States, ^10^Columbia University Irving Medical Center, New York, United States, ^11^Brigham and Women's Hospital, Harvard Medical School, Boston, United States, ^12^Duke University, Durham, United States

**Background: **Multiple broadly neutralizing antibodies (bnAbs) targeting domains of gp120 are in development for prevention of HIV‐1. PGT121.414.LS, a modification of the anti‐V3 glycan bnAb PGT121, potently neutralizes multiple HIV‐1 clades *in vitro*.

**Methods: **The ongoing, phase 1 HVTN 136/HPTN 092 trial assesses the safety, tolerability, and pharmacokinetics (PK) of PGT121.414.LS in 13 healthy adults without HIV. We evaluated IV dose‐escalation and SC infusion in four groups: 3 mg/kg IV (group T1, n=3), 10 mg/kg IV (T2, n=4), 30 mg/kg IV (T3, n=3) and 5 mg/kg SC (T4, n=3). Serum concentrations of PGT121.414.LS were measured on days 0, 1, 2, 3, 6, 14, 28, 56 and 112 after a single infusion. Non‐compartmental PK analyses were performed.

**Results: **The median participant age was 30 years; 77% were assigned female sex at birth; 15% Black and 85% White. IV and SC infusions were safe and well‐tolerated, with no related serious adverse events or dose‐limiting toxicities. Peak concentrations after IV infusions were observed on day 1, increasing linearly with higher doses (median = 525.8 in T3 vs 164.7 μg/mL in T2). Peak concentrations after SC infusion occurred on day 14. On day 112 (trough visit), T1 and T4 concentrations were similar (12.1 and 13.7 μg/mL); T2 concentrations (31.3 μg/mL) were lower than those predicted for T3 (78.8 μg/mL). Day 112 concentrations for T3 are in progress. PGT121.414.LS estimated clearance was 0.06‐0.12 liter/day in T1‐T4. PGT121.414.LS estimated elimination half‐lives were 3 times longer than its precursor, PGT121, with medians of 53.6 ‐74.3 days in T1‐T4. The estimated bioavailability of SC PGT121.414.LS was 70%, twice the bioavailability of its precursor.

**Conclusions: **PGT121.414.LS was safe and well‐tolerated following IV or SC infusion in healthy US adults. These preliminary safety and pharmacokinetic findings support further development of PGT121.414.LS in combination with other bnAbs for global HIV‐1 prevention.

**Figure:**
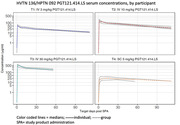
**Abstract PELBA02‐Figure 1**.

### PELBA03 *In‐vitro/ ex‐vivo* contribution of antiretroviral drug and alcohol exposure to blood‐brain barrier disruption: relevance to the pathogenesis of HIV‐1 associated neurological disorder

S.(. Huang ^1^, M.T. Hoque^1^, R. Bendayan^1^

^1^University of Toronto, Pharmaceutical Sciences, Toronto, Canada

**Background: **HIV‐1‐associated neurological disorder (HAND) and alcohol use disorder (AUD) are common among people living with HIV/AIDS and significantly compromise their quality of life. Lines of evidence suggest that the toxicity induced by alcohol and antiretroviral drugs (ARVs) in the central nervous system (CNS) can potentially contribute to the pathogenesis of HAND. We aimed to investigate the effect of several recommended first‐line ARVs and alcohol in inducing blood‐brain barrier (BBB) dysfunction using human and mouse*in‐vitro* and *ex‐vivo* BBB models.

**Methods: **Brain Microvascular endothelial cells of human (hCMEC/D3) and mouse (primary cultures) origin, and isolated mice brain capillaries were used as in‐vitro and ex‐vivo models of the BBB and treated with alcohol or ARVs at clinically relevant plasma concentrations. Gene expression of tight junction proteins, drug efflux transporters, pro‐inflammatory cytokines and oxidative stress marker were analyzed by qPCR using TaqMan gene expression assays.

**Results: **We observed a significant downregulation of TJP1/ZO‐1, OCLN and CLDN5 mRNA expression following efavirenz, dolutegravir and emtricitabine exposure, and upregulation of pro‐inflammatory cytokines (IL6, IL1β, IL8) and oxidative stress marker (NOS2/iNOS) by efavirenz, dolutegravir, emtricitabine, bictegravir and abacavir treatment in hCMEC/D3 cells. The mRNA expression of drug efflux transporters, P‐glycoprotein (ABCB1/P‐gp), Breast Cancer Resistant Protein (ABCG2/BCRP), and glucose transporter1 (SLC2A1/Glut‐1) was altered by efavirenz, dolutegravir, emtricitabine, bictegravir and abacavir. In mouse brain microvessel endothelial cells, alcohol, efavirenz and dolutegravir exposure significantly downregulated Tjp1, Ocln and Cldn5, upregulated Il6, and altered Abcb1a, Abcg2 and Slc2a1 gene expression. Experiments using mouse brain capillaries further confirmed the significant effect of efavirenz and dolutegravir in disrupting Tjp1, Ocln, Cldn5, inducing Il1β and downregulating Abcb1a, Abcg2 and Slc2a1 mRNA expression.

**Conclusions: **Our studies demonstrated the effect of alcohol and ARVs in dysregulating TJ proteins, drug efflux transporters and pro‐inflammatory cytokines at the BBB. By assessing the CNS toxicity of first line ARVs, our research reveals the potential contribution of ARVs in the pathogenesis of HAND and provides insights into optimal use of such ARVs in AUD population.(Supported by CIHR and OHTN).

### PELBB01 Final week 192 results from the ADVANCE trial: first‐line TAF/FTC/DTG, TDF/FTC/DTG vs TDF/FTC/EFV

W.D.F. Venter^1^, B. Bosch^1^, S. Sokhela^1^, G. Akpomiemie^1^, N. Chandiwana^1^, A. Tembo^1^, A. Qavi^2^, B. Simmons^3^, K. McCann^4^, A. Hill ^5^

^1^Ezintsha, University of the Witwatersrand, Johannesburg, South Africa, ^2^Imperial College London, London, United Kingdom, ^3^London School of Economics and Political Science, London, United Kingdom, ^4^University of Connecticut, Connecticut, United States, ^5^University Of Liverpool, Liverpool, United Kingdom

**Background: **Current World Health Organization guidelines recommend first‐line treatment with tenofovir disoproxil fumarate (TDF)/lamivudine (or emtricitabine (FTC)) and dolutegravir /(DTG) for HIV‐1 infection.Tenofovir alafenamide (TAF) is listed as an alternative to TDF for patients with osteoporosis or impaired renal function.

**Methods: **1,053 treatment‐naïve participants in South Africa were randomized to either TAF/FTC+DTG, TDF/FTC+DTG, or TDF/FTC/EFV and followed up to week 192 under a trial extension. HIV‐1 RNA, vital signs and renal and bone adverse events were assessed prospectively.

**Results: **BMI was balanced between arms at baseline. At 192 weeks, HIV‐1 RNA <50 copies/mL was confirmed in 218/351 (62.3%) in the TAF/FTC+DTG arm, 204/351 (58.1%) in the TDF/FTC+DTG arm, and 177/351 (50.4%) in the TDF/FTC/EFV arm. In the on treatment analysis, HIV RNA <50 copies/mL was 218/226 (96%) for TAF/FTC+DTG, 204/209 (98%) for TDF/FTC+DTG, and 177/179 (99%) for TDF/FTC/EFV. By Week 192, body weight increased by +8.9kg for TAF/FTC+DTG, +5.8kg for TDF/FTC+DTG, and +3.3kg for TDF/FTC/EFV participants (observed data analysis). By Week 192, 29% of patients on TAF/FTC+DTG, 21% on TDF/FTC+DTG and 15% on TDF/FTC/EFV had developed clinical obesity.The risk of clinical obesity was significantly higher if taking TAF/FTC/DTG (p<0.001), female patients (p<0.001) and those with higher baseline BMI (p<0.001). Among the women enrolled, 43% on TAF/FTC+DTG developed clinical obesity by Week 192 versus 27% on TDF/FTC+DTG and 20% taking TDF/FTC/EFV (p<0.001).Bone fracture and Grade 3 or 4 renal adverse events were rare and similar across arms.

**Conclusions: **In the ADVANCE trial, participants taking TAF/FTC+DTG experienced greater weight gain and clinical obesity than TDF/FTC/DTG by Week 192, particularly in women, but no significant differences in HIV RNA suppression or renal or bone‐related adverse events. Both TAF/FTC/DTG and TDF/FTC/DTG had significantly higher rates of HIV RNA suppression than TDF/FTC/EFV at Week 192 in the main ITT analysis.

**Figure:**
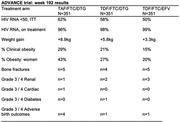

### PELBB02 Update on neural tube defects with antiretroviral exposure in the Tsepamo Study, Botswana

R. Zash ^1,2^, L.B. Holmes^3^, M. Diseko^2^, D. Jacobson^4^, G. Mayondi^2^, J. Mabuta^2^, M. Mmalane^2^, T. Gaolathe^5^, S. Lockman^6,2^, J. Makhema^2^, R. Shapiro^4,2^

^1^Beth Israel Deaconess Medical Center, Boston, United States, ^2^Botswana Harvard AIDS Institute Partnership, Gaborone, Botswana, ^3^MassGeneral Hospital for Children, Boston, United States, ^4^Harvard TH Chan School of Public Health, Boston, United States, ^5^University of Botswana, Gaborone, Botswana, ^6^Brigham and Women's Hospital, Boston, United States

**Background: **After reporting a possible association between neural tube defects (NTDs) and exposure to dolutegravir (DTG) from conception in 2018, yearly updates from the Tsepamo study have been increasingly reassuring. We report updated data collected through March 2022.

**Methods: **The Tsepamo Study conducts birth outcomes surveillance study at government hospitals throughout Botswana, covering ∼70% of all births. Midwives perform surface examinations of all live births and stillbirths and describe abnormalities. Research assistants photograph major abnormalities after maternal consent, which are reviewed by a birth defects expert blinded to exposures. Prevalence of NTDs was determined by maternal HIV and antiretroviral (ARV) exposure status (95% CI by Wilson method) and the primary analysis evaluated prevalence differences by exposure status (95% CI by Newcombe method).

**Results: **Since April 2021, 32,819 additional births were recorded, including 3,780 additional DTG conception exposures. Since August 2014, there have been a total of 224,251 deliveries; 223,797 (99.8%) had an evaluable infant surface exam, with 156 (0.07%, 95% CI 0.06%, 0.08%) NTDs identified (100 with photo, 56 by description only). Among women on DTG at conception, 10/5860 NTDs occurred (0.11%; 95%CI 0.06%, 0.19%): 4 myelomeningoceles, 2 anencephaly, 3 encephaloceles, and 1 iniencephaly. In comparison, NTDs occurred in 25/23,664 (0.11%; 95%CI 0.07%, 0.16%) women on any non‐DTG ARVs from conception, 11/14,432 (0.08%; 95%CI 0.04%, 0.14%) on efavirenz from conception, 4/6,551 (0.06%; 95%CI 0.02%, 0.16%) on dolutegravir started in pregnancy, and 108/170,723 (0.07%; 95%CI 0.05, 0.08%) among women without HIV. NTD prevalence did not differ between DTG and any non‐DTG ARVs from conception (0.00% difference; 95%CI ‐0.07%, 0.10%).

Table. Prevalence Difference of Neural Tube Defects by ARV and HIV Exposure Categories

**Table:** 

**Abstract PELBB02‐Table 1**.	
Exposure group vs. comparison group	Prevalence difference (%) (95% CI)
DTG at conception vs. Non‐DTG at conception	0.00 (‐0.07, 0.10)
DTG at conception vs. EFV at conception	0.03 (‐0.05, 0.12)
DTG at conception vs. DTG started in pregnancy	0.04 (‐0.06,0.14)
DTG at conception vs. non‐DTG started in pregnancy	0.04 (‐0.07,0.13)
DTG at conception vs. women without HIV	0.04 (‐0.01, 0.13)

**Conclusions: **The prevalence of NTDs among infants born to women on DTG at conception has declined to 0.11% and does not substantially differ from other exposure groups.

### PELBC01 Botswana achieved the Joint United Nations Programme on HIV/AIDS (UNAIDS) 95‐95‐95 targets: results from the Fifth Botswana HIV/AIDS Impact Survey (BAIS V), 2021

M. Mine ^1^, K. Stafford^2^, R.L. Laws^3^, R. Marima^4^, P. Lekone^5^, D. Ramaabya^1^, K. Makhaola^5^, P. Mapondera^4^, F. Wray‐Gordon^3^, C. Agbakwuru^2^, L. Okui^4^, E. Onyadile^6^, J. Ngidi^1^, A. Abimiku^2^, K. Bagapi^5^, B. Nkomo^1^, S. Bodika^3^, S.Y. Hong^5^, S. Matroos^6^, M. Charurat^2^, R. Selato^7^, A.C. Voetsch^3^, BAIS V group

^1^Ministry of Health and Wellness, Gaborone, Botswana, ^2^Ciheb, School of Medicine, University of Maryland Baltimore, Baltimore, United States, ^3^Division of Global HIV and TB, Centers for Disease Control and Prevention, Atlanta, United States, ^4^Ciheb, MGIC‐Botswana an affiliate of University of Maryland Baltimore, Gaborone, Botswana, ^5^Division of Global HIV and TB, Centers for Disease Control and Prevention, Gaborone, Botswana, ^6^Statistics Botswana, Gaborone, Botswana, ^7^National AIDS and Health Promotion Agency, Gaborone, Botswana

**Background: **In 2002, Botswana was the first African country to offer free HIV treatment to citizens. Since then, Botswana has expanded treatment coverage and adopted evidence‐based practices, including test‐and‐start and dolutegravir treatment. The BAIS V survey was used to measure national progress toward UNAIDS 95‐95‐95 targets (percent of persons living with HIV (PLHIV) aware of status, on treatment, virally suppressed).

**Methods: **BAIS V used a two‐stage cluster design to obtain a nationally representative sample of adults 15–64 years. During March–August 2021, survey teams consented 14,763 participants in their households, administered questionnaires, and tested blood specimens for HIV. Viral load and presence of antiretrovirals (ARVs) in blood were measured. The first and second 95 estimates were based on self‐report and adjusted for detectable ARVs. Viral load suppression (VLS) was defined as HIV RNA <1,000 copies per milliliter. Data were weighted to account for complex survey design, and jackknife methods were used to estimate variance.

**Results: **National HIV prevalence was 20.8% (men: 15.2%; women: 26.2%). Among PLHIV, 95.1% (men: 93.0%; women: 96.4%) were aware of their status, 98.0% (men: 97.2%; women: 98.4%) of those aware were on ART, and 97.9% (men: 96.6%; women: 98.6%) of those on ART achieved VLS (Table). Among PLHIV 15–24 years, 84.5% were aware of their status, 98.5% of those aware were on ART, and 91.6% of those on ART achieved VLS. VLS among all PLHIV was 91.8% (men: 88.1%; women: 94.0%).

**Table:** 

**Abstract PELBC01‐Table 1**.						
	Aware of Status N; Weighted % (95% CI)		On Treatment N; Weighted % (95% CI)		Virally Suppressed N; Weighted % (95% CI)	
**Men, 15–64 years**	989	93.0 (90.8–95.2)	920	97.2 (95.7–98.8)	899	96.6 (95.2–98.0)
15–24 years	39	89.1 (77.3–100.0)	34*	100.0 (100.0–100.0)	34	91.8 (83.9–99.8)
25–44 years	360	88.7 (85.0–92.5)	315	94.2 (90.4–98.0)	302	94.9 (91.5–98.4)
45–64 years	590	96.4 (93.6–99.2)	571	99.2 (98.4–100.0)	563	98.0 (97.2–98.8)
**Women, 15–64 years**	2,428	96.4 (95.0–97.7)	2,342	98.4 (97.5–99.2)	2,309	98.6 (98.0–99.2)
15–24 years	118	82.3 (70.1–94.5)	99	97.8 (94.4–100.0)	96	91.5 (84.2–98.8)
25–44 years	1,302	97.1 (95.9–98.2)	1,256	98.4 (97.7–99.1)	1,235	98.8 (98.2–99.4)
45–64 years	1,008	97.0 (95.0–99.0)	987	98.3 (96.3–100.0)	978	99.1 (97.9–100.0)
**Total, 15–64 years**	3,417	95.1 (93.8–96.5)	3,262	98.0 (97.2–98.7)	3,208	97.9 (97.2–98.6)

**Conclusions: **BAIS V is the first population‐based survey to confirm achievement of UNAIDS 95‐95‐95 goals, overall and among women. Men have achieved the second and third 95 targets and surpassed 90% for the first. Gaps remain in awareness among men 25–44 years and younger adults, particularly young women. Botswana has made tremendous progress in 20 years and is well‐positioned to end the AIDS epidemic by 2030.

### PELBC02 Identifying women at highest risk for HIV acquisition across sub‐Saharan Africa: a risk assessment tool based on machine‐learning methods

N.E. Rosenberg^1^, A. Young ^2^, M. Liu^2^, B.E. Shook‐Sa^2^, N.A. Sam‐Agudu^3^, L. Stranix‐Chibanda^4^, M. Yotebieng^5^, M.E. Charurat^6^, J.E. Justman^7^, B.H. Chi^8^

^1^University of North Carolina at Chapel Hill, Health Behavior, Chapel Hill, United States, ^2^University of North Carolina at Chapel Hill, Biostatistics, Chapel Hill, United States, ^3^University of Maryland School of Medicine, Institute of Human Virology, Baltimore, United States, ^4^University of Zimbabwe Faculty of Medicine, Child and Adolescent Health Unit, Harare, Zimbabwe, ^5^Albert Einstein College of Medicine, Bronx, United States, ^6^University of Maryland School of Medicine, Institute for Human Virology, Baltimore, United States, ^7^Columbia University, New York, United States, ^8^University of North Carolina at Chapel Hill, Obstetrics and Gynecology, Chapel Hill, United States

**Background: **Women in sub‐Saharan Africa disproportionately acquire HIV. Effective HIV prevention interventions are available, but identification of women in greatest need of these interventions remains a challenge. To date, HIV risk scores to identify those at highest risk of HIV have been based on non‐representative populations, have limited geographic applicability, and have demonstrated sub‐optimal performance with area under the receiver operating characteristic curve (AUC) <0.80.

**Methods: **We sought to develop a regionally representative risk assessment tool to identify women at highest risk of HIV‐1 acquisition across multiple high‐burden African countries. We pooled, weighted, and analyzed data from Population‐based HIV Impact Assessment surveys (PHIAs) from 13 countries in Southern, Eastern, and West/Central Africa. The population comprised 15‐49 year‐old women who were HIV‐uninfected or had recent HIV‐1 infection characterized by HIV‐Limiting Antigen Avidity enzyme immunoassay, HIV‐1 viral load, and antiretroviral drug concentrations. Least absolute shrinkage and selection operator (LASSO) regression models were implemented to create a predictive model for recent HIV‐1 infection based on 23 potential variables. The model was trained in 70% of the sample and tested in the remaining 30%. Model performance was evaluated using AUC. Optimal sensitivity and specificity were reported.

**Results: **Among 164,935 participants, representing 82.2 million women, 200 had evidence of recent HIV‐1 infection. Twelve variables were retained in the LASSO model and were predictive of elevated risk: 1) age 25‐34 years, 2) any educational attainment, 3) divorce/separation or widowhood, 4) age at first sex <18 years, 5) >2 recent sexual partners, 6) partner with unknown HIV status, 7) relationship entered for financial support 8) receipt of money/goods from sexual partner, 9) recent condom use, 10) use of short‐acting contraception, 11) past pregnancy, and 12) sub‐national male HIV prevalence. Model AUC was 0.81 (95% confidence interval (CI): 0.77‐0.85) in the training sample and 0.80 (CI: 0.75‐0.85) in the testing sample. The optimal threshold had 80.0% (CI: 67.7%‐89.2%) sensitivity and 71.0% (CI: 70.6%‐71.4%) specificity.

**Conclusions: **Using national surveys representing 82.2 million women, our machine learning tool outperformed earlier models and identified a set of characteristics that was highly predictive of HIV risk, identifying women who would benefit most from prevention interventions.

### PELBD01 #*SafeHandsSafeHearts*: a randomized waitlist‐controlled trial of an eHealth intervention to increase COVID‐19 knowledge and protective behaviors and reduce psychological distress among LGBTQ+ people in India and Thailand

P. Newman^1^, V. Chakrapani ^2,3^, S. Tepjan^4^, P. Akkakanjanasupar^4^, S. Rawat^3^, N. Massaqoui^5^, C. Williams^1^, S. Waewklaihong^6^, B. Mohan^3^, G. Carl^6^, S. Pengnonyang^6^, S. Roungprakhon^7^, N. Phanuphak^6^

^1^University of Toronto, Factor‐Inwentash Faculty of Social Work, Toronto, Canada, ^2^Centre for Sexuality and Health Research and Policy, Chennai, India, ^3^The Humsafar Trust, Mumbai, India, ^4^VOICES‐Thailand Foundation, Chiang Mai, Thailand, ^5^University of Toronto Scarborough, Department of Health and Society, Scarborough, Canada, ^6^Institute of HIV Research and Innovation (IHRI), Bangkok, Thailand, ^7^Rajamangala University of Technology Phra Nakhon, Computer Technology, Bangkok, Thailand

**Background: **Lesbian, gay, bisexual, transgender, queer, and other sexual/gender minority (LGBTQ+) people are at heightened vulnerability for COVID‐19 morbidity and mortality due to adverse social determinants of health, and pervasive health and mental health disparities. We tested a theory‐based, culturally‐tailored, peer‐delivered eHealth intervention to increase COVID‐19 knowledge and protective behaviors and reduce psychological distress among LGBTQ+ people in Bangkok and Mumbai.

**Methods: **#SafeHandsSafeHearts is a multisite, pragmatic, randomized waitlist‐controlled trial with 1:1 (immediate intervention group [IIG]: waitlist control [WLC]) allocation (ClinicalTrials.gov NCT04870723). Eligibility criteria: self‐identified LGBTQ+, ≥18‐years‐old, resident in Bangkok or Mumbai. Participants recruited from LGBTQ+ listservs and social media completed mobile‐optimized baseline, 2‐week post‐intervention, and 2‐month follow‐up surveys. The motivational interviewing‐ and psychoeducation‐based intervention was implemented in 3 biweekly 45‐minute online sessions by trained peer counselors. Primary outcome measures: COVID‐19 knowledge and protective behavior (e.g., masking, physical distancing) scores based on US CDC items, PHQ‐2 (depressive symptoms), and GAD‐2 (anxiety symptoms). We used multilevel models, accounting for clustering at participant‐ and country‐levels, to estimate outcomes.

**Results: **From August 2021 to February 2022, participants (n = 650; median age=29.0 years; IQR=10) completed the baseline assessment and were randomized to IIG (n = 320; 49.2%) and WLC conditions (n = 330; 50.8%). 531 (81.7%) completed post‐intervention and 452 (69.5%) completed follow‐up assessments. Compared to WLC, the IIG had statistically significant improvements in COVID‐19 knowledge scores at post‐intervention (b=.34; 95% CI .18, .50; p<.001) and follow‐up (b=.28; 95% CI .04, 51; p=.01), and COVID‐19 protective behavior scores at post‐intervention (b=1.12; 95% CI .39, 1.85; p<.01) and follow‐up (b=1.00; 95% CI .51, 1.49; p<.001). Compared to WLC, the IIG had a statistically significant reduction in depression scores at post‐intervention (b=‐.18; 95% CI ‐.22, ‐.14; p<.001) and follow‐up (b=‐.23; 95% CI ‐.35, ‐.10; p<.001), with no significant reduction in anxiety scores.

**Conclusions: **Among LGBTQ+ adults in Bangkok and Mumbai, a tailored, peer‐delivered eHealth intervention is effective in increasing COVID‐19 knowledge and protective behaviors and reducing depression. The pragmatic multisite trial design and implementation during a pandemic supports the ecological validity of the findings and suggests #SafeHandsSafeHearts may be effective among LGBTQ+ adults in other middle‐ and high‐income countries.

### PELBD02 Implementation of the first PrEP counseling and adherence protocol in a country with high need for and low access to biomedical HIV prevention

C. Lelutiu‐Weinberger ^1,2^, M. Filimon^1,2^, M. Lixandru^3^, R. Jipa^4^, C. Fierbințeanu^3^, I. Filipescu^5^, M. Bora^5^, L. Hanu^3^, S. Luculescu^6^, L. Hightow‐Weidman^7^, S. Golub^8^, A. Rochelle^7^, N. Buckner^9^, A. Streinu‐Cercel^4^, C. Jianu^5^, J. Pachankis^10^

^1^Rutgers University, Rutgers Biomedical and Health Sciences, Newark, United States, ^2^Columbia University, School of Nursing, New York, United States, ^3^The Romanian Association Against AIDS, București, Romania, ^4^The National Institue of Infectious Diseases Matei Balș, București, Romania, ^5^University of Medicine and Pharmacy Iuliu Hațieganu, Cluj‐Napoca, Romania, ^6^The Romanian Association Against AIDS, Cluj‐Napoca, Romania, ^7^University of North Carolina, Department of Medicine, Chapel Hill, United States, ^8^Hunter College, Department of Psychology, New York, United States, ^9^One Cow Standing, Durham, United States, ^10^Yale University, School of Public Health, New Haven, United States

**Background: **PrEP is not prescribed in Romania, yet the country demonstrates increasing HIV incidence, primarily driven by gay and bisexual men (GBM). Romania displays some of the highest levels of homophobia in Eastern Europe, and high PrEP demand among GBM. This study evaluates PrEP implementation rollout within the Romanian national healthcare system.

**Methods: **In Phase 1, the ADAPT‐ITT Model was used to tailor and integrate two US evidence‐based interventions for pre‐exposure prophylaxis (PrEP) implementation (SPARK, brief sex‐positive motivational counseling, and P3 [Prepared, Protected, emPowered], social networking gamified app with text‐based adherence counseling) to the local context with physicians, psychologists, and GBM. In Phase 2, *PrEP Romania* was theater‐tested in clinics in Bucharest and Cluj‐Napoca with 10 high risk GBM for 1‐month. In Phase 3, *PrEP Romania* was pilot‐tested with 20 GBM in a single‐arm pilot for *feasibility* (medical visit attendance), *acceptability* (protocol feedback, app usability), and PrEP *uptake* (filled prescriptions), *adherence* (self‐reported and dried blood spot samples [DBS]) and *persistence* (still on PrEP) after 3‐months.

**Results: **All 30 PrEP candidates (biological males; *M* age=29.17; *SD*=9.28; range=19‐53) attended medical visits and initiated PrEP, and 29/30 continued PrEP through follow‐up. All participants tracked medication use on the app (average 4/week dosing), and 98% daily PrEP adherence on the Timeline Followback. All 30 DBS samples were collected and frozen adequately (at ‐20 degrees Celsius) at baseline; 10/10 DBS samples were collected at the 1‐month and 18/20 at 3‐month follow‐ups. DBS blood plasma concentration indicating ≥4/week dosing (TFVdp concentrations ≥1,000 fmol) were found in 100% of 1‐month and 83% of 3‐month samples. On average, participants accessed the app once daily, 6 times/week, for 13 minutes/week, and read 3/4 articles and completed 3/4 activities monthly. The average intervention acceptability score by staff was 4.9 (*SD*=0.2) (out of 5) and 75 (out of 100) by participants (≥68=acceptable).

**Conclusions:** *PrEP Romania* is an acceptable and feasible hybrid in‐person + mHealth PrEP uptake and adherence program, which may empower GBM and healthcare systems in stigmatizing settings to adopt biomedical prevention. Upon future efficacy testing, this protocol blueprint can support PrEP rollout in countries with similar levels of unpreparedness for biomedical prevention.

### PELBE01 Pre‐exposure prophylaxis for people who inject drugs: opportunities to expand the program in Ukraine

I. Sazonova ^1^, R. Kulchynska^1^, I. Doan^1^, R. Keating^1^, S. Ohorodnik^2^, S. Salnikov^2^, I. Titar^2^, M. Azarskova^1^, E.J. Barzilay^1^

^1^CDC‐Ukraine, Overseas Strategy and Management Branch, Division of Global HIV & TB, U.S. Centers for Disease Control and Prevention, Kyiv, Ukraine, ^2^Center of Public Health of the Ministry of Health of Ukraine, Kyiv, Ukraine

**Background: **HIV has a disproportionate burden on people who inject drugs (PWID) in Ukraine. HIV prevalence in the PWID population is 20 times higher than the national estimate. Rough estimates suggest that 275,000 HIV‐negative PWID are at risk of HIV acquisition due to continuing injecting practices. Pre‐exposure prophylaxis (PrEP) is an effective biomedical intervention that prevents sexual transmission of HIV and reduces HIV incidence. The World Health Organization recommends that PrEP be offered to PWID with increased sexual risk behavior. This study explored PrEP awareness, willingness, and eligibility among PWID in Ukraine.

**Methods: **We analyzed data from a 2020 PWID bio‐behavioral surveillance (BBS) survey conducted in 12 cities in Ukraine. The BBS used a cross‐sectional study design and a respondent‐driven sampling (RDS) approach to recruit 6,001 participants who were PWID. We assessed their sexual risks, their awareness, use, and willingness for PrEP. In addition, using existing PWID population size estimates, we assessed the number of PWID eligible for PrEP and willing to enroll in the program.

**Results: **Nearly 80% of the PWID participants were HIV‐negative. Among them, 10% had previously heard about PrEP, and 1.4% had experience using PrEP. 35.1% of the HIV‐negative PWID reported having either increased sexual risk behavior in the past 30 days (i.e., multiple sexual partners or condomless sex with casual or exchange partners) or diagnosis of a sexually transmitted infection (i.e., syphilis, chlamydia, gonorrhea, herpes, papillomavirus). Among this group of PWID with increased sexual risk behavior or diagnosis of an STI, 34.7% expressed willingness to use PrEP. PrEP willingness varied substantially by geography (ranging from 3.3% in Odesa to 22.6% in Kyiv city). Female sex, unmarried status, a low level of education, and stimulant drug use were associated with greater PrEP willingness. We estimate that at least 33,000 active PWID are eligible for PrEP and have expressed interest in participating in the program.

**Conclusions: **There is a considerable opportunity to expand the PrEP program among PWID in Ukraine. HIV community testing programs might benefit from the development of more efficient pathways to refer and link eligible HIV‐negative PWID to PrEP services.

### PELBE02 Community led monitoring in PEPFAR Vietnam ‐ innovation and lessons learned

H. Hoang ^1^

^1^PEPFAR Coordination Office, Vietnam, U.S. Department of State, Hanoi, Viet Nam

**Background: **In 2020 PEPFAR Headquarters required all PEPFAR bilateral and regional programs to initiate in fiscal year 2021 community led monitoring (CLM). However there was very little of guidance from OGAC and no concrete example of a commonly considered "successful CLM model". Taking a serious and comprehensive research of available examples of community monitoring models and an innovative and inclusive approach that ensured the "community led" principle, the PEPFAR program in Vietnam worked closely with community representatives to launch a unique and unprecedented CLM model that now turns out to be an efficient mechanism for community feedback of PEPFAR supported services, highly appreciated by OGAC, and closely watched by regional and international civil society alliances.

**Description: **A year before CLM actually started, in coordination with in‐country stakeholders, the PEPFAR team gathered a task‐force of 15 community leaders who had rich experience in service delivery, advocacy and quantitative and qualitative research and a comprehensive understanding of the PEPFAR program. They worked out a comprehensive CLM protocol, together with concrete data collection tools for PEPFAR supported sites which were then presented in a stakeholder workshop for feedback and mutual understanding and agreed implementation principles. The model was then finalized and budgeted in COP20 for implementation in fiscal year 2021.

**Lessons learned: **The approach up to now is proved to be an efficient mechanism, securing the "community led" nature. While in most other PEPFAR programs, the independence nature of CLM is a concern, the Vietnam model is gradually and increasingly acknowledged by the host government, in‐country stakeholders, OGAC, and regional alliances. The concept of a PEPFAR funded yet independent CLM model is now widely understood by both government offices, healthcare workers, program managers, and beneficiaries.

**Conclusions/Next steps: **Available CLM data is currently at an extent that provides only snap‐shot of surveyed sites, and not representational enough to tell stories of provinces, regions, and national trends. At the global level, OGAC is supposed to draw and share widely CLM lessons from countries, particularly approaches, granting mechanisms, cost norms, and data sharing and usage. There is also expectation for CLM to be taken up in the Global Fund and national programs.

### PELBF01 HIV criminalization laws, public health trust, and uptake of COVID‐19 vaccine among people living with HIV in the United States

T. Mckay ^1^, J.C. Henne^2^, N. Kari^1^, T. Haught^3^, S. Strub^3^

^1^Vanderbilt University, Department of Medicine, Health, and Society, Nashville, United States, ^2^The Henne Group, Inc., San Francisco, United States, ^3^The Sero Project, Milford, United States

**Background: **HIV criminalization creates significant tensions in public health practice. The negative effects of HIV criminalization on HIV stigma, disclosure, testing, and engagement in care are well documented. In this study, we examine the effects of HIV criminalization laws on trust in public health and uptake of COVID‐19 vaccination among people living with HIV (PLWH) in the US.

**Methods: **We use the 2021 National HIV Criminalization Survey conducted by The Sero Project and community partners (N=610). Participants were recruited via community organizations by and for PLWH and invited to participate in an online survey from 18 August 2021 to 31 December 2021. We restrict analyses to PLWH who are 18 and older, reside in the United States, and provided information on COVID‐19 vaccination status (N=554).

**Results: **Just over half (54.6%) of PLWH lived in states with an HIV‐specific criminalization law. Over one‐third (36.7%) endorsed the statement “Public health professionals care more about enforcing laws that criminalize HIV transmission than they do about my health,” with endorsement higher among PLWH in states with HIV criminalization laws (40.4% vs 32.3%; χ^2^=4.226, *p*<.05). PLWH living in a state with HIV criminalization laws were significantly more likely to have heard of anyone in the state being arrested for nondisclosure of HIV status to a sexual partner (46.8% vs 28.2%; χ^2^=22.742, *p*<.001) and to personally know anyone accused, threatened, or arrested on an HIV‐related charge (40.1% vs 18.7%; χ^2^=32.996, *p*<.001). Uptake of COVID‐19 vaccination among PLWH was high at 90.3%, but we observe significantly lower uptake in states that had HIV criminalization laws (87.4% vs 93.7%; χ^2^=6.203, *p*<.05). In a logistic regression model adjusting for differences in vaccine uptake across individual characteristics (gender, race, age, diagnosis year, employed or volunteer in HIV work), survey timing, and geographic region, PLWH in states with HIV criminalization laws were more than 50% less likely to be fully vaccinated for COVID‐19 (OR= 0.478; 95% CI=0.233‐0.982).

**Conclusions: **Preventing HIV and COVID‐19 require trust in public health measures and practitioners. HIV‐specific criminalization laws jeopardize trust in public health and are associated with lower uptake of COVID‐19 vaccination among PLWH in the US.

### PELBF02 Follow the science ‐ addressing resistance to comprehensive sexuality education: lessons from five African countries

P. Machawira ^1^, R. Lovich^2^

^1^UNESCO, Harare, Zimbabwe, ^2^Consultant, Fairfield, United States

**Background: **Every week, 4200 girls and women aged 15‐24 in sub‐Saharan Africa acquire HIV (UNAIDS). Each one of these infections could be prevented if young people had accurate information about how HIV is transmitted, alongside the services they need to make empowered choices. In recent years, countries within the African region have experienced escalating levels of controversy and opposition surrounding Comprehensive Sexuality Education (CSE). Local concerns from religious and socially conservative entities related to the content covered and debates about the information that young people should have. CSE aims to empowers young people to build agency, develop skills, knowledge and attitudes required for preventing HIV, reducing early and unintended pregnancies, and eliminating gender‐based violence. Driven by the need for understanding the arguments of the opposition, UNESCO commissioned a study to document the experiences of five countries; Ghana, Namibia, South Africa, Uganda and Zambia with the objective to provide recommendations for governments and partners to respond to it.

**Description: **A mixed‐methods approach was used to gather information to inform the development of five case studies, consisting of a desk review, KIIs, and stakeholder analysis. Background information was gathered through a questionnaire completed by key stakeholders. Data was also collected using social listening methods from open access social media sites. Feedback sessions with case study participants were conducted to validate findings and probe for additional detail when needed. Findings were released in April 2022.

**Lessons learned: **Opposition tactics include disinformation; use of media to amplify reach of internationally generated materials; personalize attacks and politization of CSE; coordination of messages with specific events. Country response was efficient when (1)government is supported and takes firm ownership of CSE, (2)CSOs are engaged and contribute to build public support, (3)contextually relevant and timely communications strategy is in place, (3)mobilization of youth voices by CSOs.

**Conclusions/Next steps: **Focus on ensuring a government‐led response where they “take charge of the narrative” was essential. It is key to anticipate opposition and prepare for a coordinated response. Engage stakeholders, formulate common messaging on CSE, share evidence, develop a robust communication strategy using positive stories how CSE has helped learners, plan when and how to disseminate messages.

### EPLBA01 Protective potential of non‐neutralizing mAbs targeting immunodominant Env epitopes against tier‐2 HIV‐1

J. Klingler ^1,2^, X. Liu^1^, C. Upadhyay^1^, G. Li^3,4^, L. Su^3,4,5^, G. Hu^6^, S. Wang^6^, S. Lu^6^, S. Zolla‐Pazner^1^, C.E. Hioe^1,2^

^1^ICAHN School of Medicine at Mount Sinai, Division of Infectious Diseases, Department of Medicine, New York, United States, ^2^James J. Peters VA Medical Center, New York, United States, ^3^University of Maryland School of Medicine, Laboratory of Viral Pathogenesis and Immunotherapy, Division of Virology, Pathogenesis, and Cancer, Institute of Human Virology, Department of Pharmacology, Baltimore, United States, ^4^University of North Carolina, Lineberger Comprehensive Cancer Center, Department of Microbiology and Immunology, Chapel Hill, United States, ^5^University of Maryland School of Medicine, Laboratory of Viral Pathogenesis and Immunotherapy, Division of Virology, Pathogenesis and Cancer, Institute of Human Virology, Departments of Pharmacology and Microbiology & Immunology, Baltimore, United States, ^6^University of Massachusetts Medical School, Department of Medicine, Worcester, United States

**Background: **A robust non‐neutralizing antibody (Ab) response to the immunogenic regions of the gp120 envelope (Env) protein was identified as an immune correlate for reduced risk of HIV acquisition in the RV144 vaccine trial, which showed a vaccine efficacy of 31%. Our initial study showed that mAbs against V3 (2219) with poor neutralizing activities displayed protective capacity upon passive transfer to humanized mice that received a rectal challenge of an infectious molecular clone (IMC) expressing a tier‐2 Env of JRFL. To understand the immune functions contributing to protection, we examined the Fab‐ and Fc‐mediated activities of 2219 and other mAbs targeting similarly immunogenic epitopes of Env.

**Methods: **To evaluate the Fab functions of vaccine‐mediated mAbs against tier‐2 HIV‐1, we examined 1) ELISA reactivity of 2219 mAb from an infected individual versus two vaccine‐induced mAbs with solubilized Env of JRFL and REJO IMCs, 2) their neutralization capacities. To investigate their Fc‐mediated activities, we assessed 1) antibody‐dependent cell‐mediated cytotoxicity (ADCC), 2) antibody‐dependent cell‐mediated phagocytosis (ADCP), 3) Fcγ receptors binding, and 4) complement binding.

**Results: **Two vaccine‐induced IgG1 mAbs with the highest ELISA reactivity were selected: RH16 (V3 mAb) and HH1G9 (conformational C2/C5 mAb), for comparison with 2219 (V3 mAb). These three mAbs had no neutralization activity, and all mediated ADCP. Interestingly, they displayed differential complement binding and ADCC. 2219 bound C1q and C3d but had undetectable ADCC, whereas RH16 did not bind complement but displayed ADCC. In contrast, HH1G9 bound complement and showed ADCC.

To examine the Fc functions of 2219 that contribute to protection, Fc mutations that affect complement binding and ADCP were introduced. Passive transfer of these Fc variants to humanized mice challenged rectally with HIV demonstrated that the KA mutation, which abrogated mainly complement binding, reduced protection to the same or greater levels as the LALA mutations, which reduced ADCP and complement binding. Currently, Fc mutations that enhance ADCC are being tested.

**Conclusions: **Antibodies against immunogenic regions of Env have Fc‐mediated activities, especially complement binding, that can contribute to protection against mucosal HIV‐1 infection, even though these antibodies have no neutralization activity.

### EPLBA02 Analysis of the HVTN 702 Phase 2b‐3 HIV‐1 vaccine trial in South Africa assessing RV144 antibody and T cell correlates of HIV‐1 acquisition risk

Z. Moodie ^1^, S. Sawant^2,3^, O. Dintwe^1,4^, D. Grove^1^, Y. Huang^1,5,6^, H. Janes^1,7^, J. Heptinstall^2,3^, F. Laher Omar^4^, K. Cohen^1^, S.C. De Rosa^1,8^, L. Zhang^2,3^, N.L. Yates^2,3^, M. Sarzotti‐Kelsoe^3,9^, K.E. Seaton^2,3^, F. Laher^10^, L.‐G. Bekker^11^, M. Malahleha^12,13^, C. Innes^14^, S. Kassim^11^, N. Naicker^15^, V. Govender^16^, M. Sebe^17^, N. Singh^16^, P. Kotze^18^, E. Lazarus^10^, M. Nchabeleng^19^, A.M. Ward^20,21^, W. Brumskine^22^, T. Dubula^23^, A.K. Randhawa^1^, N. Grunenberg^1^, J. Jin Kee^1^, L.N. Carpp^1^, J. Hural^1^, M. Allen^24^, P. D'Souza^24^, J. Tartaglia^25^, C.A. DiazGranados^25^, M. Koutsoukos^26^, P.B. Gilbert^1,6,7^, J.G. Kublin^1^, L. Corey^1,8^, E. Andersen‐Nissen^1,4^, G.E. Gray^10,16^, G.D. Tomaras^2,3,9,27^, M.J. McElrath^1,28^, HVTN 702 Protocol Team

^1^Fred Hutchinson Cancer Center, Vaccine and Infectious Disease Division, Seattle, United States, ^2^Duke University, Center for Human Systems Immunology, Durham, United States, ^3^Duke University, Department of Surgery, Durham, United States, ^4^Hutchinson Centre Research Institute of South Africa, Cape Town HVTN Immunology Laboratory, Cape Town, South Africa, ^5^University of Washington, Department of Global Health, Seattle, United States, ^6^Fred Hutchinson Cancer Center, Public Health Sciences Division, Seattle, United States, ^7^University of Washington, Department of Biostatistics, Seattle, United States, ^8^University of Washington, Department of Laboratory Medicine and Pathology, Seattle, United States, ^9^Duke University, Department of Immunology, Durham, United States, ^10^University of the Witwatersrand, Perinatal HIV Research Unit, Faculty of Health Sciences, Johannesburg, South Africa, ^11^University of Cape Town, Desmond Tutu HIV Centre, Cape Town, South Africa, ^12^Setshaba Research Centre, Soshanguve, South Africa, ^13^Synergy Biomed Research Institute, East London, South Africa, ^14^The Aurum Institute, Klerksdorp, South Africa, ^15^Centre for the AIDS Programme of Research in South Africa, Durban, South Africa, ^16^South African Medical Research Council, Durban, South Africa, ^17^Aurum Institute, Tembisa, South Africa, ^18^Qhakaza Mbokodo Research Centre, Ladysmith, South Africa, ^19^Sefako Makgatho Health Sciences University, Mecru Clinical Research Unit, Pretoria, South Africa, ^20^University of Cape Town, Department of Medicine, Cape Town, South Africa, ^21^University of Cape Town, Wellcome Centre for Infectious Diseases Research in Africa, Institute of Infectious Disease and Molecular Medicine, Cape Town, South Africa, ^22^Aurum Institute, Johannesburg, South Africa, ^23^Walter Sisulu University, Nelson Mandela Academic Clinical Research Unit and Department of Internal Medicine and Pharmacology, Mthatha, South Africa, ^24^National Institutes of Health, National Institute of Allergy and Infectious Diseases, Bethesda, United States, ^25^Sanofi‐Pasteur, Swiftwater, United States, ^26^GSK, Wavre, Belgium, ^27^Duke University, Department of Molecular Genetics and Microbiology, Durham, United States, ^28^University of Washington, Department of Medicine, Seattle, United States

**Background: **Whether the immune correlates of HIV‐1 acquisition risk identified in the Thai HIV‐1 vaccine efficacy trial of an ALVAC/gp120 pox‐protein vaccine regimen (RV144) generalize to other at‐risk populations is a critical question. Although the clade C‐adapted vaccine regimen was not efficacious in preventing HIV‐1 acquisition in South African participants, HVTN 702 (NCT02968849) provides a unique opportunity to answer this important question and to raise hypotheses regarding the observed lack of efficacy.

**Methods: **Among 3909 female vaccinees, 60 HIV‐1‐seropositive cases and 60 matched seronegative controls were sampled. HIV‐1‐specific CD4+ T‐cell and binding antibody (bAb) responses were measured by intracellular cytokine staining and bAb multiplex assays 2 weeks post‐fourth and fifth immunizations. Three primary vaccine‐matched immunological endpoints that were strong inverse correlates of HIV‐1 risk in RV144 were assessed as predictors of HIV‐1 acquisition among vaccinees using Cox proportional hazards models: Env‐ZM96‐specific CD4+ polyfunctionality score based on six markers and total IgG and IgG3 binding antibody responses to A244 and 1086.C V1V2. Secondary endpoints included polyfunctional CD4+ T‐cell responses to other Env vaccine inserts, IgG bAbs to gp120 and Env consensus antigens, and IgA bAbs. Interactions among pre‐specified primary and secondary endpoints were also assessed using Cox models, with low/medium/high categories defined by tertiles.

**Results: **Although no significant association was observed between any T‐cell or bAb response and HIV‐1 acquisition, significant interactions were seen in pre‐specified analyses (multiplicity‐adjusted p‐values <=0.03). Among those with highest tertile IgG A244 V1V2 bAb responses, vaccine‐matched CD4+ T‐cell endpoints (polyfunctional scores to Env‐ZM96 and 1086, triple‐functional cells expressing IFN‐g, IL2, and CD40L to Env‐ZM96) were associated with decreased HIV‐1 acquisition risk with estimated hazard ratios=0.40‐0.49 per 1‐SD increase in the respective CD4+ T‐cell endpoint.

**Conclusions: **Our study interrogated previously identified correlates of HIV‐1 risk and their interplays for an ALVAC/gp120 vaccine in the South African population. We hypothesize that due to low IgG V1V2 bAb responses in HVTN 702 vs. RV144, Env‐specific CD4+ T‐cell responses were not confirmed as predictors of risk. Higher bAb V1V2 responses in combination with polyfunctional CD4+ T‐cell responses may be necessary to reduce HIV‐1 acquisition.

### EPLBA03 Transcriptome analysis to identify the changes in microRNA expression profile and their targeted pathways in the cervicovaginal mucosa of HIV‐infected women

K. Akolkar ^1^, S. Sonar^1^, A. Rao^1^, M. Mamulwar^1^, H. Bal^2^, R. Bagul^1^, U. Ghule^1^, A. Mane^1^, M. Thakar^1^, V. Saxena^1^

^1^National AIDS Research Institute, Pune, India, ^2^D Y Patil University, Pune, India

**Background: **Cervicovaginal mucosa (CVM) plays a key role in HIV acquisition among females during heterosexual contact, hence understanding of immune mechanisms and their regulatory factors is warranted. MicroRNAs are important regulators of cellular events, however limited is known about their involvement at CVM. We performed a detailed transcriptome analysis to identify dysregulated microRNAs and their associated cell‐signaling pathways at CVM during HIV infection.

**Methods: **Cervical samples collected from 25 HIV‐infected and 33 HIV‐uninfected women were tested for HBV, HCV, TV, BV, NG, CT and syphilis for screening other STIs. Cytobrush samples from STI‐negative women were used for transcriptome analysis. Briefly, cervical cells isolated from cytobrush samples of 13 HIV‐infected and 8 HIV‐uninfected women were used for small‐RNA sequencing. Dysregulated microRNAs were identified using log2fold changes and their targets using miRanda tool. Functional annotations of microRNA target genes were performed using KEGG Ortholog database and Gene Ontology.

**Results: **All the study participants tested negative for other STIs, except BV which was present in 28% HIV‐infected and 10% in HIV‐uninfected women. RNA sequencing yielded 12‐13x10^6^ total and >1‐1.8x10^6^ unique reads. Unique reads were mapped to human genome, annotated and classified into different categories of RNAs. HIV‐uninfected participants had 2% higher proportion of microRNA reads than HIV‐uninfected participants. Total 20410 microRNAs were identified, of which 314 known microRNAs were differentially expressed in HIV infected women. Using Fisher exact test, 19 known microRNAs were found to be significantly dysregulated among HIV‐infected women. Their target genes were identified and functionally categorized for biological process, cellular component and molecular functions. Majority of these were involved in transcriptional regulation, signal transduction, binding of molecules etc. Pathway enrichment data showed their involvement in important immune pathways including TLR signaling, NLR signaling NF‐ĸB signaling, MAPK signaling pathway as well as in HIV‐1 infection.

**Conclusions: **We identified the profile of dysregulated microRNAs, their key targets and associated signaling pathways using *in silico* approaches in the cervical samples of HIV infected women. These are mainly associated with innate immune responses and inflammation, which are critical during HIV infection; hence their targeting may be helpful in devising immunotherapeutic strategies to combat HIV infection/ inflammation at CVM.

### EPLBA04 CD8 depletion and N‐803 plus anti‐SIV Env RhmAbs in ART‐suppressed rhesus macaques

V. Singh ^1^, D. Burgess^1^, A. Dashti^1^, J. McBrien^2^, H. King^3^, R. Mason^3^, J. Safrit^4^, J. Lifson^5^, M. Tuyishime^6^, G. Ferrari^6^, M. Roederer^3^, G. Silvestri^2^, A. Chahroudi^1^

^1^Emory University, Pediatrics/Infectious Disease, Atlanta, United States, ^2^Emory University, Yerkes National Primate Research Center, Atlanta, United States, ^3^NIAID/NIH, Vaccine Research Center, Bethesda, United States, ^4^ImmunityBio, Infectious Diseases, Santa Monica, United States, ^5^Frederick National Laboratory, AIDS and Cancer Virus Program, Frederick, United States, ^6^Duke University, Surgical Sciences, Durham, United States

**Background: **Building upon the robust latency reversal with CD8+ cell depletion and IL‐15 superagonist N‐803 in SIV‐infected, ART‐suppressed rhesus macaques (RMs), we developed a cure strategy with these latency reversal agents (LRAs) plus a cocktail of four anti‐SIV Env‐specific rhesus IgG1 monoclonal antibodies (RhmAbs) targeting V2, CD4 binding site, CD4 binding site proximal, and membrane proximal external region (MPER) with the goal of reducing reservoir cells.

**Methods: **28 adult RMs were infected with SIV_mac239_and began ART at 8 weeks post infection (wpi). Study arms included ART controls (n=7), ART + RhmAbs controls (n=7), and intervention (ART + CD8a depletion + N‐803 + RhmAbs, n=14). After 96 weeks of ART, RhmAbs were administered; three days later, the CD8a depleting MT807R1 was co‐administered with N‐803, followed by 3 additional weekly N‐803 doses. During this period, on‐ART viremia measured by qPCR of SIV*gag* RNA. CD8+ T cell depletion efficacy was assessed by flow cytometry and serum RhmAb concentrations were measured longitudinally by indirect ELISA. RhmAb effector functions were determined using infected cell Ab binding and infected cell elimination assays.

**Results: **We first demonstrated that the combination of four RhmAbs binds to SIV‐infected cells and exerts ADCC *in vitro*.We next found that CD8a depletion was efficient (>99% in blood and ≥95% in lymph nodes)and circulating RhmAbs levels were high *in vivo*.During the first four weeks following MT807R1 + N‐803, on‐ART viremia >60 copies/ml was seen in 28 of 56 viral load measurements (50%) with 1/56 (1.8%) >1,000 copies/ml. In comparison, a historical comparison group of RMs that received MT807R1 + N‐803 without RhmAbs experienced more frequent on‐ART viremia of 73%, with 23% of measurements >1,000 copies/ml.

**Conclusions: **During latency reversal with CD8a depletion + N‐803 in combination with RhmAbs, we demonstrate a similar but diminished magnitude of on‐ART viremia compared to prior work by McBrien et al (*Nature*2020). This effect may result from RhmAb neutralization of virions released from infected cells and, combined with the additional antibody effector functions identified, supports a role for therapeutic RhmAbs as potential clearance agents in cure approaches.

### EPLBA05 Translational potency of the antisense protein ORF in the proviral DNA context

B. Barbeau ^1,2^, Y. Xiao^3^

^1^Université du Québec à Montréal, Montreal, Canada, ^2^Réseau Intersectoriel de recherche en santé de l'Université du Québec, Laval, Canada, ^3^Université du Québec à Montréal, Montréal, Canada

**Background: **Previous *in silico* analyses of HIV‐1 isolates provided strong evidence of the existence of the antisense open reading frame overlapping the HIV‐1 *envgene*, termed Antisense Protein (ASP). The detection of the resulting protein has been a challenge and, although we were successful in studying ASP in cells transfected with expression vectors, its detection and understanding of its regulation in the context of proviral DNA remain difficult. Our objective was thus to test new proviral DNA‐based constructs in which different reporter versions were inserted.

**Methods: **Proviral DNA NL4.3 and NL4.3 BaL were used to insert the Myc tag or the luciferase reporter gene at the amino/COOH end or other positions. The 11 HiBiT peptide of the split Luc‐based Nano‐Glo® HiBiT System was similarly inserted in proviral DNA. The 5’LTR was removed in certain constructs. Proviral DNA were transfected in HEK293T cells alone or with tat/rev expression vectors. Luciferase activity was measured and confocal microscopy analyses were performed.

**Results: **NL4.3‐based proviral DNA in which the luciferase gene was inserted next to the ASP initiation codon (termed NL4.3LucASP) generated a significant signal in transfected cells, but, as expected, was lower than the classical NL4.3Luc+env‐ vector. Interestingly, signals were lost in versions of NL4.3LucASP deleted of its 5’LTR (no sense expression) or of a previously identified polyA signal. Furthermore, Tat could importantly induce luciferase expression in 5’LTR‐deleted proviral DNA. Insertion of the luciferase reporter gene or the short HiBiT tag (complemented with the large Luc subunit by co‐transfection of an expression vector) at different positions in the ASP ORF showed similar luciferase activity, again suggesting that the ASP ORF is translated. Addition of the Myc tag in the ASP ORF also led to the detection of ASP signals in transfected cells by confocal microscopy.

**Conclusions: **These results further add to the growing evidence that the ASP protein exists and has functional relevance. Such proviral DNA constructs should also provide important tools to study transcriptional and post‐transcriptional regulation of ASP expression and lead to novel approaches for ASP detection in infected cells.

### EPLBA06 Deep down in the gut: analyzing the connection between epithelial cells and ART‐induced inflammation

G.G. Gornalusse ^1^, C. Levy^1^, S.M. Hughes^1^, U. Pandey^1^, A. Yi^1^, P.N. Vo^1^, J. Porter^2^, J. Smith^2^, F. Hladik^1^

^1^University of Washington, Obstetrics and Gynecology, Seattle, United States, ^2^University of Washington, Microbiology, Seattle, United States

**Background: ​**Despite virologically suppressive combination antiretroviral therapy (cART), people living with HIV are more likely than HIV‐negative people to experience comorbidities, mainly due to chronic immune activation. Here, we focused on the gastrointestinal (GI) tract because it represents the largest HIV reservoir. We tested the hypothesis that certain nucleoside/nucleotide reverse transcriptase inhibitors (NRTI) drugs, mainstay cART components, induce interferon‐stimulated‐genes (ISGs) in GI cells. Our previous work suggested that chronic exposure to NRTIs induces the proliferation of microfold (“M”) cells, a relatively rare type of enterocyte. Because M‐cells have been shown to be immunologically active, we tested whether supernatants from cultures containing M‐cells induce HIV reactivation in latently infected T cells.

**Methods: **We exposed colorectal and duodenal cell lines to tenofovir (TFV) and quantified by the level of three ISGs: *ISG15*, *IFI6* and *MX1* using digital droplet PCR. We used two models of the small intestinal epithelium: **(i)**a triple‐coculture system containing colon carcinoma Caco‐2, mucus‐producing HT29, and Raji B cells and **(ii)** an *ex vivo* duodenum and ileum organoid model, in which four differentiation factors (noggin, retinoic acid, LTα2β1 and sRANKL) generate functional M‐cells. We exposed HIV latently infected GFP‐expressing J‐Lat 11.1 cells to culture supernatants from both models (separately) and measured HIV reactivation by flow cytometry and RT‐ddPCR.

**Results**:

- After 3 days of treatment, TFV induced *IFI6* mRNA in duodenal carcinoma cells (2.5‐4‐fold increase).
- Culture supernatants from the triple‐coculture model but not from single cultures induced HIV reactivation (5‐fold increase in % GFP^+^ cells, 3‐fold increase HIV‐LTR‐polyA RNA).
- By qPCR, both the duodenum and ileum organoids expressed the M‐cell factors *SpiB*, *CCL20* and *GP2*. When tested separately, LTα2β1 but not noggin, retinoic acid or sRANKL induced HIV reactivation by ∼10‐fold.

**Conclusions: **Soluble factors related to the differentiation and activation of the GI epithelium elicited HIV reactivation. Furthermore, we show that NRTI can trigger the expression of inflammatory ISGs in duodenal cells. Collectively, this suggests that activation in the gut may influence the dynamics of the HIV reservoir.

### EPLBA07 Host genetic variants regulates CCR5 expression on immune cells: a study in people living with HIV and healthy controls

J.C. dos Santos ^1^, Z. Zhang^2^, L.E. van Eekeren^3^, E.T. Fok^4^, N. Vadaq^3^, L. van de Wijer^3^, W.A. van der Heijden^3^, V.A.C.M. Koeken^3^, H.J. Koenen^3^, M. Mhlanga^4^, M.G. Netea^3^, A.J. van der Ven^3^, Y. Li^5^

^1^Radboud University Medical Center, 6525 HP Nijmegen, the Netherlands, Internal Medicine, Nijmegen, Netherlands, the, ^2^University of Groningen, University Medical Center Groningen,, Groningen, Netherlands, the, ^3^Radboud University Medical Center, Nijmegen, Netherlands, the, ^4^Radboud University, Nijmegen, Netherlands, the, ^5^Hannover Medical School and the Helmholtz Centre for Infection Research,, Hannover, Germany

**Background: **CCR5 plays an important role in the acquisition of HIV and it is associated to immune activation in people living with HIV (PLHIV). Understanding the genetic regulation of CCR5 is crucial for HIV susceptibility and pathogenesis.

**Methods: **Quantitative trait loci (QTL) mapping analysis was performed to assess genetic variants associated with CCR5 expression on circulating immune cells in 209PLHIV using ART and 304 healthy controls, all Western European ancestry. The proportions of CCR5 positive cells and CCR5 mean fluorescence intensity (MFI) were assessed by flow cytometry in monocytes and CD4^+^and CD8^+^T cell subsets using flow cytometry.

**Results: **We identified distinct genetic variants that are associated with CCR5 cell proportions or mean fluorescence intensity in subpopulations of T cells with memory functions in both healthy and PLHIV (Figure 1). We identified the rs60939770, which is an intergenic variant in*cis*‐regionto*CCR5*gene notin linkage disequilibrium with*CCR5d32*,related to the proportion of CCR5^+^memory T regulatory cells, both in PLHIV and healthy controls.Twogenome‐wide significant loci, in linkage equilibrium with*CCR5d32*, were found to be associated with CCR5MFI ofmultiple subsets of mostly differentiated memory T cellsin both groups. The expression of nearby chemokines receptors (*CCR1*,*CCR2*,*CCR3, CCRL2*),existing in the same the same topologically associating domain, were also influenced by these genetic variants. Furthermore, we show the genetic variants which modulate CCR5 surface expression affect the production of other inflammatory mediators, including monocyte‐ and lymphocyte‐derived cytokines as well as CCL4 and IL‐8.

**Figure:**
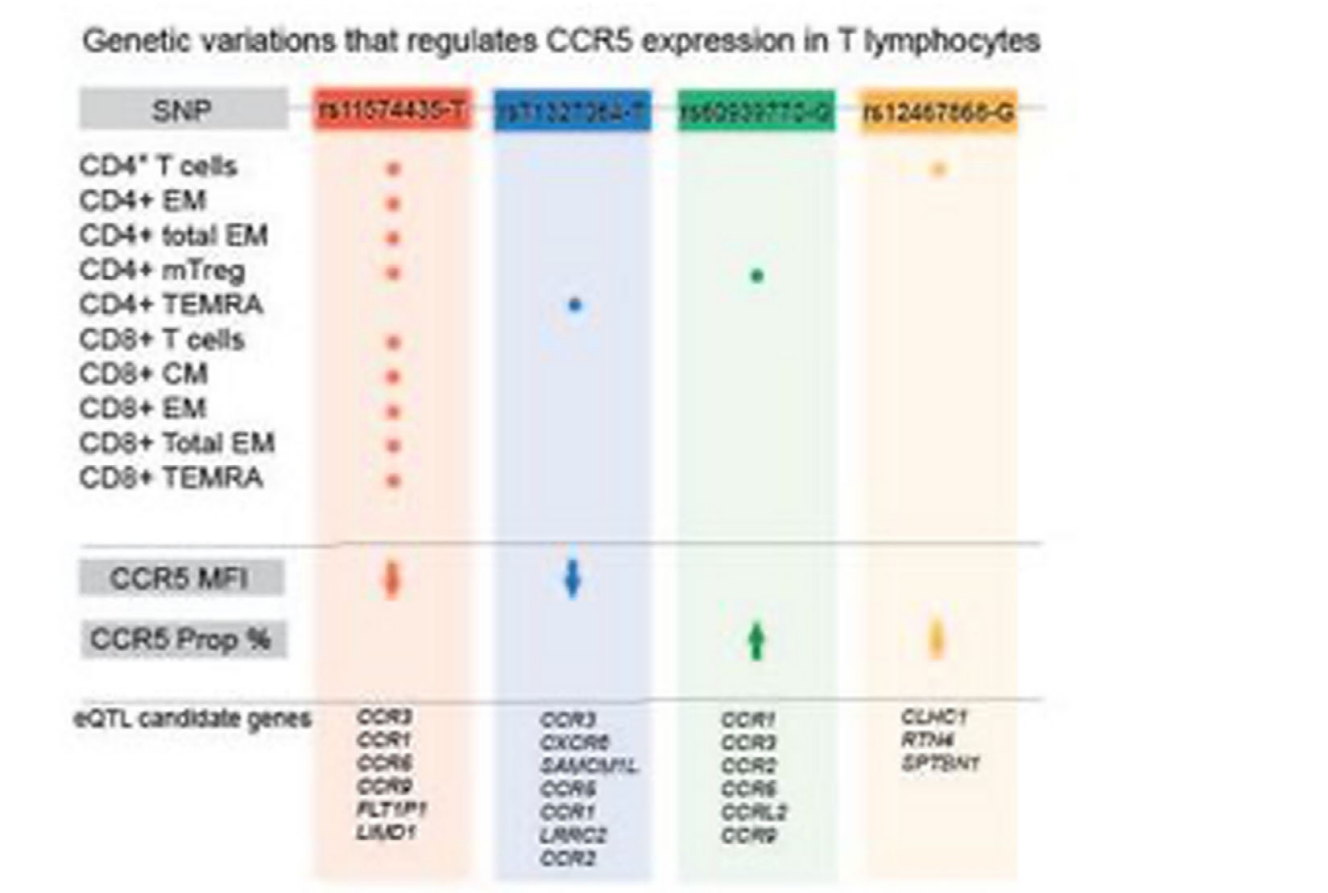
**Abstract EPLBA07‐Figure 1**.

**Conclusions: **We demonstrated that the genetic regulation of CCR5 expression is cell‐type specific and may impact HIV susceptibility and disease progression.

### EPLBA08 ILC3s in HIV‐infected lymph nodes up‐regulate inflammatory pathways linked to tissue fibrosis

N. Herbert ^1^, O.E. Asowata^1^, Y. Zungu^1^, C. Kummerlowe^2,3^, A. Singh^1^, N. Mthabela^1^, D. Ramjit^1^, F. Karim^1^, F.G. Madela^1^, V.T. Manzini^1^, F. Anderson^1^, A.K. Shalek^2,3^, A. Leslie^1^, H. Kloverpris^1^

^1^Africa Health Research Institute, Durban, South Africa, ^2^Massachusetts Institute of Technology, Program in Computational and Systems Biology, Boston, United States, ^3^Massachusetts Institute of Technology, Department of Chemistry, Boston, United States

**Background: **People living with HIV (PLWH) develop extensive fibrosis and collagen deposition throughout their lymphoid tissues not reversed by antiretroviral therapy (ART). Innate lymphoid cells (ILCs) play essential roles in tissue homeostasis and repair. However, no studies exist on ILCs in lymph nodes (LNs) during HIV infection. We hypothesized that ILCs are modulated by HIV infection and participate in the subsequent immune response.

**Methods: **We obtained fresh mesenteric, celiac and hepatic LNs immediately after gastrointestinal surgery from patients recruited from areas in South Africa which were subject to flow cytometry (n=64), F‐IHC (n=8) and scRNA‐seq (n=7).

**Results: **LNs from PLWH receiving ART exhibited extensive collagen deposition and CD4 T‐cell depletion compared to uninfected controls characteristic of HIV LN pathology. We found no correlation between CD4 T‐cell levels and ILC subsets in LNs, but reduced CD4 levels in both blood and LNs of HIV suppressed PLWH. Strikingly, we found no depletion of any of the ILC subsets in LNs, except a slight reduction of the dominant ILC3s in LNs located outside the germinal centers and close to HIV‐infected cells. In contrast, circulating ILC3s were severely depleted in PLWH and consistent with our previous work. Single‐cell transcriptional profiling revealed activation of the dominant ILC3 subset during HIV infection, suggesting ILC3s are directly involved in the HIV response. HIV‐infected LNs expressed more heterogenous ILC3 subsets, including NK‐like or ‘ex‐ILC3s’ with cytotoxic potential, suggesting that HIV infection induces trans‐differentiation away from conventional ILC3 subsets towards cytotoxic type I responses. Moreover, we consistently found elevated levels of TGF‐beta producing ILC3s. This subset was enriched in inflammatory pathways in PLWH, suggesting that these cells may play a central role in fibrosis formation through fibroblast‐induced collagen deposition. Transcriptional profiling of the myeloid populations from matched LNs identified macrophages as the dominant source of IL1‐B production and, therefore, may serve as innate sensors and drivers of ILC3 activation and differentiation in HIV‐infected LNs.

**Conclusions: **Here, we performed the first single‐cell analysis of ILCs in HIV‐infected LNs and identified ILC3s as potential contributors to lymph node fibrosis, a major pathological consequence of HIV infection that warrants further investigation.

### EPLBB01 Standard versus double dose dolutegravir in patients with HIV‐associated tuberculosis: a phase 2 non‐comparative randomized controlled trial

R. Griesel ^1,2^, Y. Zhao^3,2^, B. Simmons^4^, Z. Omar^2^, A. Hill^4^, G. Meintjes^3,2^, G. Maartens^1,2^

^1^University of Cape Town, Division of Clinical Pharmacology, Department of Medicine, Cape Town, South Africa, ^2^University of Cape Town, Wellcome Centre for Infectious Diseases Research in Africa, Institute of Infectious Diseases and Molecular Medicine, Department of Medicine, Cape Town, South Africa, ^3^University of Cape Town, Department of Medicine, Cape Town, South Africa, ^4^University of Liverpool, Department of Pharmacology and Therapeutics, Liverpool, United Kingdom

**Background: **Rifampicin reduces the exposure of co‐administered dolutegravir. This can be overcome with a supplemental dose of dolutegravir, which is difficult to implement in high burden settings. Dolutegravir trough concentrations of standard dosing with rifampicin were above the protein‐adjusted IC_90_in a healthy‐volunteer study – this finding, coupled with the long dissociative half‐life of dolutegravir from integrase, suggests supplemental dosing of dolutegravir with rifampicin may be unnecessary. We hypothesize that virologic suppression with standard dose dolutegravir‐based antiretroviral therapy (ART) will be acceptable in patients on rifampicin‐based antituberculosis therapy (ATT).

**Methods: **We conducted a phase 2, non‐comparative, randomised, double‐blind, placebo‐controlled trial of standard versus double dose dolutegravir among adults living with HIV on rifampicin‐based ATT who were ART‐naïve or had interrupted ART in Khayelitsha, Cape Town, South Africa. Participants were commenced on tenofovir/lamivudine/dolutegravir (TLD) and randomised to supplemental dolutegravir or placebo. The primary endpoint was the proportion virologically suppressed (HIV‐RNA viral load <50 copies/mL) at 24 weeks analyzed according to modified intention‐to‐treat using the FDA snapshot algorithm.

**Results: **We enrolled 108 participants: median age 35 years (IQR 32 to 41), 38% female, median baseline CD4 cell count 184 cells/mm^3^ (IQR 145 to 316) and HIV‐RNA viral load 5.2 log_10_copies/mL (IQR 4.6 to 5.7). Baseline characteristics were similar between arms. Proportions with virologic suppression at weeks 12 and 24 were similar between arms (**Table** 1). No participants developed dolutegravir resistance. Grade 3 and 4 adverse events occurred at similar rates in both arms. Insomnia developed in more participants in the double dose arm (n=12) than in the single dose arm (n=4).

**Abstract EPLBB01 1:** Proportions with viral load <50 copies/mL

	TLD + Dolutegravir arm(n=53)	TLD + Placebo arm(n=55)
**Week 12**		
mITT, n (% [95%CI])	42/53 (79% [66‐89%])	46/55 (84% [71‐92%])
PP, n (% [95%CI])	42/53 (79% [66‐89%])	46/54 (85% [73‐93%])
**Week 24**		
mITT, n (% [95%CI])	43/52 (83% [70‐92%])	44/53 (83% [70‐92%])
PP, n (% [95%CI])	43/51 (84% [71‐93%])	44/52 (85% [72‐93%])

TLD = tenofovir/lamivudine/dolutegravir, mITT = modified intention‐to‐treat, PP = per protocol

**Conclusions: **Virologic outcomes in both arms were acceptable. Our findings should be confirmed by adequately powered randomised controlled trials before implementation.

### EPLBB02 Chronic/latent viral infection prevalence and estimated all‐cause mortality risk among women living with HIV and HIV‐negative women participating in the British Columbia CARMA‐CHIWOS Collaboration (BCC3) study: preliminary findings

T. Povshedna ^1,2^, S.L. Levy^1,2^, A.R. Campbell^3,4^, S.A. Swann^3,5^, D. Pang^6^, E.M. King^3,7^, V. Nicholson^6,8^, A. Kaida^3,6^, M.C. Murray^3,4,9^, H.C. Cote^1,2,3^, the BCC3 (CIHR, CTN 335) study team

^1^The University of British Columbia, Department of Pathology and Laboratory Medicine, Vancouver, Canada, ^2^The University of British Columbia, Centre for Blood Research, Vancouver, Canada, ^3^Women's Health Research Institute, British Columbia Women's Hospital and Health Centre, Vancouver, Canada, ^4^British Columbia Women's Hospital and Health Centre, Oak Tree Clinic, Vancouver, Canada, ^5^The University of British Columbia Faculty of Medicine, Experimental Medicine, Vancouver, Canada, ^6^Simon Fraser University, Faculty of Health Sciences, Vancouver, Canada, ^7^The University of British Columbia, Department of Medicine, Faculty of Medicine, Vancouver, Canada, ^8^BC Centre for Excellence in HIV/AIDS, Epidemiology and Population Health, Vancouver, Canada, ^9^The University of British Columbia, Division of Infectious Diseases, Faculty of Medicine, Vancouver, Canada

**Background: **Women living with HIV (WLWH) have shorter life expectancy compared to HIV‐negative women, which suggests accelerated/accentuated aging. Healthy aging is affected by chronic inflammation caused by HIV and other persistent viral infections, as well as socio‐structural stressors that disproportionately affect WLWH.

**Methods: **The BCC3 cohort takes a holistic approach to examine healthy aging and enrolls WLWH and HIV‐negative women living in British Columbia, Canada. Prevalence of 8 chronic viral infections was assessed by serology (hepatitis B and C viruses (HBV, HCV), herpes simplex viruses (HSV‐1, HSV‐2), Epstein‐Barr virus (EBV), human herpesvirus‐8 (HHV‐8), and cytomegalovirus (CMV)), or self‐report (varicella‐zoster virus (VZV)). The Veterans Aging Cohort Study (VACS) index, which estimates 5‐year all‐cause mortality risk based on clinical and demographic parameters, was calculated based on data from the BCC3 survey. The groups were compared by Fisher's, Chi‐Squared, and Mann‐Whitney tests, as appropriate.

**Results: **Table 1 describes demographic characteristics of the study participants. WLWH were more likely to harbor CMV (77% vs 53%, p<0.001), HSV‐2 (73% vs 27%, p<0.0001), HCV (37% vs 7%, p<0.0001), and HBV (23% vs 4%, p<0.001), but not EBV (98% vs 91%, p=0.06), HSV‐1 (72% vs 63%, p=0.17), VZV (74% vs 80%, p=0.3), and HHV‐8 (8% vs 18%, p=0.04). After excluding participants with missing data, WLWH (n=90) had significantly higher VACS scores compared to controls (n=96) 8.2 [3.7‐23.1]% vs 4.2 [3.7‐10.7]%, p<0.001.

**Table:** 

**Abstract EPLBB02‐Table 1**.			
	WLWH (n=100)	Controls (n=100)	P‐value
Age (years), median [IQR] (range)	51 [42‐58] (20‐73)	47 [27‐56] (17‐80)	**0.01**
African/Caribbean/Black / White / Indigenous / Asian / other, %	13 / 38 / 29 / 9 / 11	3 / 53 / 3 / 24 / 7	**<0.001**
Graduated high school, %	69	96	**<0.0001**
Currently employed, %	36	57	**0.003**
Individual income <$20.000, %	64	33	**<0.0001**
Have experienced homelessness, %	51	21	**<0.001**
Current smoking, %	45	21	**<0.001**
Current substance use, %	48	38	0.15
Current opioid use, %	27	11	**0.004**

**Conclusions: **In this interim analysis, WLWH were more likely to have 4/8 chronic/latent viruses and almost twice the estimated risk of mortality within 5 years compared to controls. These observed differences may be partially mediated through biological variables, age, and/or socio‐structural factors. This type of analysis can shed light on the factors that affect aging in WLWH, to ultimately inform action(s) to improve quality of life and close the health gap between WLWH and HIV‐negative women.

### EPLBB03 Cervical cancer screening outcomes among women living with HIV In Malawi

C. Trapence^1^, O. Kumwenda ^1^, A. Abubakar^1^, C. Mhango^1^, A. Mtimuni^1^, A. Worku^1^, C. Lau^1^, J. Gama^1^, T. Van Boven^1^, V. Kanje^1^

^1^USAID, Lilongwe, Malawi

**Background**: In comparison to HIV‐negative women, women living with HIV (WLHIV) are six times more likely to develop persistent precancerous lesions that advance to cervical cancer, with more aggressive forms and higher mortality. Since 2018, PEPFAR Malawi has helped the Ministry of Health integrate cervical cancer screening and treatment into high‐volume ART clinics, increasing the number of facilities from 39 to 129. PEPFAR Malawi used a "screen and treat" strategy that included visual examination with acetic acid (VIA) and treatment of precancerous lesions with thermocoagulation, cryotherapy, and LEEP (for lesions covering more than 75% of the cervix). All PEPFAR‐supported sites have skilled providers, a continuous supply of critical goods, and at least one thermal coagulator. Our objectives is to describe patterns of cervical cancer screening using VIA testing among WLHIV in Malawi from 2020 to 2021.

**Description: **We retrospectively analyzed 2020‐2021cervical cancer program data. Our analysis focused on 116,023 women (20–49 years). Chi‐square test for bivariate analysis, and proportional tests were used to analyze independent association between individual‐level factors and women who have had VIA. Statistical significance was set at p < 0.05.

**Lessons learned: **Using program data, we calculated descriptive statistics and proportion tests to test statistical significance differences in VIA‐positivity by age and screening type. Between October 2020 and September 2021, the program conducted 116,023 cervical cancer screenings. Proportion of WLHIV who tested VIA‐positive was 3% (3,621/116,023). Among 3621 women who screened VIA‐positive, positivity was 4% (2,382/67744) from first time screening, 2% (1170/47283) from rescreening and 7% (69/996) from follow up screening. By age, positivity was 4% for women aged 20‐34 years, 3% for ages 35‐44 years and 1% for ages above 39 years. Although the age range 20‐24 years is outside the PEPFAR targeted population, we noticed a positivity of 4%.

**Conclusions/Next steps: **There is a need to discuss the possibility of including ages 20‐24 years in the PEPFAR targeted population for cervical cancer screening. This group had a positivity of 4% which is higher than positivity reported for other age categories. In addition, the positivity for follow up screens was 7% which implies a need to evaluate the effectiveness of treatment.

### EPLBB04 Randomised study of switch toDTG/RPV in subjects with HIV RNA <50c/ml and archived K103N over 48 weeks

G. Moyle ^1^, L. Assoumou^2^, J.‐M. Molina^3^, F. Post^4^, A. Curran^5^, S. Rusconi^6^, S. De Wit^7^, C. Stephan^8^, F. Raffi^9^, M. Johnson^10^, M. Del Mar Masia^11^, J. Vera^12^, K. Morris^13^, A. Duffy^13^, C. Fletcher^13^, A. Pozniak^14^

^1^Chelsea and Westminster Hosptal, HIV, London, United Kingdom, ^2^ANRS, Pierre Louis Institute of Epidemiology and Public Health, UMR‐S 1136, INSERM, Sorbonne Universités UPMC Paris Univ 06, Paris, France, ^3^Hospital Saint Louis, Paris, France, ^4^Kings College NHS Foundation Trust, London, United Kingdom, ^5^Hospital Universitario Vall d'Hebron, Barcelona, Spain, ^6^University of Milan, Department of Biomedical and Clinical Sciences "Luigi Sacco" ASST, Milano, Italy, ^7^University Hospital of Saint‐Pierre, Brussels, Belgium, ^8^Goethe‐Universitat Frankfurt, Frankfurt, Germany, ^9^University Hospital of Nantes, Hôpital Hôtel‐Dieu, Service d'Infectiologie, Nantes, France, ^10^Royal Free London NHS Foundation Trust, London, United Kingdom, ^11^General University Hospital of Elche, Infectious Diseases Unit, Elche, Spain, ^12^Elton John Centre (…)Brighton and Sussex University Hospitals, Brighton, United Kingdom, ^13^Research Organisation (Kings Cross), London, United Kingdom, ^14^Chelsea and Westminster NHS Foundation Trust, London, United Kingdom

**Background: **DTG/RPV 2‐drug regimen was studied in virologically suppressed switched subjects with no prior treatment failure history or resistance. Viruses with NNRTI resistance mutation K103N retain in‐vitro susceptibility to RPV. Potential to maintain viral suppression with DTG/RPV in subjects with documented K103N currently suppressed on other regimens was investigated.

**Methods: **This is a European, open‐label, multi‐centre, exploratory study randomised 2:1 over 48 weeks of switch to DTG/RPV vs continuing current suppressive regimen (CSR) in treatment experienced, HIV‐1 subjects with documented, prior K103N mutation. Prior PI and NRTI mutations were permitted. Mutations known to reduce susceptibility to RPV or DTG, INSTI failure history, or contraindications to DTG or RPV were exclusions.

**Results: **Results were available for all 140 randomised subjects (DTG/RPV: 95, CSR: 45), well matched for baseline characteristics, median age was 52yr, 82% male, 73% white and median CD4 570 c/uL. Baseline regimens included NRTIs 77%, PI/b 63%, INSTI 48%.

Proportion of subject with treatment success (HIV‐RNA<50 copies/mL) by ITT FDA Snapshot at week 48 was DTG/RPV 90.5% vs CSR 88.9% (‐1.5%, 95% CI ‐12.3 to +9.8). One CSR (2.2%) and three DTG/RPV (3.2%) subjects were confirmed virological failures (2x ≥50copies/ml >2weeks apart). All virological load failures were <200 copies/ml.

Adverse event occurred with DTG/RPV 80.0% vs CSR 73.3%. Drug‐related AE rate was 23.2% for DTG/RPV (mostly grade 1, and one grade 3) with none in the CSR group. Three DTG/RPV subjects out of 13 discontinuations (8 DTG/RPV; 5 CSR) were withdrawn for AEs (Aggressive behaviour; fatty faeces and flatulence; sleep disorder). There were no significant differences in changes to weight, lipids, or renal parameters.

**Conclusions: **In subjects with archived K103N currently suppressed on standard regimens, switch to DTG/RPV maintains virological suppression in the majority of subjects through week 48. Higher rates of mainly grade 1 AEs were observed, consistent with other switch studies.

### EPLBB05 Safety and effectiveness outcomes from the Carisel study: phase 3b hybrid‐3 implementation study integrating cabotegravir + rilpivirine long‐acting into European clinical settings

C. Jonsson‐Oldenbüttel ^1^, J. Ghosn^2,3^, M. van der Valk^4^, E. Florence^5^, F. Vera^6^, M. Ait‐Khaled^7^, G. Bontempo^8^, C. Latham^8^, C. A. Gutner^8^, S. Iyer^9^, R. DeMoor^10^, M. Gill^7^, M. Czarnogorski^8^, J. van Wyk^7^

^1^MUC Research GmbH, München, Germany, ^2^Université de Paris, INSERM UMR 1137 IAME, Paris, France, ^3^Hôpital Bichat–Claude Bernard, Service de Maladies Infectieuses et Tropicales, AP‐HP, Paris, France, ^4^Amsterdam UMC, Amsterdam, Netherlands, the, ^5^Instituut voor Tropische Geneeskunde, Antwerp, Belgium, ^6^Hospital General Universitario de Santa Lucía, Murcia, Spain, ^7^ViiV Healthcare, Brentford, United Kingdom, ^8^ViiV Healthcare Research Triangle Park, Durham, United States, ^9^GlaxoSmithKline, Bangalore, India, ^10^GlaxoSmithKline, Collegeville, United States

**Background: **Cabotegravir + rilpivirine long‐acting (CAB+RPV LA) dosed every 2 months (Q2M) is a recommended regimen in European and US treatment guidelines for virologically suppressed people living with HIV‐1 (PLWH) with no known CAB/RPV resistance. CARISEL is the first study in which all participants switched from daily oral therapy to CAB+RPV LA Q2M. Key safety and effectiveness Month 12 endpoints are reported.

**Methods: **This single‐arm study enrolled virologically suppressed PLWH to receive CAB+RPV LA Q2M. Clinics with no prior experience with CAB+RPV LA were preferentially selected. Effectiveness endpoints were the proportion of participants with plasma HIV‐1 RNA ≥50 copies/mL and <50 copies/mL at Month 12 (FDA Snapshot algorithm, intention‐to‐treat exposed population). Safety outcomes were also reported.

**Table:** 

**Abstract EPLBB05‐Table 1. Adverse Events Outcomes (Including ISRs)**	
	CAB+RPV LA (n=430) n (%)
Any AEs*	420 (98)
Drug‐related AEs	389 (90)
Grade 3–5 drug‐related AEs	25 (6)
AEs leading to treatment withdrawal	42 (10)
Recorded ISRs leading to treatment withdrawal	24 (6)
SAEs^†^	15 (3)

^*^No Grade 5 AEs or deaths were reported; most common AEs were injection site pain (80%); COVID‐19 infection (16%); injection site induration (10%); injection site discomfort (9%); pyrexia (9%).

One SAE was reported as drug related and led to study treatment discontinuation.

AE, adverse event; CAB, cabotegravir; ISR, injection site reaction; LA, long‐acting; RPV, rilpivirine; SAE, serious adverse event.

**Results: **Thirteen of 18 clinics (72%) had no experience with administering CAB+RPV LA at study start. 430 enrolled and treated participants were included; 25% were female (sex at birth), 18% were Black, with a mean baseline age of 44 years (30% >50 years). At Month 12, 87% (n=373/430) of participants maintained HIV‐1 RNA <50 copies/mL, and the proportion with HIV‐1 RNA ≥50 copies/mL was 0.7% (n=3/430). One participant had confirmed virologic failure (n=1/430; 0.23%) with E138A+M230L RPV resistance‐associated mutations (RAMs) and no INSTI RAMs detected in the suspected virologic failure sample at Month 10; the E138A RPV RAM was present at baseline (retrospective testing of Day 1 peripheral blood mononuclear cell pro‐viral DNA). Most AEs and drug‐related AEs were Grade 1 or 2 (86% and 94%, respectively). Injection site reactions (ISRs) were reported in 86% of participants; 98% were mild or moderate in severity. The median ISR duration was 3 days, with >80% resolving within 7 days. Few participants (6%) discontinued due to ISRs (Table).

**Conclusions**: Across diverse European clinical settings and participants, CAB+RPV LA Q2M was well tolerated and highly effective in maintaining virologic suppression with a low rate of virologic failure.

### EPLBB06 Suboptimal lopinavir exposure in infants 1‐12 months on rifampicin treatment receiving double‐dosed or semi‐superboosted lopinavir/ritonavir; results from the EMPIRICAL trial

T.G. Jacobs ^1^, V. Mumbiro^2^, C. Moraleda^3^, A. Colbers^1^, A. Passanduca^4^, S. Domínguez‐Rodríguez^3^, M. Chitsamatanga^2^, A. Tagarro^3^, A. Ballesteros^3^, K.J. Nathoo^2^, L. Madrid^3^, N. Namuziya^5^, B. Nduna^6^, W.C. Buck^4,7^, C. Chabala^5,8^, H.A. Mujuru^2^, D.M. Burger^1^, P. Rojo^3^, EMPIRICAL study team

^1^Radboud university medical center, Clinical Pharmacy, Nijmegen, Netherlands, the, ^2^University of Zimbabwe Clinical Research Centre, Harare, Zimbabwe, ^3^Hospital 12 de Octubre, Madrid, Spain, ^4^Universidade Eduardo Mondlane Faculdade de Medicina, Maputo, Mozambique, ^5^University Teaching Hospitals‐Children's Hospital, Lusaka, Zambia, ^6^Arthur Davidson Children's Hospital, Ndola, Zambia, ^7^University of California Los Angeles, David Geffen School of Medicine, Pediatrics, Los Angeles, United States, ^8^University of Zambia, School of Medicine, Lusaka, Zambia

**Background: **Double‐dosing of lopinavir/ritonavir (LPV/r) in infants and young children receiving rifampicin resulted in subtherapeutic LPV trough concentrations (<1.0mg/L) in 60% of children. However, only four infants <12 months old participated in that study while activity of LPV metabolism through CYP3A4 changes greatly during the first year of life. Super‐boosted LPV/r to a 1:1 ratio is recommended for infants being co‐treated with rifampicin. In clinical practice, however, double‐dosed LPV/r is frequently given to infants receiving rifampicin due to limited availability of single formulation ritonavir syrup. We evaluated plasma LPV concentrations in infants with HIV receiving LPV/r according to local dosing guidelines with or without rifampicin‐based TB‐treatment.

**Methods: **This is a 2‐arm pharmacokinetic sub‐study of the EMPIRICAL randomized controlled trial (#NCT03915366) for severe pneumonia in infants with HIV. Eligible infants aged 1‐12 months, weighing ≥ 4kg, receiving LPV/r with or without (control) rifampicin‐based TB‐treatment, were recruited from hospitals in Mozambique, Zambia, and Zimbabwe. Infants received double‐dosed or semi‐superboosted LPV/r (adding a ritonavir 100mg crushed tablet to the evening LPV/r dose) during rifampicin co‐treatment. Six blood samples were taken over 12 hours. This project is part of the EDCTP2 programme supported by the European Union RIA2017MC‐2013.

**Results: **In total, 13/15 included infants had evaluable pharmacokinetic curves; 8/13 had rifampicin co‐treatment (5 received double‐dosed and 3 semi‐superboosted LPV/r). The median (IQR) weight was 5.7kg (5.3‐6.7) and age 6.6 months (5.5‐9.7), 9/13 were male. 5/8 infants in rifampicin arm had LPV C_trough_ <1.0mg/L (equally divided over those receiving double‐dosed and semi‐superboosted LPV/r); median (IQR) AUC_0‐12h_ and C_trough_ were 47.6 (7.7‐96.1) h*mg/L and 0.25 (0.06‐2.8) mg/L, respectively. In the control arm, 1/5 infants had C_trough_ <1.0mg/L; AUC_0‐12h_ and C_trough_ were 64.2 (61.8‐237.2) h*mg/L and 3.4 (1.6‐15.8) mg/L, respectively. LPV apparent oral clearance was 4‐fold higher for infants receiving rifampicin.

**Conclusions: **Double‐dosed or semi‐superboosted LPV/r for infants 1‐12 months old receiving rifampicin resulted in substantial proportions of subtherapeutic LPV levels. There is an urgent need for data on alternative ARVs in infants with HIV/TB co‐infection, such as twice‐daily dolutegravir which is being evaluated in an ongoing EMPIRICAL pharmacokinetic substudy.

### EPLBB07 A point‐of‐care triage test for HIV virological failure: filling the gaps in viral load coverage

A. Saura‐Lázaro ^1^, P. Bock^2^, E. van den Bogaart^3^, J. van Vliet^3^, L. Granés^4^, K. Nel^2^, V. Naidoo^2^, Y. Saunders^2^, M. Scheepers^2^, R. Paulussen^3^, D. Naniche^1^, E. López‐Varela^1,2^

^1^Barcelona Centre for International Health Research (CRESIB), Hospital Clinic‐Universidad de Barcelona, Barcelona, Spain, ^2^Desmond Tutu TB Centre, Department of Paediatrics and Child Health, Faculty of Medicine and Health Sciences, Stellenbosch University, Cape Town, South Africa, ^3^Mondial Diagnostics, Amsterdam, Netherlands, the, ^4^Hospital Clínic de Barcelona, Barcelona, Spain

**Background: **Viral load (VL) monitoring in antiretroviral treatment (ART) patients is challenging, especially in high‐burden settings. Access to an accurate, affordable point‐of‐care test (POCT) could greatly enhance ART outcomes.IFN‐ɣ‐induced protein 10 (IP‐10) is a chemokine strongly correlated with human immunodeficiency virus (HIV) VL that could serve to predict virological failure (VF) and to triage patients requiring VL testing. This study aimed to evaluate the field performance of a semi‐quantitative prototype lateral flow IP‐10 POCT as a screening test for VF in South Africa.

**Methods: **Finger prick capillary blood was collected from patients attending a primary health clinic in the Western Cape for direct application by trained nurses onto the IP‐10 POCT (index test) and compared with a plasma VL result taken ≤1 month prior (reference test) amongst adult patients on ART for ≥ 1 year. Logistic regression with penalized likelihood was used to build an IP‐10 POCT reading values‐based model able to identify individuals with VF (VL > 1,000 copies/mL). The area under the receiver operating characteristic curves (AUC) was calculated to evaluate model prediction. Testing cost saving was estimated assuming a unit cost of 2 USD for IP‐10 POCT, 22 USD for VL test plus 60% of test‐associated costs.

**Results: **Among the 209 participants (median age 38 years and 88% female), 18% had VF. Median IP‐10 POCT reading values were higher among individuals with VF compared to those without (24.0 vs. 14.6; p < 0.001). The IP‐10 POCT predicted VF with an AUC = 0.76 (95% confidence interval (CI), 0.67–0.85). The model identified VF with 91.9% sensitivity (95% CI, 78.1%–98.3%) and 35.1% specificity (28.0%–42.7%). Projecting a VF prevalence of 18% in a simulated cohort of 1,000 ART patients, an IP‐10 screening POCT would avert > 30% of the routine VL monitoring tests and associated costs.

**Conclusions: **The IP‐10 POCT is an effective triage test for routine VL monitoring. Combining a highly sensitive, low‐cost IP‐10 POCT‐based screening with VL testing in a two‐step decision algorithm could provide a greatly needed monitoring tool in settings with low VL coverage, and result in significant savings for health systems.

### EPLBB08 Analytical treatment interruption (ATI) among African women with early ART initiation with or without VRC01 circulating at HIV acquisition: study design and early observations of viral rebound and control

S. Karuna ^1^, K. Bar^2^, A. DeCamp^3^, E. Rudnicki^3^, P.‐C. Yu^3^, P. Andrew^4^, C. Orrell^5^, A. Takalani^6^, S. Takuva^6^, L. Gama^7^, T.‐W. Chun^8^, N. Mgodi^9^, S. Dadabhai^10^, C.‐A. Mathew^11^, J. Makhema^12^, P. Hunidzarira^9^, F. Laher^13^, M. Hosseinipour^14^, R. Tressler^15^, L. Soto‐Torres^15^, M. Cohen^16^, J. Currier^17^, J. Eron^18^, L. Corey^1^, for the HVTN 805/HPTN 093/A5390 Study Team

^1^Fred Hutch Cancer Center, Vaccine & Infectious Disease Division, Seattle, United States, ^2^University of Pennsylvania, Department of Medicine, Philadelphia, United States, ^3^Fred Hutch Cancer Center, Statistical Center for HIV/AIDS Research and Prevention, Seattle, United States, ^4^FHI360, Durham, United States, ^5^Desmond Tutu Health Foundation, Cape Town, South Africa, ^6^Hutch Centre for Research in South Africa, Johannesburg, South Africa, ^7^Vaccine Research Center, NIAID, Bethesda, United States, ^8^National Institute of Allergy and Infectious Diseases, Laboratory of Immunoregulation, Bethesda, United States, ^9^University of Zimbabwe, Clinical Trials Research Centre, Harare, Zimbabwe, ^10^Johns Hopkins Research Project, Blantyre, Malawi, ^11^Wits Reproductive Health Institute, Johannesburg, South Africa, ^12^Botswana Harvard AIDS Institute Partnership, Gaborone, Botswana, ^13^Perinatal HIV Research Unit Vaccine Research Center, Johannesburg, South Africa, ^14^University of North Carolina Project‐Malawi, Lilongwe, Malawi, ^15^National Institute of Allergy and Infectious Diseases, Division of AIDS, Bethesda, United States, ^16^University of North Carolina, Institute for Global Health and Infectious Diseases, Chapel Hill, United States, ^17^University of California, Division of Infectious Diseases, Los Angeles, United States, ^18^University of North Carolina, Division of Infectious Diseases, Chapel Hill, United States

**Background: **Viremia rebounds rapidly in most people living with HIV upon ART cessation. Early ART initiation is associated with ART‐free virologic control, and broadly neutralizing anti‐HIV‐1 antibodies (bnAbs) may modulate immune responses to HIV. Durable ART‐free virologic control has been observed in 20‐25% of African women in some cohorts, significantly higher than in other populations. The HVTN 703/HPTN 081 AMP trial evaluated VRC01 bnAb‐mediated HIV‐1 prevention among African women; those who acquired HIV were linked to early ART. With African community, investigator, ethics and regulatory collaborators, an AMP ATI (HVTN 805/HPTN 093/A5390) was designed to evaluate whether early ART +/‐ VRC01 circulating at HIV acquisition is associated with virologic control post‐ATI and to assess underlying immunologic and virologic dynamics.

**Methods: **AMP ATI eligibility includes African women with an estimated HIV acquisition date within 8 weeks of receiving VRC01 or placebo in AMP, early ART initiation and ≥1 year of viral suppression. Participants complete an NNRTI switch, as needed, then stop ART and receive frequent viral load (VL) and CD4+ T‐cell count monitoring. ART re‐initiation criteria include CD4<250, VL>1,000 for 4 weeks without 0.5log decline, or participant/clinician request to restart ART.

**Results: **Nine participants from South Africa, Malawi, Botswana and Zimbabwe have enrolled, thus far; 7/9 met ART re‐initiation criteria (n=5 for VL; n=2 for participant/clinician request). One participant requesting ART re‐initiation had tenofovir levels consistent with ART use during ATI. Median time to confirmed VL>200 was 7.3 weeks (range 2.7‐20.9+). Median time to meet virologic ART re‐initiation criteria was 17.1 weeks (11‐21.3). ART was reinitiated a median of 7 days later; all re‐suppressed. No SAEs or Grade ≥2 related AEs were reported. See Figure 1.

**Figure:**
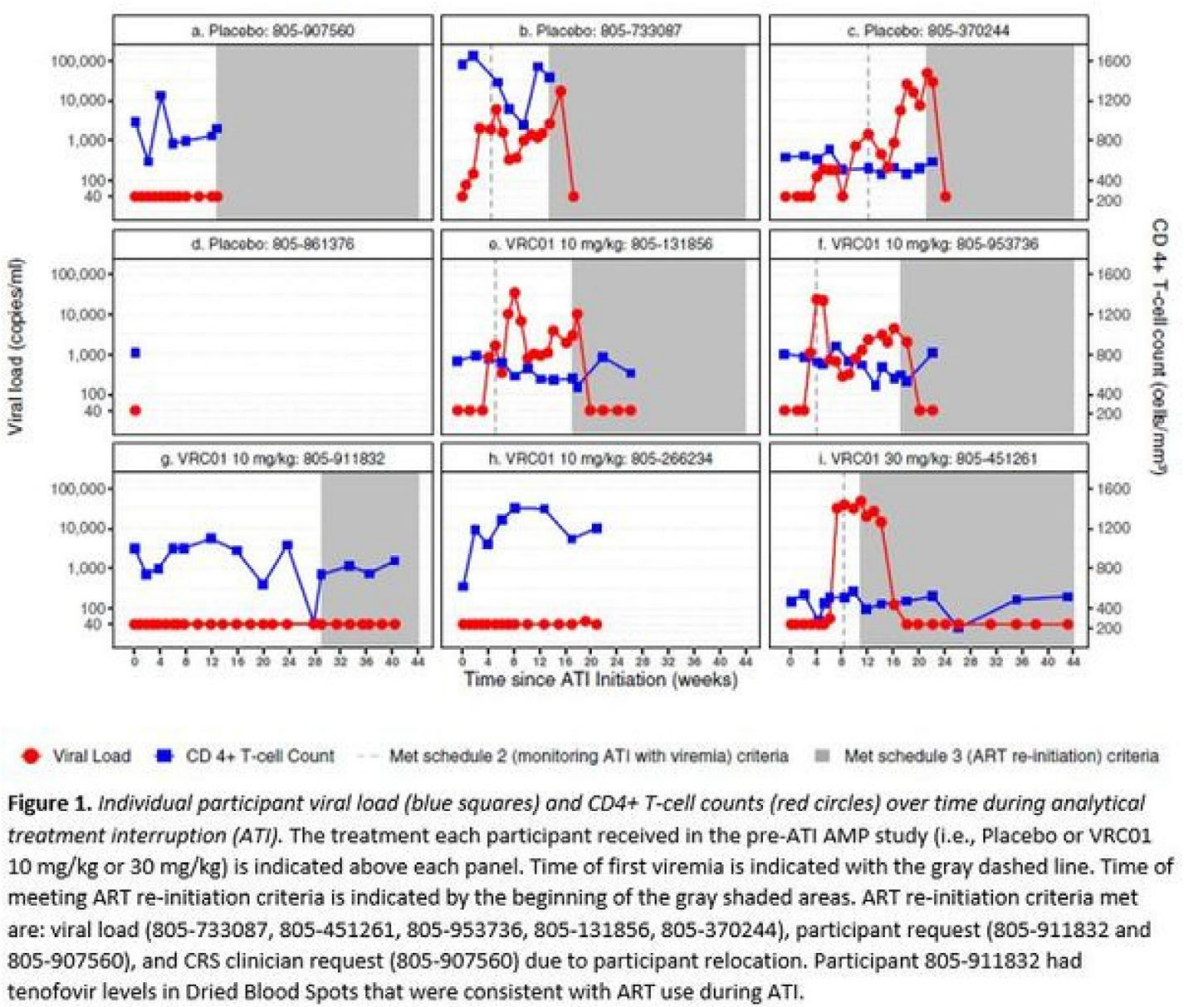
**Abstract EPLBB08‐Figure 1**.

**Conclusions: **In a safe and well‐tolerated ongoing ATI developed with local stakeholder engagement, African women with early ART initiation +/‐ prior VRC01 exhibit evidence of viral rebound and control.

### EPLBB09 Risk factors and prognoses for low‐level HIV‐1 viremia: a long‐time observational study

X. Duan ^1,2^, X. Song^1^, W. Lv^1^, Y. Li^1^, Y. Han^1^, Z. Qiu^1^, W. Cao^1^, T. Li^1,3^

^1^Peking Union Medical Hospital, Department of Infectious Diseases, Beijing, China, ^2^Chinese Academy of Medical Sciences & Peking Union Medical College, School of Clinical Medicine, Beijing, China, ^3^Tsinghua University, Medical College, Beijing, China

**Background: **The risk factors and optimal management of low‐level viremia (LLV) in HIV infection is still controversial. Here, we studied the risk factors for LLV in treated HIV patients, and the impact of viral load (VL), CD4 counts, and ART modification during LLV on virological and immunological prognoses.

**Methods: **We reviewed all outpatients at the HIV clinical center of Peking Union Medical College Hospital in Beijing, China during 2010‐2020. Patients who aged 18‐65, ever achieved virological suppression (VS), and had at least two documented VLs and CD4 counts were included in the study. A case‐control study was designed to figure out the risk factors for LLV. Patients with LLV were then studied to learn the impact of viral load, CD4 counts, and strategies of ART modification on the clinical outcomes including virological failure (VF, VL > 200 copies/ml) and poor CD4 recovery (CD4 counts < 350 cells/mm^3^).

**Results: **The case‐control study showed that the modification of ART regimens (OR =2.309, 95% CI [1.302‐4.191], *p =*0.005) and higher zenith VL (OR = 1.331, 95% CI [1.091‐1.623], *p*= 0.005) were independent risk factors for LLV. In the second part, of 43 patients with LLV, 2 (7.9%) had a subsequent VF and 17 (44.4%) showed poor CD4 recovery. Immunological suboptimal responder during LLV (HR =3.015, 95% CI [1.000‐9.094], *p* =0.050) and higher zenith CD4 (HR = 0.995, 95% CI [0.991‐0.999], *p* =0.012) were respectively independent risk and protective factors. However, LLV did not dramatically alter the dynamic of CD4 changes before and after LLV. Modification of ART regimens during LLV did not help to reverse either virological or immunological outcomes.

**Conclusions: **These findings suggested that a powerful and sustainable initial ART regimen to avoid frequent adjustment of suboptimal regimens may be key to preventing LLV. The incidence of VF post LLV was relatively low but poor CD4 recovery was common. Modification of ART regimens during LLV had no necessity or help for reversing the virological and immunological outcomes post LLV.

### EPLBC01 Mapping and estimating the size of virtual key populations: potential approaches

r. adhikary ^1^, A. Rao^2^, S. Talwar^3^

^1^WHO India Country Office, HIV, Hepatitis and STIs unit, Communicable Diseases, New Delhi, India, ^2^National AIDS Research Institute, Pune, India, ^3^Actions Research Centre, Mumbai, India

**Background: **The dynamics of key population is changing very fast all over the world including India. More and more of them shifting increasingly from traditional physical hotspots to the virtual space due to rapid development in information technology as well as other underlying factors. Some discreate efforts are being made across some countries to map the virtual sites as well as estimate the size of key populations operating from there. However, no sound, reliable and convincing methodology is yet to be available to map and estimate the size of virtual key populations for designing appropriate interventions among them.

**Methods: **National AIDS Research Institute of India and World Health Organization undertook an in‐depth assessment in 2019‐2020 to develop few potential approaches based on extensive literature review and informal consultations with key experts, program managers and key population communities.

**Results: **The in‐depth assessment recommended three alternative potential approaches for mapping and estimating the size of key populations, particularly of men who have sex with other men, transgenders and female sex workers: (1) virtual mapping and respondent driven sampling surveys (2) virtual mapping and online surveys and (3) physical mapping and respondent sampling surveys. All required adjustment factors could be calculated from any of these approaches.

**Conclusions: **The assessment concluded that the recommended three potential approaches need to be field tested for identifying the best approach for a particular key population group in a specific context. Currently, the team is gearing up to field test these approaches as the COVID pandemic is now under control in India.

### EPLBC02 Opioid‐agonist therapy in Ukraine has a large potential for expansion and demonstrates significant reduction of risky injection practices and overdose among people who inject opioid drugs

R. Kulchynska ^1^, Y. Sazonova^1^, N. Podolchak^1^, R. Keating^1^, I. Ivanchuk^2^, I. Titar^2^, M. Azarskova^1^, E.J. Barzilay^1^

^1^CDC‐Ukraine, Overseas Strategy & Management Branch, Division of Global HIV & TB, Centers for Disease Control and Prevention, Kyiv, Ukraine, ^2^Public Health Center of the MOH of Ukraine, Kyiv, Ukraine

**Background: **Opioid‐Agonist Therapy (OAT) in Ukraine is the largest program of its kind in the Eastern Europe and Central Asia (EECA) region, with current coverage of nearly 17,000 clients. Ukraine's OAT program provides methadone or buprenorphine in combination with counseling and social services. We investigated the reduction in unsafe injection practices and drug overdoses among clients who receive OAT but continue to inject drugs.

**Methods: **We analyzed the 2020 Integrated Biobehavioral Survey (IBBS) survey data among people who inject drugs (PWID) in Ukraine. Respondent‐driven sampling was used to recruit 6,001 those who injected drugs in the past 30 days across 12 cities. The questionnaire captured sociodemographics, OAT awareness, eligibility, and participation, and risk behaviors among PWID who inject opioids (n=3,681). We ran four multivariate logistic models to estimate the preventive effect of OAT on usage of non‐sterile syringes, syringe or needle sharing, use of prefilled syringes, and experience of non‐fatal overdoses.

**Results: **In a weighted analysis, 62% of respondents were aware of the OAT program and 19% reported being on OAT. While 24% of respondents expressed interest in OAT, 49% were not planning to ever enroll in it. Other PWID had current or past OAT experience (24%) or failed to provide an answer (2.4%). In adjusted logistic models, current (n=745) OAT users were 25% less likely to report non‐sterile syringe use. OAT users had 45% lower odds of sharing syringes than non‐OAT users (2,936), and they had 43% lower odds of using drugs in pre‐filled syringes. Additionally, OAT clients experienced half the risk of non‐fatal overdose compared to non‐OAT clients.

**Conclusions: **In this large PWID survey, OAT was associated with reduction of unsafe injection behaviors and non‐fatal overdose, regardless of drug cessation. Allowing clients to enroll in OAT regardless of continuing injection practices would remove barriers to enrollment in OAT programs and potentially increase the benefits of OAT in Ukraine. These results could be further used to inform a lower threshold of enrollment requirements for OAT among PWID in Ukraine as well as to increase PWIDs’ awareness of the program.

### EPLBC03 Jitegemee (rely on yourself): acceptability and feasibility of a personal savings intervention to reduce HIV risk among female sex workers in Kisumu and Siaya Counties, Kenya

K. Agot^1^, L. Odeny^1^, J. Arasa ^1^, O. Okumu^1^, M. Ochillo^1^, R. Bosire^1^, N. Okeyo^1^, J. Onyango^1^, H. Thirumurthy^2^

^1^Impact Research and Development Organization, Research, Kisumu, Kenya, ^2^University of Pennsylvania, Center for Health Incentives and Behavioral Economics, Pennsylvania, United States

**Background: **Female sex workers (FSWs) in Kenya bear a disproportionate burden of HIV, mainly due to a high premium attached to unprotected sex with partners of unknown HIV status. Jitegemee (rely on yourself) intervention aims to support FSWs to build a small reserve from their earnings for use when sex work business is not doing well or when they want to decline unsafe sex.

**Methods: **We conducted a cross‐sectional survey on acceptability and feasibility of Jitegemee – an intervention that aims to support FSW to put aside small savings from their earnings to fall back on to avoid situations of HIV risk. Eligible FSW were aged ≥18 years living in Kisumu and Siaya Counties. We explored HIV risk‐taking behaviours; earnings, savings, and spending practices; views on and concerns over Jitegemee; plans to leave sex work; how to improve savings, among other topics. To ensure representation of different FSW typologies, we enrolled participants from entertainment joints, brothels, streets, homes and beaches.

**Results: **Between 01/Feb/2022‐02/Apr/2022, we enrolled 370 FSWs (208 in Kisumu, 162 in Siaya); average age 31 years; 55% primary and 40% secondary education; 22% married/cohabiting; 56.5% joined sex work ≤5 years; average monthly income US$1,227; 86% reporting sex work as primary income. Median monthly savings was US$95 (mainly in mobile money and table banking) while expenditures consumed US$1,860; current loans was US$67. Expenditures‐plus‐loan was higher than income‐plus‐savings by US$351. To augment their income in order to save, FSWs would seek other sources of income (62%), get more customers (29%) or work longer hours (20%). Jigetemee intervention was acceptable to 95% of participants; however, 8% had concerns, of whom 42% perceived it as forcing sex workers out of sex work, 35% as disapproval of sex work, and 19% as a scheme to disappear with savings/use FSWs as a source of income.

**Conclusions: **Jitegemee is acceptable to almost all FWS and would be a sustainable approach to reduce their risk of HIV. However, in order to reduce the risk of HIV by joining a savings intervention, FSW would need to cut down on non‐essential expenses and/or diversity income without engaging in riskier sex.

### EPLBC04 Transgender women (TGW) in HPTN 083: an evaluation of safety, efficacy, and gender affirming hormonal therapy (GAHT) interactions with long‐acting cabotegravir (CAB‐LA)

B. Grinsztejn ^1^, B. Hanscom^2^, Z. Wang^2^, D. Donnell^2^, P. Richardson^3^, P. Sullivan^3^, S. Eshleman^3^, A. Jennings^4^, K. Gomez‐Feliciano^4^, K. Samitpol^5^, E. Jalil^1^, N. Cardozo^6^, B. Maia^7^, T. Khan^8^, Y. Singh^9^, J. Franks^10^, J. Valencia^11^, C. Psaros^12^, S. Safren^13^, N. Sanchez^14^, J. Lucas^4^, C. Blanchette^4^, J. Rooney^15^, A. Rinehart^16^, S. Ford^17^, A. Adeyeye^18^, M. Cohen^19^, M. McCauley^4^, R. Landovitz^20^, M. Marzinke^3^

^1^FIOCRUZ, INI, Rio de Janeiro, Brazil, ^2^Fred Hutch, Seattle, United States, ^3^Johns Hopkins University, Baltimore, United States, ^4^FHI 360, Durham, United States, ^5^Institute of HIV Research and Innovation, Bangok, Thailand, ^6^Fundaction Huesped, Buenos Aires, Argentina, ^7^University of Sao Paulo, Faculdade de Medicina, Sao Paulo, Brazil, ^8^Fenway Health, Boston, United States, ^9^Desmond Tutu HIV Centre, Cape Town, South Africa, ^10^Columbia University, New York, United States, ^11^IMPACTA‐Peru, Lima, Peru, ^12^Harvard Medical School, Cambridge, United States, ^13^Miami University, Miami, United States, ^14^University of Pennsylvania, Philadelphia, United States, ^15^GILEAD, Foster City, United States, ^16^ViiV Healthcare, London, United Kingdom, ^17^GlaxoSmithKline, Brentford, United Kingdom, ^18^NIH, DAIDS, Bethesda, United States, ^19^University of North Carolina, Chapel Hill, United States, ^20^UCLA, Los Angeles, United States

**Background: **HPTN 083 demonstrated a 66% reduced risk of HIV acquisition for long‐acting injectable cabotegravir (CAB‐LA) vs. daily oral TDF/FTC. As transgender women (TGW) remain a priority group for HIV prevention, we report the safety, prevention efficacy, and pharmacokinetics (PK) of CAB‐LA in TGW during the blinded phase of HPTN 083.

**Methods: **Participant characteristics, including history of interpersonal violence, HIV risk perception, and grade 2+ adverse events, were compared between TGW and the larger study cohort. CAB drug concentrations were measured in a subset of TGW with and without gender‐affirming hormonal therapy (GAHT) to evaluate the potential impact of GAHT on CAB PK.

**Results: **Of 4566 participants enrolled in HPTN 083, 570 (12.5%) were TGW (United States 21.9%, Latin America 36.0%, Asia 39.5%, and Africa 2.6%).During the study, 330 (57.9%) TGW reported GAHT use, with estradiol valerate (44.5%) and spironolactone (32.4%) most frequently reported. HIV incidence among TGW was 1.80% (TDF/FTC) and 0.54% (CAB‐LA) during the blinded phase of HPTN 083 (hazard ratio: 0.34, 95%, CI 0.08‐1.56).When compared to the larger HPTN 083 cohort, TGW experienced an increased frequency of sexual (56.7% vs. 45.4%), physical (30.2% vs. 19.2%), and emotional (47.4% vs. 36.4%) interpersonal violence, and lower self‐perceived HIV acquisition risk (53.3% vs. 66.5%). CAB‐LA was well tolerated in TGW; the frequency of grade 2+ adverse events did not differ between TGW receiving CAB‐LA or TDF/FTC (92.5% vs. 88.8%). CAB drug concentrations were measured in a subset of TGW who received on‐time CAB injections (23 not taking GAHT, 30 taking GAHT).CAB drug concentrations were comparable between the two groups, suggesting the lack of a GAHT effect on CAB PK (Figure 1).

**Figure:**
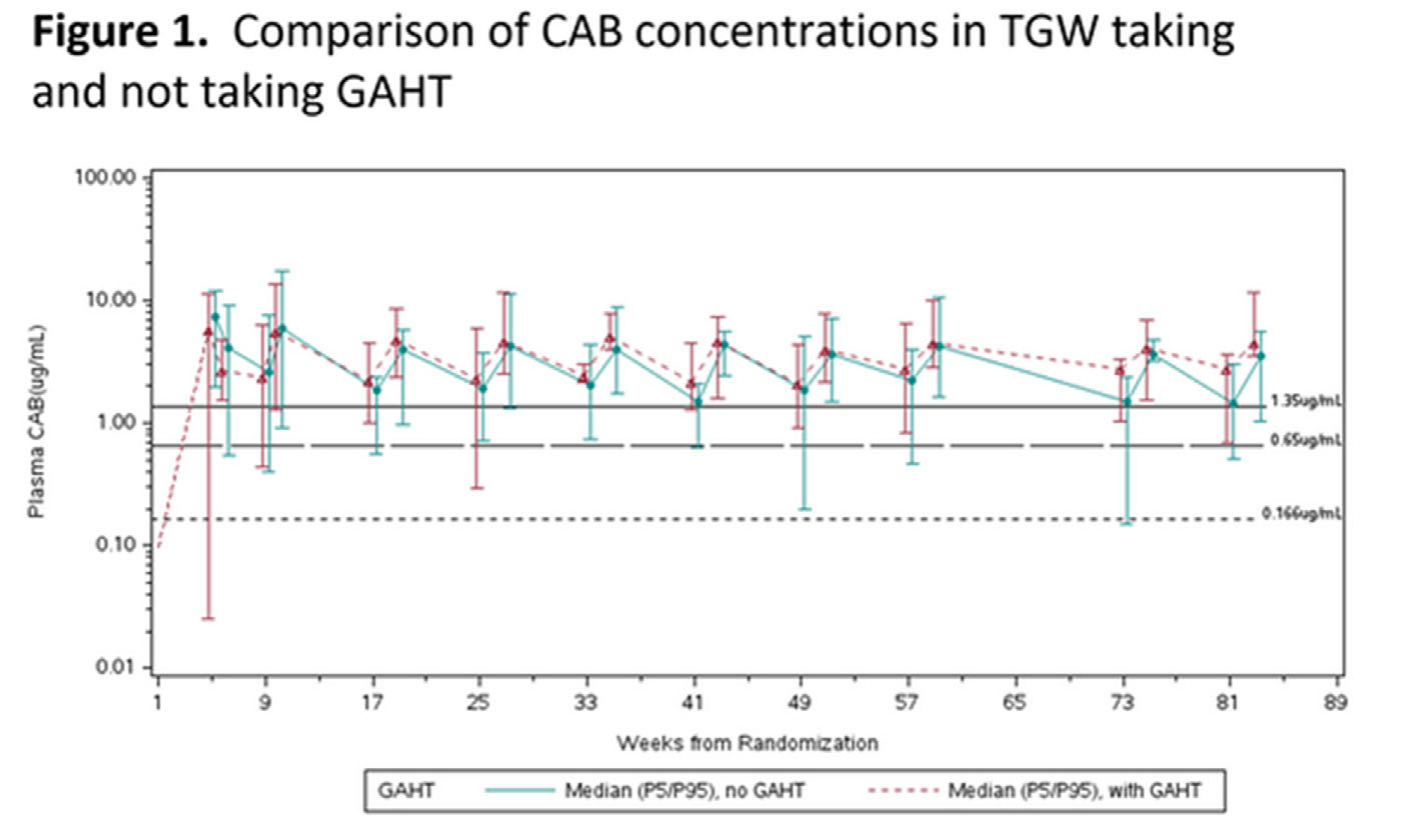
**Abstract EPLBC04‐Figure 1**.

**Conclusions: **CAB‐LA is a safe and effective HIV prevention strategy for TGW. Initial findings suggest there is no impact of GAHT on CAB concentrations.

### EPLBC05 Phase 3B, randomized, open‐label, safety study of dapivirine vaginal ring and oral emtricitabine 200mg/tenofovir disoproxil fumarate 300 mg tablet in breastfeeding mother‐infant pairs

L. Noguchi ^1,2^, M. Owor^3^, B. Gati^3^, E. Horne^4^, N. Mgodi^5^, F. Taulo^6^, R. Scheckter^7^, K. Bunge^8^, H. Gundacker^9,10^, B. Richardson^11,12^, J. Piper^13^, N. Chakhtoura^14^, J. Balkus^15,12^, MTN‐043/B‐PROTECTED Study Team

^1^Jhpiego/Johns Hopkins University, Maternal Newborn Health, Washington, United States, ^2^MWRI/UPMC, Microbicide Trials Network, Pittsburgh, United States, ^3^Makerere University ‐ Johns Hopkins University (MU‐JHU) Research Collaboration, Kampala, Uganda, ^4^Wits RHI, Shandukani Research Centre, Johannesburg, South Africa, ^5^University of Zimbabwe, Clinical Trials Research Centre, Harare, Zimbabwe, ^6^Queen Elizabeth Central Hospital, Obstetrics and Gynaecology, Blantyre, Malawi, ^7^FHI 360, Science Facilitation, Durham, United States, ^8^Magee‐Womens Hospital, University of Pittsburgh Medical Center, Obstetrics and Gynecology, Pittsburgh, United States, ^9^Statistical Center for HIV/AIDS Research and Prevention, Seattle, United States, ^10^Fred Hutchinson Cancer Research Center, Seattle, United States, ^11^University of Washington, Biostatistics and Global Health, Seattle, United States, ^12^Fred Hutchinson Cancer Research Center, Division of Vaccine and Infectious Disease Research, Seattle, United States, ^13^NIAID/National Institutes of Health, Division of AIDS, Bethesda, United States, ^14^NICHD/National Institutes of Health, Rockville, United States, ^15^University of Washington School of Public Health, Epidemiology, Seattle, United States

**Background: **World Health Organization (WHO) guidance supports provision of oral pre‐exposure prophylaxis (PrEP) for breastfeeding people at substantial risk of HIV acquisition. In January 2021, WHO recommended the dapivirine vaginal ring (VR) as an additional HIV prevention choice as part of combination prevention approaches. In March 2022, the VR was approved by the South African Health Products Regulatory Authority. However, data are lacking on VR safety during breastfeeding, a period of increased HIV acquisition risk.

**Methods: **MTN‐043 was a phase 3b, randomized, open‐label trial, with 12 weeks exposure to VR or oral 200 mg emtricitabine/300mg tenofovir disoproxil fumarate tablet. Healthy, HIV‐negative, exclusively breastfeeding mother‐infant pairs enrolled from September 2020 to July 2021 at sites in Malawi, South Africa, Uganda, and Zimbabwe and randomized in a 3:1 ratio (VR: tablet). Adverse events (AEs) were collected throughout product exposure and two weeks following product discontinuation. Primary safety outcomes for mothers and infants included serious adverse events (SAEs) and Grade 3 or higher AEs in both arms.

**Results: **Across sites, 197 mother‐infant pairs enrolled (VR: 148, oral PrEP: 49). Median age of infants was 9 weeks. Among VR arm participants, two (1%) mothers experienced an SAE and three (2%) an AE of Grade 3 or higher; four (3%) infants experienced an SAE, and 10 (7%) an AE of Grade 3 or higher (see table). No SAEs or Grade 3 or higher events in mothers or infants were deemed related to study product.

**Table:** 

**Abstract EPLBC05‐Table 1. Primary safety outcomes among breastfeeding mothers and infants enrolled in MTN‐043**								
	Mothers				Infants			
	Serious Adverse Events		Grade 3 or> Higher Adverse Events		Serious Adverse Events		Grade 3 or Higher Adverse Events	
	n/N	% (95% CI)	n/N	% (95% CI)	n/N	% (95% CI)	n/N	% (95% CI)
Dapivirine Vaginal Ring	2/148	1% (0, 5)	3/148	2% (0, 6)	4/148	3% (1, 7)	10/148	7% (3, 12)
FTC 200 mg/ TDF 300mg oral tablet	0/49	0% (0, 7)	2/49	4% (1, 14)	0/49	0% (0, 7)	1/49	2% (0, 11)

**Conclusions: **In this first evaluation of VR safety during breastfeeding, few SAEs or AEs of Grade 3 or higher occurred among mothers and infants in either study arm; most AEs were mild or moderate, and all infant AEs were unrelated to study product. This favorable safety profile, along with previous data demonstrating low drug transfer to breastmilk, support updates of WHO and other guidelines to include breastfeeding people when recommending the VR as an additional HIV prevention choice.

### EPLBC06 iSTAMP: Implementation of HIV self‐testing among Black and Hispanic MSM recruited online in 11 States, 2020‐2021

R. Mac Gowan ^1^, P. Chavez^1^, R. Dana^2^, M. Hannah^2^, J. Caldwell^2^, J. Johnson^1^, L. Hightow‐Weidman^3^, J. Jones^2^, J. Raiford^1^, T. Sanchez^2^, A. Sharma^4^, A. Smith^1^, R. Stephenson^5^, S. Sullivan^5^, P. Sullivan^2^

^1^CDC, Division of HIV Prevention, Atlanta, United States, ^2^Emory University, Rollins School of Public Health, Atlanta, United States, ^3^University of North Carolina at Chapel Hill, Institute for Global Health and Infectious Diseases, Chapel Hill, United States, ^4^University of Michigan, Department of Health Behavior and Biological Sciences, Ann Arbor, United States, ^5^University of Michigan, Department of Systems, Population and Leadership, Ann Arbor, United States

**Background: **In the US, Black/African American and Hispanic/Latino men who have sex with men (BMSM & HMSM) account for a disproportionate number of new HIV infections. Providing HIV self‐tests (HIVST) allows users to learn their HIV status, thereby contributing to achieving the “Ending the HIV Epidemic in the United States” (EHE) goal of diagnosing all people with HIV as early as possible.

**Methods: **This HIV self‐testing study evaluated the effectiveness of marketing strategies in which advertising materials were specifically developed to recruit BMSM and HMSM through 3 media channels (general‐interest social media, LGBT interest websites, gay dating apps). Eligibility included: >18 years old, not taking PrEP, no prior HIV diagnosis, completing online screener survey, baseline survey and providing contact information. Participants were mailed two HIVST. After completing a 4‐month (4M) survey, participants who did not opt‐out were sent another HIVST and a dried blood spot (DBS) kit. Participants could report HIVST results online before, during or after their 4M survey. We report test results from participants and their social network associates (SNA).

**Results: **We enrolled 2,093 participants (55% BMSM, 45% HMSM); 22% had never tested for HIV. Eighty‐five percent of BMSM were recruited from dating apps and 52% of HMSM from general‐interest social media apps; 1,742 participants provided a HIVST result online before the 4M survey, and 80% (1,668/2,093) completed the 4M survey. 457 participants gave HIVSTs to SNA. During the intervention period, positive results were reported by 9% (156/1,806) of all participants who reported at least one HIVST result, and 15% (61/396) of those never tested before enrollment. The highest percentage of all positive HIVST results were reported from participants recruited from dating sites, 12% (133/1,110). 1396 DBS kits were mailed, 515 were tested, of which 6% were reactive for HIV. Additionally, 10 SNA had a positive HIVST result.

**Table:** 

**Abstract EPLBC06‐Table 1. Reported positive HIV self‐test results by iSTAMP participants by recruitment source**					
Reporting source	Total	General‐ interest social media	LGBT interest websites	Dating sites	Unknown source
**Positive HIVST result reported during 4 month intervention period, by race/ethnicity** Black/AA MSM Hispanic/Latino MSM	**156/1806 (8.6%)** 108/982 (11.0%) 48/824 (5.8%)	5/101 (5.0%) 24/434 (5.5%)	0/3 0/12	102/826 (12.4%) 23/284 (8.1)	1/52 (1.9%) 1/94 (1.1%)
**Positive HIVST result reported during 4 month intervention period, by history of testing at enrollment** Never tested before enrollment Ever tested	**156/1806 (8.6%)** 61/396 (15.4%) 95/1410 (6.7%)	9/152 (5.9%) 20/383 (5.2%)	0 0/15	51/210 (24.3%) 74/900 (8.2%)	1/34 (2.9%) 1/112 (0.9%)
**Positive HIVST result from DBS card** Black/AA MSM Hispanic/Latino MSM	**29/515 (5.6%)** 21/262 (8.0%) 8/253 (3.1%)	1/26 (3.8%) 4/125 (3.2%)	0/2 0/7	20/215 (9.3%) 4/88 (4.6%)	0/19 0/33
**Result of HIVST given to first SNA partner (N=457)** Negative Positive Don't know No response	N (Col %) 223 (48.8%) 10 (2.2%) 27 (5.9%) 197 (43.1%)	N (Col %) 77 (51.0%) 2 (1.3%) 11 (7.3%) 61 (40.4%)	N (Col %) 5 (55.6%) 0 0 4 (44.4%)	N (Col %) 122 (47.1%) 8 (3.1%) 15 (5.8%) 114 (44.0%)	N (Col %) 19 (50.0%) 0 1 (2.6%) 18 (47.4%)
Did SNA know of prior positive result* No	9	1	0	8	0

***Abbreviations***: ST, Self‐Testing; RCT, randomized controlled trial; SNA, Social Network Associate, LGBT, Lesbian, Gay, Bisexual, and Transgender; DBS, Dried Blood Spot

^*^One respondent did not answer whether their SNA had previously tested positive for HIV

Includes all reported infections by participants in surveys or online reporting system.

**Conclusions: **The use of population‐specific ads and specific media channels, especially MSM dating and general‐interest social media sites could enable programs to remotely provide HIVST to BMSM & HMSM, including those who are not using traditional services. This approach will increase awareness of HIV status among BMSM and HMSM and contribute to achieving EHE goals.

### EPLBC07 Number of pregnancies and its impact on adherence to ART during pregnancy in women living with HIV, followed up in 2020 in a specialized health center in São Paulo, Brazil

C. Ferreira Lima ^1^, W. Cesar Ribeiro Campos^2^, N. Zanon Narchi^3^

^1^Universidade de São Paulo, Escola de Enfermagem / Faculdade de Medicina, São Paulo, Brazil, ^2^Universidade de São Paulo, Faculdade de Medicina, São Paulo, Brazil, ^3^Universidade de São Paulo / EACH‐USP, Obstetricia, Sao Paulo, Brazil

**Background: **Being a woman and living with HIV brings enormous challenges: the need to maintain treatment with ART chronically, and the concern with the planning of reproductive life, in order to reduce the risks of maternal and child morbidity and mortality. The objective of this study was to analyze adherence to ART among women living with HIV (WLHIV) who discovered pregnancy in 2020, according to the number of previous pregnancies, assisted in a Specialized Health Care Center (SAE).

**Methods: **Descriptive data analysis from cross‐reference table in SPSS software, version 26. The sample was composed of all WLHIV (n=15), with onset of pregnancy between 01/01/2020 and 12/31/2020, who knew the previous diagnosis, until the conclusion of the pregnancy process, assisted in a SAE, in São Paulo. Data were collected from the prenatal records and medical records. CEP 3.139.029 ‐ SMS/SP and 3.081.173 ‐ EEUSP/SP.

**Results: **Analyzing the variables number of pregnancies x use of ART prior to pregnancy, we identified that the group with only one pregnancy (n=4), 75% made irregular use or no use of ART, in the groups with two or three pregnancies (n=7) and four pregnancies or more (n=4), 28.6% and 25% were in the same situation. In the analysis of the variables number of pregnancies x ART use during pregnancy, analyzing the same groups, we find respective rates of 50%, 0%, and 25% in the category irregular use or no use of ART.

**Conclusions: **Pregnancy was an important factor in stimulating adherence among WLHIV, promoting a reduction in the rates of irregular use and non‐use of ART, especially among women with up to 3 pregnancies. However, it highlights the maintenance of irregular use and non‐use of ART among the group of 4 pregnancies or more. Excessive family responsibilities, psychosocial vulnerabilities and the belief that the child will not acquire HIV due to the negative diagnosis history of previous children are among the justifications reported by WLHIV in medical records for this situation. Reflection on these data denotes the need for improvement in WLHIV monitoring, adjusting strategies, with opportunities for dialogue about reproductive planning in the assistance.

### EPLBC08 Population trends in HIV service delivery, viral suppression, and incidence before and during the COVID‐19 era in Rakai, Uganda

V. Ssempijja ^1,2^, K. Grabowski^2,3^, A. Ndyanabo^2^, R. Ssekubugu^2^, L. Chang^4,2^, H. Nakawooya^2^, F. Nalugoda^5^, G. Kigozi^5^, J. Kagaayi^6^, D. Serwadda^7,8^, M. Wawer^9,7^, R. Gray^9,7^, T. Quinn^4,10^, J. Kabanda^11^, S. Alamo^11^, L. Nelson^11^, L.A. Mills^11^, D. Kabatesi^11^, S.J. Reynolds^7,10,4^

^1^Leidos Biomedical Inc, Clinical Monitoring Research Program Directorate,, Frederrick, United States, ^2^Rakai Health Sciences Program, Statistics unit, Kalisizo, Uganda, ^3^Johns Hopkins University, Pathology, Baltimore, United States, ^4^Johns Hopkins University, School of Medicine, Baltimore, United States, ^5^Rakai Health Sciences Program, Research Coordination Office, Kalisizo, Uganda, ^6^Rakai Health Sciences Program, Executive Directors Office, Kalisizo, Uganda, ^7^Rakai Health Sciences Program, Kalisizo, Uganda, ^8^Makerere Univerisity, Kampala, Uganda, ^9^Johns Hopkins University, Blook, Bloomberg School of Public Health, Department of Epidemiology, Baltimore, United States, ^10^National Institutes of Health, Division of Intramural Research, National Institute of Allergy and Infectious Diseases, Bethesda, United States, ^11^U.S. Centers for Diseases Control and Prevention, Division of Global HIV and TB, Centers for Global Health, Kampala, Uganda

**Background: **There are limited data on the impact of COVID‐19 on African HIV programs. Using data from the Rakai Community Cohort Study (RCCS), we evaluated trends in use of Combined HIV Interventions (CHI) and HIV incidence in southcentral Uganda from 2015 to 2022. During COVID‐19 lockdown, HIV services were provided through community outreach and multi‐month ART prescriptions.

**Methods: **Participants aged 15‐49 years were surveyed in three surveys before COVID‐19 (February 2015– January 2020) and one survey during COVID‐19 (February 2021 – March 2022). Participants were classified as HIV prevalent (HP; HIV‐positive from prior survey), HIV incident (HI; HIV seropositive with prior seronegative result) or newly diagnosed (ND, HIV seropositive without prior survey result). Participants self‐reported HIV testing within 12 months, knowing their HIV status, current use of anti‐retroviral therapy (ART) and being circumcised. Viral loads (VLS) (<1,000 cps/ml) were consider as suppressed. Interrupted time series analysis assessed changes in HIV services before and during COVID‐19.

**Results: (*figure 1)*** 19,390 (52.8% female) participants were included. HIV testing decreased during COVID‐19, whereas it had increased before COVID19 (p<0.001 change in trend). Knowledge of HIV positive status was stable for both HP (p=0.582) and HI/ND (p=0.134). During COVID‐19, ART use continued increasing in both HP (p<0.213) and in HI/ND (p<0.242). VLS was unchanged during COVID19 in both HP (p=0.296) and HI/ND (p=0.667). MC significantly increased during COVID19 (p<0.001). HIV incidence declined from 1.17/100 person‐years(pys) to 0.48/100 pys before COVID‐19, to 0.35/100 pys during COVID‐19 (p<0.066).

**Figure:**
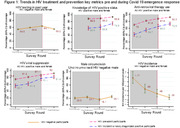
**Abstract EPLBC08‐Figure 1**.

**Conclusions: **We were concerned that the COVID‐19 pandemic and associated lockdown would negatively affect CHI services, but we did not observe such effects, potentially because of intensified/enhanced new outreach approaches. MC uptake increased possibly because post‐operative time off work during lockdown prevented income loss.

### EPLBC09 Effectiveness of COVID‐19 vaccines in people living with HIV in British Columbia: a test negative design

H. Samji ^1,2^, A. Fowokan^3^, J. Puyat^4^, N. Janjua^3,4^, J. Wilton^3^, J. Wong^2,4^, T. Grennan^3^, C. Chambers^5^, A. Kroch^5^, C. Costiniuk^6^, C. Cooper^7^, A. Burchell^8,5^, A. Anis^4^

^1^Simon Fraser University, Faculty of Health Sciences, Burnaby, Canada, ^2^British Columbia Centre for Disease Control, Clinical Prevention Services, Vancouver, Canada, ^3^British Columbia Centre for Disease Control, Vancouver, Canada, ^4^University of British Columbia, School of Population and Public Health, Vancouver, Canada, ^5^University of Toronto, Dalla Lana School of Public Health, Toronto, Canada, ^6^McGill University, Division of Infectious Diseases, Montreal, Canada, ^7^University of Ottawa, Department of Medicine, Ottawa, Canada, ^8^St. Michael's Hospital, Toronto, Department of Family and Community Medicine and MAP Centre for Urban Health, Toronto, Canada

**Background: **The efficacy of COVID‐19 vaccines against severe disease, hospitalizations, and deaths were rapidly established in drug approval trials. Less is known, however, about their effectiveness among immunocompromised individuals such as people living with HIV (PLWH). We therefore sought to estimate the effectiveness of Pfizer‐BioNTech (BNT162b2), Moderna (mRNA‐1273) and AstraZeneca (ChAdOx1) vaccines in a population‐based cohort against laboratory confirmed SARS‐CoV‐2 infections and hospitalizations among PLWH.

**Methods: **We used the British Columbia (BC) COVID‐19 Cohort (BCC19C), which integrates data on SARS‐CoV‐2 tests, COVID‐19 cases, hospitalizations, and immunization with provincial health administrative data. PLWH status was assessed using an adapted version of a previously validated case‐finding algorithm. All PLWH who were living in BC, ≥19 years old, and tested for SARS‐CoV‐2 between December 15, 2020 (when vaccines became available in BC), and November 21, 2021 (time before Omicron variant), were eligible.

Vaccine effectiveness (VE) was estimated by the test‐negative design using multivariable logistic regression to compare the odds of vaccination between test‐positive “cases” and test‐negative “controls”, adjusting for age, sex, area‐level income, health authority, number of COVID‐19 tests 3 months prior to study period, Elixhauser comorbidity index, and bi‐weekly testing periods. We used the formula (1‐AOR) x100% to compute VE.

**Results: **There were 9,116 PLWH in the dataset, 2,657 (29.1%) of whom tested for SARS‐CoV‐2 during the study period and were considered eligible. Of the eligible PLWH, 357 (13.4%) tested positive (cases), while 2300 (86.6%) tested negative (controls); 68 (19.0%) of test positive cases and 254 (11.0%) of test negative controls were unvaccinated. Adjusted VE against SARS‐CoV‐2 symptomatic infection was 78.7% (95% CI = 63.6, 87.5) ≥ seven days after two vaccine doses. VE was preserved until the period four to six months following receipt of two vaccine doses after which slight waning was observed (VE = 66.4% (95% CI = 21.6, 85.6). Adjusted VE against hospitalizations was 88.4% (95% CI= 19.9, 98.3) ≥ seven days after two vaccine doses.

**Conclusions: **Findings suggest that receipt of two COVID‐19 vaccines doses is effective against SARS‐CoV‐2 infections. Future efforts will focus on the impact of variants of concern on VE and comparing VE estimates with a matched HIV‐negative cohort.

### EPLBC10 Using lessons learnt from the implementation of HIV self‐testing in decentralized community settings to increase the uptake for community‐based COVID‐19 Antigen testing in Johannesburg, South Africa

M. Majam^1^, V. Msolomba ^1^, P. Akugizibwe^2^

^1^University of the Witwatersrand, Ezintsha, Johannesburg, South Africa, ^2^FIND, Kigali, Rwanda

**Background: **HIV self‐testing (HIVST) has been a successful strategy used in South Africa, to test hard‐to‐reach individuals and communities. Experience from HIVST points to several barriers that low‐income segments of the population face when trying to access services. These include long wait times at testing facilities, the opportunity cost of time away from their places of work, price sensitivity, and unfriendly services. Accessing target populations in high density community settings such as taxi ranks, plays a vital role in bridging the testing gap. For COVID‐19, access to affordable, time sensitive testing required a similar approach to reach those not accessing testing services.

**Description: **During the third wave of COVID‐19 infections in South Africa between July and October 2021, community based screening for COVID‐19 using point‐of‐care Antigen RDT's was employed in three high‐density taxi ranks in Johannesburg. The intervention, which was initially successfully employed during the roll out of HIV self‐testing in the country, sought to reach populations who may not otherwise access testing services

**Lessons learned: **A baseline evaluation conducted in the taxi ranks prior to the intervention showed that only 21% of respondents had previously tested for COVID‐19 using either PCR or Antigen based testing. Similar to HIV, community members cited (1) lack of access, (2) price of testing, (3) long queues at facilities, as the main reasons for not testing. During the intervention, 15 443 participants were enrolled and screened using a digital risk determination tool. Approximately 33% of all individuals screened were deemed to be at risk of having COVID‐19 and referred for testing. Testing was completed in 3997 cases, with 238 positives (6%) identified. 84% of positives completed 2 weeks of phone‐based follow up. In the general end‐line survey conducted, testing for COVID‐19 increased to 67% of the surveyed population.

**Conclusions/Next steps: **Lessons from HIVST can be applied to implementation of community based Antigen testing for COVID‐19 in order to reach individuals not accessing services. With the scale up of COVID‐19 self‐testing initiatives, utilization of decentralized approaches such as taxi ranks will be required. Coupling of COVID‐19, HIV, and other self‐care approaches has the potential to maximize screening efficiency in community settings.

### EPLBD01 HIV status disclosure and related factors among children aged 6‐14 years living with HIV in Kilimanjaro region, Tanzania

B. Mtesha ^1^, I. Swai^1^, L. Masika^1^, R. Maro^1^, K. Ngowi^1^, M. Sumari‐de Boer1^1^

^1^Kilimanjaro Clinical Research Institute, Data Management Unit, Moshi, Tanzania, The United Republic of

**Background: **In Tanzania, disclosure of the HIV status to children remains a challenge despite the recommendation from the World Health Organization (WHO) which states that children should be informed about their HIV status between the ages of 6 to12 years. The aim of this study is to determine factors associated with HIV status disclosure to children living with HIV in Kilimanjaro, Tanzania.

**Methods: **A cross sectional study using mixed‐methods was conducted from September 2021 to February 2022 among children aged 6‐14years receiving HIV care in Kilimanjaro region. Semi‐structured questionnaires were used to collect socio‐demographic data and reasons of non‐disclosure. We in‐depthly interviewed twenty caregivers of children who had disclosed and not disclosed the status to their children; we also interviewed children who's HIV status had been disclosed. Bivariate and multivariate Logistic regression analysis was performed to identify factors associated with HIV status disclosure. P<0.05 was considered statistically significant. We did thematic content analysis for qualitative data

**Results: **Out of 121 children,51 (42%) had been told they were living with HIV. Eighty‐six percent of children aged above 12years were told their HIV status compared to 28% among children aged 6‐12 years (P<0.001). The percentage of disclosure among girls was 43% and 41% among boys (P=0.8). Among children<5years on ART treatment, it was 30% while it was 53% among does on treatment more than 5 years (P=0.01). Lastly, for those with a treatment supporter, the disclosure were57% and 28% among those without (p=0.002).In the final multivariate model, HIV disclosure was more likely to children aged above 12 years compared to those aged 6‐12 years (OR= 13.5; 95%CI= 4‐46)and children who have a treatment supporter (OR=2.8; 95%CI=2‐7).Through IDI, we revealed the following themes: (1) challenges of disclosure to children, (2) importance of early disclosure, (3) risks of delayed disclosure, (4) feelings when finding out about HIV and (5) accidental disclosure.

**Conclusions: **HIV status disclosure to children living with HIV in Kilimanjaro region was associated with higher age and having a treatment supporter. New strategies should be introduced in Health facilities to make sure that children know their HIV status as recommended by the WHO.

### EPLBD02 Risky business: gender, sexual risk behaviors and vulnerability to HIV infection among South African youth

P. Burns ^1^, J. Ncayiyana^2^

^1^University of Mississippi Medical Center, Population Health Science, Jackson, United States, ^2^University of Kwa‐Zulu Natal, Department of Public Health Medicine, Durban, South Africa

**Background**: Globally, adolescents and young people account for the largest number of people living with HIV. In 2020, 410,000 young people (ages of 10 to 24) were newly infected with HIV. The 10‐19 year category represents the largest proportion of the population in sub‐Saharan Africa.

**Methods**: Utilizing the South African National HIV Prevalence, HIV Incidence, Behaviour and Communication Survey (SABSSM), we conducted a cross‐sectional analysis the association of socio‐demographics and HIV risk behaviors among youth (n=13,454) aged 10–24 years in South Africa. Frequencies and their respective percentages were determined for categorical variables and stratified by biological sex. Chi‐square analysis was used to compare categorical variables and multiple logistic regression to assess associations on two HIV‐related risk behaviorial outcomes. All data were analyzed using SAS software.

**Results: **Results: Of the 13,456 respondents, 3.75 (n=504) were HIV‐positive. The data showed 41.9 (n=3,599) had ever had sexual intercourse. The majority had their sexual debut (70.3%, n=2,977) between the ages 15‐20 years, 42.5% (n=2,869) had 3 or more lifetime sexual partners; 14.5% (n=209) had 3 or more sexual partners in the last 3 months; 18.4% (n=371) had condomless sex and 53.1% (n=169) had concurrent sexual partners in the last 12 months. Next, we assessed the role of social‐demographicphic characteristics (i.e., age, sex, race, marital status, school status), HIV risk perception, and substance disorder (i.e., alcohol, dagga use) on condom use. We found women and girls were less likely to use a condom (IRR=0.66; C.I. .54‐.81) and being married increased the likelihood of using a condom (IRR‐1.12, C.I. 1.06‐1.28). We ran the same model for the outcome number of sexual partners in the last 12 months with similar results. Being female (IRR‐0.64; C.I. .55‐.80. and marital status (IRR‐1.18, C.I. 1.07‐1.28. were consistently statistically significant.

**Conclusions: **We found South African youth suffer high rates of HIV prevalence (3.75) and being female increases your HIV vulnerability. There is an urgent need to implement programs and policies that address gender and cultural norms which sustain gender inequities, gender‐based violence and economic disparities between men and women, particularly as it relates to condomless sex and multiple sexual partners

### EPLBD03 Socio‐structural challenges and solutions to PrEP in rural communities: patient and provider perspectives

M. Teti ^1^, L. Pichon^2^

^1^University of Missouri, Public Health, Columbia, United States, ^2^University of Memphis, Public Health, Memphis, United States

**Background: **Pre‐exposure prophylaxis (PrEP) is an effective HIV prevention tool that remains underused among many at‐risk rural Americans. Improving PrEP delivery in non‐metropolitan areas is essential. HIV cases are rising in the rural U.S., alongside the opioid epidemic, compromised access to healthcare, and high rates of HIV stigma also occurring in these regions.

**Methods: **In‐depth interviews about PrEP knowledge, attitudes, and perspectives were conducted with 47 providers and 40 patients in non‐metropolitan Missouri, a state identified by U.S public health agencies as having a substantial rural HIV burden. Theme analysis was used to identify key challenges and solutions to improve PrEP access and uptake.

**Results: **Patient participants included young people (ages 18‐30) from rural or small‐town Missouri who recently visited a health clinic. Five themes were present in their interviews: low HIV risk awareness despite risk behaviors, false belief in monogamy as HIV protection, low PrEP‐related health and financial literacy, and concern that PrEP conversations were not normative in small towns. Providers included infections disease (ID), primary care (PCP), and AIDS Service Organization (ASO) providers from similar areas in Missouri. Themes included varying PrEP prescribing beliefs by provider type – ID doctors believed their expertise was important, PCPs wanted to prescribe PrEP but lacked correct information, and ASO providers believed anyone could prescribe PrEP; lack of formal PrEP training; hesitancy to treat high risk patients; desire for help from PrEP “experts” via telehealth; and need for patient awareness of prevention options to facilitate uptake.

**Conclusions: **Patients and providers need solutions relevant to their rural and small‐town context. Patients need information about HIV risk and PrEP through broad, normative, and less personal channels because it is not always safe to ask for it (e.g., incorporating into health education classes, posters in waiting rooms and clinic offices that are visible to all who enter). Providers also said it was easier to address PrEP when patients asked. Providers need education that is tailored to provider type. Telehealth has introduced a potential PrEP care model by which national experts can support both providers and patients in real‐time who have PrEP concerns that cannot be answered locally.

### EPLBD04 Gender differences in sexual experience and condom use among in‐school adolescents: a multi‐country study using the global school‐based health survey

M.A. Maruf ^1,2^, Y.‐W. Chiu^1,3^, H.‐Y. Chiou^1,4^

^1^Taipei Medical University, Global Health and Health Security, Taipei, Taiwan, Province of China, ^2^Universitas Muhammadiyah Jakarta, Faculty of Public Health, Jakarta, Indonesia, ^3^Kaohsiung Medical University, Department of Public Health, Kaohsiung, Taiwan, Province of China, ^4^National Health Research Institutes, Institute of Population Sciences, Miaoli, Taiwan, Province of China

**Background: **Adolescents are one of the groups that are vulnerable to early and risky sexual behavior. Previous studies have shown that gender plays a role in shaping differences in sexual and reproductive health behavior so that it has the potential to affect an individual's ability to make decisions regarding safe sexual behavior. This study aims to determine gender differences in sex and condom use in adolescents and their relationship on the Human Development Index and the Gender Equality Index.

**Methods: **This study is a multi‐country study using data from the Global School‐based Student Health Survey involving 267,545 global participants to identify adolescent sexual behavior and condom use. We calculated the odds ratios (OR) with 95% confidence intervals for males versus females’ sexual experience and condom use. The regression analyses were examined to look at whether gender differences in sexual experience and condom use are related to gender inequality and human development indices.

**Results: **The findings revealed significant gender differences in adolescent sexual behavior and condom use. Boys are more likely than girls to have had sex and use condoms at last sex. The effect of gender differences in sex and condom use on the Human Development Index was not found in this study. However, there is a significant effect between gender differences in condom use on the Gender Inequality Index.

**Conclusions: **The results of this study indicate the importance of sexuality education and the provision of reproductive health services for adolescents in order to minimize the consequences of risky sexual behavior, especially for women. Focusing on the health condition of adolescents by considering a gender perspective can have an impact on reducing the gap in health conditions based on gender in adulthood.

### EPLBD05 Beyond HIV stigma reduction: effects of the CHAMPs‐In‐Action inervention on stress and coping during COVID‐19

J.P.‐H. Wong^1^, A.T.‐W. Li ^2^, K. Prabakaran^2^, L. Brown O'Sullivan^3^, T. CHAMPs‐In‐Action Alliance^4^

^1^Toronto Metropolitan University, Daphne Cockwell School of Nursing, Toronto, Canada, ^2^Committee for Accessible AIDS Treatment, Toronto, Canada, ^3^Committee for Accessible AIDS Treatment, Toronot, Canada, ^4^CHAMPs‐In‐Action Alliance, Toronto, Canada

**Background: **Racialized groups in Canada bear a disproportionate burden of HIV. Their vulnerability to HIV is elevated by structural violence of racism, sexism, homophobia, transphobia, and HIV related stigma. To address these complex challenges, five community HIV/AIDS organizations in Toronto formed an Alliance and secured resources to carry out CHAMPs‐In‐Action (2017‐2022), an evidence‐based intervention that consisted of 4‐day in‐person experiential learning to promote psychological flexibility and reduce HIV related stigma. Implementation was disrupted midway by COVID‐19; the intervention was converted to six weekly online modules in 2020 to enhance access.

**Methods: **CHAMPs‐In‐Action applied psychological and group empowerment processes to reduce stigma. We engaged participants who self‐identified as: aged > 16, a service user/volunteer of an Alliance organization, living with or vulnerable to HIV, or a service provider serving people living with HIV (PLHIV). Validated scales were used pre‐, immediate post‐ and 3‐month post‐ intervention to assess effectiveness. Focus groups were used to explore participants’ experiences and assess the acceptability, feasibility and sustainability of the program. Thematic analysis was used with the focus group data. Inferential statistics and analyses of variance were used to determine the effectiveness of CHAMPs‐In‐Action over time in reducing stigma and increasing psychological flexibility.

**Results: **A total of 362 participants graduated from CHAMPs‐In‐Action: 139 (38.4%) service providers; 87 (24%) PLHIV; 38 (10.5%) gay men and MSM, and 98 (27.1%) racialized persons vulnerable to HIV. Survey results indicated significant increase in resilience, mindfulness, empowerment readiness and confidence to engage others to address HIV related stigma. Focus group results indicated that participants who graduated before the pandemic were able to apply mindfulness and defusion techniques during COVID‐19 to cope with social isolation, anxiety, stigma and stress. Participants who engaged in the online intervention during the pandemic also reported improved coping, reduced stress and better social connection.

**Conclusions: **CHAMPs‐In‐Action is effective not only in reducing stigma, but also in promoting resilience and mental health. Racialized PLHIV experience intersecting stigma and increased social isolation during a pandemic. Online programs are critical in reducing social isolation among PLHIV. Pandemic preparedness needs to address the digital divide and build mutual support networks that tap into community strengths.

### EPLBD06 Factors associated with mental health outcomes among people living with HIV co‐infected with SARS‐CoV‐2 in France: results from COVIDHIV study

I. Yaya ^1,2^, L. Yombo‐Kokule^1,2^, G. Roucoux^1,2^, F. Thonon^1,2^, D. Zucman^3,4^, C. Duvivier^5^, K. Lacombe^6^, M. Preau^7^, A. Cheret^8^, M. Duracinsky^1,2,8^

^1^URC ECO ‐ Hôpital Hôtel‐Dieu, Paris, France, ^2^Patient‐Reported Outcomes Research (PROQOL), UM1123; University de Paris, Inserm, Paris, France, ^3^Foch Hospital, Suresnes, France, ^4^Trait d'Union – Strasbourg University Hospitals, Strasbourg, France, ^5^AP‐HP ‐ Necker‐Enfants Malades Hospital, Infectious Diseases Department, Necker‐Pasteur Infectiology Center, Paris, France, IHU Imagine, Paris, France, ^6^Saint Antoine Hospital, Paris, France, ^7^Inserm Unit 1296 « Radiations : Defense, Health, Environment » ; Lyon 2 Lumière University, Lyon, France, ^8^Internal Medicine Unit, Le Kremlin Bicêtre Hospital, Bicêtre, France

**Background: **The 2019 pandemic of the coronavirus disease (COVID‐19) was found to have a negative impact on vulnerable people, including people living with HIV (PLHIV). This study aimed to investigate the mental health among PLHIV co‐infected with SARS‐CoV‐2 in France.

**Methods: **COVIDHIV is a cohort of PLHIV co‐infected with SARS‐CoV‐2 followed‐up in France. Socio‐demographic, clinical data and those on mental health were collected. The depression and anxiety symptoms, and post‐traumatic stress disorder (PTSD) were assessed by the Hospital Anxiety and Depression Scale (HADS) and PTSD Checklist (specific version) (PCL‐S), respectively. Multivariable logistic regression analysis was performed to identify factors associated with mental health outcomes at the baseline.

**Results: **A total of 397 participants were included, with a mean age (±SD) of 52 ±12.0 years. About two‐thirds of the participants (64.0%) were male, 61% were employed and half of them lived in a couple. Rates of mental health symptoms were 22.6% for depression, 34.2% for anxiety, 53.9% for insomnia, and 12.7% for PTSD. In multivariable regression adjusted for age and duration between COVID‐19 confirmation and enrolment, female gender (adjusted odds ratio (aOR) = 1.95, 95% CI: 1.13‐3.38), being professionally active (aOR = 0.52, 95% CI 0.30‐0.90), fatigue (aOR = 3.17, 95% CI 1.75‐5.75), and cannabis use (aOR = 2.73, 95% CI 1.03‐7.26) were associated with anxiety; being professionally active (aOR = 0.32, 95% CI 0.18‐0.59) and fatigue (aOR = 2.04, 95% CI 1.07‐3.88) were associated with depression; and fatigue (aOR = 3.15, 95% CI 1.24‐7.98) and self‐perceived as vulnerable to COVID‐19 (aOR = 2.16, 95% CI 1.03‐4.52) were found as associated factors for PTSD.

**Conclusions: **This study highlighted the high prevalence of mental health outcomes at the baseline, and these symptoms should be part of the management of PLHIV with SARS‐CoV‐2.

### EPLBD07 The COVID‐19 health crisis has disproportionately impacted sex workers compared to other key populations: preliminary results from the multi‐country community‐based EPIC research program

N. Lorente^1,2,3^, R.M. Delabre ^1^, L. Riegel^1^, C. Folch^2,3^, O. Apffel Font^1,4^, G. White^5,6^, J. Castro Avila^1^, R. Diagne^6^, O. Bourhaba^7,6^, L. Kretzer^8,6^, M. Magassouba^9,6^, J.M. Mutima^10,6^, E.A. Kambire^11,6^, G. Girard^12^, A. Velter^13^, C. Lacoux^14,1^, T. Cerveau^6^, A. Ben Moussa^7,6^, R. Freitas^15,1^, L. Sagaon‐Teyssier^12,16^, H. Mendoza^17,1^, B. Spire^12^, I. Aristegui^18,1^, D. Rojas Castro^1,12^, EPIC study group

^1^Coalition PLUS, Community‐ based Research Laboratory, Pantin, France, ^2^Centre d'Estudis Epidemiològics sobre les Infeccions de Transmissió Sexual i Sida de Catalunya (CEEISCAT), Departament de Salut, Generalitat de Catalunya, Badalona, Spain, ^3^Centro de Investigación Biomédica en Red de Epidemiología y Salud Pública (CIBERESP), Madrid, Spain, ^4^Coalition des organismes communautaires québécois de lutte contre le sida (COCQ‐SIDA), Montréal, Canada, ^5^PILS, Saint‐Louis, Mauritius, ^6^Coalition PLUS, Community‐ based Research Laboratory, Dakar, Senegal, ^7^Association de Lutte Contre le Sida (ALCS), Casablanca, Morocco, ^8^Corporación Kimirina, Quito, Ecuador, ^9^ANCS, Dakar, Senegal, ^10^ANSS, Bujumbura, Burundi, ^11^REVS PLUS, Bobo‐Dioulasso, Burkina Faso, ^12^Aix Marseille Univ, Inserm, IRD, SESSTIM, Sciences Economiques & Sociales de la Santé & Traitement de l'Information Médicale, ISSPAM, Marseille, France, ^13^Santé Publique France, Saint‐Maurice, France, ^14^AIDES, Pantin, France, ^15^GAT, Lisbon, Portugal, ^16^ARCAD Santé PLUS, Centre Integré de Recherche, de Soins et d'Action Communautaire (CIRSAC), Bamako, Mali, ^17^Instituto para el Desarrollo Humano (IDH), Cochabamba, Bolivia, Plurinational State of, ^18^Fundación Huésped, Buenos Aires, Argentina

**Background: **To describe the impact of the Covid‐19 health crisis on specific key populations (KPs): people living with HIV (PLHIV), sex workers (SWs), men who have sex with men (MSM), and people who use drugs (PWUD), in 27 countries.

**Methods: **Coalition PLUS, an international network of community‐based organisations fighting against HIV and hepatitis, initiated the multi‐country and community‐based research program EPIC to document the impact of the Covid‐19 health crisis on KPs and community health workers. Quantitative data were collected among N=10583 respondents from KPs, between June 2020 and March 2022, in 28 countries, mainly from Africa, Latin America, and Europe. We present preliminary data comparing PLHIV (n=3932), PWUD (n=1383), MSM (n=2965) and SWs (n=2303), using Chi‐square tests.

**Results: **Median[IQR] age of respondents was 32[26‐41], 39% self‐identified as female, 55% male, 6% transgender person. Overall, 16% of foreign‐born respondents were undocumented (22% and 25% in PWUD and SWs, respectively, vs. ≤12% in other KPs; p<0.001), and 28% were in unstable housing (60% in PWUD vs. ≤26% in other KPs). The negative impact of the crisis on quality of life was more often reported in SWs and PLHIV (48% and 47%, respectively) than in other KPs (≤39%, p<0.001). SWs also reported more often: a deterioration of their financial situation (85% vs. ≤75% in other KPs; p<0.001) and a negative impact on their personal and professional lives (83% and 84%, respectively, vs. ≤74% and ≤73%, respectively, in other KPs; p<0.001) since the beginning of the health crisis. Having asked/received support from the organisation that implemented EPIC in their country was less often reported in SWs (36%) than in other KPs (>46%, p<0.001). A significant proportion of SWs reported they felt more at risk of HIV infection with clients and non‐clients (28% and 23%, respectively), than before the crisis.

**Conclusions: **This preliminary analysis highlights the disproportionate impact of the COVID‐19 health crisis on SWs compared to other KPs, although all were highly affected by the health crisis. Deeper analyses are needed to identify possible levers for community health and other health workers to better support KPs in time of health crisis, and especially SWs.

### EPLBD08 Limited awareness of HIV status hinders uptake of treatment among female sex workers and sexually exploited adolescents in Wau and Yambio, South Sudan

A. Bolo ^1^, P. Ochira^2^, A.J. Hakim^3^, J. Katoro^4^, S. Bunga^5^, H. Pasquale^6^, V. Anib^7^, G. Caesar Arkangelo^8^, B. Nyokani^9^, A.G. Okiria^10^

^1^US Centres for Disease Control, Global Health, Juba, South Sudan, ^2^IntraHealth International, Strategic Information, Juba, South Sudan, ^3^US CDC, Surveillance Branch, Atlanta, United States, ^4^US CDC, Laboratory, Juba, South Sudan, ^5^US CDC, DGHT, Juba, South Sudan, ^6^Ministry of Health, HIV/STI, Juba, South Sudan, ^7^Ministry of Health, Health, Juba, South Sudan, ^8^MOH, Surveillance, Juba, South Sudan, ^9^IntraHealth, Surveillance, Juba, South Sudan, ^10^IntraHealth, Health, Juba, South Sudan

**Background: **HIV prevalence in Female Sex Workers (FSW) is high, approximately 2.2% globally and 29.3% in Sub‐Saharan Africa. Several factors determine uptake of HIV testing services (HTS) by female sex workers (FSW) including knowledge of their HIV status. HTS provided entry into the UNAIDS 95‐95‐95 cascade of care. Data regarding HIV burden and access to HIV services among FSW in South Sudan are limited. We conducted a cross‐sectional bio‐behavioural survey (BBS) to determine HIV prevalence and progress towards UNAIDS 95‐95‐95 cascade among this population in Wau and Yambio towns in South Sudan in 2019.

**Methods: **Respondent‐driven sampling (RDS) was used to recruit women and sexually exploited girls aged 13‐18 years who exchanged sex for goods or money in the past 6 months and resided in the town for at least 1 month. Consenting participants were interviewed using questionnaire programmed in Open Data Kit tablets and then tested for HIV. Those found HIV positive were tested for viral load (VL). Data were weighted in RDS Analyst and analyzed with Stata 13.

**Results: **A total of 1,284 participants were recruited, 679 in Wau and 605 in Yambio. HIV prevalence was 6.7% in Wau and 13.6% in Yambio. Table 1, presents the HIV cascade for Wau, Yambio and overall for both towns.

**Table:** 

**Abstract EPLBD08‐Table 1: Participants HIV Cascade**					
Participant location	HIV Prevalence	Self‐reported HIV‐positive status (1^st^ 95)	Self‐ reported on ART (2^nd^ 95)	VLS for self‐reported HIV‐positives on ART (3^rd^ 95)	VLS for all participants testing HIV‐positive
Wau	52 (6.7%) 4.1%‐9.4%	23 (35.4%) 15.9%‐51.9%	23 (100.0%)	19 (91.3%) 6.4% – 100.0%	37 (65.0%) 45.0%‐80.8%
Yambio	94 (13.6%) 10.6%‐16.5%	68 (73.0%) 62.5% ‐83.4%	62 (89.8%) 81.0%‐98.5%	55 (93.2%) 88.5%‐97.9%	77 (86.2%) 77.6%‐91.8%
Wau and Yambio	146 (11.2%) 9.3%‐13.4%	91 (64.8%) 55.0%‐73.6%	85 (91.0%) 80.1%‐96.2%	74 (93.0%) 85.9%‐96.6%	114 (81.6%) 73.2%‐87.7%

n (%) 95% CI where “n” is absolute number.

**Conclusions: **Being unaware of HIV‐positive status, limits the uptake of HIV treatment among FSW in South Sudan. This underscores the importance of optimised case‐finding approaches to increase HTS among FSW and sexually exploited minors.

### EPLBD09 Fathering a child with a female sex worker: the experience of male intimate partners of female sex workers in Kampala, Uganda

M. Mbonye ^1^, G. Siu^1^, J. Seeley^2^

^1^Makerere University, Child health and Development Centre, Mulago Hospital complex, Kampala, Uganda, ^2^London School of Hygiene and Tropical Medicine, London, United Kingdom

**Background: **The number of women in sub‐Saharan Africa who become pregnant, often unintentionally, while engaging in sex work is high. We examine the meaning of fatherhood to men in relationships with FSW, the significance of children, and how these men navigate the economic and cultural challenges of providing for their families, juxtaposed with the social stigma associated with sex work.

**Methods: **In 2019, we conducted repeat in‐depth interviews with thirteen men who were involved in relationships with female sex workers (FSW). These data were augmented by observations in the different settings of the 13 men and with information from focus group discussions with FSW who had children with non‐commercial partners.

**Results: **Accepting the role of a `father’ was a challenge for many men because of the circumstances under which they had become fathers. It was a struggle against the ideal way to become a father. Women reported how easy it was for men to shun their responsibilities when they found out about a pregnancy.

Once a FSW became a mother, men who could not severe the relationship, with time, thought about the best way to integrate the sex workers into their extended families. Men were aware that this would not be an easy task given the strong social stigma against sex workers and the moral questions involved. Women's access to money and other opportunities ensured that they were in a strong position to receive approval of the partners’ relatives.

Children became a focal point for the men that helped establish and cement relationships. Children conveyed different social meanings to the men such as leading to transitioning to adulthood and were an important resource for constructing a desired masculinity. Being a provider was perceived by the men as the most important of all the roles of a father, and this narrative, often overshadowed the discussion about other parameters of fatherhood.

**Conclusions: **Men who have children with sex workers face hurdles fitting within the social construction of ideal fatherhood and masculinity. However, when, they come to embrace fatherhood they may wish to deal with the social and economic challenges of raising children, as a couple.

### EPLBE01 Scaling up integrated HIV service delivery for key and priority populations in catholic health facilities in Kampala and Wakiso districts, Uganda

J. Muhangi ^1^, J. Nyakato^1^, L. Ocen^1^, C. Nandago^2^, J. Ekong^1^, C. Ajulong^3^, F. Arthur G^3^, R. Kamara^1^, S. Orach^1^

^1^Uganda Episcopal Conference, Uganda Catholic Medical Bureau, Kampala, Uganda, ^2^Uganda Empowerment Mission, Kampala, Uganda, ^3^US Centers for Disease Control and Prevention, Division of Global HIV & TB, Division of Global HIV & TB, Kampala, Uganda

**Background: **HIV prevalence among key and priority populations (KP/PPs) in Uganda remains higher than the national prevalence, yet these populations are stigmatized, discriminated against, and excluded from some mainstream HIV programming. Catholic healthcare networks are key partners in the HIV epidemic but are perceived as non‐inclusive to KP/PPs, so we used a multi‐pronged approach to improve KP/PP service delivery in Catholic facilities in Uganda's Kampala and Wakiso districts.

**Description: **Uganda Catholic Medical Bureau (UCMB) integrated pre‐exposure prophylaxis (PrEP), sexual and reproductive health, HIV care, and gender‐based violence services into the KP/PP services package available through Catholic facilities.UCMBtrained and mentored health workers (HWs) on stigma‐free screening and conducted Continuing Medical Education to improve HW attitudes and perceptions towards KPs. HWs conducted routine screening with KPs to identify their needs, provided psychosocial support and behavior change communication, and operationalized flexi‐hour clinics to improve access and privacy. UCMB partnered with the KP‐led Uganda Empowerment Mission (UGEM) for behavior change dialogue meetings and hotspot outreaches, engaging KP peer leaders to mobilize KPs, distribute commodities, scale up partner testing and PrEP and HIV treatment initiation, screen and treat sexually‐transmitted infections and TB, and strengthen facility referrals.

The numbers of KPs receiving comprehensive HIV prevention, testing, and treatment initiation services increased 9.7‐fold from 746 served in 4 facilities in the first quarter (Oct‐Dec 2020) to 7,255 in 21 facilities in the fourth quarter (Jul‐Sep 2021). PPs served increased 8.8‐fold (874 to 7,700); PrEP distribution to KP/PPs increased 9.0‐fold (101 to 907). Served KPs comprised 69% female sex workers, 10% men who have sex with men, 10% transgender persons, and 11% persons who inject drugs. PPs comprised adolescent girls and young women (48%), fisher‐folks (23%), migrant workers (23%), truck drivers (23%), and discordant couples (5%).

**Lessons learned: **Despite Catholic teachings on sexuality, Catholic health facilities have maintained a tradition of compassionate care. They effectively provide KP/PP services if guided by KP/PP peers and sensitized about stigmatizing and discriminatory attitudes and practices.

**Conclusions/Next steps: **UCMB will continue providing services to KP/PPs and stigma/sensitivity education to Catholic health facilities and solicit client feedback, KP‐led civil society monitoring to ensure stigma‐free services.

### EPLBE02 Assessing fidelity of mobile service provision of PrEP to adolescent girls and young women and female sex workers within decentralized priority population programming in South Africa

A. Varnauskas ^1^, C. Singh^2^, L. Shipp^1^, K. Meek^1^, M. Mcingana^2^, J. Mcloughlin^2^, B.A. Nonyane^1^, S. Baral^1^, H. Hausler^2^, S. Schwartz^1^

^1^Johns Hopkins University, International Health, Baltimore, United States, ^2^TB HIV Care, Cape Town, South Africa

**Background: **Decentralized, mobile van delivery of pre‐exposure prophylaxis (PrEP) is increasingly used to reach priority populations. We assessed the extent to which mobile van PrEP provision is being implemented as planned for female sex workers (FSW) and adolescent girls and young women (AGYW) within a large South African non‐profit service provider.

**Methods: **We assessed PrEP implementation across 13 TB HIV Care program sites from May 2021‐April 2022. An observational checklist was used to assess fidelity to standard operating procedures (SOP) and quality of service delivery, including patient‐centeredness, amongst a random sample of 116 provider‐patient visits. Weighted fidelity scores were calculated as a proportion of SOP steps completed for each observational visit, upweighting ‘essential steps’ as identified by program implementers. Scores were assessed per checklist adherence and moderators to fidelity. Case audits among a stratified sample of 1160 user records further measured the proportion of clinical and counseling steps recorded in client files.

**Results: **The weighted average fidelity score for FSW initiation and follow‐up visits was 0.52 and 0.51, respectively, and for AGYW initiation and follow‐up visits was 0.60 and 0.56, respectively. In both programs, total visit length was associated with higher fidelity; urban sites were associated with lower fidelity compared to rural sites in the FSW program. The case audits and observations indicated that essential clinical steps such as HIV testing and safety bloods were consistently completed. Quality of service delivery was high on almost all measures across programs, with over 80% of all service users scoring moderate to high on patience, empathy, and friendliness, except for at AGYW follow‐up visits where at least 50% of providers scored moderate to high.

**Conclusions: **Fidelity across programs was moderate, but critical clinical steps related to safety were followed. Other activities related to building patient comprehension and self‐efficacy to use PrEP were minimal, which stresses the urgency for programs to target social, behavioral and cultural barriers to PrEP use. While mobile PrEP provision shows promise, urgent modifications are needed to better address the persistence gap on PrEP and to have a substantial impact on HIV prevention.

### EPLBE03 Maintaining patient care in the context of major and prolonged socio‐political turmoil in Haiti through community care centers for antiretroviral therapy distribution and viral load testing

P. Joseph ^1^, R. Sun^2^, J.E. Mathon^1^, Y. Macius^1^, M.A. Jean Juste^1^, C. Guiteau^1^, S. Koenig^3^, J.W. Pape^1,4^

^1^GHESKIO, Port‐au‐Prince, Haiti, ^2^Harvard University, Department of Government, Cambridge, United States, ^3^Brigham and Women's Hospital, Boston, United States, ^4^Weill Cornell Medical College, New York, United States

**Background: **Persistent and violent civil conflict in Haiti has continued to challenge HIV care provision for patients. To support patient retention, GHESKIO transitioned essential services from its main facilities to community care centers (CCCs), providing antiretroviral therapy (ART) refills, adherence support, and collection of viral load specimens for stable patients in remote locations in and around Port‐au‐Prince.

**Methods: **CCC services were offered beginning in May 2019 to patients with at least 6 months of ART treatment. Data on all ART refills in 5/1/2019‐12/31/2021 from GHESKIO's electronic health records were analyzed. Patients were defined as on‐time if they picked up their ART prescription within 30 days of their scheduled refill date. Viral load (VL) results were analyzed using the latest test for all patients who received valid VL tests in the last 12 months. VL suppression was defined as <1,000 copies/ml.

**Results: **18,625 patients completed ≥1 drug refill visit during the 32‐month study period (41.3% male, mean±SD age 44.0±13.3 years). 51.9% of patients had ≥1 non‐clinic ART refill. Patients attended 128,740 refill visits (6.9±3.6 per patient), 21.1% and 4.9% of which were CCC and home visits, respectively. The proportion of ART refills occurring at CCC and home visits increased from 0% to 39% between May 2019 and June 2020, remaining between 23% and 45% thereafter. Every month, ≥75% of patients were able to receive on‐time ART prescriptions throughout the study period, despite persistent obstructive gang violence beginning in 2019 and increased frequency of kidnapping beginning in 2020. Among 12,252 patients who received a VL test in 2021, 92.3% were on‐time for ART refill. Overall VL suppression among patients with a VL test was 83.4%.

**Figure:**
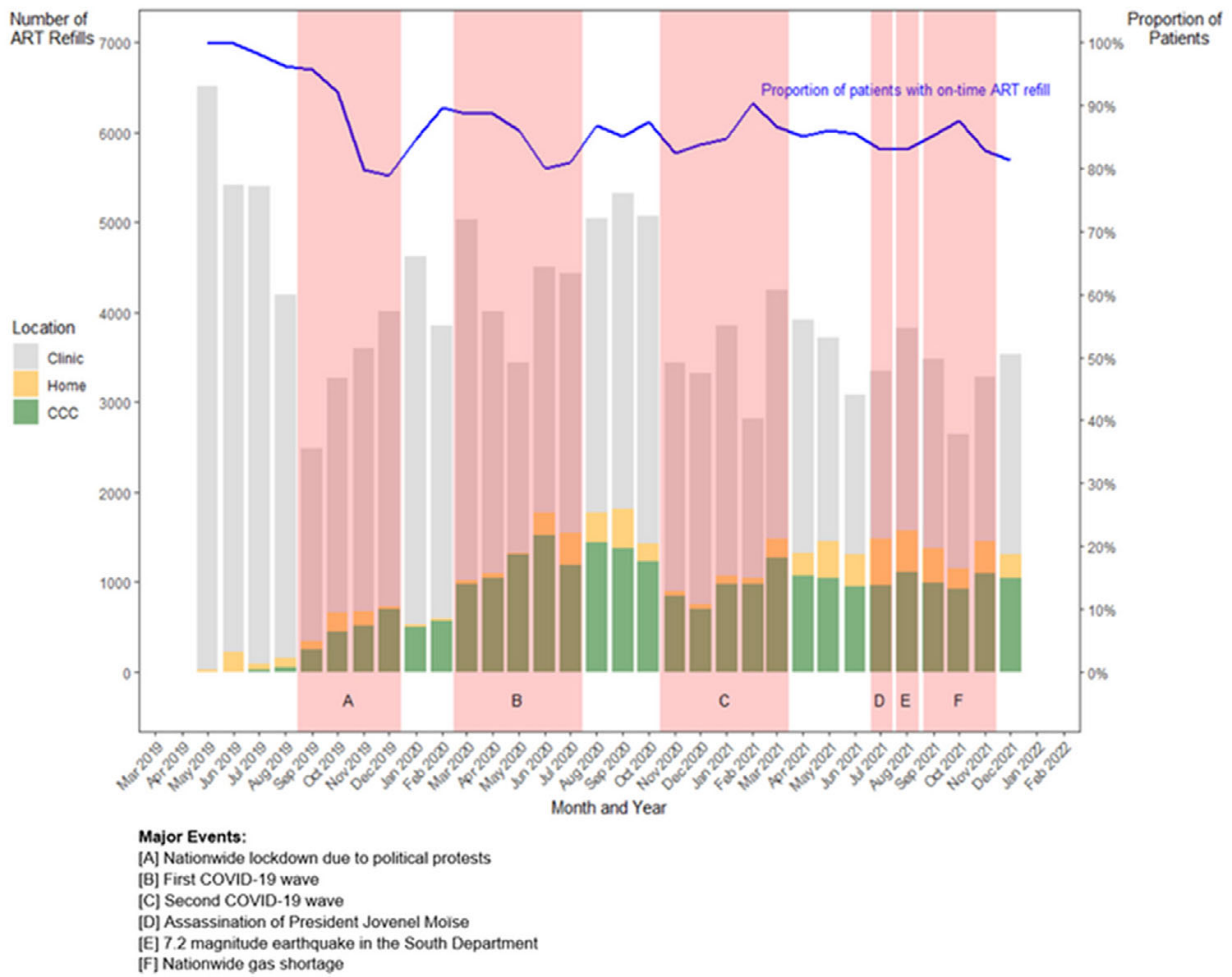
**Abstract EPLBE03‐Figure 1**.

**Conclusions: **Transitioning basic care services from facility‐based to community‐based can support health systems resilience in geographic regions prone to political instability and civil unrest.

### EPLBE04 A novel qualitative assessment tool tracking progress towards sustainability of Zimbabwe's voluntary medical male circumcision program

B. Pindiwe ^1^, S. Xaba^2^, G. Ncube^3^, D. Rama^1^, N. Zwangobani^1^, A. Svisva^1^, N. Shoko^4,5^, T. Kanyenda^5^, Y. Katanda^5^, L. Nyazema^3^, S. Nyaude^3^, P. Kunaka^3^, F. Gwarazimba^3^, B. Nachipo^3^, T. Makoni^3^

^1^Clinton Health Access Initiative, Zimbabwe, Harare, Zimbabwe, ^2^Ministry Of Health and Child Care, AIDS & TB Unit, Harare, Zimbabwe, ^3^Ministry of Health and Child Care, AIDS & TB Unit, Harare, Zimbabwe, ^4^Clinton Health Access Initiative, Boston, United States, ^5^Clinton Health Access Initiative, HIV Prevention, Boston, United States

**Background: **Since 2009, Zimbabwe has implemented voluntary medical male circumcision (VMMC) as a high‐impact, one‐time HIV prevention intervention. While the initial goal of the program was to scale VMMC services, stakeholders recognized that scale up and sustainability must be simultaneously pursued in response to differences in sub‐national performance. This led to the development of the Sustainability Transition Implementation Plan in 2019 which outlined sustainability goals for the VMMC programme. The VMMC Transition Assessment Dashboard (VTAD) assessments were developed to track progress towards country‐defined sustainability goals and to identify health system barriers and enablers to transitioning to sustainability.

**Methods: **The VTAD assessment is designed to collect data on key processes in the VMMC program while tracking progress towards the development of program characteristics necessary for sustainability by programmatic pillar[1]. Data is collected through a consultative process guided by the Ministry of Health and Child Care that prioritizes understanding the qualitative structure and implementation of VMMC activities. Findings from the assessment are incorporated into the district, provincial, and national program planning.

[1] Leadership, management, and coordination(LMC); Service delivery(SD); Programme Quality(Q); Demand Generation(DG); Strategic Information(SI) and Financing

**Results: **Progress towards sustainable LMC1 was driven by increased programme coordination, clearly defined roles and responsibilities, and district level program ownership. Lack of integrated VMMC plans and sub‐optimal engagement of stakeholders were identified as barriers to sustainable LMC1. Modified service delivery models to support program continuity during the COVID‐19 pandemic contributed to a more sustainable SD1. However, vertical and siloed VMMC programming at service delivery points impeded the integrated HIV prevention programming necessary for sustainability. Under the Q1 pillar, integrated and standardised quality assurance activities managed by the district personnel and timely detection and management of VMMC adverse events accelerated sustainability progress. SI and DG1 remained relatively stagnant after adoption of new HMIS tools and updated global guidance on the priority age group requiring new strategies to engage older VMMC clients.

**Conclusions: **The VTAD facilitates structured discourse about VMMC sustainability for all stakeholders while promoting greater district‐level program ownership, data‐driven planning, and identification and monitoring of barriers to and enablers of program sustainability.

### EPLBE05 Community and health services together to avoid interruption of HIV treatment during COVID‐19 restrictions – a person‐centered and a low‐cost initiative for antiretroviral delivery in Florianópolis, Brazil

F.d.B. Perini ^1,2^, M.d.S. Silveira^3^, F.B. Gastaldi^4^, M. Pacheco de Freitas^5^, A.P. da Silva^6^, A.S. Cardoso^6^

^1^Municipal Health Secretary of Florianópolis, Care Integration Department, Florianópolis, Brazil, ^2^Santa Catarina Federal University, POSTGRADUATE PROGRAM IN COLLECTIVE HEALTH, Florianópolis, Brazil, ^3^AIDS Prevention Support Group ‐ GAPA/SC, Florianópolis, Brazil, ^4^Acontece LGBTI+ (Happens ‐ LGBTI+ art and politics), Florianópolis, Brazil, ^5^National Network of People Living with HIV and AIDS (RNP+ Brasil/SC), Florianópolis, Brazil, ^6^Municipal Health Secretary of Florianópolis, Care Integration Department ‐ Pharmaceutical Assistance, Florianópolis, Brazil

**Background: **Avoiding treatment interruption (TI) is among the main challenges in HIV care. COVID‐19 pandemic has exacerbated TI by decreasing health services access. In this context, Florianópolis, a 500,000 inhabitants capital in southern Brazil, with antiretroviral medication (ARV) centralized in 4 municipal pharmacies, joined forces with the community to create new free options for accessing ARV: the ARV Delivery Support Project (ADSP).

**Description: **ADSP was a partnership between the Municipal Health Department of Florianópolis (MHS) and 3 local non‐governmental organizations (NGO), GAPA/SC, Acontece LGBTQI+, and RNP+Brasil/SC. ADSP happened from April 2020 to March 2021, and was publicized by means of local radio, TV, press, and social media. Users, with difficulty in getting their ARV, got in contact with NGO volunteers and had their basic data and request registered. PLHIV also informed how many days of ARV they still had, or if they run out of medication, and could select a nearby primary health care service (PHCS) to withdraw their ARV. This information was shared in a secured digital color coded spreadsheet with the MHS that, subsequently, organized the logistics to send ARV from the pharmacies to one of the 49 capillarized PHCS. NGO volunteers would then contact back PLHIV users to inform that ARV were available for withdraw.

**Lessons learned: **ADSP helped 297 PLHIV; 63% male, median age 40 yo (18‐76); to retrieve their ARV during COVID‐19 restrictions, representing 5% of total PLHIV in treatment in Florianópolis. Without new expenditures, the project carried out 573 distributions in 12 months; a median of 2 (1‐5) distributions per person, 42% of them in the first 3 months. When contacting the NGO, 45% of PLHIV still had up to 7 days of ARV, 22% more than 7 days, but 33% were already without medication. People with long‐term TI (2%) were identified and linked to treatment. Also, NGOs got more visibility and had their staff renewed.

**Conclusions/Next steps: **With innovative initiatives, community engagement, and political will, it was possible to address part of TI challenge. The project and its results have served as the basis for the new MHS ARV home delivery, in partnership with CDC and PEPFAR support.

### EPLBE06 The National Inuit Sexual Health Network ‐ an engagement model for effective health systems transformation across Inuit Nunangat

S. Scott ^1,2^

^1^Pauktuutit Inuit Women of Canada, Health, Ottawa, Canada, ^2^University of Calgary, Calgary, Canada

**Background: **Pauktuutit Inuit Women of Canada (PIWC) is the national representative organization of Inuit women in Canada. We advocate for the social, cultural, political, and economic betterment of Inuit women and their families. Most Inuit in Canada live in 53 communities across four Inuit Regions of Inuit Nunangat, which means "the place where Inuit live:"

Sexual health programming has always, and continues to be a cornerstone of PIWC's Health department since the late 1980's.*Tavva – the National Inuit Sexual Health Strategy*was developed by a group of experts representing each of the Inuit Regions and across provincial, territorial governments, and Inuit governance organizations. Following the development of Tavva, the National Inuit Sexual Health Network (NISHN) was created to implement Tavva.

**Description: **Objectives of the NISHN include:

- Identifying strengths and promising practices within Inuit regions;
- Supporting collaboration among the regions, so efforts are not being duplicated and resources can be shared;
- Providing opportunities for professional development, knowledge transfer and peer support;
- Identifying new trends, emerging issues and infections;
- Clearly identifying priority activities and expectations for the Network.

**Lessons learned:** *Tavva*provided insight to a holistic conception of sexual health beyond combatting STBBIs. As such, PIWC shifted and expanded the scope of outreach to connect with potential members such as midwives, youth, and other Inuit.

Lessons learned in the past year have been published by the NISHN on the Pauktuutit website:

*‐ Uuktuutit* brings conversations on healthy Inuit sexuality closer to the lived realities. Listed components of healthy Inuit Sexuality include positive body image, self‐determination, and intergenerational communication.

*‐ Ikajurniq*recommended 12 unique policies and practices to policymakers and healthcare providers, such as implementing strict confidentiality practices and using peer educators for health promotion.

**Conclusions/Next steps: **Upcoming activities include:

- supporting the launch of an Inuit‐specific lactation clinic
- working with territorial governments on a mobile testing clinic to address confidentiality concerns
- facilitating a land‐based retreat in the Western Arctic to explore how the pandemic has disrupted relational foundations at a community level

The NISHN has inspired next steps to include maternal health considerations and build up the relational foundations that uphold sexual health such as intergenerational communication.

### EPLBE07 Lessons learned in community‐led monitoring: early evidence from global study of the implementation landscape

A. Sharp^1^, N. Mpofu ^2^, C. Mashoko^3^, S. Dringus^1^, B. Honermann^4^, A. Russell^5^, K. Kaplan^6^, B. Ajonye^7^, M. Mikaya^8^, N. Rambau^9^, C. Aguiar^1^, N. Erondu^1^, E. Lankiewicz^4^, N. Ledan^5^, M. Kavanagh^1^

^1^Georgetown University, O'Neill Institute for National and Global Health Law, Washington, United States, ^2^Treatment Action Campaign, Johannesburg, South Africa, ^3^Advocacy Core Team, Harare, Zimbabwe, ^4^amfAR, the Foundation for AIDS Research, Public Policy Office, Washington, United States, ^5^Health GAP, Washington, United States, ^6^Asia Catalyst, Bangkok, Thailand, ^7^International Community of Women Living with HIV Eastern Africa, Kampala, Uganda, ^8^MANASO, Lilongwe, Malawi, ^9^Ritshidze, Johannesburg, South Africa

**Background: **Achieving the global 95‐95‐95 targets is critically dependent on finding the missing positives, addressing unacceptably high loss to follow‐up rates and reengaging people living with HIV into treatment and care.Community‐led monitoring (CLM) is an important approach for improving the quality of healthcare services through social empowerment and political accountability.Driven by increasing support from donors, a growing number of countries are implementing CLM, creating an optimal time to identify early best practices in CLM implementation.

**Methods: **Participants were recruited using a screening tool, disseminated by email and social media.Projects that met inclusion criteria participated in a quantitative survey and an individual interview.Surveys and interviews focused on developing a global mapping of CLM projects, identifying implementation arrangements and activities, and understanding best practices and challenges.Projects monitoring HIV, tuberculosis, malaria, human rights, and/or COVID‐19 were included in the sample.

**Results: **Thirty‐five projects, representing 23 countries, completed the survey, of which 25 additionally participated in an interview.Projects most commonly monitored indicators related to HIV (82% of projects) and TB (74%), and most countries represented were in Sub‐Saharan Africa (77%). The most commonly‐reported donor was the Global Fund (61%), followed by PEPFAR (37%). Among projects’ reported achievements were an increased capacity for local organizations to conduct advocacy (63%), collect data (60%), and more frequent and productive engagement with governments (60%).

Respondents described challenges around COVID‐19 disruption (57%), sustainability of funding (54%), and human resources (46%). Additional challenges identified during interviews included funding levels and on‐time disbursement, challenging funding models, project independence and data ownership, difficulties with timely data analysis, and the need for strengthened advocacy.

Best practices included early and continuous engagement with communities, host governments, and service users, hiring dedicated and paid teams, reducing funding intermediaries and ensuring on‐time disbursements, and strengthening technical assistance for data use and advocacy.

**Conclusions: **With the rapid expansion of CLM, this study serves as a practical guide for CLM implementers, donors, and technical assistance providers.Successful implementation of CLM requires prioritizing community ownership and leadership, donor commitment to sustainable and reliable funding, and strengthened support of projects across the data collection and advocacy lifecycle.

### EPLBE08 A modified quality improvement approach yields high TB case‐finding rates in newly diagnosed HIV‐positive patients in a district health setting in KwaZulu‐Natal province, South Africa

M.F. Tshabalala ^1^, M. Youngleson^1^, J. Ngozo^2^, P.N. Zulu^1^, N. Kamoga^1^, Z. Linda^1^, S.‐s. Tettey^3^, P. Barker^4^

^1^Institute for Healthcare Improvement, Pietermaritzburg, South Africa, ^2^KwaZulu‐Natal Provincial Department of Health, Pietermaritzburg, South Africa, ^3^Institute for Healthcare Improvement, Accra, Ghana, ^4^Institute for Healthcare Improvement, Boston, United States

**Background: **Covid‐19 adversely affected TB case finding in South Africa from 2020. A TB case finding quality improvement (QI) project aimed to rapidly restore TB case finding to pre‐Covid‐19 levels in five districts in KwaZulu‐Natal province (September 2020 ‐ June 2022) through Targeted Universal TB Testing (TUTT) in three HIV‐positive facility groups (HIV positive pregnant women at 1^st^ antenatal visit, HIV newly diagnosed clients and ART patients at their annual viral load (VL) visit). In addition, two sputum samples were taken simultaneously to increase secondary sputum culture for HIV‐positive GeneXpert negative clients.

**Description: **A modified QI approach was used, including single A4 implementation worksheets to support testing in each of the four groups. Worksheets included a description of the change, names of staff responsible for implementation and measurement, and a data table for recording monthly progress including the number needing investigation, number investigated, and number TB confirmed. Worksheets were introduced over time, updated monthly by facility QI teams, and posted on a district QI WhatsApp group from which the QI project team collated and shared the TB case finding data.

**Lessons learned: **125 facilities participated in the project. Over 12 months (April 2021 – March 2022), 85% of the HIV newly diagnosed clients were tested, with a TB yield of 8.1% (1190/14779) compared with yields of 1.1% (126/11317) and 1.4% (555/39762) in the ANC and VL visit groups respectively. An additional yield of 1.6% (322/20014) was obtained with culture. Culture data was not disaggregated by the HIV‐positive group so the specific culture yield for the newly diagnosed HIV group is unknown. However, assuming an equivalent yield across all groups, an overall TB yield of 9.7% may be achievable in HIV newly diagnosed patients in a district setting (range 14.3% ‐ 6.2% between the five districts).

**Conclusions/Next steps: **While all patients diagnosed with TB are required to have an HIV test, the standard of care currently requires that only TB symptomatic HIV newly diagnosed clients are tested for TB. These data suggest TUTT and “double‐sputum” sampling for this group, supported by a simplified QI approach, is an effective way to increase TB case finding and improve HIV/TB integration.

### EPLBE09 Impact of SARS‐CoV‐2 pandemic on routine HIV care and treatment outcomes in Kenya: a nationally representative analysis

D. Kimanga ^1^, V.N. Makory^2^, A.S. Hassan^3^, F. Ngari^2^, M.N. Mwakala^1^, K.J. Muthoka^4^, L. Odero^5^, G. Omoro^6^, A. Aoko^1^, L. Ng'ang'a^1^

^1^Centers for Disease Control and Prevention, Division of Global HIV and TB (DGHT), Nairobi, Kenya, ^2^Ministry of Health, Kenya, National AIDS and STI Control Program, Nairobi, Kenya, ^3^KEMRI/Wellcome Trust Research Programme, Kilifi, Kenya, ^4^Palladium Group, Nairobi, Kenya, ^5^United States Agency for International Development (USAID), Nairobi, Kenya, ^6^Military HIV Research Program/Walter Reed Army Institute of Research (MHRP/WRAIR), Nairobi, Kenya

**Background: **The COVID‐19 pandemic adversely disrupted global health service delivery. We aimed to assess impact of the pandemic on same‐day HIV diagnosis/ART initiation, six‐months non‐retention and initial virologic non‐suppression (VnS) among individuals starting antiretroviral therapy (ART) in Kenya.

**Methods: **Individual‐level longitudinal service delivery data hosted at a national repository were analysed. Random sampling of individuals aged >15 years starting ART between Apr’18 and Mar’21 was done. Date of ART initiation was stratified into pre‐COVID‐19 (Apr’18–Mar’19 and Apr’19–Mar’20) and COVID‐19 (Apr’20–Mar’21) pandemic periods. End‐points included (i) same‐day HIV diagnosis/ART initiation, (ii) six‐months non‐retention, defined as either dead or lost‐to‐follow‐up (missed scheduled appointments plus three months grace period), and (iii) initial VnS (first viral load test done within 12‐months of ART initiation), defined as HIV RNA>1000 copies/ml. Mixed effects generalised linear, survival and logistic regression models were used to determine the effect of COVID‐19 pandemic on same‐day HIV diagnosis/ART initiation, six‐months non‐retention and initial VnS respectively.

**Results: **Of the 7,046 individuals sampled, 35.0%, 37.1% and 27.9% started ART during Apr’18– Mar’19, Apr’19–Mar’20 and Apr’20–Mar’21, respectively. Compared to the pre‐COVID‐19 period, the COVID‐19 pandemic period had higher same‐day HIV diagnosis/ART initiation (adjusted risk ratio [95% CI], p‐value: 1.4 [1.2–1.6], p<0.001) and lower six‐months non‐retention (adjusted hazard ratio [95% CI], p‐value: 0.7 [0.6–0.8], p<0.001). Of those sampled, 3,174 (45.7%) had a viral load test done at a median 6.2 (IQR, 5.3–7.4) months after ART initiation. Compared to the pre‐COVID‐19 period, there was no significant difference in initial VnS during the COVID‐19 pandemic period (adjusted odds ratio [95% CI], p‐value: 0.8 [95% CI: 0.5–1.2], p=0.238).

**Conclusions: **Impact of the COVID‐19 pandemic on HIV care and treatment outcomes has been less adverse in Kenya. Timely, strategic and sustained COVID‐19 response may have played a critical role in mitigating adverse effects of the pandemic and point towards maturity, versatility and resilience of the HIV program in Kenya. Continued monitoring to assess long‐term impact of the COVID‐19 pandemic on HIV care and treatment program in Kenya is warranted.

### EPLBE10 Expanding viral load coverage in the pursuit of HIV epidemic control in Lualaba province in the Democratic Republic of Congo

B. KABONGO ^1^, G. Fatti^2^, R. Mohammed^2^, J.M. Kanyinda^1^, B.M. Banzua^1^, A. Grimwood^2^

^1^Kheth'impilo‐DRC, Kolwezi, Congo, Democratic Republic of the, ^2^Kheth'impilo, Cape Town, South Africa

**Background: **Lualaba, DRC's copper and cobalt‐mining hub, with industrial mining attracting regional business people and a vibrant artisanal mining trade of “entrepreneurs” of small and medium industries leading to in‐migration from rural areas and neighboring countries including truckers with associated CSW and SUD.

Viral Load testing (VL) and coverage are important indicators of HIV epidemic control. Coverage and suppression of VL are long‐standing challenges in Lualaba. A long‐term turnaround strategy was implemented using a threefold approach: pre‐lab demand generation, intra‐lab swift sample analysis and post‐lab electronic results delivery system, with optimal patient support for VL suppression.

Kheth'Impilo‐DRC (KI‐DRC) is a local Non‐Governmental Organisation supporting 77 sites in Lualaba in 14 Health Zones (HZs) with a current cohort of 26452 patients in care.

**Methods: **Demand for VL testing was increased through a micro‐management approach that included identifying eligible patients in supported sites, improving support to providers with communication credits for phone calls in following up patients for VL collection appointments, driving community VL sample collection while increasing the frequency of collection between communities, facilities and main laboratory Hubs. Additional personnel was extended to the laboratory to support internal processes and shorten result turnover time through electronic results delivery while individual IEC support was reinforced to patients with unsuppressed VL.

**Results: **This strategy resulted in an increase of VL uptake from 53% of an active cohort of 19922 in Mar2021 to 99% of an active cohort of 25017 in Dec2021. The viral suppression rate increased from 86% to 95% over the same period. Progress in VL suppression rate registered accross all age groups and gender.

**Figure:**
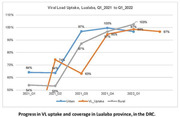
**Abstract EPLBE10‐Figure 1**.

**Conclusions: **Community VL collection combined with a singularised reinforcing approach to adherence of each patient with individualized follow‐up by peer educators of poorly adherent patients are key to improving VL coverage and suppression in pursuit of HIV epidemic control.

### EPLBF01 Community‐led humanitarian and HIV/TB interventions in the emergency response to the war in Ukraine

V. Rachynska ^1^, D. Sherembei^1^, A. Iovita^2^, D. Lohman^2^

^1^All‐Ukrainian Network of PLWH, Kyiv, Ukraine, ^2^The Global Fund to Fight AIDS, Tuberculosis and Malaria, Geneva, Switzerland

**Background: **Community‐led organizations in Ukraine play a significant role in the country's response to HIV and TB through provision of prevention services, linkages to ARV and opioid substitution treatment, and persistent efforts to remove human rights barriers such as stigma and discrimination that impede communities’ access to services. Russia's February 24^th^ invasion of Ukraine dramatically changed the operating environment for these organizations as conflict disrupted normal operations in large parts of the country and sent millions of Ukrainians fleeing for their lives.

**Description: **Community‐led organizations quickly adapted their operations to these new conditions. Embracing a “first‐save‐lives” approach, they used their deep connections in communities to evacuate members of key and vulnerable populations in areas of active combat to safety; organized temporary shelter and nutritional support; facilitated access to emergency medical services; and linked them to HIV, TB and drug treatment services in their new locations. These efforts were closely coordinated with and supported by the government, donors and other partners. As a result, key and vulnerable populations have been able to continue to receive HIV and TB services with limited disruptions despite the devastating impact of the conflict.

**Lessons learned: **Our preliminary analysis suggests that a number of key factors facilitated these community‐led organizations’ ability to respond rapidly. First, these groups generally tend to have light, horizontal organizational structures allowing them to quickly adapt to ever changing local circumstances. Their embeddedness in communities allows them to quickly pick up on changing needs. Second, the COVID‐19 pandemic gave community‐led organizations ample practice in adaptability as it forced numerous and substantial change to their operations. It also resulted in innovations such as simplified procedures for ART and substitution treatment, development of security protocols, and psychological interventions that have proven highly relevant during the conflict. Finally, the Global Fund, the main funder of community‐led programs, quickly facilitated the reprogramming of activities allowing organizations to meet the evolving needs of their communities.

**Conclusions/Next steps: **Ukraine's experience may hold broader lessons for challenging operating environments. Community organizations can be an important resource in adapting the response to HIV and TB during armed conflicts and other major disruptive events.

### EPLBF02 The Venezuelan migration phenomenon and the response to HIV and TB in Colombia, Ecuador and Peru

A. Mejia ^1^, A. Luna^2^, S. Jaramillo^3^, M.A. Torres^3^

^1^Vía Libre, LAC Platform, Lima, Peru, ^2^Via Libre, LAC Platform, Lima, Peru, ^3^ICASO, Toronto, Canada

**Background: **Venezuela has faced a humanitarian crisis of significant impact in recent years. As of November 2021, more than6 million migrants and refugees from Venezuela have been forced to leave their country, of which about 5 million reside in Latin America. According to UNAIDS, at least 8,000 Venezuelans living with HIV have left the country to continue their treatment; this number could increase given that it corresponds to 14% of people who require antiretroviral therapy. This study documents the response to HIV and TB by governments, civil society and the Global Fund in Colombia, Ecuador and Peru, countries with the highest number of Venezuelan migrants.

**Methods: **A case study methodology was developed with mixed methods and information from primary and secondary sources. In 2021, the team conducted interviews with key actors in response to HIV/TB and related documents were reviewed, the results of which were systematized and analyzed.

**Results: **Governments have maintained or improved their supportive policies to guarantee access to health services for Venezuelan migrants. International cooperation agencies have a growing interest in directly or indirectly addressing the response to HIV and TB in these countries, including the Inter‐Agency Coordination Platform for Refugees and Migrants from Venezuela (R4V).TheGlobal Fund in Colombia and Peru has plans to include services for migrants. Civil society organizations have contributed significantly to the response, mainly to HIV, they have articulated regionally with each other and with the international cooperation agencies. However, they have limited resources and require strengthening to achieve a more significant impact. The study identified technical assistance needs to improve the response of this sector, as well as barriers to access health services for migrants and gaps in the response.

**Conclusions: **The results allow us to have an overview of the response to HIV and TB in the framework of Venezuelan migration in the countries studied and the challenges that stakeholders have to improve access to services. It describes access barriers, gaps in response, and technical assistance needs for civil society organizations and provides recommendations for action. The results are also helpful in guiding the response of the Global Fund and other stakeholders.

### EPLBF03 A study on association between legal environments, society tolerance, family support and alcoholism, high‐risk substance abuse, HIV risk perception among TGE communities, post decriminalization of IPC section 377, India

V.S. Prasad ^1^, E. Michael^1^, K. Prasanna^1^, L. Honig^2^, T. Ford^2^, M. Wienstein^2^, A.S. Benzaken^3^, J. Vandenhombergh^4^

^1^AIDS Healthcare Foundation India, New Delhi, India, ^2^AIDS Healthcare Foundation, Los Angeles, United States, ^3^AIDS Healthcare Foundation, Sao Paulo, Brazil, ^4^AIDS Healthcare Foundation, Amsterdam, Netherlands, the

**Background: **Gender Based Violence on (TGE) ‐Transgender and Gender Expansive communities continue to be existing and seems to be accepted and perceived as NORMAL among the general population of India. Decriminalization of Same Sex relationships and repeal of section 377 from the Indian Penal Code opened the public discourse and added visibility of the TGE communities in India. The study was intended to explore the association between legal environments, society tolerance, family acceptance and alcoholism, high risk substance abuse, HIV risk perception and Unsafe Sexual behaviors among TGE communities.

**Description: **876 TGE living with HIV and enrolled for Anti‐Retroviral Therapy (ART) from the “THE PEOPLE'S CLINIC” New Delhi, India, were recruited and a questionnaire was shared via social media and responses collected over a period of nine months. Logistic regression was employed to test Associations between Protective environments and high‐risk substance abuse, High risk Sexual behaviors and HIV positivity and Self rated Health outcomes.

**Lessons learned: **Overall, (18%) reported support of their TGE identity by at least one close family member. Due to fear of stigma and discrimination only (3%) revealed HIV positivity status to a close family member. Owners of rental residences and housing societies exhibited most intolerance (85%) in urban areas and refused letting out their premises to TGE communities. Housing instability further pushed communities to indulge in high‐risk substance abuse (36%), alcoholism (63%) leading to High‐risk behaviors including unprotected multi‐partner SEX (89%) and leading to acquiring HIV infections. Even with repeal of punitive homophobic laws ‐ violence, abuse and body shaming was reported especially from law enforces and police (34%).

**Conclusions/Next steps: **Decriminalization of punitive laws against sexual minorities without sensitization of general population and law enforcement agencies including Police, does not necessarily bring Protective environments, Societal acceptance or gender‐based violence. The need for revised National level strategies to break structural barriers and promote protective environments for TGE communities are reflected as major findings of this study. Framing strict laws, in treating violence meted out to TGE communities as a culpable offence, would help create more tolerant and accommodative societies, reduce violence and lead to better health outcomes for the TGE communities.

### EPLBF04 Right to quality HIV care for children in trauma and rescue centers in Kenya

N. Mugao ^1^, C.A. Opinde^2^, P.T. Kadir^1^

^1^Frolics of Hope Africa, Nakuru, Kenya, ^2^The NGO Whisperer™, Manchester, United Kingdom

**Background: **The United Nations‐led Sustainable Development envisioned a future free of fear and violence. Governments and non‐government entities are putting structures that ensure the peaceful coexistence of the communities globally. Kenya is one of the many governments that has policies and mechanisms to curb all forms of violence. However, despite having these policies and guidelines in place, gender‐based violence (GBV) and Human Immune Virus (HIV) prevention services are still offered perpendicularly (Edwards, etal, 2022).

**Description: **The recent challenges of COVID‐19 have compounded this gap. Rescue and Trauma Centers for abused children and women do not have a legal framework to govern their existence and service delivery to this vulnerable group. According to the research, the crossing of HIV and GBV proves that violence against women and children exponentially raises their vulnerability to HIV (Meffert, etal, 2021). The World Health Organization report states that one in five girls and one in ten boys have gone through one or more forms of violence before they are 18 (Bhatia, etal, 2020). Currently, this figure has risen to three in five girls and two in five boys who report to have experienced sexual violence before they are adults (Annor, etal, 2022). Rescue and Trauma Centers such as Frolics of Hope Africa requires capacity building, and resources to effectively rescue, report, rehabilitate, reintegrate, and repatriate victims of GBV and human trafficking violence due to their HIV status.

**Lessons learned: **In Kenya, only about 12 per cent of the 50 Trauma and Rescue Centers have the capacity to refer or serve women and children who have gone through violence and abuse due to their HIV status. Data has shown that Intimate Partner Violence (IPV) raises a woman's chances of HIV infection due to rape by an infected partner, negligible opportunities for compromise on safer sex, and high‐risk adverse tendencies (Ndungu, 2019).

**Conclusions/Next steps: **Integration of HIV and GBV is critical, especially within the safe houses that offer rescue services for GBV survivors. The integration will reduce HIV transmission from mother to child, teen, and youth infection rates and help the survivors access the much‐needed care and support.

### EPLBF05 Post‐test and TB survivor grassroots‐based CBOs leverage HIV/TB care at The Last Mile; a case of 7 expert PWD‐led mobilization HIV prevention groups in Uganda, August 2021‐May 2022

T. Muyunga‐Mukasa ^1,2,3^

^1^Advocacy Network Africa ( AdNetA), Strategic Public Health Advocacy, Los Angeles, United States, ^2^Joyful Hope Resource Platform Center (JHRPC), Livelihood and Entrepreneurship Among the Youth, Nairobi, Kenya, ^3^Most At Risk Populations' Society in Uganda (MARPS In Uganda), Strategic Public Health Advocacy, Kampala, Uganda

**Background: **People living with HIV, key populations, and people at risk of HIV grasp such concepts like Public‐Private Mix (PPM), Communities of Practice (CoP), Integrated Service Delivery (ISD), human rights, equality, and dignity, action against harmful practices, stigma, and discrimination when there is deliberate, strategic action to take them through participatory training sessions. This study followed groups from August 2021 to May 2022 and it aimed at gauging TB/HIV‐related Prevention campaigns by CBOs which were formed as PWD‐led Post‐Test and TB Survivor Support Clubs in Uganda.

**Description: **A 9 month cross‐sectional study, using contacts to regularly check compliance to strategic plans, updates of record books, checklists and minutes to track community outreach events promoting prevention.

**Lessons learned: **The report indicates that the CBOs connected beneficiaries to HIV testing, access to treatment, promoting care, referrals for non‐communicable diseases care, attending clinic checkups for viral suppression and uptake of integrated services including addressing vertical HIV transmission, SRH services, dental and ear, Family planning, spacing cancer screening, paediatric AIDS and other services. It is clear that the 7 CBOs were the constant reminders and motivation to adherence during the COVID‐19 lockdown and during the easing for 784 Persons Living with HIV and 54 Living with TB. Through constant visibility and mobilization, they maintained a food supply‐chain, checked in on each other and mobilized money which sustained 48 who would have faced housing instability and food insecurity.

**Conclusions/Next steps: **Grassroots‐based systems can be leveraged as health and social protection continuum spaces. This in turn supports wellness, livelihood, and enabling environments for people living with, at risk of, or affected by HIV to reduce inequalities and allow them to live and thrive.

### EPLBF06 Provision of sexual and reproductive health services to adolescents and young people with disabilities living with HIV (AYPWDSLWHIV): observations from Chipinge district in Zimbabwe

C. Ndanga ^1^

^1^REPSSI, Programmes, Harare, Zimbabwe

**Background: **Despite the existence of the eloquent and sound United Nations Convention on the Rights of People with Disabilities (UNCRPWD) (2008) which articulates on inclusion people with disabilities in all development initiatives and improvement on their access to key welfare resources, it remains disheartening to pick the provision of sexual and reproductive health services (SRHS) to AYPWDLWHIV in a piecemeal worrisome state. Cultural connotations which place a taboo labelling on the provision of SRHS to AYPWDS remains the most outstanding barrier among other key issues observed.

**Description: **Inclusion of AYPWDLWHIV in discourses and platforms spearheading provision of SRHS in Chipinge district, Zimbabwe is yet to be implemented. Programming and discussion on the SRHR needs of AYPWDS remains absent in the district. The community holds negative attitude marked with some taboo connotations towards the provision of SRHS to AYPWDLWHIV. Shocking and disturbing from the human personnel of expected high integrity and ethical conduct, health workers at clinics in the district frown at AYPWDLWHIV when they seek condoms and other critical information regarding SRHR. As if that is not enough for the plight of AYPWDS, there are no evident efforts to improve their conducive physical access to SRH services.

**Lessons learned: **According to district data, over 45% of sexual harassment cases recorded in marriages and other sexual relations in the district involves AYPWDLWHIV as they lack empowerment on their sexual and reproductive health. It is against this background compounded by other factors already observed, that a lot of AYPWDLWHIV are engaging in unprotected sex which is leading to unintended pregnancies, STIs, and HIV spreading.Unplanned children and poor health are exacerbating their entrenchment in the circle of poverty

**Conclusions/Next steps: **There is need for communities to come up with a home‐grown local policy which fosters inclusion of AYPWDLWHIW in all discourses pertaining their SRH needs and also unpacking the UNCRPD article 25 to the communities. It is also important that public educational interventions are designed and implemented to demystify prevailing beliefs and practices, improve public understanding of the sexual and reproductive health needs of AYPWDLWHIV and ways the public could support AYPWDLWHIV to lead safe and satisfying sexual lives.

### EPLBF07 Advancing HIV prevention research in pregnant and breastfeeding populations (PBFP): priority advocacy objectives and next steps

S. Kristen ^1^, M. Chatani^2^, B. Lesnar^3^, D. Nhamo^4^, A.D. Lyerly^1^

^1^University of North Carolina at Chapel Hill, Center for Bioethics, Department of Social Medicine, Chapel Hill, United States, ^2^AVAC, New York, United States, ^3^AVAC, Grand Rapids, United States, ^4^Pangaea Zimbabwe AIDS Trust (PZAT), Harare, Zimbabwe

**Background: **Growing consensus around the ethical and public health imperative for the responsible inclusion of PBFP in HIV research has gained critical momentum. Milestones include Ethics Guidance from the PHASES Working Group and the WHO/IMPAACT Call to Action to accelerate the study of new HIV drugs for pregnant and breastfeeding populations (PBFP), though much work to realize this vision remains. To this end, AVAC, supported by the Coalition to Accelerate and Support Prevention Research (CASPR), and the PHASES Project convened a think tank in April 2022 to identify priority advocacy objectives, informed by consensus recommendations, and develop an action plan to accelerate HIV prevention research with PBFP.

**Description: **Significant stakeholder engagement guided the development of the think tank, in alignment with the Good Participatory Practice Guidelines. This included consultations with researchers and a pre‐meeting, co‐convened with Pangaea Zimbabwe Aids Trust, with advocates and former trial participants. Sixty‐three global stakeholders, including researchers, industry representatives, trial participants, civil society, regulators, and funders attended a half‐day virtual workshop which utilized facilitated planning sessions to identify advocacy and action priorities. Think Tank members provided input on a report and action plan and were further engaged for additional information.

**Lessons learned: **Priority action areas included the need to: strengthen advocates’ knowledge about the inclusion of PBFP in research; develop guidance specific to engaging stakeholders in the design and conduct of trials with PBFP, with particular attention to centering PBFP throughout; frame the responsible inclusion of PBFP in research as a reproductive justice issue and align with a broader coalition of advocates and allies; and support the advancement of regulatory authority to require data on PBFP. Additional priorities raised included development of resources to support ethics review boards to consider responsible inclusion of PBFP; integration of the experiences and needs of adolescents and trans and gender‐diverse PBFP in HIV prevention research; harmonization across regulatory agencies, and consistent metrics for trial outcomes.

**Conclusions/Next steps: **Assessing and implementing this action plan is ongoing. Key accomplishments to date include funder commitment to harmonize outcome measures and AVAC commitment to the development of an advocacy toolkit, and training resources. Further status updates will be provided.

### EPLBF08 The Brazilian response to HIV/AIDS & COVID‐19: double standards and rights violations

M. Malta ^1,2^

^1^Centre for Addiction and Mental Health, Institute for Mental Health Policy Research, Toronto, Canada, ^2^University of Toronto, Department of Psychiatry, Toronto, Canada

**Background: **The Brazilian response to HIV/AIDS is frequent cited as best practice. Brazil threatened to break patents, issued a compulsory license for Efavirenz and negotiated large discounts to acquire antiretroviral therapy (ART). These strategies allowed free/universal access to ART for all people living with HIV/AIDS (PLWHA) in Brazil since mid 90s’. The country was also pioneer in offering HIV self‐testing, HIV pre‐ and post‐exposure prophylaxis (PrEP/PEP). Why all this expertise was not adapted during COVID‐19 pandemic?

**Description: **The COVID‐19 pandemic exposed Brazil to an unprecedented health, social and economic challenge. The country had the third‐highest death toll in the world: over 660,000 COVID‐related deaths recorded. During COVID‐19 wave peaks, hospitals experienced shortages of oxygen and sedatives to intubate patients, ICU and hospitals were working at capacity. In spite of a well stablished immunization program, the country struggled with COVID‐19 vaccine rollout.

**Lessons learned: **Brazilian HIV/AIDS response was guided by scientific and public health evidence, developed in close collaboration with civil society and PLWHA. Policy makers, government, researchers and affected communities worked together to assure that strategies were adequate, feasible and responsive. Open dialogue was key to review problems and address concerns. Conversely, Brazilian COVID‐19 response highlighted a completely lack of dialogue with the federal government. The absence of a centrally coordinated strategy and misalignment between federal and local governments regarding COVID‐19 response, together with Brazilian president denial of scientific evidence, promotion of ineffective treatments and insufficient vaccination efforts, have led to uncontrolled spread of COVID‐19, the near‐total collapse of the health system and excess deaths. Overwhelmed by COVID‐19, health services reduced their availability of PrEP and PEP, while PLWHIA experienced difficulties in receiving their ART and scheduling follow up exams/appointments. During president Bolsonaro's ultra conservative government, several HIV prevention materials and campaigns were also censored.

**Conclusions/Next steps: **Decades of effective response to HIV/AIDS have demonstrated the essential relationship between human rights and public health. Countries should build their pandemic responses rooted in respecting science, public health and human rights principles, while hearing and working with populations most at risk. During the COVID‐19 pandemic, the current Brazilian president choose to do the opposite, with tragic consequences

## AUTHOR INDEX

### A

A bu dou re xi ti, G. OALBC0103

Abad, A. PEMOA32

Abah, S. PESAD23

Abass, M. PESAE11

Abate, A. OALBX0102

Abbah, S. OAF0405

Abbasi, S. PEMOD64

Abbott, R. OAB0403

Abdalian, S.E. PESAB01

Abdel‐Mohsen, M. OALBA0105

Abdool Karim, Q. PEMOD62, PESUE31

Abdullaev, T. OALBF0105

Abdullahi, R.M. PEMOD72

Abera, G. OAC0102

Aberle‐Grasse, J. PESAE08

Abimiku, A. PELBC01

Ablona, A. PEMOB34

Abrams, E.J. OAD0705, PEMOD60

Abu‐Laban, M. OALBX0102

Abubakar, A. EPLBB03

Abubakari, G.M. PESAD04

Abudiore, O. PESAB04

Abuna, F. OAC0502

Abuogi, L. PESUA19

Aceiton Cardona, J. OAC0402

Acheampong, N.A. PEMOD54, PESUD27

Ackerman, M. PELBA01

Adams, D. OAC0403

Adamu‐Oyegun, S. PEMOC39

Adeagbo, O. PESUD46

Adedimeji, A. PESUD28

Ademola‐Kay, T.E. OAE0103

Adepoju, V.A. OAE0103

Adesigbin, C. PESAB04

Adetosoye, A. OAE0103

Adeyeye, A. EPLBC04, OALBX0107

adhikary, r. EPLBC01

Adiibokah, E. PESUE24

Adimora, A.A. PESAB13

Adjei, B. OAF0405

Adler, M. PEMOC42

Adobea Asante, C. PESUD27

Adongo, C. OAD0603

Afriyie, R. PEMOD54, PESUD27

Agaba, P. PESAE08, PESUE20

Agada, E. PEMOB30

Agafitei, O. PEMOA38

Agbaji, O. PESAB04

Agbakwuru, C. PELBC01

Agbom, U. PESAD23

Agengo, G. OALBE0102

Agot, K. EPLBC03, PESAC05

Aguiar, C. EPLBE07

Aguilera, M. PEMOD67

Agwu, A. PESUA19

Agyei, Y. OALBX0107

Ahalawat, A. OAB0103, PESUD34

Ahlenback, V. OAF0305

Ahmad, A. PESUF15

Ahmad, F. PESAD03

Ahmadi‐Erber, S. OALBA0103

Ahmed, S. OAC0102

Ahsan, A. PEMOA36

Ahuja, D. PESUF16

Ait‐Ammar, A. PESUA17

Ait‐Khaled, M. EPLBB05, PESUB21

Aitchison, J. PEMOB32

Ajeh, R. OAE0502

Ajijelek, A. OAE0103

Ajonye, B. EPLBE07, PEMOD58

Ajulong, C. EPLBE01

Akanbi, S. PEMOB30

Akanmu, M.‐M. PESAB04

Akanmu, S. PESAB04

Akasreku, B. PESAC14

Ake, J. PEMOB27

Akinmade, M. PESAD09

Akkakanjanasupar, P. PELBD01

Ako‐Arrey, D. PESAD17

Akolkar, K. EPLBA03

Akoth, S. PEMOD60

Akpomiemie, G. PELBB01

Akugizibwe, P. EPLBC10

Akullo, J. OAE0102

Akullu, L. PEMOD59

Al Hinai, A.S. PESUB18

Al Malki, M. OALBB0104

Alagi, M. OAC0102

Alain, T. OAD0404

Alamo, S. EPLBC08

Alanio, A. PESAB06

Alba, C. OAE0303

Alcamí, J. OAA0205, PESAA13, PESUA23

Alemnji, G. PESUE20

Alfranca, A. PEMOA27

Algarin, A. OAD0304

Alger, S. PESAE10

Ali, H. PEMOA38

Ali, M. OAD0703

Alintah, O. OALBE0105

Aliu, A. PESAD23

Allan, B. PESUB25

Allen, J. PESAD04

Allen, M. EPLBA02

Althoff, K. OAE0502

Althoff, K.N. OAC0403, PESAB02

Aluh, D. PESAD23

Alvarnas, J. OALBB0104

Alves, C.R.B. PESAC02

Alwano, M.G. PEMOC42

Amamilo, I. PESAB04

Aman, K. PESAB08

Amankwa, B. PESUD27

Amanya, J. PEMOD59

Amatsombat, T. OAC0503

Ambrosioni, J. OAA0205

Ameli, V. PEMOD57

Amick, A.K. PESUE27

Amole, C. PESAB04, PESUE30

Amon, J. OAF0103, PESUF23

Amos, N. PESUC24

Amuge, P. OAB0203

Amukele, T. OAE0102

Amutuhaire, H. OAD0805, PEMOD59

An, Z. PEMOE43

Anabwani, F. OAB0202

Anand, S. PEMOC41

Anand, S.P. OAA0405

Anand, V. PEMOD64

Ananworanich, J. OAA0105, OAA0302

Anastasie, M. PESAE11

Anastos, K. PESAB13, PESUB15

Ancuta, P. OAA0103, OAA0202, PESAA07

Anderegg, N. OAC0304, PESUB17

Anderle, R.V.R. OAE0302

Andersen‐Nissen, E. EPLBA02, OALBA0102

Andersen, A. OALBD0104

Anderson, F. EPLBA08

Anderson, P.L. PESAB03

Anderson, R. PESUC18

Andifasi, P. PEMOD55

Ando, N. PESAB10

Andom, A.T. OAE0203

Andrew, P. EPLBB08, PESUA14

Andriesen, J. OALBC0102

Aneja, K. PESAF10

Angelovich, T. OAA0304, PESUA24

Angira, F. OALBX0107

Anib, V. EPLBD08

Anis, A. EPLBC09

Anis, A.H. OALBD0102, PEMOA38

Annamanthadoo, I. PESAF01, PESAF02, PESUF22

Anne Bukusi, E. PESAE02

Anok, A. PESAE16

Anoubissi, J.d.D. PESAE11

Anyasi, H. OAE0103

Anzala, O. PESAD06

Aoki, T. PESAB10

Aoko, A. EPLBE09

Aparicio‐Gómez, M. PESUA23

Aparicio‐Herguedas, M. PESUA23

Apedaile, D. PESAC04

Apffel Font, O. EPLBD07, PEMOD71

Apok, O. PESAD17

Arana, J.C. PESAD05

Aranguren, M. PESAA09

Arasa, J. EPLBC03

Archin, N.M. OAA0404

Argaw, S. PEMOE40

Arias García, S. PESUC18

Aribi, A. OALBB0104

Arije, O. PEMOD62, PEMOD72, PESUE31

Arinaitwe, I. OAD0805

Aristegui, I. EPLBD07, PEMOB35, PEMOD67

Ark, B. PESAB01

Armon, C. PEMOC48

Armstrong, D. PESAB08

Arons, M. OAC0102

Arora, A. PESAD14

Arroyo‐Flores, J. PEMOD75

Arthur G, F. EPLBE01

Arthur, M. OAD0803

Arumugam, V. PESAE05

Asalou, G. OAC0102

Asamoah Adu, C. PEMOD61

Asewe, M. PESAE02

Asfaw, M. OAE0203

Ashaba, S. OAD0805

Asowata, O.E. EPLBA08

Assoumou, L. EPLBB04

Atagher, D. PEMOC39

Athokpam, L. OAF0202

Atif Zahoor, M. PESAA05

Atuahene, D.K. PESUD27

Atuahene, K. OAF0405

Atujuna, M. PEMOC37

Atukunda, M. OAB0403

Atuma, E. OAE0103

Atwine, D. OAD0105

Atwoli, L. PESAC04

Atwongyeire, D. OAD0805

Audi, C. PESAF07

Auld, A. OAC0305

Auld, A.F. OAC0405

Aungkulanon, S. OAC0103, PESUC22

Avani, N. PESUF21

Avendaño‐Solá, C. PESUA23

Averilla, M.L. PESUE29

Avery, M. OAC0503, PESUC32

Avettand‐Fenoel, V. PESUA17

Avihingsanon, A. OALBX0105, PESAB09

Awor, A.C. PEMOC42

Ayamah, P. OAF0405, PESUD27

Ayebare, F. OAE0503

Ayeh, E. PESUF17

Ayieko, J. OAB0403, OALBE0102

Ayles, H. OAC0104, PEMOC36

Ayuku, D. PESAC04

Azangtsop, L. PEMOF29

Azarskova, M. EPLBC02, PELBE01

Azevedo, P. OAB0305

Azizi, R. OALBF0104

Azmin, R. PEMOB34

### B

Babaye, Y. OAC0405

Babirye, B. PESAE10

Babu Raj, M. PEMOD70

Babu, H. OAD0305

Bacha, J. OAB0202, OAB0203

Bachanas, P. PESAE08

Badia, J. PESAC05

Baeten, J. OAC0504, OALBX0105

Baeten, J.M. OAE0105, PEMOE41, PESAE02

Bagapi, K. PELBC01

Bagasol, E. PESUE29

Bagul, R. EPLBA03

BaharuddinNur, R.J. OALBC0104

Bahemana, E. PEMOB27

Baidoobonso, S. PEMOF29

Bailey, L. PESAE08, PESUE20

Baipluthong, B. PESUC22

Baker, M. PEMOB29, PESUB24

Bal, H. EPLBA03

Balazs, A. PELBA01

Balkus, J. EPLBC05

Ballesteros, A. EPLBB06

Ballif, M. OAC0304

Baltrusaitis, K. OAB0104

Balzer, L. OAB0403, OALBE0102

Bancroft, E. PEMOE45

Banda, B.A. PESAE09

Banda, U. PEMOC44

Banerjee, S. PESAD07, PESUD42, PESUD47

Banougnin, B. OAD0702

Banzua, B.M. EPLBE10

Bar, K. EPLBB08

Bara, H. PESAD22

Baral, R. PEMOE44

Baral, S. EPLBE02, OAD0504, OALBD0105, PEMOB33, PEMOD63, PEMOF28, PESAD09, PESUC18

Barbeau, B. EPLBA05

Barbee, L. PESAC07

Barber, T.J. PEMOA33

Bardon, A.R. OAE0105

Barkatdaoud, C. OALBF0104

Barker, P. EPLBE08

Barlow, J. PEMOD57

Barnabee, G. PEMOC44

Barnable, P. PESAA08

Barnhart, M. OAE0303

Barnighausen, T. PEMOE36

Barouch, D.H. OALBA0102

Barreto, M.L. OAE0302

Barrios, R. PEMOA38

Barroso Pacca, J. PEMOE37

Baruah, D. PEMOD69, PESUD45

Baruch‐Dominguez, R. OAD0304

Barzilay, E.J. EPLBC02, PELBE01

Baskota, R. PEMOE44

Bassetti, M. PESUB19

Bateganya, M. PESAE11

Baton, V.P. PESUE29

Batrouney, C. PESUE28

Baudi, I. OAC0104

Bautista‐Arredondo, S. PEMOE39, PESAE12

Bavinton, B. PESUC24, PESUC25

Bay, M. OAB0305

Bearden, A. PESUA19

Beattie, T. OAD0305

Beauchemin Nadeau, M.‐E. PEMOB36

Beaumont, S. OAF0402

Beeman, A. PESAD08

Behel, S. OAC0102

Behera, T. OAF0202

Beigel‐Orme, S. OALBX0107

Bekker, L.‐G. EPLBA02, OALBC0102, PEMOC37

Belayneh, M. OAE0504

Bell‐Mandla, N. PEMOC36

Bell, B. PESAA01

Bell, E. OAF0305

Bell, J. PESAE05

Bello, G. OAC0305, OAC0405

Ben Moussa, A. EPLBD07

Bendayan, R. PELBA03

Bendoumou, M. PESUA17

Benedetti, M. PESAE12

Beneri, C. OAB0104

Benhabbour, S.R. PEMOA29

Benitez, G. OALBD0104

Benjamin Mwakyosi, L. OAF0305

Benjapornpong, K. PESAA10

Benn, P. PESUB24

Benzaken, A.S. EPLBF03, PEMOF34

Bergam, S. PESUD37

Berhanu, R. OALBX0107

Berkhout, B. PESUA17

Bermejo, M. PESUA23

Bernadine, N. OAF0102

Bernard‐Henry, C. PEMOA37

Bernard, C. OAE0502

Bernard, E. OAF0402

Bernard, K. OAA0305

Bernasconi, E. OAB0302

Bernstein, M. PEMOA31

Berr, C. PESUB14

Berruti, M. PESUB19

Berry, C. OAB0402

Berry, S.A. PESAB02

Bertagnolio, S. OAB0404

Besong, M. PEMOA34

Best, B. PEMOB29

Beukes, A. OAC0102

Beyrer, C. OAC0403, PESUC29

Bhagani, S. PEMOA33

Bhattacharya, D. OAB0405

Bhebhe, L. PEMOB32

Bhengu, N. PESUD37

Bickel, M. PESAC11

Biedron, C. PEMOE34, PESAE08

Bielick, C. PESAC10

Bigoga, J. PEMOA34

Billong, S. PESAE11

Biribawa, C. PEMOC42

Bitnun, A. OAC0204

Bizet, M. PESUA17

Black, D. OALBE0102

Blanchette, C. EPLBC04

Blandford, J. PEMOE34

Blatz, S. PEMOE47

Blyum, A. PESAD24

Bock, P. EPLBB07, OALBX0107, PEMOC36

Boden, D. OAA0402

Bodika, S. PELBC01

Bodzo, P. PEMOE36

Boesecke, C. PESAC11

Bogers, S. PESAE04

Bohac, C. OAA0403

Boily, M.‐C. OAD0505, PESAC09, PESUD36

Bokhizzou, N. PESAE04

Bolan, R. PESAB01

Bolo, A. EPLBD08

Bolshakova, M. PESUD50

Bolton Moore, C. PEMOB29

Bolton, C. OALBC0105

Bolton, D. PESAA10

Bonacci, R. OAE0404

Bond, V. PEMOC36

Bonell, C. PEMOC43

Bonierbale, M. PESAD13

Bonono, L. PESAE11

Bonsall, D. OAC0104

Bontempo, G. EPLBB05, PESUB21

Boopathy, A. OALBA0103

Booth, J. PESAE01

Boothe, M. PESUC18

Bor, J. PESAE06, PESUD40

Bora, M. PELBD02

Borducchi, E. OALBA0102

Boritz, E. OALBX0102

Borja, J.C. PESAD05

Borquez, A. PESAC08

Borse, N.N. OALBE0104

Bosch, B. PELBB01

Bosch, R. PESAA12

Bose, D.L. PEMOD69

Bosire, R. EPLBC03, PESAC06

Bosomprah, S. OALBC0105

Bosque, A. OAA0102, PESUA21

Both, R. OAD0202

Bouchat, S. PESUA17

Boucicault, E. OAF0305

Boucoiran, I. OAB0105, OAC0204

Bourassa, C. OAA0405

Bourhaba, O. EPLBD07

Bourne, A. PESUC24, PESUC30

Boutin, M. OAA0405

Boutros, C. PELBA01

Boyd, A. PEMOC35, PESAC12, PESAE04

Boyd, D. PESAD04

Boyd, M. PEMOC42

Boyer‐Legault, G. PEMOB36

Boyle, C. PESUB24

Bréhin, C. OAB0205

Braccio, C. OAC0305

Brachel, M. OAD0103

Bradley, J. OAD0305

Brady, J. PELBA01

Braitstein, P. PESAC04

Branca, M. OAB0302

Branger, J. PESAE04

Brar, S.K. PEMOD56

Brassard, N. PESAA09

Braun, D.L. OAB0302

Bravo, C.A. PESUB15

Bravo, K. PESUF13

Bremer, V. PESAC11

Brennan‐Ing, M. PEMOC45

Brennan, D. OAD0704

Brenner, B. OAC0105

Brentari, C. OAF0304

Brew, B. OAA0304, PESUA24

Briedenhann, E. PEMOC34

Brimhall, D.B. PESUB24

Brinkman, K. PESAE04

Brion, S. PEMOF28

Brisson, M. PESAC09

Brites, C. OAB0305

Briz, V. PESAA03

Brochado‐Kith, Ó. PESAA03

Brockman, M.A. PEMOA38

Brockmeyer, N.H. PESAC11

Brookins, K. OALBD0104

Brophy, J. OAC0204

Brostrom, M. OAE0202

Brough, G. PESUB25

Brown O'Sullivan, L. EPLBD05

Brown, A. OAC0302

Brown, A.L. PEMOC45

Brown, B. OAD0403

Brown, C. OALBX0103

Brown, J.R.G. PEMOA33

Brown, K. OAC0305

Brown, L. OAB0104

Bruel, T. PEMOA35

Bruguera, A. OAC0402

Brumme, C.J. PEMOA38

Brumme, Z.L. PEMOA38, PESUA20

Brumskine, W. EPLBA02

Bruneau, J. PEMOB36

Bryant, H. PESUE17

Bryson, M. PEMOD74

Bryson, Y. PESAB01

Bubb‐Humfryes, O. OAE0403

Bucha, M. OAC0503

Buchacz, K. PEMOC48

Buchbinder, S. OALBA0102, OALBX0103

Buchholz, C. PEMOD59

Buck, W.C. EPLBB06

Buckner, N. PELBD02

Buisson, S. OAB0104

Bukusi, D. PESAC06

Bukusi, E. OALBE0102

Bunga, S. EPLBD08

Bunge, K. EPLBC05

Bunis, D. OALBX0102

Burchell, A. EPLBC09, OALBD0102

Burchell, A.N. PEMOB34

Burger, D.M. EPLBB06

Burgess, D. EPLBA04

Burke, R. OALBB0103

Burnham, R. OALBA0102

Burnie, J. PESAA02

Burns, F. PEMOA33

Burns, F.M. PEMOC43

Burns, L. PEMOA38

Burns, P. EPLBD02

Burrone, E. OAE0403

Bush, C. PEMOC33

Businge, J. PESAE10

Busman‐Sahay, K. PESUA24

Butchart, A. PEMOC47

Buule, P. PESUA20

Buzón, M.J. PEMOA27

Buzaalirwa, D.L. PEMOB30

Buzzi, M. OAB0302

Bvumbwe, M. OAB0202

Bwale, C. PESUC26

Bwalya, J. PEMOC36

Bwembelo, B. PESUC26

Bwembya, K.P.M. PESAD19

Byrnes, S. PESUA24

Byrns, M. PESAA09

### C

Cáceres, C.F. PEMOD71

Cabrera, N. PEMOB35, PESUD50

Caceres, C.F. PESAC08, PESAE12

Caesar Arkangelo, G. EPLBD08

Cahn, P. OAB0303

Cahn, P.E. PESUB21

Cai, Y. OALBA0105

Caldeira, D.B. PESAC02

Caldwell, J. EPLBC06

Calmy, A. OAB0302

Calonge, E. PESAA13

Calvet‐Mirabent, M. PEMOA27

Calvo, G.M. PESAC08

Calzada, M.J. PEMOA27

Camacho‐González, A. PEMOD51

Cambria, E. PESUD42, PESUD47

Cameron, D.W. PEMOB32

Camlin, C. OALBE0102

Camp, D. OAD0703

Campbell, A.R. EPLBB02

Campi‐Portaluppi, J. PESUF13

Canagasabey, D. OAC0505

Canton, J. PEMOA28

Cao Duc, P. OAE0505

Cao, W. EPLBB09

Capparelli, E. OALBB0102, PEMOB29

Card, K. PESAE13

Cardoso, A. OALBB0104

Cardoso, A.S. EPLBE05

Cardozo, N. EPLBC04, PEMOD67

Carfi, A. OAA0204

Carl, G. PELBD01

Carlisle, N. PESAE01

Carlson, K. PEMOC48

Carpino, T. OALBD0105

Carpp, L.N. EPLBA02

Carr, B. OALBA0103

Carrico, A. PEMOD75

Carter, C. PEMOC33

Casabona, J. OAC0402

Casadellà, M. OAA0205

Casado‐Fernandez, G. PEMOA28

Casado, G. PESUA25

Casado, J.L. PEMOA32

Cascajero, A. PESUA23

Caseiro, M.M. PESAC02

Caskey, M. OAA0305

Cassamo Omar Abdula, A. OAD0405

Cassim, N. PESUC19

Castellanos, E. OAF0302

Castillo‐de la Osa, M. PESUA23

Castro Avila, J. EPLBD07, PEMOD67, PEMOD71

Castro‐Álvarez, J.M. PESAA03

Caswell, G. PESUD33, PESUF17

Cattle, J. PEMOE34

Cavanaugh, J. PEMOB27

Cavassini, M. OAB0302

Cecchini, D. OAB0303

Cele, S. PEMOA31

Celum, C. OAC0504, OALBX0103

Cenderello, G. PESUB19

Cepeda, J. PESAD24

Cermakian, N. OAA0103

Cerveau, T. EPLBD07, PEMOD73

Cervero, M. PEMOA28

Cesar Ribeiro Campos, W. EPLBC07

Cesar, C. PEMOB35

Ceschel, M. OAB0303

Cezar, R. PESUB14

Chabala, C. EPLBB06

Chadwick, E. PESUA19

Chagaris, K. PEMOC48

Chagnon‐Choquet, J. PESAA09

Chaguala, L. PEMOC46

Chahroudi, A. EPLBA04, OAA0404, PESUA19

Chaillon, A. OALBB0104

Chaiphosri, P. OAC0103

Chaisson, L. PESAB08

Chaiyahong, P. PESAB09

Chaiyavilaskul, P. PESAB09

Chakhtoura, N. EPLBC05, OAB0405

Chakrapani, V. OAB0103, OAD0203, PELBD01, PESUD34, PESUD45

Chambers, C. EPLBC09, OALBD0102

Chamie, G. OAB0403

Chammard, T.B. PESAB06

CHAMPs‐In‐Action Alliance, T. EPLBD05

Chan, P. PESAA10

Chan, R. PESUD42, PESUD47

Chancham, A. OAC0503

Chandiwana, N. PELBB01

Chandy, S. PEMOB37

Chang, A. OAB0405

Chang, L. EPLBC08

Chang, L.W. PESAE16

Chang, S. OALBB0104

Chapin‐Bardales, J. OAC0303

Chapman, K. OAE0303

Charlebois, E. OAB0403, OALBE0102, OALBX0103

Chartrand‐Lefebvre, C. OAA0202

Charurat, M. PELBC01

Charurat, M.E. PELBC02

Chasekwa, B. PESAB07

Chatani, M. EPLBF07

Chatterjee, D. OAA0103

Chauca, A. PEMOE37

Chaudron, S.E. OAC0104

Chavez, P. EPLBC06

Chavez, P.O. PESUB15

Chege, W. PELBA02, PESUA14

Chemnasiri, T. PESUC29

Chen, J. PESAB03

Chen, T.‐W. PESUC30

Chen, Y. OAC0203

Cheneau, C. PEMOA37

Cheng, W. OAD0303

Cheptoris, C. PESAD17

Chere, M. OAE0203

Cheret, A. EPLBD06

Chernoff, M. OAB0104

Chetane, P. OAE0203

Cheung, P.K. PEMOA38

Cheung, S.Y.A. PEMOB29

Chi, B.H. PELBC02

Chiale, S. PESUE17

Chiboma, I. OAE0104

Chikuba‐McLeod, M.N.J.M.N.S.H.M. PESAD19

Chikwanda, P. OAC0102

Chilengi, R. OALBC0102

Chilyabanyama, O. PEMOC42

Chima, K. PEMOF32

Chimbetete, C. OAC0304, PESUB17

Chimwaza, A. PESUE27

Chingombe, B. PESAD18, PESAD22

Chingono, R.M.S. PESUB16

Chintala, S. PESAE05

Chiosi, J. PESUC21

Chiou, H.‐Y. EPLBD04

Chipeta, S. OAC0405

Chipukuma, J. PESUC26

Chirairo, H. PEMOC44

Chirenje, Z. OALBC0102

Chirwa, L. PEMOB28

Chisenga Chungu, M. PEMOF28

Chitsamatanga, M. EPLBB06

Chiu, Y.‐W. EPLBD04

Chiwala, L. OAC0405

Cho, Y.S. PESAB09

Chohan, B. PESAC06

Choi, S. OALBX0102

Chokephaibulkit, K. OAA0105, OAA0302

Cholico, W. OAD0205

Chollier, M. PESAD13

Chomont, N. OAA0105, OAA0302

Choonara, S. PEMOD62, PESUE31

Chopra, N. PESUD34

Chopra, N.S. OAB0103

Chougar, S. PEMOB36

Chowdhury, E.I. PESUE18

Christ, B. OAC0304

Christensen, C. OAD0403

Chu, I.Y.‐H. PEMOE46

Chu, S.K.H. OAF0103, PESAF03, PESUF23

Chua‐Intra, B. PESUC29

Chukwu, E. PEMOB30

Chun, H. OAE0504

Chun, T.‐W. EPLBB08

Chung, S. PEMOC38

Chungu, C. PEMOC42

Churchill, M. OAA0304, PESUA24

Ciaranello, A. PESUC21, PESUE27

Ciaranello, A.L. OAE0303

Cicilionytè, A. PESUA17

Cissé, M. PEMOD73

Citro, B. OALBF0105

Ciuffi, A. PESUA16

Claassen, C.W. PESUC26

Clark, A. OAA0405

Clark, I. OALBX0102

Clarke, C. PESAF01

Claypool, A. PESUE27

Climent, N. OAA0205

Cluver, L. OAD0204, OAD0503, OAD0702, PEMOB31, PEMOC47, PEMOD65, PESAC15

Cochrane, C. OAA0304, PESUA24

Coelho, M. PESUD38

Coetzee, J. PEMOE33

Cogle, A. PEMOF30

Cohen, K. EPLBA02

Cohen, M. EPLBB08, EPLBC04, OALBX0107

Cohen, S. OALBX0103

Coiras, M. PEMOA28, PESUA25

Colasanti, J.A. PESAB02

Colbers, A. EPLBB06

Cole, B. OAA0402

Coleman, J. PESUD43

Coletti, A. OALBB0102, PESUA19

Collaco‐Moraes, Y. PEMOC43

Collins, L.F. PESAB13

Collora, J. PEMOA30

Colmegna, I. OALBD0102

Comeau, J. OAC0204

Comins, C. PEMOB33, PEMOD63

Connor, D. PESAE08

Connor, N. OAC0302

Conroy, A. OAD0804

Conroy, A.A. PESUD39

Conroy, J. PESAB04

Cook, A.R. PESAD07

Cooney, E.E. OAC0403

Cooper, C. EPLBC09, OALBD0102, PEMOA38

Copertino, D. OAA0305, PESUA21

Corazza, F. PESUA17

Corbeau, P. PESUB14

Corbett, E. OALBB0103

Corciega, J.O. PESUE29

Cordes, C. PESAC11

Corey, L. EPLBA02, EPLBB08, OALBA0102, OALBC0102

Cornell, M. OAC0304

Corona, M. PESUA25

Cortado, R. PESAB01

Cortes, E. OAF0304

Costagliola, D. PESUC20

Costello, D. OALBB0102

Costiniuk, C. EPLBC09, OALBD0102

Costiniuk, C.T. PEMOA38

Cote, H.C. EPLBB02

Cotshott, J. OAA0403

Cotton, M. OALBB0102, PESAA11

Cottrell, M. PEMOA29

Cowan, F. PESUB16, PESUC18

Cox, J. OAD0302, PEMOD74, PESAD14

Crane, H. PESUD30

Creegan, M. PESAA10

Crespo‐Bermejo, C. PESAA03

Cristinelli, S. PESUA16

Croll, M. OALBD0104

Crosby, H. PESUD25

Crowell, T.A. PESAC11

Csete, J. OAF0103, PESUF23

Cuadros, D. OAC0202

Cuevas, G. PESAA03

Cui, Z. PEMOC35

Cunningham, C.K. OAE0303

Curran, A. EPLBB04

Currier, J. EPLBB08

Currier, J.S. OAB0405

Cuvin, J. PESUB22

Cyktor, J. PESAA12

Cymerman, P. OAF0304

Czarnogorski, M. EPLBB05

### D

D'Antoni Brogan, M. OALBX0105

Díez‐Fuertes, F. PESUA23

D'Souza, P. EPLBA02

Da Rocha, M. PESAA01

da Silva, A.P. EPLBE05

da Silva, J. OAE0504

Da Silva, P. PESUC19

Dadabhai, S. EPLBB08, OALBC0102, OALBX0107

Dadirai, T. PESAD02

Dadwal, S. OALBB0104

Dai, L. OALBC0103

Dale, H. OAC0102

Dale, S. PESUD25

Damazio, F. PESAF12

Dambasea, A. PESUE24

Dana, R. EPLBC06

Danda, T. PEMOC44

Dang, C. OAB0102

Dang, N. PESUB25

Dange, A. PEMOD64

Daniel, L. OAF0102

Danion, F. PEMOA37

Darbes, L. OAD0804

Darbes, L.A. PESUD39

Darcis, G. PESUA17

Darko, S. OALBX0102

Daroya, E. OAD0302

Darrow de Mora, D. PESUF24

Das, M. OAE0403, PEMOC33

Dash, R. OAF0202

Dash, S. OAF0202

Dashti, A. EPLBA04, OAA0404

Date, A. PEMOE45

Datta, D. OAB0103

Datwani, S. PEMOA38

David, A. PESUA16

David, R. PEMOD60

David, S. OALBE0104, PESAD23

Davidovich, U. PESAE04

Davies, M.‐A. OAC0304

Davis, K. PEMOC36

Davis, S.L.M. PESUF17

Davy‐Mendez, T. PESAB02

Dawad, S. PEMOE34

Day, S. PEMOF32

Ddaaki, W. PESAE16

De Anda, S. PEMOE34

De Angelis, D. OAC0302

de Bony, E.J. OAA0104

de Bree, G. PESAE04

de la Fuente, H. PEMOA27

De La Torre‐Tarazona, H.E. PESUA23

De la Fuente, S. PESAA03

de los Santos, I. PEMOA27, PESAA03

de Montigny, C. PEMOB36

de Pokomandy, A. PESAD14

De Rosa, S.C. EPLBA02, OALBA0102

de Souza, M. OAA0105, OAA0302

De Vos, M. OAD0502

de Vries, C. OALBA0105

De Wever, R. PESAD13

De Wit, S. EPLBB04, PESUA17

Deal, C. PELBA01

Dear, N. PEMOB27

Deas Van ONACKER, J. PESUC31

DeCamp, A. EPLBB08

Decosterd, L. OAB0302

Deeks, S. OALBA0105, OALBX0102

Degenhardt, L. PESUC18

Deikus, G. OAA0303

del Canto, S. OALBD0102

Del Mar Masia, M. EPLBB04

del Pino, J. PEMOA32

Del Rio, C. PEMOD51

Delabre, R. PEMOD73

Delabre, R.M. EPLBD07

Delacourt, N. PESUA17

Delaney, J. PESUD30

Delannoy, A. PESAA01

Delany‐Moretlwe, S. OAC0504, OAE0304, OALBX0107, PESAC09

Delaporte, E. OAB0304

Deleage, C. PESUA24

Delgado‐Arévalo, C. PEMOA27

Delhomme, F. PEMOF30

DeLong, A. PESAC04

Delpech, V. OAC0302

DeMarco, M.L. PEMOA38

Dembélé Keïta, B. PEMOD73

DeMoor, R. EPLBB05

Denoeud, L. PEMOD55

Deschenes, M. PESUB18

DesLauriers, N. PESAC06

Desmond, C. PEMOC47

Destefano, M. OAB0303

Detels, R. PEMOC45

Dettinger, J. OAC0502

Devanga, A. PESAE05

Devezin, T. OAB0203

Deville, J. OAB0104

Devine, J. PESAE08

Dewaele, J. PESUD42, PESUD47

Dhungel, R. PESUF18

Di Biagio, A. PESUB19

Di Caccio, M. PEMOD71

Diaba, K. PEMOD61

Diabaté, S. OAD0505

Diagne, R. EPLBD07

Dias, P. PESAD01

Diawara, Y. PESUC20

Diaz Rodriguez, Y. OAC0402

Diaz, D. PESAE12

Diaz, J. OAB0404

DiazGranados, C.A. EPLBA02

Dickter, J. OALBB0104

Dicto, A. PESAD05

Dietze, P. PEMOC35

Diez‐Fuertes, F. PESAA13

Dikgale, K. PEMOD62, PESUE31

Ding, S. OAA0405

Dingake, O. OAF0404

Dintwe, O. EPLBA02, OALBA0102

Diouf, P. PESAD01

Diseko, M. PELBB02

Djemai, A. PESUE30

Dladlama, T. OAF0104

Dlamini, S. OAB0202, OAB0203

Do Quang, K. OAE0505

Do Tuan, K. OAE0505

Doan, I. PELBE01

Dobbs, T. OAC0305

Doblecki‐Lewis, S. PEMOD75

Dodd, M. OAB0402

Doherty, M. OAB0404

Dolezal, C. OAD0705

Domínguez‐Rodríguez, S. EPLBB06

Domínguez‐Rodríguez, S.D.‐R. OAB0205

Domínguez, L. PESAA03

Dombrowski, J. OALBX0103

Domerçant, J.W. OAF0102

Domingo, P. OAC0402

Donald, K.A. OAB0204

Donazzolo, Y. PESUB26

Dondbzanga, D. PEMOD73

Dong, D.D. PESUF17

Dong, H. PEMOC35

Dongmo Tsague, L. PEMOD56

Donnell, D. EPLBC04, OAC0504, OALBX0103

Doraivelu, K. OAD0703, PEMOD51

Dorey, D. PESUB24

dos Passos Cunha, M. PEMOA35

dos Santos, J.C. EPLBA07

Doudtchitzky, N. PEMOB35

Douek, D. OALBX0102

Dougherty, S. PESUD30

Dourado, I. OAE0302

Dovbakh, G. PESUF14

Dowdy, D. PESAB08

Doyle, J. PESAC12, PESAC13

Doyon‐Laliberté, K. PESAA09

Draenos, C. PESAE13

Dringus, S. EPLBE07

Dromer, F. PESAB06

Drumright, L. PESUD30

Duan, X. EPLBB09

Dubé, M. PESUA22

Dubois, M. PEMOB32

DuBose, S. OALBX0106

Dubula, T. EPLBA02

Duby, Z. PEMOC37

Ducruet, T. OAB0105

Duerr, A. PESAC07

Duffy, A. EPLBB04

Duffy, F. PEMOB32

Dufour, C. OAA0105, OAA0302

Dugdale, C.M. OAE0303

Dula, D. OAB0405

Dulce, H. OALBD0104

Dumond, J. PESUA14

Dunaway, K. PEMOF28

Duncan, M.C. PEMOA38

Dunham, R.M. OAA0404

Dunn, D.T. PEMOC43

Dupuis, E. PEMOF26

Duracinsky, M. EPLBD06

Durand, M. OAA0202, PESAA07

Durojaye, P.E. OAE0205

Duru, E. PESAD23

Dutilleul, A. PESUA16

Duval, N. PESUC31

Duvivier, C. EPLBD06

Dwivedi, S. PEMOF34

Dye, B. PESUA14

Dyer, J. PESAC05

Dyson, A. PESAE14

Dzamatira, F. PESAD02

Dzinotyiweyi, E. PEMOC44

Dzumbunu, S. OAD0503

### E

Easley, K. PEMOD51

Eaton, J. OAD0505, PESUC18

Ebonwu, J. PESAC16

Edlefsen, P.T. PEMOB32

Edmiston, L. PESAF03

Eduardo, S. OAB0305

Edupuganti, S. PELBA02

Edward, J. PEMOB34

Edwards, A. OAC0202, PESAE16

Edwards, O.W. OALBD0105

Egan, J.E. PEMOC45

Egger, M. OAB0302, OAC0304, PESUB17

Eigege, W. PESAB04

Ekem‐Ferguson, G. PEMOD61

Ekong, J. EPLBE01

Ekstrand, M.L. PEMOB37

El‐Far, M. OAA0202, PESAA07

El‐Sadr, W. OAC0404, PEMOC38

El‐Sadr, W.M. PESAC01

Ellard, J. PESUD41

Elliott, R. OALBF0102, PESAF01, PESAF03, PESUF23

Ellsworth, G. OAA0305

Elorreaga, O. PESAE12

Elorreaga, O.A. PESAC08

Elyanu, P. OALBC0102

Elyanu, P.J. OAB0202

Emery, M. OAA0303

Emmanuel, E. OAF0102

Emslie, G. OAB0104

Enane, L.A. OAE0502

Encarnacion, M. PESUE29

Enciso Durand, J.C. PEMOD71

Ene, A. PEMOB30

Enel, P. PESAD13

Enemo, A. PEMOD72, PESUE31

Engamba, D. OALBB0105, PEMOB28

Engelman, A. OAA0303

Engler, K. PESAD14

English, T. PEMOE47

Ennis, S. PEMOA38

Ensminger, A. PEMOC44

Enugu, A. PESAE05

Epie, T. PESAE11

Erkola, T. PESAF10

Eron, J. EPLBB08, PESAA12

Eron, J.J. PESAB02

Erondu, N. EPLBE07

Esber, A. PEMOB27, PESAC11

Esemu Livo, F. PEMOA34

Eshleman, S. EPLBC04

Eshleman, S.H. OALBX0107

Essam, T. PESUE20

Esser, S. PESAC11

Essien, I. PEMOB30

Estes, J. OAA0304, PESUA24

Etowa, E. PEMOF29

Etowa, J. PEMOF29

Evans, C. PESAB07

Evans, T. PESAE14, PESUF16

Ewane, P.‐P. PESAE11

Excellent, M.L. OAF0102

Exner, T. PESUD37

Eze, U. PESAD23

Ezechi, O. PEMOF32

Ezeh, A. PESAD23

Ezenwa, U. PEMOC39

### F

Facciolla Paiva, V.S. PESAD10

Fafi‐Kremer, S. PEMOA37

Fahey, C.A. PEMOE42

Falcó, V. OAC0402

Falcinelli, S.D. OAA0404

Falcone, S. OAA0204

Falkard, B. OALBA0103

Fallon, S. PEMOD75

Fane, K. OAC0205

Fane, O. OAC0205

Farach, N. OAC0102

Farahani, M. PEMOC38

Farber, E. OAD0703

Farid, M.S. PESUE18

Farirai, J. OAB0202

Farley, S.M. OAC0305

Farquhar, C. PESAC06

Farrior, J. OALBX0107

Fast, P. OAE0303

Fatti, G. EPLBE10

Faucher, L. OAC0303

Fazzari, M.J. PESUB15

Feaster, D. OAB0102

Feather, J. OAF0305

Fedosik, A. OAF0105

Feinstein, M.J. PESUB15

Felsen, U.R. PESUB15

Feng, Y. OALBB0104

Fenn, T. PEMOC42

Ferguson, L. PESUD50

Fernández‐Rodríguez, A. PESAA03

Fernandes, J.C. PESAC02

Fernandez, A. PESUD28

Ferrari, G. EPLBA04, OAA0403, OALBA0102, PEMOE36

Ferreira Lima, C. EPLBC07

Ferreira, E. PEMOE37

Feutz, E. PEMOE41

Feyznezhad, R. PESAA06

Fidler, S. OAC0104, PEMOC36

Fielding, K. OAB0402, OALBB0103

Fierbințeanu, C. PELBD02

Figueroa, M.I. OAB0303, PEMOB35

Filali, A. OAA0202

Filimon, M. PELBD02

Filipescu, I. PELBD02

Fink, V. OAB0303, PEMOB35

Finlayson, T. OAC0303

Finzi, A. OAA0405, PESUA22

Fischer Walker, C. PEMOE38

Fischer, P. PEMOA37

Fischl, M.A. PESAB13

Fisher, K. OAE0504

Fisher, L. OALBC0102

Fisher, P.W. OAD0705

Fitzmaurice, A.G. PEMOC40

Fitzsimmons, E. PESUD30

Flanagan, C. PESUE27

Flanzer, J. OAD0205

Flaxman, S. PEMOC47

Fletcher, C. EPLBB04

Fliedner, P. OALBD0104

Florence, E. EPLBB05

Flores Anato, J.L. PESUD36

Floyd, S. OAC0104

Foalem, M. OAB0304

Fockele, J. OAD0205

Fok, E.T. EPLBA07

Folayan, M. PEMOD62

Folch, C. EPLBD07

Fong, Y. OALBA0102

Fonseca Do Rosario, N. OAA0202

Fonseca, F. PESAF09

Fonseca, F.F. PEMOF34

Ford, N. OAB0404

Ford, S. EPLBC04, PEMOB29

Ford, T. EPLBF03

Forman, S. OALBB0104

Forno, D. PEMOE45

Forrest, D. OAB0102

Forster, N. PEMOC44

Fosua Clement, N. PESUF24

Fouche, J.‐P. OAB0204

Fouda, G.G. OAE0303

Fowke, K.R. PESUA15

Fowler, M.G. OAB0405

Fowler, R. OAB0404

Fowokan, A. EPLBC09

Fox, M.P. PESAE06

Franks, J. EPLBC04

Fraser, C. OAC0104

Fraser, D. PESUC25

Fredericksen, R. PESUD30

Freedberg, K. PESUC21

Freedberg, K.A. OAE0303, PESUE27

Freeman, A. OAE0502

Freitas, R. EPLBD07

French, A.L. PESAB13

Freshman, M. OAD0602

Friedman, M.R. PEMOC45

Frola, C. PEMOB35

Fromentin, R. OAA0105, OAA0302

Fu, N. PESUD50

Fuhrer, J. PEMOC48

Fuks, F. PESUA17

Fundulu, E. PESUC26

Funsani, P. OALBC0105

Fwoloshi, S. OALBB0105, PEMOB28

### G

Gómez, L. OAC0502

Górgolas, M. PESUB21

Günthard, H.F. OAB0302

Gaborets, T. OAC0505

Gabriel, M.M. PEMOC43

Gabrielle Delgado, G. PESUA22

Gadam Rao, P. PESAA06

Gadama, D. OALBX0107

Gaebler, C. OAA0305

Gafos, M. OAD0305, PEMOC43, PESAD06

Gakindi, J.F. PESUC31

Gakuo, S. OAE0105

Galea, J.T. OAD0403

Galinskas, J. PESAC02

Galons, H. PESUA16

Gama, J. EPLBB03

Gama, L. EPLBB08, OALBB0102, PESUA14

Gamazina, K. OAC0505

Gamble, C. PESAD01

Gamble, T. PELBA02, PESUA14

Ganase, B. PEMOA38

Gandhi, M. PESAB03, PESUA19

Gandhi, R. PESAA12

Ganesan, K. OAC0404, PESAC01

Ganga, Y. PEMOA31

Gantner, P. PEMOA37

Gao, L. PESAD06

Gao, Y. OALBC0103

Gaolathe, T. PELBB02

García‐Fraile, L. PEMOA27

García‐Gutierrez, V. PESUA25

García‐Pérez, J. PESUA23

Garcia de Leon Moreno, C. PEMOF28

Garcia‐Beltran, W. PELBA01

Garcia‐Iglesias, J. PEMOD68

Garcia, J.V. OAA0403

Garcia, M. PEMOC49

Garis, C. PESUB24

Garrett, N. OALBC0102

Gartner, M. OAA0304

Gaspar, M. PEMOB34

Gasper, M.A. PEMOB32

Gastaldi, F.B. EPLBE05

Gatanaga, H. PESAB10, PESAC17

Gatechompol, S. PESAB09

Gati, B. EPLBC05

Gaur, A.H. PEMOB29

Gauthier, T. PESAC09

Gautier, V. PESUA17

Gavhi, D. OAF0104

Gavrysh, L. PESAD21

Gay, C. PESUA14

Gay, C.L. OAA0403

Gazi, I. PEMOA31

Gbaja‐Biamila, T. PEMOF32

Gebo, K.A. PESAB02

Geerlings, S. PESAE04

Geleziunas, R. OALBA0103

Gendron‐Lepage, G. OAA0405, PESUA22

Gendzekhadze, K. OALBB0104

Geoffroy, E. OAE0202

George, K. OAB0405

Georgina, N. PESAE03

Gerard Audace, H.N. PEMOE32

Gerke, D. OAD0602

Germann, J. OAE0102

Getwongsa, P. OAC0503

Getzler, A. PESAA04

Ghazaryan, L. OAE0303

Ghelani, S. PESUE30

Ghosh, S. PESAE05

Ghosn, J. EPLBB05

Ghule, U. EPLBA03

Giaquinto, C. OAB0205

Gibb, D.M. OAB0204

Gibbs, T. PESAD08

Gilbert, P. OALBC0102

Gilbert, P.B. EPLBA02, OALBA0102

Gill, M. EPLBB05

Gill, M.J. PESAB02

Gillis, J. PEMOB34

Ginda, A. PESUA18

Ginsberg, M.S. PESUB15

Giordana, G. OALBF0104

Girard, G. EPLBD07

Girish, S. PESUB23

Giron, L. OALBA0105

Gitau, E. PESAC06

Githiru, F. OAF0404

Gittings, L. OAD0204, PEMOD65

Glashoff, R. PESAA11

Glick, S. PESAC07

Glidden, D.V. PESAB03

Glotfelty, J. OAD0602

Gobah, P. PESUF24

Gobburu, J. OAA0403

Godfrey, C. OAE0504, PEMOE45, PESAE08, PESUE20

Godin, G.H. OALBD0102

Godoy, S. PESAD15

Golden, M. PEMOC44

Goldenberg, S. OAD0103

Goldman, A. OALBA0105

Goldman, P. PEMOC47

Goldstein, D. PEMOE45

Goldstein, M. PEMOD51

Golichenko, M. OAF0103, PESUF23

Golin, C. PESUD31

Gollapalli, K. OAF0202

Golmei, K. OAF0202

Golub, E.T. PESAB13

Golub, S. OAB0103, PELBD02

Golub, S.A. PESUD34

Gombakomba, G. PEMOD55

Gomes, D. PEMOE45

Gomes, M.F. OAC0405

Gomez‐Feliciano, K. EPLBC04

Gonese, E. OAC0102

Gonese, G. PESAD18, PESAD22

González, T. OAA0205

Gonzalez, S. PESUD25

Gorelick, R.J. OAA0403

Gornalusse, G.G. EPLBA06

Gosselin, A. OAA0202, PESAA07

Govender, V. EPLBA02, OAB0405

Govha, M. PESAB07

Govindasamy, D. PEMOE36

Goyal, N. PEMOB29

Goyette, A. PEMOB36

Goyette, G. OAA0405

Grabar, S. PESUC20

Grabbe, K. OAE0103

Grabinsky, S. PESAA02

Grabow, C. OAC0504, OALBX0103

Grabowski, K. EPLBC08

Grace, D. OAD0302, PEMOB34

Graham, H. PESUB22

Graham, O. PEMOD54

Grainger, K. PESUE30

Granés, L. EPLBB07

Grant, K. OAF0404

Grard, E. PESUC18

Gray, C.M. PEMOB32

Gray, G. OALBC0102, PEMOE33

Gray, G.E. EPLBA02, OALBA0102, PESAC16

Gray, R. EPLBC08

Green, K. OAE0505, OALBE0103, PESUF16

Green, P. PEMOC47

Greene, K.Y. OAD0403

Gregson, S. PEMOC36, PESAD02

Grennan, T. EPLBC09, PEMOB34

Grenville ‐ Grey, T. PEMOD53

Grey, C. OAD0302

Griesel, R. EPLBB01

Grimsrud, A. OAE0104

Grimwood, A. EPLBE10

Grinsztejn, B. EPLBC04, PESAE12

Groenewold, N.A. OAB0204

Gross, J. PEMOC42

Grosso, A. PESAD09

Grove, D. EPLBA02

Grove, R. PESUB21

Grulich, A. PESUC25

Grund, J.M. PEMOE34

Grundy, R. PEMOD59

Grunenberg, N. EPLBA02

Gruskin, S. PESUD50

Guadamuz, T. PESUC24

Guanira, J. PESAC08

Guanira, J.V. PESAE12

Guanizo, A. OAA0304

Guaraldi, G. PESUB18

Guethina, G. OAF0102

Guillén‐Díaz‐Barriga, C. PESAE12

Guillemi, S. PEMOA38

Guimarães, N.S. OAE0302

Guindo, M. PEMOD73

Guiteau, C. EPLBE03

Guivel‐Benhassine, F. PEMOA35

Gulemye, I. OAD0603

Gulfam, F. OAB0103, PESUD34

Gumbie, R. PEMOD59

Gun, A. OAB0303

Gundacker, H. EPLBC05

Gunst, J.D. PEMOA26

Guo, S. OALBA0105

Gupta‐Wright, A. OALBB0103

Gupta, A. OAB0103, PESUD34

Gupta, A.K. PEMOB34

Gupta, V. OAD0203

Guthrie, B. PESAC06

Gutierrez, C. OAC0102

Gutierrez, L.‐A. PESUB14

Gutin, S. OAD0804

Gutin, S.A. PESUD39

Gutner, C.A. EPLBB05

Guy, R. PESUC25

Guzzo, C. PESAA02

Gwanmesia, P. PEMOA34

Gwarazimba, F. EPLBE04

Gwimile, J. OAB0202

### H

Ha, T. PESUF17

Haag, K. PEMOB31

Haas, A. OAE0502

Habanyama, M. OALBD0102

Haberlen, S. PEMOC45

Habib, T. PESAA01

Habiyambere, V. OAE0305

Hachiya, A. PESAC17

Hadjad, J. PEMOA35

Hahn, A. PESUD30

Haile, A. OAC0102

Haimbe, P. OAE0104, PESUE23

Hakim, A.J. EPLBD08

Hale, F. OAF0305

Hallstrom, H. OAD0405

Hambayi, M. OALBF0104

Hamouda, M. OALBX0102

Hamp, A. PEMOE43

Han, K. PEMOB29, PESUB24

Han, Y. EPLBB09

Hanass‐Hancock, J. PESUD37

Haney, J. PESAD01

Haniffa, R. OAB0404

Hanna, D.B. PESUB15

Hannah, M. EPLBC06, OALBD0105

Hans, L. PESUC19

Hanscom, B. EPLBC04, OALBX0107, PESUA14

Hansmann, Y. PEMOA37

Hanu, L. PELBD02

Hanunka, B. OALBC0105

Happel, A.‐U. PEMOB32

Haq, H. OAB0202

Harases, B. OALBE0104

Harding, P. OALBB0102

Hargreaves, J. PEMOC36

Harney, B. PESAC13

Harper, G. PESUD46

Harrington, C. PEMOB29

Harris Requejo, J. PEMOD56

Harris, L. PESUF24

Harris, M. PEMOA38

Harris, R. OAC0302

Harris, S. OAC0303

Harris, T.G. PEMOD60

Harrison, A. PESUD37

Harrison, S. PESUF16

Harrison, T. PESAB06

Harrison, T.S. PESAB11

Hart, T. OAD0302, OAD0704

Hartikainen, J. PESAC11

Hasen, N. PEMOD53

Hassan, A.S. EPLBE09

Hassan, Z. OAE0103

Hassman, M.A. PESUB24

Hast, M. PEMOC42

Hatt, E. OAF0402

Hauck, K. PEMOC36

Haufiku, B. OALBE0104

Haught, T. PELBF01

Hausler, H. EPLBE02, PEMOB33, PEMOD63

Hausmann, K. OALBD0104

Havens, J. PESUD43

Havlir, D. OAB0403, OALBE0102, OALBX0103

Haw, J.S. OAC0403

Hawk, C. PESUE28

Hawken, M. PEMOD60

Hayashi, K. PEMOC35

Hayes, R. OAC0104, PEMOC36

Hayward, K. PESUE30

He, Z. PESUA14

Heath, S. PESAE01

Hedgcock, M. PEMOA38

Heerten‐Rodriguez, L. PESUD43

Heffron, R. PEMOE41

Hegde, A. OALBE0103

Hellard, M. PESAC12, PESAC13

Hemat, N. PEMOA33

Hempel, S. PESUD50

Henderson, G.E. PESUD31

Hendrickson, C. PESUE23

Hendriksz, S. PESAE15

Henne, J.C. PELBF01, PESAE14

Henry, A. OALBX0102

Heptinstall, J. EPLBA02, OALBA0102, PELBA02

Herbeck, J. OAC0104, PESAC06

Herbert, N. EPLBA08

Herna Sari, A. OALBF0105

Hernalsteens, O. PESUA16

Hernandez‐Avila, M. OAD0304

Hertzog, L. OAD0702

Heylen, E. PEMOB37

Hickey, A.C. PESUC29

Hickman, M. PEMOC35

Hicks, L. OALBE0103

Hien, B.T.T. OAC0102

Higgins, R. PESAE13

Hightow‐Weidman, L. EPLBC06, PELBD02

Hill, A. EPLBB01, PELBB01, PEMOB28, PESUC24

Hillis, S. PEMOC47

Himansu, S. OAA0204

Hindman, J. OALBX0105

Hinman, K. OAF0103, PESUF23

Hioe, C.E. EPLBA01

Hipp, P. OALBX0106

Hisham, S. PEMOA36

Hladik, F. EPLBA06

Hlongwe, K. PEMOE33

Ho, W.Y. PEMOA36

Ho, Y.‐C. PEMOA30

Hoagland, B. PESAE12

Hoang, H. PELBE02

Hocqueloux, L. PEMOA35

Hoddinott, G. PEMOC36

Hoejrup, A. OAD0104

Hoffman, N. OAB0204

Hoffman, R.M. PESAE09

Hoffman, S. PESUD37

Hoffmann, C. PEMOA28

Hogan, B.C. PESAB02

Hoh, R. OALBX0102

Hoj, S. PEMOB36

Holmes, D. PEMOA38

Holmes, L.B. PELBB02

Holt, M. PEMOF30, PESUC24

Honermann, B. EPLBE07, PEMOE35

Hong, K.Y. OALBA0105

Hong, L. PEMOF34

Hong, S.Y. PELBC01

Honglawan, K. PEMOE46

Honig, L. EPLBF03

Hope, R. PESAD08

Hoque, M.T. PELBA03

Horberg, M.A. PESAB02

Horn, T. PEMOE43

Horne, E. EPLBC05

Horner, A.M. OAA0404

Horsfall, S. OAE0303

Hosek, S. OAC0504, OALBX0107, PEMOC49

Hosseini, P. OAC0102

Hosseinipour, M. EPLBB08, OALBX0107

Hosseinipour, M.C. OAE0304

Hou, Q. PEMOC48

Hoving, J.C. PESAB06

Howard, J.N. OAA0102

Howard, S.A. PEMOA29

Hrapcak, S. PEMOD55

Hsiao, Y.‐H. PESUB25

Hu, A. PESAE13

Hu, G. EPLBA01

Huang, J. PEMOB29

Huang, P. PESUC30

Huang, S.‐H. PESAA12

Huang, S. PELBA03, PESUA19

Huang, Y. EPLBA02, OAD0303, OALBC0102, PELBA02, PESUA14

Huber, A. PESUE23

Huber, A.N. PESUE19

Huggins, R. OAE0404

Hughes, J.P. OALBX0107

Hughes, S.M. EPLBA06

Hui, C. OAD0704, OALBD0102

Hull, M. PEMOA38

Humes, E. OAC0403

Humphrey, J.H. PESAB07

Humphries, H. PEMOD62, PESUE31

Hundal, A. PEMOD69

Hung, C.‐C. OALBX0105

Hung, P. PESUF16

Hunidzarira, P. EPLBB08, OALBX0107

Hunter, A. PEMOA33

Hural, J. EPLBA02, OALBC0102, PESUA14

Hurt, C. PELBA02

Hurtado, I. PESAA13

Hussen, S. OAD0703, PEMOD51

Hwang, T.‐F. PESUB25

Hyder, S. PESUD42, PESUD47

Hyle, E. PESUC21

Hyrien, O. OALBA0102, PESUA14

### I

I‐Ching, J. PEMOA36

Iacono, M. OAF0305

Iannantuono, M.V. PEMOB35

Ibáñez‐Carrasco, F. PESAE13

Ibanescu, R.‐I. OAC0105

Ibrahim, A. OAF0303

Ichihara, M.Y. OAE0302

Igbebor, I. PEMOB30

Igbiri, S. OALBE0105

Igweta, R. PESAF07

Ihekanandu, U. OAE0402

Ijagason, T. PESAD17

Ijeoma‐Johnson, U. OAE0103

Ikpeazu, A. OAE0103, PESAB04

Ilika, F. OAE0402

Imahashi, M. PESAC17

Imalingat, T. PESUF17

Imaz, A. OAC0402

Indongo, R. OALBE0104

Indravudh, P. PESAD06

Innes, C. EPLBA02

Innes, S. OALBX0107

Inyang, A. PESAB04

Iovita, A. EPLBF01, PESUF21, PESUF23

Iroezindu, M. PEMOB27

Iseniyi, J. OAE0103

Islam, D.S. PESUE18

Itoh, M. PEMOC42

Ivanchuk, I. EPLBC02

Iversen, E.F. PEMOA26

Iwasaki, A. PEMOA30

Iwase, S.C. PEMOB32

Iwelunmor, J. PEMOF32

Iyer, S. EPLBB05

Izard, D. PEMOB29

### J

Jackie, N. PEMOD58

Jacobs, T.G. EPLBB06

Jacobson, D. PELBB02

Jacobson, J.M. PESAB02

Jahn, A. OAC0305

Jailani, A.M. PESUF15

Jain, R. PESUD42, PESUD47

Jalali, M.S. PESUE27

Jalil, E. EPLBC04

Jamal Eddine, J. OAA0304

Jamieson, L. OAE0104, OAE0304, PESUE19

Janamnuaysook, R. OAC0503, OAF0204

Janes, H. EPLBA02

Jang, G.M. OAA0104

Janini, L.M.R. PESAC02

Janjua, N. EPLBC09

Janse van Rensburg, A. OAB0205

Jansen, K. PESAC11

Janthawilai, K. OAF0204

Janyam, S. PESUC29

Jao, J. PEMOB32

Jaramillo, S. EPLBF02

Jardim dos Santos, M. PESAD10

Jarrin, I. PESAC12

Jarvis, J.N. PESAB06, PESAB11

Jaspan, H.B. PEMOB32

Jassat, W. OAB0404

Javadekar, S. PESUE21

Jaya, A PESUD45

Jayaweera, D. OAB0102

Jean Juste, M.A. EPLBE03

Jean, M. OAF0305

Jeanne, B. OAB0105

Jefferys, L. OAC0304

Jennings, A. EPLBC04

Jere, J. OAE0202

Jessen, H. PESAC11

Jesus, G.S. OAE0302

Jewkes, R. PEMOE33

Jiang, H. OAD0303

Jianu, C. PELBD02

Jiménez‐Sousa, M.Á. PESAA03

Jiménez, P. PESUA23

Jin Kee, J. EPLBA02

Jin, D. OALBA0103

Jinga, N. PESAE06, PESUE19

Jipa, R. PELBD02

Jochim, J. PEMOB31, PESAC15

Joekes, E. OALBB0103

Jogiraju, V. PESUB22

Johannsen, I.M. PEMOA26

Johansen, I.S. OAC0402

John‐Dada, I. OAE0103

John‐Stewart, G. OAC0502, PESAC05

Johnson, A. PEMOD75

Johnson, C. PESAF01

Johnson, J. EPLBC06, OAC0303

Johnson, K. PEMOD52

Johnson, K.A. PESAB03

Johnson, L.F. OAE0304

Johnson, M. EPLBB04

Johnson, M.A. PEMOA33

Johnston, C. OAA0305

Jollimore, J. OAD0302

Jonah, L. PEMOD74

Jones, B. PESUB21

Jones, G. PEMOF26

Jones, J. EPLBC06

Jones, R.B. OAA0305, PESAA12, PESUA20, PESUA21

Jonsson‐Oldenbüttel, C. EPLBB05

Joseph, P. EPLBE03

Joshi, S.H. OAB0204

Jossefa, M.V. PEMOC46

Judge, M. PESAF12

Juga, A. PEMOC46

Juhasz, M. OALBX0106

Juma, E. PESAC06

Jupimai, T. OAA0105, OAA0302

Jurgens, R. PESUF21, PESUF23

Justice, A.C. PESAB02

Justman, J. PEMOC38

Justman, J.E. PELBC02

Jutile, L. OAF0102

### K

K, P. PESAD12

Ka Hon Chu, S. PESAF02, PESUF22

Kaakyo, M. PEMOC42

Kabaghe, A. OAC0305

Kabaghe, A.N. OAC0405

Kabami, J. OAB0403, OALBE0102

Kabanda, J. EPLBC08

Kabanda, R. OAD0105

Kabatesi, D. EPLBC08

Kablawi, D. PESUB18

KABONGO, B. EPLBE10

Kabwe, M. PESUD46

Kadama, H. PEMOE41

Kadir, P.T. EPLBF04

Kaeuffer, C. PEMOA37

Kagaayi, J. EPLBC08, PEMOC42

Kagoli, M. OAC0405

Kagongwe, S. PEMOC42

Kaida, A. EPLBB02, PEMOF29

Kailasom, A. PESAE05

Kajanga, C. PESAB06

Kakande, E. OAB0403

Kakende, M. OALBE0104

Kakkar, F. OAB0105, OAC0204

Kalamya, J.N. PESAE10

Kalata, N. OAC0305

Kalikawe, R. PEMOA38

Kalokhe, A. OAD0703

Kalonji, D. OALBX0107

Kalubula, M. OALBC0105

Kalule, H.N. PEMOC49

Kamali, A. PESAD06

Kamanga, G. PESUF24

Kamanga, P. OALBE0104

Kamara, R. EPLBE01, PEMOC40

Kamarulzaman, A. PEMOA36, PESUF15

Kamat, N. PEMOC46

Kambanzera, J. PEMOC44

Kambara, K. PESUB25

Kambire, E.A. EPLBD07

Kambugu, A. OAE0102

Kamere, N. PEMOE34

Kamgaing, N. PESAE11

Kamissoko, A. PEMOD73

Kamoga, N. EPLBE08

Kampamba, D. PEMOB28

Kamugisha, B. OALBE0102

Kamwiine, C. PESAD11

Kamya, M. OAB0403, OALBE0102, PESAB08

Kamzati, M. OAC0405

Kanamori, M. PEMOD75

Kaneko, N. PESUC24

Kanje, V. EPLBB03

Kankindi, I. OAC0102

Kannemeyer, N. PEMOD65

Kanphukiew, A. PESUC22

Kansiime, L. OAD0603

Kanyanda, J. OAD0104

Kanyenda, T. EPLBE04

Kanyinda, J.M. EPLBE10

Kanywa, J.B. OAB0202

Kaplan, K. EPLBE07

Kaplan, R.C. PESUB15

Kapogiannis, B. OALBE0102

Karanja, E. OAD0102

Karg, C. PESUA14

Kari, N. PELBF01

Karim, F. EPLBA08, PEMOA31

Karki, K. PESUF18

Karris, M.Y. PESAB02

Karuna, S. EPLBB08, PESUA14

Kashuba, A. PEMOA29

Kasoka, K. PESUD50

Kassanjee, R. OAC0304

Kassaye, S. PESAB13

Kassim, S. EPLBA02

Kasule, J. OAA0303, PESUA20

Katabaro, E. PEMOE42

Katahoire, A. OAE0503

Katanda, Y. EPLBE04

Katekwe, M. PESUD33

Katirayi, L. PEMOD55

Katoro, J. EPLBD08

Katumba, K. PESAD06

Katwesigye, R. OAE0503

Katz, I.T. PEMOE41

Kaufmann, D. PESAA09

Kaufmann, D.E. PESUA22

Kaul, R. OAD0305

Kaur, J. OAB0103, PESUD34, PESUD45

Kaur, M. OAD0203

Kaushic, C. PESAA05, PESUA15

Kavanagh, M. EPLBE07, PESAF10

Kawungezi, P.C. OAD0805

Kay, A. OAB0203

Kayesu, I. PESAD06

Kayigamba, F. OAC0305

Kazatchkine, C. OAF0103, PESAF01, PESUF23

Kazounis, E. OAB0402

Kearns, C. PESUA18

Keating, R. EPLBC02, PELBE01

Keating, S. PESAA12

Keim‐Malpass, J. PEMOE43

Kelly, J. OAD0204, PEMOD65

Kelly, M. PESAD20

Kelvin, E. PESAC14

Kempf, M.‐C. PESAB13

Kendrick, C. PEMOF35

Kenji Mfuh, O. PEMOA34

Kennard, B. OAB0104

Kennedy, C.E. PEMOD56, PESAE16

Kenny, A. OALBA0102

Kerbay, C. PESUF24

Kerin, T. PESAB01

Kerndt, P. PEMOE45

Kerr, S.J. PESAB09

Kerrigan, D. OAD0103

Ketter, N. OALBC0102

Kgokolo, M. OAF0104

Kgwaadira, B. OAC0205

Khadka, P. OAA0305, PESUA20

Khaitan, A. OALBB0102

Khan, A.R. PEMOF26

Khan, F. PESAD12

Khan, K. PEMOA31

Khan, T. EPLBC04

Khaydarova, T. OAF0305

Khosropour, C. PESAC07

Khoza, N. OAC0504

Khozomba, N. OAE0202

Khumalo, S. PESUD37

Khuzwayo, S. OALBA0102

Kiatchanon, W. PESUC22

Kibugi, J. PESAC05

Kibuuka, H. PEMOB27

Kibuuka, J. PEMOE41

Kidambi, T. OALBB0104

Kiertiburanakul, S. OALBX0105

Kiesa, D. OAE0504

Kiesman, L. PESUC23

Kigozi, G. EPLBC08

Kigozi, M. PESAE03

Kigozi, S. OAE0503

Kihika, E. OAD0603

Kikuchi, T. PESAC17

Kilam, D. OALBX0102

Killelea, A. PEMOE43

Kim, B. PESUB26

Kim, E. OAC0305, OAC0405

Kim, H.‐Y. OAC0202

Kim, J. OAF0203

Kim, K. PESUB26

Kim, U.‐I. PESUB26

Kimanga, D. EPLBE09

Kimani, J. OAD0305, PESUA15

Kimbui, R. OAE0102

Kimera, E. PESUD49

Kimera, I. OAE0503

Kindra, G. PEMOE34

King, E.M. EPLBB02

King, H. EPLBA04, OAA0404

Kingston, H. PESAC06

Kinker, C. PESAE14

Kinuthia, J. OAC0502

Kinyanjui, D.G. OAD0102

Kiptinness, C. OAE0105

Kiragga, A. OAE0102

Kirchner, H.L. OAB0203

Kironde, J. OAB0403

Kisio, J. OAD0102

Kisubi, N. OAD0802, PESUD38

Kittinunvorakoon, C. OAC0102, OAC0103

Kityamuweesi, T. PESUA20

Kizer, J.R. PESUB15

Kizhner, A. OAB0102

Klassen, B. OAD0302, PESAE13

Klausner, J. PESAB12

Klein, J. PEMOA30

Klein, M. PESAC12, PESAD14, PESUB18, PESUC23

Klein, N. OAB0205

Klingler, J. EPLBA01

Kloverpris, H. EPLBA08, PEMOA31

Kluisza, L. OAD0705

Knowles, K. OAB0104

Knowles, S. PESUB24

Ko, S.H. OALBX0102

Kobayashi, K. PESAB10

Koeken, V.A.C.M. EPLBA07

Koenen, H.J. EPLBA07

Koenig, E. OALBX0105

Koenig, S. EPLBE03

Kogilwaimath, S. PESUC23

Kohler, P. PESAC05, PESAE02

Kojima, N. PESAB12

Kombo, B. PESAD06

Konda, K. PESAE12

Konda, K.A. PESAC08

Kondratyuk, S. PESAF08

Kong, A. PEMOC33

Kong, X. PESAE16

Kongkapan, J. OAC0503

Konrad, C.V. PEMOA26

Korchia, T. PESAD13

Korn, A.K. PESAD18

Kosia, A. PESAC03

Kosloff, B. OAC0104

Kotze, P. EPLBA02, OALBC0102

Kouanfack, C. OAB0304

Koulla‐Shiro, S. OAB0304

Koutsoukos, M. EPLBA02

Kowalska, J. OALBF0103

Kowatsch, M.M. PESUA15

Kpodo, I.E. PESUF17

Krüsi, A. OAD0103

Kra, A. PESAF12

Kraamwinkel, C. PESAE15

Krause, R. PEMOA31

Kreniske, P. OAD0705

Kretzer, L. EPLBD07, PEMOD67

Kristen, S. EPLBF07

Kroch, A. EPLBC09, OALBD0102

Kroch, A.E. OAD0704

Krogan, N.J. OAA0104

Kronfli, N. PESAD14

Krotje, C. OAB0104, OALBB0102

Ku, S.W.‐W. PESUC30

Kublin, J.G. EPLBA02

Kubota, H. PESAB10

Kuchukhidze, S. OAD0505

Kuczynski, K.J. PESUD31

Kuhn, L. OAB0205, PESAC16

Kuhne, M. OALBA0103

Kukreja, A. PEMOA36

Kulchynska, R. EPLBC02, PELBE01

Kumar, A. PEMOB28

Kumar, D. OAD0203

Kumar, M. PEMOF34, PESAC05

Kumar, P.N. PESUB21

Kumar, S. PEMOD64, PESAD12

Kummerlowe, C. EPLBA08

Kumwenda, O. EPLBB03

Kunaka, P. EPLBE04

Kundura, L. PESUB14

Kuo, A. PESAE02

Kurcevič, E. PESUF14

Kurmanalieva, A. PESAD24

Kurniawan, R. OALBC0104

Kuteesa, M. PESAD06

Kuwane, B. PEMOD62

Kwariisima, D. OAB0403

Kwarisiima, D. OAD0105

Kwobah, E. OAE0502

Kwok, C. PESAD07

Kyambadde, P. PESUD38

Kyendikuwa, A.N. OAD0605

### L

Léonard, M. OAF0102

López‐Varela, E. EPLBB07

Lacerda, M. OAB0305

Lachowsky, N. OAD0302, PESAE13

Lacombe, K. EPLBD06, PESAC12, PESUB21

Lacorazza, A. PESAD08

Lacoux, C. EPLBD07, OAD0404

Lagarde, M. PESAA03

Laher Omar, F. EPLBA02

Laher, F. EPLBA02, EPLBB08

Lahouel, N. OAF0305

Lai, L. OALBB0104

Lai, Y.‐T. OAA0204

Lailo, J.M. PESUD26

Lain, M.G. OAB0205

Lairikyengbam, R. OAF0202

Lajoie, J. PESUA15

Lakatos, B. OALBF0103

Lakhi, S. OALBB0105

Lakshmikanth, K. PEMOD70

Lalak, K. PEMOF28

Lam, E. PELBA01

Lamare, N. OAB0304

Lamarre, V. OAB0105

Lambrechts, L. OAA0402

Lamont, S.‐K. PEMOB36

Lamontagne, E. OAE0406, PEMOD62, PEMOD72, PESUE31

Lampe, F.C. PEMOC43

Lancaster, K. OAE0502

Landay, A. PESAA07

Landay, A.L. OAA0202

Landovitz, R. EPLBC04

Landovitz, R.J. PELBA02

Lane, R. OALBD0104

Langwenya, N. OAD0503, PESAC15

Lankiewicz, E. EPLBE07, OAF0302

Lapointe, H.R. PEMOA38

Lara‐Aguilar, V. PESAA03

Larasati, A. PESAF04

Larmarange, J. PESUD36

Larouche‐Anctil, E. PESAA07

Larsen, A. OAC0502

Larsen, M. OAD0205

Lash, R. PESUD38

Lassaunière, R. PESAC16

Lataillade, M. PESUB24

Latham, C. EPLBB05

Lau, C. EPLBB03, PESAD15

Laube, C. OAE0103

Lauer, W. OAA0303

Laughton, B. PESAA11

Laumaea, A. OAA0405

Lauterbach, H. OALBA0103

Lavreys, L. OALBA0102

Lawal, I. PEMOE45

Lawrence, D. PESAB06

Lawrence, D.S. PESAB11

Laws, R.L. PELBC01

Laxmeshwar, C. OALBE0103

Layunta‐Acero, R. PESUA23

Lazarus, E. EPLBA02

Le Douce, V. PESUA17

Le Gouez, C. OAD0404

Le Van, T. OAE0505

Le, D. PESAD07

Leandre, R. PEMOB36

Lebouché, B. PESAD14

Lebouche, B. PESUB18

Lechiile, K. PESAB06

Ledan, N. EPLBE07

Ledin, C. PEMOD68

Lee, A. PESUB20

Lee, C. OAD0803

Lee, C.S. PEMOA36

Lee, G. OAA0305

Lee, G.Q. PESUA20

Lee, J. OALBX0102

Lee, K. OAC0303

Lee, L. PESAE08, PESUE20

Lee, M.‐P. OALBX0105

Lee, T. OAC0204

Leemereise, C. PESUB24

Lefebvre, N. PEMOA37

Lehmann, C. PESAC11

Leider, J. PESUB20

Leke, R. PEMOA34

Lekodeba, N. PESUE23

Lekone, P. PELBC01

Lelutiu‐Weinberger, C. PELBD02

Lemire, B. PESAD14

Leong, C.L. OALBX0105

Leontieva, S. OAC0505

Leroy, V. OAE0303

Lertpiriyasuwat, C. PESUC22

Leslie, A. EPLBA08, OAA0203, PEMOA31

Lesnar, B. EPLBF07

Lessard, D. OAD0302

Leung, V. PEMOA38

Levin, A. OAF0105

Levine, A. OAD0705

Levine, J. PESAD08

Levy‐Braide, B. PESAB04

Levy, C. EPLBA06

Levy, L. OAB0104

Levy, S.L. EPLBB02

Lewin, S. OAA0304

Lewis, L. PEMOD62, PESUE30, PESUE31

Leyre, L. OAA0105, OAA0302, OAA0305

Li, A. OALBC0103

Li, A.T.‐W. EPLBD05

Li, C.‐W. PESUC30

Li, C. PESAA04

Li, G. EPLBA01

Li, H. OAA0403

Li, J. OAD0303, OALBC0103, PEMOC48

Li, S. OALBB0104

Li, T. EPLBB09, OALBX0105

Li, X. PESUD35, PESUD46

Li, Y. EPLBA07, EPLBB09

Li, Z. OALBC0103, PESUD35

Liang, B. OAC0203

Lichtenberg, M. OAD0104, OAD0405

Lifson, J. EPLBA04

Lifson, J.D. OAA0404

Light, L. OAD0704

Light, S. PESAF10

Likoro, E. PEMOC44

Liku, J. OAD0305

Lim, H. PESUF21, PESUF23

Lim, S.H. PESUC24

Limacher, A. OAB0302

Limbada, M. OAC0104

Lin, A. OAA0404

Linda, Z. EPLBE08

Lindsay, B. PESUC26

Lindsey, J. OALBB0102

Ling, J. PESUB22

Linjongrat, D. PESUC29

Liotta, L. OAD0705

Lipesa, S. PESAD06

Lipman, M. PEMOA33

Lise, M. PESUC20

Liu, A. OALBC0103

Liu, M. PELBC02

Liu, Q. OAA0204, PESAA02

Liu, R. PEMOA30

Liu, X. EPLBA01

LiU, Y. PESUC28

Lixandru, M. PELBD02

Llibre, J.M. OAC0402

Lo Hog Tian, J.M. PESUD44

Lockman, S. PELBB02

Lodi, S. PEMOC35

Loeb, T. PEMOC41

Logie, C. OAD0204, OAD0802, PEMOD65, PESUD38

Loh, C.Y. PEMOA36

Loh, P. OALBC0104, OALBE0103

Lohman, D. EPLBF01, OAF0103, PESUF23

Longenecker, C. OAE0503

Loots, B. PESAF12

Looze, P. PEMOF28

Lopardo, G. OAB0303

Lopes, C.A.F. PESAC02

Lopez, C. OALBX0103

Lorente, N. EPLBD07, PEMOD71

Lortholary, O. PESAB06

Losina, E. PESUC21

Louey, M. PESUE30

Loutet, M. OAD0802, PESUD38

Loutfy, M. PESAB02

Lovich, R. PELBF02

Low, A. OAC0404, PESAC01

Lowe, C. PEMOA38

Lu, H. OALBX0105

Lu, S. EPLBA01

Lu, Y. PESUD29

Lua, I. OAE0302

Lubis, M. PESUE26

Lucas, C. PEMOA30

Lucas, J. EPLBC04

Lucas, L. PEMOF28

Lucas, T. PEMOE45

Lucero‐Prisno III, D.E. OAF0303

Luculescu, S. PELBD02

Ludwig‐Barron, N. PESAC06

Luedtke, A. OALBA0102

Luetkemeyer, A. OALBX0103

Lufadeju, F. PESAB04

Lukhele, B. OAB0203

Lumano‐Mulenga, P. OAE0104

Lumenze, P.F. PEMOB30

Lumpa, M. OALBC0105

Luna Siachoque, M.S. PESAF11

Luna, A. EPLBF02

Lunani, L.L. PESAD06

Luo, G. OAC0203

Lusso, P. OAA0204, PESAA02

Lutz, T. PESUB21

Luz, E. OAB0305

Luz, I. OAB0305

Lv, W. EPLBB09

Lwamba, C. PEMOD56

Lwatula, C. PESUD46

Lwin, H.M.S. PESAB09

Lyerly, A.D. EPLBF07

Lynch, S. PESAF10

Lyon, T. PEMOC42

Lyons, B. PEMOB34

Lyons, C. PEMOF28

Lyu, S. PESAA04

### M

Ma, J. PESUD30

Maartens, G. EPLBB01

Mabathoana, J. OAE0203

Mabilat, J. PESUF23

Mabirizi, D. PEMOC42

Mabuta, J. PELBB02

Mac Gowan, R. EPLBC06

Macías‐González, F. PEMOE39

Macatangay, B. PESAA12, PESUA19

MacDonald, S. PELBA01

Macedo, A.B. PESUA21

Machado, D.B. OAE0302

Machawira, P. PELBF02

Machekano, R. PEMOE40

Machinda, A. PESAD20

Machira, Y. PESAD06

Macinko, J. OAE0302

Macinthyre‐Crockett, G. OAC0104

Macintyre, A.N. OAA0403

Macius, Y. EPLBE03

MacLeod, W. PESAE06

MacPherson, P. OALBB0103, PEMOB34

Madela, F.G. EPLBA08

Madge, S. PEMOA33

Madhu, A. PESUD25

Madrid, L. EPLBB06

Maduabum, E. OAE0205

Madubela, N. OAD0502

Magassouba, M. EPLBD07

Magnani, R.J. OALBC0104, OALBE0103

Magnekou, R. PEMOA34

Magno, L. OAE0302

Magnoumba Legnanga, M.M. OAA0203

Magutshwa, S. PESUD37

Mah, Z.H. PESAD07

Mahaka, I. PESAD18, PESAD22

Mahender, T OAF0202

Maher, L. PEMOC35

Maheu‐Giroux, M. OAD0505, PESAC09, PESUC18, PESUD36

Mahler, H. PESAE11

Mahnken, J. PEMOC48

Mahungu, T. PEMOA33

Mahy, M. PESUC18

Maia Acuña, S. PEMOA35

Maia, B. EPLBC04

Maiga, A.I. OAB0205

Maina, A. PESAD06

Maina, G. PESAE02

Majachani, M. OAD0402

Majam, M. EPLBC10

Majji, S. OALBB0102

Majo, F.D. PESAB07

Makadzange, T. OALBA0103

Makama, S. PEMOB33

Makasa, P. PESUC26

Makhaola, K. PELBC01

Makhema, J. EPLBB08, OALBC0102, OALBX0107, PELBB02

Makinson, A. PESUB14

Makiwa, R. PESAD20

Makoni, T. EPLBE04

Makory, V.N. EPLBE09

Makunike, B. PESAD18, PESAD22

Makuyana, C. OALBE0104

Makyao, N. PESAC03

Malaba, R. PESAD18, PESAD22

Malahleha, M. EPLBA02

Malala, L. OAF0104

Malama, K. PESUD38

Malamba, S. OAC0102

Malar, J. OALBF0105

Malati, C. OAE0504

Maldonado, A. PESUB25

Maleche, A. PESUF17

Malee, K. PESUA19

Malhotra, S. OAE0303

Malinski, C. OALBX0103

Mallolas, J. OAA0205

Malogo, R. PESAD06

Malone, S. PEMOD53

Malta, M. EPLBF08

Mamulwar, M. EPLBA03

Manasa, J. PESUB17

Mancuso, N. PEMOC49

Mandalakas, A. OAB0203

Mandarino, E. OALBD0102

Mandima, P. OALBX0107

Mandizvidza, P. PESAD02

Mane, A. EPLBA03

Mangale, D. OAE0105

Manganye, M. OAF0104

Mangold, M. PEMOD60

Mann, C. OAE0303

Mannheimer, S. PESUA14

Manyando, M. OALBE0104

Manyanga, P. PESAD22

Manzanares, M. PEMOA28

Manzini, V.T. EPLBA08

Maphane, L. PESAB11

Maphosa, T. PEMOD55

Mapondera, P. PELBC01

Maponga, T. PESAA11

Maquigneau, A. PESAD13

Marchitto, L. OAA0405

Marcus, U. PESAC11

Margolese, S. OALBD0102

Margolis, D.M. OAA0403, OAA0404

Marima, R. PELBC01

Marinosci, A. OAB0302

Mariyappan, K. OALBE0103

Marley, G. PESUD29

Marlow, M. PEMOB31

Maro, A. PESAC03

Maro, R. EPLBD01

Marquez, C. OAB0403

Marray, T. PESUA16

Martín‐Cófreces, N. PEMOA27

Martín‐Carbonero, L. PESAA03

Martín‐Colmenarejo, S. PEMOA32

Martín‐Gayo, E. PEMOA27

Martín‐Hondarza, A. PEMOA32

Martin‐Iguacel, R. OAC0402

Martin, C. OALBD0102

Martin, H. OALBX0105

Martin, J.N. PESAB02

Martin, M. OAC0102, PESAF05

Martin, V. OAC0302

Martinez Quesada, C. PESAF11

Maruf, M.A. EPLBD04

Marwa, M. OAC0502

Maryono, M. PESUE26

Marzinke, M. EPLBC04, PEMOB29

Marzinke, M.A. OALBX0107

Masching, R. PEMOF35

Masciotra, S. OAC0303

Mascola, J. PESUA14

Mashapa, R. PESAD18

Mashoko, C. EPLBE07

Mashudu, L. PEMOE34

Masika, L. EPLBD01

Mason, R. EPLBA04

Mason, R.D. OAA0404

Masoni, D. OAE0102

Massanella, M. OAA0105, OAA0302

Massaqoui, N. PELBD01

Massawe, D. PEMOD62

Maswai, J. PEMOB27

Maswera, R. PESAD02

Masyuko, S. PESAC06

Matano, T. PESAC17

Matatiyo, B. OAC0405

Matendawafa, A. PEMOD62, PESUE31

Mateos, E. PESUA25

Mathew, C.‐A. EPLBB08

Mathew, C. OALBX0107

Mathews, C. PEMOE36

Mathon, J.E. EPLBE03

Matoba, J. PEMOC42

Matoko, M. OAE0203

Matovu, F.K. OAB0405

Matroos, S. PELBC01

Matse, S. PESAD09

Matthews, G. PESAC12

Mattur R, D. PESUE21

Mattur, D. OAE0305

Maturavongsadit, P. PEMOA29

Matyushina‐Ocheret, D. PEMOF28

Maunder, R.G. PESUD44

Mavasa, N. PESUF20

Mavigner, M. OAA0404

Mayanja, Y. PESAD06

Mayaud, P. PESAC09

Mayer, C. PEMOC48

Mayer, K. PESUD30

Mayer, K.H. OAC0403, PELBA02

Mayondi, G. PELBB02

Mayoral‐Muñoz, M. PESAA03

Mazibuko, M. PEMOA31

Maziya, S. PESAD09

Mbabali, I. PESAE16

Mbaire, S. OAE0105

Mbangiwa, T. PESAB06

Mbewe, N. OALBB0105, PEMOB28

Mbizvo, M.T. OAF0403

Mbofana, F. OAD0505

Mbogo, L. PESAC06

Mbokile, W. PESUC26

Mbonye, M. EPLBD09

Mbowane, V. PEMOE33

Mbuliro, M. OAE0503

McAlpine, A. OAD0802

McBrien, J. EPLBA04

McCabe, L. PEMOC43

McCann, K. PELBB01

McCann, N.C. PESUE27

McCauley, M. EPLBC04

McClelland, A. PESAF01, PESAF02

McCluskey, M.M. OAE0303

McCoig, C. PEMOB29

McCormack, S. PEMOC43

McCoy, S.I. PEMOE42

McDougall, P. PEMOF35

McDowell, H. PESUE30

McElrath, M.J. EPLBA02, OALBA0102

McFall, A. PESAE05

McFall, A.M. PEMOC41

McFarland, E. OALBB0102

McFarland, E.J. OAE0303

McGary, C. PESUB20

McGinnis, K.A. PESAB02

McGuckin Wuertz, K. PESAA10

McGuinness, P. OALBX0106

McHardy, J. PESAF10

Mcingana, M. EPLBE02, PEMOB33, PEMOD63

Mckay, T. PELBF01, PESAE14

McLaughlin, T. PESUB20

McLemore, M. OAF0103, PESUF23

Mcloughlin, J. EPLBE02

McMahon, D. PESAA12

McManus, H. PESUC25

McManus, K. PEMOE43, PESAC10

Meanley, S. PEMOC45

Meda, N. PESAC09

Medjahed, H. OAA0405, PESUA22

Medland, N. PESUC25

Medley, S. OAD0204

Medrano, T. PESUE17

Meek, K. EPLBE02

Mehta, C.C. PESAB13

Mehta, K. PEMOD70

Mehta, P. PESUA19

Mehta, S. PESAE05

Mehta, S.H. PEMOC41

Meinck, F. OAD0503, PEMOD57

Meintjes, G. EPLBB01

Meisner, A. OAC0504, PEMOE41

Mejia Mercado, H. PESAF11

Mejia, A. EPLBF02

Melese, E. OALBE0104

Mellins, C.A. OAD0705, OAF0204

Mellors, J. PESAA12

Mellouk, O. PESAF08

Melounou, C. PEMOA37

Mendez Reyes, J. OAB0203

Mendoza, H. EPLBD07

Meng, X. PESUB26

Menon, A. PESUD46

Mensah, J. PEMOF36

Mernies, G. OAB0303

Mesquita, P. PESUB20

Mestdagh, P. OAA0104

Metras, M.E. OAB0105

Metzner, K.J. OAB0302

Meya, D. PESAB11

Meyer‐Rath, G. OAE0304

Meyer, B. PESAE15

Meyer, N. PEMOA37

Mgodi, N. EPLBB08, EPLBC05, OALBC0102, OALBX0107

Mhango, C. EPLBB03

Mharadze, T. PESAD18, PESAD22

Mhlanga, M. EPLBA07

Miñano‐González, C. PESUA23

Mia, S. OAF0404

Miao, H. OAA0204, PESAA02

Michael, C. PEMOB30

Michael, E. EPLBF03, PEMOF34

Michaud, L. PESAF02

Michels, D. OAD0404

Mikaya, M. EPLBE07

Milic, J. PESUB18

Miller, L. PESUD37

Miller, S.J. PESUF16

Mills, L.A. EPLBC08

Mills, S. OAC0503, PESUC32

Milovanovic, M. PEMOE33

Mimbé, E. OAB0304

Minami, R. PESAC17

Minchella, P. OAC0102, OALBC0105

Mine, M. PELBC01

Minga, A. OAE0502

Minnis, A. PEMOC37

Minoprio, P. PEMOA35

Miró, J.M. OAA0205, OAC0402

Mircovic, K. OAC0305

Mirembe, I. PESAD17

Mirhaji, P. PESUB15

Mitchell, J. OAA0105, OAA0302

Mitchell, K.M. PESUD36

Mitobe, M. PESAB10

Mitra, R. PESAD12

Mittal, S. OAD0203

Miyake, H. PESAB10

Mizushima, D. PESAB10

Mjwana, N. PESUF17

Mkandawire, J. OAD0804, PESUD39

Mkandawire, K. OAD0604

Mkhize, M. OAF0104

Mkhize, N. PEMOD62

Mkungudza, J. OAC0405

Mkwanazi, E. OAC0504

Mmalane, M. PELBB02

Mmasa, N. PEMOB32

Mmolai‐Chalmers, A. PEMOF37

Mngadi, K. OALBA0102

Modi, C. PEMOF26

Mogere, P. OAE0105, PESAE02

Mohammad, G.S. PESUD46

Mohammadi, T. OALBD0102

Mohammed Abdullah, R. PESUE31

Mohammed, R. EPLBE10

Mohan, B. PELBD01

Mohanty, S.C. OAF0202

Mohd Salleh, N.A. PESUF15

Mohlouoa, T. OAE0203

Mohtashemi, N. OAB0405

Moirangthem, A. OAF0202

Mokhele, I. PESUD40, PESUE23

Mokoena, M. OAE0203

Molande, E. PESAD18, PESAD22

Molefi, T. OAC0205

Molewa, T. OAF0104

Molina, J.‐M. EPLBB04

Mon, S.H.H. PESUC29

Monceaux, V. PESUA16

Money, D. OAC0204

Monita, P. OAC0102

Monroe‐Wise, A. PESAC06

Montañez, N. PESUA19

Montaner, J.S.G. PEMOA38

Montefiori, D. PELBA02, PESUA14

Montgomery, E. PEMOC37

Moodie, Z. EPLBA02

Moodley, D. OAB0405

Moodley, R. PESUB21

Mookleemas, P. OAC0103

Moon, J.R. PESAF10

Moore, C. OAC0405

Moore, D. OAD0302

Moore, R.D. PESAB02

Moore, S. OAD0703

Moorhouse, L. PESAD02

Moosa, Y. OAC0202

Morais, G.A.S. OAE0302

Moraleda, C. EPLBB06

Moran‐Garcia, N. PEMOA38

Moreira Gabriel, E. PESAA07

Moreno Fornes, S. OAC0402

Moreno, A. PEMOA32

Morgan, T. OAA0403

Morgello, S. OAA0303

Morhason‐Bello, I. PESAC09

Mori, L. PESAA04

Morin, S. OAE0403

Morris, K. EPLBB04

Morrison, C. OAD0705

Morrow, M. PESAB03

Morton, J. OAC0504

Morton, Z.P. PESAB13

Mosepele, M. PESAB06

Moses, A. PESAE09

Moshashane, N. PESAB11

Mota, M.T.d.O. PESAC02

Motala, A. PESUD50

Motsamai, S. OAE0203

Motsumi, K. PEMOB32

Motta, I. OAB0402

Moucheraud, C. PESAE09

Mouquet, H. PEMOA35

Moye, J. OALBB0102

Moyle, G. EPLBB04

Moyo, B. OAC0102

Moyo, N. PEMOC42

Moyo, P. PESAD18, PESAD22

Moyo, S. PEMOB32

Mpagi, D. OAD0603

Mpendo, J. OALBX0107

Mpofu, N. EPLBE07

Mposula, H. PEMOC49

Mpoudi Ngole, E. PEMOA34

Mpoudi‐Etame, M. OAB0304

Msekandiana, A. OAB0203

Msolomba, V. EPLBC10

Msuya, M. OAE0203

Mtesha, B. EPLBD01

Mthabela, N. EPLBA08, PEMOA31

Mtimuni, A. EPLBB03

Muñoz‐Fernández, M.Á. PEMOA27

Mubiru, A. OAC0102

Muchara, A. PESAD18

Muchemwa, C. PESAD22

Muchoka, M. PESUC26

Mudaliar, M.S. OAF0202

Muddu, M. OAE0503

Mudenda, M. OALBC0105

Mudvari, P. OALBX0102

Mudzviti, T. PESUB17

Muema, D. PEMOA31

Mugabe, A. PESAD17

Mugabe, D. PEMOC46

Mugabe, F. OAE0503

Mugao, N. EPLBF04

Mugo, C. PESAC05

Mugo, N.R. OAE0105

Mugocha, C. PEMOC49

Mugoma, W.E. OALBE0102

Mugundu, P. PESAE05

Muhairwe, J. OAC0304

Muhammad, A. PEMOD72, PESUE31

Muhangi, J. EPLBE01

Muhindo, B. PEMOD59

Mujugira, A. PEMOE41

Mujuru, H.A. EPLBB06

Mukasa, B. PESAE10

Mukherjee, J. PEMOD69

Mukhopadhyay, S. PESAA08

Mukose, A. PEMOC42

Mukundane, A. OAD0105

Mukuru, M. PESAE07

Mukwekwerere, P. OALBX0107

Mulamba, J. PESAE16

Mulang, R. PEMOC44

Mulenga, L. OALBB0105, OALBC0105, PEMOB28

Mulenga, P. PESUE23

Muleya, C. PESUC26

Muleya, R.M. PESUC27

Mullick, S. PEMOC34

Mulucha, J. OAF0302

Mulumba, N. PEMOB33, PEMOD63

Mulwanda, W. OAD0604

Mumbiro, V. EPLBB06

Munagala, V.R. PESAE05

Munatsi, V. PESAD16

Mundingi, R. PESAD18

Mungati, M. PEMOD55

Munsongo, C. PESUC26

Munthali, G. PEMOC42

Munyayi, F. PEMOC44

Murandu, M. PEMOD55

Murape, T. PESUE27

Murciano‐Anton, M.A. PEMOA28

Muronga, D. PEMOC44

Murphy, D. PESUD41

Murray, M.C. EPLBB02

Murray, S. OAD0504, OALBD0105

Murry, J. OALBA0103

Murthy, S. PESUB15

Murungu, J. OAD0402, PESAD18, PESAD22

Muruti, R. PEMOC44

Museka, T. PESAD02

Mushangwe, B. PESAD22

Mushavi, A. PEMOD55, PESUE27

Mushayanembwa, P. PESAB07

Musinguzi, J. OAD0105

Musonda, B. PESUC26

Musonda, M. PESAD19

Musonza, A. PESAD18

Mutabazi, T. OAE0102

Mutale, J. OALBC0105

Mutasa, K. PESAB07

Mutembo, S. PEMOC42

Muthoka, K.J. EPLBE09

Muthui, A. PESUF17

Mutima, J.M. EPLBD07

Mutungi, G. OAE0503

Muturi‐Kioi, V. OAE0303

Muula, A.S. OAC0405

Muwonge, T.R. PEMOE41

Muyunda, B. OAC0305

Muyunga‐Mukasa, T. EPLBF05

Mvinjelwa, P. PEMOC49

Mwakala, M.N. EPLBE09

Mwamba, D. OALBC0105

Mwandumba, H. PESAB06

Mwango, L. PESUC26

Mwangwa, F. OAB0403, OALBE0102

Mwansa, M. OAE0104, OALBC0105

Mweebo, K. OALBC0105

Mweemba, A. PEMOB28

Mwenechanya, M.M. OAE0104

Mwesezi, H. PEMOC40

Mwila, A. PESUC26

Mwimanzi, F. PEMOA38

Mwiya, M. PEMOC42

Mworeko, L. PEMOD58

### N

Nabaggala, G. PESAB11

Nabalwanyi, Z. PESAD06

Nabimanya, H. PEMOD59

Nabuduwa, A. PEMOC42

Nabulumba, N. PESAD11

Nachipo, B. EPLBE04

Nagarajan, S. OAB0404

Nagel, M. OALBA0103

Naggie, S. PESAB13

Nahirya Ntege, P. OALBX0107

Naicker, N. EPLBA02

Naidoo, N. PEMOC34

Naidoo, S. PESAA11

Naidoo, V. EPLBB07

Naiga, Z. PEMOD66

Naitore, D. PEMOD60

Naiwatanakul, T. OAC0103, PESUC22

Najjeke, R. PEMOD66

Nakabiito, C. OALBX0107

Nakabugo, L. PEMOE41

Nakato, H. OALBE0102

Nakaweesi, J. PESAE10

Nakawooya, H. EPLBC08

Nakazzi, J. PESAD17

Nakigozi, G. PESAE16

Nakigudde, J. OALBE0102

Nakyanjo, N. PESAE16

Nalugoda, F. EPLBC08

Naluwooza, R. PESAD06

Namaganda Aleserwe Hope, S. PESAE10

Namanda, C. PEMOC42

Nambi, F. PEMOE41

Nampalli, N. OAF0202

Nampungu, J. OAD0603

Namugenyi, C. OAE0503

Namukwaya, S. PESAD06

Namuziya, N. EPLBB06

Nana Poku, F. OAF0405

Nana, C. PEMOA34

Nana, M. PEMOA34

Nance, R. PESUD30

Nandago, C. EPLBE01

Nandagopal, P. PEMOC41

Naniche, D. EPLBB07

Napravnik, S. PESAB02

Narayan, P. PEMOC41

Narayanan, E. OAA0204

Narr, K.L. OAB0204

Nash, D. OAE0502

Nasser, M. OALBX0103

Natarajan, H. PELBA01

Nathoo, K.J. EPLBB06

Nawina, C. OALBF0105

Nazli, A. PESAA05

Nazziwa, E. PEMOC42

Ncayiyana, J. EPLBD02

Nchabeleng, M. EPLBA02

Ncube, G. EPLBE04

Ncube, J. PESAD18

Ndanga, C. EPLBF06

Ndayizigiye, M. OAE0203

Ndede, K. PEMOD60

Ndeloa, C. OAD0603

Ndlovu, N. OAC0504

Ndlovu, P. PESAD18

Ndlovu, S. PESUC19

Ndubani, P. PEMOC42

Ndugile, Y. PEMOE42

Ndukwe, D. OAE0103

Nduna, B. EPLBB06

Ndung'u, T. OAA0203, PEMOA31

Ndyanabo, A. EPLBC08

Necsoi, C. PESUA17

Needham, J. OALBD0102

Negumbo, E. OALBE0104

Neilands, T. OAD0804

Neilands, T.B. PESUD39

Neja, M. OAA0404

Nekkalapudi, A. OALBA0103

Nekorchuk, M. PESUA24

Nel, K. EPLBB07

Nelson, C. PEMOC47

Nelson, C.M. OAB0102

Nelson, L. EPLBC08

Nelson, L.J. PEMOC40

Nelson, R. PESUD45

Neri, S. OALBE0104

Nestola, L. PESUA16, PESUA17

Netea, M.G. EPLBA07, PEMOB32

Nethra Raju, G. PEMOD70

Netto, E.M. OAB0305

Neukom, J. PESUD26

Neuman, M. PEMOE36

Newman, P. PELBD01

Newman, P.A. OAD0203, PESUD45

Ng'ang'a, L. EPLBE09

Ng, B.C. PESUD42, PESUD47

Ng, K. PEMOA38

Ngari, F. EPLBE09

Ngassaki‐Yoka, C.‐D. OAA0103

Ngidi, J. PELBC01

Ngo Thuy, N. OAE0505

Ngoepe, A. PEMOA31

Ngokere, A. PESAB05

Ngosa, L. PESUD46

Ngowi, K. EPLBD01

Ngozo, J. EPLBE08

Ngugi, M. OAF0404

Ngumbau, N. OAC0502

Ngure, K. OAE0105, PEMOC49, PESAE02

Nguyen Anh, P. OAE0505

Nguyen Trong, K. OAE0505

Nguyen, A.L. OAD0403

Nguyen, J. PESUD30

Nguyen, N. OAD0705

Nguyen, Q. OAC0102

Nguywen, D. OAB0202

Nhamo, D. EPLBF07

Nhampossa, T. OAB0205

Ni, Y. PESUD29

Nibogora, B.D. OAD0604

Nicholls, E.J. PEMOC43

Nichols, B. PESUE19, PESUE23

Nichols, B.E. OAE0104, OAE0304

Nichols, S. PESUA19

Nicholson, V. EPLBB02

Nielsen‐Saines, K. PESAB01

Niikura, M. PEMOA38

Nijhawan, A. PESAB02

Nijs, E. OAA0402

Ning, H. PESUD35

Nininahazwe, C. PESUF17

Nishimoto, L. PESAE11

Nishimura, H. PESAE16

Nishizawa, M. PESAC17

Nitido, A. PELBA01

Njau, P.F. PEMOE42

Njengwa, R. PEMOC44

Njogu, V. PEMOF25

Njonkep, C. OAB0304

Nkomana, N. PEMOC37

Nkomo, B. OAC0205, PELBC01

Nkonge, I. PEMOD59

Nkundanyirazo, P. OAE0203

Nliwasa, M. OALBB0103

Nobleman, R. PESAF02

Noguchi, L. EPLBC05

Noguera, M. OAA0205

Nonyane, B.A. EPLBE02

Nookhai, S. OAC0103

Noppe, Y. OAA0402

Nordstrom, J.L. OAA0403

Norouzi, V. PESAA02

Northbrook, S. OAC0103, PESUC22

Norwood, A. OALBD0104

Nsenga, M. PEMOC42

Nsoh, M. PESAE11

Nsonde, D. OAE0502

Nsubuga ‐Nyombi, T. PESAE10

Ntjikelane, V. PESUE23

Ntlamelle, L. OAE0203

Nugent, J. OAB0403

Nugroho, A. PESUE26

Nur, M. OALBE0104

Nurhidayat, Y. PESUE26

Nuwagaba‐Biribonwoha, H. OALBC0102, OALBX0107

Nwabueze, D.E. PEMOB30

Nwafor, C. OALBE0105

Nwaokenneya, P. PESAB04

Nwaozuru, U. PEMOF32

Nwi‐ue, J. OALBE0105

Nwizu, J. OAE0402

Nwokolo, N. PESUB25

Nworie, K. PESAD23

Nyabuti, M. OALBE0102

Nyagani, D. PESUE31

Nyakato, J. EPLBE01

Nyakato, P. OAC0304

Nyakuwa, S. PESAD18, PESAD22

Nyambura, C. OAF0305

Nyamhuno, S. PESUE25

Nyamukapa, C. PESAD02

Nyang'wa, B.‐T. OAB0402

Nyangulu, M. OAC0305

Nyariki, E. OAD0305

Nyarko, T. PEMOD54

Nyasulu, I. OAC0405

Nyathi, M. PEMOE35

Nyaude, S. EPLBE04

Nyawasha, T. OAF0104

Nyazema, L. EPLBE04

Nyimbili, S. OALBC0105

Nyirenda, A. PESAC03

Nyirenda, R. OAC0305, OAC0405, OALBB0103

Nyirenda, S. OALBB0103

Nyokabi, J. OAE0105

Nyokani, B. EPLBD08

Nzarubara, B. OALBE0102

Nzima, V. PESAE11

Nzoutchoum, O. OAC0102

### O

O'Bryan, G. PEMOC44

O'Connor, N. OAF0302

O'Doherty, U. PESUA18

O'Malley, G. OAC0405

O'Malley, J. PESAF12

Obiefuna, A. OALBF0105

Obiezu‐Umeh, C. PEMOF32

Obiri‐Yeboah, D. PESAC09

Ocasio, M. PESAB01

Oceguera, A. PESUA18

Ocen, L. EPLBE01

Ochieng, B. OAC0502

Ochillo, M. EPLBC03

Ochinokwu, P. PEMOB30

Ochira, P. EPLBD08

Ochola, C. OAD0102

Ochs, A. OAD0602

Odeny, L. EPLBC03

Odero, L. EPLBE09

Odhiambo, A. PEMOF29

Odondi, J. PEMOD60

Odong, S. OAD0802, PESUD38

Odoyo, J. PESAE02

Oeffinger, J. OALBD0104

Offenbecker, K. PESUB24

Ofotokun, I. PESAB13

Ogbuabor, D. PESUE22

Ogeta, A. OAF0302

Ogollah, F. OAC0305

Ogunleye, A. PEMOF29

Ogunsanya, A. OAD0603

Ogunsanya, P. OAD0603

Ohazurike, C. OAE0402

Ohorodnik, S. PELBE01

Ohrnberger, J. PEMOC36

Oka, S. PESAB10

Okeke, B. OAE0205

Okello, F. PEMOC42

Okey, J.U. PEMOB30

Okeyo, N. EPLBC03

Okiria, A.G. EPLBD08

Okiwu, H. PEMOD72, PESUE31

Okocha, B. PEMOB34

Okochi, H. PESAB03

Okoli, C. PESUB21, PESUB25

Okon, R. PEMOB30

Okoye, J. PESAB05

Oktariani, A. OAF0305

Okui, L. PELBC01

Okuku, J. PESUC26

Okumu, E.A. PESUD31

Okumu, M. OAD0802, PESUD38

Okumu, O. EPLBC03

Olabanjo, O. PEMOC39

Olabode‐Dada, S. PESAD08

Oladele, R. PESAB04

Olansky, E. OAC0303

Olatosi, B. PESUD35

Olete, R. PEMOE46

Olinga, J. OAB0304

Oliveira da Silva, L. PESAD10

Oliveira, M. OAC0105

Oliver, B. PESAA13

Oliver, D. PEMOC42

Olivier, J. PEMOE34

Olivier, S. OAC0202

Olorunfemi, G. PESAB04

Olowski, P. PESUC26

Olowu, A. OAE0103

Olujimi, A. OALBD0104

Oluwatoyin Folayan, M. PEMOD72, PESUE31

Omar, F.L. OALBA0102

Omar, Z. EPLBB01

Omgba Bassega, P. OAB0304

Omoighe, S. OAE0103

Omollo, K. PESUA15

Omollo, V. PESAE02

Omom, C. PESAD06

Omondi, D. PESAD06

Omondi, F.H. PEMOA38

Omondi, I. OALBF0104

Omondi, M. PESAD08

Omondi, P. OAC0502

Omony, J. OAC0504

Omoro, G. EPLBE09

Omosa‐Manyonyi, G. PESAD06

Omotuyi, O. PESAB05

Oniyire, A. OAE0103

Onoya, D. PESAE06, PESUD40

Onwuatuelo, I. PEMOC39

Onwujekwe, O. PESUE22

Onyadile, E. PELBC01

Onyango, J. EPLBC03

Onyekwum, C. PESAD23

Onyenekwe, C. PESAB05

Onyezue, O. PEMOC39

Opel, F. OALBE0102

Opinde, C.A. EPLBF04

Opira, B. PESAB08

Oppong, S. PESUD27

Orach, S. EPLBE01

Oraro‐Lawrence, T. PESUD50

Orellano, G. PEMOD67

Orkin, M. OAD0503

Orlinger, K. OALBA0103

Ormsby, N. PESUD31

Orrell, C. EPLBB08

Ortblad, K.F. OAE0105, PESAE02

Ortiz, Z. OAB0303

Osi‐Samuels, J. PEMOC39

Østergaard, L.J. PEMOA26

Osuji, J. PEMOF29

Otani, M. PESAC17

Otchere‐Darko, J. PEMOE34

Oto‐Obong, N. PEMOB30

Otubu, N. PESAB04

Otwombe, K. OAB0205, PEMOE33

Ouma, C. OALBF0104

Ouma, J. OAD0605, PESUD33

Over, M. OAE0406

Owaraganise, A. OAB0403

Owens, T. PESAE02

Owino, M. OALBD0102

Owor, M. EPLBC05

Owuoth, J. PEMOB27

Owusu, E. PESUE24

Owusu, M. PESUE24

Owusu, S.E. PEMOD61

Oyugi, J. PESUA15

### P

Péré, H. PEMOA35

Pérez‐Elías, M.J. PEMOA32

Pérez‐Olmeda, M. PESUA23

Pachankis, J. PELBD02

Pacheco de Freitas, M. EPLBE05

Packel, L. PEMOE42

Paez, C. PELBA02, PESUA14

Pagliuzza, A. OAA0105, OAA0302

Pahwa, R. OAB0102

Pahwa, S. OAB0102

Paiola, S. PESAB01

Palanee‐Phillips, T. PEMOC37

Palaparthy, R. PESUB22, PESUB23

Palella, F. PEMOC48

Palella, F.J. PESAB13

Pallikkuth, S. OAB0102

Panagiotoglou, D. OAD0505

Panchia, R. OALBX0107

Pandey, U. EPLBA06

Pandya, A. PESUC21

Pang, D. EPLBB02

Pang, J. PESUF15

Paniconi, M. PESAA09

Panis, N.K. PEMOB35

Panneh, M. OAD0305

Pantophlet, R. PEMOA38

Paone, J. PEMOC33

Pape, J.W. EPLBE03

Pape, W. OAE0502

Paquette, D. PEMOD74

Parcesepe, A. OAE0502

Pardons, M. OAA0402

Paredes, R. OAA0205

Parekh, B. OAC0102

Paris, K. PEMOC42

Park, B. PEMOE45

Park, S. PESUB25

Parkes‐Ratanshi, R. OAE0102

Parkpien, P. OAC0503

Parsons, D. PELBA01

Parsons, J.A. PESUD44

Pascoe, S. PESUE19, PESUE23

Pashchuk, O. OAF0205

Pasipamire, M. OAC0102

Pasquale, H. EPLBD08

Passanduca, A. EPLBB06

Pasternak, A.O. PESUA17

Pastore, M. PESUB14

Pasupula, J.R. OAF0202

Patel, D. PEMOC33, PESUE20

Patel, P. PEMOB29

Patel, R. PESUB25

Patel, V.V. OAB0103, PESUD34

Pati, R. OAE0504

Patnaik, K. OAF0202

Paton, M. PESUF16

Pattacini, L. OALBA0102

Pattarapayoon, N. OAC0103

Patterson, P. OAB0303

Patterson, T.L. PESAD24

Pattery, T. OAE0102

Patton, M.E. PEMOE34

Pau, M.G. OALBA0102

Paul, H. PESAF07

Paul, M. OAB0104

Paulin, H. PEMOE34

Paulussen, R. EPLBB07

Paximadis, M. PESAC16

Payne, D. OAC0102, OAC0305

Peabody, R. PEMOC43

Pearce, J. PESUD30

Pearson, J. OAD0103

Peay, H.L. PESUD31

Peck, R. PESAF01, PESAF02

Peeters, M. OAB0304

Pelaez, S. OALBD0102

Pelletier‐Marcotte, L. PESAF01

Pelloquin, R. OAB0304

Peng, J. OALBE0102

Pengnonyang, S. PELBD01

Penner, M.C. PESAE14

Penzhorn, Z. PESAD20

Peppa, D. PEMOA33

Pereira Bittencourt Passaes, C. PESUA17

Pereira, M. PEMOB37

Perez, C.F. PEMOB35

Perini, F.d.B. EPLBE05

Perkins, R. OALBX0103

Perlowski, C. OALBB0102

Permar, S.R. OAE0303

Persaud, A.T. PESAA02

Persaud, D. OALBB0102, PESUA19

Pescarini, J. OAE0302

Pessoa Vilela, A.P. PEMOA35

Peters, M. OAB0405

Petersen, M. OAB0403

Pham, T. PESUF17

Phanuphak, N. OAC0503, OAF0204, PELBD01, PESAA10, PESUC32

Phanuphak, P. OAC0503, PESUC32

Phelps, M. PELBA01

Philbin, M.M. OAF0204

Phillips, A.N. PEMOC43

Phillips, P. PESAB08

Philpott, S. PESUD41

Phiri, B. OAE0104, PESUE23

Phiri, C. OALBC0105

Phiri, G. PEMOB28

Phiri, K. PESAE09

Phiri, M. OAE0202

Phiri, S. PESAE09

Phiri, S.J OAC0304

Piñeirúa‐Menendez, A. PEMOE39

Pichon, L. EPLBD03

Pickles, M. PEMOC36, PESAC09

Pietersen, I. OAC0102

Pillinger, M. PESAF10

Pilusa, M. OAF0104

Pimenta, C. PESAE12

Pina, I.L. PESUB15

Pincino, R. PESUB19

Pindiwe, B. EPLBE04

Pinini, Z. OAF0104, PEMOE34

Pinto, G. OAB0305

Pinto, R.M. OAD0803

Pintye, J. OAC0502

Pinzone, M. PESUA18

Piper, J. EPLBC05

Pipkin, M. PESAA04

Pitsillides, P. PEMOD53

Piwowar‐Manning, E. OALBX0107, PESUA14

Plagianos, M.G. PESAA08

Plana, M. OAA0205

Planas, D. PEMOA35

Planchais, C. PEMOA35

Planelles, V. PEMOA28

Plankey, M.W. PEMOC45

Plant, E. PESUA17

Platt, L. PESUC18

Plax, K. OAD0602

Pleaner, M. PEMOC34

Pliakas, T. PEMOC36

Podmore, M. PESUF17

Podolchak, N. EPLBC02

Poku, F.N. PESUD27

Polakowski, L. PESUA14

Pollard, R. PESAE05, PESAE16

Polonijo, A.N. OAD0403

Polyak, C. PEMOB27

Ponda, F. PEMOC46

Ponde, T. PESAD01

Poonkasetwattana, M. PEMOE46

Poopola, D.V. PEMOB30

Poovan, N. OAC0504

Porrot, F. PEMOA35

Porter, J. EPLBA06

Post, F. EPLBB04

Poteat, T. OAC0403

Poudrier, J. PESAA09

Pounds, C. PEMOC42

Povshedna, T. EPLBB02

Powell, J. OAA0303

Powell, M. PESUD30

Powis, K.M. PEMOB32

Pozniak, A. EPLBB04

Prévost, J. OAA0405, PESUA22

Prabakaran, K. EPLBD05

Prachniak‐Rincón, C. PESUF19

Prasad, V.S. EPLBF03, PEMOF34

Prasanna Viswanath, K. PEMOF34

Prasanna, K. EPLBF03

Prasher, A. PEMOA29

Prazuck, T. PEMOA35

Preau, M. EPLBD06

Prendergast, A.J. PESAB07

Presanis, A. OAC0302

Prestage, G. PESUD41

Price, Y. OAD0204, PEMOD65

Prieto, M. PEMOE37

Prins, M. PEMOC35, PESAC12

Probert, W. PEMOC36

Prodger, J. OAA0303, PESUA20

Prueksakaew, P. PESAA10

Prusente, S. PEMOE34

Pry, J. OALBC0105

Prystajecky, N. PEMOA38

Przybylek, S. PESUC28

Psaros, C. EPLBC04

Pujari, R. PEMOD64

Pulerwitz, J. PESUD28

Purswani, M. PESUA19

Puspitasari, N. PESAF04

Puthanakit, T. OAA0105, OAA0302

Puyat, J. EPLBC09, OAB0105

### Q

Qaisar, M.N. PESAD03

Qaragholi, N. PESUC29

Qavi, A. PELBB01

Qian, H. OALBD0102

Qiao, S. PESUD46

Qiu, Z. EPLBB09

Quaife, M. PESAD06

Quaitey, F. OAE0103

Quarles, R.C. PESAE14

Quesnel‐Vallée, A. PESAD14

Quinlivan, D. OAE0204

Quinn, T. EPLBC08, PESUA20

Quintal, M.‐C. PESAA09

Quintana, F. OALBX0102

### R

Rönn, M. PESAC09

Rabie, L. PEMOD52

Rabkin, C.S. PESAB02

Rachas, A. PESUC20

Rachynska, V. EPLBF01, PESAF06

Radix, A. OAC0403

Radusky, P. PEMOD67

Raffi, F. EPLBB04

Raghavan, S.S. OAF0202

Rahayu, S. OALBC0104

Rahim, S. PESAF01

Rahman, D.L. PESUE18

Rahman, S. OALBX0105

Raiford, J. EPLBC06

Raizes, E. OAE0504, PEMOE34

Rajasuriar, R. PEMOA36

Ralayo, N. OAD0204, PEMOD65

Ramírez‐García, A. PESUA23

Rama, D. EPLBE04

Ramaabya, D. PELBC01

Ramachandran, S. PEMOE40

Ramakrishnan, L. OAF0202

Ramautarsing, R. OAC0503

Ramautarsing, R.A. PESUC32

Rambally Greener, L. PEMOD53

Rambau, N. EPLBE07, PEMOE35, PESUF20

Ramjit, D. EPLBA08

Ramoran, E.G. PESUD26

Ramos Martín, F. PESUA25

Ramos‐Martin, F. PEMOA28

Randhawa, A.K. EPLBA02

Ranebennur, V. PEMOE38

Rao, A. EPLBA03, EPLBC01

Rasella, D. OAE0302

Rashbass, J. PEMOF31

Rathakrishnan, D. PESAB04

Rauch, A. PESAC12

Rawat, S. OAD0203, PELBD01, PEMOD64, PEMOD69, PESUD45

Rawlings, L. PEMOC47

Raymond Marchand, L. OAA0103

Redd, A. OAA0303

Redd, A.D. PESUA20

Reddy, K. OAA0203, PEMOC37

Reddy, L. PEMOD70

Reddy, S. OAB0103, PESUD34

Regenold, S. PELBA02

Reggee, S. OAF0302

Rehman, A.M. OAB0204

Reid, R. PESUD25

Reirden, D. OAB0104

Reisner, S.L. OAC0403

Reiss, P. PESAE04

Remch, M. OAE0502

Rendina, J. PESUD34

Rendon, I.Q. PEMOE46

Rennie, S. PEMOF32

Renzi, I. OAA0204

Resino, S. PESAA03

Reveiz, L. OAB0404

Rey, D. PEMOA37

Reyes Díaz, M. PEMOD71

Reyes‐Urueña, J. OAC0402

Reynes, C. PESUB14

Reynes, J. OAB0304, PESUB14

Reynolds, S. PESUA20

Reynolds, S.J. EPLBC08

Rhee, M. PESUB22, PESUB23

Rice, B. PESUC18

Rich, K. PESUC21

Richard *, J. PESUA22

Richard, C. OAA0105, OAA0302

Richard, J. OAA0405

Richardson, B. EPLBC05

Richardson, P. EPLBC04

Richman, D. PESUA19

Riegel, L. EPLBD07, PEMOD67, PEMOD71, PEMOD73

Rinehart, A. EPLBC04, OALBX0107

Rivadeneira, E. PEMOC42, PEMOD55

Rivona, B. OAF0305

Riyadi, F. PESUE26

Rizarri, R. PESUE29

Robbins, R.N. OAD0705

Roberts, K. PEMOB31

Roberts, S.T. PEMOC49

Robin, E.G. PESUC31

Roche, M. OAA0304, PESUA24

Rochelle, A. PELBD02

Rodger, A.J. PEMOC43

Rodgers, J. PESAE01

Rodríguez Mora, S. PESUA25

Rodrigues, W. PESAB03

Rodriguez Sherman, H. PESUF24

Rodriguez‐Cruz, A. PESAD14

Rodriguez‐Mora, S. PEMOA28

Rodriguez, A. OAB0102

Rodriguez, A.E. OAC0403

Rodriguez, C. OAF0305

Rodriguez, E. PEMOD75

Roederer, M. EPLBA04, OAA0404

Roger, M. OAC0105, PESAA09

Rogers, D. OAA0204

Rohr, O. PESUA16, PESUA17

Rojas Castro, D. EPLBD07, PEMOD67, PEMOD71, PEMOD73

Rojo, P. EPLBB06, OAB0205

Rolon, M.J. OAB0303

Roman, N. OAC0505

Romero, M. PEMOD67

Romio, S. OAD0203

Romney, M.G. PEMOA38

Rooney, J. EPLBC04

Rooney, J.R. OALBX0107

Roos, A. OAB0204

Rose, S. OALBX0107

Rosen, J.G. OAF0403, PEMOD56

Rosen, S. OAE0104, PESAE06, PESUE19, PESUE23

Rosenberg, N.E. PELBC02

Rosenes, R. PEMOB34

Rosenfield, J. OAE0504

Ross, J. OAB0103, OAE0502, OAF0204, OALBB0104, PESUD34

Rossi, P. OAB0205

Roucoux, G. EPLBD06

Rougier, E. PEMOA37

Roungprakhon, S. PELBD01

Rouquette, C. OAD0404

Rourke, S.B. PESUD44

Routy, J.‐P. OAA0103, OAA0202, OAC0105, PESAA07, PESAA09, PESAD14

Rouzioux, C. PESUA17

Rowan, D. PESAF05

Rowe, J. PESAF03

Rozelle, M. PESUD50

Ruark, A. OAD0804, PESUD39

Rubeni, M. OALBE0104

Rubio, F. PESUE29

Ruch, Y. PEMOA37

Rucinski, K. PEMOD63

Ruderman, S. PESUD30

Rudisill, C.A. PESUF16

Rudnicki, E. EPLBB08

Rueannak, J. OAC0503

Ruel, T. OAB0403, OALBE0102

Rufurwadzo, T. PEMOF26, PESUD33

Ruiz, E. OAD0403

Rukobo, S. PESAB07

Rukundo, G.Z. OAD0805

Rusconi, S. EPLBB04

Russell, A. EPLBE07

Russell, C. OAE0304

Russo, L.V. OAF0304

Ruth, S. PESUE28

Rutsaert, S. OAA0402

Rutter, L. PEMOE35, PESUF20

Ruzibe, S. PESAD18

Ryan, P. PESAA03

### S

Sáez‐Cirión, A. PESUA17

Sánchez‐Cerrillo, I. PEMOA27

Sánchez‐Madrid, F. PEMOA27

Sánchez‐Palomino, S. OAA0205

Søgaard, O.S. PEMOA26

S, A. PEMOD70

Saal, W. OAD0702, PESAC15

Sabasaba, A. PEMOE42

Sabin, K. PEMOD72, PESUC18

Sabounchi, N. PESAC14

Sacdalan, C. PESAA10

Sacha, J. OAA0404

Sachs, D. OAA0303

Sackitey, R. OAF0405

Sacks‐Davis, R. PESAC12

Sadamasu, K. PESAB10

Sadate‐Ngatchou, P. PESAE11

Sadik Shaik, J. PESUB24

Saez‐Cirion, A. PESUA16

Saez, A.J. PESUA25

Safren, S. EPLBC04, OAB0103, PEMOD75

Safren, S.A. PESUD34

Safrit, J. EPLBA04

Safrit, J.T. OAA0404

Sagaon‐Teyssier, L. EPLBD07, PEMOD73

Saha, P. PEMOD69

Saiwaew, S. PESUC32

Salcedo, M. OAD0404

Salit, I. PEMOB34

Salnikov, S. PELBE01

Sam‐Agudu, N.A. PELBC02

Samandari, T. OALBC0102

Sambai, B. PESAC06

Samitpol, K. EPLBC04, OAC0503

Samji, H. EPLBC09, OALBD0102

Sana Ullah, M.F. PESUF22

Sanche, S. PESUC23

Sanchez, A. PEMOF26

Sanchez, M. OAB0303

Sanchez, N. EPLBC04

Sanchez, T. EPLBC06, OAD0304, OAD0504, OALBD0105

Sandoval Figueroa, C. PEMOD71

Sandoval, M.M. PEMOB35

Sandset, T.J. PESUF17

Sang, E. PESAC04

Sang, Y. PEMOA38

Sangma, L.R. PEMOC41

Sangtong, S. PESUC32

Sannier, G. PESUA22

Santangelo, M. PESUA17

Santos, C.A.S.T. OAE0302

Santos, E.L.B.d. PESAC02

Sanz, J. PEMOA27

Sarang, S. PESUC19

Sardinha, L. OAD0505

Sarwitri, S. PESUE26

Sarzotti‐Kelsoe, M. EPLBA02

Sathane, I. PESUC18

Saunders, Y. EPLBB07

Saura‐Lázaro, A. EPLBB07

Sauve, L. OAC0204

Savory, T. OALBC0105

Sawangsinth, P. OAA0105, OAA0302

Sawant, S. EPLBA02, OALBA0102

Saxena, V. EPLBA03

Sazonova, I. PELBE01

Sazonova, Y. EPLBC02

Schechter, M. PESAC02

Scheckter, R. EPLBC05

Scheepers, M. EPLBB07

Schelling, E. OALBD0104

Schenkel, S. PEMOB32

Schim van der Loeff, M. PESAE04

Schleimann, M.H. PEMOA26

Schlosser, D. OAD0603

Schmid, P. OAB0302

Schmidt, S. OALBA0103

Schmitz, J.L. OAA0403

Schmitz, M. OAC0102

Schnarrs, P.W. OALBD0104

Schneeweiß, S. PESAC11

Schneider, J. OAC0403

Scholten, S. PESAC11

Schomaker, M. OAC0304

Schoof, N. OAA0404

Schrubbe, L. OAD0505

Schulha, S. OALBA0103

Schurman, E. PEMOE43

Schwab, J. OALBE0102

Schwabe, C. PESUB24

Schwartz, C. PESUA17

Schwartz, J. OAE0503

Schwartz, O. PEMOA35

Schwartz, S. EPLBE02, OAD0205, PEMOB33, PEMOD63, PESAE14

Schynkel, T. OAA0104

Scott, C. PEMOE34

Scott, H. OALBX0103

Scott, S. EPLBE06

Sculier, D. OAB0302

Seaton, K. PELBA02, PESUA14

Seaton, K.E. EPLBA02

Sebastian, A. OAD0203, PESUD45

Sebastiani, G. PESAD14, PESUB18

Sebayang, M. OALBF0105

Sebe, M. EPLBA02

Sebotsa, M. PESUF20

Sebra, R. OAA0303

Seeley, J. EPLBD09, OAD0305, PEMOE36, PESAB11

Seenappa, B. PEMOD70

Sekimpi, L. PEMOC40

Selato, R. PELBC01

Selzer, L. OALBA0105

Semchuk, N. OAF0205

Semitala, F. PESAB08

Semitala, F.C. OAE0503

Senapati, N. OAF0202

SenGupta, D. OALBA0105

Senior, L. OAA0304

Senyimba, C. PESAE10

Seo, Y. OAA0204

Sereda, P. PEMOA38

Serioni, E. PESAD17

Serrano Amaya, J.F. PESAF11

Serwadda, D. EPLBC08

Seshoka, L. OAF0104

Seth, K. PEMOD69

Setshogelo, B. PEMOE35

Seumko'o, R. PEMOA34

Seve, A. PEMOA35

Shabalala, S. PEMOD53

Shade, S. OALBE0102

Shadie, M.M. OAF0305

Shah, N. PEMOB27

Shah, P. OAD0305

Shah, S. PEMOF32

Shaharuddin, S. PEMOA36

Shahi, S. OAF0305

Shaik, N. PESUB23

Shakwelele, H. OAE0104, PESUE23

Shalek, A.K. EPLBA08

Shamu, T. PESUB17

Shanaube, K. OAC0104, PEMOC36

Shanley, A. OAE0204

Shannon, K. OAD0103

Shao, Y. OALBC0103

Shapiro, D. OAB0104

Shapiro, R. PELBB02

Sharma, A. EPLBC06, PESUD46

Sharma, B. OALBA0103

Sharma, R. PESAD12

Sharma, V. PESAA10

Sharp, A. EPLBE07, PEMOE35

Sharp, E. OAC0102

Shaw, A. PESUF21, PESUF23

Shebl, F. PESUC21

Shenvi, N. PEMOD51

Sherembei, D. EPLBF01

Sherr, L. OAD0503, PEMOB31, PEMOC47, PESUB16

Sherwood, J. OAF0302, PESUF20

Shet, A. PEMOD70

Sheth, A.N. PESAB13

Shi, F. PESUD35

Shiino, T. PESAC17

Shikuku, J. PESAD06

Shilomboleni, M. OALBE0104

Shin, J.G. PESAB09

Shinkai, T. PESAB10

Shinners, C. PESAE14

Ship, H. OAB0405

Shipp, L. EPLBE02, PEMOB33, PEMOD63

Shirley‐Beavan, S. OAF0304

Shivakumar, K. OAF0202

Shoko, N. EPLBE04

Shook‐Sa, B.E. PELBC02

Shoyemi, E. PESUD28

Shrader, C.H. PEMOD75

Shrivastava, R. PEMOA29

Shrom, J. OALBE0102

Shublaq, D. PEMOD74

Shumba, K. PESAE06

Shumskaya, N. PESAD24

Shunmugam, M. OAD0203, PESUD45

Sianyinda, C. OALBC0105

Siberry, G. OAE0504

Sibisi, P. PESUE31

Sibisi, S. PESUF20

Siddhant, G. OAD0203

Siedner, M. OAC0202

Siegel, M. PELBA02

Sievers, J. PESUB24

Siew, W.H. PESUF15

Sigal, A. PEMOA31

Sigal, M. PESUD30

Sigaloff, K. PESAE04

Sikazwe, I. OALBC0105

Silva, A.F. OAE0302

Silva, R. OAB0404

Silveira, M.d.S. EPLBE05

Silverberg, M.J. PESAB02

Silvestri, G. EPLBA04, OAA0404

Simmons, B. EPLBB01, PELBB01

Simms, V. PESUB16

Simoncini, G. PEMOC48

Simoni, J. PEMOE41

Simons, B. PEMOB28

Simons, J. PEMOA38

Simons, S. PESAF03

Simpasa, B. PESAD19

Sindimba, M. PEMOC44

Sineke, T. PESUD40

Singer, J. OAB0105, OAC0204, OALBD0102

Singh, A. EPLBA08, PESAE05

Singh, C. EPLBE02

Singh, D. OAD0203

Singh, N. EPLBA02, OALBX0107

Singh, P. OAA0303

Singh, R. PESUB22, PESUB23

Singh, U. OAC0202

Singh, V. EPLBA04

Singh, Y. EPLBC04, PEMOD64

Singoei, V. PEMOB27

Sinkala, T. OALBC0105

Sinkele, W. PESAC06

Sirait, A.I. OALBC0104, OALBE0103

Siregar, K.N. OALBC0104

Sirivongrangson, P. PESUC29

Sithole, B. PESAD09

Siu, G. EPLBD09

Sivasubramanian, M. PEMOD64

Sivile, S. OALBC0105, PEMOB28

Siwingwa, M. PEMOB28

Siziba, B. OALBX0107

Skakoon‐Sparling, S. OAD0302

Skalland, T. PESUA14

Skovdel, M. PESAD02

Skrzat‐Klapaczyńska, A. OALBF0103

Skvoretz, J. PEMOD75

Small, L. PESAD15

Smith‐Sreen, J. OAC0405

Smith, A. EPLBC06, OAC0303

Smith, C. PEMOA33, PEMOB31

Smith, D. OAE0404

Smith, D.E. PESUB21

Smith, D.K. OALBX0106

Smith, J. EPLBA06

Smith, L. OAD0304

Smith, L.R. PESAD24

Smith, M. OAA0303

Smith, S. OALBX0102

Sobhie Diaz, R. PESAC02

Sobieszczyk, M. PESUA14

Sobieszczyk, M.E. PELBA02

Soge, O. OALBX0103

Sohn, A.H. OAF0204

Sok, D. OAE0303

Sokhela, S. PELBB01

Solomon, S. PESAE05

Solomon, S.S. PEMOC41

Solt, L. OAA0103

Soltan, V. OALBF0105

Somwe, P. OALBC0105

Sonar, S. EPLBA03

Song, X. EPLBB09

Sophonphan, J. PESAB09

Sorge, J. PEMOD74

Soto‐Torres, L. EPLBB08, PEMOC49

Soto, J. OAA0303

Soudeyns, H. OAB0105

Sousa, B. PESUE17

Sousa, M.D.G. PESAC02

Souza, L.E. OAE0302

Sowale, O. PESAB04

Spadaccini, L. PEMOB35

Speakman, A. PEMOC43

Speckmaier, S. PEMOA38

Spector, S. PESUA19

Spelman, T. PESAC12

Spence, C. PESUC23

Spencer, B.H. OAD0705

Spiegel, H. OALBB0102, PESUA19

Spinelli, F. PESUB21

Spinelli, M. PESAB03

Spinner, C. PESAC11

Spire, B. EPLBD07, OAD0404

Spooner, E. OALBX0107

Sprague, L. PEMOF28

Spreen, W. PESUB24

Sprinz, E. PESAC02

Spudich, S. PESAA10, PESUA19

Srikrishnan, A.K. PEMOC41

Srimanus, P. OAC0503, OAF0204

Srinivasan, K. PEMOB37

Ssali, A. PESAB11

Ssegujja, E. PESAE07

Ssekajja, B. OAD0202

Ssekaynzi, B. OAB0403

Ssekubugu, R. EPLBC08

Ssemanda, J. PESAD11

Ssemata, J.L. OAE0102

Ssemmondo, E. OAB0403

Ssempijja, V. EPLBC08

Ssengooba, F. PESAE07

Ssenyonjo, R. OAE0503

Ssesaazi, P. OAE0102

Ssinabulya, I. OAE0503

Stöckl, H. OAD0505

Stackpool‐Moore, L. PESUD50

Stafford, K. PELBC01

Stafford, S. PESAD20

Stahmer, A. PESUD33

Stan, R. OALBB0104

Stanfield‐Oakley, S. OALBA0102

Stankevitz, K. PEMOE38

Stannah, J. PESUD36

Staropoli, I. PEMOA35

Stecker, C. PEMOC42, PEMOD55

Steen, A. PEMOE43

Steffen, G. PESAC11

Steffy, T. OAB0202, OAB0203

Stein, D.J. OAB0204

Steinmetz, S. PESAB08

Stephan, C. EPLBB04

Stephens, J. PESAE08

Stephenson, K. PELBA02

Stephenson, R. EPLBC06

Stern, J. OAC0502

Stevens, O. PESUC18

Stevens, W.S. PESUC19

Stevenson, E. OAA0305, PESAA12

Stevenson, M. OAC0403

Stewart‐Jones, G. OAA0204

Stewart, A. PESAC12

Steyn, J. PEMOA31

Stieh, D.J. OALBA0102

Stillson, C. OALBE0105

Stjuste, M. PESUD25

Stockton, M. OAE0502

Stoeckle, M. OAB0302

Stoehr, A. PESAC11

Stoler, J. PEMOD75

Stone, E.M. OAF0403

Stoové, M. PESAC13

Stoove, M. PESAC12

Storey, A. OALBE0105

Storrow, L. PESAF05

Stover, C. PESUA21

Stover, J. OAE0406

Strachan, M. OAE0103

Straitz, S. PEMOE47

Stranix‐Chibanda, L. OAE0303, PELBC02

Strathdee, S. OAD0304

Stratton, T. PESAF03

Streeck, H. PESAC11

Strehlau, R. PESAC16

Streinu‐Cercel, A. PELBD02

Strong, C. PESUC30

Strub, S. PELBF01

Strumpf, A. PEMOE43, PESAC10

Stuart‐Brown, A. PESUC18

Stubbs, L. PEMOE47

Sturny‐Leclere, A. PESAB06

Su, L. EPLBA01

Subeliani, D. OAF0105

Subramoney, S. OAB0204

Sudderuddin, H. PEMOA38

Sued, O. OAB0303, PEMOB35

Sugiura, W. PESAC17

Sukkestad, S. OAA0404

Sullivan, P. EPLBC04, EPLBC06

Sullivan, P.S. OALBX0106

Sullivan, S. EPLBC06

Sumari‐de Boer1, M. EPLBD01

Sun, L. OALBC0103

Sun, N. OAF0103, PESUF23

Sun, R. EPLBE03

Sun, X. PESUD35

Sunday, A. PEMOD72, PESUE31

Sung, J. OALBA0103

Suntarattiwong, P. OAA0105, OAA0302

Suon, S. PESUD26

Supervie, V. PESUC20

Supparatpinyo, K. OALBX0105

Svisva, A. EPLBE04

Swai, I. EPLBD01

Swain, S. OAF0202

Swaminathan, V. PEMOC41

Swann, S. PEMOF35

Swann, S.A. EPLBB02

Swendeman, D. PESAB01

Syarif, O. PEMOF28

Syed Omar, S.F. PEMOA36

Sykes, C. PEMOA29

Szydlo, D. PEMOC49

### T

Taasi, G. PEMOC42

Taback‐Esra, R. PEMOE34

Tabengwa, M. OAF0404, PESAF12

Tac‐an, I. PESUE29

Tagarro, A. EPLBB06, OAB0205

Tagoe, H. PESUE24

Tahlil, K. PEMOF32

Taillefer, S. OAB0105

Takalani, A. EPLBB08

Takano, M. PESAB10

Takaya, S. PESUC23

Takuva, S. EPLBB08

Tally, L. OALBC0105

Talwar, S. EPLBC01

Tamundele, S. PESAF12

Tan, A. PESAD07

Tan, C. PESAD07

Tan, D.H.S. OAD0302, PEMOB34

Tan, L. PEMOB29

Tan, R.K.J. PESAD07, PESUD29, PESUD42, PESUD47

Tang, V.A. PESAA02

Tang, W. PESUD29

Tangmunkongvorakul, A. OAF0204

Tanno, S. PELBA01

Tanpradech, S. OAC0103

Tanser, F. OAC0202

Tanuma, J. OAE0502

Tao, L. PEMOC33

Tapley, A. OALBC0102

Taplitz, R. OALBB0104

Taramasso, L. PESUB19

Tarasuk, J. PEMOD74

Tartaglia, J. EPLBA02

Tasaneeyapan, T. OAC0103

Tasomboon, W. OAC0503

Tasya, I.A. OALBC0104

Tatenda Bhila, J. OAF0305

Tattersall, T. PEMOB34

Taulo, F. EPLBC05, OAC0405

Tavengwa, N.V. PESAB07

Taylor, D. PESUB24

Taylor, J. OAD0403

Taylor, T.N. PESAB13

Tedaldi, E. PEMOC48

Tedla, Y. OAC0102

Teleshova, N. PESAA08

Tembo, A. PELBB01

Tembo, K. PESUC26

Tembo, S. PESUC26

Tene, H. PEMOA34

Tenthani, L. OAC0305

Tenza, S. PEMOC37

Tepjan, S. PELBD01

Terrier, B. PEMOA35

Terry, S. OAA0305

Tesfay, N. PESUD37

Teti, M. EPLBD03

Tettey, S.‐s. EPLBE08

Thakar, M. EPLBA03

Thakker, J. PESAE05

Thammajaruk, N. PESUC32

Thaploo, S. OALBX0102

Thapwong, P. PESUC32

Thaya, L. PESAA02

Thermidor, G.E. PESUC31

Thirumurthy, H. EPLBC03

Tholanah, M. OAF0305

Thomas, A. OAD0204, PEMOD65, PESAA12

Thomas, D. PEMOE41

Thomas, F.T. PEMOD70

Thomas, K. OAE0105, PEMOE41

Thomas, R. OAC0105, PEMOC36

Thompson, L. OAC0104

Thomson, K. OAC0405

Thongsuksangcharoen, S. PESUC32

Thonon, F. EPLBD06

Thorburn, J. PEMOC33

Thorne, J.E. PESAB02

Thorsen, V. PEMOD55

Thwin, S.S. OAB0404

Tiam, A. PEMOE40

Tiemann, C. PESAC11

Tiemessen, C.T. PESAC16

Tien, P.C. PESAB13

Tierney, C. OAB0405, OALBB0102, PESUA19

Timothy, Z. OAD0603

Tinkham, K. OAD0205

Tippanna Chitti, K. PEMOD70

Titar, I. EPLBC02, PELBE01

Tlali, M. OAE0502

Tlali, R. OAE0203

Tobian, A. OAA0303

Tolmay, J. PESAC15

Tolstrup, M. PEMOA26

Tomaka, F. OALBA0102

Tomaras, G. OALBA0102, PELBA02, PESUA14

Tomaras, G.D. EPLBA02

Tongo, M. OAB0304

Tookes, H. OAB0102

Tordoff, D. PESAC07

Torres, M. PEMOA28, PESUA25

Torres, M.A. EPLBF02

Torres, T.S. PESAE12

Torruella, R. OAF0304

Toska, E. OAD0204, OAD0503, OAD0702, PEMOB31, PEMOD65, PESAC15

Tovar Sanchez, T. OAB0304

Townley, E. OAB0104

Toy, J. PEMOA38

Traeger, M. PESAC13

Traite, S. OAB0104

Tran Khanh, A. OAE0505

Tran Khanh, L. OAE0505

Tran Thi Huong, L. OAE0505

Traoré, D. PEMOD73

Trapence, C. EPLBB03

Trautmann, L. OAA0105, OAA0302

Treistman, M.S. PESAC02

Tremblay, C. OAA0202, PESAA07, PESAA09

Tressler, R. EPLBB08

Triamwichanon, R. PESUC22

Trichavaroj, R. PESUC32

Trihandini, I. OALBC0104

Tripon‐Manguiat, M.A. PESUD26

Trollope, G. OAA0304

Truong, H. OAC0102

Trypsteen, W. OAA0104

Tse‐Chang, A. OAC0204

Tsenesa, B. PESAD02

Tshabalala, M.F. EPLBE08

Tsotako, M. OAB0202

Tsukalov, I. PEMOA27

Tucker, J.D. PEMOF32

Tumwesigye, N.M. PEMOC42

Tun, W. PESUD28

Turpin, G. PEMOF28

Tusingweire, E. OAD0805

Tuyishime, M. EPLBA04

Twabi, H. OALBB0103

Tyler, A. PESAD07

### U

Uakuramenua, C. OALBE0104

Ubi, I. PEMOB30

Ubolyam, S. PESAB09

Udenkwo, D.I. PEMOB30

Ueaphongsukkit, T. PESAB09

Uemura, H. PESAB10

Ukwishaka, J. PESUC31

ul Hadi, S. PEMOD69

Umebido, C. OAE0103

Umviligihozo, G. PEMOA38

Unwin, J. PEMOC47

Upadhyay, C. EPLBA01, PESAA06

Ursenbach, A. PEMOA37

Usang, S. PEMOB30

Utomo, B. OALBC0104

### V

Vézina, D. OAA0405

Vadaq, N. EPLBA07

Valencia, D. OAC0405

Valencia, J. EPLBC04

Valente, S. PESAA04

Valenzuela Lara, M. OAD0304

Vallabhaneni, S. OAE0504, PEMOE45

Valle Millares, D. PESAA03

Vallejo, A. PEMOA32

Valois, S. OAB0105

van Bergen, J. PESAE04

Van Boven, T. EPLBB03

van de Wijer, L. EPLBA07

van den Bogaart, E. EPLBB07

van den Boom, W. PEMOC35

van der Heijden, W.A. EPLBA07

van der Valk, M. EPLBB05, PESAC12, PESAE04

van der Ven, A.J. EPLBA07

van Dijk, J. OAC0304

van Duijn, J. OALBA0102

van Eekeren, L.E. EPLBA07

Van Gulck, E. OAA0402

Van Handel, M. OAE0404

Van Leeuwen, J. PEMOD59

van Lettow, M. OAC0304

Van Lint, C. PESUA16, PESUA17

van Oosterhout, J. PESAE09

van Santen, D. PESAC12

van Santen, D.K. PEMOC35

van Snippenberg, W. OAA0104

van Vliet, J. EPLBB07

van Wyk, J. EPLBB05

Vandekerckhove, L. OAA0104, OAA0402

Vandenhombergh, J. EPLBF03, PEMOF34

VanderVeen, L. OALBA0105

Vanhulle, C. PESUA16, PESUA17

Vargas, S.K. PESAC08

Varloteaux, M. OAB0304

Varnauskas, A. EPLBE02

Vasan, S. PESAA10

Vasantiuppapokakorn, M. PESUC22

Vaudry, W. OAC0204

Vaughan, M. PEMOD52

Vaynos, M. OAD0502

Vedel, I. PESAD14

Vega‐Ramirez, H. PESAE12

Veldsman, K. PESAA11

Velloza, J. OAC0504

Veloso, V.G. PESAE12

Velter, A. EPLBD07

Vendrame, E. OALBA0105

Venter, W.D.F. PELBB01

Vera, F. EPLBB05

Vera, J. EPLBB04

Verdikt, R. PESUA16, PESUA17

Vergara, T.R.C. PESAC02

Verhasselt, B. PESUA16

Verma, N. PESAA08

Vermandere, H. PEMOE39, PESAE12

Vernazza, P. OAB0302

Verniers, K. OAA0104

Veyer, D. PEMOA35

Vhembo, T. OAB0405

Vicari, M. OAD0503

Vicente‐Izquierdo, L. PESUA23

Vicente, S. PESAD14

Vichea, O. PESUD26

Vigón, L. PESUA25

Vigon, L. PEMOA28

Vilaveces, A. PEMOC47

Villaester, M.J. PESUE29

Villaran, M. OALBC0102

Villardi, P. PESAF09

Villes, V. PEMOD67

Vinc, L. OAF0105

Vindevogel, S. PESUD49

Vinikoor, M.J. OAC0304

Vittangkrun, A. OAC0103

Vivancos, M.J. PEMOA32

Vizcarra, P. PEMOA32

Vo, P.N. EPLBA06

Voetsch, A.C. PELBC01

von Drehle, C. OAE0403

Vorontsova, L. PEMOF27

Voster, S. OAD0502

Vrbanac, V. PELBA01

Vu Ngoc, B. OAE0505

Vundamina, N. PEMOC34

### W

Wa Mwanza, M. OALBC0105

Wacharachaisurapol, N. PESAB09

Wadonda‐Kabondo, N. OAC0102, OAC0405

Wadonda, N. OAC0305

Waewklaihong, S. PELBD01

Wagner, C. OAD0502

Wagner, T. PESUA19

Wahl, A.R. OAA0403

Wainberg, M.L. OAF0204

Wainipitapong, S. OAF0204

Walker, D. PEMOD56

Walker, N. OALBB0103

Wallach, S. PESAD18, PESAD22

Wallin, J. OALBA0105

Walmsley, S. PESUB21

Walsh, S. PESUA14

Walsh, S.R. PELBA02

Walter, L. PESAE01

Wamalwa, D. PESAC05

Wamboya, D. PESAD17

Wambui, J.M. PESAD08

Wan Alias, W.M.H. PEMOA36

Wandeler, G. OAB0302

Wang, C. PEMOC38

Wang, G. PESAB03

Wang, H. OALBX0105

Wang, J. PESUF18

Wang, Q. PESUD29

Wang, S. EPLBA01

Wang, T. PESAB13

Wang, X. OALBC0103

Wang, Z. EPLBC04, OALBC0103

Wanicek, E. OAA0304

Wanjir, R. OAD0305

Wanjiru, M. OAD0305

Wanyenze, R. OAD0505

Warapornmongkholkul, A. PESUC29

Warburton, P. OAA0303

Ward, A. OAA0305, PESAA12

Ward, A.M. EPLBA02

Ward, D. PEMOC43, PESAF05

Ware, D. PEMOC45

Ware, N. PEMOE41

Waring, E. OAA0304

Warwick‐Sanders, M. PESUB24

Watala, K. PESUC26

Watanabe, D. PESAC17

Watanabe, K. PESAB10

Watkins, M. PESUE30

Watson, J. PESUD44

Wawer, M. EPLBC08, PESAE16

Wawrzyniak, A.J. OAC0403

Weatherburn, P. PEMOC43

Webb, K.A. PESUE27

Weber, R. PEMOD55, PESAD22

Wedderburn, C.J. OAB0204

Wei, H. OALBC0103

Wei, Y. PEMOA30

Weibel, S. OALBB0104

Weinreich, M.A. PESUB15

Weinstein, M.C. OAE0303

Weir, B.W. PESUC29

Weiss, H.A. OAD0305

Weissman, S. PESUD35, PESUF16

Wejnert, C. OAC0303

Wells, N. PESUD41

Wendler, C. PESAD20

Wendling, M.‐J. PEMOA37

Werb, D. PEMOF33, PESAD24

Were, N. PESUF17

West, C.A. OAC0305

West, S. PESUB22

Wester, C.W. PEMOB28

Wheeler, M. OALBX0102

Whitbread, J. OAF0305

Whitby, S. OAC0303, OALBX0106

White, G. EPLBD07

Whitehead, H.S. PESAE09

Whiteley, L. OAB0104

Whitney, B. PESUD30

Wiche Salinas, T. PESAA07

Wiche Salinas, T.R. OAA0103, OAA0202

Wienstein, M. EPLBF03

Wiginton, J.M. OAD0504, OALBD0105

Wiktor, S. PESAD18

Wilcher, R. PESAE11

Wilhelm, E. PESAA01

Wilkin, T. OAA0305

Wilkins, G. PESAF05

Wilkins, M. OAB0104, PESUA19

Wilkinson, E. PESAC06

Willems, W. OALBA0102

William Wester, C. OAE0502

Williams Sherlock, M. PESAE08

Williams, C. PELBD01

Williams, K. PEMOC49

Williamson, D. OAC0405

Willig, A. PESUD30

Willis, K. PESUC18

Wilson, E.C. PEMOF32

Wilton, J. EPLBC09

Wimmer, R. OALBA0103

Win, B. PESUB24

Windsor, L. OAD0803

Winters, S. PEMOE42

Wirtz, A.L. OAC0403, PESUC29

Wirya, A. PESAF04

Witkor, S. PESAD22

Wittesaele, C. PESAC15

Wittkop, L. PESAC12

Wittmer, R. PESAD14

Witzel, T.C. PEMOC43

Wiwa, O. OALBE0105, PESAB04

Wiznia, A. OAD0705, PESUA19

Wolfe, M. PESAD01

Wolinsky, S. PEMOC45

Wolstenholme, A. PESUB24

Wong, C.M. PEMOC34

Wong, C.S. PESUD42, PESUD47

Wong, E. OAC0202, PEMOD74

Wong, G. PEMOD57

Wong, J. EPLBC09

Wong, J.P.‐H. EPLBD05

Wong, M. PEMOD74

Wong, M.L. PESAD07

Worku, A. EPLBB03

Wosornu, S. PESUE24

Wosornu, S.K. PEMOD61

Wray‐Gordon, F. PELBC01

Wu, D. PESUD29

Wu, H.‐J. PESUC30

Wu, R.E. OALBC0103

Wu, T.‐H. PESUC30

Wu, X. OAC0203

Wudel, B. PESUC23

Wyatt, M.A. PEMOE41

Wyen, C. PEMOA28

Wynne, B. PESUB21

### X

Xaba, S. EPLBE04, PEMOE35

Xiao, Y. EPLBA05

Xin, R. OALBC0103

### Y

Yacovone, M. OALBC0102, PESUA14

Yagyu, S. PESUD50

Yakusik, A. OAE0406, PEMOD62, PEMOD72, PESUE31

Yalley, C. PEMOD61

Yan, X. PESUD29

Yanagawa, Y. PESAB10

Yang, C. OAC0203

Yang, K.J. PESAB12

Yang, L. PESAC09

Yang, X. PESUD35

Yang, Y. OAD0303, OALBD0102

Yanusa Nyako, H. PEMOD72

Yaseen, F. PEMOA38

Yates, A. PESAA10

Yates, N.L. EPLBA02

Yawa, A. PEMOE35

Yaya, I. EPLBD06

Ye, J. OALBC0103

Yeager, S. PESAD24

Yeh, P.T. PEMOD56

Yekeye, I. PESAF12

Yen, C. PELBA02

Yerly, S. OAB0302

Yeruva, A.R. PESAE05

Yeung, S. OAB0204

Yi, A. EPLBA06

Yiannoutsos, C.T. OAC0304

Yildrim, M. PESUE27

Yin, D. OALBB0102

Yombo‐Kokule, L. EPLBD06

Yoo‐Jeong, M. OAD0403

Yoon, C. PESAB08

Yoshimura, K. PESAC17

Yotebieng, M. PELBC02

Young, A. PELBC02

Young, B. PESUB25

Young, J. PESAC12

Young, L. PEMOA38

Younger, S. PESUA24

Youngleson, M. EPLBE08

Yu, C. PELBA02, PESUA14

Yu, F. PESUB25

Yu, P.‐C. EPLBB08

Yufenyuy, E. OAC0102

Yumnam, S. OAF0202

Yunusa Nyako, H. PESUE31

### Z

Zaia, J. OALBB0104

Zaikos, T. PESUA21

Zakaria, S. OALBX0102

Zakumumpa, H. PESAE07

Zambo, B. PEMOA34

Zanon Narchi, N. EPLBC07

Zanoni, B. PEMOD51

Zar, H.J. OAB0204

Zash, R. PELBB02

Zech, J.M. PEMOD60

Zender, N. OAC0102, PEMOE45

Zenengeya, E. OAC0405

Zeng, C. PESUD35

Zerbe Buba, A. PEMOD60

Zhai, K. OAC0203

Zhang, C. PESUC28

Zhang, F. OALBX0105

Zhang, H. OALBC0103, PESUB23

Zhang, J. PEMOD74, PESUD35

Zhang, K. PEMOD74

Zhang, L. EPLBA02, OALBA0105

Zhang, P. OAA0204

Zhang, W. OALBD0102

Zhang, Y. OAA0103, OAA0202

Zhang, Z. EPLBA07

Zhao, J. PESUC18

Zhao, L. OAC0104

Zhao, Y. EPLBB01

Zhou, S. OAD0503

Zhou, X. OAC0203

Zhou, Y. PESUD29

Zimunhu, E. OAD0104

Zinyemba, C. PESAF07

Zolla‐Pazner, S. EPLBA01

Zou, H. OAC0203

Zucman, D. EPLBD06

Zucoloto, G. PESAF09

Zulhaimi, N.S. PEMOA36

Zulu, A. OAD0104

Zulu, I. PESAE08, PESUE20

Zulu, M. OAE0203

Zulu, P.N. EPLBE08

Zungu, Y. EPLBA08, PEMOA31

Zuniga, J. OALBD0104

Zuniga, J.M. PESUF19

Zwangobani, N. EPLBE04

Zyambo, Z. PEMOC42

